# Ductal keratin 15^+^ luminal progenitors in normal breast exhibit a basal-like breast cancer transcriptomic signature

**DOI:** 10.1038/s41523-022-00444-8

**Published:** 2022-07-12

**Authors:** Katharina Theresa Kohler, Nadine Goldhammer, Samuel Demharter, Ulrich Pfisterer, Konstantin Khodosevich, Lone Rønnov-Jessen, Ole William Petersen, René Villadsen, Jiyoung Kim

**Affiliations:** 1 Department of Cellular and Molecular Medicine, Faculty of Health and Medical Sciences, University of Copenhagen, 2200 Copenhagen N, Denmark; 2 Novo Nordisk Foundation Center for Stem Cell Biology, Faculty of Health and Medical Sciences, University of Copenhagen, 2200 Copenhagen N, Denmark; 3 Biotech Research and Innovation Center, Faculty of Health and Medical Sciences, University of Copenhagen, 2200 Copenhagen N, Denmark; 4 Section for Cell Biology and Physiology, Department of Biology, Faculty of Science, University of Copenhagen, 2100 Copenhagen Ø, Denmark

**Keywords:** Mammary stem cells, Gene expression, Tumour heterogeneity, Differentiation

## Abstract

Normal breast luminal epithelial progenitors have been implicated as cell of origin in basal-like breast cancer, but their anatomical localization remains understudied. Here, we combine collection under the microscope of organoids from reduction mammoplasties and single-cell mRNA sequencing (scRNA-seq) of FACS-sorted luminal epithelial cells with multicolor imaging to profile ducts and terminal duct lobular units (TDLUs) and compare them with breast cancer subtypes. Unsupervised clustering reveals eleven distinct clusters and a differentiation trajectory starting with keratin 15^+^ (K15^+^) progenitors enriched in ducts. Spatial mapping of luminal progenitors is confirmed at the protein level by staining with critical duct markers. Comparison of the gene expression profiles of normal luminal cells with those of breast cancer subtypes suggests a strong correlation between normal breast ductal progenitors and basal-like breast cancer. We propose that K15^+^ basal-like breast cancers originate in ductal progenitors, which emphasizes the importance of not only lineages but also cellular position within the ductal-lobular tree.

## Introduction

Breast cancer is not a single disease. Rather, it relies on several different tumor subtypes each with their own phenotype and clinical outcome^1^ (for review see ref. ^2^). One of the most difficult-to-treat subtypes is the basal-like. Basal-like breast cancer originates from progenitor cells within the normal breast, typically among premenopausal women. We and others have previously narrowed down a luminal progenitor, which is double positive for K14 and K19 as a likely candidate cell of origin of basal-like breast cancer^3–6^. In basal-like breast cancer, apparent equivalents to double-positive cells are believed to contribute to aggressive behavior by taking on a leader role in invasion^7^. Indeed, knockdown of K14 in these cancer cells is sufficient to block what is referred to as collective invasion^7^. In primary tumors, the basal-like cells, reminiscent of normal double-positive cells are considered progenitors. In a tumor setting, these cells exhibit the potential of acquiring a hybrid epithelial-mesenchymal transition (EMT) state with a permanent aggressive potential^8^. Moreover, the EMT state seems to govern the level of progenitor activity as well as malignant behavior^8–11^. It is therefore important to understand in more detail the relationship between double-positive cells in the normal breast and the cells of the basal-like subtype of breast cancer.

Previous studies have emphasized the importance of spatial mapping based on combining micro-collection of organoids directly from reduction mammoplasties with quantitative fluorescence-activated cell sorting (FACS) and multicolor imaging to investigate human breast progenitors^3,12^. These and other studies have provided compelling evidence for the site-specific presence of distinct progenitors in ducts and TDLUs^3,12–14^. This observation is potentially much more far-reaching if viewed in context with data of distinct disease-free survival rates exclusively determined by cell of origin in ducts and lobules as determined by mammography and histology^15,16^. That duct-derived tumors may exhibit the worst prognosis^15,16^ emphasizes the importance of establishing further evidence for a relationship between normal double-positive progenitors in ducts and basal-like breast cancer.

Studies by others have recently employed scRNA-seq to describe the diversity of the epithelial cells in the human breast^17–20^. While these confirm the existence of three distinct epithelial populations irrespective of donor age and measures taken to enrich for epithelial cells prior to analysis, it remains unanswered whether the luminal epithelial compartment can be resolved further and whether transcriptional profiles relate to anatomical position. To address this, we here used micro-collected primary normal breast organoids to isolate trophoblast antigen 2^+^ (TROP2^+^)/CD271^−^ luminal epithelial cells from ducts and TDLUs and performed scRNA-seq. We discovered that the most immature luminal human breast epithelial cells reside in ducts and exhibit a unique expression profile that includes high levels of *KRT15*. This signature was found to correlate strongly with basal-like breast cancer. Together, our data provide a new level of resolution of phenotypes in the human normal breast for precision of cancer cell of origin studies.

## Results

### Combined micro-collection of organoids, FACS, and scRNA-seq lead to spatial mapping of differentially expressed genes of luminal epithelial cells in the human breast gland

**Fig. 1:**
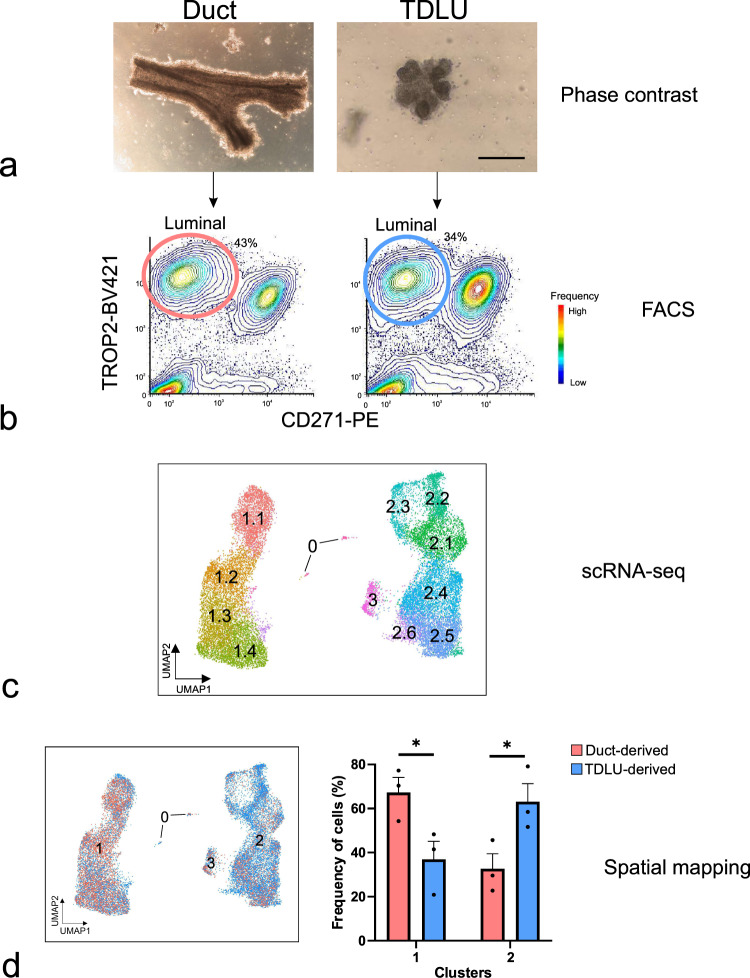
Combined micro-collection, FACS, and scRNA-seq assign duct- and TDLU-derived cells to separate clusters. **a** Phase-contrast micrographs of micro-collected ducts (left) and TDLUs (right) from human breast organoids. Scale bar = 100 μm. **b** FACS diagrams showing the gating of luminal cells (TROP2^+^/CD271^−^) from ducts (red circle, left) and TDLUs (blue circle, right) for scRNA-seq. Colors in FACS diagrams indicate frequency (red = high, blue = low frequency). **c** Uniform Manifold Approximation and Projection (UMAP) plot showing the clustering of luminal cells of three age-matched biopsies (20,286 cells). Clusters are denoted by color and labeled in the plot. Clusters: 0 as immune cells, 1.1–1.4 as luminal progenitors, and 2.1 to 2.6 and 3 as mature luminal cells according to differentially expressed genes (DEGs), some of which are shown in Fig. 2a. **d** (left) Same UMAP plot as in **c** showing contribution of duct- (red) and TDLU-derived (blue) luminal cells. (right) Bar graph illustrating contribution (relative frequency of cells in percent) of duct- (red) and TDLU-derived (blue) luminal cells to groups 1 and 2 of clusters. Error bars represent mean + standard error of the mean (SEM), *n* = 3 biopsies. *p < 0.05 by two-tailed *t*-test. Source data are provided as a Source Data file.

The presence of a luminal stem cell zone in ducts based on functional assays has been well described^3,5,21^. However, relatively little is known about the molecular constitution enabling the cells to function as progenitors or about the relationship between the stem cell hierarchy and tissue architecture. To address these questions, we micro-collected ducts and TDLUs from reduction mammoplasties, used lineage-specific cell-surface markers to enrich for luminal epithelial cells by a FACS protocol including TROP2 instead of EpCAM in combination with CD271 previously shown to optimize the separation of luminal and myoepithelial cells^12,22,23^, and subjected the resulting populations to scRNA-seq (Fig. 1a–c). To minimize variation due to age, parity, and ductal-lobular ratio^12,24,25^, we used biopsies from three same-aged young adults (age 18), collected in the range of 30 to 50 TDLUs and ducts, respectively, from each, and sorted a total number of 36,000 ductal- and 36,000 TDLU- derived cells for scRNA-seq. For an integrative analysis of the luminal lineage, we performed clustering of a total of 20,286 cells and on average 1300 genes per cell using Seurat (version 3.0)^26^, which identified 12 clusters with distinct gene expression profiles (Fig. 1c). Analysis of cluster entropy indicated unskewed contribution from the three biopsies to each cluster (Supplementary Fig. 1a). The cluster designated 0 included a minor proportion of cells (146 cells, 0.7% of the total population) reflecting the presence of immune cells, and thus, was excluded from further analysis. The cells were separated in two major groups of ten clusters labeled 1.1–1.4 and 2.1–2.6, respectively. The remaining cluster located in between was labeled cluster 3. Since the clustering relied on compiled data from ducts and lobules in separate, the contribution of each to the collective image was readily resolved and revealed a higher contribution of duct-derived cells to clusters 1.1–1.4, and to a significant level in cluster 1.2, and TDLU-derived cells to clusters 2.1–2.6 (Fig. 1d and Supplementary Fig. 1b).

### The ductal-enriched group comprises immature luminal progenitors including K14^+^/K19^+^ double-positive cells and a human-specific population of K15^+^ cells

**Fig. 2:**
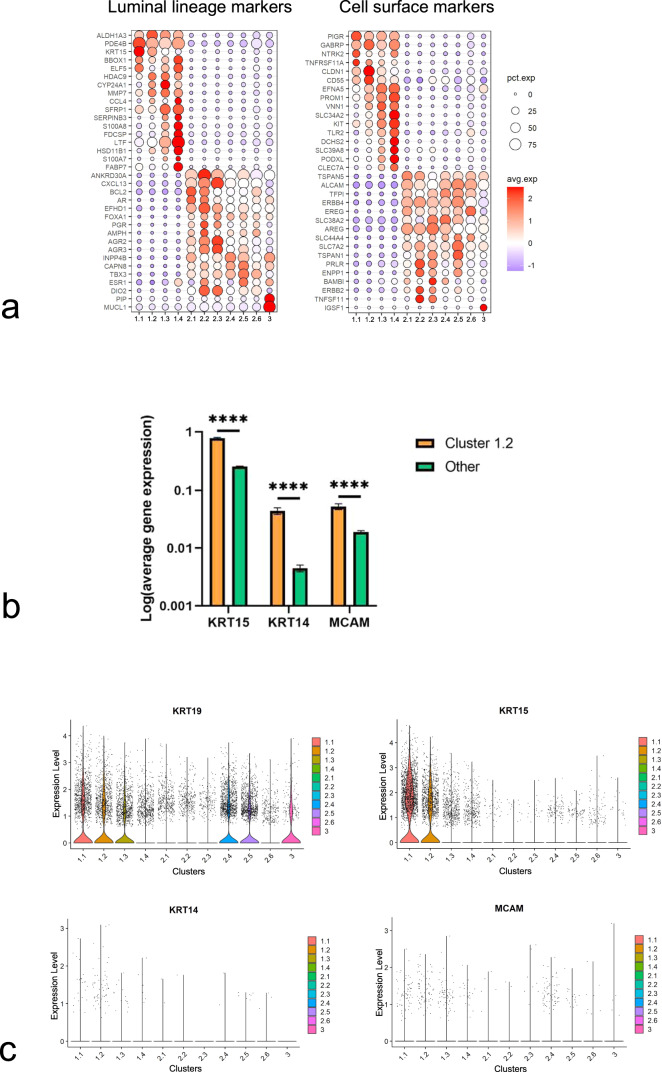

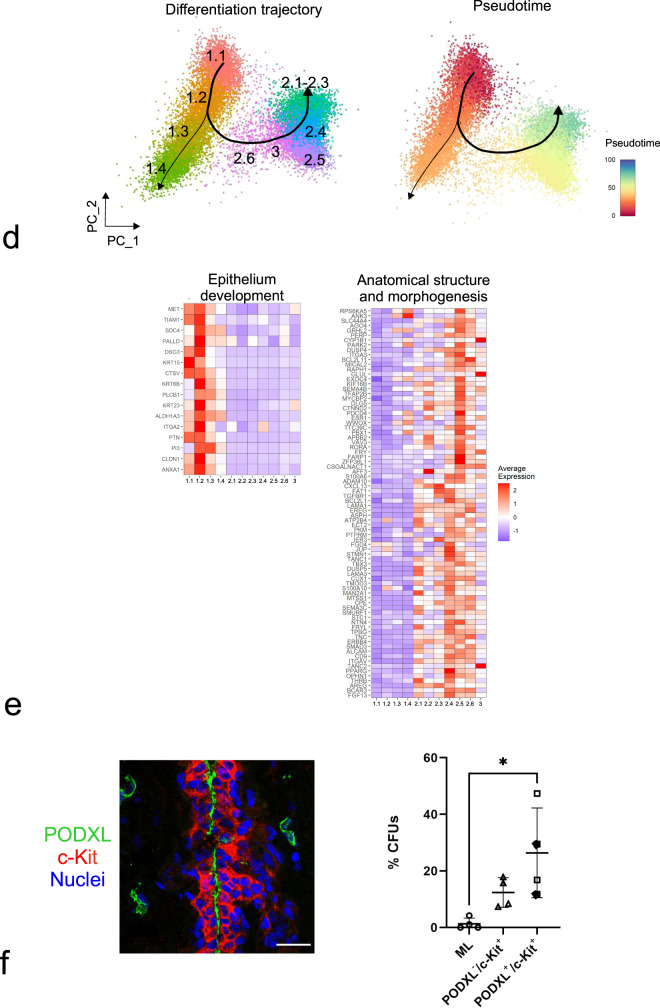
The ductal-enriched clusters 1.1–1.4 include immature progenitors. **a** Bubble plots showing a subset of cluster-specific DEGs of luminal lineage markers (left) and DEGs encoding cell-surface proteins (right). Circle size denotes the percentage of cells expressing the gene in each cluster, while color indicates the average expression level (red = high expression, blue = low expression). **b** Bar graph showing log-transformed gene expression of *KRT15*, *KRT14*, and *MCAM* in cluster 1.2 (orange) and in the other clusters (green). Error bars represent mean ± SEM. ****p < 0.001 by multiple two-tailed *t*-tests using False Discovery Rate (FDR) for multiple comparisons. **c** Violin plots showing expression of *KRT19, KRT15, KRT14*, and *MCAM* in all luminal clusters. **d** Principal component (PC) plots showing the trajectories of luminal differentiation (left) and pseudotime (right) using principal curves. Colors denote clusters (left) and pseudotime (right). The origin and endpoints were identified without supervision. Slingshot inferred cluster 1.1 as the origin and arrow heads indicate endpoints. **e** Heat maps showing expression of genes related to epithelium development (adj *p* < 0.001, g: Profiler analysis, left) and anatomical structure and morphogenesis (adj *p* < 0.0001, g:Profiler analysis, right) in the luminal clusters. **f** (left) Representative fluorescence multicolor imaging of normal breast stained for PODXL (green), c-Kit (red), and nuclei (blue). Scale bar = 25 μm. (right) Dot plot showing percentage of colony-forming units (CFUs) per 96 well-plate of sorted PODXL^−^/c-Kit^-^ mature luminal (ML), PODXL^−^/c-Kit^+^, and PODXL^+^/c-Kit^+^ luminal cells. Filled squares indicate PODXL^+^/c-Kit^−/+^. PODXL^+^ cells form significantly more colonies compared to ML cells (*n* = 4 biopsies). Error bars represent mean ± standard deviation (SD). *p < 0.05 by Kruskal–Wallis test with Dunn’s multiple comparisons test. Source data are provided as a Source Data file.

To infer the roles of the clusters, differentially expressed genes (DEGs) were examined and summarized in Fig. 2a and Supplementary Data 1. From these data, it became obvious that clusters 1.1–1.4 in general may represent immature progenitors by expression of, e.g., *ALDH1A3* (aldehyde dehydrogenase 1 family member A3) and *KRT15*^3,27–29^, whereas clusters 2.1–2.6 and 3 represent more mature luminal epithelial cells by expression of *BCL2*, *FOXA1* (forkhead box A1) and several endocrine receptors (Fig. 2a)^28,30,31^. This hierarchical division was echoed in a screen of lineage-specific cell-surface markers within the list of the in silico human surfaceome^32^. Thus, we found expression of established progenitor markers *TNFRSF11A* (RANK), *CD55*, *PROM1* (prominin 1) and *KIT* (c-Kit)^33–39^ in clusters 1.1–1.4 and differentiated luminal epithelial markers *ALCAM* (CD166), *AREG* (amphiregulin), and *TNFSF11* (RANKL) in clusters 2.1–2.6 (Fig. 2a)^28,40–42^. The full list of DEGs encoding cell-surface proteins is available in Supplementary Data 2. Intriguingly, the significantly duct-enriched cluster 1.2 accumulates *MCAM* (CD146), *KRT14* and *KRT15* expressing cells on a *KRT19*-positive background (Fig. 2b, c)—a combination of phenotypes, which have been amply validated in functional progenitor assays and which have been localized primarily to ducts at the protein level^3,21,28^. Cluster 3 was characterized by a significant expression of prolactin induced protein (*PIP*) and mucin like 1 (*MUCL1*) compared to other clusters. Also, immunoglobulin superfamily 1 (*IGSF1*) encoding a cell-surface molecule is exclusively upregulated in cluster 3 (Fig. 2a). To uncover any species related controversy concerning the generation of epithelial lineages^43^, we compared our data with existing scRNA-seq data based on the mouse mammary gland^44^. Of note, in accordance with others^45^, we found that expression of *KRT15* in the luminal compartment is specific for the human breast, and moreover that the mapping of the human stem cell hierarchy differs from that of mice (Supplementary Fig. 2a).

To further substantiate the analysis of maturation among clusters based on DEGs, we applied the lineage inference algorithm Slingshot^46^ in the search for a potential hierarchy in an unbiased and unsupervised manner. In short, this method identifies a trajectory based on a minimum spanning tree algorithm towards the most differentiated state. Cells that are placed closer to the beginning of the trajectory belong to an early time point in the lineage. Using our single-cell transcriptome data of luminal epithelial cells, Slingshot built several trajectories all starting in cluster 1.1 and ending in either clusters 2.1–2.3 or cluster 1.4, as summarized in Fig. 2d. Accordingly, estimation of pseudotime places the least differentiated cells in clusters 1.1–1.2 and the most differentiated cells of the luminal lineage in clusters 2.1–2.3 (Fig. 2d). These data were confirmed by geneset enrichment analysis showing that whereas clusters 1.1 and 1.2 are particularly high in genes involved in epithelium development (adj *p* < 0.001, Fig. 2e), suggesting a role upstream of epithelial differentiation within a hierarchy, clusters 2.1–2.6 are enriched for genes involved in anatomical structure morphogenesis (adj *p* < 0.0001, Fig. 2e). Additional gene sets in support of a hierarchical organization with respect to response to extracellular signaling, MAPK signaling, mammary gland development, nuclear receptor and ERBB signaling are highlighted in Supplementary Fig. 2b (adj *p* < 0.05). However, cluster 1.4 is somewhat of a conundrum by being the end of a separate trajectory never leaving the progenitor compartment (Fig. 2d). Therefore, in order to characterize this subcluster relative to the rest of cluster 1, we sought for a marker suitable for prospective isolation of subcluster 1.4 progenitors in a FACS-based protocol. We identified podocalyxin-like (*PODXL*) as an ideal candidate. *PODXL* is a gene encoding a member of the CD34 sialomucin protein family, which is expressed in hematopoietic stem cells^47^, and whose expression has been associated with basal-like breast cancer^48^. At the protein level, it is expressed at the apical surface of a subset of c-Kit^+^ luminal progenitors^35^ (Fig. 2f and Supplementary Fig. 2c), and PODXL^+^ cells from reduction mammoplasties can be readily identified in a FACS protocol with c-Kit included (Supplementary Fig. 2d). By this protocol we defined mature luminal cells as PODXL^−^/c-Kit^-^, c-Kit^+^ progenitors as PODXL^−^/c-Kit^+^, and PODXL^+^ progenitors as PODXL^+^/c-Kit^+/−^, respectively (Supplementary Fig. 2d). These three cell types were gated for and plated at clonal density in a colony formation assay (Supplementary Fig. 2e). Indeed, PODXL^+^ cells turned out to exhibit the highest colony-forming capability (Fig. 2f), confirming also at the functional level that cluster 1.4 represents a progenitor population.

### Micro-collection- and cluster-based spatial mapping is confirmed by multicolor imaging in situ

**Fig. 3:**
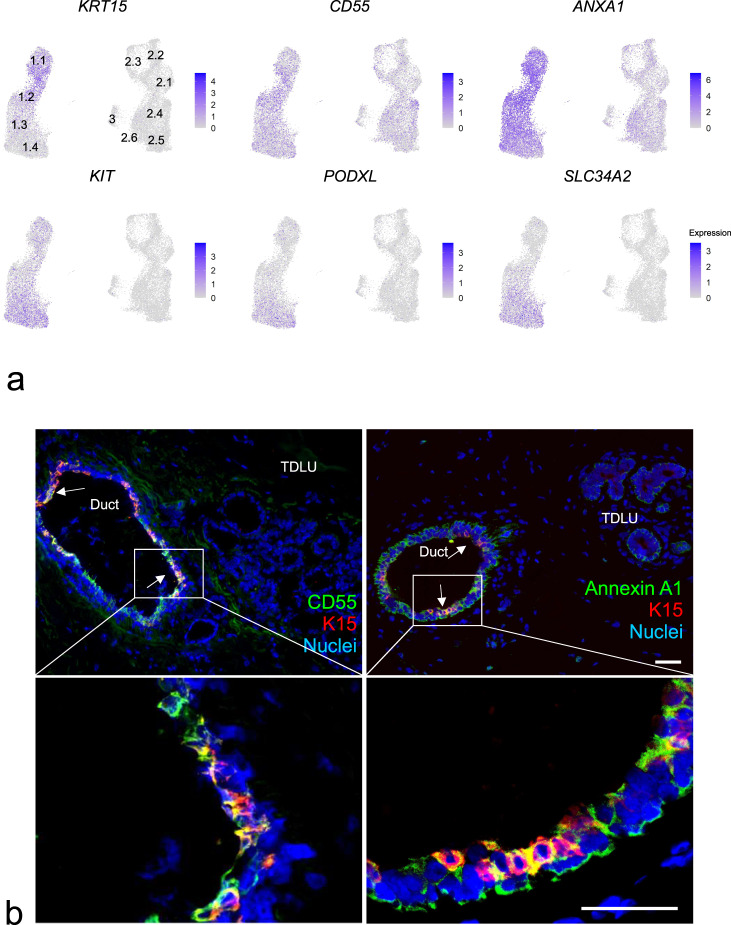
Cluster 1.2-associated CD55, annexin A1, and K15 preferentially localize to ducts. **a** Feature plots showing highest expression of *KRT15*, *CD55*, and *ANXA1* (annexin A1) in the area of cluster 1.2 and highest expression of *KIT*, *SLC34A2*, and *PODXL* in the area of cluster 1.4. **b** Representative fluorescence multicolor imaging of cryostat sections of normal breast stained for CD55 or annexin A1 (green) and K15 (red), and nuclei (blue). Arrows indicate CD55^+^/K15^+^ or annexin A1^+^/K15^+^ luminal cells. Images with higher magnification of selected areas (white squares) are shown below. Note that annexin A1 also stains myoepithelial cells. Annexin A1 and CD55 staining colocalizes with K15 preferentially in ductal luminal cells. Scale bars = 50 μm.

In order to validate the scRNA-seq cluster profiling and the apparent enrichment of duct-derived progenitors in cluster 1.2 and late progenitors in cluster 1.4 at the protein level with particular emphasis on surface markers, we searched our antibody repository and identified CD55 or annexin A1 and SLC34A2, respectively, as promising candidates. In line with our scRNA-seq data, we have previously found ductal-enriched expression of K15 and heterogeneous distribution of c-Kit expression by immunostaining^3,33^. Here, in an effort to classify cluster 1.2 we found that K15, due to its higher expression level, was superior to K14, which we have otherwise used as a ductal progenitor marker^3,21^. Thus, here we compared cluster 1.2-associated markers CD55 or annexin A1 with K15 as well as a cluster 1.4-associated marker SLC34A2 with c-Kit in ten different human breast biopsies using multicolor imaging. As inferred from the scRNA-seq (Fig. 3a), the majority of biopsies showed strong co-staining of CD55 with K15 (7 out of 10 biopsies, Fig. 3b) and to some extent co-staining of annexin A1 with K15 (5 out of 10 biopsies) in ducts compared to TDLUs (Fig. 3b), while SLC34A2 essentially co-stained with c-Kit (10 out of 10 biopsies) in both ducts and TDLUs (Supplementary Fig. 3a). To assess whether CD55 added to the established c-Kit protocol^28^ for enrichment of progenitors, smears were recovered from different gate combinations of FACS and stained for K15 (Supplementary Fig. 3b). A significantly higher frequency of strongly K15^+^ (K15^high^) cells was seen from the combined CD55/c-Kit gate compared to c-Kit alone (Supplementary Fig. 3c). We have previously found evidence of progenitor heterogeneity by comparing c-Kit^+^ and CD146^+^ cells functionally^21^. Our present data add to these differentiation programs, since CD55 co-stained with CD146 in ductal luminal cells, while CD146 rarely overlapped with the cell-surface marker PODXL, which is a cluster 1.4-associated cell-surface marker as shown above (Supplementary Fig. 3d, e)—all in line with the scRNA-seq data. The lack of overlap between the preferentially ductal CD55 and PODXL did not only unfold in ducts. Rather, the strongest PODXL staining was seen in CD55^neg^ TDLUs including the lobules proper (Supplementary Fig. 3f). Since we have previously shown that primarily the lobules and TDLUs are rich in hormone receptor-positive late progenitors and differentiated cells^28,49^, we here co-stained with PODXL and the surrogate marker, Ks20.8, of estrogen/progesterone receptor-positive cells^28^. Clearly, the hormone receptor-positive-cells were negative for PODXL (Supplementary Fig. 4a). Collectively, these data are in favor of the existence of a ductal luminal progenitor CD55^+^/K15^high^ cell, which is phenotypically and functionally distinct from those of TDLUs as summarized in Supplementary Fig. 4b.

### Comparison of duct and TDLU expression profiles with those of breast cancer subtypes

**Fig. 4:**
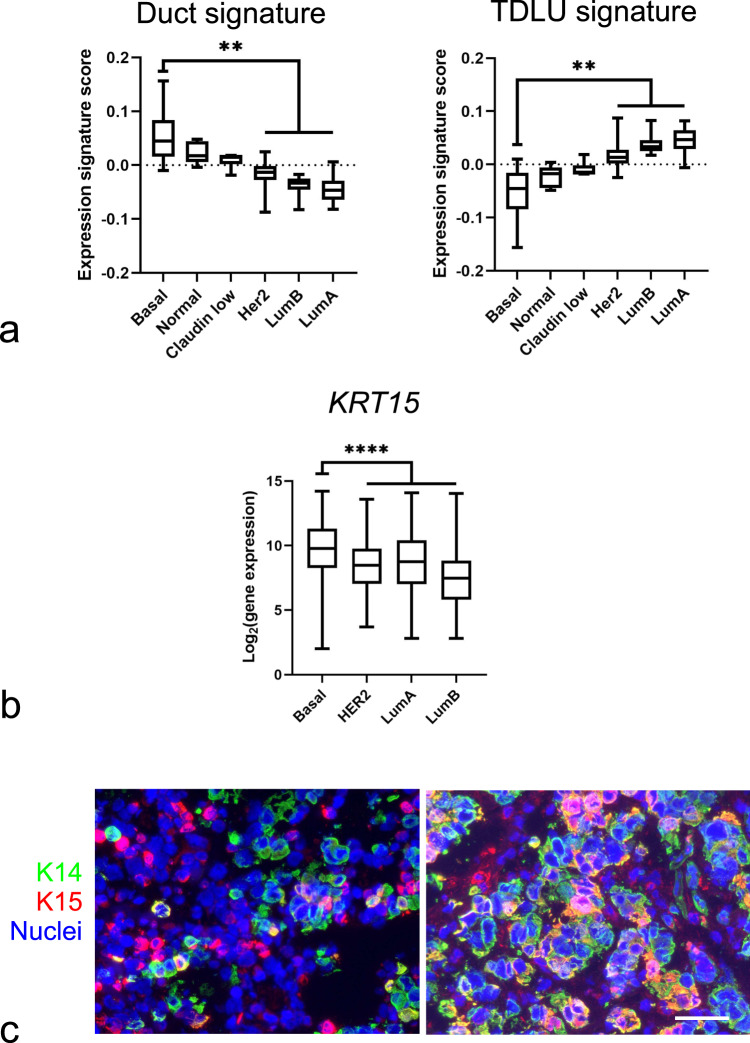
Correlation between expression profiles of duct- and TDLU-derived normal luminal cells and breast cancer subtypes. **a** Whisker box plots of expression signature scores comparing DEGs of duct- (left) and TDLU-derived (right) luminal cells with gene expression profiles of different breast cancer subtypes (basal-like (Basal), normal-like (Normal), Claudin^low^ (Claudin low), HER2-enriched (Her2), luminal type B (LumB), and luminal type A (LumA)). Boxes represent median ± quantiles. Whiskers indicate the minimum and maximum values. **p < 0.01 by Kruskal–Wallis test with Dunn’s multiple comparisons test. **b** Whisker box plot showing *KRT15* gene expression in the different breast cancer subtypes. *KRT15* is significantly upregulated in basal-like breast cancer compared to the other breast cancer subtypes (*n* = 2164, GENT2 database). Boxes represent median ± quantiles. Whiskers indicate the minimum and maximum values. ****p < 0.001 by one-way ANOVA with Tukey’s multiple comparison test. Source data are provided as a Source Data file. **c** Representative fluorescence multicolor imaging of cryostat sections of basal-like breast carcinomas stained for K15 (red), K14 (green), and nuclei (blue). Nine out of 36 basal-like breast carcinomas stained positive for both K15 and K14. Scale bar = 50 μm.

Finally, we investigated whether the identified single-cell transcriptome signatures overlapped with those of breast cancer. Gene expression and molecular characteristics of breast cancer have allowed the classification of breast cancer into several subtypes^1,50^. It has previously been shown that gene expression signatures of luminal progenitors are significantly correlated with basal-like breast cancer^35^. By adapting the method by Lim et al.^35^, we calculated the expression signature scores of ductal and TDLU luminal DEGs (Supplementary Data 3) and compared them to those of breast cancer subtypes^51^. As shown in Fig. 4a, the transcriptome signature of luminal cells in ducts exhibited an expression profile that was much more similar to those of the basal-like subtype of breast cancer than those of the luminal subtypes. The TDLU-derived luminal signature, on the other hand, showed a stronger compatibility with the luminal breast cancer subtypes (Fig. 4a). In addition, we asked which clusters aligned with the basal-like breast cancer subtypes using the established Prediction Analysis of Microarray 50 (PAM50) subtyping^52^, as well as a new subtyping classifier based on scRNAseq of breast cancer cells using the “SCSubtype” gene signatures^53^. Accordingly, PAM50 subtyping showed that clusters 1.1–1.4, in contrast to clusters 2 and 3, are more closely related to basal-like breast cancer (Supplementary Table 1). Likewise, upon comparison with SCSubtype gene signatures, clusters 1.2, 1.3, and 1.4 show a positive correlation with basal-like breast cancer (Supplementary Fig. 5). Recent molecular profiling has stratified basal-like breast cancer within triple-negative breast cancer (TNBC)^54–56^. We therefore further compared TNBC subtype gene signatures with clusters 1.1 to 1.4. Our analysis according to TNBC subtyping of DEGs among cluster 1, reveals that clusters 1.1–1.4 are related to basal-like 1/mesenchymal, basal-like 2/mesenchymal, basal-like 2 and immunomodulatory subtypes, respectively (Supplementary Fig. 6 and Supplementary Data 4). Intriguingly, assessment of the expression level of *KRT15* in a dataset of 2,164 breast cancer biopsies subdivided according to the most widely used classification showed a significantly higher expression level in basal-like breast cancer compared to any of the other subtypes (Fig. 4b)^57^. While others have reported that K15 protein is expressed among TNBC, HER2 and Luminal A carcinomas^58^, we here sought to corroborate that K15 is a marker of basal-like breast cancer also at the protein level. In a series of TNBC biopsies, 22/36 (61%) biopsies stained positive for K14 and 9/36 (25%) of these stained positive for K15. All nine K15^+^ biopsies contained K14^+^/K15^+^ cells in addition to K14^+^ and K15^+^ cells (Fig. 4c). Taken together, the data suggest that progenitors with a ductal profile represent the most immature cell type within the luminal lineage of the human breast and a likely source of basal-like breast cancer.

## Discussion

The present work demonstrates that the two major segments of the human breast ductal tree, i.e., the ducts and the TDLUs, are specifically enriched for cells reminiscent of the major breast cancer subtypes. This agrees with clinical data that duct- and TDLU-derived breast carcinomas exhibit unique histological and radiological appearances as well as clinical outcomes^15^. Our study opens for precision cell of origins comparisons with breast cancer subtypes. For example, we find that within the luminal epithelial lineage the most immature progenitors are characterized by the expression of basal-like breast cancer-associated K15 and a localization preferentially to ducts. Furthermore, we provide a proof of principle that luminal progenitors close to the apex of the hierarchy can be isolated by new combinations of surface markers revealing progenitors lending themselves to mechanistic studies of breast cancer subtype specific transformation and evolution. In the present study, scRNA-seq is based on biopsies from three age-matched young women. While this may serve as a starting point, importantly, we and others have shown that aberrant basal-like luminal cells accumulate with age^25,59,60^, thus implying that further studies of the resemblance between the transcriptomic profiles of normal luminal breast cells and breast cancer should take the age of the donors into account.

It is becoming increasingly clear not least by scRNA-seq that the luminal epithelial compartment consists of a multitude of progenitors and differentiated cells exhibiting molecular profiles overlapping with breast cancer subtypes^18,25,61–64^. Nevertheless, as far as cell of origin of breast cancer is concerned, the current view is centered around an estrogen receptor-negative, c-Kit-positive progenitor, which constitutes a relatively large proportion of cells widely distributed along the entire ductal-lobular tree^35,65,66^. This concurs with the widely held notion in the mouse mammary gland field based on early transplantation experiments that any part of the gland can give rise to the entire complement of the ductal-lobular tree if transplanted to a cleared fat pad (for review see^67^). Therefore, the alternative explanation to breast cancer subtypes is that they depend on the order and magnitude of genomic aberrations rather than distinct cells of origin, which is furthered here (for review see ref. ^67^). Indeed, early studies based on X-chromosome inactivation patterns showed that an entire duct and ductal-lobular unit had the same genomic constitution^68^. Therefore, the differences recorded in the present and previous studies are most likely governed by microenvironmental cues eventually leading to spatially determined, more permanent states of immaturity or differentiation^12–14,69^.

The present work highlights the value of ductal expression of K15 as a marker of an immature progenitor cell zone. K15 expression has been widely used as a biomarker for epithelial stem cells^70–72^. The antibody clone used here against K15 (LHK15) has been exhaustively shown to be entirely specific for K15^73^. However, the protein expression pattern in ducts of the human breast appears to be broader than what one would expect from cells near the apex of a differentiation hierarchy^43^. In general, human breast stem cells are believed to reside in the basal layer^12,74^. K15 is, however, expressed by luminal cells and not basal cells and as such it may have another function more relevant for progenitor or even differentiated cells. Analogously, in the esophagus, K15^+^ progenitors are found in the non-stem cell suprabasal layer (for review see also ref. ^75^). The fact that K15 in some tissues stain stem cells and in other tissues stain their progeny has been explained by different mechanisms of K15 regulation: a differentiation-specific mechanism involving the PKC/AP1 pathway and a basal-specific mechanism mediated by FOXM1^76^. Therefore, K15 expression in the normal breast undoubtedly covers more progenitors than those suspected of being cells of origin to breast cancer.

We also discovered additional markers of human breast progenitors. One of these, CD55, is enriched in cluster 1.2, spatially mapped primarily to ducts, and expressed coordinately to a great extent with K15. CD55 is a glycosylphosphatidylinositol-anchored protein, which regulates complement activation pathway, and it is also referred to as decay-accelerating factor 1 (Daf1). In human breast cancer cell lines, it renders cells resistant to apoptosis and thus facilitates tumorigenesis^77^. However, its function in normal breast is not known. A recent scRNA-seq study in the mouse mammary gland suggests CD55 as an early progenitor subset marker in the basal compartment and a marker of luminal transit cells during development^78^. In the adult mouse gland, CD55^+^ cells were found exclusively in the luminal epithelial compartment^78^. In addition, based on colony-forming assays, the CD55^+^ cells exhibited an about three times higher colony-forming capacity as an indication of their progenitor status^78^. Our colony formation data including c-Kit^+^ and PODXL^+^ luminal cells representing cells in group 1 of clusters are in good agreement with a progenitor status of this entire group of cells. We have previously shown a progenitor potential of CD146^+^ cells^21^. Whereas CD55 adds to the value of c-Kit for identifying progenitors in this compartment, the PODXL^+^ cells seem to mark a separate progenitor compartment. PODXL expression is of particular interest since others have reported that high PODXL expression is associated with higher risk categories, and that breast carcinomas with high PODXL expression are more likely to exhibit characteristics of basal-like cancers^48^. In support of such association between PODXL expression and prognosis, silencing of PODXL expression in a basal-like human breast cancer cell line reduces primary tumor formation and metastasis^79^, and analysis of the EMT program in an immortalized transformed human breast cell line reveals PODXL as a key promotor of extravasation^80^. The finding of PODXL primarily outside the CD55^+^ compartment combined with functional progenitor activity implicates the existence of a residential progenitor zone within the lobules. This may be at variance with our previous findings of limited colony-forming activity in cells from TDLUs^3^. It is possible, though, that the differences can be explained either by the use of different culture media in the two studies or that in the present study, both ductal and TDLU-derived PODXL^+^ cells contribute to the CFU activity. Importantly, however, we here find good agreement between staining, FACS and mRNA as far as CD146, CD55 and PODXL are concerned. Nevertheless, this does not exclude that broader populations of cells may be captured in particular at the protein level depending, e.g., on the threshold setting of detection^81^.

Collectively, we here provide evidence for a hitherto underappreciated spatial distribution of luminal progenitors and unravel a progenitor cell population in the ducts of the human breast with resemblance to basal-like breast cancer. These findings emphasize the relevance of cells of origin in breast cancer in general and pave the way for further investigation of the development and progression of basal-like breast cancer in particular.

## Methods

### Human tissue

The use of human tissue has been approved by the Scientific Ethical Committee of Region Hovedstaden and the Danish Data Protection Agency with reference to H-2-2011-052 and 2011-41-6722, respectively, and patients agreed to donate tissue by written consent. Normal breast tissue was acquired from 29 female donors undergoing reduction mammoplasty for cosmetic purposes. Donors remain anonymous except their ages at the time of surgery. 36 breast carcinoma specimens were donated by women undergoing mastectomy for primary breast cancer. Tissue was cut into pieces for cryo-sectioning or cut finely prior to dissociation using 900 U/ml collagenase solution (Worthington Biochemical) in DMEM/F12 (Gibco) supplemented with 2 mM glutamine and 50 μg/ml gentamycin (Biological Industries) to release epithelial organoids, upon collagenase digestion comprised of epithelium and adjacent stromal cells^82^, which were then stored in liquid nitrogen with 90% fetal bovine serum (F7524, Sigma-Aldrich) and 10% dimethyl sulfoxide (D2650, Sigma-Aldrich), which we find, is the optimal condition for freezing, thawing and survival^83^. Some of the biopsies used in this study have been included in previous studies^21,23,28,84^.

### Fluorescence-activated cell sorting (FACS)

Primary breast organoids were used for micro-collection (collection under the microscope) of ducts and TDLUs under the Leica DMIL microscope^3,12,23^. Organoids were dissociated using 0.25% trypsin in 1 mM EDTA (Sigma, E5134), following resuspension in HEPES buffer (Sigma, H3375) and filtering with a 100 μm filter^12,22^. Details regarding antibodies including the dilutions and catalog numbers used for all experiments included are summarized in Supplementary Table 2. Hereafter, samples were incubated at 4 °C with antibodies against an epithelial marker, TROP2 (brilliant violet (BV) 421 or BV510 conjugated) and a myoepithelial marker, CD271 (PE or APC conjugated) when sorting cells for scRNA-seq or with additional antibodies against c-Kit (BV421 or PE conjugated) and PODXL when cells were used for colony formation assays. Secondary antibody BV421-anti mouse IgM was added for staining the non-conjugated PODXL primary antibody. The secondary antibody was added to the control. For the comparison of PODXL and CD146, following antibodies were used; a luminal marker, EpCAM (CD326, BV786 conjugated), CD271 (PE conjugated), CD146 (AF647 conjugated) and PODXL, which is mentioned above. To sort luminal cells according to their expression of CD55 and c-Kit, TROP2 (BV510 conjugated), CD271 (APC conjugated) were used together with c-Kit (PE conjugated) and unconjugated CD55 in combinations with BV421-anti mouse IgM. After incubation, cells were washed twice with HEPES buffer and filtered through a 20 μm filter (BD, 340624) following addition of 1 μg/ml Fixable Viability Stain 780 (BD Horizon, 565388). Cell sorting was performed using the FACSAria™ Fusion Cytometer (BD) or the BD FACSAria™ III Cytometer with a 100 μm nozzle and prior multicolor compensations.

### Single-cell RNA sequencing

Organoids representing ducts and TDLUs (30–50 of each from each biopsy) from normal breast tissue of three age matched (18 years old) individuals were micro-collected and TROP2^+^/CD271^−^ luminal cells of each sample were sorted using FACS as described above. The myoepithelial cells were used in a separate study^23^.

Chromium Single-Cell 3’ Reagent Kits were employed for single-cell transcriptome sequencing. Version 2 (first two donors, PN-120237, PN-120236, PN-120262) or version 3 (last donor, PN-1000075, PN-1000073) of the kits were used for RNA isolation, cDNA amplification and library preparation. Hereafter, the Illumina® NextSeq500/550 High Output Kit v2 for 150 cycles (20024907) was used according to the manufacturer’s instructions for sequencing. Resulting files were demultiplexed and aligned to the human reference genome GRCh38-1.2.0.pre-mRNA. Hereafter, the data were filtered and barcodes as well as unique molecular identifiers were counted (10x Genomics Cell Ranger software). The R package “Seurat” version 3.0 was used for quality control, pre-processing (filtering, normalization, integration) and data analysis (clustering, data visualization, detection of differentially expressed genes)^26^. During filtering, cells with a feature count out of a range between 200 and 2000 as well as with a mitochondrial count higher than 10% were excluded. In order to analyze samples of three different donors together, datasets were integrated according to the “Integration and Label Transfer” tutorial of Seurat^26^. The equal contribution of biopsies to each cluster and cluster entropies were calculated to confirm successful integration of the data with an adapted function of the R package “Conos” (version 1.4.5)^85^. After clustering, the dimensionality reduction technique UMAP was applied to the whole dataset for visualization. DEGs for clusters were defined using a log_2_ fold-change (FC) cutoff of 0.1, and a threshold of 1% for the relative number of cells expressing the gene in the given group. Finally, only statistically significant genes (adjusted *p*-value < 0.05, Wilcoxon rank sum test together with Bonferroni correction for multiple testing) were used for further analysis. The in silico human surfaceome was utilized to search for genes encoding cell-surface proteins^32^.

### Comparison of DEGs to molecular breast cancer subtype classifiers

DEGs between ductal and TDLU luminal cells (Supplementary Data 3) were compared to the gene expression profile of breast cancer subtypes using a method that calculates “expression signature scores” based on log_2_FCs of DEGs in a geneset and gene expression values of the same genes in a reference dataset.^35^. Gene expression data of breast cancer were extracted from the Gene Expression Omnibus (GEO) with accession number GSE3165 and the platform GPL887 explained in more detail by Herschkowitz et al.^51^. The expression values were background-corrected and normalized by loess normalization using the R packages “affy” (version 1.68.0) and “limma” (version 3.46.0)^86,87^. An expression signature score was calculated for each combination of DEGs of ductal to TDLU and samples of breast cancer subtypes (Basal: *n* = 28, Normal: *n* = 6, Claudin low: *n* = 6, Her2: *n* = 14, LumB: *n* = 17, LumA: *n* = 22). Used values were the log_2_FC of marker genes in our single-cell sequencing data, including markers with a negative fold-change, and the expression value of the same genes in the breast cancer samples. A high log_2_FC as well as a high expression of a gene in the breast cancer subtype resulted in a larger expression signature score. Thus, high scores indicate a high similarity of the single-cell cluster with the cancer sample. Kruskal–Wallis one-way analysis of variance with Dunn’s multiple comparisons test was used for statistical analysis. In order to compare to molecular subtypes based on PAM50 gene signature^52^, we employed R package “genefu” (version 2.26.0)^88^ and estimated the probability of each cluster belonging to each subtype. For comparison with a single-cell method of breast cancer subtype classification “SCSubtype”^53^, expression signature scores were computated based on similarity between cluster-specific DEGs (Supplementary Data 1) and SCSubtype gene lists according to Lim et al.^35^. To further examine the basal-like breast cancer signatures within clusters 1.1 to 1.4, Lehmann´s TNBC gene signatures^55^ were compared to DEGs among clusters 1.1, 1.2, 1.3, 1.4 (Supplementary Data 4) using Lim et al.’s method above. Since only the gene lists and not the actual expression values were available for SCSubtype and TNBC gene signatures, we set a value of 1 as upregulated genes, while a value of –1 was used for downregulated genes for the calculations of expression signature scores.

### Pathway analysis

The functional enrichment analysis was performed using g:Profiler (version e102_eg49_p15_7a9b4d6, https://biit.cs.ut.ee/gprofiler) with a significance threshold of 0.05 after correcting for multiple testing^89^. Among the DEGs for cell clusters (Supplementary Data 1), genes of log_2_FC > 0.5, with >1.5 fold-change of the percentage of cells expressing the gene in a cluster compared to the rest of cells were tested for the “g:GOSt functional profiling” function and genes were sorted descendingly by log_2_FC.

### Trajectory inference

By use of a lineage inference tool, “Slingshot” (version 1.8.0)^46^, we calculated a differentiation trajectory and pseudotime of luminal cells. Cells from cluster 0 (immune cells) were excluded from the analysis. The values from the principal component analysis (PCA) and Seurat-annotated clusters were used as input. Neither starting nor ending clusters were pre-defined and trajectories were identified in an unsupervised manner.

### *KRT15* gene expression in breast cancer subtypes and in mice

*KRT15* gene expression in breast cancer subtypes was retrieved from the gene expression database of normal and tumor tissues (GENT2)^57^. Gene expression data of *Krt15* in the mouse mammary gland was obtained from Tabula Muris, a compendium of scRNA-seq data derived from mouse tissue^44^.

### Colony formation assay

Using FACS as described above, PODXL^+^, KIT^+^ and KIT^-^/PODXL^−^ cells were sorted into BioCoat™ Collagen I 96 Clear Well Plates (Corning, 354407) with 5 cells per well (1 cell/6.4 mm^2^). Cells were incubated for 3 weeks in TGFβR2i medium ((DMEM (Gibco, 21068028) and Ham’s F12 Nutrient Mix (Gibco, 21765029) 3:1 with 2 mM glutamine (Sigma, G7513), 5% FCS (Sigma-Aldrich, F7524), 0.5 μg/ml hydrocortisone (Sigma-Aldrich, H-0888), 5 μg/ml insulin (Sigma-Aldrich, I6634), 10 ng/ml cholera toxin (Sigma-Aldrich, C-8052), 10 ng/ml EGF (Peprotech, AF-100-15), 180 μM adenine (Sigma-Aldrich, A3159), 10 μM Y-27632 (AbMole BioScience, M1817), 5 nM amphiregulin (Peprotech, 100-55B), 25 μM RepSox (Sigma-Aldrich, R0158), and 10 μM SB431542 (Axon Medchem, 1661))^21,28^. Hereafter, cells were fixed with methanol (VWR Chemicals, 20847.307) for 5 min at –20 °C and stained with hematoxylin (Sigma-Aldrich, MHS16). For representative images, cells were stained with 0.4% crystal violet (Sigma Life Sciences, C6158) in 1:1 PBS and 96% ethanol. The number of wells with colonies was counted under a microscope (Leica DM2000), using a cutoff of 15 cells to define a colony. The assay was performed with four different biopsies.

### Immunohistochemistry and immunocytochemistry

Snap-frozen normal breast biopsies from reduction mammoplasties and archival TNBCs, characterized as such as estrogen receptor-negative, progesterone receptor-negative and human epidermal growth factor receptor 2-low/negative, K5-positive and/or K17-positive, were cut into 6 μm thick sections using a CryoStar NX50 cryostat (Thermo Scientific). Cryostat sections and cell smears upon FACS were fixed either with methanol (VWR Chemicals, 20847.307) for 5 min at –20 °C, or with 3.7% formaldehyde (Merck, 104002) for 10 min at room temperature following permeabilization with 0.1% Triton X-100 (Sigma-Aldrich, X-100)^3,28^. Sections were incubated with primary antibodies for 2 h (Supplementary Table 2) and secondary antibodies for 30 min, with PBS washes in between. Finally, ProLong™ Gold anti fade reagent with DAPI (Molecular Probes, P36934) was applied. Images were acquired using confocal microscopes (Leica DM5500B equipped with a DFC550 camera, or a Zeiss LSM710 confocal system). Staining results were strictly dependent on the fixation protocol and dilutions of antibodies as specified in Supplementary Table 2. For the quantification of K15^high^ cells in smears, ImageJ (version 1.53k) was used with a lower threshold of 20 for detection of cells with intense K15 staining. The archival breast carcinomas that had been characterized as TNBCs^84^ were stained for K5 and K17, before co-staining for K14 and K15.

### Statistical analysis

All statistical analyses were performed using the softwares R Studio (version 1.2.5001 and version 3.6.2) or GraphPad Prism (version 9.0.0). Data were tested for normal distribution using Shapiro–Wilk and Kolmogorov–Smirnov tests. Tests to determine significant differences between datasets were chosen separately for each experiment and are specified in the figure legends. Significance is indicated as follows: *p* < 0.05*, *p* < 0.01**, *p* < 0.005***, *p* < 0.001****.

## Supplementary information

Supplementary File

## Data Availability

Raw data of scRNA-seq are available in EGA European Genome-Phenome Archive with Study ID: EGAS00001005963 and all DEGs are provided as Supplementary Data 1 to 4. The source data underlying Figs. 1d, 2b, f, 3b, and 4a, b and Supplementary Figs. 1b, 3a, c, f, 5 and 6 are provided as a Source Data file.

## Source data

Source Data
Dataset1
Dataset2
Dataset 3
Dataset 4

## References

[1] Sørlie T (2001). Gene expression patterns of breast carcinomas distinguish tumor subclasses with clinical implications. Proc. Natl Acad. Sci. USA.

[2] Polyak K (2007). Breast cancer: origins and evolution. J. Clin. Invest.

[3] Villadsen R (2007). Evidence for a stem cell hierarchy in the adult human breast. J. Cell Biol..

[4] Keller PJ (2012). Defining the cellular precursors to human breast cancer. Proc. Natl Acad. Sci. USA.

[5] Engelsen AST (2020). AXL is a driver of stemness in normal mammary gland and breast cancer. iScience.

[6] Britschgi A (2017). The Hippo kinases LATS1 and 2 control human breast cell fate via crosstalk with ERalpha. Nature.

[7] Cheung KJ, Gabrielson E, Werb Z, Ewald AJ (2013). Collective invasion in breast cancer requires a conserved basal epithelial program. Cell.

[8] Kroger C (2019). Acquisition of a hybrid E/M state is essential for tumorigenicity of basal breast cancer cells. Proc. Natl Acad. Sci. USA.

[9] Petersen OW (1990). Differential tumorigenicity of two autologous human breast carcinoma cell lines, HMT-3909S1 and HMT-3909S8, established in serum-free medium. Cancer Res..

[10] Petersen OW (2001). The plasticity of human breast carcinoma cells is more than epithelial to mesenchymal conversion. Breast Cancer Res..

[11] Petersen OW (2003). Epithelial to mesenchymal transition in human breast cancer can provide a nonmalignant stroma. Am. J. Pathol..

[12] Fridriksdottir AJ (2017). Proof of region-specific multipotent progenitors in human breast epithelia. Proc. Natl Acad. Sci. USA.

[13] Arendt LM (2014). Anatomical localization of progenitor cells in human breast tissue reveals enrichment of uncommitted cells within immature lobules. Breast Cancer Res..

[14] Honeth G (2015). Models of breast morphogenesis based on localization of stem cells in the developing mammary lobule. Stem Cell Rep..

[15] Tabar L (2014). A proposal to unify the classification of breast and prostate cancers based on the anatomic site of cancer origin and on long-term patient outcome. Breast Cancer (Auckl..

[16] Tabar L (2022). A new approach to breast cancer terminology based on the anatomic site of tumour origin: The importance of radiologic imaging biomarkers. Eur. J. Radio..

[17] Nguyen QH (2018). Profiling human breast epithelial cells using single cell RNA sequencing identifies cell diversity. Nat. Commun..

[18] Bhat-Nakshatri P (2021). A single-cell atlas of the healthy breast tissues reveals clinically relevant clusters of breast epithelial cells. Cell Rep. Med..

[19] Pal B (2021). A single-cell RNA expression atlas of normal, preneoplastic and tumorigenic states in the human breast. EMBO J..

[20] Peng, S., Hebert, L. L., Eschbacher, J. M. & Kim, S. Single-Cell RNA Sequencing of a Postmenopausal Normal Breast Tissue Identifies Multiple Cell Types That Contribute to Breast Cancer. *Cancers (Basel)* **12**, 10.3390/cancers12123639 (2020). 10.3390/cancers12123639 PMC7761899 33291647

[21] Isberg OG (2019). A CD146 FACS protocol enriches for luminal keratin 14/19 double positive human breast progenitors. Sci. Rep..

[22] Goldhammer N, Kim J, Timmermans-Wielenga V, Petersen OW (2019). Characterization of organoid cultured human breast cancer. Breast Cancer Res..

[23] Goldhammer N, Kim J, Villadsen R, Ronnov-Jessen L, Petersen OW (2022). Myoepithelial progenitors as founder cells of hyperplastic human breast lesions upon PIK3CA transformation. Commun. Biol..

[24] Russo J, Rivera R, Russo IH (1992). Influence of age and parity on the development of the human breast. Breast Cancer Res Treat..

[25] Pelissier Vatter FA (2018). High-dimensional phenotyping identifies age-emergent cells in human mammary epithelia. Cell Rep..

[26] Stuart T (2019). Comprehensive integration of single-cell data. Cell.

[27] Ginestier C (2007). ALDH1 is a marker of normal and malignant human mammary stem cells and a predictor of poor clinical outcome. Cell Stem Cell.

[28] Fridriksdottir AJ (2015). Propagation of oestrogen receptor-positive and oestrogen-responsive normal human breast cells in culture. Nat. Commun..

[29] Celis JE (2007). Identification of a subset of breast carcinomas characterized by expression of cytokeratin 15: relationship between CK15+ progenitor/amplified cells and pre-malignant lesions and invasive disease. Mol. Oncol..

[30] Bernardo GM (2010). FOXA1 is an essential determinant of ER alpha expression and mammary ductal morphogenesis. Development.

[31] Lu QL, Abel P, Foster CS, Lalani EN (1996). bcl-2: role in epithelial differentiation and oncogenesis. Hum. Pathol..

[32] Bausch-Fluck D (2018). The in silico human surfaceome. Proc. Natl Acad. Sci. USA.

[33] Kim J, Villadsen R (2018). Expression of luminal progenitor marker CD117 in the human breast gland. J. Histochem. Cytochem..

[34] Anderson LH, Boulanger CA, Smith GH, Carmeliet P, Watson CJ (2011). Stem cell marker prominin-1 regulates branching morphogenesis, but not regenerative capacity, in the mammary gland. Dev. Dyn..

[35] Lim E (2009). Aberrant luminal progenitors as the candidate target population for basal tumor development in BRCA1 mutation carriers. Nat. Med.

[36] Joshi PA (2015). RANK signaling amplifies WNT-responsive mammary progenitors through R-SPONDIN1. Stem Cell Rep..

[37] Sigl V (2016). RANKL/RANK control Brca1 mutation. Cell Res..

[38] Regan JL (2012). c-Kit is required for growth and survival of the cells of origin of Brca1-mutation-associated breast cancer. Oncogene.

[39] Nolan E (2016). RANK ligand as a potential target for breast cancer prevention in BRCA1-mutation carriers. Nat. Med..

[40] Sternlicht MD (2005). Mammary ductal morphogenesis requires paracrine activation of stromal EGFR via ADAM17-dependent shedding of epithelial amphiregulin. Development.

[41] Burkhardt M (2006). Cytoplasmic overexpression of ALCAM is prognostic of disease progression in breast cancer. J. Clin. Pathol..

[42] Tanos T (2013). Progesterone/RANKL is a major regulatory axis in the human breast. Sci. Transl. Med.

[43] Dontu G, Ince TA (2015). Of mice and women: a comparative tissue biology perspective of breast stem cells and differentiation. J. Mammary Gland Biol. Neoplasia.

[44] Tabula Muris C (2018). Single-cell transcriptomics of 20 mouse organs creates a Tabula Muris. Nature.

[45] Saeki K (2021). Mammary cell gene expression atlas links epithelial cell remodeling events to breast carcinogenesis. Commun. Biol..

[46] Street K (2018). Slingshot: cell lineage and pseudotime inference for single-cell transcriptomics. BMC Genomics.

[47] Nielsen JS, McNagny KM (2008). Novel functions of the CD34 family. J. Cell Sci..

[48] Forse CL (2013). Elevated expression of podocalyxin is associated with lymphatic invasion, basal-like phenotype, and clinical outcome in axillary lymph node-negative breast cancer. Breast Cancer Res. Treat..

[49] Petersen OW, Høyer PE, van Deurs B (1987). Frequency and distribution of estrogen receptor-positive cells in normal, nonlactating human breast tissue. Cancer Res..

[50] Perou CM (2000). Molecular portraits of human breast tumours. Nature.

[51] Herschkowitz JI (2007). Identification of conserved gene expression features between murine mammary carcinoma models and human breast tumors. Genome Biol..

[52] Parker JS (2009). Supervised risk predictor of breast cancer based on intrinsic subtypes. J. Clin. Oncol..

[53] Wu SZ (2021). A single-cell and spatially resolved atlas of human breast cancers. Nat. Genet..

[54] van de Rijn M (2002). Expression of cytokeratins 17 and 5 identifies a group of breast carcinomas with poor clinical outcome. Am. J. Pathol..

[55] Lehmann BD (2011). Identification of human triple-negative breast cancer subtypes and preclinical models for selection of targeted therapies. J. Clin. Invest.

[56] Tang P, Tse GM (2016). Immunohistochemical surrogates for molecular classification of breast carcinoma: a 2015 update. Arch. Pathol. Lab. Med..

[57] Park SJ, Yoon BH, Kim SK, Kim SY (2019). GENT2: an updated gene expression database for normal and tumor tissues. BMC Med Genomics.

[58] Moreira JM (2010). Tissue proteomics of the human mammary gland: towards an abridged definition of the molecular phenotypes underlying epithelial normalcy. Mol. Oncol..

[59] Garbe JC (2012). Accumulation of multipotent progenitors with a basal differentiation bias during aging of human mammary epithelia. Cancer Res.

[60] Shalabi SF (2021). Evidence for accelerated aging in mammary epithelia of women carrying germline BRCA1 or BRCA2 mutations. Nat. Aging.

[61] Chen W (2019). Single-cell landscape in mammary epithelium reveals bipotent-like cells associated with breast cancer risk and outcome. Commun. Biol..

[62] Rosenbluth JM (2020). Organoid cultures from normal and cancer-prone human breast tissues preserve complex epithelial lineages. Nat. Commun..

[63] Santagata S (2014). Taxonomy of breast cancer based on normal cell phenotype predicts outcome. J. Clin. Invest.

[64] Hu T, Zhao G, Liu Y, Long M (2021). A machine learning approach to differentiate two specific breast cancer subtypes using androgen receptor pathway genes. Technol. Cancer Res. Treat..

[65] Molyneux G (2010). BRCA1 basal-like breast cancers originate from luminal epithelial progenitors and not from basal stem cells. Cell Stem Cell.

[66] Proia TA (2011). Genetic predisposition directs breast cancer phenotype by dictating progenitor cell fate. Cell Stem Cell.

[67] Visvader, J. E. & Smith, G. H. Murine mammary epithelial stem cells: discovery, function, and current status. *Cold Spring Harb. Perspect. Biol* **3**, 10.1101/cshperspect.a004879 (2011). 10.1101/cshperspect.a004879 PMC3039534 20926515

[68] Deng G, Lu Y, Zlotnikov G, Thor AD, Smith HS (1996). Loss of heterozygosity in normal tissue adjacent to breast carcinomas. Science.

[69] Morsing M (2020). Fibroblasts direct differentiation of human breast epithelial progenitors. Breast Cancer Res..

[70] Yoshida S (2006). Cytokeratin 15 can be used to identify the limbal phenotype in normal and diseased ocular surfaces. Invest. Ophthalmol. Vis. Sci..

[71] Liu Y, Lyle S, Yang Z, Cotsarelis G (2003). Keratin 15 promoter targets putative epithelial stem cells in the hair follicle bulge. J. Invest. Dermatol.

[72] Giroux V (2017). Long-lived keratin 15+ esophageal progenitor cells contribute to homeostasis and regeneration. J. Clin. Invest.

[73] Aldehlawi H (2019). The monoclonal antibody EPR1614Y against the stem cell biomarker keratin K15 lacks specificity and reacts with other keratins. Sci. Rep..

[74] Eirew P (2008). A method for quantifying normal human mammary epithelial stem cells with in vivo regenerative ability. Nat. Med..

[75] Bose A, Teh MT, Mackenzie IC, Waseem A (2013). Keratin k15 as a biomarker of epidermal stem cells. Int. J. Mol. Sci..

[76] Bose A (2012). Two mechanisms regulate keratin K15 expression in keratinocytes: role of PKC/AP-1 and FOXM1 mediated signalling. PLoS ONE.

[77] Ikeda J (2008). Prognostic significance of CD55 expression in breast cancer. Clin. Cancer Res..

[78] Pal B (2017). Construction of developmental lineage relationships in the mouse mammary gland by single-cell RNA profiling. Nat. Commun..

[79] Snyder KA (2015). Podocalyxin enhances breast tumor growth and metastasis and is a target for monoclonal antibody therapy. Breast Cancer Res..

[80] Frose J (2018). Epithelial-mesenchymal transition induces podocalyxin to promote extravasation via Ezrin Signaling. Cell Rep..

[81] Virtanen S, Schulte R, Stingl J, Caldas C, Shehata M (2021). High-throughput surface marker screen on primary human breast tissues reveals further cellular heterogeneity. Breast Cancer Res.

[82] Stampfer M, Hallowes RC, Hackett AJ (1980). Growth of normal human mammary cells in culture. Vitro.

[83] Rønnov-Jessen L, Petersen OW (1993). Induction of alpha-smooth muscle actin by transforming growth factor-beta 1 in quiescent human breast gland fibroblasts. Implications for myofibroblast generation in breast neoplasia. Lab. Invest..

[84] Bechmann MB, Brydholm AV, Codony VL, Kim J, Villadsen R (2020). Heterogeneity of CEACAM5 in breast cancer. Oncotarget.

[85] Barkas N (2019). Joint analysis of heterogeneous single-cell RNA-seq dataset collections. Nat. Methods.

[86] Gautier L, Cope L, Bolstad BM, Irizarry RA (2004). affy-analysis of Affymetrix GeneChip data at the probe level. Bioinformatics.

[87] Ritchie ME (2015). limma powers differential expression analyses for RNA-sequencing and microarray studies. Nucleic Acids Res..

[88] Gendoo DM (2016). Genefu: an R/Bioconductor package for computation of gene expression-based signatures in breast cancer. Bioinformatics.

[89] Raudvere U (2019). g:Profiler: a web server for functional enrichment analysis and conversions of gene lists (2019 update). Nucleic Acids Res..

