# Supplementary material for: Ductal keratin 15+ luminal progenitors in normal breast exhibit a basal-like breast cancer transcriptomic signature
Source: NPJ Breast Cancer. 2022 Jul 12;8:81. doi: 10.1038/s41523-022-00444-8 (PMC9276673; doi:10.1038/s41523-022-00444-8)
Supplement: Supplementary file 1 — Supplementary File [file 41523_2022_444_MOESM1_ESM.pdf]

Supplementary Figure 1

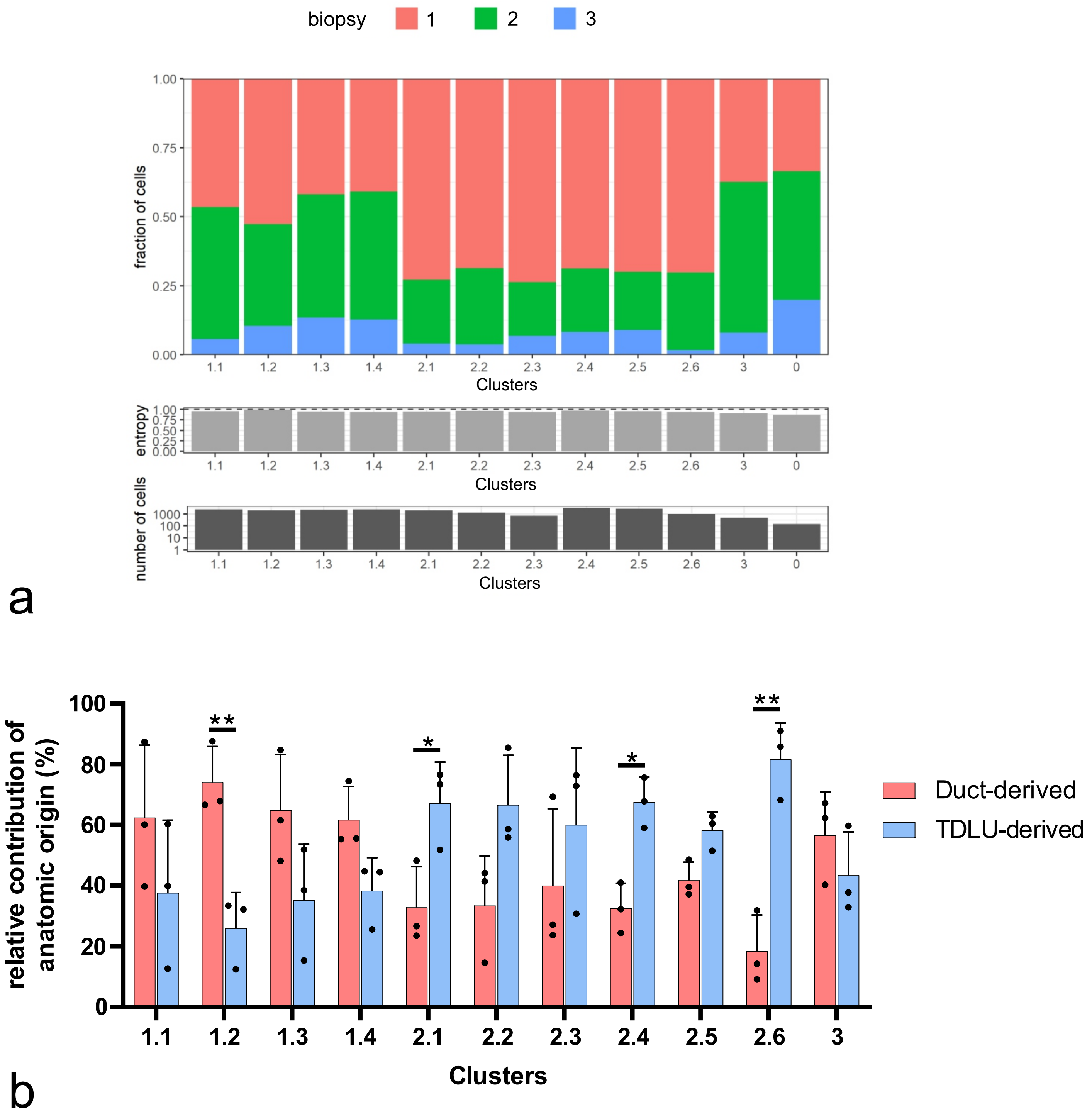

Supplementary Figure 1. Combined micro-collection, FACS, and scRNA-seq assign duct- and TDLU-derived cells to separate clusters. (a) Analysis of the contribution of each biopsy to the clusters from the scRNA-seq analysis. Stacked bar chart indicates the fraction of cells of each sample in a given cluster (top). Middle bar graph shows the cluster entropy for each cluster, indicating unskewed contribution of biopsies, while the bottom bar graph denotes the total number of cells in each cluster. (b) Bar graph showing the relative contribution in percentage of duct- (red) and TDLU-derived (blue) luminal cells to each of the clusters. Error bars represent mean + SD, n = 3 biopsies. *$p<0.05$, **$p<0.01$ by multiple two-tailed t tests. Source data are provided as a Source Data file.

Supplementary Figure 2

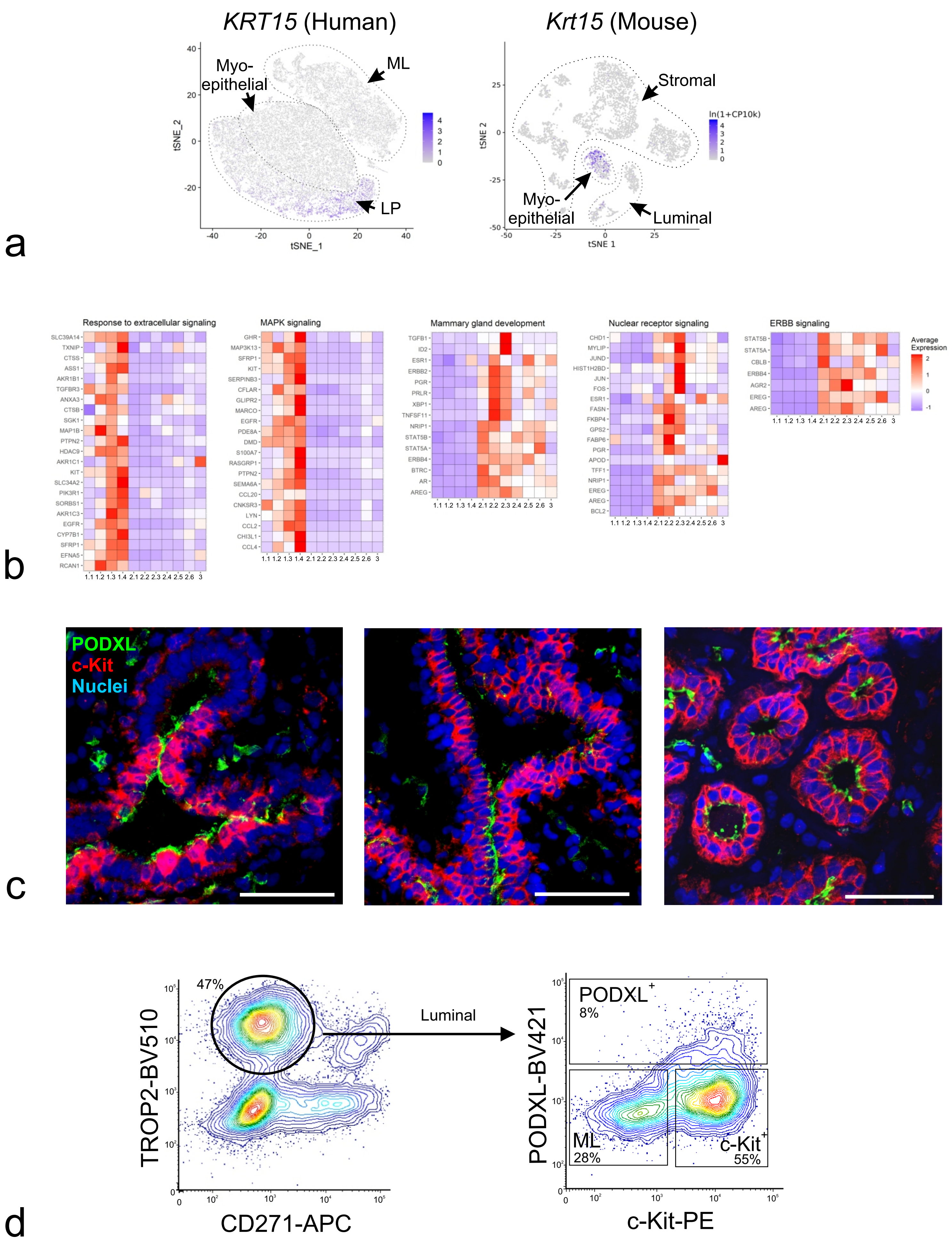

Supplementary Figure 2 continued

ML

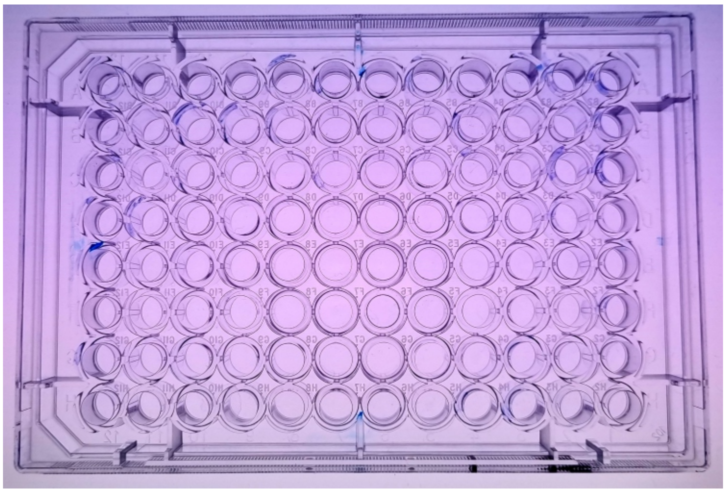

PODXL$^-$/c-Kit$^+$

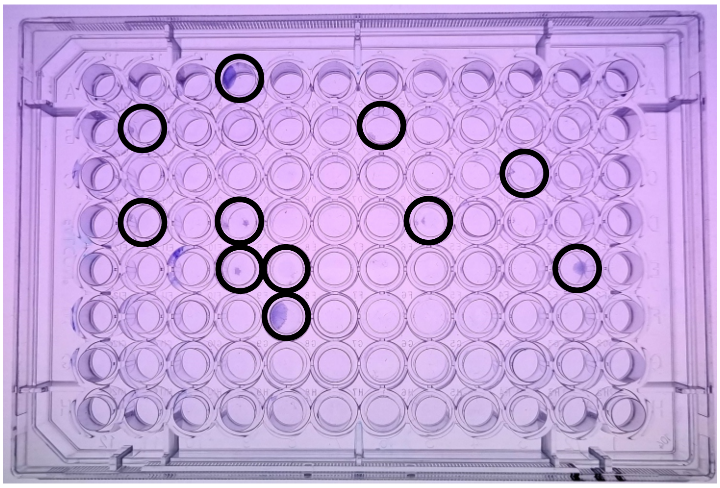

PODXL$^+$/c-Kit$^+$

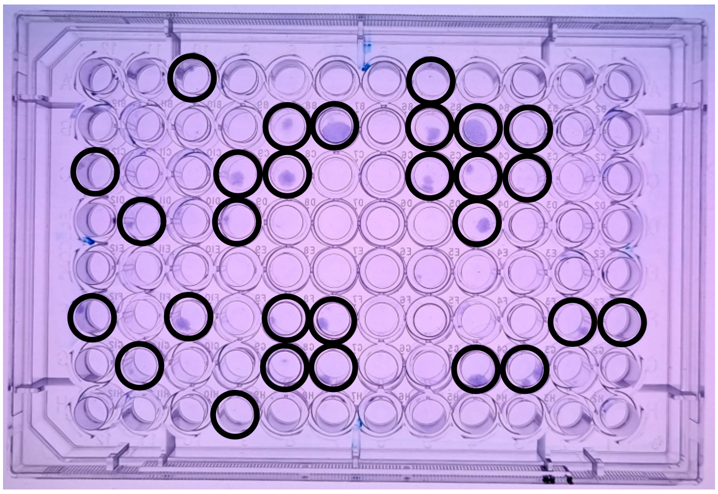

e

Supplementary Figure 2. PODXL$^+$ cells exhibit progenitor capacity. (a) tSNE plots showing expression of KRT15 in normal epithelial cells of human breast (left) and mouse mammary gland (right). Note that human scRNA data include epithelial cells (myoepithelial cells, luminal progenitor cells (LPs) and mature luminal cells (ML)), whereas mouse data, which are adapted from Tabula Muris, include epithelial cells as well as stromal and immune cells (Stromal). While *KRT15* is expressed in a subset of luminal progenitor cells (LP) in human, *Krt15* in the mouse mammary gland is expressed in a subset of myoepithelial cells. (Blue = high, grey = low expression of *KRT15* or *Krt15*) (b) Heat maps showing expression of statistically significant gene sets related to response to extracellular signaling (adj $p < 0.01$), MAPK signaling (adj $p < 0.005$), mammary gland development (adj $p < 0.05$), nuclear receptor signaling (adj $p < 0.01$), and ErbB signaling (adj $p < 0.05$) in the luminal clusters. (c) Fluorescence multicolor imaging of normal breast stained for PODXL (green), c-Kit (red) and nuclei (blue) showing that expression of PODXL is more restricted than c-Kit. Scale bars = 50 μm. (d) FACS plots showing gating for luminal cells (TROP2$^+$/CD271$^-$, left) that were further subdivided into c-Kit$^-$/PODXL$^-$ ML cells, c-Kit$^+$ and PODXL$^+$ luminal progenitors (right). (e) Representative images of 96 well plates with colonies stained with crystal violet to illustrate difference in colony formation capacity among PODXL$^-$/c-Kit$^-$ (ML), PODXL$^-$/c-Kit$^+$ and PODXL$^+$/c-Kit$^+$ luminal cell populations.

Supplementary Figure 3

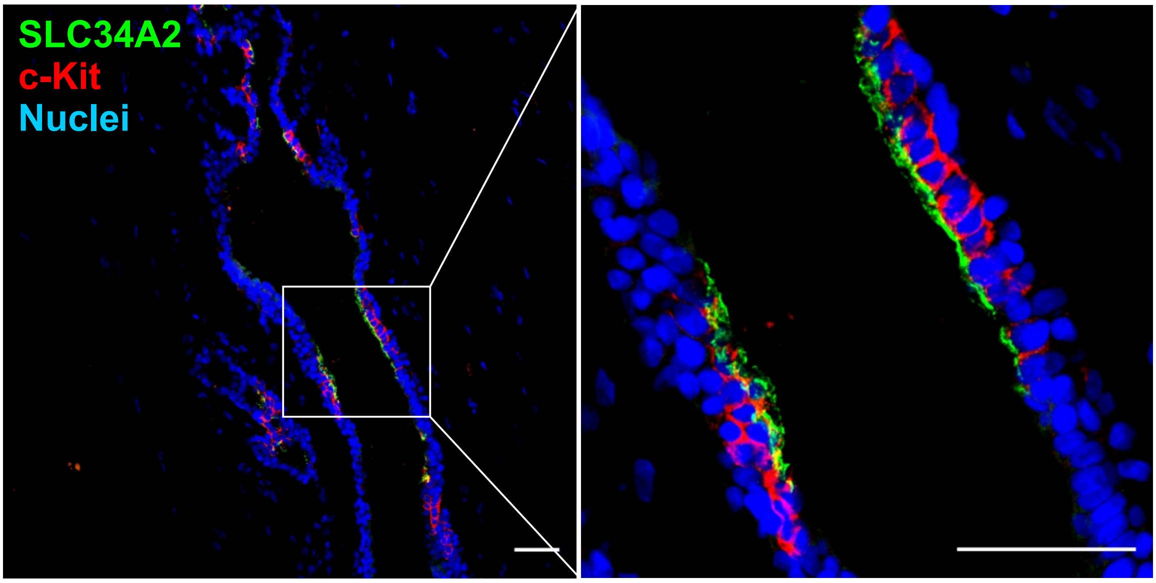

a

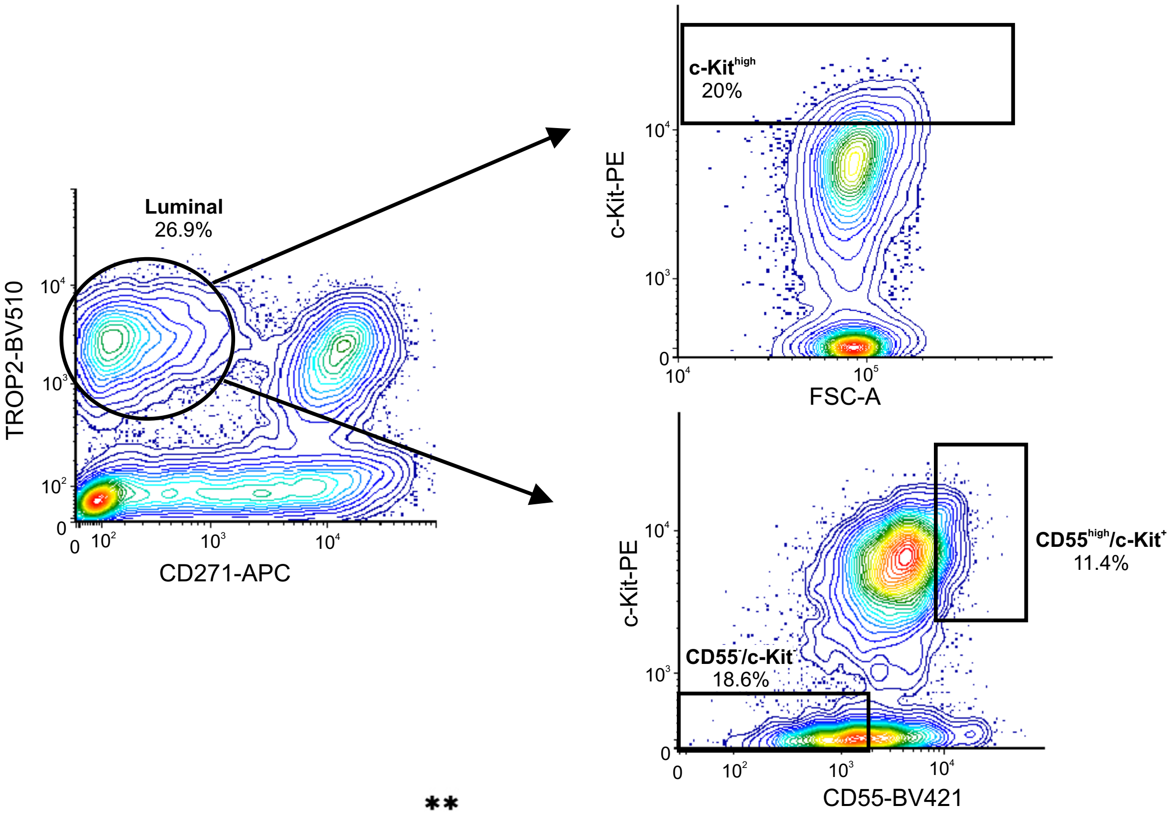

b

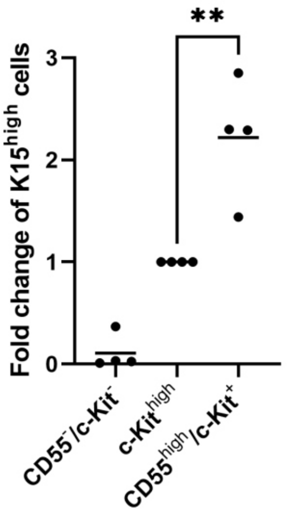

c

Supplementary Figure 3 continued

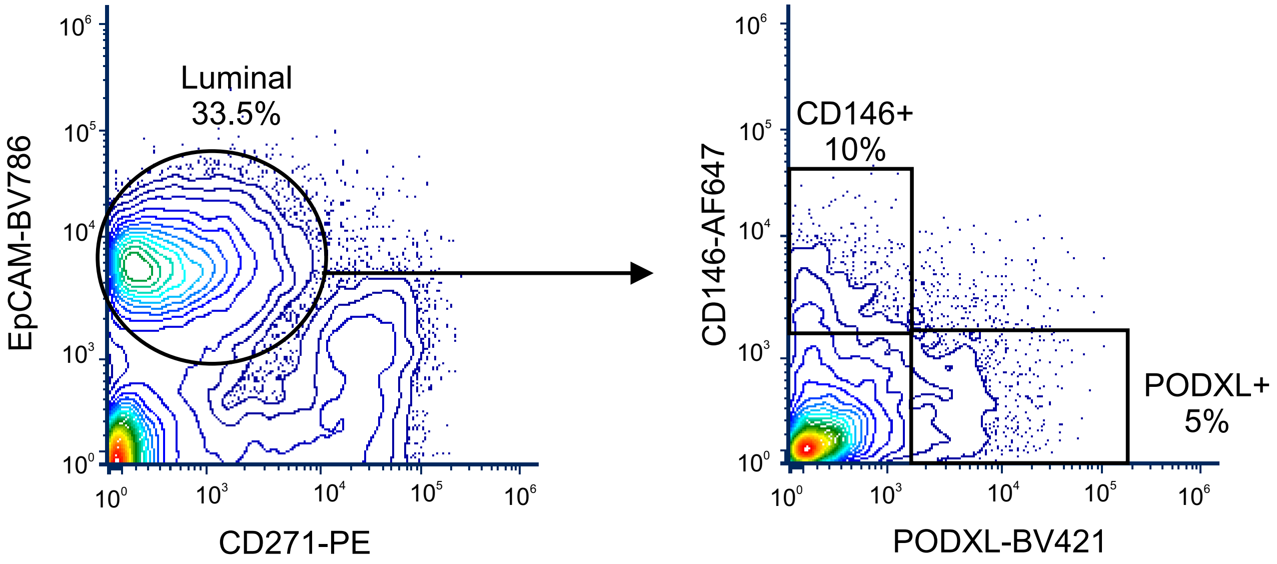

d

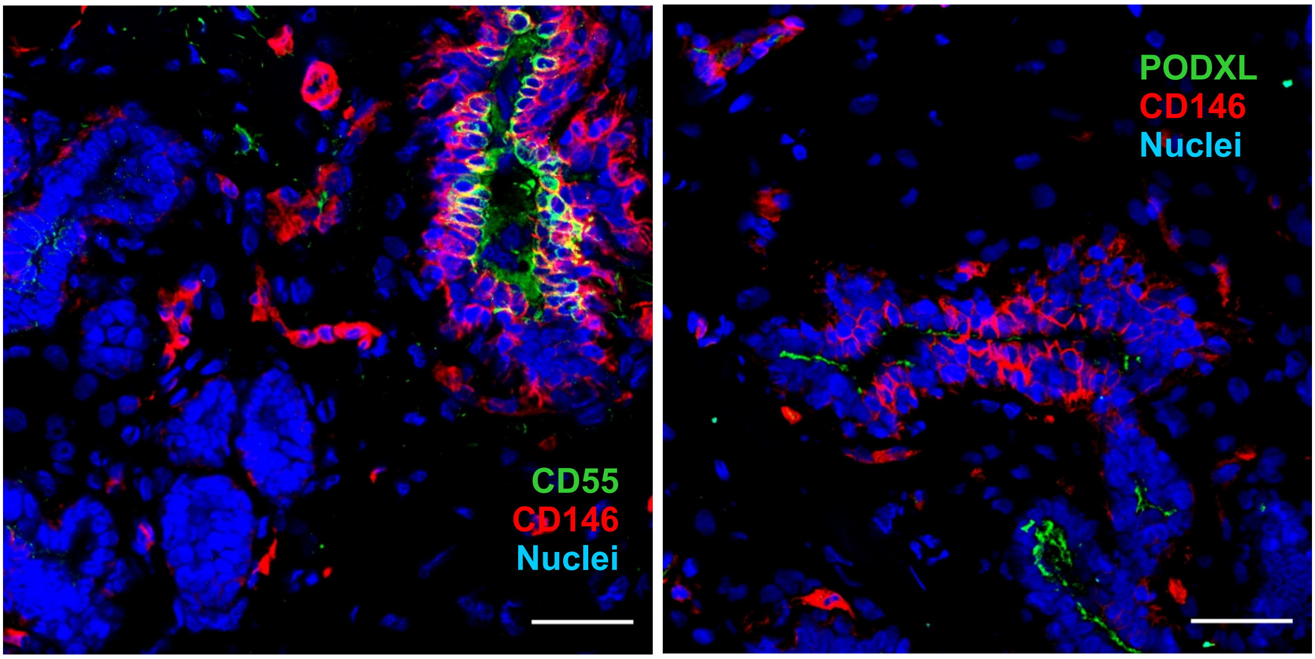

e

Supplementary Figure 3 continued

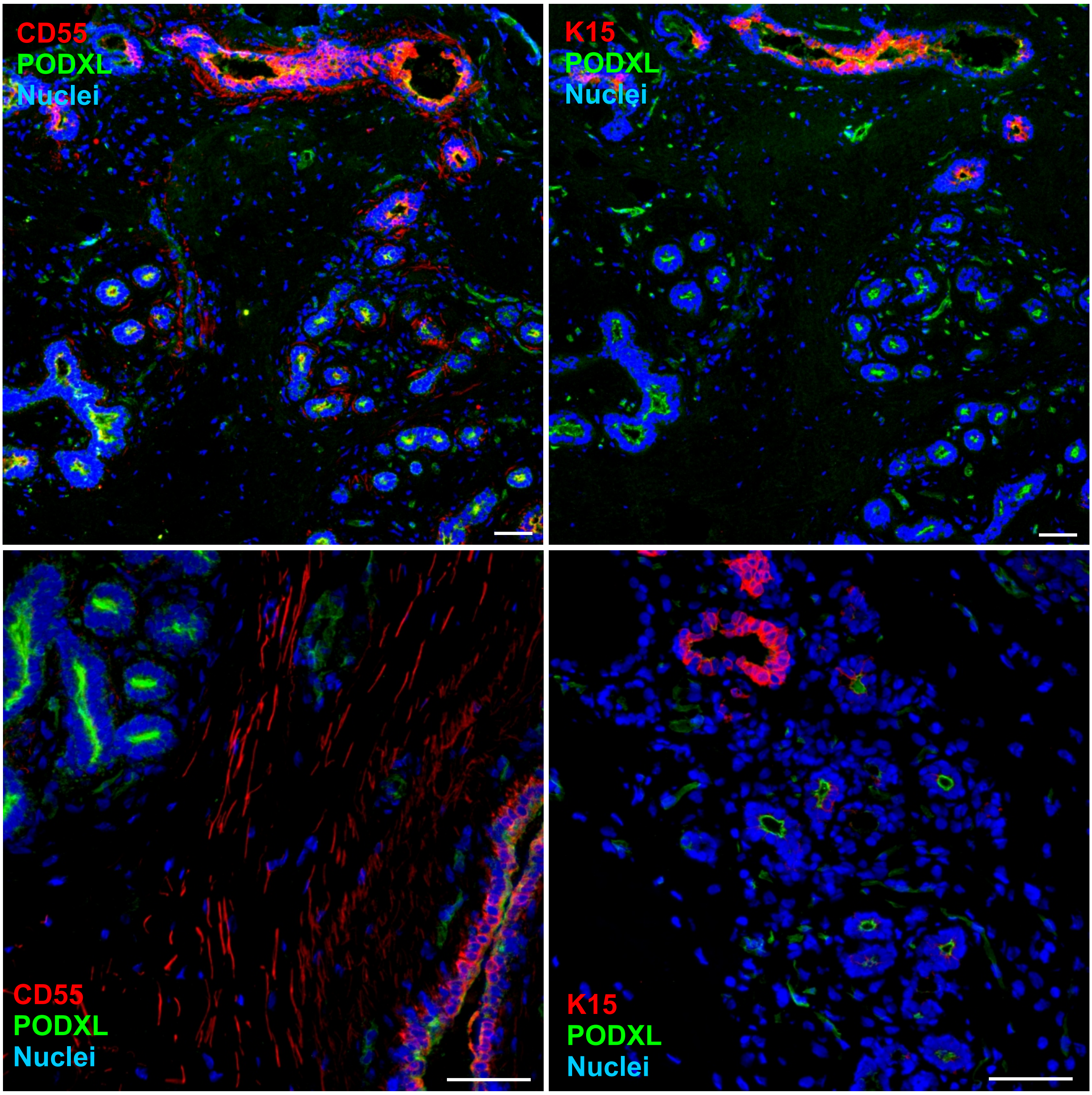

f

Supplementary Figure 3. CD55 co-stains $CD146^+$ ductal luminal cells, while PODXL stains a different luminal cell population *in situ*. (a) Representative multicolor imaging of cryostat sections of normal human breast stained for SLC34A2 (green), c-Kit (red), and nuclei (blue) shows overlap of markers (left). A high magnification image of a ductal area shows co-staining of SLC34A2 and c-Kit (right). Scale bars = 50 µm. (b) FACS graphs of the distribution of $CD55^+$ and $c\text{-}Kit^+$ luminal cells. $TROP2^+/CD271^-$ luminal cells (left) are gated for sorting $CD55^-/c\text{-}Kit^-$ and $CD55^{high}/c\text{-}Kit^+$ (lower right) or $c\text{-}Kit^{high}$ (upper right) cells. (c) Fold difference of strongly $K15^+$ ($K15^{high}$) cells determined by staining smears of sorted cells from (b) in each gate compared to the $c\text{-}Kit^{high}$ gate. Note that the $CD55^{high}/c\text{-}Kit^+$ population significantly enriches for cells that are strongly positive for K15 compared to the other populations. Lines represent mean values. The difference between $c\text{-}Kit^{high}$ and $CD55^{high}/c\text{-}Kit^+$ is indicated in the graph, $^{**}p < 0.01$ by ordinary one-way ANOVA with Tukey's multiple comparison test (n = 4 biopsies). Source data are provided as a Source Data file. (d) FACS graphs of the distribution of $PODXL^+$ and $CD146^+$ luminal cells. $EpCAM^+/CD271^-$ luminal cells (left) are gated for $CD146^+$ and $PODXL^+$ populations of cells (right). This shows that CD146 and PODXL expressions tend not to overlap. (e) An example of fluorescence imaging of cryostat sections of normal human breast stained for CD55 (green), CD146 (red) and nuclei (blue) (left) and PODXL (green), CD146 (red) and nuclei (blue) (right). Luminal CD146 staining overlaps with CD55 in ducts and is often mutually exclusive to PODXL. Scale bars = 50 µm. (f) Representative multicolor imaging of cryostat sections of normal human breast stained for CD55 or K15 (red), PODXL (green) and nuclei (blue). Note that CD55 also stains stromal cells. Both CD55 and K15 are preferentially located in ducts, while PODXL is strongly expressed in TDLUs. Scale bars = 50 µm.

Supplementary Figure 4

a

b

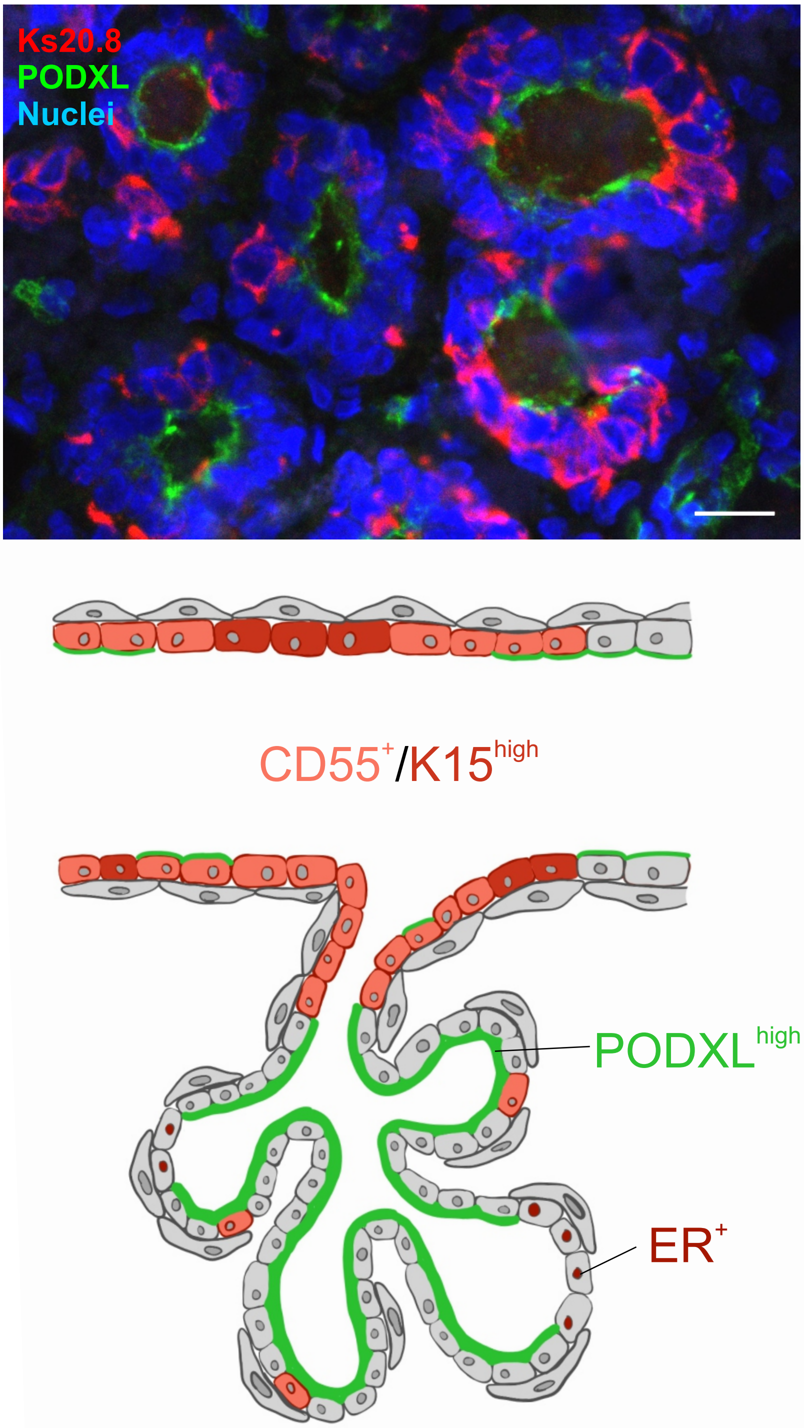

Supplementary Figure 4. PODXL stains a subpopulation in TDLUs that are negative for Ks20.8. (a) Representative multicolor imaging of normal human breast cryosections stained for PODXL (green), a surrogate marker of endocrine receptor-positive cells, Ks20.8 (red), and nuclei (blue). While strong PODXL staining is seen in TDLUs, endocrine receptor-positive cells tend to be negative for PODXL. Scale bar = 20 μm. (b) Schematic example of the spatial distribution of $CD55^+/K15^{high}$, $PODXL^{high}$ and estrogen receptor $(ER)^+$ cells in the human breast. While $CD55^+$ and $K15^{high}$ cells are co-localized in ducts, $PODXL^{high}$ cells are preferentially located in TDLUs with little overlap with endocrine receptor-positive cells.

Supplementary Figure 5

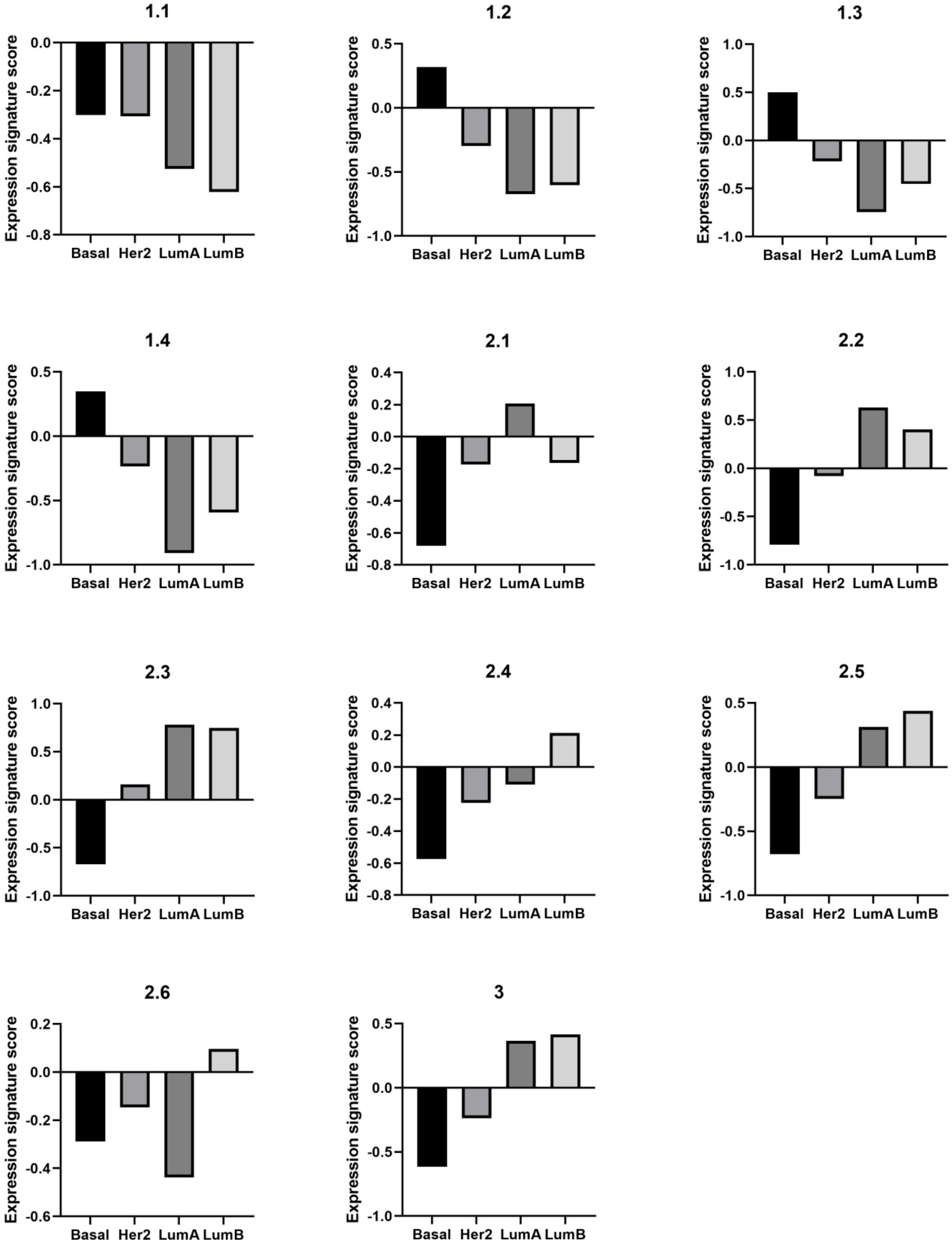

Supplementary Figure 5. Clusters 1.2-1.4 are closely related to SCSubtype defined basal-like breast cancer. Bar graphs showing expression signature scores of luminal clusters with gene signatures of single-cell derived molecular subtypes (basal-like (Basal), HER2-enriched (Her2), luminal type B (LumB), and luminal type A (LumA). Clusters 1.2-1.4 show a positive correlation with Basal, while clusters 2.1-3 are more closely related to the LumA, LumB and Her2. Source data are provided as a Source Data file.

Supplementary Figure 6

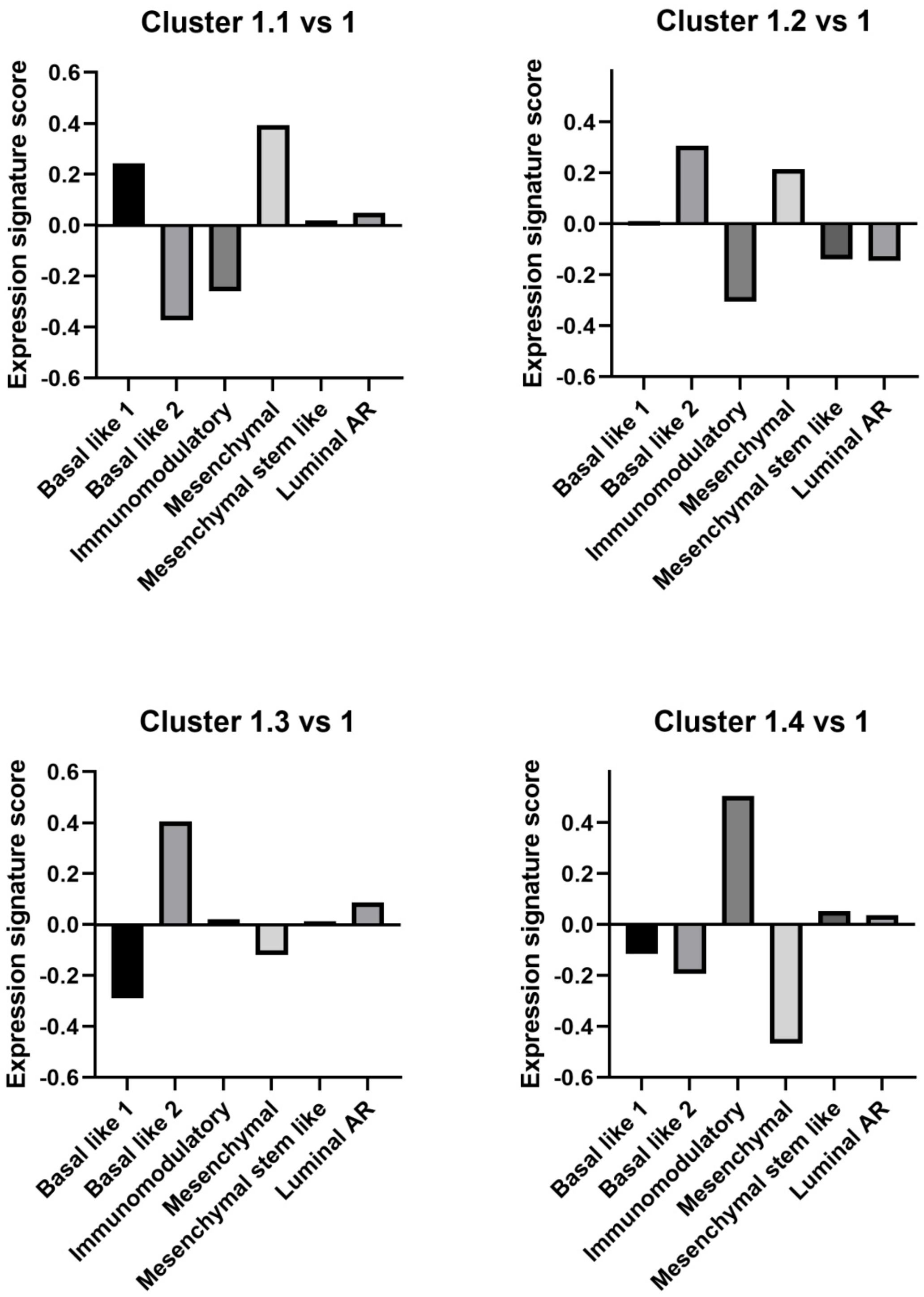

Supplementary Figure 6. Clusters 1.1-1.4 relate to different TNBC subtypes. Expression signature scores, indicating the similarity between DEGs of clusters 1.1-1.4 and gene signatures of TNBC subtypes, shown in bar graphs. Cluster 1.1 has a positive correlation with basal-like 1 and mesenchymal subtype, cluster 1.2 is most similar to basal-like 2 and the mesenchymal subtype, while cluster 1.3 has a positive correlation to the basal-like 2 subtype and cluster 1.4 is closely related to the immunomodulatory subtype of TNBC. Source data are provided as a Source Data file.

Supplementary Table 1

Cluster	PAM50 based Subtype
1.1	Basal
1.2	Basal
1.3	Basal
1.4	Basal
2.1	Her2
2.2	LumA
2.3	LumB
2.4	Lum B
2.5	LumA
2.6	LumB
3	LumA

Supplementary Table 1. Classification of clusters based on PAM50 subtype gene signature.

Supplementary Table 2

Antibody	Clone	Company	Cat. No.	Dilution IHC	Dilution FACS	Fixation IHC
ANXA1	EPR19342	Abcam	ab214486	1:10,000	-	formaldehyde
BV421 anti mouse IgM	RMM-1	BioLegend	406532	-	1:20	-
CD55	MEM-118	Nordic BioSite	AFC-1OBPQ-0.1	1:50	1:100	methanol
CD146	EPR3208	Abcam	ab75769	1:50	-	formaldehyde
CD146	P1H12	Abcam	ab24577	1:100	-	methanol
CD146-AF647	P1H12	BD Biosciences	56619	-	1:20	-
CD271-APC	ME20.4	Cedarlane Laboratories	CL10013APC	-	1:50	-
CD271-PE	ME20.4	BioLegend	345106	-	1:50	-
c-Kit	K45	Epredia	MS-289-P1	1:50	-	formaldehyde / methanol
c-Kit-BV421, c-Kit-PE	104D2	BD Biosciences	563856, 332785	-	1:20	-
EpCAM-BV785	9C4	BioLegend	324238	-	1:50	-
K5	XM26	Novocastra	NCL-CK5	1:250	-	methanol
K14	LL002	Nordic BioSite	MonX10687	1:50	-	formaldehyde
K15	LHK15	Abcam	ab80522	1:500	-	formaldehyde / methanol
K15	LHK15	Neomarkers	MS-1068-P0	1:100	-	formaldehyde
K17	E3	Dako	M7046	1:100	-	methanol
K20	Ks20.8	Dako	M7019	1:10	-	formaldehyde
PODXL		Invitrogen	433140	-	1:20	-
PODXL	3D3	Invitrogen	39-3800	1:50	-	formaldehyde
SLC34A2		Novus Biologicals	NBP1-81013	1:200	-	formaldehyde
TROP2-BV421, TROP2-BV510	162-46	BD Biosciences	563243, 563244	-	1:50	-

Supplementary Table 2. List of antibodies used for immunohisto-/immunocytochemistry and FACS.
