# Supplementary material for: Ductal keratin 15+ luminal progenitors in normal breast exhibit a basal-like breast cancer transcriptomic signature
Source: NPJ Breast Cancer. 2022 Jul 12;8:81. doi: 10.1038/s41523-022-00444-8 (PMC9276673; doi:10.1038/s41523-022-00444-8)
Supplement: Supplementary file 3 — Dataset1 [file 41523_2022_444_MOESM3_ESM.pdf]

Supplementary Data 1.

DEGs among clusters. Data include all cluster specific gene markers analyzed with Seurat.
DEGs are identified using a Wilcoxon Rank Sum test.
The gene list was further filtered using a threshold of 0.05 for the adjusted p-value, and 0.1 for the average log2(FC).

Abbreviations:
- p_val = p-value
- avg_log2FC = average log2(fold change)
- pct.1 = percentage of cells in which the gene is detected in a given cluster
- pct.2 = percentage of cell in which the gene is detected in the rest of cells
- p_val_adj = adjusted p-value

	p_val	avg_log2FC	pct.1	pct.2	p_val_adj	cluster	gene	pct.1/pct.2
UBC	0	2.193931057	0.988	0.887	0	1.1	UBC	1.113866967
CYR61	0	2.130701775	0.546	0.235	0	1.1	CYR61	2.323404255
KRT15	0	2.018226939	0.601	0.113	0	1.1	KRT15	5.318584071
OSER1	0	1.94396573	0.857	0.446	0	1.1	OSER1	1.921524664
PDE4B	0	1.895859847	0.931	0.463	0	1.1	PDE4B	2.010799136
RNF145	0	1.84864534	0.744	0.284	0	1.1	RNF145	2.61971831
CRYAB	0	1.770366085	0.647	0.206	0	1.1	CRYAB	3.140776699
OSBPL9	0	1.596732585	0.712	0.331	0	1.1	OSBPL9	2.151057402
PIGR	0	1.552539684	0.559	0.208	0	1.1	PIGR	2.6875
LRRFIP2	0	1.535040934	0.94	0.739	0	1.1	LRRFIP2	1.271989175
ARHGEF28	0	1.518382005	0.551	0.238	0	1.1	ARHGEF28	2.31512605
COBL	0	1.465949764	0.672	0.297	0	1.1	COBL	2.262626263
HNRNPA1	0	1.454192372	0.956	0.733	0	1.1	HNRNPA1	1.304229195
RASA2	0	1.394212928	0.792	0.507	0	1.1	RASA2	1.562130178
IGF2BP2	0	1.385138887	0.563	0.234	0	1.1	IGF2BP2	2.405982906
CALD1	0	1.378453878	0.633	0.254	0	1.1	CALD1	2.492125984
LHFPL2	0	1.355453858	0.597	0.254	0	1.1	LHFPL2	2.350393701
DAPK2	0	1.346307997	0.527	0.195	0	1.1	DAPK2	2.702564103
NPM1	0	1.333745619	0.938	0.685	0	1.1	NPM1	1.369343066
PSME4	0	1.315027537	0.846	0.614	0	1.1	PSME4	1.377850163
ANXA1	0	1.281223808	0.889	0.573	0	1.1	ANXA1	1.551483421
GBP1	0	1.264341253	0.336	0.07	0	1.1	GBP1	4.8
EEF1B2	0	1.228496461	0.897	0.707	0	1.1	EEF1B2	1.26874116
EIF3E	0	1.217793521	0.842	0.54	0	1.1	EIF3E	1.559259259
RPSA	0	1.141024565	0.932	0.794	0	1.1	RPSA	1.173803526
RPS23	0	1.11042398	0.994	0.965	0	1.1	RPS23	1.030051813
RPL11	0	1.076003953	0.991	0.955	0	1.1	RPL11	1.037696335
RPL35A	0	1.057113847	0.992	0.95	0	1.1	RPL35A	1.044210526
RPL14	0	1.056370197	0.99	0.957	0	1.1	RPL14	1.034482759
RPS27A	0	1.052317655	0.994	0.968	0	1.1	RPS27A	1.026859504
RPL32	0	1.043693378	0.995	0.973	0	1.1	RPL32	1.022610483
HSP90AB1	0	1.034165252	0.96	0.823	0	1.1	HSP90AB1	1.166464156
RPL30	0	1.028284072	0.99	0.949	0	1.1	RPL30	1.043203372
TOMM7	0	1.01872447	0.929	0.802	0	1.1	TOMM7	1.158354115
BTF3	0	1.015738963	0.957	0.88	0	1.1	BTF3	1.0875
RPL4	0	1.014541284	0.967	0.881	0	1.1	RPL4	1.097616345
RPL21	0	1.013291055	0.976	0.856	0	1.1	RPL21	1.140186916
NACA	0	1.010628493	0.979	0.9	0	1.1	NACA	1.087777778
RPL22	0	0.994398065	0.843	0.627	0	1.1	RPL22	1.344497608
RPL5	0	0.979853125	0.986	0.935	0	1.1	RPL5	1.054545455
RPS3A	0	0.972970575	0.972	0.889	0	1.1	RPS3A	1.09336333
RPS6	0	0.965404863	0.994	0.976	0	1.1	RPS6	1.018442623
PLEKHA6	0	0.962801916	0.942	0.818	0	1.1	PLEKHA6	1.151589242
ROPN1B	0	0.9523142	0.281	0.033	0	1.1	ROPN1B	8.515151515
RPS15A	0	0.941784928	0.984	0.936	0	1.1	RPS15A	1.051282051
SLC47A1	0	0.94152737	0.972	0.886	0	1.1	SLC47A1	1.097065463
RPS4X	0	0.928729225	0.994	0.977	0	1.1	RPS4X	1.017400205
RPL31	0	0.927400994	0.983	0.917	0	1.1	RPL31	1.071973828
EEF1A1	0	0.925503036	0.993	0.972	0	1.1	EEF1A1	1.021604938
APOO	0	0.899479458	0.991	0.963	0	1.1	APOO	1.029075805
RPL34	0	0.859159823	0.996	0.976	0	1.1	RPL34	1.020491803

RPS7	0	0.858672528	0.976	0.908	0	1.1	RPS7	1.074889868
RPL7A	0	0.853200518	0.986	0.954	0	1.1	RPL7A	1.033542977
RPS24	0	0.818347149	0.993	0.963	0	1.1	RPS24	1.031152648
SIK3	0	0.805219562	0.99	0.97	0	1.1	SIK3	1.020618557
ZFAS1	0	0.785519621	0.979	0.922	0	1.1	ZFAS1	1.061822126
OOEP	0	0.737709125	0.994	0.962	0	1.1	OOEP	1.033264033
RPS17	0	0.663649388	0.979	0.936	0	1.1	RPS17	1.045940171
PLEKHA7	0	0.594383	0.995	0.978	0	1.1	PLEKHA7	1.017382413
ROPN1	0	0.577585071	0.16	0.015	0	1.1	ROPN1	10.66666667
RSL1D1	1.58E-305	1.128740508	0.705	0.419	3.81E-301	1.1	RSL1D1	1.682577566
DHFR	1.96E-305	0.944544364	0.914	0.774	4.72E-301	1.1	DHFR	1.180878553
BAIAP2L1	5.22E-302	1.192626562	0.978	0.919	1.26E-297	1.1	BAIAP2L1	1.064200218
SRRM1	1.94E-301	1.067128723	0.766	0.506	4.67E-297	1.1	SRRM1	1.513833992
LDHB	8.52E-301	1.080168709	0.788	0.535	2.05E-296	1.1	LDHB	1.472897196
MLLT4	1.47E-299	1.087435417	0.845	0.619	3.54E-295	1.1	MLLT4	1.365105008
RPL24	1.16E-297	0.702375389	0.975	0.909	2.79E-293	1.1	RPL24	1.072607261
RPL7	6.59E-293	1.152381037	0.971	0.864	1.59E-288	1.1	RPL7	1.123842593
FAM13A	9.02E-289	1.497882968	0.524	0.23	2.17E-284	1.1	FAM13A	2.27826087
RIPK2	2.10E-288	1.267538461	0.55	0.245	5.07E-284	1.1	RIPK2	2.244897959
MAMDC2	1.49E-280	0.928207551	0.944	0.838	3.59E-276	1.1	MAMDC2	1.126491647
NFKBIZ	5.47E-273	1.024011235	0.796	0.528	1.32E-268	1.1	NFKBIZ	1.507575758
SRSF3	6.98E-270	1.035034705	0.703	0.397	1.68E-265	1.1	SRSF3	1.770780856
LRP6	1.25E-259	1.095285612	0.428	0.16	3.01E-255	1.1	LRP6	2.675
HSPA5	2.40E-258	1.368088504	0.703	0.418	5.80E-254	1.1	HSPA5	1.681818182
DDX21	4.80E-258	1.031633866	0.742	0.494	1.16E-253	1.1	DDX21	1.502024291
EIF3L	5.24E-252	0.919037601	0.792	0.563	1.26E-247	1.1	EIF3L	1.406749556
PFDN5	1.61E-250	0.764621358	0.912	0.837	3.89E-246	1.1	PFDN5	1.089605735
MAML2	5.35E-249	0.962374336	0.724	0.376	1.29E-244	1.1	MAML2	1.925531915
EIF4A2	7.52E-249	0.882594732	0.862	0.675	1.81E-244	1.1	EIF4A2	1.277037037
UQCRH	1.73E-246	0.900881625	0.863	0.734	4.16E-242	1.1	UQCRH	1.175749319
MECOM	1.04E-245	0.807750013	0.904	0.791	2.51E-241	1.1	MECOM	1.142857143
RPL27	2.28E-238	0.607186166	0.97	0.901	5.49E-234	1.1	RPL27	1.076581576
PDLIM5	7.43E-238	0.913024982	0.876	0.726	1.79E-233	1.1	PDLIM5	1.20661157
ILF2	2.62E-234	1.012209286	0.664	0.4	6.32E-230	1.1	ILF2	1.66
RPL41	7.70E-233	0.92474513	0.964	0.895	1.86E-228	1.1	RPL41	1.077094972
SEC24D	4.43E-231	1.048674725	0.423	0.165	1.07E-226	1.1	SEC24D	2.563636364
USP53	2.12E-228	1.223261342	0.798	0.601	5.12E-224	1.1	USP53	1.327787022
EIF2AK3	3.06E-228	1.169206472	0.603	0.332	7.37E-224	1.1	EIF2AK3	1.81626506
CXCR4	3.42E-227	1.355305737	0.449	0.188	8.25E-223	1.1	CXCR4	2.388297872
RPS25	3.85E-224	1.003316356	0.862	0.723	9.28E-220	1.1	RPS25	1.192254495
ATP5G2	2.65E-223	0.91923606	0.835	0.726	6.40E-219	1.1	ATP5G2	1.150137741
RP11-608O21.1	2.87E-222	0.938462359	0.747	0.511	6.93E-218	1.1	RP11-608O	1.46183953
ABI1	1.29E-215	0.901388603	0.831	0.659	3.10E-211	1.1	ABI1	1.261001517
ZMYM4	8.54E-213	0.781399888	0.893	0.806	2.06E-208	1.1	ZMYM4	1.107940447
S100B	1.49E-212	0.482134736	0.117	0.012	3.58E-208	1.1	S100B	9.75
KIAA0922	1.60E-212	1.369871091	0.409	0.17	3.87E-208	1.1	KIAA0922	2.405882353
CNKSR3	8.72E-209	1.003413261	0.554	0.276	2.10E-204	1.1	CNKSR3	2.007246377
TBC1D3P1-DHX40P1	6.67E-208	0.965940762	0.757	0.567	1.61E-203	1.1	TBC1D3P1-	1.335097002
RPL23	3.33E-201	0.542748409	0.968	0.909	8.02E-197	1.1	RPL23	1.064906491
RPS20	5.91E-201	0.913810581	0.962	0.919	1.43E-196	1.1	RPS20	1.046789989
RPL10	4.08E-198	1.170904181	0.977	0.93	9.83E-194	1.1	RPL10	1.050537634
LPIN1	1.09E-197	0.996681705	0.419	0.178	2.64E-193	1.1	LPIN1	2.353932584

METTL17	2.32E-197	0.524309755	0.148	0.024	5.58E-193	1.1	METTL17	6.166666667
TCF7L1	3.99E-195	0.9939512	0.39	0.157	9.63E-191	1.1	TCF7L1	2.484076433
TRIO	4.36E-195	0.82194139	0.905	0.797	1.05E-190	1.1	TRIO	1.135508156
ELF5	1.56E-194	0.855341859	0.336	0.118	3.76E-190	1.1	ELF5	2.847457627
RPS13	9.95E-193	1.074048007	0.829	0.687	2.40E-188	1.1	RPS13	1.206695779
CORO1C	5.70E-192	0.871493011	0.398	0.162	1.38E-187	1.1	CORO1C	2.456790123
RPS3	1.08E-191	1.272567063	0.927	0.861	2.62E-187	1.1	RPS3	1.076655052
RBM3	9.18E-189	0.837479932	0.627	0.379	2.21E-184	1.1	RBM3	1.654353562
DTNA	2.19E-187	0.803178523	0.882	0.742	5.29E-183	1.1	DTNA	1.188679245
SYNPO2	2.53E-187	0.902970348	0.268	0.079	6.11E-183	1.1	SYNPO2	3.392405063
GLIPR1	6.21E-183	0.966177533	0.389	0.158	1.50E-178	1.1	GLIPR1	2.462025316
NACA2	6.38E-182	0.952958735	0.662	0.443	1.54E-177	1.1	NACA2	1.494356659
SELK	1.45E-180	0.867304727	0.767	0.591	3.49E-176	1.1	SELK	1.297800338
RSL24D1	7.91E-180	0.737678981	0.754	0.534	1.91E-175	1.1	RSL24D1	1.411985019
UBA52	2.35E-178	0.556280052	0.957	0.914	5.66E-174	1.1	UBA52	1.047045952
FAM129A	6.15E-175	0.993413248	0.498	0.244	1.48E-170	1.1	FAM129A	2.040983607
RPS27	1.83E-174	0.923054897	0.79	0.617	4.42E-170	1.1	RPS27	1.280388979
EIF3K	1.16E-171	0.992176358	0.528	0.306	2.81E-167	1.1	EIF3K	1.725490196
EHF	2.27E-169	1.000460001	0.661	0.45	5.46E-165	1.1	EHF	1.468888889
GABRP	3.42E-168	0.748212357	0.496	0.235	8.26E-164	1.1	GABRP	2.110638298
TAF1D	5.62E-168	0.864964656	0.478	0.241	1.36E-163	1.1	TAF1D	1.98340249
HLA-DRA	1.78E-166	0.666789854	0.225	0.063	4.28E-162	1.1	HLA-DRA	3.571428571
BACH2	2.78E-166	1.032476921	0.549	0.309	6.71E-162	1.1	BACH2	1.776699029
GRB14	9.89E-165	0.961280386	0.613	0.383	2.38E-160	1.1	GRB14	1.600522193
PHLPP1	8.39E-164	1.035935434	0.586	0.376	2.02E-159	1.1	PHLPP1	1.558510638
ESD	1.02E-162	0.820287888	0.519	0.288	2.47E-158	1.1	ESD	1.802083333
WAC	2.01E-162	0.774518813	0.769	0.611	4.86E-158	1.1	WAC	1.258592471
TIMM9	4.35E-162	0.705120159	0.307	0.115	1.05E-157	1.1	TIMM9	2.669565217
ARRDC3	2.75E-160	0.989160683	0.596	0.357	6.64E-156	1.1	ARRDC3	1.669467787
ARHGAP17	4.55E-160	0.87144267	0.338	0.138	1.10E-155	1.1	ARHGAP17	2.449275362
SGMS2	5.47E-158	0.760031852	0.276	0.095	1.32E-153	1.1	SGMS2	2.905263158
RPL38	6.60E-157	0.518833235	0.935	0.886	1.59E-152	1.1	RPL38	1.05530474
RPL19	2.11E-156	0.911425843	0.935	0.897	5.10E-152	1.1	RPL19	1.042363434
EIF3M	7.86E-156	0.804104705	0.526	0.307	1.90E-151	1.1	EIF3M	1.713355049
NDUFS5	9.11E-156	0.618007805	0.866	0.76	2.20E-151	1.1	NDUFS5	1.139473684
EDN1	1.62E-154	0.86347031	0.299	0.112	3.90E-150	1.1	EDN1	2.669642857
SMS	2.60E-154	1.11867638	0.493	0.282	6.27E-150	1.1	SMS	1.74822695
HSP90B1	1.56E-153	0.826342867	0.684	0.485	3.77E-149	1.1	HSP90B1	1.410309278
DAAM1	1.65E-153	0.943153706	0.672	0.488	3.97E-149	1.1	DAAM1	1.37704918
LINC01198	4.61E-153	0.877604051	0.327	0.131	1.11E-148	1.1	LINC01198	2.496183206
RGS2	6.04E-153	1.499045536	0.287	0.108	1.46E-148	1.1	RGS2	2.657407407
RPL22L1	7.90E-153	0.80583772	0.615	0.397	1.90E-148	1.1	RPL22L1	1.549118388
RLF	1.56E-152	0.981318791	0.538	0.323	3.77E-148	1.1	RLF	1.665634675
GOLGA4	1.32E-151	0.739326719	0.764	0.602	3.19E-147	1.1	GOLGA4	1.26910299
ARL4C	1.75E-151	0.706449143	0.257	0.087	4.23E-147	1.1	ARL4C	2.954022989
MGP	1.48E-150	1.748451701	0.893	0.809	3.57E-146	1.1	MGP	1.103831891
HSP90AA1	2.15E-149	0.671847621	0.917	0.846	5.18E-145	1.1	HSP90AA1	1.08392435
HELZ	1.33E-148	0.826758645	0.457	0.241	3.21E-144	1.1	HELZ	1.89626556
NOCT	2.73E-147	0.723557093	0.255	0.088	6.58E-143	1.1	NOCT	2.897727273
TATDN1	3.03E-146	0.733157327	0.397	0.189	7.31E-142	1.1	TATDN1	2.100529101
SSR3	3.65E-146	0.82025105	0.629	0.421	8.81E-142	1.1	SSR3	1.494061758
RPL37	6.64E-146	0.481089993	0.987	0.94	1.60E-141	1.1	RPL37	1.05

EIF2A	1.10E-145	0.766618161	0.502	0.286	2.66E-141	1.1	EIF2A	1.755244755
C4orf3	1.33E-144	0.890968953	0.694	0.516	3.21E-140	1.1	C4orf3	1.34496124
SERAC1	1.55E-144	0.583004474	0.172	0.043	3.73E-140	1.1	SERAC1	4
MARCH5	2.95E-144	0.832903991	0.417	0.205	7.12E-140	1.1	MARCH5	2.034146341
HADH	4.78E-144	0.795328341	0.251	0.086	1.15E-139	1.1	HADH	2.918604651
IFRD1	1.68E-141	0.861996972	0.542	0.327	4.04E-137	1.1	IFRD1	1.657492355
BDNF-AS	2.40E-141	0.95043672	0.667	0.485	5.80E-137	1.1	BDNF-AS	1.375257732
RARRES3	2.87E-141	0.781772164	0.406	0.195	6.92E-137	1.1	RARRES3	2.082051282
CRIM1	3.73E-141	0.945677292	0.611	0.418	9.00E-137	1.1	CRIM1	1.461722488
ADAM9	1.68E-140	0.913973408	0.705	0.551	4.06E-136	1.1	ADAM9	1.279491833
RPL23A	1.76E-140	1.027624977	0.707	0.559	4.25E-136	1.1	RPL23A	1.264758497
CHCHD3	8.78E-139	0.619429874	0.897	0.829	2.12E-134	1.1	CHCHD3	1.082026538
SASH1	3.38E-137	0.745696081	0.327	0.137	8.14E-133	1.1	SASH1	2.386861314
NFATC2	7.04E-137	0.786538932	0.226	0.075	1.70E-132	1.1	NFATC2	3.013333333
AUH	1.58E-135	0.869025646	0.536	0.331	3.80E-131	1.1	AUH	1.619335347
RPL10A	4.73E-134	0.836827108	0.796	0.707	1.14E-129	1.1	RPL10A	1.125884017
PTPN14	5.20E-134	1.050936927	0.478	0.279	1.25E-129	1.1	PTPN14	1.713261649
PTN	9.94E-133	1.434841346	0.257	0.095	2.40E-128	1.1	PTN	2.705263158
MYO5B	3.96E-132	0.935463155	0.729	0.587	9.55E-128	1.1	MYO5B	1.241908007
HERC1	5.70E-132	0.916434098	0.487	0.291	1.37E-127	1.1	HERC1	1.673539519
CCL28	1.14E-131	0.436160759	0.609	0.334	2.76E-127	1.1	CCL28	1.823353293
CCNB1IP1	1.96E-130	0.721928567	0.431	0.23	4.73E-126	1.1	CCNB1IP1	1.873913043
DIAPH3	2.14E-129	0.653218013	0.195	0.058	5.16E-125	1.1	DIAPH3	3.362068966
NTRK2	3.32E-129	0.595435209	0.24	0.084	8.01E-125	1.1	NTRK2	2.857142857
SPINK5	4.40E-129	0.377964901	0.057	0.004	1.06E-124	1.1	SPINK5	14.25
DDIT3	7.86E-129	0.786402262	0.246	0.089	1.90E-124	1.1	DDIT3	2.764044944
NAV2	2.71E-127	1.029863729	0.577	0.395	6.54E-123	1.1	NAV2	1.460759494
RAD21	3.37E-127	0.782890024	0.512	0.314	8.13E-123	1.1	RAD21	1.630573248
KMT2C	8.86E-126	0.817010734	0.702	0.587	2.14E-121	1.1	KMT2C	1.195911414
FAM172A	9.55E-126	0.900959259	0.75	0.601	2.30E-121	1.1	FAM172A	1.247920133
DAPL1	2.24E-125	0.419581588	0.108	0.02	5.39E-121	1.1	DAPL1	5.4
UQCRC2	2.26E-125	0.724670707	0.505	0.308	5.44E-121	1.1	UQCRC2	1.63961039
LIPH	1.12E-124	1.055467475	0.487	0.287	2.69E-120	1.1	LIPH	1.696864111
BCAS3	1.71E-124	0.58593674	0.824	0.728	4.12E-120	1.1	BCAS3	1.131868132
RPL39	1.90E-124	0.555961877	0.963	0.89	4.58E-120	1.1	RPL39	1.082022472
SNRPD2	1.44E-120	0.674429204	0.761	0.662	3.47E-116	1.1	SNRPD2	1.149546828
COPS2	1.47E-120	0.673227724	0.433	0.236	3.54E-116	1.1	COPS2	1.834745763
NCALD	1.80E-118	0.840443456	0.269	0.11	4.35E-114	1.1	NCALD	2.445454545
LINC00152	4.08E-118	0.88777416	0.49	0.302	9.84E-114	1.1	LINC00152	1.622516556
EML4	1.06E-117	0.689137479	0.321	0.146	2.55E-113	1.1	EML4	2.198630137
WTAP	3.12E-117	1.118356774	0.66	0.495	7.52E-113	1.1	WTAP	1.333333333
PDIA6	3.55E-117	0.754072637	0.398	0.21	8.55E-113	1.1	PDIA6	1.895238095
RPS12	9.67E-117	1.164360037	0.717	0.641	2.33E-112	1.1	RPS12	1.118564743
GBP2	1.71E-116	0.748354224	0.75	0.597	4.12E-112	1.1	GBP2	1.256281407
CPEB3	9.44E-116	0.827408124	0.499	0.307	2.28E-111	1.1	CPEB3	1.625407166
AIMP1	2.82E-114	0.728398557	0.476	0.28	6.81E-110	1.1	AIMP1	1.7
RPLP0	5.04E-114	0.81214735	0.891	0.863	1.22E-109	1.1	RPLP0	1.032444959
AGFG1	8.95E-114	0.807184004	0.486	0.3	2.16E-109	1.1	AGFG1	1.62
HNRNPC	6.82E-113	0.5857762	0.866	0.773	1.64E-108	1.1	HNRNPC	1.120310479
EIF2B5	9.31E-113	0.925923069	0.595	0.445	2.25E-108	1.1	EIF2B5	1.337078652
TNFRSF11A	1.58E-110	0.570674532	0.172	0.053	3.82E-106	1.1	TNFRSF11A	3.245283019
RUSC2	4.29E-110	0.530518288	0.176	0.055	1.03E-105	1.1	RUSC2	3.2

DAPK1	5.94E-110	0.754920998	0.664	0.517	1.43E-105	1.1	DAPK1	1.284332689
PLIN2	2.71E-109	0.747938252	0.216	0.079	6.53E-105	1.1	PLIN2	2.734177215
CTC-471J1.9	1.18E-108	0.674334224	0.742	0.623	2.84E-104	1.1	CTC-471J1.	1.191011236
BTAF1	3.71E-107	0.704701342	0.376	0.198	8.96E-103	1.1	BTAF1	1.898989899
ZFAND1	1.48E-106	0.612871652	0.334	0.164	3.56E-102	1.1	ZFAND1	2.036585366
APEX1	4.92E-106	0.648869252	0.348	0.175	1.19E-101	1.1	APEX1	1.988571429
KLK5	9.48E-106	0.453109466	0.108	0.023	2.29E-101	1.1	KLK5	4.695652174
RP5-945F2.3	9.67E-106	0.772360816	0.128	0.032	2.33E-101	1.1	RP5-945F2.	4
RIOK3	8.95E-105	0.735595605	0.428	0.248	2.16E-100	1.1	RIOK3	1.725806452
FGFR1	1.30E-103	0.801570575	0.345	0.174	3.13E-99	1.1	FGFR1	1.982758621
SLC20A2	1.85E-102	0.762832234	0.489	0.305	4.46E-98	1.1	SLC20A2	1.603278689
LDHA	1.56E-101	0.809242946	0.761	0.656	3.77E-97	1.1	LDHA	1.160060976
CAMK1D	4.72E-100	0.695253816	0.252	0.107	1.14E-95	1.1	CAMK1D	2.355140187
MIR222HG	6.34E-100	0.515345491	0.15	0.045	1.53E-95	1.1	MIR222HG	3.333333333
QKI	9.95E-100	0.757897164	0.655	0.526	2.40E-95	1.1	QKI	1.245247148
STEAP1B	3.81E-99	0.727868026	0.975	0.928	9.18E-95	1.1	STEAP1B	1.050646552
BCL2L14	7.85E-99	0.538346302	0.226	0.089	1.89E-94	1.1	BCL2L14	2.539325843
CASC8	8.84E-99	0.333396564	0.128	0.034	2.13E-94	1.1	CASC8	3.764705882
MKL1	8.67E-97	0.841328413	0.623	0.482	2.09E-92	1.1	MKL1	1.29253112
SERTAD2	1.26E-96	0.686380999	0.305	0.148	3.05E-92	1.1	SERTAD2	2.060810811
CBX1	7.26E-96	0.575254429	0.232	0.096	1.75E-91	1.1	CBX1	2.416666667
BBOX1	1.47E-94	0.730571761	0.251	0.108	3.54E-90	1.1	BBOX1	2.324074074
CTSV	3.20E-93	1.079968157	0.235	0.099	7.73E-89	1.1	CTSV	2.373737374
RPL37A	5.63E-93	0.380297048	0.995	0.972	1.36E-88	1.1	RPL37A	1.023662551
DENND5A	7.88E-92	0.679902942	0.494	0.32	1.90E-87	1.1	DENND5A	1.54375
FXR1	6.51E-91	0.632548138	0.501	0.331	1.57E-86	1.1	FXR1	1.513595166
RANBP2	7.97E-91	0.636881832	0.366	0.202	1.92E-86	1.1	RANBP2	1.811881188
SNRPD1	4.75E-90	0.641714592	0.434	0.267	1.15E-85	1.1	SNRPD1	1.625468165
TGFBR3	2.26E-89	0.616098399	0.212	0.085	5.46E-85	1.1	TGFBR3	2.494117647
TRIM2	3.80E-89	0.629263905	0.307	0.154	9.15E-85	1.1	TRIM2	1.993506494
BARX2	4.18E-89	0.739500262	0.401	0.231	1.01E-84	1.1	BARX2	1.735930736
BIRC3	1.54E-88	0.746164948	0.738	0.596	3.71E-84	1.1	BIRC3	1.238255034
SSR2	2.67E-87	0.531512278	0.765	0.676	6.44E-83	1.1	SSR2	1.131656805
KIZ-AS1	2.84E-86	0.584043206	0.925	0.833	6.84E-82	1.1	KIZ-AS1	1.110444178
MT-ND4	1.34E-85	0.36355877	0.996	0.993	3.24E-81	1.1	MT-ND4	1.003021148
CRABP1	4.17E-85	0.121235167	0.04	0.003	1.01E-80	1.1	CRABP1	13.33333333
ALDH1A3	5.15E-85	0.533253298	0.439	0.255	1.24E-80	1.1	ALDH1A3	1.721568627
B2M	5.60E-85	0.236424362	0.995	0.968	1.35E-80	1.1	B2M	1.027892562
FNBP1L	7.22E-85	0.767009728	0.55	0.403	1.74E-80	1.1	FNBP1L	1.364764268
GJA1	7.82E-85	0.393806032	0.093	0.021	1.89E-80	1.1	GJA1	4.428571429
EIF3H	1.46E-84	0.519332186	0.719	0.613	3.51E-80	1.1	EIF3H	1.172920065
TEX14	1.77E-84	0.88701034	0.276	0.134	4.28E-80	1.1	TEX14	2.059701493
RP11-286E11.1	1.19E-83	0.369577839	0.103	0.026	2.88E-79	1.1	RP11-286E:	3.961538462
TM4SF1	5.76E-83	0.710879909	0.936	0.903	1.39E-78	1.1	TM4SF1	1.03654485
EPB41L2	1.46E-82	0.772676494	0.223	0.098	3.52E-78	1.1	EPB41L2	2.275510204
PDK1	1.92E-82	0.536854711	0.204	0.083	4.64E-78	1.1	PDK1	2.457831325
HK2	2.64E-82	0.592547159	0.298	0.151	6.37E-78	1.1	HK2	1.973509934
EIF4A1	5.19E-82	0.565585767	0.553	0.385	1.25E-77	1.1	EIF4A1	1.436363636
SOSTDC1	7.86E-82	0.252362251	0.053	0.007	1.90E-77	1.1	SOSTDC1	7.571428571
TGFB2	1.40E-81	0.664088168	0.325	0.172	3.36E-77	1.1	TGFB2	1.889534884
SLC9A7	2.76E-81	0.705169266	0.424	0.265	6.65E-77	1.1	SLC9A7	1.6
FLNB	3.42E-81	0.580837827	0.596	0.44	8.25E-77	1.1	FLNB	1.354545455

RP11-817J15.2	4.29E-81	0.407967667	0.133	0.042	1.03E-76	1.1	RP11-817J1	3.166666667
GSTA1	5.85E-81	0.714903537	0.115	0.032	1.41E-76	1.1	GSTA1	3.59375
SNX18	6.99E-81	0.317694529	0.11	0.03	1.68E-76	1.1	SNX18	3.666666667
KDM5B	2.82E-80	0.638246885	0.635	0.534	6.79E-76	1.1	KDM5B	1.189138577
RAD23B	5.42E-80	0.626739336	0.611	0.469	1.31E-75	1.1	RAD23B	1.302771855
SKP1	6.26E-80	0.477897724	0.874	0.798	1.51E-75	1.1	SKP1	1.095238095
IGFBP3	1.01E-79	0.571954088	0.143	0.047	2.43E-75	1.1	IGFBP3	3.042553191
AMBRA1	1.13E-79	0.632092758	0.737	0.67	2.71E-75	1.1	AMBRA1	1.1
PFDN4	1.17E-79	0.585369583	0.48	0.318	2.83E-75	1.1	PFDN4	1.509433962
EPB42	7.75E-79	0.65874273	0.961	0.887	1.87E-74	1.1	EPB42	1.083427283
DMD	8.47E-78	0.654277724	0.404	0.242	2.04E-73	1.1	DMD	1.669421488
IL22RA2	1.64E-77	0.152778029	0.043	0.005	3.96E-73	1.1	IL22RA2	8.6
RPL6	1.67E-77	0.441482976	0.966	0.947	4.02E-73	1.1	RPL6	1.020063358
CRY1	3.64E-77	0.696480962	0.523	0.369	8.78E-73	1.1	CRY1	1.417344173
CLDN1	4.12E-77	0.76381677	0.367	0.21	9.93E-73	1.1	CLDN1	1.747619048
MAP2K6	6.44E-77	0.227720427	0.064	0.011	1.55E-72	1.1	MAP2K6	5.818181818
ETV6	9.65E-77	0.591792243	0.684	0.564	2.33E-72	1.1	ETV6	1.212765957
RP11-244M2.1	4.91E-76	0.596233918	0.535	0.394	1.18E-71	1.1	RP11-244M	1.35786802
ITGB8	1.33E-75	0.504582047	0.654	0.477	3.21E-71	1.1	ITGB8	1.371069182
SH3GL1	2.59E-75	0.432481875	0.155	0.057	6.25E-71	1.1	SH3GL1	2.719298246
FAS	9.85E-75	0.505870817	0.201	0.086	2.37E-70	1.1	FAS	2.337209302
PIK3C2G	1.80E-74	0.423659338	0.15	0.053	4.33E-70	1.1	PIK3C2G	2.830188679
NDRG1	1.95E-74	0.962926895	0.475	0.336	4.71E-70	1.1	NDRG1	1.413690476
CXCL3	2.13E-73	0.624477003	0.143	0.05	5.13E-69	1.1	CXCL3	2.86
PDK3	2.99E-73	0.756893025	0.358	0.213	7.21E-69	1.1	PDK3	1.680751174
GCNT1	8.05E-73	0.502154546	0.136	0.047	1.94E-68	1.1	GCNT1	2.893617021
PTPRJ	2.03E-72	0.661822803	0.438	0.291	4.89E-68	1.1	PTPRJ	1.505154639
TSC22D2	6.17E-72	0.42064462	0.614	0.449	1.49E-67	1.1	TSC22D2	1.367483296
FBL	1.21E-71	0.437073919	0.248	0.12	2.92E-67	1.1	FBL	2.066666667
IGF2BP2-AS1	1.68E-71	0.272591388	0.074	0.016	4.05E-67	1.1	IGF2BP2-AS	4.625
CCDC6	8.34E-71	0.652747624	0.449	0.302	2.01E-66	1.1	CCDC6	1.486754967
ATP13A3	3.21E-70	0.685147907	0.387	0.246	7.73E-66	1.1	ATP13A3	1.573170732
HILPDA	5.49E-70	0.408922313	0.428	0.26	1.32E-65	1.1	HILPDA	1.646153846
ENAH	8.23E-70	0.619757792	0.655	0.541	1.99E-65	1.1	ENAH	1.210720887
EMP1	1.03E-69	1.163682762	0.696	0.687	2.48E-65	1.1	EMP1	1.013100437
PPP1CB	1.51E-69	0.639186743	0.478	0.339	3.65E-65	1.1	PPP1CB	1.410029499
MTRNR2L8	2.12E-69	0.469165455	0.254	0.126	5.11E-65	1.1	MTRNR2L8	2.015873016
HCAR2	2.81E-69	0.811637307	0.202	0.091	6.78E-65	1.1	HCAR2	2.21978022
UQCRHL	9.28E-69	0.510594464	0.302	0.168	2.24E-64	1.1	UQCRHL	1.797619048
RP11-511B23.2	1.69E-68	0.513361987	0.617	0.494	4.08E-64	1.1	RP11-511B	1.248987854
EIF4B	1.05E-67	0.523925341	0.511	0.365	2.52E-63	1.1	EIF4B	1.4
CCT2	1.93E-66	0.533506083	0.435	0.286	4.66E-62	1.1	CCT2	1.520979021
RPF2	4.82E-66	0.476540241	0.243	0.121	1.16E-61	1.1	RPF2	2.008264463
GALNT1	1.89E-65	0.505802641	0.206	0.096	4.56E-61	1.1	GALNT1	2.145833333
NAA25	2.73E-65	0.564991442	0.484	0.335	6.59E-61	1.1	NAA25	1.444776119
RPS11	4.02E-65	0.7017662	0.78	0.769	9.69E-61	1.1	RPS11	1.014304291
SAR1B	5.34E-65	0.666808858	0.496	0.357	1.29E-60	1.1	SAR1B	1.389355742
RP5-896L10.1	8.67E-65	0.491938731	0.355	0.214	2.09E-60	1.1	RP5-896L1(	1.658878505
RSRC2	2.27E-64	0.546660352	0.708	0.615	5.47E-60	1.1	RSRC2	1.151219512
ARID5B	2.68E-64	0.519990196	0.791	0.727	6.46E-60	1.1	ARID5B	1.088033012
LIMS1	4.35E-64	0.752305711	0.359	0.229	1.05E-59	1.1	LIMS1	1.56768559
NUP153	6.52E-64	0.557789183	0.414	0.267	1.57E-59	1.1	NUP153	1.550561798

USP54	8.37E-64	0.631200938	0.607	0.483	2.02E-59	1.1	USP54	1.256728778
SESTD1	1.38E-63	0.544120615	0.603	0.474	3.32E-59	1.1	SESTD1	1.272151899
GGA2	1.54E-63	0.457810356	0.202	0.094	3.72E-59	1.1	GGA2	2.14893617
SH3BP5	2.72E-63	0.331366124	0.129	0.046	6.55E-59	1.1	SH3BP5	2.804347826
MT-CO1	4.68E-63	0.236417338	0.996	0.991	1.13E-58	1.1	MT-CO1	1.005045409
TULP4	5.99E-63	0.683847228	0.59	0.491	1.44E-58	1.1	TULP4	1.201629328
KPNA1	9.42E-63	0.619354318	0.387	0.252	2.27E-58	1.1	KPNA1	1.535714286
PPP2CB	1.27E-62	0.554750816	0.464	0.324	3.05E-58	1.1	PPP2CB	1.432098765
MTRNR2L1	1.83E-62	0.416150379	0.216	0.102	4.41E-58	1.1	MTRNR2L1	2.117647059
ATP6V0A1	2.11E-62	0.485011945	0.247	0.126	5.09E-58	1.1	ATP6V0A1	1.96031746
HCFC2	2.27E-62	0.221591097	0.068	0.015	5.46E-58	1.1	HCFC2	4.533333333
PIM1	2.93E-62	0.196195186	0.057	0.011	7.06E-58	1.1	PIM1	5.181818182
CYP2U1	4.14E-62	0.157795371	0.051	0.009	9.99E-58	1.1	CYP2U1	5.666666667
ACTN4	5.72E-62	0.56229782	0.399	0.258	1.38E-57	1.1	ACTN4	1.546511628
TPT1-AS1	9.08E-62	0.520515552	0.358	0.219	2.19E-57	1.1	TPT1-AS1	1.634703196
PTEN	1.21E-61	0.594328887	0.862	0.801	2.92E-57	1.1	PTEN	1.076154806
SEC62	3.26E-61	0.540325794	0.679	0.566	7.85E-57	1.1	SEC62	1.199646643
C5orf46	4.05E-61	0.497640402	0.217	0.105	9.76E-57	1.1	C5orf46	2.066666667
RPS8	4.35E-61	0.499694364	0.758	0.736	1.05E-56	1.1	RPS8	1.029891304
NDUFA4	8.44E-61	0.432693984	0.807	0.742	2.03E-56	1.1	NDUFA4	1.087601078
ESYT2	1.22E-60	0.610461351	0.614	0.5	2.93E-56	1.1	ESYT2	1.228
HNRNPA3	8.33E-60	0.496000591	0.386	0.248	2.01E-55	1.1	HNRNPA3	1.556451613
STRN	4.41E-59	0.578954251	0.513	0.383	1.06E-54	1.1	STRN	1.339425587
RPL9	5.68E-59	0.480610895	0.668	0.557	1.37E-54	1.1	RPL9	1.199281867
DDX24	7.08E-59	0.557193542	0.607	0.494	1.71E-54	1.1	DDX24	1.228744939
PELI1	9.60E-59	0.639700068	0.447	0.314	2.31E-54	1.1	PELI1	1.423566879
ARF4	1.18E-58	0.481858485	0.702	0.609	2.85E-54	1.1	ARF4	1.15270936
EPHA4	5.39E-58	0.310209022	0.076	0.02	1.30E-53	1.1	EPHA4	3.8
TMEM123	7.60E-58	0.585838478	0.419	0.289	1.83E-53	1.1	TMEM123	1.44982699
RPL12	9.60E-58	0.537020192	0.406	0.272	2.31E-53	1.1	RPL12	1.492647059
IPO7	1.20E-57	0.511962642	0.371	0.238	2.90E-53	1.1	IPO7	1.558823529
CD83	1.25E-57	0.479773263	0.146	0.059	3.02E-53	1.1	CD83	2.474576271
HEY2	2.09E-57	0.20651543	0.058	0.012	5.03E-53	1.1	HEY2	4.833333333
PPP1R14C	2.40E-57	0.480379338	0.185	0.086	5.79E-53	1.1	PPP1R14C	2.151162791
IFFO2	3.46E-57	0.472812551	0.213	0.104	8.34E-53	1.1	IFFO2	2.048076923
SNRPE	3.51E-57	0.447253301	0.622	0.509	8.46E-53	1.1	SNRPE	1.222003929
CELF2	4.77E-57	0.453919667	0.221	0.112	1.15E-52	1.1	CELF2	1.973214286
RPS29	5.29E-57	0.334647477	0.976	0.918	1.28E-52	1.1	RPS29	1.063180828
PPTC7	9.14E-57	0.608447795	0.306	0.181	2.20E-52	1.1	PPTC7	1.690607735
UQCRB	1.18E-56	0.329536532	0.853	0.8	2.85E-52	1.1	UQCRB	1.06625
FAM168A	1.22E-56	0.412575394	0.277	0.155	2.93E-52	1.1	FAM168A	1.787096774
MICAL3	6.83E-56	0.598721617	0.349	0.221	1.65E-51	1.1	MICAL3	1.57918552
COL6A2	1.48E-55	0.476811966	0.142	0.058	3.57E-51	1.1	COL6A2	2.448275862
MRPS33	1.64E-55	0.500239396	0.354	0.226	3.95E-51	1.1	MRPS33	1.566371681
MT-CYB	1.78E-55	0.267038219	0.992	0.991	4.30E-51	1.1	MT-CYB	1.001009082
RSBN1L	1.91E-55	0.482743038	0.189	0.089	4.60E-51	1.1	RSBN1L	2.123595506
NCOA7	3.37E-54	0.487944331	0.573	0.424	8.13E-50	1.1	NCOA7	1.351415094
TGFBR2	7.76E-54	0.464806616	0.171	0.079	1.87E-49	1.1	TGFBR2	2.164556962
MEST	1.15E-53	0.268815615	0.078	0.022	2.77E-49	1.1	MEST	3.545454545
C11orf1	1.31E-53	0.418369211	0.231	0.123	3.16E-49	1.1	C11orf1	1.87804878
ZNF292	7.15E-53	0.504557836	0.652	0.554	1.72E-48	1.1	ZNF292	1.176895307
IQCG	7.88E-53	0.539883012	0.277	0.162	1.90E-48	1.1	IQCG	1.709876543

MT-ND4L	1.02E-52	0.484867765	0.554	0.442	2.45E-48	1.1	MT-ND4L	1.253393665
SVIL	3.81E-52	0.328308396	0.72	0.568	9.19E-48	1.1	SVIL	1.267605634
BACE2	7.10E-52	0.547563445	0.422	0.299	1.71E-47	1.1	BACE2	1.411371237
WWTR1	1.56E-51	0.587010693	0.453	0.333	3.76E-47	1.1	WWTR1	1.36036036
KCTD9	2.27E-51	0.660204076	0.339	0.221	5.47E-47	1.1	KCTD9	1.533936652
SEC24A	2.84E-51	0.543384011	0.276	0.163	6.84E-47	1.1	SEC24A	1.693251534
ADGRL3-AS1	3.20E-51	0.499836297	0.692	0.586	7.71E-47	1.1	ADGRL3-AS	1.180887372
CYCS	4.24E-51	0.479544272	0.624	0.508	1.02E-46	1.1	CYCS	1.228346457
SNHG8	4.65E-51	0.493018093	0.336	0.213	1.12E-46	1.1	SNHG8	1.577464789
MET	5.57E-51	0.715126535	0.326	0.214	1.34E-46	1.1	MET	1.523364486
MEAF6	6.34E-51	0.446484006	0.273	0.156	1.53E-46	1.1	MEAF6	1.75
RASGEF1C	2.19E-50	0.327274068	0.093	0.031	5.27E-46	1.1	RASGEF1C	3
ELF3	2.36E-50	0.636128514	0.605	0.503	5.70E-46	1.1	ELF3	1.2027833
MAP4K5	4.81E-50	0.618554631	0.391	0.273	1.16E-45	1.1	MAP4K5	1.432234432
CLINT1	5.02E-50	0.58212068	0.353	0.235	1.21E-45	1.1	CLINT1	1.50212766
FOXK2	5.37E-50	0.522247303	0.326	0.209	1.29E-45	1.1	FOXK2	1.559808612
PTRF	9.79E-50	0.412422228	0.206	0.106	2.36E-45	1.1	PTRF	1.943396226
MYC	1.18E-49	0.466590213	0.196	0.099	2.84E-45	1.1	MYC	1.97979798
PEG10	1.66E-49	0.29470272	0.088	0.029	4.01E-45	1.1	PEG10	3.034482759
FMO2	2.25E-49	0.299859158	0.125	0.05	5.43E-45	1.1	FMO2	2.5
FERMT2	2.92E-49	0.449816177	0.228	0.123	7.05E-45	1.1	FERMT2	1.853658537
ZNF652	4.12E-49	0.490183179	0.541	0.434	9.93E-45	1.1	ZNF652	1.246543779
DBI	7.06E-49	0.555473227	0.709	0.648	1.70E-44	1.1	DBI	1.094135802
BFAR	1.67E-48	0.402298331	0.236	0.131	4.03E-44	1.1	BFAR	1.801526718
UBB	1.89E-48	0.422759902	0.781	0.714	4.56E-44	1.1	UBB	1.093837535
FRMD6	2.17E-48	0.534232078	0.29	0.176	5.24E-44	1.1	FRMD6	1.647727273
RP11-386I14.4	2.39E-48	0.188234501	0.055	0.013	5.77E-44	1.1	RP11-386I1	4.230769231
UBAP1	2.74E-48	0.496484436	0.431	0.309	6.62E-44	1.1	UBAP1	1.394822006
RP5-968J1.1	4.41E-48	0.145075412	0.047	0.01	1.06E-43	1.1	RP5-968J1.	4.7
NSA2	1.45E-47	0.467122895	0.451	0.333	3.50E-43	1.1	NSA2	1.354354354
KCMF1	2.29E-47	0.54300567	0.441	0.325	5.53E-43	1.1	KCMF1	1.356923077
TLE4	3.77E-47	0.569641302	0.442	0.327	9.08E-43	1.1	TLE4	1.351681957
CHIC2	8.04E-47	0.550949019	0.253	0.149	1.94E-42	1.1	CHIC2	1.697986577
SAP18	2.00E-46	0.388872869	0.652	0.562	4.82E-42	1.1	SAP18	1.160142349
NFAT5	8.32E-46	0.361680483	0.705	0.62	2.01E-41	1.1	NFAT5	1.137096774
SEC24B	8.43E-46	0.476434532	0.348	0.231	2.03E-41	1.1	SEC24B	1.506493506
SAMD5	1.76E-45	0.222485822	0.079	0.025	4.24E-41	1.1	SAMD5	3.16
KCTD3	2.82E-45	0.498668648	0.293	0.183	6.80E-41	1.1	KCTD3	1.601092896
RIF1	4.92E-45	0.481642219	0.327	0.213	1.19E-40	1.1	RIF1	1.535211268
CTA-293F17.1	1.03E-44	0.439380229	0.139	0.063	2.48E-40	1.1	CTA-293F1	2.206349206
AKT3	1.52E-44	0.803013643	0.432	0.336	3.67E-40	1.1	AKT3	1.285714286
PTBP2	1.55E-44	0.533441419	0.441	0.332	3.75E-40	1.1	PTBP2	1.328313253
NPEPPS	2.10E-44	0.579466086	0.576	0.486	5.07E-40	1.1	NPEPPS	1.185185185
ELL2	2.52E-44	0.46061667	0.757	0.689	6.09E-40	1.1	ELL2	1.098693759
KLF5	2.64E-44	0.450501829	0.259	0.153	6.37E-40	1.1	KLF5	1.692810458
TIAM1	3.51E-44	0.698201903	0.339	0.231	8.45E-40	1.1	TIAM1	1.467532468
THBS1	4.52E-44	0.455035653	0.151	0.07	1.09E-39	1.1	THBS1	2.157142857
FNBP1	4.78E-44	0.512005285	0.606	0.518	1.15E-39	1.1	FNBP1	1.16988417
IFIT2	6.33E-44	0.462584444	0.072	0.022	1.53E-39	1.1	IFIT2	3.272727273
RNMT	8.67E-44	0.517610977	0.426	0.317	2.09E-39	1.1	RNMT	1.34384858
RP11-473I1.5	8.70E-44	0.211363867	0.081	0.027	2.10E-39	1.1	RP11-473I1	3
AFTPH	2.39E-43	0.460524907	0.419	0.302	5.76E-39	1.1	AFTPH	1.387417219

EIF4E	2.71E-43	0.462980271	0.482	0.37	6.53E-39	1.1	EIF4E	1.302702703
ATP5L	4.41E-43	0.284876291	0.851	0.804	1.06E-38	1.1	ATP5L	1.058457711
LSM5	7.14E-43	0.429712574	0.444	0.335	1.72E-38	1.1	LSM5	1.325373134
FAM160A1	8.15E-43	0.459369974	0.715	0.642	1.97E-38	1.1	FAM160A1	1.113707165
TFG	6.86E-42	0.484964753	0.391	0.282	1.65E-37	1.1	TFG	1.386524823
COX4I1	1.11E-41	0.461792963	0.77	0.775	2.69E-37	1.1	COX4I1	0.993548387
LDHC	1.41E-41	0.263984251	0.064	0.019	3.40E-37	1.1	LDHC	3.368421053
RP11-111E14.1	6.07E-41	0.218759554	0.074	0.024	1.46E-36	1.1	RP11-111E	3.083333333
LINC00887	6.21E-41	0.327667557	0.083	0.03	1.50E-36	1.1	LINC00887	2.766666667
RGCC	1.13E-40	0.243182761	0.084	0.03	2.72E-36	1.1	RGCC	2.8
WDR45B	1.24E-40	0.492063883	0.308	0.205	2.99E-36	1.1	WDR45B	1.502439024
MORF4L2	2.05E-40	0.299471513	0.795	0.736	4.95E-36	1.1	MORF4L2	1.080163043
NDUFAF4	2.12E-40	0.344575044	0.141	0.067	5.12E-36	1.1	NDUFAF4	2.104477612
EXOSC8	2.35E-40	0.378132443	0.201	0.11	5.66E-36	1.1	EXOSC8	1.827272727
ZFP36L2	3.84E-40	0.579374398	0.476	0.36	9.25E-36	1.1	ZFP36L2	1.322222222
RAB9A	3.95E-40	0.45078117	0.254	0.154	9.53E-36	1.1	RAB9A	1.649350649
PALMD	7.68E-40	0.153198983	0.05	0.013	1.85E-35	1.1	PALMD	3.846153846
SWAP70	9.31E-40	0.488079251	0.396	0.288	2.24E-35	1.1	SWAP70	1.375
SNRPF	1.36E-39	0.396485616	0.285	0.181	3.28E-35	1.1	SNRPF	1.574585635
JMJD1C	1.44E-39	0.458901221	0.736	0.693	3.48E-35	1.1	JMJD1C	1.062049062
OTUD3	1.47E-39	0.298842737	0.101	0.041	3.55E-35	1.1	OTUD3	2.463414634
LRP2	1.52E-39	0.498700348	0.264	0.164	3.68E-35	1.1	LRP2	1.609756098
SFRP1	1.66E-39	0.345561731	0.33	0.213	4.01E-35	1.1	SFRP1	1.549295775
MARCH7	1.68E-39	0.44824887	0.35	0.242	4.05E-35	1.1	MARCH7	1.446280992
CD74	2.17E-39	0.281915852	0.158	0.078	5.23E-35	1.1	CD74	2.025641026
MT-CO2	2.22E-39	0.1980003	0.985	0.987	5.35E-35	1.1	MT-CO2	0.997973658
STX12	2.29E-39	0.593659041	0.414	0.31	5.51E-35	1.1	STX12	1.335483871
UBE2H	2.42E-39	0.336828462	0.796	0.751	5.84E-35	1.1	UBE2H	1.059920107
ADM	2.55E-39	0.215962774	0.063	0.019	6.14E-35	1.1	ADM	3.315789474
DYRK1A	2.80E-39	0.525171688	0.468	0.372	6.76E-35	1.1	DYRK1A	1.258064516
TMEM159	5.96E-39	0.430629077	0.405	0.3	1.44E-34	1.1	TMEM159	1.35
ATF3	1.20E-38	0.562070061	0.493	0.397	2.89E-34	1.1	ATF3	1.241813602
MTUS1	1.22E-38	0.495821191	0.473	0.367	2.94E-34	1.1	MTUS1	1.288828338
ARHGEF10	1.72E-38	0.48255606	0.312	0.208	4.16E-34	1.1	ARHGEF10	1.5
ADAM17	3.98E-38	0.498182299	0.469	0.372	9.60E-34	1.1	ADAM17	1.260752688
C2orf88	5.58E-38	0.444319521	0.442	0.338	1.35E-33	1.1	C2orf88	1.307692308
CCT8	5.74E-38	0.426217051	0.376	0.27	1.38E-33	1.1	CCT8	1.392592593
USP6NL	6.20E-38	0.467788728	0.276	0.176	1.50E-33	1.1	USP6NL	1.568181818
RAPGEF2	1.01E-37	0.546878456	0.506	0.412	2.44E-33	1.1	RAPGEF2	1.22815534
TES	1.12E-37	0.44648535	0.404	0.299	2.70E-33	1.1	TES	1.351170569
WDR33	1.79E-37	0.418348817	0.359	0.254	4.32E-33	1.1	WDR33	1.413385827
C1orf198	3.14E-37	0.261538859	0.103	0.043	7.58E-33	1.1	C1orf198	2.395348837
SLC39A2	3.34E-37	0.101378187	0.025	0.004	8.05E-33	1.1	SLC39A2	6.25
PUM1	3.37E-37	0.435514018	0.57	0.489	8.12E-33	1.1	PUM1	1.165644172
MAP3K13	6.27E-37	0.431463597	0.7	0.611	1.51E-32	1.1	MAP3K13	1.145662848
TNRC6A	8.46E-37	0.471883904	0.312	0.212	2.04E-32	1.1	TNRC6A	1.471698113
GNL2	8.86E-37	0.313187921	0.149	0.075	2.14E-32	1.1	GNL2	1.986666667
HADHA	1.10E-36	0.418974636	0.373	0.272	2.66E-32	1.1	HADHA	1.371323529
ZNF592	1.90E-36	0.375065202	0.192	0.107	4.59E-32	1.1	ZNF592	1.794392523
TOMM20	2.39E-36	0.347331826	0.284	0.182	5.77E-32	1.1	TOMM20	1.56043956
SLC2A1	3.36E-36	0.3483585	0.15	0.075	8.11E-32	1.1	SLC2A1	2
PSMB7	3.80E-36	0.333924766	0.554	0.44	9.17E-32	1.1	PSMB7	1.259090909

SPIRE1	6.65E-36	0.410119359	0.347	0.241	1.60E-31	1.1	SPIRE1	1.439834025
ARHGEF7	6.70E-36	0.413172414	0.217	0.129	1.62E-31	1.1	ARHGEF7	1.682170543
RTN4	6.77E-36	0.38882655	0.711	0.647	1.63E-31	1.1	RTN4	1.098918083
KRT19	9.56E-36	0.626833382	0.376	0.276	2.30E-31	1.1	KRT19	1.362318841
PRKCI	9.93E-36	0.424459258	0.266	0.17	2.39E-31	1.1	PRKCI	1.564705882
STX19	1.00E-35	0.144190489	0.049	0.014	2.42E-31	1.1	STX19	3.5
ARL4A	1.06E-35	0.386879409	0.194	0.109	2.55E-31	1.1	ARL4A	1.779816514
TBCA	1.28E-35	0.327497034	0.627	0.533	3.09E-31	1.1	TBCA	1.176360225
NDUFS4	1.86E-35	0.402994478	0.494	0.402	4.50E-31	1.1	NDUFS4	1.228855721
SLC16A13	2.23E-35	0.524248	0.076	0.028	5.38E-31	1.1	SLC16A13	2.714285714
GPRC5A	2.50E-35	0.766468958	0.46	0.383	6.02E-31	1.1	GPRC5A	1.201044386
PAICS	2.93E-35	0.32601094	0.235	0.143	7.06E-31	1.1	PAICS	1.643356643
PHF3	3.38E-35	0.398761474	0.393	0.287	8.15E-31	1.1	PHF3	1.369337979
KRT7	3.75E-35	0.447238156	0.357	0.252	9.04E-31	1.1	KRT7	1.416666667
PABPC1	6.65E-35	0.309755761	0.747	0.728	1.60E-30	1.1	PABPC1	1.026098901
PID1	7.36E-35	0.40197907	0.138	0.069	1.77E-30	1.1	PID1	2
CLCA4	1.45E-34	0.25652319	0.047	0.013	3.49E-30	1.1	CLCA4	3.615384615
RP11-795H16.3	1.69E-34	0.546790657	0.448	0.351	4.06E-30	1.1	RP11-795H	1.276353276
RAI14	2.00E-34	0.481879119	0.253	0.161	4.82E-30	1.1	RAI14	1.571428571
TPRKB	2.34E-34	0.373330362	0.24	0.149	5.65E-30	1.1	TPRKB	1.610738255
MTRNR2L12	2.63E-34	0.385013514	0.315	0.213	6.34E-30	1.1	MTRNR2L1	1.478873239
ITGB1	2.67E-34	0.551370771	0.416	0.327	6.44E-30	1.1	ITGB1	1.272171254
RFC1	2.80E-34	0.376753505	0.283	0.188	6.74E-30	1.1	RFC1	1.505319149
NFIL3	3.03E-34	0.377317235	0.275	0.179	7.30E-30	1.1	NFIL3	1.536312849
PIGP	3.39E-34	0.30660127	0.204	0.118	8.18E-30	1.1	PIGP	1.728813559
AP001439.2	5.62E-34	0.328826875	0.136	0.068	1.36E-29	1.1	AP001439.:	2
PCBP1	7.67E-34	0.422979681	0.56	0.499	1.85E-29	1.1	PCBP1	1.122244489
RPL15	8.40E-34	0.346117968	0.789	0.777	2.03E-29	1.1	RPL15	1.015444015
RP11-317G6.1	1.15E-33	0.230628855	0.076	0.029	2.78E-29	1.1	RP11-317G	2.620689655
RPL36AL	1.16E-33	0.226305479	0.912	0.883	2.81E-29	1.1	RPL36AL	1.032842582
ZNF143	1.29E-33	0.360861441	0.237	0.147	3.11E-29	1.1	ZNF143	1.612244898
TIMM10	1.33E-33	0.355207765	0.281	0.185	3.20E-29	1.1	TIMM10	1.518918919
ANKRD37	1.56E-33	0.275469113	0.12	0.057	3.75E-29	1.1	ANKRD37	2.105263158
COX7B	1.59E-33	0.341257913	0.682	0.628	3.84E-29	1.1	COX7B	1.085987261
LIAS	2.12E-33	0.246130427	0.089	0.037	5.10E-29	1.1	LIAS	2.405405405
MORC3	3.32E-33	0.406710068	0.328	0.23	8.02E-29	1.1	MORC3	1.426086957
LMBRD1	4.30E-33	0.374144109	0.305	0.207	1.04E-28	1.1	LMBRD1	1.473429952
RBFOX2	5.18E-33	0.363517061	0.978	0.948	1.25E-28	1.1	RBFOX2	1.03164557
CAMK2D	7.07E-33	0.512922777	0.254	0.164	1.71E-28	1.1	CAMK2D	1.548780488
TMEM57	8.18E-33	0.392050873	0.24	0.151	1.97E-28	1.1	TMEM57	1.589403974
ERGIC3	8.20E-33	0.445625772	0.404	0.316	1.98E-28	1.1	ERGIC3	1.278481013
CTGF	9.49E-33	0.332705447	0.058	0.019	2.29E-28	1.1	CTGF	3.052631579
NEDD4L	1.14E-32	0.247405551	0.692	0.588	2.75E-28	1.1	NEDD4L	1.176870748
PPIA	1.34E-32	0.282708393	0.823	0.803	3.22E-28	1.1	PPIA	1.0249066
RCAN1	1.87E-32	0.234195643	0.51	0.382	4.51E-28	1.1	RCAN1	1.335078534
HIST1H4C	2.18E-32	0.440627923	0.246	0.158	5.25E-28	1.1	HIST1H4C	1.556962025
DCTN6	2.20E-32	0.408695593	0.364	0.268	5.30E-28	1.1	DCTN6	1.358208955
PVT1	2.20E-32	0.431169769	0.43	0.335	5.31E-28	1.1	PVT1	1.28358209
WBP5	3.39E-32	0.423046323	0.42	0.322	8.17E-28	1.1	WBP5	1.304347826
TMEM126A	4.36E-32	0.275672444	0.164	0.09	1.05E-27	1.1	TMEM126/	1.822222222
NONO	8.94E-32	0.36422657	0.401	0.304	2.16E-27	1.1	NONO	1.319078947
C16orf72	1.18E-31	0.332137259	0.218	0.133	2.85E-27	1.1	C16orf72	1.639097744

MCTS1	1.27E-31	0.361937844	0.298	0.206	3.06E-27	1.1		MCTS1	1.446601942
NOD2	2.12E-31	0.207969188	0.085	0.035	5.12E-27	1.1		NOD2	2.428571429
RABGGTB	3.10E-31	0.3009941	0.135	0.069	7.47E-27	1.1		RABGGTB	1.956521739
SEL1L	6.25E-31	0.36632849	0.167	0.094	1.51E-26	1.1		SEL1L	1.776595745
RPS18	8.74E-31	0.374881691	0.694	0.629	2.11E-26	1.1		RPS18	1.103338633
TPM4	9.13E-31	0.358907494	0.459	0.363	2.20E-26	1.1		TPM4	1.26446281
FAM126B	1.13E-30	0.411924621	0.206	0.126	2.72E-26	1.1		FAM126B	1.634920635
AC009313.1	1.18E-30	0.318679027	0.116	0.057	2.85E-26	1.1		AC009313.	2.035087719
RTCB	1.48E-30	0.421547639	0.482	0.4	3.57E-26	1.1		RTCB	1.205
LALBA	1.75E-30	0.77672953	0.023	0.004	4.21E-26	1.1		LALBA	5.75
METAP1	2.13E-30	0.260547256	0.115	0.056	5.13E-26	1.1		METAP1	2.053571429
OXSR1	4.52E-30	0.397857904	0.332	0.237	1.09E-25	1.1		OXSR1	1.400843882
GPM6B	5.88E-30	0.413665301	0.194	0.116	1.42E-25	1.1		GPM6B	1.672413793
MDH1	6.18E-30	0.379617562	0.358	0.266	1.49E-25	1.1		MDH1	1.345864662
CSRP1	9.08E-30	0.299862647	0.196	0.117	2.19E-25	1.1		CSRP1	1.675213675
TFCP2L1	1.33E-29	0.48068853	0.152	0.084	3.20E-25	1.1		TFCP2L1	1.80952381
POLR1D	1.39E-29	0.33538841	0.309	0.215	3.35E-25	1.1		POLR1D	1.437209302
AC026202.3	1.59E-29	0.434435798	0.286	0.198	3.84E-25	1.1		AC026202.:	1.444444444
RNF150	2.93E-29	0.36434206	0.124	0.063	7.07E-25	1.1		RNF150	1.968253968
RCC1	3.00E-29	0.445217279	0.351	0.264	7.24E-25	1.1		RCC1	1.329545455
TPD52	3.75E-29	0.345217319	0.199	0.121	9.03E-25	1.1		TPD52	1.644628099
TAGAP	4.90E-29	0.15488162	0.037	0.01	1.18E-24	1.1		TAGAP	3.7
BRIX1	5.15E-29	0.359709953	0.175	0.102	1.24E-24	1.1		BRIX1	1.715686275
KLK7	5.36E-29	0.172556359	0.046	0.014	1.29E-24	1.1		KLK7	3.285714286
RDX	5.67E-29	0.394134228	0.229	0.148	1.37E-24	1.1		RDX	1.547297297
PIP5K1A	5.81E-29	0.391857599	0.385	0.293	1.40E-24	1.1		PIP5K1A	1.313993174
TSLP	6.79E-29	0.245397684	0.079	0.033	1.64E-24	1.1		TSLP	2.393939394
AC072062.1	7.28E-29	0.38024129	0.548	0.464	1.76E-24	1.1		AC072062.	1.181034483
HNRNPA1L2	7.32E-29	0.171924738	0.085	0.036	1.77E-24	1.1		HNRNPA1L	2.361111111
RBPMS	8.27E-29	0.375020485	0.715	0.653	1.99E-24	1.1		RBPMS	1.094946401
MXD1	1.01E-28	0.348199072	0.298	0.202	2.43E-24	1.1		MXD1	1.475247525
MAGI1	1.19E-28	0.374141857	0.783	0.75	2.86E-24	1.1		MAGI1	1.044
DYRK2	2.46E-28	0.153610799	0.06	0.022	5.92E-24	1.1		DYRK2	2.727272727
FABP3	2.57E-28	0.357884203	0.035	0.009	6.19E-24	1.1		FABP3	3.888888889
ANXA2	2.71E-28	0.291838778	0.93	0.927	6.53E-24	1.1		ANXA2	1.003236246
GPAT3	2.87E-28	0.264959504	0.092	0.041	6.91E-24	1.1		GPAT3	2.243902439
AC090498.1	3.38E-28	0.380191072	0.384	0.298	8.16E-24	1.1		AC090498.	1.288590604
HINT1	4.89E-28	0.420229835	0.627	0.599	1.18E-23	1.1		HINT1	1.046744574
MEF2A	6.67E-28	0.487428067	0.488	0.415	1.61E-23	1.1		MEF2A	1.175903614
CCNY	7.35E-28	0.710438669	0.331	0.254	1.77E-23	1.1		CCNY	1.303149606
CSN3	8.25E-28	0.167724582	0.017	0.002	1.99E-23	1.1		CSN3	8.5
DSG3	1.04E-27	0.673004665	0.07	0.029	2.50E-23	1.1		DSG3	2.413793103
WEE1	1.22E-27	0.353324551	0.397	0.3	2.93E-23	1.1		WEE1	1.323333333
SQSTM1	1.42E-27	0.402140443	0.321	0.231	3.43E-23	1.1		SQSTM1	1.38961039
HPS5	1.58E-27	0.369723579	0.206	0.13	3.80E-23	1.1		HPS5	1.584615385
COX7C	2.22E-27	0.211703574	0.905	0.869	5.36E-23	1.1		COX7C	1.041426928
GJC3	2.55E-27	0.317945792	0.153	0.087	6.15E-23	1.1		GJC3	1.75862069
FOXC1	2.91E-27	0.195196623	0.077	0.033	7.03E-23	1.1		FOXC1	2.333333333
SMC5	6.01E-27	0.45323024	0.378	0.296	1.45E-22	1.1		SMC5	1.277027027
ADAMTS9	6.90E-27	0.161376832	0.304	0.201	1.66E-22	1.1		ADAMTS9	1.512437811
SLC25A33	1.02E-26	0.477355695	0.153	0.088	2.47E-22	1.1		SLC25A33	1.738636364
KARS	2.10E-26	0.305424219	0.327	0.237	5.06E-22	1.1		KARS	1.379746835

GNG12	2.50E-26	0.432234549	0.4	0.32	6.03E-22	1.1	GNG12	1.25
GNA13	2.64E-26	0.37259212	0.286	0.2	6.36E-22	1.1	GNA13	1.43
PRKCA	4.19E-26	0.321900008	0.116	0.061	1.01E-21	1.1	PRKCA	1.901639344
RASAL2	5.77E-26	0.584141782	0.59	0.537	1.39E-21	1.1	RASAL2	1.098696462
METAP2	5.96E-26	0.335633148	0.324	0.234	1.44E-21	1.1	METAP2	1.384615385
YARS	1.21E-25	0.40320877	0.356	0.271	2.93E-21	1.1	YARS	1.313653137
PREP	1.24E-25	0.261594345	0.144	0.081	2.99E-21	1.1	PREP	1.777777778
EIF2S1	1.28E-25	0.351707958	0.272	0.191	3.09E-21	1.1	EIF2S1	1.42408377
RP11-496N12.9	1.57E-25	0.310888895	0.027	0.006	3.78E-21	1.1	RP11-496N	4.5
RP11-68E19.2	1.64E-25	0.224166681	0.094	0.045	3.95E-21	1.1	RP11-68E1	2.088888889
VIM	1.66E-25	0.321703792	0.188	0.117	4.00E-21	1.1	VIM	1.606837607
CARD16	2.34E-25	0.21918265	0.087	0.04	5.64E-21	1.1	CARD16	2.175
RBM39	2.70E-25	0.31733743	0.523	0.446	6.51E-21	1.1	RBM39	1.17264574
NAP1L1	3.82E-25	0.330984459	0.434	0.349	9.22E-21	1.1	NAP1L1	1.243553009
MRPL45	3.87E-25	0.287328486	0.213	0.139	9.33E-21	1.1	MRPL45	1.532374101
MYO9B	4.97E-25	0.401387972	0.258	0.179	1.20E-20	1.1	MYO9B	1.441340782
PUM3	4.98E-25	0.357946645	0.239	0.162	1.20E-20	1.1	PUM3	1.475308642
ARHGAP29	6.90E-25	0.674291187	0.605	0.571	1.66E-20	1.1	ARHGAP29	1.059544658
UFC1	1.14E-24	0.32094493	0.544	0.478	2.75E-20	1.1	UFC1	1.138075314
RBPJ	1.46E-24	0.361477166	0.422	0.343	3.52E-20	1.1	RBPJ	1.2303207
AFF4	1.75E-24	0.379151477	0.499	0.428	4.22E-20	1.1	AFF4	1.16588785
ATP5F1	1.83E-24	0.363446225	0.441	0.365	4.41E-20	1.1	ATP5F1	1.208219178
HERC3	1.90E-24	0.410340659	0.226	0.151	4.59E-20	1.1	HERC3	1.496688742
FAM19A2	1.93E-24	0.345459815	0.671	0.585	4.65E-20	1.1	FAM19A2	1.147008547
FOSB	2.17E-24	0.450180055	0.458	0.388	5.23E-20	1.1	FOSB	1.180412371
AMOTL1	2.20E-24	0.176204275	0.088	0.041	5.31E-20	1.1	AMOTL1	2.146341463
RP11-306I1.2	2.24E-24	0.101206686	0.03	0.008	5.41E-20	1.1	RP11-306I1	3.75
HSF2	2.52E-24	0.240559146	0.12	0.065	6.08E-20	1.1	HSF2	1.846153846
SEPP1	3.38E-24	0.441611303	0.378	0.305	8.14E-20	1.1	SEPP1	1.239344262
FNBP4	3.42E-24	0.371783886	0.471	0.394	8.25E-20	1.1	FNBP4	1.195431472
SET	4.50E-24	0.331459997	0.477	0.401	1.08E-19	1.1	SET	1.189526185
ZCCHC8	5.23E-24	0.303235824	0.183	0.114	1.26E-19	1.1	ZCCHC8	1.605263158
GNB2L1	5.57E-24	0.352035138	0.693	0.67	1.34E-19	1.1	GNB2L1	1.034328358
GPBP1	6.59E-24	0.235564936	0.725	0.685	1.59E-19	1.1	GPBP1	1.058394161
NFIX	7.02E-24	0.236611348	0.115	0.061	1.69E-19	1.1	NFIX	1.885245902
S100A1	7.15E-24	0.187685695	0.078	0.035	1.72E-19	1.1	S100A1	2.228571429
TMEM150C	7.98E-24	0.307329335	0.278	0.198	1.92E-19	1.1	TMEM150(	1.404040404
SPCS2	9.90E-24	0.352022592	0.376	0.294	2.39E-19	1.1	SPCS2	1.278911565
SARNP	1.01E-23	0.39582104	0.267	0.191	2.43E-19	1.1	SARNP	1.397905759
DYRK3	1.03E-23	0.237792138	0.087	0.042	2.48E-19	1.1	DYRK3	2.071428571
FBLIM1	1.05E-23	0.284696096	0.165	0.1	2.52E-19	1.1	FBLIM1	1.65
DSG2	1.77E-23	0.338131077	0.336	0.255	4.27E-19	1.1	DSG2	1.317647059
RP11-481C4.1	2.14E-23	0.257794812	0.11	0.057	5.17E-19	1.1	RP11-481C	1.929824561
VPS37B	2.29E-23	0.272219784	0.191	0.121	5.53E-19	1.1	VPS37B	1.578512397
RRAGD	2.52E-23	0.209994687	0.095	0.048	6.08E-19	1.1	RRAGD	1.979166667
HNRNPDL	2.76E-23	0.313600998	0.363	0.28	6.65E-19	1.1	HNRNPDL	1.296428571
DYNLL1	3.35E-23	0.353952785	0.32	0.236	8.07E-19	1.1	DYNLL1	1.355932203
CROCC	4.44E-23	0.232448985	0.1	0.051	1.07E-18	1.1	CROCC	1.960784314
COX7A2L	5.07E-23	0.309593644	0.562	0.491	1.22E-18	1.1	COX7A2L	1.144602851
SEC11A	5.35E-23	0.303890602	0.546	0.476	1.29E-18	1.1	SEC11A	1.147058824
FOXJ3	9.32E-23	0.398020156	0.335	0.258	2.25E-18	1.1	FOXJ3	1.298449612
RNF19A	9.35E-23	0.438079461	0.619	0.569	2.26E-18	1.1	RNF19A	1.087873462

PNN	9.57E-23	0.363494012	0.391	0.315	2.31E-18	1.1	PNN	1.241269841
COMMD6	1.20E-22	0.338831496	0.423	0.347	2.88E-18	1.1	COMMD6	1.219020173
CHD6	1.84E-22	0.393268029	0.35	0.275	4.44E-18	1.1	CHD6	1.272727273
TMEM27	2.78E-22	0.178678919	0.055	0.022	6.71E-18	1.1	TMEM27	2.5
ARHGAP26-AS1	4.20E-22	0.378362565	0.208	0.14	1.01E-17	1.1	ARHGAP26	1.485714286
HNRNPD	4.22E-22	0.32691775	0.354	0.275	1.02E-17	1.1	HNRNPD	1.287272727
SH3RF1	4.86E-22	0.380544829	0.295	0.217	1.17E-17	1.1	SH3RF1	1.359447005
SF3B6	5.15E-22	0.269959043	0.649	0.604	1.24E-17	1.1	SF3B6	1.074503311
EIF2S3	5.58E-22	0.324738338	0.364	0.284	1.35E-17	1.1	EIF2S3	1.281690141
NUDT2	6.47E-22	0.25080481	0.184	0.118	1.56E-17	1.1	NUDT2	1.559322034
AC004878.2	7.54E-22	0.141660738	0.046	0.017	1.82E-17	1.1	AC004878.	2.705882353
POLR1E	9.91E-22	0.134489298	0.054	0.021	2.39E-17	1.1	POLR1E	2.571428571
TMEM263	1.19E-21	0.230608808	0.145	0.086	2.86E-17	1.1	TMEM263	1.686046512
ATG14	1.49E-21	0.267069242	0.155	0.095	3.60E-17	1.1	ATG14	1.631578947
RASSF3	1.82E-21	0.281219268	0.181	0.115	4.38E-17	1.1	RASSF3	1.573913043
LINC01492	1.90E-21	0.196640168	0.132	0.076	4.58E-17	1.1	LINC01492	1.736842105
ITGA2	2.09E-21	0.495124731	0.453	0.382	5.04E-17	1.1	ITGA2	1.185863874
KHDRBS1	2.27E-21	0.309890601	0.36	0.283	5.48E-17	1.1	KHDRBS1	1.272084806
SLC6A14	3.45E-21	0.303490766	0.16	0.099	8.31E-17	1.1	SLC6A14	1.616161616
PDIA3	3.72E-21	0.367439676	0.378	0.299	8.98E-17	1.1	PDIA3	1.264214047
EIF3D	4.50E-21	0.330080135	0.393	0.32	1.09E-16	1.1	EIF3D	1.228125
AK2	6.28E-21	0.256168998	0.17	0.107	1.52E-16	1.1	AK2	1.588785047
MAP1B	9.88E-21	0.263715842	0.346	0.259	2.38E-16	1.1	MAP1B	1.335907336
MGAT5	1.04E-20	0.37679059	0.329	0.257	2.50E-16	1.1	MGAT5	1.280155642
MAP2	1.20E-20	0.35638797	0.148	0.091	2.89E-16	1.1	MAP2	1.626373626
MID1	1.61E-20	0.249995071	0.108	0.059	3.87E-16	1.1	MID1	1.830508475
MPZL1	1.65E-20	0.429030728	0.431	0.368	3.98E-16	1.1	MPZL1	1.171195652
MORF4L1	1.74E-20	0.246848459	0.679	0.625	4.19E-16	1.1	MORF4L1	1.0864
CYLD	1.88E-20	0.382501061	0.28	0.205	4.52E-16	1.1	CYLD	1.365853659
INTS6-AS1	1.94E-20	0.241824464	0.178	0.116	4.67E-16	1.1	INTS6-AS1	1.534482759
NUS1	2.10E-20	0.260002453	0.129	0.076	5.07E-16	1.1	NUS1	1.697368421
RPS2	2.53E-20	0.297033667	0.346	0.266	6.10E-16	1.1	RPS2	1.30075188
RP11-443B7.1	2.64E-20	0.107965497	0.047	0.018	6.36E-16	1.1	RP11-443B	2.611111111
SLC25A37	2.72E-20	0.267250165	0.474	0.374	6.55E-16	1.1	SLC25A37	1.267379679
CHSY1	2.91E-20	0.212432732	0.146	0.088	7.02E-16	1.1	CHSY1	1.659090909
GTF2F2	3.05E-20	0.245083491	0.157	0.098	7.35E-16	1.1	GTF2F2	1.602040816
TCF7L2	4.29E-20	0.424027899	0.441	0.376	1.03E-15	1.1	TCF7L2	1.17287234
ZC3H15	4.49E-20	0.309947895	0.301	0.228	1.08E-15	1.1	ZC3H15	1.320175439
RP11-840I19.3	6.05E-20	0.239697367	0.103	0.056	1.46E-15	1.1	RP11-840I1	1.839285714
CLEC2B	7.35E-20	0.330234857	0.106	0.059	1.77E-15	1.1	CLEC2B	1.796610169
CENPU	8.66E-20	0.116889612	0.036	0.012	2.09E-15	1.1	CENPU	3
INTS12	8.71E-20	0.341038206	0.274	0.203	2.10E-15	1.1	INTS12	1.349753695
RP11-817J15.3	1.05E-19	0.106997218	0.034	0.011	2.54E-15	1.1	RP11-817J1	3.090909091
CA5B	1.10E-19	0.289135319	0.181	0.119	2.66E-15	1.1	CA5B	1.521008403
CNN3	1.34E-19	0.389453072	0.557	0.511	3.22E-15	1.1	CNN3	1.090019569
IPO5	2.16E-19	0.261185994	0.158	0.101	5.21E-15	1.1	IPO5	1.564356436
ATP11B	2.17E-19	0.343149396	0.21	0.146	5.24E-15	1.1	ATP11B	1.438356164
WDR48	2.33E-19	0.300386381	0.251	0.182	5.61E-15	1.1	WDR48	1.379120879
RP11-356C4.5	2.40E-19	0.448159205	0.203	0.143	5.78E-15	1.1	RP11-356C	1.41958042
GSTO2	3.49E-19	0.210685843	0.147	0.09	8.41E-15	1.1	GSTO2	1.633333333
SNHG12	3.49E-19	0.194096406	0.08	0.04	8.42E-15	1.1	SNHG12	2
TMEM258	3.66E-19	0.228559018	0.649	0.593	8.82E-15	1.1	TMEM258	1.094435076

MT-CO3	3.86E-19	0.174554921	0.998	0.995	9.31E-15	1.1	MT-CO3	1.003015075
KIF13B	5.43E-19	0.45729419	0.314	0.251	1.31E-14	1.1	KIF13B	1.250996016
CPA4	6.15E-19	0.101143338	0.024	0.006	1.48E-14	1.1	CPA4	4
WDR43	8.41E-19	0.253621328	0.159	0.101	2.03E-14	1.1	WDR43	1.574257426
RP11-314N13.10	9.15E-19	0.402317356	0.17	0.112	2.21E-14	1.1	RP11-314N	1.517857143
CSN1S1	9.83E-19	0.435818396	0.009	0.001	2.37E-14	1.1	CSN1S1	9
PCNXL2	1.53E-18	0.352898681	0.812	0.758	3.69E-14	1.1	PCNXL2	1.071240106
SDK2	1.71E-18	0.236148372	0.101	0.056	4.12E-14	1.1	SDK2	1.803571429
TCEAL8	1.73E-18	0.27655208	0.297	0.226	4.16E-14	1.1	TCEAL8	1.314159292
EEPD1	1.89E-18	0.165837789	0.086	0.045	4.57E-14	1.1	EEPD1	1.911111111
WBP4	1.90E-18	0.246863216	0.146	0.092	4.59E-14	1.1	WBP4	1.586956522
PIAS2	1.99E-18	0.228673573	0.115	0.067	4.79E-14	1.1	PIAS2	1.71641791
DPH5	5.51E-18	0.163939824	0.096	0.053	1.33E-13	1.1	DPH5	1.811320755
FBXO42	5.60E-18	0.331306005	0.222	0.159	1.35E-13	1.1	FBXO42	1.396226415
BEX5	6.45E-18	0.136903546	0.055	0.024	1.56E-13	1.1	BEX5	2.291666667
EIF3I	8.39E-18	0.230095802	0.635	0.59	2.02E-13	1.1	EIF3I	1.076271186
SPAG9	1.67E-17	0.357156502	0.403	0.339	4.02E-13	1.1	SPAG9	1.18879056
RP1-117O3.2	1.84E-17	0.184725684	0.067	0.033	4.43E-13	1.1	RP1-117O3	2.03030303
RP11-557C18.3	2.19E-17	0.239922242	0.084	0.045	5.28E-13	1.1	RP11-557C	1.866666667
ARF6	2.26E-17	0.323645092	0.265	0.198	5.46E-13	1.1	ARF6	1.338383838
SEMA3B	2.32E-17	0.134319993	0.045	0.018	5.60E-13	1.1	SEMA3B	2.5
OSTC	2.35E-17	0.266844003	0.512	0.452	5.66E-13	1.1	OSTC	1.132743363
SPHKAP	3.12E-17	0.12156897	0.047	0.02	7.53E-13	1.1	SPHKAP	2.35
LARS	3.24E-17	0.285215949	0.256	0.19	7.81E-13	1.1	LARS	1.347368421
RAB8B	4.34E-17	0.32515612	0.2	0.141	1.05E-12	1.1	RAB8B	1.418439716
ENO1	5.26E-17	0.195122173	0.445	0.375	1.27E-12	1.1	ENO1	1.186666667
PREPL	5.43E-17	0.224446863	0.155	0.1	1.31E-12	1.1	PREPL	1.55
AFAP1	5.89E-17	0.20068407	0.132	0.082	1.42E-12	1.1	AFAP1	1.609756098
PLEKHH2	8.52E-17	0.195246176	0.106	0.062	2.05E-12	1.1	PLEKHH2	1.709677419
SYNE1	9.73E-17	0.180189458	0.05	0.022	2.35E-12	1.1	SYNE1	2.272727273
VAPA	1.10E-16	0.276918815	0.521	0.469	2.66E-12	1.1	VAPA	1.1108742
TRA2A	1.21E-16	0.308450232	0.455	0.396	2.92E-12	1.1	TRA2A	1.148989899
CCDC82	1.26E-16	0.305208899	0.209	0.15	3.04E-12	1.1	CCDC82	1.393333333
TMEM99	1.35E-16	0.193144881	0.118	0.071	3.25E-12	1.1	TMEM99	1.661971831
LINC00866	1.72E-16	0.267337512	0.038	0.015	4.14E-12	1.1	LINC00866	2.533333333
HOTAIRM1	1.90E-16	0.128925591	0.064	0.031	4.58E-12	1.1	HOTAIRM1	2.064516129
VPS29	1.90E-16	0.303181593	0.381	0.314	4.59E-12	1.1	VPS29	1.213375796
ARHGDIB	2.01E-16	0.327945025	0.208	0.15	4.84E-12	1.1	ARHGDIB	1.386666667
HERPUD1	3.20E-16	0.299865496	0.284	0.217	7.71E-12	1.1	HERPUD1	1.30875576
TCP1	3.45E-16	0.298803114	0.222	0.162	8.32E-12	1.1	TCP1	1.37037037
CDK6	3.72E-16	0.277941483	0.18	0.124	8.96E-12	1.1	CDK6	1.451612903
ATP5G1	3.81E-16	0.269540912	0.239	0.177	9.20E-12	1.1	ATP5G1	1.350282486
DAPK3	4.13E-16	0.18285671	0.091	0.051	9.96E-12	1.1	DAPK3	1.784313725
UBE3A	4.16E-16	0.340858754	0.357	0.294	1.00E-11	1.1	UBE3A	1.214285714
ZMYM5	4.27E-16	0.279308557	0.201	0.143	1.03E-11	1.1	ZMYM5	1.405594406
PRDM2	4.43E-16	0.195629752	0.358	0.29	1.07E-11	1.1	PRDM2	1.234482759
NF2	5.39E-16	0.200160907	0.104	0.061	1.30E-11	1.1	NF2	1.704918033
OAT	5.82E-16	0.364811595	0.332	0.271	1.40E-11	1.1	OAT	1.225092251
TOP2B	6.47E-16	0.253447728	0.251	0.186	1.56E-11	1.1	TOP2B	1.349462366
YWHAE	7.85E-16	0.257665237	0.652	0.626	1.89E-11	1.1	YWHAE	1.041533546
MFSD14A	7.92E-16	0.258595232	0.176	0.122	1.91E-11	1.1	MFSD14A	1.442622951
SEC63	1.17E-15	0.2905004	0.281	0.218	2.81E-11	1.1	SEC63	1.288990826

RP11-299J3.8	1.42E-15	0.155077795	0.079	0.042	3.42E-11	1.1	RP11-299J3	1.880952381
CTTNBP2NL	1.44E-15	0.330989891	0.264	0.204	3.48E-11	1.1	CTTNBP2N	1.294117647
ANP32B	1.58E-15	0.229089786	0.203	0.144	3.81E-11	1.1	ANP32B	1.409722222
ACTR3	1.69E-15	0.30352562	0.326	0.265	4.06E-11	1.1	ACTR3	1.230188679
MRPL22	1.91E-15	0.273179781	0.271	0.21	4.60E-11	1.1	MRPL22	1.29047619
MIR4435-2HG	1.98E-15	0.345866972	0.544	0.499	4.78E-11	1.1	MIR4435-2	1.090180361
CKS1B	5.19E-15	0.266798899	0.368	0.308	1.25E-10	1.1	CKS1B	1.194805195
RP11-307P5.1	5.48E-15	0.112517349	0.044	0.019	1.32E-10	1.1	RP11-307P.	2.315789474
CCDC59	6.77E-15	0.208480509	0.244	0.181	1.63E-10	1.1	CCDC59	1.348066298
MEF2C	6.83E-15	0.117462294	0.036	0.014	1.65E-10	1.1	MEF2C	2.571428571
LINC00486	7.08E-15	0.117746631	0.988	0.987	1.71E-10	1.1	LINC00486	1.001013171
MBIP	7.38E-15	0.236544198	0.208	0.151	1.78E-10	1.1	MBIP	1.377483444
RAP2B	8.85E-15	0.327197483	0.232	0.175	2.13E-10	1.1	RAP2B	1.325714286
CLIC4	9.31E-15	0.268080013	0.598	0.557	2.24E-10	1.1	CLIC4	1.073608618
HSPA9	9.94E-15	0.268423285	0.272	0.211	2.40E-10	1.1	HSPA9	1.289099526
AC074289.1	1.07E-14	0.110228184	0.044	0.019	2.58E-10	1.1	AC074289.	2.315789474
MCF2L2	1.26E-14	0.262926964	0.143	0.095	3.03E-10	1.1	MCF2L2	1.505263158
LATS2	1.28E-14	0.213900498	0.157	0.106	3.09E-10	1.1	LATS2	1.481132075
LYRM4	1.36E-14	0.224952692	0.189	0.134	3.27E-10	1.1	LYRM4	1.410447761
SAR1A	1.38E-14	0.259110663	0.243	0.184	3.33E-10	1.1	SAR1A	1.320652174
SARS	1.40E-14	0.239178188	0.228	0.171	3.37E-10	1.1	SARS	1.333333333
RAPGEF1	2.46E-14	0.215702934	0.145	0.097	5.94E-10	1.1	RAPGEF1	1.494845361
EIF1B	2.71E-14	0.27052905	0.277	0.218	6.54E-10	1.1	EIF1B	1.270642202
ITGB5-AS1	2.75E-14	0.314913781	0.049	0.023	6.63E-10	1.1	ITGB5-AS1	2.130434783
JAZF1	3.22E-14	0.184236623	0.128	0.082	7.75E-10	1.1	JAZF1	1.56097561
FGF2	3.34E-14	0.182824799	0.073	0.039	8.05E-10	1.1	FGF2	1.871794872
RPL13	3.69E-14	0.268656507	0.468	0.413	8.90E-10	1.1	RPL13	1.133171913
HLA-DRB1	3.76E-14	0.155613439	0.081	0.046	9.06E-10	1.1	HLA-DRB1	1.760869565
EXT1	3.85E-14	0.443926437	0.531	0.493	9.28E-10	1.1	EXT1	1.077079108
MAMLD1	3.98E-14	0.127324243	0.062	0.032	9.59E-10	1.1	MAMLD1	1.9375
TRAM1	4.01E-14	0.244541056	0.247	0.189	9.68E-10	1.1	TRAM1	1.306878307
PCGF5	4.54E-14	0.29607723	0.223	0.168	1.09E-09	1.1	PCGF5	1.327380952
NMB	6.27E-14	0.10950079	0.036	0.015	1.51E-09	1.1	NMB	2.4
CACUL1	6.69E-14	0.302026424	0.426	0.374	1.61E-09	1.1	CACUL1	1.139037433
ARMCX3	7.10E-14	0.202770493	0.174	0.122	1.71E-09	1.1	ARMCX3	1.426229508
CASP1	7.95E-14	0.14235512	0.055	0.028	1.92E-09	1.1	CASP1	1.964285714
DNAJB6	8.40E-14	0.354603383	0.428	0.378	2.03E-09	1.1	DNAJB6	1.132275132
ATP5C1	9.39E-14	0.243530947	0.336	0.279	2.27E-09	1.1	ATP5C1	1.204301075
DSC2	9.63E-14	0.294863165	0.26	0.203	2.32E-09	1.1	DSC2	1.280788177
MAPKAPK2	1.02E-13	0.210439737	0.196	0.141	2.46E-09	1.1	MAPKAPK2	1.390070922
INSIG2	1.13E-13	0.342959531	0.261	0.206	2.72E-09	1.1	INSIG2	1.266990291
GADD45A	1.14E-13	0.314822773	0.322	0.261	2.75E-09	1.1	GADD45A	1.233716475
KIT	1.14E-13	0.221733518	0.228	0.17	2.76E-09	1.1	KIT	1.341176471
FAM46B	1.21E-13	0.213617021	0.087	0.05	2.91E-09	1.1	FAM46B	1.74
SNX8	1.60E-13	0.230532078	0.081	0.047	3.85E-09	1.1	SNX8	1.723404255
UBQLN1	2.12E-13	0.237867285	0.295	0.236	5.11E-09	1.1	UBQLN1	1.25
SERP1	2.21E-13	0.226211418	0.308	0.248	5.34E-09	1.1	SERP1	1.241935484
BCL7A	2.45E-13	0.12078195	0.053	0.026	5.90E-09	1.1	BCL7A	2.038461538
TNFRSF10B	3.18E-13	0.283409849	0.257	0.2	7.68E-09	1.1	TNFRSF10E	1.285
C12orf45	4.13E-13	0.115038279	0.051	0.025	9.96E-09	1.1	C12orf45	2.04
PISD	4.24E-13	0.265180572	0.214	0.161	1.02E-08	1.1	PISD	1.329192547
NOP58	4.62E-13	0.235635129	0.258	0.2	1.12E-08	1.1	NOP58	1.29

YWHAQ	4.89E-13	0.312366362	0.411	0.362	1.18E-08	1.1	YWHAQ	1.135359116
FAM133B	5.33E-13	0.19125484	0.141	0.096	1.28E-08	1.1	FAM133B	1.46875
TP53BP2	7.54E-13	0.276935336	0.166	0.119	1.82E-08	1.1	TP53BP2	1.394957983
AC016995.3	9.68E-13	0.16882613	0.169	0.118	2.33E-08	1.1	AC016995.:	1.43220339
ITGBL1	1.03E-12	0.131281784	0.048	0.024	2.49E-08	1.1	ITGBL1	2
ACSL4	1.08E-12	0.211951628	0.083	0.05	2.60E-08	1.1	ACSL4	1.66
RP11-84A19.4	1.23E-12	0.166619075	0.086	0.051	2.98E-08	1.1	RP11-84A1	1.68627451
RP11-685M7.5	1.36E-12	0.16236316	0.073	0.041	3.29E-08	1.1	RP11-685M	1.780487805
CENPW	1.44E-12	0.241507075	0.106	0.068	3.48E-08	1.1	CENPW	1.558823529
EFNB2	1.57E-12	0.199852382	0.146	0.1	3.80E-08	1.1	EFNB2	1.46
DDX47	1.67E-12	0.201468169	0.059	0.032	4.03E-08	1.1	DDX47	1.84375
CDC27	1.89E-12	0.33839094	0.31	0.258	4.56E-08	1.1	CDC27	1.201550388
RP11-758H9.2	1.95E-12	0.126103662	0.035	0.015	4.71E-08	1.1	RP11-758H	2.333333333
RP11-87M18.2	2.19E-12	0.124550363	0.079	0.046	5.29E-08	1.1	RP11-87M1	1.717391304
DAB2IP	2.24E-12	0.205062717	0.119	0.078	5.40E-08	1.1	DAB2IP	1.525641026
GNA12	2.37E-12	0.35509583	0.341	0.295	5.72E-08	1.1	GNA12	1.155932203
B3GNT5	2.85E-12	0.181320252	0.088	0.054	6.87E-08	1.1	B3GNT5	1.62962963
MIER1	3.19E-12	0.295028745	0.218	0.168	7.69E-08	1.1	MIER1	1.297619048
PKIG	3.31E-12	0.15243382	0.082	0.049	7.99E-08	1.1	PKIG	1.673469388
CD2AP	3.32E-12	0.362560662	0.466	0.425	8.00E-08	1.1	CD2AP	1.096470588
SSBP2	3.65E-12	0.361876734	0.481	0.432	8.79E-08	1.1	SSBP2	1.113425926
HOXA3	3.81E-12	0.102388174	0.044	0.021	9.19E-08	1.1	HOXA3	2.095238095
MORN2	4.26E-12	0.129896701	0.095	0.058	1.03E-07	1.1	MORN2	1.637931034
LHFPL3	4.45E-12	0.241589465	0.309	0.255	1.07E-07	1.1	LHFPL3	1.211764706
RIC3	4.56E-12	0.455500328	0.086	0.054	1.10E-07	1.1	RIC3	1.592592593
ITGA6	4.59E-12	0.188798331	0.118	0.077	1.11E-07	1.1	ITGA6	1.532467532
ASUN	5.03E-12	0.123406553	0.062	0.034	1.21E-07	1.1	ASUN	1.823529412
CDC5L	5.41E-12	0.240145963	0.247	0.193	1.30E-07	1.1	CDC5L	1.279792746
UHRF1BP1L	5.65E-12	0.259167194	0.253	0.2	1.36E-07	1.1	UHRF1BP1l	1.265
RAN	6.91E-12	0.217826662	0.563	0.526	1.67E-07	1.1	RAN	1.070342205
INSR	7.39E-12	0.236611769	0.586	0.551	1.78E-07	1.1	INSR	1.063520871
RICTOR	8.46E-12	0.152080611	0.66	0.619	2.04E-07	1.1	RICTOR	1.066235864
HBS1L	1.01E-11	0.252556085	0.231	0.18	2.44E-07	1.1	HBS1L	1.283333333
LRRC75A	1.12E-11	0.250361586	0.179	0.133	2.69E-07	1.1	LRRC75A	1.345864662
PPP1R3B	1.14E-11	0.122199519	0.069	0.039	2.75E-07	1.1	PPP1R3B	1.769230769
RPS19	1.16E-11	0.190435915	0.241	0.18	2.80E-07	1.1	RPS19	1.338888889
CRBN	2.07E-11	0.199427557	0.188	0.14	4.98E-07	1.1	CRBN	1.342857143
MBD2	2.16E-11	0.32640052	0.35	0.304	5.21E-07	1.1	MBD2	1.151315789
TBC1D8	2.17E-11	0.261514492	0.474	0.425	5.24E-07	1.1	TBC1D8	1.115294118
RECK	2.29E-11	0.122472764	0.049	0.025	5.53E-07	1.1	RECK	1.96
SRSF12	2.56E-11	0.126397964	0.068	0.039	6.17E-07	1.1	SRSF12	1.743589744
AP000487.6	2.72E-11	0.288617752	0.208	0.159	6.56E-07	1.1	AP000487.(	1.308176101
GHR	2.76E-11	0.296748641	0.118	0.08	6.66E-07	1.1	GHR	1.475
FAM221A	3.36E-11	0.102696792	0.038	0.018	8.11E-07	1.1	FAM221A	2.111111111
C5orf56	3.39E-11	0.244022369	0.125	0.086	8.18E-07	1.1	C5orf56	1.453488372
APTR	3.66E-11	0.198619438	0.089	0.056	8.84E-07	1.1	APTR	1.589285714
DYNC2LI1	3.97E-11	0.170269149	0.125	0.086	9.56E-07	1.1	DYNC2LI1	1.453488372
RAB1A	4.08E-11	0.29073396	0.481	0.453	9.83E-07	1.1	RAB1A	1.061810155
PPHLN1	4.09E-11	0.281310899	0.381	0.334	9.87E-07	1.1	PPHLN1	1.140718563
TBC1D22B	4.45E-11	0.269782445	0.208	0.16	1.07E-06	1.1	TBC1D22B	1.3
C8orf59	5.73E-11	0.251615518	0.332	0.282	1.38E-06	1.1	C8orf59	1.177304965
S100A13	6.52E-11	0.177945027	0.553	0.51	1.57E-06	1.1	S100A13	1.084313725

ZCCHC2	6.60E-11	0.305348539	0.272	0.225	1.59E-06	1.1	ZCCHC2	1.208888889
MAP3K14	7.20E-11	0.26434316	0.083	0.052	1.74E-06	1.1	MAP3K14	1.596153846
MMP7	7.24E-11	0.178704734	0.196	0.146	1.75E-06	1.1	MMP7	1.342465753
SCARB1	7.69E-11	0.113963986	0.055	0.03	1.85E-06	1.1	SCARB1	1.833333333
PAK3	1.09E-10	0.147191559	0.071	0.042	2.63E-06	1.1	PAK3	1.69047619
ASXL1	1.09E-10	0.227730788	0.304	0.252	2.64E-06	1.1	ASXL1	1.206349206
SPEN	1.18E-10	0.270208162	0.313	0.262	2.84E-06	1.1	SPEN	1.194656489
SMURF2	1.58E-10	0.342465784	0.331	0.287	3.80E-06	1.1	SMURF2	1.153310105
RUVBL1	1.61E-10	0.195562332	0.148	0.106	3.88E-06	1.1	RUVBL1	1.396226415
KDM3A	1.66E-10	0.200670499	0.148	0.106	4.01E-06	1.1	KDM3A	1.396226415
ARL8B	1.84E-10	0.287960932	0.314	0.268	4.43E-06	1.1	ARL8B	1.171641791
FAM49A	1.86E-10	0.123989	0.053	0.029	4.48E-06	1.1	FAM49A	1.827586207
KIF15	1.86E-10	0.131792811	0.024	0.009	4.48E-06	1.1	KIF15	2.666666667
TMEM71	2.32E-10	0.10592193	0.05	0.027	5.60E-06	1.1	TMEM71	1.851851852
ELK3	2.59E-10	0.220215009	0.153	0.112	6.24E-06	1.1	ELK3	1.366071429
PHACTR4	2.65E-10	0.324827821	0.368	0.327	6.40E-06	1.1	PHACTR4	1.125382263
FAXC	2.93E-10	0.134289855	0.043	0.022	7.06E-06	1.1	FAXC	1.954545455
VDAC3	2.93E-10	0.242523737	0.31	0.264	7.07E-06	1.1	VDAC3	1.174242424
SMR3B	3.05E-10	0.178191063	0.01	0.002	7.36E-06	1.1	SMR3B	5
EIF1AX	3.35E-10	0.258394357	0.284	0.236	8.09E-06	1.1	EIF1AX	1.203389831
RP11-659O3.1	4.31E-10	0.274786789	0.205	0.161	1.04E-05	1.1	RP11-659O	1.273291925
CTD-2515A14.1	4.92E-10	0.123901418	0.052	0.029	1.19E-05	1.1	CTD-2515A	1.793103448
FAR1	5.87E-10	0.198381598	0.126	0.089	1.41E-05	1.1	FAR1	1.415730337
RP11-122M14.3	6.95E-10	0.119411508	0.021	0.008	1.68E-05	1.1	RP11-122M	2.625
SCD5	7.06E-10	0.15967612	0.063	0.037	1.70E-05	1.1	SCD5	1.702702703
RPS26	8.20E-10	0.163776467	0.153	0.112	1.98E-05	1.1	RPS26	1.366071429
DDX18	8.95E-10	0.238224627	0.332	0.284	2.16E-05	1.1	DDX18	1.169014085
RP11-293M10.1	9.18E-10	0.158609635	0.071	0.044	2.21E-05	1.1	RP11-293M	1.613636364
ENO2	9.30E-10	0.129838006	0.072	0.044	2.24E-05	1.1	ENO2	1.636363636
PRSS8	9.40E-10	0.435276424	0.065	0.039	2.27E-05	1.1	PRSS8	1.666666667
ERRFI1	9.51E-10	0.263920397	0.574	0.537	2.29E-05	1.1	ERRFI1	1.068901304
ABCE1	1.22E-09	0.174684111	0.124	0.088	2.95E-05	1.1	ABCE1	1.409090909
MLF1	1.22E-09	0.252496847	0.29	0.242	2.95E-05	1.1	MLF1	1.198347107
MRPL24	1.26E-09	0.145437028	0.094	0.062	3.03E-05	1.1	MRPL24	1.516129032
GSTA4	1.44E-09	0.103958755	0.086	0.055	3.48E-05	1.1	GSTA4	1.563636364
RNF114	1.54E-09	0.224385865	0.163	0.123	3.71E-05	1.1	RNF114	1.325203252
CDC42	1.58E-09	0.182462068	0.722	0.716	3.80E-05	1.1	CDC42	1.008379888
NEK7	1.63E-09	0.275730522	0.215	0.172	3.92E-05	1.1	NEK7	1.25
NCL	1.71E-09	0.187944664	0.211	0.165	4.12E-05	1.1	NCL	1.278787879
MPDZ	1.74E-09	0.241894737	0.176	0.135	4.20E-05	1.1	MPDZ	1.303703704
NGFRAP1	1.79E-09	0.103106865	0.068	0.041	4.32E-05	1.1	NGFRAP1	1.658536585
SLC39A14	1.96E-09	0.22007926	0.241	0.194	4.73E-05	1.1	SLC39A14	1.242268041
CCT5	2.10E-09	0.191107592	0.342	0.291	5.07E-05	1.1	CCT5	1.175257732
PNPLA8	2.17E-09	0.379266355	0.312	0.276	5.23E-05	1.1	PNPLA8	1.130434783
TOX4	2.36E-09	0.222870437	0.218	0.176	5.69E-05	1.1	TOX4	1.238636364
CAPZA2	2.40E-09	0.27668748	0.346	0.304	5.79E-05	1.1	CAPZA2	1.138157895
NT5C3A	2.59E-09	0.245980839	0.198	0.155	6.23E-05	1.1	NT5C3A	1.277419355
PCBP2	2.92E-09	0.222135596	0.572	0.572	7.05E-05	1.1	PCBP2	1
MPP5	3.16E-09	0.271293084	0.251	0.207	7.61E-05	1.1	MPP5	1.212560386
ACTN1	3.17E-09	0.340933536	0.386	0.339	7.64E-05	1.1	ACTN1	1.138643068
RP1-292B18.4	3.55E-09	0.171328759	0.257	0.21	8.57E-05	1.1	RP1-292B1	1.223809524
CD55	3.67E-09	0.265751624	0.327	0.285	8.85E-05	1.1	CD55	1.147368421

SLC12A3	4.52E-09	0.103063607	0.06	0.035	0.000109054	1.1	SLC12A3	1.714285714
PRICKLE1	4.70E-09	0.232249372	0.078	0.05	0.000113442	1.1	PRICKLE1	1.56
EGLN1	5.79E-09	0.148755839	0.126	0.09	0.000139728	1.1	EGLN1	1.4
GIPC2	6.07E-09	0.138013286	0.057	0.034	0.00014647	1.1	GIPC2	1.676470588
GAS2L3	6.48E-09	0.101569926	0.049	0.028	0.000156343	1.1	GAS2L3	1.75
TJP2	6.64E-09	0.313607419	0.35	0.319	0.000160012	1.1	TJP2	1.097178683
MAX	6.98E-09	0.182899894	0.171	0.13	0.000168421	1.1	MAX	1.315384615
IQGAP1	7.93E-09	0.22916652	0.469	0.435	0.00019123	1.1	IQGAP1	1.07816092
BANF1	8.00E-09	0.203969308	0.218	0.177	0.000192926	1.1	BANF1	1.231638418
TRA2B	8.46E-09	0.273308461	0.252	0.21	0.000204068	1.1	TRA2B	1.2
ARHGEF38	8.89E-09	0.186283005	0.525	0.481	0.000214332	1.1	ARHGEF38	1.091476091
C12orf60	9.46E-09	0.210466681	0.102	0.071	0.000227989	1.1	C12orf60	1.436619718
HIP1R	9.76E-09	0.100306254	0.035	0.018	0.00023531	1.1	HIP1R	1.944444444
GLRX	9.80E-09	0.314128065	0.33	0.284	0.00023633	1.1	GLRX	1.161971831
FABP6	1.25E-08	0.194828317	0.06	0.036	0.000300512	1.1	FABP6	1.666666667
MAGT1	1.26E-08	0.199715985	0.218	0.175	0.000303651	1.1	MAGT1	1.245714286
SETD5	1.34E-08	0.218356498	0.546	0.526	0.000323144	1.1	SETD5	1.038022814
IMPDH2	1.37E-08	0.131323115	0.076	0.049	0.000330407	1.1	IMPDH2	1.551020408
NIFK	1.45E-08	0.168867226	0.101	0.07	0.000349709	1.1	NIFK	1.442857143
CTC-425F1.4	1.86E-08	0.193498386	0.107	0.075	0.000447558	1.1	CTC-425F1.	1.426666667
CLVS1	2.03E-08	0.134558148	0.091	0.062	0.00048923	1.1	CLVS1	1.467741935
PFKFB4	2.05E-08	0.156088176	0.06	0.037	0.0004948	1.1	PFKFB4	1.621621622
ANP32E	2.07E-08	0.173402311	0.113	0.081	0.000498851	1.1	ANP32E	1.395061728
PPIH	2.13E-08	0.153498623	0.128	0.094	0.000514619	1.1	PPIH	1.361702128
COPS4	2.15E-08	0.172909478	0.136	0.101	0.000517372	1.1	COPS4	1.346534653
CAMSAP1	2.21E-08	0.180848114	0.092	0.063	0.000533859	1.1	CAMSAP1	1.46031746
CCDC126	2.37E-08	0.150978473	0.103	0.072	0.000572602	1.1	CCDC126	1.430555556
SH3YL1	2.79E-08	0.223179416	0.241	0.2	0.000672115	1.1	SH3YL1	1.205
CALCOCO2	3.17E-08	0.228060626	0.29	0.248	0.000763921	1.1	CALCOCO2	1.169354839
LINC01235	3.18E-08	0.159636325	0.124	0.09	0.000766525	1.1	LINC01235	1.377777778
TANK	3.63E-08	0.310297118	0.49	0.473	0.000875974	1.1	TANK	1.035940803
NAA50	3.95E-08	0.225805875	0.258	0.217	0.000952597	1.1	NAA50	1.188940092
ADPRM	4.19E-08	0.144213787	0.089	0.061	0.001010167	1.1	ADPRM	1.459016393
RPP30	4.20E-08	0.14565087	0.105	0.074	0.001012136	1.1	RPP30	1.418918919
PFKFB3	4.34E-08	0.133137916	0.089	0.06	0.001046807	1.1	PFKFB3	1.483333333
EIF3J	4.40E-08	0.194311616	0.156	0.12	0.001060955	1.1	EIF3J	1.3
PLEKHG1	4.57E-08	0.197043701	0.134	0.1	0.001101596	1.1	PLEKHG1	1.34
FAM162A	5.29E-08	0.166309772	0.165	0.127	0.001275903	1.1	FAM162A	1.299212598
CSDE1	5.41E-08	0.176667547	0.569	0.547	0.001304131	1.1	CSDE1	1.040219378
FMR1	5.89E-08	0.145401718	0.106	0.075	0.001420546	1.1	FMR1	1.413333333
RASSF8	6.87E-08	0.204802271	0.137	0.103	0.00165651	1.1	RASSF8	1.330097087
SEC61G	7.42E-08	0.155184194	0.623	0.597	0.001789787	1.1	SEC61G	1.043551089
ARHGAP10	7.46E-08	0.241414363	0.15	0.116	0.001798789	1.1	ARHGAP10	1.293103448
MYNN	9.02E-08	0.14752861	0.098	0.069	0.002174105	1.1	MYNN	1.420289855
COPZ1	9.35E-08	0.21256512	0.355	0.319	0.002253994	1.1	COPZ1	1.112852665
DAPP1	1.14E-07	0.249625295	0.277	0.238	0.002739634	1.1	DAPP1	1.163865546
MYH9	1.21E-07	0.215936666	0.547	0.512	0.002911223	1.1	MYH9	1.068359375
PDGFC	1.27E-07	0.236765855	0.261	0.221	0.00306975	1.1	PDGFC	1.180995475
SBF2	1.46E-07	0.28581791	0.622	0.641	0.003525672	1.1	SBF2	0.970358814
ALDH7A1	1.47E-07	0.160070898	0.11	0.08	0.003534558	1.1	ALDH7A1	1.375
KIAA0226L	1.52E-07	0.126177524	0.073	0.048	0.003655038	1.1	KIAA0226L	1.520833333
RAB14	1.77E-07	0.202246392	0.164	0.129	0.004257111	1.1	RAB14	1.271317829

PIGA	1.77E-07	0.166459164	0.125	0.093	0.004259857	1.1	PIGA	1.344086022
LAMP2	1.83E-07	0.270900084	0.278	0.242	0.004420264	1.1	LAMP2	1.148760331
UBR5	1.97E-07	0.183330912	0.5	0.468	0.004746513	1.1	UBR5	1.068376068
SMC3	2.00E-07	0.147928559	0.131	0.099	0.00483299	1.1	SMC3	1.323232323
ZRANB1	2.29E-07	0.201576263	0.186	0.149	0.005511305	1.1	ZRANB1	1.248322148
USP38	2.35E-07	0.151799272	0.065	0.043	0.005655833	1.1	USP38	1.511627907
SRP72	2.43E-07	0.159225897	0.192	0.155	0.005847733	1.1	SRP72	1.238709677
HSD17B6	2.46E-07	0.209213401	0.032	0.017	0.005927094	1.1	HSD17B6	1.882352941
ARHGEF10L	2.63E-07	0.193617278	0.201	0.164	0.006345263	1.1	ARHGEF10I	1.225609756
AC008074.1	2.81E-07	0.107794804	0.062	0.04	0.006785502	1.1	AC008074.	1.55
MAP7D1	2.82E-07	0.20119399	0.186	0.15	0.006800163	1.1	MAP7D1	1.24
PPIL4	2.92E-07	0.18837321	0.162	0.127	0.007051836	1.1	PPIL4	1.275590551
CTR9	3.22E-07	0.162882547	0.113	0.083	0.007774688	1.1	CTR9	1.361445783
C5orf34	3.42E-07	0.105082453	0.077	0.052	0.008242018	1.1	C5orf34	1.480769231
BTG3	3.67E-07	0.187484808	0.213	0.176	0.008839164	1.1	BTG3	1.210227273
TTC37	3.86E-07	0.172573137	0.166	0.131	0.009302566	1.1	TTC37	1.267175573
C6orf48	4.10E-07	0.128472085	0.099	0.071	0.009885787	1.1	C6orf48	1.394366197
PA2G4	4.33E-07	0.117000802	0.126	0.094	0.010450455	1.1	PA2G4	1.340425532
GLIS3	4.37E-07	0.336262837	0.189	0.157	0.010537436	1.1	GLIS3	1.203821656
FTL	4.40E-07	0.138441645	0.556	0.529	0.010621678	1.1	FTL	1.051039698
SNRPB2	4.72E-07	0.193666252	0.378	0.341	0.011382048	1.1	SNRPB2	1.108504399
U2SURP	4.80E-07	0.209071819	0.268	0.235	0.011579351	1.1	U2SURP	1.140425532
GBE1	5.00E-07	0.39730191	0.384	0.357	0.012063049	1.1	GBE1	1.075630252
SRFBP1	5.06E-07	0.224543491	0.179	0.146	0.012201615	1.1	SRFBP1	1.226027397
RP11-445F6.2	5.23E-07	0.22810956	0.023	0.011	0.012615876	1.1	RP11-445F(	2.090909091
NIT2	5.37E-07	0.15050133	0.135	0.103	0.012958905	1.1	NIT2	1.310679612
PAFAH1B1	5.58E-07	0.256174694	0.396	0.367	0.013462494	1.1	PAFAH1B1	1.079019074
TAB2	5.62E-07	0.22788546	0.316	0.281	0.01354066	1.1	TAB2	1.12455516
RLIM	5.76E-07	0.130631413	0.082	0.057	0.013894294	1.1	RLIM	1.438596491
SNX2	6.65E-07	0.170941959	0.166	0.133	0.016040184	1.1	SNX2	1.248120301
RP11-398K22.12	6.98E-07	0.103355064	0.057	0.036	0.016834998	1.1	RP11-398K	1.583333333
MTDH	7.40E-07	0.192902842	0.331	0.292	0.017849357	1.1	MTDH	1.133561644
PJA2	7.52E-07	0.201960972	0.278	0.239	0.018122782	1.1	PJA2	1.163179916
ST6GALNAC5	7.56E-07	0.160369549	0.224	0.182	0.018233886	1.1	ST6GALNA(	1.230769231
KCNQ5	7.96E-07	0.146513106	0.071	0.048	0.019186085	1.1	KCNQ5	1.479166667
TCERG1	8.57E-07	0.149948046	0.17	0.135	0.020660668	1.1	TCERG1	1.259259259
CMTM7	8.73E-07	0.254216579	0.188	0.155	0.021046322	1.1	CMTM7	1.212903226
HDAC2	9.28E-07	0.188375752	0.213	0.179	0.022379697	1.1	HDAC2	1.189944134
RBMS2	9.45E-07	0.190289121	0.166	0.133	0.022777212	1.1	RBMS2	1.248120301
PFDN1	1.01E-06	0.162714317	0.258	0.218	0.024237738	1.1	PFDN1	1.183486239
GMPR2	1.10E-06	0.116396822	0.087	0.061	0.026622052	1.1	GMPR2	1.426229508
RP11-392O1.4	1.11E-06	0.133166817	0.038	0.022	0.026730184	1.1	RP11-392O	1.727272727
KPNB1	1.11E-06	0.252533946	0.425	0.404	0.026863848	1.1	KPNB1	1.051980198
HLA-B	1.19E-06	0.128548123	0.566	0.54	0.028626682	1.1	HLA-B	1.048148148
RND3	1.28E-06	0.253097077	0.381	0.348	0.030796418	1.1	RND3	1.094827586
PROL1	1.33E-06	0.189265943	0.02	0.009	0.031996006	1.1	PROL1	2.222222222
ADH5	1.33E-06	0.164366523	0.11	0.083	0.032143125	1.1	ADH5	1.325301205
RPL13A	1.34E-06	0.190479869	0.506	0.476	0.032277172	1.1	RPL13A	1.06302521
ETS1	1.38E-06	0.176826516	0.09	0.065	0.033367861	1.1	ETS1	1.384615385
HSPD1	1.46E-06	0.151019837	0.291	0.249	0.035109856	1.1	HSPD1	1.168674699
DRG1	1.47E-06	0.178803788	0.209	0.174	0.035530915	1.1	DRG1	1.201149425
SUMO1	1.84E-06	0.177243182	0.515	0.495	0.044263433	1.1	SUMO1	1.04040404

ATF2	2.00E-06	0.186829475	0.17	0.138	0.048189552	1.1	ATF2	1.231884058
MTMR1	2.07E-06	0.186598661	0.15	0.12	0.04996879	1.1	MTMR1	1.25
ANXA12	0	2.122008455	0.974	0.57	0	1.2	ANXA1	1.70877193
CLDN12	0	2.095629329	0.658	0.182	0	1.2	CLDN1	3.615384615
ADAMTS91	0	1.988700505	0.621	0.169	0	1.2	ADAMTS9	3.674556213
CALD11	0	1.962903275	0.77	0.248	0	1.2	CALD1	3.10483871
LIPH1	0	1.890244431	0.657	0.273	0	1.2	LIPH	2.406593407
RCAN11	0	1.678397456	0.808	0.352	0	1.2	RCAN1	2.295454545
SLPI	0	1.646534433	0.864	0.517	0	1.2	SLPI	1.671179884
CCL281	0	1.572377805	0.735	0.326	0	1.2	CCL28	2.254601227
GLIPR11	0	1.528737247	0.557	0.145	0	1.2	GLIPR1	3.84137931
ITGA21	0	1.421118923	0.737	0.353	0	1.2	ITGA2	2.087818697
BCL2A1	0	1.390616965	0.314	0.058	0	1.2	BCL2A1	5.413793103
ALDH1A31	0	1.341112332	0.652	0.236	0	1.2	ALDH1A3	2.762711864
GABRP1	0	1.289855248	0.594	0.23	0	1.2	GABRP	2.582608696
DAPP11	1.52E-306	1.263331754	0.55	0.209	3.67E-302	1.2	DAPP1	2.631578947
MAP3K131	5.32E-303	1.254517734	0.872	0.594	1.28E-298	1.2	MAP3K13	1.468013468
ST6GALNAC51	2.22E-291	1.353254199	0.477	0.156	5.36E-287	1.2	ST6GALNA(	3.057692308
B2M1	5.59E-284	0.884570667	0.988	0.97	1.35E-279	1.2	B2M	1.018556701
IGF2BP21	1.57E-274	1.065579955	0.586	0.238	3.78E-270	1.2	IGF2BP2	2.462184874
SVIL1	1.19E-273	1.031632085	0.86	0.556	2.87E-269	1.2	SVIL	1.54676259
PDE4B1	1.40E-263	0.956470207	0.864	0.48	3.38E-259	1.2	PDE4B	1.8
MAP1B1	6.06E-258	1.15221267	0.564	0.237	1.46E-253	1.2	MAP1B	2.379746835
RPL241	1.32E-249	0.747942645	0.963	0.911	3.18E-245	1.2	RPL24	1.057080132
RPS27A1	1.05E-248	0.661594764	0.991	0.969	2.52E-244	1.2	RPS27A	1.022703818
PTN1	7.58E-243	1.4331657	0.335	0.09	1.83E-238	1.2	PTN	3.722222222
KRT231	3.34E-239	1.236998557	0.384	0.124	8.05E-235	1.2	KRT23	3.096774194
KRT151	2.93E-233	0.996598515	0.428	0.143	7.07E-229	1.2	KRT15	2.993006993
RPL341	3.59E-230	0.628602641	0.989	0.977	8.65E-226	1.2	RPL34	1.012282497
NPM11	1.81E-224	0.822740119	0.913	0.693	4.37E-220	1.2	NPM1	1.317460317
RPL211	2.10E-224	0.73696513	0.962	0.86	5.05E-220	1.2	RPL21	1.118604651
ADAMTS9-AS2	1.91E-215	0.986284643	0.398	0.136	4.60E-211	1.2	ADAMTS9-,	2.926470588
SLC47A11	3.74E-204	0.688273903	0.947	0.89	9.03E-200	1.2	SLC47A1	1.064044944
MAP21	2.99E-203	0.812042075	0.286	0.077	7.20E-199	1.2	MAP2	3.714285714
USP531	7.81E-203	1.063025373	0.826	0.603	1.88E-198	1.2	USP53	1.369817579
ZPLD1	1.18E-199	1.053091083	0.226	0.051	2.86E-195	1.2	ZPLD1	4.431372549
ANKRD36C	2.67E-199	1.043048212	0.734	0.43	6.44E-195	1.2	ANKRD36C	1.706976744
SLC25A371	1.82E-197	1.001135832	0.68	0.354	4.39E-193	1.2	SLC25A37	1.920903955
RPL35A1	1.83E-196	0.593944493	0.987	0.951	4.42E-192	1.2	RPL35A	1.03785489
PLCB1	2.28E-195	1.115653902	0.474	0.198	5.50E-191	1.2	PLCB1	2.393939394
MYL12A	4.76E-195	0.782470489	0.945	0.859	1.15E-190	1.2	MYL12A	1.100116414
HSP90AB11	3.69E-192	0.748467228	0.948	0.828	8.89E-188	1.2	HSP90AB1	1.144927536
STX121	8.58E-192	1.02876339	0.574	0.295	2.07E-187	1.2	STX12	1.945762712
ELK31	6.47E-191	0.750904942	0.312	0.096	1.56E-186	1.2	ELK3	3.25
TPT1-AS11	1.32E-189	0.926088944	0.479	0.209	3.18E-185	1.2	TPT1-AS1	2.291866029
RPL301	5.94E-188	0.533405357	0.989	0.95	1.43E-183	1.2	RPL30	1.041052632
KRT6B	1.04E-187	1.007632718	0.201	0.042	2.50E-183	1.2	KRT6B	4.785714286
ZFAS11	4.73E-187	0.64907236	0.97	0.924	1.14E-182	1.2	ZFAS1	1.04978355
PI3	6.57E-187	1.997901817	0.358	0.125	1.59E-182	1.2	PI3	2.864
PALLD	1.67E-185	0.937602434	0.654	0.375	4.02E-181	1.2	PALLD	1.744
RPS71	9.02E-185	0.58331927	0.978	0.91	2.18E-180	1.2	RPS7	1.074725275
EMP11	1.41E-183	1.065701245	0.841	0.671	3.39E-179	1.2	EMP1	1.253353204

ITGB81	3.28E-183	0.899872401	0.748	0.47	7.91E-179	1.2	ITGB8	1.591489362
MET1	4.55E-183	0.898978919	0.471	0.201	1.10E-178	1.2	MET	2.343283582
ANXA21	2.31E-181	0.778025578	0.973	0.922	5.58E-177	1.2	ANXA2	1.055314534
DAPK21	1.84E-180	0.875017061	0.48	0.207	4.44E-176	1.2	DAPK2	2.31884058
COL6A21	1.51E-179	0.808411542	0.219	0.051	3.64E-175	1.2	COL6A2	4.294117647
TPM41	3.85E-170	0.935031298	0.615	0.348	9.29E-166	1.2	TPM4	1.767241379
MTHFD2L	7.36E-170	1.18599326	0.646	0.376	1.77E-165	1.2	MTHFD2L	1.718085106
SOD2	9.43E-170	0.710179704	0.955	0.758	2.27E-165	1.2	SOD2	1.259894459
MAMDC21	1.30E-169	0.709316301	0.948	0.84	3.14E-165	1.2	MAMDC2	1.128571429
PGK1	1.83E-168	0.819729723	0.776	0.532	4.41E-164	1.2	PGK1	1.458646617
RPL141	8.69E-168	0.500661695	0.989	0.957	2.10E-163	1.2	RPL14	1.033437827
RPS231	1.55E-162	0.488499733	0.99	0.966	3.73E-158	1.2	RPS23	1.02484472
PLEKHA61	2.07E-161	0.637343703	0.92	0.823	5.00E-157	1.2	PLEKHA6	1.117861482
RPS3A1	1.32E-160	0.600879524	0.952	0.893	3.18E-156	1.2	RPS3A	1.066069429
CTSV1	4.41E-160	1.003692251	0.294	0.096	1.06E-155	1.2	CTSV	3.0625
RPS61	1.74E-159	0.515151628	0.989	0.977	4.18E-155	1.2	RPS6	1.012282497
NFKBIZ1	2.21E-158	0.770713968	0.788	0.535	5.33E-154	1.2	NFKBIZ	1.472897196
RPL51	4.60E-157	0.519998644	0.977	0.938	1.11E-152	1.2	RPL5	1.041577825
CELF21	1.60E-156	0.726549326	0.308	0.105	3.87E-152	1.2	CELF2	2.933333333
MAP4K4	4.72E-155	0.875702089	0.457	0.213	1.14E-150	1.2	MAP4K4	2.145539906
RPL111	1.91E-154	0.501504465	0.987	0.956	4.60E-150	1.2	RPL11	1.032426778
CFLAR	3.72E-153	1.007159636	0.559	0.318	8.97E-149	1.2	CFLAR	1.757861635
NACA1	9.24E-152	0.499124766	0.968	0.902	2.23E-147	1.2	NACA	1.073170732
DSG31	2.34E-151	0.974061558	0.133	0.023	5.64E-147	1.2	DSG3	5.782608696
APOO1	6.91E-150	0.449604886	0.988	0.964	1.67E-145	1.2	APOO	1.024896266
SAA4	1.09E-148	0.812629455	0.313	0.112	2.63E-144	1.2	SAA4	2.794642857
EIF3E1	4.05E-146	0.680528104	0.785	0.553	9.76E-142	1.2	EIF3E	1.419529837
SUB11	7.51E-142	0.717171278	0.795	0.622	1.81E-137	1.2	SUB1	1.278135048
RSL24D11	7.94E-142	0.721523703	0.759	0.539	1.91E-137	1.2	RSL24D1	1.408163265
CDCP1	1.08E-140	0.816405995	0.496	0.256	2.61E-136	1.2	CDCP1	1.9375
GLRX1	2.73E-140	1.156680388	0.503	0.266	6.59E-136	1.2	GLRX	1.890977444
RPL321	6.04E-138	0.433332014	0.991	0.974	1.46E-133	1.2	RPL32	1.017453799
TIAM11	1.46E-137	0.899931502	0.454	0.22	3.52E-133	1.2	TIAM1	2.063636364
HCAR21	6.09E-137	0.875870497	0.261	0.087	1.47E-132	1.2	HCAR2	3
RPS15A1	1.34E-136	0.491360285	0.978	0.938	3.24E-132	1.2	RPS15A	1.042643923
FGF21	1.05E-134	0.570192916	0.15	0.032	2.54E-130	1.2	FGF2	4.6875
MYL6	1.68E-134	0.590583264	0.972	0.93	4.06E-130	1.2	MYL6	1.04516129
LUCAT1	3.44E-134	0.888455047	0.408	0.181	8.29E-130	1.2	LUCAT1	2.254143646
C5orf461	1.92E-133	0.701384799	0.283	0.1	4.62E-129	1.2	C5orf46	2.83
BIRC31	3.38E-133	0.812083941	0.815	0.591	8.14E-129	1.2	BIRC3	1.379018613
TSHZ21	1.62E-132	0.778300451	0.471	0.227	3.91E-128	1.2	TSHZ2	2.074889868
AKT31	3.33E-132	0.91267711	0.562	0.324	8.03E-128	1.2	AKT3	1.734567901
SEC61G1	2.38E-129	0.759738932	0.764	0.582	5.73E-125	1.2	SEC61G	1.312714777
ARHGEF101	4.94E-128	0.811224543	0.416	0.199	1.19E-123	1.2	ARHGEF10	2.090452261
PDLIM51	5.98E-127	0.694298398	0.864	0.73	1.44E-122	1.2	PDLIM5	1.183561644
RPS171	9.77E-124	0.450216663	0.969	0.938	2.35E-119	1.2	RPS17	1.033049041
MAML21	3.00E-123	0.651836964	0.672	0.389	7.23E-119	1.2	MAML2	1.727506427
PIGR1	1.26E-121	0.698077038	0.467	0.226	3.05E-117	1.2	PIGR	2.066371681
RPL311	1.18E-119	0.470989352	0.965	0.92	2.84E-115	1.2	RPL31	1.048913043
RASA21	3.83E-118	0.696535632	0.729	0.52	9.23E-114	1.2	RASA2	1.401923077
OOEP1	7.80E-118	0.421687392	0.99	0.963	1.88E-113	1.2	OOEP	1.028037383
EEF1A11	9.99E-118	0.438414642	0.987	0.973	2.41E-113	1.2	EEF1A1	1.014388489

EIF4A21	2.29E-113	0.667498937	0.821	0.684	5.52E-109	1.2	EIF4A2	1.200292398
C4orf31	2.13E-111	0.761642881	0.702	0.519	5.14E-107	1.2	C4orf3	1.352601156
HDAC9	4.91E-108	0.696490511	0.274	0.107	1.18E-103	1.2	HDAC9	2.560747664
SELK1	2.70E-106	0.665953949	0.761	0.596	6.51E-102	1.2	SELK	1.276845638
RPS241	2.14E-105	0.363640738	0.988	0.964	5.16E-101	1.2	RPS24	1.024896266
SRPK1	3.43E-105	0.736469771	0.658	0.469	8.28E-101	1.2	SRPK1	1.402985075
RPL42	1.52E-103	0.428749565	0.951	0.884	3.66E-99	1.2	RPL4	1.075791855
DSG21	6.48E-103	0.665909087	0.445	0.245	1.56E-98	1.2	DSG2	1.816326531
GLIS31	1.25E-101	0.793562338	0.321	0.143	3.02E-97	1.2	GLIS3	2.244755245
RP11-608O21.11	5.02E-101	0.61613477	0.725	0.519	1.21E-96	1.2	RP11-608O	1.396917148
WFDC2	7.41E-100	1.007946038	0.513	0.305	1.79E-95	1.2	WFDC2	1.681967213
SDC4	8.62E-100	0.936951348	0.508	0.311	2.08E-95	1.2	SDC4	1.633440514
MICAL31	1.63E-99	0.735869922	0.415	0.216	3.92E-95	1.2	MICAL3	1.921296296
SLC28A3	4.68E-99	0.679817147	0.341	0.159	1.13E-94	1.2	SLC28A3	2.144654088
EGFR	1.25E-98	0.697051048	0.345	0.162	3.01E-94	1.2	EGFR	2.12962963
MGP1	1.35E-98	0.844935606	0.896	0.811	3.26E-94	1.2	MGP	1.104808878
BACE21	1.85E-98	0.694884887	0.498	0.293	4.46E-94	1.2	BACE2	1.699658703
CRYAB1	1.15E-97	0.669103182	0.455	0.237	2.78E-93	1.2	CRYAB	1.919831224
PTRF1	2.50E-97	0.562563254	0.257	0.102	6.03E-93	1.2	PTRF	2.519607843
TNFAIP6	2.68E-97	1.184024117	0.377	0.186	6.46E-93	1.2	TNFAIP6	2.02688172
SDCBP	2.93E-97	0.738119062	0.771	0.602	7.07E-93	1.2	SDCBP	1.280730897
EIF4A11	6.15E-97	0.649248421	0.595	0.384	1.48E-92	1.2	EIF4A1	1.549479167
DDX211	2.77E-96	0.691292944	0.679	0.506	6.67E-92	1.2	DDX21	1.341897233
RPL231	5.76E-96	0.415633466	0.966	0.911	1.39E-91	1.2	RPL23	1.060373216
FAM172A1	1.12E-95	0.746012421	0.755	0.604	2.70E-91	1.2	FAM172A	1.25
SLC5A1	1.90E-94	0.478263937	0.149	0.042	4.58E-90	1.2	SLC5A1	3.547619048
MYO5B1	2.36E-94	0.555162935	0.782	0.585	5.69E-90	1.2	MYO5B	1.336752137
TMEM41A1	5.48E-94	0.604015254	0.284	0.125	1.32E-89	1.2	TMEM41A	2.272
EDN11	3.70E-93	0.632890255	0.277	0.118	8.93E-89	1.2	EDN1	2.347457627
INSR1	6.58E-93	0.764534368	0.7	0.54	1.59E-88	1.2	INSR	1.296296296
AC159540.1	8.14E-93	0.702653616	0.233	0.091	1.96E-88	1.2	AC159540.	2.56043956
CD551	3.35E-92	0.686607747	0.467	0.271	8.08E-88	1.2	CD55	1.723247232
SSR31	3.79E-92	0.6409612	0.613	0.427	9.13E-88	1.2	SSR3	1.43559719
RPL71	5.38E-92	0.397981335	0.958	0.868	1.30E-87	1.2	RPL7	1.103686636
PROM1	1.24E-91	0.620074936	0.404	0.209	3.00E-87	1.2	PROM1	1.933014354
CCL20	3.81E-91	1.786661768	0.338	0.167	9.18E-87	1.2	CCL20	2.023952096
FAM129A1	1.15E-90	0.813124775	0.449	0.255	2.77E-86	1.2	FAM129A	1.760784314
CMIP1	1.45E-90	0.654104253	0.441	0.249	3.49E-86	1.2	CMIP	1.771084337
LPIN11	2.08E-90	0.741919321	0.367	0.189	5.03E-86	1.2	LPIN1	1.941798942
MACC1	2.86E-90	0.771476705	0.595	0.395	6.89E-86	1.2	MACC1	1.506329114
ETS11	3.95E-90	0.469707835	0.174	0.056	9.52E-86	1.2	ETS1	3.107142857
ILF21	4.74E-90	0.635181259	0.605	0.412	1.14E-85	1.2	ILF2	1.468446602
CAST	6.71E-90	0.639159565	0.663	0.497	1.62E-85	1.2	CAST	1.334004024
LYN	7.98E-90	0.5416848	0.62	0.387	1.92E-85	1.2	LYN	1.602067183
GBP21	9.24E-90	0.637285442	0.757	0.6	2.23E-85	1.2	GBP2	1.261666667
ARL4C1	1.09E-89	0.524136962	0.237	0.093	2.63E-85	1.2	ARL4C	2.548387097
SLC6A141	5.14E-89	0.605650226	0.235	0.092	1.24E-84	1.2	SLC6A14	2.554347826
FAM177B	8.76E-89	0.807141448	0.667	0.474	2.11E-84	1.2	FAM177B	1.407172996
KLK51	5.93E-88	0.343048608	0.109	0.025	1.43E-83	1.2	KLK5	4.36
MYO3B1	6.01E-88	0.732742762	0.294	0.137	1.45E-83	1.2	MYO3B	2.145985401
SMS1	6.87E-88	0.720912034	0.479	0.288	1.66E-83	1.2	SMS	1.663194444
MYO1E	5.57E-87	0.712513656	0.64	0.459	1.34E-82	1.2	MYO1E	1.394335512

MMP71	7.10E-87	0.82033263	0.297	0.136	1.71E-82	1.2	MMP7	2.183823529
SIK31	2.88E-86	0.426215676	0.986	0.971	6.93E-82	1.2	SIK3	1.015447992
PLAUR	9.93E-86	0.730456546	0.347	0.176	2.40E-81	1.2	PLAUR	1.971590909
MAMLD11	6.54E-84	0.388269508	0.111	0.027	1.58E-79	1.2	MAMLD1	4.111111111
RPL36AL1	1.89E-83	0.433178685	0.933	0.882	4.55E-79	1.2	RPL36AL	1.057823129
PTGS2	2.93E-83	0.472223223	0.059	0.008	7.06E-79	1.2	PTGS2	7.375
LAMC2	4.00E-83	0.809152222	0.422	0.248	9.65E-79	1.2	LAMC2	1.701612903
LRRFIP21	7.20E-82	0.510788551	0.856	0.753	1.74E-77	1.2	LRRFIP2	1.136786189
CA8	7.58E-82	0.695406317	0.401	0.218	1.83E-77	1.2	CA8	1.839449541
PAM	2.75E-81	0.59563144	0.626	0.451	6.64E-77	1.2	PAM	1.388026608
IGFBP31	8.01E-81	0.557281116	0.153	0.048	1.93E-76	1.2	IGFBP3	3.1875
DMD1	1.84E-80	0.700126247	0.427	0.244	4.43E-76	1.2	DMD	1.75
CTTNBP2NL1	2.25E-80	0.54928748	0.366	0.195	5.42E-76	1.2	CTTNBP2N	1.876923077
SLC20A21	4.10E-80	0.693900452	0.493	0.309	9.88E-76	1.2	SLC20A2	1.595469256
RPS4X1	4.62E-80	0.330649228	0.992	0.978	1.11E-75	1.2	RPS4X	1.014314928
RCC11	6.70E-80	0.621314103	0.436	0.257	1.62E-75	1.2	RCC1	1.696498054
PLEKHA71	3.40E-79	0.294019482	0.988	0.979	8.20E-75	1.2	PLEKHA7	1.009193054
CAV1	1.81E-78	0.359475771	0.101	0.024	4.36E-74	1.2	CAV1	4.208333333
GBE11	7.62E-77	0.830817156	0.519	0.343	1.84E-72	1.2	GBE1	1.513119534
RPL411	9.68E-77	0.582966405	0.951	0.898	2.33E-72	1.2	RPL41	1.059020045
GRB141	3.55E-76	0.710573349	0.564	0.393	8.55E-72	1.2	GRB14	1.435114504
FOSL11	1.43E-75	0.495867339	0.181	0.066	3.44E-71	1.2	FOSL1	2.742424242
ANXA3	3.38E-75	0.64729075	0.522	0.345	8.15E-71	1.2	ANXA3	1.513043478
LOX1	8.47E-75	0.345318559	0.092	0.021	2.04E-70	1.2	LOX	4.380952381
DHFR1	1.17E-74	0.555963509	0.862	0.783	2.82E-70	1.2	DHFR	1.100893997
RPS251	3.49E-74	0.531515397	0.851	0.727	8.41E-70	1.2	RPS25	1.170563961
RPL7A1	1.76E-73	0.317076949	0.985	0.955	4.24E-69	1.2	RPL7A	1.031413613
DNAJC61	3.77E-73	0.446876739	0.153	0.052	9.08E-69	1.2	DNAJC6	2.942307692
PDE8A1	4.48E-73	0.604637335	0.493	0.311	1.08E-68	1.2	PDE8A	1.585209003
CRIM11	1.12E-72	0.623803043	0.596	0.424	2.70E-68	1.2	CRIM1	1.405660377
NKX3-11	1.24E-72	0.434009574	0.137	0.043	2.99E-68	1.2	NKX3-1	3.186046512
RPL391	2.49E-72	0.450475365	0.943	0.894	6.01E-68	1.2	RPL39	1.054809843
TSLP1	2.54E-72	0.341746294	0.112	0.031	6.13E-68	1.2	TSLP	3.612903226
LDHA1	1.81E-71	0.468451349	0.793	0.655	4.37E-67	1.2	LDHA	1.210687023
PPP1R14C1	2.32E-71	0.488132401	0.208	0.086	5.59E-67	1.2	PPP1R14C	2.418604651
FMNL2	2.41E-71	0.650102829	0.508	0.336	5.82E-67	1.2	FMNL2	1.511904762
CLMN1	3.69E-71	0.610824452	0.67	0.519	8.89E-67	1.2	CLMN	1.290944123
CAPN2	3.84E-71	0.59875806	0.448	0.277	9.26E-67	1.2	CAPN2	1.61732852
CCDC821	5.49E-71	0.495897637	0.29	0.143	1.32E-66	1.2	CCDC82	2.027972028
MBP	6.40E-71	0.511868697	0.234	0.104	1.54E-66	1.2	MBP	2.25
RPL271	1.10E-69	0.333173068	0.958	0.903	2.65E-65	1.2	RPL27	1.060908084
HSP90AA11	1.45E-69	0.602136928	0.914	0.848	3.49E-65	1.2	HSP90AA1	1.077830189
RASGEF1C1	2.28E-69	0.322088555	0.11	0.031	5.50E-65	1.2	RASGEF1C	3.548387097
HS3ST4	2.16E-68	0.671869451	0.328	0.174	5.22E-64	1.2	HS3ST4	1.885057471
INSIG21	2.64E-68	0.498907818	0.358	0.197	6.38E-64	1.2	INSIG2	1.817258883
CDK14	1.14E-67	0.599926325	0.481	0.311	2.75E-63	1.2	CDK14	1.546623794
BAIAP2L11	2.34E-67	0.318640581	0.971	0.922	5.65E-63	1.2	BAIAP2L1	1.053145336
RPS131	3.90E-67	0.44745786	0.84	0.689	9.40E-63	1.2	RPS13	1.2191582
L3MBTL4	5.11E-67	0.546150684	0.313	0.163	1.23E-62	1.2	L3MBTL4	1.920245399
CDK61	9.01E-67	0.503252364	0.251	0.118	2.17E-62	1.2	CDK6	2.127118644
EEF1B21	1.03E-66	0.398943046	0.853	0.716	2.48E-62	1.2	EEF1B2	1.191340782
VIM1	2.14E-66	0.525016182	0.242	0.112	5.15E-62	1.2	VIM	2.160714286

RPSA1	3.88E-66	0.36553073	0.9	0.801	9.35E-62	1.2	RPSA	1.123595506
TUBB6	8.98E-66	0.273053369	0.095	0.025	2.17E-61	1.2	TUBB6	3.8
TANK1	1.24E-65	0.508386189	0.626	0.458	3.00E-61	1.2	TANK	1.366812227
KLK10	1.78E-65	0.307540015	0.089	0.022	4.29E-61	1.2	KLK10	4.045454545
ANKRD36B	1.54E-64	0.47533874	0.17	0.066	3.71E-60	1.2	ANKRD36B	2.575757576
GPM6B1	1.91E-64	0.500231233	0.242	0.113	4.60E-60	1.2	GPM6B	2.14159292
TOMM71	1.96E-64	0.369657339	0.89	0.809	4.72E-60	1.2	TOMM7	1.100123609
HNRNPA11	6.18E-64	0.427949759	0.862	0.748	1.49E-59	1.2	HNRNPA1	1.152406417
MIR222HG1	3.12E-63	0.437658418	0.139	0.048	7.51E-59	1.2	MIR222HG	2.895833333
RPL371	4.75E-63	0.33680965	0.977	0.942	1.15E-58	1.2	RPL37	1.037154989
ANKRD36	9.43E-63	0.496590792	0.188	0.078	2.27E-58	1.2	ANKRD36	2.41025641
CMTM71	1.02E-62	0.531151712	0.284	0.146	2.46E-58	1.2	CMTM7	1.945205479
HMGA11	1.55E-62	0.599594949	0.182	0.075	3.75E-58	1.2	HMGA1	2.426666667
PLEKHH21	2.28E-62	0.356331372	0.155	0.058	5.50E-58	1.2	PLEKHH2	2.672413793
RBPMS1	6.67E-62	0.493667839	0.783	0.647	1.61E-57	1.2	RBPMS	1.210200927
EFNA5	4.09E-61	0.532967318	0.437	0.265	9.87E-57	1.2	EFNA5	1.649056604
RPS271	5.89E-61	0.680065437	0.753	0.625	1.42E-56	1.2	RPS27	1.2048
KLK71	7.53E-61	0.231744855	0.064	0.013	1.82E-56	1.2	KLK7	4.923076923
PGBD5	1.06E-60	0.293372823	0.108	0.033	2.55E-56	1.2	PGBD5	3.272727273
S100A21	1.07E-59	0.908101324	0.136	0.048	2.59E-55	1.2	S100A2	2.833333333
TBC1D3P1-DHX40P11	1.17E-59	0.478621391	0.72	0.575	2.82E-55	1.2	TBC1D3P1-	1.252173913
HADH1	1.45E-59	0.447403249	0.21	0.094	3.49E-55	1.2	HADH	2.234042553
SESTD11	2.38E-58	0.496254708	0.619	0.475	5.74E-54	1.2	SESTD1	1.303157895
TJP21	2.61E-58	0.610883124	0.456	0.308	6.29E-54	1.2	TJP2	1.480519481
NFATC21	4.25E-58	0.458374471	0.191	0.082	1.03E-53	1.2	NFATC2	2.329268293
LINC004861	4.38E-58	0.255126034	0.993	0.986	1.06E-53	1.2	LINC00486	1.007099391
PRNP	6.17E-58	0.449470698	0.224	0.105	1.49E-53	1.2	PRNP	2.133333333
CD59	8.83E-58	0.486157888	0.939	0.876	2.13E-53	1.2	CD59	1.071917808
KIZ-AS11	1.62E-57	0.485036917	0.894	0.838	3.90E-53	1.2	KIZ-AS1	1.066825776
TNFRSF11B	1.73E-57	0.467725245	0.159	0.062	4.17E-53	1.2	TNFRSF11E	2.564516129
RPL37A1	8.56E-57	0.309322609	0.989	0.973	2.06E-52	1.2	RPL37A	1.016443988
MECOM1	1.28E-56	0.396107451	0.863	0.798	3.09E-52	1.2	MECOM	1.081453634
BTF31	1.46E-56	0.289871628	0.943	0.883	3.53E-52	1.2	BTF3	1.06795017
TM2D2	3.00E-56	0.296073518	0.121	0.041	7.22E-52	1.2	TM2D2	2.951219512
THUMPD3-AS1	4.59E-56	0.515305322	0.355	0.212	1.11E-51	1.2	THUMPD3-	1.674528302
CLIC41	7.62E-56	0.514544484	0.685	0.549	1.84E-51	1.2	CLIC4	1.247723133
FOXN2	7.62E-56	0.427310904	0.165	0.067	1.84E-51	1.2	FOXN2	2.462686567
GLS	1.02E-55	0.570388229	0.325	0.187	2.46E-51	1.2	GLS	1.737967914
FAM78B	1.40E-55	0.336094179	0.097	0.029	3.38E-51	1.2	FAM78B	3.344827586
SERPINB51	2.36E-55	0.214347959	0.052	0.009	5.69E-51	1.2	SERPINB5	5.777777778
NCOA71	3.62E-55	0.525594924	0.597	0.424	8.72E-51	1.2	NCOA7	1.408018868
KRT16	6.66E-55	0.314643746	0.053	0.01	1.61E-50	1.2	KRT16	5.3
YWHAH1	1.00E-54	0.484468234	0.337	0.198	2.42E-50	1.2	YWHAH	1.702020202
CPAMD81	1.35E-54	0.413569436	0.146	0.056	3.27E-50	1.2	CPAMD8	2.607142857
PRKX	1.43E-54	0.429582412	0.2	0.092	3.44E-50	1.2	PRKX	2.173913043
TPM1	1.55E-54	0.509029322	0.646	0.514	3.73E-50	1.2	TPM1	1.256809339
GALNT31	1.13E-53	0.517057157	0.325	0.188	2.73E-49	1.2	GALNT3	1.728723404
COL6A1	1.63E-53	0.314284685	0.069	0.016	3.93E-49	1.2	COL6A1	4.3125
SAMD4A	1.75E-53	0.434934704	0.752	0.611	4.21E-49	1.2	SAMD4A	1.230769231
SLC39A141	2.08E-53	0.502125955	0.324	0.186	5.02E-49	1.2	SLC39A14	1.741935484
SPATA5	2.41E-53	0.454428876	0.176	0.077	5.80E-49	1.2	SPATA5	2.285714286
UAP1	3.35E-53	0.49911456	0.257	0.134	8.09E-49	1.2	UAP1	1.917910448

FRMD4A	5.93E-53	0.488950326	0.419	0.263	1.43E-48	1.2	FRMD4A	1.593155894
RPL61	1.03E-52	0.281867024	0.976	0.946	2.47E-48	1.2	RPL6	1.031712474
GPAT31	3.58E-52	0.285471047	0.116	0.04	8.64E-48	1.2	GPAT3	2.9
IFNAR2	3.72E-52	0.527732822	0.315	0.184	8.96E-48	1.2	IFNAR2	1.711956522
LAMB31	6.60E-52	1.000656629	0.353	0.229	1.59E-47	1.2	LAMB3	1.541484716
CHIC21	1.05E-51	0.450754338	0.274	0.149	2.54E-47	1.2	CHIC2	1.838926174
PTCHD11	1.14E-51	0.286288625	0.105	0.035	2.76E-47	1.2	PTCHD1	3
CTC-471J1.91	1.76E-51	0.476823584	0.728	0.627	4.25E-47	1.2	CTC-471J1.	1.16108453
LINC00969	1.99E-51	0.464246623	0.33	0.195	4.80E-47	1.2	LINC00969	1.692307692
NUDCD1	2.07E-51	0.362561285	0.168	0.072	4.98E-47	1.2	NUDCD1	2.333333333
DSC31	2.75E-51	0.291623881	0.06	0.013	6.63E-47	1.2	DSC3	4.615384615
SKP11	5.43E-51	0.438919915	0.863	0.801	1.31E-46	1.2	SKP1	1.077403246
CENPW1	1.15E-50	0.360784385	0.154	0.064	2.78E-46	1.2	CENPW	2.40625
TXNRD1	2.36E-50	0.738172819	0.507	0.365	5.68E-46	1.2	TXNRD1	1.389041096
CTSS	3.36E-50	0.421240167	0.244	0.127	8.11E-46	1.2	CTSS	1.921259843
TRAF1	3.45E-50	0.35433434	0.105	0.035	8.31E-46	1.2	TRAF1	3
RPL221	1.94E-49	0.367818592	0.755	0.641	4.69E-45	1.2	RPL22	1.177847114
PLSCR1	2.64E-49	0.459857352	0.31	0.182	6.38E-45	1.2	PLSCR1	1.703296703
SGK1	4.11E-49	0.441651859	0.201	0.096	9.91E-45	1.2	SGK1	2.09375
SH3PXD2A	6.27E-49	0.456624814	0.19	0.089	1.51E-44	1.2	SH3PXD2A	2.134831461
TUBA1C	1.08E-48	0.480614712	0.416	0.279	2.59E-44	1.2	TUBA1C	1.491039427
EPB421	1.31E-48	0.597192076	0.923	0.893	3.16E-44	1.2	EPB42	1.033594625
TPTEP1	1.35E-48	0.456237211	0.266	0.145	3.25E-44	1.2	TPTEP1	1.834482759
PCNXL21	7.90E-48	0.679346076	0.813	0.759	1.90E-43	1.2	PCNXL2	1.071146245
UQCRH1	1.31E-47	0.335417749	0.832	0.74	3.15E-43	1.2	UQCRH	1.124324324
DENND2C	2.18E-47	0.283397495	0.094	0.03	5.25E-43	1.2	DENND2C	3.133333333
BPGM	3.38E-47	0.421294367	0.176	0.081	8.14E-43	1.2	BPGM	2.172839506
TNFRSF11A1	4.04E-47	0.326749878	0.143	0.059	9.74E-43	1.2	TNFRSF11A	2.423728814
FEZ21	5.44E-47	0.574991247	0.462	0.328	1.31E-42	1.2	FEZ2	1.408536585
KRT14	6.20E-47	0.148655314	0.03	0.004	1.50E-42	1.2	KRT14	7.5
TMEM1591	6.38E-47	0.471710043	0.432	0.299	1.54E-42	1.2	TMEM159	1.444816054
SGMS21	8.93E-47	0.427458969	0.213	0.106	2.15E-42	1.2	SGMS2	2.009433962
RP11-66B24.4	1.90E-46	0.295625655	0.1	0.034	4.59E-42	1.2	RP11-66B2	2.941176471
CNIH4	2.31E-46	0.468340306	0.422	0.289	5.57E-42	1.2	CNIH4	1.460207612
RSL1D11	3.09E-46	0.399202289	0.582	0.439	7.44E-42	1.2	RSL1D1	1.325740319
RPL91	3.77E-46	0.477443819	0.666	0.56	9.09E-42	1.2	RPL9	1.189285714
SRRM11	3.97E-46	0.426509527	0.645	0.525	9.57E-42	1.2	SRRM1	1.228571429
ZMYM41	1.15E-45	0.297613178	0.876	0.81	2.77E-41	1.2	ZMYM4	1.081481481
BCAS31	1.17E-45	0.325425517	0.817	0.731	2.83E-41	1.2	BCAS3	1.117647059
MTHFD1L1	1.45E-45	0.534791913	0.329	0.206	3.49E-41	1.2	MTHFD1L	1.597087379
SEMA6A-AS1	1.66E-45	0.475111619	0.23	0.12	4.01E-41	1.2	SEMA6A-A	1.916666667
RP11-356C4.51	1.94E-45	0.577390743	0.251	0.139	4.67E-41	1.2	RP11-356C	1.805755396
KCTD91	2.66E-45	0.523791159	0.35	0.222	6.42E-41	1.2	KCTD9	1.576576577
NPAS2	2.76E-45	0.457669649	0.505	0.359	6.66E-41	1.2	NPAS2	1.406685237
TMEM271	3.04E-45	0.197798297	0.073	0.021	7.33E-41	1.2	TMEM27	3.476190476
RANBP17	3.85E-45	0.461956332	0.194	0.095	9.28E-41	1.2	RANBP17	2.042105263
TACSTD2	7.46E-45	0.343034763	0.832	0.72	1.80E-40	1.2	TACSTD2	1.155555556
COBL1	2.92E-44	0.540903325	0.471	0.328	7.05E-40	1.2	COBL	1.43597561
RASSF81	2.97E-44	0.39879818	0.196	0.097	7.17E-40	1.2	RASSF8	2.020618557
DDX241	4.51E-44	0.47006101	0.612	0.496	1.09E-39	1.2	DDX24	1.233870968
CYP24A1	5.26E-44	0.769236559	0.208	0.106	1.27E-39	1.2	CYP24A1	1.962264151
BDNF-AS1	1.05E-43	0.53914614	0.629	0.493	2.54E-39	1.2	BDNF-AS	1.275862069

STEAP1B1	1.31E-43	0.573674418	0.947	0.932	3.17E-39	1.2	STEAP1B	1.016094421
CMPK1	1.34E-43	0.472450988	0.323	0.197	3.23E-39	1.2	CMPK1	1.639593909
RASSF31	3.01E-43	0.380113277	0.218	0.113	7.26E-39	1.2	RASSF3	1.92920354
LINC014921	3.13E-43	0.311231996	0.162	0.074	7.54E-39	1.2	LINC01492	2.189189189
PTPN141	3.78E-43	0.361579136	0.428	0.289	9.11E-39	1.2	PTPN14	1.480968858
LINGO2	4.92E-43	0.395526617	0.087	0.029	1.19E-38	1.2	LINGO2	3
NMB1	5.02E-43	0.183365942	0.055	0.013	1.21E-38	1.2	NMB	4.230769231
C6orf132	6.31E-43	0.402753735	0.256	0.143	1.52E-38	1.2	C6orf132	1.79020979
PTEN1	8.80E-43	0.525605631	0.851	0.804	2.12E-38	1.2	PTEN	1.058457711
BRINP1	1.47E-42	0.7816635	0.191	0.095	3.55E-38	1.2	BRINP1	2.010526316
FAS1	1.62E-42	0.427710511	0.184	0.091	3.91E-38	1.2	FAS	2.021978022
WNT2B	2.27E-42	0.305361881	0.1	0.036	5.48E-38	1.2	WNT2B	2.777777778
XDH1	2.37E-42	0.442372767	0.126	0.051	5.71E-38	1.2	XDH	2.470588235
SON	4.76E-42	0.548465571	0.755	0.672	1.15E-37	1.2	SON	1.123511905
PABPC11	7.51E-42	0.366597878	0.796	0.723	1.81E-37	1.2	PABPC1	1.100968188
GJA11	1.01E-41	0.255010593	0.078	0.024	2.43E-37	1.2	GJA1	3.25
AC004231.2	1.31E-41	0.293597225	0.091	0.031	3.17E-37	1.2	AC004231.:	2.935483871
ADGRL3-AS11	1.42E-41	0.486086946	0.68	0.59	3.43E-37	1.2	ADGRL3-AS	1.152542373
CALU	1.61E-41	0.444201549	0.337	0.216	3.88E-37	1.2	CALU	1.560185185
ADAMTS9-AS1	1.75E-41	0.177240564	0.045	0.009	4.21E-37	1.2	ADAMTS9-/	5
CAMK2D1	3.43E-41	0.483746433	0.279	0.164	8.28E-37	1.2	CAMK2D	1.701219512
RRAGD1	7.91E-41	0.263958231	0.116	0.046	1.91E-36	1.2	RRAGD	2.52173913
BACH21	1.16E-40	0.408714805	0.461	0.324	2.79E-36	1.2	BACH2	1.422839506
KRT81	1.26E-40	0.36453931	0.062	0.017	3.05E-36	1.2	KRT81	3.647058824
ARRDC31	1.77E-40	0.339911228	0.525	0.37	4.27E-36	1.2	ARRDC3	1.418918919
RP11-314N13.101	1.81E-40	0.503506268	0.208	0.109	4.36E-36	1.2	RP11-314N	1.908256881
HLA-B1	4.27E-40	0.444221082	0.636	0.533	1.03E-35	1.2	HLA-B	1.193245779
NXN1	9.63E-40	0.355715178	0.177	0.087	2.32E-35	1.2	NXN	2.034482759
CCT21	1.13E-39	0.473620858	0.415	0.292	2.71E-35	1.2	CCT2	1.421232877
TLE41	1.24E-39	0.424802515	0.462	0.328	3.00E-35	1.2	TLE4	1.408536585
KPNB11	1.25E-39	0.436731254	0.515	0.395	3.01E-35	1.2	KPNB1	1.303797468
MLLT41	2.28E-39	0.30449407	0.755	0.633	5.50E-35	1.2	MLLT4	1.192733017
VNN1	2.86E-39	0.53319124	0.217	0.117	6.90E-35	1.2	VNN1	1.854700855
SLC35F3	4.88E-39	0.257763913	0.082	0.027	1.18E-34	1.2	SLC35F3	3.037037037
SEMA6A1	6.27E-39	0.480079883	0.336	0.211	1.51E-34	1.2	SEMA6A	1.592417062
ANXA11	7.60E-39	0.464569605	0.353	0.232	1.83E-34	1.2	ANXA11	1.521551724
ACTB	8.96E-39	0.297442689	0.737	0.634	2.16E-34	1.2	ACTB	1.162460568
NACA21	1.12E-38	0.383796411	0.586	0.456	2.71E-34	1.2	NACA2	1.285087719
HMOX1	1.22E-38	0.483335248	0.113	0.046	2.93E-34	1.2	HMOX1	2.456521739
CFL21	1.68E-38	0.316291922	0.148	0.068	4.06E-34	1.2	CFL2	2.176470588
VMP1	6.99E-38	0.382615019	0.801	0.742	1.69E-33	1.2	VMP1	1.079514825
RPS201	1.15E-37	0.272472723	0.955	0.921	2.76E-33	1.2	RPS20	1.036916395
NDRG11	2.20E-37	0.465515863	0.466	0.34	5.30E-33	1.2	NDRG1	1.370588235
ATP13A31	2.22E-37	0.447695647	0.372	0.251	5.35E-33	1.2	ATP13A3	1.482071713
EPS81	2.58E-37	0.501222586	0.431	0.309	6.23E-33	1.2	EPS8	1.394822006
CAV2	3.30E-37	0.244149445	0.098	0.038	7.96E-33	1.2	CAV2	2.578947368
RP11-631N16.2	3.70E-37	0.250881033	0.098	0.038	8.92E-33	1.2	RP11-631N	2.578947368
PFDN41	5.79E-37	0.391386298	0.453	0.324	1.40E-32	1.2	PFDN4	1.398148148
CARD161	5.89E-37	0.239733102	0.102	0.04	1.42E-32	1.2	CARD16	2.55
PNN1	1.06E-36	0.435636338	0.429	0.313	2.56E-32	1.2	PNN	1.370607029
ITGB11	1.14E-36	0.448420483	0.448	0.326	2.76E-32	1.2	ITGB1	1.374233129
SLC18B11	1.69E-36	0.393234923	0.186	0.097	4.08E-32	1.2	SLC18B1	1.917525773

RPL23A1	1.98E-36	0.456742845	0.684	0.564	4.77E-32	1.2	RPL23A	1.212765957
ABI11	2.58E-36	0.34813493	0.759	0.67	6.23E-32	1.2	ABI1	1.132835821
LINC00342	2.84E-36	0.251610531	0.092	0.034	6.85E-32	1.2	LINC00342	2.705882353
NDRG21	3.34E-36	0.458652626	0.283	0.174	8.06E-32	1.2	NDRG2	1.626436782
ZC3H12C	3.41E-36	0.382523883	0.201	0.108	8.22E-32	1.2	ZC3H12C	1.861111111
EIF3L1	4.98E-36	0.32635476	0.7	0.578	1.20E-31	1.2	EIF3L	1.211072664
FBLIM11	1.25E-35	0.325177597	0.188	0.099	3.02E-31	1.2	FBLIM1	1.898989899
UFM1	1.44E-35	0.436752333	0.432	0.314	3.48E-31	1.2	UFM1	1.375796178
MTRNR2L121	1.82E-35	0.434045922	0.328	0.214	4.39E-31	1.2	MTRNR2L1	1.53271028
RP4-678D15.1	1.96E-35	0.335075101	0.156	0.076	4.74E-31	1.2	RP4-678D1	2.052631579
TMEM217	2.02E-35	0.291433902	0.129	0.058	4.88E-31	1.2	TMEM217	2.224137931
NT5C2	2.38E-35	0.398267384	0.378	0.259	5.74E-31	1.2	NT5C2	1.459459459
CLINT11	3.16E-35	0.424423221	0.354	0.238	7.61E-31	1.2	CLINT1	1.487394958
DAPK11	4.79E-35	0.519669762	0.611	0.526	1.15E-30	1.2	DAPK1	1.161596958
RP11-111E14.11	5.87E-35	0.20048736	0.075	0.025	1.42E-30	1.2	RP11-111E	3
KANK1	5.98E-35	0.40126924	0.252	0.15	1.44E-30	1.2	KANK1	1.68
CORO1C1	8.40E-35	0.344477253	0.291	0.179	2.03E-30	1.2	CORO1C	1.625698324
MORF4L11	9.21E-35	0.350890834	0.71	0.623	2.22E-30	1.2	MORF4L1	1.13964687
COL22A1	1.09E-34	0.167378219	0.051	0.014	2.64E-30	1.2	COL22A1	3.642857143
RP11-795H16.31	1.14E-34	0.500986701	0.458	0.352	2.76E-30	1.2	RP11-795H	1.301136364
SPOCD1	1.84E-34	0.119255566	0.036	0.007	4.45E-30	1.2	SPOCD1	5.142857143
COG5	2.24E-34	0.43788392	0.543	0.437	5.40E-30	1.2	COG5	1.242562929
ALPL1	2.51E-34	0.280203434	0.075	0.026	6.06E-30	1.2	ALPL	2.884615385
GSAP	3.17E-34	0.345946291	0.165	0.084	7.65E-30	1.2	GSAP	1.964285714
PITPNC1	3.33E-34	0.583127552	0.265	0.165	8.02E-30	1.2	PITPNC1	1.606060606
B3GNT51	9.16E-34	0.250440951	0.117	0.051	2.21E-29	1.2	B3GNT5	2.294117647
GBP11	1.05E-33	0.293330203	0.179	0.093	2.52E-29	1.2	GBP1	1.924731183
RP5-945F2.31	1.42E-33	0.310196217	0.096	0.038	3.43E-29	1.2	RP5-945F2.	2.526315789
AMOTL11	1.62E-33	0.273999273	0.1	0.041	3.90E-29	1.2	AMOTL1	2.43902439
SLCO3A1	1.71E-33	0.457513541	0.28	0.176	4.13E-29	1.2	SLCO3A1	1.590909091
IFI16	1.77E-33	0.321328298	0.222	0.126	4.28E-29	1.2	IFI16	1.761904762
ADAM91	2.46E-33	0.340541607	0.668	0.558	5.94E-29	1.2	ADAM9	1.197132616
ZRANB2	3.03E-33	0.375447371	0.332	0.222	7.30E-29	1.2	ZRANB2	1.495495495
CCNB1IP11	6.30E-33	0.367825449	0.355	0.243	1.52E-28	1.2	CCNB1IP1	1.46090535
MT-ND41	6.44E-33	0.243935422	0.994	0.994	1.55E-28	1.2	MT-ND4	1
GPR87	7.09E-33	0.226995443	0.071	0.024	1.71E-28	1.2	GPR87	2.958333333
MTM1	7.55E-33	0.399668204	0.277	0.175	1.82E-28	1.2	MTM1	1.582857143
GRIN2A	9.17E-33	0.424120795	0.109	0.047	2.21E-28	1.2	GRIN2A	2.319148936
NHSL2	1.22E-32	0.404178291	0.379	0.27	2.94E-28	1.2	NHSL2	1.403703704
GTPBP4	1.87E-32	0.349674742	0.186	0.101	4.51E-28	1.2	GTPBP4	1.841584158
LIF	1.92E-32	0.23459222	0.056	0.017	4.62E-28	1.2	LIF	3.294117647
FRAS1	2.85E-32	0.293366821	0.088	0.034	6.88E-28	1.2	FRAS1	2.588235294
TES1	2.88E-32	0.367876288	0.417	0.3	6.95E-28	1.2	TES	1.39
CCT51	3.05E-32	0.492735287	0.391	0.287	7.35E-28	1.2	CCT5	1.362369338
PLAGL11	1.51E-31	0.214508515	0.089	0.035	3.64E-27	1.2	PLAGL1	2.542857143
PLEKHG11	1.83E-31	0.388968037	0.178	0.096	4.42E-27	1.2	PLEKHG1	1.854166667
LIMS11	2.26E-31	0.407129421	0.342	0.234	5.46E-27	1.2	LIMS1	1.461538462
GSTA11	2.77E-31	0.442223012	0.091	0.037	6.67E-27	1.2	GSTA1	2.459459459
SEC24A1	3.99E-31	0.321175066	0.268	0.166	9.63E-27	1.2	SEC24A	1.614457831
FSTL1	4.00E-31	0.253828393	0.136	0.066	9.64E-27	1.2	FSTL1	2.060606061
RBM8A	4.50E-31	0.376802767	0.529	0.42	1.08E-26	1.2	RBM8A	1.25952381
ZDHHC91	6.65E-31	0.286988723	0.161	0.084	1.60E-26	1.2	ZDHHC9	1.916666667

DEFB1	7.35E-31	0.740134379	0.55	0.428	1.77E-26	1.2	DEFB1	1.285046729
PSMB71	1.04E-30	0.407116031	0.546	0.443	2.51E-26	1.2	PSMB7	1.232505643
RPL381	1.18E-30	0.237234786	0.922	0.889	2.85E-26	1.2	RPL38	1.03712036
GCLM	2.83E-30	0.42220649	0.231	0.141	6.82E-26	1.2	GCLM	1.638297872
TNFRSF81	2.91E-30	0.315792893	0.161	0.085	7.03E-26	1.2	TNFRSF8	1.894117647
RBFOX21	3.03E-30	0.328111074	0.969	0.95	7.31E-26	1.2	RBFOX2	1.02
MFGE8	3.23E-30	0.474080164	0.167	0.089	7.79E-26	1.2	MFGE8	1.876404494
IPO51	3.82E-30	0.301042557	0.182	0.1	9.20E-26	1.2	IPO5	1.82
SNRPD11	3.96E-30	0.365988204	0.387	0.275	9.54E-26	1.2	SNRPD1	1.407272727
NUDT6	5.15E-30	0.143114	0.044	0.012	1.24E-25	1.2	NUDT6	3.666666667
SMC51	5.76E-30	0.374238241	0.408	0.295	1.39E-25	1.2	SMC5	1.383050847
KIAA0226L1	9.32E-30	0.238719583	0.104	0.046	2.25E-25	1.2	KIAA0226L	2.260869565
PFDN51	2.55E-29	0.24328977	0.887	0.842	6.16E-25	1.2	PFDN5	1.053444181
DTNB	5.29E-29	0.386622813	0.53	0.417	1.28E-24	1.2	DTNB	1.270983213
KLF51	6.29E-29	0.327776405	0.251	0.157	1.52E-24	1.2	KLF5	1.598726115
DNTTIP2	6.81E-29	0.383619045	0.252	0.161	1.64E-24	1.2	DNTTIP2	1.565217391
SAR1B1	6.83E-29	0.337990691	0.478	0.362	1.65E-24	1.2	SAR1B	1.320441989
PID11	9.02E-29	0.376871504	0.139	0.07	2.17E-24	1.2	PID1	1.985714286
RPS291	2.03E-28	0.251883489	0.969	0.92	4.90E-24	1.2	RPS29	1.05326087
CXCL16	2.50E-28	0.295589487	0.221	0.133	6.04E-24	1.2	CXCL16	1.661654135
EIF3M1	2.81E-28	0.323147609	0.433	0.322	6.77E-24	1.2	EIF3M	1.344720497
IGF2BP2-AS11	3.67E-28	0.162924863	0.058	0.019	8.84E-24	1.2	IGF2BP2-A!	3.052631579
RAB8B1	4.18E-28	0.368039319	0.225	0.139	1.01E-23	1.2	RAB8B	1.618705036
RIC31	5.20E-28	0.463957915	0.11	0.052	1.25E-23	1.2	RIC3	2.115384615
NEURL3	5.34E-28	0.221675063	0.071	0.027	1.29E-23	1.2	NEURL3	2.62962963
AC008074.3	5.45E-28	0.246798987	0.101	0.045	1.32E-23	1.2	AC008074.:	2.244444444
CTD-2020K17.1	5.68E-28	0.127949401	0.037	0.009	1.37E-23	1.2	CTD-2020K	4.111111111
HDAC7	6.42E-28	0.316713746	0.167	0.091	1.55E-23	1.2	HDAC7	1.835164835
SRSF31	7.09E-28	0.511720594	0.522	0.424	1.71E-23	1.2	SRSF3	1.231132075
ZNF83	8.83E-28	0.477224954	0.401	0.305	2.13E-23	1.2	ZNF83	1.314754098
TBCA1	9.18E-28	0.325605829	0.631	0.535	2.21E-23	1.2	TBCA	1.179439252
CCDC146	1.26E-27	0.35805964	0.216	0.131	3.04E-23	1.2	CCDC146	1.648854962
ARF41	1.26E-27	0.341949772	0.694	0.612	3.05E-23	1.2	ARF4	1.133986928
SMURF21	1.54E-27	0.403707571	0.383	0.282	3.71E-23	1.2	SMURF2	1.358156028
KCNH8	1.84E-27	0.219148261	0.061	0.021	4.43E-23	1.2	KCNH8	2.904761905
PSG41	1.92E-27	0.157678337	0.054	0.017	4.64E-23	1.2	PSG4	3.176470588
MCFD2	3.30E-27	0.287963581	0.204	0.121	7.96E-23	1.2	MCFD2	1.685950413
FAM134B	6.23E-27	0.3274891	0.161	0.089	1.50E-22	1.2	FAM134B	1.808988764
CH17-80A12.1	7.76E-27	0.123373401	0.032	0.007	1.87E-22	1.2	CH17-80A1	4.571428571
MYC1	8.34E-27	0.323168057	0.181	0.103	2.01E-22	1.2	MYC	1.757281553
SYNPO21	1.12E-26	0.388816877	0.169	0.094	2.69E-22	1.2	SYNPO2	1.79787234
ZFAND11	1.17E-26	0.3332465	0.266	0.175	2.82E-22	1.2	ZFAND1	1.52
SF3B61	1.22E-26	0.372495323	0.673	0.602	2.94E-22	1.2	SF3B6	1.117940199
ACTN41	1.33E-26	0.459408405	0.367	0.265	3.20E-22	1.2	ACTN4	1.38490566
IL20	1.43E-26	0.183950487	0.027	0.005	3.45E-22	1.2	IL20	5.4
BARX21	2.24E-26	0.430211146	0.342	0.242	5.41E-22	1.2	BARX2	1.41322314
SMARCA1	2.91E-26	0.277042644	0.181	0.104	7.02E-22	1.2	SMARCA1	1.740384615
PNRC1	3.01E-26	0.383420027	0.508	0.409	7.27E-22	1.2	PNRC1	1.24205379
BNIP31	4.63E-26	0.277402118	0.156	0.085	1.12E-21	1.2	BNIP3	1.835294118
ATP6V1B2	5.31E-26	0.297341024	0.149	0.08	1.28E-21	1.2	ATP6V1B2	1.8625
TFCP2L11	5.77E-26	0.371010288	0.155	0.085	1.39E-21	1.2	TFCP2L1	1.823529412
SNRPB21	5.78E-26	0.376267853	0.433	0.336	1.39E-21	1.2	SNRPB2	1.288690476

GARS	6.63E-26	0.348808977	0.263	0.171	1.60E-21	1.2	GARS	1.538011696
COTL1	8.01E-26	0.180097746	0.066	0.025	1.93E-21	1.2	COTL1	2.64
S100A11	1.50E-25	0.224495454	0.083	0.036	3.62E-21	1.2	S100A1	2.305555556
RP11-286E11.11	1.60E-25	0.194564533	0.076	0.031	3.86E-21	1.2	RP11-286E	2.451612903
PDCD10	2.09E-25	0.369899322	0.34	0.244	5.04E-21	1.2	PDCD10	1.393442623
NEAT1	2.59E-25	0.154923218	0.986	0.943	6.25E-21	1.2	NEAT1	1.045599152
MFHAS1	2.78E-25	0.213047292	0.075	0.03	6.70E-21	1.2	MFHAS1	2.5
BRK11	3.36E-25	0.302355358	0.681	0.599	8.11E-21	1.2	BRK1	1.136894825
SORBS21	3.37E-25	0.468209858	0.476	0.37	8.12E-21	1.2	SORBS2	1.286486486
COX7A2L1	5.74E-25	0.307300679	0.582	0.491	1.38E-20	1.2	COX7A2L	1.185336049
RNF1221	7.30E-25	0.162089949	0.077	0.032	1.76E-20	1.2	RNF122	2.40625
NCMAP1	7.49E-25	0.291971061	0.089	0.04	1.81E-20	1.2	NCMAP	2.225
SAV1	7.70E-25	0.36181009	0.382	0.287	1.86E-20	1.2	SAV1	1.331010453
PDP1	8.02E-25	0.242889255	0.11	0.054	1.94E-20	1.2	PDP1	2.037037037
CHD9	9.85E-25	0.32829515	0.494	0.393	2.37E-20	1.2	CHD9	1.256997455
HSD17B61	1.08E-24	0.34006815	0.048	0.015	2.59E-20	1.2	HSD17B6	3.2
KRT17	1.16E-24	0.263123122	0.063	0.023	2.79E-20	1.2	KRT17	2.739130435
EXT11	1.46E-24	0.488149791	0.571	0.49	3.51E-20	1.2	EXT1	1.165306122
ADGRA3	1.69E-24	0.306058537	0.139	0.075	4.07E-20	1.2	ADGRA3	1.853333333
GPC5	2.21E-24	0.32074832	0.078	0.033	5.33E-20	1.2	GPC5	2.363636364
ENO21	2.84E-24	0.171620258	0.093	0.042	6.85E-20	1.2	ENO2	2.214285714
P3H21	4.06E-24	0.299175535	0.121	0.062	9.78E-20	1.2	P3H2	1.951612903
FAM49B	7.07E-24	0.284167739	0.287	0.196	1.71E-19	1.2	FAM49B	1.464285714
SNHG81	7.76E-24	0.315275536	0.312	0.218	1.87E-19	1.2	SNHG8	1.431192661
ACTR31	1.29E-23	0.317632895	0.359	0.263	3.11E-19	1.2	ACTR3	1.365019011
SNHG121	2.18E-23	0.189813758	0.089	0.04	5.25E-19	1.2	SNHG12	2.225
AC129778.2	2.20E-23	0.104844756	0.029	0.007	5.29E-19	1.2	AC129778.	4.142857143
BLZF1	3.71E-23	0.272174646	0.142	0.079	8.93E-19	1.2	BLZF1	1.797468354
UGP2	5.44E-23	0.298985855	0.522	0.427	1.31E-18	1.2	UGP2	1.222482436
RP5-896L10.11	5.74E-23	0.28257157	0.314	0.222	1.38E-18	1.2	RP5-896L1(	1.414414414
IRS2	5.86E-23	0.297051902	0.414	0.313	1.41E-18	1.2	IRS2	1.322683706
CBLC	7.27E-23	0.24243902	0.119	0.061	1.75E-18	1.2	CBLC	1.950819672
CLDN16	9.03E-23	0.132858579	0.05	0.017	2.18E-18	1.2	CLDN16	2.941176471
FOXC11	1.32E-22	0.1741942	0.077	0.034	3.18E-18	1.2	FOXC1	2.264705882
LRRC49	1.48E-22	0.322159336	0.135	0.074	3.56E-18	1.2	LRRC49	1.824324324
PSMA31	1.54E-22	0.354795573	0.423	0.33	3.72E-18	1.2	PSMA3	1.281818182
ZNF8271	1.60E-22	0.255036294	0.18	0.108	3.86E-18	1.2	ZNF827	1.666666667
PDE9A	1.67E-22	0.177859505	0.082	0.036	4.02E-18	1.2	PDE9A	2.277777778
NHSL11	1.86E-22	0.550056817	0.25	0.17	4.49E-18	1.2	NHSL1	1.470588235
SAA2-SAA4	2.03E-22	0.252859756	0.267	0.174	4.89E-18	1.2	SAA2-SAA4	1.534482759
CEACAM1	2.10E-22	0.310007645	0.131	0.072	5.06E-18	1.2	CEACAM1	1.819444444
TAX1BP1	2.31E-22	0.280948049	0.679	0.619	5.56E-18	1.2	TAX1BP1	1.096930533
SACS	2.39E-22	0.184747324	0.107	0.054	5.76E-18	1.2	SACS	1.981481481
TRIM21	2.49E-22	0.280232143	0.247	0.164	6.01E-18	1.2	TRIM2	1.506097561
HPCAL1	2.51E-22	0.290520525	0.145	0.081	6.06E-18	1.2	HPCAL1	1.790123457
FBXO281	2.59E-22	0.324742779	0.225	0.147	6.23E-18	1.2	FBXO28	1.530612245
ZDHHC13	2.67E-22	0.245102974	0.134	0.073	6.44E-18	1.2	ZDHHC13	1.835616438
IL341	3.17E-22	0.386834783	0.137	0.076	7.64E-18	1.2	IL34	1.802631579
CLIP2	3.24E-22	0.302537254	0.202	0.127	7.81E-18	1.2	CLIP2	1.590551181
GNA121	3.60E-22	0.365774103	0.379	0.292	8.67E-18	1.2	GNA12	1.297945205
CHORDC1	3.61E-22	0.280572421	0.134	0.074	8.70E-18	1.2	CHORDC1	1.810810811
DIAPH31	3.61E-22	0.230452642	0.128	0.069	8.71E-18	1.2	DIAPH3	1.855072464

FGFBP1	5.17E-22	0.26316282	0.075	0.032	1.25E-17	1.2	FGFBP1	2.34375
PLS3	5.50E-22	0.208093207	0.164	0.095	1.33E-17	1.2	PLS3	1.726315789
ZCCHC21	6.62E-22	0.443173826	0.307	0.222	1.60E-17	1.2	ZCCHC2	1.382882883
C3	7.89E-22	0.23346258	0.219	0.14	1.90E-17	1.2	C3	1.564285714
KRT51	9.49E-22	0.131865674	0.04	0.012	2.29E-17	1.2	KRT5	3.333333333
NFIB	1.26E-21	0.372300002	0.788	0.736	3.03E-17	1.2	NFIB	1.070652174
EIF2A1	2.11E-21	0.27690749	0.397	0.302	5.08E-17	1.2	EIF2A	1.314569536
POU5F1	2.36E-21	0.117202122	0.036	0.01	5.69E-17	1.2	POU5F1	3.6
CEPT1	2.85E-21	0.288792963	0.254	0.172	6.86E-17	1.2	CEPT1	1.476744186
ERC1	3.00E-21	0.340271009	0.361	0.272	7.24E-17	1.2	ERC1	1.327205882
EHBP1	3.77E-21	0.388739201	0.323	0.236	9.08E-17	1.2	EHBP1	1.368644068
FYN1	5.11E-21	0.128614352	0.059	0.023	1.23E-16	1.2	FYN	2.565217391
RIF11	5.19E-21	0.315801289	0.304	0.218	1.25E-16	1.2	RIF1	1.394495413
DUSP141	5.26E-21	0.182266741	0.063	0.026	1.27E-16	1.2	DUSP14	2.423076923
PDLIM7	5.45E-21	0.119834683	0.052	0.019	1.32E-16	1.2	PDLIM7	2.736842105
MT-ND4L1	5.79E-21	0.375560162	0.519	0.448	1.40E-16	1.2	MT-ND4L	1.158482143
CFLAR-AS1	5.83E-21	0.220008256	0.094	0.046	1.41E-16	1.2	CFLAR-AS1	2.043478261
AK6	5.88E-21	0.289064955	0.239	0.159	1.42E-16	1.2	AK6	1.503144654
COPS21	6.11E-21	0.296534007	0.339	0.251	1.47E-16	1.2	COPS2	1.35059761
PHACTR41	7.22E-21	0.333534296	0.41	0.323	1.74E-16	1.2	PHACTR4	1.269349845
NFE2L3	7.50E-21	0.365859688	0.139	0.079	1.81E-16	1.2	NFE2L3	1.759493671
MTRNR2L13	7.63E-21	0.279749951	0.177	0.109	1.84E-16	1.2	MTRNR2L1	1.623853211
SSR21	9.31E-21	0.249957313	0.738	0.681	2.24E-16	1.2	SSR2	1.083700441
LINC011981	9.59E-21	0.278691756	0.225	0.146	2.31E-16	1.2	LINC01198	1.54109589
SYNJ2	9.71E-21	0.348813139	0.303	0.219	2.34E-16	1.2	SYNJ2	1.383561644
PPIA1	1.14E-20	0.22798113	0.832	0.802	2.74E-16	1.2	PPIA	1.037406484
PRSS81	1.19E-20	0.287966247	0.081	0.038	2.86E-16	1.2	PRSS8	2.131578947
SCD51	1.22E-20	0.203653014	0.078	0.036	2.94E-16	1.2	SCD5	2.166666667
PVT11	1.56E-20	0.446532323	0.422	0.338	3.77E-16	1.2	PVT1	1.24852071
CTD-3252C9.4	1.58E-20	0.212653127	0.123	0.067	3.82E-16	1.2	CTD-3252C	1.835820896
PRELID3B	1.85E-20	0.282128237	0.318	0.233	4.47E-16	1.2	PRELID3B	1.364806867
MAP4K51	1.90E-20	0.317779613	0.37	0.278	4.57E-16	1.2	MAP4K5	1.330935252
ALOX5	1.90E-20	0.276045678	0.17	0.104	4.59E-16	1.2	ALOX5	1.634615385
KCNQ51	2.20E-20	0.292813369	0.093	0.046	5.31E-16	1.2	KCNQ5	2.02173913
CYCS1	2.60E-20	0.336302552	0.586	0.515	6.28E-16	1.2	CYCS	1.137864078
KIF1B1	2.79E-20	0.393461053	0.484	0.397	6.72E-16	1.2	KIF1B	1.219143577
IAH1	3.48E-20	0.287041605	0.171	0.105	8.39E-16	1.2	IAH1	1.628571429
PITPNB	3.53E-20	0.299140078	0.392	0.302	8.50E-16	1.2	PITPNB	1.298013245
CALCOCO21	3.86E-20	0.313345288	0.327	0.245	9.30E-16	1.2	CALCOCO2	1.334693878
SET1	4.36E-20	0.380199828	0.482	0.402	1.05E-15	1.2	SET	1.199004975
KIF13B1	4.46E-20	0.350223891	0.336	0.25	1.07E-15	1.2	KIF13B	1.344
MPZL11	4.71E-20	0.32419838	0.455	0.367	1.13E-15	1.2	MPZL1	1.239782016
HSD11B1	5.44E-20	0.277250142	0.154	0.091	1.31E-15	1.2	HSD11B1	1.692307692
UBA521	5.46E-20	0.169064703	0.952	0.915	1.32E-15	1.2	UBA52	1.040437158
SPIRE11	5.83E-20	0.285487676	0.334	0.245	1.41E-15	1.2	SPIRE1	1.363265306
RGS10	6.06E-20	0.218394043	0.112	0.06	1.46E-15	1.2	RGS10	1.866666667
RAP2B1	7.19E-20	0.241558851	0.255	0.174	1.73E-15	1.2	RAP2B	1.465517241
ATP2B4	8.02E-20	0.38569	0.435	0.351	1.93E-15	1.2	ATP2B4	1.239316239
TBC1D22B1	1.03E-19	0.296105008	0.235	0.159	2.49E-15	1.2	TBC1D22B	1.477987421
CDC421	1.27E-19	0.24632268	0.772	0.711	3.06E-15	1.2	CDC42	1.085794655
NCAM1	1.35E-19	0.179298135	0.049	0.018	3.25E-15	1.2	NCAM1	2.722222222
FAM129B	1.46E-19	0.304007095	0.15	0.089	3.51E-15	1.2	FAM129B	1.685393258

CCT81	1.57E-19	0.317792223	0.357	0.275	3.78E-15	1.2	CCT8	1.298181818
RECK1	1.86E-19	0.161466323	0.059	0.024	4.47E-15	1.2	RECK	2.458333333
C5orf28	1.93E-19	0.264807	0.19	0.122	4.65E-15	1.2	C5orf28	1.557377049
BID	2.71E-19	0.282652451	0.196	0.127	6.55E-15	1.2	BID	1.543307087
DNTTIP1	3.04E-19	0.18730806	0.088	0.044	7.32E-15	1.2	DNTTIP1	2
EPPK1	3.64E-19	0.209661947	0.098	0.05	8.79E-15	1.2	EPPK1	1.96
ANKRD6	3.96E-19	0.219501256	0.091	0.046	9.55E-15	1.2	ANKRD6	1.97826087
RPL22L11	5.52E-19	0.330016149	0.501	0.414	1.33E-14	1.2	RPL22L1	1.210144928
NUB1	5.97E-19	0.35341246	0.261	0.186	1.44E-14	1.2	NUB1	1.403225806
G0S2	6.17E-19	0.153779981	0.051	0.02	1.49E-14	1.2	G0S2	2.55
PRPSAP11	6.99E-19	0.231948135	0.138	0.08	1.69E-14	1.2	PRPSAP1	1.725
LIPG	7.59E-19	0.151529841	0.046	0.017	1.83E-14	1.2	LIPG	2.705882353
LDHB1	7.80E-19	0.20211574	0.652	0.555	1.88E-14	1.2	LDHB	1.174774775
CBX6	7.83E-19	0.110156116	0.05	0.019	1.89E-14	1.2	CBX6	2.631578947
RAN1	7.84E-19	0.307008116	0.593	0.523	1.89E-14	1.2	RAN	1.133843212
PTBP21	8.57E-19	0.305981687	0.42	0.337	2.07E-14	1.2	PTBP2	1.246290801
MSN	8.83E-19	0.247295049	0.495	0.413	2.13E-14	1.2	MSN	1.198547215
CLDN41	1.21E-18	0.426736065	0.269	0.191	2.91E-14	1.2	CLDN4	1.408376963
ZNF6521	1.34E-18	0.28520129	0.522	0.438	3.23E-14	1.2	ZNF652	1.191780822
MAX1	2.11E-18	0.244850825	0.197	0.129	5.08E-14	1.2	MAX	1.527131783
RP11-511B23.21	2.17E-18	0.259842207	0.581	0.501	5.22E-14	1.2	RP11-511B	1.159680639
MAT2A	2.61E-18	0.205511409	0.092	0.048	6.30E-14	1.2	MAT2A	1.916666667
PNPLA81	2.71E-18	0.320452842	0.355	0.272	6.53E-14	1.2	PNPLA8	1.305147059
ADAM171	2.76E-18	0.34010259	0.456	0.375	6.65E-14	1.2	ADAM17	1.216
AC090498.11	3.32E-18	0.274558191	0.386	0.3	8.01E-14	1.2	AC090498.	1.286666667
PDE1C	3.84E-18	0.182809923	0.079	0.038	9.27E-14	1.2	PDE1C	2.078947368
GTF2B	3.99E-18	0.292826008	0.236	0.165	9.63E-14	1.2	GTF2B	1.43030303
SNX91	4.73E-18	0.363933568	0.491	0.417	1.14E-13	1.2	SNX9	1.177458034
OFD11	4.93E-18	0.366441911	0.319	0.244	1.19E-13	1.2	OFD1	1.307377049
OSBPL10	5.20E-18	0.268121175	0.16	0.099	1.25E-13	1.2	OSBPL10	1.616161616
IRF2	6.02E-18	0.317530812	0.301	0.225	1.45E-13	1.2	IRF2	1.337777778
ZNF562	6.73E-18	0.280231265	0.22	0.151	1.62E-13	1.2	ZNF562	1.456953642
STOX2	7.11E-18	0.272907503	0.144	0.087	1.72E-13	1.2	STOX2	1.655172414
ETV61	7.59E-18	0.389194851	0.628	0.572	1.83E-13	1.2	ETV6	1.097902098
TAGLN	8.03E-18	0.462358678	0.035	0.011	1.94E-13	1.2	TAGLN	3.181818182
RNF19A1	1.29E-17	0.404261279	0.621	0.57	3.10E-13	1.2	RNF19A	1.089473684
YWHAZ	1.33E-17	0.254732824	0.664	0.599	3.20E-13	1.2	YWHAZ	1.10851419
BMS1P14	1.40E-17	0.11344049	0.041	0.015	3.37E-13	1.2	BMS1P14	2.733333333
SNRPG	1.40E-17	0.333120914	0.578	0.512	3.38E-13	1.2	SNRPG	1.12890625
TPI1	1.70E-17	0.322225386	0.433	0.352	4.09E-13	1.2	TPI1	1.230113636
POU2F3	1.99E-17	0.262685465	0.17	0.108	4.79E-13	1.2	POU2F3	1.574074074
RAPGEF5	2.05E-17	0.358043878	0.413	0.332	4.95E-13	1.2	RAPGEF5	1.243975904
FRMD5	2.12E-17	0.385948701	0.192	0.128	5.10E-13	1.2	FRMD5	1.5
B4GALT5	2.21E-17	0.32124809	0.379	0.299	5.32E-13	1.2	B4GALT5	1.267558528
INADL	2.71E-17	0.193184591	0.876	0.815	6.53E-13	1.2	INADL	1.074846626
GCNT11	2.73E-17	0.190795138	0.099	0.053	6.58E-13	1.2	GCNT1	1.867924528
STX7	3.21E-17	0.242292776	0.182	0.118	7.73E-13	1.2	STX7	1.542372881
RPS261	3.62E-17	0.247515703	0.173	0.111	8.72E-13	1.2	RPS26	1.558558559
USP44	3.76E-17	0.104019851	0.041	0.015	9.07E-13	1.2	USP44	2.733333333
TGFA	4.69E-17	0.193702761	0.114	0.064	1.13E-12	1.2	TGFA	1.78125
RDX1	5.25E-17	0.267964901	0.218	0.151	1.27E-12	1.2	RDX	1.443708609
FBXO11	6.32E-17	0.306393916	0.467	0.386	1.52E-12	1.2	FBXO11	1.20984456

ENO11	6.78E-17	0.334319991	0.462	0.375	1.63E-12	1.2	ENO1	1.232
MIR4435-2HG1	6.92E-17	0.311998433	0.565	0.498	1.67E-12	1.2	MIR4435-2	1.134538153
RP11-7K24.3	8.30E-17	0.108273743	0.038	0.014	2.00E-12	1.2	RP11-7K24	2.714285714
SRFBP11	8.61E-17	0.263667797	0.21	0.143	2.08E-12	1.2	SRFBP1	1.468531469
GABRE	9.85E-17	0.142608539	0.061	0.028	2.37E-12	1.2	GABRE	2.178571429
MFSD4	1.13E-16	0.108521773	0.045	0.017	2.72E-12	1.2	MFSD4	2.647058824
IL15RA	1.13E-16	0.176190684	0.064	0.03	2.73E-12	1.2	IL15RA	2.133333333
PRKCI1	1.27E-16	0.262889773	0.246	0.174	3.07E-12	1.2	PRKCI	1.413793103
LINC001521	1.33E-16	0.284432663	0.396	0.316	3.20E-12	1.2	LINC00152	1.253164557
PRDX1	1.49E-16	0.36434057	0.732	0.679	3.58E-12	1.2	PRDX1	1.078055965
SPTLC2	1.61E-16	0.241715979	0.214	0.146	3.89E-12	1.2	SPTLC2	1.465753425
MT-CO21	1.67E-16	0.153650623	0.987	0.986	4.04E-12	1.2	MT-CO2	1.001014199
PM20D2	2.00E-16	0.228250383	0.138	0.085	4.83E-12	1.2	PM20D2	1.623529412
BAG51	2.02E-16	0.204226658	0.145	0.089	4.87E-12	1.2	BAG5	1.629213483
RHOA	2.09E-16	0.30398954	0.484	0.414	5.03E-12	1.2	RHOA	1.169082126
MEX3A	2.14E-16	0.153829894	0.075	0.037	5.16E-12	1.2	MEX3A	2.027027027
WFDC3	2.15E-16	0.21829532	0.107	0.061	5.19E-12	1.2	WFDC3	1.754098361
FRK	2.30E-16	0.253825419	0.198	0.133	5.56E-12	1.2	FRK	1.488721805
CXCL8	2.39E-16	1.233193598	0.501	0.441	5.75E-12	1.2	CXCL8	1.136054422
LAMC1	3.07E-16	0.294851271	0.33	0.255	7.40E-12	1.2	LAMC1	1.294117647
MTRNR2L81	3.18E-16	0.198865877	0.201	0.134	7.67E-12	1.2	MTRNR2L8	1.5
STAC	3.78E-16	0.138785629	0.05	0.021	9.13E-12	1.2	STAC	2.380952381
ACSL41	3.93E-16	0.168304548	0.092	0.049	9.48E-12	1.2	ACSL4	1.87755102
ARHGAP101	3.97E-16	0.304477026	0.173	0.114	9.57E-12	1.2	ARHGAP10	1.51754386
JPH2	5.10E-16	0.119073087	0.057	0.025	1.23E-11	1.2	JPH2	2.28
ZNF90	6.21E-16	0.28619407	0.126	0.075	1.50E-11	1.2	ZNF90	1.68
CDH3	7.16E-16	0.223828698	0.18	0.118	1.73E-11	1.2	CDH3	1.525423729
FAM49A1	7.46E-16	0.137132895	0.062	0.029	1.80E-11	1.2	FAM49A	2.137931034
WWTR11	8.19E-16	0.275476009	0.417	0.339	1.97E-11	1.2	WWTR1	1.230088496
HOTAIRM11	8.60E-16	0.134894376	0.066	0.032	2.07E-11	1.2	HOTAIRM1	2.0625
KRT191	8.60E-16	0.401028325	0.359	0.28	2.07E-11	1.2	KRT19	1.282142857
ZBTB38	8.82E-16	0.277026427	0.396	0.318	2.13E-11	1.2	ZBTB38	1.245283019
TAF1D1	1.02E-15	0.254818533	0.341	0.261	2.45E-11	1.2	TAF1D	1.30651341
POMP	1.46E-15	0.295582815	0.677	0.628	3.52E-11	1.2	POMP	1.078025478
ATP5L1	1.52E-15	0.174593968	0.833	0.808	3.67E-11	1.2	ATP5L	1.030940594
SGPP2	1.93E-15	0.254993179	0.163	0.106	4.64E-11	1.2	SGPP2	1.537735849
DEGS1	1.95E-15	0.192424611	0.103	0.058	4.70E-11	1.2	DEGS1	1.775862069
PTTG11	2.11E-15	0.160712953	0.06	0.028	5.08E-11	1.2	PTTG1	2.142857143
RP11-37B2.1	2.17E-15	0.408837116	0.287	0.221	5.24E-11	1.2	RP11-37B2	1.298642534
CSNK1E	2.70E-15	0.316866458	0.291	0.222	6.52E-11	1.2	CSNK1E	1.310810811
GATA6	2.75E-15	0.165402641	0.072	0.036	6.64E-11	1.2	GATA6	2
PSMC1	2.90E-15	0.309614985	0.345	0.273	7.00E-11	1.2	PSMC1	1.263736264
B4GALNT3	3.24E-15	0.177737511	0.077	0.039	7.81E-11	1.2	B4GALNT3	1.974358974
SSBP1	3.41E-15	0.246161332	0.431	0.354	8.22E-11	1.2	SSBP1	1.217514124
DTX2	3.46E-15	0.188030094	0.109	0.063	8.33E-11	1.2	DTX2	1.73015873
ATP11B1	3.65E-15	0.21861088	0.213	0.147	8.81E-11	1.2	ATP11B	1.448979592
SKAP2	3.89E-15	0.262591208	0.258	0.189	9.39E-11	1.2	SKAP2	1.365079365
MYRFL	4.01E-15	0.231814402	0.117	0.07	9.67E-11	1.2	MYRFL	1.671428571
STK10	4.07E-15	0.179549312	0.098	0.055	9.81E-11	1.2	STK10	1.781818182
ST6GAL1	4.24E-15	0.25682824	0.321	0.244	1.02E-10	1.2	ST6GAL1	1.31557377
FXR11	5.45E-15	0.222621157	0.423	0.344	1.31E-10	1.2	FXR1	1.229651163
TRMT10C	5.79E-15	0.214213754	0.137	0.086	1.40E-10	1.2	TRMT10C	1.593023256

PDZD21	5.88E-15	0.255127955	0.538	0.466	1.42E-10	1.2	PDZD2	1.154506438
PELI11	6.70E-15	0.269443426	0.399	0.323	1.62E-10	1.2	PELI1	1.235294118
EIF2S11	7.08E-15	0.338264504	0.257	0.194	1.71E-10	1.2	EIF2S1	1.324742268
CCL4	7.68E-15	1.133134161	0.085	0.046	1.85E-10	1.2	CCL4	1.847826087
S100B1	8.28E-15	0.116192243	0.05	0.022	2.00E-10	1.2	S100B	2.272727273
SASH11	9.38E-15	0.261534338	0.217	0.153	2.26E-10	1.2	SASH1	1.418300654
HEATR9	1.04E-14	0.124974409	0.065	0.032	2.51E-10	1.2	HEATR9	2.03125
SLC1A5	1.19E-14	0.135835208	0.063	0.031	2.86E-10	1.2	SLC1A5	2.032258065
NLGN4X	1.24E-14	0.204908039	0.08	0.043	3.00E-10	1.2	NLGN4X	1.860465116
MAPKBP11	1.25E-14	0.219320622	0.117	0.07	3.02E-10	1.2	MAPKBP1	1.671428571
UBASH3B	1.29E-14	0.175015266	0.083	0.044	3.12E-10	1.2	UBASH3B	1.886363636
EPB41L3	1.30E-14	0.124933506	0.039	0.015	3.13E-10	1.2	EPB41L3	2.6
PNISR	1.43E-14	0.204814715	0.737	0.674	3.44E-10	1.2	PNISR	1.09347181
DAAM11	1.48E-14	0.289745785	0.561	0.504	3.56E-10	1.2	DAAM1	1.113095238
KIAA0355	1.62E-14	0.215395024	0.16	0.104	3.92E-10	1.2	KIAA0355	1.538461538
VPS291	2.23E-14	0.220726885	0.392	0.314	5.38E-10	1.2	VPS29	1.248407643
SLC15A4	2.79E-14	0.153989678	0.091	0.051	6.74E-10	1.2	SLC15A4	1.784313725
CEACAM19	2.89E-14	0.139277528	0.062	0.03	6.97E-10	1.2	CEACAM19	2.066666667
BBOX11	2.91E-14	0.211589859	0.179	0.119	7.03E-10	1.2	BBOX1	1.504201681
SUPT3H	3.04E-14	0.250107877	0.161	0.106	7.34E-10	1.2	SUPT3H	1.518867925
SDCBP2	3.07E-14	0.102221515	0.039	0.015	7.39E-10	1.2	SDCBP2	2.6
UBE2N	3.24E-14	0.265042611	0.263	0.199	7.82E-10	1.2	UBE2N	1.32160804
MINK1	3.26E-14	0.210086135	0.147	0.095	7.87E-10	1.2	MINK1	1.547368421
MPZL2	3.50E-14	0.184026516	0.129	0.081	8.43E-10	1.2	MPZL2	1.592592593
SPATS2L	3.51E-14	0.294278471	0.302	0.232	8.46E-10	1.2	SPATS2L	1.301724138
PRKCA1	3.56E-14	0.19086879	0.107	0.063	8.57E-10	1.2	PRKCA	1.698412698
KARS1	4.03E-14	0.240948935	0.311	0.241	9.71E-10	1.2	KARS	1.290456432
TMEM51	4.14E-14	0.231773032	0.206	0.146	9.98E-10	1.2	TMEM51	1.410958904
RNF19B	4.88E-14	0.29812728	0.196	0.138	1.18E-09	1.2	RNF19B	1.420289855
KCNK1	5.43E-14	0.245813172	0.215	0.153	1.31E-09	1.2	KCNK1	1.405228758
FAM60A1	5.56E-14	0.227223106	0.377	0.304	1.34E-09	1.2	FAM60A	1.240131579
RP11-231C18.3	7.39E-14	0.252565119	0.226	0.163	1.78E-09	1.2	RP11-231C	1.386503067
MIR155HG1	7.90E-14	0.100878456	0.027	0.009	1.90E-09	1.2	MIR155HG	3
HUWE1	8.28E-14	0.296413167	0.333	0.267	2.00E-09	1.2	HUWE1	1.247191011
PHLPP11	8.35E-14	0.301706018	0.466	0.393	2.01E-09	1.2	PHLPP1	1.185750636
GABPB1-AS1	8.43E-14	0.263964696	0.193	0.135	2.03E-09	1.2	GABPB1-AS	1.42962963
FAM157C	8.54E-14	0.19903361	0.127	0.08	2.06E-09	1.2	FAM157C	1.5875
XPNPEP1	8.89E-14	0.188720036	0.127	0.079	2.14E-09	1.2	XPNPEP1	1.607594937
RPS31	1.10E-13	0.186606664	0.914	0.864	2.66E-09	1.2	RPS3	1.05787037
GNL31	1.18E-13	0.274360446	0.305	0.241	2.85E-09	1.2	GNL3	1.265560166
FAM222B	1.27E-13	0.242483439	0.269	0.203	3.06E-09	1.2	FAM222B	1.325123153
SVIL-AS1	1.34E-13	0.174987242	0.153	0.1	3.23E-09	1.2	SVIL-AS1	1.53
KDM7A1	1.46E-13	0.270300816	0.339	0.269	3.51E-09	1.2	KDM7A	1.260223048
SPDYE16	1.55E-13	0.163980357	0.1	0.059	3.75E-09	1.2	SPDYE16	1.694915254
NNT	1.58E-13	0.221969607	0.1	0.059	3.81E-09	1.2	NNT	1.694915254
NDUFS51	1.59E-13	0.186650146	0.808	0.769	3.83E-09	1.2	NDUFS5	1.050715215
EYA2	1.74E-13	0.249472023	0.154	0.102	4.21E-09	1.2	EYA2	1.509803922
SERTAD21	1.84E-13	0.193635917	0.225	0.16	4.43E-09	1.2	SERTAD2	1.40625
LHFPL21	1.88E-13	0.157110262	0.366	0.287	4.53E-09	1.2	LHFPL2	1.275261324
SEC31A	1.88E-13	0.260411497	0.462	0.391	4.54E-09	1.2	SEC31A	1.181585678
MCAM1	1.94E-13	0.103550529	0.038	0.015	4.68E-09	1.2	MCAM	2.533333333
ARHGAP171	2.30E-13	0.207636221	0.218	0.156	5.56E-09	1.2	ARHGAP17	1.397435897

SNRPE1	2.73E-13	0.215731183	0.581	0.516	6.58E-09	1.2	SNRPE	1.125968992
CHCHD31	2.98E-13	0.229483961	0.857	0.835	7.18E-09	1.2	CHCHD3	1.026347305
VDAC31	2.99E-13	0.257209859	0.33	0.263	7.20E-09	1.2	VDAC3	1.254752852
PCSK51	4.63E-13	0.186931627	0.062	0.031	1.12E-08	1.2	PCSK5	2
PPFIA11	4.66E-13	0.2881694	0.3	0.235	1.12E-08	1.2	PPFIA1	1.276595745
TCP11	4.72E-13	0.219311475	0.224	0.163	1.14E-08	1.2	TCP1	1.374233129
ADRBK2	4.89E-13	0.196052808	0.124	0.078	1.18E-08	1.2	ADRBK2	1.58974359
SOS2	5.12E-13	0.286581551	0.483	0.42	1.24E-08	1.2	SOS2	1.15
LRRK1	6.45E-13	0.175377482	0.092	0.053	1.55E-08	1.2	LRRK1	1.735849057
TRAF3IP2	6.74E-13	0.261401523	0.175	0.122	1.62E-08	1.2	TRAF3IP2	1.43442623
EZR	6.86E-13	0.227544926	0.445	0.372	1.65E-08	1.2	EZR	1.196236559
GGACT	7.04E-13	0.219183163	0.193	0.137	1.70E-08	1.2	GGACT	1.408759124
SFT2D21	7.23E-13	0.211138301	0.183	0.128	1.74E-08	1.2	SFT2D2	1.4296875
FAM221A1	8.00E-13	0.100896866	0.041	0.018	1.93E-08	1.2	FAM221A	2.277777778
AGFG11	8.76E-13	0.272111333	0.384	0.316	2.11E-08	1.2	AGFG1	1.215189873
AIMP11	1.01E-12	0.22830058	0.368	0.296	2.43E-08	1.2	AIMP1	1.243243243
FGGY	1.03E-12	0.208420944	0.191	0.134	2.49E-08	1.2	FGGY	1.425373134
TCEB1	1.18E-12	0.313202693	0.366	0.302	2.84E-08	1.2	TCEB1	1.21192053
MAD1L1	1.37E-12	0.163793246	0.076	0.042	3.30E-08	1.2	MAD1L1	1.80952381
DMTF1	1.44E-12	0.219551764	0.154	0.104	3.46E-08	1.2	DMTF1	1.480769231
ARHGEF281	1.50E-12	0.107034005	0.342	0.268	3.62E-08	1.2	ARHGEF28	1.276119403
NCOA3	1.50E-12	0.256629572	0.313	0.248	3.62E-08	1.2	NCOA3	1.262096774
PRDM1	1.60E-12	0.138446533	0.06	0.03	3.85E-08	1.2	PRDM1	2
SNX24	1.72E-12	0.211361593	0.224	0.165	4.15E-08	1.2	SNX24	1.357575758
RELA	1.93E-12	0.153804402	0.124	0.078	4.67E-08	1.2	RELA	1.58974359
HERC11	2.10E-12	0.199905812	0.38	0.307	5.05E-08	1.2	HERC1	1.237785016
PLEKHA2	2.21E-12	0.115352926	0.05	0.023	5.34E-08	1.2	PLEKHA2	2.173913043
TMEM150C1	2.22E-12	0.25986134	0.261	0.202	5.35E-08	1.2	TMEM150(	1.292079208
PRICKLE11	2.40E-12	0.191253824	0.087	0.05	5.79E-08	1.2	PRICKLE1	1.74
MT-CO11	2.66E-12	0.19675899	0.991	0.992	6.42E-08	1.2	MT-CO1	0.998991935
VCL1	2.81E-12	0.188005893	0.38	0.305	6.77E-08	1.2	VCL	1.245901639
TNFAIP8	3.64E-12	0.412007095	0.438	0.381	8.79E-08	1.2	TNFAIP8	1.149606299
TEAD1	3.74E-12	0.170178113	0.524	0.447	9.01E-08	1.2	TEAD1	1.172259508
RP11-398K22.121	3.82E-12	0.149511717	0.067	0.036	9.22E-08	1.2	RP11-398K	1.861111111
PHACTR21	3.95E-12	0.176527651	0.105	0.064	9.53E-08	1.2	PHACTR2	1.640625
TMEM1231	4.03E-12	0.17843077	0.367	0.298	9.72E-08	1.2	TMEM123	1.231543624
TRNAU1AP	4.08E-12	0.217015633	0.289	0.226	9.85E-08	1.2	TRNAU1AP	1.278761062
EIF1B1	4.54E-12	0.215657861	0.282	0.218	1.09E-07	1.2	EIF1B	1.293577982
CASC81	4.96E-12	0.120056526	0.075	0.042	1.20E-07	1.2	CASC8	1.785714286
RIT1	5.49E-12	0.143436901	0.089	0.052	1.32E-07	1.2	RIT1	1.711538462
PSMD12	5.52E-12	0.200811198	0.145	0.097	1.33E-07	1.2	PSMD12	1.494845361
MPP51	5.52E-12	0.2192713	0.269	0.206	1.33E-07	1.2	MPP5	1.305825243
CAMSAP11	5.68E-12	0.153431637	0.102	0.062	1.37E-07	1.2	CAMSAP1	1.64516129
PLA2G16	5.76E-12	0.240888834	0.136	0.09	1.39E-07	1.2	PLA2G16	1.511111111
DFFA	5.91E-12	0.126743387	0.075	0.042	1.42E-07	1.2	DFFA	1.785714286
NCOA2	6.64E-12	0.349570417	0.487	0.43	1.60E-07	1.2	NCOA2	1.13255814
NARS	6.69E-12	0.282102584	0.31	0.252	1.61E-07	1.2	NARS	1.23015873
RTN41	6.78E-12	0.190215473	0.698	0.65	1.63E-07	1.2	RTN4	1.073846154
RGS6	6.91E-12	0.293811994	0.165	0.115	1.67E-07	1.2	RGS6	1.434782609
CTA-293F17.11	6.94E-12	0.163376129	0.11	0.068	1.67E-07	1.2	CTA-293F1	1.617647059
SUMO11	7.68E-12	0.242078436	0.543	0.493	1.85E-07	1.2	SUMO1	1.101419878
TPK11	7.74E-12	0.252305075	0.262	0.203	1.87E-07	1.2	TPK1	1.290640394

NOD21	8.49E-12	0.13837335	0.069	0.038	2.05E-07	1.2	NOD2	1.815789474
EIF3D1	9.24E-12	0.241737002	0.385	0.322	2.23E-07	1.2	EIF3D	1.195652174
SEC621	9.65E-12	0.211467732	0.624	0.574	2.33E-07	1.2	SEC62	1.087108014
HSPA8	9.75E-12	0.302354057	0.511	0.453	2.35E-07	1.2	HSPA8	1.12803532
Sep-11	1.05E-11	0.202101682	0.145	0.098	2.52E-07	1.2	Sep-11	1.479591837
NLRC5	1.33E-11	0.132218316	0.052	0.026	3.20E-07	1.2	NLRC5	2
RNF1141	1.35E-11	0.214327833	0.173	0.122	3.25E-07	1.2	RNF114	1.418032787
TGFB21	1.43E-11	0.302276922	0.244	0.184	3.44E-07	1.2	TGFB2	1.326086957
RIPK21	1.45E-11	0.219200258	0.344	0.274	3.49E-07	1.2	RIPK2	1.255474453
CDC5L1	1.51E-11	0.214809786	0.252	0.194	3.63E-07	1.2	CDC5L	1.298969072
MCF2L21	1.85E-11	0.21247454	0.142	0.096	4.47E-07	1.2	MCF2L2	1.479166667
NONO1	2.07E-11	0.227241437	0.371	0.309	4.98E-07	1.2	NONO	1.200647249
KCNK5	2.09E-11	0.104441598	0.036	0.015	5.03E-07	1.2	KCNK5	2.4
DDX181	2.28E-11	0.277695596	0.347	0.283	5.49E-07	1.2	DDX18	1.22614841
IGFBP5	2.33E-11	0.221645999	0.067	0.037	5.61E-07	1.2	IGFBP5	1.810810811
SMYD2	2.43E-11	0.159869524	0.069	0.038	5.86E-07	1.2	SMYD2	1.815789474
MUC16	2.47E-11	0.125271186	0.049	0.024	5.96E-07	1.2	MUC16	2.041666667
PCNP1	2.79E-11	0.222988462	0.395	0.333	6.74E-07	1.2	PCNP	1.186186186
ZRANB11	3.15E-11	0.208716912	0.203	0.148	7.59E-07	1.2	ZRANB1	1.371621622
MYADM	3.26E-11	0.125789328	0.077	0.044	7.87E-07	1.2	MYADM	1.75
SEC11A1	3.40E-11	0.216897254	0.532	0.479	8.20E-07	1.2	SEC11A	1.110647182
LINC01389	3.46E-11	0.10300799	0.04	0.018	8.35E-07	1.2	LINC01389	2.222222222
AGPS	3.64E-11	0.141987905	0.069	0.039	8.79E-07	1.2	AGPS	1.769230769
CCNC	3.69E-11	0.231835571	0.27	0.212	8.90E-07	1.2	CCNC	1.273584906
COPB11	3.71E-11	0.208267887	0.244	0.187	8.95E-07	1.2	COPB1	1.304812834
PAK31	4.13E-11	0.111181251	0.075	0.042	9.96E-07	1.2	PAK3	1.785714286
RUSC21	4.15E-11	0.183713746	0.105	0.066	1.00E-06	1.2	RUSC2	1.590909091
ATP6V1D	4.27E-11	0.306301597	0.326	0.271	1.03E-06	1.2	ATP6V1D	1.20295203
TGFBR31	4.36E-11	0.240749965	0.14	0.095	1.05E-06	1.2	TGFBR3	1.473684211
CCL2	5.05E-11	0.690160631	0.319	0.253	1.22E-06	1.2	CCL2	1.260869565
HSPG2	5.24E-11	0.177834477	0.12	0.078	1.26E-06	1.2	HSPG2	1.538461538
WTAP1	5.46E-11	0.177194771	0.57	0.509	1.32E-06	1.2	WTAP	1.119842829
KRT71	5.54E-11	0.252695742	0.321	0.258	1.34E-06	1.2	KRT7	1.244186047
EIF3I1	6.29E-11	0.221250507	0.64	0.591	1.52E-06	1.2	EIF3I	1.082910321
ITGBL11	6.36E-11	0.142431127	0.049	0.024	1.53E-06	1.2	ITGBL1	2.041666667
PHGDH	6.79E-11	0.113190421	0.047	0.023	1.64E-06	1.2	PHGDH	2.043478261
S100A10	7.59E-11	0.276752969	0.45	0.381	1.83E-06	1.2	S100A10	1.181102362
RCAN31	7.79E-11	0.469577269	0.238	0.185	1.88E-06	1.2	RCAN3	1.286486486
RP11-452H21.1	8.65E-11	0.166025798	0.105	0.066	2.09E-06	1.2	RP11-452H	1.590909091
HEBP2	9.09E-11	0.202403199	0.205	0.152	2.19E-06	1.2	HEBP2	1.348684211
TP53BP21	9.91E-11	0.18821534	0.168	0.119	2.39E-06	1.2	TP53BP2	1.411764706
RP11-840I19.31	1.00E-10	0.146224237	0.094	0.058	2.41E-06	1.2	RP11-840I1	1.620689655
MAP2K3	1.06E-10	0.165173338	0.094	0.058	2.55E-06	1.2	MAP2K3	1.620689655
RP11-417F21.1	1.12E-10	0.177101011	0.136	0.093	2.70E-06	1.2	RP11-417F:	1.462365591
PEA15	1.13E-10	0.226345937	0.246	0.192	2.72E-06	1.2	PEA15	1.28125
MAP2K1	1.13E-10	0.221548252	0.311	0.248	2.72E-06	1.2	MAP2K1	1.254032258
PRELID2	1.16E-10	0.179915849	0.107	0.068	2.80E-06	1.2	PRELID2	1.573529412
TBX191	1.17E-10	0.151848243	0.091	0.055	2.81E-06	1.2	TBX19	1.654545455
LINC008871	1.37E-10	0.134480723	0.061	0.033	3.30E-06	1.2	LINC00887	1.848484848
ZNF195	1.55E-10	0.149204366	0.111	0.072	3.74E-06	1.2	ZNF195	1.541666667
SH3YL11	1.60E-10	0.189442453	0.256	0.199	3.86E-06	1.2	SH3YL1	1.286432161
NSRP1	1.62E-10	0.229044112	0.26	0.206	3.90E-06	1.2	NSRP1	1.262135922

OCLN	1.63E-10	0.150576629	0.404	0.332	3.92E-06	1.2	OCLN	1.21686747
RP11-244M2.11	1.64E-10	0.168465085	0.473	0.404	3.96E-06	1.2	RP11-244M	1.170792079
NUFIP2	1.70E-10	0.207828337	0.236	0.181	4.10E-06	1.2	NUFIP2	1.303867403
ATP1B3	1.72E-10	0.215197599	0.31	0.251	4.15E-06	1.2	ATP1B3	1.235059761
IFFO21	1.84E-10	0.219822292	0.159	0.112	4.43E-06	1.2	IFFO2	1.419642857
BEND7	1.94E-10	0.141798637	0.113	0.073	4.68E-06	1.2	BEND7	1.547945205
PLAC9	2.24E-10	0.106257639	0.044	0.021	5.40E-06	1.2	PLAC9	2.095238095
GAB2	2.25E-10	0.229277283	0.25	0.195	5.43E-06	1.2	GAB2	1.282051282
DYRK31	2.37E-10	0.148698383	0.075	0.044	5.71E-06	1.2	DYRK3	1.704545455
PPHLN11	2.40E-10	0.20729436	0.392	0.334	5.78E-06	1.2	PPHLN1	1.173652695
FAR11	2.41E-10	0.160255732	0.133	0.089	5.81E-06	1.2	FAR1	1.494382022
PRMT1	2.63E-10	0.180294274	0.044	0.021	6.34E-06	1.2	PRMT1	2.095238095
STIL	2.65E-10	0.16894168	0.079	0.048	6.40E-06	1.2	STIL	1.645833333
KPNA2	2.68E-10	0.192678682	0.185	0.135	6.47E-06	1.2	KPNA2	1.37037037
SARS1	2.70E-10	0.214162705	0.226	0.172	6.51E-06	1.2	SARS	1.313953488
EXT2	2.82E-10	0.185894638	0.15	0.104	6.80E-06	1.2	EXT2	1.442307692
EIF4B1	2.96E-10	0.183921341	0.436	0.377	7.13E-06	1.2	EIF4B	1.156498674
TNFRSF1B	2.96E-10	0.124922003	0.062	0.034	7.14E-06	1.2	TNFRSF1B	1.823529412
TAF7	2.99E-10	0.202090327	0.156	0.11	7.21E-06	1.2	TAF7	1.418181818
C8orf591	3.00E-10	0.187730903	0.342	0.282	7.23E-06	1.2	C8orf59	1.212765957
SEC24B1	3.01E-10	0.230145371	0.298	0.239	7.25E-06	1.2	SEC24B	1.246861925
MGST3	3.14E-10	0.253573631	0.475	0.42	7.57E-06	1.2	MGST3	1.130952381
TEC	3.23E-10	0.165550724	0.075	0.044	7.78E-06	1.2	TEC	1.704545455
EIF5B	4.08E-10	0.25037506	0.256	0.204	9.84E-06	1.2	EIF5B	1.254901961
SLC7A11	4.40E-10	0.1258928	0.08	0.048	1.06E-05	1.2	SLC7A11	1.666666667
ESD1	4.55E-10	0.16394861	0.374	0.309	1.10E-05	1.2	ESD	1.210355987
MLXIP	4.77E-10	0.208427752	0.18	0.132	1.15E-05	1.2	MLXIP	1.363636364
SH3RF2	4.83E-10	0.107894684	0.047	0.024	1.16E-05	1.2	SH3RF2	1.958333333
CEBPZ	5.06E-10	0.221588249	0.196	0.148	1.22E-05	1.2	CEBPZ	1.324324324
CECR7	6.66E-10	0.120120136	0.072	0.042	1.61E-05	1.2	CECR7	1.714285714
TXN	6.96E-10	0.267744388	0.7	0.673	1.68E-05	1.2	TXN	1.040118871
NUTM2A-AS1	7.08E-10	0.16395648	0.36	0.298	1.71E-05	1.2	NUTM2A-A	1.208053691
CAPZA1	7.22E-10	0.209854226	0.471	0.415	1.74E-05	1.2	CAPZA1	1.134939759
SCAPER	7.67E-10	0.226376459	0.207	0.157	1.85E-05	1.2	SCAPER	1.318471338
CPSF6	7.75E-10	0.174873837	0.167	0.121	1.87E-05	1.2	CPSF6	1.380165289
NUS11	8.79E-10	0.152390629	0.117	0.078	2.12E-05	1.2	NUS1	1.5
MAP4	9.33E-10	0.192086012	0.533	0.477	2.25E-05	1.2	MAP4	1.117400419
RPF21	9.34E-10	0.166221971	0.179	0.131	2.25E-05	1.2	RPF2	1.366412214
BTBD10	9.49E-10	0.153396372	0.086	0.053	2.29E-05	1.2	BTBD10	1.622641509
MFN1	9.49E-10	0.180812209	0.168	0.121	2.29E-05	1.2	MFN1	1.388429752
RP11-530C5.1	9.55E-10	0.143624577	0.057	0.031	2.30E-05	1.2	RP11-530C	1.838709677
RABGGTB1	1.14E-09	0.154425646	0.111	0.073	2.74E-05	1.2	RABGGTB	1.520547945
RTP41	1.17E-09	0.142303237	0.07	0.041	2.83E-05	1.2	RTP4	1.707317073
CD741	1.38E-09	0.143629414	0.124	0.084	3.34E-05	1.2	CD74	1.476190476
MAP3K9	1.53E-09	0.187614165	0.212	0.16	3.69E-05	1.2	MAP3K9	1.325
IL12RB1	1.56E-09	0.106406372	0.039	0.019	3.76E-05	1.2	IL12RB1	2.052631579
EIF2S31	1.57E-09	0.18547815	0.345	0.288	3.80E-05	1.2	EIF2S3	1.197916667
BACH1	1.64E-09	0.237788506	0.36	0.304	3.95E-05	1.2	BACH1	1.184210526
ELK41	1.70E-09	0.120869237	0.098	0.063	4.09E-05	1.2	ELK4	1.555555556
NCL1	1.79E-09	0.284013404	0.215	0.166	4.31E-05	1.2	NCL	1.295180723
PLEKHM1	1.91E-09	0.113528084	0.069	0.041	4.62E-05	1.2	PLEKHM1	1.682926829
FBXW11	1.96E-09	0.222960614	0.343	0.288	4.72E-05	1.2	FBXW11	1.190972222

USP31	1.96E-09	0.174089232	0.157	0.112	4.74E-05	1.2	USP31	1.401785714
YEATS2	2.18E-09	0.174543459	0.158	0.113	5.26E-05	1.2	YEATS2	1.398230088
TNFRSF10D	2.48E-09	0.130681351	0.065	0.037	5.97E-05	1.2	TNFRSF10D	1.756756757
RP11-122M14.31	2.85E-09	0.17681306	0.022	0.008	6.86E-05	1.2	RP11-122M	2.75
UBD	2.94E-09	0.494763047	0.125	0.086	7.09E-05	1.2	UBD	1.453488372
ST14	3.00E-09	0.192738227	0.223	0.172	7.22E-05	1.2	ST14	1.296511628
MPRIP	3.11E-09	0.202176167	0.314	0.257	7.50E-05	1.2	MPRIP	1.221789883
ARFGEF31	3.49E-09	0.191376769	0.331	0.272	8.42E-05	1.2	ARFGEF3	1.216911765
RP11-440L14.1	4.08E-09	0.100655017	0.062	0.035	9.84E-05	1.2	RP11-440L1	1.771428571
EIF3J1	4.09E-09	0.173262547	0.165	0.12	9.86E-05	1.2	EIF3J	1.375
RNF181	4.21E-09	0.239494325	0.436	0.383	0.000101503	1.2	RNF181	1.138381201
GTF2F21	4.32E-09	0.161895485	0.142	0.101	0.000104115	1.2	GTF2F2	1.405940594
FTL1	4.35E-09	0.744864344	0.541	0.531	0.000104835	1.2	FTL	1.018832392
ZNF791	4.58E-09	0.158106546	0.134	0.094	0.000110321	1.2	ZNF791	1.425531915
SRSF121	4.77E-09	0.1246393	0.068	0.04	0.000115088	1.2	SRSF12	1.7
Sep-07	4.88E-09	0.182285258	0.425	0.37	0.000117625	1.2	Sep-07	1.148648649
GALNT18	5.03E-09	0.140088963	0.07	0.042	0.000121388	1.2	GALNT18	1.666666667
METAP11	5.69E-09	0.141249039	0.092	0.06	0.000137108	1.2	METAP1	1.533333333
SNRPD21	5.93E-09	0.155565959	0.706	0.67	0.000142902	1.2	SNRPD2	1.053731343
ACTG21	6.09E-09	0.137878637	0.023	0.009	0.000146916	1.2	ACTG2	2.555555556
ABTB2	6.16E-09	0.153888118	0.301	0.242	0.000148543	1.2	ABTB2	1.243801653
UBE3C	6.39E-09	0.200633915	0.289	0.237	0.000154189	1.2	UBE3C	1.219409283
PMM2	6.76E-09	0.168807145	0.105	0.07	0.000163092	1.2	PMM2	1.5
EPS15	6.78E-09	0.196896372	0.252	0.2	0.00016345	1.2	EPS15	1.26
MYH14	7.06E-09	0.168932713	0.205	0.157	0.000170233	1.2	MYH14	1.305732484
C8orf37-AS1	7.23E-09	0.225162387	0.293	0.242	0.000174426	1.2	C8orf37-AS	1.210743802
PPP2R5C	7.37E-09	0.172404025	0.216	0.168	0.000177601	1.2	PPP2R5C	1.285714286
UQCRHL1	7.49E-09	0.163573974	0.229	0.179	0.000180563	1.2	UQCRHL	1.279329609
IRF6	7.76E-09	0.250863928	0.281	0.229	0.000187044	1.2	IRF6	1.227074236
CREB1	8.37E-09	0.163561244	0.238	0.186	0.000201767	1.2	CREB1	1.279569892
NPC11	8.89E-09	0.199786093	0.372	0.313	0.000214326	1.2	NPC1	1.188498403
WDR45B1	8.89E-09	0.191216421	0.263	0.212	0.000214341	1.2	WDR45B	1.240566038
TXNDC9	9.20E-09	0.145667903	0.152	0.11	0.000221724	1.2	TXNDC9	1.381818182
WBP51	9.51E-09	0.170287463	0.385	0.328	0.000229242	1.2	WBP5	1.173780488
SESN2	9.77E-09	0.17949532	0.151	0.109	0.000235477	1.2	SESN2	1.385321101
ARNTL2	1.01E-08	0.177574188	0.189	0.142	0.000243057	1.2	ARNTL2	1.330985915
ENY2	1.03E-08	0.272668011	0.432	0.387	0.00024773	1.2	ENY2	1.11627907
U2SURP1	1.05E-08	0.222434438	0.283	0.234	0.000254239	1.2	U2SURP	1.209401709
OSMR	1.05E-08	0.222317178	0.354	0.302	0.000254361	1.2	OSMR	1.17218543
FAM46B1	1.06E-08	0.119225654	0.082	0.052	0.000254582	1.2	FAM46B	1.576923077
CTB-147C22.9	1.07E-08	0.10490522	0.05	0.027	0.000256828	1.2	CTB-147C2	1.851851852
SOX9	1.14E-08	0.190450842	0.237	0.188	0.000275433	1.2	SOX9	1.260638298
SLC16A1-AS1	1.23E-08	0.147315765	0.098	0.065	0.000296385	1.2	SLC16A1-A:	1.507692308
RPL101	1.25E-08	0.207622685	0.961	0.932	0.000301923	1.2	RPL10	1.03111588
MFSD14C	1.28E-08	0.194379137	0.214	0.166	0.000309676	1.2	MFSD14C	1.289156627
OSBPL91	1.31E-08	0.112622536	0.426	0.37	0.000316615	1.2	OSBPL9	1.151351351
DENND3	1.43E-08	0.117695962	0.049	0.027	0.000345237	1.2	DENND3	1.814814815
TRAPPC2	1.46E-08	0.11484813	0.088	0.057	0.000352395	1.2	TRAPPC2	1.543859649
LINC012351	1.57E-08	0.182881322	0.128	0.091	0.000379326	1.2	LINC01235	1.406593407
TATDN11	1.65E-08	0.155145398	0.26	0.208	0.000397508	1.2	TATDN1	1.25
PPP1R13L	1.70E-08	0.160404138	0.132	0.093	0.000409631	1.2	PPP1R13L	1.419354839
BIRC6-AS2	1.71E-08	0.226375146	0.25	0.202	0.000411839	1.2	BIRC6-AS2	1.237623762

MYO1D	1.87E-08	0.195581648	0.374	0.313	0.000451762	1.2	MYO1D	1.194888179
TLR2	1.88E-08	0.209721344	0.211	0.166	0.000453701	1.2	TLR2	1.271084337
MRPS18C	1.93E-08	0.191189599	0.19	0.146	0.000465587	1.2	MRPS18C	1.301369863
RPS121	1.95E-08	0.217122066	0.672	0.648	0.000470252	1.2	RPS12	1.037037037
FERMT21	2.00E-08	0.129609875	0.177	0.131	0.00048279	1.2	FERMT2	1.351145038
TIAM2	2.13E-08	0.265858004	0.272	0.226	0.000514297	1.2	TIAM2	1.203539823
GTF2IRD1	2.14E-08	0.188101325	0.21	0.163	0.000516711	1.2	GTF2IRD1	1.288343558
EML41	2.15E-08	0.206512374	0.208	0.162	0.000517618	1.2	EML4	1.283950617
ITGB5-AS11	2.19E-08	0.182880033	0.045	0.024	0.000529276	1.2	ITGB5-AS1	1.875
SH3BGRL3	2.20E-08	0.268282601	0.084	0.054	0.000530552	1.2	SH3BGRL3	1.555555556
SEC24D1	2.37E-08	0.126616657	0.242	0.191	0.000570473	1.2	SEC24D	1.267015707
ARHGEF10L1	2.48E-08	0.185085661	0.21	0.164	0.000597976	1.2	ARHGEF10I	1.280487805
CNN31	2.48E-08	0.121821434	0.57	0.511	0.000598454	1.2	CNN3	1.115459883
TNFAIP1	2.70E-08	0.126863653	0.105	0.071	0.000651202	1.2	TNFAIP1	1.478873239
MARCKS1	2.89E-08	0.20137601	0.4	0.346	0.000697021	1.2	MARCKS	1.156069364
TLE2	2.95E-08	0.106921652	0.062	0.037	0.000712497	1.2	TLE2	1.675675676
IQCG1	2.97E-08	0.262019555	0.215	0.171	0.000716877	1.2	IQCG	1.257309942
AC016831.7	3.08E-08	0.255136893	0.301	0.253	0.000742685	1.2	AC016831.[illegible]	1.18972332
EEA11	3.21E-08	0.188604566	0.165	0.123	0.000775121	1.2	EEA1	1.341463415
CBR4	3.35E-08	0.199481989	0.28	0.231	0.000806592	1.2	CBR4	1.212121212
NSFL1C1	3.37E-08	0.146720403	0.104	0.071	0.000812534	1.2	NSFL1C	1.464788732
FBL1	3.38E-08	0.160568248	0.174	0.131	0.000814932	1.2	FBL	1.328244275
DSP	3.39E-08	0.211748443	0.399	0.352	0.000816363	1.2	DSP	1.133522727
HPS51	3.42E-08	0.176521175	0.178	0.135	0.000823776	1.2	HPS5	1.318518519
ZNF267	3.57E-08	0.169968303	0.205	0.159	0.000860002	1.2	ZNF267	1.289308176
UBE2V2	3.72E-08	0.196690602	0.295	0.246	0.000896246	1.2	UBE2V2	1.199186992
LINC01549	3.78E-08	0.137580943	0.033	0.016	0.000912274	1.2	LINC01549	2.0625
TIMM17A	3.79E-08	0.192164393	0.265	0.217	0.000914004	1.2	TIMM17A	1.221198157
MCTP2	3.79E-08	0.181765986	0.126	0.09	0.00091447	1.2	MCTP2	1.4
CPNE4	4.28E-08	0.162179078	0.059	0.035	0.001031551	1.2	CPNE4	1.685714286
CISD11	5.10E-08	0.161729233	0.172	0.131	0.001230515	1.2	CISD1	1.312977099
C12orf601	5.25E-08	0.139517103	0.104	0.071	0.001265678	1.2	C12orf60	1.464788732
PTPRJ1	5.63E-08	0.187432244	0.357	0.303	0.001357003	1.2	PTPRJ	1.178217822
TMPRSS3	5.83E-08	0.110239881	0.071	0.044	0.001406123	1.2	TMPRSS3	1.613636364
DOCK5	5.93E-08	0.247406677	0.251	0.205	0.001429488	1.2	DOCK5	1.224390244
FBXO46	6.03E-08	0.109308711	0.071	0.044	0.001453454	1.2	FBXO46	1.613636364
DDX5	6.08E-08	0.15487672	0.544	0.495	0.001466976	1.2	DDX5	1.098989899
KLC1	6.29E-08	0.126175959	0.069	0.043	0.001516472	1.2	KLC1	1.604651163
SOX61	6.37E-08	0.19295682	0.116	0.082	0.00153636	1.2	SOX6	1.414634146
RPL191	6.39E-08	0.182083978	0.927	0.899	0.001541708	1.2	RPL19	1.031145717
WNT9A	8.62E-08	0.101874373	0.052	0.03	0.002077765	1.2	WNT9A	1.733333333
LRCH1	1.03E-07	0.223141123	0.349	0.3	0.002487243	1.2	LRCH1	1.163333333
RPL18	1.09E-07	0.346720686	0.123	0.089	0.002622099	1.2	RPL18	1.382022472
GREB1L1	1.10E-07	0.2131496	0.136	0.1	0.002641391	1.2	GREB1L	1.36
TOMM201	1.10E-07	0.169961049	0.236	0.19	0.002662768	1.2	TOMM20	1.242105263
PSME41	1.18E-07	0.127642875	0.68	0.637	0.002851448	1.2	PSME4	1.067503925
FNBP41	1.20E-07	0.16815356	0.446	0.399	0.002887872	1.2	FNBP4	1.117794486
IARS	1.22E-07	0.199518766	0.172	0.132	0.002936382	1.2	IARS	1.303030303
TNFRSF10A	1.23E-07	0.159493229	0.096	0.066	0.002974445	1.2	TNFRSF10A	1.454545455
PFKP	1.27E-07	0.132891974	0.051	0.029	0.003074186	1.2	PFKP	1.75862069
KIT1	1.29E-07	0.195822127	0.218	0.172	0.003108075	1.2	KIT	1.26744186
HMOX2	1.30E-07	0.106431403	0.062	0.038	0.003130646	1.2	HMOX2	1.631578947

PLSCR2	1.32E-07	0.152199654	0.086	0.057	0.003176816	1.2	PLSCR2	1.50877193
ARPC2	1.36E-07	0.184289845	0.223	0.179	0.003285903	1.2	ARPC2	1.245810056
RPS21	1.41E-07	0.243358301	0.322	0.271	0.003394892	1.2	RPS2	1.188191882
CCDC50	1.44E-07	0.147432796	0.215	0.168	0.003469301	1.2	CCDC50	1.279761905
KEAP1	1.50E-07	0.247359462	0.049	0.028	0.003621688	1.2	KEAP1	1.75
LITAF	1.58E-07	0.174181509	0.482	0.434	0.003820945	1.2	LITAF	1.110599078
TPD521	1.67E-07	0.189686049	0.166	0.127	0.004030471	1.2	TPD52	1.307086614
CYYR1	1.70E-07	0.116824683	0.059	0.035	0.004110031	1.2	CYYR1	1.685714286
TRIM56	2.00E-07	0.179003248	0.191	0.15	0.004822324	1.2	TRIM56	1.273333333
PPP2R3A	2.04E-07	0.205153447	0.373	0.323	0.004924658	1.2	PPP2R3A	1.154798762
CLDND1	2.04E-07	0.175368377	0.245	0.199	0.00492705	1.2	CLDND1	1.231155779
DKC1	2.06E-07	0.101176472	0.076	0.049	0.004964031	1.2	DKC1	1.551020408
NCK1	2.19E-07	0.156436399	0.14	0.104	0.005286175	1.2	NCK1	1.346153846
APOLD11	2.32E-07	0.100996005	0.072	0.046	0.00559138	1.2	APOLD1	1.565217391
BFAR1	2.41E-07	0.139972664	0.181	0.14	0.005807169	1.2	BFAR	1.292857143
ZC3H151	2.49E-07	0.14166805	0.281	0.231	0.005996437	1.2	ZC3H15	1.216450216
YBX1	2.80E-07	0.191334736	0.336	0.291	0.006763609	1.2	YBX1	1.154639175
FOXK21	2.86E-07	0.175769918	0.264	0.218	0.006898376	1.2	FOXK2	1.211009174
PTPN12	2.87E-07	0.177155941	0.473	0.429	0.006913788	1.2	PTPN12	1.102564103
ZCCHC11	3.19E-07	0.193181193	0.248	0.206	0.007687607	1.2	ZCCHC11	1.203883495
TNF1	3.29E-07	0.241000102	0.115	0.082	0.00793984	1.2	TNF	1.402439024
CSDE11	3.31E-07	0.187052528	0.579	0.546	0.007979736	1.2	CSDE1	1.06043956
NR3C2	3.49E-07	0.211894864	0.113	0.082	0.008424762	1.2	NR3C2	1.37804878
ZFR	3.55E-07	0.213412249	0.367	0.32	0.008559604	1.2	ZFR	1.146875
NEMF	3.55E-07	0.184496207	0.306	0.261	0.008565211	1.2	NEMF	1.172413793
GCC2-AS1	3.56E-07	0.172649669	0.049	0.029	0.008589432	1.2	GCC2-AS1	1.689655172
LRP61	3.66E-07	0.136831047	0.232	0.187	0.008830069	1.2	LRP6	1.240641711
RP11-1H15.2	3.69E-07	0.121854854	0.102	0.071	0.008886626	1.2	RP11-1H15	1.436619718
RNF241	3.85E-07	0.168250145	0.379	0.328	0.009276033	1.2	RNF24	1.155487805
PSMB4	3.92E-07	0.162496834	0.148	0.112	0.009450139	1.2	PSMB4	1.321428571
LAMTOR5	4.87E-07	0.152844111	0.623	0.584	0.011734821	1.2	LAMTOR5	1.066780822
ATP11A	4.95E-07	0.147212298	0.084	0.056	0.011939593	1.2	ATP11A	1.5
RP11-66B24.5	5.64E-07	0.145724919	0.075	0.05	0.013603197	1.2	RP11-66B2	1.5
EIF2S2	5.68E-07	0.248554064	0.298	0.257	0.013688777	1.2	EIF2S2	1.159533074
UBAP2	5.70E-07	0.161522812	0.193	0.153	0.013732653	1.2	UBAP2	1.261437908
INTS6-AS11	5.94E-07	0.12646873	0.157	0.119	0.014315536	1.2	INTS6-AS1	1.319327731
FAM126B1	6.23E-07	0.184227733	0.17	0.132	0.015025048	1.2	FAM126B	1.287878788
RPS28	7.62E-07	0.319452975	0.115	0.084	0.01836884	1.2	RPS28	1.369047619
PHAX	7.83E-07	0.121481727	0.122	0.089	0.018892444	1.2	PHAX	1.370786517
LINGO1	7.88E-07	0.103838041	0.909	0.887	0.019007555	1.2	LINGO1	1.024802706
ZNF609	8.10E-07	0.216065525	0.522	0.488	0.019527421	1.2	ZNF609	1.069672131
KCNQ1OT1	8.16E-07	0.140783386	0.117	0.085	0.019685878	1.2	KCNQ1OT1	1.376470588
TTC1	8.34E-07	0.179847942	0.314	0.269	0.020118412	1.2	TTC1	1.167286245
POLR1D1	8.76E-07	0.149190225	0.267	0.222	0.021119848	1.2	POLR1D	1.202702703
PTPN2	8.79E-07	0.214164182	0.288	0.243	0.021202377	1.2	PTPN2	1.185185185
CNTNAP3B	9.16E-07	0.121008112	0.088	0.06	0.022098442	1.2	CNTNAP3B	1.466666667
ALDOA	9.19E-07	0.344384956	0.136	0.103	0.022162025	1.2	ALDOA	1.32038835
CD82	9.69E-07	0.153149176	0.075	0.05	0.023354832	1.2	CD82	1.5
COX7B1	9.80E-07	0.161944394	0.656	0.632	0.023629103	1.2	COX7B	1.037974684
EIF3K1	9.82E-07	0.139134048	0.378	0.327	0.023683325	1.2	EIF3K	1.155963303
DCAF6	1.10E-06	0.178207452	0.469	0.426	0.026616096	1.2	DCAF6	1.100938967
TPD52L1	1.11E-06	0.147089389	0.178	0.139	0.026716086	1.2	TPD52L1	1.28057554

FUBP11	1.15E-06	0.130262879	0.236	0.191	0.02783443	1.2	FUBP1	1.235602094
SLC4A7	1.17E-06	0.18142787	0.325	0.277	0.028119833	1.2	SLC4A7	1.173285199
DPM1	1.29E-06	0.186532463	0.282	0.24	0.031142567	1.2	DPM1	1.175
SLC26A3	1.41E-06	0.247359545	0.791	0.79	0.03395167	1.2	SLC26A3	1.001265823
HLA-E	1.47E-06	0.195218927	0.121	0.089	0.035399936	1.2	HLA-E	1.359550562
PLIN21	1.52E-06	0.163747638	0.125	0.092	0.036602274	1.2	PLIN2	1.358695652
PSME2	1.58E-06	0.216125896	0.331	0.29	0.038013958	1.2	PSME2	1.14137931
C6orf481	1.60E-06	0.117138145	0.101	0.071	0.038472453	1.2	C6orf48	1.422535211
AFAP11	1.61E-06	0.178902018	0.115	0.084	0.038731407	1.2	AFAP1	1.369047619
PPL	1.61E-06	0.123951315	0.096	0.067	0.038938332	1.2	PPL	1.432835821
MED28	1.66E-06	0.118902396	0.11	0.079	0.039918137	1.2	MED28	1.392405063
C5orf341	1.71E-06	0.110308496	0.078	0.053	0.041324749	1.2	C5orf34	1.471698113
RAB9A1	1.75E-06	0.153303788	0.203	0.162	0.042218603	1.2	RAB9A	1.25308642
RP11-392O1.41	1.95E-06	0.119989355	0.04	0.023	0.047113907	1.2	RP11-392O	1.739130435
CLIC1	2.05E-06	0.192366065	0.619	0.592	0.049428788	1.2	CLIC1	1.045608108
SAA1	0	2.116159501	0.99	0.698	0	1.3	SAA1	1.418338109
PI31	0	2.107640196	0.467	0.107	0	1.3	PI3	4.364485981
OVOS2	0	1.796696257	0.656	0.22	0	1.3	OVOS2	2.981818182
SLPI1	0	1.594133321	0.891	0.507	0	1.3	SLPI	1.75739645
DEFB11	0	1.538337021	0.773	0.398	0	1.3	DEFB1	1.942211055
TSHZ22	0	1.481989552	0.649	0.199	0	1.3	TSHZ2	3.261306533
LUCAT11	0	1.470357011	0.621	0.15	0	1.3	LUCAT1	4.14
CYP24A11	0	1.464911699	0.422	0.077	0	1.3	CYP24A1	5.480519481
RARRES1	0	1.464495421	0.624	0.16	0	1.3	RARRES1	3.9
SLC12A21	0	1.35687569	0.731	0.369	0	1.3	SLC12A2	1.98102981
SOD21	0	1.320360622	0.996	0.75	0	1.3	SOD2	1.328
BIRC32	0	1.264794408	0.886	0.578	0	1.3	BIRC3	1.532871972
LTF	0	1.257893102	0.803	0.285	0	1.3	LTF	2.81754386
CXCL171	0	1.230384812	0.334	0.074	0	1.3	CXCL17	4.513513514
RCAN12	0	1.19735208	0.755	0.351	0	1.3	RCAN1	2.150997151
EFNA51	0	1.132118479	0.642	0.236	0	1.3	EFNA5	2.720338983
SFRP12	0	1.127227264	0.596	0.179	0	1.3	SFRP1	3.329608939
PLCB11	0	1.10530553	0.559	0.182	0	1.3	PLCB1	3.071428571
SAA2-SAA41	0	1.068817898	0.52	0.14	0	1.3	SAA2-SAA4	3.714285714
ST6GALNAC52	0	1.064491822	0.476	0.15	0	1.3	ST6GALNA(	3.173333333
VNN11	0	1.037221471	0.387	0.094	0	1.3	VNN1	4.117021277
LYN1	0	1.023782497	0.8	0.359	0	1.3	LYN	2.228412256
MAML22	0	1.0135753	0.821	0.366	0	1.3	MAML2	2.243169399
RP5-1198O20.4	0	1.00007178	0.332	0.051	0	1.3	RP5-1198O	6.509803922
AC005152.31	0	0.989551421	0.287	0.054	0	1.3	AC005152.:	5.314814815
ARRDC32	0	0.986616919	0.737	0.34	0	1.3	ARRDC3	2.167647059
EGFR1	0	0.982529134	0.461	0.144	0	1.3	EGFR	3.201388889
AKR1C3	0	0.979002288	0.365	0.068	0	1.3	AKR1C3	5.367647059
MGST11	0	0.976406883	0.958	0.848	0	1.3	MGST1	1.129716981
ANXA13	0	0.971153616	0.932	0.569	0	1.3	ANXA1	1.637961336
L3MBTL41	0	0.948510729	0.47	0.141	0	1.3	L3MBTL4	3.333333333
SLC25A372	0	0.922660271	0.771	0.336	0	1.3	SLC25A37	2.294642857
PROM11	0	0.916845435	0.549	0.187	0	1.3	PROM1	2.935828877
LINC011521	0	0.859060868	0.238	0.037	0	1.3	LINC01152	6.432432432
CCL21	5.55E-299	2.182253303	0.549	0.222	1.34E-294	1.3	CCL2	2.472972973
TNFAIP61	8.44E-287	1.159185762	0.494	0.168	2.04E-282	1.3	TNFAIP6	2.94047619
C31	4.97E-285	0.819314379	0.399	0.115	1.20E-280	1.3	C3	3.469565217

WFDC21	1.63E-284	1.692722017	0.642	0.285	3.92E-280	1.3	WFDC2	2.252631579
SORBS11	1.56E-283	0.9044757	0.478	0.163	3.76E-279	1.3	SORBS1	2.932515337
CCL282	2.68E-281	0.9467245	0.708	0.322	6.45E-277	1.3	CCL28	2.198757764
SLC34A21	5.76E-279	0.8466063	0.382	0.108	1.39E-274	1.3	SLC34A2	3.537037037
RPS72	2.95E-273	0.7216962	0.962	0.911	7.11E-269	1.3	RPS7	1.055982437
CHI3L2	6.88E-273	0.9282846	0.579	0.23	1.66E-268	1.3	CHI3L2	2.517391304
ST8SIA1	2.81E-271	0.7586075	0.297	0.069	6.78E-267	1.3	ST8SIA1	4.304347826
MTHFD2L1	1.68E-270	0.9640361	0.721	0.362	4.04E-266	1.3	MTHFD2L	1.991712707
RPS27A2	3.05E-270	0.6715851	0.979	0.97	7.35E-266	1.3	RPS27A	1.009278351
SLC28A31	1.81E-260	0.7744168	0.44	0.144	4.36E-256	1.3	SLC28A3	3.055555556
CYP7B1	4.33E-260	1.093486	0.512	0.195	1.05E-255	1.3	CYP7B1	2.625641026
KIT2	2.76E-259	0.8415138	0.436	0.143	6.64E-255	1.3	KIT	3.048951049
SEMA6A2	8.14E-256	0.9806467	0.503	0.187	1.96E-251	1.3	SEMA6A	2.689839572
GUCY1A31	9.19E-246	0.7233194	0.326	0.088	2.22E-241	1.3	GUCY1A3	3.704545455
B2M2	1.04E-245	0.7826485	0.979	0.971	2.51E-241	1.3	B2M	1.008238929
RP4-678D15.11	2.50E-244	0.7471375	0.264	0.06	6.03E-240	1.3	RP4-678D1	4.4
MGAM21	6.37E-242	0.9093916	0.419	0.139	1.54E-237	1.3	MGAM2	3.014388489
CRYAB2	2.64E-240	0.7908976	0.55	0.221	6.36E-236	1.3	CRYAB	2.488687783
FBLN5	9.53E-236	1.019721	0.407	0.136	2.30E-231	1.3	FBLN5	2.992647059
HSPA81	5.85E-235	1.037306	0.71	0.427	1.41E-230	1.3	HSPA8	1.662763466
MMP72	5.07E-233	0.9731212	0.38	0.122	1.22E-228	1.3	MMP7	3.114754098
FRMD4A1	1.54E-232	0.8075167	0.573	0.24	3.72E-228	1.3	FRMD4A	2.3875
GLIS32	1.67E-232	0.8124677	0.397	0.131	4.03E-228	1.3	GLIS3	3.030534351
PLXDC2	1.10E-229	0.7121401	0.333	0.097	2.65E-225	1.3	PLXDC2	3.432989691
EVA1C	2.69E-229	0.8040843	0.589	0.261	6.48E-225	1.3	EVA1C	2.256704981
ADAMTS92	6.84E-228	0.9384859	0.475	0.179	1.65E-223	1.3	ADAMTS9	2.653631285
PDE4B2	2.13E-224	0.5497585	0.885	0.471	5.13E-220	1.3	PDE4B	1.878980892
ALDH1A32	6.97E-223	0.9443188	0.56	0.241	1.68E-218	1.3	ALDH1A3	2.323651452
NFIB1	4.08E-221	0.8171117	0.932	0.717	9.83E-217	1.3	NFIB	1.29986053
PIGR2	6.70E-218	0.8174345	0.524	0.214	1.61E-213	1.3	PIGR	2.448598131
SLC26A2	2.88E-217	0.7293254	0.251	0.06	6.94E-213	1.3	SLC26A2	4.183333333
PAPSS12	7.69E-214	0.7814693	0.483	0.197	1.85E-209	1.3	PAPSS1	2.45177665
SVIL2	1.35E-212	0.744079	0.856	0.551	3.24E-208	1.3	SVIL	1.55353902
PIK3R1	3.06E-212	0.8737731	0.401	0.143	7.38E-208	1.3	PIK3R1	2.804195804
ITGB82	8.25E-209	0.7823841	0.786	0.461	1.99E-204	1.3	ITGB8	1.704989154
IFI161	3.72E-208	0.6411351	0.343	0.108	8.96E-204	1.3	IFI16	3.175925926
VNN3	3.65E-207	0.8452252	0.351	0.113	8.80E-203	1.3	VNN3	3.10619469
PLEKHS1	9.41E-207	0.6982924	0.333	0.104	2.27E-202	1.3	PLEKHS1	3.201923077
ADAMTS9-AS21	5.51E-206	0.7115196	0.385	0.133	1.33E-201	1.3	ADAMTS9-/	2.894736842
TPD52L11	2.03E-201	0.7201487	0.349	0.116	4.89E-197	1.3	TPD52L1	3.00862069
RPS232	6.34E-201	0.5530691	0.977	0.967	1.53E-196	1.3	RPS23	1.010341262
AKR1C1	2.73E-200	0.7873215	0.228	0.054	6.58E-196	1.3	AKR1C1	4.222222222
ARHGAP26	4.30E-198	0.7097238	0.907	0.636	1.04E-193	1.3	ARHGAP26	1.426100629
HDAC91	3.07E-197	0.7246146	0.318	0.099	7.41E-193	1.3	HDAC9	3.212121212
SLC6A142	1.99E-196	0.7226555	0.287	0.083	4.80E-192	1.3	SLC6A14	3.457831325
CTD-2015G9.2	3.07E-195	0.5419261	0.251	0.065	7.40E-191	1.3	CTD-2015G	3.861538462
GLRX2	4.72E-192	0.7272447	0.555	0.255	1.14E-187	1.3	GLRX	2.176470588
SKAP21	4.97E-190	0.699132	0.426	0.167	1.20E-185	1.3	SKAP2	2.550898204
RPL142	1.79E-189	0.5264059	0.975	0.959	4.31E-185	1.3	RPL14	1.016684046
MEIS21	3.61E-188	0.6861104	0.324	0.105	8.70E-184	1.3	MEIS2	3.085714286
DTNB1	4.61E-188	0.7466784	0.705	0.393	1.11E-183	1.3	DTNB	1.79389313
ACAT22	1.64E-187	0.8321563	0.426	0.173	3.95E-183	1.3	ACAT2	2.462427746

SASH12	3.31E-186	0.67592	0.371	0.133	7.98E-182	1.3	SASH1	2.789473684
SEMA6A-AS11	9.31E-185	0.7407268	0.324	0.106	2.24E-180	1.3	SEMA6A-A!	3.056603774
ANKRD36C1	4.54E-184	0.9221852	0.72	0.426	1.10E-179	1.3	ANKRD36C	1.690140845
PDZK1IP1	2.23E-182	0.7162802	0.254	0.07	5.38E-178	1.3	PDZK1IP1	3.628571429
EHBP11	1.18E-181	0.7659506	0.482	0.214	2.83E-177	1.3	EHBP1	2.252336449
NPAS21	1.52E-178	0.8241179	0.632	0.34	3.67E-174	1.3	NPAS2	1.858823529
RPL62	3.50E-178	0.538728	0.967	0.947	8.43E-174	1.3	RPL6	1.021119324
C5orf462	1.15E-177	0.6447766	0.298	0.095	2.77E-173	1.3	C5orf46	3.136842105
PHLDA1	1.80E-176	0.5528044	0.253	0.072	4.35E-172	1.3	PHLDA1	3.513888889
ZNF521	1.41E-175	0.5559512	0.254	0.072	3.40E-171	1.3	ZNF521	3.527777778
APOO2	4.00E-175	0.4630318	0.976	0.965	9.64E-171	1.3	APOO	1.011398964
RPL52	7.41E-174	0.5197665	0.968	0.938	1.79E-169	1.3	RPL5	1.031982942
FAM177B1	1.08E-173	0.9921688	0.757	0.46	2.62E-169	1.3	FAM177B	1.645652174
TRIM24	1.71E-173	0.5755775	0.384	0.145	4.13E-169	1.3	TRIM2	2.648275862
TACC11	1.40E-172	0.7206846	0.372	0.143	3.37E-168	1.3	TACC1	2.601398601
MT-CO22	3.83E-171	0.5852096	0.986	0.986	9.23E-167	1.3	MT-CO2	1
RPL72	2.49E-170	0.7294833	0.959	0.866	6.00E-166	1.3	RPL7	1.1073903
LINC012352	2.90E-168	0.5760779	0.253	0.074	6.98E-164	1.3	LINC01235	3.418918919
PRDX11	7.99E-168	0.8924123	0.838	0.665	1.93E-163	1.3	PRDX1	1.260150376
RPL112	1.79E-167	0.4972377	0.972	0.957	4.33E-163	1.3	RPL11	1.015673981
CD591	4.98E-165	0.5767924	0.966	0.871	1.20E-160	1.3	CD59	1.109070034
GALNT15	2.52E-164	0.7892509	0.209	0.054	6.08E-160	1.3	GALNT15	3.87037037
MT-CO32	2.53E-163	0.4706098	0.992	0.996	6.11E-159	1.3	MT-CO3	0.995983936
ADGRF1	7.44E-161	0.3988262	0.129	0.021	1.79E-156	1.3	ADGRF1	6.142857143
HS3ST41	1.05E-160	0.7715139	0.398	0.163	2.52E-156	1.3	HS3ST4	2.441717791
SORBS22	1.86E-160	0.7176834	0.635	0.348	4.48E-156	1.3	SORBS2	1.824712644
LINC01184	2.11E-160	0.639628	0.326	0.119	5.09E-156	1.3	LINC01184	2.739495798
MFGE81	7.51E-160	0.8122977	0.252	0.077	1.81E-155	1.3	MFGE8	3.272727273
FDPS	7.60E-159	0.7752646	0.681	0.426	1.83E-154	1.3	FDPS	1.598591549
PPIA2	3.27E-158	0.5647072	0.909	0.792	7.89E-154	1.3	PPIA	1.147727273
PTPN21	9.82E-156	0.6718423	0.473	0.218	2.37E-151	1.3	PTPN2	2.169724771
SGK11	2.05E-154	0.5858006	0.267	0.086	4.95E-150	1.3	SGK1	3.104651163
NPM12	7.89E-154	0.5384151	0.902	0.691	1.90E-149	1.3	NPM1	1.305354559
RPL322	9.10E-154	0.4572391	0.979	0.975	2.20E-149	1.3	RPL32	1.004102564
MAP3K132	2.35E-151	0.5772301	0.838	0.594	5.67E-147	1.3	MAP3K13	1.410774411
DMD2	4.89E-150	0.5921736	0.494	0.232	1.18E-145	1.3	DMD	2.129310345
SOX10	4.93E-149	0.4639521	0.184	0.046	1.19E-144	1.3	SOX10	4
RPL302	9.53E-149	0.4220357	0.973	0.951	2.30E-144	1.3	RPL30	1.023133544
LINC011982	3.10E-148	0.585491	0.342	0.13	7.47E-144	1.3	LINC01198	2.630769231
S100A8	1.15E-147	1.264406	0.282	0.099	2.78E-143	1.3	S100A8	2.848484848
BRINP11	2.20E-147	0.8196213	0.26	0.085	5.29E-143	1.3	BRINP1	3.058823529
RPL35A2	1.14E-144	0.4546318	0.975	0.952	2.74E-140	1.3	RPL35A	1.024159664
RPL43	3.90E-144	0.4883363	0.951	0.883	9.40E-140	1.3	RPL4	1.077010193
RPS62	2.87E-142	0.4501236	0.979	0.978	6.92E-138	1.3	RPS6	1.001022495
ASS1	4.10E-142	0.5433998	0.23	0.071	9.89E-138	1.3	ASS1	3.23943662
NCOA72	4.25E-142	0.5807004	0.702	0.408	1.02E-137	1.3	NCOA7	1.720588235
EEF1B22	3.29E-141	0.5915344	0.876	0.711	7.92E-137	1.3	EEF1B2	1.232067511
RAPGEF51	1.48E-140	0.6938615	0.573	0.31	3.57E-136	1.3	RAPGEF5	1.848387097
RPL212	1.78E-140	0.5045762	0.948	0.86	4.29E-136	1.3	RPL21	1.102325581
FZD7	3.37E-140	0.3508707	0.136	0.027	8.12E-136	1.3	FZD7	5.037037037
STOX21	4.20E-140	0.4881573	0.237	0.074	1.01E-135	1.3	STOX2	3.202702703
PFDN42	1.15E-139	0.6067932	0.568	0.307	2.78E-135	1.3	PFDN4	1.850162866

MAP1B2	1.14E-138	0.619031	0.491	0.241	2.74E-134	1.3	MAP1B	2.037344398
SPIRE12	2.30E-138	0.6312027	0.469	0.226	5.55E-134	1.3	SPIRE1	2.075221239
MAMDC22	1.92E-137	0.5263194	0.941	0.839	4.63E-133	1.3	MAMDC2	1.121573302
C10orf10	1.19E-136	0.4628759	0.256	0.085	2.88E-132	1.3	C10orf10	3.011764706
LINGO21	5.18E-135	0.7739808	0.122	0.023	1.25E-130	1.3	LINGO2	5.304347826
SERPINB7	8.01E-134	0.6414666	0.236	0.076	1.93E-129	1.3	SERPINB7	3.105263158
NFKBIZ2	4.53E-133	0.5401117	0.798	0.529	1.09E-128	1.3	NFKBIZ	1.508506616
RPS172	7.13E-132	0.4248989	0.953	0.94	1.72E-127	1.3	RPS17	1.013829787
ECHDC1	2.22E-130	0.5554088	0.401	0.182	5.35E-126	1.3	ECHDC1	2.203296703
CALD12	2.23E-130	0.4893589	0.541	0.268	5.37E-126	1.3	CALD1	2.018656716
CLIP21	1.95E-126	0.5860177	0.295	0.113	4.70E-122	1.3	CLIP2	2.610619469
PSTPIP2	3.10E-126	0.6191528	0.427	0.2	7.46E-122	1.3	PSTPIP2	2.135
CACHD1	6.35E-126	0.4098158	0.154	0.038	1.53E-121	1.3	CACHD1	4.052631579
SGPP21	5.37E-125	0.5055719	0.259	0.092	1.29E-120	1.3	SGPP2	2.815217391
PSMB72	7.43E-125	0.6773353	0.659	0.427	1.79E-120	1.3	PSMB7	1.543325527
RSL24D12	9.17E-125	0.5633997	0.762	0.534	2.21E-120	1.3	RSL24D1	1.426966292
UAP11	1.14E-124	0.5800247	0.311	0.125	2.76E-120	1.3	UAP1	2.488
SUB12	9.95E-124	0.5590028	0.809	0.617	2.40E-119	1.3	SUB1	1.311183144
SLC47A12	1.58E-123	0.4650979	0.933	0.891	3.82E-119	1.3	SLC47A1	1.047138047
AGAP1	1.37E-122	0.6416766	0.72	0.48	3.31E-118	1.3	AGAP1	1.5
DAPK22	7.30E-122	0.4865793	0.443	0.207	1.76E-117	1.3	DAPK2	2.140096618
RPSA2	1.75E-120	0.4920808	0.92	0.796	4.22E-116	1.3	RPSA	1.155778894
ENO12	9.60E-119	0.7541512	0.596	0.356	2.32E-114	1.3	ENO1	1.674157303
NACA3	1.44E-118	0.4053555	0.951	0.904	3.48E-114	1.3	NACA	1.05199115
GABRP2	3.42E-118	0.4980675	0.487	0.238	8.24E-114	1.3	GABRP	2.046218487
KYNU	4.22E-118	0.6300609	0.444	0.217	1.02E-113	1.3	KYNU	2.046082949
CA81	3.33E-117	0.5205548	0.44	0.21	8.03E-113	1.3	CA8	2.095238095
CLLU1OS	7.28E-117	0.3746511	0.141	0.034	1.76E-112	1.3	CLLU1OS	4.147058824
KRT6B1	5.75E-116	0.4449939	0.163	0.044	1.39E-111	1.3	KRT6B	3.704545455
DIAPH2	3.12E-115	0.5994179	0.38	0.178	7.53E-111	1.3	DIAPH2	2.134831461
CTSS1	5.93E-115	0.538846	0.294	0.119	1.43E-110	1.3	CTSS	2.470588235
PRICKLE12	9.49E-115	0.406069	0.155	0.041	2.29E-110	1.3	PRICKLE1	3.780487805
TBCA2	1.08E-114	0.5887413	0.737	0.519	2.60E-110	1.3	TBCA	1.420038536
RIN2	1.31E-113	0.5880177	0.392	0.188	3.16E-109	1.3	RIN2	2.085106383
SERPINB3	2.44E-113	0.7905193	0.129	0.03	5.88E-109	1.3	SERPINB3	4.3
DOCK7	5.67E-113	0.5259141	0.316	0.135	1.37E-108	1.3	DOCK7	2.340740741
AC159540.11	1.85E-112	0.5979169	0.241	0.088	4.47E-108	1.3	AC159540.	2.738636364
EPS82	5.90E-111	0.6217455	0.526	0.295	1.42E-106	1.3	EPS8	1.783050847
AKR1C2	5.12E-110	0.3801781	0.135	0.033	1.23E-105	1.3	AKR1C2	4.090909091
CNKSR31	8.85E-110	0.7147083	0.511	0.283	2.13E-105	1.3	CNKSR3	1.80565371
S100A111	1.33E-109	0.5922655	0.879	0.705	3.21E-105	1.3	S100A11	1.246808511
AKR1B1	2.02E-109	0.5557379	0.14	0.036	4.88E-105	1.3	AKR1B1	3.888888889
RPL7A2	9.47E-109	0.396614	0.966	0.957	2.28E-104	1.3	RPL7A	1.009404389
LRP1B	1.91E-108	1.289077	0.306	0.136	4.59E-104	1.3	LRP1B	2.25
CHPT1	2.30E-108	0.7390185	0.507	0.287	5.55E-104	1.3	CHPT1	1.766550523
RPS132	8.14E-108	0.5988411	0.854	0.684	1.96E-103	1.3	RPS13	1.248538012
SAA41	1.92E-107	0.4307569	0.283	0.113	4.63E-103	1.3	SAA4	2.504424779
RPS3A2	2.15E-106	0.4176699	0.941	0.894	5.19E-102	1.3	RPS3A	1.052572707
RPS202	2.44E-106	0.5526822	0.951	0.921	5.88E-102	1.3	RPS20	1.03257329
SERPINB4	3.15E-105	0.7539195	0.137	0.035	7.59E-101	1.3	SERPINB4	3.914285714
UQCRH2	4.38E-105	0.4404545	0.868	0.734	1.06E-100	1.3	UQCRH	1.182561308
SLC39A142	4.72E-105	0.5302942	0.373	0.177	1.14E-100	1.3	SLC39A14	2.107344633

ZC3H12C1	5.02E-105	0.4602319	0.256	0.099	1.21E-100	1.3	ZC3H12C	2.585858586
CNTNAP3B1	1.57E-104	0.3733508	0.167	0.05	3.79E-100	1.3	CNTNAP3B	3.34
LDHB2	2.75E-104	0.4783196	0.762	0.539	6.62E-100	1.3	LDHB	1.413729128
NALCN	1.84E-103	0.4176044	0.193	0.064	4.43E-99	1.3	NALCN	3.015625
EIF3E2	2.81E-103	0.4225025	0.784	0.549	6.76E-99	1.3	EIF3E	1.428051002
HSP90AA12	3.14E-103	0.4741609	0.933	0.845	7.57E-99	1.3	HSP90AA1	1.104142012
RP11-536O18.11	4.55E-103	0.3330211	0.154	0.044	1.10E-98	1.3	RP11-536O	3.5
RPS252	4.87E-103	0.5078678	0.876	0.722	1.17E-98	1.3	RPS25	1.213296399
PLEKHA62	3.11E-102	0.4289381	0.916	0.822	7.50E-98	1.3	PLEKHA6	1.114355231
BTF32	3.77E-102	0.3881594	0.948	0.881	9.08E-98	1.3	BTF3	1.076049943
CISD12	4.69E-102	0.4198516	0.281	0.116	1.13E-97	1.3	CISD1	2.422413793
RPS272	5.34E-102	0.5891739	0.801	0.616	1.29E-97	1.3	RPS27	1.300324675
PPP2R3A1	2.29E-101	0.5726517	0.525	0.303	5.51E-97	1.3	PPP2R3A	1.732673267
RPL23A2	4.50E-101	0.6241997	0.75	0.554	1.09E-96	1.3	RPL23A	1.353790614
EIF4A12	6.82E-101	0.5025268	0.611	0.378	1.65E-96	1.3	EIF4A1	1.616402116
EIF4E21	4.35E-100	0.5177356	0.518	0.299	1.05E-95	1.3	EIF4E2	1.732441472
ZMYM42	3.60E-99	0.4322937	0.914	0.804	8.68E-95	1.3	ZMYM4	1.13681592
CELF22	6.58E-98	0.4619804	0.264	0.107	1.59E-93	1.3	CELF2	2.46728972
ARHGAP44	9.84E-98	0.545146	0.338	0.159	2.37E-93	1.3	ARHGAP44	2.125786164
RPL232	3.77E-97	0.3534783	0.957	0.911	9.08E-93	1.3	RPL23	1.050493963
PGK11	4.83E-97	0.5808931	0.734	0.533	1.17E-92	1.3	PGK1	1.377110694
S100A132	5.80E-97	0.5607457	0.694	0.492	1.40E-92	1.3	S100A13	1.410569106
ELF51	2.29E-96	0.4050427	0.293	0.125	5.53E-92	1.3	ELF5	2.344
S100A9	1.44E-95	0.7647583	0.107	0.024	3.46E-91	1.3	S100A9	4.458333333
PLSCR11	1.51E-95	0.452232	0.358	0.173	3.64E-91	1.3	PLSCR1	2.069364162
TXNIP	1.66E-95	0.5367358	0.593	0.357	4.01E-91	1.3	TXNIP	1.661064426
MAP4K41	1.37E-94	0.5149849	0.414	0.214	3.31E-90	1.3	MAP4K4	1.934579439
BDNF-AS2	2.76E-94	0.5471736	0.7	0.482	6.65E-90	1.3	BDNF-AS	1.452282158
ANKRD361	3.24E-94	0.4969311	0.204	0.074	7.82E-90	1.3	ANKRD36	2.756756757
RP11-608O21.12	5.33E-94	0.476337	0.736	0.513	1.28E-89	1.3	RP11-608O	1.434697856
PODXL	5.90E-94	0.4006486	0.224	0.085	1.42E-89	1.3	PODXL	2.635294118
CCL201	1.06E-93	0.9582265	0.339	0.164	2.55E-89	1.3	CCL20	2.067073171
EYA21	2.45E-93	0.4271031	0.233	0.091	5.90E-89	1.3	EYA2	2.56043956
RPS15A2	7.29E-93	0.3577607	0.958	0.94	1.76E-88	1.3	RPS15A	1.019148936
IGF2BP22	1.09E-92	0.3095329	0.473	0.247	2.64E-88	1.3	IGF2BP2	1.914979757
CHODL	1.96E-92	0.4791147	0.254	0.104	4.73E-88	1.3	CHODL	2.442307692
WFDC31	4.65E-92	0.3156056	0.165	0.053	1.12E-87	1.3	WFDC3	3.113207547
BAIAP2L12	5.13E-92	0.509772	0.958	0.922	1.24E-87	1.3	BAIAP2L1	1.039045553
TANK2	6.46E-92	0.4604885	0.684	0.448	1.56E-87	1.3	TANK	1.526785714
OOEP2	9.41E-92	0.3474153	0.972	0.965	2.27E-87	1.3	OOEP	1.007253886
PRDM11	1.02E-91	0.2855337	0.104	0.024	2.47E-87	1.3	PRDM1	4.333333333
HSP90AB12	2.59E-91	0.3902747	0.928	0.828	6.24E-87	1.3	HSP90AB1	1.120772947
ARL4C2	3.40E-91	0.4203096	0.231	0.091	8.20E-87	1.3	ARL4C	2.538461538
RPS32	1.23E-90	0.658106	0.928	0.861	2.96E-86	1.3	RPS3	1.077816492
RNASE1	2.27E-90	0.3005801	0.078	0.014	5.47E-86	1.3	RNASE1	5.571428571
ANXA31	3.74E-90	0.5723835	0.541	0.339	9.01E-86	1.3	ANXA3	1.595870206
RPS122	3.77E-90	0.7177599	0.758	0.636	9.10E-86	1.3	RPS12	1.191823899
FAM134B1	5.77E-90	0.4651772	0.212	0.081	1.39E-85	1.3	FAM134B	2.617283951
AC005863.1	1.68E-89	0.1275954	0.047	0.005	4.05E-85	1.3	AC005863.	9.4
CYYR11	2.32E-89	0.2794693	0.113	0.028	5.60E-85	1.3	CYYR1	4.035714286
TBC1D3P1-DHX40P12	6.07E-89	0.4801966	0.745	0.569	1.46E-84	1.3	TBC1D3P1-	1.309314587
PTPN142	6.93E-89	0.4672335	0.494	0.278	1.67E-84	1.3	PTPN14	1.776978417

HPS52	1.65E-88	0.4075095	0.277	0.121	3.97E-84	1.3	HPS5	2.289256198
NEURL31	3.82E-88	0.2939441	0.099	0.022	9.22E-84	1.3	NEURL3	4.5
ST3GAL61	8.25E-88	0.3855604	0.16	0.052	1.99E-83	1.3	ST3GAL6	3.076923077
FMN1	1.82E-87	0.4131073	0.228	0.092	4.39E-83	1.3	FMN1	2.47826087
RPL22L12	3.97E-87	0.547746	0.605	0.399	9.58E-83	1.3	RPL22L1	1.516290727
IFNAR21	5.88E-87	0.4941051	0.354	0.177	1.42E-82	1.3	IFNAR2	2
PSMC11	6.20E-87	0.5407429	0.45	0.258	1.49E-82	1.3	PSMC1	1.744186047
CDK62	7.74E-87	0.4806683	0.263	0.114	1.87E-82	1.3	CDK6	2.307017544
RPLP02	9.34E-87	0.5869063	0.915	0.86	2.25E-82	1.3	RPLP0	1.063953488
SDCBP1	9.71E-87	0.5807704	0.779	0.598	2.34E-82	1.3	SDCBP	1.302675585
CAMK2D2	1.32E-86	0.4311142	0.327	0.156	3.18E-82	1.3	CAMK2D	2.096153846
TRABD2B	1.74E-86	0.4667744	0.144	0.044	4.19E-82	1.3	TRABD2B	3.272727273
UGP21	2.30E-86	0.4872419	0.627	0.412	5.55E-82	1.3	UGP2	1.52184466
RPL342	5.86E-86	0.3241631	0.977	0.979	1.41E-81	1.3	RPL34	0.997957099
RPL412	2.18E-85	0.4750773	0.952	0.896	5.25E-81	1.3	RPL41	1.0625
RPS242	2.25E-85	0.281445	0.975	0.965	5.42E-81	1.3	RPS24	1.010362694
NUDT5	3.10E-85	0.3916436	0.273	0.121	7.48E-81	1.3	NUDT5	2.256198347
CEPT11	5.38E-85	0.4501741	0.329	0.161	1.30E-80	1.3	CEPT1	2.043478261
GABRE1	8.63E-85	0.2377451	0.097	0.022	2.08E-80	1.3	GABRE	4.409090909
ST6GAL11	1.05E-84	0.4967493	0.422	0.23	2.53E-80	1.3	ST6GAL1	1.834782609
EEF1A12	3.09E-84	0.3124305	0.978	0.974	7.46E-80	1.3	EEF1A1	1.004106776
PDE8A2	4.36E-84	0.51392	0.509	0.306	1.05E-79	1.3	PDE8A	1.663398693
CTSV2	1.00E-83	0.378011	0.241	0.099	2.41E-79	1.3	CTSV	2.434343434
CLDN13	1.24E-83	0.4070355	0.397	0.207	2.99E-79	1.3	CLDN1	1.917874396
ZPLD11	1.53E-83	0.4299038	0.164	0.056	3.68E-79	1.3	ZPLD1	2.928571429
BACE22	4.91E-83	0.5140475	0.494	0.29	1.19E-78	1.3	BACE2	1.703448276
NUB11	6.56E-83	0.7642073	0.336	0.175	1.58E-78	1.3	NUB1	1.92
SEC61G2	9.32E-83	0.5522213	0.748	0.581	2.25E-78	1.3	SEC61G	1.287435456
DENND2D	9.48E-83	0.3427789	0.19	0.071	2.29E-78	1.3	DENND2D	2.676056338
RP11-114H23.1	1.94E-82	0.4757434	0.224	0.091	4.68E-78	1.3	RP11-114H	2.461538462
PSMA32	2.56E-82	0.5368631	0.517	0.317	6.16E-78	1.3	PSMA3	1.630914826
MT-ND42	3.69E-82	0.3326746	0.987	0.994	8.89E-78	1.3	MT-ND4	0.992957746
EIF3L2	5.22E-82	0.4134727	0.763	0.567	1.26E-77	1.3	EIF3L	1.345679012
SPDYE21	6.66E-82	0.3799343	0.263	0.117	1.61E-77	1.3	SPDYE2	2.247863248
FAM20C	8.08E-82	0.4316923	0.142	0.045	1.95E-77	1.3	FAM20C	3.155555556
NT5C21	9.02E-82	0.4488501	0.443	0.248	2.17E-77	1.3	NT5C2	1.786290323
RP11-244M2.12	2.35E-81	0.4940566	0.59	0.388	5.68E-77	1.3	RP11-244M	1.520618557
UBA522	5.42E-81	0.3290015	0.954	0.914	1.31E-76	1.3	UBA52	1.043763676
CMPK11	1.45E-80	0.4718118	0.362	0.189	3.51E-76	1.3	CMPK1	1.915343915
ZRANB21	1.59E-80	0.4317896	0.394	0.212	3.84E-76	1.3	ZRANB2	1.858490566
ZCCHC171	1.74E-80	0.4803708	0.424	0.239	4.19E-76	1.3	ZCCHC17	1.774058577
IL342	1.78E-80	0.3896031	0.185	0.069	4.29E-76	1.3	IL34	2.68115942
ATRNL1	2.29E-80	0.4153575	0.132	0.04	5.51E-76	1.3	ATRNL1	3.3
GPR871	2.52E-80	0.2504932	0.091	0.021	6.07E-76	1.3	GPR87	4.333333333
TLR21	5.44E-80	0.4109611	0.314	0.152	1.31E-75	1.3	TLR2	2.065789474
TLN21	9.84E-80	0.4133269	0.157	0.054	2.37E-75	1.3	TLN2	2.907407407
SLC18B12	1.24E-79	0.3972109	0.221	0.091	2.98E-75	1.3	SLC18B1	2.428571429
ACP12	1.54E-79	0.4431808	0.485	0.288	3.71E-75	1.3	ACP1	1.684027778
MUC15	3.35E-79	0.2227634	0.077	0.015	8.08E-75	1.3	MUC15	5.133333333
TGFBR32	4.99E-79	0.5665627	0.211	0.086	1.20E-74	1.3	TGFBR3	2.453488372
CYB5R21	5.69E-79	0.2285204	0.103	0.026	1.37E-74	1.3	CYB5R2	3.961538462
RP11-142C4.6	1.09E-78	0.3492097	0.157	0.054	2.63E-74	1.3	RP11-142C	2.907407407

SPATA51	1.16E-78	0.3772047	0.191	0.073	2.80E-74	1.3	SPATA5	2.616438356
OSMR1	1.39E-78	0.473109	0.482	0.285	3.36E-74	1.3	OSMR	1.69122807
EGF	3.41E-78	0.2641643	0.113	0.031	8.23E-74	1.3	EGF	3.64516129
BTBD11	4.19E-78	0.3476523	0.116	0.033	1.01E-73	1.3	BTBD11	3.515151515
SFT2D22	1.25E-77	0.367189	0.26	0.117	3.01E-73	1.3	SFT2D2	2.222222222
PLA2R1	2.57E-77	0.4583044	0.337	0.171	6.19E-73	1.3	PLA2R1	1.970760234
IFITM3	3.20E-77	0.4936551	0.328	0.167	7.73E-73	1.3	IFITM3	1.964071856
FDFT1	6.10E-77	0.5072293	0.482	0.294	1.47E-72	1.3	FDFT1	1.639455782
BRK12	1.01E-76	0.4410007	0.761	0.588	2.43E-72	1.3	BRK1	1.294217687
CNIH41	1.68E-76	0.4299223	0.472	0.281	4.05E-72	1.3	CNIH4	1.679715302
CDK141	2.06E-76	0.5017504	0.498	0.306	4.97E-72	1.3	CDK14	1.62745098
PNISR1	2.13E-76	0.4138289	0.837	0.66	5.14E-72	1.3	PNISR	1.268181818
NACA22	2.58E-76	0.4496067	0.644	0.446	6.21E-72	1.3	NACA2	1.443946188
THADA	4.73E-76	0.6790847	0.345	0.184	1.14E-71	1.3	THADA	1.875
ITPR2	6.58E-76	0.5354378	0.642	0.454	1.59E-71	1.3	ITPR2	1.414096916
CCT52	7.48E-76	0.4941368	0.462	0.276	1.80E-71	1.3	CCT5	1.673913043
HSD11B11	9.78E-76	0.4325731	0.205	0.083	2.36E-71	1.3	HSD11B1	2.469879518
TNFRSF11B1	1.50E-75	0.4652477	0.165	0.06	3.62E-71	1.3	TNFRSF11E	2.75
PSMB11	1.76E-75	0.4873928	0.565	0.37	4.24E-71	1.3	PSMB1	1.527027027
CHI3L1	1.98E-75	0.5700956	0.213	0.089	4.77E-71	1.3	CHI3L1	2.393258427
SSBP11	5.41E-75	0.5052297	0.532	0.34	1.31E-70	1.3	SSBP1	1.564705882
PPM1H	2.64E-74	0.516251	0.481	0.293	6.38E-70	1.3	PPM1H	1.641638225
COA1	4.35E-74	0.4057612	0.373	0.199	1.05E-69	1.3	COA1	1.874371859
MGST31	5.33E-73	0.454422	0.598	0.404	1.28E-68	1.3	MGST3	1.48019802
RPL312	2.10E-71	0.3017046	0.959	0.92	5.07E-67	1.3	RPL31	1.042391304
ANKRD36B1	3.06E-71	0.4063944	0.169	0.064	7.38E-67	1.3	ANKRD36B	2.640625
C10orf90	4.99E-71	0.3022835	0.137	0.046	1.20E-66	1.3	C10orf90	2.97826087
ETS12	1.36E-70	0.3653843	0.156	0.056	3.27E-66	1.3	ETS1	2.785714286
IGFBP51	3.19E-70	0.3820541	0.107	0.031	7.70E-66	1.3	IGFBP5	3.451612903
PRELID1	3.78E-70	0.3272737	0.184	0.074	9.13E-66	1.3	PRELID1	2.486486486
MT-ATP6	4.14E-70	0.3523046	0.988	0.991	9.98E-66	1.3	MT-ATP6	0.996972755
SLC11A21	1.18E-69	0.4390471	0.518	0.327	2.85E-65	1.3	SLC11A2	1.584097859
LCN2	1.40E-69	0.3694489	0.062	0.012	3.38E-65	1.3	LCN2	5.166666667
KLK101	1.55E-69	0.2436588	0.086	0.021	3.73E-65	1.3	KLK10	4.095238095
RGS101	2.13E-69	0.3085383	0.15	0.054	5.14E-65	1.3	RGS10	2.777777778
JPX	3.25E-69	0.3759009	0.331	0.173	7.84E-65	1.3	JPX	1.913294798
ATP6V1B21	3.29E-69	0.3188064	0.186	0.074	7.94E-65	1.3	ATP6V1B2	2.513513514
CLEC7A	4.33E-69	0.304898	0.162	0.06	1.04E-64	1.3	CLEC7A	2.7
RPL192	6.46E-69	0.480548	0.947	0.896	1.56E-64	1.3	RPL19	1.056919643
MCF2L22	6.53E-69	0.3861251	0.205	0.087	1.57E-64	1.3	MCF2L2	2.356321839
TFCP2L12	6.54E-69	0.5071843	0.191	0.079	1.58E-64	1.3	TFCP2L1	2.417721519
LINC00511	1.04E-68	0.3985646	0.177	0.07	2.52E-64	1.3	LINC00511	2.528571429
THUMPD3-AS11	2.54E-68	0.3778204	0.373	0.207	6.12E-64	1.3	THUMPD3-	1.801932367
LTBP11	2.68E-68	0.5454237	0.336	0.185	6.45E-64	1.3	LTBP1	1.816216216
CCT3	8.79E-68	0.4095808	0.529	0.34	2.12E-63	1.3	CCT3	1.555882353
DMKN	1.28E-67	0.3111884	0.175	0.069	3.09E-63	1.3	DMKN	2.536231884
FGFBP11	1.78E-67	0.4488859	0.1	0.028	4.29E-63	1.3	FGFBP1	3.571428571
GLIPR21	4.47E-67	0.2638557	0.145	0.052	1.08E-62	1.3	GLIPR2	2.788461538
PDE1C1	4.78E-67	0.3086834	0.111	0.034	1.15E-62	1.3	PDE1C	3.264705882
PTCHD12	4.85E-67	0.2460048	0.11	0.033	1.17E-62	1.3	PTCHD1	3.333333333
S100A12	1.36E-66	0.2372253	0.107	0.032	3.28E-62	1.3	S100A1	3.34375
DAPP12	5.15E-66	0.3014926	0.399	0.223	1.24E-61	1.3	DAPP1	1.789237668

ZNF385D	8.41E-66	0.3236621	0.116	0.037	2.03E-61	1.3	ZNF385D	3.135135135
CTDSPL	1.07E-65	0.2408015	0.107	0.032	2.57E-61	1.3	CTDSPL	3.34375
ARG2	4.26E-65	0.2679999	0.232	0.106	1.03E-60	1.3	ARG2	2.188679245
SLC39A8	6.86E-65	0.360418	0.193	0.082	1.65E-60	1.3	SLC39A8	2.353658537
GNE	7.65E-65	0.3821334	0.244	0.116	1.85E-60	1.3	GNE	2.103448276
LRRC491	1.13E-64	0.3509398	0.171	0.069	2.71E-60	1.3	LRRC49	2.47826087
PLEKHG12	4.30E-64	0.3304164	0.207	0.091	1.04E-59	1.3	PLEKHG1	2.274725275
CCDC822	8.00E-64	0.3606706	0.28	0.141	1.93E-59	1.3	CCDC82	1.985815603
MMADHC1	1.74E-63	0.3810654	0.466	0.289	4.19E-59	1.3	MMADHC	1.612456747
CNTNAP3	1.76E-63	0.2391427	0.09	0.025	4.25E-59	1.3	CNTNAP3	3.6
FADS1	2.48E-63	0.1812107	0.064	0.013	5.98E-59	1.3	FADS1	4.923076923
CFLAR1	2.59E-63	0.4143853	0.505	0.321	6.24E-59	1.3	CFLAR	1.573208723
RP11-449D8.5	3.77E-63	0.3002208	0.111	0.035	9.10E-59	1.3	RP11-449D	3.171428571
BARX22	4.57E-63	0.4300807	0.4	0.232	1.10E-58	1.3	BARX2	1.724137931
GHITM	5.04E-63	0.4641685	0.595	0.426	1.21E-58	1.3	GHITM	1.396713615
PM20D21	5.51E-63	0.2841182	0.185	0.078	1.33E-58	1.3	PM20D2	2.371794872
CHST91	6.32E-63	0.2170681	0.072	0.017	1.52E-58	1.3	CHST9	4.235294118
SRSF32	1.05E-62	0.3928317	0.605	0.411	2.52E-58	1.3	SRSF3	1.472019465
ENOSF1	1.44E-62	0.3828121	0.297	0.156	3.46E-58	1.3	ENOSF1	1.903846154
NCALD1	1.59E-62	0.3886528	0.241	0.115	3.83E-58	1.3	NCALD	2.095652174
ACSL42	2.69E-62	0.2679837	0.128	0.044	6.48E-58	1.3	ACSL4	2.909090909
PAM1	3.10E-62	0.387128	0.642	0.446	7.47E-58	1.3	PAM	1.439461883
MCTP21	3.93E-62	0.3378363	0.19	0.081	9.47E-58	1.3	MCTP2	2.345679012
CCT22	5.54E-62	0.3862301	0.461	0.284	1.34E-57	1.3	CCT2	1.623239437
CTSC	6.20E-62	0.2136769	0.096	0.028	1.50E-57	1.3	CTSC	3.428571429
TEX142	7.15E-62	0.4413797	0.268	0.136	1.72E-57	1.3	TEX14	1.970588235
RNF242	8.52E-62	0.4071516	0.496	0.312	2.06E-57	1.3	RNF24	1.58974359
NCMAP2	9.28E-62	0.2755573	0.112	0.036	2.24E-57	1.3	NCMAP	3.111111111
COX7A2L2	9.62E-62	0.366757	0.669	0.478	2.32E-57	1.3	COX7A2L	1.39958159
USP13	9.99E-62	0.2412353	0.118	0.039	2.41E-57	1.3	USP13	3.025641026
SNRPB22	1.16E-61	0.3759129	0.51	0.324	2.80E-57	1.3	SNRPB2	1.574074074
C15orf52	1.22E-61	0.1918975	0.079	0.02	2.94E-57	1.3	C15orf52	3.95
CCND3	1.61E-61	0.4817453	0.43	0.265	3.89E-57	1.3	CCND3	1.622641509
RPS4X2	2.39E-61	0.2494489	0.977	0.979	5.76E-57	1.3	RPS4X	0.997957099
PADI2	3.01E-61	0.3557327	0.181	0.076	7.26E-57	1.3	PADI2	2.381578947
SYNJ21	3.38E-61	0.4176725	0.366	0.21	8.14E-57	1.3	SYNJ2	1.742857143
TTLL41	3.68E-61	0.3441539	0.22	0.102	8.87E-57	1.3	TTLL4	2.156862745
TCEAL82	6.04E-61	0.3306404	0.38	0.216	1.46E-56	1.3	TCEAL8	1.759259259
SYNM	1.85E-60	0.385783	0.174	0.073	4.46E-56	1.3	SYNM	2.383561644
ADARB1	2.25E-60	0.2305711	0.095	0.028	5.43E-56	1.3	ADARB1	3.392857143
WTAP2	3.06E-60	0.3598575	0.676	0.494	7.37E-56	1.3	WTAP	1.368421053
HAX11	3.23E-60	0.3794618	0.401	0.237	7.80E-56	1.3	HAX1	1.691983122
SLC16A1-AS11	3.26E-60	0.3249786	0.149	0.058	7.86E-56	1.3	SLC16A1-A:	2.568965517
GPRIN3	5.31E-60	0.1751731	0.058	0.011	1.28E-55	1.3	GPRIN3	5.272727273
OAT1	9.85E-60	0.3954922	0.425	0.259	2.37E-55	1.3	OAT	1.640926641
TTC11	9.87E-60	0.3876492	0.42	0.255	2.38E-55	1.3	TTC1	1.647058824
FANCL	1.06E-59	0.2528853	0.117	0.04	2.56E-55	1.3	FANCL	2.925
MT-CO12	1.19E-59	0.4410232	0.986	0.992	2.87E-55	1.3	MT-CO1	0.993951613
CKS1B1	1.57E-59	0.4033598	0.467	0.296	3.77E-55	1.3	CKS1B	1.577702703
DPYD	2.32E-59	0.4513326	0.704	0.497	5.59E-55	1.3	DPYD	1.416498994
DAPK12	3.11E-59	0.451233	0.673	0.516	7.49E-55	1.3	DAPK1	1.304263566
BCAS32	3.50E-59	0.3327137	0.849	0.725	8.45E-55	1.3	BCAS3	1.171034483

PSMA4	3.96E-59	0.5389847	0.589	0.422	9.56E-55	1.3	PSMA4	1.395734597
FTH11	4.16E-59	0.5152658	0.915	0.835	1.00E-54	1.3	FTH1	1.095808383
MRAS	5.08E-59	0.2097817	0.098	0.03	1.23E-54	1.3	MRAS	3.266666667
TM4SF18	6.16E-59	0.4225593	0.183	0.081	1.49E-54	1.3	TM4SF18	2.259259259
CARD61	1.23E-58	0.2207518	0.088	0.025	2.97E-54	1.3	CARD6	3.52
SNRPD12	1.39E-58	0.3577924	0.437	0.267	3.35E-54	1.3	SNRPD1	1.63670412
TBC1D5	1.52E-58	0.4445799	0.511	0.335	3.66E-54	1.3	TBC1D5	1.525373134
EPB41L1	2.63E-58	0.3121926	0.152	0.06	6.34E-54	1.3	EPB41L1	2.533333333
EIF3K2	3.61E-58	0.3593695	0.489	0.312	8.71E-54	1.3	EIF3K	1.567307692
GS1-24F4.2	4.65E-58	0.3109627	0.147	0.057	1.12E-53	1.3	GS1-24F4.2	2.578947368
METAP21	6.00E-58	0.3514917	0.387	0.227	1.45E-53	1.3	METAP2	1.704845815
DDX182	6.49E-58	0.3891171	0.436	0.271	1.56E-53	1.3	DDX18	1.608856089
GSDMC	1.38E-57	0.1623333	0.064	0.014	3.32E-53	1.3	GSDMC	4.571428571
ALPK1	1.98E-57	0.3765214	0.355	0.202	4.77E-53	1.3	ALPK1	1.757425743
CTSB	3.39E-57	0.5742065	0.423	0.275	8.18E-53	1.3	CTSB	1.538181818
NRG11	4.38E-57	0.4346599	0.166	0.07	1.06E-52	1.3	NRG1	2.371428571
DARS	5.96E-57	0.340544	0.391	0.23	1.44E-52	1.3	DARS	1.7
KANK11	9.82E-57	0.364188	0.279	0.145	2.37E-52	1.3	KANK1	1.924137931
TXNDC91	1.04E-56	0.3238616	0.215	0.101	2.50E-52	1.3	TXNDC9	2.128712871
RP11-66B24.41	1.18E-56	0.3069462	0.102	0.033	2.84E-52	1.3	RP11-66B2	3.090909091
EIF2S32	1.30E-56	0.3527226	0.44	0.275	3.13E-52	1.3	EIF2S3	1.6
ZNF6522	1.31E-56	0.4073383	0.599	0.427	3.16E-52	1.3	ZNF652	1.402810304
MPZL21	1.66E-56	0.270446	0.173	0.074	4.01E-52	1.3	MPZL2	2.337837838
DYNLT1	3.28E-56	0.3558431	0.74	0.577	7.92E-52	1.3	DYNLT1	1.282495667
PNRC11	3.67E-56	0.391496	0.579	0.398	8.86E-52	1.3	PNRC1	1.454773869
FGGY1	5.44E-56	0.3337299	0.249	0.126	1.31E-51	1.3	FGGY	1.976190476
PSMC21	1.75E-55	0.3538494	0.352	0.203	4.21E-51	1.3	PSMC2	1.733990148
GLYATL2	2.29E-55	0.2115314	0.113	0.039	5.52E-51	1.3	GLYATL2	2.897435897
MET2	2.74E-55	0.337197	0.365	0.21	6.60E-51	1.3	MET	1.738095238
PRKX1	3.44E-55	0.3141385	0.197	0.09	8.30E-51	1.3	PRKX	2.188888889
NPAS3	4.47E-55	0.3522037	0.198	0.091	1.08E-50	1.3	NPAS3	2.175824176
ATP5L2	1.03E-54	0.312591	0.879	0.801	2.49E-50	1.3	ATP5L	1.097378277
SDC41	1.13E-54	0.4598464	0.476	0.311	2.72E-50	1.3	SDC4	1.530546624
TKT	1.17E-54	0.3196769	0.197	0.091	2.81E-50	1.3	TKT	2.164835165
COBLL1	2.46E-54	0.3259798	0.167	0.072	5.93E-50	1.3	COBLL1	2.319444444
LINC01183	2.47E-54	0.2283817	0.087	0.026	5.96E-50	1.3	LINC01183	3.346153846
NR2F2-AS1	3.64E-54	0.2705318	0.153	0.063	8.79E-50	1.3	NR2F2-AS1	2.428571429
GABPB1-AS11	4.01E-54	0.3018574	0.249	0.127	9.67E-50	1.3	GABPB1-AS	1.960629921
ZNF5621	4.61E-54	0.3255314	0.272	0.143	1.11E-49	1.3	ZNF562	1.902097902
GSAP1	6.42E-54	0.2852354	0.18	0.081	1.55E-49	1.3	GSAP	2.222222222
COL6A22	7.62E-54	0.2490665	0.145	0.058	1.84E-49	1.3	COL6A2	2.5
ZCCHC22	8.41E-54	0.4457201	0.358	0.214	2.03E-49	1.3	ZCCHC2	1.672897196
IGFBP32	1.48E-53	0.3336521	0.13	0.049	3.56E-49	1.3	IGFBP3	2.653061224
KIAA0226L2	1.68E-53	0.2719123	0.118	0.043	4.04E-49	1.3	KIAA0226L	2.744186047
MOB3B1	1.86E-53	0.2904225	0.261	0.134	4.48E-49	1.3	MOB3B	1.947761194
SAMD51	1.96E-53	0.2282471	0.085	0.025	4.73E-49	1.3	SAMD5	3.4
GK5	2.63E-53	0.2496685	0.118	0.043	6.35E-49	1.3	GK5	2.744186047
SOS1	3.29E-53	0.451474	0.511	0.354	7.93E-49	1.3	SOS1	1.443502825
SLCO3A11	3.74E-53	0.3735677	0.307	0.171	9.02E-49	1.3	SLCO3A1	1.795321637
FDCSP	6.21E-53	1.038629	0.231	0.119	1.50E-48	1.3	FDCSP	1.941176471
VIM2	6.35E-53	0.3243757	0.227	0.112	1.53E-48	1.3	VIM	2.026785714
EIF3M2	8.91E-53	0.3042558	0.489	0.313	2.15E-48	1.3	EIF3M	1.562300319

LINC015491	9.05E-53	0.306776	0.057	0.013	2.18E-48	1.3	LINC01549	4.384615385
NKX1-21	1.96E-52	0.1001804	0.045	0.008	4.73E-48	1.3	NKX1-2	5.625
SLC24A3	2.38E-52	0.3294607	0.215	0.103	5.73E-48	1.3	SLC24A3	2.087378641
BPGM1	2.52E-52	0.2918944	0.177	0.079	6.07E-48	1.3	BPGM	2.240506329
RP11-793A3.21	4.07E-52	0.2077773	0.063	0.015	9.82E-48	1.3	RP11-793A	4.2
FOXN3	4.08E-52	0.3711955	0.297	0.166	9.85E-48	1.3	FOXN3	1.789156627
STAC1	4.49E-52	0.2010819	0.069	0.018	1.08E-47	1.3	STAC	3.833333333
MT-ND21	4.89E-52	0.28116	0.979	0.977	1.18E-47	1.3	MT-ND2	1.002047083
CENPW2	9.45E-52	0.2537111	0.151	0.063	2.28E-47	1.3	CENPW	2.396825397
MID12	1.33E-51	0.3102823	0.138	0.056	3.21E-47	1.3	MID1	2.464285714
C2CD2	1.38E-51	0.1851458	0.077	0.022	3.32E-47	1.3	C2CD2	3.5
CXCL161	2.30E-51	0.2771134	0.248	0.128	5.55E-47	1.3	CXCL16	1.9375
RP5-896L10.12	2.49E-51	0.2941023	0.363	0.214	6.00E-47	1.3	RP5-896L1(	1.696261682
ANK3	2.78E-51	0.4168947	0.449	0.288	6.70E-47	1.3	ANK3	1.559027778
POLR1D2	3.22E-51	0.3494852	0.355	0.21	7.76E-47	1.3	POLR1D	1.69047619
SUGT1	3.46E-51	0.2744181	0.205	0.098	8.33E-47	1.3	SUGT1	2.091836735
ZNF518A	3.49E-51	0.3294553	0.234	0.119	8.41E-47	1.3	ZNF518A	1.966386555
LDLRAD3	3.54E-51	0.3959747	0.369	0.222	8.53E-47	1.3	LDLRAD3	1.662162162
BBOX12	4.59E-51	0.3244711	0.226	0.112	1.11E-46	1.3	BBOX1	2.017857143
RPL102	6.59E-51	0.5827012	0.965	0.931	1.59E-46	1.3	RPL10	1.036519871
TOMM72	6.74E-51	0.2822228	0.888	0.807	1.62E-46	1.3	TOMM7	1.100371747
TUBA1C1	7.89E-51	0.3067728	0.436	0.274	1.90E-46	1.3	TUBA1C	1.591240876
P3H22	9.73E-51	0.270115	0.143	0.058	2.35E-46	1.3	P3H2	2.465517241
PDZRN3	2.25E-50	0.3151196	0.191	0.09	5.41E-46	1.3	PDZRN3	2.122222222
CALU1	2.32E-50	0.3165353	0.358	0.211	5.59E-46	1.3	CALU	1.696682464
FSTL11	2.62E-50	0.2583354	0.15	0.063	6.31E-46	1.3	FSTL1	2.380952381
PPP1R9A	2.99E-50	0.336495	0.265	0.143	7.20E-46	1.3	PPP1R9A	1.853146853
LINC01191	3.68E-50	0.220252	0.098	0.033	8.87E-46	1.3	LINC01191	2.96969697
SESN31	5.39E-50	0.3201872	0.114	0.042	1.30E-45	1.3	SESN3	2.714285714
SERPINE2	6.51E-50	0.2541496	0.097	0.033	1.57E-45	1.3	SERPINE2	2.939393939
USP39	6.86E-50	0.3283871	0.487	0.32	1.65E-45	1.3	USP39	1.521875
TUBA1B	7.12E-50	0.3957118	0.4	0.25	1.72E-45	1.3	TUBA1B	1.6
FUT31	1.86E-49	0.1407073	0.049	0.01	4.50E-45	1.3	FUT3	4.9
RPL122	3.11E-49	0.3993574	0.423	0.27	7.49E-45	1.3	RPL12	1.566666667
CLDN8	3.33E-49	0.2075672	0.127	0.049	8.03E-45	1.3	CLDN8	2.591836735
HADH2	3.81E-49	0.2331595	0.197	0.094	9.20E-45	1.3	HADH	2.095744681
RPS192	4.24E-49	0.8590969	0.3	0.173	1.02E-44	1.3	RPS19	1.734104046
CLEC2B2	4.77E-49	0.3227917	0.136	0.055	1.15E-44	1.3	CLEC2B	2.472727273
SUPT3H1	5.85E-49	0.3086022	0.204	0.1	1.41E-44	1.3	SUPT3H	2.04
UBASH3B1	6.50E-49	0.2816288	0.11	0.04	1.57E-44	1.3	UBASH3B	2.75
RP11-903H12.3	6.53E-49	0.148566	0.042	0.007	1.58E-44	1.3	RP11-903H	6
ATP5F11	6.89E-49	0.3368175	0.525	0.355	1.66E-44	1.3	ATP5F1	1.478873239
TMEM126B	8.48E-49	0.2466972	0.2	0.096	2.04E-44	1.3	TMEM126E	2.083333333
VPS13D	9.34E-49	0.3614634	0.414	0.261	2.25E-44	1.3	VPS13D	1.586206897
RBM8A1	1.09E-48	0.350038	0.577	0.412	2.62E-44	1.3	RBM8A	1.400485437
ZNF221	1.21E-48	0.2979295	0.274	0.151	2.92E-44	1.3	ZNF22	1.814569536
ZBTB381	1.27E-48	0.3536847	0.47	0.308	3.07E-44	1.3	ZBTB38	1.525974026
RPL272	1.76E-48	0.2426038	0.942	0.905	4.24E-44	1.3	RPL27	1.040883978
GLIPR12	1.83E-48	0.1541401	0.311	0.17	4.41E-44	1.3	GLIPR1	1.829411765
RAN2	1.90E-48	0.3324343	0.669	0.512	4.57E-44	1.3	RAN	1.306640625
PSMD111	6.71E-48	0.3722197	0.386	0.242	1.62E-43	1.3	PSMD11	1.595041322
UGT2B71	8.07E-48	0.1890674	0.069	0.019	1.95E-43	1.3	UGT2B7	3.631578947

MTMR21	1.01E-47	0.2579122	0.146	0.062	2.45E-43	1.3	MTMR2	2.35483871
FTX	1.46E-47	0.2932142	0.738	0.535	3.53E-43	1.3	FTX	1.379439252
PLRG1	1.60E-47	0.2419037	0.161	0.072	3.87E-43	1.3	PLRG1	2.236111111
CDCP11	2.11E-47	0.3239941	0.419	0.261	5.08E-43	1.3	CDCP1	1.605363985
RARS1	2.31E-47	0.3151015	0.347	0.207	5.57E-43	1.3	RARS	1.676328502
MRPS18C1	2.55E-47	0.2860936	0.254	0.137	6.14E-43	1.3	MRPS18C	1.854014599
SNX241	2.76E-47	0.3333363	0.279	0.157	6.67E-43	1.3	SNX24	1.777070064
HSD17B7	3.93E-47	0.3229009	0.17	0.079	9.49E-43	1.3	HSD17B7	2.151898734
PNN2	5.43E-47	0.3114697	0.468	0.306	1.31E-42	1.3	PNN	1.529411765
MTHFD1L2	5.48E-47	0.3320238	0.341	0.202	1.32E-42	1.3	MTHFD1L	1.688118812
B4GALT6	6.85E-47	0.1514706	0.066	0.018	1.65E-42	1.3	B4GALT6	3.666666667
MPP61	7.76E-47	0.2097192	0.11	0.041	1.87E-42	1.3	MPP6	2.682926829
WIPF31	8.40E-47	0.1811324	0.073	0.021	2.03E-42	1.3	WIPF3	3.476190476
NSRP11	9.02E-47	0.3358258	0.33	0.196	2.17E-42	1.3	NSRP1	1.683673469
PARP41	1.31E-46	0.3288372	0.257	0.14	3.17E-42	1.3	PARP4	1.835714286
FRAS11	1.52E-46	0.1883123	0.094	0.032	3.67E-42	1.3	FRAS1	2.9375
INVS	1.77E-46	0.3134354	0.187	0.09	4.26E-42	1.3	INVS	2.077777778
ERH	2.65E-46	0.3096844	0.658	0.487	6.40E-42	1.3	ERH	1.351129363
SIK32	3.44E-46	0.2569736	0.977	0.972	8.29E-42	1.3	SIK3	1.005144033
ODAM	3.52E-46	0.2144929	0.043	0.008	8.48E-42	1.3	ODAM	5.375
CDC26	4.65E-46	0.2862288	0.296	0.169	1.12E-41	1.3	CDC26	1.75147929
RP11-712B9.2	4.71E-46	0.2267657	0.081	0.026	1.14E-41	1.3	RP11-712B	3.115384615
EIF2B51	4.76E-46	0.4484045	0.579	0.447	1.15E-41	1.3	EIF2B5	1.295302013
RHOJ	5.46E-46	0.1481754	0.062	0.016	1.32E-41	1.3	RHOJ	3.875
LINC004862	5.91E-46	0.2062122	0.993	0.986	1.42E-41	1.3	LINC00486	1.007099391
AC009313.12	6.03E-46	0.2779719	0.132	0.055	1.45E-41	1.3	AC009313.	2.4
MECOM2	6.98E-46	0.2812811	0.883	0.794	1.68E-41	1.3	MECOM	1.11209068
SEL1L31	1.13E-45	0.2091918	0.081	0.026	2.71E-41	1.3	SEL1L3	3.115384615
SRPK11	1.18E-45	0.3364365	0.633	0.469	2.84E-41	1.3	SRPK1	1.349680171
SLC35F31	1.30E-45	0.2044263	0.082	0.026	3.13E-41	1.3	SLC35F3	3.153846154
KARS2	1.40E-45	0.3206889	0.373	0.231	3.38E-41	1.3	KARS	1.614718615
TCF7L12	1.56E-45	0.2812829	0.299	0.17	3.76E-41	1.3	TCF7L1	1.758823529
PLEKHH22	1.79E-45	0.2632935	0.137	0.058	4.32E-41	1.3	PLEKHH2	2.362068966
CCSER1	4.52E-45	0.2732073	0.6	0.423	1.09E-40	1.3	CCSER1	1.418439716
MCTS11	5.18E-45	0.3035214	0.336	0.201	1.25E-40	1.3	MCTS1	1.671641791
SLC5A11	5.82E-45	0.2148623	0.114	0.044	1.40E-40	1.3	SLC5A1	2.590909091
RPS92	7.52E-45	0.7811098	0.349	0.215	1.81E-40	1.3	RPS9	1.623255814
SF3B31	1.45E-44	0.2898766	0.251	0.137	3.50E-40	1.3	SF3B3	1.832116788
TXN1	1.48E-44	0.3559922	0.771	0.664	3.56E-40	1.3	TXN	1.161144578
PPP1R14C2	1.80E-44	0.2613479	0.182	0.087	4.35E-40	1.3	PPP1R14C	2.091954023
CCT82	2.63E-44	0.3097086	0.414	0.266	6.34E-40	1.3	CCT8	1.556390977
DCHS2	3.16E-44	0.3062079	0.149	0.066	7.63E-40	1.3	DCHS2	2.257575758
S100A22	3.63E-44	0.308033	0.121	0.049	8.75E-40	1.3	S100A2	2.469387755
RP11-66B24.51	4.04E-44	0.2510274	0.113	0.044	9.73E-40	1.3	RP11-66B2	2.568181818
NIPAL3	4.17E-44	0.2321855	0.12	0.048	1.00E-39	1.3	NIPAL3	2.5
PTN2	4.64E-44	0.4046572	0.203	0.103	1.12E-39	1.3	PTN	1.970873786
RPL10A2	4.66E-44	0.381184	0.791	0.708	1.12E-39	1.3	RPL10A	1.117231638
AMBRA11	5.13E-44	0.5108732	0.75	0.668	1.24E-39	1.3	AMBRA1	1.122754491
RPL242	5.60E-44	0.2461127	0.942	0.913	1.35E-39	1.3	RPL24	1.031763417
KRT232	6.74E-44	0.302504	0.249	0.136	1.62E-39	1.3	KRT23	1.830882353
C15orf41	1.19E-43	0.24494	0.112	0.044	2.88E-39	1.3	C15orf41	2.545454545
LOXL4	1.21E-43	0.1137547	0.031	0.004	2.92E-39	1.3	LOXL4	7.75

SMC52	1.32E-43	0.3051689	0.442	0.289	3.17E-39	1.3	SMC5	1.529411765
HNRNPA12	1.33E-43	0.1809192	0.874	0.745	3.20E-39	1.3	HNRNPA1	1.173154362
FAM172A2	1.43E-43	0.3262011	0.752	0.601	3.44E-39	1.3	FAM172A	1.25124792
LAP3	1.66E-43	0.2956913	0.208	0.108	4.01E-39	1.3	LAP3	1.925925926
PKD2	2.03E-43	0.2456608	0.121	0.049	4.89E-39	1.3	PKD2	2.469387755
NR3C21	3.42E-43	0.2839967	0.16	0.075	8.26E-39	1.3	NR3C2	2.133333333
COL6A11	3.45E-43	0.1815532	0.061	0.016	8.32E-39	1.3	COL6A1	3.8125
EIF3I2	4.73E-43	0.3381851	0.717	0.58	1.14E-38	1.3	EIF3I	1.236206897
SLC4A71	4.77E-43	0.3361554	0.408	0.266	1.15E-38	1.3	SLC4A7	1.533834586
PLOD11	6.06E-43	0.1893818	0.102	0.038	1.46E-38	1.3	PLOD1	2.684210526
ANPEP1	7.82E-43	0.2080852	0.061	0.017	1.89E-38	1.3	ANPEP	3.588235294
TUSC31	1.30E-42	0.2622661	0.192	0.096	3.14E-38	1.3	TUSC3	2
VMP11	1.51E-42	0.3352938	0.838	0.736	3.64E-38	1.3	VMP1	1.138586957
FAM60A2	1.88E-42	0.3732124	0.441	0.294	4.54E-38	1.3	FAM60A	1.5
LINC003421	2.46E-42	0.2111948	0.092	0.033	5.92E-38	1.3	LINC00342	2.787878788
PGM1	2.51E-42	0.3233444	0.16	0.075	6.05E-38	1.3	PGM1	2.133333333
PLEKHA72	2.91E-42	0.1885805	0.977	0.98	7.02E-38	1.3	PLEKHA7	0.996938776
FAM157C1	3.49E-42	0.2606513	0.16	0.075	8.43E-38	1.3	FAM157C	2.133333333
EBP	3.83E-42	0.2613244	0.205	0.106	9.23E-38	1.3	EBP	1.933962264
KIFC3	4.02E-42	0.1694977	0.07	0.021	9.70E-38	1.3	KIFC3	3.333333333
OTUD6B-AS1	4.07E-42	0.2648523	0.279	0.159	9.82E-38	1.3	OTUD6B-AS	1.754716981
TPT1-AS12	4.17E-42	0.2685836	0.36	0.22	1.01E-37	1.3	TPT1-AS1	1.636363636
CTC-471J1.92	4.88E-42	0.2950131	0.765	0.62	1.18E-37	1.3	CTC-471J1.	1.233870968
FRMD51	5.77E-42	0.3460714	0.226	0.123	1.39E-37	1.3	FRMD5	1.837398374
MAGED1	5.97E-42	0.2826812	0.166	0.08	1.44E-37	1.3	MAGED1	2.075
AKAP6	7.54E-42	0.1962657	0.079	0.026	1.82E-37	1.3	AKAP6	3.038461538
TMEM1232	8.86E-42	0.3283287	0.433	0.288	2.14E-37	1.3	TMEM123	1.503472222
NLGN4X1	1.07E-41	0.2591566	0.102	0.039	2.58E-37	1.3	NLGN4X	2.615384615
RPS22	1.07E-41	0.4087664	0.401	0.26	2.58E-37	1.3	RPS2	1.542307692
MDH11	1.12E-41	0.3137786	0.403	0.261	2.70E-37	1.3	MDH1	1.544061303
CACNB2	1.17E-41	0.3255001	0.176	0.087	2.82E-37	1.3	CACNB2	2.022988506
PID12	1.26E-41	0.3348641	0.148	0.068	3.03E-37	1.3	PID1	2.176470588
NMI	1.28E-41	0.2200596	0.145	0.066	3.10E-37	1.3	NMI	2.196969697
HELB	1.47E-41	0.2670289	0.186	0.093	3.54E-37	1.3	HELB	2
AARD	2.00E-41	0.1065907	0.04	0.008	4.83E-37	1.3	AARD	5
RP11-77K12.4	2.87E-41	0.126865	0.054	0.014	6.93E-37	1.3	RP11-77K1	3.857142857
B3GNT52	4.82E-41	0.2597695	0.119	0.05	1.16E-36	1.3	B3GNT5	2.38
SPDYE161	6.66E-41	0.2254011	0.127	0.055	1.61E-36	1.3	SPDYE16	2.309090909
SSR32	7.22E-41	0.2527839	0.596	0.426	1.74E-36	1.3	SSR3	1.399061033
C1RL	8.37E-41	0.2548057	0.151	0.07	2.02E-36	1.3	C1RL	2.157142857
RNF2171	1.16E-40	0.2596406	0.135	0.06	2.80E-36	1.3	RNF217	2.25
C1S	1.94E-40	0.2234153	0.138	0.062	4.67E-36	1.3	C1S	2.225806452
PDE9A1	2.79E-40	0.2011424	0.093	0.034	6.74E-36	1.3	PDE9A	2.735294118
ZNF33B	3.22E-40	0.2801331	0.228	0.124	7.77E-36	1.3	ZNF33B	1.838709677
HMGA12	4.56E-40	0.3340738	0.158	0.076	1.10E-35	1.3	HMGA1	2.078947368
RASGRP1	6.12E-40	0.2073858	0.138	0.062	1.48E-35	1.3	RASGRP1	2.225806452
FGF23	1.19E-39	0.2322327	0.096	0.037	2.87E-35	1.3	FGF2	2.594594595
TC2N1	1.32E-39	0.3108061	0.41	0.27	3.18E-35	1.3	TC2N	1.518518519
C8orf592	1.41E-39	0.285013	0.414	0.271	3.40E-35	1.3	C8orf59	1.527675277
MRPL13	1.45E-39	0.2600172	0.27	0.155	3.50E-35	1.3	MRPL13	1.741935484
TNFSF10	1.49E-39	0.1047139	0.638	0.453	3.60E-35	1.3	TNFSF10	1.408388521
APP	1.65E-39	0.3724386	0.866	0.764	3.98E-35	1.3	APP	1.133507853

OSBPL101	1.90E-39	0.3424106	0.185	0.094	4.58E-35	1.3	OSBPL10	1.968085106
PELI21	2.34E-39	0.2168832	0.11	0.045	5.63E-35	1.3	PELI2	2.444444444
XDH2	2.39E-39	0.3094656	0.12	0.051	5.76E-35	1.3	XDH	2.352941176
CALM1	2.41E-39	0.3129369	0.336	0.209	5.82E-35	1.3	CALM1	1.607655502
ANKRD62	2.50E-39	0.2041799	0.107	0.043	6.03E-35	1.3	ANKRD6	2.488372093
GGPS1	2.94E-39	0.2581174	0.299	0.178	7.09E-35	1.3	GGPS1	1.679775281
RBX1	3.28E-39	0.3014094	0.575	0.423	7.90E-35	1.3	RBX1	1.359338061
UBE2V21	4.08E-39	0.2872385	0.368	0.236	9.85E-35	1.3	UBE2V2	1.559322034
MOB4	4.32E-39	0.2547385	0.256	0.146	1.04E-34	1.3	MOB4	1.753424658
EPB41L22	4.37E-39	0.1708744	0.198	0.102	1.05E-34	1.3	EPB41L2	1.941176471
ARHGAP24	5.19E-39	0.2515966	0.08	0.028	1.25E-34	1.3	ARHGAP24	2.857142857
MINK11	5.32E-39	0.2722115	0.177	0.09	1.28E-34	1.3	MINK1	1.966666667
VPS292	5.57E-39	0.3158902	0.45	0.305	1.34E-34	1.3	VPS29	1.475409836
MBP1	7.90E-39	0.2676884	0.201	0.106	1.91E-34	1.3	MBP	1.896226415
MED31	1.02E-38	0.2715216	0.28	0.165	2.47E-34	1.3	MED31	1.696969697
SPIDR	1.09E-38	0.3254078	0.746	0.584	2.63E-34	1.3	SPIDR	1.27739726
UCK21	1.12E-38	0.234686	0.177	0.089	2.69E-34	1.3	UCK2	1.988764045
ARHGEF102	1.30E-38	0.2373026	0.335	0.206	3.14E-34	1.3	ARHGEF10	1.626213592
LSM81	1.35E-38	0.2207236	0.32	0.193	3.25E-34	1.3	LSM8	1.658031088
FOXO11	1.72E-38	0.305847	0.299	0.181	4.14E-34	1.3	FOXO1	1.651933702
RNF13	1.85E-38	0.3096922	0.395	0.259	4.47E-34	1.3	RNF13	1.525096525
R3HDM2	1.86E-38	0.3119308	0.203	0.108	4.48E-34	1.3	R3HDM2	1.87962963
MT-CYB2	1.93E-38	0.18166	0.986	0.992	4.66E-34	1.3	MT-CYB	0.993951613
FAM3B	1.95E-38	0.1807682	0.097	0.037	4.71E-34	1.3	FAM3B	2.621621622
DNER	2.05E-38	0.3080781	0.098	0.039	4.94E-34	1.3	DNER	2.512820513
LRRC75A2	2.18E-38	0.3472456	0.227	0.127	5.25E-34	1.3	LRRC75A	1.787401575
SLC23A21	2.42E-38	0.1730073	0.086	0.031	5.84E-34	1.3	SLC23A2	2.774193548
PGBD51	2.52E-38	0.1898028	0.09	0.034	6.07E-34	1.3	PGBD5	2.647058824
C15orf48	2.84E-38	0.7437974	0.593	0.488	6.84E-34	1.3	C15orf48	1.215163934
LINC009691	3.53E-38	0.2515565	0.317	0.194	8.50E-34	1.3	LINC00969	1.634020619
RBPMS2	3.83E-38	0.2849917	0.794	0.643	9.23E-34	1.3	RBPMS	1.234836703
RAB29	5.63E-38	0.1753373	0.106	0.043	1.36E-33	1.3	RAB29	2.465116279
TRMT10C1	6.75E-38	0.2048139	0.166	0.082	1.63E-33	1.3	TRMT10C	2.024390244
C1R	1.27E-37	0.2046463	0.086	0.032	3.06E-33	1.3	C1R	2.6875
SMIM81	1.32E-37	0.1911506	0.119	0.051	3.18E-33	1.3	SMIM8	2.333333333
FIGN	2.00E-37	0.3172097	0.182	0.094	4.83E-33	1.3	FIGN	1.936170213
RSL1D12	2.08E-37	0.1975464	0.603	0.434	5.03E-33	1.3	RSL1D1	1.389400922
YBX11	2.10E-37	0.3245062	0.413	0.28	5.06E-33	1.3	YBX1	1.475
HIBADH1	2.84E-37	0.2820552	0.206	0.112	6.85E-33	1.3	HIBADH	1.839285714
IDI1	3.57E-37	0.2607127	0.333	0.209	8.60E-33	1.3	IDI1	1.593301435
CEBPZOS	3.96E-37	0.2446059	0.199	0.106	9.55E-33	1.3	CEBPZOS	1.877358491
VDAC32	5.05E-37	0.2799174	0.388	0.254	1.22E-32	1.3	VDAC3	1.527559055
GAPDH	5.45E-37	0.5220987	0.682	0.552	1.32E-32	1.3	GAPDH	1.235507246
ZNF33A	8.44E-37	0.2791655	0.326	0.203	2.03E-32	1.3	ZNF33A	1.60591133
STIL1	8.98E-37	0.2110841	0.105	0.044	2.16E-32	1.3	STIL	2.386363636
FUT9	1.32E-36	0.1255055	0.052	0.014	3.17E-32	1.3	FUT9	3.714285714
SNX23	1.36E-36	0.2344188	0.225	0.125	3.27E-32	1.3	SNX2	1.8
ESD2	1.85E-36	0.2296419	0.448	0.299	4.47E-32	1.3	ESD	1.498327759
SAV11	1.86E-36	0.2902794	0.42	0.28	4.48E-32	1.3	SAV1	1.5
NRG2	2.42E-36	0.2046489	0.072	0.025	5.83E-32	1.3	NRG2	2.88
NAPG	3.41E-36	0.2449629	0.283	0.169	8.23E-32	1.3	NAPG	1.674556213
RAP1GAP2	3.50E-36	0.3349006	0.231	0.132	8.44E-32	1.3	RAP1GAP2	1.75

OLFM4	3.74E-36	0.3333829	0.053	0.015	9.01E-32	1.3	OLFM4	3.533333333
MRPS14	3.81E-36	0.2245056	0.217	0.12	9.18E-32	1.3	MRPS14	1.808333333
PDCD101	4.14E-36	0.290783	0.368	0.239	9.99E-32	1.3	PDCD10	1.539748954
UTP202	4.68E-36	0.1869535	0.103	0.042	1.13E-31	1.3	UTP20	2.452380952
ARPC3	5.11E-36	0.2915906	0.766	0.649	1.23E-31	1.3	ARPC3	1.18027735
GRB142	5.20E-36	0.3079709	0.54	0.394	1.25E-31	1.3	GRB14	1.370558376
NFIX1	5.57E-36	0.2581814	0.13	0.06	1.34E-31	1.3	NFIX	2.166666667
BTF3L4	6.99E-36	0.2518454	0.344	0.22	1.69E-31	1.3	BTF3L4	1.563636364
SSR22	7.47E-36	0.2529681	0.79	0.673	1.80E-31	1.3	SSR2	1.17384844
ATP1A1	8.60E-36	0.3201923	0.635	0.491	2.07E-31	1.3	ATP1A1	1.293279022
HSDL2	8.97E-36	0.2151537	0.155	0.076	2.16E-31	1.3	HSDL2	2.039473684
UQCRHL2	1.04E-35	0.2335007	0.285	0.171	2.51E-31	1.3	UQCRHL	1.666666667
ASCC1	1.40E-35	0.2541957	0.231	0.131	3.37E-31	1.3	ASCC1	1.763358779
MARCH3	1.57E-35	0.3725644	0.244	0.144	3.79E-31	1.3	MARCH3	1.694444444
ARL15	1.82E-35	0.2980249	0.313	0.196	4.38E-31	1.3	ARL15	1.596938776
LY96	1.88E-35	0.1140408	0.041	0.01	4.53E-31	1.3	LY96	4.1
ACO1	1.89E-35	0.1506765	0.086	0.033	4.55E-31	1.3	ACO1	2.606060606
AIMP12	2.00E-35	0.2764819	0.425	0.287	4.82E-31	1.3	AIMP1	1.480836237
RP1-28O10.1	2.20E-35	0.1553749	0.084	0.031	5.30E-31	1.3	RP1-28O10	2.709677419
CRTC3	2.26E-35	0.2743354	0.313	0.197	5.45E-31	1.3	CRTC3	1.588832487
SOX62	3.35E-35	0.3050363	0.153	0.076	8.08E-31	1.3	SOX6	2.013157895
DDR21	3.99E-35	0.2724797	0.147	0.072	9.62E-31	1.3	DDR2	2.041666667
PELI12	4.23E-35	0.2679148	0.459	0.314	1.02E-30	1.3	PELI1	1.461783439
ATP1B31	4.77E-35	0.292125	0.367	0.242	1.15E-30	1.3	ATP1B3	1.516528926
RGL1	6.70E-35	0.1788859	0.107	0.046	1.62E-30	1.3	RGL1	2.326086957
HGSNAT	9.29E-35	0.2526627	0.219	0.124	2.24E-30	1.3	HGSNAT	1.766129032
RASSF82	9.77E-35	0.2704111	0.183	0.097	2.36E-30	1.3	RASSF8	1.886597938
TRIM561	1.00E-34	0.2777391	0.244	0.143	2.41E-30	1.3	TRIM56	1.706293706
RSU1	1.18E-34	0.3060033	0.292	0.182	2.83E-30	1.3	RSU1	1.604395604
PSAT11	1.38E-34	0.1127429	0.046	0.012	3.33E-30	1.3	PSAT1	3.833333333
BBS121	1.63E-34	0.1143749	0.045	0.012	3.93E-30	1.3	BBS12	3.75
SIRPA	1.97E-34	0.1489193	0.081	0.03	4.74E-30	1.3	SIRPA	2.7
ZNF124	2.97E-34	0.1862483	0.111	0.048	7.16E-30	1.3	ZNF124	2.3125
TNS3	3.46E-34	0.2896544	0.132	0.063	8.35E-30	1.3	TNS3	2.095238095
HINT11	3.93E-34	0.3060311	0.71	0.589	9.47E-30	1.3	HINT1	1.205432937
TPM42	4.14E-34	0.3812522	0.493	0.359	9.97E-30	1.3	TPM4	1.373259053
FAM3C	7.36E-34	0.2910004	0.227	0.131	1.77E-29	1.3	FAM3C	1.732824427
TFCP2	7.60E-34	0.2324668	0.188	0.102	1.83E-29	1.3	TFCP2	1.843137255
RP11-631N16.21	7.89E-34	0.1879098	0.092	0.037	1.90E-29	1.3	RP11-631N	2.486486486
LINC00693	7.93E-34	0.1648062	0.054	0.016	1.91E-29	1.3	LINC00693	3.375
CYB5B	8.73E-34	0.2193024	0.22	0.124	2.11E-29	1.3	CYB5B	1.774193548
C11orf80	8.78E-34	0.2871748	0.283	0.175	2.12E-29	1.3	C11orf80	1.617142857
RP11-817J15.22	8.94E-34	0.1497608	0.107	0.046	2.16E-29	1.3	RP11-817J1	2.326086957
DNAJC62	9.54E-34	0.2125021	0.12	0.054	2.30E-29	1.3	DNAJC6	2.222222222
RNF144B	1.04E-33	0.3426094	0.398	0.274	2.50E-29	1.3	RNF144B	1.452554745
ZNF708	1.14E-33	0.2380549	0.128	0.06	2.75E-29	1.3	ZNF708	2.133333333
ACSS21	1.33E-33	0.2509406	0.248	0.146	3.21E-29	1.3	ACSS2	1.698630137
TUBB61	2.83E-33	0.1493593	0.073	0.026	6.83E-29	1.3	TUBB6	2.807692308
CHCHD32	3.13E-33	0.259972	0.89	0.83	7.55E-29	1.3	CHCHD3	1.072289157
C5orf281	3.37E-33	0.2341755	0.21	0.118	8.12E-29	1.3	C5orf28	1.779661017
DTX21	3.48E-33	0.2378235	0.127	0.06	8.40E-29	1.3	DTX2	2.116666667
PDGFC1	3.55E-33	0.2373352	0.333	0.212	8.56E-29	1.3	PDGFC	1.570754717

XKR6	3.73E-33	0.30906	0.245	0.147	8.99E-29	1.3	XKR6	1.666666667
TRPS1	3.89E-33	0.3073221	0.846	0.725	9.38E-29	1.3	TRPS1	1.166896552
CEACAM11	3.94E-33	0.1895309	0.141	0.069	9.50E-29	1.3	CEACAM1	2.043478261
CECR71	4.08E-33	0.1676117	0.094	0.039	9.84E-29	1.3	CECR7	2.41025641
FBXO2	4.25E-33	0.1246862	0.049	0.014	1.03E-28	1.3	FBXO2	3.5
SPX	4.82E-33	0.1245169	0.06	0.02	1.16E-28	1.3	SPX	3
PSME21	5.71E-33	0.4632211	0.395	0.281	1.38E-28	1.3	PSME2	1.40569395
DDX242	6.10E-33	0.239248	0.644	0.49	1.47E-28	1.3	DDX24	1.314285714
ST5	6.83E-33	0.1630691	0.476	0.325	1.65E-28	1.3	ST5	1.464615385
PLAGL12	7.46E-33	0.181499	0.086	0.034	1.80E-28	1.3	PLAGL1	2.529411765
NXN2	7.81E-33	0.2104832	0.166	0.086	1.88E-28	1.3	NXN	1.930232558
SPARCL12	9.85E-33	0.182761	0.086	0.034	2.38E-28	1.3	SPARCL1	2.529411765
SESTD12	1.03E-32	0.2750933	0.619	0.472	2.50E-28	1.3	SESTD1	1.311440678
IRAK2	1.07E-32	0.2669277	0.21	0.119	2.59E-28	1.3	IRAK2	1.764705882
TCN1	1.11E-32	0.2829862	0.053	0.016	2.67E-28	1.3	TCN1	3.3125
LRRC16A1	1.31E-32	0.2861727	0.253	0.152	3.16E-28	1.3	LRRC16A	1.664473684
C1QTNF1	1.44E-32	0.1471866	0.057	0.018	3.47E-28	1.3	C1QTNF1	3.166666667
ATR	1.78E-32	0.2276555	0.223	0.128	4.30E-28	1.3	ATR	1.7421875
NDUFS52	2.41E-32	0.246739	0.847	0.763	5.81E-28	1.3	NDUFS5	1.110091743
CAV11	2.64E-32	0.1482031	0.072	0.026	6.36E-28	1.3	CAV1	2.769230769
CCAR1	2.90E-32	0.2876327	0.348	0.229	6.98E-28	1.3	CCAR1	1.519650655
MAPK14	3.02E-32	0.2566026	0.244	0.145	7.27E-28	1.3	MAPK14	1.682758621
DHCR24	3.36E-32	0.2257204	0.286	0.177	8.09E-28	1.3	DHCR24	1.615819209
CSTB1	6.16E-32	0.4211735	0.226	0.135	1.48E-27	1.3	CSTB	1.674074074
CCDC1461	6.73E-32	0.27279	0.22	0.129	1.62E-27	1.3	CCDC146	1.705426357
TMOD11	6.89E-32	0.2362421	0.139	0.069	1.66E-27	1.3	TMOD1	2.014492754
PLA2G161	8.18E-32	0.2883279	0.162	0.086	1.97E-27	1.3	PLA2G16	1.88372093
KNOP1	8.58E-32	0.2150923	0.232	0.136	2.07E-27	1.3	KNOP1	1.705882353
TPM11	9.05E-32	0.3468497	0.645	0.512	2.18E-27	1.3	TPM1	1.259765625
TMCO4	1.03E-31	0.214322	0.127	0.061	2.49E-27	1.3	TMCO4	2.081967213
DIAPH2-AS1	1.15E-31	0.1589813	0.064	0.022	2.78E-27	1.3	DIAPH2-AS	2.909090909
SNED1	1.20E-31	0.1564482	0.076	0.029	2.88E-27	1.3	SNED1	2.620689655
CLINT12	1.30E-31	0.2328665	0.361	0.235	3.14E-27	1.3	CLINT1	1.536170213
CSTA	1.36E-31	0.1058457	0.061	0.02	3.27E-27	1.3	CSTA	3.05
FAF1	1.66E-31	0.3214812	0.335	0.22	4.00E-27	1.3	FAF1	1.522727273
DOCK1	1.83E-31	0.3136089	0.406	0.278	4.42E-27	1.3	DOCK1	1.460431655
STEAP3	2.25E-31	0.1555545	0.066	0.023	5.43E-27	1.3	STEAP3	2.869565217
OGFRL11	3.59E-31	0.2275977	0.28	0.174	8.65E-27	1.3	OGFRL1	1.609195402
PITPNC11	4.85E-31	0.3175926	0.262	0.164	1.17E-26	1.3	PITPNC1	1.597560976
PDSS2	6.76E-31	0.2560169	0.204	0.117	1.63E-26	1.3	PDSS2	1.743589744
RPS82	6.83E-31	0.3777277	0.787	0.732	1.65E-26	1.3	RPS8	1.075136612
RANBP171	6.99E-31	0.2617432	0.175	0.095	1.69E-26	1.3	RANBP17	1.842105263
CMIP2	7.31E-31	0.3060254	0.373	0.254	1.76E-26	1.3	CMIP	1.468503937
SNRPE2	7.59E-31	0.2401356	0.648	0.506	1.83E-26	1.3	SNRPE	1.280632411
AGPS1	7.77E-31	0.1915618	0.087	0.036	1.87E-26	1.3	AGPS	2.416666667
LINC01138	8.09E-31	0.257775	0.257	0.159	1.95E-26	1.3	LINC01138	1.616352201
ENPP6	8.94E-31	0.1176662	0.062	0.021	2.16E-26	1.3	ENPP6	2.952380952
XPNPEP11	1.05E-30	0.1904874	0.147	0.076	2.53E-26	1.3	XPNPEP1	1.934210526
FBXO321	1.21E-30	0.517713	0.469	0.358	2.91E-26	1.3	FBXO32	1.310055866
RP11-366L20.2	1.31E-30	0.1006847	0.037	0.009	3.17E-26	1.3	RP11-366L:	4.111111111
BPNT1	1.69E-30	0.1563	0.093	0.04	4.09E-26	1.3	BPNT1	2.325
PRKCE	2.50E-30	0.3484593	0.289	0.186	6.03E-26	1.3	PRKCE	1.553763441

LINC-PINT	2.64E-30	0.3384954	0.703	0.561	6.37E-26	1.3	LINC-PINT	1.25311943
CRIPT	2.92E-30	0.2136172	0.276	0.172	7.04E-26	1.3	CRIPT	1.604651163
AKT32	3.15E-30	0.2744186	0.466	0.332	7.60E-26	1.3	AKT3	1.403614458
AC008074.31	3.58E-30	0.1621801	0.1	0.044	8.64E-26	1.3	AC008074.	2.272727273
CLDN161	3.68E-30	0.1262039	0.052	0.017	8.87E-26	1.3	CLDN16	3.058823529
TNFRSF21	3.84E-30	0.1893067	0.107	0.049	9.25E-26	1.3	TNFRSF21	2.183673469
RPS52	3.99E-30	0.5791431	0.319	0.215	9.63E-26	1.3	RPS5	1.48372093
NEMF1	4.07E-30	0.2370729	0.373	0.252	9.81E-26	1.3	NEMF	1.48015873
HTATIP2	4.63E-30	0.1967123	0.161	0.086	1.12E-25	1.3	HTATIP2	1.872093023
DDIT42	4.89E-30	0.3069721	0.243	0.15	1.18E-25	1.3	DDIT4	1.62
SNCA	5.05E-30	0.1254325	0.037	0.009	1.22E-25	1.3	SNCA	4.111111111
PSMB8	5.54E-30	0.1964463	0.141	0.072	1.34E-25	1.3	PSMB8	1.958333333
PAK32	6.43E-30	0.1437183	0.092	0.039	1.55E-25	1.3	PAK3	2.358974359
RPL132	6.84E-30	0.3277314	0.537	0.405	1.65E-25	1.3	RPL13	1.325925926
BMS1	9.65E-30	0.2060829	0.145	0.075	2.33E-25	1.3	BMS1	1.933333333
NCK11	1.02E-29	0.2325976	0.178	0.099	2.45E-25	1.3	NCK1	1.797979798
RP11-230B22.1	1.32E-29	0.1224063	0.048	0.014	3.18E-25	1.3	RP11-230B	3.428571429
POU2F31	1.94E-29	0.2215497	0.186	0.105	4.68E-25	1.3	POU2F3	1.771428571
ZNF1211	2.10E-29	0.1951033	0.121	0.058	5.05E-25	1.3	ZNF121	2.086206897
KIAA11471	2.58E-29	0.1924052	0.12	0.058	6.21E-25	1.3	KIAA1147	2.068965517
MYO3B2	2.58E-29	0.3802624	0.229	0.142	6.22E-25	1.3	MYO3B	1.612676056
MFSD6	2.58E-29	0.1984668	0.128	0.064	6.23E-25	1.3	MFSD6	2
TNIP3	2.95E-29	0.1566549	0.08	0.033	7.11E-25	1.3	TNIP3	2.424242424
RP11-356C4.52	3.08E-29	0.2593483	0.231	0.139	7.42E-25	1.3	RP11-356C	1.661870504
PLEKHA5	3.24E-29	0.2340022	0.521	0.377	7.81E-25	1.3	PLEKHA5	1.381962865
LARP11	3.33E-29	0.2269299	0.215	0.128	8.02E-25	1.3	LARP1	1.6796875
ZFR1	3.36E-29	0.2684339	0.438	0.31	8.10E-25	1.3	ZFR	1.412903226
UQCRC22	3.78E-29	0.2170375	0.45	0.316	9.11E-25	1.3	UQCRC2	1.424050633
MRPL451	3.79E-29	0.1835153	0.231	0.137	9.14E-25	1.3	MRPL45	1.686131387
CLIC42	4.13E-29	0.3432868	0.678	0.547	9.95E-25	1.3	CLIC4	1.239488117
DRAM2	5.30E-29	0.2425555	0.313	0.205	1.28E-24	1.3	DRAM2	1.526829268
DNTTIP21	5.90E-29	0.2010943	0.257	0.159	1.42E-24	1.3	DNTTIP2	1.616352201
QDPR	7.91E-29	0.1680679	0.095	0.042	1.91E-24	1.3	QDPR	2.261904762
LPIN12	8.19E-29	0.1387248	0.306	0.194	1.98E-24	1.3	LPIN1	1.577319588
ADGRA31	8.74E-29	0.1921721	0.142	0.073	2.11E-24	1.3	ADGRA3	1.945205479
PPL1	9.45E-29	0.2212045	0.127	0.063	2.28E-24	1.3	PPL	2.015873016
SNW12	1.02E-28	0.2046258	0.305	0.195	2.46E-24	1.3	SNW1	1.564102564
EVA1A	1.13E-28	0.1106525	0.041	0.011	2.72E-24	1.3	EVA1A	3.727272727
PHF5A1	1.31E-28	0.1889648	0.242	0.146	3.16E-24	1.3	PHF5A	1.657534247
AC005042.41	1.32E-28	0.1870608	0.087	0.037	3.18E-24	1.3	AC005042.	2.351351351
ZFYVE9	1.40E-28	0.2266789	0.143	0.075	3.38E-24	1.3	ZFYVE9	1.906666667
RP11-307P5.11	1.66E-28	0.153381	0.054	0.018	3.99E-24	1.3	RP11-307P.	3
CMTM81	2.19E-28	0.2591362	0.251	0.156	5.28E-24	1.3	CMTM8	1.608974359
PTRH21	2.68E-28	0.2218547	0.221	0.131	6.46E-24	1.3	PTRH2	1.687022901
VRK2	2.83E-28	0.216991	0.214	0.128	6.82E-24	1.3	VRK2	1.671875
THOC7	2.95E-28	0.1581555	0.138	0.071	7.12E-24	1.3	THOC7	1.943661972
ZNF6091	3.11E-28	0.2608953	0.609	0.477	7.49E-24	1.3	ZNF609	1.27672956
GSTP11	3.18E-28	0.3414794	0.107	0.051	7.66E-24	1.3	GSTP1	2.098039216
SCAPER1	3.21E-28	0.247453	0.244	0.151	7.75E-24	1.3	SCAPER	1.61589404
CHKA	3.42E-28	0.2600044	0.176	0.099	8.26E-24	1.3	CHKA	1.777777778
PDE7A	3.52E-28	0.294207	0.339	0.229	8.49E-24	1.3	PDE7A	1.480349345
RP4-765H13.11	3.96E-28	0.1141126	0.049	0.016	9.54E-24	1.3	RP4-765H1	3.0625

PSMB2	4.82E-28	0.2145222	0.195	0.114	1.16E-23	1.3	PSMB2	1.710526316
CMTM72	5.45E-28	0.2357223	0.241	0.149	1.31E-23	1.3	CMTM7	1.617449664
TRPC11	5.77E-28	0.1671025	0.067	0.025	1.39E-23	1.3	TRPC1	2.68
GLTP1	5.85E-28	0.2223846	0.207	0.123	1.41E-23	1.3	GLTP	1.682926829
S100A7	6.30E-28	0.3327208	0.042	0.012	1.52E-23	1.3	S100A7	3.5
IL27RA1	7.05E-28	0.1129959	0.058	0.02	1.70E-23	1.3	IL27RA	2.9
EIF4A22	7.13E-28	0.2445063	0.788	0.686	1.72E-23	1.3	EIF4A2	1.148688047
TUBB1	8.23E-28	0.2756681	0.449	0.324	1.98E-23	1.3	TUBB	1.385802469
KCNN41	9.60E-28	0.1185918	0.042	0.012	2.32E-23	1.3	KCNN4	3.5
PSMB41	9.66E-28	0.2332705	0.185	0.106	2.33E-23	1.3	PSMB4	1.745283019
BCL11A	1.03E-27	0.1348176	0.072	0.028	2.48E-23	1.3	BCL11A	2.571428571
ALOX51	1.24E-27	0.2309887	0.179	0.102	2.99E-23	1.3	ALOX5	1.754901961
PXDN1	1.34E-27	0.2008795	0.157	0.086	3.24E-23	1.3	PXDN	1.825581395
CXCL5	1.73E-27	0.1102201	0.029	0.006	4.18E-23	1.3	CXCL5	4.833333333
DRG11	1.82E-27	0.2154897	0.264	0.167	4.40E-23	1.3	DRG1	1.580838323
B3GALT52	1.84E-27	0.1428847	0.063	0.023	4.44E-23	1.3	B3GALT5	2.739130435
LRIG3	2.20E-27	0.1450441	0.065	0.025	5.31E-23	1.3	LRIG3	2.6
RPL292	2.29E-27	0.6843686	0.329	0.226	5.52E-23	1.3	RPL29	1.455752212
PDXK	2.71E-27	0.2656262	0.182	0.105	6.54E-23	1.3	PDXK	1.733333333
DPYD-AS1	2.79E-27	0.2272911	0.134	0.069	6.73E-23	1.3	DPYD-AS1	1.942028986
CHD91	3.00E-27	0.2365342	0.524	0.388	7.23E-23	1.3	CHD9	1.350515464
RPS262	3.62E-27	0.1915012	0.186	0.108	8.73E-23	1.3	RPS26	1.722222222
NARS1	3.62E-27	0.2477102	0.358	0.245	8.73E-23	1.3	NARS	1.46122449
RFX7	3.65E-27	0.2422819	0.19	0.111	8.80E-23	1.3	RFX7	1.711711712
TEFM	3.82E-27	0.1650226	0.08	0.034	9.21E-23	1.3	TEFM	2.352941176
ZNF901	4.07E-27	0.2929184	0.137	0.073	9.82E-23	1.3	ZNF90	1.876712329
CASP4	4.29E-27	0.2345328	0.339	0.228	1.03E-22	1.3	CASP4	1.486842105
IRF21	4.55E-27	0.2484441	0.326	0.221	1.10E-22	1.3	IRF2	1.475113122
CAPZA11	5.13E-27	0.2440976	0.535	0.406	1.24E-22	1.3	CAPZA1	1.31773399
PPIL31	5.19E-27	0.1540733	0.141	0.074	1.25E-22	1.3	PPIL3	1.905405405
AC073283.4	5.54E-27	0.1495122	0.124	0.063	1.34E-22	1.3	AC073283.4	1.968253968
LRSAM1	5.70E-27	0.210567	0.155	0.085	1.37E-22	1.3	LRSAM1	1.823529412
LYPLAL1	5.73E-27	0.1686917	0.101	0.047	1.38E-22	1.3	LYPLAL1	2.14893617
ATP5C11	6.72E-27	0.2158125	0.392	0.272	1.62E-22	1.3	ATP5C1	1.441176471
RP11-290O12.2	6.82E-27	0.2748439	0.118	0.059	1.64E-22	1.3	RP11-290O	2
SYCP3	7.34E-27	0.1353714	0.049	0.016	1.77E-22	1.3	SYCP3	3.0625
PGD	7.76E-27	0.1714038	0.1	0.046	1.87E-22	1.3	PGD	2.173913043
MITD1	8.06E-27	0.1934357	0.168	0.094	1.94E-22	1.3	MITD1	1.787234043
CXADR1	9.96E-27	0.2736068	0.264	0.17	2.40E-22	1.3	CXADR	1.552941176
MLH3	1.23E-26	0.1960358	0.128	0.066	2.97E-22	1.3	MLH3	1.939393939
NSF	1.35E-26	0.2758181	0.229	0.142	3.24E-22	1.3	NSF	1.612676056
TLR1	1.76E-26	0.1247846	0.068	0.027	4.25E-22	1.3	TLR1	2.518518519
HUWE11	2.39E-26	0.2731152	0.374	0.261	5.77E-22	1.3	HUWE1	1.432950192
SLC1A51	2.61E-26	0.1339302	0.072	0.029	6.29E-22	1.3	SLC1A5	2.482758621
TLDC1	2.79E-26	0.172743	0.16	0.088	6.72E-22	1.3	TLDC1	1.818181818
CTA-293F17.12	3.01E-26	0.1446838	0.127	0.065	7.26E-22	1.3	CTA-293F1	1.953846154
TTC9C	3.49E-26	0.1980111	0.191	0.112	8.42E-22	1.3	TTC9C	1.705357143
EHF1	3.71E-26	0.1967636	0.607	0.458	8.94E-22	1.3	EHF	1.325327511
LINC00998	3.84E-26	0.183338	0.204	0.121	9.27E-22	1.3	LINC00998	1.685950413
EIF2S12	4.84E-26	0.2202678	0.287	0.189	1.17E-21	1.3	EIF2S1	1.518518519
CSF1	4.91E-26	0.2191313	0.107	0.052	1.18E-21	1.3	CSF1	2.057692308
RPL92	5.28E-26	0.2792575	0.67	0.557	1.27E-21	1.3	RPL9	1.202872531

SET2	5.52E-26	0.2314765	0.524	0.396	1.33E-21	1.3	SET	1.323232323
CRYBG32	6.11E-26	0.1496607	0.091	0.041	1.47E-21	1.3	CRYBG3	2.219512195
EIF3D2	6.18E-26	0.2396578	0.435	0.315	1.49E-21	1.3	EIF3D	1.380952381
SLC6A9	6.91E-26	0.1076406	0.046	0.014	1.67E-21	1.3	SLC6A9	3.285714286
CX3CL1	7.14E-26	0.337906	0.326	0.221	1.72E-21	1.3	CX3CL1	1.475113122
ATP11A1	7.16E-26	0.2145243	0.108	0.053	1.73E-21	1.3	ATP11A	2.037735849
TTC7B1	9.00E-26	0.113623	0.057	0.021	2.17E-21	1.3	TTC7B	2.714285714
ZDHHC131	9.72E-26	0.1894072	0.136	0.072	2.34E-21	1.3	ZDHHC13	1.888888889
IK	9.99E-26	0.2292557	0.27	0.177	2.41E-21	1.3	IK	1.525423729
TAGLN1	1.07E-25	0.3269249	0.037	0.011	2.57E-21	1.3	TAGLN	3.363636364
FAAH2	1.20E-25	0.2714835	0.294	0.195	2.88E-21	1.3	FAAH2	1.507692308
MINA	1.22E-25	0.1286783	0.085	0.038	2.94E-21	1.3	MINA	2.236842105
TPI11	1.46E-25	0.2523912	0.467	0.347	3.52E-21	1.3	TPI1	1.345821326
LSS	1.74E-25	0.1539259	0.081	0.035	4.19E-21	1.3	LSS	2.314285714
GPHN	1.96E-25	0.2438833	0.333	0.227	4.73E-21	1.3	GPHN	1.466960352
TNFRSF82	2.21E-25	0.2623916	0.153	0.085	5.32E-21	1.3	TNFRSF8	1.8
PLSCR4	2.51E-25	0.1576373	0.098	0.046	6.05E-21	1.3	PLSCR4	2.130434783
SMARCA11	2.94E-25	0.1947485	0.177	0.103	7.10E-21	1.3	SMARCA1	1.718446602
NSD1	3.29E-25	0.1977686	0.198	0.118	7.93E-21	1.3	NSD1	1.677966102
ZNF37A	3.53E-25	0.1908583	0.117	0.059	8.51E-21	1.3	ZNF37A	1.983050847
HIST1H4E1	3.73E-25	0.1757807	0.14	0.075	9.00E-21	1.3	HIST1H4E	1.866666667
SOX5	3.91E-25	0.2025644	0.079	0.035	9.43E-21	1.3	SOX5	2.257142857
PUM32	4.57E-25	0.1744736	0.254	0.161	1.10E-20	1.3	PUM3	1.577639752
HNRNPH3	4.75E-25	0.1997539	0.42	0.302	1.15E-20	1.3	HNRNPH3	1.390728477
C4BPB	5.64E-25	0.1237182	0.051	0.018	1.36E-20	1.3	C4BPB	2.833333333
RUNX2	6.93E-25	0.1259527	0.055	0.02	1.67E-20	1.3	RUNX2	2.75
SHROOM41	6.95E-25	0.1292467	0.051	0.018	1.68E-20	1.3	SHROOM4	2.833333333
FAM171A1	7.30E-25	0.1836927	0.087	0.039	1.76E-20	1.3	FAM171A1	2.230769231
UBD1	7.90E-25	0.3229452	0.148	0.083	1.90E-20	1.3	UBD	1.78313253
GLS1	9.83E-25	0.2448561	0.286	0.189	2.37E-20	1.3	GLS	1.513227513
EIF2A2	1.02E-24	0.1737092	0.423	0.297	2.47E-20	1.3	EIF2A	1.424242424
RIN31	1.20E-24	0.1173584	0.054	0.019	2.90E-20	1.3	RIN3	2.842105263
SRSF51	1.34E-24	0.222334	0.408	0.292	3.22E-20	1.3	SRSF5	1.397260274
PML1	1.47E-24	0.169587	0.137	0.074	3.53E-20	1.3	PML	1.851351351
C21orf91	1.54E-24	0.1481044	0.081	0.036	3.72E-20	1.3	C21orf91	2.25
STAC2	1.58E-24	0.1510488	0.055	0.021	3.81E-20	1.3	STAC2	2.619047619
SSB	1.72E-24	0.1823069	0.221	0.136	4.14E-20	1.3	SSB	1.625
MTERF4	1.73E-24	0.1476732	0.109	0.054	4.18E-20	1.3	MTERF4	2.018518519
BMPR1A	1.96E-24	0.2567237	0.346	0.241	4.74E-20	1.3	BMPR1A	1.435684647
LARP7	2.00E-24	0.184687	0.231	0.145	4.81E-20	1.3	LARP7	1.593103448
PTCHD1-AS1	2.16E-24	0.2674932	0.143	0.079	5.22E-20	1.3	PTCHD1-AS	1.810126582
DLD	2.58E-24	0.2252524	0.179	0.106	6.22E-20	1.3	DLD	1.688679245
USP441	2.64E-24	0.1126277	0.043	0.014	6.37E-20	1.3	USP44	3.071428571
EPRS	3.55E-24	0.1841139	0.215	0.132	8.56E-20	1.3	EPRS	1.628787879
RHPN21	4.19E-24	0.2278659	0.332	0.229	1.01E-19	1.3	RHPN2	1.449781659
LRRK11	4.28E-24	0.1384785	0.104	0.051	1.03E-19	1.3	LRRK1	2.039215686
MNAT11	4.69E-24	0.2303454	0.373	0.264	1.13E-19	1.3	MNAT1	1.412878788
ADH51	4.72E-24	0.1441196	0.143	0.078	1.14E-19	1.3	ADH5	1.833333333
GAB1	5.33E-24	0.348982	0.486	0.374	1.29E-19	1.3	GAB1	1.299465241
PTK71	6.01E-24	0.1718339	0.119	0.062	1.45E-19	1.3	PTK7	1.919354839
GXYLT2	6.26E-24	0.1745146	0.095	0.045	1.51E-19	1.3	GXYLT2	2.111111111
MIR646HG	7.16E-24	0.1786998	0.1	0.049	1.73E-19	1.3	MIR646HG	2.040816327

STAMBP	9.05E-24	0.2110492	0.241	0.155	2.18E-19	1.3	STAMBP	1.55483871
TRNAU1AP1	9.56E-24	0.2094963	0.322	0.221	2.31E-19	1.3	TRNAU1AP	1.457013575
RPS27L	9.91E-24	0.1900883	0.633	0.503	2.39E-19	1.3	RPS27L	1.258449304
RPS112	1.02E-23	0.3355349	0.797	0.767	2.45E-19	1.3	RPS11	1.039113429
POMP1	1.02E-23	0.3028688	0.713	0.622	2.45E-19	1.3	POMP	1.146302251
LMO4	1.14E-23	0.1429734	0.139	0.076	2.75E-19	1.3	LMO4	1.828947368
GPAT32	1.17E-23	0.1636106	0.09	0.042	2.81E-19	1.3	GPAT3	2.142857143
HOTAIRM12	1.34E-23	0.1401382	0.071	0.03	3.24E-19	1.3	HOTAIRM1	2.366666667
TALDO11	1.54E-23	0.198147	0.123	0.065	3.70E-19	1.3	TALDO1	1.892307692
COX4I11	1.56E-23	0.2506808	0.818	0.769	3.75E-19	1.3	COX4I1	1.063719116
IDH3A	1.84E-23	0.181562	0.111	0.057	4.45E-19	1.3	IDH3A	1.947368421
FBXL4	1.87E-23	0.1802796	0.12	0.063	4.51E-19	1.3	FBXL4	1.904761905
ARL11	2.05E-23	0.2219514	0.343	0.239	4.94E-19	1.3	ARL1	1.435146444
GPRC5B1	2.19E-23	0.1168302	0.069	0.029	5.28E-19	1.3	GPRC5B	2.379310345
GNB2L11	2.34E-23	0.3027961	0.743	0.664	5.63E-19	1.3	GNB2L1	1.118975904
DOCK51	2.85E-23	0.2762051	0.292	0.199	6.88E-19	1.3	DOCK5	1.467336683
MRPS18B1	2.88E-23	0.1595072	0.183	0.109	6.95E-19	1.3	MRPS18B	1.678899083
ITGB4	3.69E-23	0.1850742	0.077	0.035	8.90E-19	1.3	ITGB4	2.2
FAM129A2	3.76E-23	0.1705536	0.374	0.261	9.07E-19	1.3	FAM129A	1.432950192
SLC25A25	4.26E-23	0.3512428	0.186	0.115	1.03E-18	1.3	SLC25A25	1.617391304
HNRNPA32	4.64E-23	0.2206686	0.357	0.252	1.12E-18	1.3	HNRNPA3	1.416666667
SLC1A3	5.37E-23	0.1176593	0.062	0.025	1.30E-18	1.3	SLC1A3	2.48
LITAF1	5.49E-23	0.2457016	0.548	0.425	1.32E-18	1.3	LITAF	1.289411765
KCNQ1	5.75E-23	0.1902726	0.111	0.057	1.39E-18	1.3	KCNQ1	1.947368421
HDAC71	6.65E-23	0.202602	0.158	0.091	1.60E-18	1.3	HDAC7	1.736263736
RP11-123O10.4	6.79E-23	0.2272752	0.299	0.203	1.64E-18	1.3	RP11-123O	1.472906404
ERGIC2	6.92E-23	0.1899863	0.252	0.164	1.67E-18	1.3	ERGIC2	1.536585366
RHBDF21	7.97E-23	0.1942154	0.076	0.034	1.92E-18	1.3	RHBDF2	2.235294118
MT2A	8.14E-23	1.119828	0.105	0.055	1.96E-18	1.3	MT2A	1.909090909
MFSD14C1	9.45E-23	0.2351062	0.246	0.161	2.28E-18	1.3	MFSD14C	1.527950311
TMEM150C2	1.09E-22	0.1813358	0.292	0.197	2.64E-18	1.3	TMEM150(	1.482233503
SDCCAG8	1.25E-22	0.2190228	0.221	0.141	3.02E-18	1.3	SDCCAG8	1.567375887
MBOAT2	1.33E-22	0.1801333	0.147	0.083	3.21E-18	1.3	MBOAT2	1.771084337
LAMC21	1.36E-22	0.2562959	0.354	0.254	3.29E-18	1.3	LAMC2	1.393700787
SYPL11	1.37E-22	0.216699	0.478	0.359	3.29E-18	1.3	SYPL1	1.331476323
RNF170	1.37E-22	0.1357029	0.077	0.035	3.30E-18	1.3	RNF170	2.2
PRELID3B1	1.38E-22	0.1774147	0.332	0.229	3.33E-18	1.3	PRELID3B	1.449781659
PHKB	1.60E-22	0.2215868	0.246	0.16	3.86E-18	1.3	PHKB	1.5375
PYGB	1.68E-22	0.17224	0.072	0.031	4.05E-18	1.3	PYGB	2.322580645
BPTF	1.92E-22	0.2030809	0.444	0.324	4.62E-18	1.3	BPTF	1.37037037
CLCA42	1.97E-22	0.1129389	0.042	0.014	4.76E-18	1.3	CLCA4	3
LSM51	2.01E-22	0.1853377	0.454	0.334	4.85E-18	1.3	LSM5	1.359281437
GTF2B1	2.05E-22	0.1799474	0.249	0.162	4.93E-18	1.3	GTF2B	1.537037037
NCAM11	2.22E-22	0.1385458	0.049	0.018	5.35E-18	1.3	NCAM1	2.722222222
AMOTL12	2.79E-22	0.1449146	0.087	0.041	6.73E-18	1.3	AMOTL1	2.12195122
PPID	3.25E-22	0.1323006	0.112	0.059	7.85E-18	1.3	PPID	1.898305085
PHF14	3.76E-22	0.1932325	0.191	0.118	9.08E-18	1.3	PHF14	1.618644068
KALRN	4.11E-22	0.1813205	0.14	0.079	9.90E-18	1.3	KALRN	1.772151899
ELOVL6	4.22E-22	0.1284612	0.13	0.071	1.02E-17	1.3	ELOVL6	1.830985915
CATSPER2	4.42E-22	0.1562246	0.103	0.053	1.07E-17	1.3	CATSPER2	1.943396226
SAMD12	5.52E-22	0.2331359	0.341	0.24	1.33E-17	1.3	SAMD12	1.420833333
LAMB32	5.96E-22	0.1519801	0.332	0.229	1.44E-17	1.3	LAMB3	1.449781659

NDUFA41	7.10E-22	0.1805608	0.833	0.739	1.71E-17	1.3	NDUFA4	1.127198917
PSMC5	7.16E-22	0.1347043	0.156	0.09	1.73E-17	1.3	PSMC5	1.733333333
TUBA1A1	7.41E-22	0.1230583	0.349	0.244	1.79E-17	1.3	TUBA1A	1.430327869
HLA-B2	7.44E-22	0.2589223	0.647	0.53	1.79E-17	1.3	HLA-B	1.220754717
TNFAIP2	7.55E-22	0.271637	0.36	0.258	1.82E-17	1.3	TNFAIP2	1.395348837
INPP5B	7.56E-22	0.1304591	0.078	0.036	1.82E-17	1.3	INPP5B	2.166666667
HSPG21	7.61E-22	0.1945278	0.135	0.076	1.83E-17	1.3	HSPG2	1.776315789
LYRM11	7.88E-22	0.1458087	0.15	0.086	1.90E-17	1.3	LYRM1	1.744186047
DCTN61	8.35E-22	0.1873726	0.375	0.267	2.01E-17	1.3	DCTN6	1.404494382
HSD17B12	8.61E-22	0.2354813	0.292	0.2	2.08E-17	1.3	HSD17B12	1.46
KIAA1551	8.68E-22	0.1525379	0.192	0.118	2.09E-17	1.3	KIAA1551	1.627118644
FOXK22	9.14E-22	0.2140843	0.307	0.212	2.20E-17	1.3	FOXK2	1.448113208
HOMER2	9.39E-22	0.2454288	0.244	0.161	2.26E-17	1.3	HOMER2	1.51552795
C4orf19	9.48E-22	0.203968	0.215	0.137	2.28E-17	1.3	C4orf19	1.569343066
EML42	1.26E-21	0.2119793	0.24	0.157	3.04E-17	1.3	EML4	1.52866242
VDR	1.28E-21	0.1945593	0.135	0.076	3.09E-17	1.3	VDR	1.776315789
NMT1	1.33E-21	0.2004752	0.267	0.177	3.20E-17	1.3	NMT1	1.508474576
RHBDL2	1.52E-21	0.1379052	0.069	0.03	3.66E-17	1.3	RHBDL2	2.3
GBE12	1.62E-21	0.1903945	0.462	0.347	3.90E-17	1.3	GBE1	1.331412104
TAF71	1.64E-21	0.1653147	0.177	0.107	3.96E-17	1.3	TAF7	1.654205607
AP1B1	1.64E-21	0.1658576	0.056	0.023	3.96E-17	1.3	AP1B1	2.434782609
PSMA71	1.73E-21	0.1647541	0.235	0.153	4.16E-17	1.3	PSMA7	1.535947712
SNRPD3	1.73E-21	0.1995134	0.423	0.313	4.18E-17	1.3	SNRPD3	1.3514377
PDP11	1.82E-21	0.1446486	0.104	0.053	4.40E-17	1.3	PDP1	1.962264151
TNFRSF1B1	2.18E-21	0.1342766	0.072	0.032	5.25E-17	1.3	TNFRSF1B	2.25
TTC91	2.27E-21	0.2236839	0.241	0.159	5.47E-17	1.3	TTC9	1.51572327
DFFA1	2.43E-21	0.1331301	0.084	0.04	5.86E-17	1.3	DFFA	2.1
TPRKB2	2.67E-21	0.1556832	0.234	0.15	6.43E-17	1.3	TPRKB	1.56
MRPL33	2.77E-21	0.2016407	0.53	0.409	6.68E-17	1.3	MRPL33	1.295843521
TFB1M	2.83E-21	0.1421508	0.098	0.05	6.81E-17	1.3	TFB1M	1.96
UBAP21	2.97E-21	0.2056978	0.228	0.148	7.15E-17	1.3	UBAP2	1.540540541
SUPT4H1	3.11E-21	0.1795045	0.358	0.254	7.49E-17	1.3	SUPT4H1	1.409448819
WWC1	3.14E-21	0.2498836	0.523	0.407	7.57E-17	1.3	WWC1	1.285012285
TLR5	3.16E-21	0.1159076	0.06	0.025	7.63E-17	1.3	TLR5	2.4
FOCAD	3.27E-21	0.2138477	0.238	0.156	7.89E-17	1.3	FOCAD	1.525641026
TMEM1592	3.47E-21	0.1591713	0.415	0.299	8.38E-17	1.3	TMEM159	1.387959866
C9orf3	3.72E-21	0.215273	0.207	0.13	8.98E-17	1.3	C9orf3	1.592307692
RPL222	3.90E-21	0.1292618	0.757	0.639	9.40E-17	1.3	RPL22	1.184663537
LSM6	3.96E-21	0.1020907	0.06	0.025	9.54E-17	1.3	LSM6	2.4
GTF2H5	4.32E-21	0.1696575	0.3	0.206	1.04E-16	1.3	GTF2H5	1.45631068
AFAP12	4.33E-21	0.1788261	0.14	0.081	1.04E-16	1.3	AFAP1	1.728395062
TCAF21	4.54E-21	0.1906092	0.156	0.093	1.09E-16	1.3	TCAF2	1.677419355
ESRRA	5.07E-21	0.138119	0.1	0.051	1.22E-16	1.3	ESRRA	1.960784314
LLPH	5.36E-21	0.1497958	0.191	0.118	1.29E-16	1.3	LLPH	1.618644068
CNTN4	5.37E-21	0.2355791	0.203	0.129	1.30E-16	1.3	CNTN4	1.573643411
MARCO	5.43E-21	0.1325833	0.088	0.043	1.31E-16	1.3	MARCO	2.046511628
NHSL21	5.56E-21	0.1841417	0.373	0.269	1.34E-16	1.3	NHSL2	1.3866171
PTBP22	5.79E-21	0.1917423	0.446	0.332	1.40E-16	1.3	PTBP2	1.343373494
CIAPIN1	5.84E-21	0.1470124	0.122	0.067	1.41E-16	1.3	CIAPIN1	1.820895522
MPDZ2	6.00E-21	0.1928601	0.207	0.131	1.45E-16	1.3	MPDZ	1.580152672
GATA61	6.49E-21	0.1476751	0.076	0.035	1.56E-16	1.3	GATA6	2.171428571
NR2F2	6.88E-21	0.1522123	0.134	0.076	1.66E-16	1.3	NR2F2	1.763157895

SH3PXD2A1	7.07E-21	0.1820458	0.155	0.092	1.70E-16	1.3	SH3PXD2A	1.684782609
EPS151	7.21E-21	0.1987149	0.284	0.195	1.74E-16	1.3	EPS15	1.456410256
ACACA	8.15E-21	0.1820888	0.234	0.153	1.97E-16	1.3	ACACA	1.529411765
PAICS2	8.18E-21	0.1532687	0.225	0.145	1.97E-16	1.3	PAICS	1.551724138
FBL2	8.59E-21	0.1737979	0.2	0.127	2.07E-16	1.3	FBL	1.57480315
CD742	9.04E-21	0.1124463	0.141	0.081	2.18E-16	1.3	CD74	1.740740741
IGBP11	9.97E-21	0.2029215	0.498	0.384	2.40E-16	1.3	IGBP1	1.296875
DPF2	1.16E-20	0.1402627	0.136	0.077	2.80E-16	1.3	DPF2	1.766233766
MORF4L12	1.24E-20	0.2007013	0.721	0.62	3.00E-16	1.3	MORF4L1	1.162903226
ANTXR1	1.53E-20	0.1339036	0.07	0.031	3.70E-16	1.3	ANTXR1	2.258064516
MRPL50	1.54E-20	0.1312753	0.199	0.124	3.70E-16	1.3	MRPL50	1.60483871
XPO4	1.81E-20	0.1800682	0.173	0.106	4.37E-16	1.3	XPO4	1.632075472
DAP3	2.13E-20	0.170141	0.408	0.299	5.15E-16	1.3	DAP3	1.364548495
VGLL41	2.14E-20	0.2472795	0.304	0.214	5.15E-16	1.3	VGLL4	1.420560748
SNRNP35	2.26E-20	0.1490583	0.125	0.07	5.44E-16	1.3	SNRNP35	1.785714286
SHTN1	2.38E-20	0.1743234	0.195	0.122	5.74E-16	1.3	SHTN1	1.598360656
TNFRSF11A2	2.82E-20	0.1442928	0.113	0.061	6.79E-16	1.3	TNFRSF11A	1.852459016
FAXDC21	2.85E-20	0.1182878	0.055	0.022	6.87E-16	1.3	FAXDC2	2.5
ZNHIT3	3.01E-20	0.1843326	0.258	0.173	7.26E-16	1.3	ZNHIT3	1.49132948
MALL1	3.15E-20	0.1025824	0.031	0.009	7.59E-16	1.3	MALL	3.444444444
RGS61	3.29E-20	0.2195054	0.18	0.112	7.94E-16	1.3	RGS6	1.607142857
CLIP41	3.54E-20	0.1748766	0.279	0.191	8.54E-16	1.3	CLIP4	1.460732984
DEK1	3.56E-20	0.1490001	0.275	0.185	8.59E-16	1.3	DEK	1.486486486
HRSP12	4.13E-20	0.1159158	0.121	0.067	9.97E-16	1.3	HRSP12	1.805970149
PFDN52	4.16E-20	0.1469257	0.888	0.841	1.00E-15	1.3	PFDN5	1.05588585
ST132	4.27E-20	0.1727251	0.381	0.275	1.03E-15	1.3	ST13	1.385454545
HSPD12	4.50E-20	0.1400804	0.345	0.243	1.08E-15	1.3	HSPD1	1.419753086
NNT1	4.62E-20	0.1934938	0.107	0.057	1.11E-15	1.3	NNT	1.877192982
BACH11	4.71E-20	0.2167814	0.4	0.298	1.14E-15	1.3	BACH1	1.342281879
DISC1	4.93E-20	0.1802383	0.134	0.076	1.19E-15	1.3	DISC1	1.763157895
FLOT2	5.02E-20	0.112339	0.071	0.033	1.21E-15	1.3	FLOT2	2.151515152
EML51	5.05E-20	0.1772708	0.112	0.061	1.22E-15	1.3	EML5	1.836065574
SOAT11	5.21E-20	0.1222078	0.072	0.033	1.26E-15	1.3	SOAT1	2.181818182
STRBP	5.51E-20	0.2221197	0.223	0.148	1.33E-15	1.3	STRBP	1.506756757
KIAA0319L	6.23E-20	0.2383093	0.22	0.146	1.50E-15	1.3	KIAA0319L	1.506849315
CNTN3	6.32E-20	0.1383936	0.048	0.018	1.52E-15	1.3	CNTN3	2.666666667
NSMAF	6.60E-20	0.16385	0.122	0.068	1.59E-15	1.3	NSMAF	1.794117647
ZDHHC92	6.65E-20	0.1690493	0.144	0.085	1.60E-15	1.3	ZDHHC9	1.694117647
MRPL221	7.87E-20	0.2011837	0.296	0.207	1.90E-15	1.3	MRPL22	1.429951691
NFYB	7.98E-20	0.1371909	0.1	0.053	1.92E-15	1.3	NFYB	1.886792453
ATL2	8.00E-20	0.2301474	0.309	0.217	1.93E-15	1.3	ATL2	1.423963134
LOX2	9.77E-20	0.1092297	0.058	0.024	2.35E-15	1.3	LOX	2.416666667
HIBCH	9.83E-20	0.1433028	0.171	0.105	2.37E-15	1.3	HIBCH	1.628571429
MRPL27	1.16E-19	0.1461105	0.263	0.176	2.79E-15	1.3	MRPL27	1.494318182
C14orf1191	1.23E-19	0.1658047	0.28	0.191	2.97E-15	1.3	C14orf119	1.465968586
RP11-20I20.2	1.24E-19	0.110104	0.055	0.023	3.00E-15	1.3	RP11-20I2(	2.391304348
NSUN4	1.34E-19	0.1504078	0.111	0.061	3.24E-15	1.3	NSUN4	1.819672131
ACTR6	1.37E-19	0.1448014	0.23	0.151	3.29E-15	1.3	ACTR6	1.523178808
MPZL12	1.38E-19	0.2157328	0.476	0.363	3.33E-15	1.3	MPZL1	1.311294766
LYRM2	1.41E-19	0.1320279	0.156	0.093	3.39E-15	1.3	LYRM2	1.677419355
COMMD10	1.43E-19	0.2244151	0.281	0.194	3.46E-15	1.3	COMMD10	1.448453608
RP11-382A20.5	1.47E-19	0.1415897	0.096	0.049	3.54E-15	1.3	RP11-382A	1.959183673

MAGI2	1.55E-19	0.2297761	0.104	0.056	3.73E-15	1.3	MAGI2	1.857142857
SRFBP12	1.60E-19	0.1501784	0.218	0.141	3.85E-15	1.3	SRFBP1	1.546099291
RP11-711K1.8	1.60E-19	0.1836447	0.091	0.046	3.85E-15	1.3	RP11-711K	1.97826087
RTP42	1.61E-19	0.1042529	0.081	0.039	3.88E-15	1.3	RTP4	2.076923077
RPL151	1.62E-19	0.3527286	0.806	0.775	3.90E-15	1.3	RPL15	1.04
DOPEY1	1.76E-19	0.1675737	0.135	0.078	4.25E-15	1.3	DOPEY1	1.730769231
SGCE	1.99E-19	0.1049446	0.079	0.038	4.79E-15	1.3	SGCE	2.078947368
SH3YL12	2.05E-19	0.1790424	0.283	0.195	4.95E-15	1.3	SH3YL1	1.451282051
CDK181	2.30E-19	0.1155496	0.043	0.016	5.55E-15	1.3	CDK18	2.6875
TJP22	2.66E-19	0.1882092	0.417	0.31	6.42E-15	1.3	TJP2	1.34516129
NAP1L12	2.86E-19	0.1936694	0.458	0.346	6.90E-15	1.3	NAP1L1	1.323699422
TIMM101	3.06E-19	0.1382844	0.275	0.187	7.39E-15	1.3	TIMM10	1.470588235
HAPLN31	3.11E-19	0.1151155	0.043	0.016	7.50E-15	1.3	HAPLN3	2.6875
DST	3.37E-19	0.1868634	0.669	0.556	8.13E-15	1.3	DST	1.20323741
NDUFAF42	3.52E-19	0.11011	0.124	0.07	8.49E-15	1.3	NDUFAF4	1.771428571
ATXN7	3.63E-19	0.1761778	0.166	0.103	8.75E-15	1.3	ATXN7	1.611650485
MRRF1	3.87E-19	0.1779959	0.196	0.126	9.33E-15	1.3	MRRF	1.555555556
NHSL12	3.93E-19	0.4149879	0.245	0.169	9.47E-15	1.3	NHSL1	1.449704142
RIF12	4.23E-19	0.2172996	0.306	0.216	1.02E-14	1.3	RIF1	1.416666667
ENOX2	4.56E-19	0.1600868	0.083	0.041	1.10E-14	1.3	ENOX2	2.024390244
PALLD1	5.09E-19	0.1721559	0.51	0.389	1.23E-14	1.3	PALLD	1.311053985
RAB30-AS1	5.16E-19	0.1320948	0.127	0.072	1.24E-14	1.3	RAB30-AS1	1.763888889
ATG7	5.21E-19	0.1943005	0.291	0.204	1.26E-14	1.3	ATG7	1.426470588
UBE2L31	5.41E-19	0.2101965	0.537	0.427	1.30E-14	1.3	UBE2L3	1.257611241
PTK2B1	5.66E-19	0.1544422	0.069	0.032	1.37E-14	1.3	PTK2B	2.15625
SOCS51	5.68E-19	0.2072656	0.194	0.126	1.37E-14	1.3	SOCS5	1.53968254
ADGRL21	7.37E-19	0.2018543	0.128	0.074	1.78E-14	1.3	ADGRL2	1.72972973
HMGN3	7.85E-19	0.1261891	0.181	0.113	1.89E-14	1.3	HMGN3	1.601769912
RRAGD2	8.97E-19	0.1406239	0.092	0.048	2.16E-14	1.3	RRAGD	1.916666667
SF3A3	9.08E-19	0.1357713	0.104	0.057	2.19E-14	1.3	SF3A3	1.824561404
LINC01122	9.53E-19	0.1477906	0.068	0.031	2.30E-14	1.3	LINC01122	2.193548387
CYP27A1	1.08E-18	0.1539152	0.103	0.056	2.60E-14	1.3	CYP27A1	1.839285714
UTP11L1	1.19E-18	0.1177438	0.115	0.064	2.88E-14	1.3	UTP11L	1.796875
PABPC12	1.24E-18	0.1783205	0.805	0.721	2.99E-14	1.3	PABPC1	1.116504854
TPTEP11	1.25E-18	0.1729397	0.225	0.149	3.02E-14	1.3	TPTEP1	1.510067114
FAM76A	1.30E-18	0.1116254	0.065	0.03	3.12E-14	1.3	FAM76A	2.166666667
NCK2	1.39E-18	0.1936459	0.202	0.131	3.35E-14	1.3	NCK2	1.541984733
KPNA21	1.45E-18	0.1487745	0.204	0.132	3.49E-14	1.3	KPNA2	1.545454545
ZNF429	1.48E-18	0.1387036	0.108	0.06	3.57E-14	1.3	ZNF429	1.8
VPS45	1.56E-18	0.1264208	0.127	0.073	3.77E-14	1.3	VPS45	1.739726027
PHAX1	1.62E-18	0.1252595	0.143	0.085	3.90E-14	1.3	PHAX	1.682352941
AC097724.32	1.66E-18	0.1133045	0.08	0.04	4.01E-14	1.3	AC097724.3	2
RPL33	1.67E-18	0.353368	0.386	0.295	4.04E-14	1.3	RPL3	1.308474576
LRAT1	1.76E-18	0.104271	0.051	0.021	4.24E-14	1.3	LRAT	2.428571429
PIK3C2G1	1.76E-18	0.1644285	0.108	0.059	4.25E-14	1.3	PIK3C2G	1.830508475
ATF7IP	1.85E-18	0.1833197	0.247	0.167	4.45E-14	1.3	ATF7IP	1.479041916
SGPL1	2.38E-18	0.1285033	0.166	0.103	5.73E-14	1.3	SGPL1	1.611650485
GRHL2	2.51E-18	0.2212262	0.373	0.273	6.04E-14	1.3	GRHL2	1.366300366
TRIAP1	2.78E-18	0.1334694	0.127	0.074	6.71E-14	1.3	TRIAP1	1.716216216
TMA16	2.89E-18	0.1034754	0.068	0.032	6.96E-14	1.3	TMA16	2.125
RABGAP1L	3.03E-18	0.2343391	0.306	0.22	7.30E-14	1.3	RABGAP1L	1.390909091
NUDCD11	3.12E-18	0.1305159	0.13	0.075	7.52E-14	1.3	NUDCD1	1.733333333

SNRPG1	3.45E-18	0.2077052	0.613	0.507	8.33E-14	1.3	SNRPG	1.209072978
DDX1	3.55E-18	0.163596	0.148	0.09	8.56E-14	1.3	DDX1	1.644444444
SACS1	4.33E-18	0.1335603	0.1	0.054	1.04E-13	1.3	SACS	1.851851852
HLA-E1	4.56E-18	0.2203558	0.142	0.086	1.10E-13	1.3	HLA-E	1.651162791
ENTPD1-AS1	4.78E-18	0.1535142	0.091	0.048	1.15E-13	1.3	ENTPD1-AS	1.895833333
PHF21A	4.79E-18	0.2121842	0.317	0.23	1.16E-13	1.3	PHF21A	1.37826087
GNPTAB	5.06E-18	0.1750402	0.147	0.089	1.22E-13	1.3	GNPTAB	1.651685393
MAP22	5.09E-18	0.1370714	0.15	0.091	1.23E-13	1.3	MAP2	1.648351648
CLEC2D	5.17E-18	0.1531952	0.133	0.079	1.25E-13	1.3	CLEC2D	1.683544304
VBP1	6.09E-18	0.1571529	0.206	0.135	1.47E-13	1.3	VBP1	1.525925926
SF3B62	6.40E-18	0.1815565	0.708	0.596	1.54E-13	1.3	SF3B6	1.187919463
ANXA7	6.90E-18	0.1732444	0.378	0.28	1.66E-13	1.3	ANXA7	1.35
TRMT10B	7.07E-18	0.1396637	0.136	0.081	1.70E-13	1.3	TRMT10B	1.679012346
GGACT1	7.57E-18	0.1460155	0.204	0.134	1.82E-13	1.3	GGACT	1.52238806
RAD50	8.70E-18	0.1769346	0.135	0.081	2.10E-13	1.3	RAD50	1.666666667
PROL12	8.77E-18	0.1316106	0.028	0.008	2.11E-13	1.3	PROL1	3.5
RPL36AL2	8.84E-18	0.1515961	0.914	0.883	2.13E-13	1.3	RPL36AL	1.035107588
SLC5A6	9.28E-18	0.1521793	0.144	0.087	2.24E-13	1.3	SLC5A6	1.655172414
SNRPD22	1.02E-17	0.154407	0.758	0.662	2.45E-13	1.3	SNRPD2	1.145015106
FAM189A21	1.06E-17	0.159772	0.112	0.063	2.57E-13	1.3	FAM189A2	1.777777778
CHORDC11	1.08E-17	0.1141759	0.127	0.074	2.60E-13	1.3	CHORDC1	1.716216216
ERCC6L2	1.08E-17	0.1497336	0.114	0.065	2.60E-13	1.3	ERCC6L2	1.753846154
FMNL21	1.09E-17	0.2004711	0.451	0.341	2.63E-13	1.3	FMNL2	1.322580645
CH17-189H20.1	1.14E-17	0.1707981	0.144	0.087	2.75E-13	1.3	CH17-189H	1.655172414
CBR41	1.14E-17	0.1827156	0.314	0.226	2.75E-13	1.3	CBR4	1.389380531
ARHGEF10L2	1.16E-17	0.2487995	0.234	0.16	2.79E-13	1.3	ARHGEF10I	1.4625
TRMT111	1.20E-17	0.1602231	0.153	0.094	2.89E-13	1.3	TRMT11	1.627659574
SP140L	1.26E-17	0.1303483	0.063	0.029	3.03E-13	1.3	SP140L	2.172413793
ARHGAP26-AS11	1.29E-17	0.1873118	0.21	0.14	3.12E-13	1.3	ARHGAP26	1.5
TBX192	1.32E-17	0.1418399	0.099	0.054	3.18E-13	1.3	TBX19	1.833333333
FAF2	1.34E-17	0.1522067	0.228	0.153	3.23E-13	1.3	FAF2	1.490196078
Sep-71	1.47E-17	0.1961027	0.468	0.363	3.54E-13	1.3	Sep-07	1.289256198
RPL181	1.51E-17	0.387055	0.139	0.086	3.64E-13	1.3	RPL18	1.61627907
NTRK21	1.71E-17	0.1358764	0.156	0.096	4.11E-13	1.3	NTRK2	1.625
RUSC22	1.75E-17	0.1128434	0.113	0.064	4.23E-13	1.3	RUSC2	1.765625
XPO52	1.80E-17	0.1435251	0.148	0.091	4.35E-13	1.3	XPO5	1.626373626
EEF2	1.83E-17	0.3910738	0.495	0.401	4.41E-13	1.3	EEF2	1.234413965
RPL36	1.89E-17	0.4838898	0.268	0.192	4.55E-13	1.3	RPL36	1.395833333
RPL392	1.95E-17	0.2259615	0.932	0.894	4.70E-13	1.3	RPL39	1.042505593
EIF2S21	1.96E-17	0.1827596	0.343	0.251	4.73E-13	1.3	EIF2S2	1.366533865
FRK1	2.03E-17	0.1614688	0.201	0.132	4.89E-13	1.3	FRK	1.522727273
DEGS11	2.13E-17	0.1269156	0.104	0.058	5.14E-13	1.3	DEGS1	1.793103448
ATXN3	2.20E-17	0.1743455	0.207	0.137	5.31E-13	1.3	ATXN3	1.510948905
TXNRD11	2.29E-17	0.55198	0.466	0.367	5.53E-13	1.3	TXNRD1	1.269754768
HDDC21	2.32E-17	0.1334194	0.106	0.059	5.59E-13	1.3	HDDC2	1.796610169
MPHOSPH8	2.39E-17	0.166578	0.214	0.142	5.75E-13	1.3	MPHOSPH8	1.507042254
WDR45B2	2.45E-17	0.2100223	0.29	0.208	5.90E-13	1.3	WDR45B	1.394230769
ABCB10	2.68E-17	0.1062378	0.064	0.03	6.46E-13	1.3	ABCB10	2.133333333
DCAF131	2.73E-17	0.1415256	0.125	0.073	6.58E-13	1.3	DCAF13	1.712328767
STEAP1	2.74E-17	0.1103637	0.091	0.049	6.62E-13	1.3	STEAP1	1.857142857
ITGBL12	2.79E-17	0.1290159	0.053	0.023	6.72E-13	1.3	ITGBL1	2.304347826
SERGEF	2.79E-17	0.11065	0.067	0.032	6.73E-13	1.3	SERGEF	2.09375

CCDC91	3.00E-17	0.1613751	0.443	0.337	7.23E-13	1.3	CCDC91	1.314540059
IDH1	3.21E-17	0.1090929	0.13	0.076	7.74E-13	1.3	IDH1	1.710526316
DIRC2	3.26E-17	0.1591933	0.097	0.053	7.86E-13	1.3	DIRC2	1.830188679
TMPRSS31	3.56E-17	0.1170909	0.082	0.042	8.58E-13	1.3	TMPRSS3	1.952380952
NIN	3.86E-17	0.1622152	0.156	0.098	9.30E-13	1.3	NIN	1.591836735
LRP5	4.02E-17	0.1098305	0.055	0.025	9.69E-13	1.3	LRP5	2.2
RP11-111E14.12	4.08E-17	0.1119571	0.059	0.026	9.85E-13	1.3	RP11-111E	2.269230769
NOL3	4.50E-17	0.1198839	0.078	0.04	1.08E-12	1.3	NOL3	1.95
IPO52	4.50E-17	0.1423317	0.162	0.101	1.09E-12	1.3	IPO5	1.603960396
EIF4B2	4.66E-17	0.1584594	0.477	0.37	1.12E-12	1.3	EIF4B	1.289189189
FAM49B1	4.81E-17	0.1607023	0.279	0.195	1.16E-12	1.3	FAM49B	1.430769231
ZFAND12	4.94E-17	0.1237912	0.255	0.175	1.19E-12	1.3	ZFAND1	1.457142857
LPL	5.27E-17	0.1006472	0.055	0.025	1.27E-12	1.3	LPL	2.2
GRIP1	5.73E-17	0.2316205	0.257	0.182	1.38E-12	1.3	GRIP1	1.412087912
CREG11	5.94E-17	0.1445195	0.089	0.048	1.43E-12	1.3	CREG1	1.854166667
RP11-577H5.5	6.67E-17	0.1024667	0.093	0.05	1.61E-12	1.3	RP11-577H	1.86
ZNF721	7.18E-17	0.1906384	0.26	0.182	1.73E-12	1.3	ZNF721	1.428571429
COL27A1	7.42E-17	0.1504303	0.097	0.053	1.79E-12	1.3	COL27A1	1.830188679
SLC35E3	8.57E-17	0.1237847	0.125	0.074	2.07E-12	1.3	SLC35E3	1.689189189
TASP1	8.69E-17	0.1911733	0.189	0.124	2.10E-12	1.3	TASP1	1.524193548
CD552	8.79E-17	0.1397915	0.378	0.279	2.12E-12	1.3	CD55	1.35483871
CCDC90B	9.50E-17	0.1221176	0.135	0.082	2.29E-12	1.3	CCDC90B	1.646341463
MSN1	9.59E-17	0.1841516	0.524	0.408	2.31E-12	1.3	MSN	1.284313725
CCT6A1	9.87E-17	0.1180728	0.156	0.097	2.38E-12	1.3	CCT6A	1.608247423
ZC3H81	1.03E-16	0.1225938	0.114	0.066	2.49E-12	1.3	ZC3H8	1.727272727
BANF11	1.05E-16	0.1101183	0.252	0.173	2.54E-12	1.3	BANF1	1.456647399
SCAI	1.10E-16	0.1397347	0.106	0.06	2.66E-12	1.3	SCAI	1.766666667
TFAP2C1	1.14E-16	0.141631	0.13	0.078	2.74E-12	1.3	TFAP2C	1.666666667
BIRC6-AS21	1.22E-16	0.1845262	0.277	0.197	2.94E-12	1.3	BIRC6-AS2	1.406091371
RP11-541P9.31	1.25E-16	0.1438533	0.087	0.046	3.00E-12	1.3	RP11-541P!	1.891304348
CCNH	1.27E-16	0.1205974	0.131	0.079	3.07E-12	1.3	CCNH	1.658227848
ALG14	1.30E-16	0.1448443	0.127	0.075	3.12E-12	1.3	ALG14	1.693333333
IQGAP2	1.38E-16	0.1611942	0.082	0.043	3.33E-12	1.3	IQGAP2	1.906976744
FAM222B1	1.39E-16	0.1783441	0.281	0.201	3.36E-12	1.3	FAM222B	1.39800995
DAP1	1.41E-16	0.1287132	0.099	0.055	3.41E-12	1.3	DAP	1.8
NAA20	1.47E-16	0.1516789	0.172	0.111	3.54E-12	1.3	NAA20	1.54954955
PTPN4	1.47E-16	0.1656756	0.134	0.081	3.54E-12	1.3	PTPN4	1.654320988
EIF3J-AS1	1.48E-16	0.1182119	0.085	0.045	3.58E-12	1.3	EIF3J-AS1	1.888888889
BCAP29	1.50E-16	0.1743467	0.213	0.145	3.62E-12	1.3	BCAP29	1.468965517
LDLRAD4	1.56E-16	0.2265156	0.237	0.163	3.75E-12	1.3	LDLRAD4	1.45398773
MAGI11	1.60E-16	0.1686704	0.846	0.742	3.86E-12	1.3	MAGI1	1.140161725
NRBF2	1.67E-16	0.1204052	0.089	0.048	4.03E-12	1.3	NRBF2	1.854166667
CWC152	1.68E-16	0.1797324	0.367	0.275	4.05E-12	1.3	CWC15	1.334545455
AC090498.12	1.72E-16	0.1367148	0.399	0.296	4.16E-12	1.3	AC090498.	1.347972973
GPM6B2	1.84E-16	0.1576209	0.182	0.118	4.43E-12	1.3	GPM6B	1.542372881
RPS161	1.85E-16	0.4694306	0.388	0.301	4.47E-12	1.3	RPS16	1.289036545
CARD162	1.86E-16	0.113583	0.08	0.041	4.48E-12	1.3	CARD16	1.951219512
TMEM167A	1.87E-16	0.177576	0.345	0.256	4.51E-12	1.3	TMEM167/	1.34765625
FBXO282	1.94E-16	0.1953315	0.215	0.147	4.68E-12	1.3	FBXO28	1.462585034
C9orf85	1.97E-16	0.1112932	0.108	0.062	4.75E-12	1.3	C9orf85	1.741935484
HADHA1	2.19E-16	0.1496479	0.368	0.273	5.28E-12	1.3	HADHA	1.347985348
LARS1	2.21E-16	0.1510845	0.267	0.189	5.32E-12	1.3	LARS	1.412698413

XYLT11	2.29E-16	0.1036592	0.13	0.078	5.53E-12	1.3	XYLT1	1.666666667
ELK32	2.40E-16	0.1378938	0.171	0.11	5.79E-12	1.3	ELK3	1.554545455
CRBN2	2.64E-16	0.1565353	0.205	0.138	6.38E-12	1.3	CRBN	1.485507246
NCOA4	2.96E-16	0.1412515	0.207	0.138	7.14E-12	1.3	NCOA4	1.5
UBA3	3.21E-16	0.1653295	0.312	0.227	7.73E-12	1.3	UBA3	1.374449339
LINC013891	3.25E-16	0.1294892	0.042	0.017	7.83E-12	1.3	LINC01389	2.470588235
ITCH1	3.32E-16	0.1764829	0.42	0.316	8.01E-12	1.3	ITCH	1.329113924
COL19A1	3.59E-16	0.1383491	0.081	0.043	8.65E-12	1.3	COL19A1	1.88372093
RALGDS	3.63E-16	0.156774	0.088	0.047	8.76E-12	1.3	RALGDS	1.872340426
MRPS9	3.83E-16	0.1025406	0.094	0.052	9.23E-12	1.3	MRPS9	1.807692308
KCTD92	3.95E-16	0.2140177	0.306	0.225	9.53E-12	1.3	KCTD9	1.36
PTGFRN	4.09E-16	0.1419518	0.15	0.094	9.85E-12	1.3	PTGFRN	1.595744681
MED281	4.33E-16	0.1245634	0.127	0.077	1.04E-11	1.3	MED28	1.649350649
RBMX2	4.37E-16	0.1150566	0.142	0.088	1.05E-11	1.3	RBMX2	1.613636364
RP11-138A9.2	4.39E-16	0.1615968	0.169	0.109	1.06E-11	1.3	RP11-138A	1.550458716
KIAA1143	4.61E-16	0.1244623	0.15	0.094	1.11E-11	1.3	KIAA1143	1.595744681
UST	4.93E-16	0.295602	0.248	0.178	1.19E-11	1.3	UST	1.393258427
PHYKPL	4.98E-16	0.139495	0.075	0.038	1.20E-11	1.3	PHYKPL	1.973684211
PWWP2A	5.08E-16	0.1404223	0.096	0.054	1.23E-11	1.3	PWWP2A	1.777777778
DDX501	5.45E-16	0.1475356	0.13	0.08	1.31E-11	1.3	DDX50	1.625
UGGT2	5.49E-16	0.1794405	0.208	0.14	1.32E-11	1.3	UGGT2	1.485714286
MAPK1IP1L1	5.74E-16	0.1536909	0.234	0.161	1.38E-11	1.3	MAPK1IP1I	1.453416149
PAIP11	5.93E-16	0.1998411	0.218	0.15	1.43E-11	1.3	PAIP1	1.453333333
TMCC1	6.05E-16	0.1777025	0.333	0.244	1.46E-11	1.3	TMCC1	1.364754098
MBNL1	6.15E-16	0.2153557	0.504	0.397	1.48E-11	1.3	MBNL1	1.269521411
TAF4B	6.43E-16	0.1067993	0.089	0.048	1.55E-11	1.3	TAF4B	1.854166667
EIF2AK2	6.67E-16	0.1579753	0.137	0.085	1.61E-11	1.3	EIF2AK2	1.611764706
NRG3	7.35E-16	0.1466026	0.11	0.064	1.77E-11	1.3	NRG3	1.71875
ATG5	7.48E-16	0.1319847	0.163	0.104	1.80E-11	1.3	ATG5	1.567307692
ORMDL2	7.73E-16	0.1217806	0.226	0.154	1.86E-11	1.3	ORMDL2	1.467532468
NIFK2	8.03E-16	0.1143054	0.116	0.068	1.94E-11	1.3	NIFK	1.705882353
HMGB1	8.20E-16	0.1915911	0.63	0.535	1.98E-11	1.3	HMGB1	1.177570093
SPDYE51	1.12E-15	0.1237455	0.099	0.056	2.69E-11	1.3	SPDYE5	1.767857143
KRR12	1.18E-15	0.146034	0.214	0.146	2.84E-11	1.3	KRR1	1.465753425
SLC25A6	1.19E-15	0.2491332	0.166	0.109	2.88E-11	1.3	SLC25A6	1.52293578
COMT	1.28E-15	0.1246435	0.075	0.039	3.08E-11	1.3	COMT	1.923076923
CEP63	1.40E-15	0.149795	0.115	0.068	3.38E-11	1.3	CEP63	1.691176471
RPS15	1.46E-15	0.3722126	0.202	0.137	3.51E-11	1.3	RPS15	1.474452555
PRDX61	1.65E-15	0.1746016	0.238	0.166	3.99E-11	1.3	PRDX6	1.43373494
PXK	1.76E-15	0.1231751	0.087	0.047	4.25E-11	1.3	PXK	1.85106383
DANT2	1.85E-15	0.1446547	0.1	0.057	4.47E-11	1.3	DANT2	1.754385965
AK61	1.88E-15	0.125047	0.23	0.159	4.54E-11	1.3	AK6	1.446540881
EZH1	1.96E-15	0.1366885	0.129	0.079	4.72E-11	1.3	EZH1	1.632911392
FANCC	1.99E-15	0.1666861	0.185	0.123	4.80E-11	1.3	FANCC	1.504065041
MDM4	2.07E-15	0.1628183	0.325	0.24	4.99E-11	1.3	MDM4	1.354166667
C6orf89	2.09E-15	0.1268441	0.102	0.059	5.03E-11	1.3	C6orf89	1.728813559
SLC25A431	2.15E-15	0.1076646	0.068	0.034	5.18E-11	1.3	SLC25A43	2
MLLT10	2.35E-15	0.1594355	0.22	0.151	5.68E-11	1.3	MLLT10	1.456953642
AC023590.1	2.39E-15	0.1015273	0.055	0.026	5.77E-11	1.3	AC023590.	2.115384615
ZNF426	2.45E-15	0.1191704	0.085	0.046	5.91E-11	1.3	ZNF426	1.847826087
PILRB	2.51E-15	0.1632166	0.081	0.043	6.05E-11	1.3	PILRB	1.88372093
KIN	2.51E-15	0.1176267	0.103	0.059	6.06E-11	1.3	KIN	1.745762712

ADPRM2	2.62E-15	0.1349531	0.103	0.059	6.31E-11	1.3	ADPRM	1.745762712
PHACTR42	2.75E-15	0.1480682	0.417	0.321	6.62E-11	1.3	PHACTR4	1.299065421
CDC5L2	2.76E-15	0.1387608	0.268	0.191	6.65E-11	1.3	CDC5L	1.403141361
SMOC1	2.80E-15	0.103247	0.06	0.029	6.76E-11	1.3	SMOC1	2.068965517
RPS281	2.88E-15	0.3763047	0.129	0.081	6.94E-11	1.3	RPS28	1.592592593
AUTS2	2.89E-15	0.2021902	0.628	0.517	6.98E-11	1.3	AUTS2	1.214700193
CCNB1IP12	2.90E-15	0.1354515	0.331	0.244	6.99E-11	1.3	CCNB1IP1	1.356557377
C14orf1661	2.95E-15	0.1068348	0.296	0.211	7.10E-11	1.3	C14orf166	1.402843602
CUL5	3.00E-15	0.1235184	0.174	0.114	7.23E-11	1.3	CUL5	1.526315789
MAT2A1	3.00E-15	0.1326788	0.087	0.047	7.23E-11	1.3	MAT2A	1.85106383
GREB1L2	3.21E-15	0.1298468	0.152	0.097	7.74E-11	1.3	GREB1L	1.567010309
CEBPD	3.37E-15	0.2057236	0.252	0.181	8.13E-11	1.3	CEBPD	1.392265193
CPAMD82	3.62E-15	0.1448077	0.104	0.06	8.73E-11	1.3	CPAMD8	1.733333333
OLMALINC	4.11E-15	0.1044452	0.077	0.04	9.91E-11	1.3	OLMALINC	1.925
GANC	4.16E-15	0.1344556	0.129	0.079	1.00E-10	1.3	GANC	1.632911392
RPL281	4.30E-15	0.6299473	0.148	0.098	1.04E-10	1.3	RPL28	1.510204082
KDM1B	4.44E-15	0.1111503	0.09	0.05	1.07E-10	1.3	KDM1B	1.8
IRX2	4.50E-15	0.1098359	0.102	0.059	1.09E-10	1.3	IRX2	1.728813559
PLA2G4A	4.53E-15	0.1091263	0.048	0.021	1.09E-10	1.3	PLA2G4A	2.285714286
PARVA	4.60E-15	0.1645291	0.176	0.117	1.11E-10	1.3	PARVA	1.504273504
LAMTOR51	4.88E-15	0.1534341	0.681	0.576	1.18E-10	1.3	LAMTOR5	1.182291667
ICA1	5.67E-15	0.1595195	0.351	0.26	1.37E-10	1.3	ICA1	1.35
CDC42SE2	6.01E-15	0.1943704	0.276	0.201	1.45E-10	1.3	CDC42SE2	1.373134328
FOS	6.37E-15	0.2883391	0.297	0.224	1.53E-10	1.3	FOS	1.325892857
TLK1	6.67E-15	0.1704116	0.263	0.187	1.61E-10	1.3	TLK1	1.406417112
STX71	6.77E-15	0.1486648	0.176	0.117	1.63E-10	1.3	STX7	1.504273504
GTF2F22	7.02E-15	0.1373217	0.153	0.099	1.69E-10	1.3	GTF2F2	1.545454545
VPS54	7.33E-15	0.1481475	0.254	0.181	1.77E-10	1.3	VPS54	1.403314917
CTTNBP2NL2	7.76E-15	0.1187046	0.283	0.202	1.87E-10	1.3	CTTNBP2N	1.400990099
ANP32E1	8.17E-15	0.1122113	0.128	0.079	1.97E-10	1.3	ANP32E	1.620253165
ZNF397	8.20E-15	0.1385293	0.141	0.089	1.98E-10	1.3	ZNF397	1.584269663
IL321	8.30E-15	0.1535447	0.091	0.051	2.00E-10	1.3	IL32	1.784313725
CCDC71L	8.70E-15	0.1015713	0.054	0.025	2.10E-10	1.3	CCDC71L	2.16
TPST1	9.50E-15	0.2197474	0.158	0.104	2.29E-10	1.3	TPST1	1.519230769
PRKDC	9.91E-15	0.135132	0.16	0.103	2.39E-10	1.3	PRKDC	1.553398058
DDX52	9.94E-15	0.1463278	0.151	0.098	2.40E-10	1.3	DDX52	1.540816327
INSIG1	1.01E-14	0.1203779	0.164	0.107	2.45E-10	1.3	INSIG1	1.53271028
TCP12	1.08E-14	0.1331537	0.231	0.161	2.60E-10	1.3	TCP1	1.434782609
ALDOA1	1.12E-14	0.2460757	0.153	0.1	2.70E-10	1.3	ALDOA	1.53
PLCG2	1.14E-14	0.1113383	0.086	0.048	2.75E-10	1.3	PLCG2	1.791666667
DLG2	1.14E-14	0.2391645	0.224	0.158	2.75E-10	1.3	DLG2	1.417721519
LINC00623	1.15E-14	0.1045685	0.055	0.026	2.76E-10	1.3	LINC00623	2.115384615
MGA	1.20E-14	0.1409423	0.107	0.063	2.90E-10	1.3	MGA	1.698412698
KRCC1	1.22E-14	0.1090138	0.093	0.052	2.95E-10	1.3	KRCC1	1.788461538
GSTA12	1.33E-14	0.1725039	0.072	0.038	3.20E-10	1.3	GSTA1	1.894736842
YEATS21	1.39E-14	0.1614643	0.168	0.111	3.34E-10	1.3	YEATS2	1.513513514
NUS12	1.40E-14	0.1026758	0.125	0.076	3.37E-10	1.3	NUS1	1.644736842
C9orf721	1.68E-14	0.1148768	0.093	0.053	4.05E-10	1.3	C9orf72	1.754716981
HNRNPA2B1	1.90E-14	0.2013211	0.502	0.403	4.58E-10	1.3	HNRNPA2E	1.245657568
PLEKHM3	1.90E-14	0.1255191	0.058	0.028	4.59E-10	1.3	PLEKHM3	2.071428571
KLHL20	1.93E-14	0.1435061	0.098	0.057	4.65E-10	1.3	KLHL20	1.719298246
PPP1CC	1.95E-14	0.1154338	0.142	0.09	4.70E-10	1.3	PPP1CC	1.577777778

CDC42SE1	2.07E-14	0.1623489	0.207	0.143	4.99E-10	1.3	CDC42SE1	1.447552448
GOSR1	2.09E-14	0.134377	0.208	0.143	5.03E-10	1.3	GOSR1	1.454545455
MRPL20	2.16E-14	0.1252098	0.096	0.055	5.21E-10	1.3	MRPL20	1.745454545
ROR1	2.23E-14	0.1718353	0.048	0.022	5.37E-10	1.3	ROR1	2.181818182
FOXN21	2.24E-14	0.1430913	0.117	0.072	5.39E-10	1.3	FOXN2	1.625
NDUFA12	2.46E-14	0.1260364	0.281	0.204	5.94E-10	1.3	NDUFA12	1.37745098
DNAJC8	2.53E-14	0.1462505	0.29	0.213	6.10E-10	1.3	DNAJC8	1.361502347
GPX1	2.54E-14	0.1287403	0.064	0.032	6.11E-10	1.3	GPX1	2
IKZF2	2.70E-14	0.1138443	0.073	0.039	6.52E-10	1.3	IKZF2	1.871794872
RFC12	2.77E-14	0.1379225	0.266	0.19	6.68E-10	1.3	RFC1	1.4
THAP6	2.78E-14	0.1087947	0.091	0.051	6.70E-10	1.3	THAP6	1.784313725
DSG22	2.96E-14	0.1661331	0.338	0.256	7.13E-10	1.3	DSG2	1.3203125
RBMX	3.11E-14	0.1888641	0.259	0.187	7.49E-10	1.3	RBMX	1.385026738
DLEU1	3.17E-14	0.1367446	0.184	0.124	7.64E-10	1.3	DLEU1	1.483870968
PSMD7	3.64E-14	0.1365707	0.209	0.144	8.78E-10	1.3	PSMD7	1.451388889
SCFD2	3.65E-14	0.1135351	0.067	0.035	8.81E-10	1.3	SCFD2	1.914285714
PCNXL4	3.88E-14	0.1425165	0.155	0.101	9.35E-10	1.3	PCNXL4	1.534653465
LIMK2	3.90E-14	0.1390502	0.149	0.096	9.40E-10	1.3	LIMK2	1.552083333
TYW3	4.00E-14	0.1096729	0.096	0.056	9.64E-10	1.3	TYW3	1.714285714
N4BP2L2	4.00E-14	0.129352	0.825	0.714	9.65E-10	1.3	N4BP2L2	1.155462185
MFAP11	4.16E-14	0.1216825	0.208	0.143	1.00E-09	1.3	MFAP1	1.454545455
MLXIP1	4.49E-14	0.1442247	0.19	0.13	1.08E-09	1.3	MLXIP	1.461538462
RP11-304M2.31	4.82E-14	0.1156638	0.062	0.031	1.16E-09	1.3	RP11-304M	2
HOOK2	5.08E-14	0.1566488	0.214	0.15	1.23E-09	1.3	HOOK2	1.426666667
OSMR-AS1	5.15E-14	0.1543203	0.233	0.165	1.24E-09	1.3	OSMR-AS1	1.412121212
ZBED5	5.23E-14	0.1334356	0.096	0.056	1.26E-09	1.3	ZBED5	1.714285714
ZNF544	5.53E-14	0.1440364	0.129	0.081	1.33E-09	1.3	ZNF544	1.592592593
CATSPERB	5.55E-14	0.1499272	0.27	0.197	1.34E-09	1.3	CATSPERB	1.370558376
CCNC1	5.57E-14	0.1450248	0.285	0.209	1.34E-09	1.3	CCNC	1.363636364
NUPL2	5.61E-14	0.1017976	0.09	0.051	1.35E-09	1.3	NUPL2	1.764705882
PSMB5	5.68E-14	0.1397674	0.315	0.233	1.37E-09	1.3	PSMB5	1.35193133
MRPS31	5.80E-14	0.1307823	0.145	0.094	1.40E-09	1.3	MRPS31	1.542553191
CD821	5.85E-14	0.1364813	0.085	0.048	1.41E-09	1.3	CD82	1.770833333
PARK7	6.03E-14	0.1627986	0.545	0.446	1.45E-09	1.3	PARK7	1.221973094
NHP21	6.16E-14	0.117495	0.042	0.018	1.48E-09	1.3	NHP2	2.333333333
ITPKB1	6.96E-14	0.1033396	0.074	0.04	1.68E-09	1.3	ITPKB	1.85
MLX	7.09E-14	0.1108227	0.089	0.051	1.71E-09	1.3	MLX	1.745098039
STARD13	8.48E-14	0.1039551	0.27	0.195	2.04E-09	1.3	STARD13	1.384615385
AKAP1	8.72E-14	0.101941	0.052	0.025	2.10E-09	1.3	AKAP1	2.08
PIN4	9.24E-14	0.1343955	0.342	0.259	2.23E-09	1.3	PIN4	1.32046332
THSD4-AS11	9.40E-14	0.1387524	0.553	0.447	2.27E-09	1.3	THSD4-AS1	1.237136465
KDM3B	9.62E-14	0.1714886	0.216	0.151	2.32E-09	1.3	KDM3B	1.430463576
PRKCI2	9.96E-14	0.1217136	0.244	0.173	2.40E-09	1.3	PRKCI	1.410404624
TGFBR21	1.03E-13	0.1114318	0.133	0.084	2.48E-09	1.3	TGFBR2	1.583333333
LETMD1	1.13E-13	0.1259684	0.098	0.058	2.73E-09	1.3	LETMD1	1.689655172
WDPCP	1.16E-13	0.1531977	0.171	0.115	2.81E-09	1.3	WDPCP	1.486956522
NEDD11	1.17E-13	0.1069164	0.093	0.054	2.82E-09	1.3	NEDD1	1.722222222
RABGAP1	1.18E-13	0.1649069	0.356	0.268	2.84E-09	1.3	RABGAP1	1.328358209
ATXN10	1.18E-13	0.1612123	0.201	0.141	2.85E-09	1.3	ATXN10	1.425531915
PDZD22	1.39E-13	0.1534791	0.555	0.463	3.35E-09	1.3	PDZD2	1.198704104
FRG11	1.41E-13	0.1345928	0.169	0.113	3.39E-09	1.3	FRG1	1.495575221
XRCC5	1.42E-13	0.1594637	0.44	0.346	3.41E-09	1.3	XRCC5	1.271676301

PCID2	1.52E-13	0.1179952	0.137	0.088	3.66E-09	1.3		PCID2	1.556818182
TRAF3IP21	1.55E-13	0.1430809	0.177	0.12	3.74E-09	1.3		TRAF3IP2	1.475
BRCC3	1.61E-13	0.1193535	0.086	0.049	3.88E-09	1.3		BRCC3	1.755102041
PRKD31	1.62E-13	0.1370934	0.161	0.108	3.90E-09	1.3		PRKD3	1.490740741
HIP1	1.62E-13	0.1199519	0.137	0.088	3.91E-09	1.3		HIP1	1.556818182
FAS2	1.79E-13	0.1311216	0.144	0.094	4.33E-09	1.3		FAS	1.531914894
RNF1751	1.85E-13	0.1171135	0.056	0.028	4.47E-09	1.3		RNF175	2
GCC2	2.16E-13	0.1322198	0.387	0.297	5.20E-09	1.3		GCC2	1.303030303
TRAF11	2.16E-13	0.1232073	0.071	0.038	5.21E-09	1.3		TRAF1	1.868421053
CDC42EP11	2.19E-13	0.102631	0.063	0.032	5.29E-09	1.3		CDC42EP1	1.96875
RNF1501	2.24E-13	0.1642603	0.107	0.065	5.40E-09	1.3		RNF150	1.646153846
RPL13A1	2.33E-13	0.3875354	0.549	0.471	5.63E-09	1.3		RPL13A	1.165605096
LRIG1	2.49E-13	0.2213065	0.294	0.222	6.00E-09	1.3		LRIG1	1.324324324
GDI2	2.54E-13	0.1389378	0.257	0.185	6.12E-09	1.3		GDI2	1.389189189
SRSF122	2.60E-13	0.1125456	0.072	0.039	6.26E-09	1.3		SRSF12	1.846153846
MAN1A2	2.60E-13	0.1501168	0.222	0.157	6.26E-09	1.3		MAN1A2	1.414012739
DNTTIP11	2.69E-13	0.1108403	0.079	0.044	6.50E-09	1.3		DNTTIP1	1.795454545
PSMA5	2.98E-13	0.1382257	0.281	0.207	7.18E-09	1.3		PSMA5	1.357487923
PPM1L	3.01E-13	0.1327267	0.114	0.07	7.25E-09	1.3		PPM1L	1.628571429
USP161	3.03E-13	0.1503568	0.209	0.149	7.30E-09	1.3		USP16	1.402684564
ZNHIT6	3.30E-13	0.1463206	0.205	0.144	7.97E-09	1.3		ZNHIT6	1.423611111
RPL7L1	3.57E-13	0.1186117	0.173	0.117	8.62E-09	1.3		RPL7L1	1.478632479
MCFD21	3.58E-13	0.1006623	0.181	0.122	8.64E-09	1.3		MCFD2	1.483606557
TCEB2	3.60E-13	0.1756923	0.069	0.037	8.68E-09	1.3		TCEB2	1.864864865
TECPR2	3.68E-13	0.1055689	0.065	0.034	8.88E-09	1.3		TECPR2	1.911764706
RTTN	3.85E-13	0.1355993	0.107	0.065	9.29E-09	1.3		RTTN	1.646153846
MAGOH2	3.88E-13	0.1427935	0.212	0.149	9.34E-09	1.3		MAGOH	1.422818792
MRPL1	3.93E-13	0.1042795	0.097	0.057	9.47E-09	1.3		MRPL1	1.701754386
MT-ND1	4.15E-13	0.1589725	0.977	0.98	1.00E-08	1.3		MT-ND1	0.996938776
LIMS12	4.20E-13	0.1753699	0.312	0.236	1.01E-08	1.3		LIMS1	1.322033898
DBI1	4.28E-13	0.1054997	0.74	0.644	1.03E-08	1.3		DBI	1.149068323
TMEM63A	4.45E-13	0.1167851	0.094	0.056	1.07E-08	1.3		TMEM63A	1.678571429
CLCN3	4.58E-13	0.1471938	0.226	0.161	1.11E-08	1.3		CLCN3	1.403726708
NIF3L1	4.93E-13	0.1091777	0.091	0.054	1.19E-08	1.3		NIF3L1	1.685185185
ZDHHC17	5.05E-13	0.1451223	0.118	0.074	1.22E-08	1.3		ZDHHC17	1.594594595
NDRG22	5.40E-13	0.1240221	0.246	0.177	1.30E-08	1.3		NDRG2	1.389830508
SREBF2	5.80E-13	0.13269	0.465	0.368	1.40E-08	1.3		SREBF2	1.263586957
CEBPZ1	6.20E-13	0.1342187	0.207	0.146	1.50E-08	1.3		CEBPZ	1.417808219
CNIH1	6.51E-13	0.1327851	0.316	0.236	1.57E-08	1.3		CNIH1	1.338983051
PPP2R5C1	7.96E-13	0.1245953	0.231	0.165	1.92E-08	1.3		PPP2R5C	1.4
RPRD2	7.97E-13	0.1686607	0.203	0.143	1.92E-08	1.3		RPRD2	1.41958042
AC004231.21	8.13E-13	0.1242142	0.064	0.034	1.96E-08	1.3		AC004231.	1.882352941
MRPS36	9.18E-13	0.1085867	0.236	0.169	2.21E-08	1.3		MRPS36	1.396449704
SIPA1L1	9.66E-13	0.1317135	0.471	0.375	2.33E-08	1.3		SIPA1L1	1.256
GBAS	9.66E-13	0.1453904	0.231	0.166	2.33E-08	1.3		GBAS	1.391566265
PDK31	1.06E-12	0.119966	0.295	0.221	2.56E-08	1.3		PDK3	1.334841629
CTDSP2	1.10E-12	0.1105261	0.234	0.168	2.65E-08	1.3		CTDSP2	1.392857143
TRAPPC11	1.14E-12	0.1095704	0.08	0.046	2.75E-08	1.3		TRAPPC11	1.739130435
STAG1	1.16E-12	0.1451216	0.605	0.502	2.79E-08	1.3		STAG1	1.205179283
CAPRIN1	1.24E-12	0.1375883	0.267	0.196	2.99E-08	1.3		CAPRIN1	1.362244898
TMEM45A	1.25E-12	0.1123241	0.186	0.129	3.01E-08	1.3		TMEM45A	1.441860465
ABCE12	1.26E-12	0.1213012	0.134	0.087	3.03E-08	1.3		ABCE1	1.540229885

SHFM1	1.29E-12	0.1445182	0.706	0.618	3.12E-08	1.3	SHFM1	1.142394822
MRPL47	1.58E-12	0.1281345	0.23	0.166	3.80E-08	1.3	MRPL47	1.385542169
EEF1D	1.61E-12	0.2009485	0.148	0.1	3.89E-08	1.3	EEF1D	1.48
COMMD61	1.65E-12	0.11787	0.439	0.346	3.97E-08	1.3	COMMD6	1.268786127
LGALS8	1.71E-12	0.1547121	0.159	0.108	4.13E-08	1.3	LGALS8	1.472222222
PRKRIP1	1.74E-12	0.1492321	0.17	0.117	4.19E-08	1.3	PRKRIP1	1.452991453
CCDC14	1.87E-12	0.142766	0.142	0.094	4.50E-08	1.3	CCDC14	1.510638298
TMEM242	2.11E-12	0.1046034	0.086	0.05	5.09E-08	1.3	TMEM242	1.72
PIK3IP1-AS1	2.16E-12	0.1026811	0.068	0.037	5.22E-08	1.3	PIK3IP1-AS	1.837837838
UBE2E3	2.44E-12	0.1122145	0.23	0.165	5.89E-08	1.3	UBE2E3	1.393939394
CAV21	2.47E-12	0.1104966	0.072	0.04	5.96E-08	1.3	CAV2	1.8
TMEM41A2	2.61E-12	0.138515	0.191	0.134	6.29E-08	1.3	TMEM41A	1.425373134
GLMN	2.74E-12	0.1012175	0.079	0.045	6.61E-08	1.3	GLMN	1.755555556
POLR2F	2.82E-12	0.130181	0.091	0.054	6.81E-08	1.3	POLR2F	1.685185185
PRPF38B1	3.11E-12	0.141937	0.312	0.234	7.49E-08	1.3	PRPF38B	1.333333333
DNAJC51	3.21E-12	0.1276985	0.105	0.065	7.73E-08	1.3	DNAJC5	1.615384615
RPL35	3.31E-12	0.3637602	0.342	0.269	7.99E-08	1.3	RPL35	1.271375465
UBE2N1	3.32E-12	0.1216916	0.267	0.197	8.00E-08	1.3	UBE2N	1.355329949
GTF2IRD2	3.49E-12	0.1170569	0.117	0.074	8.43E-08	1.3	GTF2IRD2	1.581081081
DNAJC71	3.69E-12	0.152495	0.236	0.173	8.91E-08	1.3	DNAJC7	1.36416185
C1orf56	3.79E-12	0.1305971	0.197	0.14	9.15E-08	1.3	C1orf56	1.407142857
CASC4	3.86E-12	0.1541662	0.248	0.182	9.30E-08	1.3	CASC4	1.362637363
RFC3	4.01E-12	0.1077722	0.082	0.048	9.67E-08	1.3	RFC3	1.708333333
MMP241	4.22E-12	0.1163308	0.127	0.082	1.02E-07	1.3	MMP24	1.548780488
CTNNBL1	4.32E-12	0.109089	0.11	0.069	1.04E-07	1.3	CTNNBL1	1.594202899
AGK	4.45E-12	0.103957	0.058	0.03	1.07E-07	1.3	AGK	1.933333333
RRAS21	4.67E-12	0.1386882	0.309	0.232	1.12E-07	1.3	RRAS2	1.331896552
PSMC6	4.85E-12	0.1427938	0.39	0.307	1.17E-07	1.3	PSMC6	1.270358306
HERC2	4.89E-12	0.1372528	0.11	0.069	1.18E-07	1.3	HERC2	1.594202899
SLC25A13	4.92E-12	0.1378519	0.107	0.067	1.19E-07	1.3	SLC25A13	1.597014925
NONO2	5.34E-12	0.1124712	0.394	0.305	1.29E-07	1.3	NONO	1.291803279
EIF3H2	5.37E-12	0.1055364	0.704	0.616	1.30E-07	1.3	EIF3H	1.142857143
PUS10	5.70E-12	0.1014329	0.083	0.049	1.38E-07	1.3	PUS10	1.693877551
CDH31	6.08E-12	0.108492	0.172	0.118	1.47E-07	1.3	CDH3	1.457627119
BBOX1-AS1	6.41E-12	0.1081019	0.031	0.013	1.54E-07	1.3	BBOX1-AS1	2.384615385
SMURF22	6.48E-12	0.1835287	0.362	0.283	1.56E-07	1.3	SMURF2	1.279151943
CFDP1	6.95E-12	0.1207596	0.234	0.17	1.68E-07	1.3	CFDP1	1.376470588
SLC25A5	7.17E-12	0.1637599	0.195	0.139	1.73E-07	1.3	SLC25A5	1.402877698
SDAD1	7.23E-12	0.117717	0.11	0.07	1.74E-07	1.3	SDAD1	1.571428571
CALCOCO22	7.36E-12	0.1210885	0.319	0.244	1.77E-07	1.3	CALCOCO2	1.307377049
PIWIL4	7.40E-12	0.1011321	0.056	0.029	1.78E-07	1.3	PIWIL4	1.931034483
TFDP2	7.52E-12	0.1489244	0.264	0.197	1.81E-07	1.3	TFDP2	1.340101523
DCUN1D11	7.91E-12	0.1165133	0.173	0.12	1.91E-07	1.3	DCUN1D1	1.441666667
FRMD3	8.60E-12	0.1720993	0.125	0.082	2.07E-07	1.3	FRMD3	1.524390244
JKAMP	8.79E-12	0.1255712	0.129	0.085	2.12E-07	1.3	JKAMP	1.517647059
SNX29	9.09E-12	0.1578465	0.14	0.093	2.19E-07	1.3	SNX29	1.505376344
CYB5R3	9.25E-12	0.1184655	0.104	0.065	2.23E-07	1.3	CYB5R3	1.6
SRGAP3	9.38E-12	0.1071628	0.082	0.048	2.26E-07	1.3	SRGAP3	1.708333333
CAMKMT	9.89E-12	0.1662311	0.227	0.167	2.39E-07	1.3	CAMKMT	1.359281437
MAGOHB	1.00E-11	0.1016665	0.086	0.051	2.42E-07	1.3	MAGOHB	1.68627451
NCBP2	1.03E-11	0.1066875	0.143	0.096	2.48E-07	1.3	NCBP2	1.489583333
SFXN1	1.06E-11	0.1082282	0.082	0.048	2.55E-07	1.3	SFXN1	1.708333333

COX7A22	1.06E-11	0.148594	0.809	0.757	2.56E-07	1.3	COX7A2	1.068692206
ANAPC161	1.07E-11	0.1296388	0.313	0.24	2.59E-07	1.3	ANAPC16	1.304166667
LGR41	1.11E-11	0.1319678	0.374	0.295	2.68E-07	1.3	LGR4	1.26779661
VPS351	1.24E-11	0.109954	0.33	0.252	2.99E-07	1.3	VPS35	1.30952381
AK1	1.31E-11	0.1023293	0.031	0.013	3.17E-07	1.3	AK1	2.384615385
ANKUB11	1.33E-11	0.1087991	0.167	0.115	3.20E-07	1.3	ANKUB1	1.452173913
MAVS	1.39E-11	0.1043843	0.081	0.048	3.34E-07	1.3	MAVS	1.6875
CCT71	1.42E-11	0.1196752	0.052	0.027	3.43E-07	1.3	CCT7	1.925925926
UXS11	1.60E-11	0.1327729	0.133	0.088	3.86E-07	1.3	UXS1	1.511363636
RP11-415J8.3	1.72E-11	0.1234969	0.081	0.048	4.16E-07	1.3	RP11-415J8	1.6875
ZNF595	1.99E-11	0.1172105	0.071	0.041	4.81E-07	1.3	ZNF595	1.731707317
TAX1BP11	2.19E-11	0.1577117	0.703	0.615	5.27E-07	1.3	TAX1BP1	1.143089431
SUMO12	2.27E-11	0.1264423	0.577	0.488	5.48E-07	1.3	SUMO1	1.182377049
SMARCAD1	2.39E-11	0.1096859	0.094	0.058	5.76E-07	1.3	SMARCAD1	1.620689655
SNX61	2.53E-11	0.1379029	0.391	0.309	6.11E-07	1.3	SNX6	1.265372168
AL592183.1	2.69E-11	0.1089651	0.101	0.063	6.49E-07	1.3	AL592183.1	1.603174603
IPMK1	2.78E-11	0.1128327	0.131	0.087	6.70E-07	1.3	IPMK	1.505747126
C11orf58	2.82E-11	0.1547169	0.345	0.27	6.81E-07	1.3	C11orf58	1.277777778
LRRC8B1	2.87E-11	0.1181786	0.148	0.101	6.92E-07	1.3	LRRC8B	1.465346535
PIBF1	2.93E-11	0.1675029	0.23	0.17	7.06E-07	1.3	PIBF1	1.352941176
NCOA1	3.01E-11	0.1606087	0.457	0.371	7.27E-07	1.3	NCOA1	1.23180593
RPL37A2	3.03E-11	0.1416353	0.973	0.975	7.31E-07	1.3	RPL37A	0.997948718
ZNF106	3.19E-11	0.1177462	0.148	0.101	7.69E-07	1.3	ZNF106	1.465346535
MICALL11	3.30E-11	0.1009127	0.075	0.044	7.95E-07	1.3	MICALL1	1.704545455
HDAC11	3.30E-11	0.107737	0.144	0.097	7.95E-07	1.3	HDAC1	1.484536082
PSMD1	3.31E-11	0.1340755	0.202	0.145	7.98E-07	1.3	PSMD1	1.393103448
WIPI1	3.31E-11	0.1234096	0.196	0.14	7.99E-07	1.3	WIPI1	1.4
TMTC2	3.40E-11	0.1788379	0.46	0.369	8.20E-07	1.3	TMTC2	1.246612466
ROR21	3.60E-11	0.164634	0.124	0.083	8.67E-07	1.3	ROR2	1.493975904
PRDX5	3.73E-11	0.151255	0.097	0.061	8.99E-07	1.3	PRDX5	1.590163934
RASGEF1A	3.82E-11	0.1124336	0.052	0.027	9.22E-07	1.3	RASGEF1A	1.925925926
PCNXL22	3.97E-11	0.3878781	0.814	0.758	9.58E-07	1.3	PCNXL2	1.073878628
ACSL1	4.18E-11	0.1272246	0.264	0.198	1.01E-06	1.3	ACSL1	1.333333333
TTC14	5.42E-11	0.1158663	0.107	0.069	1.31E-06	1.3	TTC14	1.550724638
VPS36	5.45E-11	0.1293452	0.145	0.099	1.32E-06	1.3	VPS36	1.464646465
IDE	5.61E-11	0.1178418	0.104	0.067	1.35E-06	1.3	IDE	1.552238806
RP11-795H16.32	5.90E-11	0.1838169	0.425	0.354	1.42E-06	1.3	RP11-795H	1.200564972
PCBP21	6.18E-11	0.1469802	0.65	0.562	1.49E-06	1.3	PCBP2	1.15658363
RP11-417F21.11	6.58E-11	0.1237927	0.136	0.092	1.59E-06	1.3	RP11-417F2	1.47826087
H1F0	6.62E-11	0.1357	0.21	0.154	1.60E-06	1.3	H1F0	1.363636364
LYPLA1	7.10E-11	0.1201692	0.15	0.103	1.71E-06	1.3	LYPLA1	1.45631068
MACROD2	7.35E-11	0.1689097	0.303	0.236	1.77E-06	1.3	MACROD2	1.283898305
KIAA1217	7.70E-11	0.1794685	0.874	0.793	1.86E-06	1.3	KIAA1217	1.102143758
RPL8	7.74E-11	0.3649084	0.213	0.158	1.87E-06	1.3	RPL8	1.348101266
NKX3-12	8.23E-11	0.1048686	0.081	0.049	1.98E-06	1.3	NKX3-1	1.653061224
IMMP2L	8.66E-11	0.1892836	0.361	0.287	2.09E-06	1.3	IMMP2L	1.257839721
GHR1	9.03E-11	0.1057562	0.121	0.08	2.18E-06	1.3	GHR	1.5125
ERRFI11	9.31E-11	0.1415697	0.628	0.53	2.24E-06	1.3	ERRFI1	1.18490566
NUDCD3	9.57E-11	0.1376729	0.149	0.103	2.31E-06	1.3	NUDCD3	1.446601942
RBM41	9.87E-11	0.121404	0.108	0.07	2.38E-06	1.3	RBM41	1.542857143
C2orf68	9.99E-11	0.1008707	0.095	0.059	2.41E-06	1.3	C2orf68	1.610169492
CBWD1	1.01E-10	0.1053008	0.182	0.129	2.44E-06	1.3	CBWD1	1.410852713

WARS	1.09E-10	0.1121815	0.101	0.064	2.62E-06	1.3	WARS	1.578125
CD46	1.12E-10	0.2270901	0.476	0.394	2.70E-06	1.3	CD46	1.208121827
LRRC37A3	1.33E-10	0.1150044	0.153	0.107	3.20E-06	1.3	LRRC37A3	1.429906542
ADRBK21	1.39E-10	0.1007936	0.118	0.078	3.36E-06	1.3	ADRBK2	1.512820513
EPN2	1.41E-10	0.1359088	0.158	0.111	3.40E-06	1.3	EPN2	1.423423423
RPL26L1	1.46E-10	0.1064105	0.215	0.157	3.52E-06	1.3	RPL26L1	1.369426752
HEBP21	1.56E-10	0.105642	0.207	0.151	3.77E-06	1.3	HEBP2	1.370860927
FMO21	1.83E-10	0.1158866	0.089	0.055	4.42E-06	1.3	FMO2	1.618181818
PHB2	2.63E-10	0.1018835	0.13	0.087	6.33E-06	1.3	PHB2	1.494252874
USP28	3.00E-10	0.100297	0.062	0.035	7.24E-06	1.3	USP28	1.771428571
CHIC22	3.03E-10	0.1085681	0.21	0.155	7.31E-06	1.3	CHIC2	1.35483871
PPP1R12B	3.20E-10	0.1272059	0.281	0.213	7.73E-06	1.3	PPP1R12B	1.319248826
DIS3L2	3.29E-10	0.1217568	0.135	0.092	7.93E-06	1.3	DIS3L2	1.467391304
SS18	3.44E-10	0.1466755	0.183	0.133	8.30E-06	1.3	SS18	1.37593985
ZNF43	3.47E-10	0.1334133	0.178	0.128	8.37E-06	1.3	ZNF43	1.390625
C12orf57	3.96E-10	0.1211369	0.062	0.035	9.54E-06	1.3	C12orf57	1.771428571
KATNBL1	4.34E-10	0.1137195	0.189	0.138	1.05E-05	1.3	KATNBL1	1.369565217
EIF1B2	4.45E-10	0.1333817	0.282	0.217	1.07E-05	1.3	EIF1B	1.299539171
HS6ST3	4.67E-10	0.1237982	0.062	0.035	1.13E-05	1.3	HS6ST3	1.771428571
SNX3	4.78E-10	0.1169062	0.214	0.159	1.15E-05	1.3	SNX3	1.34591195
PPFIBP12	5.10E-10	0.1139143	0.194	0.141	1.23E-05	1.3	PPFIBP1	1.375886525
RNF213	5.24E-10	0.1372944	0.233	0.175	1.26E-05	1.3	RNF213	1.331428571
RSRC1	5.40E-10	0.1261895	0.354	0.279	1.30E-05	1.3	RSRC1	1.268817204
PHC3	5.40E-10	0.1242258	0.222	0.164	1.30E-05	1.3	PHC3	1.353658537
TNPO11	6.00E-10	0.1082551	0.258	0.196	1.45E-05	1.3	TNPO1	1.316326531
NBR1	6.51E-10	0.1154281	0.225	0.168	1.57E-05	1.3	NBR1	1.339285714
SPNS2	7.27E-10	0.1267962	0.081	0.05	1.75E-05	1.3	SPNS2	1.62
MARK1	7.75E-10	0.1071942	0.095	0.062	1.87E-05	1.3	MARK1	1.532258065
KDM7A2	7.86E-10	0.1275791	0.341	0.267	1.90E-05	1.3	KDM7A	1.277153558
ARL6IP5	8.87E-10	0.109829	0.568	0.48	2.14E-05	1.3	ARL6IP5	1.183333333
IDH2	9.17E-10	0.1171626	0.112	0.075	2.21E-05	1.3	IDH2	1.493333333
RP11-452H21.11	1.04E-09	0.105625	0.101	0.066	2.51E-05	1.3	RP11-452H	1.53030303
RPL27A	1.11E-09	0.3050422	0.493	0.416	2.67E-05	1.3	RPL27A	1.185096154
KCND21	1.34E-09	0.1305928	0.087	0.055	3.23E-05	1.3	KCND2	1.581818182
EAPP	1.34E-09	0.1013802	0.344	0.272	3.24E-05	1.3	EAPP	1.264705882
MSH3	1.47E-09	0.1407347	0.138	0.097	3.55E-05	1.3	MSH3	1.422680412
CADM1	1.49E-09	0.1208609	0.107	0.072	3.59E-05	1.3	CADM1	1.486111111
GPATCH8	1.54E-09	0.1172306	0.306	0.239	3.72E-05	1.3	GPATCH8	1.280334728
GNAQ	1.54E-09	0.1425081	0.375	0.304	3.72E-05	1.3	GNAQ	1.233552632
TCEB11	1.75E-09	0.1092853	0.375	0.3	4.22E-05	1.3	TCEB1	1.25
ZNF98	1.83E-09	0.1044906	0.13	0.09	4.41E-05	1.3	ZNF98	1.444444444
MVP	2.14E-09	0.1064747	0.092	0.059	5.17E-05	1.3	MVP	1.559322034
RFWD2	2.16E-09	0.13856	0.368	0.294	5.21E-05	1.3	RFWD2	1.25170068
NARS2	2.49E-09	0.1058217	0.099	0.065	6.01E-05	1.3	NARS2	1.523076923
SEC22A	3.04E-09	0.1172733	0.16	0.116	7.34E-05	1.3	SEC22A	1.379310345
SLC25A26	3.12E-09	0.1010124	0.119	0.081	7.52E-05	1.3	SLC25A26	1.469135802
KDSR	3.47E-09	0.1015894	0.141	0.099	8.36E-05	1.3	KDSR	1.424242424
LNPEP	3.88E-09	0.1000966	0.125	0.086	9.36E-05	1.3	LNPEP	1.453488372
MYH141	4.33E-09	0.115871	0.207	0.156	0.000104341	1.3	MYH14	1.326923077
STX17	5.66E-09	0.1152094	0.159	0.115	0.000136365	1.3	STX17	1.382608696
TSFM	6.06E-09	0.1020751	0.069	0.042	0.000146123	1.3	TSFM	1.642857143
RP4-533D7.51	6.12E-09	0.1258064	0.042	0.022	0.000147589	1.3	RP4-533D7	1.909090909

RERG	6.15E-09	0.1321188	0.248	0.192	0.000148228	1.3	RERG	1.291666667
AP000487.62	6.37E-09	0.1330614	0.211	0.159	0.000153578	1.3	AP000487.(	1.327044025
PRKAG1	7.04E-09	0.1086812	0.183	0.136	0.000169816	1.3	PRKAG1	1.345588235
RAPGEF11	8.18E-09	0.107088	0.139	0.098	0.000197333	1.3	RAPGEF1	1.418367347
SEL1L2	9.34E-09	0.1235136	0.024	0.01	0.000225263	1.3	SEL1L2	2.4
SH3PXD2B1	1.04E-08	0.1102683	0.162	0.118	0.000249909	1.3	SH3PXD2B	1.372881356
PTAR1	1.05E-08	0.1018947	0.102	0.069	0.000253556	1.3	PTAR1	1.47826087
RPS181	1.06E-08	0.2010071	0.703	0.628	0.000254535	1.3	RPS18	1.119426752
ZNF235	1.06E-08	0.1218448	0.107	0.073	0.000255663	1.3	ZNF235	1.465753425
PITPNA	1.09E-08	0.1255017	0.178	0.132	0.000263118	1.3	PITPNA	1.348484848
WWOX	1.12E-08	0.2424693	0.286	0.231	0.000270164	1.3	WWOX	1.238095238
CCDC174	1.33E-08	0.100054	0.224	0.17	0.000321701	1.3	CCDC174	1.317647059
EDF11	1.44E-08	0.1897595	0.097	0.066	0.00034678	1.3	EDF1	1.46969697
PPP1CB1	1.54E-08	0.1136152	0.427	0.347	0.000371189	1.3	PPP1CB	1.23054755
ATM	1.64E-08	0.1017248	0.095	0.063	0.000396636	1.3	ATM	1.507936508
CSNK1E1	1.66E-08	0.1169147	0.283	0.222	0.000400228	1.3	CSNK1E	1.274774775
RP11-440L14.11	1.73E-08	0.1049476	0.059	0.035	0.000417209	1.3	RP11-440L1	1.685714286
FBXL5	1.92E-08	0.1188921	0.172	0.127	0.000462895	1.3	FBXL5	1.354330709
APTX	1.95E-08	0.1011513	0.166	0.123	0.000470316	1.3	APTX	1.349593496
ATP6V1E1	1.96E-08	0.1275992	0.352	0.287	0.000471577	1.3	ATP6V1E1	1.226480836
SEPT111	2.38E-08	0.112308	0.137	0.098	0.000574226	1.3	Sep-11	1.397959184
IL6R	2.49E-08	0.1279314	0.094	0.063	0.000599792	1.3	IL6R	1.492063492
HIST1H2BB	2.52E-08	0.1682343	0.214	0.166	0.000606467	1.3	HIST1H2BB	1.289156627
CASP7	2.64E-08	0.1111368	0.205	0.156	0.000636857	1.3	CASP7	1.314102564
SCFD1	2.68E-08	0.102063	0.269	0.211	0.000645219	1.3	SCFD1	1.274881517
MFN11	2.77E-08	0.1118472	0.163	0.121	0.000669059	1.3	MFN1	1.347107438
CARHSP1	2.99E-08	0.155728	0.328	0.266	0.000719997	1.3	CARHSP1	1.233082707
GREB11	3.12E-08	0.2279127	0.101	0.069	0.000752521	1.3	GREB1	1.463768116
RSRP1	3.20E-08	0.1186294	0.313	0.25	0.000772234	1.3	RSRP1	1.252
RBM26	3.43E-08	0.148352	0.242	0.189	0.000827873	1.3	RBM26	1.28042328
AGTPBP1	3.73E-08	0.1176545	0.107	0.073	0.000900385	1.3	AGTPBP1	1.465753425
SON1	4.01E-08	0.2003324	0.73	0.674	0.000965976	1.3	SON	1.083086053
UBE3C1	4.19E-08	0.1109755	0.298	0.235	0.001009725	1.3	UBE3C	1.268085106
SLC7A111	4.47E-08	0.1037865	0.075	0.048	0.001078039	1.3	SLC7A11	1.5625
AP3B1	4.50E-08	0.1076519	0.233	0.182	0.00108473	1.3	AP3B1	1.28021978
TPK12	4.79E-08	0.1420718	0.257	0.203	0.001155901	1.3	TPK1	1.266009852
GAB21	5.25E-08	0.1084498	0.248	0.194	0.001266008	1.3	GAB2	1.278350515
SH2B1	5.60E-08	0.1417379	0.03	0.015	0.001349846	1.3	SH2B1	2
TTC371	7.29E-08	0.1045432	0.175	0.131	0.001757789	1.3	TTC37	1.335877863
ACBD6	7.64E-08	0.1325615	0.11	0.077	0.001841576	1.3	ACBD6	1.428571429
ATP6V1D1	7.65E-08	0.1018032	0.334	0.27	0.001844898	1.3	ATP6V1D	1.237037037
CDKAL1	1.14E-07	0.1160616	0.292	0.234	0.002747901	1.3	CDKAL1	1.247863248
CDC371	1.20E-07	0.1096595	0.046	0.027	0.002881783	1.3	CDC37	1.703703704
ZNF608	1.32E-07	0.1000324	0.225	0.175	0.00319276	1.3	ZNF608	1.285714286
AHR	1.70E-07	0.1203847	0.254	0.2	0.004110613	1.3	AHR	1.27
KCTD71	1.78E-07	0.1070471	0.146	0.108	0.004284915	1.3	KCTD7	1.351851852
FAM114A1	1.90E-07	0.1287942	0.2	0.155	0.004591316	1.3	FAM114A1	1.290322581
HP	1.96E-07	0.1068538	0.026	0.012	0.004716354	1.3	HP	2.166666667
NUBPL	2.03E-07	0.1077101	0.079	0.053	0.004901803	1.3	NUBPL	1.490566038
LINC01252	2.11E-07	0.1021213	0.046	0.027	0.005090297	1.3	LINC01252	1.703703704
SERPINB12	2.11E-07	0.1355316	0.14	0.103	0.005091727	1.3	SERPINB1	1.359223301
PPP2R5A	2.17E-07	0.1010472	0.202	0.156	0.005238497	1.3	PPP2R5A	1.294871795

C10orf76	2.81E-07	0.1261573	0.169	0.128	0.006784298	1.3	C10orf76	1.3203125
KLF3	2.84E-07	0.1110311	0.148	0.109	0.006846788	1.3	KLF3	1.357798165
POLR2L	3.19E-07	0.1032255	0.057	0.036	0.007691766	1.3	POLR2L	1.583333333
PRMT2	3.20E-07	0.1158029	0.169	0.128	0.007717069	1.3	PRMT2	1.3203125
EPS8L2	3.94E-07	0.1375314	0.043	0.025	0.009510594	1.3	EPS8L2	1.72
R3HCC1L	5.35E-07	0.1046456	0.166	0.127	0.01289272	1.3	R3HCC1L	1.307086614
SYMPK	5.61E-07	0.1060167	0.086	0.059	0.01351576	1.3	SYMPK	1.457627119
TOMM202	6.32E-07	0.1268282	0.236	0.189	0.01523758	1.3	TOMM20	1.248677249
TBCK	6.46E-07	0.1059564	0.156	0.119	0.01557966	1.3	TBCK	1.31092437
SGCD1	6.65E-07	0.1073454	0.064	0.041	0.01603064	1.3	SGCD	1.56097561
PTEN2	7.10E-07	0.1987278	0.838	0.804	0.01711163	1.3	PTEN	1.042288557
RPLP2	7.49E-07	0.3790721	0.265	0.222	0.01805376	1.3	RPLP2	1.193693694
HIPK3	7.61E-07	0.1346627	0.236	0.188	0.01834093	1.3	HIPK3	1.255319149
SRSF7	8.29E-07	0.1154	0.31	0.253	0.01998086	1.3	SRSF7	1.225296443
SECISBP2L	1.01E-06	0.1102324	0.263	0.211	0.0243737	1.3	SECISBP2L	1.246445498
EGR1	1.01E-06	0.1275054	0.102	0.073	0.02440164	1.3	EGR1	1.397260274
NUMB	1.02E-06	0.1034982	0.501	0.43	0.02454298	1.3	NUMB	1.165116279
KCNQ52	1.05E-06	0.1044562	0.072	0.048	0.02526578	1.3	KCNQ5	1.5
WDR60	1.18E-06	0.1336721	0.156	0.12	0.02846401	1.3	WDR60	1.3
CLTA	1.30E-06	0.1005971	0.19	0.148	0.03138005	1.3	CLTA	1.283783784
COG51	1.32E-06	0.1064915	0.512	0.439	0.0317416	1.3	COG5	1.166287016
ECHDC2	2.02E-06	0.1045691	0.156	0.118	0.04859659	1.3	ECHDC2	1.322033898
FDCSP1	0	3.479832	0.364	0.101	0	1.4	FDCSP	3.603960396
S100A81	0	2.913251	0.381	0.086	0	1.4	S100A8	4.430232558
SAA11	0	2.683349	0.995	0.697	0	1.4	SAA1	1.427546628
KYNU1	0	2.635694	0.777	0.173	0	1.4	KYNU	4.49132948
LTF1	0	2.580376	0.932	0.267	0	1.4	LTF	3.490636704
FABP7	0	2.457187	0.4	0.069	0	1.4	FABP7	5.797101449
OVOS21	0	2.447478	0.769	0.204	0	1.4	OVOS2	3.769607843
CHI3L11	0	2.212951	0.453	0.057	0	1.4	CHI3L1	7.947368421
FBLN51	0	2.163622	0.664	0.101	0	1.4	FBLN5	6.574257426
CHI3L21	0	2.134689	0.761	0.205	0	1.4	CHI3L2	3.712195122
CCL22	0	2.098008	0.676	0.205	0	1.4	CCL2	3.297560976
FRMD4A2	0	2.038777	0.796	0.21	0	1.4	FRMD4A	3.79047619
RARRES11	0	2.023326	0.716	0.147	0	1.4	RARRES1	4.870748299
SLPI2	0	1.936807	0.879	0.508	0	1.4	SLPI	1.730314961
TNFAIP62	0	1.891871	0.654	0.146	0	1.4	TNFAIP6	4.479452055
ARHGAP261	0	1.782003	0.982	0.625	0	1.4	ARHGAP26	1.5712
LYN2	0	1.774238	0.922	0.342	0	1.4	LYN	2.695906433
SERPINB41	0	1.772606	0.202	0.026	0	1.4	SERPINB4	7.769230769
EVA1C1	0	1.759207	0.802	0.231	0	1.4	EVA1C	3.471861472
VNN31	0	1.728935	0.539	0.087	0	1.4	VNN3	6.195402299
SORBS23	0	1.726753	0.78	0.328	0	1.4	SORBS2	2.37804878
MGAM22	0	1.709629	0.603	0.114	0	1.4	MGAM2	5.289473684
SAA2-SAA42	0	1.617659	0.661	0.12	0	1.4	SAA2-SAA4	5.508333333
SLC25A373	0	1.610857	0.892	0.319	0	1.4	SLC25A37	2.796238245
SERPINB71	0	1.599854	0.41	0.052	0	1.4	SERPINB7	7.884615385
CYP7B11	0	1.579719	0.636	0.178	0	1.4	CYP7B1	3.573033708
PSTPIP21	0	1.509049	0.67	0.168	0	1.4	PSTPIP2	3.988095238
UBD2	0	1.505735	0.301	0.063	0	1.4	UBD	4.777777778
CA82	0	1.449499	0.665	0.18	0	1.4	CA8	3.694444444
MAML23	0	1.436108	0.889	0.355	0	1.4	MAML2	2.504225352

TXNIP1	0	1.399408	0.745	0.336	0	1.4	TXNIP	2.217261905
SOD22	0	1.39352	0.997	0.749	0	1.4	SOD2	1.331108144
PLEKHS11	0	1.380521	0.521	0.078	0	1.4	PLEKHS1	6.679487179
CHODL1	0	1.37964	0.446	0.078	0	1.4	CHODL	5.717948718
SLC34A22	0	1.366499	0.542	0.086	0	1.4	SLC34A2	6.302325581
DOCK41	0	1.343173	0.699	0.263	0	1.4	DOCK4	2.657794677
CCL283	0	1.332183	0.78	0.312	0	1.4	CCL28	2.5
AGAP11	0	1.332108	0.834	0.464	0	1.4	AGAP1	1.797413793
THADA1	0	1.310047	0.493	0.164	0	1.4	THADA	3.006097561
PROM12	0	1.297729	0.64	0.174	0	1.4	PROM1	3.67816092
RP11-114H23.11	0	1.2623	0.357	0.073	0	1.4	RP11-114H	4.890410959
KIT3	0	1.25362	0.565	0.125	0	1.4	KIT	4.52
DCHS21	0	1.236639	0.362	0.038	0	1.4	DCHS2	9.526315789
MARCH31	0	1.235274	0.424	0.12	0	1.4	MARCH3	3.533333333
CNKSR32	0	1.229286	0.642	0.265	0	1.4	CNKSR3	2.422641509
WFDC22	0	1.229204	0.662	0.281	0	1.4	WFDC2	2.355871886
EFNA52	0	1.22489	0.653	0.233	0	1.4	EFNA5	2.802575107
SVIL3	0	1.21579	0.909	0.543	0	1.4	SVIL	1.674033149
SEMA6A3	0	1.214029	0.565	0.178	0	1.4	SEMA6A	3.174157303
CNTN41	0	1.213674	0.419	0.1	0	1.4	CNTN4	4.19
MTHFD2L2	0	1.192647	0.773	0.354	0	1.4	MTHFD2L	2.183615819
C32	0	1.18007	0.508	0.1	0	1.4	C3	5.08
HS3ST42	0	1.176277	0.516	0.146	0	1.4	HS3ST4	3.534246575
PIK3R11	0	1.175636	0.486	0.131	0	1.4	PIK3R1	3.709923664
NCOA73	0	1.175401	0.813	0.392	0	1.4	NCOA7	2.073979592
RAPGEF52	0	1.173599	0.694	0.293	0	1.4	RAPGEF5	2.368600683
SFRP13	0	1.171654	0.609	0.176	0	1.4	SFRP1	3.460227273
ALPK11	0	1.163068	0.575	0.173	0	1.4	ALPK1	3.323699422
SORBS12	0	1.146185	0.555	0.151	0	1.4	SORBS1	3.675496689
PDE4B3	0	1.138603	0.961	0.46	0	1.4	PDE4B	2.089130435
HSD11B12	0	1.129923	0.377	0.06	0	1.4	HSD11B1	6.283333333
CCND31	0	1.119279	0.62	0.239	0	1.4	CCND3	2.594142259
MAP3K133	0	1.097066	0.916	0.582	0	1.4	MAP3K13	1.573883162
BBOX13	0	1.079669	0.389	0.091	0	1.4	BBOX1	4.274725275
PTPN22	0	1.07619	0.578	0.203	0	1.4	PTPN2	2.84729064
SLC28A32	0	1.074968	0.536	0.13	0	1.4	SLC28A3	4.123076923
GABRP3	0	1.064567	0.628	0.218	0	1.4	GABRP	2.880733945
ST51	0	1.047752	0.693	0.296	0	1.4	ST5	2.341216216
ANK31	0	1.047005	0.626	0.264	0	1.4	ANK3	2.371212121
SLC26A21	0	1.026377	0.313	0.051	0	1.4	SLC26A2	6.137254902
PDZRN31	0	1.024124	0.37	0.066	0	1.4	PDZRN3	5.606060606
PADI21	0	1.018897	0.368	0.051	0	1.4	PADI2	7.215686275
PLXDC21	0	1.008537	0.42	0.085	0	1.4	PLXDC2	4.941176471
RIN21	0	0.9926191	0.514	0.171	0	1.4	RIN2	3.005847953
MEIS22	0	0.9805151	0.405	0.094	0	1.4	MEIS2	4.308510638
ELF52	0	0.9792623	0.439	0.105	0	1.4	ELF5	4.180952381
ZNF5211	0	0.9752873	0.345	0.06	0	1.4	ZNF521	5.75
NR2F2-AS11	0	0.9648049	0.322	0.04	0	1.4	NR2F2-AS1	8.05
RASGRP11	0	0.9619503	0.33	0.036	0	1.4	RASGRP1	9.166666667
VNN12	0	0.936522	0.389	0.093	0	1.4	VNN1	4.182795699
GUCY1A32	0	0.9187783	0.358	0.083	0	1.4	GUCY1A3	4.313253012
PODXL1	0	0.8960945	0.36	0.067	0	1.4	PODXL	5.373134328

IFI162	0	0.8741174	0.404	0.1	0	1.4	IFI16	4.04
CTD-2015G9.21	0	0.8397638	0.332	0.054	0	1.4	CTD-2015G	6.148148148
ZNF518A1	0	0.8330162	0.377	0.099	0	1.4	ZNF518A	3.808080808
SAA42	0	0.7675089	0.385	0.099	0	1.4	SAA4	3.888888889
MARCO1	0	0.7503821	0.227	0.024	0	1.4	MARCO	9.458333333
C10orf901	0	0.7422067	0.25	0.031	0	1.4	C10orf90	8.064516129
NALCN1	0	0.7314488	0.279	0.052	0	1.4	NALCN	5.365384615
RP11-449D8.51	0	0.7179585	0.198	0.023	0	1.4	RP11-449D	8.608695652
RP11-739G5.1	0	0.7118937	0.131	0.007	0	1.4	RP11-739G	18.71428571
GLIPR22	0	0.711588	0.253	0.037	0	1.4	GLIPR2	6.837837838
CLEC7A1	0	0.6804386	0.258	0.047	0	1.4	CLEC7A	5.489361702
SCARA31	0	0.6479124	0.181	0.013	0	1.4	SCARA3	13.92307692
TNIP31	0	0.5620333	0.185	0.019	0	1.4	TNIP3	9.736842105
FMN11	5.50E-307	0.8225176	0.331	0.078	1.33E-302	1.4	FMN1	4.243589744
TRABD2B1	1.42E-305	0.7751819	0.22	0.034	3.41E-301	1.4	TRABD2B	6.470588235
SLC39A81	1.02E-301	0.9372409	0.302	0.067	2.47E-297	1.4	SLC39A8	4.507462687
FAM129A3	1.23E-299	1.111697	0.587	0.232	2.97E-295	1.4	FAM129A	2.530172414
TLR22	2.67E-298	0.8456721	0.436	0.136	6.44E-294	1.4	TLR2	3.205882353
GLRX3	1.55E-296	0.8602602	0.609	0.247	3.74E-292	1.4	GLRX	2.465587045
AKR1C31	2.93E-294	0.7292614	0.317	0.073	7.07E-290	1.4	AKR1C3	4.342465753
B2M3	6.47E-294	0.88522	0.981	0.97	1.56E-289	1.4	B2M	1.011340206
ITPR21	7.93E-294	1.11776	0.774	0.436	1.91E-289	1.4	ITPR2	1.775229358
PDZK1IP11	6.26E-293	0.9264813	0.294	0.064	1.51E-288	1.4	PDZK1IP1	4.59375
EYA22	5.11E-289	0.7904003	0.324	0.078	1.23E-284	1.4	EYA2	4.153846154
RP11-142C4.61	1.18E-287	0.6769519	0.239	0.043	2.86E-283	1.4	RP11-142C	5.558139535
CHPT11	2.85E-287	1.222849	0.614	0.272	6.86E-283	1.4	CHPT1	2.257352941
DEFB12	3.15E-284	0.9865559	0.767	0.397	7.59E-280	1.4	DEFB1	1.931989924
LBP	8.19E-284	0.5448661	0.11	0.006	1.97E-279	1.4	LBP	18.33333333
C10orf101	9.49E-282	0.7651404	0.317	0.077	2.29E-277	1.4	C10orf10	4.116883117
L3MBTL42	1.63E-281	0.819734	0.446	0.143	3.92E-277	1.4	L3MBTL4	3.118881119
SYNM1	1.40E-278	0.840201	0.275	0.059	3.37E-274	1.4	SYNM	4.661016949
ARRDC33	6.10E-278	0.8025684	0.725	0.34	1.47E-273	1.4	ARRDC3	2.132352941
RP11-519G16.5	5.78E-276	0.7782739	0.214	0.036	1.39E-271	1.4	RP11-519G	5.944444444
FBN1	1.18E-274	0.5626452	0.156	0.017	2.84E-270	1.4	FBN1	9.176470588
ST8SIA11	6.05E-271	0.6907139	0.294	0.069	1.46E-266	1.4	ST8SIA1	4.260869565
SERPINB31	2.55E-264	1.473287	0.174	0.024	6.15E-260	1.4	SERPINB3	7.25
RP11-536O18.12	2.88E-263	0.6087359	0.21	0.036	6.95E-259	1.4	RP11-536O	5.833333333
COL27A11	3.28E-263	0.7407393	0.214	0.038	7.91E-259	1.4	COL27A1	5.631578947
RP11-519G16.3	3.85E-263	0.9405226	0.816	0.47	9.29E-259	1.4	RP11-519G	1.736170213
CTSS2	1.74E-262	0.7646311	0.37	0.108	4.20E-258	1.4	CTSS	3.425925926
DMD3	1.46E-261	0.9071942	0.554	0.223	3.52E-257	1.4	DMD	2.484304933
C1S1	2.93E-257	0.5429202	0.24	0.048	7.07E-253	1.4	C1S	5
SKAP22	2.43E-256	0.7613799	0.459	0.161	5.86E-252	1.4	SKAP2	2.850931677
AC016995.32	2.59E-256	0.8686721	0.339	0.096	6.25E-252	1.4	AC016995.	3.53125
SEMA6A-AS12	1.30E-255	0.8616123	0.353	0.102	3.14E-251	1.4	SEMA6A-A	3.460784314
MOB3B2	2.94E-255	0.6747678	0.386	0.118	7.10E-251	1.4	MOB3B	3.271186441
C1R1	8.09E-252	0.4998373	0.162	0.021	1.95E-247	1.4	C1R	7.714285714
TNFAIP21	1.43E-249	1.24767	0.555	0.232	3.45E-245	1.4	TNFAIP2	2.392241379
RPS27A3	2.27E-248	0.6250003	0.978	0.97	5.48E-244	1.4	RPS27A	1.008247423
RP11-266O8.1	2.20E-247	0.6682324	0.14	0.015	5.30E-243	1.4	RP11-266O	9.333333333
GHR2	2.81E-247	0.8367993	0.264	0.061	6.76E-243	1.4	GHR	4.327868852
DTNB2	2.35E-246	0.8639787	0.731	0.388	5.67E-242	1.4	DTNB	1.884020619

RGL11	1.97E-244	0.5206064	0.198	0.034	4.76E-240	1.4	RGL1	5.823529412
SLFN51	7.53E-242	0.6734937	0.56	0.235	1.82E-237	1.4	SLFN5	2.382978723
PHLDA11	1.75E-240	0.659442	0.277	0.068	4.21E-236	1.4	PHLDA1	4.073529412
ZC3H12C2	7.32E-239	0.7320203	0.322	0.09	1.77E-234	1.4	ZC3H12C	3.577777778
UCK22	1.94E-238	0.7382553	0.288	0.074	4.67E-234	1.4	UCK2	3.891891892
RPS73	1.59E-237	0.6592078	0.961	0.911	3.83E-233	1.4	RPS7	1.054884742
LUCAT12	1.27E-236	0.8497608	0.462	0.17	3.06E-232	1.4	LUCAT1	2.717647059
RNF1502	3.35E-236	0.7308597	0.232	0.049	8.07E-232	1.4	RNF150	4.734693878
CLLU1OS1	3.96E-236	0.4792052	0.18	0.029	9.54E-232	1.4	CLLU1OS	6.206896552
C4orf191	1.35E-233	0.8308833	0.365	0.117	3.25E-229	1.4	C4orf19	3.11965812
ARHGAP26-AS12	1.52E-232	0.8538076	0.371	0.119	3.68E-228	1.4	ARHGAP26	3.117647059
DENND2D1	3.94E-230	0.5628155	0.259	0.062	9.51E-226	1.4	DENND2D	4.177419355
PAPSS13	4.89E-229	0.7985507	0.491	0.195	1.18E-224	1.4	PAPSS1	2.517948718
MMP73	7.39E-226	0.7075538	0.378	0.122	1.78E-221	1.4	MMP7	3.098360656
FAM157C2	1.52E-223	0.5869371	0.257	0.062	3.68E-219	1.4	FAM157C	4.14516129
CACNB21	1.37E-222	0.7073633	0.28	0.074	3.30E-218	1.4	CACNB2	3.783783784
CLU1	9.05E-220	1.10152	0.256	0.063	2.18E-215	1.4	CLU	4.063492063
ASS11	2.03E-219	0.6134716	0.262	0.066	4.90E-215	1.4	ASS1	3.96969697
SOX101	3.08E-219	0.5877475	0.208	0.042	7.43E-215	1.4	SOX10	4.952380952
EGFR2	3.69E-219	0.7532834	0.418	0.149	8.91E-215	1.4	EGFR	2.805369128
RPS233	8.35E-219	0.5769108	0.975	0.967	2.01E-214	1.4	RPS23	1.008273009
LINC012353	5.79E-216	0.6052939	0.272	0.071	1.40E-211	1.4	LINC01235	3.830985915
CEPT12	2.13E-215	0.6826488	0.413	0.15	5.15E-211	1.4	CEPT1	2.753333333
SMARCD31	1.52E-213	0.3500409	0.099	0.008	3.65E-209	1.4	SMARCD3	12.375
OSMR2	4.88E-210	0.8360945	0.574	0.272	1.18E-205	1.4	OSMR	2.110294118
ENPP61	6.40E-210	0.3964432	0.12	0.013	1.54E-205	1.4	ENPP6	9.230769231
RPL63	2.78E-208	0.5793727	0.967	0.947	6.69E-204	1.4	RPL6	1.021119324
SGPP22	2.81E-208	0.6631985	0.299	0.086	6.77E-204	1.4	SGPP2	3.476744186
CCL202	8.49E-208	1.220871	0.409	0.155	2.05E-203	1.4	CCL20	2.638709677
PIGR3	2.32E-206	0.8321327	0.516	0.214	5.61E-202	1.4	PIGR	2.411214953
SLC39A143	6.38E-205	0.7454439	0.438	0.168	1.54E-200	1.4	SLC39A14	2.607142857
CYP1B1	2.44E-204	0.5444326	0.308	0.091	5.88E-200	1.4	CYP1B1	3.384615385
IFITM31	3.19E-203	0.8051613	0.408	0.156	7.70E-199	1.4	IFITM3	2.615384615
PPP2R3A2	9.00E-202	0.8196082	0.593	0.293	2.17E-197	1.4	PPP2R3A	2.023890785
PRELID11	7.33E-201	0.5387406	0.25	0.064	1.77E-196	1.4	PRELID1	3.90625
PIWIL41	7.78E-200	0.3975901	0.135	0.019	1.88E-195	1.4	PIWIL4	7.105263158
UST1	8.58E-198	0.8428318	0.409	0.157	2.07E-193	1.4	UST	2.605095541
BIRC33	3.33E-197	0.6846147	0.862	0.58	8.02E-193	1.4	BIRC3	1.486206897
TRPS11	4.90E-196	0.8228939	0.909	0.716	1.18E-191	1.4	TRPS1	1.269553073
NFIB2	6.49E-196	0.7128299	0.925	0.717	1.57E-191	1.4	NFIB	1.290097629
LINC011841	2.20E-195	0.6367625	0.346	0.116	5.30E-191	1.4	LINC01184	2.982758621
TANK3	2.65E-195	0.7700992	0.739	0.44	6.38E-191	1.4	TANK	1.679545455
FGGY2	1.32E-194	0.6576362	0.339	0.113	3.17E-190	1.4	FGGY	3
GXYLT21	8.41E-194	0.476465	0.177	0.034	2.03E-189	1.4	GXYLT2	5.205882353
BOC1	1.16E-193	0.3416734	0.096	0.008	2.81E-189	1.4	BOC	12
C15orf481	3.61E-192	1.264505	0.722	0.47	8.71E-188	1.4	C15orf48	1.536170213
CMPK12	1.63E-191	0.6874719	0.442	0.178	3.94E-187	1.4	CMPK1	2.483146067
MT-CO33	8.77E-191	0.4640066	0.99	0.996	2.12E-186	1.4	MT-CO3	0.993975904
MIR646HG1	1.16E-190	0.5323199	0.183	0.037	2.79E-186	1.4	MIR646HG	4.945945946
PNISR2	1.55E-189	0.701153	0.879	0.654	3.73E-185	1.4	PNISR	1.344036697
HP1	6.44E-189	0.9007689	0.08	0.005	1.55E-184	1.4	HP	16
FMO22	2.46E-188	0.5691124	0.192	0.042	5.94E-184	1.4	FMO2	4.571428571

EEF1B23	5.18E-185	0.6285546	0.902	0.707	1.25E-180	1.4	EEF1B2	1.275813296
DOCK71	6.03E-185	0.6986857	0.358	0.128	1.45E-180	1.4	DOCK7	2.796875
GALNT151	4.11E-184	0.7073194	0.216	0.053	9.92E-180	1.4	GALNT15	4.075471698
TCF7L13	1.20E-183	0.683772	0.405	0.155	2.90E-179	1.4	TCF7L1	2.612903226
RPL323	1.76E-183	0.5094811	0.975	0.976	4.24E-179	1.4	RPL32	0.99897541
RANBP172	9.75E-183	0.6017773	0.275	0.082	2.35E-178	1.4	RANBP17	3.353658537
LINC011221	2.66E-182	0.5062801	0.138	0.022	6.42E-178	1.4	LINC01122	6.272727273
SLC5A61	6.07E-181	0.5545489	0.255	0.072	1.46E-176	1.4	SLC5A6	3.541666667
C1RL1	1.16E-180	0.5673908	0.229	0.06	2.80E-176	1.4	C1RL	3.816666667
RPL303	1.24E-180	0.4740505	0.969	0.952	2.98E-176	1.4	RPL30	1.017857143
RP11-141O11.21	1.35E-179	0.3335646	0.092	0.009	3.26E-175	1.4	RP11-141O	10.22222222
ALDH1A33	4.72E-179	0.6890239	0.533	0.243	1.14E-174	1.4	ALDH1A3	2.193415638
CCL41	1.21E-178	2.587351	0.168	0.034	2.91E-174	1.4	CCL4	4.941176471
LINC011381	3.71E-178	0.6592114	0.376	0.143	8.93E-174	1.4	LINC01138	2.629370629
TPST11	1.44E-177	0.6940212	0.282	0.087	3.48E-173	1.4	TPST1	3.24137931
FAM177B2	5.00E-177	0.9883458	0.749	0.46	1.21E-172	1.4	FAM177B	1.62826087
NPAS31	4.50E-175	0.6930932	0.268	0.081	1.09E-170	1.4	NPAS3	3.308641975
SLC11A22	1.45E-174	0.6941155	0.607	0.315	3.51E-170	1.4	SLC11A2	1.926984127
HMGN31	8.16E-174	0.5534468	0.299	0.097	1.97E-169	1.4	HMGN3	3.082474227
SPX1	8.39E-174	0.3479703	0.107	0.013	2.02E-169	1.4	SPX	8.230769231
PPP1R9A1	2.75E-173	0.6637992	0.355	0.131	6.64E-169	1.4	PPP1R9A	2.709923664
SLC18B13	2.95E-173	0.6021685	0.274	0.084	7.12E-169	1.4	SLC18B1	3.261904762
LINC011983	1.84E-171	0.6274197	0.353	0.128	4.43E-167	1.4	LINC01198	2.7578125
RPL44	4.27E-171	0.5258175	0.959	0.882	1.03E-166	1.4	RPL4	1.087301587
RPL113	1.42E-170	0.505058	0.973	0.957	3.42E-166	1.4	RPL11	1.016718913
ADAMTS9-AS22	2.59E-170	0.6458016	0.362	0.136	6.24E-166	1.4	ADAMTS9-/	2.661764706
PLSCR12	4.68E-170	0.6265603	0.408	0.166	1.13E-165	1.4	PLSCR1	2.457831325
PPM1H1	3.31E-169	0.8224031	0.555	0.283	7.98E-165	1.4	PPM1H	1.961130742
SLC12A22	4.03E-169	0.8281934	0.657	0.378	9.71E-165	1.4	SLC12A2	1.738095238
RP11-66B24.52	1.07E-168	0.4433643	0.171	0.036	2.57E-164	1.4	RP11-66B2	4.75
STOX22	2.81E-168	0.5415715	0.247	0.072	6.78E-164	1.4	STOX2	3.430555556
FAM20C1	2.42E-166	0.5638074	0.178	0.04	5.83E-162	1.4	FAM20C	4.45
FAM20A1	4.72E-166	0.433056	0.156	0.031	1.14E-161	1.4	FAM20A	5.032258065
ST6GAL12	1.61E-165	0.7214817	0.481	0.222	3.88E-161	1.4	ST6GAL1	2.166666667
RP5-1198O20.41	4.16E-165	0.6341207	0.23	0.064	1.00E-160	1.4	RP5-1198O	3.59375
ITGB83	3.72E-164	0.6692705	0.761	0.463	8.97E-160	1.4	ITGB8	1.64362851
C4BPA1	7.63E-164	0.3581435	0.098	0.011	1.84E-159	1.4	C4BPA	8.909090909
KREMEN11	8.68E-164	0.4296423	0.164	0.034	2.09E-159	1.4	KREMEN1	4.823529412
RBPMS3	4.55E-162	0.697085	0.864	0.634	1.10E-157	1.4	RBPMS	1.362776025
IL343	5.02E-162	0.5764589	0.228	0.063	1.21E-157	1.4	IL34	3.619047619
AL109761.51	6.63E-161	0.3006087	0.105	0.014	1.60E-156	1.4	AL109761.!	7.5
JPX1	1.08E-159	0.632546	0.395	0.164	2.61E-155	1.4	JPX	2.408536585
ABCC4	2.99E-159	0.3975046	0.1	0.012	7.21E-155	1.4	ABCC4	8.333333333
PLA2R11	5.08E-159	0.7069965	0.393	0.163	1.22E-154	1.4	PLA2R1	2.411042945
TNFRSF1B2	1.80E-158	0.3363875	0.135	0.024	4.34E-154	1.4	TNFRSF1B	5.625
BRINP12	2.98E-156	0.7423706	0.264	0.084	7.20E-152	1.4	BRINP1	3.142857143
SYNPO22	5.65E-156	0.8317045	0.257	0.081	1.36E-151	1.4	SYNPO2	3.172839506
RPL53	1.37E-154	0.4790236	0.965	0.938	3.30E-150	1.4	RPL5	1.028784648
ST6GALNAC53	1.75E-154	0.687033	0.393	0.16	4.21E-150	1.4	ST6GALNA(	2.45625
GNE1	9.13E-154	0.5844723	0.303	0.108	2.20E-149	1.4	GNE	2.805555556
NRG21	1.18E-153	0.393292	0.117	0.018	2.84E-149	1.4	NRG2	6.5
CCSER11	2.44E-153	0.66641	0.685	0.411	5.88E-149	1.4	CCSER1	1.666666667

SIK33	1.44E-152	0.5321016	0.982	0.971	3.47E-148	1.4	SIK3	1.011328527
PDE8A3	1.77E-151	0.790588	0.555	0.299	4.27E-147	1.4	PDE8A	1.856187291
AC097724.33	7.20E-151	0.3852535	0.148	0.031	1.74E-146	1.4	AC097724.	4.774193548
RPL143	2.10E-150	0.4297317	0.97	0.959	5.05E-146	1.4	RPL14	1.011470282
C4BPB1	3.19E-150	0.2869103	0.095	0.012	7.68E-146	1.4	C4BPB	7.916666667
APOO3	6.55E-149	0.4190634	0.973	0.966	1.58E-144	1.4	APOO	1.007246377
FAS3	1.42E-148	0.4692818	0.253	0.08	3.42E-144	1.4	FAS	3.1625
KCNJ21	3.80E-148	0.2077021	0.065	0.004	9.17E-144	1.4	KCNJ2	16.25
RPL73	5.18E-148	0.7032684	0.951	0.867	1.25E-143	1.4	RPL7	1.096885813
RPL35A3	5.76E-148	0.4630743	0.971	0.953	1.39E-143	1.4	RPL35A	1.018887723
RP1-28O10.11	7.47E-148	0.3376383	0.132	0.025	1.80E-143	1.4	RP1-28O10	5.28
LRIG11	1.29E-147	0.7925388	0.438	0.203	3.12E-143	1.4	LRIG1	2.157635468
C1QTNF11	2.00E-147	0.3112818	0.097	0.013	4.82E-143	1.4	C1QTNF1	7.461538462
HIBADH2	7.09E-147	0.5404353	0.287	0.101	1.71E-142	1.4	HIBADH	2.841584158
RPS63	1.88E-146	0.444926	0.975	0.978	4.53E-142	1.4	RPS6	0.996932515
PNRC12	2.62E-146	0.6566411	0.649	0.388	6.33E-142	1.4	PNRC1	1.672680412
C21orf91-OT11	1.65E-145	0.3059305	0.097	0.013	3.97E-141	1.4	C21orf91-C	7.461538462
RPSA3	1.77E-145	0.5526185	0.928	0.795	4.26E-141	1.4	RPSA	1.167295597
AKR1C11	4.93E-144	0.5949479	0.203	0.057	1.19E-139	1.4	AKR1C1	3.561403509
MT-ND43	6.51E-144	0.4154276	0.986	0.995	1.57E-139	1.4	MT-ND4	0.990954774
ENOSF11	8.61E-144	0.6094775	0.358	0.147	2.08E-139	1.4	ENOSF1	2.43537415
RPS273	1.38E-143	0.8667051	0.819	0.613	3.33E-139	1.4	RPS27	1.336052202
MT-CO23	6.09E-143	0.4939102	0.982	0.987	1.47E-138	1.4	MT-CO2	0.994934144
AC009313.13	1.25E-142	0.4213524	0.185	0.048	3.01E-138	1.4	AC009313.	3.854166667
RP11-290O12.21	5.29E-142	0.5931139	0.188	0.05	1.27E-137	1.4	RP11-290O	3.76
RPL213	1.79E-141	0.5275462	0.948	0.86	4.32E-137	1.4	RPL21	1.102325581
MINA1	2.01E-141	0.3384652	0.143	0.03	4.85E-137	1.4	MINA	4.766666667
SLC24A31	2.48E-141	0.5328712	0.274	0.095	5.98E-137	1.4	SLC24A3	2.884210526
PDXK1	1.88E-140	0.5356666	0.269	0.093	4.54E-136	1.4	PDXK	2.892473118
USP131	2.63E-140	0.4156582	0.152	0.034	6.35E-136	1.4	USP13	4.470588235
ARHGAP441	1.91E-139	0.5643842	0.369	0.154	4.60E-135	1.4	ARHGAP44	2.396103896
EEF1A13	1.02E-137	0.4174945	0.975	0.975	2.47E-133	1.4	EEF1A1	1
RP11-356C4.53	1.80E-137	0.6062469	0.325	0.127	4.35E-133	1.4	RP11-356C	2.559055118
VCAM1	2.08E-137	0.2468133	0.053	0.003	5.01E-133	1.4	VCAM1	17.66666667
TLR51	2.77E-137	0.2864916	0.11	0.018	6.69E-133	1.4	TLR5	6.111111111
OSMR-AS11	9.64E-137	0.5648104	0.356	0.148	2.32E-132	1.4	OSMR-AS1	2.405405405
LAP31	1.29E-135	0.5547849	0.274	0.099	3.11E-131	1.4	LAP3	2.767676768
NMI1	2.40E-135	0.4061152	0.201	0.058	5.80E-131	1.4	NMI	3.465517241
SFT2D23	1.21E-134	0.5070916	0.297	0.111	2.91E-130	1.4	SFT2D2	2.675675676
CFLAR2	3.64E-134	0.6897727	0.566	0.312	8.78E-130	1.4	CFLAR	1.814102564
RPS133	3.76E-134	0.6790416	0.869	0.682	9.07E-130	1.4	RPS13	1.274193548
LRP1B1	7.27E-134	1.082953	0.321	0.134	1.75E-129	1.4	LRP1B	2.395522388
MFGE82	1.46E-133	0.6863544	0.237	0.078	3.51E-129	1.4	MFGE8	3.038461538
WTAP3	1.51E-132	0.6652052	0.734	0.486	3.63E-128	1.4	WTAP	1.510288066
STAC21	4.30E-132	0.3396203	0.098	0.015	1.04E-127	1.4	STAC2	6.533333333
LGALS21	1.14E-131	0.3440406	0.083	0.01	2.74E-127	1.4	LGALS2	8.3
ARHGEF103	2.52E-131	0.5868167	0.418	0.194	6.07E-127	1.4	ARHGEF10	2.154639175
SGCZ	1.06E-130	0.6534617	0.173	0.046	2.57E-126	1.4	SGCZ	3.760869565
AUTS21	3.36E-130	0.6076227	0.757	0.5	8.11E-126	1.4	AUTS2	1.514
FOXN31	9.34E-130	0.5966605	0.361	0.157	2.25E-125	1.4	FOXN3	2.299363057
CAMK2D3	1.19E-129	0.5973968	0.356	0.151	2.87E-125	1.4	CAMK2D	2.357615894
SASH13	1.37E-129	0.4886183	0.337	0.136	3.30E-125	1.4	SASH1	2.477941176

TSHZ23	1.65E-129	0.5704144	0.461	0.223	3.98E-125	1.4	TSHZ2	2.067264574
NFKBIZ3	2.19E-129	0.4991793	0.803	0.528	5.29E-125	1.4	NFKBIZ	1.520833333
CCL5	5.60E-129	0.3005079	0.067	0.006	1.35E-124	1.4	CCL5	11.16666667
TPT1-AS13	8.01E-129	0.5299548	0.441	0.208	1.93E-124	1.4	TPT1-AS1	2.120192308
KCNQ31	1.01E-128	0.4305678	0.112	0.02	2.43E-124	1.4	KCNQ3	5.6
RNF1453	1.45E-128	0.4241607	0.582	0.307	3.50E-124	1.4	RNF145	1.895765472
NUB12	1.83E-128	0.7464519	0.374	0.169	4.42E-124	1.4	NUB1	2.213017751
P3H23	2.31E-128	0.5101407	0.186	0.052	5.57E-124	1.4	P3H2	3.576923077
LINC01344	1.99E-126	0.3906075	0.119	0.023	4.80E-122	1.4	LINC01344	5.173913043
CTC-340D7.11	1.13E-125	0.2308032	0.071	0.008	2.73E-121	1.4	CTC-340D7	8.875
BCL11A1	3.80E-125	0.3172128	0.115	0.022	9.15E-121	1.4	BCL11A	5.227272727
GPHN1	6.89E-125	0.531473	0.442	0.212	1.66E-120	1.4	GPHN	2.08490566
NACA4	8.69E-125	0.413578	0.953	0.903	2.10E-120	1.4	NACA	1.055370986
LINC011831	1.42E-124	0.3340146	0.114	0.022	3.42E-120	1.4	LINC01183	5.181818182
TRIM562	1.56E-124	0.5511431	0.32	0.133	3.75E-120	1.4	TRIM56	2.406015038
KIAA12171	1.78E-124	0.5853366	0.932	0.785	4.30E-120	1.4	KIAA1217	1.187261146
SPDYE22	2.60E-123	0.4567195	0.29	0.113	6.27E-119	1.4	SPDYE2	2.566371681
DAPP13	1.03E-122	0.5005317	0.449	0.216	2.48E-118	1.4	DAPP1	2.078703704
SLC47A13	1.22E-122	0.453157	0.932	0.891	2.94E-118	1.4	SLC47A1	1.046015713
HSD17B21	1.24E-122	0.3407667	0.137	0.031	3.00E-118	1.4	HSD17B2	4.419354839
GABPB1-AS12	1.26E-122	0.4776355	0.301	0.12	3.04E-118	1.4	GABPB1-AS	2.508333333
GSAP2	1.32E-122	0.4503079	0.224	0.074	3.19E-118	1.4	GSAP	3.027027027
IFNAR22	7.50E-122	0.5180143	0.381	0.173	1.81E-117	1.4	IFNAR2	2.202312139
RPS203	7.98E-122	0.5341434	0.958	0.92	1.92E-117	1.4	RPS20	1.041304348
RPS3A3	1.29E-121	0.444836	0.942	0.893	3.11E-117	1.4	RPS3A	1.054871221
RP11-142M10.21	3.72E-121	0.2362386	0.065	0.006	8.96E-117	1.4	RP11-142M	10.83333333
TMCO41	6.34E-121	0.4003632	0.184	0.053	1.53E-116	1.4	TMCO4	3.471698113
S100A91	1.25E-120	0.7458288	0.115	0.023	3.01E-116	1.4	S100A9	5
ST3GAL62	2.63E-120	0.3885004	0.176	0.05	6.35E-116	1.4	ST3GAL6	3.52
CCDC141	5.72E-120	0.4213933	0.236	0.082	1.38E-115	1.4	CCDC14	2.87804878
FHIT	1.27E-119	0.6980234	0.57	0.33	3.05E-115	1.4	FHIT	1.727272727
LPL1	1.90E-119	0.3016529	0.102	0.018	4.57E-115	1.4	LPL	5.666666667
OOEP3	1.93E-118	0.4549553	0.969	0.965	4.66E-114	1.4	OOEP	1.004145078
RPS15A3	2.13E-118	0.4280869	0.96	0.939	5.15E-114	1.4	RPS15A	1.022364217
PLA2G4A1	3.39E-118	0.2563809	0.093	0.015	8.17E-114	1.4	PLA2G4A	6.2
IGFBP7	5.48E-118	0.2961525	0.063	0.006	1.32E-113	1.4	IGFBP7	10.5
RPL343	5.70E-118	0.3981015	0.975	0.979	1.37E-113	1.4	RPL34	0.995914198
IKBKE1	2.82E-117	0.2709608	0.091	0.015	6.80E-113	1.4	IKBKE	6.066666667
ZNHIT61	4.22E-117	0.556785	0.31	0.13	1.02E-112	1.4	ZNHIT6	2.384615385
MGC32805	1.01E-116	0.2021205	0.063	0.006	2.44E-112	1.4	MGC32805	10.5
RPS243	1.12E-116	0.355248	0.975	0.965	2.69E-112	1.4	RPS24	1.010362694
METAP22	1.57E-116	0.5088069	0.439	0.22	3.80E-112	1.4	METAP2	1.995454545
LINC014821	6.26E-116	0.2934358	0.076	0.01	1.51E-111	1.4	LINC01482	7.6
RP11-37B2.11	1.19E-115	0.5815358	0.418	0.202	2.87E-111	1.4	RP11-37B2	2.069306931
ATL21	1.84E-115	0.5856874	0.414	0.203	4.45E-111	1.4	ATL2	2.039408867
BPGM2	6.19E-115	0.4781436	0.217	0.074	1.49E-110	1.4	BPGM	2.932432432
TOMM73	7.06E-115	0.4332808	0.918	0.803	1.70E-110	1.4	TOMM7	1.143212951
FAM78B2	8.81E-115	0.3332134	0.117	0.025	2.12E-110	1.4	FAM78B	4.68
KCNQ11	2.08E-114	0.4003799	0.17	0.049	5.02E-110	1.4	KCNQ1	3.469387755
EIF3L3	4.51E-114	0.5106783	0.782	0.564	1.09E-109	1.4	EIF3L	1.386524823
RPS123	7.17E-114	0.7373751	0.785	0.632	1.73E-109	1.4	RPS12	1.242088608
DPYD1	7.75E-114	0.5188084	0.755	0.49	1.87E-109	1.4	DPYD	1.540816327

TBC1D3P1-DHX40P13	1.24E-112	0.5998175	0.776	0.565	2.99E-108	1.4	TBC1D3P1-	1.373451327
ZNF1241	1.54E-112	0.3663146	0.155	0.042	3.70E-108	1.4	ZNF124	3.69047619
LDHB3	2.14E-112	0.4447915	0.78	0.536	5.16E-108	1.4	LDHB	1.455223881
GPRC5B2	9.78E-112	0.3239868	0.112	0.023	2.36E-107	1.4	GPRC5B	4.869565217
DIMT11	1.55E-111	0.2923919	0.125	0.029	3.74E-107	1.4	DIMT1	4.310344828
NPM13	2.18E-111	0.4200079	0.893	0.691	5.27E-107	1.4	NPM1	1.292329957
BAIAP2L13	8.16E-111	0.6033742	0.96	0.922	1.97E-106	1.4	BAIAP2L1	1.041214751
FAAH21	9.41E-111	0.5465683	0.385	0.183	2.27E-106	1.4	FAAH2	2.103825137
CYP1B1-AS11	9.59E-111	0.283663	0.098	0.018	2.31E-106	1.4	CYP1B1-AS	5.444444444
TLR11	1.62E-110	0.3141304	0.107	0.021	3.91E-106	1.4	TLR1	5.095238095
RGS102	3.60E-110	0.3763141	0.171	0.051	8.69E-106	1.4	RGS10	3.352941176
SLC30A42	4.11E-110	0.4752684	0.237	0.087	9.91E-106	1.4	SLC30A4	2.724137931
RPS253	5.09E-110	0.5855685	0.872	0.722	1.23E-105	1.4	RPS25	1.207756233
ZMYM43	1.32E-109	0.448081	0.904	0.805	3.19E-105	1.4	ZMYM4	1.122981366
GBP23	2.67E-109	0.4854758	0.808	0.59	6.43E-105	1.4	GBP2	1.369491525
SLC16A1-AS12	2.80E-109	0.4094542	0.176	0.054	6.75E-105	1.4	SLC16A1-A:	3.259259259
DARS1	3.90E-109	0.5068917	0.438	0.224	9.41E-105	1.4	DARS	1.955357143
R3HDM21	1.49E-108	0.4959511	0.258	0.101	3.58E-104	1.4	R3HDM2	2.554455446
CECR72	2.08E-108	0.3152547	0.134	0.033	5.01E-104	1.4	CECR7	4.060606061
RP11-475O6.1	4.28E-108	0.3815318	0.12	0.027	1.03E-103	1.4	RP11-475O	4.444444444
AC027119.11	4.49E-108	0.1850611	0.052	0.004	1.08E-103	1.4	AC027119.	13
PLEKHA63	5.08E-108	0.4351781	0.916	0.822	1.22E-103	1.4	PLEKHA6	1.114355231
LRIG31	5.24E-108	0.2629909	0.101	0.02	1.26E-103	1.4	LRIG3	5.05
JARID2	8.02E-108	0.4420928	0.44	0.229	1.93E-103	1.4	JARID2	1.92139738
NT5C22	1.00E-107	0.5367123	0.462	0.245	2.42E-103	1.4	NT5C2	1.885714286
PI32	1.03E-107	0.4609446	0.304	0.127	2.49E-103	1.4	PI3	2.393700787
FTH12	1.14E-107	0.5415806	0.939	0.832	2.75E-103	1.4	FTH1	1.128605769
DIAPH21	3.81E-107	0.5607771	0.373	0.179	9.18E-103	1.4	DIAPH2	2.083798883
RP11-608O21.13	4.44E-107	0.5591792	0.739	0.512	1.07E-102	1.4	RP11-608O	1.443359375
SNED11	7.93E-107	0.3055161	0.111	0.024	1.91E-102	1.4	SNED1	4.625
RCAN13	9.60E-107	0.6064433	0.607	0.369	2.32E-102	1.4	RCAN1	1.64498645
CATSPER21	9.91E-107	0.3355716	0.158	0.045	2.39E-102	1.4	CATSPER2	3.511111111
RPL7A3	2.34E-106	0.3618762	0.967	0.957	5.64E-102	1.4	RPL7A	1.010449321
ZRANB22	3.13E-106	0.4910147	0.416	0.208	7.55E-102	1.4	ZRANB2	2
SIK2	4.13E-106	0.5674733	0.526	0.3	9.97E-102	1.4	SIK2	1.753333333
BDNF-AS3	7.91E-106	0.6342306	0.703	0.481	1.91E-101	1.4	BDNF-AS	1.461538462
NPAS22	2.28E-105	0.5934929	0.583	0.346	5.49E-101	1.4	NPAS2	1.684971098
DRAM21	4.10E-105	0.4730972	0.395	0.194	9.89E-101	1.4	DRAM2	2.036082474
ADGRL22	4.61E-105	0.5280762	0.195	0.065	1.11E-100	1.4	ADGRL2	3
ZNF1952	2.21E-104	0.3993668	0.187	0.061	5.34E-100	1.4	ZNF195	3.06557377
COA11	4.74E-104	0.4723858	0.395	0.195	1.14E-99	1.4	COA1	2.025641026
RPL23A3	5.41E-104	0.6348322	0.745	0.554	1.31E-99	1.4	RPL23A	1.344765343
CISD13	1.03E-103	0.4045681	0.28	0.115	2.49E-99	1.4	CISD1	2.434782609
GANC1	1.52E-103	0.3987923	0.202	0.07	3.67E-99	1.4	GANC	2.885714286
CH17-189H20.11	1.63E-103	0.4315066	0.216	0.078	3.94E-99	1.4	CH17-189H	2.769230769
CSF11	1.79E-103	0.4448082	0.157	0.046	4.30E-99	1.4	CSF1	3.413043478
MRAS1	2.14E-103	0.2884476	0.117	0.027	5.16E-99	1.4	MRAS	4.333333333
RTP43	2.98E-103	0.2872685	0.13	0.033	7.19E-99	1.4	RTP4	3.939393939
AKR1C21	3.20E-103	0.3226259	0.131	0.033	7.72E-99	1.4	AKR1C2	3.96969697
AC073283.41	7.63E-103	0.3694945	0.176	0.056	1.84E-98	1.4	AC073283.4	3.142857143
CYP27A11	9.62E-103	0.3631865	0.162	0.048	2.32E-98	1.4	CYP27A1	3.375
SPIRE13	1.60E-102	0.5437041	0.439	0.229	3.87E-98	1.4	SPIRE1	1.917030568

CYP27B11	1.83E-102	0.2630266	0.091	0.017	4.41E-98	1.4	CYP27B1	5.352941176
KALRN1	1.90E-102	0.4141854	0.203	0.07	4.59E-98	1.4	KALRN	2.9
RPLP03	5.95E-102	0.6164275	0.919	0.86	1.44E-97	1.4	RPLP0	1.068604651
RBP51	8.11E-102	0.2254192	0.088	0.016	1.96E-97	1.4	RBP5	5.5
HGSNAT1	9.59E-102	0.4804473	0.277	0.116	2.31E-97	1.4	HGSNAT	2.387931034
FANCL1	1.65E-101	0.2994882	0.138	0.037	3.98E-97	1.4	FANCL	3.72972973
ATP13A52	1.85E-101	0.3446744	0.089	0.016	4.46E-97	1.4	ATP13A5	5.5625
MECOM3	5.47E-101	0.4720671	0.899	0.792	1.32E-96	1.4	MECOM	1.13510101
EIF3E3	1.26E-100	0.4280058	0.78	0.549	3.04E-96	1.4	EIF3E	1.420765027
NTRK22	2.52E-100	0.4259348	0.23	0.086	6.08E-96	1.4	NTRK2	2.674418605
SLCO1A21	1.87E-99	0.2219165	0.063	0.008	4.52E-95	1.4	SLCO1A2	7.875
HCAR22	5.99E-99	0.3317693	0.232	0.087	1.44E-94	1.4	HCAR2	2.666666667
RP11-867G2.81	6.09E-99	0.2557452	0.078	0.013	1.47E-94	1.4	RP11-867G	6
ZNF222	6.31E-99	0.4395924	0.318	0.144	1.52E-94	1.4	ZNF22	2.208333333
C1orf1471	7.92E-99	0.2191961	0.071	0.011	1.91E-94	1.4	C1orf147	6.454545455
TTLL42	9.78E-99	0.4197317	0.246	0.098	2.36E-94	1.4	TTLL4	2.510204082
RPS4X3	2.01E-98	0.3265642	0.973	0.98	4.86E-94	1.4	RPS4X	0.992857143
SNCAIP	4.27E-98	0.2730104	0.07	0.01	1.03E-93	1.4	SNCAIP	7
PFDN43	4.28E-98	0.4287552	0.541	0.31	1.03E-93	1.4	PFDN4	1.74516129
MUC162	4.51E-98	0.3074261	0.091	0.018	1.09E-93	1.4	MUC16	5.055555556
DAPK13	7.01E-98	0.6302371	0.706	0.512	1.69E-93	1.4	DAPK1	1.37890625
HSD17B71	1.28E-97	0.4357191	0.205	0.074	3.09E-93	1.4	HSD17B7	2.77027027
MCTP22	1.80E-97	0.4164741	0.212	0.078	4.33E-93	1.4	MCTP2	2.717948718
EHF2	4.15E-97	0.4866957	0.68	0.448	1.00E-92	1.4	EHF	1.517857143
TRIM221	9.02E-97	0.3515254	0.181	0.06	2.17E-92	1.4	TRIM22	3.016666667
MAGI21	1.19E-96	0.5024189	0.158	0.049	2.87E-92	1.4	MAGI2	3.224489796
BCAS33	2.29E-96	0.4118946	0.87	0.722	5.52E-92	1.4	BCAS3	1.20498615
C11orf801	2.57E-96	0.4805593	0.347	0.166	6.19E-92	1.4	C11orf80	2.090361446
VPS13D1	3.10E-96	0.4883677	0.463	0.254	7.47E-92	1.4	VPS13D	1.822834646
C21orf911	8.00E-96	0.2932414	0.122	0.031	1.93E-91	1.4	C21orf91	3.935483871
CTB-113D17.1	1.34E-95	0.1502152	0.043	0.003	3.24E-91	1.4	CTB-113D1	14.33333333
SCAPER2	1.81E-95	0.453147	0.311	0.142	4.35E-91	1.4	SCAPER	2.190140845
RPS173	4.62E-95	0.345541	0.952	0.94	1.11E-90	1.4	RPS17	1.012765957
CNTNAP31	1.52E-94	0.3067735	0.102	0.023	3.66E-90	1.4	CNTNAP3	4.434782609
PKD21	3.26E-94	0.3437063	0.151	0.045	7.85E-90	1.4	PKD2	3.355555556
RPS33	3.92E-94	0.6037126	0.937	0.86	9.45E-90	1.4	RPS3	1.089534884
EPS83	7.38E-94	0.5574055	0.514	0.296	1.78E-89	1.4	EPS8	1.736486486
CCDC1462	1.18E-93	0.497022	0.277	0.121	2.85E-89	1.4	CCDC146	2.289256198
IDO1	1.22E-93	0.5939788	0.091	0.019	2.94E-89	1.4	IDO1	4.789473684
RP11-1069G10.11	1.84E-93	0.1943196	0.067	0.01	4.43E-89	1.4	RP11-1069	6.7
DISC11	4.98E-93	0.394252	0.192	0.068	1.20E-88	1.4	DISC1	2.823529412
RARRES31	7.65E-93	0.4524021	0.39	0.197	1.84E-88	1.4	RARRES3	1.979695431
ZNF33A1	2.95E-92	0.435623	0.385	0.195	7.11E-88	1.4	ZNF33A	1.974358974
ISLR	6.47E-92	0.134301	0.042	0.003	1.56E-87	1.4	ISLR	14
TBC1D51	9.44E-92	0.5261419	0.547	0.329	2.28E-87	1.4	TBC1D5	1.662613982
TMEM176B1	1.04E-91	0.2435924	0.091	0.019	2.52E-87	1.4	TMEM176E	4.789473684
CFB	1.28E-91	0.34143	0.303	0.136	3.08E-87	1.4	CFB	2.227941176
PELI22	2.95E-91	0.3523691	0.139	0.041	7.11E-87	1.4	PELI2	3.390243902
THUMPD3-AS12	9.58E-91	0.4123675	0.396	0.203	2.31E-86	1.4	THUMPD3-	1.950738916
RHBDF22	9.70E-91	0.2532105	0.115	0.029	2.34E-86	1.4	RHBDF2	3.965517241
MT-ATP61	1.47E-90	0.3477464	0.987	0.991	3.55E-86	1.4	MT-ATP6	0.995963673
RPL413	1.84E-89	0.5923611	0.948	0.897	4.43E-85	1.4	RPL41	1.056856187

SYNJ22	1.95E-89	0.5194872	0.393	0.206	4.70E-85	1.4	SYNJ2	1.90776699
ZNF6523	2.05E-89	0.5150041	0.638	0.421	4.95E-85	1.4	ZNF652	1.51543943
RELB1	2.30E-89	0.4352225	0.272	0.119	5.55E-85	1.4	RELB	2.285714286
COBLL11	2.85E-89	0.3851002	0.191	0.069	6.88E-85	1.4	COBLL1	2.768115942
SMARCA2	6.99E-89	0.4780129	0.495	0.288	1.69E-84	1.4	SMARCA2	1.71875
WDPCP1	9.85E-89	0.4555228	0.249	0.105	2.37E-84	1.4	WDPCP	2.371428571
BMPR1A1	4.04E-88	0.4829727	0.426	0.23	9.73E-84	1.4	BMPR1A	1.852173913
IQGAP21	8.91E-88	0.2902997	0.129	0.037	2.15E-83	1.4	IQGAP2	3.486486486
GS1-24F4.21	1.72E-87	0.44258	0.163	0.054	4.16E-83	1.4	GS1-24F4.2	3.018518519
ADAMTS93	2.45E-87	0.5063343	0.379	0.191	5.90E-83	1.4	ADAMTS9	1.984293194
RPL313	2.51E-87	0.3539981	0.951	0.921	6.05E-83	1.4	RPL31	1.03257329
AC005152.32	3.08E-87	0.443565	0.185	0.067	7.42E-83	1.4	AC005152.3	2.76119403
DANT21	3.29E-87	0.3353348	0.155	0.05	7.93E-83	1.4	DANT2	3.1
IL15RA2	4.15E-87	0.2827066	0.102	0.024	1.00E-82	1.4	IL15RA	4.25
MAMDC23	4.18E-87	0.3930762	0.928	0.84	1.01E-82	1.4	MAMDC2	1.104761905
XDH3	4.49E-87	0.5121947	0.149	0.047	1.08E-82	1.4	XDH	3.170212766
CATSPERB1	5.47E-87	0.4640534	0.366	0.184	1.32E-82	1.4	CATSPERB	1.989130435
CD743	5.86E-87	0.3292492	0.198	0.073	1.41E-82	1.4	CD74	2.712328767
RP11-244M2.13	9.40E-87	0.4773029	0.598	0.386	2.27E-82	1.4	RP11-244M	1.549222798
RAB291	1.67E-86	0.2518973	0.135	0.039	4.03E-82	1.4	RAB29	3.461538462
SAMD121	2.77E-86	0.5348262	0.421	0.229	6.69E-82	1.4	SAMD12	1.838427948
EPB41L11	6.70E-86	0.4100994	0.168	0.058	1.62E-81	1.4	EPB41L1	2.896551724
TMOD12	8.79E-86	0.3556048	0.179	0.064	2.12E-81	1.4	TMOD1	2.796875
ATR1	1.49E-85	0.4215884	0.272	0.122	3.59E-81	1.4	ATR	2.229508197
CTC-471J1.93	1.05E-84	0.5315701	0.788	0.617	2.52E-80	1.4	CTC-471J1.	1.277147488
CTB-91J4.1	2.17E-84	0.190629	0.035	0.002	5.23E-80	1.4	CTB-91J4.1	17.5
MGAT31	5.62E-84	0.1545864	0.048	0.005	1.35E-79	1.4	MGAT3	9.6
NACA23	1.15E-83	0.4782361	0.647	0.445	2.78E-79	1.4	NACA2	1.453932584
NNT2	1.66E-83	0.3603791	0.154	0.051	4.00E-79	1.4	NNT	3.019607843
CX3CL11	4.34E-83	0.5464506	0.394	0.211	1.05E-78	1.4	CX3CL1	1.867298578
NCOA11	6.76E-83	0.5437304	0.561	0.357	1.63E-78	1.4	NCOA1	1.571428571
NXN3	7.38E-83	0.4250359	0.206	0.081	1.78E-78	1.4	NXN	2.543209877
BTF33	7.62E-83	0.3499434	0.937	0.882	1.84E-78	1.4	BTF3	1.062358277
N4BP2L21	1.10E-82	0.4011309	0.883	0.706	2.66E-78	1.4	N4BP2L2	1.250708215
EML52	1.26E-82	0.3520321	0.16	0.054	3.05E-78	1.4	EML5	2.962962963
CSTB2	1.28E-82	0.5467289	0.277	0.128	3.09E-78	1.4	CSTB	2.1640625
LINC009981	2.34E-82	0.3428798	0.26	0.113	5.65E-78	1.4	LINC00998	2.300884956
GABRG31	2.59E-82	0.2981474	0.087	0.019	6.24E-78	1.4	GABRG3	4.578947368
PELI13	3.37E-82	0.5206048	0.505	0.307	8.12E-78	1.4	PELI1	1.64495114
BTBD111	5.45E-82	0.3022152	0.117	0.032	1.31E-77	1.4	BTBD11	3.65625
PLCB12	2.20E-81	0.3919815	0.388	0.203	5.30E-77	1.4	PLCB1	1.911330049
MT-CO13	2.60E-81	0.5083414	0.983	0.993	6.27E-77	1.4	MT-CO1	0.989929507
NUDT71	5.62E-81	0.2091271	0.09	0.02	1.36E-76	1.4	NUDT7	4.5
LINC005361	7.60E-81	0.3585603	0.149	0.049	1.83E-76	1.4	LINC00536	3.040816327
NSD11	1.35E-80	0.4016177	0.251	0.111	3.26E-76	1.4	NSD1	2.261261261
TNFSF101	1.38E-80	0.2282368	0.686	0.446	3.34E-76	1.4	TNFSF10	1.538116592
ATP6V1B22	1.40E-80	0.3706671	0.19	0.073	3.37E-76	1.4	ATP6V1B2	2.602739726
RPL193	1.68E-80	0.5460871	0.942	0.897	4.06E-76	1.4	RPL19	1.050167224
HRSP121	2.10E-80	0.3220471	0.169	0.06	5.07E-76	1.4	HRSP12	2.816666667
CNTNAP3B2	3.60E-80	0.3337151	0.152	0.051	8.68E-76	1.4	CNTNAP3B	2.980392157
FOXO12	4.41E-80	0.4222972	0.343	0.175	1.06E-75	1.4	FOXO1	1.96
MARK11	6.59E-80	0.3203217	0.157	0.053	1.59E-75	1.4	MARK1	2.962264151

HSPD13	3.46E-79	0.4233667	0.418	0.233	8.35E-75	1.4	HSPD1	1.793991416
BPI1	4.89E-79	0.2127672	0.077	0.016	1.18E-74	1.4	BPI	4.8125
FLOT21	5.23E-79	0.27974	0.106	0.028	1.26E-74	1.4	FLOT2	3.785714286
TC2N2	8.21E-79	0.5120675	0.454	0.263	1.98E-74	1.4	TC2N	1.726235741
CXCL172	1.49E-78	0.4526196	0.215	0.089	3.59E-74	1.4	CXCL17	2.415730337
RPL223	1.98E-78	0.3671185	0.816	0.631	4.78E-74	1.4	RPL22	1.29318542
ZNF4291	2.43E-78	0.3104956	0.155	0.053	5.86E-74	1.4	ZNF429	2.924528302
ANTXR11	5.59E-78	0.2670703	0.103	0.027	1.35E-73	1.4	ANTXR1	3.814814815
RPL233	7.67E-78	0.3224137	0.961	0.91	1.85E-73	1.4	RPL23	1.056043956
TPM12	1.73E-77	0.4950929	0.693	0.505	4.18E-73	1.4	TPM1	1.372277228
MOB41	2.31E-77	0.3770209	0.292	0.14	5.58E-73	1.4	MOB4	2.085714286
AKAP61	6.71E-77	0.2723112	0.096	0.024	1.62E-72	1.4	AKAP6	4
FTX1	9.07E-77	0.4692047	0.755	0.532	2.19E-72	1.4	FTX	1.419172932
RNF1752	1.20E-76	0.2805593	0.093	0.023	2.90E-72	1.4	RNF175	4.043478261
STX171	1.80E-76	0.3211718	0.239	0.104	4.34E-72	1.4	STX17	2.298076923
MT-ND22	1.87E-76	0.3161022	0.977	0.977	4.51E-72	1.4	MT-ND2	1
UQCRH3	2.31E-76	0.3708022	0.85	0.736	5.57E-72	1.4	UQCRH	1.154891304
RP11-66B24.11	2.93E-76	0.1717554	0.062	0.011	7.07E-72	1.4	RP11-66B2	5.636363636
MARCKS3	4.42E-76	0.4898362	0.526	0.329	1.07E-71	1.4	MARCKS	1.598784195
TRAF3IP22	5.63E-76	0.3830835	0.248	0.111	1.36E-71	1.4	TRAF3IP2	2.234234234
CACHD11	9.89E-76	0.3123229	0.13	0.041	2.39E-71	1.4	CACHD1	3.170731707
FGFR21	1.21E-75	0.2469718	0.079	0.017	2.93E-71	1.4	FGFR2	4.647058824
HDAC92	1.36E-75	0.4870282	0.241	0.108	3.28E-71	1.4	HDAC9	2.231481481
PAM2	3.42E-75	0.4313927	0.657	0.443	8.24E-71	1.4	PAM	1.483069977
PPP1R14C3	4.42E-75	0.3463433	0.205	0.084	1.07E-70	1.4	PPP1R14C	2.44047619
SDCCAG81	4.97E-75	0.4275888	0.279	0.133	1.20E-70	1.4	SDCCAG8	2.097744361
DBI2	5.69E-75	0.472875	0.799	0.636	1.37E-70	1.4	DBI	1.256289308
TFAP2C2	5.72E-75	0.3346993	0.183	0.071	1.38E-70	1.4	TFAP2C	2.577464789
C5orf282	8.86E-75	0.3766242	0.249	0.113	2.14E-70	1.4	C5orf28	2.203539823
RP11-318C2.11	9.19E-75	0.2395969	0.099	0.026	2.22E-70	1.4	RP11-318C	3.807692308
SLC1A31	1.06E-74	0.2194065	0.09	0.022	2.57E-70	1.4	SLC1A3	4.090909091
TEX143	1.37E-74	0.4714734	0.279	0.134	3.30E-70	1.4	TEX14	2.082089552
C21orf62-AS11	1.64E-74	0.17989	0.07	0.014	3.95E-70	1.4	C21orf62-A	5
EHBP12	2.69E-74	0.4496649	0.4	0.224	6.48E-70	1.4	EHBP1	1.785714286
GMDS-AS11	3.01E-74	0.4975351	0.224	0.097	7.26E-70	1.4	GMDS-AS1	2.309278351
TACC12	6.81E-74	0.5202897	0.3	0.152	1.64E-69	1.4	TACC1	1.973684211
PARP42	9.34E-74	0.3800036	0.283	0.137	2.25E-69	1.4	PARP4	2.065693431
ZNF5622	1.26E-73	0.3742253	0.288	0.141	3.04E-69	1.4	ZNF562	2.042553191
FAM3B1	1.53E-73	0.2471757	0.116	0.035	3.68E-69	1.4	FAM3B	3.314285714
HIVEP31	4.15E-73	0.5569064	0.484	0.296	1.00E-68	1.4	HIVEP3	1.635135135
RPS210	5.24E-73	0.5425857	0.434	0.255	1.26E-68	1.4	RPS2	1.701960784
IMMP2L1	5.74E-73	0.5152268	0.457	0.274	1.38E-68	1.4	IMMP2L	1.667883212
EGF1	7.14E-73	0.2769108	0.109	0.031	1.72E-68	1.4	EGF	3.516129032
VNN21	8.34E-73	0.2043484	0.065	0.012	2.01E-68	1.4	VNN2	5.416666667
ENTPD1-AS11	9.70E-73	0.2967383	0.131	0.043	2.34E-68	1.4	ENTPD1-AS	3.046511628
FAF11	1.22E-72	0.4468091	0.384	0.213	2.93E-68	1.4	FAF1	1.802816901
SPDYE162	1.35E-72	0.2887743	0.148	0.052	3.26E-68	1.4	SPDYE16	2.846153846
RP11-817J15.23	1.88E-72	0.2523428	0.131	0.042	4.53E-68	1.4	RP11-817J1	3.119047619
LINC004863	2.13E-72	0.2646523	0.997	0.986	5.14E-68	1.4	LINC00486	1.011156187
PRDM12	2.73E-72	0.22564	0.096	0.025	6.58E-68	1.4	PRDM1	3.84
TBCA3	3.08E-72	0.394727	0.714	0.522	7.44E-68	1.4	TBCA	1.367816092
IDH21	5.96E-72	0.3264776	0.173	0.067	1.44E-67	1.4	IDH2	2.582089552

SMC53	8.49E-72	0.4227528	0.473	0.284	2.05E-67	1.4	SMC5	1.665492958
INSR3	9.14E-72	0.472473	0.717	0.534	2.20E-67	1.4	INSR	1.342696629
CRTC31	2.17E-71	0.3968951	0.355	0.191	5.23E-67	1.4	CRTC3	1.858638743
RASSF41	3.44E-71	0.1434502	0.054	0.008	8.29E-67	1.4	RASSF4	6.75
NFIX2	3.77E-71	0.3875848	0.154	0.056	9.09E-67	1.4	NFIX	2.75
UGGT21	3.80E-71	0.5124966	0.271	0.132	9.15E-67	1.4	UGGT2	2.053030303
LDLRAD41	6.79E-71	0.4580578	0.304	0.154	1.64E-66	1.4	LDLRAD4	1.974025974
LINC011522	1.02E-70	0.3367	0.142	0.049	2.46E-66	1.4	LINC01152	2.897959184
PGM11	1.24E-70	0.3053002	0.181	0.072	2.98E-66	1.4	PGM1	2.513888889
OAT2	2.66E-70	0.3868618	0.439	0.257	6.42E-66	1.4	OAT	1.708171206
MPZL13	1.11E-69	0.4176233	0.549	0.353	2.67E-65	1.4	MPZL1	1.555240793
TRMT10B1	1.39E-69	0.2885724	0.185	0.075	3.36E-65	1.4	TRMT10B	2.466666667
LYPLAL11	2.30E-69	0.2679125	0.129	0.043	5.54E-65	1.4	LYPLAL1	3
MIR3142HG1	3.56E-69	0.1290761	0.047	0.006	8.60E-65	1.4	MIR3142H(	7.833333333
RNF144B1	7.36E-69	0.5070054	0.44	0.269	1.77E-64	1.4	RNF144B	1.635687732
CASP41	7.81E-69	0.3707138	0.39	0.221	1.88E-64	1.4	CASP4	1.764705882
NHS	1.07E-68	0.3486015	0.302	0.152	2.58E-64	1.4	NHS	1.986842105
PNN3	1.18E-68	0.3601742	0.493	0.302	2.84E-64	1.4	PNN	1.632450331
PLPP3	1.66E-68	0.4740625	0.404	0.234	4.01E-64	1.4	PLPP3	1.726495726
RPL10A3	1.74E-68	0.4800633	0.81	0.706	4.19E-64	1.4	RPL10A	1.147308782
EIF4A13	3.10E-68	0.3705583	0.582	0.381	7.48E-64	1.4	EIF4A1	1.527559055
KCND22	6.22E-68	0.5332247	0.137	0.048	1.50E-63	1.4	KCND2	2.854166667
CLINT13	9.07E-68	0.3576504	0.404	0.229	2.19E-63	1.4	CLINT1	1.76419214
TPTEP12	1.03E-67	0.4030465	0.281	0.141	2.47E-63	1.4	TPTEP1	1.992907801
SLCO3A12	1.82E-67	0.4350565	0.319	0.169	4.39E-63	1.4	SLCO3A1	1.887573964
PBX1	4.50E-67	0.4458479	0.418	0.244	1.08E-62	1.4	PBX1	1.713114754
STRA6	1.71E-66	0.1097796	0.031	0.003	4.13E-62	1.4	STRA6	10.33333333
GBP31	2.12E-66	0.3839842	0.258	0.126	5.10E-62	1.4	GBP3	2.047619048
CLEC1A	6.17E-66	0.1620623	0.066	0.014	1.49E-61	1.4	CLEC1A	4.714285714
NIPAL31	1.00E-65	0.2979239	0.133	0.046	2.42E-61	1.4	NIPAL3	2.891304348
ETV63	2.77E-65	0.4075976	0.745	0.556	6.67E-61	1.4	ETV6	1.339928058
LIMK21	4.03E-65	0.3430806	0.203	0.089	9.72E-61	1.4	LIMK2	2.280898876
PUS101	6.76E-65	0.2460748	0.127	0.043	1.63E-60	1.4	PUS10	2.953488372
ETS13	9.69E-65	0.3109775	0.151	0.057	2.34E-60	1.4	ETS1	2.649122807
C1RL-AS11	1.54E-64	0.1816828	0.06	0.012	3.71E-60	1.4	C1RL-AS1	5
TMEM1233	2.50E-64	0.3771241	0.467	0.283	6.04E-60	1.4	TMEM123	1.650176678
CYYR12	3.34E-64	0.2525054	0.1	0.03	8.04E-60	1.4	CYYR1	3.333333333
ZNF33B1	3.52E-64	0.3302825	0.251	0.121	8.48E-60	1.4	ZNF33B	2.074380165
FBXO322	4.91E-64	0.7274184	0.519	0.351	1.18E-59	1.4	FBXO32	1.478632479
ENO13	7.12E-64	0.3996653	0.553	0.361	1.72E-59	1.4	ENO1	1.531855956
ITFG11	8.07E-64	0.4118537	0.426	0.254	1.95E-59	1.4	ITFG1	1.677165354
CDC42EP52	8.25E-64	0.1663905	0.061	0.012	1.99E-59	1.4	CDC42EP5	5.083333333
SLC26A4	1.13E-63	0.3204145	0.058	0.011	2.73E-59	1.4	SLC26A4	5.272727273
ODAM1	1.62E-63	0.3791549	0.048	0.008	3.91E-59	1.4	ODAM	6
FAM135A1	2.20E-63	0.30059	0.147	0.055	5.30E-59	1.4	FAM135A	2.672727273
NRG12	2.40E-63	0.4000078	0.17	0.069	5.80E-59	1.4	NRG1	2.463768116
FGFBP12	3.22E-63	0.3268063	0.098	0.028	7.76E-59	1.4	FGFBP1	3.5
HELB1	4.21E-63	0.3286727	0.203	0.09	1.01E-58	1.4	HELB	2.255555556
SLC25A271	4.48E-63	0.1686741	0.058	0.011	1.08E-58	1.4	SLC25A27	5.272727273
CASP13	4.73E-63	0.1953415	0.087	0.023	1.14E-58	1.4	CASP1	3.782608696
TGFBR33	4.76E-63	0.4234431	0.198	0.087	1.15E-58	1.4	TGFBR3	2.275862069
DDR22	5.15E-63	0.3532012	0.17	0.069	1.24E-58	1.4	DDR2	2.463768116

LRP22	5.43E-63	0.4578025	0.302	0.159	1.31E-58	1.4	LRP2	1.899371069
SPIDR1	7.56E-63	0.4282422	0.769	0.58	1.82E-58	1.4	SPIDR	1.325862069
OLFM41	9.03E-63	0.4334919	0.064	0.014	2.18E-58	1.4	OLFM4	4.571428571
FAM171A11	1.42E-62	0.2593602	0.112	0.036	3.41E-58	1.4	FAM171A1	3.111111111
TIMM23B1	1.53E-62	0.3781198	0.302	0.16	3.70E-58	1.4	TIMM23B	1.8875
SF3B32	2.03E-62	0.3557309	0.267	0.134	4.90E-58	1.4	SF3B3	1.992537313
RAD51B1	2.40E-62	0.3876908	0.373	0.214	5.79E-58	1.4	RAD51B	1.742990654
TRIM381	5.68E-62	0.3055849	0.195	0.086	1.37E-57	1.4	TRIM38	2.26744186
NRG31	8.01E-62	0.3539723	0.151	0.059	1.93E-57	1.4	NRG3	2.559322034
NTN12	8.79E-62	0.3320658	0.22	0.102	2.12E-57	1.4	NTN1	2.156862745
PTBP23	1.09E-61	0.3571532	0.509	0.323	2.64E-57	1.4	PTBP2	1.575851393
SLC15A11	1.48E-61	0.2021241	0.069	0.016	3.56E-57	1.4	SLC15A1	4.3125
IL12RB11	1.65E-61	0.170752	0.066	0.015	3.99E-57	1.4	IL12RB1	4.4
RP11-353M9.12	2.21E-61	0.1599436	0.045	0.007	5.32E-57	1.4	RP11-353M	6.428571429
RIF13	3.26E-61	0.3606487	0.365	0.208	7.86E-57	1.4	RIF1	1.754807692
CFLAR-AS12	3.33E-61	0.2481524	0.122	0.042	8.02E-57	1.4	CFLAR-AS1	2.904761905
VRK21	3.36E-61	0.3255748	0.251	0.122	8.10E-57	1.4	VRK2	2.057377049
PLCB41	3.95E-61	0.3233952	0.192	0.084	9.52E-57	1.4	PLCB4	2.285714286
TPD52L12	4.30E-61	0.3774022	0.256	0.128	1.04E-56	1.4	TPD52L1	2
NCOA22	5.08E-61	0.4167803	0.599	0.414	1.22E-56	1.4	NCOA2	1.446859903
ALPL2	6.45E-61	0.2465103	0.085	0.023	1.56E-56	1.4	ALPL	3.695652174
PTK2B2	7.58E-61	0.2197855	0.096	0.029	1.83E-56	1.4	PTK2B	3.310344828
CYP24A12	1.01E-60	0.3643101	0.221	0.102	2.42E-56	1.4	CYP24A1	2.166666667
SPP11	1.10E-60	0.5761622	0.05	0.009	2.65E-56	1.4	SPP1	5.555555556
GSDMC1	1.29E-60	0.158755	0.064	0.014	3.12E-56	1.4	GSDMC	4.571428571
RP11-93K22.61	2.97E-60	0.1250437	0.047	0.007	7.17E-56	1.4	RP11-93K2	6.714285714
UGT2B72	3.28E-60	0.1718304	0.074	0.018	7.92E-56	1.4	UGT2B7	4.111111111
ADARB11	7.96E-60	0.2103931	0.094	0.028	1.92E-55	1.4	ADARB1	3.357142857
SRPK12	1.02E-59	0.3968998	0.648	0.466	2.46E-55	1.4	SRPK1	1.39055794
SIRPA1	1.26E-59	0.2153392	0.094	0.028	3.03E-55	1.4	SIRPA	3.357142857
MAGED11	1.44E-59	0.3002418	0.181	0.078	3.47E-55	1.4	MAGED1	2.320512821
S100A71	2.92E-59	0.9222163	0.054	0.01	7.05E-55	1.4	S100A7	5.4
SCGB2B21	3.14E-59	0.2437449	0.129	0.046	7.56E-55	1.4	SCGB2B2	2.804347826
EIF4E22	4.74E-59	0.3515905	0.481	0.303	1.14E-54	1.4	EIF4E2	1.587458746
NCK12	7.01E-59	0.3007041	0.207	0.095	1.69E-54	1.4	NCK1	2.178947368
ITGB41	1.23E-58	0.2822241	0.1	0.031	2.96E-54	1.4	ITGB4	3.225806452
RP11-66B24.42	1.57E-58	0.2468475	0.102	0.032	3.79E-54	1.4	RP11-66B2	3.1875
UBA523	2.27E-58	0.2703943	0.957	0.914	5.48E-54	1.4	UBA52	1.047045952
B4GALT61	2.56E-58	0.190888	0.071	0.017	6.17E-54	1.4	B4GALT6	4.176470588
RP4-765H13.12	2.87E-58	0.1859568	0.063	0.014	6.92E-54	1.4	RP4-765H1	4.5
UAP12	4.69E-58	0.2852258	0.26	0.131	1.13E-53	1.4	UAP1	1.984732824
MID13	5.09E-58	0.3362884	0.142	0.055	1.23E-53	1.4	MID1	2.581818182
SEL1L32	5.73E-58	0.2064407	0.086	0.025	1.38E-53	1.4	SEL1L3	3.44
MAP4K42	6.26E-58	0.3341209	0.377	0.218	1.51E-53	1.4	MAP4K4	1.729357798
PRKX2	2.07E-57	0.2743407	0.198	0.09	4.98E-53	1.4	PRKX	2.2
PARP141	2.29E-57	0.3505255	0.244	0.122	5.53E-53	1.4	PARP14	2
C5orf561	2.71E-57	0.29628	0.181	0.079	6.53E-53	1.4	C5orf56	2.291139241
PLEKHA51	3.06E-57	0.3328698	0.56	0.372	7.37E-53	1.4	PLEKHA5	1.505376344
MAPK141	3.34E-57	0.3509406	0.27	0.141	8.06E-53	1.4	MAPK14	1.914893617
INVS1	3.49E-57	0.3384838	0.195	0.089	8.42E-53	1.4	INVS	2.191011236
PARK2	5.05E-57	0.3874366	0.305	0.167	1.22E-52	1.4	PARK2	1.826347305
LRRC492	5.20E-57	0.2881896	0.165	0.069	1.25E-52	1.4	LRRC49	2.391304348

KIAA0319L1	5.52E-57	0.3425639	0.269	0.139	1.33E-52	1.4	KIAA0319L	1.935251799
RASGEF1A1	5.82E-57	0.212268	0.082	0.023	1.40E-52	1.4	RASGEF1A	3.565217391
RARS3	7.89E-57	0.3552969	0.356	0.205	1.90E-52	1.4	RARS	1.736585366
FZD71	9.87E-57	0.2114087	0.099	0.032	2.38E-52	1.4	FZD7	3.09375
ESD3	1.95E-56	0.332334	0.469	0.295	4.70E-52	1.4	ESD	1.589830508
RNASEH2B1	2.12E-56	0.1788133	0.085	0.024	5.12E-52	1.4	RNASEH2B	3.541666667
RGS22	2.13E-56	0.4583129	0.233	0.115	5.14E-52	1.4	RGS2	2.026086957
HLA-B3	2.47E-56	0.3799826	0.703	0.522	5.96E-52	1.4	HLA-B	1.346743295
WIPI11	3.29E-56	0.323767	0.257	0.132	7.94E-52	1.4	WIPI1	1.946969697
ECHDC11	3.70E-56	0.3689737	0.333	0.191	8.93E-52	1.4	ECHDC1	1.743455497
ANKRD63	4.56E-56	0.2504346	0.118	0.042	1.10E-51	1.4	ANKRD6	2.80952381
CPAMD83	6.07E-56	0.2831136	0.14	0.055	1.46E-51	1.4	CPAMD8	2.545454545
MINK12	8.17E-56	0.3319938	0.191	0.088	1.97E-51	1.4	MINK1	2.170454545
EMC22	8.91E-56	0.270947	0.263	0.134	2.15E-51	1.4	EMC2	1.962686567
LRRC8B2	9.21E-56	0.2289987	0.203	0.093	2.22E-51	1.4	LRRC8B	2.182795699
TMEM1593	1.26E-55	0.3355125	0.463	0.292	3.03E-51	1.4	TMEM159	1.585616438
TCEAL83	1.47E-55	0.3536378	0.369	0.217	3.55E-51	1.4	TCEAL8	1.700460829
LINC008532	1.57E-55	0.1482876	0.056	0.012	3.79E-51	1.4	LINC00853	4.666666667
MTERF41	1.72E-55	0.2333817	0.133	0.051	4.15E-51	1.4	MTERF4	2.607843137
CCDC1221	1.74E-55	0.2022801	0.097	0.031	4.20E-51	1.4	CCDC122	3.129032258
GLTP2	2.04E-55	0.3115026	0.238	0.118	4.93E-51	1.4	GLTP	2.016949153
MT-CYB3	2.33E-55	0.2134555	0.985	0.992	5.62E-51	1.4	MT-CYB	0.992943548
KRIT11	2.83E-55	0.2903733	0.214	0.102	6.82E-51	1.4	KRIT1	2.098039216
IGF2BP23	3.39E-55	0.1566084	0.431	0.252	8.16E-51	1.4	IGF2BP2	1.71031746
PRKCE1	4.28E-55	0.4596908	0.32	0.182	1.03E-50	1.4	PRKCE	1.758241758
ARHGEF10L3	4.54E-55	0.3691101	0.284	0.153	1.09E-50	1.4	ARHGEF10I	1.85620915
SUPT3H2	5.83E-55	0.3382604	0.208	0.099	1.41E-50	1.4	SUPT3H	2.101010101
PDE1C2	6.46E-55	0.2898447	0.103	0.034	1.56E-50	1.4	PDE1C	3.029411765
THAP61	7.32E-55	0.2230162	0.126	0.047	1.77E-50	1.4	THAP6	2.680851064
HIBCH1	7.66E-55	0.299642	0.209	0.099	1.85E-50	1.4	HIBCH	2.111111111
RPS83	1.05E-54	0.4312751	0.819	0.728	2.52E-50	1.4	RPS8	1.125
H6PD1	1.06E-54	0.2156167	0.098	0.031	2.55E-50	1.4	H6PD	3.161290323
DOPEY11	1.06E-54	0.2623901	0.17	0.074	2.55E-50	1.4	DOPEY1	2.297297297
RNF2131	1.64E-54	0.3255628	0.302	0.165	3.96E-50	1.4	RNF213	1.83030303
SEPP11	2.34E-54	0.2681457	0.468	0.293	5.63E-50	1.4	SEPP1	1.597269625
SAMD9L	2.49E-54	0.1713509	0.063	0.015	6.01E-50	1.4	SAMD9L	4.2
EIF3M3	2.70E-54	0.2876738	0.492	0.312	6.51E-50	1.4	EIF3M	1.576923077
PIBF11	2.86E-54	0.3453606	0.294	0.161	6.91E-50	1.4	PIBF1	1.826086957
ZNF37A1	3.27E-54	0.2471699	0.141	0.056	7.88E-50	1.4	ZNF37A	2.517857143
NOD23	3.31E-54	0.2136968	0.1	0.033	7.98E-50	1.4	NOD2	3.03030303
KIAA0226L3	4.27E-54	0.1943608	0.118	0.042	1.03E-49	1.4	KIAA0226L	2.80952381
CARD163	4.55E-54	0.212239	0.109	0.037	1.10E-49	1.4	CARD16	2.945945946
RNF1701	6.24E-54	0.2056883	0.098	0.032	1.50E-49	1.4	RNF170	3.0625
B3GALT53	6.51E-54	0.2048741	0.077	0.021	1.57E-49	1.4	B3GALT5	3.666666667
PFDN53	1.01E-53	0.2766171	0.901	0.839	2.44E-49	1.4	PFDN5	1.073897497
C2CD21	1.15E-53	0.1732644	0.077	0.021	2.78E-49	1.4	C2CD2	3.666666667
CLDN81	1.34E-53	0.2262119	0.129	0.049	3.23E-49	1.4	CLDN8	2.632653061
ZNF3971	1.37E-53	0.2699316	0.184	0.083	3.31E-49	1.4	ZNF397	2.21686747
SPARCL13	1.66E-53	0.1803595	0.099	0.032	4.01E-49	1.4	SPARCL1	3.09375
LCN21	1.70E-53	0.2614317	0.056	0.012	4.10E-49	1.4	LCN2	4.666666667
RP4-678D15.12	1.80E-53	0.2897461	0.167	0.072	4.34E-49	1.4	RP4-678D1	2.319444444
AMBRA12	3.03E-53	0.4936044	0.776	0.665	7.32E-49	1.4	AMBRA1	1.166917293

TRMT113	3.94E-53	0.2786225	0.191	0.089	9.50E-49	1.4	TRMT11	2.146067416
BARX23	5.39E-53	0.4203671	0.383	0.234	1.30E-48	1.4	BARX2	1.636752137
CSF3R	6.02E-53	0.2026405	0.094	0.03	1.45E-48	1.4	CSF3R	3.133333333
RP11-692D12.11	6.22E-53	0.200156	0.069	0.018	1.50E-48	1.4	RP11-692D	3.833333333
SHPRH1	6.94E-53	0.2823082	0.177	0.079	1.67E-48	1.4	SHPRH	2.240506329
ADGRA32	7.72E-53	0.248226	0.163	0.07	1.86E-48	1.4	ADGRA3	2.328571429
EIF2B52	1.35E-52	0.4216233	0.602	0.444	3.25E-48	1.4	EIF2B5	1.355855856
C9orf722	1.63E-52	0.2241603	0.126	0.048	3.93E-48	1.4	C9orf72	2.625
GLYATL21	1.90E-52	0.1993731	0.11	0.039	4.58E-48	1.4	GLYATL2	2.820512821
MLH31	2.89E-52	0.2527111	0.15	0.063	6.97E-48	1.4	MLH3	2.380952381
ZNF780B1	4.42E-52	0.1680587	0.077	0.022	1.07E-47	1.4	ZNF780B	3.5
ASCC11	6.32E-52	0.2961604	0.248	0.129	1.52E-47	1.4	ASCC1	1.92248062
SDC42	6.63E-52	0.4020208	0.475	0.311	1.60E-47	1.4	SDC4	1.52733119
SEC22A1	6.67E-52	0.3011433	0.219	0.108	1.61E-47	1.4	SEC22A	2.027777778
RP11-367G18.1	9.28E-52	0.1260213	0.043	0.007	2.24E-47	1.4	RP11-367G	6.142857143
LITAF2	1.01E-51	0.3663415	0.592	0.419	2.44E-47	1.4	LITAF	1.412887828
EML43	1.14E-51	0.3117449	0.279	0.151	2.75E-47	1.4	EML4	1.847682119
ZNF618	1.39E-51	0.1412389	0.056	0.013	3.35E-47	1.4	ZNF618	4.307692308
MCC1	1.87E-51	0.3026446	0.197	0.094	4.50E-47	1.4	MCC	2.095744681
TNFAIP82	2.00E-51	0.4929942	0.533	0.368	4.82E-47	1.4	TNFAIP8	1.448369565
ZNF7081	2.13E-51	0.3578858	0.142	0.058	5.14E-47	1.4	ZNF708	2.448275862
STIL2	2.37E-51	0.2369225	0.115	0.042	5.70E-47	1.4	STIL	2.738095238
VPS541	2.42E-51	0.3401194	0.308	0.174	5.84E-47	1.4	VPS54	1.770114943
RP11-115D19.1	4.09E-51	0.2414425	0.077	0.022	9.87E-47	1.4	RP11-115D	3.5
CREB3L21	4.68E-51	0.3029698	0.164	0.072	1.13E-46	1.4	CREB3L2	2.277777778
PAK33	4.82E-51	0.2895933	0.106	0.037	1.16E-46	1.4	PAK3	2.864864865
HUWE12	6.74E-51	0.3267855	0.413	0.256	1.63E-46	1.4	HUWE1	1.61328125
ZBTB201	7.37E-51	0.3570739	0.691	0.501	1.78E-46	1.4	ZBTB20	1.379241517
AC005042.42	1.24E-50	0.2580779	0.101	0.035	2.99E-46	1.4	AC005042.4	2.885714286
COX7A2L3	1.90E-50	0.3112033	0.655	0.479	4.57E-46	1.4	COX7A2L	1.36743215
ATRNL11	2.29E-50	0.3012942	0.114	0.042	5.52E-46	1.4	ATRNL1	2.714285714
TBX193	2.42E-50	0.250054	0.127	0.05	5.84E-46	1.4	TBX19	2.54
PML2	3.00E-50	0.2628776	0.16	0.07	7.24E-46	1.4	PML	2.285714286
EIF3K3	3.52E-50	0.2920766	0.484	0.312	8.48E-46	1.4	EIF3K	1.551282051
ACO11	3.76E-50	0.2040381	0.094	0.031	9.08E-46	1.4	ACO1	3.032258065
VWA5A1	6.78E-50	0.2359178	0.17	0.077	1.63E-45	1.4	VWA5A	2.207792208
LRRC75A3	8.46E-50	0.3479629	0.239	0.125	2.04E-45	1.4	LRRC75A	1.912
FBL3	1.04E-49	0.2471316	0.238	0.122	2.50E-45	1.4	FBL	1.950819672
ARHGEF91	1.33E-49	0.1862522	0.087	0.028	3.20E-45	1.4	ARHGEF9	3.107142857
TFCP21	1.55E-49	0.3057911	0.203	0.099	3.73E-45	1.4	TFCP2	2.050505051
TYW51	1.72E-49	0.2181144	0.117	0.044	4.14E-45	1.4	TYW5	2.659090909
RP5-896L10.13	2.33E-49	0.2791623	0.362	0.213	5.63E-45	1.4	RP5-896L1(	1.699530516
ZNF280D1	2.40E-49	0.2741617	0.174	0.08	5.80E-45	1.4	ZNF280D	2.175
CDKAL11	2.60E-49	0.3689552	0.368	0.224	6.27E-45	1.4	CDKAL1	1.642857143
PLEKHM31	2.98E-49	0.1767369	0.082	0.025	7.18E-45	1.4	PLEKHM3	3.28
ZKSCAN1	3.16E-49	0.3405741	0.388	0.239	7.62E-45	1.4	ZKSCAN1	1.623430962
RPL273	3.17E-49	0.2500373	0.948	0.904	7.64E-45	1.4	RPL27	1.048672566
NSMCE11	3.27E-49	0.237118	0.172	0.078	7.89E-45	1.4	NSMCE1	2.205128205
CLEC2B3	3.94E-49	0.222936	0.136	0.055	9.51E-45	1.4	CLEC2B	2.472727273
ATXN31	1.26E-48	0.3086858	0.247	0.131	3.04E-44	1.4	ATXN3	1.885496183
AC026167.1	1.46E-48	0.10023	0.033	0.004	3.51E-44	1.4	AC026167.	8.25
SMIM82	1.63E-48	0.2343329	0.126	0.05	3.94E-44	1.4	SMIM8	2.52

CIR11	2.15E-48	0.3073192	0.39	0.24	5.19E-44	1.4	CIR1	1.625
DPYSL21	2.35E-48	0.2939897	0.215	0.109	5.66E-44	1.4	DPYSL2	1.972477064
RPL103	3.43E-48	0.5515124	0.954	0.933	8.26E-44	1.4	RPL10	1.022508039
SOS11	3.71E-48	0.3852825	0.516	0.352	8.94E-44	1.4	SOS1	1.465909091
ROR11	3.77E-48	0.2435133	0.069	0.019	9.09E-44	1.4	ROR1	3.631578947
XPNPEP12	5.19E-48	0.2482083	0.162	0.073	1.25E-43	1.4	XPNPEP1	2.219178082
RP1-122P22.21	5.82E-48	0.1899243	0.067	0.018	1.40E-43	1.4	RP1-122P2	3.722222222
ADGRF11	5.85E-48	0.2027812	0.085	0.027	1.41E-43	1.4	ADGRF1	3.148148148
PDZD23	6.49E-48	0.357329	0.618	0.454	1.56E-43	1.4	PDZD2	1.36123348
PLA2G4C1	1.13E-47	0.2241227	0.106	0.039	2.74E-43	1.4	PLA2G4C	2.717948718
GAB11	1.29E-47	0.542115	0.515	0.37	3.10E-43	1.4	GAB1	1.391891892
RP11-304M2.32	1.45E-47	0.1989947	0.086	0.028	3.51E-43	1.4	RP11-304M	3.071428571
ATF7IP1	1.92E-47	0.3469232	0.287	0.162	4.62E-43	1.4	ATF7IP	1.771604938
CALCOCO23	2.12E-47	0.2995158	0.382	0.236	5.10E-43	1.4	CALCOCO2	1.618644068
PHF141	2.19E-47	0.329998	0.22	0.114	5.28E-43	1.4	PHF14	1.929824561
TMEM176A2	2.22E-47	0.1973615	0.081	0.025	5.36E-43	1.4	TMEM176A	3.24
SESTD13	2.24E-47	0.3168079	0.654	0.467	5.40E-43	1.4	SESTD1	1.400428266
WBP2NL2	2.57E-47	0.1856203	0.088	0.029	6.20E-43	1.4	WBP2NL	3.034482759
KCNK52	2.63E-47	0.1708934	0.054	0.013	6.34E-43	1.4	KCNK5	4.153846154
FIGN1	2.87E-47	0.3153648	0.191	0.093	6.93E-43	1.4	FIGN	2.053763441
WWOX1	3.62E-47	0.5949524	0.352	0.222	8.72E-43	1.4	WWOX	1.585585586
CDK63	4.13E-47	0.2475451	0.228	0.118	9.96E-43	1.4	CDK6	1.93220339
NLGN4X2	4.70E-47	0.2070232	0.105	0.039	1.13E-42	1.4	NLGN4X	2.692307692
CDC42EP12	5.39E-47	0.2601783	0.088	0.029	1.30E-42	1.4	CDC42EP1	3.034482759
CRBN3	5.82E-47	0.2541195	0.247	0.132	1.40E-42	1.4	CRBN	1.871212121
ZNF5441	5.84E-47	0.2416867	0.166	0.076	1.41E-42	1.4	ZNF544	2.184210526
SPG111	6.57E-47	0.3571826	0.433	0.279	1.58E-42	1.4	SPG11	1.551971326
HOMER21	7.33E-47	0.3776794	0.277	0.156	1.77E-42	1.4	HOMER2	1.775641026
CTA-292E10.61	7.47E-47	0.2509965	0.17	0.078	1.80E-42	1.4	CTA-292E1	2.179487179
RSL1D13	7.90E-47	0.2412508	0.621	0.431	1.91E-42	1.4	RSL1D1	1.440835267
ATM1	8.91E-47	0.2555837	0.137	0.058	2.15E-42	1.4	ATM	2.362068966
RBMX1	1.24E-46	0.2941412	0.31	0.18	2.99E-42	1.4	RBMX	1.722222222
RABGAP11	1.25E-46	0.3142186	0.41	0.261	3.01E-42	1.4	RABGAP1	1.570881226
GABRE2	1.37E-46	0.1597987	0.079	0.025	3.30E-42	1.4	GABRE	3.16
H1F01	1.42E-46	0.3216465	0.264	0.147	3.43E-42	1.4	H1F0	1.795918367
SLC25A12	1.61E-46	0.2753123	0.161	0.073	3.87E-42	1.4	SLC25A12	2.205479452
DNER1	1.97E-46	0.2866596	0.103	0.038	4.75E-42	1.4	DNER	2.710526316
SLC35E31	2.86E-46	0.2601398	0.155	0.069	6.90E-42	1.4	SLC35E3	2.246376812
ABCA5	3.90E-46	0.2649402	0.218	0.112	9.39E-42	1.4	ABCA5	1.946428571
NBN1	6.67E-46	0.2095996	0.115	0.045	1.61E-41	1.4	NBN	2.555555556
OGFRL12	7.79E-46	0.2881729	0.298	0.172	1.88E-41	1.4	OGFRL1	1.73255814
LINC011911	8.41E-46	0.1684485	0.094	0.033	2.03E-41	1.4	LINC01191	2.848484848
CTSB1	1.48E-45	0.3827405	0.419	0.275	3.57E-41	1.4	CTSB	1.523636364
IRF22	1.55E-45	0.2889035	0.356	0.217	3.74E-41	1.4	IRF2	1.640552995
PSMB81	2.03E-45	0.2272882	0.155	0.07	4.89E-41	1.4	PSMB8	2.214285714
LRRC37A31	2.15E-45	0.2448407	0.201	0.1	5.20E-41	1.4	LRRC37A3	2.01
USP391	2.56E-45	0.2729809	0.484	0.319	6.18E-41	1.4	USP39	1.517241379
C2	3.34E-45	0.1486913	0.065	0.018	8.05E-41	1.4	C2	3.611111111
CCDC661	3.71E-45	0.2688376	0.235	0.125	8.94E-41	1.4	CCDC66	1.88
CEBPD1	5.45E-45	0.2883302	0.3	0.174	1.31E-40	1.4	CEBPD	1.724137931
STRBP1	6.34E-45	0.2989524	0.258	0.143	1.53E-40	1.4	STRBP	1.804195804
RAP1GAP21	6.71E-45	0.3588663	0.24	0.13	1.62E-40	1.4	RAP1GAP2	1.846153846

ITSN21	7.90E-45	0.3298535	0.412	0.264	1.91E-40	1.4	ITSN2	1.560606061
SYCP31	8.60E-45	0.1705718	0.058	0.015	2.07E-40	1.4	SYCP3	3.866666667
RPS193	8.73E-45	0.8093295	0.294	0.173	2.10E-40	1.4	RPS19	1.699421965
RHOXF1-AS11	9.06E-45	0.1156608	0.043	0.009	2.19E-40	1.4	RHOXF1-AS	4.777777778
DDX60	9.27E-45	0.1512394	0.055	0.014	2.23E-40	1.4	DDX60	3.928571429
NDRG23	1.09E-44	0.34099	0.294	0.171	2.64E-40	1.4	NDRG2	1.719298246
SLC5A12	2.15E-44	0.2033941	0.113	0.044	5.18E-40	1.4	SLC5A1	2.568181818
RP11-759A24.3	2.15E-44	0.1279199	0.072	0.022	5.19E-40	1.4	RP11-759A	3.272727273
MUC151	2.36E-44	0.1463408	0.062	0.017	5.68E-40	1.4	MUC15	3.647058824
HSD17B4	2.58E-44	0.3132191	0.284	0.163	6.22E-40	1.4	HSD17B4	1.742331288
NCMAP3	2.62E-44	0.2181677	0.1	0.037	6.32E-40	1.4	NCMAP	2.702702703
CEP2951	2.82E-44	0.1783152	0.105	0.039	6.80E-40	1.4	CEP295	2.692307692
DLEU11	3.17E-44	0.2339119	0.226	0.118	7.64E-40	1.4	DLEU1	1.915254237
RP11-796E2.41	3.17E-44	0.1546117	0.057	0.015	7.64E-40	1.4	RP11-796E	3.8
HAPLN32	3.26E-44	0.1730176	0.056	0.014	7.85E-40	1.4	HAPLN3	4
ZFYVE91	3.37E-44	0.2443995	0.158	0.073	8.12E-40	1.4	ZFYVE9	2.164383562
DTWD11	3.95E-44	0.2258985	0.13	0.055	9.51E-40	1.4	DTWD1	2.363636364
ZBED51	5.22E-44	0.2232625	0.125	0.052	1.26E-39	1.4	ZBED5	2.403846154
ATP5F12	8.98E-44	0.2908184	0.515	0.356	2.16E-39	1.4	ATP5F1	1.446629213
FAM120C1	1.01E-43	0.208501	0.095	0.034	2.44E-39	1.4	FAM120C	2.794117647
MICU11	1.30E-43	0.3142903	0.307	0.181	3.14E-39	1.4	MICU1	1.696132597
FUT91	1.73E-43	0.1468712	0.055	0.014	4.16E-39	1.4	FUT9	3.928571429
SLC23A22	1.90E-43	0.1865179	0.088	0.031	4.57E-39	1.4	SLC23A2	2.838709677
G0S22	1.92E-43	0.138906	0.063	0.017	4.63E-39	1.4	G0S2	3.705882353
LSM52	1.99E-43	0.2669064	0.495	0.328	4.81E-39	1.4	LSM5	1.509146341
NIFK-AS11	2.04E-43	0.1665882	0.079	0.026	4.92E-39	1.4	NIFK-AS1	3.038461538
UQCRHL3	2.49E-43	0.2586223	0.293	0.17	6.01E-39	1.4	UQCRHL	1.723529412
CD471	2.93E-43	0.3660953	0.561	0.397	7.06E-39	1.4	CD47	1.413098237
PDE7A1	3.13E-43	0.3992215	0.357	0.226	7.54E-39	1.4	PDE7A	1.579646018
SAV12	3.57E-43	0.372064	0.421	0.28	8.61E-39	1.4	SAV1	1.503571429
MFSD14C2	3.98E-43	0.2736003	0.275	0.157	9.59E-39	1.4	MFSD14C	1.751592357
POU2F32	4.12E-43	0.306441	0.2	0.103	9.94E-39	1.4	POU2F3	1.941747573
ENKUR1	5.71E-43	0.1091524	0.042	0.009	1.38E-38	1.4	ENKUR	4.666666667
SGCE1	6.58E-43	0.2070159	0.097	0.036	1.59E-38	1.4	SGCE	2.694444444
NR2F21	7.18E-43	0.2112014	0.157	0.073	1.73E-38	1.4	NR2F2	2.150684932
NR3C22	1.02E-42	0.2660648	0.159	0.075	2.45E-38	1.4	NR3C2	2.12
ALDH9A1	1.37E-42	0.2137916	0.146	0.065	3.31E-38	1.4	ALDH9A1	2.246153846
TNFSF151	1.99E-42	0.1437386	0.054	0.014	4.79E-38	1.4	TNFSF15	3.857142857
CCAR11	2.00E-42	0.2958738	0.361	0.227	4.81E-38	1.4	CCAR1	1.59030837
FIBIN1	2.08E-42	0.1007818	0.031	0.005	5.01E-38	1.4	FIBIN	6.2
ACP13	2.34E-42	0.2867181	0.442	0.293	5.64E-38	1.4	ACP1	1.508532423
EIF2S33	2.92E-42	0.284957	0.424	0.276	7.04E-38	1.4	EIF2S3	1.536231884
RFX3-AS11	3.00E-42	0.2479958	0.126	0.054	7.23E-38	1.4	RFX3-AS1	2.333333333
RPS6KA51	3.02E-42	0.3278931	0.335	0.206	7.27E-38	1.4	RPS6KA5	1.626213592
DTX22	3.12E-42	0.2275034	0.135	0.059	7.53E-38	1.4	DTX2	2.288135593
NCALD2	3.29E-42	0.1854853	0.223	0.117	7.92E-38	1.4	NCALD	1.905982906
STEAP31	3.32E-42	0.1876625	0.071	0.022	8.00E-38	1.4	STEAP3	3.227272727
CRYBG33	3.50E-42	0.1927522	0.102	0.039	8.45E-38	1.4	CRYBG3	2.615384615
NEMF2	3.66E-42	0.2953348	0.39	0.249	8.82E-38	1.4	NEMF	1.56626506
PTCH11	3.69E-42	0.1187848	0.038	0.007	8.90E-38	1.4	PTCH1	5.428571429
KRCC11	3.88E-42	0.1924717	0.119	0.049	9.35E-38	1.4	KRCC1	2.428571429
ABCB101	3.89E-42	0.1544907	0.081	0.027	9.37E-38	1.4	ABCB10	3

ERCC6L21	4.08E-42	0.2394411	0.139	0.062	9.83E-38	1.4	ERCC6L2	2.241935484
ZNF1331	4.14E-42	0.1414462	0.067	0.02	9.99E-38	1.4	ZNF133	3.35
NSRP12	4.60E-42	0.2662613	0.326	0.196	1.11E-37	1.4	NSRP1	1.663265306
HINT12	6.04E-42	0.3101607	0.721	0.587	1.46E-37	1.4	HINT1	1.228279387
CHSY3	6.90E-42	0.1932706	0.045	0.01	1.66E-37	1.4	CHSY3	4.5
ZER11	8.12E-42	0.2499287	0.161	0.077	1.96E-37	1.4	ZER1	2.090909091
ROR22	8.49E-42	0.268198	0.162	0.078	2.05E-37	1.4	ROR2	2.076923077
RALGDS1	1.17E-41	0.1910785	0.111	0.044	2.82E-37	1.4	RALGDS	2.522727273
HSPG22	1.22E-41	0.2514699	0.155	0.073	2.94E-37	1.4	HSPG2	2.123287671
RP5-1180E21.52	1.26E-41	0.1550907	0.064	0.019	3.03E-37	1.4	RP5-1180E2	3.368421053
NAP1L13	1.27E-41	0.2905794	0.495	0.341	3.05E-37	1.4	NAP1L1	1.451612903
PTAR11	2.18E-41	0.2308463	0.14	0.063	5.25E-37	1.4	PTAR1	2.222222222
TGM21	2.56E-41	0.1910834	0.179	0.088	6.17E-37	1.4	TGM2	2.034090909
LINC006231	2.95E-41	0.1714238	0.073	0.023	7.12E-37	1.4	LINC00623	3.173913043
HNRNPA13	3.17E-41	0.1861082	0.879	0.743	7.64E-37	1.4	HNRNPA1	1.183041723
HPS53	3.28E-41	0.2569914	0.232	0.127	7.90E-37	1.4	HPS5	1.826771654
HAX12	3.52E-41	0.265609	0.378	0.24	8.48E-37	1.4	HAX1	1.575
SDCBP3	4.54E-41	0.2598848	0.764	0.599	1.09E-36	1.4	SDCBP	1.275459098
CTSC1	5.54E-41	0.1798518	0.084	0.029	1.34E-36	1.4	CTSC	2.896551724
CELF23	6.19E-41	0.1986548	0.214	0.113	1.49E-36	1.4	CELF2	1.89380531
AP000998.21	7.58E-41	0.1334413	0.028	0.004	1.83E-36	1.4	AP000998.2	7
SOS22	8.14E-41	0.3015264	0.567	0.408	1.96E-36	1.4	SOS2	1.389705882
TSFM1	8.24E-41	0.1848495	0.1	0.038	1.99E-36	1.4	TSFM	2.631578947
PLEKHA73	1.09E-40	0.1796005	0.979	0.98	2.64E-36	1.4	PLEKHA7	0.998979592
FBXL41	1.15E-40	0.2097321	0.136	0.061	2.76E-36	1.4	FBXL4	2.229508197
NOL43	1.58E-40	0.1874099	0.067	0.021	3.81E-36	1.4	NOL4	3.19047619
ACSL11	1.59E-40	0.3068418	0.316	0.191	3.84E-36	1.4	ACSL1	1.654450262
RBM411	2.24E-40	0.1803991	0.143	0.065	5.40E-36	1.4	RBM41	2.2
TBCK1	2.25E-40	0.2852736	0.209	0.112	5.42E-36	1.4	TBCK	1.866071429
MRPL452	2.90E-40	0.2435137	0.242	0.135	6.98E-36	1.4	MRPL45	1.792592593
PPFIBP21	3.02E-40	0.2510016	0.138	0.062	7.28E-36	1.4	PPFIBP2	2.225806452
ATXN71	3.16E-40	0.2367407	0.192	0.099	7.62E-36	1.4	ATXN7	1.939393939
FAM49A3	3.55E-40	0.1693286	0.077	0.026	8.56E-36	1.4	FAM49A	2.961538462
HOOK21	3.85E-40	0.2897768	0.254	0.144	9.27E-36	1.4	HOOK2	1.763888889
C14orf1591	5.53E-40	0.1891173	0.12	0.051	1.33E-35	1.4	C14orf159	2.352941176
EGFLAM1	6.22E-40	0.1575915	0.052	0.013	1.50E-35	1.4	EGFLAM	4
CARF	7.19E-40	0.1714051	0.074	0.024	1.73E-35	1.4	CARF	3.083333333
PLSCR41	7.38E-40	0.2268057	0.109	0.045	1.78E-35	1.4	PLSCR4	2.422222222
UBE2E31	7.70E-40	0.2981261	0.273	0.159	1.86E-35	1.4	UBE2E3	1.716981132
C9orf91	9.86E-40	0.1653075	0.073	0.024	2.38E-35	1.4	C9orf91	3.041666667
RPL123	1.09E-39	0.3070262	0.411	0.271	2.64E-35	1.4	RPL12	1.516605166
POLK1	1.29E-39	0.2360345	0.198	0.103	3.11E-35	1.4	POLK	1.922330097
LRCH12	1.43E-39	0.3565287	0.429	0.288	3.45E-35	1.4	LRCH1	1.489583333
SNX25	1.45E-39	0.2473983	0.226	0.125	3.49E-35	1.4	SNX2	1.808
ZNF6301	1.65E-39	0.1607243	0.063	0.019	3.98E-35	1.4	ZNF630	3.315789474
SSPN1	1.97E-39	0.1940256	0.076	0.026	4.76E-35	1.4	SSPN	2.923076923
PLEKHG13	2.31E-39	0.2492733	0.183	0.094	5.58E-35	1.4	PLEKHG1	1.946808511
KDM7A3	2.49E-39	0.349284	0.394	0.26	6.02E-35	1.4	KDM7A	1.515384615
METTL151	3.24E-39	0.2603919	0.216	0.117	7.82E-35	1.4	METTL15	1.846153846
RSRP11	3.52E-39	0.3176631	0.374	0.241	8.48E-35	1.4	RSRP1	1.55186722
EPB41L23	3.83E-39	0.1871524	0.196	0.102	9.23E-35	1.4	EPB41L2	1.921568627
TAF151	4.53E-39	0.3219402	0.418	0.278	1.09E-34	1.4	TAF15	1.503597122

HPRT12	5.26E-39	0.2102201	0.095	0.036	1.27E-34	1.4	HPRT1	2.638888889
CCT31	8.34E-39	0.2894806	0.493	0.344	2.01E-34	1.4	CCT3	1.433139535
LARP71	8.43E-39	0.2468792	0.25	0.143	2.03E-34	1.4	LARP7	1.748251748
PNPLA31	9.47E-39	0.1858946	0.075	0.026	2.28E-34	1.4	PNPLA3	2.884615385
BPTF1	9.66E-39	0.3040048	0.465	0.321	2.33E-34	1.4	BPTF	1.448598131
TMCC11	9.77E-39	0.3198456	0.371	0.239	2.35E-34	1.4	TMCC1	1.552301255
SNRPB23	1.16E-38	0.252638	0.481	0.327	2.79E-34	1.4	SNRPB2	1.470948012
SS181	1.21E-38	0.2408709	0.229	0.127	2.91E-34	1.4	SS18	1.803149606
MAP2K51	1.32E-38	0.2826789	0.218	0.119	3.17E-34	1.4	MAP2K5	1.831932773
SP11	1.37E-38	0.2419611	0.209	0.112	3.31E-34	1.4	SP1	1.866071429
TCF7L21	1.68E-38	0.3124879	0.525	0.365	4.06E-34	1.4	TCF7L2	1.438356164
OTUD6B-AS11	1.70E-38	0.2329856	0.274	0.16	4.09E-34	1.4	OTUD6B-A!	1.7125
GPM6B3	2.27E-38	0.2421478	0.211	0.114	5.48E-34	1.4	GPM6B	1.850877193
RSL24D13	2.33E-38	0.2350963	0.709	0.541	5.62E-34	1.4	RSL24D1	1.310536044
RP11-230B22.11	2.55E-38	0.1253912	0.052	0.014	6.14E-34	1.4	RP11-230B	3.714285714
CTD-2315E11.1	2.78E-38	0.122018	0.05	0.013	6.71E-34	1.4	CTD-2315E	3.846153846
RPS53	2.86E-38	0.5111827	0.333	0.213	6.90E-34	1.4	RPS5	1.563380282
APOL31	3.52E-38	0.1256759	0.052	0.014	8.48E-34	1.4	APOL3	3.714285714
LINC005111	3.70E-38	0.3639807	0.15	0.073	8.92E-34	1.4	LINC00511	2.054794521
RPL13A2	3.90E-38	0.4853086	0.607	0.463	9.41E-34	1.4	RPL13A	1.311015119
MCCC11	4.17E-38	0.2144821	0.155	0.075	1.01E-33	1.4	MCCC1	2.066666667
MFSD61	4.75E-38	0.2335847	0.135	0.062	1.15E-33	1.4	MFSD6	2.177419355
UQCRC23	4.86E-38	0.2407618	0.463	0.314	1.17E-33	1.4	UQCRC2	1.474522293
TCHP1	5.27E-38	0.2391349	0.07	0.023	1.27E-33	1.4	TCHP	3.043478261
HOXA32	6.23E-38	0.1383349	0.062	0.019	1.50E-33	1.4	HOXA3	3.263157895
CDC25B1	6.78E-38	0.230304	0.078	0.028	1.64E-33	1.4	CDC25B	2.785714286
XPO41	7.61E-38	0.222633	0.195	0.103	1.83E-33	1.4	XPO4	1.893203883
TAMM411	7.93E-38	0.1565768	0.076	0.026	1.91E-33	1.4	TAMM41	2.923076923
DPH51	8.09E-38	0.167373	0.117	0.05	1.95E-33	1.4	DPH5	2.34
HOTAIRM13	9.41E-38	0.1687673	0.081	0.029	2.27E-33	1.4	HOTAIRM1	2.793103448
WWC11	1.05E-37	0.3432592	0.555	0.403	2.53E-33	1.4	WWC1	1.377171216
GPATCH81	1.17E-37	0.2922461	0.36	0.231	2.82E-33	1.4	GPATCH8	1.558441558
ZNF8273	1.31E-37	0.2244044	0.196	0.104	3.17E-33	1.4	ZNF827	1.884615385
TLN22	1.67E-37	0.2309065	0.127	0.057	4.01E-33	1.4	TLN2	2.228070175
GBP4	1.99E-37	0.1233994	0.045	0.011	4.80E-33	1.4	GBP4	4.090909091
GRAMD21	2.00E-37	0.1086986	0.038	0.008	4.81E-33	1.4	GRAMD2	4.75
GNB2L12	2.16E-37	0.3766948	0.756	0.662	5.20E-33	1.4	GNB2L1	1.141993958
APTX1	2.24E-37	0.2433761	0.212	0.116	5.41E-33	1.4	APTX	1.827586207
LINC009692	2.75E-37	0.2426749	0.313	0.194	6.63E-33	1.4	LINC00969	1.613402062
TRIM28	3.31E-37	0.2503088	0.269	0.159	7.98E-33	1.4	TRIM2	1.691823899
BHLHE413	3.35E-37	0.2222072	0.097	0.039	8.07E-33	1.4	BHLHE41	2.487179487
RP11-793A3.22	4.15E-37	0.158739	0.056	0.016	1.00E-32	1.4	RP11-793A	3.5
C8orf593	4.17E-37	0.2606905	0.407	0.272	1.01E-32	1.4	C8orf59	1.496323529
LINC013892	4.19E-37	0.1383656	0.055	0.015	1.01E-32	1.4	LINC01389	3.666666667
FBXO91	6.01E-37	0.1804144	0.126	0.056	1.45E-32	1.4	FBXO9	2.25
MIS18BP11	6.57E-37	0.233957	0.186	0.097	1.58E-32	1.4	MIS18BP1	1.917525773
DHRS7B1	1.06E-36	0.2053439	0.123	0.055	2.56E-32	1.4	DHRS7B	2.236363636
GTF2IRD2B	1.14E-36	0.2278348	0.15	0.073	2.74E-32	1.4	GTF2IRD2B	2.054794521
DIRC21	1.28E-36	0.2119886	0.116	0.051	3.09E-32	1.4	DIRC2	2.274509804
TCN11	1.31E-36	0.1784916	0.055	0.016	3.15E-32	1.4	TCN1	3.4375
PARVA1	1.45E-36	0.2541838	0.206	0.113	3.49E-32	1.4	PARVA	1.82300885
RP11-514P8.6	2.23E-36	0.1397352	0.071	0.024	5.39E-32	1.4	RP11-514P.	2.958333333

PHKB1	2.38E-36	0.2696801	0.265	0.158	5.74E-32	1.4	PHKB	1.67721519
CHCHD33	2.47E-36	0.359896	0.885	0.831	5.96E-32	1.4	CHCHD3	1.064981949
BTN3A22	2.69E-36	0.1269489	0.06	0.018	6.49E-32	1.4	BTN3A2	3.333333333
ZFP141	2.78E-36	0.1792978	0.126	0.057	6.70E-32	1.4	ZFP14	2.210526316
PPA12	4.62E-36	0.2778366	0.32	0.201	1.11E-31	1.4	PPA1	1.592039801
CLEC2D1	5.83E-36	0.1982826	0.154	0.076	1.41E-31	1.4	CLEC2D	2.026315789
MNAT12	5.88E-36	0.2957299	0.391	0.262	1.42E-31	1.4	MNAT1	1.492366412
ORC31	6.00E-36	0.1963643	0.135	0.063	1.45E-31	1.4	ORC3	2.142857143
KB-1991G8.12	6.57E-36	0.1014668	0.038	0.008	1.59E-31	1.4	KB-1991G8	4.75
RFTN21	7.26E-36	0.168744	0.042	0.01	1.75E-31	1.4	RFTN2	4.2
PEX33	7.84E-36	0.2110772	0.103	0.043	1.89E-31	1.4	PEX3	2.395348837
YIPF41	8.77E-36	0.2135449	0.147	0.072	2.11E-31	1.4	YIPF4	2.041666667
RPL133	9.23E-36	0.3580428	0.543	0.404	2.22E-31	1.4	RPL13	1.344059406
LETMD11	9.59E-36	0.1859953	0.122	0.055	2.31E-31	1.4	LETMD1	2.218181818
POLR2F1	1.16E-35	0.2228754	0.115	0.051	2.79E-31	1.4	POLR2F	2.254901961
ADAMTS101	1.46E-35	0.1112346	0.038	0.008	3.53E-31	1.4	ADAMTS10	4.75
PDE9A2	1.52E-35	0.1933349	0.089	0.035	3.67E-31	1.4	PDE9A	2.542857143
MCEE	1.90E-35	0.1425202	0.069	0.023	4.59E-31	1.4	MCEE	3
CCDC71L1	2.03E-35	0.1380742	0.069	0.023	4.89E-31	1.4	CCDC71L	3
ASAP31	2.16E-35	0.132468	0.062	0.02	5.21E-31	1.4	ASAP3	3.1
CBR42	2.27E-35	0.2381112	0.347	0.221	5.48E-31	1.4	CBR4	1.570135747
FDPS1	2.79E-35	0.2515426	0.582	0.438	6.74E-31	1.4	FDPS	1.328767123
CDC261	2.89E-35	0.2347167	0.282	0.17	6.97E-31	1.4	CDC26	1.658823529
KARS3	3.96E-35	0.2537286	0.357	0.233	9.54E-31	1.4	KARS	1.532188841
CHD92	4.03E-35	0.2980693	0.532	0.386	9.71E-31	1.4	CHD9	1.378238342
THOC71	4.25E-35	0.1793879	0.144	0.07	1.02E-30	1.4	THOC7	2.057142857
LINC011371	5.93E-35	0.1833511	0.111	0.049	1.43E-30	1.4	LINC01137	2.265306122
MBP2	6.26E-35	0.208394	0.196	0.106	1.51E-30	1.4	MBP	1.849056604
DIS3L21	6.72E-35	0.2391497	0.169	0.088	1.62E-30	1.4	DIS3L2	1.920454545
CXCL162	6.74E-35	0.2342811	0.228	0.13	1.62E-30	1.4	CXCL16	1.753846154
CCDC125	7.21E-35	0.2104782	0.126	0.058	1.74E-30	1.4	CCDC125	2.172413793
GBAS1	7.76E-35	0.2317181	0.268	0.161	1.87E-30	1.4	GBAS	1.664596273
CCDC170	8.95E-35	0.2336947	0.113	0.05	2.16E-30	1.4	CCDC170	2.26
TMEM108	9.29E-35	0.1876842	0.042	0.011	2.24E-30	1.4	TMEM108	3.818181818
RP11-410L14.21	9.48E-35	0.1201186	0.052	0.015	2.29E-30	1.4	RP11-410L1	3.466666667
SH3BP52	9.70E-35	0.181032	0.111	0.049	2.34E-30	1.4	SH3BP5	2.265306122
COL22A12	1.08E-34	0.1411236	0.048	0.013	2.60E-30	1.4	COL22A1	3.692307692
PLRG11	1.09E-34	0.1918291	0.148	0.073	2.62E-30	1.4	PLRG1	2.02739726
UFL11	1.09E-34	0.1968244	0.107	0.046	2.63E-30	1.4	UFL1	2.326086957
TJP23	1.69E-34	0.2280188	0.45	0.306	4.08E-30	1.4	TJP2	1.470588235
RHPN22	1.70E-34	0.2522928	0.35	0.226	4.10E-30	1.4	RHPN2	1.548672566
ZNF7101	2.36E-34	0.2162448	0.161	0.083	5.70E-30	1.4	ZNF710	1.939759036
CH507-528H12.11	2.52E-34	0.129481	0.063	0.021	6.07E-30	1.4	CH507-528	3
SLC12A81	2.53E-34	0.1400282	0.072	0.025	6.10E-30	1.4	SLC12A8	2.88
GUCY1B31	2.94E-34	0.126529	0.047	0.013	7.09E-30	1.4	GUCY1B3	3.615384615
MPHOSPH81	3.61E-34	0.2332902	0.239	0.139	8.72E-30	1.4	MPHOSPH8	1.71942446
MDH12	4.23E-34	0.2855033	0.391	0.262	1.02E-29	1.4	MDH1	1.492366412
CADM11	5.44E-34	0.2545152	0.139	0.067	1.31E-29	1.4	CADM1	2.074626866
MRPS141	5.56E-34	0.1935722	0.214	0.12	1.34E-29	1.4	MRPS14	1.783333333
ZBTB332	6.20E-34	0.1266444	0.07	0.025	1.50E-29	1.4	ZBTB33	2.8
RPL310	7.96E-34	0.3974429	0.42	0.29	1.92E-29	1.4	RPL3	1.448275862
GLUL	8.06E-34	0.3848798	0.494	0.363	1.94E-29	1.4	GLUL	1.360881543

CTC-254B4.12	9.12E-34	0.134947	0.047	0.013	2.20E-29	1.4	CTC-254B4	3.615384615
RP1-167A14.2	1.30E-33	0.1986838	0.106	0.046	3.14E-29	1.4	RP1-167A1	2.304347826
MBOAT21	1.42E-33	0.222622	0.159	0.082	3.42E-29	1.4	MBOAT2	1.93902439
LRSAM11	1.61E-33	0.2217117	0.162	0.084	3.88E-29	1.4	LRSAM1	1.928571429
RNF243	1.61E-33	0.2582564	0.456	0.317	3.89E-29	1.4	RNF24	1.438485804
SNHG83	1.90E-33	0.2132673	0.335	0.213	4.59E-29	1.4	SNHG8	1.572769953
ITPKB2	2.07E-33	0.1636212	0.091	0.037	4.99E-29	1.4	ITPKB	2.459459459
SUGT11	2.28E-33	0.1979192	0.186	0.1	5.51E-29	1.4	SUGT1	1.86
RP11-666F17.11	2.31E-33	0.1312307	0.052	0.015	5.57E-29	1.4	RP11-666F:	3.466666667
TTC231	2.41E-33	0.1685553	0.102	0.044	5.81E-29	1.4	TTC23	2.318181818
TRAPPC111	2.75E-33	0.1778036	0.1	0.043	6.62E-29	1.4	TRAPPC11	2.325581395
ZNF2601	2.79E-33	0.1518037	0.138	0.067	6.73E-29	1.4	ZNF260	2.059701493
CARD141	2.98E-33	0.1390103	0.059	0.019	7.19E-29	1.4	CARD14	3.105263158
HSDL21	3.22E-33	0.2071167	0.151	0.077	7.76E-29	1.4	HSDL2	1.961038961
C15orf521	4.48E-33	0.1399649	0.064	0.022	1.08E-28	1.4	C15orf52	2.909090909
RP11-106M3.21	4.64E-33	0.1519358	0.076	0.028	1.12E-28	1.4	RP11-106M	2.714285714
MAP3K51	8.39E-33	0.2081118	0.502	0.35	2.02E-28	1.4	MAP3K5	1.434285714
RP11-572M11.41	1.05E-32	0.1078819	0.041	0.01	2.54E-28	1.4	RP11-572M	4.1
AL592183.11	1.14E-32	0.1997437	0.126	0.06	2.74E-28	1.4	AL592183.:	2.1
GPRIN31	1.22E-32	0.1258443	0.046	0.013	2.94E-28	1.4	GPRIN3	3.538461538
FAM172A3	1.28E-32	0.251803	0.737	0.603	3.08E-28	1.4	FAM172A	1.222222222
PHF21A1	1.32E-32	0.2891587	0.346	0.226	3.17E-28	1.4	PHF21A	1.530973451
LDLRAD31	1.38E-32	0.3415418	0.341	0.226	3.33E-28	1.4	LDLRAD3	1.508849558
MMP242	1.40E-32	0.1913293	0.153	0.078	3.38E-28	1.4	MMP24	1.961538462
MGST32	1.41E-32	0.2761603	0.555	0.409	3.40E-28	1.4	MGST3	1.356968215
GOSR11	1.51E-32	0.2053544	0.237	0.139	3.65E-28	1.4	GOSR1	1.705035971
GTF2IRD21	1.70E-32	0.2269021	0.142	0.071	4.11E-28	1.4	GTF2IRD2	2
RP11-711K1.81	1.92E-32	0.2266707	0.102	0.045	4.63E-28	1.4	RP11-711K	2.266666667
RAB30-AS11	3.39E-32	0.2121299	0.14	0.07	8.16E-28	1.4	RAB30-AS1	2
MTRNR2L14	3.85E-32	0.2122554	0.191	0.106	9.27E-28	1.4	MTRNR2L1	1.801886792
CCDC251	4.42E-32	0.1610953	0.164	0.085	1.07E-27	1.4	CCDC25	1.929411765
TTC39B1	4.78E-32	0.2045008	0.16	0.084	1.15E-27	1.4	TTC39B	1.904761905
GLIS33	4.82E-32	0.1964182	0.251	0.149	1.16E-27	1.4	GLIS3	1.684563758
CMTM82	5.59E-32	0.2817329	0.255	0.156	1.35E-27	1.4	CMTM8	1.634615385
DCLK21	6.45E-32	0.1522877	0.066	0.023	1.55E-27	1.4	DCLK2	2.869565217
MGST12	7.74E-32	0.2213093	0.909	0.854	1.87E-27	1.4	MGST1	1.06440281
OSBPL1A	7.98E-32	0.1312673	0.266	0.161	1.92E-27	1.4	OSBPL1A	1.652173913
PPM1L1	8.53E-32	0.2109791	0.136	0.067	2.06E-27	1.4	PPM1L	2.029850746
KIF1B3	1.25E-31	0.2315589	0.538	0.388	3.01E-27	1.4	KIF1B	1.386597938
PIK3IP11	1.42E-31	0.1548444	0.119	0.056	3.43E-27	1.4	PIK3IP1	2.125
LINC015541	1.63E-31	0.1011326	0.033	0.007	3.93E-27	1.4	LINC01554	4.714285714
EXT12	1.93E-31	0.2454286	0.622	0.482	4.66E-27	1.4	EXT1	1.290456432
KDM3B1	2.09E-31	0.2014544	0.246	0.147	5.05E-27	1.4	KDM3B	1.673469388
XYLT12	2.42E-31	0.261201	0.147	0.075	5.84E-27	1.4	XYLT1	1.96
DHCR241	2.54E-31	0.2269122	0.283	0.177	6.13E-27	1.4	DHCR24	1.598870056
GRIN2B	2.79E-31	0.1646964	0.061	0.021	6.72E-27	1.4	GRIN2B	2.904761905
TKT1	2.86E-31	0.188083	0.173	0.094	6.90E-27	1.4	TKT	1.840425532
TAF72	3.03E-31	0.2160352	0.188	0.105	7.30E-27	1.4	TAF7	1.79047619
ZNF433	3.16E-31	0.227306	0.213	0.124	7.63E-27	1.4	ZNF43	1.717741935
RP11-180I4.21	3.51E-31	0.1050271	0.041	0.011	8.47E-27	1.4	RP11-180I4	3.727272727
MSH31	3.58E-31	0.2887077	0.17	0.092	8.63E-27	1.4	MSH3	1.847826087
FOXC13	3.73E-31	0.1217846	0.081	0.032	8.99E-27	1.4	FOXC1	2.53125

MPPED21	3.77E-31	0.1148753	0.038	0.009	9.08E-27	1.4	MPPED2	4.222222222
PTPN143	3.99E-31	0.1439474	0.424	0.287	9.62E-27	1.4	PTPN14	1.477351916
CYP4A22-AS11	4.48E-31	0.111607	0.041	0.011	1.08E-26	1.4	CYP4A22-A	3.727272727
CUL51	4.62E-31	0.226927	0.195	0.111	1.12E-26	1.4	CUL5	1.756756757
GRHL21	4.70E-31	0.2337241	0.395	0.27	1.13E-26	1.4	GRHL2	1.462962963
FAM60A3	5.50E-31	0.2870071	0.422	0.296	1.33E-26	1.4	FAM60A	1.425675676
QRSL11	5.58E-31	0.1601469	0.108	0.049	1.35E-26	1.4	QRSL1	2.204081633
ARSB1	5.69E-31	0.1819555	0.071	0.027	1.37E-26	1.4	ARSB	2.62962963
CLSTN31	6.32E-31	0.2041773	0.082	0.033	1.52E-26	1.4	CLSTN3	2.484848485
RP11-452L6.11	6.67E-31	0.1299495	0.066	0.023	1.61E-26	1.4	RP11-452L(	2.869565217
RPRD21	6.96E-31	0.245545	0.232	0.139	1.68E-26	1.4	RPRD2	1.669064748
HSPA82	7.25E-31	0.2137313	0.591	0.442	1.75E-26	1.4	HSPA8	1.337104072
ENOX21	1.10E-30	0.2056735	0.093	0.04	2.65E-26	1.4	ENOX2	2.325
PHC31	1.14E-30	0.2108378	0.26	0.159	2.74E-26	1.4	PHC3	1.635220126
SP140L1	1.14E-30	0.1393249	0.073	0.028	2.76E-26	1.4	SP140L	2.607142857
AC008074.32	1.18E-30	0.1733136	0.1	0.044	2.84E-26	1.4	AC008074.:	2.272727273
PRKRIP11	1.19E-30	0.2079238	0.199	0.113	2.87E-26	1.4	PRKRIP1	1.761061947
AC023590.11	1.45E-30	0.1247908	0.066	0.024	3.50E-26	1.4	AC023590.	2.75
SLC38A61	1.72E-30	0.1378577	0.071	0.027	4.15E-26	1.4	SLC38A6	2.62962963
RNASE11	1.73E-30	0.121195	0.053	0.017	4.18E-26	1.4	RNASE1	3.117647059
IGBP12	1.86E-30	0.2220578	0.524	0.38	4.48E-26	1.4	IGBP1	1.378947368
BBX1	2.02E-30	0.2493247	0.527	0.385	4.86E-26	1.4	BBX	1.368831169
CEBPZOS1	2.04E-30	0.1722843	0.19	0.107	4.92E-26	1.4	CEBPZOS	1.775700935
NSMCE21	2.07E-30	0.2128266	0.509	0.366	4.98E-26	1.4	NSMCE2	1.390710383
ADH52	2.18E-30	0.1630774	0.15	0.077	5.26E-26	1.4	ADH5	1.948051948
BCL7A2	2.34E-30	0.1297197	0.067	0.024	5.64E-26	1.4	BCL7A	2.791666667
PPIP5K11	2.48E-30	0.156765	0.075	0.029	5.97E-26	1.4	PPIP5K1	2.586206897
PSME22	2.59E-30	0.4337565	0.393	0.281	6.26E-26	1.4	PSME2	1.398576512
DOCK11	2.75E-30	0.2762589	0.401	0.278	6.62E-26	1.4	DOCK1	1.442446043
GRB143	2.77E-30	0.2442041	0.53	0.394	6.67E-26	1.4	GRB14	1.345177665
XRN1	3.25E-30	0.2745438	0.425	0.297	7.84E-26	1.4	XRN1	1.430976431
TYW1B2	3.58E-30	0.2426281	0.209	0.121	8.62E-26	1.4	TYW1B	1.727272727
TIMM93	4.22E-30	0.1466351	0.22	0.127	1.02E-25	1.4	TIMM9	1.732283465
MLXIP2	4.41E-30	0.2102246	0.216	0.126	1.06E-25	1.4	MLXIP	1.714285714
FOXP21	4.63E-30	0.1353669	0.056	0.018	1.12E-25	1.4	FOXP2	3.111111111
LMF11	5.25E-30	0.1342847	0.071	0.027	1.27E-25	1.4	LMF1	2.62962963
RP11-544A12.81	5.61E-30	0.1490642	0.07	0.026	1.35E-25	1.4	RP11-544A	2.692307692
MMADHC2	5.92E-30	0.2155259	0.426	0.294	1.43E-25	1.4	MMADHC	1.448979592
PDCD4	7.53E-30	0.2976861	0.341	0.229	1.82E-25	1.4	PDCD4	1.489082969
RP11-283G6.41	8.84E-30	0.1131558	0.038	0.01	2.13E-25	1.4	RP11-283G	3.8
SLC25A61	9.15E-30	0.3034087	0.186	0.106	2.21E-25	1.4	SLC25A6	1.754716981
TRIM51	9.17E-30	0.2640277	0.19	0.109	2.21E-25	1.4	TRIM5	1.743119266
PARN1	1.21E-29	0.2034688	0.146	0.076	2.91E-25	1.4	PARN	1.921052632
CXADR2	1.28E-29	0.2609293	0.269	0.169	3.09E-25	1.4	CXADR	1.591715976
DYNLT11	1.29E-29	0.2084117	0.735	0.577	3.10E-25	1.4	DYNLT1	1.273830156
PLEKHH23	1.36E-29	0.206836	0.122	0.06	3.28E-25	1.4	PLEKHH2	2.033333333
DPYD-AS11	1.48E-29	0.2391637	0.135	0.069	3.57E-25	1.4	DPYD-AS1	1.956521739
ZCCHC112	1.57E-29	0.2034546	0.308	0.197	3.78E-25	1.4	ZCCHC11	1.563451777
ESR1	1.60E-29	0.2595442	0.277	0.175	3.85E-25	1.4	ESR1	1.582857143
C6orf891	1.97E-29	0.1577341	0.118	0.057	4.75E-25	1.4	C6orf89	2.070175439
GTF3C31	2.01E-29	0.1501869	0.103	0.047	4.83E-25	1.4	GTF3C3	2.191489362
SRSF123	2.01E-29	0.1162262	0.088	0.037	4.85E-25	1.4	SRSF12	2.378378378

C7orf491	2.07E-29	0.1351584	0.055	0.018	4.99E-25	1.4	C7orf49	3.055555556
RNF1801	2.14E-29	0.1502801	0.059	0.021	5.15E-25	1.4	RNF180	2.80952381
CEACAM12	2.37E-29	0.193449	0.136	0.07	5.71E-25	1.4	CEACAM1	1.942857143
RRAGD3	2.54E-29	0.1319368	0.103	0.047	6.13E-25	1.4	RRAGD	2.191489362
WFDC32	2.54E-29	0.1814218	0.12	0.058	6.13E-25	1.4	WFDC3	2.068965517
PSMA33	2.58E-29	0.2729103	0.453	0.324	6.22E-25	1.4	PSMA3	1.398148148
EIF4B3	2.70E-29	0.2055705	0.51	0.366	6.51E-25	1.4	EIF4B	1.393442623
CHKA1	3.09E-29	0.2472796	0.177	0.099	7.46E-25	1.4	CHKA	1.787878788
PRKAG21	3.28E-29	0.3140865	0.23	0.141	7.92E-25	1.4	PRKAG2	1.631205674
LRP51	3.39E-29	0.1348723	0.064	0.023	8.17E-25	1.4	LRP5	2.782608696
PCID21	3.42E-29	0.1827486	0.158	0.085	8.25E-25	1.4	PCID2	1.858823529
HIST1H2AC1	3.52E-29	0.302879	0.483	0.352	8.48E-25	1.4	HIST1H2AC	1.372159091
RPS93	3.66E-29	0.69796	0.324	0.218	8.82E-25	1.4	RPS9	1.486238532
PITPNA1	3.72E-29	0.2296728	0.214	0.127	8.96E-25	1.4	PITPNA	1.68503937
DDX12	3.83E-29	0.1625398	0.163	0.088	9.23E-25	1.4	DDX1	1.852272727
CRHR11	4.87E-29	0.1659429	0.079	0.032	1.17E-24	1.4	CRHR1	2.46875
VPS451	5.87E-29	0.1979407	0.138	0.071	1.41E-24	1.4	VPS45	1.943661972
JAZF12	5.93E-29	0.2163272	0.15	0.079	1.43E-24	1.4	JAZF1	1.898734177
POLR1D3	7.71E-29	0.210162	0.324	0.214	1.86E-24	1.4	POLR1D	1.514018692
RP11-577H5.51	8.74E-29	0.1667393	0.104	0.048	2.11E-24	1.4	RP11-577H	2.166666667
SERPINA3	9.97E-29	0.3934544	0.365	0.253	2.40E-24	1.4	SERPINA3	1.442687747
INPP5B1	1.14E-28	0.1366761	0.084	0.035	2.74E-24	1.4	INPP5B	2.4
ZNF1061	1.14E-28	0.1678563	0.175	0.097	2.76E-24	1.4	ZNF106	1.804123711
MRPS332	1.18E-28	0.174851	0.345	0.228	2.85E-24	1.4	MRPS33	1.513157895
PPP1CB2	1.23E-28	0.2394019	0.473	0.34	2.97E-24	1.4	PPP1CB	1.391176471
STX122	1.30E-28	0.1736103	0.44	0.307	3.14E-24	1.4	STX12	1.433224756
CREG12	1.32E-28	0.1439752	0.101	0.046	3.19E-24	1.4	CREG1	2.195652174
BACE23	1.60E-28	0.1891193	0.427	0.298	3.86E-24	1.4	BACE2	1.432885906
NEK91	1.88E-28	0.1587217	0.121	0.06	4.53E-24	1.4	NEK9	2.016666667
PM20D22	1.99E-28	0.1736289	0.153	0.082	4.81E-24	1.4	PM20D2	1.865853659
NAA381	2.20E-28	0.19504	0.109	0.052	5.30E-24	1.4	NAA38	2.096153846
ELOVL61	2.30E-28	0.1954682	0.135	0.07	5.54E-24	1.4	ELOVL6	1.928571429
GCA1	2.32E-28	0.1547901	0.097	0.044	5.60E-24	1.4	GCA	2.204545455
KLHL201	2.47E-28	0.1471337	0.113	0.054	5.96E-24	1.4	KLHL20	2.092592593
MTMR22	2.48E-28	0.1978938	0.127	0.064	5.98E-24	1.4	MTMR2	1.984375
SNX31	2.49E-28	0.1899137	0.25	0.154	6.01E-24	1.4	SNX3	1.623376623
CLCN31	2.50E-28	0.212831	0.253	0.158	6.03E-24	1.4	CLCN3	1.601265823
ADRBK22	2.74E-28	0.2046748	0.142	0.075	6.62E-24	1.4	ADRBK2	1.893333333
SULF2	3.25E-28	0.22769	0.179	0.101	7.83E-24	1.4	SULF2	1.772277228
PDSS21	3.62E-28	0.254135	0.2	0.117	8.72E-24	1.4	PDSS2	1.709401709
ZNF5651	3.64E-28	0.1924238	0.24	0.146	8.77E-24	1.4	ZNF565	1.643835616
SLC6A143	3.78E-28	0.2002602	0.173	0.097	9.13E-24	1.4	SLC6A14	1.783505155
ZFR2	4.04E-28	0.2377914	0.437	0.31	9.74E-24	1.4	ZFR	1.409677419
CD822	5.49E-28	0.1695636	0.1	0.046	1.32E-23	1.4	CD82	2.173913043
TMEM150C3	5.61E-28	0.1948963	0.302	0.195	1.35E-23	1.4	TMEM150(	1.548717949
LMO41	5.62E-28	0.1863628	0.143	0.075	1.35E-23	1.4	LMO4	1.906666667
PPP2R1B	5.65E-28	0.1948465	0.154	0.083	1.36E-23	1.4	PPP2R1B	1.855421687
BTN2A2	5.84E-28	0.1446674	0.084	0.036	1.41E-23	1.4	BTN2A2	2.333333333
TASP11	6.29E-28	0.2018469	0.206	0.122	1.52E-23	1.4	TASP1	1.68852459
MCTS12	7.38E-28	0.1722549	0.315	0.204	1.78E-23	1.4	MCTS1	1.544117647
ZNF6081	7.84E-28	0.247252	0.267	0.17	1.89E-23	1.4	ZNF608	1.570588235
LINC012521	8.79E-28	0.1353819	0.064	0.024	2.12E-23	1.4	LINC01252	2.666666667

MACROD21	8.79E-28	0.3202562	0.336	0.231	2.12E-23	1.4	MACROD2	1.454545455
PSMA41	9.64E-28	0.2942668	0.565	0.424	2.33E-23	1.4	PSMA4	1.33254717
DOK53	9.69E-28	0.1390098	0.061	0.022	2.34E-23	1.4	DOK5	2.772727273
BTF3L41	9.72E-28	0.1991909	0.333	0.221	2.34E-23	1.4	BTF3L4	1.50678733
BCL2A11	9.95E-28	0.2781831	0.142	0.075	2.40E-23	1.4	BCL2A1	1.893333333
AKT33	1.00E-27	0.2722588	0.46	0.333	2.41E-23	1.4	AKT3	1.381381381
LINC008691	1.21E-27	0.1521024	0.085	0.037	2.92E-23	1.4	LINC00869	2.297297297
RP11-66B24.22	1.26E-27	0.1357011	0.063	0.023	3.03E-23	1.4	RP11-66B2	2.739130435
LSM82	1.28E-27	0.2152082	0.299	0.196	3.09E-23	1.4	LSM8	1.525510204
PSMB91	1.39E-27	0.1129764	0.047	0.015	3.35E-23	1.4	PSMB9	3.133333333
ATP5C12	1.51E-27	0.1875222	0.396	0.271	3.63E-23	1.4	ATP5C1	1.461254613
RNF19B1	1.79E-27	0.232831	0.219	0.133	4.32E-23	1.4	RNF19B	1.646616541
NRBF21	1.95E-27	0.1265489	0.1	0.046	4.71E-23	1.4	NRBF2	2.173913043
TPK13	1.98E-27	0.3231995	0.297	0.198	4.78E-23	1.4	TPK1	1.5
ICE2	2.37E-27	0.141139	0.099	0.045	5.71E-23	1.4	ICE2	2.2
LRP62	2.62E-27	0.1810943	0.28	0.18	6.32E-23	1.4	LRP6	1.555555556
LINC009071	2.82E-27	0.1200902	0.05	0.016	6.80E-23	1.4	LINC00907	3.125
ENSA	2.86E-27	0.2461705	0.484	0.355	6.90E-23	1.4	ENSA	1.363380282
DDX243	2.87E-27	0.2099925	0.639	0.49	6.91E-23	1.4	DDX24	1.304081633
FMNL22	2.87E-27	0.2271514	0.469	0.338	6.93E-23	1.4	FMNL2	1.387573964
CALCOCO11	4.12E-27	0.1492323	0.119	0.059	9.94E-23	1.4	CALCOCO1	2.016949153
TMA161	4.23E-27	0.1388397	0.074	0.031	1.02E-22	1.4	TMA16	2.387096774
NOS1AP	4.23E-27	0.377328	0.31	0.207	1.02E-22	1.4	NOS1AP	1.497584541
LARP12	4.38E-27	0.1914807	0.213	0.127	1.06E-22	1.4	LARP1	1.677165354
EIF2AK21	4.64E-27	0.186758	0.152	0.083	1.12E-22	1.4	EIF2AK2	1.831325301
SMC61	4.74E-27	0.170368	0.131	0.068	1.14E-22	1.4	SMC6	1.926470588
RCOR3	5.28E-27	0.2236721	0.159	0.088	1.27E-22	1.4	RCOR3	1.806818182
FAM134B2	5.33E-27	0.1973224	0.158	0.087	1.28E-22	1.4	FAM134B	1.816091954
ACSL43	5.47E-27	0.1417087	0.101	0.047	1.32E-22	1.4	ACSL4	2.14893617
WDR271	7.12E-27	0.1429471	0.073	0.03	1.72E-22	1.4	WDR27	2.433333333
CARD62	8.36E-27	0.1053505	0.069	0.027	2.02E-22	1.4	CARD6	2.555555556
EVL	8.63E-27	0.1974436	0.121	0.062	2.08E-22	1.4	EVL	1.951612903
ANAPC162	9.86E-27	0.1877963	0.349	0.235	2.38E-22	1.4	ANAPC16	1.485106383
RPL293	1.02E-26	0.630232	0.328	0.226	2.46E-22	1.4	RPL29	1.451327434
CD461	1.05E-26	0.3492674	0.51	0.39	2.53E-22	1.4	CD46	1.307692308
SNX82	1.11E-26	0.1656274	0.096	0.045	2.67E-22	1.4	SNX8	2.133333333
SOCS52	1.33E-26	0.2377986	0.206	0.124	3.21E-22	1.4	SOCS5	1.661290323
ZNF1462	1.38E-26	0.1747929	0.164	0.092	3.33E-22	1.4	ZNF146	1.782608696
ANKRD362	1.46E-26	0.2186333	0.149	0.081	3.52E-22	1.4	ANKRD36	1.839506173
CD831	1.46E-26	0.1901756	0.123	0.062	3.52E-22	1.4	CD83	1.983870968
C10orf761	1.47E-26	0.1689252	0.208	0.123	3.54E-22	1.4	C10orf76	1.691056911
IFNGR1	1.54E-26	0.2669776	0.374	0.26	3.71E-22	1.4	IFNGR1	1.438461538
VDR1	1.73E-26	0.1666821	0.141	0.075	4.17E-22	1.4	VDR	1.88
TMEM41A3	1.99E-26	0.1842757	0.215	0.13	4.80E-22	1.4	TMEM41A	1.653846154
CCDC911	2.08E-26	0.2333071	0.463	0.334	5.01E-22	1.4	CCDC91	1.386227545
RNF1301	2.11E-26	0.1927475	0.264	0.167	5.09E-22	1.4	RNF130	1.580838323
ANKRD13C1	2.29E-26	0.2166148	0.181	0.105	5.53E-22	1.4	ANKRD13C	1.723809524
PRKD32	2.32E-26	0.1764063	0.182	0.105	5.60E-22	1.4	PRKD3	1.733333333
DAPK23	2.60E-26	0.1089335	0.337	0.221	6.27E-22	1.4	DAPK2	1.524886878
EIF3J-AS11	2.74E-26	0.1264735	0.095	0.044	6.61E-22	1.4	EIF3J-AS1	2.159090909
GRIP11	2.75E-26	0.258389	0.275	0.179	6.64E-22	1.4	GRIP1	1.536312849
SNX291	2.77E-26	0.2604747	0.16	0.09	6.69E-22	1.4	SNX29	1.777777778

KLHDC11	2.91E-26	0.1334548	0.059	0.022	7.02E-22	1.4	KLHDC1	2.681818182
NBR11	2.95E-26	0.1799898	0.259	0.163	7.11E-22	1.4	NBR1	1.588957055
TUSC32	3.46E-26	0.193835	0.173	0.098	8.34E-22	1.4	TUSC3	1.765306122
SPATS2L1	3.82E-26	0.2746524	0.33	0.227	9.22E-22	1.4	SPATS2L	1.453744493
RP11-286N22.81	3.91E-26	0.1409876	0.082	0.036	9.42E-22	1.4	RP11-286N	2.277777778
MTR1	4.08E-26	0.1768238	0.113	0.056	9.85E-22	1.4	MTR	2.017857143
KB-1562D12.11	4.12E-26	0.1400175	0.071	0.029	9.94E-22	1.4	KB-1562D1	2.448275862
STAT1	5.04E-26	0.1421872	0.135	0.071	1.22E-21	1.4	STAT1	1.901408451
NHLRC21	5.10E-26	0.1618072	0.108	0.053	1.23E-21	1.4	NHLRC2	2.037735849
PLB11	5.16E-26	0.1000397	0.066	0.026	1.24E-21	1.4	PLB1	2.538461538
AC159540.12	5.35E-26	0.2577404	0.169	0.097	1.29E-21	1.4	AC159540.	1.742268041
SACS2	5.36E-26	0.1725371	0.107	0.053	1.29E-21	1.4	SACS	2.018867925
KCNC42	5.53E-26	0.1396756	0.05	0.017	1.33E-21	1.4	KCNC4	2.941176471
SMPDL3A	5.59E-26	0.1044696	0.051	0.018	1.35E-21	1.4	SMPDL3A	2.833333333
GCDH3	5.80E-26	0.1452875	0.124	0.064	1.40E-21	1.4	GCDH	1.9375
RNF2172	5.87E-26	0.1570382	0.121	0.062	1.42E-21	1.4	RNF217	1.951612903
TAF4B1	6.11E-26	0.1558514	0.099	0.047	1.47E-21	1.4	TAF4B	2.106382979
CCNB1IP13	7.29E-26	0.1665528	0.355	0.241	1.76E-21	1.4	CCNB1IP1	1.473029046
PSMB12	7.33E-26	0.1953215	0.514	0.377	1.77E-21	1.4	PSMB1	1.363395225
ST133	7.72E-26	0.2051227	0.392	0.273	1.86E-21	1.4	ST13	1.435897436
CYB5R22	8.28E-26	0.1160688	0.073	0.03	2.00E-21	1.4	CYB5R2	2.433333333
GGA11	8.52E-26	0.1546609	0.077	0.033	2.06E-21	1.4	GGA1	2.333333333
SRSF33	8.80E-26	0.1711275	0.555	0.417	2.12E-21	1.4	SRSF3	1.330935252
ZNF529-AS12	1.03E-25	0.1372328	0.08	0.035	2.48E-21	1.4	ZNF529-AS	2.285714286
RPL27A1	1.05E-25	0.3355484	0.531	0.411	2.53E-21	1.4	RPL27A	1.291970803
ATG71	1.05E-25	0.211858	0.303	0.202	2.54E-21	1.4	ATG7	1.5
NUDT51	1.09E-25	0.1726899	0.212	0.129	2.62E-21	1.4	NUDT5	1.643410853
BRE	1.18E-25	0.2588934	0.297	0.199	2.84E-21	1.4	BRE	1.492462312
PXK1	1.21E-25	0.127596	0.098	0.046	2.92E-21	1.4	PXK	2.130434783
PTPN41	1.22E-25	0.1816317	0.146	0.08	2.94E-21	1.4	PTPN4	1.825
ATP6AP1L	1.38E-25	0.1157066	0.063	0.024	3.32E-21	1.4	ATP6AP1L	2.625
TMPRSS32	1.49E-25	0.1497268	0.09	0.041	3.60E-21	1.4	TMPRSS3	2.195121951
DNAJC72	1.50E-25	0.1944597	0.263	0.169	3.61E-21	1.4	DNAJC7	1.556213018
PSMC22	1.56E-25	0.1915867	0.31	0.208	3.76E-21	1.4	PSMC2	1.490384615
TTC9C1	1.57E-25	0.1748313	0.189	0.112	3.77E-21	1.4	TTC9C	1.6875
NSF1	1.61E-25	0.2208207	0.228	0.142	3.88E-21	1.4	NSF	1.605633803
RIPK22	1.62E-25	0.1954398	0.382	0.267	3.92E-21	1.4	RIPK2	1.43071161
ARHGAP241	1.76E-25	0.1717114	0.07	0.029	4.23E-21	1.4	ARHGAP24	2.413793103
AIMP13	1.81E-25	0.1941819	0.407	0.289	4.37E-21	1.4	AIMP1	1.408304498
RPS19BP1	1.83E-25	0.1397081	0.051	0.018	4.41E-21	1.4	RPS19BP1	2.833333333
ERRFI12	2.04E-25	0.2159783	0.661	0.526	4.91E-21	1.4	ERRFI1	1.256653992
RPS113	2.33E-25	0.3601178	0.786	0.769	5.62E-21	1.4	RPS11	1.022106632
THUMPD11	2.57E-25	0.1647223	0.135	0.072	6.20E-21	1.4	THUMPD1	1.875
C9orf31	2.77E-25	0.3084333	0.208	0.13	6.69E-21	1.4	C9orf3	1.6
SLC9A81	2.79E-25	0.1732355	0.147	0.081	6.73E-21	1.4	SLC9A8	1.814814815
PDLIM52	2.85E-25	0.1309327	0.85	0.729	6.87E-21	1.4	PDLIM5	1.165980796
RAD501	2.87E-25	0.1760134	0.145	0.079	6.92E-21	1.4	RAD50	1.835443038
MBD5	2.88E-25	0.2282427	0.414	0.294	6.95E-21	1.4	MBD5	1.408163265
DLEU2	2.89E-25	0.1792124	0.315	0.211	6.98E-21	1.4	DLEU2	1.492890995
TTC141	3.17E-25	0.1855447	0.126	0.066	7.64E-21	1.4	TTC14	1.909090909
N4BP2L1	3.35E-25	0.1945229	0.176	0.102	8.08E-21	1.4	N4BP2L1	1.725490196
SLC25A332	3.36E-25	0.2262101	0.155	0.088	8.11E-21	1.4	SLC25A33	1.761363636

SLC6A16	3.37E-25	0.1471948	0.091	0.042	8.13E-21	1.4	SLC6A16	2.166666667
RPL393	3.86E-25	0.2958624	0.933	0.894	9.31E-21	1.4	RPL39	1.043624161
ZNF7911	4.21E-25	0.1709832	0.159	0.09	1.01E-20	1.4	ZNF791	1.766666667
SEC24D2	4.47E-25	0.1210057	0.285	0.184	1.08E-20	1.4	SEC24D	1.548913043
CRLF32	4.59E-25	0.1904282	0.167	0.096	1.11E-20	1.4	CRLF3	1.739583333
KIF9-AS11	4.76E-25	0.1620776	0.085	0.038	1.15E-20	1.4	KIF9-AS1	2.236842105
RBMX21	5.22E-25	0.1439199	0.154	0.086	1.26E-20	1.4	RBMX2	1.790697674
RPL93	5.45E-25	0.2362591	0.681	0.556	1.31E-20	1.4	RPL9	1.224820144
ZC3H6	5.86E-25	0.1690881	0.129	0.069	1.41E-20	1.4	ZC3H6	1.869565217
KCTD72	6.35E-25	0.2002532	0.176	0.103	1.53E-20	1.4	KCTD7	1.708737864
PIK3IP1-AS11	6.98E-25	0.1496126	0.081	0.036	1.68E-20	1.4	PIK3IP1-AS	2.25
FAM49B2	7.25E-25	0.2204017	0.29	0.193	1.75E-20	1.4	FAM49B	1.502590674
SECISBP2L1	7.75E-25	0.1972467	0.308	0.205	1.87E-20	1.4	SECISBP2L	1.502439024
SLC2A91	8.30E-25	0.1179089	0.071	0.029	2.00E-20	1.4	SLC2A9	2.448275862
CTA-293F17.13	8.40E-25	0.1241476	0.125	0.065	2.03E-20	1.4	CTA-293F1	1.923076923
FOXP1	8.68E-25	0.1295106	0.786	0.616	2.09E-20	1.4	FOXP1	1.275974026
CCNC2	8.82E-25	0.220348	0.305	0.206	2.13E-20	1.4	CCNC	1.480582524
ZNF1312	9.60E-25	0.1987323	0.193	0.117	2.31E-20	1.4	ZNF131	1.64957265
FAM189A22	1.16E-24	0.1691479	0.12	0.062	2.80E-20	1.4	FAM189A2	1.935483871
ANKRA2	1.16E-24	0.1396035	0.122	0.063	2.81E-20	1.4	ANKRA2	1.936507937
TLK11	1.18E-24	0.2279444	0.28	0.185	2.84E-20	1.4	TLK1	1.513513514
EXOC41	1.19E-24	0.2443226	0.448	0.325	2.86E-20	1.4	EXOC4	1.378461538
SRGAP31	1.24E-24	0.2015386	0.096	0.046	2.99E-20	1.4	SRGAP3	2.086956522
ZCCHC6	1.43E-24	0.2403121	0.447	0.327	3.44E-20	1.4	ZCCHC6	1.366972477
USP401	1.71E-24	0.1810924	0.129	0.069	4.13E-20	1.4	USP40	1.869565217
RAB241	1.76E-24	0.1273007	0.058	0.022	4.24E-20	1.4	RAB24	2.636363636
STYXL11	1.99E-24	0.1287886	0.092	0.043	4.80E-20	1.4	STYXL1	2.139534884
IL27RA2	1.99E-24	0.113043	0.055	0.02	4.81E-20	1.4	IL27RA	2.75
KRR13	2.22E-24	0.2120288	0.227	0.144	5.35E-20	1.4	KRR1	1.576388889
NSUN41	2.23E-24	0.1421721	0.116	0.06	5.39E-20	1.4	NSUN4	1.933333333
PSMF11	2.24E-24	0.1652406	0.134	0.072	5.41E-20	1.4	PSMF1	1.861111111
SLC26A81	2.27E-24	0.1186723	0.052	0.019	5.48E-20	1.4	SLC26A8	2.736842105
PACRGL	2.34E-24	0.1141452	0.074	0.032	5.64E-20	1.4	PACRGL	2.3125
NSMAF1	2.37E-24	0.1671417	0.127	0.067	5.71E-20	1.4	NSMAF	1.895522388
RP11-631N16.22	2.40E-24	0.1567596	0.084	0.038	5.80E-20	1.4	RP11-631N	2.210526316
RP11-513G19.11	2.50E-24	0.1110758	0.038	0.011	6.02E-20	1.4	RP11-513G	3.454545455
FAM217B1	2.59E-24	0.1001106	0.071	0.03	6.24E-20	1.4	FAM217B	2.366666667
CHDH1	2.73E-24	0.1655419	0.096	0.046	6.59E-20	1.4	CHDH	2.086956522
FBXW111	3.07E-24	0.1950871	0.396	0.28	7.40E-20	1.4	FBXW11	1.414285714
PGBD52	3.26E-24	0.1295703	0.079	0.035	7.87E-20	1.4	PGBD5	2.257142857
ARMT11	3.27E-24	0.1671585	0.14	0.076	7.88E-20	1.4	ARMT1	1.842105263
ZNF2351	3.60E-24	0.1725663	0.129	0.069	8.67E-20	1.4	ZNF235	1.869565217
SPDYE12	4.38E-24	0.1132739	0.07	0.03	1.06E-19	1.4	SPDYE1	2.333333333
PPA21	4.96E-24	0.2171987	0.28	0.185	1.20E-19	1.4	PPA2	1.513513514
RPS162	5.27E-24	0.4188612	0.407	0.298	1.27E-19	1.4	RPS16	1.365771812
SUB13	5.50E-24	0.131216	0.75	0.624	1.33E-19	1.4	SUB1	1.201923077
TBC1D141	5.72E-24	0.1827606	0.124	0.065	1.38E-19	1.4	TBC1D14	1.907692308
LHPP1	5.97E-24	0.1039585	0.046	0.016	1.44E-19	1.4	LHPP	2.875
LINC014812	6.36E-24	0.1632736	0.079	0.035	1.53E-19	1.4	LINC01481	2.257142857
NFE2L21	7.23E-24	0.2592658	0.486	0.369	1.74E-19	1.4	NFE2L2	1.317073171
SENP51	8.29E-24	0.1993684	0.282	0.187	2.00E-19	1.4	SENP5	1.50802139
WDSUB11	8.91E-24	0.1472027	0.101	0.05	2.15E-19	1.4	WDSUB1	2.02

PLOD12	9.95E-24	0.1230032	0.086	0.04	2.40E-19	1.4	PLOD1	2.15
HLCS1	1.08E-23	0.177438	0.137	0.075	2.61E-19	1.4	HLCS	1.826666667
ZNF6771	1.11E-23	0.1233161	0.083	0.038	2.68E-19	1.4	ZNF677	2.184210526
RP11-712B9.21	1.25E-23	0.1287161	0.066	0.027	3.01E-19	1.4	RP11-712B	2.444444444
TRPC12	1.40E-23	0.1139754	0.063	0.026	3.39E-19	1.4	TRPC1	2.423076923
LINC015492	1.46E-23	0.2595731	0.043	0.014	3.52E-19	1.4	LINC01549	3.071428571
MRPL91	1.47E-23	0.1023491	0.055	0.021	3.54E-19	1.4	MRPL9	2.619047619
MRPS18B2	1.56E-23	0.1495288	0.184	0.109	3.76E-19	1.4	MRPS18B	1.688073394
ZC3H82	1.68E-23	0.1236123	0.123	0.065	4.06E-19	1.4	ZC3H8	1.892307692
RABGAP1L1	1.87E-23	0.1888454	0.319	0.218	4.50E-19	1.4	RABGAP1L	1.463302752
TRIM131	1.88E-23	0.133606	0.136	0.075	4.54E-19	1.4	TRIM13	1.813333333
MITD11	1.98E-23	0.1499919	0.163	0.095	4.78E-19	1.4	MITD1	1.715789474
ZNF2541	2.29E-23	0.193843	0.238	0.153	5.52E-19	1.4	ZNF254	1.555555556
ECHDC21	2.55E-23	0.1801077	0.189	0.114	6.14E-19	1.4	ECHDC2	1.657894737
SPPL3	2.64E-23	0.2017494	0.361	0.254	6.36E-19	1.4	SPPL3	1.421259843
ANKRD26	2.68E-23	0.159718	0.131	0.071	6.47E-19	1.4	ANKRD26	1.845070423
XPNPEP31	2.88E-23	0.1340182	0.1	0.05	6.94E-19	1.4	XPNPEP3	2
CPD	3.11E-23	0.2862775	0.326	0.231	7.50E-19	1.4	CPD	1.411255411
CASC41	3.30E-23	0.2234896	0.269	0.179	7.96E-19	1.4	CASC4	1.502793296
BET11	3.37E-23	0.1814946	0.196	0.12	8.12E-19	1.4	BET1	1.633333333
KIZ1	3.40E-23	0.115134	0.059	0.023	8.21E-19	1.4	KIZ	2.565217391
CMC11	3.42E-23	0.1474957	0.089	0.042	8.25E-19	1.4	CMC1	2.119047619
MFN12	4.45E-23	0.1751481	0.192	0.117	1.07E-18	1.4	MFN1	1.641025641
ZNF3021	4.47E-23	0.1001962	0.062	0.025	1.08E-18	1.4	ZNF302	2.48
PRR5L1	4.67E-23	0.1208866	0.053	0.02	1.13E-18	1.4	PRR5L	2.65
MRRF2	4.69E-23	0.1812395	0.203	0.125	1.13E-18	1.4	MRRF	1.624
MON2	5.24E-23	0.1786175	0.324	0.223	1.26E-18	1.4	MON2	1.452914798
PRICKLE13	5.60E-23	0.1824565	0.097	0.048	1.35E-18	1.4	PRICKLE1	2.020833333
TBC1D32	6.24E-23	0.2167278	0.102	0.052	1.50E-18	1.4	TBC1D32	1.961538462
ATP11A2	7.20E-23	0.1498068	0.104	0.053	1.74E-18	1.4	ATP11A	1.962264151
PPID1	7.75E-23	0.131042	0.113	0.058	1.87E-18	1.4	PPID	1.948275862
SUSD4	8.07E-23	0.1169503	0.071	0.031	1.95E-18	1.4	SUSD4	2.290322581
RP11-541G9.11	8.17E-23	0.1030786	0.039	0.012	1.97E-18	1.4	RP11-541G	3.25
TCP13	8.38E-23	0.1366513	0.247	0.159	2.02E-18	1.4	TCP1	1.553459119
RP11-545I5.31	8.49E-23	0.1000306	0.044	0.015	2.05E-18	1.4	RP11-545I5	2.933333333
NUP371	9.03E-23	0.1167831	0.084	0.039	2.18E-18	1.4	NUP37	2.153846154
RPL373	9.58E-23	0.2138182	0.963	0.943	2.31E-18	1.4	RPL37	1.021208908
NEURL32	9.68E-23	0.1195895	0.064	0.027	2.33E-18	1.4	NEURL3	2.37037037
DPH61	1.02E-22	0.1888349	0.115	0.061	2.46E-18	1.4	DPH6	1.885245902
C6orf2031	1.02E-22	0.1082248	0.061	0.025	2.47E-18	1.4	C6orf203	2.44
KDM2B1	1.11E-22	0.1432739	0.076	0.034	2.68E-18	1.4	KDM2B	2.235294118
TRAF32	1.12E-22	0.230904	0.186	0.115	2.70E-18	1.4	TRAF3	1.617391304
NUBPL1	1.22E-22	0.1661982	0.099	0.05	2.94E-18	1.4	NUBPL	1.98
AMY2B	1.24E-22	0.1131289	0.078	0.035	2.98E-18	1.4	AMY2B	2.228571429
INTS10	1.25E-22	0.1671437	0.201	0.124	3.01E-18	1.4	INTS10	1.620967742
AKAP11	1.26E-22	0.1286365	0.059	0.024	3.03E-18	1.4	AKAP1	2.458333333
C7orf601	1.31E-22	0.1524769	0.124	0.067	3.15E-18	1.4	C7orf60	1.850746269
ZNF4381	1.32E-22	0.1658734	0.122	0.066	3.19E-18	1.4	ZNF438	1.848484848
RP11-541P9.32	1.54E-22	0.1465156	0.093	0.045	3.72E-18	1.4	RP11-541P9	2.066666667
RP11-440L14.12	1.96E-22	0.127111	0.074	0.033	4.72E-18	1.4	RP11-440L1	2.242424242
KAT2B1	1.97E-22	0.120307	0.063	0.026	4.76E-18	1.4	KAT2B	2.423076923
TAPT1-AS11	2.93E-22	0.1651027	0.109	0.057	7.06E-18	1.4	TAPT1-AS1	1.912280702

MAGI12	3.32E-22	0.1801981	0.857	0.74	8.02E-18	1.4	MAGI1	1.158108108
SCAI1	4.53E-22	0.1827215	0.111	0.059	1.09E-17	1.4	SCAI	1.881355932
FCHSD2	4.57E-22	0.1601608	0.199	0.123	1.10E-17	1.4	FCHSD2	1.617886179
TATDN13	4.59E-22	0.1393438	0.301	0.202	1.11E-17	1.4	TATDN1	1.49009901
IDE1	4.69E-22	0.1497784	0.12	0.064	1.13E-17	1.4	IDE	1.875
SIL11	4.74E-22	0.2044514	0.147	0.085	1.14E-17	1.4	SIL1	1.729411765
MRPS311	5.76E-22	0.1515567	0.157	0.092	1.39E-17	1.4	MRPS31	1.706521739
FRK2	6.44E-22	0.1878563	0.207	0.131	1.55E-17	1.4	FRK	1.580152672
CLIC43	7.01E-22	0.2186878	0.668	0.548	1.69E-17	1.4	CLIC4	1.218978102
ELMOD31	7.08E-22	0.1301353	0.144	0.081	1.71E-17	1.4	ELMOD3	1.777777778
BBIP11	7.53E-22	0.1531406	0.164	0.097	1.82E-17	1.4	BBIP1	1.690721649
DDIT43	8.03E-22	0.1716877	0.234	0.151	1.94E-17	1.4	DDIT4	1.549668874
PARG1	8.48E-22	0.1616902	0.14	0.08	2.04E-17	1.4	PARG	1.75
ICK1	8.59E-22	0.1133707	0.078	0.036	2.07E-17	1.4	ICK	2.166666667
TMEM1171	9.03E-22	0.1408768	0.07	0.031	2.18E-17	1.4	TMEM117	2.258064516
MDM41	9.08E-22	0.2291676	0.336	0.239	2.19E-17	1.4	MDM4	1.405857741
FOCAD1	9.22E-22	0.1785029	0.239	0.155	2.22E-17	1.4	FOCAD	1.541935484
CCDC823	9.32E-22	0.1555528	0.229	0.148	2.25E-17	1.4	CCDC82	1.547297297
IQCK	9.79E-22	0.1679419	0.101	0.052	2.36E-17	1.4	IQCK	1.942307692
COMMD101	1.03E-21	0.2025522	0.283	0.193	2.48E-17	1.4	COMMD10	1.466321244
TTC92	1.21E-21	0.1990839	0.242	0.159	2.92E-17	1.4	TTC9	1.522012579
APTR2	1.32E-21	0.1257155	0.105	0.054	3.18E-17	1.4	APTR	1.944444444
C1orf1861	1.45E-21	0.1026121	0.046	0.017	3.50E-17	1.4	C1orf186	2.705882353
JAK2	1.68E-21	0.3509327	0.159	0.097	4.05E-17	1.4	JAK2	1.639175258
PPIA3	1.85E-21	0.1405083	0.879	0.795	4.46E-17	1.4	PPIA	1.105660377
APOL1	1.92E-21	0.1162594	0.069	0.03	4.62E-17	1.4	APOL1	2.3
PID13	1.94E-21	0.2019652	0.127	0.07	4.67E-17	1.4	PID1	1.814285714
GNPTAB1	2.16E-21	0.176166	0.151	0.088	5.20E-17	1.4	GNPTAB	1.715909091
TCTN3	2.30E-21	0.1183847	0.072	0.032	5.54E-17	1.4	TCTN3	2.25
RAB27A1	2.55E-21	0.1442336	0.12	0.065	6.14E-17	1.4	RAB27A	1.846153846
CSTF31	2.71E-21	0.1705325	0.175	0.107	6.53E-17	1.4	CSTF3	1.635514019
LARS21	2.76E-21	0.1381187	0.074	0.034	6.65E-17	1.4	LARS2	2.176470588
USP162	2.98E-21	0.1345539	0.228	0.146	7.20E-17	1.4	USP16	1.561643836
ZNFX1	3.36E-21	0.2003209	0.194	0.122	8.09E-17	1.4	ZNFX1	1.590163934
CAPRIN11	3.44E-21	0.1628592	0.286	0.194	8.29E-17	1.4	CAPRIN1	1.474226804
DHTKD1	3.50E-21	0.1027446	0.065	0.028	8.45E-17	1.4	DHTKD1	2.321428571
DDX502	3.53E-21	0.1498355	0.138	0.078	8.51E-17	1.4	DDX50	1.769230769
DDX53	3.65E-21	0.240978	0.617	0.484	8.80E-17	1.4	DDX5	1.274793388
CEP951	3.66E-21	0.173525	0.202	0.127	8.83E-17	1.4	CEP95	1.590551181
SOAT12	3.97E-21	0.1229388	0.073	0.033	9.58E-17	1.4	SOAT1	2.212121212
NCK21	3.97E-21	0.2225131	0.204	0.131	9.58E-17	1.4	NCK2	1.557251908
ZFHX31	4.25E-21	0.1898234	0.181	0.112	1.03E-16	1.4	ZFHX3	1.616071429
ZNHIT31	4.63E-21	0.1471236	0.26	0.173	1.12E-16	1.4	ZNHIT3	1.502890173
SMURF23	5.31E-21	0.1974615	0.386	0.28	1.28E-16	1.4	SMURF2	1.378571429
LINC008873	5.32E-21	0.1044695	0.07	0.031	1.28E-16	1.4	LINC00887	2.258064516
CTPS2	5.56E-21	0.1532169	0.12	0.065	1.34E-16	1.4	CTPS2	1.846153846
CMIP3	5.65E-21	0.1734893	0.36	0.255	1.36E-16	1.4	CMIP	1.411764706
NASP1	5.84E-21	0.1739118	0.23	0.15	1.41E-16	1.4	NASP	1.533333333
DNAJC111	6.33E-21	0.123234	0.078	0.036	1.53E-16	1.4	DNAJC11	2.166666667
ATG10	6.51E-21	0.2398219	0.211	0.136	1.57E-16	1.4	ATG10	1.551470588
C11orf581	7.30E-21	0.1769423	0.372	0.266	1.76E-16	1.4	C11orf58	1.398496241
MRPS51	7.32E-21	0.1076506	0.085	0.041	1.76E-16	1.4	MRPS5	2.073170732

FDFT11	7.59E-21	0.2301158	0.408	0.303	1.83E-16	1.4	FDFT1	1.346534653
CCDC571	7.92E-21	0.1619253	0.099	0.051	1.91E-16	1.4	CCDC57	1.941176471
SRGN	9.31E-21	0.1401799	0.057	0.024	2.24E-16	1.4	SRGN	2.375
NONO3	9.58E-21	0.1545974	0.416	0.302	2.31E-16	1.4	NONO	1.377483444
DPF21	1.03E-20	0.1472284	0.135	0.077	2.49E-16	1.4	DPF2	1.753246753
PSMB73	1.08E-20	0.1363493	0.571	0.438	2.62E-16	1.4	PSMB7	1.303652968
VGLL42	1.18E-20	0.1908271	0.307	0.213	2.85E-16	1.4	VGLL4	1.441314554
MPP62	1.30E-20	0.140762	0.088	0.043	3.12E-16	1.4	MPP6	2.046511628
C7orf731	1.42E-20	0.1583426	0.165	0.1	3.43E-16	1.4	C7orf73	1.65
PC1	1.48E-20	0.1026588	0.051	0.02	3.57E-16	1.4	PC	2.55
SRSF52	1.58E-20	0.174049	0.403	0.293	3.80E-16	1.4	SRSF5	1.375426621
RP11-745L13.22	1.59E-20	0.129186	0.09	0.045	3.83E-16	1.4	RP11-745L1	2
C9orf851	1.60E-20	0.1509883	0.113	0.061	3.85E-16	1.4	C9orf85	1.852459016
RPL361	1.91E-20	0.4567692	0.274	0.191	4.62E-16	1.4	RPL36	1.434554974
PRDX62	2.06E-20	0.1289108	0.25	0.165	4.97E-16	1.4	PRDX6	1.515151515
UBAP22	2.29E-20	0.1804439	0.224	0.148	5.51E-16	1.4	UBAP2	1.513513514
TMLHE1	2.60E-20	0.1179442	0.088	0.044	6.28E-16	1.4	TMLHE	2
DMKN1	2.75E-20	0.132642	0.132	0.075	6.64E-16	1.4	DMKN	1.76
RBM221	2.78E-20	0.1372505	0.126	0.071	6.70E-16	1.4	RBM22	1.774647887
ACAT23	2.92E-20	0.1732481	0.28	0.192	7.04E-16	1.4	ACAT2	1.458333333
CNIH11	2.98E-20	0.1519546	0.333	0.234	7.19E-16	1.4	CNIH1	1.423076923
XKR61	3.20E-20	0.2150045	0.226	0.149	7.70E-16	1.4	XKR6	1.516778523
ACTR3C1	3.26E-20	0.1633757	0.094	0.048	7.85E-16	1.4	ACTR3C	1.958333333
VMP12	3.65E-20	0.2009818	0.841	0.735	8.80E-16	1.4	VMP1	1.144217687
SNX62	4.77E-20	0.1897469	0.412	0.306	1.15E-15	1.4	SNX6	1.346405229
NRDE21	4.83E-20	0.1057111	0.062	0.027	1.16E-15	1.4	NRDE2	2.296296296
LRRC4C	4.89E-20	0.1797793	0.099	0.052	1.18E-15	1.4	LRRC4C	1.903846154
ESR21	5.13E-20	0.1634187	0.165	0.1	1.24E-15	1.4	ESR2	1.65
ZCCHC172	5.24E-20	0.1725175	0.348	0.248	1.26E-15	1.4	ZCCHC17	1.403225806
ZNF638	5.40E-20	0.1754328	0.555	0.43	1.30E-15	1.4	ZNF638	1.290697674
MAX3	5.97E-20	0.1154207	0.201	0.127	1.44E-15	1.4	MAX	1.582677165
MRPS18C2	6.02E-20	0.1496722	0.218	0.141	1.45E-15	1.4	MRPS18C	1.546099291
ZNF902	6.07E-20	0.237381	0.129	0.074	1.46E-15	1.4	ZNF90	1.743243243
PDGFC2	6.58E-20	0.1673544	0.309	0.215	1.59E-15	1.4	PDGFC	1.437209302
CCDC1741	7.00E-20	0.1616093	0.25	0.167	1.69E-15	1.4	CCDC174	1.497005988
AKR1B11	7.07E-20	0.156527	0.086	0.043	1.71E-15	1.4	AKR1B1	2
IL152	7.10E-20	0.1282812	0.058	0.024	1.71E-15	1.4	IL15	2.416666667
SPOP1	7.31E-20	0.1191907	0.137	0.079	1.76E-15	1.4	SPOP	1.734177215
EEA13	7.44E-20	0.160946	0.189	0.119	1.79E-15	1.4	EEA1	1.588235294
INTS6-AS13	7.75E-20	0.1307373	0.184	0.115	1.87E-15	1.4	INTS6-AS1	1.6
SESN32	7.95E-20	0.175826	0.088	0.045	1.92E-15	1.4	SESN3	1.955555556
TEFM1	8.11E-20	0.1088567	0.074	0.035	1.96E-15	1.4	TEFM	2.114285714
NMNAT21	8.14E-20	0.2057221	0.05	0.02	1.96E-15	1.4	NMNAT2	2.5
ZNF1691	9.54E-20	0.1082502	0.049	0.019	2.30E-15	1.4	ZNF169	2.578947368
HLA-A1	1.01E-19	0.2170037	0.412	0.308	2.44E-15	1.4	HLA-A	1.337662338
GALK2	1.04E-19	0.1644308	0.124	0.07	2.52E-15	1.4	GALK2	1.771428571
KDM4C1	1.08E-19	0.1365408	0.107	0.058	2.59E-15	1.4	KDM4C	1.844827586
PHYKPL1	1.08E-19	0.1245941	0.078	0.038	2.60E-15	1.4	PHYKPL	2.052631579
ABCA31	1.11E-19	0.1199561	0.065	0.029	2.69E-15	1.4	ABCA3	2.24137931
ADCY31	1.15E-19	0.1088886	0.056	0.023	2.77E-15	1.4	ADCY3	2.434782609
FAXDC22	1.16E-19	0.1003412	0.054	0.022	2.80E-15	1.4	FAXDC2	2.454545455
PALLD2	1.20E-19	0.1772292	0.511	0.389	2.90E-15	1.4	PALLD	1.313624679

ROBO1	1.22E-19	0.1550969	0.114	0.063	2.93E-15	1.4	ROBO1	1.80952381
GCC21	1.25E-19	0.185865	0.398	0.296	3.02E-15	1.4	GCC2	1.344594595
TBC1D191	1.39E-19	0.1141424	0.049	0.019	3.36E-15	1.4	TBC1D19	2.578947368
EEF21	1.44E-19	0.3475186	0.503	0.399	3.47E-15	1.4	EEF2	1.260651629
HADH3	1.47E-19	0.1246307	0.162	0.098	3.54E-15	1.4	HADH	1.653061224
UBE2V22	2.47E-19	0.1836581	0.335	0.24	5.96E-15	1.4	UBE2V2	1.395833333
MRE11A1	2.75E-19	0.1431695	0.107	0.058	6.64E-15	1.4	MRE11A	1.844827586
SPDYE52	2.83E-19	0.1313505	0.103	0.055	6.83E-15	1.4	SPDYE5	1.872727273
DYM1	2.88E-19	0.1592894	0.311	0.218	6.95E-15	1.4	DYM	1.426605505
HMGXB4	2.92E-19	0.1663767	0.308	0.217	7.03E-15	1.4	HMGXB4	1.419354839
LNPEP1	3.08E-19	0.1513644	0.141	0.083	7.44E-15	1.4	LNPEP	1.698795181
ZADH2	3.18E-19	0.1090214	0.064	0.029	7.67E-15	1.4	ZADH2	2.206896552
TTN-AS11	3.59E-19	0.1363983	0.086	0.043	8.66E-15	1.4	TTN-AS1	2
SNAPC31	3.74E-19	0.1209162	0.116	0.065	9.03E-15	1.4	SNAPC3	1.784615385
TMEM14C1	3.98E-19	0.1232678	0.132	0.076	9.60E-15	1.4	TMEM14C	1.736842105
CTDSP21	4.43E-19	0.144966	0.247	0.166	1.07E-14	1.4	CTDSP2	1.487951807
FZD61	4.45E-19	0.1371497	0.085	0.043	1.07E-14	1.4	FZD6	1.976744186
ZNF5861	4.99E-19	0.1458137	0.154	0.093	1.20E-14	1.4	ZNF586	1.655913978
IFT741	5.11E-19	0.1277384	0.083	0.042	1.23E-14	1.4	IFT74	1.976190476
NFX11	5.15E-19	0.142068	0.22	0.144	1.24E-14	1.4	NFX1	1.527777778
NUP2141	5.51E-19	0.1444105	0.115	0.064	1.33E-14	1.4	NUP214	1.796875
EIF3D3	5.54E-19	0.1503477	0.425	0.316	1.34E-14	1.4	EIF3D	1.344936709
HDAC81	5.75E-19	0.1867952	0.327	0.235	1.39E-14	1.4	HDAC8	1.391489362
EZH11	6.24E-19	0.1431962	0.134	0.078	1.50E-14	1.4	EZH1	1.717948718
TMEM63A1	6.40E-19	0.1924811	0.101	0.055	1.54E-14	1.4	TMEM63A	1.836363636
DDI21	6.69E-19	0.1599399	0.151	0.092	1.61E-14	1.4	DDI2	1.641304348
ZNF321	7.41E-19	0.1101706	0.069	0.032	1.79E-14	1.4	ZNF32	2.15625
SENP6	9.52E-19	0.1622922	0.407	0.3	2.30E-14	1.4	SENP6	1.356666667
SEC31A2	9.93E-19	0.1488391	0.503	0.384	2.39E-14	1.4	SEC31A	1.309895833
CCDC1491	1.02E-18	0.1116925	0.054	0.023	2.46E-14	1.4	CCDC149	2.347826087
HM131	1.04E-18	0.1451468	0.194	0.124	2.50E-14	1.4	HM13	1.564516129
KIAA11091	1.04E-18	0.1618575	0.176	0.111	2.50E-14	1.4	KIAA1109	1.585585586
FOXK23	1.15E-18	0.1495932	0.303	0.212	2.77E-14	1.4	FOXK2	1.429245283
RFC13	1.19E-18	0.1266821	0.275	0.189	2.86E-14	1.4	RFC1	1.455026455
RBM51	1.22E-18	0.163331	0.218	0.144	2.94E-14	1.4	RBM5	1.513888889
PMM22	1.23E-18	0.1321584	0.119	0.067	2.96E-14	1.4	PMM2	1.776119403
GLG11	1.29E-18	0.1813844	0.266	0.184	3.11E-14	1.4	GLG1	1.445652174
ERGIC21	1.32E-18	0.174865	0.243	0.165	3.17E-14	1.4	ERGIC2	1.472727273
RP11-427J23.11	1.32E-18	0.1030162	0.047	0.018	3.17E-14	1.4	RP11-427J2	2.611111111
SNX242	1.38E-18	0.1479338	0.24	0.162	3.33E-14	1.4	SNX24	1.481481481
GPR841	1.41E-18	0.1573465	0.013	0.002	3.41E-14	1.4	GPR84	6.5
SMARCA41	1.45E-18	0.1690506	0.193	0.125	3.49E-14	1.4	SMARCA4	1.544
CSNK1E2	1.52E-18	0.1591928	0.307	0.219	3.67E-14	1.4	CSNK1E	1.401826484
ZMYM4-AS11	1.58E-18	0.1011089	0.07	0.033	3.80E-14	1.4	ZMYM4-AS	2.121212121
RFX71	1.59E-18	0.169486	0.177	0.112	3.85E-14	1.4	RFX7	1.580357143
MPZL22	1.63E-18	0.1401847	0.134	0.079	3.94E-14	1.4	MPZL2	1.696202532
AC007566.101	1.71E-18	0.1114716	0.077	0.038	4.13E-14	1.4	AC007566.	2.026315789
CEP112	1.73E-18	0.108465	0.069	0.032	4.16E-14	1.4	CEP112	2.15625
HAUS61	1.78E-18	0.1065568	0.06	0.027	4.29E-14	1.4	HAUS6	2.222222222
TUG11	1.90E-18	0.1416082	0.167	0.104	4.58E-14	1.4	TUG1	1.605769231
TECPR21	1.92E-18	0.1278934	0.07	0.033	4.64E-14	1.4	TECPR2	2.121212121
SUPT20H	2.20E-18	0.1302535	0.129	0.075	5.30E-14	1.4	SUPT20H	1.72

PHB21	2.26E-18	0.112169	0.144	0.085	5.46E-14	1.4	PHB2	1.694117647
CTD-2337A12.1	2.27E-18	0.2587924	0.272	0.191	5.48E-14	1.4	CTD-2337A	1.42408377
GLMN1	2.39E-18	0.1317023	0.086	0.044	5.77E-14	1.4	GLMN	1.954545455
MVP1	2.48E-18	0.1198407	0.105	0.058	5.98E-14	1.4	MVP	1.810344828
VPS531	2.69E-18	0.1576969	0.304	0.214	6.48E-14	1.4	VPS53	1.420560748
WDR11	2.87E-18	0.1427547	0.136	0.08	6.91E-14	1.4	WDR11	1.7
TYW31	2.91E-18	0.1226146	0.101	0.055	7.02E-14	1.4	TYW3	1.836363636
NUPL21	3.26E-18	0.1048324	0.095	0.05	7.86E-14	1.4	NUPL2	1.9
ATXN7L11	3.46E-18	0.1383797	0.107	0.059	8.35E-14	1.4	ATXN7L1	1.813559322
GATAD1	3.62E-18	0.1143948	0.073	0.035	8.74E-14	1.4	GATAD1	2.085714286
ATN11	3.79E-18	0.1195349	0.083	0.042	9.13E-14	1.4	ATN1	1.976190476
PDLIM31	3.89E-18	0.1718257	0.091	0.048	9.39E-14	1.4	PDLIM3	1.895833333
UBLCP11	3.93E-18	0.1179326	0.1	0.054	9.47E-14	1.4	UBLCP1	1.851851852
TMEM165	3.94E-18	0.1226089	0.633	0.508	9.51E-14	1.4	TMEM165	1.246062992
C15orf411	3.97E-18	0.1363287	0.089	0.047	9.57E-14	1.4	C15orf41	1.893617021
DPP8	4.38E-18	0.1721995	0.142	0.086	1.06E-13	1.4	DPP8	1.651162791
SPNS21	4.56E-18	0.1508402	0.092	0.049	1.10E-13	1.4	SPNS2	1.87755102
PPP2R5C2	4.65E-18	0.1622723	0.24	0.163	1.12E-13	1.4	PPP2R5C	1.472392638
NEK11	4.72E-18	0.1543757	0.121	0.07	1.14E-13	1.4	NEK1	1.728571429
HLA-E2	4.79E-18	0.1823802	0.142	0.086	1.15E-13	1.4	HLA-E	1.651162791
MCPH11	5.83E-18	0.1891669	0.243	0.168	1.41E-13	1.4	MCPH1	1.446428571
ZNF7211	6.59E-18	0.1963313	0.262	0.182	1.59E-13	1.4	ZNF721	1.43956044
PPL2	6.87E-18	0.1146873	0.114	0.065	1.66E-13	1.4	PPL	1.753846154
RP11-849H4.21	7.39E-18	0.1001593	0.053	0.023	1.78E-13	1.4	RP11-849H	2.304347826
NNMT	9.17E-18	0.2505632	0.162	0.102	2.21E-13	1.4	NNMT	1.588235294
TNS31	9.36E-18	0.1935199	0.114	0.065	2.26E-13	1.4	TNS3	1.753846154
GALNT111	9.49E-18	0.15052	0.131	0.078	2.29E-13	1.4	GALNT11	1.679487179
PMPCB1	9.83E-18	0.1163496	0.085	0.044	2.37E-13	1.4	PMPCB	1.931818182
CASP81	1.13E-17	0.1191456	0.075	0.037	2.72E-13	1.4	CASP8	2.027027027
NOL31	1.31E-17	0.1247499	0.078	0.04	3.15E-13	1.4	NOL3	1.95
TATDN3	1.36E-17	0.1291524	0.059	0.027	3.29E-13	1.4	TATDN3	2.185185185
PHF5A2	1.39E-17	0.1283544	0.222	0.149	3.35E-13	1.4	PHF5A	1.489932886
NDUFAF71	1.44E-17	0.1185463	0.099	0.054	3.47E-13	1.4	NDUFAF7	1.833333333
PXDN2	1.45E-17	0.1156942	0.144	0.087	3.49E-13	1.4	PXDN	1.655172414
MT2A1	1.47E-17	0.5274459	0.1	0.055	3.54E-13	1.4	MT2A	1.818181818
CEP1701	1.50E-17	0.1197039	0.116	0.066	3.62E-13	1.4	CEP170	1.757575758
RP11-499P20.21	1.57E-17	0.1096472	0.071	0.035	3.79E-13	1.4	RP11-499P.	2.028571429
TFDP21	1.61E-17	0.1355694	0.277	0.195	3.89E-13	1.4	TFDP2	1.420512821
CBR31	1.72E-17	0.1224717	0.055	0.024	4.15E-13	1.4	CBR3	2.291666667
SLC25A251	1.75E-17	0.1984786	0.178	0.116	4.23E-13	1.4	SLC25A25	1.534482759
VIM3	1.76E-17	0.111904	0.183	0.117	4.24E-13	1.4	VIM	1.564102564
HADHA2	1.85E-17	0.1472925	0.371	0.272	4.46E-13	1.4	HADHA	1.363970588
EPS152	1.85E-17	0.181375	0.276	0.196	4.47E-13	1.4	EPS15	1.408163265
PPP1R12B1	1.87E-17	0.1996867	0.295	0.211	4.50E-13	1.4	PPP1R12B	1.398104265
NEO1	1.88E-17	0.1596336	0.15	0.092	4.54E-13	1.4	NEO1	1.630434783
SLC30A71	2.11E-17	0.1803171	0.18	0.116	5.10E-13	1.4	SLC30A7	1.551724138
WDR701	2.16E-17	0.179072	0.208	0.139	5.21E-13	1.4	WDR70	1.496402878
WDR71	2.17E-17	0.1449651	0.091	0.048	5.24E-13	1.4	WDR7	1.895833333
DRG12	2.27E-17	0.129826	0.247	0.169	5.47E-13	1.4	DRG1	1.461538462
TRAF3IP2-AS11	2.28E-17	0.1328132	0.154	0.096	5.51E-13	1.4	TRAF3IP2-A	1.604166667
ZBTB46	2.36E-17	0.1180372	0.099	0.055	5.70E-13	1.4	ZBTB46	1.8
AASDH1	2.50E-17	0.1030757	0.093	0.05	6.03E-13	1.4	AASDH	1.86

TUBD11	2.58E-17	0.104843	0.073	0.036	6.21E-13	1.4	TUBD1	2.027777778
DAB2IP3	2.83E-17	0.144897	0.129	0.077	6.82E-13	1.4	DAB2IP	1.675324675
VPS361	2.89E-17	0.1409015	0.157	0.098	6.98E-13	1.4	VPS36	1.602040816
PGS11	3.04E-17	0.10465	0.036	0.013	7.33E-13	1.4	PGS1	2.769230769
HIST1H4C3	3.15E-17	0.1722294	0.233	0.16	7.59E-13	1.4	HIST1H4C	1.45625
ARRDC3-AS12	3.32E-17	0.1300866	0.064	0.03	8.02E-13	1.4	ARRDC3-AS	2.133333333
VDAC33	3.34E-17	0.1337614	0.354	0.258	8.05E-13	1.4	VDAC3	1.372093023
GREB12	3.86E-17	0.2274356	0.115	0.067	9.31E-13	1.4	GREB1	1.71641791
HERC21	3.93E-17	0.1570514	0.117	0.068	9.48E-13	1.4	HERC2	1.720588235
SERPINE21	4.05E-17	0.1427718	0.072	0.036	9.77E-13	1.4	SERPINE2	2
ERAP12	4.77E-17	0.1017388	0.111	0.063	1.15E-12	1.4	ERAP1	1.761904762
PIK3C2G2	5.02E-17	0.1908615	0.105	0.06	1.21E-12	1.4	PIK3C2G	1.75
ZFAND13	5.21E-17	0.1206023	0.253	0.175	1.26E-12	1.4	ZFAND1	1.445714286
INTS31	5.26E-17	0.1111066	0.093	0.051	1.27E-12	1.4	INTS3	1.823529412
GGA22	5.74E-17	0.1529155	0.159	0.1	1.38E-12	1.4	GGA2	1.59
TEAD21	5.75E-17	0.1215634	0.12	0.07	1.39E-12	1.4	TEAD2	1.714285714
MCF2L23	5.93E-17	0.175153	0.15	0.094	1.43E-12	1.4	MCF2L2	1.595744681
BRCC31	6.05E-17	0.1040147	0.091	0.048	1.46E-12	1.4	BRCC3	1.895833333
PSMC12	6.09E-17	0.1351501	0.365	0.269	1.47E-12	1.4	PSMC1	1.356877323
MFF	7.19E-17	0.1160838	0.124	0.073	1.73E-12	1.4	MFF	1.698630137
C5orf45	7.20E-17	0.1260667	0.068	0.033	1.74E-12	1.4	C5orf45	2.060606061
FAM120B1	8.09E-17	0.1578993	0.176	0.114	1.95E-12	1.4	FAM120B	1.543859649
CFDP11	8.23E-17	0.1794388	0.242	0.169	1.98E-12	1.4	CFDP1	1.431952663
CLK4	8.30E-17	0.1294161	0.115	0.066	2.00E-12	1.4	CLK4	1.742424242
PSMB21	9.25E-17	0.127746	0.179	0.115	2.23E-12	1.4	PSMB2	1.556521739
SCLT1	1.01E-16	0.1678418	0.089	0.048	2.44E-12	1.4	SCLT1	1.854166667
PDGFRL2	1.17E-16	0.2695004	0.082	0.044	2.82E-12	1.4	PDGFRL	1.863636364
PLAGL13	1.23E-16	0.1026368	0.072	0.036	2.96E-12	1.4	PLAGL1	2
N4BP11	1.33E-16	0.1657911	0.245	0.171	3.21E-12	1.4	N4BP1	1.432748538
VPS13C1	1.51E-16	0.1559146	0.321	0.234	3.65E-12	1.4	VPS13C	1.371794872
HYDIN	1.70E-16	0.1332937	0.052	0.023	4.09E-12	1.4	HYDIN	2.260869565
TMEM51-AS12	1.81E-16	0.1014235	0.045	0.019	4.35E-12	1.4	TMEM51-A	2.368421053
GTF2H51	1.82E-16	0.1112924	0.293	0.207	4.39E-12	1.4	GTF2H5	1.415458937
RPS282	2.01E-16	0.3580799	0.131	0.081	4.84E-12	1.4	RPS28	1.617283951
LINC015151	2.02E-16	0.1087695	0.063	0.03	4.86E-12	1.4	LINC01515	2.1
CCT83	2.08E-16	0.1353854	0.369	0.272	5.00E-12	1.4	CCT8	1.356617647
COPS42	2.15E-16	0.1241796	0.156	0.098	5.19E-12	1.4	COPS4	1.591836735
LYRM21	2.24E-16	0.1313275	0.149	0.094	5.40E-12	1.4	LYRM2	1.585106383
RPL383	2.72E-16	0.1290255	0.927	0.887	6.57E-12	1.4	RPL38	1.045095829
VPS511	2.87E-16	0.1224345	0.083	0.044	6.92E-12	1.4	VPS51	1.886363636
OXCT11	2.87E-16	0.1093927	0.06	0.028	6.93E-12	1.4	OXCT1	2.142857143
TCAF22	3.17E-16	0.1647561	0.148	0.093	7.65E-12	1.4	TCAF2	1.591397849
SUPT4H11	3.25E-16	0.1479276	0.346	0.255	7.84E-12	1.4	SUPT4H1	1.356862745
CLEC16A	3.28E-16	0.1429275	0.222	0.151	7.90E-12	1.4	CLEC16A	1.470198675
SOX63	3.29E-16	0.1601821	0.13	0.079	7.93E-12	1.4	SOX6	1.64556962
ZNF6211	3.30E-16	0.109436	0.083	0.044	7.96E-12	1.4	ZNF621	1.886363636
RFWD21	3.60E-16	0.1611377	0.387	0.291	8.69E-12	1.4	RFWD2	1.329896907
MCTP11	3.88E-16	0.2142356	0.148	0.094	9.35E-12	1.4	MCTP1	1.574468085
PAIP12	3.93E-16	0.1385431	0.22	0.15	9.47E-12	1.4	PAIP1	1.466666667
NEDD12	4.01E-16	0.1207159	0.096	0.054	9.66E-12	1.4	NEDD1	1.777777778
ATXN101	4.11E-16	0.1475914	0.207	0.14	9.91E-12	1.4	ATXN10	1.478571429
IRAK21	4.37E-16	0.1397534	0.185	0.122	1.05E-11	1.4	IRAK2	1.516393443

RP11-415J8.31	4.79E-16	0.1377707	0.087	0.047	1.15E-11	1.4	RP11-415J8	1.85106383
KEAP11	4.91E-16	0.1786759	0.057	0.027	1.18E-11	1.4	KEAP1	2.111111111
USP33	5.03E-16	0.1615051	0.299	0.216	1.21E-11	1.4	USP33	1.384259259
GIT22	5.35E-16	0.1169944	0.098	0.055	1.29E-11	1.4	GIT2	1.781818182
ARL151	5.68E-16	0.1772806	0.278	0.2	1.37E-11	1.4	ARL15	1.39
PUM33	5.81E-16	0.1389823	0.237	0.163	1.40E-11	1.4	PUM3	1.45398773
XPO53	6.13E-16	0.1351313	0.145	0.091	1.48E-11	1.4	XPO5	1.593406593
GNAQ1	6.30E-16	0.1576414	0.398	0.3	1.52E-11	1.4	GNAQ	1.326666667
AGAP91	6.61E-16	0.1056852	0.036	0.014	1.59E-11	1.4	AGAP9	2.571428571
ADPRM3	6.86E-16	0.1132642	0.103	0.059	1.65E-11	1.4	ADPRM	1.745762712
FBXW4	6.87E-16	0.137911	0.134	0.082	1.66E-11	1.4	FBXW4	1.634146341
ALOX12-AS11	7.25E-16	0.1288894	0.104	0.06	1.75E-11	1.4	ALOX12-AS	1.733333333
CFL23	7.50E-16	0.1080977	0.119	0.07	1.81E-11	1.4	CFL2	1.7
IGFL11	7.52E-16	0.1042252	0.019	0.005	1.81E-11	1.4	IGFL1	3.8
PURA	7.87E-16	0.1019599	0.11	0.064	1.90E-11	1.4	PURA	1.71875
ALOX52	8.03E-16	0.1680279	0.161	0.104	1.94E-11	1.4	ALOX5	1.548076923
GTF2B2	8.19E-16	0.121707	0.237	0.163	1.98E-11	1.4	GTF2B	1.45398773
CLTA1	8.76E-16	0.1333805	0.213	0.145	2.11E-11	1.4	CLTA	1.468965517
SMARCAD11	9.54E-16	0.1159935	0.1	0.057	2.30E-11	1.4	SMARCAD1	1.754385965
WARS1	9.74E-16	0.1474772	0.108	0.063	2.35E-11	1.4	WARS	1.714285714
OFD13	9.91E-16	0.1404497	0.327	0.242	2.39E-11	1.4	OFD1	1.351239669
ZZZ31	9.92E-16	0.1775136	0.236	0.165	2.39E-11	1.4	ZZZ3	1.43030303
CDH32	1.04E-15	0.1500095	0.177	0.117	2.50E-11	1.4	CDH3	1.512820513
FAF21	1.07E-15	0.1631086	0.223	0.154	2.58E-11	1.4	FAF2	1.448051948
ZNF718	1.17E-15	0.1277555	0.105	0.061	2.81E-11	1.4	ZNF718	1.721311475
RPL152	1.21E-15	0.3039601	0.815	0.774	2.92E-11	1.4	RPL15	1.052971576
AGFG12	1.33E-15	0.1321857	0.412	0.311	3.21E-11	1.4	AGFG1	1.324758842
ZNF1411	1.33E-15	0.1170138	0.091	0.05	3.21E-11	1.4	ZNF141	1.82
PTGFRN1	1.41E-15	0.1447073	0.148	0.094	3.41E-11	1.4	PTGFRN	1.574468085
RP11-123O10.41	1.57E-15	0.1562561	0.284	0.204	3.78E-11	1.4	RP11-123O	1.392156863
S100A133	1.66E-15	0.1239278	0.621	0.501	4.01E-11	1.4	S100A13	1.239520958
ZDHHC171	1.66E-15	0.1515676	0.122	0.074	4.01E-11	1.4	ZDHHC17	1.648648649
SLC25A432	2.08E-15	0.1034196	0.068	0.034	5.01E-11	1.4	SLC25A43	2
KRBOX41	2.32E-15	0.131052	0.068	0.035	5.60E-11	1.4	KRBOX4	1.942857143
ARL17B	2.33E-15	0.200342	0.117	0.07	5.62E-11	1.4	ARL17B	1.671428571
FARS21	2.47E-15	0.1637679	0.129	0.08	5.97E-11	1.4	FARS2	1.6125
LRIF11	2.62E-15	0.1061039	0.106	0.062	6.31E-11	1.4	LRIF1	1.709677419
GAPDH1	2.65E-15	0.2271625	0.657	0.555	6.38E-11	1.4	GAPDH	1.183783784
SCOC1	3.00E-15	0.1281007	0.317	0.232	7.25E-11	1.4	SCOC	1.36637931
DAB1	3.42E-15	0.1262769	0.094	0.054	8.24E-11	1.4	DAB1	1.740740741
MMS191	3.44E-15	0.106166	0.076	0.04	8.29E-11	1.4	MMS19	1.9
S100PBP1	3.71E-15	0.1448038	0.159	0.103	8.95E-11	1.4	S100PBP	1.54368932
PLCH11	3.91E-15	0.1052101	0.058	0.028	9.43E-11	1.4	PLCH1	2.071428571
GK51	3.97E-15	0.1067942	0.085	0.047	9.58E-11	1.4	GK5	1.808510638
TMEM512	3.99E-15	0.1255935	0.211	0.144	9.63E-11	1.4	TMEM51	1.465277778
TSTD2	4.23E-15	0.1024792	0.083	0.045	1.02E-10	1.4	TSTD2	1.844444444
LRRK12	4.30E-15	0.1256154	0.093	0.052	1.04E-10	1.4	LRRK1	1.788461538
OVOL21	4.32E-15	0.1063455	0.103	0.059	1.04E-10	1.4	OVOL2	1.745762712
APBA2	4.81E-15	0.1136249	0.093	0.052	1.16E-10	1.4	APBA2	1.788461538
HIST2H2BF1	4.91E-15	0.1038681	0.063	0.032	1.18E-10	1.4	HIST2H2BF	1.96875
ZCCHC23	5.36E-15	0.156326	0.301	0.221	1.29E-10	1.4	ZCCHC2	1.36199095
KMT5B1	6.15E-15	0.1413861	0.224	0.155	1.48E-10	1.4	KMT5B	1.44516129

RBM61	6.15E-15	0.1498612	0.432	0.329	1.48E-10	1.4	RBM6	1.313069909
SRD5A3	6.79E-15	0.104775	0.112	0.066	1.64E-10	1.4	SRD5A3	1.696969697
PHF20L1	7.64E-15	0.1564201	0.176	0.117	1.84E-10	1.4	PHF20L1	1.504273504
CCDC90B1	8.17E-15	0.1151661	0.132	0.082	1.97E-10	1.4	CCDC90B	1.609756098
PRPF40B1	8.62E-15	0.1242082	0.058	0.028	2.08E-10	1.4	PRPF40B	2.071428571
ANXA4	9.28E-15	0.118042	0.158	0.102	2.24E-10	1.4	ANXA4	1.549019608
GATA62	9.55E-15	0.1017523	0.069	0.036	2.30E-10	1.4	GATA6	1.916666667
SCMH11	9.86E-15	0.1330785	0.314	0.231	2.38E-10	1.4	SCMH1	1.359307359
STAT2	1.04E-14	0.1435094	0.13	0.081	2.50E-10	1.4	STAT2	1.604938272
EPS8L21	1.04E-14	0.1576817	0.052	0.024	2.50E-10	1.4	EPS8L2	2.166666667
SMYD41	1.04E-14	0.1219692	0.05	0.023	2.51E-10	1.4	SMYD4	2.173913043
PAXBP1	1.13E-14	0.1263877	0.14	0.089	2.73E-10	1.4	PAXBP1	1.573033708
RAD54L21	1.19E-14	0.1100165	0.068	0.035	2.88E-10	1.4	RAD54L2	1.942857143
TIPRL1	1.22E-14	0.1070215	0.099	0.057	2.94E-10	1.4	TIPRL	1.736842105
SETDB11	1.24E-14	0.1300207	0.121	0.074	2.98E-10	1.4	SETDB1	1.635135135
COMMD62	1.36E-14	0.1271855	0.443	0.345	3.29E-10	1.4	COMMD6	1.284057971
C11orf541	1.40E-14	0.1313216	0.152	0.099	3.38E-10	1.4	C11orf54	1.535353535
ZNF248	1.51E-14	0.1336	0.09	0.051	3.64E-10	1.4	ZNF248	1.764705882
LHFP1	1.55E-14	0.1247335	0.058	0.029	3.73E-10	1.4	LHFP	2
STAMBP1	1.55E-14	0.1368638	0.225	0.157	3.74E-10	1.4	STAMBP	1.433121019
MAPRE21	1.57E-14	0.1692438	0.181	0.122	3.79E-10	1.4	MAPRE2	1.483606557
RNF131	1.63E-14	0.1497521	0.351	0.264	3.93E-10	1.4	RNF13	1.329545455
SLC30A61	1.83E-14	0.1174883	0.091	0.052	4.40E-10	1.4	SLC30A6	1.75
MTERF1	1.89E-14	0.1325023	0.128	0.08	4.55E-10	1.4	MTERF1	1.6
SH3PXD2A2	2.03E-14	0.10208	0.145	0.093	4.89E-10	1.4	SH3PXD2A	1.559139785
PRKAG11	2.04E-14	0.1095153	0.197	0.134	4.93E-10	1.4	PRKAG1	1.470149254
CIRBP1	2.35E-14	0.1285536	0.067	0.035	5.66E-10	1.4	CIRBP	1.914285714
UGP22	2.45E-14	0.1461833	0.532	0.424	5.90E-10	1.4	UGP2	1.254716981
TTC171	2.56E-14	0.1553818	0.376	0.287	6.17E-10	1.4	TTC17	1.31010453
DDX521	2.80E-14	0.1228211	0.151	0.097	6.76E-10	1.4	DDX52	1.556701031
R3HDM11	2.82E-14	0.1142055	0.101	0.059	6.79E-10	1.4	R3HDM1	1.711864407
TRNAU1AP2	2.83E-14	0.1163633	0.305	0.223	6.82E-10	1.4	TRNAU1AP	1.367713004
DMGDH	2.91E-14	0.1124283	0.08	0.044	7.02E-10	1.4	DMGDH	1.818181818
SSBP12	3.04E-14	0.1226486	0.45	0.35	7.33E-10	1.4	SSBP1	1.285714286
FAM196A1	3.04E-14	0.1210705	0.099	0.058	7.34E-10	1.4	FAM196A	1.706896552
ZNF2741	3.04E-14	0.1255724	0.133	0.083	7.34E-10	1.4	ZNF274	1.602409639
PWWP2A1	3.10E-14	0.1120183	0.093	0.054	7.47E-10	1.4	PWWP2A	1.722222222
DIS32	3.13E-14	0.1270137	0.106	0.064	7.54E-10	1.4	DIS3	1.65625
TMTC21	3.22E-14	0.1620982	0.47	0.368	7.76E-10	1.4	TMTC2	1.277173913
KDM1B1	3.34E-14	0.1044509	0.088	0.05	8.04E-10	1.4	KDM1B	1.76
DEK2	3.53E-14	0.1129579	0.26	0.187	8.50E-10	1.4	DEK	1.390374332
ZNF2131	3.69E-14	0.1036427	0.049	0.023	8.91E-10	1.4	ZNF213	2.130434783
PYGB1	3.87E-14	0.106715	0.063	0.032	9.34E-10	1.4	PYGB	1.96875
RP11-530C5.11	4.07E-14	0.125948	0.06	0.03	9.81E-10	1.4	RP11-530C	2
PCBP22	4.39E-14	0.1498209	0.665	0.559	1.06E-09	1.4	PCBP2	1.189624329
AGBL51	5.56E-14	0.1005445	0.071	0.038	1.34E-09	1.4	AGBL5	1.868421053
RP11-382A20.51	5.56E-14	0.1166475	0.088	0.05	1.34E-09	1.4	RP11-382A	1.76
SRSF71	5.57E-14	0.1339782	0.334	0.25	1.34E-09	1.4	SRSF7	1.336
PMS11	5.58E-14	0.1069008	0.081	0.045	1.35E-09	1.4	PMS1	1.8
ERH1	5.87E-14	0.1064577	0.617	0.492	1.42E-09	1.4	ERH	1.254065041
IL4R	6.28E-14	0.1228597	0.198	0.136	1.51E-09	1.4	IL4R	1.455882353
CENPO2	6.38E-14	0.176768	0.037	0.015	1.54E-09	1.4	CENPO	2.466666667

MAK	6.44E-14	0.1336939	0.135	0.086	1.55E-09	1.4	MAK	1.569767442
RP11-417F21.12	6.51E-14	0.1167528	0.141	0.091	1.57E-09	1.4	RP11-417F:	1.549450549
TTF11	7.55E-14	0.1004376	0.167	0.11	1.82E-09	1.4	TTF1	1.518181818
GSTO21	7.56E-14	0.1020968	0.142	0.091	1.82E-09	1.4	GSTO2	1.56043956
KIAA11472	7.66E-14	0.1106263	0.101	0.06	1.85E-09	1.4	KIAA1147	1.683333333
STX72	8.28E-14	0.1212031	0.174	0.117	2.00E-09	1.4	STX7	1.487179487
TMC4	8.28E-14	0.1147471	0.063	0.032	2.00E-09	1.4	TMC4	1.96875
ITGB3BP2	8.82E-14	0.123012	0.121	0.075	2.13E-09	1.4	ITGB3BP	1.613333333
HIST2H2BE	9.15E-14	0.1559692	0.16	0.106	2.21E-09	1.4	HIST2H2BE	1.509433962
RUFY21	9.73E-14	0.100698	0.112	0.068	2.35E-09	1.4	RUFY2	1.647058824
YLPM11	1.00E-13	0.1391763	0.177	0.12	2.42E-09	1.4	YLPM1	1.475
SNX10	1.02E-13	0.1087633	0.109	0.066	2.47E-09	1.4	SNX10	1.651515152
RDX3	1.09E-13	0.1034342	0.216	0.15	2.62E-09	1.4	RDX	1.44
MRPS272	1.12E-13	0.1078463	0.103	0.062	2.69E-09	1.4	MRPS27	1.661290323
RPL182	1.17E-13	0.2484909	0.133	0.086	2.83E-09	1.4	RPL18	1.546511628
RBPJ1	1.26E-13	0.1274832	0.437	0.341	3.04E-09	1.4	RBPJ	1.281524927
RP5-1101C3.1	1.27E-13	0.1175986	0.095	0.056	3.06E-09	1.4	RP5-1101C	1.696428571
CMTM73	1.30E-13	0.1282559	0.216	0.152	3.14E-09	1.4	CMTM7	1.421052632
LTA4H3	1.31E-13	0.1280998	0.123	0.077	3.15E-09	1.4	LTA4H	1.597402597
CELF12	1.35E-13	0.1426974	0.377	0.288	3.25E-09	1.4	CELF1	1.309027778
RPS182	1.40E-13	0.3211685	0.688	0.63	3.37E-09	1.4	RPS18	1.092063492
SLC33A11	1.41E-13	0.1170137	0.103	0.062	3.40E-09	1.4	SLC33A1	1.661290323
RPL37A3	1.48E-13	0.1684879	0.975	0.975	3.56E-09	1.4	RPL37A	1
CNDP2	1.50E-13	0.1171546	0.177	0.12	3.63E-09	1.4	CNDP2	1.475
TMEM87A	1.73E-13	0.1652997	0.468	0.376	4.16E-09	1.4	TMEM87A	1.244680851
BRK13	1.82E-13	0.1374732	0.703	0.595	4.38E-09	1.4	BRK1	1.181512605
TRIB21	1.92E-13	0.105127	0.088	0.051	4.64E-09	1.4	TRIB2	1.725490196
PTCHD13	2.11E-13	0.1082875	0.07	0.038	5.08E-09	1.4	PTCHD1	1.842105263
IRAK1BP1	2.33E-13	0.1370987	0.11	0.067	5.61E-09	1.4	IRAK1BP1	1.641791045
RP11-795H16.33	2.38E-13	0.2056437	0.434	0.353	5.74E-09	1.4	RP11-795H	1.229461756
ALDH7A11	2.40E-13	0.1193394	0.124	0.078	5.78E-09	1.4	ALDH7A1	1.58974359
RPS292	2.63E-13	0.2314914	0.961	0.92	6.33E-09	1.4	RPS29	1.044565217
CNIH42	3.49E-13	0.1324115	0.378	0.292	8.41E-09	1.4	CNIH4	1.294520548
SGCD2	3.79E-13	0.1168191	0.073	0.04	9.13E-09	1.4	SGCD	1.825
SLC10A71	3.79E-13	0.119636	0.088	0.051	9.14E-09	1.4	SLC10A7	1.725490196
ZNF720	4.07E-13	0.1023806	0.128	0.081	9.83E-09	1.4	ZNF720	1.580246914
DNHD12	4.08E-13	0.1176363	0.089	0.052	9.83E-09	1.4	DNHD1	1.711538462
NMT11	4.12E-13	0.1168744	0.247	0.18	9.94E-09	1.4	NMT1	1.372222222
SORL11	4.22E-13	0.1039797	0.104	0.063	1.02E-08	1.4	SORL1	1.650793651
RP11-840I19.33	4.23E-13	0.1208592	0.096	0.057	1.02E-08	1.4	RP11-840I1	1.684210526
RAD9A1	4.33E-13	0.1117816	0.155	0.103	1.05E-08	1.4	RAD9A	1.504854369
MAPK1IP1L2	4.84E-13	0.1030356	0.227	0.162	1.17E-08	1.4	MAPK1IP1L	1.401234568
HMG20A	4.88E-13	0.1002017	0.102	0.062	1.18E-08	1.4	HMG20A	1.64516129
SVIL-AS12	5.28E-13	0.1140632	0.15	0.099	1.27E-08	1.4	SVIL-AS1	1.515151515
ATP1A11	5.32E-13	0.1264567	0.612	0.493	1.28E-08	1.4	ATP1A1	1.24137931
USO12	5.46E-13	0.1343743	0.223	0.158	1.32E-08	1.4	USO1	1.411392405
PAPD4	5.69E-13	0.1258154	0.392	0.305	1.37E-08	1.4	PAPD4	1.285245902
CDC14B2	5.70E-13	0.1086649	0.103	0.062	1.37E-08	1.4	CDC14B	1.661290323
PHIP	6.38E-13	0.1396503	0.538	0.43	1.54E-08	1.4	PHIP	1.251162791
IRF2BPL1	7.02E-13	0.1036118	0.222	0.157	1.69E-08	1.4	IRF2BPL	1.414012739
SIPA1L11	8.38E-13	0.106672	0.476	0.374	2.02E-08	1.4	SIPA1L1	1.272727273
MED282	8.47E-13	0.1138049	0.121	0.077	2.04E-08	1.4	MED28	1.571428571

ANKRD36B2	8.51E-13	0.1445238	0.113	0.071	2.05E-08	1.4	ANKRD36B	1.591549296
TRIP41	9.54E-13	0.1162648	0.073	0.041	2.30E-08	1.4	TRIP4	1.780487805
UVRAG	1.13E-12	0.1185252	0.563	0.454	2.73E-08	1.4	UVRAG	1.240088106
FKBP5	1.31E-12	0.1749558	0.413	0.328	3.16E-08	1.4	FKBP5	1.259146341
XRRA12	1.32E-12	0.1069089	0.146	0.097	3.18E-08	1.4	XRRA1	1.505154639
WDR45B3	1.44E-12	0.1284476	0.281	0.208	3.47E-08	1.4	WDR45B	1.350961538
CEP70	1.44E-12	0.1056816	0.113	0.071	3.48E-08	1.4	CEP70	1.591549296
OCIAD11	1.62E-12	0.1051115	0.331	0.25	3.91E-08	1.4	OCIAD1	1.324
DCAF101	1.63E-12	0.1165688	0.247	0.18	3.93E-08	1.4	DCAF10	1.372222222
CEP57	1.69E-12	0.1127291	0.141	0.093	4.08E-08	1.4	CEP57	1.516129032
DENND4C	1.74E-12	0.1292628	0.286	0.212	4.19E-08	1.4	DENND4C	1.349056604
WDR411	1.91E-12	0.1039972	0.13	0.085	4.60E-08	1.4	WDR41	1.529411765
FAM179B	1.97E-12	0.1115544	0.126	0.081	4.74E-08	1.4	FAM179B	1.555555556
DEGS12	2.06E-12	0.1208411	0.096	0.058	4.98E-08	1.4	DEGS1	1.655172414
BIRC6-AS22	2.07E-12	0.1069593	0.269	0.198	5.00E-08	1.4	BIRC6-AS2	1.358585859
HEATR4	2.15E-12	0.1145633	0.053	0.027	5.17E-08	1.4	HEATR4	1.962962963
GOLGA8A1	2.19E-12	0.1200873	0.059	0.031	5.29E-08	1.4	GOLGA8A	1.903225806
LINC008941	2.26E-12	0.1025664	0.096	0.058	5.44E-08	1.4	LINC00894	1.655172414
CDC42SE21	2.46E-12	0.120856	0.272	0.202	5.94E-08	1.4	CDC42SE2	1.346534653
LHFPL33	2.98E-12	0.1027552	0.331	0.253	7.20E-08	1.4	LHFPL3	1.308300395
IPMK2	3.14E-12	0.11628	0.132	0.087	7.58E-08	1.4	IPMK	1.517241379
TCAIM1	3.40E-12	0.1092431	0.095	0.058	8.19E-08	1.4	TCAIM	1.637931034
RPS151	3.40E-12	0.3411496	0.192	0.138	8.20E-08	1.4	RPS15	1.391304348
AC015849.191	3.45E-12	0.1088557	0.053	0.027	8.31E-08	1.4	AC015849.	1.962962963
FANCC1	3.45E-12	0.1323935	0.178	0.124	8.31E-08	1.4	FANCC	1.435483871
RPAIN3	3.87E-12	0.1188599	0.277	0.207	9.32E-08	1.4	RPAIN	1.338164251
FBXW81	4.02E-12	0.1024436	0.058	0.031	9.69E-08	1.4	FBXW8	1.870967742
CEP631	4.06E-12	0.1046994	0.109	0.069	9.78E-08	1.4	CEP63	1.579710145
PRPSAP13	4.13E-12	0.1075784	0.124	0.081	9.96E-08	1.4	PRPSAP1	1.530864198
OPTN2	5.27E-12	0.1256607	0.211	0.152	1.27E-07	1.4	OPTN	1.388157895
FBXW72	5.92E-12	0.1527908	0.356	0.275	1.43E-07	1.4	FBXW7	1.294545455
RPL282	6.78E-12	0.4629856	0.143	0.099	1.64E-07	1.4	RPL28	1.444444444
B4GALT51	7.08E-12	0.1322335	0.383	0.297	1.71E-07	1.4	B4GALT5	1.28956229
PDK32	7.37E-12	0.1270926	0.291	0.222	1.78E-07	1.4	PDK3	1.310810811
DLG21	7.91E-12	0.1219384	0.22	0.158	1.91E-07	1.4	DLG2	1.392405063
FOS1	8.35E-12	0.1569015	0.291	0.225	2.01E-07	1.4	FOS	1.293333333
CBWD71	8.68E-12	0.1124158	0.102	0.064	2.09E-07	1.4	CBWD7	1.59375
LUC7L1	9.15E-12	0.1116453	0.137	0.091	2.21E-07	1.4	LUC7L	1.505494505
ARMC81	9.80E-12	0.1246929	0.194	0.138	2.36E-07	1.4	ARMC8	1.405797101
LINC-PINT1	1.11E-11	0.1430625	0.678	0.563	2.67E-07	1.4	LINC-PINT	1.204262877
ZDHHC132	1.14E-11	0.1032805	0.116	0.074	2.74E-07	1.4	ZDHHC13	1.567567568
ZNF1601	1.16E-11	0.1039209	0.077	0.045	2.80E-07	1.4	ZNF160	1.711111111
MASTL1	1.33E-11	0.1099191	0.083	0.049	3.21E-07	1.4	MASTL	1.693877551
CASP71	1.37E-11	0.113431	0.214	0.155	3.29E-07	1.4	CASP7	1.380645161
SHTN11	1.41E-11	0.110736	0.178	0.125	3.41E-07	1.4	SHTN1	1.424
HDAC72	1.51E-11	0.13301	0.139	0.094	3.63E-07	1.4	HDAC7	1.478723404
TGFA2	1.54E-11	0.1236394	0.103	0.065	3.72E-07	1.4	TGFA	1.584615385
DFNB591	1.74E-11	0.1893903	0.033	0.014	4.19E-07	1.4	DFNB59	2.357142857
DCAF62	1.79E-11	0.1120888	0.516	0.419	4.31E-07	1.4	DCAF6	1.23150358
GSTP12	1.85E-11	0.10271	0.088	0.053	4.46E-07	1.4	GSTP1	1.660377358
IWS1	1.93E-11	0.1044483	0.202	0.144	4.66E-07	1.4	IWS1	1.402777778
ARL17A	2.00E-11	0.103079	0.099	0.062	4.82E-07	1.4	ARL17A	1.596774194

NUCKS1	2.03E-11	0.1213801	0.37	0.289	4.89E-07	1.4	NUCKS1	1.280276817
ANXA71	2.36E-11	0.1056633	0.363	0.282	5.70E-07	1.4	ANXA7	1.287234043
RFX31	2.63E-11	0.1400301	0.222	0.163	6.34E-07	1.4	RFX3	1.36196319
CDC42SE11	2.91E-11	0.1099377	0.2	0.144	7.03E-07	1.4	CDC42SE1	1.388888889
BCAP291	2.92E-11	0.1476924	0.202	0.146	7.05E-07	1.4	BCAP29	1.383561644
PRRC2C1	2.99E-11	0.1383936	0.709	0.603	7.21E-07	1.4	PRRC2C	1.175787728
ICA11	3.24E-11	0.1231739	0.337	0.262	7.80E-07	1.4	ICA1	1.286259542
MED27	3.24E-11	0.1097266	0.09	0.055	7.81E-07	1.4	MED27	1.636363636
ALDH1A2	3.29E-11	0.1379854	0.076	0.045	7.93E-07	1.4	ALDH1A2	1.688888889
LIPE-AS11	3.64E-11	0.1305104	0.113	0.073	8.78E-07	1.4	LIPE-AS1	1.547945205
RPL351	3.90E-11	0.3235244	0.335	0.27	9.40E-07	1.4	RPL35	1.240740741
DZANK13	4.19E-11	0.1200746	0.049	0.025	1.01E-06	1.4	DZANK1	1.96
UBR3	4.79E-11	0.1150367	0.255	0.189	1.16E-06	1.4	UBR3	1.349206349
SP100	4.89E-11	0.1334897	0.206	0.151	1.18E-06	1.4	SP100	1.364238411
RP11-1H15.22	6.11E-11	0.1407225	0.108	0.07	1.47E-06	1.4	RP11-1H15	1.542857143
SH3YL13	6.15E-11	0.1131894	0.262	0.197	1.48E-06	1.4	SH3YL1	1.329949239
MFI21	6.35E-11	0.100583	0.038	0.018	1.53E-06	1.4	MFI2	2.111111111
CC2D1A	6.40E-11	0.1010698	0.064	0.036	1.54E-06	1.4	CC2D1A	1.777777778
AAMDC	6.49E-11	0.1355724	0.218	0.161	1.56E-06	1.4	AAMDC	1.354037267
RAB131	6.98E-11	0.1002491	0.192	0.138	1.68E-06	1.4	RAB13	1.391304348
IFT43	7.28E-11	0.1126875	0.134	0.09	1.76E-06	1.4	IFT43	1.488888889
ANKRD101	7.29E-11	0.1310752	0.569	0.477	1.76E-06	1.4	ANKRD10	1.192872117
NFIC1	7.44E-11	0.1252006	0.242	0.18	1.79E-06	1.4	NFIC	1.344444444
EFCAB2	8.04E-11	0.1061424	0.071	0.041	1.94E-06	1.4	EFCAB2	1.731707317
ZNF385D1	9.29E-11	0.1174124	0.072	0.042	2.24E-06	1.4	ZNF385D	1.714285714
TRAPPC91	9.47E-11	0.1251878	0.268	0.201	2.28E-06	1.4	TRAPPC9	1.333333333
DAP2	9.80E-11	0.1034102	0.09	0.056	2.36E-06	1.4	DAP	1.607142857
BMPR2	1.08E-10	0.1200673	0.334	0.261	2.62E-06	1.4	BMPR2	1.279693487
TRAP12	1.49E-10	0.1012822	0.059	0.033	3.60E-06	1.4	TRAP1	1.787878788
EEF1D1	1.72E-10	0.16312	0.143	0.1	4.15E-06	1.4	EEF1D	1.43
PRPF3	1.75E-10	0.122434	0.177	0.127	4.22E-06	1.4	PRPF3	1.393700787
ZNF3263	1.77E-10	0.109484	0.191	0.138	4.28E-06	1.4	ZNF326	1.384057971
RHBDD1	1.80E-10	0.1040438	0.188	0.135	4.34E-06	1.4	RHBDD1	1.392592593
PCLO	1.85E-10	0.1400362	0.08	0.048	4.47E-06	1.4	PCLO	1.666666667
CAMKMT1	1.85E-10	0.1388178	0.225	0.167	4.47E-06	1.4	CAMKMT	1.347305389
FKBP15	2.25E-10	0.1014246	0.088	0.055	5.43E-06	1.4	FKBP15	1.6
GCAT1	2.91E-10	0.1097511	0.058	0.032	7.02E-06	1.4	GCAT	1.8125
RPS141	3.26E-10	0.2585872	0.481	0.403	7.86E-06	1.4	RPS14	1.193548387
SMARCA12	3.33E-10	0.1044294	0.151	0.106	8.03E-06	1.4	SMARCA1	1.424528302
ZNF76	4.02E-10	0.1088898	0.052	0.029	9.69E-06	1.4	ZNF76	1.793103448
RP11-91P24.7	5.04E-10	0.104371	0.07	0.042	1.22E-05	1.4	RP11-91P2	1.666666667
NIN1	5.55E-10	0.1134735	0.144	0.099	1.34E-05	1.4	NIN	1.454545455
RP4-605O3.41	7.03E-10	0.1360122	0.124	0.085	1.70E-05	1.4	RP4-605O3	1.458823529
OPA11	7.98E-10	0.1255767	0.144	0.101	1.92E-05	1.4	OPA1	1.425742574
MT-ND4L3	8.80E-10	0.1008452	0.534	0.444	2.12E-05	1.4	MT-ND4L	1.202702703
PPP2R5A1	1.02E-09	0.1038286	0.209	0.155	2.46E-05	1.4	PPP2R5A	1.348387097
CABIN11	1.32E-09	0.1146921	0.104	0.068	3.17E-05	1.4	CABIN1	1.529411765
BCL6	1.58E-09	0.1882375	0.427	0.35	3.80E-05	1.4	BCL6	1.22
METTL161	1.89E-09	0.1202266	0.229	0.173	4.55E-05	1.4	METTL16	1.323699422
SFI11	2.01E-09	0.1040146	0.082	0.052	4.84E-05	1.4	SFI1	1.576923077
CXCL101	2.03E-09	0.215318	0.013	0.004	4.90E-05	1.4	CXCL10	3.25
AC004231.22	2.04E-09	0.1071878	0.059	0.034	4.91E-05	1.4	AC004231.	1.735294118

ZSWIM71	2.58E-09	0.1216462	0.1	0.066	6.23E-05	1.4	ZSWIM7	1.515151515
RP11-479O9.4	2.69E-09	0.1027905	0.088	0.056	6.49E-05	1.4	RP11-479O	1.571428571
EYA31	2.69E-09	0.1018456	0.132	0.091	6.49E-05	1.4	EYA3	1.450549451
LTBP12	3.16E-09	0.1175321	0.253	0.195	7.61E-05	1.4	LTBP1	1.297435897
CNN21	3.64E-09	0.1094931	0.043	0.023	8.78E-05	1.4	CNN2	1.869565217
CEP192	4.22E-09	0.1291606	0.154	0.111	0.000101814	1.4	CEP192	1.387387387
TAPBP	4.26E-09	0.1173557	0.188	0.139	0.000102702	1.4	TAPBP	1.352517986
LDLR1	4.71E-09	0.1069106	0.22	0.165	0.000113658	1.4	LDLR	1.333333333
KCNQ1OT11	4.98E-09	0.1047063	0.121	0.083	0.000120075	1.4	KCNQ1OT1	1.457831325
MTX2	4.99E-09	0.1013803	0.118	0.081	0.000120249	1.4	MTX2	1.456790123
DDX17	5.52E-09	0.1346078	0.508	0.417	0.000133198	1.4	DDX17	1.21822542
ZBTB7C1	5.69E-09	0.1031668	0.064	0.039	0.000137242	1.4	ZBTB7C	1.641025641
DPP101	6.37E-09	0.1170995	0.076	0.048	0.00015358	1.4	DPP10	1.583333333
SCFD11	6.55E-09	0.1074494	0.27	0.21	0.000157828	1.4	SCFD1	1.285714286
MED13L	6.66E-09	0.1699724	0.763	0.68	0.00016069	1.4	MED13L	1.122058824
MYO3B3	8.38E-09	0.1766887	0.191	0.147	0.000202161	1.4	MYO3B	1.299319728
PLA2G162	9.98E-09	0.1240538	0.127	0.09	0.000240709	1.4	PLA2G16	1.411111111
RPL81	1.22E-08	0.3102754	0.206	0.159	0.000295136	1.4	RPL8	1.295597484
LRRC28	1.44E-08	0.1186028	0.102	0.069	0.000346629	1.4	LRRC28	1.47826087
MLLT101	1.47E-08	0.1003619	0.203	0.153	0.000354369	1.4	MLLT10	1.326797386
UTRN	1.56E-08	0.1273311	0.342	0.272	0.000377098	1.4	UTRN	1.257352941
ZNF1481	1.74E-08	0.1157465	0.342	0.273	0.000420372	1.4	ZNF148	1.252747253
C20orf203	2.23E-08	0.1046941	0.022	0.01	0.000537443	1.4	C20orf203	2.2
PREPL2	2.55E-08	0.1210951	0.141	0.102	0.000615553	1.4	PREPL	1.382352941
FBXL17	2.59E-08	0.1063582	0.327	0.26	0.000625317	1.4	FBXL17	1.257692308
HIPK31	3.53E-08	0.108575	0.241	0.187	0.000852359	1.4	HIPK3	1.288770053
CLIP22	5.66E-08	0.1340353	0.171	0.129	0.001365252	1.4	CLIP2	1.325581395
NFATC31	8.30E-08	0.1007484	0.217	0.166	0.002001002	1.4	NFATC3	1.307228916
PCNXL23	1.44E-07	0.2785627	0.811	0.758	0.003466586	1.4	PCNXL2	1.069920844
MLLT6	1.80E-07	0.1115197	0.121	0.087	0.004330937	1.4	MLLT6	1.390804598
GEMIN51	3.47E-07	0.1112797	0.032	0.017	0.008364771	1.4	GEMIN5	1.882352941
KIAA1033	3.59E-07	0.1110047	0.139	0.103	0.008648993	1.4	KIAA1033	1.349514563
ZDHHC20	3.78E-07	0.1013345	0.22	0.173	0.009112746	1.4	ZDHHC20	1.271676301
LIFR	5.26E-07	0.1081005	0.19	0.148	0.01268262	1.4	LIFR	1.283783784
FAU1	5.93E-07	0.1937843	0.231	0.186	0.01430876	1.4	FAU	1.241935484
RAB11FIP31	7.67E-07	0.133574	0.107	0.077	0.01849001	1.4	RAB11FIP3	1.38961039
B4GALT1	9.89E-07	0.1084049	0.506	0.433	0.02383706	1.4	B4GALT1	1.168591224
H3F3B	1.27E-06	0.1321039	0.456	0.387	0.03072829	1.4	H3F3B	1.178294574
MAST4	1.29E-06	0.1167305	0.595	0.521	0.03105796	1.4	MAST4	1.142034549
CD24	1.67E-06	0.1235739	0.641	0.573	0.04021768	1.4	CD24	1.118673647
RPLP21	1.75E-06	0.2976366	0.266	0.221	0.04210245	1.4	RPLP2	1.20361991
RP1-78O14.1	0	1.633987	0.515	0.173	0	2.1	RP1-78O14	2.976878613
NR6A1	0	1.532101	0.676	0.319	0	2.1	NR6A1	2.119122257
MYO61	0	1.270477	0.928	0.743	0	2.1	MYO6	1.248990579
RICTOR1	0	1.245223	0.859	0.599	0	2.1	RICTOR	1.434056761
ANKRD30A	0	1.042348	0.881	0.431	0	2.1	ANKRD30A	2.044083527
AREG	1.64E-303	1.217541	0.791	0.417	3.94E-299	2.1	AREG	1.896882494
DTNA1	1.07E-294	1.187511	0.919	0.742	2.57E-290	2.1	DTNA	1.238544474
TMEM2	4.86E-287	1.363091	0.65	0.317	1.17E-282	2.1	TMEM2	2.050473186
DNAJC12	1.47E-274	1.099486	0.727	0.357	3.54E-270	2.1	DNAJC12	2.036414566
TRIO2	1.23E-256	0.9813895	0.946	0.796	2.97E-252	2.1	TRIO	1.188442211
MACF1	2.08E-250	1.08192	0.872	0.706	5.01E-246	2.1	MACF1	1.235127479

TSC22D21	9.61E-242	1.47351	0.722	0.442	2.32E-237	2.1	TSC22D2	1.633484163
HSPA51	9.40E-236	1.391482	0.715	0.424	2.27E-231	2.1	HSPA5	1.686320755
AR	3.85E-231	1.119676	0.402	0.136	9.29E-227	2.1	AR	2.955882353
UBN2	4.15E-229	1.078308	0.543	0.247	1.00E-224	2.1	UBN2	2.198380567
GNAS	1.58E-222	0.8722538	0.902	0.738	3.80E-218	2.1	GNAS	1.222222222
RP11-554F20.1	4.84E-220	0.9549696	0.207	0.039	1.17E-215	2.1	RP11-554F.	5.307692308
CHD2	3.02E-218	1.074213	0.771	0.526	7.29E-214	2.1	CHD2	1.465779468
ZFAND3	1.98E-214	0.8375056	0.907	0.746	4.79E-210	2.1	ZFAND3	1.215817694
CXCL13	2.98E-212	1.120639	0.718	0.381	7.19E-208	2.1	CXCL13	1.884514436
TSPAN5	1.43E-211	1.113683	0.587	0.281	3.46E-207	2.1	TSPAN5	2.088967972
RPS6KA3	2.57E-209	1.145938	0.703	0.441	6.21E-205	2.1	RPS6KA3	1.594104308
ATXN1	2.65E-208	1.17818	0.734	0.494	6.40E-204	2.1	ATXN1	1.48582996
PPP2R2A1	8.86E-204	1.086807	0.733	0.511	2.14E-199	2.1	PPP2R2A	1.43444227
LRBA	2.49E-201	1.180629	0.808	0.622	6.01E-197	2.1	LRBA	1.29903537
EREG	2.27E-195	1.007322	0.533	0.234	5.46E-191	2.1	EREG	2.277777778
ALCAM	1.22E-194	0.8236185	0.788	0.485	2.94E-190	2.1	ALCAM	1.624742268
BCL2	2.46E-189	1.293647	0.519	0.249	5.94E-185	2.1	BCL2	2.084337349
UGDH	1.89E-188	0.96437	0.486	0.212	4.56E-184	2.1	UGDH	2.29245283
OTUD7B	9.51E-186	0.9960397	0.542	0.27	2.29E-181	2.1	OTUD7B	2.007407407
CACNB4	4.84E-185	1.095167	0.315	0.1	1.17E-180	2.1	CACNB4	3.15
ZNF644	4.61E-181	1.177627	0.682	0.45	1.11E-176	2.1	ZNF644	1.515555556
RAB11FIP1	4.43E-180	0.7156369	0.918	0.715	1.07E-175	2.1	RAB11FIP1	1.283916084
RASEF	8.03E-179	0.9767797	0.513	0.249	1.94E-174	2.1	RASEF	2.060240964
THRB	7.90E-177	1.086204	0.686	0.438	1.90E-172	2.1	THRB	1.566210046
MALAT1	1.12E-176	0.4626054	0.999	1	2.69E-172	2.1	MALAT1	0.999
ANKRD12	4.98E-171	0.9104013	0.816	0.659	1.20E-166	2.1	ANKRD12	1.238239757
ERBB4	1.29E-167	0.8464465	0.769	0.465	3.11E-163	2.1	ERBB4	1.653763441
AKAP13	1.45E-165	0.9341448	0.775	0.595	3.49E-161	2.1	AKAP13	1.302521008
ERBB2IP	5.07E-164	0.981188	0.643	0.407	1.22E-159	2.1	ERBB2IP	1.57985258
HIVEP2	3.95E-159	1.071925	0.657	0.43	9.52E-155	2.1	HIVEP2	1.527906977
ARHGAP291	8.86E-159	0.8810927	0.771	0.554	2.14E-154	2.1	ARHGAP29	1.391696751
ANO6	2.08E-157	0.8878721	0.717	0.5	5.01E-153	2.1	ANO6	1.434
DENND4A	5.25E-154	0.9566139	0.76	0.578	1.27E-149	2.1	DENND4A	1.314878893
BTRC	2.73E-149	0.9494723	0.478	0.243	6.58E-145	2.1	BTRC	1.967078189
EFHD1	3.50E-149	0.9071621	0.531	0.278	8.44E-145	2.1	EFHD1	1.910071942
GSTM3	5.74E-149	0.8424408	0.559	0.305	1.38E-144	2.1	GSTM3	1.832786885
LARP1B	9.01E-149	1.018972	0.421	0.198	2.17E-144	2.1	LARP1B	2.126262626
COX6C	1.65E-147	0.8208295	0.853	0.754	3.98E-143	2.1	COX6C	1.131299735
KCNMA1	8.40E-145	1.312373	0.331	0.131	2.03E-140	2.1	KCNMA1	2.526717557
SYTL2	4.25E-143	0.7844581	0.704	0.427	1.03E-138	2.1	SYTL2	1.648711944
AF127936.9	5.07E-143	0.8065297	0.331	0.128	1.22E-138	2.1	AF127936.!	2.5859375
HILPDA1	1.62E-142	1.745543	0.484	0.258	3.92E-138	2.1	HILPDA	1.875968992
AGR2	3.80E-141	0.8947078	0.472	0.222	9.17E-137	2.1	AGR2	2.126126126
C8orf4	4.30E-139	0.854627	0.779	0.494	1.04E-134	2.1	C8orf4	1.576923077
ADAM32	7.63E-139	1.008864	0.431	0.21	1.84E-134	2.1	ADAM32	2.052380952
YTHDC11	1.05E-138	0.8715612	0.586	0.365	2.52E-134	2.1	YTHDC1	1.605479452
TJP1	2.21E-137	0.8697263	0.685	0.49	5.33E-133	2.1	TJP1	1.397959184
PHLDB2	2.69E-133	0.8735323	0.622	0.4	6.49E-129	2.1	PHLDB2	1.555
ARPP19	6.90E-132	0.8630079	0.616	0.412	1.66E-127	2.1	ARPP19	1.495145631
MYBPC1	9.59E-132	0.6541068	0.452	0.211	2.31E-127	2.1	MYBPC1	2.142180095
NIPBL	1.24E-131	0.8557192	0.707	0.533	2.98E-127	2.1	NIPBL	1.326454034
MYO5B2	4.80E-129	0.7564272	0.773	0.586	1.16E-124	2.1	MYO5B	1.319112628

HERC4	3.66E-128	0.8538589	0.696	0.511	8.83E-124	2.1	HERC4	1.362035225
PEAK1	1.13E-127	1.086993	0.464	0.248	2.72E-123	2.1	PEAK1	1.870967742
NEBL	1.36E-127	0.8347055	0.805	0.65	3.28E-123	2.1	NEBL	1.238461538
RERE	2.48E-127	0.8559016	0.719	0.556	5.99E-123	2.1	RERE	1.293165468
SH3BGRL	4.48E-127	0.8110121	0.599	0.383	1.08E-122	2.1	SH3BGRL	1.563968668
MLLT42	1.19E-126	0.6964746	0.789	0.63	2.87E-122	2.1	MLLT4	1.252380952
RALGPS2	1.36E-126	0.9384819	0.497	0.276	3.28E-122	2.1	RALGPS2	1.800724638
KTN1	5.22E-125	0.7622852	0.709	0.527	1.26E-120	2.1	KTN1	1.345351044
C1orf21	1.06E-124	0.953538	0.509	0.295	2.55E-120	2.1	C1orf21	1.725423729
USP47	1.10E-123	0.8840325	0.604	0.404	2.65E-119	2.1	USP47	1.495049505
LMO7	2.10E-116	0.966531	0.362	0.168	5.07E-112	2.1	LMO7	2.154761905
C3orf52	6.55E-116	0.7478776	0.332	0.145	1.58E-111	2.1	C3orf52	2.289655172
LRRFIP22	1.71E-115	0.5499177	0.877	0.751	4.12E-111	2.1	LRRFIP2	1.167776298
RP11-60I3.4	3.02E-112	0.4141649	0.101	0.017	7.28E-108	2.1	RP11-60I3.4	5.941176471
GPBP11	3.08E-110	0.6382426	0.806	0.677	7.42E-106	2.1	GPBP1	1.190546529
ITGAV	3.82E-110	0.7390965	0.592	0.381	9.21E-106	2.1	ITGAV	1.553805774
KIF13A	7.13E-110	0.9765775	0.566	0.379	1.72E-105	2.1	KIF13A	1.493403694
UBE2H1	1.33E-109	0.6385906	0.842	0.748	3.20E-105	2.1	UBE2H	1.125668449
PAN3	2.64E-108	0.7030421	0.762	0.627	6.36E-104	2.1	PAN3	1.215311005
SPOPL	4.04E-108	0.7912097	0.441	0.243	9.75E-104	2.1	SPOPL	1.814814815
ARHGEF121	5.75E-106	0.729039	0.722	0.564	1.39E-101	2.1	ARHGEF12	1.280141844
CPEB2	1.40E-105	0.6356832	0.521	0.289	3.37E-101	2.1	CPEB2	1.802768166
NRIP1	6.13E-105	0.8177522	0.451	0.251	1.48E-100	2.1	NRIP1	1.796812749
IBTK	8.93E-104	0.9518375	0.386	0.206	2.15E-99	2.1	IBTK	1.873786408
INPP4B	2.89E-102	0.8459877	0.527	0.31	6.97E-98	2.1	INPP4B	1.7
MYL12B	4.85E-102	0.5617299	0.906	0.812	1.17E-97	2.1	MYL12B	1.115763547
PPP6R3	2.17E-101	0.7156229	0.655	0.494	5.22E-97	2.1	PPP6R3	1.325910931
DUSP5	1.09E-99	0.9530936	0.406	0.222	2.62E-95	2.1	DUSP5	1.828828829
NAA251	4.14E-99	0.9178902	0.516	0.335	9.99E-95	2.1	NAA25	1.540298507
MBNL2	5.73E-99	0.6940312	0.69	0.529	1.38E-94	2.1	MBNL2	1.304347826
TBC1D9	4.14E-98	0.6893746	0.628	0.432	9.97E-94	2.1	TBC1D9	1.453703704
FAM160A12	1.51E-97	0.83561	0.753	0.64	3.64E-93	2.1	FAM160A1	1.1765625
HSP90B11	8.28E-95	0.8982709	0.651	0.494	2.00E-90	2.1	HSP90B1	1.317813765
KLRD1	1.01E-94	0.765224	0.317	0.148	2.43E-90	2.1	KLRD1	2.141891892
MYO1B	7.13E-94	0.7926185	0.606	0.433	1.72E-89	2.1	MYO1B	1.399538106
RAB11A1	7.55E-93	0.7094898	0.745	0.632	1.82E-88	2.1	RAB11A	1.178797468
ZNRF2	5.19E-92	0.8713301	0.426	0.247	1.25E-87	2.1	ZNRF2	1.724696356
CASC15	1.09E-91	1.159016	0.656	0.51	2.64E-87	2.1	CASC15	1.28627451
STAT5B	2.19E-91	0.7557397	0.497	0.317	5.29E-87	2.1	STAT5B	1.567823344
CAMTA11	4.44E-91	0.6131416	0.716	0.569	1.07E-86	2.1	CAMTA1	1.258347979
SLC39A6	1.80E-90	0.616514	0.481	0.286	4.34E-86	2.1	SLC39A6	1.681818182
SMCHD1	4.88E-89	0.7212333	0.562	0.393	1.18E-84	2.1	SMCHD1	1.430025445
BHLHE40	1.93E-88	0.7205815	0.342	0.173	4.64E-84	2.1	BHLHE40	1.976878613
STAT5A	9.47E-88	0.7859557	0.33	0.166	2.28E-83	2.1	STAT5A	1.987951807
RAP1B	1.49E-86	0.7034014	0.61	0.462	3.60E-82	2.1	RAP1B	1.32034632
ACRC	2.09E-86	0.6157249	0.225	0.089	5.03E-82	2.1	ACRC	2.528089888
BNIP3L	8.49E-86	0.7879776	0.565	0.406	2.05E-81	2.1	BNIP3L	1.391625616
TFPI	3.96E-85	0.5617088	0.525	0.307	9.55E-81	2.1	TFPI	1.71009772
MAP7D2	6.34E-85	0.4707505	0.138	0.039	1.53E-80	2.1	MAP7D2	3.538461538
ELF1	1.32E-84	0.5999854	0.672	0.515	3.17E-80	2.1	ELF1	1.304854369
CBLB	2.75E-83	0.8454796	0.474	0.307	6.63E-79	2.1	CBLB	1.543973941
LRRFIP11	2.86E-83	0.5823101	0.748	0.619	6.89E-79	2.1	LRRFIP1	1.208400646

PTK2	5.62E-83	0.5872544	0.748	0.632	1.35E-78	2.1	PTK2	1.183544304
MAN2A1	3.04E-82	0.7891257	0.47	0.306	7.33E-78	2.1	MAN2A1	1.535947712
GSPT1	8.58E-82	0.6618585	0.52	0.354	2.07E-77	2.1	GSPT1	1.468926554
FNBP11	9.95E-82	0.6559605	0.661	0.514	2.40E-77	2.1	FNBP1	1.285992218
CTD-2587M2.1	1.63E-80	0.3690968	0.096	0.021	3.94E-76	2.1	CTD-2587M	4.571428571
SLC2A11	2.47E-80	0.651313	0.194	0.072	5.95E-76	2.1	SLC2A1	2.694444444
SETD51	7.00E-80	0.6261876	0.647	0.516	1.69E-75	2.1	SETD5	1.253875969
ARID4B	1.04E-79	0.6635503	0.669	0.546	2.50E-75	2.1	ARID4B	1.225274725
ECT2	7.02E-79	0.6843206	0.353	0.192	1.69E-74	2.1	ECT2	1.838541667
SDC2	2.96E-78	0.517653	0.188	0.069	7.14E-74	2.1	SDC2	2.724637681
SEMA3C	4.01E-78	0.7810585	0.439	0.265	9.68E-74	2.1	SEMA3C	1.656603774
MIR181A2HG	1.51E-77	0.5493096	0.195	0.075	3.63E-73	2.1	MIR181A2H	2.6
FUT8	2.72E-77	0.7963676	0.414	0.246	6.56E-73	2.1	FUT8	1.682926829
TOP1	3.44E-77	0.6842827	0.585	0.433	8.30E-73	2.1	TOP1	1.351039261
PDZD8	4.25E-77	0.6698664	0.381	0.217	1.02E-72	2.1	PDZD8	1.755760369
TOM1L2	5.93E-77	0.7043624	0.45	0.287	1.43E-72	2.1	TOM1L2	1.567944251
SMG1	1.94E-76	0.6818788	0.476	0.314	4.69E-72	2.1	SMG1	1.515923567
WASL	2.14E-76	0.6840735	0.377	0.217	5.17E-72	2.1	WASL	1.737327189
SMIM14	2.93E-75	0.5793547	0.562	0.384	7.06E-71	2.1	SMIM14	1.463541667
EFNA1	3.01E-74	0.7322566	0.386	0.225	7.25E-70	2.1	EFNA1	1.715555556
UGCG	5.22E-74	0.6114742	0.632	0.476	1.26E-69	2.1	UGCG	1.327731092
LAMA3	2.40E-73	0.7472879	0.334	0.181	5.79E-69	2.1	LAMA3	1.845303867
FASN	2.45E-73	0.4138622	0.138	0.043	5.90E-69	2.1	FASN	3.209302326
MORF4L22	5.03E-73	0.590254	0.791	0.737	1.21E-68	2.1	MORF4L2	1.073270014
LRRC1	1.20E-72	0.6823404	0.274	0.133	2.90E-68	2.1	LRRC1	2.060150376
DGKD	3.05E-72	0.6007243	0.247	0.113	7.36E-68	2.1	DGKD	2.185840708
WDR49	3.81E-72	0.4013896	0.102	0.026	9.18E-68	2.1	WDR49	3.923076923
USP9X	8.11E-72	0.635577	0.541	0.393	1.96E-67	2.1	USP9X	1.376590331
CNOT4	5.00E-71	0.6581222	0.533	0.388	1.20E-66	2.1	CNOT4	1.37371134
ACADSB	1.65E-69	0.6158905	0.346	0.19	3.98E-65	2.1	ACADSB	1.821052632
HPX	1.04E-68	0.6346567	0.368	0.201	2.52E-64	2.1	HPX	1.830845771
FRYL	2.19E-68	0.6727691	0.528	0.385	5.28E-64	2.1	FRYL	1.371428571
CDK8	7.39E-68	0.674549	0.379	0.228	1.78E-63	2.1	CDK8	1.662280702
MAST2	1.20E-67	0.703331	0.359	0.211	2.90E-63	2.1	MAST2	1.701421801
USP121	1.03E-65	0.6595098	0.317	0.178	2.48E-61	2.1	USP12	1.780898876
PDLIM53	1.58E-65	0.5367336	0.831	0.734	3.82E-61	2.1	PDLIM5	1.132152589
P4HA1	1.15E-64	0.9063382	0.506	0.374	2.76E-60	2.1	P4HA1	1.352941176
MAPK8	3.58E-64	0.7210765	0.467	0.322	8.64E-60	2.1	MAPK8	1.450310559
LIMCH1	1.66E-62	0.7510446	0.607	0.477	3.99E-58	2.1	LIMCH1	1.272536688
CTA-392E5.1	7.09E-62	0.4417376	0.097	0.027	1.71E-57	2.1	CTA-392E5	3.592592593
ERO1A	7.45E-62	0.9461618	0.543	0.431	1.80E-57	2.1	ERO1A	1.259860789
AUH1	2.17E-61	0.6603538	0.485	0.342	5.24E-57	2.1	AUH	1.418128655
FAM171B	2.48E-61	0.4105449	0.105	0.031	5.97E-57	2.1	FAM171B	3.387096774
PLOD2	2.57E-61	0.9513787	0.366	0.227	6.19E-57	2.1	PLOD2	1.612334802
TOX3	2.86E-61	0.5688606	0.297	0.159	6.90E-57	2.1	TOX3	1.867924528
SAMSN1	3.55E-61	0.2965191	0.081	0.019	8.57E-57	2.1	SAMSN1	4.263157895
MIPOL1	9.45E-61	0.6825692	0.308	0.173	2.28E-56	2.1	MIPOL1	1.780346821
HIPK2	1.04E-60	0.5949877	0.497	0.362	2.50E-56	2.1	HIPK2	1.372928177
FGF13	1.13E-60	0.6829484	0.596	0.439	2.73E-56	2.1	FGF13	1.357630979
OSER12	1.74E-60	0.4652601	0.626	0.48	4.19E-56	2.1	OSER1	1.304166667
SLC7A2	2.47E-60	0.4696608	0.461	0.286	5.94E-56	2.1	SLC7A2	1.611888112
ENAH1	2.52E-60	0.5528372	0.658	0.543	6.07E-56	2.1	ENAH	1.211786372

DUSP10	3.16E-60	0.5430666	0.359	0.212	7.62E-56	2.1	DUSP10	1.693396226
TPH2	3.57E-60	0.3101391	0.072	0.016	8.61E-56	2.1	TPH2	4.5
MAP3K1	7.10E-60	0.5365419	0.605	0.444	1.71E-55	2.1	MAP3K1	1.362612613
NEDD4L1	8.04E-60	0.7647892	0.671	0.592	1.94E-55	2.1	NEDD4L	1.133445946
RP11-507B12.2	1.22E-59	0.4659939	0.207	0.092	2.93E-55	2.1	RP11-507B	2.25
ELOVL5	2.15E-59	0.4566434	0.441	0.282	5.17E-55	2.1	ELOVL5	1.563829787
ZSWIM6	2.51E-59	0.558187	0.617	0.487	6.05E-55	2.1	ZSWIM6	1.266940452
IGFBP4	3.57E-59	0.543973	0.257	0.129	8.62E-55	2.1	IGFBP4	1.992248062
NFKB11	3.71E-59	0.659944	0.564	0.44	8.95E-55	2.1	NFKB1	1.281818182
SMAD3	8.28E-59	0.6055378	0.407	0.258	2.00E-54	2.1	SMAD3	1.57751938
INTS6	8.38E-59	0.6787923	0.41	0.27	2.02E-54	2.1	INTS6	1.518518519
NPR3	2.12E-57	0.2261846	0.052	0.009	5.12E-53	2.1	NPR3	5.777777778
NME7	1.15E-56	0.6421152	0.26	0.138	2.78E-52	2.1	NME7	1.884057971
ACTN11	1.54E-56	0.5710714	0.475	0.33	3.70E-52	2.1	ACTN1	1.439393939
MAST41	2.10E-56	0.6254765	0.643	0.518	5.07E-52	2.1	MAST4	1.241312741
MED13	5.95E-56	0.5856499	0.518	0.388	1.44E-51	2.1	MED13	1.335051546
CCNG2	9.51E-56	0.5936134	0.335	0.201	2.29E-51	2.1	CCNG2	1.666666667
NCEH1	5.02E-55	0.6047724	0.544	0.402	1.21E-50	2.1	NCEH1	1.353233831
RANBP9	1.25E-54	0.6044745	0.421	0.288	3.02E-50	2.1	RANBP9	1.461805556
DAAM12	1.48E-54	0.5509852	0.609	0.499	3.57E-50	2.1	DAAM1	1.220440882
RP11-554D13.1	4.80E-54	0.3236285	0.067	0.015	1.16E-49	2.1	RP11-554D	4.466666667
DCP2	5.27E-54	0.5971657	0.29	0.166	1.27E-49	2.1	DCP2	1.746987952
AF165138.7	8.94E-54	0.2262025	0.058	0.012	2.15E-49	2.1	AF165138.7	4.833333333
ATP2B41	1.01E-53	0.7255304	0.474	0.347	2.45E-49	2.1	ATP2B4	1.365994236
ZFC3H1	2.08E-53	0.6152707	0.377	0.248	5.01E-49	2.1	ZFC3H1	1.52016129
KLHL24	3.11E-53	0.585361	0.467	0.34	7.51E-49	2.1	KLHL24	1.373529412
HSPH1	3.68E-53	0.5344348	0.413	0.273	8.88E-49	2.1	HSPH1	1.512820513
BTG1	3.78E-53	0.4834286	0.676	0.573	9.11E-49	2.1	BTG1	1.179755672
SH3BP4	7.68E-53	0.5396674	0.27	0.149	1.85E-48	2.1	SH3BP4	1.812080537
MEF2A1	2.09E-52	0.5911693	0.535	0.411	5.04E-48	2.1	MEF2A	1.301703163
LCOR	2.61E-52	0.6183192	0.384	0.255	6.29E-48	2.1	LCOR	1.505882353
SYT1	6.40E-52	0.2948592	0.077	0.02	1.54E-47	2.1	SYT1	3.85
SHROOM3	8.59E-52	0.4737181	0.722	0.623	2.07E-47	2.1	SHROOM3	1.158908507
CORIN	9.87E-52	0.2971033	0.069	0.017	2.38E-47	2.1	CORIN	4.058823529
TBC1D10A	1.17E-51	0.7801783	0.266	0.145	2.81E-47	2.1	TBC1D10A	1.834482759
UBR4	2.60E-51	0.574392	0.346	0.22	6.28E-47	2.1	UBR4	1.572727273
TGIF1	2.84E-51	0.6049931	0.334	0.209	6.85E-47	2.1	TGIF1	1.598086124
RP11-282I1.1	3.22E-51	0.3232303	0.09	0.027	7.77E-47	2.1	RP11-282I1	3.333333333
AAK1	3.81E-51	0.5420354	0.278	0.157	9.19E-47	2.1	AAK1	1.770700637
SCP2	1.16E-50	0.5420282	0.441	0.313	2.81E-46	2.1	SCP2	1.408945687
PJA21	1.45E-50	0.620007	0.355	0.231	3.49E-46	2.1	PJA2	1.536796537
TSPAN15	4.20E-50	0.4776462	0.198	0.095	1.01E-45	2.1	TSPAN15	2.084210526
FNDC3B	1.73E-49	0.5226912	0.802	0.77	4.16E-45	2.1	FNDC3B	1.041558442
ACTG1	2.31E-49	0.6250293	0.789	0.719	5.57E-45	2.1	ACTG1	1.097357441
GCNT2	6.19E-49	0.8556479	0.528	0.438	1.49E-44	2.1	GCNT2	1.205479452
CDH14	8.65E-49	0.4925809	0.691	0.598	2.08E-44	2.1	CDH1	1.155518395
BAZ1A	1.16E-48	0.5854735	0.492	0.375	2.79E-44	2.1	BAZ1A	1.312
RP4-800F24.1	1.52E-48	0.2759155	0.066	0.016	3.67E-44	2.1	RP4-800F24	4.125
XG	2.09E-48	0.2490544	0.048	0.009	5.04E-44	2.1	XG	5.333333333
DNAJB9	2.65E-48	0.4507568	0.178	0.082	6.38E-44	2.1	DNAJB9	2.170731707
ANGPTL1	5.50E-48	0.5303419	0.247	0.135	1.33E-43	2.1	ANGPTL1	1.82962963
FLNB1	2.02E-47	0.5491271	0.564	0.448	4.86E-43	2.1	FLNB	1.258928571

AC026202.31	2.60E-47	0.5862807	0.318	0.197	6.26E-43	2.1	AC026202.:	1.614213198
NEU3	2.70E-47	0.2582004	0.076	0.021	6.51E-43	2.1	NEU3	3.619047619
UBR51	2.99E-47	0.5252045	0.568	0.461	7.22E-43	2.1	UBR5	1.232104121
CCDC73	5.33E-47	0.5571304	0.199	0.098	1.29E-42	2.1	CCDC73	2.030612245
FXYD3	6.95E-47	0.47194	0.709	0.627	1.68E-42	2.1	FXYD3	1.130781499
ANKRD17	8.56E-47	0.52195	0.597	0.501	2.06E-42	2.1	ANKRD17	1.191616766
DIAPH1	1.23E-46	0.5630864	0.409	0.288	2.96E-42	2.1	DIAPH1	1.420138889
ARIH1	2.15E-46	0.4850016	0.605	0.5	5.18E-42	2.1	ARIH1	1.21
UBE2G1	2.39E-46	0.5828861	0.366	0.247	5.75E-42	2.1	UBE2G1	1.481781377
CD2AP2	3.37E-46	0.6229858	0.526	0.419	8.14E-42	2.1	CD2AP	1.255369928
MXD11	3.46E-46	0.6525659	0.323	0.202	8.34E-42	2.1	MXD1	1.599009901
MAP2K11	4.89E-46	0.5570008	0.363	0.243	1.18E-41	2.1	MAP2K1	1.49382716
RSRC21	7.25E-46	0.5086428	0.683	0.62	1.75E-41	2.1	RSRC2	1.101612903
REL	9.05E-46	0.5624793	0.413	0.29	2.18E-41	2.1	REL	1.424137931
PPP2CB1	2.29E-45	0.5099012	0.446	0.33	5.52E-41	2.1	PPP2CB	1.351515152
ELMO2	2.61E-45	0.4407742	0.181	0.087	6.29E-41	2.1	ELMO2	2.08045977
MAP41	3.21E-45	0.5400921	0.572	0.473	7.73E-41	2.1	MAP4	1.209302326
JUND	3.95E-44	0.4981503	0.278	0.165	9.51E-40	2.1	JUND	1.684848485
TOR1AIP21	5.74E-44	0.5371555	0.374	0.26	1.38E-39	2.1	TOR1AIP2	1.438461538
RAB21	1.41E-43	0.5365163	0.372	0.253	3.39E-39	2.1	RAB21	1.470355731
RLF1	1.91E-43	0.4896185	0.458	0.337	4.60E-39	2.1	RLF	1.359050445
HNRNPM	1.92E-43	0.5030583	0.371	0.253	4.64E-39	2.1	HNRNPM	1.466403162
OLA1	2.91E-43	0.571166	0.428	0.315	7.02E-39	2.1	OLA1	1.358730159
GOLGA41	3.39E-43	0.4320645	0.683	0.615	8.17E-39	2.1	GOLGA4	1.110569106
PGM2L1	5.10E-43	0.5747942	0.341	0.224	1.23E-38	2.1	PGM2L1	1.522321429
ABCD3	5.74E-43	0.5626859	0.282	0.171	1.39E-38	2.1	ABCD3	1.649122807
RASAL22	7.07E-43	0.6094288	0.613	0.536	1.71E-38	2.1	RASAL2	1.143656716
OCLN1	7.61E-43	0.5207461	0.439	0.328	1.84E-38	2.1	OCLN	1.338414634
PTHLH	1.22E-42	0.3189704	0.367	0.224	2.94E-38	2.1	PTHLH	1.638392857
AGR3	1.42E-42	0.4512829	0.337	0.206	3.43E-38	2.1	AGR3	1.63592233
PVRL2	1.42E-42	0.4878793	0.398	0.274	3.43E-38	2.1	PVRL2	1.452554745
SLC9C1	2.03E-42	0.4033082	0.126	0.051	4.90E-38	2.1	SLC9C1	2.470588235
PIAS1	2.25E-42	0.5004929	0.569	0.478	5.43E-38	2.1	PIAS1	1.190376569
SRPK2	2.35E-42	0.6302112	0.506	0.405	5.67E-38	2.1	SRPK2	1.249382716
IGF1R	3.32E-42	0.4965016	0.59	0.496	8.01E-38	2.1	IGF1R	1.189516129
BMPR1B1	1.14E-41	0.5545485	0.127	0.052	2.76E-37	2.1	BMPR1B	2.442307692
NBEAL1	1.68E-41	0.360326	0.767	0.711	4.04E-37	2.1	NBEAL1	1.078762307
LSAMP	1.71E-41	0.4481734	0.589	0.461	4.12E-37	2.1	LSAMP	1.277657267
MAGI3	2.83E-41	0.5061625	0.493	0.374	6.82E-37	2.1	MAGI3	1.318181818
TBC1D23	4.56E-41	0.5205244	0.287	0.18	1.10E-36	2.1	TBC1D23	1.594444444
WWC2	6.03E-41	0.5669757	0.3	0.189	1.45E-36	2.1	WWC2	1.587301587
FBP1	8.06E-41	0.2795262	0.091	0.032	1.94E-36	2.1	FBP1	2.84375
GSTK1	1.57E-40	0.429395	0.234	0.132	3.78E-36	2.1	GSTK1	1.772727273
GSK3B	1.64E-40	0.5054916	0.499	0.391	3.95E-36	2.1	GSK3B	1.276214834
TM9SF2	1.84E-40	0.5333619	0.359	0.249	4.43E-36	2.1	TM9SF2	1.441767068
CTBP2	3.71E-40	0.5199897	0.443	0.336	8.95E-36	2.1	CTBP2	1.318452381
DPY19L1	3.73E-40	0.4222619	0.178	0.089	9.00E-36	2.1	DPY19L1	2
PUM21	3.76E-40	0.5139794	0.503	0.406	9.06E-36	2.1	PUM2	1.238916256
CCDC83	3.81E-40	0.2335463	0.062	0.017	9.19E-36	2.1	CCDC83	3.647058824
EPB41L5	5.51E-40	0.5473984	0.398	0.287	1.33E-35	2.1	EPB41L5	1.386759582
SND11	1.33E-39	0.5714127	0.412	0.309	3.21E-35	2.1	SND1	1.333333333
RAD23B1	2.17E-39	0.4415338	0.571	0.477	5.22E-35	2.1	RAD23B	1.19706499

UBR2		2.73E-39	0.5124776	0.482	0.381	6.59E-35	2.1	UBR2	1.265091864
TMOD3		2.88E-39	0.4868746	0.502	0.394	6.94E-35	2.1	TMOD3	1.274111675
TMEM1651		3.68E-39	0.6082975	0.587	0.516	8.86E-35	2.1	TMEM165	1.137596899
PIP4K2A		3.94E-39	0.5860103	0.203	0.111	9.49E-35	2.1	PIP4K2A	1.828828829
AIM1		4.08E-39	0.5597875	0.541	0.438	9.83E-35	2.1	AIM1	1.235159817
NUP1531		5.56E-39	0.506106	0.385	0.274	1.34E-34	2.1	NUP153	1.405109489
ARL5B		5.58E-39	0.3916244	0.193	0.1	1.34E-34	2.1	ARL5B	1.93
GATSL2		8.21E-39	0.2857821	0.094	0.034	1.98E-34	2.1	GATSL2	2.764705882
CMYA5		9.12E-39	0.4554645	0.189	0.098	2.20E-34	2.1	CMYA5	1.928571429
NFIL31		1.32E-38	0.4757215	0.287	0.18	3.19E-34	2.1	NFIL3	1.594444444
CXCL81		1.96E-38	0.4980531	0.551	0.436	4.73E-34	2.1	CXCL8	1.263761468
AP1S3		3.36E-38	0.4292503	0.211	0.115	8.11E-34	2.1	AP1S3	1.834782609
SCUBE2		5.23E-38	0.375357	0.127	0.055	1.26E-33	2.1	SCUBE2	2.309090909
HERPUD11		7.85E-38	0.5911265	0.319	0.215	1.89E-33	2.1	HERPUD1	1.48372093
MANF		8.05E-38	0.3348436	0.129	0.057	1.94E-33	2.1	MANF	2.263157895
NFAT51		8.77E-38	0.4423623	0.682	0.624	2.11E-33	2.1	NFAT5	1.092948718
NPEPPS1		9.60E-38	0.5087538	0.569	0.489	2.32E-33	2.1	NPEPPS	1.163599182
STX18		1.03E-37	0.4526244	0.269	0.166	2.48E-33	2.1	STX18	1.620481928
PDIA31		1.79E-37	0.4769335	0.409	0.298	4.32E-33	2.1	PDIA3	1.372483221
HNRNPC1		2.09E-37	0.3320027	0.807	0.782	5.05E-33	2.1	HNRNPC	1.031969309
ZNF165		2.40E-37	0.475246	0.181	0.094	5.78E-33	2.1	ZNF165	1.925531915
TLL2		2.85E-37	0.2162406	0.053	0.013	6.86E-33	2.1	TLL2	4.076923077
MSRB3		4.17E-37	0.2261773	0.056	0.015	1.01E-32	2.1	MSRB3	3.733333333
DUSP16		5.37E-37	0.4873783	0.519	0.413	1.29E-32	2.1	DUSP16	1.256658596
PRDX3		5.39E-37	0.4664059	0.324	0.217	1.30E-32	2.1	PRDX3	1.493087558
ABI12		1.57E-36	0.3971978	0.731	0.674	3.79E-32	2.1	ABI1	1.084569733
PLEKHA8		1.86E-36	0.4534367	0.201	0.111	4.50E-32	2.1	PLEKHA8	1.810810811
RP11-90K6.1		2.53E-36	0.2018691	0.042	0.009	6.10E-32	2.1	RP11-90K6	4.666666667
ATXN2		3.43E-36	0.5438614	0.425	0.327	8.27E-32	2.1	ATXN2	1.29969419
APLP2		5.93E-36	0.4617677	0.378	0.269	1.43E-31	2.1	APLP2	1.405204461
ECE1		6.57E-36	0.5059003	0.281	0.179	1.59E-31	2.1	ECE1	1.569832402
GLRA1		2.42E-35	0.2315948	0.054	0.014	5.83E-31	2.1	GLRA1	3.857142857
LYST		3.01E-35	0.493375	0.372	0.265	7.27E-31	2.1	LYST	1.403773585
LINC01031		4.59E-35	0.1861743	0.051	0.013	1.11E-30	2.1	LINC01031	3.923076923
FANK1		4.79E-35	0.4749256	0.238	0.143	1.15E-30	2.1	FANK1	1.664335664
AFF3		4.83E-35	0.1679763	0.474	0.327	1.17E-30	2.1	AFF3	1.449541284
MKLN1		5.28E-35	0.3844364	0.713	0.659	1.27E-30	2.1	MKLN1	1.081942337
	Sep-04	5.94E-35	0.1740413	0.055	0.015	1.43E-30	2.1	Sep-04	3.666666667
WDR44		6.75E-35	0.4611584	0.253	0.156	1.63E-30	2.1	WDR44	1.621794872
PPP3CA		7.87E-35	0.4708959	0.638	0.558	1.90E-30	2.1	PPP3CA	1.143369176
ZBTB10		1.16E-34	0.5098104	0.35	0.249	2.79E-30	2.1	ZBTB10	1.40562249
ARSJ		1.56E-34	0.3291181	0.115	0.05	3.77E-30	2.1	ARSJ	2.3
SELK2		1.95E-34	0.5649251	0.655	0.608	4.69E-30	2.1	SELK	1.077302632
RNF10		2.79E-34	0.4893459	0.317	0.217	6.72E-30	2.1	RNF10	1.460829493
CTNND1		2.84E-34	0.4797334	0.474	0.389	6.85E-30	2.1	CTNND1	1.218508997
TNRC6B		3.44E-34	0.5134507	0.554	0.482	8.29E-30	2.1	TNRC6B	1.149377593
KDM2A		3.89E-34	0.4741967	0.51	0.431	9.37E-30	2.1	KDM2A	1.183294664
GPBP1L1		4.07E-34	0.4844382	0.43	0.336	9.81E-30	2.1	GPBP1L1	1.279761905
CERS6		4.56E-34	0.4662414	0.256	0.158	1.10E-29	2.1	CERS6	1.620253165
UBE2R2		1.01E-33	0.4707505	0.451	0.363	2.44E-29	2.1	UBE2R2	1.242424242
TMEM41B		1.12E-33	0.522404	0.339	0.242	2.70E-29	2.1	TMEM41B	1.400826446
PIK3C2A		1.76E-33	0.5484421	0.393	0.301	4.24E-29	2.1	PIK3C2A	1.305647841

MANBA	1.92E-33	0.4076707	0.127	0.059	4.63E-29	2.1	MANBA	2.152542373
SEMA3E	2.56E-33	0.4657707	0.226	0.135	6.17E-29	2.1	SEMA3E	1.674074074
COPA	2.88E-33	0.5824942	0.398	0.311	6.96E-29	2.1	COPA	1.279742765
RAB181	3.14E-33	0.4739969	0.429	0.332	7.58E-29	2.1	RAB18	1.292168675
SERF2	3.44E-33	0.3881839	0.491	0.386	8.30E-29	2.1	SERF2	1.272020725
TMCO3	3.58E-33	0.4144551	0.22	0.13	8.63E-29	2.1	TMCO3	1.692307692
CHMP4B	9.54E-33	0.4121209	0.282	0.184	2.30E-28	2.1	CHMP4B	1.532608696
PRKG1	1.20E-32	0.6816778	0.234	0.141	2.89E-28	2.1	PRKG1	1.659574468
PSME42	1.33E-32	0.2942496	0.705	0.634	3.21E-28	2.1	PSME4	1.111987382
SLC7A8	1.95E-32	0.2399762	0.097	0.039	4.70E-28	2.1	SLC7A8	2.487179487
DENND5A1	3.76E-32	0.4598177	0.427	0.331	9.07E-28	2.1	DENND5A	1.290030211
WAC1	4.01E-32	0.3757826	0.677	0.625	9.66E-28	2.1	WAC	1.0832
CD99	5.37E-32	0.3752733	0.221	0.129	1.29E-27	2.1	CD99	1.713178295
RB1CC1	6.80E-32	0.4341471	0.552	0.477	1.64E-27	2.1	RB1CC1	1.157232704
CYTH1	9.36E-32	0.391192	0.237	0.146	2.26E-27	2.1	CYTH1	1.623287671
PTPN13	9.98E-32	0.4962256	0.269	0.172	2.41E-27	2.1	PTPN13	1.563953488
CNOT2	1.20E-31	0.5324322	0.474	0.398	2.88E-27	2.1	CNOT2	1.190954774
GLI3	1.22E-31	0.4045662	0.218	0.129	2.95E-27	2.1	GLI3	1.689922481
CALR	1.36E-31	0.4781815	0.318	0.219	3.28E-27	2.1	CALR	1.452054795
GADD45A1	1.44E-31	0.4620872	0.361	0.258	3.48E-27	2.1	GADD45A	1.399224806
AC017101.10	1.52E-31	0.3803176	0.163	0.086	3.67E-27	2.1	AC017101.	1.895348837
GADD45B	2.22E-31	0.4640195	0.186	0.103	5.35E-27	2.1	GADD45B	1.805825243
HS6ST2	4.21E-31	0.4171281	0.232	0.14	1.01E-26	2.1	HS6ST2	1.657142857
PLEKHB2	5.05E-31	0.3957255	0.249	0.156	1.22E-26	2.1	PLEKHB2	1.596153846
STX17-AS1	5.28E-31	0.1869151	0.047	0.013	1.27E-26	2.1	STX17-AS1	3.615384615
USP34	7.59E-31	0.3630463	0.697	0.648	1.83E-26	2.1	USP34	1.075617284
USP25	7.92E-31	0.536388	0.344	0.252	1.91E-26	2.1	USP25	1.365079365
TFF1	9.50E-31	0.5342343	0.182	0.099	2.29E-26	2.1	TFF1	1.838383838
ABHD18	9.85E-31	0.5123625	0.429	0.337	2.38E-26	2.1	ABHD18	1.272997033
NDUFV2	1.03E-30	0.4446587	0.362	0.263	2.48E-26	2.1	NDUFV2	1.376425856
RNF149	1.28E-30	0.5012165	0.427	0.341	3.08E-26	2.1	RNF149	1.252199413
HK21	1.78E-30	0.5186967	0.25	0.16	4.30E-26	2.1	HK2	1.5625
BMP2K	3.32E-30	0.4275716	0.171	0.094	8.00E-26	2.1	BMP2K	1.819148936
ZNF1431	3.35E-30	0.4449479	0.236	0.149	8.07E-26	2.1	ZNF143	1.583892617
LNX2	3.73E-30	0.4254632	0.228	0.14	9.00E-26	2.1	LNX2	1.628571429
CAPZB	4.62E-30	0.4440775	0.273	0.182	1.11E-25	2.1	CAPZB	1.5
PIP4K2C	5.42E-30	0.3533453	0.142	0.072	1.31E-25	2.1	PIP4K2C	1.972222222
PRPS2	6.08E-30	0.296035	0.147	0.075	1.47E-25	2.1	PRPS2	1.96
LVRN	8.06E-30	0.1442521	0.035	0.008	1.94E-25	2.1	LVRN	4.375
TNIK	8.86E-30	0.4440068	0.246	0.154	2.14E-25	2.1	TNIK	1.597402597
OSBPL8	1.30E-29	0.5703314	0.322	0.232	3.13E-25	2.1	OSBPL8	1.387931034
WWP1	1.61E-29	0.47511	0.394	0.304	3.89E-25	2.1	WWP1	1.296052632
SMURF1	1.83E-29	0.488445	0.447	0.36	4.40E-25	2.1	SMURF1	1.241666667
NPTN	2.33E-29	0.4280872	0.281	0.189	5.61E-25	2.1	NPTN	1.486772487
KCMF11	2.34E-29	0.3996637	0.424	0.33	5.63E-25	2.1	KCMF1	1.284848485
EDEM1	2.53E-29	0.2645442	0.091	0.038	6.10E-25	2.1	EDEM1	2.394736842
PICALM	2.54E-29	0.4453512	0.515	0.442	6.13E-25	2.1	PICALM	1.165158371
RP1-313I6.12	2.74E-29	0.3663275	0.166	0.09	6.61E-25	2.1	RP1-313I6.	1.844444444
QKI1	3.05E-29	0.4290424	0.601	0.535	7.35E-25	2.1	QKI	1.123364486
MALRD1	3.32E-29	0.3045291	0.096	0.041	8.01E-25	2.1	MALRD1	2.341463415
NBPF14	3.75E-29	0.4230051	0.238	0.151	9.05E-25	2.1	NBPF14	1.57615894
RIC1	4.11E-29	0.5070044	0.256	0.17	9.91E-25	2.1	RIC1	1.505882353

HEPACAM2	4.51E-29	0.229894	0.092	0.038	1.09E-24	2.1	HEPACAM2	2.421052632
PIK3CA	5.42E-29	0.4432939	0.369	0.279	1.31E-24	2.1	PIK3CA	1.322580645
RP11-274H2.2	9.61E-29	0.4175747	0.121	0.058	2.32E-24	2.1	RP11-274H	2.086206897
TMEM136	9.81E-29	0.442093	0.157	0.085	2.37E-24	2.1	TMEM136	1.847058824
RP3-404K8.2	1.41E-28	0.1367761	0.035	0.008	3.39E-24	2.1	RP3-404K8	4.375
ZNF395	1.60E-28	0.2928202	0.095	0.041	3.86E-24	2.1	ZNF395	2.317073171
DCUN1D2	1.68E-28	0.3444015	0.131	0.066	4.06E-24	2.1	DCUN1D2	1.984848485
SLAIN2	1.68E-28	0.4193976	0.217	0.134	4.06E-24	2.1	SLAIN2	1.619402985
RP11-84A19.41	2.20E-28	0.2750541	0.109	0.05	5.30E-24	2.1	RP11-84A1	2.18
TMED2	2.28E-28	0.4191782	0.278	0.189	5.50E-24	2.1	TMED2	1.470899471
FILIP1L	2.33E-28	0.3729295	0.184	0.106	5.63E-24	2.1	FILIP1L	1.735849057
GPR75-ASB3	3.57E-28	0.4731908	0.245	0.161	8.61E-24	2.1	GPR75-ASB	1.52173913
DLC1	5.38E-28	0.4048373	0.123	0.06	1.30E-23	2.1	DLC1	2.05
MKL11	6.66E-28	0.4146643	0.558	0.492	1.60E-23	2.1	MKL1	1.134146341
CREBZF	7.30E-28	0.34262	0.126	0.062	1.76E-23	2.1	CREBZF	2.032258065
LINC00290	1.29E-27	0.35982	0.07	0.026	3.12E-23	2.1	LINC00290	2.692307692
PLAC91	1.32E-27	0.2102927	0.059	0.02	3.17E-23	2.1	PLAC9	2.95
DIO2	2.33E-27	0.185494	0.327	0.215	5.62E-23	2.1	DIO2	1.520930233
ASB15	2.40E-27	0.1103281	0.028	0.005	5.80E-23	2.1	ASB15	5.6
SLC41A2	2.70E-27	0.4737919	0.19	0.114	6.52E-23	2.1	SLC41A2	1.666666667
LARGE	3.00E-27	0.5275998	0.405	0.321	7.23E-23	2.1	LARGE	1.261682243
RANBP10	3.81E-27	0.3667609	0.148	0.08	9.18E-23	2.1	RANBP10	1.85
KMT2A1	3.90E-27	0.41104	0.489	0.415	9.41E-23	2.1	KMT2A	1.178313253
NFRKB	4.10E-27	0.3533807	0.144	0.077	9.90E-23	2.1	NFRKB	1.87012987
CSGALNACT2	4.60E-27	0.3503583	0.199	0.12	1.11E-22	2.1	CSGALNAC	1.658333333
SNX311	4.62E-27	0.2409222	0.081	0.033	1.11E-22	2.1	SNX31	2.454545455
UGDH-AS1	6.00E-27	0.4575708	0.179	0.104	1.45E-22	2.1	UGDH-AS1	1.721153846
GOLM1	7.11E-27	0.4144388	0.322	0.226	1.71E-22	2.1	GOLM1	1.424778761
RAB30	9.07E-27	0.3980229	0.194	0.116	2.19E-22	2.1	RAB30	1.672413793
GS1-114I9.3	1.22E-26	0.4799667	0.281	0.197	2.95E-22	2.1	GS1-114I9.	1.426395939
PPP1R15B	1.68E-26	0.3241183	0.13	0.067	4.05E-22	2.1	PPP1R15B	1.940298507
CKS2	1.81E-26	0.4351158	0.297	0.211	4.37E-22	2.1	CKS2	1.407582938
FSIP1	1.95E-26	0.301285	0.104	0.048	4.70E-22	2.1	FSIP1	2.166666667
AFTPH1	2.21E-26	0.4567931	0.393	0.308	5.32E-22	2.1	AFTPH	1.275974026
BANP	2.22E-26	0.3507139	0.146	0.079	5.35E-22	2.1	BANP	1.848101266
SLC38A1	2.85E-26	0.4475289	0.468	0.387	6.88E-22	2.1	SLC38A1	1.209302326
EGOT	3.28E-26	0.407258	0.185	0.11	7.90E-22	2.1	EGOT	1.681818182
TSPAN13	3.31E-26	0.333074	0.182	0.107	7.98E-22	2.1	TSPAN13	1.700934579
KAT6B	3.58E-26	0.4828641	0.337	0.25	8.62E-22	2.1	KAT6B	1.348
SCGB2A2	4.36E-26	0.3268719	0.374	0.265	1.05E-21	2.1	SCGB2A2	1.411320755
CD44	5.12E-26	0.3632058	0.647	0.585	1.23E-21	2.1	CD44	1.105982906
CDYL	5.59E-26	0.4315186	0.392	0.306	1.35E-21	2.1	CDYL	1.281045752
RP11-390E23.3	5.63E-26	0.1511743	0.038	0.01	1.36E-21	2.1	RP11-390E	3.8
RAPGEF4	7.08E-26	0.4658294	0.201	0.124	1.71E-21	2.1	RAPGEF4	1.620967742
LPAR3	7.65E-26	0.21241	0.087	0.037	1.85E-21	2.1	LPAR3	2.351351351
FAM188A	7.81E-26	0.3564595	0.166	0.095	1.88E-21	2.1	FAM188A	1.747368421
LINC01476	8.24E-26	0.2509632	0.063	0.023	1.99E-21	2.1	LINC01476	2.739130435
IER3	9.77E-26	0.5815977	0.404	0.312	2.36E-21	2.1	IER3	1.294871795
IMMP1L	1.10E-25	0.3814624	0.21	0.133	2.64E-21	2.1	IMMP1L	1.578947368
AP3S1	1.10E-25	0.3791399	0.176	0.105	2.64E-21	2.1	AP3S1	1.676190476
EFR3A	1.38E-25	0.4816052	0.284	0.203	3.33E-21	2.1	EFR3A	1.399014778
BRWD1	1.51E-25	0.4794372	0.441	0.369	3.63E-21	2.1	BRWD1	1.195121951

MORC31	1.63E-25	0.4289769	0.317	0.233	3.92E-21	2.1	MORC3	1.360515021
LEMD3	2.07E-25	0.3361273	0.153	0.086	4.98E-21	2.1	LEMD3	1.779069767
PAWR	2.78E-25	0.4163599	0.603	0.546	6.69E-21	2.1	PAWR	1.104395604
PGR	3.17E-25	0.3281165	0.143	0.077	7.65E-21	2.1	PGR	1.857142857
KIAA0430	3.33E-25	0.3941344	0.199	0.125	8.03E-21	2.1	KIAA0430	1.592
F11R1	3.52E-25	0.4247648	0.276	0.194	8.49E-21	2.1	F11R	1.422680412
LIFR1	3.82E-25	0.4943424	0.223	0.145	9.22E-21	2.1	LIFR	1.537931034
IL1RN	5.48E-25	0.2614415	0.101	0.047	1.32E-20	2.1	IL1RN	2.14893617
RSF1	6.83E-25	0.4279711	0.442	0.368	1.65E-20	2.1	RSF1	1.201086957
LPP	7.65E-25	0.2350203	0.933	0.914	1.84E-20	2.1	LPP	1.020787746
RAB3IP	8.10E-25	0.3893921	0.212	0.135	1.95E-20	2.1	RAB3IP	1.57037037
CFAP1611	9.69E-25	0.327439	0.082	0.035	2.34E-20	2.1	CFAP161	2.342857143
ZSCAN16-AS1	1.24E-24	0.3207699	0.152	0.086	2.98E-20	2.1	ZSCAN16-A	1.76744186
CFAP70	1.33E-24	0.2625559	0.087	0.038	3.21E-20	2.1	CFAP70	2.289473684
GAPVD11	1.38E-24	0.4574005	0.305	0.226	3.32E-20	2.1	GAPVD1	1.349557522
CASK	1.49E-24	0.4894929	0.436	0.367	3.59E-20	2.1	CASK	1.188010899
RP11-48B3.4	1.51E-24	0.1675803	0.046	0.014	3.64E-20	2.1	RP11-48B3	3.285714286
SBNO1	1.54E-24	0.3926423	0.222	0.145	3.72E-20	2.1	SBNO1	1.531034483
SLC17A8	1.83E-24	0.1925008	0.048	0.015	4.41E-20	2.1	SLC17A8	3.2
AKIRIN1	1.83E-24	0.3698261	0.194	0.121	4.42E-20	2.1	AKIRIN1	1.603305785
PHF12	1.93E-24	0.352996	0.163	0.095	4.65E-20	2.1	PHF12	1.715789474
HMGN5	2.05E-24	0.3000703	0.123	0.063	4.94E-20	2.1	HMGN5	1.952380952
BICC1	2.61E-24	0.3019586	0.077	0.033	6.30E-20	2.1	BICC1	2.333333333
PCBP11	2.68E-24	0.3957315	0.555	0.501	6.47E-20	2.1	PCBP1	1.107784431
FOXK1	2.83E-24	0.4327727	0.256	0.177	6.82E-20	2.1	FOXK1	1.446327684
DRC1	3.65E-24	0.10694	0.03	0.007	8.80E-20	2.1	DRC1	4.285714286
FNIP2	5.02E-24	0.3878482	0.173	0.102	1.21E-19	2.1	FNIP2	1.696078431
PLK2	5.34E-24	0.5683975	0.2	0.128	1.29E-19	2.1	PLK2	1.5625
JAG1	5.53E-24	0.2865306	0.086	0.038	1.33E-19	2.1	JAG1	2.263157895
ZDHHC7	7.51E-24	0.2756659	0.118	0.061	1.81E-19	2.1	ZDHHC7	1.93442623
PARD6B	8.59E-24	0.407505	0.255	0.175	2.07E-19	2.1	PARD6B	1.457142857
RBMS1	1.21E-23	0.4885207	0.409	0.339	2.91E-19	2.1	RBMS1	1.206489676
EIF2AK31	1.32E-23	0.3326418	0.447	0.355	3.18E-19	2.1	EIF2AK3	1.25915493
CBL	1.44E-23	0.4122784	0.181	0.111	3.48E-19	2.1	CBL	1.630630631
RBBP6	1.62E-23	0.4084879	0.355	0.275	3.90E-19	2.1	RBBP6	1.290909091
YPEL2	1.83E-23	0.3556964	0.18	0.11	4.41E-19	2.1	YPEL2	1.636363636
ATF21	2.20E-23	0.3366711	0.209	0.134	5.31E-19	2.1	ATF2	1.559701493
KDM6A	2.31E-23	0.4406006	0.431	0.362	5.57E-19	2.1	KDM6A	1.190607735
AC106900.6	2.44E-23	0.1503444	0.038	0.011	5.89E-19	2.1	AC106900.(	3.454545455
PTP4A2	3.21E-23	0.4047478	0.216	0.142	7.75E-19	2.1	PTP4A2	1.521126761
DEPDC1B	3.45E-23	0.1832693	0.037	0.01	8.33E-19	2.1	DEPDC1B	3.7
QSOX1	3.80E-23	0.3870873	0.223	0.146	9.16E-19	2.1	QSOX1	1.52739726
WHSC1	4.07E-23	0.4171141	0.25	0.173	9.81E-19	2.1	WHSC1	1.445086705
NPFFR2	4.37E-23	0.1974932	0.06	0.023	1.05E-18	2.1	NPFFR2	2.608695652
DCAF63	5.23E-23	0.4101354	0.486	0.424	1.26E-18	2.1	DCAF6	1.146226415
ATP5EP2	5.97E-23	0.4207164	0.379	0.298	1.44E-18	2.1	ATP5EP2	1.271812081
RP11-96O20.4	7.14E-23	0.1224436	0.033	0.009	1.72E-18	2.1	RP11-96O2	3.666666667
DNAJC3	8.12E-23	0.4555698	0.329	0.254	1.96E-18	2.1	DNAJC3	1.295275591
FAM69A	8.22E-23	0.4545408	0.15	0.086	1.98E-18	2.1	FAM69A	1.744186047
SGMS1	8.22E-23	0.5022197	0.38	0.305	1.98E-18	2.1	SGMS1	1.245901639
TCF12	8.64E-23	0.3964307	0.627	0.592	2.08E-18	2.1	TCF12	1.059121622
PDGFB	1.34E-22	0.2747109	0.082	0.036	3.22E-18	2.1	PDGFB	2.277777778

SPDYA	1.49E-22	0.3887085	0.165	0.098	3.59E-18	2.1	SPDYA	1.683673469
LMCD1	1.57E-22	0.3526299	0.173	0.103	3.79E-18	2.1	LMCD1	1.67961165
NUTM2A-AS11	1.85E-22	0.4878765	0.366	0.297	4.47E-18	2.1	NUTM2A-A	1.232323232
SCAF41	1.89E-22	0.3277587	0.166	0.099	4.55E-18	2.1	SCAF4	1.676767677
ZFAND2A	1.95E-22	0.2335819	0.08	0.035	4.69E-18	2.1	ZFAND2A	2.285714286
DIXDC1	2.09E-22	0.5274392	0.179	0.111	5.05E-18	2.1	DIXDC1	1.612612613
KANSL1	2.40E-22	0.3907901	0.474	0.407	5.80E-18	2.1	KANSL1	1.164619165
NBPF19	2.62E-22	0.4202714	0.288	0.209	6.33E-18	2.1	NBPF19	1.377990431
RAPGEF22	2.85E-22	0.3724658	0.493	0.416	6.87E-18	2.1	RAPGEF2	1.185096154
CDC271	3.58E-22	0.3883914	0.335	0.257	8.63E-18	2.1	CDC27	1.303501946
SFMBT2	3.61E-22	0.3971656	0.241	0.163	8.71E-18	2.1	SFMBT2	1.478527607
ARHGEF381	3.72E-22	0.5312059	0.53	0.481	8.96E-18	2.1	ARHGEF38	1.101871102
MEGF9	3.79E-22	0.3973374	0.199	0.128	9.15E-18	2.1	MEGF9	1.5546875
MLEC	5.36E-22	0.2807405	0.136	0.076	1.29E-17	2.1	MLEC	1.789473684
PKP2	6.73E-22	0.3700176	0.152	0.09	1.62E-17	2.1	PKP2	1.688888889
ELF2	7.94E-22	0.4336166	0.449	0.384	1.91E-17	2.1	ELF2	1.169270833
UBE2K	8.85E-22	0.416756	0.435	0.377	2.13E-17	2.1	UBE2K	1.153846154
LINC01214	8.99E-22	0.1368631	0.038	0.011	2.17E-17	2.1	LINC01214	3.454545455
ATP5E	9.25E-22	0.3868128	0.589	0.554	2.23E-17	2.1	ATP5E	1.063176895
WDR481	1.02E-21	0.3884735	0.256	0.183	2.45E-17	2.1	WDR48	1.398907104
MTURN	1.35E-21	0.4181458	0.228	0.157	3.27E-17	2.1	MTURN	1.452229299
KLF12	1.95E-21	0.4292337	0.215	0.141	4.71E-17	2.1	KLF12	1.524822695
GSG1L	3.43E-21	0.1515864	0.044	0.015	8.28E-17	2.1	GSG1L	2.933333333
YY1	3.64E-21	0.3736313	0.289	0.215	8.77E-17	2.1	YY1	1.344186047
PGM32	3.70E-21	0.3313951	0.159	0.096	8.91E-17	2.1	PGM3	1.65625
ARID5B1	3.93E-21	0.2932847	0.756	0.732	9.47E-17	2.1	ARID5B	1.032786885
MSL1	3.98E-21	0.3775629	0.218	0.147	9.59E-17	2.1	MSL1	1.482993197
ACVR1C	4.11E-21	0.2183008	0.058	0.023	9.91E-17	2.1	ACVR1C	2.52173913
CMBL	4.23E-21	0.1677361	0.055	0.021	1.02E-16	2.1	CMBL	2.619047619
SYT9	5.72E-21	0.2351679	0.086	0.04	1.38E-16	2.1	SYT9	2.15
RAPH1	7.44E-21	0.4274001	0.403	0.331	1.79E-16	2.1	RAPH1	1.217522659
FEM1C	7.99E-21	0.3238444	0.133	0.075	1.93E-16	2.1	FEM1C	1.773333333
COL4A5	8.25E-21	0.3340951	0.298	0.213	1.99E-16	2.1	COL4A5	1.399061033
LMTK2	8.63E-21	0.4339901	0.259	0.187	2.08E-16	2.1	LMTK2	1.385026738
SLMAP1	8.73E-21	0.3418467	0.694	0.673	2.10E-16	2.1	SLMAP	1.031203566
NF1	8.91E-21	0.403064	0.544	0.502	2.15E-16	2.1	NF1	1.083665339
INTS2	1.41E-20	0.2635647	0.086	0.041	3.40E-16	2.1	INTS2	2.097560976
RNF11	1.65E-20	0.3508046	0.317	0.241	3.99E-16	2.1	RNF11	1.315352697
CTD-2528L19.4	1.75E-20	0.2154519	0.091	0.044	4.22E-16	2.1	CTD-2528L	2.068181818
RP11-486O13.4	1.79E-20	0.3064816	0.163	0.099	4.32E-16	2.1	RP11-486O	1.646464646
MAP3K19	2.21E-20	0.1728912	0.053	0.02	5.32E-16	2.1	MAP3K19	2.65
LAPTM4A	2.29E-20	0.4026877	0.392	0.323	5.52E-16	2.1	LAPTM4A	1.213622291
HTT	2.62E-20	0.3641979	0.233	0.162	6.31E-16	2.1	HTT	1.438271605
MIB1	2.97E-20	0.3888474	0.445	0.379	7.15E-16	2.1	MIB1	1.17414248
RP13-270P17.3	3.24E-20	0.3571478	0.073	0.033	7.82E-16	2.1	RP13-270P	2.212121212
THRAP3	3.88E-20	0.4277528	0.348	0.282	9.36E-16	2.1	THRAP3	1.234042553
PEBP4	5.83E-20	0.2847262	0.153	0.091	1.41E-15	2.1	PEBP4	1.681318681
SOX4	6.57E-20	0.2582769	0.882	0.835	1.58E-15	2.1	SOX4	1.056287425
AMPH	6.66E-20	0.368946	0.159	0.096	1.61E-15	2.1	AMPH	1.65625
MGLL	7.57E-20	0.367861	0.255	0.178	1.82E-15	2.1	MGLL	1.43258427
ITSN1	7.69E-20	0.4059823	0.263	0.191	1.85E-15	2.1	ITSN1	1.376963351
PIK3C3	7.79E-20	0.3094282	0.171	0.108	1.88E-15	2.1	PIK3C3	1.583333333

FBXO111	9.83E-20	0.3834348	0.45	0.387	2.37E-15	2.1	FBXO11	1.162790698
CCDC13	1.03E-19	0.1402279	0.042	0.014	2.48E-15	2.1	CCDC13	3
PDIA61	1.05E-19	0.3829832	0.298	0.225	2.52E-15	2.1	PDIA6	1.324444444
KLHL13	1.11E-19	0.2940357	0.17	0.104	2.69E-15	2.1	KLHL13	1.634615385
GPS2	1.18E-19	0.3980414	0.235	0.165	2.85E-15	2.1	GPS2	1.424242424
AZGP1	1.24E-19	0.2631791	0.629	0.541	2.99E-15	2.1	AZGP1	1.162661738
ATP5I	1.27E-19	0.3019633	0.208	0.138	3.06E-15	2.1	ATP5I	1.507246377
TSC1	1.39E-19	0.2517746	0.099	0.051	3.34E-15	2.1	TSC1	1.941176471
SARAF	1.42E-19	0.3599813	0.416	0.348	3.41E-15	2.1	SARAF	1.195402299
FGFR11	1.47E-19	0.4312638	0.259	0.187	3.53E-15	2.1	FGFR1	1.385026738
STAU1	1.54E-19	0.3720865	0.301	0.23	3.72E-15	2.1	STAU1	1.308695652
AC107218.3	1.62E-19	0.2212566	0.066	0.029	3.90E-15	2.1	AC107218.3	2.275862069
FEM1B1	2.08E-19	0.355454	0.234	0.164	5.01E-15	2.1	FEM1B	1.426829268
PCMTD11	2.17E-19	0.3770671	0.398	0.337	5.23E-15	2.1	PCMTD1	1.181008902
TBX3	2.66E-19	0.2655695	0.352	0.264	6.41E-15	2.1	TBX3	1.333333333
KMT2E	2.76E-19	0.3898897	0.514	0.462	6.67E-15	2.1	KMT2E	1.112554113
PPP2R2D	2.90E-19	0.3788543	0.245	0.175	6.99E-15	2.1	PPP2R2D	1.4
SEC23IP	2.97E-19	0.3175009	0.155	0.096	7.17E-15	2.1	SEC23IP	1.614583333
MGEA5	3.61E-19	0.4034893	0.459	0.405	8.71E-15	2.1	MGEA5	1.133333333
MYH92	4.61E-19	0.2704158	0.572	0.51	1.11E-14	2.1	MYH9	1.121568627
CTD-2033A16.3	5.30E-19	0.1720888	0.055	0.022	1.28E-14	2.1	CTD-2033A	2.5
AZIN1	5.31E-19	0.3665272	0.271	0.199	1.28E-14	2.1	AZIN1	1.361809045
CYTH3	6.17E-19	0.3669148	0.174	0.112	1.49E-14	2.1	CYTH3	1.553571429
ITGA91	6.79E-19	0.2250114	0.063	0.027	1.64E-14	2.1	ITGA9	2.333333333
GOLGB11	7.54E-19	0.3597595	0.432	0.37	1.82E-14	2.1	GOLGB1	1.167567568
BRWD3	7.92E-19	0.4449193	0.252	0.186	1.91E-14	2.1	BRWD3	1.35483871
SAMD4A1	8.52E-19	0.3726169	0.665	0.62	2.05E-14	2.1	SAMD4A	1.072580645
ADAM10	1.07E-18	0.3831355	0.434	0.37	2.59E-14	2.1	ADAM10	1.172972973
NFKBIA1	1.14E-18	0.3288049	0.664	0.625	2.76E-14	2.1	NFKBIA	1.0624
CCBE1	1.17E-18	0.2522358	0.091	0.046	2.82E-14	2.1	CCBE1	1.97826087
TOP2B1	1.22E-18	0.3466918	0.258	0.187	2.94E-14	2.1	TOP2B	1.379679144
CDK13	2.06E-18	0.3546781	0.478	0.42	4.96E-14	2.1	CDK13	1.138095238
KLHL28	2.34E-18	0.2807585	0.161	0.102	5.63E-14	2.1	KLHL28	1.578431373
ERP44	2.56E-18	0.3652136	0.202	0.138	6.17E-14	2.1	ERP44	1.463768116
TTC28	2.67E-18	0.4068891	0.225	0.158	6.43E-14	2.1	TTC28	1.424050633
PTPRK	2.72E-18	0.3428334	0.772	0.772	6.57E-14	2.1	PTPRK	1
RAB22A	3.85E-18	0.3078992	0.198	0.135	9.28E-14	2.1	RAB22A	1.466666667
AP000695.6	3.88E-18	0.1165515	0.026	0.007	9.36E-14	2.1	AP000695.6	3.714285714
KIAA1324	3.95E-18	0.3154643	0.297	0.223	9.52E-14	2.1	KIAA1324	1.331838565
RP11-252E2.2	4.10E-18	0.1269866	0.033	0.01	9.88E-14	2.1	RP11-252E2	3.3
FRZB	4.11E-18	0.1204669	0.036	0.012	9.92E-14	2.1	FRZB	3
DNAH5	4.90E-18	0.2571072	0.115	0.064	1.18E-13	2.1	DNAH5	1.796875
ROCK21	5.27E-18	0.3981551	0.351	0.285	1.27E-13	2.1	ROCK2	1.231578947
RP11-212D19.4	5.73E-18	0.1692116	0.07	0.032	1.38E-13	2.1	RP11-212D	2.1875
CDC37L1	6.02E-18	0.2718673	0.111	0.062	1.45E-13	2.1	CDC37L1	1.790322581
ITGA5	6.49E-18	0.3003304	0.154	0.096	1.57E-13	2.1	ITGA5	1.604166667
DNAH12	9.07E-18	0.2143545	0.054	0.022	2.19E-13	2.1	DNAH12	2.454545455
WEE11	9.10E-18	0.3610379	0.37	0.306	2.19E-13	2.1	WEE1	1.209150327
EMSY	9.44E-18	0.3587876	0.211	0.148	2.28E-13	2.1	EMSY	1.425675676
STRN1	9.55E-18	0.3767211	0.451	0.393	2.30E-13	2.1	STRN	1.147582697
RBFOX22	1.06E-17	0.2249268	0.942	0.953	2.54E-13	2.1	RBFOX2	0.988457503
TTC27	1.09E-17	0.4107239	0.099	0.054	2.63E-13	2.1	TTC27	1.833333333

RPS10-NUDT3	1.10E-17	0.2055314	0.086	0.044	2.66E-13	2.1	RPS10-NUI	1.954545455
EP300	1.23E-17	0.3062687	0.284	0.215	2.97E-13	2.1	EP300	1.320930233
CNN32	1.29E-17	0.3173982	0.56	0.512	3.12E-13	2.1	CNN3	1.09375
RP11-174G6.1	1.49E-17	0.2919256	0.109	0.061	3.59E-13	2.1	RP11-174G	1.786885246
AC093901.1	1.60E-17	0.1097901	0.028	0.008	3.87E-13	2.1	AC093901.	3.5
UACA1	1.61E-17	0.400103	0.259	0.195	3.88E-13	2.1	UACA	1.328205128
UBTD21	1.65E-17	0.3590254	0.209	0.146	3.99E-13	2.1	UBTD2	1.431506849
LINC01588	1.72E-17	0.2980207	0.154	0.097	4.15E-13	2.1	LINC01588	1.587628866
HOMER1	1.91E-17	0.360875	0.17	0.11	4.60E-13	2.1	HOMER1	1.545454545
RBM44	1.99E-17	0.1310953	0.038	0.013	4.79E-13	2.1	RBM44	2.923076923
ZBTB18	2.06E-17	0.2748947	0.119	0.069	4.98E-13	2.1	ZBTB18	1.724637681
PITPNB1	2.15E-17	0.3747688	0.366	0.305	5.18E-13	2.1	PITPNB	1.2
ERO1B	2.21E-17	0.3084644	0.15	0.094	5.32E-13	2.1	ERO1B	1.595744681
MTHFD2	2.58E-17	0.3080561	0.188	0.126	6.22E-13	2.1	MTHFD2	1.492063492
ARL8B1	3.08E-17	0.3672552	0.332	0.267	7.42E-13	2.1	ARL8B	1.243445693
MN1	3.41E-17	0.1286327	0.036	0.012	8.23E-13	2.1	MN1	3
STYK1	3.62E-17	0.3220107	0.111	0.063	8.73E-13	2.1	STYK1	1.761904762
GSR	4.12E-17	0.2350074	0.065	0.03	9.93E-13	2.1	GSR	2.166666667
EIF5AL1	4.12E-17	0.2179597	0.086	0.044	9.94E-13	2.1	EIF5AL1	1.954545455
PXDNL	4.25E-17	0.2186127	0.071	0.034	1.03E-12	2.1	PXDNL	2.088235294
NUSAP1	4.88E-17	0.2297676	0.09	0.047	1.18E-12	2.1	NUSAP1	1.914893617
PDK11	5.11E-17	0.3318883	0.147	0.092	1.23E-12	2.1	PDK1	1.597826087
CRY11	5.14E-17	0.3287063	0.447	0.381	1.24E-12	2.1	CRY1	1.173228346
REC114	5.27E-17	0.1122651	0.032	0.01	1.27E-12	2.1	REC114	3.2
CHSY11	5.41E-17	0.3607375	0.144	0.089	1.31E-12	2.1	CHSY1	1.617977528
BARD1	5.67E-17	0.3208883	0.141	0.088	1.37E-12	2.1	BARD1	1.602272727
STT3B	5.74E-17	0.3437835	0.243	0.179	1.38E-12	2.1	STT3B	1.357541899
LINC00607	5.98E-17	0.2442927	0.073	0.036	1.44E-12	2.1	LINC00607	2.027777778
ZNRF1	6.03E-17	0.3591427	0.226	0.162	1.46E-12	2.1	ZNRF1	1.395061728
TANC2	6.70E-17	0.2888598	0.543	0.471	1.62E-12	2.1	TANC2	1.152866242
DIP2B	7.12E-17	0.4090163	0.362	0.302	1.72E-12	2.1	DIP2B	1.198675497
MSI2	7.39E-17	0.3668701	0.399	0.339	1.78E-12	2.1	MSI2	1.17699115
BRAF	9.03E-17	0.3646038	0.469	0.417	2.18E-12	2.1	BRAF	1.12470024
GLUD1	1.41E-16	0.3504504	0.342	0.271	3.40E-12	2.1	GLUD1	1.26199262
TMEM59	1.73E-16	0.2895039	0.501	0.45	4.18E-12	2.1	TMEM59	1.113333333
IER5	1.86E-16	0.3295905	0.165	0.109	4.49E-12	2.1	IER5	1.513761468
VCL2	1.88E-16	0.429015	0.366	0.307	4.53E-12	2.1	VCL	1.19218241
RARA	2.08E-16	0.1511697	0.048	0.019	5.02E-12	2.1	RARA	2.526315789
EIF1	2.17E-16	0.2171578	0.537	0.476	5.24E-12	2.1	EIF1	1.128151261
FAM177A1	2.20E-16	0.2891107	0.179	0.121	5.32E-12	2.1	FAM177A1	1.479338843
GOLT1B	2.48E-16	0.3319456	0.199	0.139	5.98E-12	2.1	GOLT1B	1.431654676
DDB1	2.66E-16	0.3165554	0.184	0.124	6.40E-12	2.1	DDB1	1.483870968
ASPH	2.75E-16	0.3458019	0.35	0.28	6.63E-12	2.1	ASPH	1.25
NQO1	3.08E-16	0.1870487	0.107	0.06	7.44E-12	2.1	NQO1	1.783333333
AK3	3.56E-16	0.3514365	0.265	0.203	8.59E-12	2.1	AK3	1.305418719
GNAI3	4.24E-16	0.3104817	0.246	0.182	1.02E-11	2.1	GNAI3	1.351648352
WLS	4.96E-16	0.2724856	0.237	0.17	1.20E-11	2.1	WLS	1.394117647
C3orf35	5.34E-16	0.2763592	0.1	0.056	1.29E-11	2.1	C3orf35	1.785714286
P4HB	5.46E-16	0.3405024	0.192	0.134	1.32E-11	2.1	P4HB	1.432835821
TMA7	5.80E-16	0.3012948	0.615	0.602	1.40E-11	2.1	TMA7	1.021594684
WAPL	6.47E-16	0.3424409	0.251	0.189	1.56E-11	2.1	WAPL	1.328042328
VEZT	7.31E-16	0.3632973	0.418	0.368	1.76E-11	2.1	VEZT	1.135869565

ACVR1	7.46E-16	0.3240104	0.194	0.135	1.80E-11	2.1	ACVR1	1.437037037
LINC01280	7.50E-16	0.108654	0.033	0.011	1.81E-11	2.1	LINC01280	3
SPAG91	8.38E-16	0.2955554	0.405	0.341	2.02E-11	2.1	SPAG9	1.187683284
EIF4G3	8.58E-16	0.3453904	0.565	0.508	2.07E-11	2.1	EIF4G3	1.112204724
DICER1	8.83E-16	0.3756235	0.261	0.199	2.13E-11	2.1	DICER1	1.311557789
CD164	1.04E-15	0.3414603	0.341	0.278	2.52E-11	2.1	CD164	1.226618705
IGFBP2	1.12E-15	0.199559	0.087	0.046	2.70E-11	2.1	IGFBP2	1.891304348
CFAP69	1.20E-15	0.2847794	0.156	0.101	2.89E-11	2.1	CFAP69	1.544554455
SEL1L1	1.20E-15	0.3034026	0.152	0.098	2.90E-11	2.1	SEL1L	1.551020408
TEAD11	1.27E-15	0.313743	0.504	0.449	3.06E-11	2.1	TEAD1	1.122494432
CST3	1.31E-15	0.2336563	0.109	0.063	3.16E-11	2.1	CST3	1.73015873
ABAT	1.34E-15	0.2536607	0.097	0.055	3.23E-11	2.1	ABAT	1.763636364
ANLN	1.80E-15	0.2195257	0.076	0.039	4.35E-11	2.1	ANLN	1.948717949
TRIM36	2.01E-15	0.1952648	0.065	0.031	4.86E-11	2.1	TRIM36	2.096774194
UQCRQ	2.03E-15	0.2699953	0.166	0.111	4.89E-11	2.1	UQCRQ	1.495495495
UBXN7	2.06E-15	0.2828921	0.155	0.103	4.97E-11	2.1	UBXN7	1.504854369
SPEN1	2.37E-15	0.3109395	0.325	0.262	5.72E-11	2.1	SPEN	1.240458015
CYB5A	2.47E-15	0.2533883	0.325	0.253	5.96E-11	2.1	CYB5A	1.28458498
BTBD7	2.61E-15	0.3597639	0.246	0.186	6.29E-11	2.1	BTBD7	1.322580645
RP11-1021N1.1	2.75E-15	0.1022294	0.028	0.009	6.63E-11	2.1	RP11-1021I	3.111111111
CSNK1A1	2.76E-15	0.359984	0.659	0.656	6.66E-11	2.1	CSNK1A1	1.004573171
PPM1B	2.95E-15	0.3365507	0.264	0.204	7.12E-11	2.1	PPM1B	1.294117647
ELOVL7	3.05E-15	0.3050476	0.157	0.102	7.36E-11	2.1	ELOVL7	1.539215686
UBE2E1	3.32E-15	0.3743923	0.392	0.342	8.01E-11	2.1	UBE2E1	1.14619883
RNF128	3.34E-15	0.2988718	0.181	0.123	8.05E-11	2.1	RNF128	1.471544715
ATG16L1	3.57E-15	0.2772042	0.083	0.044	8.60E-11	2.1	ATG16L1	1.886363636
TMEM156	3.63E-15	0.3352543	0.088	0.047	8.74E-11	2.1	TMEM156	1.872340426
GABARAP	3.66E-15	0.2795801	0.423	0.357	8.83E-11	2.1	GABARAP	1.18487395
CHST7	3.95E-15	0.1573059	0.052	0.023	9.52E-11	2.1	CHST7	2.260869565
RAC1	4.13E-15	0.3040406	0.54	0.509	9.95E-11	2.1	RAC1	1.060903733
EGLN3	4.32E-15	0.4098732	0.199	0.142	1.04E-10	2.1	EGLN3	1.401408451
NUP58	4.44E-15	0.3099303	0.188	0.132	1.07E-10	2.1	NUP58	1.424242424
HSPA13	4.48E-15	0.180097	0.091	0.05	1.08E-10	2.1	HSPA13	1.82
MYOF	6.44E-15	0.2503618	0.634	0.597	1.55E-10	2.1	MYOF	1.061976549
TAF1D2	6.53E-15	0.3224884	0.324	0.263	1.58E-10	2.1	TAF1D	1.231939163
ATP6AP2	6.56E-15	0.281307	0.198	0.14	1.58E-10	2.1	ATP6AP2	1.414285714
SLC18A2	6.80E-15	0.1425198	0.044	0.018	1.64E-10	2.1	SLC18A2	2.444444444
AGO21	7.10E-15	0.3413366	0.266	0.207	1.71E-10	2.1	AGO2	1.285024155
CCDC591	7.15E-15	0.3722016	0.239	0.183	1.72E-10	2.1	CCDC59	1.306010929
RTKN2	7.42E-15	0.2372686	0.091	0.05	1.79E-10	2.1	RTKN2	1.82
UBL5	7.43E-15	0.3339254	0.535	0.508	1.79E-10	2.1	UBL5	1.053149606
RP3-523E19.2	7.93E-15	0.2217637	0.076	0.04	1.91E-10	2.1	RP3-523E1!	1.9
RASSF32	8.53E-15	0.3638202	0.171	0.118	2.06E-10	2.1	RASSF3	1.449152542
KHDRBS11	9.29E-15	0.3910272	0.343	0.286	2.24E-10	2.1	KHDRBS1	1.199300699
SLC38A2	9.48E-15	0.2573178	0.448	0.377	2.29E-10	2.1	SLC38A2	1.188328912
WASF2	9.54E-15	0.3445746	0.306	0.246	2.30E-10	2.1	WASF2	1.243902439
HYOU1	1.00E-14	0.2313667	0.101	0.058	2.41E-10	2.1	HYOU1	1.74137931
FAM81B	1.06E-14	0.1123896	0.038	0.015	2.55E-10	2.1	FAM81B	2.533333333
SERPINA11	1.13E-14	0.1565887	0.066	0.032	2.72E-10	2.1	SERPINA11	2.0625
SMAD2	1.28E-14	0.3508191	0.354	0.3	3.09E-10	2.1	SMAD2	1.18
ANKLE21	1.34E-14	0.2990411	0.169	0.116	3.24E-10	2.1	ANKLE2	1.456896552
DRAM1	1.36E-14	0.3825413	0.349	0.293	3.28E-10	2.1	DRAM1	1.19112628

CHD1	1.64E-14	0.3239028	0.324	0.267	3.95E-10	2.1	CHD1	1.213483146
MREG	1.65E-14	0.2941178	0.194	0.137	3.98E-10	2.1	MREG	1.416058394
FNBP1L1	1.79E-14	0.358819	0.465	0.416	4.32E-10	2.1	FNBP1L	1.117788462
CACUL11	1.91E-14	0.3431159	0.423	0.376	4.61E-10	2.1	CACUL1	1.125
LINC01170	2.60E-14	0.2697668	0.061	0.029	6.27E-10	2.1	LINC01170	2.103448276
NCKAP1	3.01E-14	0.2980637	0.471	0.431	7.26E-10	2.1	NCKAP1	1.092807425
ZBED5-AS11	3.24E-14	0.1481095	0.059	0.028	7.81E-10	2.1	ZBED5-AS1	2.107142857
RPS6KB1	3.37E-14	0.234585	0.139	0.09	8.12E-10	2.1	RPS6KB1	1.544444444
KIF16B	4.49E-14	0.3294068	0.242	0.182	1.08E-09	2.1	KIF16B	1.32967033
RP11-350N15.41	4.78E-14	0.1189454	0.038	0.015	1.15E-09	2.1	RP11-350N	2.533333333
OSBP	6.53E-14	0.3502781	0.203	0.149	1.57E-09	2.1	OSBP	1.362416107
FGD6	7.12E-14	0.3958354	0.39	0.341	1.72E-09	2.1	FGD6	1.143695015
NFYA	7.88E-14	0.2238176	0.118	0.073	1.90E-09	2.1	NFYA	1.616438356
S100A141	8.84E-14	0.3161064	0.742	0.735	2.13E-09	2.1	S100A14	1.00952381
DCXR	8.88E-14	0.1137935	0.039	0.016	2.14E-09	2.1	DCXR	2.4375
SIDT2	9.08E-14	0.1601589	0.066	0.033	2.19E-09	2.1	SIDT2	2
GPD2	9.64E-14	0.3870357	0.378	0.328	2.33E-09	2.1	GPD2	1.152439024
CCNK	1.02E-13	0.3211357	0.196	0.142	2.46E-09	2.1	CCNK	1.38028169
ARFGEF2	1.10E-13	0.362712	0.41	0.37	2.64E-09	2.1	ARFGEF2	1.108108108
PPP2R5E	1.13E-13	0.4337636	0.335	0.287	2.73E-09	2.1	PPP2R5E	1.167247387
NEDD91	1.14E-13	0.3380999	0.426	0.368	2.74E-09	2.1	NEDD9	1.157608696
TMEM254	1.15E-13	0.1529591	0.077	0.041	2.77E-09	2.1	TMEM254	1.87804878
MARCH51	1.16E-13	0.2995846	0.284	0.224	2.80E-09	2.1	MARCH5	1.267857143
LSMEM1	1.28E-13	0.2299459	0.112	0.068	3.09E-09	2.1	LSMEM1	1.647058824
KDM3A1	1.36E-13	0.2901529	0.157	0.106	3.29E-09	2.1	KDM3A	1.481132075
MARK3	1.36E-13	0.2977907	0.502	0.467	3.29E-09	2.1	MARK3	1.074946467
CDR21	1.53E-13	0.3353285	0.225	0.17	3.69E-09	2.1	CDR2	1.323529412
PGRMC1	1.54E-13	0.2501352	0.128	0.082	3.72E-09	2.1	PGRMC1	1.56097561
HSPB8	1.62E-13	0.221269	0.117	0.072	3.90E-09	2.1	HSPB8	1.625
IL6ST	1.98E-13	0.3019881	0.382	0.33	4.78E-09	2.1	IL6ST	1.157575758
TMBIM6	2.20E-13	0.1262308	0.875	0.86	5.31E-09	2.1	TMBIM6	1.01744186
STX3	2.35E-13	0.2844159	0.189	0.136	5.66E-09	2.1	STX3	1.389705882
ACOT12	2.64E-13	0.1035628	0.027	0.009	6.36E-09	2.1	ACOT12	3
SPPL2A	2.88E-13	0.3172944	0.315	0.26	6.94E-09	2.1	SPPL2A	1.211538462
LUC7L3	2.96E-13	0.3348489	0.379	0.335	7.13E-09	2.1	LUC7L3	1.131343284
LMBR1	3.35E-13	0.3189235	0.196	0.144	8.08E-09	2.1	LMBR1	1.361111111
KLHDC10	3.45E-13	0.2879575	0.186	0.133	8.32E-09	2.1	KLHDC10	1.398496241
MARCH61	3.73E-13	0.2988542	0.442	0.398	8.99E-09	2.1	MARCH6	1.110552764
DHX15	4.92E-13	0.3216208	0.311	0.257	1.19E-08	2.1	DHX15	1.210116732
MTMR31	5.38E-13	0.3315004	0.249	0.196	1.30E-08	2.1	MTMR3	1.270408163
CLSTN2	6.26E-13	0.1883308	0.111	0.068	1.51E-08	2.1	CLSTN2	1.632352941
CTD-2060L22.1	6.26E-13	0.1606342	0.045	0.02	1.51E-08	2.1	CTD-2060L	2.25
CAPN8	7.01E-13	0.1841075	0.283	0.214	1.69E-08	2.1	CAPN8	1.322429907
DGKH	7.39E-13	0.4177065	0.357	0.305	1.78E-08	2.1	DGKH	1.170491803
MBTD1	7.45E-13	0.3499971	0.237	0.185	1.80E-08	2.1	MBTD1	1.281081081
FAM222A	7.58E-13	0.1808702	0.075	0.041	1.83E-08	2.1	FAM222A	1.829268293
TRIP12	7.69E-13	0.3141053	0.372	0.325	1.85E-08	2.1	TRIP12	1.144615385
PCNX1	8.08E-13	0.3277765	0.258	0.205	1.95E-08	2.1	PCNX	1.258536585
ENPP1	8.28E-13	0.2692558	0.184	0.131	2.00E-08	2.1	ENPP1	1.404580153
ANKRD11	9.55E-13	0.2767953	0.37	0.317	2.30E-08	2.1	ANKRD11	1.167192429
CAND1	9.97E-13	0.3030379	0.208	0.156	2.40E-08	2.1	CAND1	1.333333333
KIF2A1	1.01E-12	0.3504967	0.232	0.182	2.43E-08	2.1	KIF2A	1.274725275

DMXL1	1.02E-12	0.3238091	0.216	0.163	2.46E-08	2.1	DMXL1	1.325153374
GLO1	1.05E-12	0.318477	0.222	0.168	2.53E-08	2.1	GLO1	1.321428571
CUL3	1.06E-12	0.2904397	0.441	0.401	2.57E-08	2.1	CUL3	1.099750623
GLRX31	1.09E-12	0.2806621	0.169	0.119	2.62E-08	2.1	GLRX3	1.420168067
HELLS	1.15E-12	0.1724894	0.053	0.025	2.78E-08	2.1	HELLS	2.12
PKP41	1.16E-12	0.4030706	0.519	0.503	2.80E-08	2.1	PKP4	1.031809145
CRKL	1.18E-12	0.2279831	0.117	0.074	2.86E-08	2.1	CRKL	1.581081081
ATP6V1G1	1.33E-12	0.280833	0.431	0.387	3.21E-08	2.1	ATP6V1G1	1.11369509
ARHGEF21	1.48E-12	0.223103	0.125	0.08	3.57E-08	2.1	ARHGEF2	1.5625
RAB26	1.63E-12	0.1562448	0.066	0.034	3.93E-08	2.1	RAB26	1.941176471
MEAF61	1.72E-12	0.2881675	0.219	0.165	4.15E-08	2.1	MEAF6	1.327272727
ZC3HAV1	1.82E-12	0.3398705	0.237	0.185	4.39E-08	2.1	ZC3HAV1	1.281081081
GOLPH3L2	1.83E-12	0.317008	0.246	0.194	4.42E-08	2.1	GOLPH3L	1.268041237
ATF31	1.86E-12	0.2497798	0.456	0.403	4.49E-08	2.1	ATF3	1.131513648
EIF4G2	1.97E-12	0.2817826	0.434	0.39	4.75E-08	2.1	EIF4G2	1.112820513
TBC1D15	2.31E-12	0.3514249	0.358	0.317	5.58E-08	2.1	TBC1D15	1.129337539
KIAA0895	2.51E-12	0.1911674	0.086	0.049	6.05E-08	2.1	KIAA0895	1.755102041
TMEM106A	2.78E-12	0.2156358	0.092	0.055	6.71E-08	2.1	TMEM106/	1.672727273
RAB31	3.24E-12	0.3321443	0.241	0.187	7.80E-08	2.1	RAB31	1.288770053
ELOVL2	3.54E-12	0.1039726	0.026	0.009	8.54E-08	2.1	ELOVL2	2.888888889
RP11-384F7.2	3.75E-12	0.2015985	0.1	0.06	9.04E-08	2.1	RP11-384F	1.666666667
DHRSX	4.13E-12	0.3771466	0.322	0.274	9.96E-08	2.1	DHRSX	1.175182482
ZNF385B	4.72E-12	0.2500352	0.041	0.018	1.14E-07	2.1	ZNF385B	2.277777778
OS9	4.97E-12	0.2431703	0.136	0.091	1.20E-07	2.1	OS9	1.494505495
GSTCD	5.18E-12	0.2892443	0.154	0.106	1.25E-07	2.1	GSTCD	1.452830189
GPR107	5.70E-12	0.3164089	0.199	0.151	1.37E-07	2.1	GPR107	1.317880795
CUL4A	5.80E-12	0.3144896	0.227	0.177	1.40E-07	2.1	CUL4A	1.282485876
CLK11	6.76E-12	0.3005739	0.452	0.415	1.63E-07	2.1	CLK1	1.089156627
LAMA1	6.87E-12	0.5874029	0.148	0.101	1.66E-07	2.1	LAMA1	1.465346535
RP11-131L23.1	6.91E-12	0.3436921	0.143	0.097	1.67E-07	2.1	RP11-131L	1.474226804
OST4	7.11E-12	0.3147182	0.442	0.414	1.71E-07	2.1	OST4	1.06763285
BRIP1	7.79E-12	0.2264049	0.075	0.042	1.88E-07	2.1	BRIP1	1.785714286
MAP3K8	8.09E-12	0.3206042	0.348	0.297	1.95E-07	2.1	MAP3K8	1.171717172
PEBP1	8.60E-12	0.2752137	0.219	0.17	2.07E-07	2.1	PEBP1	1.288235294
BAG1	1.05E-11	0.2510625	0.141	0.096	2.53E-07	2.1	BAG1	1.46875
AEBP2	1.06E-11	0.3955972	0.301	0.255	2.55E-07	2.1	AEBP2	1.180392157
DGKI	1.11E-11	0.2134667	0.063	0.034	2.67E-07	2.1	DGKI	1.852941176
KCNAB1	1.17E-11	0.109827	0.034	0.014	2.81E-07	2.1	KCNAB1	2.428571429
BCOR	1.22E-11	0.1994042	0.582	0.507	2.94E-07	2.1	BCOR	1.147928994
ZBTB431	1.29E-11	0.3066654	0.226	0.176	3.12E-07	2.1	ZBTB43	1.284090909
UCHL3	1.43E-11	0.3046176	0.19	0.142	3.45E-07	2.1	UCHL3	1.338028169
DACH1	1.54E-11	0.2630181	0.139	0.094	3.72E-07	2.1	DACH1	1.478723404
SLK	1.62E-11	0.3004042	0.283	0.232	3.92E-07	2.1	SLK	1.219827586
TNFRSF19	1.84E-11	0.1711966	0.058	0.03	4.43E-07	2.1	TNFRSF19	1.933333333
IARS1	1.95E-11	0.3094159	0.179	0.131	4.70E-07	2.1	IARS	1.366412214
SLC17A5	1.97E-11	0.2549756	0.137	0.094	4.75E-07	2.1	SLC17A5	1.457446809
DERA	2.12E-11	0.2348274	0.105	0.067	5.11E-07	2.1	DERA	1.567164179
ITPR1	2.13E-11	0.2526327	0.144	0.099	5.13E-07	2.1	ITPR1	1.454545455
TMED7-TICAM2	2.42E-11	0.1757197	0.081	0.047	5.83E-07	2.1	TMED7-TIC	1.723404255
TNKS1	2.78E-11	0.3414385	0.327	0.283	6.70E-07	2.1	TNKS	1.155477032
RAI2	3.00E-11	0.1455959	0.048	0.023	7.23E-07	2.1	RAI2	2.086956522
TTC33	3.07E-11	0.223195	0.107	0.069	7.41E-07	2.1	TTC33	1.550724638

MSMO1	3.15E-11	0.2613163	0.383	0.33	7.59E-07	2.1	MSMO1	1.160606061
COG3	3.36E-11	0.2442323	0.108	0.069	8.10E-07	2.1	COG3	1.565217391
MTUS11	3.46E-11	0.3452457	0.412	0.376	8.35E-07	2.1	MTUS1	1.095744681
CAB39	3.55E-11	0.2856275	0.404	0.359	8.57E-07	2.1	CAB39	1.125348189
CHEK1	3.57E-11	0.1203361	0.029	0.011	8.62E-07	2.1	CHEK1	2.636363636
RP11-302B13.5	3.79E-11	0.1150627	0.038	0.017	9.15E-07	2.1	RP11-302B	2.235294118
RP11-166B2.8	3.80E-11	0.2286954	0.097	0.061	9.15E-07	2.1	RP11-166B	1.590163934
PGRMC2	3.98E-11	0.2655455	0.172	0.126	9.59E-07	2.1	PGRMC2	1.365079365
NEB	3.99E-11	0.1901277	0.076	0.044	9.63E-07	2.1	NEB	1.727272727
BATF	4.30E-11	0.2266486	0.111	0.071	1.04E-06	2.1	BATF	1.563380282
P4HA3	4.45E-11	0.1592355	0.062	0.033	1.07E-06	2.1	P4HA3	1.878787879
SNX1	4.52E-11	0.3017571	0.206	0.159	1.09E-06	2.1	SNX1	1.295597484
SPTAN1	4.56E-11	0.2869991	0.267	0.219	1.10E-06	2.1	SPTAN1	1.219178082
GRB10	4.91E-11	0.3588433	0.227	0.179	1.18E-06	2.1	GRB10	1.268156425
RNF1691	5.10E-11	0.3353629	0.305	0.261	1.23E-06	2.1	RNF169	1.168582375
ATP13A4	5.64E-11	0.1316818	0.061	0.032	1.36E-06	2.1	ATP13A4	1.90625
GLCCI1	5.92E-11	0.1575183	0.364	0.297	1.43E-06	2.1	GLCCI1	1.225589226
PKD1L3	6.65E-11	0.1443952	0.059	0.032	1.60E-06	2.1	PKD1L3	1.84375
PMEPA1	6.73E-11	0.191232	0.094	0.058	1.62E-06	2.1	PMEPA1	1.620689655
NCOR1	7.19E-11	0.3000607	0.447	0.422	1.73E-06	2.1	NCOR1	1.059241706
FAM208B	7.38E-11	0.3140124	0.279	0.233	1.78E-06	2.1	FAM208B	1.197424893
ATP6V1C1	8.26E-11	0.3250534	0.174	0.13	1.99E-06	2.1	ATP6V1C1	1.338461538
PABPC1L	8.35E-11	0.1220357	0.037	0.017	2.01E-06	2.1	PABPC1L	2.176470588
VEGFA	8.39E-11	0.3286002	0.277	0.226	2.02E-06	2.1	VEGFA	1.225663717
RHOV	8.69E-11	0.2322523	0.078	0.047	2.09E-06	2.1	RHOV	1.659574468
PCM1	9.48E-11	0.3362723	0.299	0.256	2.29E-06	2.1	PCM1	1.16796875
KDM5B1	1.01E-10	0.2338847	0.563	0.544	2.43E-06	2.1	KDM5B	1.034926471
RP11-12M5.4	1.04E-10	0.1245133	0.036	0.016	2.52E-06	2.1	RP11-12M5	2.25
THSD4	1.06E-10	0.2216568	0.602	0.56	2.56E-06	2.1	THSD4	1.075
FNDC3A	1.08E-10	0.3182881	0.403	0.368	2.61E-06	2.1	FNDC3A	1.095108696
COQ3	1.08E-10	0.1323532	0.045	0.022	2.61E-06	2.1	COQ3	2.045454545
CTC-490E21.12	1.10E-10	0.2416094	0.089	0.055	2.64E-06	2.1	CTC-490E2	1.618181818
ADAMTS4	1.15E-10	0.1737308	0.082	0.049	2.77E-06	2.1	ADAMTS4	1.673469388
RP11-295K2.3	1.18E-10	0.1034364	0.034	0.015	2.84E-06	2.1	RP11-295K	2.266666667
USF3	1.19E-10	0.2411863	0.103	0.066	2.87E-06	2.1	USF3	1.560606061
ENOX1	1.19E-10	0.3522229	0.184	0.136	2.87E-06	2.1	ENOX1	1.352941176
YAP1	1.22E-10	0.2722605	0.545	0.535	2.95E-06	2.1	YAP1	1.018691589
RNF4	1.27E-10	0.2763314	0.211	0.164	3.06E-06	2.1	RNF4	1.286585366
ZNF428	1.28E-10	0.1457934	0.039	0.018	3.08E-06	2.1	ZNF428	2.166666667
VAV3	1.49E-10	0.2419425	0.337	0.283	3.60E-06	2.1	VAV3	1.190812721
PNP	1.60E-10	0.2487617	0.117	0.078	3.87E-06	2.1	PNP	1.5
ITGA10	1.73E-10	0.1785051	0.085	0.051	4.17E-06	2.1	ITGA10	1.666666667
MYB	1.75E-10	0.1163358	0.043	0.021	4.21E-06	2.1	MYB	2.047619048
EPT1	1.82E-10	0.2102163	0.135	0.093	4.38E-06	2.1	EPT1	1.451612903
SIAH2	1.84E-10	0.1807693	0.113	0.075	4.44E-06	2.1	SIAH2	1.506666667
SAP130	1.91E-10	0.2300864	0.123	0.084	4.61E-06	2.1	SAP130	1.464285714
AC073218.1	1.95E-10	0.2461118	0.127	0.086	4.70E-06	2.1	AC073218.	1.476744186
DCP1A	1.96E-10	0.315926	0.3	0.261	4.74E-06	2.1	DCP1A	1.149425287
CHAF1B1	2.04E-10	0.180274	0.061	0.033	4.92E-06	2.1	CHAF1B	1.848484848
TMEM254-AS1	2.12E-10	0.1257067	0.045	0.022	5.10E-06	2.1	TMEM254-	2.045454545
ANKFY1	2.23E-10	0.2491138	0.115	0.078	5.38E-06	2.1	ANKFY1	1.474358974
OGT1	2.75E-10	0.2919968	0.431	0.402	6.64E-06	2.1	OGT	1.072139303

CA21	2.77E-10	0.2720557	0.13	0.089	6.68E-06	2.1	CA2	1.460674157
LINC00862	2.79E-10	0.1799245	0.067	0.038	6.73E-06	2.1	LINC00862	1.763157895
F3	3.48E-10	0.2239629	0.152	0.108	8.38E-06	2.1	F3	1.407407407
GNB1	3.54E-10	0.2784963	0.38	0.343	8.52E-06	2.1	GNB1	1.10787172
EDEM3	3.56E-10	0.248612	0.125	0.086	8.59E-06	2.1	EDEM3	1.453488372
CDC73	3.85E-10	0.312059	0.229	0.184	9.28E-06	2.1	CDC73	1.244565217
WHSC1L1	3.97E-10	0.3006333	0.301	0.258	9.57E-06	2.1	WHSC1L1	1.166666667
VAPA1	4.24E-10	0.2703056	0.488	0.474	1.02E-05	2.1	VAPA	1.029535865
MAPK6	4.41E-10	0.2808767	0.315	0.27	1.06E-05	2.1	MAPK6	1.166666667
PMP22	4.60E-10	0.1378213	0.044	0.021	1.11E-05	2.1	PMP22	2.095238095
UQCRB1	5.07E-10	0.1667718	0.796	0.807	1.22E-05	2.1	UQCRB	0.986369269
ZCCHC61	5.34E-10	0.2970631	0.373	0.338	1.29E-05	2.1	ZCCHC6	1.103550296
UVRAG1	5.40E-10	0.3059946	0.485	0.465	1.30E-05	2.1	UVRAG	1.043010753
RALY	5.48E-10	0.2728702	0.223	0.178	1.32E-05	2.1	RALY	1.252808989
HNRNPA0	5.51E-10	0.2472719	0.134	0.095	1.33E-05	2.1	HNRNPA0	1.410526316
LIN52	5.80E-10	0.2243562	0.095	0.061	1.40E-05	2.1	LIN52	1.557377049
ZFAND5	5.80E-10	0.2673074	0.464	0.433	1.40E-05	2.1	ZFAND5	1.071593533
PHEX	5.83E-10	0.1436829	0.042	0.02	1.41E-05	2.1	PHEX	2.1
RP11-371F15.3	6.17E-10	0.2400493	0.122	0.083	1.49E-05	2.1	RP11-371F1	1.469879518
MED15	6.63E-10	0.2969875	0.213	0.169	1.60E-05	2.1	MED15	1.26035503
RSPRY1	7.67E-10	0.2301535	0.15	0.108	1.85E-05	2.1	RSPRY1	1.388888889
MAL2	7.77E-10	0.2334807	0.398	0.351	1.87E-05	2.1	MAL2	1.133903134
TTLL5	8.33E-10	0.3322667	0.323	0.281	2.01E-05	2.1	TTLL5	1.149466192
MYO10	8.63E-10	0.3311147	0.294	0.251	2.08E-05	2.1	MYO10	1.171314741
DDR1	8.78E-10	0.3123066	0.258	0.213	2.12E-05	2.1	DDR1	1.211267606
MAP7	8.91E-10	0.3442404	0.39	0.358	2.15E-05	2.1	MAP7	1.089385475
BCL3	9.01E-10	0.2250023	0.141	0.1	2.17E-05	2.1	BCL3	1.41
DOCK111	9.25E-10	0.1372532	0.048	0.025	2.23E-05	2.1	DOCK11	1.92
TNIP1	1.01E-09	0.257001	0.255	0.209	2.44E-05	2.1	TNIP1	1.220095694
RP11-644C3.1	1.07E-09	0.2242722	0.07	0.041	2.59E-05	2.1	RP11-644C	1.707317073
SDCBP2-AS1	1.07E-09	0.2447156	0.097	0.063	2.59E-05	2.1	SDCBP2-AS	1.53968254
RCL1	1.08E-09	0.1880917	0.103	0.067	2.60E-05	2.1	RCL1	1.537313433
AC018816.3	1.19E-09	0.2380528	0.137	0.096	2.88E-05	2.1	AC018816.	1.427083333
CCNI	1.28E-09	0.2249959	0.636	0.628	3.08E-05	2.1	CCNI	1.012738854
USP152	1.28E-09	0.3336971	0.363	0.33	3.09E-05	2.1	USP15	1.1
MYOM1	1.29E-09	0.264614	0.077	0.047	3.11E-05	2.1	MYOM1	1.638297872
PLAT	1.30E-09	0.249493	0.124	0.085	3.14E-05	2.1	PLAT	1.458823529
TWSG1	1.31E-09	0.1785388	0.117	0.079	3.17E-05	2.1	TWSG1	1.481012658
ITPR3	1.40E-09	0.2010347	0.112	0.076	3.36E-05	2.1	ITPR3	1.473684211
C14orf37	1.42E-09	0.2785859	0.142	0.103	3.43E-05	2.1	C14orf37	1.378640777
NUP210L	1.58E-09	0.1465844	0.056	0.031	3.82E-05	2.1	NUP210L	1.806451613
MAP2K4	1.60E-09	0.2244585	0.48	0.441	3.85E-05	2.1	MAP2K4	1.088435374
ATF4	1.61E-09	0.3005081	0.425	0.395	3.89E-05	2.1	ATF4	1.075949367
PXN	1.75E-09	0.2129654	0.131	0.092	4.22E-05	2.1	PXN	1.423913043
AGAP3	1.76E-09	0.1804981	0.086	0.054	4.25E-05	2.1	AGAP3	1.592592593
TNC	1.96E-09	0.2384192	0.388	0.328	4.72E-05	2.1	TNC	1.182926829
CCDC181	1.99E-09	0.1110049	0.033	0.015	4.80E-05	2.1	CCDC181	2.2
RCAN32	2.33E-09	0.2219747	0.132	0.197	5.62E-05	2.1	RCAN3	0.670050761
PHF20	2.35E-09	0.2924545	0.384	0.355	5.67E-05	2.1	PHF20	1.081690141
IKBKB	2.37E-09	0.3074374	0.205	0.163	5.70E-05	2.1	IKBKB	1.257668712
DCAF5	2.45E-09	0.2959775	0.185	0.143	5.91E-05	2.1	DCAF5	1.293706294
PFKFB41	2.63E-09	0.1355885	0.065	0.037	6.34E-05	2.1	PFKFB4	1.756756757

TIMM17A2	2.64E-09	0.3467193	0.256	0.218	6.36E-05	2.1	TIMM17A	1.174311927
CDC14A	2.86E-09	0.270527	0.203	0.158	6.90E-05	2.1	CDC14A	1.284810127
C9orf24	3.18E-09	0.1250214	0.037	0.018	7.68E-05	2.1	C9orf24	2.055555556
ABCC1	3.19E-09	0.2478615	0.14	0.101	7.69E-05	2.1	ABCC1	1.386138614
ATG101	3.32E-09	0.1154696	0.054	0.03	8.01E-05	2.1	ATG101	1.8
AKAP17A	3.42E-09	0.1375323	0.064	0.037	8.25E-05	2.1	AKAP17A	1.72972973
RAB32	3.64E-09	0.1967134	0.13	0.092	8.79E-05	2.1	RAB32	1.413043478
SLC9A71	3.70E-09	0.2704732	0.321	0.28	8.93E-05	2.1	SLC9A7	1.146428571
JAK1	3.93E-09	0.2667986	0.437	0.406	9.47E-05	2.1	JAK1	1.07635468
HCG171	3.98E-09	0.2337648	0.095	0.063	9.59E-05	2.1	HCG17	1.507936508
ASH1L	4.06E-09	0.2641393	0.562	0.551	9.79E-05	2.1	ASH1L	1.019963702
COX6B1	4.08E-09	0.186404	0.739	0.763	9.84E-05	2.1	COX6B1	0.968545216
OSTC2	4.15E-09	0.2968441	0.471	0.458	0.00010018	2.1	OSTC	1.028384279
MCF2	4.32E-09	0.1303471	0.045	0.023	0.000104225	2.1	MCF2	1.956521739
SH3KBP1	4.33E-09	0.2097604	0.142	0.101	0.000104398	2.1	SH3KBP1	1.405940594
PTGER3	4.87E-09	0.2034204	0.065	0.038	0.000117534	2.1	PTGER3	1.710526316
CASZ1	4.99E-09	0.2215271	0.133	0.095	0.000120262	2.1	CASZ1	1.4
SNIP11	5.21E-09	0.1522057	0.073	0.044	0.00012571	2.1	SNIP1	1.659090909
RP11-37N22.1	5.57E-09	0.1500211	0.058	0.033	0.000134203	2.1	RP11-37N2	1.757575758
SP3	5.59E-09	0.2801563	0.253	0.211	0.000134831	2.1	SP3	1.199052133
RP11-347P5.1	6.46E-09	0.1851989	0.072	0.044	0.000155826	2.1	RP11-347P.	1.636363636
NFXL11	6.72E-09	0.2200757	0.089	0.058	0.00016214	2.1	NFXL1	1.534482759
AC104667.3	6.79E-09	0.1221624	0.031	0.014	0.000163673	2.1	AC104667.	2.214285714
SAFB	6.79E-09	0.203733	0.108	0.073	0.00016383	2.1	SAFB	1.479452055
RP11-813I20.2	6.96E-09	0.1275529	0.043	0.022	0.000167868	2.1	RP11-813I2	1.954545455
NBAS	7.13E-09	0.3278311	0.187	0.147	0.000171958	2.1	NBAS	1.272108844
AVIL1	7.38E-09	0.2178821	0.09	0.059	0.000177873	2.1	AVIL	1.525423729
PSD3	7.90E-09	0.4268631	0.375	0.345	0.000190528	2.1	PSD3	1.086956522
RND31	8.61E-09	0.2755602	0.386	0.349	0.000207587	2.1	RND3	1.106017192
UQCC2	8.76E-09	0.1496374	0.082	0.052	0.000211279	2.1	UQCC2	1.576923077
UBL3	8.84E-09	0.2804545	0.242	0.2	0.000213263	2.1	UBL3	1.21
GGNBP2	9.42E-09	0.2892841	0.265	0.227	0.000227173	2.1	GGNBP2	1.167400881
MEX3D	1.03E-08	0.1275593	0.057	0.033	0.000248599	2.1	MEX3D	1.727272727
ARID4A1	1.06E-08	0.2386162	0.228	0.185	0.000254648	2.1	ARID4A	1.232432432
SRGAP1	1.09E-08	0.3835696	0.391	0.365	0.000263457	2.1	SRGAP1	1.071232877
TANC1	1.10E-08	0.3427713	0.375	0.348	0.000265355	2.1	TANC1	1.077586207
CANX	1.20E-08	0.2731731	0.37	0.337	0.000289077	2.1	CANX	1.097922849
GATAD2A	1.28E-08	0.3111232	0.19	0.15	0.000308859	2.1	GATAD2A	1.266666667
ALDH6A1	1.31E-08	0.1063418	0.05	0.027	0.000316155	2.1	ALDH6A1	1.851851852
FHL2	1.32E-08	0.2520941	0.212	0.17	0.000317585	2.1	FHL2	1.247058824
LINGO11	1.32E-08	0.1769684	0.889	0.89	0.00031765	2.1	LINGO1	0.998876404
MACC11	1.34E-08	0.2059981	0.449	0.411	0.000322619	2.1	MACC1	1.092457421
RAB2A	1.36E-08	0.2922244	0.343	0.311	0.000327448	2.1	RAB2A	1.102893891
ARHGAP30	1.42E-08	0.141532	0.048	0.026	0.000342439	2.1	ARHGAP30	1.846153846
CDK7	1.44E-08	0.2658513	0.154	0.117	0.00034651	2.1	CDK7	1.316239316
UBE2I	1.44E-08	0.2222606	0.16	0.121	0.000347873	2.1	UBE2I	1.32231405
MLIP	1.53E-08	0.114907	0.031	0.014	0.000368546	2.1	MLIP	2.214285714
ITM2B	1.60E-08	0.2648043	0.378	0.349	0.000384756	2.1	ITM2B	1.083094556
GABPB1	1.60E-08	0.2824183	0.153	0.116	0.000385021	2.1	GABPB1	1.318965517
CACNA2D1	1.70E-08	0.2466936	0.199	0.154	0.000408729	2.1	CACNA2D1	1.292207792
TBL1XR1	1.73E-08	0.1911988	0.597	0.582	0.000417931	2.1	TBL1XR1	1.025773196
FAM222A-AS1	1.93E-08	0.1426794	0.052	0.029	0.000465402	2.1	FAM222A-/	1.793103448

RRAGC	2.01E-08	0.182464	0.109	0.075	0.0004845	2.1	RRAGC	1.453333333
STRN3	2.03E-08	0.2827998	0.348	0.316	0.000488496	2.1	STRN3	1.101265823
TTC34	2.24E-08	0.3127992	0.336	0.303	0.000540476	2.1	TTC3	1.108910891
RP11-148B18.1	2.34E-08	0.2303119	0.072	0.045	0.000564603	2.1	RP11-148B	1.6
ADGRV1	2.42E-08	0.3387931	0.202	0.163	0.000583376	2.1	ADGRV1	1.239263804
CD109	2.52E-08	0.1327194	0.05	0.028	0.000608764	2.1	CD109	1.785714286
GDE1	2.55E-08	0.2268711	0.182	0.142	0.00061401	2.1	GDE1	1.281690141
RPS293	2.55E-08	0.1514752	0.9	0.927	0.000615405	2.1	RPS29	0.970873786
MBD21	2.84E-08	0.298849	0.338	0.306	0.000685627	2.1	MBD2	1.104575163
ASAH1	2.95E-08	0.2004198	0.136	0.099	0.000710837	2.1	ASAH1	1.373737374
IRS1	3.04E-08	0.169964	0.13	0.093	0.000732251	2.1	IRS1	1.397849462
MTF22	3.11E-08	0.2866848	0.247	0.209	0.000749901	2.1	MTF2	1.181818182
RYR2	3.17E-08	0.209902	0.155	0.116	0.000765145	2.1	RYR2	1.336206897
COX7C1	3.34E-08	0.1391318	0.856	0.875	0.000804792	2.1	COX7C	0.978285714
SHOC2	3.47E-08	0.2826419	0.293	0.258	0.000837371	2.1	SHOC2	1.135658915
NOTCH2	3.66E-08	0.2894547	0.275	0.237	0.000882912	2.1	NOTCH2	1.160337553
ARHGAP32	3.70E-08	0.2509042	0.443	0.415	0.000891794	2.1	ARHGAP32	1.06746988
PLSCR21	4.09E-08	0.2214952	0.086	0.057	0.000985835	2.1	PLSCR2	1.50877193
WSB1	4.18E-08	0.4241794	0.411	0.39	0.001007602	2.1	WSB1	1.053846154
GIGYF2	4.25E-08	0.282781	0.323	0.292	0.00102486	2.1	GIGYF2	1.106164384
BOD1L1	4.32E-08	0.2648125	0.176	0.14	0.001040878	2.1	BOD1L1	1.257142857
ACTB1	4.61E-08	0.2668724	0.653	0.643	0.001111535	2.1	ACTB	1.0155521
MTCL1	4.65E-08	0.1685425	0.081	0.053	0.001122276	2.1	MTCL1	1.528301887
IST1	4.73E-08	0.2582158	0.359	0.329	0.001141166	2.1	IST1	1.09118541
RIOK31	4.77E-08	0.2658339	0.303	0.266	0.001149247	2.1	RIOK3	1.139097744
UBAP11	4.86E-08	0.2608212	0.354	0.32	0.001171918	2.1	UBAP1	1.10625
ABHD3	4.92E-08	0.3840725	0.209	0.172	0.001186942	2.1	ABHD3	1.215116279
PIP5K1A1	5.01E-08	0.2680112	0.334	0.301	0.001207228	2.1	PIP5K1A	1.109634551
MCCC2	5.04E-08	0.2469396	0.194	0.156	0.001215535	2.1	MCCC2	1.243589744
MAN1A1	5.13E-08	0.2778612	0.266	0.226	0.001237722	2.1	MAN1A1	1.17699115
H2AFY	5.26E-08	0.2502449	0.184	0.146	0.001269337	2.1	H2AFY	1.260273973
LINC00504	5.34E-08	0.1540706	0.124	0.087	0.001288152	2.1	LINC00504	1.425287356
ACSL3	5.62E-08	0.2654279	0.369	0.335	0.001354431	2.1	ACSL3	1.101492537
PPFIA2	5.97E-08	0.1315734	0.061	0.036	0.001438694	2.1	PPFIA2	1.694444444
COCH	6.28E-08	0.2146603	0.156	0.12	0.001514754	2.1	COCH	1.3
HBP1	6.30E-08	0.2527047	0.296	0.26	0.001518222	2.1	HBP1	1.138461538
MYLIP	6.47E-08	0.1747443	0.125	0.09	0.001560291	2.1	MYLIP	1.388888889
GLRX5	6.83E-08	0.1449695	0.056	0.033	0.00164598	2.1	GLRX5	1.696969697
CXCL1	7.17E-08	0.3566616	0.307	0.268	0.001728127	2.1	CXCL1	1.145522388
GNA131	7.37E-08	0.2405932	0.246	0.206	0.001776711	2.1	GNA13	1.194174757
RP11-665G4.1	7.51E-08	0.2037534	0.121	0.087	0.001810171	2.1	RP11-665G	1.390804598
RP3-331H24.5	7.90E-08	0.1613976	0.053	0.031	0.001904559	2.1	RP3-331H2	1.709677419
TUFT11	7.91E-08	0.2806571	0.218	0.181	0.001906704	2.1	TUFT1	1.20441989
LRRC59	8.66E-08	0.168021	0.097	0.067	0.002088232	2.1	LRRC59	1.447761194
RP11-241G9.31	8.92E-08	0.1579879	0.066	0.041	0.00215177	2.1	RP11-241G	1.609756098
HERC31	8.98E-08	0.3316273	0.192	0.157	0.002165774	2.1	HERC3	1.222929936
ANXA5	9.07E-08	0.1972817	0.401	0.363	0.002186228	2.1	ANXA5	1.104683196
ZC3H12A	9.67E-08	0.1941916	0.096	0.066	0.002331763	2.1	ZC3H12A	1.454545455
VCPIP1	1.05E-07	0.1699193	0.083	0.055	0.002539938	2.1	VCPIP1	1.509090909
TMED9	1.10E-07	0.1959663	0.133	0.099	0.002658242	2.1	TMED9	1.343434343
MXI1	1.12E-07	0.2772264	0.227	0.19	0.002711324	2.1	MXI1	1.194736842
MAGT11	1.17E-07	0.2392735	0.215	0.177	0.002827078	2.1	MAGT1	1.214689266

NPC12	1.17E-07	0.2744958	0.347	0.316	0.002827505	2.1	NPC1	1.098101266
FAM234B	1.19E-07	0.1289542	0.072	0.046	0.002876929	2.1	FAM234B	1.565217391
FAM110C	1.20E-07	0.1244686	0.064	0.039	0.002891042	2.1	FAM110C	1.641025641
SLC10A1	1.21E-07	0.1631768	0.075	0.049	0.002906198	2.1	SLC10A1	1.530612245
FAM46A	1.21E-07	0.2818282	0.208	0.168	0.002906846	2.1	FAM46A	1.238095238
SPIN1	1.22E-07	0.2578377	0.25	0.214	0.002939907	2.1	SPIN1	1.168224299
BCAR3	1.27E-07	0.1662302	0.326	0.276	0.003058275	2.1	BCAR3	1.18115942
MITF	1.30E-07	0.1236632	0.054	0.032	0.003131057	2.1	MITF	1.6875
SFXN5	1.33E-07	0.1328308	0.047	0.027	0.003201982	2.1	SFXN5	1.740740741
MAOA	1.34E-07	0.1507417	0.085	0.056	0.003222105	2.1	MAOA	1.517857143
PPTC71	1.36E-07	0.252058	0.231	0.192	0.003273437	2.1	PPTC7	1.203125
DNAH3	1.43E-07	0.1346855	0.046	0.026	0.003456016	2.1	DNAH3	1.769230769
GFPT12	1.47E-07	0.2935117	0.226	0.19	0.003554463	2.1	GFPT1	1.189473684
RALA	1.50E-07	0.2669065	0.25	0.213	0.003622061	2.1	RALA	1.17370892
YARS3	1.56E-07	0.2516999	0.312	0.278	0.003766557	2.1	YARS	1.122302158
PPME1	1.61E-07	0.2082431	0.111	0.079	0.003889245	2.1	PPME1	1.405063291
MKRN1	1.72E-07	0.2346294	0.19	0.153	0.004158486	2.1	MKRN1	1.241830065
MOK1	1.82E-07	0.1398851	0.079	0.053	0.004392853	2.1	MOK	1.490566038
LINC00704	1.82E-07	0.103858	0.048	0.027	0.004395965	2.1	LINC00704	1.777777778
NPIPB5	1.85E-07	0.1859016	0.119	0.087	0.004462734	2.1	NPIPB5	1.367816092
IPPK	1.89E-07	0.1043828	0.036	0.019	0.004551419	2.1	IPPK	1.894736842
CYCS2	2.08E-07	0.2600818	0.534	0.52	0.005018851	2.1	CYCS	1.026923077
C2orf882	2.22E-07	0.2306162	0.374	0.348	0.005354026	2.1	C2orf88	1.074712644
ZFP36L21	2.23E-07	0.3649103	0.395	0.371	0.005370527	2.1	ZFP36L2	1.064690027
MPRIP-AS12	2.26E-07	0.1708818	0.086	0.058	0.00544581	2.1	MPRIP-AS1	1.482758621
FBXL20	2.29E-07	0.2877616	0.339	0.316	0.005528876	2.1	FBXL20	1.07278481
RP2	2.35E-07	0.1299993	0.053	0.031	0.005662684	2.1	RP2	1.709677419
BBOF1	2.36E-07	0.1690495	0.076	0.05	0.005694062	2.1	BBOF1	1.52
IL1RAPL2	2.37E-07	0.1795126	0.115	0.083	0.005713801	2.1	IL1RAPL2	1.385542169
FOXA1	2.45E-07	0.1925009	0.153	0.116	0.005911621	2.1	FOXA1	1.318965517
CTNNAL11	2.86E-07	0.2507731	0.161	0.126	0.006887869	2.1	CTNNAL1	1.277777778
PDIA5	2.86E-07	0.122162	0.055	0.033	0.006907662	2.1	PDIA5	1.666666667
RP11-486A14.2	2.88E-07	0.1055869	0.029	0.014	0.00695197	2.1	RP11-486A	2.071428571
USP32	3.02E-07	0.2843259	0.264	0.23	0.007289214	2.1	USP32	1.147826087
EXOC6B	3.03E-07	0.3182766	0.304	0.271	0.007304145	2.1	EXOC6B	1.121771218
HDAC3	3.04E-07	0.1802629	0.088	0.06	0.007338148	2.1	HDAC3	1.466666667
TNKS2	3.10E-07	0.2573261	0.289	0.259	0.007481997	2.1	TNKS2	1.115830116
CYLD3	3.12E-07	0.1941723	0.25	0.21	0.007520451	2.1	CYLD	1.19047619
ETNK1	3.17E-07	0.2567178	0.232	0.198	0.007637717	2.1	ETNK1	1.171717172
ZSWIM8	3.35E-07	0.1659944	0.09	0.062	0.008089227	2.1	ZSWIM8	1.451612903
HSPA1A	3.55E-07	0.3146401	0.107	0.077	0.00856616	2.1	HSPA1A	1.38961039
RP11-703M24.5	3.57E-07	0.173454	0.085	0.058	0.008603078	2.1	RP11-703M	1.465517241
RP11-277P12.202	3.61E-07	0.2082097	0.096	0.067	0.00870934	2.1	RP11-277P	1.432835821
SEC24A2	3.69E-07	0.264459	0.209	0.173	0.008897785	2.1	SEC24A	1.208092486
LACTB	3.71E-07	0.1509557	0.089	0.061	0.008954401	2.1	LACTB	1.459016393
RALGAPA1	3.81E-07	0.2559611	0.335	0.304	0.009177633	2.1	RALGAPA1	1.101973684
EIF2AK1	3.82E-07	0.5038823	0.144	0.111	0.009203449	2.1	EIF2AK1	1.297297297
NAP1L4	3.88E-07	0.2462726	0.235	0.201	0.009354101	2.1	NAP1L4	1.169154229
RAB1A1	4.09E-07	0.2560972	0.461	0.456	0.009854113	2.1	RAB1A	1.010964912
POU2F1	4.47E-07	0.2616815	0.199	0.164	0.01076972	2.1	POU2F1	1.213414634
PPP1R15A	4.59E-07	0.1243057	0.051	0.03	0.01105977	2.1	PPP1R15A	1.7
USP22	4.67E-07	0.2023852	0.154	0.119	0.01126653	2.1	USP22	1.294117647

ADAM92	4.69E-07	0.1622668	0.578	0.568	0.01129942	2.1	ADAM9	1.017605634
GRB2	4.79E-07	0.2066007	0.195	0.16	0.01155593	2.1	GRB2	1.21875
TPRG1	4.87E-07	0.2659818	0.15	0.116	0.01174516	2.1	TPRG1	1.293103448
WDR19	4.98E-07	0.2317302	0.133	0.101	0.01200185	2.1	WDR19	1.316831683
SRP14	5.03E-07	0.111938	0.86	0.876	0.01213442	2.1	SRP14	0.98173516
MYO5A	5.05E-07	0.2621702	0.137	0.105	0.01218527	2.1	MYO5A	1.304761905
ASB7	5.40E-07	0.1394222	0.056	0.035	0.01301474	2.1	ASB7	1.6
PRKAR2B	5.50E-07	0.1818053	0.079	0.053	0.0132654	2.1	PRKAR2B	1.490566038
NAA35	5.51E-07	0.248164	0.166	0.133	0.01328671	2.1	NAA35	1.248120301
AF127577.11	5.62E-07	0.150715	0.069	0.045	0.01355901	2.1	AF127577.1	1.533333333
RP11-517O13.1	5.90E-07	0.1213008	0.044	0.025	0.01422116	2.1	RP11-517O	1.76
ITGB6	6.11E-07	0.2407812	0.412	0.382	0.01472668	2.1	ITGB6	1.078534031
MAPK10	6.19E-07	0.1397889	0.068	0.044	0.0149216	2.1	MAPK10	1.545454545
JADE3	6.34E-07	0.1738516	0.081	0.055	0.01529758	2.1	JADE3	1.472727273
ADM1	6.52E-07	0.1026666	0.041	0.023	0.01573165	2.1	ADM	1.782608696
CTB-12O2.1	6.55E-07	0.181731	0.038	0.021	0.01580342	2.1	CTB-12O2.1	1.80952381
AC004893.11	6.77E-07	0.1243973	0.064	0.04	0.01632352	2.1	AC004893.	1.6
MEX3C	6.94E-07	0.1485751	0.093	0.065	0.01674324	2.1	MEX3C	1.430769231
LRCH3	7.23E-07	0.2943475	0.367	0.346	0.01742651	2.1	LRCH3	1.060693642
TRRAP	7.77E-07	0.1837594	0.087	0.061	0.01874611	2.1	TRRAP	1.426229508
NUCB2	8.39E-07	0.2169686	0.161	0.127	0.02021887	2.1	NUCB2	1.267716535
RP11-507B12.1	8.53E-07	0.1007212	0.047	0.027	0.02057235	2.1	RP11-507B	1.740740741
DLGAP1	8.63E-07	0.4100021	0.239	0.202	0.02081718	2.1	DLGAP1	1.183168317
PDHX	8.97E-07	0.1772902	0.103	0.074	0.02162459	2.1	PDHX	1.391891892
PI4KB	9.01E-07	0.1779845	0.083	0.057	0.02173437	2.1	PI4KB	1.456140351
STXBP4	9.64E-07	0.1622246	0.067	0.044	0.02324237	2.1	STXBP4	1.522727273
TMCC3	9.99E-07	0.1393004	0.059	0.037	0.02409292	2.1	TMCC3	1.594594595
TMEM185A	9.99E-07	0.1270437	0.067	0.044	0.02409373	2.1	TMEM185A	1.522727273
GDPD4	1.01E-06	0.2131384	0.023	0.01	0.0243298	2.1	GDPD4	2.3
REEP5	1.08E-06	0.1833771	0.288	0.251	0.02612247	2.1	REEP5	1.147410359
IL12RB2	1.10E-06	0.1000589	0.038	0.021	0.0264649	2.1	IL12RB2	1.80952381
PKM	1.11E-06	0.1943566	0.381	0.351	0.02665265	2.1	PKM	1.085470085
SIX4	1.13E-06	0.1749521	0.107	0.078	0.02734483	2.1	SIX4	1.371794872
RBFOX1	1.15E-06	0.1664672	0.134	0.102	0.02784102	2.1	RBFOX1	1.31372549
PCF11	1.18E-06	0.2496824	0.155	0.123	0.02852427	2.1	PCF11	1.260162602
DYNLL11	1.20E-06	0.2477706	0.277	0.243	0.02900605	2.1	DYNLL1	1.139917695
COL12A1	1.22E-06	0.184259	0.073	0.048	0.02934636	2.1	COL12A1	1.520833333
GPR160	1.22E-06	0.1741541	0.124	0.093	0.02953767	2.1	GPR160	1.333333333
TFG1	1.23E-06	0.2407996	0.321	0.292	0.02975486	2.1	TFG	1.099315068
ZC3H7A2	1.27E-06	0.1645457	0.206	0.17	0.03050723	2.1	ZC3H7A	1.211764706
BCL101	1.27E-06	0.1929111	0.176	0.143	0.03057213	2.1	BCL10	1.230769231
IL13RA1	1.32E-06	0.2000781	0.155	0.122	0.03174193	2.1	IL13RA1	1.270491803
BAALC-AS1	1.41E-06	0.1285604	0.046	0.027	0.03408737	2.1	BAALC-AS1	1.703703704
TM9SF31	1.45E-06	0.2497825	0.336	0.31	0.03488973	2.1	TM9SF3	1.083870968
CCNL1	1.50E-06	0.2501964	0.424	0.412	0.03615701	2.1	CCNL1	1.029126214
RAB7A	1.54E-06	0.2098507	0.464	0.452	0.03715003	2.1	RAB7A	1.026548673
SC5D	1.57E-06	0.1665914	0.22	0.182	0.03776965	2.1	SC5D	1.208791209
IMPAD1	1.72E-06	0.1292444	0.09	0.063	0.04150599	2.1	IMPAD1	1.428571429
ELP2	1.72E-06	0.2656922	0.244	0.213	0.04153035	2.1	ELP2	1.145539906
FLT3	1.75E-06	0.1286134	0.056	0.035	0.04229041	2.1	FLT3	1.6
ATOX1	1.89E-06	0.1568113	0.12	0.09	0.04563787	2.1	ATOX1	1.333333333
RPH3A	1.92E-06	0.1211881	0.066	0.043	0.0463679	2.1	RPH3A	1.534883721

ABCA12	2.00E-06	0.115851	0.039	0.022	0.04818959	2.1	ABCA12	1.772727273
LMAN13	2.01E-06	0.2638901	0.213	0.181	0.04849685	2.1	LMAN1	1.17679558
TRAPPC8	2.02E-06	0.1915793	0.143	0.111	0.04866317	2.1	TRAPPC8	1.288288288
ANKRD30A1	0	2.202454	0.962	0.441	0	2.2	ANKRD30A	2.181405896
MYBPC11	0	2.07511	0.691	0.204	0	2.2	MYBPC1	3.387254902
GSTM31	0	2.058135	0.779	0.3	0	2.2	GSTM3	2.596666667
ELOVL51	0	1.928783	0.765	0.266	0	2.2	ELOVL5	2.87593985
XBP1	0	1.843671	0.776	0.367	0	2.2	XBP1	2.114441417
TNFSF11	0	1.337634	0.304	0.047	0	2.2	TNFSF11	6.468085106
C8orf41	1.10E-305	1.725306	0.897	0.496	2.66E-301	2.2	C8orf4	1.808467742
CXCL131	1.14E-298	1.880267	0.818	0.386	2.75E-294	2.2	CXCL13	2.119170984
AFF31	9.40E-296	1.905245	0.74	0.314	2.27E-291	2.2	AFF3	2.356687898
AGR21	9.75E-295	1.462608	0.635	0.219	2.35E-290	2.2	AGR2	2.899543379
EFHD11	2.06E-282	1.401908	0.682	0.276	4.97E-278	2.2	EFHD1	2.471014493
HEPACAM21	4.80E-278	0.9158496	0.237	0.03	1.16E-273	2.2	HEPACAM2	7.9
DIO21	6.16E-274	1.983679	0.59	0.201	1.48E-269	2.2	DIO2	2.935323383
RP11-507B12.21	3.87E-260	1.407982	0.374	0.085	9.34E-256	2.2	RP11-507B	4.4
DNAJC121	8.91E-255	1.356759	0.772	0.367	2.15E-250	2.2	DNAJC12	2.103542234
COX6C1	6.51E-243	1.379199	0.915	0.753	1.57E-238	2.2	COX6C	1.215139442
SH3BGRL1	1.45E-241	1.366227	0.727	0.382	3.49E-237	2.2	SH3BGRL	1.903141361
RP11-384F7.21	1.06E-217	1.270707	0.267	0.05	2.55E-213	2.2	RP11-384F	5.34
AGR31	3.48E-215	1.340566	0.54	0.197	8.38E-211	2.2	AGR3	2.741116751
APELA1	5.64E-212	0.641068	0.136	0.012	1.36E-207	2.2	APELA	11.33333333
PTHLH1	3.55E-206	1.474658	0.546	0.217	8.55E-202	2.2	PTHLH	2.516129032
AREG1	1.83E-205	1.038275	0.807	0.429	4.41E-201	2.2	AREG	1.881118881
IGFBP41	1.12E-183	1.101682	0.397	0.124	2.71E-179	2.2	IGFBP4	3.201612903
TMA71	2.19E-181	1.172431	0.779	0.591	5.27E-177	2.2	TMA7	1.318104907
CADPS21	3.82E-175	2.202855	0.655	0.389	9.20E-171	2.2	CADPS2	1.683804627
ERBB41	9.63E-174	1.004542	0.833	0.472	2.32E-169	2.2	ERBB4	1.764830508
UGDH1	2.96E-172	1.268066	0.527	0.219	7.15E-168	2.2	UGDH	2.406392694
FXYD31	1.40E-159	1.072436	0.833	0.621	3.38E-155	2.2	FXYD3	1.341384863
LPAR31	4.43E-159	0.74112	0.185	0.032	1.07E-154	2.2	LPAR3	5.78125
F31	2.69E-157	1.127204	0.329	0.098	6.49E-153	2.2	F3	3.357142857
FAM105A1	3.45E-156	0.6011882	0.141	0.019	8.31E-152	2.2	FAM105A	7.421052632
SLC39A61	1.90E-153	1.064908	0.58	0.286	4.58E-149	2.2	SLC39A6	2.027972028
HSPH11	2.04E-147	1.400543	0.542	0.269	4.93E-143	2.2	HSPH1	2.014869888
RP11-507B12.11	8.26E-146	0.689311	0.145	0.021	1.99E-141	2.2	RP11-507B	6.904761905
SDC21	4.83E-138	0.8923888	0.26	0.068	1.16E-133	2.2	SDC2	3.823529412
NEDD4L2	4.53E-137	1.169576	0.778	0.588	1.09E-132	2.2	NEDD4L	1.323129252
TNIK1	1.01E-136	1.105084	0.39	0.147	2.45E-132	2.2	TNIK	2.653061224
NDUFB11	2.74E-136	1.276058	0.587	0.344	6.61E-132	2.2	NDUFB1	1.706395349
DBI3	2.08E-130	0.9245912	0.822	0.643	5.02E-126	2.2	DBI	1.278382582
PRDX31	4.15E-130	0.9453925	0.463	0.211	1.00E-125	2.2	PRDX3	2.194312796
ATP5E1	2.54E-129	1.132719	0.723	0.546	6.13E-125	2.2	ATP5E	1.324175824
PRLR1	1.06E-128	1.053263	0.481	0.222	2.55E-124	2.2	PRLR	2.166666667
DIO2-AS11	1.70E-128	0.9571079	0.259	0.073	4.09E-124	2.2	DIO2-AS1	3.547945205
APOL4	1.20E-127	0.3890655	0.097	0.01	2.88E-123	2.2	APOL4	9.7
SCP21	5.50E-122	0.9643454	0.562	0.31	1.33E-117	2.2	SCP2	1.812903226
UQCRB2	2.61E-121	0.7939216	0.879	0.801	6.29E-117	2.2	UQCRB	1.097378277
ACSL31	3.87E-116	1.097075	0.567	0.323	9.33E-112	2.2	ACSL3	1.755417957
HPX1	5.65E-116	1.080545	0.454	0.201	1.36E-111	2.2	HPX	2.258706468
NDUFA5	1.05E-114	0.9462276	0.537	0.297	2.53E-110	2.2	NDUFA5	1.808080808

UQCR10	4.01E-114	1.019336	0.68	0.498	9.67E-110	2.2	UQCR10	1.365461847
GPBP12	7.84E-114	0.9680366	0.8	0.682	1.89E-109	2.2	GPBP1	1.173020528
PGR1	1.93E-112	0.8568612	0.246	0.072	4.65E-108	2.2	PGR	3.416666667
LINC005041	2.71E-108	0.8256343	0.256	0.08	6.54E-104	2.2	LINC00504	3.2
COX6B11	5.09E-107	0.7496011	0.846	0.755	1.23E-102	2.2	COX6B1	1.120529801
ATP5G23	7.38E-107	0.7376931	0.843	0.732	1.78E-102	2.2	ATP5G2	1.151639344
HMGN51	2.21E-106	0.7016368	0.214	0.059	5.32E-102	2.2	HMGN5	3.627118644
LRRC11	2.53E-106	0.9870996	0.339	0.134	6.09E-102	2.2	LRRC1	2.529850746
RP11-53O19.1	4.58E-106	0.443126	0.093	0.012	1.11E-101	2.2	RP11-53O1	7.75
ATP13A41	6.19E-106	0.5877538	0.143	0.028	1.49E-101	2.2	ATP13A4	5.107142857
GSTK11	5.09E-105	0.8777858	0.331	0.129	1.23E-100	2.2	GSTK1	2.565891473
UBB2	5.82E-105	0.8573278	0.837	0.715	1.40E-100	2.2	UBB	1.170629371
SFMBT21	1.44E-104	0.9835795	0.371	0.157	3.47E-100	2.2	SFMBT2	2.363057325
BCL21	8.54E-104	1.049547	0.504	0.259	2.06E-99	2.2	BCL2	1.945945946
IL20RA	1.63E-103	0.7309293	0.184	0.046	3.93E-99	2.2	IL20RA	4
COX7C2	6.55E-103	0.6697906	0.908	0.871	1.58E-98	2.2	COX7C	1.042479908
BHLHE401	7.87E-101	0.9152173	0.392	0.175	1.90E-96	2.2	BHLHE40	2.24
Sep-41	3.29E-100	0.3660522	0.097	0.014	7.92E-96	2.2	Sep-04	6.928571429
ENPP11	4.79E-100	0.8278489	0.321	0.123	1.15E-95	2.2	ENPP1	2.609756098
ATP5I1	8.61E-99	0.9021504	0.326	0.133	2.08E-94	2.2	ATP5I	2.45112782
AR1	3.02E-98	0.9683406	0.356	0.148	7.29E-94	2.2	AR	2.405405405
EGOT1	7.60E-98	0.8608328	0.288	0.106	1.83E-93	2.2	EGOT	2.716981132
ADGRL31	1.43E-96	0.9385722	0.276	0.098	3.45E-92	2.2	ADGRL3	2.816326531
GCNT21	5.06E-96	1.374725	0.616	0.435	1.22E-91	2.2	GCNT2	1.416091954
SLC7A21	6.58E-95	0.7918076	0.542	0.287	1.59E-90	2.2	SLC7A2	1.888501742
CTC-558O2.11	1.22E-94	0.2826186	0.056	0.004	2.93E-90	2.2	CTC-558O2	14
ZNF689	1.58E-93	0.3739681	0.084	0.011	3.82E-89	2.2	ZNF689	7.636363636
HES1	5.20E-92	1.313742	0.537	0.331	1.25E-87	2.2	HES1	1.622356495
MPHOSPH6	8.38E-92	0.8209065	0.301	0.118	2.02E-87	2.2	MPHOSPH(	2.550847458
MLPH1	2.12E-90	0.7110246	0.211	0.065	5.12E-86	2.2	MLPH	3.246153846
DENND4A1	3.30E-89	1.103746	0.711	0.588	7.96E-85	2.2	DENND4A	1.209183673
TMEM21	3.32E-89	1.140136	0.542	0.336	8.01E-85	2.2	TMEM2	1.613095238
ATP5EP21	1.71E-88	0.9464375	0.497	0.293	4.11E-84	2.2	ATP5EP2	1.696245734
RP1	2.26E-88	0.2661589	0.052	0.004	5.44E-84	2.2	RP1	13
FTO1	2.30E-88	0.8560246	0.452	0.243	5.53E-84	2.2	FTO	1.860082305
C3orf14	2.46E-88	0.7932833	0.274	0.104	5.93E-84	2.2	C3orf14	2.634615385
UBL51	2.73E-87	0.934341	0.66	0.501	6.59E-83	2.2	UBL5	1.317365269
TSPAN51	3.97E-86	0.8900428	0.523	0.296	9.57E-82	2.2	TSPAN5	1.766891892
RCL11	4.41E-86	0.6865944	0.202	0.062	1.06E-81	2.2	RCL1	3.258064516
MAP3K11	4.57E-85	0.8128553	0.663	0.446	1.10E-80	2.2	MAP3K1	1.486547085
NFKB12	5.50E-84	1.037532	0.61	0.441	1.33E-79	2.2	NFKB1	1.383219955
AMPH1	5.30E-83	0.8299315	0.254	0.092	1.28E-78	2.2	AMPH	2.760869565
RAB11A2	4.41E-82	0.9681773	0.731	0.637	1.06E-77	2.2	RAB11A	1.147566719
CCBE11	4.97E-82	0.6744539	0.161	0.043	1.20E-77	2.2	CCBE1	3.744186047
SUSD61	1.50E-81	0.8961799	0.469	0.268	3.62E-77	2.2	SUSD6	1.75
UQCC21	5.14E-81	0.5614197	0.169	0.047	1.24E-76	2.2	UQCC2	3.595744681
REEP51	6.56E-81	0.8544482	0.44	0.242	1.58E-76	2.2	REEP5	1.818181818
APBB2	1.61E-80	0.9234745	0.453	0.246	3.89E-76	2.2	APBB2	1.841463415
MSMO11	1.70E-80	0.9285917	0.528	0.322	4.10E-76	2.2	MSMO1	1.639751553
C9orf152	2.72E-80	0.5720205	0.156	0.041	6.56E-76	2.2	C9orf152	3.804878049
SLC2A10	3.23E-80	0.3471165	0.078	0.011	7.80E-76	2.2	SLC2A10	7.090909091
GALNT61	1.85E-79	0.6138185	0.186	0.056	4.45E-75	2.2	GALNT6	3.321428571

MALAT11	2.05E-79	0.3595439	1	1	4.93E-75	2.2	MALAT1	1
FBP11	3.10E-79	0.4979548	0.133	0.031	7.47E-75	2.2	FBP1	4.290322581
ATP8B1	7.71E-79	1.138663	0.571	0.395	1.86E-74	2.2	ATP8B1	1.44556962
AZGP11	1.01E-78	0.6677332	0.74	0.536	2.45E-74	2.2	AZGP1	1.380597015
ATP5L4	1.99E-78	0.5910223	0.877	0.806	4.79E-74	2.2	ATP5L	1.08808933
C1orf211	1.25E-77	0.8998225	0.504	0.303	3.02E-73	2.2	C1orf21	1.663366337
C14orf21	1.36E-77	0.7592973	0.77	0.705	3.28E-73	2.2	C14orf2	1.092198582
PTPN131	3.62E-77	0.85675	0.357	0.169	8.73E-73	2.2	PTPN13	2.112426036
TCEAL41	1.92E-76	0.8973404	0.401	0.215	4.63E-72	2.2	TCEAL4	1.865116279
MORN22	2.87E-76	0.5411825	0.18	0.054	6.93E-72	2.2	MORN2	3.333333333
PPCS	6.13E-75	0.4741929	0.146	0.038	1.48E-70	2.2	PPCS	3.842105263
PEBP41	8.01E-75	0.7358931	0.236	0.087	1.93E-70	2.2	PEBP4	2.712643678
SRP141	2.20E-74	0.5187575	0.916	0.871	5.31E-70	2.2	SRP14	1.051664753
TBC1D91	2.64E-74	0.8102456	0.624	0.439	6.37E-70	2.2	TBC1D9	1.421412301
MB21D21	4.76E-74	0.7854057	0.235	0.088	1.15E-69	2.2	MB21D2	2.670454545
NDUFA42	2.17E-73	0.6286881	0.821	0.745	5.22E-69	2.2	NDUFA4	1.102013423
PLEKHB21	1.24E-72	0.7646046	0.326	0.154	2.98E-68	2.2	PLEKHB2	2.116883117
ERBB2IP1	2.17E-72	1.042042	0.578	0.42	5.24E-68	2.2	ERBB2IP	1.376190476
MT1E	2.95E-72	0.4284959	0.156	0.043	7.11E-68	2.2	MT1E	3.627906977
CSNK1A11	4.46E-72	0.7659387	0.756	0.649	1.07E-67	2.2	CSNK1A1	1.164869029
PANK31	7.64E-72	0.7884728	0.345	0.168	1.84E-67	2.2	PANK3	2.053571429
PDIA32	1.84E-71	0.8814373	0.481	0.297	4.44E-67	2.2	PDIA3	1.61952862
NDUFB4	3.10E-71	0.9061913	0.571	0.428	7.48E-67	2.2	NDUFB4	1.33411215
TSC22D22	6.15E-71	0.8357505	0.634	0.458	1.48E-66	2.2	TSC22D2	1.384279476
ATOX11	2.04E-70	0.6114155	0.224	0.084	4.91E-66	2.2	ATOX1	2.666666667
SEC622	4.39E-70	0.7225688	0.708	0.57	1.06E-65	2.2	SEC62	1.242105263
STON2	9.27E-70	0.6162512	0.15	0.043	2.24E-65	2.2	STON2	3.488372093
WLS1	3.88E-69	0.7844035	0.341	0.166	9.37E-65	2.2	WLS	2.054216867
NQO11	5.01E-68	0.54987	0.178	0.057	1.21E-63	2.2	NQO1	3.122807018
NFAT52	5.29E-68	1.017998	0.697	0.625	1.28E-63	2.2	NFAT5	1.1152
PRR15L	1.25E-67	0.4283651	0.12	0.029	3.00E-63	2.2	PRR15L	4.137931034
HSPA1A1	9.73E-65	0.7553454	0.2	0.072	2.35E-60	2.2	HSPA1A	2.777777778
KIAA13241	1.17E-64	0.7550944	0.394	0.219	2.82E-60	2.2	KIAA1324	1.799086758
ATP5J1	1.20E-64	0.7672748	0.624	0.492	2.89E-60	2.2	ATP5J	1.268292683
CRACR2A1	1.32E-64	0.3941083	0.09	0.018	3.18E-60	2.2	CRACR2A	5
C1orf168	2.88E-64	0.2219503	0.041	0.004	6.95E-60	2.2	C1orf168	10.25
FASN1	5.21E-64	0.5482078	0.152	0.046	1.26E-59	2.2	FASN	3.304347826
GNAS1	6.33E-64	0.5672052	0.838	0.748	1.53E-59	2.2	GNAS	1.120320856
MEPE1	1.20E-63	0.3016736	0.069	0.011	2.88E-59	2.2	MEPE	6.272727273
QPRT1	1.80E-63	0.2466197	0.062	0.009	4.34E-59	2.2	QPRT	6.888888889
HSPA61	4.71E-63	0.1709173	0.031	0.002	1.14E-58	2.2	HSPA6	15.5
ALOX15B	1.42E-62	0.5363947	0.158	0.05	3.43E-58	2.2	ALOX15B	3.16
TTC39A1	3.94E-62	0.5154951	0.148	0.045	9.50E-58	2.2	TTC39A	3.288888889
SEPP12	2.38E-61	0.8868992	0.472	0.303	5.75E-57	2.2	SEPP1	1.557755776
NDUFA11	1.15E-60	0.7048093	0.696	0.602	2.76E-56	2.2	NDUFA1	1.156146179
ZSCAN16-AS11	1.29E-60	0.5546073	0.214	0.084	3.10E-56	2.2	ZSCAN16-A	2.547619048
AC008268.1	6.26E-60	0.1966825	0.034	0.002	1.51E-55	2.2	AC008268.	17
FKBP41	3.91E-59	0.5574349	0.182	0.066	9.43E-55	2.2	FKBP4	2.757575758
ATP5G12	5.62E-59	0.7477431	0.333	0.174	1.36E-54	2.2	ATP5G1	1.913793103
PARK71	5.86E-59	0.7007715	0.588	0.448	1.41E-54	2.2	PARK7	1.3125
SLIRP2	7.09E-59	0.8405801	0.458	0.301	1.71E-54	2.2	SLIRP	1.521594684
HSPA1B1	7.76E-59	0.6600099	0.173	0.06	1.87E-54	2.2	HSPA1B	2.883333333

ZFP36L22	1.00E-58	0.6066508	0.558	0.361	2.42E-54	2.2	ZFP36L2	1.545706371
KLHL131	1.51E-58	0.8043619	0.24	0.102	3.64E-54	2.2	KLHL13	2.352941176
DLGAP11	1.63E-58	0.9103335	0.362	0.195	3.93E-54	2.2	DLGAP1	1.856410256
CTNND21	1.79E-58	0.7452787	0.301	0.146	4.32E-54	2.2	CTNND2	2.061643836
IRX3	1.24E-57	0.6044838	0.21	0.084	2.99E-53	2.2	IRX3	2.5
SCGB2A21	1.46E-57	1.620867	0.441	0.264	3.51E-53	2.2	SCGB2A2	1.670454545
CRY12	2.50E-57	0.8891688	0.535	0.377	6.03E-53	2.2	CRY1	1.419098143
LSAMP1	5.65E-57	0.8863306	0.622	0.463	1.36E-52	2.2	LSAMP	1.343412527
MAGI31	1.11E-56	0.8330907	0.539	0.375	2.67E-52	2.2	MAGI3	1.437333333
PDZD81	3.99E-56	0.7336445	0.387	0.223	9.61E-52	2.2	PDZD8	1.735426009
ZSWIM61	7.24E-56	0.7740365	0.632	0.491	1.75E-51	2.2	ZSWIM6	1.287169043
CMBL1	3.84E-55	0.3358365	0.089	0.02	9.25E-51	2.2	CMBL	4.45
MRPL241	3.98E-55	0.4767204	0.168	0.059	9.59E-51	2.2	MRPL24	2.847457627
UQCRQ1	2.69E-54	0.608029	0.242	0.108	6.49E-50	2.2	UQCRQ	2.240740741
ERBB21	2.78E-54	0.612732	0.245	0.11	6.70E-50	2.2	ERBB2	2.227272727
AC018816.31	6.40E-54	0.6417032	0.219	0.092	1.54E-49	2.2	AC018816.3	2.380434783
SLC40A1	1.02E-53	0.2995705	0.085	0.019	2.46E-49	2.2	SLC40A1	4.473684211
NRIP11	2.75E-53	0.7792682	0.423	0.26	6.64E-49	2.2	NRIP1	1.626923077
ARID4B1	5.51E-53	0.8500515	0.629	0.553	1.33E-48	2.2	ARID4B	1.137432188
RP11-140K17.3	7.96E-53	0.4164561	0.126	0.038	1.92E-48	2.2	RP11-140K	3.315789474
HS6ST21	1.88E-52	0.6909583	0.286	0.139	4.54E-48	2.2	HS6ST2	2.057553957
HSPA52	1.93E-52	0.5347802	0.612	0.441	4.65E-48	2.2	HSPA5	1.387755102
SERF21	5.99E-52	0.6660597	0.545	0.386	1.44E-47	2.2	SERF2	1.411917098
SLC7A81	6.70E-52	0.4139238	0.128	0.039	1.62E-47	2.2	SLC7A8	3.282051282
SPRED2	1.37E-51	0.7037343	0.359	0.204	3.30E-47	2.2	SPRED2	1.759803922
IRX5	1.01E-50	0.3952788	0.106	0.029	2.44E-46	2.2	IRX5	3.655172414
MCCC21	1.06E-50	0.6780621	0.29	0.151	2.56E-46	2.2	MCCC2	1.920529801
PPP3CA1	6.30E-50	0.6852381	0.676	0.558	1.52E-45	2.2	PPP3CA	1.211469534
OST41	2.82E-49	0.6900366	0.534	0.408	6.80E-45	2.2	OST4	1.308823529
ITPR11	3.51E-49	0.5581298	0.217	0.095	8.46E-45	2.2	ITPR1	2.284210526
TMEM135	8.60E-49	0.7014288	0.225	0.102	2.07E-44	2.2	TMEM135	2.205882353
ZBED5-AS12	9.84E-49	0.3262706	0.1	0.027	2.37E-44	2.2	ZBED5-AS1	3.703703704
RGS221	1.71E-48	0.2780255	0.052	0.008	4.11E-44	2.2	RGS22	6.5
SRP91	1.97E-48	0.6388088	0.543	0.416	4.75E-44	2.2	SRP9	1.305288462
CHD21	3.48E-48	0.8448474	0.622	0.544	8.40E-44	2.2	CHD2	1.143382353
EIF2AK32	6.82E-48	0.7085965	0.507	0.354	1.65E-43	2.2	EIF2AK3	1.43220339
NRXN31	8.18E-48	0.7257138	0.252	0.12	1.97E-43	2.2	NRXN3	2.1
AK5	4.18E-47	0.376091	0.094	0.024	1.01E-42	2.2	AK5	3.916666667
CETN2	1.38E-46	0.6153958	0.314	0.175	3.34E-42	2.2	CETN2	1.794285714
RP11-22N19.21	5.23E-46	0.2858727	0.066	0.014	1.26E-41	2.2	RP11-22N1	4.714285714
NDUFB31	1.42E-45	0.7022541	0.345	0.208	3.43E-41	2.2	NDUFB3	1.658653846
RP11-460M2.11	1.70E-45	0.2641835	0.065	0.013	4.11E-41	2.2	RP11-460M	5
RPN21	1.74E-45	0.6346806	0.403	0.261	4.19E-41	2.2	RPN2	1.544061303
NBPF191	8.96E-45	0.67824	0.348	0.208	2.16E-40	2.2	NBPF19	1.673076923
SLC9A3R1	1.06E-44	0.5774716	0.201	0.09	2.55E-40	2.2	SLC9A3R1	2.233333333
EREG1	5.27E-44	0.4603165	0.43	0.251	1.27E-39	2.2	EREG	1.71314741
WNT4	1.24E-43	0.2559973	0.055	0.01	2.98E-39	2.2	WNT4	5.5
COX7A23	3.71E-43	0.5184783	0.795	0.761	8.95E-39	2.2	COX7A2	1.044678055
DECR11	6.16E-43	0.6084647	0.281	0.152	1.48E-38	2.2	DECR1	1.848684211
NBPF141	1.35E-42	0.6354325	0.28	0.152	3.26E-38	2.2	NBPF14	1.842105263
MUCL1	1.93E-42	0.5092453	0.408	0.266	4.64E-38	2.2	MUCL1	1.533834586
CCDC831	2.38E-42	0.2744171	0.074	0.018	5.74E-38	2.2	CCDC83	4.111111111

VAV31	4.23E-42	0.6471848	0.426	0.278	1.02E-37	2.2	VAV3	1.532374101
PREX11	4.96E-42	0.4571339	0.136	0.049	1.20E-37	2.2	PREX1	2.775510204
HSBP1	8.95E-42	0.5548015	0.647	0.55	2.16E-37	2.2	HSBP1	1.176363636
TOP11	2.00E-41	0.6874852	0.549	0.441	4.83E-37	2.2	TOP1	1.244897959
ABRACL1	2.07E-41	0.6332933	0.536	0.416	4.99E-37	2.2	ABRACL	1.288461538
NME5	3.55E-41	0.3269409	0.085	0.023	8.57E-37	2.2	NME5	3.695652174
LFNG	3.76E-41	0.1286945	0.03	0.003	9.06E-37	2.2	LFNG	10
PCCA1	4.15E-41	0.7420848	0.323	0.193	1.00E-36	2.2	PCCA	1.67357513
GLA1	7.63E-41	0.5865155	0.199	0.091	1.84E-36	2.2	GLA	2.186813187
SAP181	2.67E-40	0.5181245	0.648	0.567	6.43E-36	2.2	SAP18	1.142857143
KTN11	3.78E-40	0.5933613	0.628	0.538	9.12E-36	2.2	KTN1	1.167286245
MIR4458HG	6.08E-40	0.4727577	0.156	0.063	1.47E-35	2.2	MIR4458H(	2.476190476
CPEB21	8.59E-40	0.4763319	0.467	0.301	2.07E-35	2.2	CPEB2	1.551495017
DCXR1	1.41E-39	0.241372	0.065	0.015	3.39E-35	2.2	DCXR	4.333333333
LARP4	1.90E-39	0.5976636	0.251	0.133	4.58E-35	2.2	LARP4	1.887218045
CD1641	3.01E-39	0.6470968	0.405	0.276	7.26E-35	2.2	CD164	1.467391304
GSTA42	3.69E-39	0.4158093	0.139	0.053	8.89E-35	2.2	GSTA4	2.622641509
TMCO12	7.58E-39	0.6030334	0.461	0.34	1.83E-34	2.2	TMCO1	1.355882353
AIG1	9.49E-39	0.5858248	0.332	0.2	2.29E-34	2.2	AIG1	1.66
GTF2H52	1.02E-38	0.6382601	0.334	0.209	2.45E-34	2.2	GTF2H5	1.598086124
U47924.271	1.19E-38	0.3486778	0.099	0.031	2.86E-34	2.2	U47924.27	3.193548387
SMIM141	2.83E-38	0.5773076	0.532	0.392	6.81E-34	2.2	SMIM14	1.357142857
YTHDC12	3.15E-38	0.6409168	0.506	0.378	7.59E-34	2.2	YTHDC1	1.338624339
TSPAN1	3.92E-38	0.5380049	0.363	0.22	9.44E-34	2.2	TSPAN1	1.65
ATF6	4.23E-38	0.6300165	0.323	0.198	1.02E-33	2.2	ATF6	1.631313131
HSPE1-MOB4	4.56E-38	0.2299493	0.049	0.009	1.10E-33	2.2	HSPE1-MO	5.444444444
TXN3	1.01E-37	0.5014291	0.738	0.672	2.43E-33	2.2	TXN	1.098214286
UBR52	1.10E-37	0.618051	0.564	0.466	2.65E-33	2.2	UBR5	1.210300429
SMG11	1.58E-37	0.7270945	0.438	0.322	3.80E-33	2.2	SMG1	1.360248447
B3GALNT11	1.60E-37	0.1951567	0.053	0.011	3.85E-33	2.2	B3GALNT1	4.818181818
LSM31	1.85E-37	0.6447138	0.438	0.324	4.45E-33	2.2	LSM3	1.351851852
SHROOM31	3.08E-37	0.564843	0.705	0.628	7.43E-33	2.2	SHROOM3	1.122611465
CYTH31	3.59E-37	0.7053898	0.219	0.111	8.66E-33	2.2	CYTH3	1.972972973
GPS21	4.32E-37	0.5853968	0.288	0.164	1.04E-32	2.2	GPS2	1.756097561
NUP1532	6.09E-37	0.6171867	0.405	0.276	1.47E-32	2.2	NUP153	1.467391304
RHOBTB31	1.01E-36	0.5951726	0.242	0.127	2.42E-32	2.2	RHOBTB3	1.905511811
NCKAP5	1.44E-36	0.5338854	0.301	0.171	3.47E-32	2.2	NCKAP5	1.760233918
MAFB	1.52E-36	0.4420912	0.122	0.045	3.66E-32	2.2	MAFB	2.711111111
OLA11	2.29E-36	0.6174594	0.441	0.318	5.52E-32	2.2	OLA1	1.386792453
ENTPD3-AS1	2.39E-36	0.2906144	0.079	0.022	5.76E-32	2.2	ENTPD3-AS	3.590909091
STOM1	2.48E-36	0.6066981	0.387	0.255	5.98E-32	2.2	STOM	1.517647059
FGF141	2.74E-36	0.5238149	0.183	0.082	6.60E-32	2.2	FGF14	2.231707317
WDR61	3.57E-36	0.5079973	0.214	0.108	8.61E-32	2.2	WDR61	1.981481481
CYCS3	5.07E-36	0.6492672	0.61	0.516	1.22E-31	2.2	CYCS	1.182170543
NR4A11	5.48E-36	0.4357754	0.111	0.039	1.32E-31	2.2	NR4A1	2.846153846
LL22NC03-104C7.11	5.55E-36	0.1116972	0.03	0.004	1.34E-31	2.2	LL22NC03-:	7.5
MRPL511	6.15E-36	0.6194357	0.524	0.429	1.48E-31	2.2	MRPL51	1.221445221
ATF7IP2	8.07E-36	0.4743791	0.112	0.04	1.95E-31	2.2	ATF7IP2	2.8
PPP2R2A2	1.31E-35	0.5566734	0.624	0.526	3.15E-31	2.2	PPP2R2A	1.186311787
AC020571.3	1.38E-35	0.1196848	0.026	0.003	3.34E-31	2.2	AC020571.:	8.666666667
DYNC1I2	1.65E-35	0.6364816	0.481	0.377	3.98E-31	2.2	DYNC1I2	1.275862069
SETD52	3.11E-35	0.6608089	0.593	0.524	7.50E-31	2.2	SETD5	1.131679389

AC018890.61	3.83E-35	0.6297416	0.354	0.233	9.22E-31	2.2	AC018890.(	1.519313305
WDR433	4.93E-35	0.6113126	0.203	0.102	1.19E-30	2.2	WDR43	1.990196078
SCGB1D21	5.02E-35	0.5191684	0.226	0.113	1.21E-30	2.2	SCGB1D2	2
KCNK6	6.74E-35	0.3882233	0.114	0.041	1.63E-30	2.2	KCNK6	2.780487805
DNAJB1	7.71E-35	0.6564631	0.219	0.115	1.86E-30	2.2	DNAJB1	1.904347826
ZBTB101	1.12E-34	0.6743241	0.373	0.251	2.70E-30	2.2	ZBTB10	1.486055777
ARSD	1.14E-34	0.3786998	0.121	0.046	2.74E-30	2.2	ARSD	2.630434783
DYNLL12	1.43E-34	0.580098	0.362	0.238	3.44E-30	2.2	DYNLL1	1.521008403
AKAP131	1.44E-34	0.6299689	0.664	0.609	3.47E-30	2.2	AKAP13	1.090311987
IL1R1	2.04E-34	0.4341013	0.155	0.067	4.92E-30	2.2	IL1R1	2.313432836
SNX5	2.58E-34	0.4197075	0.147	0.062	6.21E-30	2.2	SNX5	2.370967742
DHFR3	2.97E-34	0.5498354	0.857	0.786	7.16E-30	2.2	DHFR	1.090330789
RP11-6N13.1	3.58E-34	0.4262962	0.114	0.041	8.64E-30	2.2	RP11-6N13	2.780487805
PIAS11	4.61E-34	0.647302	0.558	0.482	1.11E-29	2.2	PIAS1	1.157676349
AZIN11	5.84E-34	0.6124914	0.318	0.199	1.41E-29	2.2	AZIN1	1.59798995
SCN9A1	6.03E-34	0.1957788	0.046	0.009	1.45E-29	2.2	SCN9A	5.111111111
ARHGEF382	6.03E-34	0.6831032	0.566	0.481	1.46E-29	2.2	ARHGEF38	1.176715177
DTNA2	6.19E-34	0.6957895	0.766	0.758	1.49E-29	2.2	DTNA	1.01055409
CLIC6	7.66E-34	0.7093657	0.333	0.212	1.85E-29	2.2	CLIC6	1.570754717
CERS61	8.66E-34	0.609907	0.276	0.16	2.09E-29	2.2	CERS6	1.725
EMP2	9.29E-34	0.4637692	0.225	0.117	2.24E-29	2.2	EMP2	1.923076923
ALCAM1	3.48E-33	0.3861185	0.655	0.504	8.38E-29	2.2	ALCAM	1.299603175
GATM	7.71E-33	0.2361098	0.065	0.017	1.86E-28	2.2	GATM	3.823529412
SPA17	1.60E-32	0.3329389	0.098	0.034	3.86E-28	2.2	SPA17	2.882352941
CREB3L4	2.67E-32	0.2167421	0.047	0.01	6.45E-28	2.2	CREB3L4	4.7
ZFAND31	3.72E-32	0.5240355	0.772	0.761	8.96E-28	2.2	ZFAND3	1.014454665
ELP21	4.05E-32	0.5588577	0.326	0.209	9.75E-28	2.2	ELP2	1.559808612
DIO1	4.26E-32	0.4490685	0.051	0.011	1.03E-27	2.2	DIO1	4.636363636
CCDC102B	4.38E-32	0.3103508	0.087	0.028	1.06E-27	2.2	CCDC102B	3.107142857
VTCN1	5.27E-32	0.5698235	0.203	0.104	1.27E-27	2.2	VTCN1	1.951923077
HPGD1	7.13E-32	0.1898892	0.047	0.01	1.72E-27	2.2	HPGD	4.7
LINC015501	7.98E-32	0.1109116	0.027	0.003	1.92E-27	2.2	LINC01550	9
DNAJC31	8.10E-32	0.5815872	0.369	0.254	1.95E-27	2.2	DNAJC3	1.452755906
GOLGA42	9.09E-32	0.5740246	0.663	0.619	2.19E-27	2.2	GOLGA4	1.071082391
TCEA21	1.14E-31	0.4430986	0.055	0.013	2.76E-27	2.2	TCEA2	4.230769231
RASEF1	1.26E-31	0.5949576	0.391	0.266	3.05E-27	2.2	RASEF	1.469924812
IL1RN1	1.35E-31	0.3475598	0.121	0.047	3.26E-27	2.2	IL1RN	2.574468085
TSPAN131	1.38E-31	0.4815272	0.207	0.108	3.32E-27	2.2	TSPAN13	1.916666667
TXNDC17	2.04E-31	0.4225657	0.147	0.066	4.91E-27	2.2	TXNDC17	2.227272727
RP4-568C11.4	2.05E-31	0.3280163	0.104	0.038	4.94E-27	2.2	RP4-568C1	2.736842105
TFAP2B	2.07E-31	0.3685932	0.144	0.062	4.99E-27	2.2	TFAP2B	2.322580645
ZFAND2A1	2.54E-31	0.418729	0.1	0.036	6.14E-27	2.2	ZFAND2A	2.777777778
MGST23	3.11E-31	0.4921079	0.257	0.15	7.49E-27	2.2	MGST2	1.713333333
MREG1	3.23E-31	0.5285245	0.242	0.136	7.79E-27	2.2	MREG	1.779411765
C2orf742	3.25E-31	0.3386804	0.093	0.032	7.83E-27	2.2	C2orf74	2.90625
APLP21	3.68E-31	0.4873976	0.397	0.272	8.88E-27	2.2	APLP2	1.459558824
PLEKHA74	3.92E-31	0.2511013	0.983	0.979	9.46E-27	2.2	PLEKHA7	1.004085802
MRPS21	4.29E-31	0.5592997	0.581	0.515	1.04E-26	2.2	MRPS21	1.12815534
ITGA101	6.43E-31	0.4121479	0.123	0.05	1.55E-26	2.2	ITGA10	2.46
ANAPC131	7.20E-31	0.5109929	0.245	0.141	1.74E-26	2.2	ANAPC13	1.737588652
UGDH-AS11	9.22E-31	0.6812606	0.201	0.105	2.22E-26	2.2	UGDH-AS1	1.914285714
FAM210B1	1.24E-30	0.391874	0.136	0.059	3.00E-26	2.2	FAM210B	2.305084746

CTD-2336O2.11	1.52E-30	0.1888699	0.041	0.008	3.67E-26	2.2	CTD-2336C	5.125
STK32B	1.69E-30	0.3025977	0.072	0.021	4.07E-26	2.2	STK32B	3.428571429
CSPP11	1.96E-30	0.5504772	0.281	0.172	4.72E-26	2.2	CSPP1	1.63372093
TBC1D41	2.12E-30	0.5684943	0.302	0.19	5.10E-26	2.2	TBC1D4	1.589473684
PRPS21	2.40E-30	0.4144741	0.164	0.077	5.79E-26	2.2	PRPS2	2.12987013
POLR2K	2.41E-30	0.586991	0.462	0.365	5.82E-26	2.2	POLR2K	1.265753425
CTA-392E5.11	5.42E-30	0.5010328	0.088	0.03	1.31E-25	2.2	CTA-392E5	2.933333333
LINGO12	6.86E-30	0.3949583	0.875	0.89	1.65E-25	2.2	LINGO1	0.983146067
ASTN21	9.09E-30	0.6108706	0.382	0.268	2.19E-25	2.2	ASTN2	1.425373134
SC5D1	1.87E-29	0.532628	0.29	0.178	4.51E-25	2.2	SC5D	1.629213483
HNRNPM1	2.10E-29	0.5544301	0.369	0.257	5.06E-25	2.2	HNRNPM	1.435797665
ESR11	2.14E-29	0.5270251	0.291	0.18	5.17E-25	2.2	ESR1	1.616666667
FAM81B1	2.30E-29	0.2092899	0.056	0.014	5.54E-25	2.2	FAM81B	4
NPFFR21	2.46E-29	0.3376631	0.074	0.023	5.94E-25	2.2	NPFFR2	3.217391304
DMXL11	2.98E-29	0.5275639	0.267	0.162	7.19E-25	2.2	DMXL1	1.648148148
CRNDE1	3.95E-29	0.4731152	0.19	0.099	9.52E-25	2.2	CRNDE	1.919191919
RAB301	4.34E-29	0.6034283	0.214	0.117	1.05E-24	2.2	RAB30	1.829059829
ERO1B1	1.42E-28	0.5090085	0.183	0.093	3.43E-24	2.2	ERO1B	1.967741935
ATP5H2	1.54E-28	0.5817733	0.44	0.345	3.71E-24	2.2	ATP5H	1.275362319
MEGF91	1.79E-28	0.546204	0.228	0.128	4.31E-24	2.2	MEGF9	1.78125
ECE11	1.83E-28	0.5371629	0.291	0.182	4.42E-24	2.2	ECE1	1.598901099
TMPRSS11E	2.10E-28	0.3597891	0.122	0.05	5.07E-24	2.2	TMPRSS11I	2.44
RBM472	2.34E-28	0.5053207	0.698	0.634	5.65E-24	2.2	RBM47	1.100946372
NOTCH21	3.54E-28	0.5847295	0.337	0.234	8.53E-24	2.2	NOTCH2	1.44017094
MBNL21	5.00E-28	0.5584644	0.61	0.54	1.20E-23	2.2	MBNL2	1.12962963
SSX2IP	5.40E-28	0.2329308	0.055	0.015	1.30E-23	2.2	SSX2IP	3.666666667
DUSP101	6.00E-28	0.4948841	0.334	0.219	1.45E-23	2.2	DUSP10	1.525114155
USMG51	6.12E-28	0.4846672	0.677	0.627	1.48E-23	2.2	USMG5	1.079744817
MORF4L23	6.35E-28	0.3931311	0.768	0.741	1.53E-23	2.2	MORF4L2	1.036437247
HSP90B12	7.49E-28	0.3473885	0.609	0.502	1.81E-23	2.2	HSP90B1	1.21314741
HSPB11	8.32E-28	0.4499174	0.186	0.097	2.01E-23	2.2	HSPB11	1.917525773
ERP441	9.89E-28	0.540194	0.235	0.138	2.39E-23	2.2	ERP44	1.702898551
ACADM	1.02E-27	0.4171869	0.162	0.079	2.45E-23	2.2	ACADM	2.050632911
CAPN81	1.05E-27	0.4727249	0.332	0.213	2.53E-23	2.2	CAPN8	1.558685446
MAPK81	1.77E-27	0.6170178	0.433	0.329	4.26E-23	2.2	MAPK8	1.316109422
COMMD63	2.59E-27	0.5320175	0.444	0.35	6.24E-23	2.2	COMMD6	1.268571429
MAOA1	2.67E-27	0.3307895	0.126	0.054	6.44E-23	2.2	MAOA	2.333333333
RAB7A1	3.29E-27	0.5668505	0.523	0.449	7.94E-23	2.2	RAB7A	1.16481069
ALG8	3.95E-27	0.3307568	0.105	0.042	9.53E-23	2.2	ALG8	2.5
BCL2L15	4.89E-27	0.1341834	0.028	0.004	1.18E-22	2.2	BCL2L15	7
ABCD31	4.92E-27	0.5131925	0.278	0.175	1.19E-22	2.2	ABCD3	1.588571429
MRPL402	9.19E-27	0.4260379	0.174	0.09	2.22E-22	2.2	MRPL40	1.933333333
NUDT4	1.14E-26	0.4053174	0.147	0.069	2.75E-22	2.2	NUDT4	2.130434783
FAM198B	1.94E-26	0.1323902	0.028	0.004	4.68E-22	2.2	FAM198B	7
HMGN22	1.95E-26	0.3727927	0.122	0.053	4.71E-22	2.2	HMGN2	2.301886792
CDC14A1	2.36E-26	0.5533393	0.256	0.156	5.69E-22	2.2	CDC14A	1.641025641
NDUFA121	2.44E-26	0.5343266	0.306	0.206	5.88E-22	2.2	NDUFA12	1.485436893
LINC002901	3.59E-26	0.4483428	0.079	0.027	8.65E-22	2.2	LINC00290	2.925925926
DDB11	3.67E-26	0.4806808	0.216	0.124	8.84E-22	2.2	DDB1	1.741935484
LARP1B1	3.80E-26	0.5963433	0.316	0.212	9.17E-22	2.2	LARP1B	1.490566038
HNRNPA2B12	4.46E-26	0.4955788	0.493	0.409	1.08E-21	2.2	HNRNPA2E	1.205378973
LMCD11	7.14E-26	0.5472722	0.195	0.104	1.72E-21	2.2	LMCD1	1.875

DRAIC	7.82E-26	0.2126721	0.051	0.013	1.89E-21	2.2	DRAIC	3.923076923
MYB1	8.70E-26	0.2463373	0.065	0.02	2.10E-21	2.2	MYB	3.25
ELF11	1.15E-25	0.503262	0.595	0.526	2.78E-21	2.2	ELF1	1.131178707
NIT21	1.29E-25	0.4204065	0.188	0.102	3.12E-21	2.2	NIT2	1.843137255
LRIG12	1.53E-25	0.4961086	0.33	0.224	3.70E-21	2.2	LRIG1	1.473214286
SPTSSB	1.74E-25	0.4791439	0.169	0.086	4.19E-21	2.2	SPTSSB	1.965116279
NDUFB9	1.93E-25	0.6493735	0.314	0.218	4.65E-21	2.2	NDUFB9	1.440366972
CHMP4B1	2.05E-25	0.5004547	0.288	0.187	4.94E-21	2.2	CHMP4B	1.540106952
LEMD31	2.10E-25	0.418372	0.169	0.087	5.06E-21	2.2	LEMD3	1.942528736
SEPT4-AS12	4.35E-25	0.1420618	0.037	0.008	1.05E-20	2.2	SEPT4-AS1	4.625
RP3-523E19.21	4.73E-25	0.3141707	0.099	0.039	1.14E-20	2.2	RP3-523E1!	2.538461538
MARCH62	4.91E-25	0.4815336	0.484	0.397	1.18E-20	2.2	MARCH6	1.219143577
YWHAE1	5.31E-25	0.4325911	0.672	0.626	1.28E-20	2.2	YWHAE	1.073482428
LINC009982	5.86E-25	0.4483361	0.214	0.125	1.41E-20	2.2	LINC00998	1.712
IGFL4	6.17E-25	0.4846508	0.044	0.011	1.49E-20	2.2	IGFL4	4
HSPA41	7.72E-25	0.573581	0.228	0.137	1.86E-20	2.2	HSPA4	1.664233577
RPS27L1	8.96E-25	0.4329225	0.598	0.513	2.16E-20	2.2	RPS27L	1.165692008
C4orf33	9.51E-25	0.1791314	0.049	0.013	2.29E-20	2.2	C4orf33	3.769230769
UBXN81	1.01E-24	0.2061108	0.068	0.022	2.43E-20	2.2	UBXN8	3.090909091
APOD	1.11E-24	0.8692256	0.118	0.052	2.69E-20	2.2	APOD	2.269230769
ERLIN2	1.15E-24	0.313235	0.104	0.043	2.76E-20	2.2	ERLIN2	2.418604651
RASSF6	1.27E-24	0.3913661	0.139	0.066	3.06E-20	2.2	RASSF6	2.106060606
ZNF385B1	1.32E-24	0.2816932	0.058	0.017	3.18E-20	2.2	ZNF385B	3.411764706
ASAH11	1.83E-24	0.3813007	0.182	0.098	4.41E-20	2.2	ASAH1	1.857142857
COL4A51	2.07E-24	0.4823552	0.321	0.215	4.99E-20	2.2	COL4A5	1.493023256
ABCC81	2.19E-24	0.1568598	0.031	0.006	5.29E-20	2.2	ABCC8	5.166666667
FAM234B1	2.29E-24	0.3208672	0.106	0.045	5.53E-20	2.2	FAM234B	2.355555556
UCP2	2.43E-24	0.2581491	0.084	0.031	5.87E-20	2.2	UCP2	2.709677419
IQCJ-SCHIP1	3.44E-24	0.582567	0.175	0.094	8.30E-20	2.2	IQCJ-SCHIP	1.861702128
CNDP21	4.35E-24	0.481142	0.207	0.121	1.05E-19	2.2	CNDP2	1.710743802
SEL1L4	8.60E-24	0.3989594	0.181	0.098	2.07E-19	2.2	SEL1L	1.846938776
PBDC1	9.39E-24	0.4949907	0.215	0.13	2.26E-19	2.2	PBDC1	1.653846154
TAT	1.11E-23	0.2563976	0.041	0.01	2.67E-19	2.2	TAT	4.1
RNF2201	1.51E-23	1.06881	0.14	0.07	3.64E-19	2.2	RNF220	2
RPS294	1.56E-23	0.3328221	0.917	0.925	3.77E-19	2.2	RPS29	0.991351351
HMGN32	1.57E-23	0.4206248	0.201	0.115	3.78E-19	2.2	HMGN3	1.747826087
RP5-857K21.4	1.74E-23	0.5742415	0.501	0.431	4.19E-19	2.2	RP5-857K2	1.162412993
TMEM2582	2.02E-23	0.4481449	0.638	0.598	4.88E-19	2.2	TMEM258	1.066889632
RBM34	2.35E-23	0.4540956	0.487	0.403	5.66E-19	2.2	RBM3	1.208436725
PPP1R1B2	3.11E-23	0.2750115	0.082	0.031	7.50E-19	2.2	PPP1R1B	2.64516129
PEBP11	3.37E-23	0.4906344	0.26	0.169	8.12E-19	2.2	PEBP1	1.538461538
TMEM2301	3.45E-23	0.3616577	0.161	0.085	8.32E-19	2.2	TMEM230	1.894117647
MACF11	4.29E-23	0.4961893	0.732	0.721	1.03E-18	2.2	MACF1	1.015256588
LINC00467	4.50E-23	0.2431983	0.076	0.027	1.09E-18	2.2	LINC00467	2.814814815
DTWD21	4.84E-23	0.3005891	0.11	0.048	1.17E-18	2.2	DTWD2	2.291666667
NUDT12	5.04E-23	0.3381387	0.093	0.038	1.21E-18	2.2	NUDT12	2.447368421
KLF121	6.71E-23	0.5119854	0.235	0.143	1.62E-18	2.2	KLF12	1.643356643
WAC2	8.84E-23	0.4589453	0.65	0.628	2.13E-18	2.2	WAC	1.035031847
GABARAP1	9.04E-23	0.5029836	0.443	0.358	2.18E-18	2.2	GABARAP	1.237430168
SNX14	9.07E-23	0.4595086	0.248	0.157	2.19E-18	2.2	SNX1	1.579617834
GREB13	1.02E-22	0.28141	0.14	0.068	2.45E-18	2.2	GREB1	2.058823529
KIF16B1	1.67E-22	0.460759	0.274	0.182	4.02E-18	2.2	KIF16B	1.505494505

MT1A1	1.89E-22	0.4178629	0.052	0.015	4.56E-18	2.2	MT1A	3.466666667
GUSB1	1.96E-22	0.2805282	0.101	0.043	4.73E-18	2.2	GUSB	2.348837209
SERPINA111	2.08E-22	0.282563	0.083	0.032	5.01E-18	2.2	SERPINA11	2.59375
HNRNPA14	2.81E-22	0.1828539	0.833	0.754	6.77E-18	2.2	HNRNPA1	1.104774536
ATP6V1G11	2.89E-22	0.4855644	0.465	0.387	6.96E-18	2.2	ATP6V1G1	1.201550388
GFM1	2.92E-22	0.3228672	0.112	0.051	7.03E-18	2.2	GFM1	2.196078431
GSTM41	3.39E-22	0.1256344	0.034	0.008	8.17E-18	2.2	GSTM4	4.25
PDK41	3.51E-22	0.4758444	0.149	0.076	8.46E-18	2.2	PDK4	1.960526316
PAN31	3.55E-22	0.5220071	0.646	0.639	8.57E-18	2.2	PAN3	1.010954617
C18orf32	3.73E-22	0.4469644	0.241	0.15	8.98E-18	2.2	C18orf32	1.606666667
RAB182	5.62E-22	0.4862584	0.42	0.336	1.35E-17	2.2	RAB18	1.25
ANAPC163	5.97E-22	0.4880307	0.332	0.242	1.44E-17	2.2	ANAPC16	1.371900826
PNPLA4	7.17E-22	0.2016969	0.068	0.024	1.73E-17	2.2	PNPLA4	2.833333333
MEAF62	9.33E-22	0.4048915	0.257	0.164	2.25E-17	2.2	MEAF6	1.567073171
SLC50A1	1.14E-21	0.3018073	0.096	0.041	2.74E-17	2.2	SLC50A1	2.341463415
TMEM26	1.29E-21	0.1210939	0.032	0.007	3.12E-17	2.2	TMEM26	4.571428571
ERVK3-1	1.40E-21	0.2443024	0.084	0.033	3.38E-17	2.2	ERVK3-1	2.545454545
HERPUD12	1.44E-21	0.4872166	0.313	0.219	3.47E-17	2.2	HERPUD1	1.429223744
DLG5	1.44E-21	0.465318	0.361	0.258	3.48E-17	2.2	DLG5	1.399224806
SCOC2	1.49E-21	0.4873286	0.327	0.236	3.59E-17	2.2	SCOC	1.38559322
DYX1C1	1.55E-21	0.245178	0.067	0.024	3.75E-17	2.2	DYX1C1	2.791666667
RAB2A1	1.78E-21	0.5210404	0.391	0.309	4.29E-17	2.2	RAB2A	1.265372168
RPL26L11	1.96E-21	0.4881598	0.242	0.158	4.72E-17	2.2	RPL26L1	1.53164557
RORA	2.07E-21	0.4056047	0.644	0.57	5.00E-17	2.2	RORA	1.129824561
FNIP21	2.47E-21	0.4734092	0.184	0.104	5.95E-17	2.2	FNIP2	1.769230769
ATP6V1C11	3.22E-21	0.448207	0.211	0.129	7.75E-17	2.2	ATP6V1C1	1.635658915
FAM104B	3.25E-21	0.3158704	0.097	0.042	7.84E-17	2.2	FAM104B	2.30952381
PPM1K1	3.51E-21	0.4588451	0.233	0.146	8.47E-17	2.2	PPM1K	1.595890411
CD631	3.67E-21	0.4779891	0.471	0.378	8.85E-17	2.2	CD63	1.246031746
TBC1D10A1	3.74E-21	0.8937604	0.238	0.151	9.02E-17	2.2	TBC1D10A	1.57615894
HNMT	4.14E-21	0.4154544	0.387	0.291	9.98E-17	2.2	HNMT	1.329896907
RPL384	5.18E-21	0.2662882	0.902	0.891	1.25E-16	2.2	RPL38	1.012345679
RLF2	6.29E-21	0.5297472	0.428	0.343	1.52E-16	2.2	RLF	1.247813411
MYCBP1	7.48E-21	0.1925561	0.059	0.02	1.80E-16	2.2	MYCBP	2.95
CHD11	8.52E-21	0.5326959	0.355	0.266	2.05E-16	2.2	CHD1	1.334586466
DNAJC19	8.86E-21	0.2306289	0.071	0.026	2.14E-16	2.2	DNAJC19	2.730769231
GALNT71	9.07E-21	0.4806462	0.243	0.157	2.19E-16	2.2	GALNT7	1.547770701
KDM6A1	1.09E-20	0.6009523	0.438	0.364	2.63E-16	2.2	KDM6A	1.203296703
TMEM981	1.28E-20	0.1308623	0.034	0.008	3.07E-16	2.2	TMEM98	4.25
FOXA11	1.28E-20	0.4040278	0.196	0.115	3.09E-16	2.2	FOXA1	1.704347826
LAMA11	1.59E-20	0.5753024	0.18	0.101	3.83E-16	2.2	LAMA1	1.782178218
ALDH6A11	2.46E-20	0.2082264	0.071	0.027	5.92E-16	2.2	ALDH6A1	2.62962963
PUM22	3.35E-20	0.5200085	0.482	0.411	8.09E-16	2.2	PUM2	1.172749392
CAPN131	4.00E-20	0.2672598	0.086	0.036	9.64E-16	2.2	CAPN13	2.388888889
USP471	4.77E-20	0.4991318	0.49	0.419	1.15E-15	2.2	USP47	1.169451074
TAT-AS1	6.67E-20	0.1497211	0.034	0.008	1.61E-15	2.2	TAT-AS1	4.25
TMBIM61	6.90E-20	0.2733053	0.872	0.86	1.66E-15	2.2	TMBIM6	1.013953488
UFC11	9.11E-20	0.4371136	0.539	0.482	2.20E-15	2.2	UFC1	1.118257261
MEF2A2	1.08E-19	0.5349731	0.484	0.419	2.60E-15	2.2	MEF2A	1.155131265
DOCK8	1.15E-19	0.3098551	0.119	0.058	2.77E-15	2.2	DOCK8	2.051724138
HNRNPC2	1.52E-19	0.3004706	0.793	0.784	3.68E-15	2.2	HNRNPC	1.011479592
ECI2	2.21E-19	0.3725694	0.139	0.074	5.32E-15	2.2	ECI2	1.878378378

PTGIS	2.36E-19	0.2243821	0.057	0.019	5.68E-15	2.2	PTGIS	3
RHOH	2.37E-19	0.2691462	0.076	0.03	5.71E-15	2.2	RHOH	2.533333333
PPP6R31	2.38E-19	0.4897306	0.549	0.507	5.73E-15	2.2	PPP6R3	1.082840237
DNAH51	2.83E-19	0.4381198	0.128	0.065	6.83E-15	2.2	DNAH5	1.969230769
DICER11	3.54E-19	0.5274417	0.281	0.2	8.54E-15	2.2	DICER1	1.405
AC007682.1	4.94E-19	0.3100976	0.06	0.021	1.19E-14	2.2	AC007682.	2.857142857
SLC13A11	5.97E-19	0.1539788	0.044	0.013	1.44E-14	2.2	SLC13A1	3.384615385
ERH2	6.90E-19	0.496152	0.541	0.504	1.66E-14	2.2	ERH	1.073412698
TNC1	7.04E-19	0.3419013	0.434	0.327	1.70E-14	2.2	TNC	1.327217125
ANKRD30B1	7.09E-19	0.1871806	0.054	0.018	1.71E-14	2.2	ANKRD30B	3
RP11-539L10.31	7.90E-19	0.2229518	0.073	0.029	1.90E-14	2.2	RP11-539L1	2.517241379
RSPH1	8.74E-19	0.1292939	0.035	0.009	2.11E-14	2.2	RSPH1	3.888888889
FSIP2	8.78E-19	0.1716493	0.037	0.01	2.12E-14	2.2	FSIP2	3.7
SPOPL1	9.54E-19	0.4975424	0.341	0.257	2.30E-14	2.2	SPOPL	1.326848249
STAT5A1	1.53E-18	0.4346258	0.263	0.177	3.69E-14	2.2	STAT5A	1.485875706
C15orf65	1.60E-18	0.1533291	0.04	0.011	3.86E-14	2.2	C15orf65	3.636363636
TMEM101	1.72E-18	0.2001547	0.052	0.017	4.15E-14	2.2	TMEM101	3.058823529
HGD1	2.64E-18	0.1677914	0.048	0.015	6.37E-14	2.2	HGD	3.2
JAG11	4.25E-18	0.2806355	0.09	0.04	1.02E-13	2.2	JAG1	2.25
CAT1	5.70E-18	0.2754558	0.103	0.049	1.38E-13	2.2	CAT	2.102040816
COX201	5.84E-18	0.4032799	0.207	0.132	1.41E-13	2.2	COX20	1.568181818
GSPT11	6.06E-18	0.4038322	0.439	0.366	1.46E-13	2.2	GSPT1	1.199453552
RAP1GDS1	6.30E-18	0.4613279	0.196	0.122	1.52E-13	2.2	RAP1GDS1	1.606557377
UBE2G11	6.37E-18	0.4782324	0.335	0.253	1.54E-13	2.2	UBE2G1	1.324110672
APOA1BP1	6.99E-18	0.2129224	0.069	0.028	1.69E-13	2.2	APOA1BP	2.464285714
NOP10	7.83E-18	0.4288255	0.587	0.556	1.89E-13	2.2	NOP10	1.055755396
MARCKSL1	8.14E-18	0.3689594	0.228	0.149	1.96E-13	2.2	MARCKSL1	1.530201342
TGFB3	8.85E-18	0.1843402	0.083	0.035	2.13E-13	2.2	TGFB3	2.371428571
POLR3GL	9.60E-18	0.1407364	0.037	0.01	2.32E-13	2.2	POLR3GL	3.7
NBEAL11	1.15E-17	0.3034867	0.735	0.715	2.77E-13	2.2	NBEAL1	1.027972028
NAA151	1.22E-17	0.4276426	0.214	0.139	2.94E-13	2.2	NAA15	1.539568345
FKBP51	1.41E-17	0.4028635	0.421	0.332	3.39E-13	2.2	FKBP5	1.268072289
SEMA3E1	1.73E-17	0.4197557	0.215	0.139	4.17E-13	2.2	SEMA3E	1.54676259
IRF11	1.85E-17	0.4859333	0.263	0.182	4.46E-13	2.2	IRF1	1.445054945
MED131	2.11E-17	0.6094276	0.454	0.397	5.08E-13	2.2	MED13	1.143576826
SLC26A32	2.13E-17	0.2550017	0.832	0.787	5.15E-13	2.2	SLC26A3	1.057179161
WISP31	4.09E-17	0.1051963	0.027	0.006	9.86E-13	2.2	WISP3	4.5
TBX31	4.11E-17	0.3121361	0.366	0.266	9.90E-13	2.2	TBX3	1.37593985
ATP6AP21	4.58E-17	0.3480831	0.217	0.141	1.10E-12	2.2	ATP6AP2	1.539007092
HIPK21	4.68E-17	0.3894579	0.445	0.37	1.13E-12	2.2	HIPK2	1.202702703
MSRB2	4.98E-17	0.1412953	0.029	0.007	1.20E-12	2.2	MSRB2	4.142857143
ITGB3BP3	5.27E-17	0.3098954	0.139	0.076	1.27E-12	2.2	ITGB3BP	1.828947368
CPEB4	5.57E-17	0.4965491	0.335	0.258	1.34E-12	2.2	CPEB4	1.298449612
ZFP36	5.91E-17	0.4867835	0.186	0.114	1.43E-12	2.2	ZFP36	1.631578947
CD163L12	6.12E-17	0.2923507	0.11	0.056	1.48E-12	2.2	CD163L1	1.964285714
RWDD2B1	6.45E-17	0.1966919	0.058	0.022	1.56E-12	2.2	RWDD2B	2.636363636
AP2B11	9.12E-17	0.4094385	0.214	0.14	2.20E-12	2.2	AP2B1	1.528571429
RHOB	9.21E-17	0.4184077	0.178	0.108	2.22E-12	2.2	RHOB	1.648148148
MAPK101	9.94E-17	0.2846098	0.092	0.043	2.40E-12	2.2	MAPK10	2.139534884
MYO101	1.05E-16	0.5022715	0.327	0.25	2.53E-12	2.2	MYO10	1.308
NCSTN1	1.18E-16	0.2708851	0.103	0.051	2.84E-12	2.2	NCSTN	2.019607843
SDHC2	1.39E-16	0.3944737	0.233	0.16	3.35E-12	2.2	SDHC	1.45625

MRPS333	1.44E-16	0.4290629	0.313	0.237	3.47E-12	2.2	MRPS33	1.320675105
NOP581	1.48E-16	0.5382273	0.277	0.202	3.56E-12	2.2	NOP58	1.371287129
TWISTNB	1.48E-16	0.254761	0.075	0.032	3.56E-12	2.2	TWISTNB	2.34375
PDSS11	1.53E-16	0.3127659	0.086	0.039	3.70E-12	2.2	PDSS1	2.205128205
KCTD31	1.55E-16	0.4022194	0.27	0.191	3.75E-12	2.2	KCTD3	1.413612565
GATAD2A1	1.80E-16	0.4416747	0.223	0.15	4.35E-12	2.2	GATAD2A	1.486666667
PIGK	1.94E-16	0.3416554	0.142	0.08	4.67E-12	2.2	PIGK	1.775
HSPA91	2.03E-16	0.4146274	0.289	0.213	4.90E-12	2.2	HSPA9	1.356807512
DACH11	2.24E-16	0.3730546	0.161	0.094	5.41E-12	2.2	DACH1	1.712765957
NEDD92	2.25E-16	0.4379995	0.441	0.369	5.42E-12	2.2	NEDD9	1.195121951
DPY301	2.36E-16	0.4489255	0.248	0.177	5.70E-12	2.2	DPY30	1.401129944
STX181	2.71E-16	0.4197366	0.248	0.171	6.52E-12	2.2	STX18	1.450292398
BTBD71	2.94E-16	0.5010466	0.262	0.187	7.09E-12	2.2	BTBD7	1.401069519
DPCD	3.10E-16	0.2890091	0.143	0.081	7.47E-12	2.2	DPCD	1.765432099
IQUB	3.26E-16	0.1625411	0.04	0.012	7.87E-12	2.2	IQUB	3.333333333
TMEM144	3.38E-16	0.154288	0.047	0.016	8.14E-12	2.2	TMEM144	2.9375
TET1	3.50E-16	0.3045536	0.122	0.065	8.45E-12	2.2	TET1	1.876923077
TOP2B2	3.63E-16	0.445283	0.266	0.189	8.76E-12	2.2	TOP2B	1.407407407
BANF12	3.97E-16	0.3871759	0.252	0.177	9.57E-12	2.2	BANF1	1.423728814
IRS11	4.37E-16	0.3486251	0.157	0.092	1.05E-11	2.2	IRS1	1.706521739
IDH12	4.81E-16	0.3265924	0.139	0.078	1.16E-11	2.2	IDH1	1.782051282
TMEM237	5.06E-16	0.1286556	0.044	0.014	1.22E-11	2.2	TMEM237	3.142857143
RP11-84A19.42	5.26E-16	0.244849	0.104	0.052	1.27E-11	2.2	RP11-84A1	2
PIP4K2C1	6.62E-16	0.3077103	0.135	0.075	1.60E-11	2.2	PIP4K2C	1.8
EID1	6.64E-16	0.3246564	0.138	0.078	1.60E-11	2.2	EID1	1.769230769
PAWR1	6.92E-16	0.4683487	0.584	0.549	1.67E-11	2.2	PAWR	1.063752277
NXPE3	8.29E-16	0.1755055	0.048	0.017	2.00E-11	2.2	NXPE3	2.823529412
JUND1	8.45E-16	0.3904991	0.248	0.171	2.04E-11	2.2	JUND	1.450292398
DNAJB62	1.01E-15	0.3980464	0.445	0.38	2.43E-11	2.2	DNAJB6	1.171052632
ZYG11A1	1.11E-15	0.1836617	0.048	0.017	2.67E-11	2.2	ZYG11A	2.823529412
SELENBP1	1.12E-15	0.1565044	0.044	0.015	2.70E-11	2.2	SELENBP1	2.933333333
PUS7L1	1.21E-15	0.2179391	0.064	0.026	2.92E-11	2.2	PUS7L	2.461538462
NDUFA3	1.47E-15	0.1417976	0.044	0.015	3.54E-11	2.2	NDUFA3	2.933333333
SKP13	1.51E-15	0.2597481	0.814	0.807	3.63E-11	2.2	SKP1	1.008674102
KIF9	1.89E-15	0.4692832	0.132	0.073	4.56E-11	2.2	KIF9	1.808219178
EFNA11	2.03E-15	0.3922478	0.318	0.235	4.89E-11	2.2	EFNA1	1.353191489
GLRX51	2.03E-15	0.2138344	0.074	0.033	4.89E-11	2.2	GLRX5	2.242424242
CMTM61	2.05E-15	0.464719	0.371	0.3	4.93E-11	2.2	CMTM6	1.236666667
NOS1AP1	2.06E-15	0.5002531	0.289	0.214	4.96E-11	2.2	NOS1AP	1.35046729
DYNLRB21	2.13E-15	0.1106716	0.028	0.007	5.14E-11	2.2	DYNLRB2	4
TP53TG1	2.32E-15	0.1366705	0.041	0.013	5.61E-11	2.2	TP53TG1	3.153846154
EAF2	2.33E-15	0.1908884	0.051	0.019	5.61E-11	2.2	EAF2	2.684210526
IL6ST1	2.43E-15	0.4854209	0.392	0.331	5.86E-11	2.2	IL6ST	1.18429003
HDHD2	2.77E-15	0.1704236	0.051	0.019	6.67E-11	2.2	HDHD2	2.684210526
C14orf1662	3.26E-15	0.4048512	0.286	0.216	7.85E-11	2.2	C14orf166	1.324074074
FAM204A	3.46E-15	0.442936	0.291	0.218	8.33E-11	2.2	FAM204A	1.334862385
OSBPL81	3.55E-15	0.5074325	0.309	0.236	8.56E-11	2.2	OSBPL8	1.309322034
OSBPL1A1	3.74E-15	0.5118877	0.24	0.169	9.02E-11	2.2	OSBPL1A	1.420118343
AC104667.31	3.75E-15	0.159154	0.042	0.014	9.05E-11	2.2	AC104667.:	3
MEA11	4.10E-15	0.3610518	0.18	0.116	9.88E-11	2.2	MEA1	1.551724138
CHMP3	4.92E-15	0.2983825	0.135	0.077	1.19E-10	2.2	CHMP3	1.753246753
BCOR1	5.10E-15	0.4064939	0.571	0.51	1.23E-10	2.2	BCOR	1.119607843

PIGP2	5.78E-15	0.3698775	0.189	0.124	1.39E-10	2.2	PIGP	1.524193548
FMOD1	5.89E-15	0.1115694	0.028	0.007	1.42E-10	2.2	FMOD	4
CRYZ1	5.99E-15	0.2112184	0.068	0.029	1.44E-10	2.2	CRYZ	2.344827586
METTL7A1	6.04E-15	0.2574712	0.1	0.051	1.46E-10	2.2	METTL7A	1.960784314
RERE1	6.29E-15	0.3501003	0.61	0.569	1.52E-10	2.2	RERE	1.072056239
TSPAN151	6.32E-15	0.3283792	0.165	0.101	1.52E-10	2.2	TSPAN15	1.633663366
CST31	6.48E-15	0.259555	0.119	0.064	1.56E-10	2.2	CST3	1.859375
SYT16	7.10E-15	0.1127941	0.023	0.005	1.71E-10	2.2	SYT16	4.6
C20orf961	8.06E-15	0.1881199	0.081	0.038	1.94E-10	2.2	C20orf96	2.131578947
RAB211	8.33E-15	0.3768812	0.339	0.259	2.01E-10	2.2	RAB21	1.308880309
MUM1L11	1.08E-14	0.1012978	0.023	0.005	2.59E-10	2.2	MUM1L1	4.6
ATP2A2	1.10E-14	0.4846118	0.328	0.262	2.66E-10	2.2	ATP2A2	1.251908397
ARHGEF371	1.19E-14	0.2908907	0.098	0.05	2.88E-10	2.2	ARHGEF37	1.96
TMSB101	1.24E-14	0.1507821	0.761	0.732	3.00E-10	2.2	TMSB10	1.039617486
ATF32	1.30E-14	0.4292767	0.467	0.404	3.13E-10	2.2	ATF3	1.155940594
ARL6IP52	1.44E-14	0.4119732	0.526	0.487	3.48E-10	2.2	ARL6IP5	1.080082136
RP11-60I3.41	1.61E-14	0.1627117	0.058	0.023	3.88E-10	2.2	RP11-60I3.4	2.52173913
CLDN82	1.72E-14	0.2805397	0.104	0.055	4.14E-10	2.2	CLDN8	1.890909091
CTC-338M12.52	1.77E-14	0.1087783	0.026	0.006	4.27E-10	2.2	CTC-338M1	4.333333333
POLE2	1.93E-14	0.4338591	0.306	0.235	4.66E-10	2.2	POLE2	1.30212766
ACSF2	2.32E-14	0.2348129	0.076	0.035	5.60E-10	2.2	ACSF2	2.171428571
MRPS35	2.38E-14	0.3011386	0.118	0.066	5.73E-10	2.2	MRPS35	1.787878788
H3F3B1	2.42E-14	0.3133573	0.459	0.39	5.82E-10	2.2	H3F3B	1.176923077
TNFRSF10B1	2.42E-14	0.4435725	0.273	0.202	5.84E-10	2.2	TNFRSF10E	1.351485149
AHSA2	2.44E-14	0.2366897	0.068	0.03	5.89E-10	2.2	AHSA2	2.266666667
ARPC31	3.00E-14	0.3275506	0.672	0.662	7.25E-10	2.2	ARPC3	1.01510574
EFHC1	3.14E-14	0.2897076	0.118	0.066	7.58E-10	2.2	EFHC1	1.787878788
MOCS22	3.25E-14	0.2926	0.118	0.065	7.84E-10	2.2	MOCS2	1.815384615
GPD1L	3.27E-14	0.1283008	0.033	0.01	7.89E-10	2.2	GPD1L	3.3
RNF101	4.05E-14	0.3711092	0.292	0.223	9.77E-10	2.2	RNF10	1.30941704
GMDS1	4.36E-14	0.4721289	0.504	0.457	1.05E-09	2.2	GMDS	1.102844639
TRA2B1	4.40E-14	0.4260872	0.278	0.211	1.06E-09	2.2	TRA2B	1.317535545
SNRPF4	4.40E-14	0.4364009	0.257	0.189	1.06E-09	2.2	SNRPF	1.35978836
STT3B1	4.48E-14	0.4034124	0.249	0.181	1.08E-09	2.2	STT3B	1.375690608
SORD1	5.55E-14	0.1937208	0.07	0.032	1.34E-09	2.2	SORD	2.1875
TFF11	5.82E-14	0.3975275	0.17	0.103	1.40E-09	2.2	TFF1	1.650485437
SLC25A39	7.19E-14	0.1606065	0.044	0.016	1.73E-09	2.2	SLC25A39	2.75
CPNE3	8.39E-14	0.3794629	0.251	0.183	2.02E-09	2.2	CPNE3	1.371584699
COX7B2	8.50E-14	0.3025013	0.652	0.633	2.05E-09	2.2	COX7B	1.030015798
ALAD	8.61E-14	0.123713	0.037	0.012	2.08E-09	2.2	ALAD	3.083333333
FGFR1OP	1.04E-13	0.2577489	0.102	0.054	2.51E-09	2.2	FGFR1OP	1.888888889
UBN21	1.13E-13	0.4223345	0.341	0.271	2.73E-09	2.2	UBN2	1.258302583
GSTO22	1.14E-13	0.3173319	0.151	0.093	2.75E-09	2.2	GSTO2	1.623655914
TTC6	1.16E-13	0.4594118	0.24	0.172	2.80E-09	2.2	TTC6	1.395348837
TMEM591	1.33E-13	0.3674204	0.497	0.452	3.21E-09	2.2	TMEM59	1.099557522
NOSTRIN	1.39E-13	0.2206206	0.072	0.033	3.36E-09	2.2	NOSTRIN	2.181818182
MAGED2	1.40E-13	0.1945187	0.062	0.026	3.37E-09	2.2	MAGED2	2.384615385
C2CD4A1	1.44E-13	0.1996564	0.092	0.046	3.48E-09	2.2	C2CD4A	2
NIPBL1	1.56E-13	0.5213287	0.553	0.549	3.76E-09	2.2	NIPBL	1.007285974
STIP11	1.63E-13	0.3221917	0.107	0.058	3.93E-09	2.2	STIP1	1.844827586
GNA151	1.70E-13	0.1997353	0.064	0.028	4.09E-09	2.2	GNA15	2.285714286
GADD45B1	1.76E-13	0.4226951	0.17	0.107	4.25E-09	2.2	GADD45B	1.588785047

EIF2AK11	1.82E-13	0.5414819	0.171	0.111	4.38E-09	2.2	EIF2AK1	1.540540541
CDH15	1.92E-13	0.2761769	0.642	0.605	4.62E-09	2.2	CDH1	1.061157025
ZMAT11	2.38E-13	0.1299152	0.034	0.011	5.74E-09	2.2	ZMAT1	3.090909091
ANKRD171	2.44E-13	0.462728	0.532	0.509	5.88E-09	2.2	ANKRD17	1.04518664
LRRFIP23	2.60E-13	0.217698	0.769	0.763	6.26E-09	2.2	LRRFIP2	1.007863696
SOD1	2.97E-13	0.3925389	0.18	0.119	7.16E-09	2.2	SOD1	1.512605042
HELB2	3.14E-13	0.3925664	0.157	0.1	7.58E-09	2.2	HELB	1.57
AMIGO21	3.18E-13	0.1764683	0.055	0.022	7.67E-09	2.2	AMIGO2	2.5
NFKBIA2	3.51E-13	0.4097772	0.644	0.628	8.47E-09	2.2	NFKBIA	1.025477707
RBFOX23	3.86E-13	0.2498916	0.951	0.952	9.31E-09	2.2	RBFOX2	0.99894958
TFF31	3.91E-13	0.2541249	0.106	0.057	9.42E-09	2.2	TFF3	1.859649123
TPM13	4.58E-13	0.2722805	0.575	0.524	1.10E-08	2.2	TPM1	1.097328244
PKIB1	4.63E-13	0.2686605	0.145	0.088	1.12E-08	2.2	PKIB	1.647727273
PFDN12	4.84E-13	0.4162758	0.282	0.219	1.17E-08	2.2	PFDN1	1.287671233
PPME11	5.04E-13	0.3368094	0.133	0.079	1.22E-08	2.2	PPME1	1.683544304
BATF1	5.40E-13	0.3214889	0.124	0.072	1.30E-08	2.2	BATF	1.722222222
EGR11	5.43E-13	0.3400564	0.125	0.073	1.31E-08	2.2	EGR1	1.712328767
CD2AP3	5.52E-13	0.4005296	0.473	0.427	1.33E-08	2.2	CD2AP	1.107728337
PER1	5.95E-13	0.2473676	0.076	0.037	1.43E-08	2.2	PER1	2.054054054
EIF5AL11	6.00E-13	0.2531538	0.089	0.046	1.45E-08	2.2	EIF5AL1	1.934782609
SIDT1	6.30E-13	0.2597356	0.083	0.041	1.52E-08	2.2	SIDT1	2.024390244
ADAMTS15	6.53E-13	0.1479936	0.027	0.007	1.57E-08	2.2	ADAMTS15	3.857142857
PSD31	6.59E-13	0.4384744	0.412	0.344	1.59E-08	2.2	PSD3	1.197674419
ABCC12	7.28E-13	0.319907	0.16	0.101	1.76E-08	2.2	ABCC1	1.584158416
HNRNPU1	7.67E-13	0.3763333	0.179	0.12	1.85E-08	2.2	HNRNPU	1.491666667
MALRD11	7.68E-13	0.2622258	0.086	0.043	1.85E-08	2.2	MALRD1	2
GTF3C61	7.97E-13	0.2456634	0.106	0.058	1.92E-08	2.2	GTF3C6	1.827586207
RP11-295K2.31	8.66E-13	0.1450745	0.041	0.015	2.09E-08	2.2	RP11-295K	2.733333333
ADD31	8.75E-13	0.3749086	0.26	0.193	2.11E-08	2.2	ADD3	1.347150259
ALDH3B21	9.19E-13	0.1076259	0.032	0.01	2.22E-08	2.2	ALDH3B2	3.2
TPT12	9.38E-13	0.1172713	0.867	0.851	2.26E-08	2.2	TPT1	1.01880141
NUDT24	1.03E-12	0.332837	0.181	0.122	2.49E-08	2.2	NUDT2	1.483606557
JUNB	1.04E-12	0.4601032	0.078	0.038	2.51E-08	2.2	JUNB	2.052631579
SPCS11	1.07E-12	0.3083796	0.139	0.084	2.58E-08	2.2	SPCS1	1.654761905
RP5-1101C3.11	1.15E-12	0.2972346	0.104	0.057	2.78E-08	2.2	RP5-1101C	1.824561404
ACADSB1	1.22E-12	0.292786	0.275	0.2	2.94E-08	2.2	ACADSB	1.375
TGM31	1.37E-12	0.2543443	0.054	0.022	3.30E-08	2.2	TGM3	2.454545455
TBC1D30	1.50E-12	0.2397561	0.076	0.037	3.61E-08	2.2	TBC1D30	2.054054054
HMGCR	1.55E-12	0.3060534	0.143	0.088	3.75E-08	2.2	HMGCR	1.625
MPP71	1.58E-12	0.3606669	0.174	0.114	3.80E-08	2.2	MPP7	1.526315789
TSPYL5	1.71E-12	0.1223536	0.035	0.012	4.12E-08	2.2	TSPYL5	2.916666667
SMARCA21	1.78E-12	0.3911264	0.365	0.308	4.28E-08	2.2	SMARCA2	1.185064935
RPL375	1.93E-12	0.1806671	0.947	0.946	4.65E-08	2.2	RPL37	1.001057082
OSBP1	2.01E-12	0.335586	0.214	0.15	4.84E-08	2.2	OSBP	1.426666667
GTF3A1	2.02E-12	0.2440015	0.102	0.056	4.88E-08	2.2	GTF3A	1.821428571
NAMPT	2.15E-12	0.2541942	0.693	0.647	5.17E-08	2.2	NAMPT	1.071097372
DEPDC1B1	2.24E-12	0.1316394	0.034	0.011	5.40E-08	2.2	DEPDC1B	3.090909091
PLAT1	2.35E-12	0.2916817	0.14	0.085	5.66E-08	2.2	PLAT	1.647058824
OCIAD12	2.59E-12	0.4019173	0.313	0.255	6.24E-08	2.2	OCIAD1	1.22745098
BRDT1	2.69E-12	0.1993931	0.07	0.033	6.49E-08	2.2	BRDT	2.121212121
IGF1R1	3.05E-12	0.4462708	0.522	0.504	7.36E-08	2.2	IGF1R	1.035714286
GJC31	3.06E-12	0.2925347	0.147	0.091	7.38E-08	2.2	GJC3	1.615384615

RPS114	3.07E-12	0.1209703	0.818	0.768	7.40E-08	2.2	RPS11	1.065104167
RP1-78O14.11	3.26E-12	0.3812148	0.275	0.201	7.85E-08	2.2	RP1-78O14	1.368159204
GSTZ1	3.26E-12	0.1201319	0.03	0.01	7.86E-08	2.2	GSTZ1	3
IPO73	3.33E-12	0.4612025	0.311	0.25	8.03E-08	2.2	IPO7	1.244
RGL21	3.48E-12	0.2462473	0.081	0.041	8.40E-08	2.2	RGL2	1.975609756
TMEM991	3.53E-12	0.2724108	0.123	0.073	8.52E-08	2.2	TMEM99	1.684931507
BEX4	3.54E-12	0.1713622	0.052	0.022	8.54E-08	2.2	BEX4	2.363636364
IGFBP21	3.66E-12	0.2462318	0.09	0.048	8.82E-08	2.2	IGFBP2	1.875
BCL61	3.74E-12	0.5392625	0.401	0.356	9.01E-08	2.2	BCL6	1.126404494
TMEM254-AS11	3.96E-12	0.1420388	0.053	0.022	9.56E-08	2.2	TMEM254-	2.409090909
TCEANC2	4.29E-12	0.3590855	0.306	0.236	1.03E-07	2.2	TCEANC2	1.296610169
COPRS	4.34E-12	0.1240793	0.033	0.011	1.05E-07	2.2	COPRS	3
RPL10A4	4.45E-12	0.1691464	0.763	0.715	1.07E-07	2.2	RPL10A	1.067132867
PSORS1C11	5.14E-12	0.2553571	0.091	0.049	1.24E-07	2.2	PSORS1C1	1.857142857
COX17	5.32E-12	0.255289	0.108	0.061	1.28E-07	2.2	COX17	1.770491803
MLEC1	6.17E-12	0.2637073	0.13	0.079	1.49E-07	2.2	MLEC	1.64556962
NRXN11	6.68E-12	0.2636668	0.08	0.04	1.61E-07	2.2	NRXN1	2
HIBCH2	6.81E-12	0.4116054	0.164	0.109	1.64E-07	2.2	HIBCH	1.504587156
ITGAV1	6.82E-12	0.2723456	0.473	0.397	1.64E-07	2.2	ITGAV	1.191435768
RASGEF1B2	7.08E-12	0.238999	0.871	0.893	1.71E-07	2.2	RASGEF1B	0.975363942
AF127936.91	7.21E-12	0.3559857	0.206	0.143	1.74E-07	2.2	AF127936.9	1.440559441
LRRIQ1	7.27E-12	0.1301288	0.036	0.013	1.75E-07	2.2	LRRIQ1	2.769230769
TMEM14A1	7.67E-12	0.2002978	0.103	0.057	1.85E-07	2.2	TMEM14A	1.807017544
ATRAID	8.25E-12	0.2326728	0.089	0.047	1.99E-07	2.2	ATRAID	1.893617021
PROM21	8.31E-12	0.2310882	0.09	0.048	2.00E-07	2.2	PROM2	1.875
TMEM241	8.99E-12	0.2814371	0.137	0.086	2.17E-07	2.2	TMEM241	1.593023256
EIF3A1	9.51E-12	0.3703896	0.383	0.326	2.29E-07	2.2	EIF3A	1.174846626
IKZF5	9.71E-12	0.20153	0.083	0.043	2.34E-07	2.2	IKZF5	1.930232558
IMMP1L1	1.16E-11	0.3010666	0.197	0.136	2.80E-07	2.2	IMMP1L	1.448529412
RP11-108M9.41	1.21E-11	0.1690663	0.059	0.027	2.93E-07	2.2	RP11-108M	2.185185185
DYNC2LI11	1.33E-11	0.318264	0.138	0.087	3.21E-07	2.2	DYNC2LI1	1.586206897
BAG11	1.46E-11	0.2979671	0.15	0.097	3.53E-07	2.2	BAG1	1.546391753
PLA2G2A1	1.50E-11	0.1022272	0.03	0.01	3.61E-07	2.2	PLA2G2A	3
SPDEF	1.52E-11	0.1013441	0.025	0.007	3.66E-07	2.2	SPDEF	3.571428571
CSRNP1	1.56E-11	0.2562692	0.084	0.044	3.75E-07	2.2	CSRNP1	1.909090909
SRSF34	1.60E-11	0.2037744	0.495	0.429	3.87E-07	2.2	SRSF3	1.153846154
SRP14-AS1	1.62E-11	0.1044328	0.03	0.01	3.91E-07	2.2	SRP14-AS1	3
LYRM51	1.82E-11	0.3295394	0.161	0.107	4.39E-07	2.2	LYRM5	1.504672897
OTUD7B1	1.90E-11	0.367418	0.354	0.292	4.58E-07	2.2	OTUD7B	1.212328767
RAB3D	1.97E-11	0.2103889	0.065	0.032	4.76E-07	2.2	RAB3D	2.03125
QKI2	1.98E-11	0.4040846	0.551	0.541	4.78E-07	2.2	QKI	1.018484288
TRIO3	2.05E-11	0.2679617	0.822	0.809	4.95E-07	2.2	TRIO	1.016069221
EWSR11	2.32E-11	0.3468982	0.176	0.121	5.59E-07	2.2	EWSR1	1.454545455
HNRNPD1	2.42E-11	0.3628778	0.336	0.281	5.83E-07	2.2	HNRNPD	1.195729537
ABCA121	2.71E-11	0.1862139	0.051	0.022	6.53E-07	2.2	ABCA12	2.318181818
REL1	2.96E-11	0.3628921	0.356	0.298	7.14E-07	2.2	REL	1.194630872
CUL31	3.21E-11	0.4067358	0.443	0.402	7.75E-07	2.2	CUL3	1.10199005
SURF11	3.37E-11	0.1004311	0.034	0.012	8.13E-07	2.2	SURF1	2.833333333
RYBP1	3.42E-11	0.3707204	0.239	0.178	8.25E-07	2.2	RYBP	1.342696629
CCDC63	3.64E-11	0.4312385	0.367	0.316	8.77E-07	2.2	CCDC6	1.161392405
C3orf521	3.76E-11	0.311243	0.222	0.159	9.08E-07	2.2	C3orf52	1.396226415
H2AFV1	3.95E-11	0.2831124	0.139	0.088	9.51E-07	2.2	H2AFV	1.579545455

RAB30-AS12	4.11E-11	0.260458	0.122	0.075	9.91E-07	2.2	RAB30-AS1	1.626666667
PPT1	4.40E-11	0.1889019	0.074	0.038	1.06E-06	2.2	PPT1	1.947368421
RP11-35G9.5	4.50E-11	0.3507215	0.147	0.095	1.08E-06	2.2	RP11-35G9	1.547368421
BTG2	4.51E-11	0.3730353	0.103	0.06	1.09E-06	2.2	BTG2	1.716666667
RPS6KA31	4.69E-11	0.4596019	0.489	0.464	1.13E-06	2.2	RPS6KA3	1.05387931
TAF9B1	4.81E-11	0.1169204	0.038	0.015	1.16E-06	2.2	TAF9B	2.533333333
TMEM126A3	4.95E-11	0.2914169	0.146	0.095	1.19E-06	2.2	TMEM126A	1.536842105
DIXDC11	5.77E-11	0.5579152	0.169	0.114	1.39E-06	2.2	DIXDC1	1.48245614
STYK11	5.83E-11	0.268952	0.11	0.065	1.41E-06	2.2	STYK1	1.692307692
ENPP51	6.41E-11	0.1931191	0.066	0.033	1.55E-06	2.2	ENPP5	2
DNAJC27-AS1	6.41E-11	0.1787379	0.054	0.024	1.55E-06	2.2	DNAJC27-A	2.25
LSM53	6.51E-11	0.3959626	0.39	0.345	1.57E-06	2.2	LSM5	1.130434783
COPZ12	6.70E-11	0.3489345	0.371	0.32	1.62E-06	2.2	COPZ1	1.159375
KIAA0825	6.98E-11	0.3106637	0.143	0.093	1.68E-06	2.2	KIAA0825	1.537634409
PPP2CB2	7.03E-11	0.3927728	0.387	0.338	1.69E-06	2.2	PPP2CB	1.144970414
LAMTOR2	7.29E-11	0.2119657	0.081	0.043	1.76E-06	2.2	LAMTOR2	1.88372093
DHX151	7.76E-11	0.3397477	0.316	0.259	1.87E-06	2.2	DHX15	1.22007722
CHN1	8.00E-11	0.2124884	0.104	0.06	1.93E-06	2.2	CHN1	1.733333333
RP11-481C4.11	9.06E-11	0.2391615	0.104	0.061	2.18E-06	2.2	RP11-481C	1.704918033
HSPD14	9.67E-11	0.496168	0.302	0.251	2.33E-06	2.2	HSPD1	1.203187251
RALY-AS1	1.01E-10	0.1053473	0.029	0.01	2.44E-06	2.2	RALY-AS1	2.9
APEX12	1.04E-10	0.3270188	0.251	0.192	2.52E-06	2.2	APEX1	1.307291667
CTNND11	1.05E-10	0.37467	0.435	0.395	2.53E-06	2.2	CTNND1	1.101265823
RPL37A5	1.08E-10	0.1921767	0.975	0.975	2.60E-06	2.2	RPL37A	1
PTPN20	1.08E-10	0.107437	0.03	0.01	2.60E-06	2.2	PTPN20	3
ARHGEF261	1.18E-10	0.3453336	0.154	0.102	2.84E-06	2.2	ARHGEF26	1.509803922
TNS1	1.18E-10	0.2057773	0.081	0.043	2.85E-06	2.2	TNS1	1.88372093
S100A16	1.23E-10	0.2876117	0.212	0.153	2.96E-06	2.2	S100A16	1.385620915
USP153	1.38E-10	0.4476389	0.375	0.33	3.32E-06	2.2	USP15	1.136363636
NPIPB51	1.38E-10	0.2801117	0.136	0.087	3.32E-06	2.2	NPIPB5	1.563218391
LRRC591	1.46E-10	0.214024	0.112	0.067	3.53E-06	2.2	LRRC59	1.671641791
PIN42	1.64E-10	0.4283111	0.317	0.265	3.96E-06	2.2	PIN4	1.196226415
IFT222	1.70E-10	0.1912684	0.082	0.044	4.10E-06	2.2	IFT22	1.863636364
ISOC1	1.71E-10	0.176208	0.057	0.027	4.12E-06	2.2	ISOC1	2.111111111
SUCLG1	1.74E-10	0.242894	0.115	0.07	4.18E-06	2.2	SUCLG1	1.642857143
MED41	1.83E-10	0.3247985	0.21	0.154	4.41E-06	2.2	MED4	1.363636364
CTD-2369P2.51	2.17E-10	0.1771185	0.057	0.027	5.22E-06	2.2	CTD-2369P	2.111111111
VWA8	2.19E-10	0.398731	0.149	0.099	5.28E-06	2.2	VWA8	1.505050505
GNPNAT1	2.19E-10	0.1489838	0.057	0.027	5.29E-06	2.2	GNPNAT1	2.111111111
CTC1	2.29E-10	0.4597735	0.102	0.06	5.53E-06	2.2	CTC1	1.7
SUMO13	2.36E-10	0.2806155	0.528	0.496	5.70E-06	2.2	SUMO1	1.064516129
RANBP91	2.38E-10	0.3756221	0.35	0.297	5.74E-06	2.2	RANBP9	1.178451178
MTMR41	2.38E-10	0.2152866	0.085	0.047	5.74E-06	2.2	MTMR4	1.808510638
ARHGAP40	2.38E-10	0.1321395	0.038	0.015	5.75E-06	2.2	ARHGAP40	2.533333333
ARG21	2.41E-10	0.2948567	0.061	0.125	5.82E-06	2.2	ARG2	0.488
TMEM179B	2.50E-10	0.2000058	0.065	0.033	6.03E-06	2.2	TMEM179B	1.96969697
DNMBP-AS1	2.53E-10	0.1017896	0.025	0.008	6.10E-06	2.2	DNMBP-AS	3.125
FLNB2	2.56E-10	0.2958305	0.5	0.456	6.18E-06	2.2	FLNB	1.096491228
PGRMC11	2.63E-10	0.2882656	0.13	0.083	6.34E-06	2.2	PGRMC1	1.56626506
HMGN1	2.66E-10	0.3849744	0.231	0.178	6.42E-06	2.2	HMGN1	1.297752809
COL4A6	2.66E-10	0.2532103	0.102	0.06	6.42E-06	2.2	COL4A6	1.7
CHMP5	2.70E-10	0.313219	0.475	0.436	6.52E-06	2.2	CHMP5	1.089449541

C1orf123	2.85E-10	0.1664135	0.052	0.024	6.87E-06	2.2	C1orf123	2.166666667
EFS	2.85E-10	0.1356516	0.038	0.015	6.88E-06	2.2	EFS	2.533333333
DUSP161	3.03E-10	0.3284037	0.468	0.42	7.30E-06	2.2	DUSP16	1.114285714
RABEP1	3.04E-10	0.3911214	0.212	0.158	7.32E-06	2.2	RABEP1	1.341772152
MT-ND52	3.05E-10	0.3162451	0.832	0.854	7.35E-06	2.2	MT-ND5	0.974238876
EIF4E3	3.25E-10	0.2980119	0.429	0.38	7.83E-06	2.2	EIF4E	1.128947368
ASH1L1	3.26E-10	0.3725366	0.559	0.552	7.85E-06	2.2	ASH1L	1.012681159
KCNMA11	3.33E-10	0.1353384	0.209	0.146	8.02E-06	2.2	KCNMA1	1.431506849
SIAH21	3.42E-10	0.2491465	0.121	0.075	8.24E-06	2.2	SIAH2	1.613333333
AFTPH2	3.48E-10	0.3866891	0.36	0.313	8.38E-06	2.2	AFTPH	1.150159744
NUCB21	3.56E-10	0.3098736	0.18	0.127	8.59E-06	2.2	NUCB2	1.417322835
ARFGEF21	3.79E-10	0.4367165	0.408	0.372	9.13E-06	2.2	ARFGEF2	1.096774194
HSP90AA13	3.83E-10	0.2154337	0.882	0.853	9.25E-06	2.2	HSP90AA1	1.033997655
PPIC	4.06E-10	0.1905407	0.074	0.039	9.80E-06	2.2	PPIC	1.897435897
COX161	4.20E-10	0.1223452	0.034	0.013	1.01E-05	2.2	COX16	2.615384615
DUT1	4.67E-10	0.1765438	0.062	0.031	1.13E-05	2.2	DUT	2
CREB3L1	4.85E-10	0.171936	0.053	0.025	1.17E-05	2.2	CREB3L1	2.12
RANBP101	4.95E-10	0.2987935	0.13	0.083	1.19E-05	2.2	RANBP10	1.56626506
NDE1	5.25E-10	0.2472229	0.083	0.046	1.27E-05	2.2	NDE1	1.804347826
SSBP21	5.71E-10	0.3210003	0.486	0.435	1.38E-05	2.2	SSBP2	1.117241379
COA3	5.90E-10	0.1473929	0.051	0.023	1.42E-05	2.2	COA3	2.217391304
DHX9	5.93E-10	0.3149002	0.251	0.195	1.43E-05	2.2	DHX9	1.287179487
RPA3	5.98E-10	0.3206652	0.189	0.138	1.44E-05	2.2	RPA3	1.369565217
RETSAT	6.51E-10	0.1601678	0.054	0.025	1.57E-05	2.2	RETSAT	2.16
SLC10A6	6.85E-10	0.2153479	0.052	0.024	1.65E-05	2.2	SLC10A6	2.166666667
TMEM126B2	8.27E-10	0.293147	0.154	0.105	1.99E-05	2.2	TMEM126E	1.466666667
CANX1	8.36E-10	0.351174	0.38	0.338	2.02E-05	2.2	CANX	1.124260355
EIF4G21	8.47E-10	0.2988054	0.433	0.391	2.04E-05	2.2	EIF4G2	1.10741688
EIF3H4	8.77E-10	0.234105	0.642	0.625	2.12E-05	2.2	EIF3H	1.0272
LAMP11	8.86E-10	0.297456	0.143	0.095	2.14E-05	2.2	LAMP1	1.505263158
VIPR1	8.89E-10	0.1605692	0.049	0.022	2.14E-05	2.2	VIPR1	2.227272727
PMP221	8.90E-10	0.1476495	0.048	0.022	2.15E-05	2.2	PMP22	2.181818182
PLEKHF2	9.29E-10	0.3334245	0.148	0.1	2.24E-05	2.2	PLEKHF2	1.48
IMPACT1	9.30E-10	0.1579389	0.051	0.024	2.24E-05	2.2	IMPACT	2.125
RP11-49I11.11	9.38E-10	0.1929796	0.072	0.038	2.26E-05	2.2	RP11-49I11	1.894736842
IRF2BP21	9.39E-10	0.3796159	0.181	0.13	2.27E-05	2.2	IRF2BP2	1.392307692
BANK11	9.93E-10	0.1259768	0.057	0.027	2.39E-05	2.2	BANK1	2.111111111
GLOD4	1.10E-09	0.2988097	0.148	0.101	2.65E-05	2.2	GLOD4	1.465346535
MYEOV2	1.12E-09	0.2183494	0.088	0.05	2.70E-05	2.2	MYEOV2	1.76
YWHAB	1.24E-09	0.3628062	0.359	0.314	2.98E-05	2.2	YWHAB	1.143312102
GANAB	1.26E-09	0.295937	0.161	0.113	3.03E-05	2.2	GANAB	1.424778761
ATP7A	1.27E-09	0.2060322	0.082	0.046	3.07E-05	2.2	ATP7A	1.782608696
EIF4A31	1.30E-09	0.4313676	0.142	0.096	3.13E-05	2.2	EIF4A3	1.479166667
RP11-420K8.11	1.38E-09	0.1189224	0.042	0.018	3.33E-05	2.2	RP11-420K	2.333333333
RP11-166B2.81	1.43E-09	0.2326994	0.102	0.061	3.45E-05	2.2	RP11-166B	1.672131148
NFIA	1.44E-09	0.3396768	0.245	0.188	3.47E-05	2.2	NFIA	1.303191489
KLHDC101	1.48E-09	0.3324624	0.186	0.135	3.58E-05	2.2	KLHDC10	1.377777778
CCNL11	1.49E-09	0.3468583	0.443	0.411	3.60E-05	2.2	CCNL1	1.077858881
NOC3L	1.50E-09	0.1535194	0.051	0.024	3.62E-05	2.2	NOC3L	2.125
LAMTOR1	1.54E-09	0.2048858	0.072	0.039	3.72E-05	2.2	LAMTOR1	1.846153846
LRTOMT1	1.61E-09	0.180863	0.05	0.023	3.88E-05	2.2	LRTOMT	2.173913043
AMZ21	1.73E-09	0.1557531	0.059	0.03	4.18E-05	2.2	AMZ2	1.966666667

PTGES31	1.80E-09	0.4264263	0.289	0.242	4.35E-05	2.2	PTGES3	1.194214876
LINC001601	1.95E-09	0.1290034	0.036	0.014	4.71E-05	2.2	LINC00160	2.571428571
OLFM21	2.09E-09	0.2396281	0.058	0.029	5.05E-05	2.2	OLFM2	2
PYURF1	2.52E-09	0.193278	0.076	0.042	6.08E-05	2.2	PYURF	1.80952381
NEU31	2.53E-09	0.1606827	0.052	0.025	6.10E-05	2.2	NEU3	2.08
CDYL2	2.54E-09	0.1882154	0.065	0.034	6.13E-05	2.2	CDYL2	1.911764706
DNAJC24	2.62E-09	0.2374122	0.077	0.043	6.32E-05	2.2	DNAJC24	1.790697674
RP11-977G19.11	2.65E-09	0.110474	0.034	0.013	6.39E-05	2.2	RP11-977G	2.615384615
ABCC111	2.86E-09	0.1672537	0.034	0.014	6.90E-05	2.2	ABCC11	2.428571429
CLSTN21	3.05E-09	0.1917844	0.112	0.069	7.36E-05	2.2	CLSTN2	1.623188406
AP1M2	3.15E-09	0.2389243	0.12	0.077	7.60E-05	2.2	AP1M2	1.558441558
PCYOX1	3.16E-09	0.2699198	0.083	0.047	7.61E-05	2.2	PCYOX1	1.765957447
KLHL42	3.36E-09	0.2823264	0.112	0.071	8.11E-05	2.2	KLHL42	1.577464789
IBTK1	3.43E-09	0.4261175	0.27	0.22	8.26E-05	2.2	IBTK	1.227272727
ADAM281	3.50E-09	0.1122411	0.043	0.019	8.44E-05	2.2	ADAM28	2.263157895
NUCKS11	3.62E-09	0.3338087	0.341	0.296	8.74E-05	2.2	NUCKS1	1.152027027
RPH3AL	3.75E-09	0.1759065	0.043	0.019	9.05E-05	2.2	RPH3AL	2.263157895
CGNL11	3.79E-09	0.3715581	0.241	0.189	9.15E-05	2.2	CGNL1	1.275132275
FAM155A	4.06E-09	0.2380803	0.096	0.057	9.78E-05	2.2	FAM155A	1.684210526
ACSS3	4.22E-09	0.1475979	0.044	0.02	0.000101816	2.2	ACSS3	2.2
ATP5F13	4.32E-09	0.3067229	0.412	0.372	0.000104097	2.2	ATP5F1	1.107526882
CCPG1	4.38E-09	0.2739656	0.141	0.096	0.000105719	2.2	CCPG1	1.46875
TJP11	4.43E-09	0.4206913	0.516	0.509	0.000106727	2.2	TJP1	1.013752456
PRPF40A	5.10E-09	0.3467048	0.222	0.172	0.000123044	2.2	PRPF40A	1.290697674
NGFRAP12	5.13E-09	0.1873914	0.076	0.042	0.000123742	2.2	NGFRAP1	1.80952381
SNRPE4	5.23E-09	0.3266077	0.538	0.521	0.000125999	2.2	SNRPE	1.032629559
AP1S31	5.24E-09	0.2814347	0.171	0.121	0.000126365	2.2	AP1S3	1.41322314
FAM188A1	5.34E-09	0.2936618	0.145	0.098	0.000128809	2.2	FAM188A	1.479591837
BAZ1A1	5.62E-09	0.5500082	0.408	0.385	0.000135578	2.2	BAZ1A	1.05974026
TP53I3	7.02E-09	0.1250574	0.034	0.014	0.000169209	2.2	TP53I3	2.428571429
USP9X1	7.43E-09	0.3990319	0.432	0.406	0.000179231	2.2	USP9X	1.064039409
ENTPD7	7.95E-09	0.1094016	0.031	0.012	0.000191716	2.2	ENTPD7	2.583333333
CYB5R1	8.08E-09	0.1733839	0.059	0.031	0.00019487	2.2	CYB5R1	1.903225806
IER51	8.55E-09	0.3080391	0.158	0.111	0.00020621	2.2	IER5	1.423423423
SF3B63	9.07E-09	0.2591587	0.607	0.609	0.000218662	2.2	SF3B6	0.996715928
AHCY	9.22E-09	0.2533818	0.094	0.058	0.000222205	2.2	AHCY	1.620689655
POLR2G1	9.86E-09	0.3588489	0.219	0.172	0.000237694	2.2	POLR2G	1.273255814
PSMG3	9.92E-09	0.2390778	0.04	0.018	0.00023916	2.2	PSMG3	2.222222222
THSD4-AS12	1.00E-08	0.3258865	0.485	0.457	0.000241418	2.2	THSD4-AS1	1.061269147
PXYLP1	1.02E-08	0.1682983	0.057	0.029	0.000247098	2.2	PXYLP1	1.965517241
TIMMDC11	1.06E-08	0.2093263	0.09	0.054	0.000254504	2.2	TIMMDC1	1.666666667
TCEAL1	1.22E-08	0.1705163	0.051	0.025	0.000294882	2.2	TCEAL1	2.04
KIZ-AS14	1.24E-08	0.1418452	0.841	0.844	0.000298415	2.2	KIZ-AS1	0.996445498
TMEM25	1.25E-08	0.1003259	0.033	0.013	0.000301597	2.2	TMEM25	2.538461538
PDCL3	1.27E-08	0.1659954	0.061	0.032	0.000306236	2.2	PDCL3	1.90625
HEBP1	1.34E-08	0.2405751	0.103	0.065	0.000322075	2.2	HEBP1	1.584615385
TSPAN6	1.58E-08	0.3351197	0.223	0.176	0.000382063	2.2	TSPAN6	1.267045455
ETFA	1.71E-08	0.296541	0.176	0.129	0.000413038	2.2	ETFA	1.364341085
STX8	1.73E-08	0.3494168	0.277	0.23	0.000418353	2.2	STX8	1.204347826
METAP1D1	1.99E-08	0.111695	0.039	0.017	0.000480405	2.2	METAP1D	2.294117647
RGMB-AS11	2.05E-08	0.1075173	0.031	0.013	0.000493715	2.2	RGMB-AS1	2.384615385
PDE11A	2.11E-08	0.2051118	0.063	0.034	0.000508393	2.2	PDE11A	1.852941176

ZFAND51	2.13E-08	0.3193637	0.461	0.435	0.000514038	2.2	ZFAND5	1.059770115
AGA1	2.15E-08	0.1640691	0.06	0.032	0.000518622	2.2	AGA	1.875
HRSP122	2.16E-08	0.2267534	0.109	0.07	0.000521693	2.2	HRSP12	1.557142857
GGCT2	2.30E-08	0.2113836	0.09	0.054	0.000554526	2.2	GGCT	1.666666667
LLNLR-245B6.1	2.34E-08	0.2105853	0.099	0.062	0.000564091	2.2	LLNLR-245I	1.596774194
CHMP2A	2.44E-08	0.2414153	0.133	0.09	0.000587647	2.2	CHMP2A	1.477777778
SERTAD41	2.60E-08	0.1002272	0.026	0.009	0.000627223	2.2	SERTAD4	2.888888889
DCAF51	2.61E-08	0.3755263	0.19	0.145	0.000628326	2.2	DCAF5	1.310344828
KNOP12	2.62E-08	0.381914	0.19	0.144	0.000632117	2.2	KNOP1	1.319444444
RSRC22	2.72E-08	0.2209847	0.618	0.626	0.000655408	2.2	RSRC2	0.987220447
LGMN1	2.74E-08	0.177871	0.084	0.05	0.00066138	2.2	LGMN	1.68
CCNG21	2.82E-08	0.2956254	0.261	0.21	0.000680239	2.2	CCNG2	1.242857143
RP11-347P5.11	2.91E-08	0.1880153	0.077	0.044	0.00070079	2.2	RP11-347P.	1.75
DCP1A1	2.91E-08	0.3672328	0.304	0.262	0.00070106	2.2	DCP1A	1.160305344
INTS61	3.07E-08	0.364163	0.33	0.281	0.000740576	2.2	INTS6	1.174377224
DNALI11	3.23E-08	0.1015835	0.038	0.017	0.000779411	2.2	DNALI1	2.235294118
LAMTOR52	3.29E-08	0.2809767	0.593	0.587	0.00079335	2.2	LAMTOR5	1.010221465
DHX30	3.33E-08	0.2262263	0.103	0.065	0.000802299	2.2	DHX30	1.584615385
RP4-633O19--A.12	3.35E-08	0.1510829	0.041	0.019	0.000808899	2.2	RP4-633O1	2.157894737
MAP9	3.36E-08	0.1439977	0.049	0.024	0.000810586	2.2	MAP9	2.041666667
PKIA1	3.45E-08	0.1200396	0.033	0.014	0.000831504	2.2	PKIA	2.357142857
PDIA62	3.54E-08	0.3366378	0.276	0.229	0.000852458	2.2	PDIA6	1.205240175
ANKRD121	3.92E-08	0.3012118	0.66	0.675	0.000945554	2.2	ANKRD12	0.977777778
PCCB	3.94E-08	0.1406906	0.058	0.031	0.000950898	2.2	PCCB	1.870967742
RAD211	4.06E-08	0.3435932	0.369	0.335	0.000979219	2.2	RAD21	1.101492537
ARL5A1	4.22E-08	0.2701727	0.146	0.103	0.001018473	2.2	ARL5A	1.417475728
EDEM31	4.30E-08	0.2560369	0.129	0.087	0.001037835	2.2	EDEM3	1.482758621
FAM229B	4.34E-08	0.2168098	0.098	0.062	0.001045512	2.2	FAM229B	1.580645161
SERP12	4.64E-08	0.3420765	0.292	0.252	0.001118043	2.2	SERP1	1.158730159
APH1A	4.91E-08	0.1864221	0.065	0.036	0.00118447	2.2	APH1A	1.805555556
COPA1	4.99E-08	0.3769139	0.352	0.317	0.001203769	2.2	COPA	1.110410095
XRN21	5.01E-08	0.2592521	0.256	0.207	0.001208597	2.2	XRN2	1.236714976
PHKG11	5.02E-08	0.2485778	0.104	0.066	0.001210661	2.2	PHKG1	1.575757576
BECN1	5.24E-08	0.2307053	0.106	0.069	0.00126445	2.2	BECN1	1.536231884
ANKH2	5.36E-08	0.2415318	0.129	0.088	0.001292136	2.2	ANKH	1.465909091
CCDC252	5.58E-08	0.2880758	0.132	0.092	0.001346073	2.2	CCDC25	1.434782609
PDZD111	5.68E-08	0.2392903	0.128	0.087	0.00137072	2.2	PDZD11	1.471264368
SMARCC11	5.92E-08	0.3341611	0.332	0.289	0.001428026	2.2	SMARCC1	1.148788927
PSMC51	6.13E-08	0.2679849	0.136	0.095	0.001479105	2.2	PSMC5	1.431578947
FSIP11	6.14E-08	0.1861045	0.085	0.051	0.001481591	2.2	FSIP1	1.666666667
RRAGC1	6.29E-08	0.2141696	0.115	0.076	0.00151681	2.2	RRAGC	1.513157895
SELT	6.89E-08	0.2309249	0.115	0.076	0.00166019	2.2	SELT	1.513157895
AHSA1	6.97E-08	0.1647265	0.063	0.035	0.001679892	2.2	AHSA1	1.8
AF127577.111	7.02E-08	0.1868492	0.077	0.045	0.001692717	2.2	AF127577.:	1.711111111
S100A134	7.13E-08	0.2681376	0.536	0.513	0.001718406	2.2	S100A13	1.044834308
ACAA2	7.39E-08	0.1795948	0.062	0.034	0.001782193	2.2	ACAA2	1.823529412
ZBTB411	7.46E-08	0.1945445	0.076	0.044	0.001799316	2.2	ZBTB41	1.727272727
PAFAH2	7.62E-08	0.1217326	0.043	0.02	0.001836701	2.2	PAFAH2	2.15
CALM3	7.65E-08	0.1225777	0.046	0.023	0.001845317	2.2	CALM3	2
RAPH11	7.73E-08	0.3965503	0.371	0.336	0.00186468	2.2	RAPH1	1.104166667
SPAG92	8.04E-08	0.4154888	0.375	0.345	0.00193814	2.2	SPAG9	1.086956522
NR6A11	8.69E-08	0.4915637	0.387	0.351	0.002094744	2.2	NR6A1	1.102564103

TM9SF32	9.57E-08	0.334084	0.347	0.31	0.002308704	2.2	TM9SF3	1.119354839
LNX21	1.00E-07	0.3131009	0.191	0.146	0.002415727	2.2	LNX2	1.308219178
FAM111A2	1.04E-07	0.1775354	0.058	0.031	0.002498883	2.2	FAM111A	1.870967742
CCDC342	1.05E-07	0.1119136	0.044	0.021	0.002543145	2.2	CCDC34	2.095238095
EBAG9	1.06E-07	0.14904	0.063	0.035	0.002560351	2.2	EBAG9	1.8
USP71	1.10E-07	0.2639792	0.134	0.093	0.002664218	2.2	USP7	1.440860215
VDAC1	1.18E-07	0.2895773	0.255	0.211	0.002839883	2.2	VDAC1	1.208530806
PAK4	1.18E-07	0.2173993	0.074	0.044	0.002846805	2.2	PAK4	1.681818182
GDPD41	1.25E-07	0.3523734	0.027	0.01	0.003002537	2.2	GDPD4	2.7
CUX2	1.27E-07	0.1833839	0.058	0.031	0.003070196	2.2	CUX2	1.870967742
SIAE1	1.28E-07	0.1916014	0.076	0.045	0.003088242	2.2	SIAE	1.688888889
MDK	1.29E-07	0.1358859	0.057	0.031	0.003107563	2.2	MDK	1.838709677
FAM174B	1.36E-07	0.2239397	0.109	0.072	0.003286776	2.2	FAM174B	1.513888889
SYK	1.37E-07	0.1033386	0.034	0.015	0.003300101	2.2	SYK	2.266666667
C8orf594	1.40E-07	0.3393849	0.323	0.285	0.003365945	2.2	C8orf59	1.133333333
ENAH2	1.42E-07	0.4192269	0.549	0.555	0.003436016	2.2	ENAH	0.989189189
ADIRF1	1.43E-07	0.2707043	0.104	0.066	0.00344008	2.2	ADIRF	1.575757576
ERP29	1.45E-07	0.2626082	0.166	0.122	0.003491664	2.2	ERP29	1.360655738
SLC38A11	1.46E-07	0.2977506	0.426	0.393	0.00353178	2.2	SLC38A1	1.083969466
CDC422	1.50E-07	0.2053606	0.705	0.718	0.003620397	2.2	CDC42	0.98189415
RPS10-NUDT31	1.51E-07	0.1788461	0.077	0.046	0.003643337	2.2	RPS10-NUI	1.673913043
EI241	1.56E-07	0.2524189	0.115	0.077	0.003764896	2.2	EI24	1.493506494
H2AFY1	1.56E-07	0.3188813	0.19	0.147	0.003766935	2.2	H2AFY	1.292517007
VPS251	1.65E-07	0.274675	0.18	0.137	0.00397576	2.2	VPS25	1.313868613
STK17B1	1.69E-07	0.3122421	0.203	0.158	0.004065414	2.2	STK17B	1.284810127
TMEM2541	1.81E-07	0.185263	0.072	0.043	0.00435529	2.2	TMEM254	1.674418605
TMEM41B1	1.81E-07	0.3332995	0.291	0.248	0.004358327	2.2	TMEM41B	1.173387097
PHLDB21	1.87E-07	0.3479506	0.453	0.419	0.004508469	2.2	PHLDB2	1.081145585
ZFC3H11	1.87E-07	0.3601772	0.298	0.258	0.004517793	2.2	ZFC3H1	1.15503876
DYNLL2	1.98E-07	0.1597712	0.066	0.038	0.004784151	2.2	DYNLL2	1.736842105
DPH62	1.99E-07	0.2527703	0.1	0.065	0.004804596	2.2	DPH6	1.538461538
PCSK6	2.01E-07	0.2134155	0.069	0.04	0.004844655	2.2	PCSK6	1.725
CDC272	2.29E-07	0.3692025	0.302	0.262	0.005528546	2.2	CDC27	1.152671756
ZNF101	2.57E-07	0.1072452	0.037	0.017	0.006199529	2.2	ZNF101	2.176470588
PCLO1	2.57E-07	0.1839136	0.082	0.05	0.006208576	2.2	PCLO	1.64
KMT2E1	2.58E-07	0.5563074	0.461	0.468	0.006218874	2.2	KMT2E	0.985042735
PEMT1	2.58E-07	0.3585534	0.066	0.038	0.006226444	2.2	PEMT	1.736842105
BMP2K1	2.73E-07	0.2354432	0.14	0.099	0.006581999	2.2	BMP2K	1.414141414
STX31	2.89E-07	0.277684	0.182	0.138	0.006967387	2.2	STX3	1.31884058
USP241	3.01E-07	0.3087307	0.212	0.169	0.007258848	2.2	USP24	1.25443787
PEAK11	3.01E-07	0.473696	0.306	0.266	0.007267929	2.2	PEAK1	1.15037594
HMGCLL1	3.02E-07	0.1467691	0.07	0.041	0.007271916	2.2	HMGCLL1	1.707317073
BRIX11	3.02E-07	0.2715234	0.15	0.108	0.007278751	2.2	BRIX1	1.388888889
SNU131	3.05E-07	0.2814674	0.494	0.477	0.007362062	2.2	SNU13	1.035639413
FAM213A	3.32E-07	0.302381	0.16	0.119	0.008011921	2.2	FAM213A	1.344537815
LINC00578	3.41E-07	0.5600703	0.11	0.073	0.008219862	2.2	LINC00578	1.506849315
ATP5B	3.49E-07	0.2966077	0.268	0.227	0.008412126	2.2	ATP5B	1.18061674
GRAP2	3.54E-07	0.1127876	0.035	0.016	0.008544719	2.2	GRAP2	2.1875
QSOX11	3.60E-07	0.2780766	0.196	0.151	0.008670028	2.2	QSOX1	1.298013245
CHD4	3.66E-07	0.2685425	0.171	0.129	0.008823733	2.2	CHD4	1.325581395
TMEM147	3.84E-07	0.1391592	0.046	0.024	0.0092608	2.2	TMEM147	1.916666667
DNAJA41	4.21E-07	0.1488073	0.049	0.026	0.01014832	2.2	DNAJA4	1.884615385

TSPAN312	4.28E-07	0.2186283	0.085	0.054	0.01032155	2.2	TSPAN31	1.574074074
KRT18	4.33E-07	0.2342646	0.149	0.108	0.01043207	2.2	KRT18	1.37962963
NCEH11	4.35E-07	0.3225301	0.449	0.413	0.01048301	2.2	NCEH1	1.08716707
PACSIN2	4.49E-07	0.3152176	0.316	0.274	0.01083123	2.2	PACSIN2	1.153284672
TBC1D12	4.59E-07	0.2330664	0.115	0.078	0.01107209	2.2	TBC1D12	1.474358974
MAP2K41	4.74E-07	0.2703482	0.47	0.443	0.01142213	2.2	MAP2K4	1.060948081
ILF3	4.85E-07	0.1796581	0.09	0.057	0.01168807	2.2	ILF3	1.578947368
TRA2A1	4.85E-07	0.3398622	0.422	0.401	0.01170082	2.2	TRA2A	1.052369077
POLE4	5.22E-07	0.1211486	0.04	0.019	0.01258877	2.2	POLE4	2.105263158
RPS184	5.65E-07	0.16135	0.652	0.635	0.0136329	2.2	RPS18	1.026771654
HIPK11	5.97E-07	0.2643882	0.175	0.133	0.01439364	2.2	HIPK1	1.315789474
DRC3	6.09E-07	0.1819926	0.061	0.035	0.01467533	2.2	DRC3	1.742857143
DGKD1	6.42E-07	0.2680719	0.165	0.123	0.01547965	2.2	DGKD	1.341463415
EPT11	6.91E-07	0.2269415	0.133	0.095	0.01665608	2.2	EPT1	1.4
SRPRB1	6.93E-07	0.1539333	0.064	0.037	0.01671158	2.2	SRPRB	1.72972973
LINC014202	6.96E-07	0.291648	0.402	0.375	0.01679278	2.2	LINC01420	1.072
RAB27B1	7.08E-07	0.1947666	0.199	0.151	0.01707773	2.2	RAB27B	1.317880795
BRK14	7.30E-07	0.2550622	0.599	0.608	0.01759355	2.2	BRK1	0.985197368
RARA1	7.44E-07	0.1519415	0.041	0.021	0.01793881	2.2	RARA	1.952380952
NSA24	7.55E-07	0.2771961	0.376	0.345	0.01820704	2.2	NSA2	1.089855072
GATSL21	7.72E-07	0.1544626	0.065	0.038	0.01862491	2.2	GATSL2	1.710526316
TCEB3-AS1	7.81E-07	0.1399322	0.041	0.021	0.01883265	2.2	TCEB3-AS1	1.952380952
ACSM31	7.83E-07	0.1979781	0.072	0.043	0.01887135	2.2	ACSM3	1.674418605
PDHX1	7.90E-07	0.2264407	0.109	0.075	0.01905464	2.2	PDHX	1.453333333
GPR39	7.99E-07	0.122166	0.035	0.017	0.01926053	2.2	GPR39	2.058823529
VAPA2	8.47E-07	0.25602	0.482	0.475	0.02042079	2.2	VAPA	1.014736842
ZBTB181	9.03E-07	0.2563406	0.106	0.072	0.02177799	2.2	ZBTB18	1.472222222
THYN12	9.28E-07	0.1510296	0.06	0.035	0.02237146	2.2	THYN1	1.714285714
VBP11	9.69E-07	0.2798102	0.181	0.141	0.02335603	2.2	VBP1	1.283687943
MAP3K191	9.72E-07	0.1072084	0.043	0.022	0.02343839	2.2	MAP3K19	1.954545455
KMT2A2	9.72E-07	0.3539078	0.433	0.421	0.02344784	2.2	KMT2A	1.028503563
ZMPSTE24	9.74E-07	0.2348192	0.107	0.073	0.02349249	2.2	ZMPSTE24	1.465753425
GABPB11	1.03E-06	0.2953466	0.157	0.117	0.02479387	2.2	GABPB1	1.341880342
MRPL37	1.03E-06	0.157883	0.078	0.048	0.02488614	2.2	MRPL3	1.625
NHSL22	1.04E-06	0.2968594	0.314	0.278	0.02505861	2.2	NHSL2	1.129496403
MIEN1	1.05E-06	0.1972518	0.089	0.057	0.02540827	2.2	MIEN1	1.561403509
NET11	1.06E-06	0.3848051	0.229	0.19	0.02556437	2.2	NET1	1.205263158
HIVEP21	1.06E-06	0.3975711	0.465	0.451	0.02566609	2.2	HIVEP2	1.031042129
EPCAM	1.18E-06	0.2862189	0.252	0.212	0.02855069	2.2	EPCAM	1.188679245
SREK1	1.18E-06	0.2938641	0.203	0.164	0.02856342	2.2	SREK1	1.237804878
ERGIC32	1.20E-06	0.2564132	0.359	0.324	0.02894635	2.2	ERGIC3	1.108024691
NAALADL2	1.25E-06	0.2349081	0.54	0.502	0.03008615	2.2	NAALADL2	1.075697211
COX7A2L4	1.41E-06	0.2566129	0.511	0.499	0.03393768	2.2	COX7A2L	1.024048096
ATF22	1.41E-06	0.235621	0.18	0.139	0.03397981	2.2	ATF2	1.294964029
EMC3	1.43E-06	0.3055374	0.275	0.236	0.03441495	2.2	EMC3	1.165254237
USP251	1.44E-06	0.3880216	0.295	0.259	0.03472988	2.2	USP25	1.138996139
JTB1	1.49E-06	0.1961012	0.08	0.05	0.03594221	2.2	JTB	1.6
CTD-2349B8.11	1.52E-06	0.2997443	0.03	0.014	0.03663996	2.2	CTD-2349B	2.142857143
HINT13	1.53E-06	0.1928753	0.615	0.602	0.0370019	2.2	HINT1	1.021594684
CASZ11	1.56E-06	0.2389688	0.133	0.096	0.03766365	2.2	CASZ1	1.385416667
ARMCX32	1.58E-06	0.277207	0.164	0.126	0.03798812	2.2	ARMCX3	1.301587302
VSIG2	1.58E-06	0.1034094	0.024	0.01	0.03805486	2.2	VSIG2	2.4

USP541	1.63E-06	0.286642	0.51	0.496	0.0392926	2.2	USP54	1.028225806
WDR35	1.63E-06	0.1427323	0.046	0.024	0.03940674	2.2	WDR35	1.916666667
MRPL132	1.70E-06	0.2825536	0.204	0.166	0.04092257	2.2	MRPL13	1.228915663
ELMO21	1.70E-06	0.2761737	0.13	0.094	0.0410605	2.2	ELMO2	1.382978723
INTS122	1.71E-06	0.283043	0.249	0.209	0.04118378	2.2	INTS12	1.19138756
LYPD6	1.71E-06	0.1910469	0.08	0.051	0.04133562	2.2	LYPD6	1.568627451
NDUFB51	1.74E-06	0.1855773	0.07	0.043	0.04194799	2.2	NDUFB5	1.627906977
NENF	1.74E-06	0.208753	0.1	0.067	0.04195705	2.2	NENF	1.492537313
INTU	1.74E-06	0.1852871	0.082	0.052	0.04206821	2.2	INTU	1.576923077
ADI1	1.89E-06	0.1117641	0.041	0.021	0.04553999	2.2	ADI1	1.952380952
MTRNR2L122	1.90E-06	0.4407833	0.259	0.223	0.04570152	2.2	MTRNR2L1	1.161434978
CPE1	1.93E-06	0.1578804	0.254	0.203	0.04645804	2.2	CPE	1.251231527
GSTCD1	1.94E-06	0.2371758	0.147	0.108	0.04686752	2.2	GSTCD	1.361111111
GMNN1	1.95E-06	0.1137498	0.037	0.018	0.04703036	2.2	GMNN	2.055555556
FABP61	1.96E-06	0.6807932	0.063	0.037	0.04715043	2.2	FABP6	1.702702703
SPRED12	2.02E-06	0.2253689	0.114	0.08	0.04862009	2.2	SPRED1	1.425
CTSZ1	2.02E-06	0.1927455	0.096	0.064	0.0487802	2.2	CTSZ	1.5
ATP5A1	2.03E-06	0.3149292	0.225	0.188	0.04906044	2.2	ATP5A1	1.196808511
PDE4C	0	1.700709	0.355	0.013	0	2.3	PDE4C	27.30769231
BRIP11	0	1.59251	0.388	0.033	0	2.3	BRIP1	11.75757576
THEMIS	0	1.433846	0.257	0.004	0	2.3	THEMIS	64.25
PPP1R36	0	1.1843	0.271	0.009	0	2.3	PPP1R36	30.11111111
SNX312	0	1.158716	0.313	0.027	0	2.3	SNX31	11.59259259
SERPINE31	0	0.9810794	0.24	0.013	0	2.3	SERPINE3	18.46153846
AC019117.11	0	0.9363484	0.208	0.01	0	2.3	AC019117.	20.8
KIAA0319	0	0.8524833	0.191	0.003	0	2.3	KIAA0319	63.66666667
RP11-838N2.5	0	0.693588	0.139	0.004	0	2.3	RP11-838N	34.75
DNAH121	2.53E-285	1.109763	0.232	0.018	6.10E-281	2.3	DNAH12	12.88888889
GLRA11	1.41E-283	0.797412	0.195	0.012	3.39E-279	2.3	GLRA1	16.25
TRIM72	6.32E-277	0.5200048	0.115	0.003	1.52E-272	2.3	TRIM72	38.33333333
BMPR1B2	2.08E-274	1.760554	0.362	0.049	5.00E-270	2.3	BMPR1B	7.387755102
SEMA4A	1.32E-266	0.9805392	0.235	0.02	3.19E-262	2.3	SEMA4A	11.75
GSTM32	1.03E-259	2.310478	0.802	0.313	2.49E-255	2.3	GSTM3	2.562300319
LRRC231	4.90E-229	1.509332	0.412	0.076	1.18E-224	2.3	LRRC23	5.421052632
CXCL132	5.13E-226	2.237112	0.881	0.397	1.24E-221	2.3	CXCL13	2.219143577
IL19	2.18E-225	0.3865265	0.086	0.002	5.25E-221	2.3	IL19	43
AGR22	3.92E-221	1.779388	0.695	0.229	9.44E-217	2.3	AGR2	3.034934498
C3orf581	1.33E-212	1.004788	0.256	0.031	3.20E-208	2.3	C3orf58	8.258064516
MANBA1	1.74E-210	1.128195	0.341	0.055	4.19E-206	2.3	MANBA	6.2
PTGER31	2.09E-209	1.153409	0.263	0.033	5.05E-205	2.3	PTGER3	7.96969697
COX6C2	4.55E-206	1.621179	0.954	0.757	1.10E-201	2.3	COX6C	1.260237781
GUCY2C1	2.27E-202	0.5273996	0.117	0.005	5.48E-198	2.3	GUCY2C	23.4
NOP2	1.25E-199	0.919281	0.221	0.024	3.02E-195	2.3	NOP2	9.208333333
RP11-215A19.21	2.76E-198	0.3807727	0.09	0.003	6.65E-194	2.3	RP11-215A	30
RAB261	9.42E-197	1.020379	0.243	0.03	2.27E-192	2.3	RAB26	8.1
RAB44	1.79E-191	0.4190113	0.09	0.003	4.32E-187	2.3	RAB44	30
MLIP1	2.43E-187	0.7458298	0.149	0.011	5.86E-183	2.3	MLIP	13.54545455
CPED11	2.58E-182	0.6387163	0.152	0.012	6.21E-178	2.3	CPED1	12.66666667
RNASEH2A	6.20E-182	0.5294947	0.112	0.006	1.50E-177	2.3	RNASEH2A	18.66666667
AMPD3	4.42E-179	0.9458427	0.237	0.031	1.06E-174	2.3	AMPD3	7.64516129
DNAJC122	6.80E-177	1.471731	0.812	0.378	1.64E-172	2.3	DNAJC12	2.148148148
DNAJB14	2.79E-172	1.396849	0.445	0.116	6.73E-168	2.3	DNAJB14	3.836206897

MAP7D22	4.46E-172	0.9541408	0.267	0.041	1.08E-167	2.3	MAP7D2	6.512195122
C8orf42	3.66E-171	1.603616	0.909	0.507	8.82E-167	2.3	C8orf4	1.792899408
C5orf47	4.63E-171	0.3555634	0.073	0.002	1.12E-166	2.3	C5orf47	36.5
ANKRD30A2	1.06E-167	1.169413	0.92	0.458	2.55E-163	2.3	ANKRD30A	2.008733624
LLfos-48D6.2	3.13E-162	0.6405589	0.177	0.019	7.54E-158	2.3	LLfos-48D6	9.315789474
AREG2	2.29E-161	1.397413	0.861	0.438	5.52E-157	2.3	AREG	1.965753425
RP11-342D14.1	1.13E-160	0.3169915	0.069	0.002	2.72E-156	2.3	RP11-342D	34.5
RYBP2	1.08E-158	1.402273	0.521	0.169	2.61E-154	2.3	RYBP	3.082840237
INTS21	1.36E-156	0.8651931	0.247	0.038	3.29E-152	2.3	INTS2	6.5
LINC01004	4.50E-156	1.166345	0.333	0.07	1.08E-151	2.3	LINC01004	4.757142857
PRX	9.50E-155	0.3843071	0.069	0.002	2.29E-150	2.3	PRX	34.5
GPR75-ASB31	6.41E-151	1.439405	0.494	0.157	1.55E-146	2.3	GPR75-ASB	3.146496815
AGR32	5.83E-149	1.572717	0.577	0.206	1.41E-144	2.3	AGR3	2.800970874
HEXIM1	5.35E-146	1.176485	0.301	0.061	1.29E-141	2.3	HEXIM1	4.93442623
TPH21	7.08E-143	0.5927313	0.157	0.017	1.71E-138	2.3	TPH2	9.235294118
RP11-863K10.2	3.48E-141	0.2311424	0.042	0	8.38E-137	2.3	RP11-863K	#DIV/0!
INTS62	5.60E-141	1.317405	0.642	0.271	1.35E-136	2.3	INTS6	2.36900369
RP11-110L15.2	1.36E-140	0.3380525	0.069	0.002	3.27E-136	2.3	RP11-110L1	34.5
HOMER12	2.24E-140	1.214677	0.399	0.106	5.39E-136	2.3	HOMER1	3.764150943
ACOT121	3.62E-139	0.501513	0.107	0.007	8.74E-135	2.3	ACOT12	15.28571429
ELOVL52	1.99E-137	1.337235	0.657	0.284	4.80E-133	2.3	ELOVL5	2.313380282
SDC22	3.97E-137	1.051569	0.324	0.072	9.57E-133	2.3	SDC2	4.5
PTHLH2	2.11E-136	1.348186	0.597	0.225	5.08E-132	2.3	PTHLH	2.653333333
RP11-554D13.11	8.55E-135	0.5782514	0.149	0.016	2.06E-130	2.3	RP11-554D	9.3125
NME71	3.59E-134	1.388272	0.449	0.138	8.66E-130	2.3	NME7	3.253623188
FXYD32	1.42E-132	1.409986	0.872	0.626	3.41E-128	2.3	FXYD3	1.392971246
SH3BGRL4	4.89E-132	1.246476	0.743	0.391	1.18E-127	2.3	SH3BGRL	1.900255754
HMP19	1.09E-131	0.4149695	0.074	0.003	2.63E-127	2.3	HMP19	24.66666667
AZGP12	4.48E-130	1.181189	0.869	0.538	1.08E-125	2.3	AZGP1	1.615241636
SERP21	8.62E-130	0.6764402	0.167	0.021	2.08E-125	2.3	SERP2	7.952380952
DIO22	4.90E-129	1.797863	0.566	0.213	1.18E-124	2.3	DIO2	2.657276995
TNFSF111	8.72E-129	1.024455	0.275	0.055	2.10E-124	2.3	TNFSF11	5
RP11-114G22.11	1.09E-126	0.5805929	0.152	0.018	2.62E-122	2.3	RP11-114G	8.444444444
GRIK41	2.21E-124	0.675472	0.129	0.013	5.33E-120	2.3	GRIK4	9.923076923
EZH21	4.24E-124	0.9619224	0.289	0.063	1.02E-119	2.3	EZH2	4.587301587
ASB31	9.99E-123	0.7040181	0.169	0.023	2.41E-118	2.3	ASB3	7.347826087
TRHDE1	1.06E-122	0.3812723	0.079	0.004	2.56E-118	2.3	TRHDE	19.75
ATF33	2.70E-122	1.34731	0.726	0.396	6.52E-118	2.3	ATF3	1.833333333
RP11-1017G21.4	5.46E-122	0.3776174	0.09	0.006	1.32E-117	2.3	RP11-1017	15
SLIT22	9.82E-120	0.9324601	0.208	0.035	2.37E-115	2.3	SLIT2	5.942857143
THBS11	1.42E-119	1.13382	0.303	0.072	3.42E-115	2.3	THBS1	4.208333333
ZSWIM62	1.43E-118	1.265175	0.784	0.489	3.45E-114	2.3	ZSWIM6	1.603271984
CCDC1411	3.60E-117	0.2770917	0.063	0.003	8.68E-113	2.3	CCDC141	21
NDUFAF1	8.83E-117	0.9306055	0.285	0.065	2.13E-112	2.3	NDUFAF1	4.384615385
COL21A11	1.63E-116	0.6947476	0.125	0.013	3.94E-112	2.3	COL21A1	9.615384615
CCDC1811	3.91E-116	0.5403041	0.125	0.013	9.42E-112	2.3	CCDC181	9.615384615
BTBD2	1.43E-115	0.6280558	0.135	0.015	3.44E-111	2.3	BTBD2	9
UGDH2	2.09E-115	1.189376	0.563	0.226	5.04E-111	2.3	UGDH	2.491150442
HEXIM2	1.10E-114	0.3341223	0.063	0.003	2.66E-110	2.3	HEXIM2	21
TPRG11	1.65E-113	1.37589	0.371	0.11	3.99E-109	2.3	TPRG1	3.372727273
EFCAB8	2.58E-113	0.3041055	0.069	0.003	6.21E-109	2.3	EFCAB8	23
SLC7A82	3.97E-113	0.7564425	0.213	0.038	9.58E-109	2.3	SLC7A8	5.605263158

TMEM247	5.39E-112	0.1669643	0.031	0	1.30E-107	2.3	TMEM247	#DIV/0!
SLC39A62	1.51E-111	1.148642	0.633	0.292	3.65E-107	2.3	SLC39A6	2.167808219
TANGO6	4.06E-111	1.004493	0.27	0.06	9.80E-107	2.3	TANGO6	4.5
BICC11	1.60E-110	0.8689254	0.19	0.031	3.85E-106	2.3	BICC1	6.129032258
CTB-50L17.16	3.78E-110	0.9507231	0.103	0.009	9.12E-106	2.3	CTB-50L17.	11.44444444
ASAH21	4.03E-110	0.2479237	0.066	0.003	9.73E-106	2.3	ASAH2	22
MYBPC12	5.30E-110	1.094077	0.563	0.222	1.28E-105	2.3	MYBPC1	2.536036036
KIAA1683	3.77E-109	0.6624966	0.164	0.024	9.10E-105	2.3	KIAA1683	6.833333333
CTD-2033A16.32	3.09E-108	0.5480585	0.152	0.021	7.44E-104	2.3	CTD-2033A	7.238095238
RP1-78O14.12	2.12E-107	1.063453	0.521	0.194	5.10E-103	2.3	RP1-78O14	2.68556701
NR4A12	5.14E-107	0.9494228	0.206	0.038	1.24E-102	2.3	NR4A1	5.421052632
HIPK4	1.55E-106	0.304971	0.067	0.004	3.73E-102	2.3	HIPK4	16.75
NFE2L32	1.16E-104	0.8878491	0.302	0.077	2.81E-100	2.3	NFE2L3	3.922077922
FAM46C1	9.83E-104	0.6611691	0.187	0.032	2.37E-99	2.3	FAM46C	5.84375
S100Z1	2.50E-101	0.3116735	0.067	0.004	6.03E-97	2.3	S100Z	16.75
FAM171B2	6.03E-101	0.6123161	0.187	0.032	1.45E-96	2.3	FAM171B	5.84375
ATP1B2	1.17E-100	0.2566972	0.056	0.003	2.82E-96	2.3	ATP1B2	18.66666667
RP11-973N13.3	1.30E-100	0.4191594	0.079	0.006	3.15E-96	2.3	RP11-973N	13.16666667
METTL12	1.84E-100	1.25439	0.101	0.01	4.44E-96	2.3	METTL12	10.1
TRIM591	2.06E-100	0.260365	0.063	0.003	4.96E-96	2.3	TRIM59	21
EGR12	5.93E-100	1.439778	0.275	0.069	1.43E-95	2.3	EGR1	3.985507246
KIF251	6.80E-100	0.3523767	0.076	0.005	1.64E-95	2.3	KIF25	15.2
TMBIM62	5.85E-99	0.8589779	0.942	0.858	1.41E-94	2.3	TMBIM6	1.097902098
NIM1K	3.31E-97	0.4475483	0.098	0.01	7.99E-93	2.3	NIM1K	9.8
GSTCD2	6.87E-97	1.102541	0.338	0.103	1.66E-92	2.3	GSTCD	3.281553398
WBSCR28	1.68E-94	0.107525	0.027	0	4.05E-90	2.3	WBSCR28	#DIV/0!
SCUBE21	1.80E-93	0.7834986	0.24	0.055	4.34E-89	2.3	SCUBE2	4.363636364
RP11-644C3.11	6.75E-93	0.7517209	0.197	0.039	1.63E-88	2.3	RP11-644C	5.051282051
SLC10A11	3.92E-92	0.7067532	0.213	0.045	9.45E-88	2.3	SLC10A1	4.733333333
OPN51	4.01E-92	0.2245173	0.048	0.002	9.68E-88	2.3	OPN5	24
RP11-390E23.31	2.02E-90	0.4229887	0.096	0.01	4.88E-86	2.3	RP11-390E2	9.6
TGFB1	3.41E-90	0.9263135	0.211	0.045	8.22E-86	2.3	TGFB1	4.688888889
HSPH12	1.48E-89	1.010321	0.58	0.276	3.57E-85	2.3	HSPH1	2.101449275
HPX2	2.88E-88	1.228066	0.501	0.206	6.94E-84	2.3	HPX	2.432038835
FAM184A	4.27E-88	0.6172885	0.162	0.028	1.03E-83	2.3	FAM184A	5.785714286
CPT1C	4.92E-88	0.2688173	0.053	0.003	1.19E-83	2.3	CPT1C	17.66666667
CHD41	2.56E-87	1.065291	0.36	0.124	6.16E-83	2.3	CHD4	2.903225806
ATP5E2	4.89E-87	1.10848	0.768	0.55	1.18E-82	2.3	ATP5E	1.396363636
ELAVL3	8.12E-87	0.194453	0.035	0.001	1.96E-82	2.3	ELAVL3	35
SFXN51	1.05E-86	0.5747554	0.149	0.024	2.53E-82	2.3	SFXN5	6.208333333
TRIM69	1.19E-84	0.5746611	0.16	0.029	2.87E-80	2.3	TRIM69	5.517241379
RP11-1100L3.7	2.91E-84	0.1928966	0.029	0.001	7.02E-80	2.3	RP11-1100	29
GALM	5.42E-84	0.9099846	0.291	0.085	1.31E-79	2.3	GALM	3.423529412
TMPRSS71	6.62E-84	0.2305314	0.06	0.004	1.60E-79	2.3	TMPRSS7	15
NUP210L1	2.73E-83	0.6367354	0.159	0.029	6.57E-79	2.3	NUP210L	5.482758621
FAM117A	1.49E-82	0.5355472	0.1	0.012	3.59E-78	2.3	FAM117A	8.333333333
IGFBP42	1.79E-82	0.9621188	0.375	0.133	4.32E-78	2.3	IGFBP4	2.819548872
RAI22	2.37E-82	0.5162107	0.136	0.021	5.71E-78	2.3	RAI2	6.476190476
GOLGA6D	6.97E-82	0.1647934	0.034	0.001	1.68E-77	2.3	GOLGA6D	34
CCDC190	1.32E-81	0.2018116	0.039	0.001	3.19E-77	2.3	CCDC190	39
NUSAP11	4.89E-81	0.7027675	0.204	0.046	1.18E-76	2.3	NUSAP1	4.434782609
TMEM223	5.78E-81	0.4014267	0.084	0.008	1.39E-76	2.3	TMEM223	10.5

LINC00310	1.15E-80	0.5174958	0.135	0.021	2.77E-76	2.3	LINC00310	6.428571429
RP4-800F24.11	1.39E-80	0.4552406	0.121	0.017	3.36E-76	2.3	RP4-800F2	7.117647059
NQO12	3.13E-79	0.6835473	0.232	0.059	7.55E-75	2.3	NQO1	3.93220339
AC008592.4	3.98E-79	0.2853344	0.067	0.005	9.59E-75	2.3	AC008592.	13.4
APELA2	7.84E-79	0.5787466	0.115	0.016	1.89E-74	2.3	APELA	7.1875
TAF4B2	9.33E-79	0.8082273	0.204	0.047	2.25E-74	2.3	TAF4B	4.340425532
DRAIC1	1.25E-78	0.3947398	0.101	0.013	3.01E-74	2.3	DRAIC	7.769230769
HEPACAM22	1.91E-78	0.6197399	0.183	0.038	4.60E-74	2.3	HEPACAM2	4.815789474
ID21	3.42E-78	1.030784	0.306	0.099	8.26E-74	2.3	ID2	3.090909091
SHBG	1.77E-77	0.40023	0.067	0.006	4.27E-73	2.3	SHBG	11.16666667
PKLR	3.72E-77	0.1465369	0.038	0.001	8.97E-73	2.3	PKLR	38
ITGAM	4.72E-77	0.383639	0.084	0.009	1.14E-72	2.3	ITGAM	9.333333333
ABHD6	6.80E-77	0.4158249	0.069	0.006	1.64E-72	2.3	ABHD6	11.5
RBBP61	7.34E-77	1.053409	0.539	0.273	1.77E-72	2.3	RBBP6	1.974358974
TMPRSS11E1	1.17E-76	0.6922195	0.208	0.049	2.83E-72	2.3	TMPRSS11	4.244897959
ATP5EP22	7.19E-75	1.051361	0.558	0.297	1.73E-70	2.3	ATP5EP2	1.878787879
LMCD12	9.78E-75	0.852829	0.315	0.102	2.36E-70	2.3	LMCD1	3.088235294
XBP11	1.72E-74	1.294038	0.622	0.384	4.15E-70	2.3	XBP1	1.619791667
RP11-605B16.1	3.85E-74	0.2198972	0.041	0.002	9.28E-70	2.3	RP11-605B	20.5
APLP22	7.49E-74	0.8679672	0.539	0.27	1.81E-69	2.3	APLP2	1.996296296
FBXL18	1.81E-73	0.611569	0.128	0.021	4.37E-69	2.3	FBXL18	6.095238095
AC124944.5	4.99E-73	0.1793097	0.032	0.001	1.20E-68	2.3	AC124944.	32
RP11-517O13.11	2.25E-72	0.5164932	0.133	0.023	5.43E-68	2.3	RP11-517O	5.782608696
ITGA92	2.65E-72	0.4799836	0.143	0.026	6.39E-68	2.3	ITGA9	5.5
HES11	1.28E-71	1.507938	0.581	0.336	3.09E-67	2.3	HES1	1.729166667
RPL3L	2.45E-71	0.1897743	0.039	0.002	5.91E-67	2.3	RPL3L	19.5
GSTK12	3.29E-71	0.8992587	0.355	0.134	7.93E-67	2.3	GSTK1	2.649253731
OSBPL82	6.39E-71	1.004613	0.483	0.232	1.54E-66	2.3	OSBPL8	2.081896552
PNP1	7.89E-71	0.9149992	0.254	0.076	1.90E-66	2.3	PNP	3.342105263
CREBZF1	2.71E-70	0.76796	0.229	0.063	6.53E-66	2.3	CREBZF	3.634920635
CATSPERG	8.11E-70	0.4061031	0.098	0.013	1.96E-65	2.3	CATSPERG	7.538461538
BRD21	1.99E-69	1.022857	0.508	0.262	4.80E-65	2.3	BRD2	1.938931298
CCNG22	5.67E-69	1.013671	0.449	0.205	1.37E-64	2.3	CCNG2	2.190243902
UBL52	8.06E-69	0.9827332	0.711	0.503	1.94E-64	2.3	UBL5	1.413518887
COX7C3	8.99E-69	0.7339878	0.917	0.872	2.17E-64	2.3	COX7C	1.051605505
LDHC1	3.47E-68	0.4970999	0.122	0.021	8.36E-64	2.3	LDHC	5.80952381
SCP22	8.92E-68	0.9656556	0.565	0.317	2.15E-63	2.3	SCP2	1.782334385
PLEKHG21	1.02E-67	0.4384339	0.104	0.015	2.47E-63	2.3	PLEKHG2	6.933333333
CTD-2277K2.1	1.19E-67	0.3570295	0.067	0.006	2.86E-63	2.3	CTD-2277K	11.16666667
SC5D2	3.52E-67	0.9391932	0.41	0.177	8.49E-63	2.3	SC5D	2.316384181
MYL12B2	4.28E-67	0.7131043	0.934	0.817	1.03E-62	2.3	MYL12B	1.143206854
PRDX32	4.63E-67	0.8193598	0.466	0.218	1.12E-62	2.3	PRDX3	2.137614679
RP11-809N8.4	6.23E-67	0.2010845	0.041	0.002	1.50E-62	2.3	RP11-809N	20.5
AC098617.11	8.52E-67	0.1584334	0.041	0.002	2.06E-62	2.3	AC098617.	20.5
RP11-381K20.2	4.79E-66	0.3324694	0.08	0.009	1.15E-61	2.3	RP11-381K	8.888888889
BNIP3L1	5.28E-66	0.9309358	0.652	0.413	1.27E-61	2.3	BNIP3L	1.578692494
SPATA16	5.52E-66	0.2407131	0.045	0.003	1.33E-61	2.3	SPATA16	15
BTK	9.23E-66	0.1833893	0.037	0.002	2.22E-61	2.3	BTK	18.5
HMMR1	1.06E-65	0.2806872	0.065	0.006	2.55E-61	2.3	HMMR	10.83333333
APOL41	1.58E-65	0.3913255	0.094	0.013	3.80E-61	2.3	APOL4	7.230769231
RP1-167G20.11	4.02E-65	0.2797911	0.058	0.005	9.69E-61	2.3	RP1-167G2	11.6
PPP3CA2	4.19E-65	0.8676393	0.775	0.558	1.01E-60	2.3	PPP3CA	1.388888889

EFHD12	5.48E-65	0.9136214	0.558	0.293	1.32E-60	2.3	EFHD1	1.90443686
GDF91	6.74E-65	0.1671352	0.045	0.003	1.62E-60	2.3	GDF9	15
KCNMB41	5.66E-64	0.3435689	0.096	0.014	1.37E-59	2.3	KCNMB4	6.857142857
FGF181	6.10E-64	0.2576437	0.056	0.005	1.47E-59	2.3	FGF18	11.2
NDUFB12	1.21E-63	1.043712	0.573	0.352	2.91E-59	2.3	NDUFB1	1.627840909
TULP21	1.53E-63	0.5753738	0.147	0.031	3.69E-59	2.3	TULP2	4.741935484
UBE2S	1.93E-63	0.3823778	0.086	0.011	4.65E-59	2.3	UBE2S	7.818181818
BMP8A1	2.55E-63	0.2155408	0.055	0.004	6.15E-59	2.3	BMP8A	13.75
LSAMP2	3.11E-63	0.7891867	0.726	0.464	7.51E-59	2.3	LSAMP	1.564655172
TSPAN11	4.12E-63	0.8824666	0.468	0.22	9.93E-59	2.3	TSPAN1	2.127272727
DIO2-AS12	5.19E-63	0.822354	0.251	0.078	1.25E-58	2.3	DIO2-AS1	3.217948718
REEP52	9.36E-63	0.8642547	0.486	0.246	2.26E-58	2.3	REEP5	1.975609756
DHDH	9.31E-62	0.1684186	0.041	0.002	2.25E-57	2.3	DHDH	20.5
TEX351	1.43E-61	0.1442565	0.031	0.001	3.46E-57	2.3	TEX35	31
NEB1	2.04E-61	0.6118732	0.174	0.043	4.92E-57	2.3	NEB	4.046511628
RP11-93I21.31	3.26E-61	0.3596015	0.087	0.012	7.87E-57	2.3	RP11-93I21	7.25
ASIP	3.77E-61	0.2567162	0.056	0.005	9.09E-57	2.3	ASIP	11.2
F32	7.49E-61	0.872471	0.295	0.106	1.81E-56	2.3	F3	2.783018868
MYLIP1	1.03E-60	0.7630529	0.263	0.087	2.49E-56	2.3	MYLIP	3.022988506
CNNM4	1.08E-60	0.7589809	0.225	0.067	2.61E-56	2.3	CNNM4	3.358208955
ZFP36L23	1.64E-60	1.16226	0.604	0.365	3.96E-56	2.3	ZFP36L2	1.654794521
ANKRD42	1.18E-59	0.5358504	0.173	0.043	2.84E-55	2.3	ANKRD42	4.023255814
FAM222A1	1.20E-59	0.5331064	0.166	0.04	2.89E-55	2.3	FAM222A	4.15
DLG23	1.64E-59	1.017241	0.365	0.158	3.94E-55	2.3	DLG2	2.310126582
RP5-1185I7.1	3.55E-59	0.1010085	0.028	0.001	8.55E-55	2.3	RP5-1185I7	28
CFAP701	4.04E-59	0.6716098	0.162	0.038	9.75E-55	2.3	CFAP70	4.263157895
RP4-665J23.1	4.98E-59	0.1597172	0.034	0.002	1.20E-54	2.3	RP4-665J23	17
BMPR1B-AS11	1.10E-58	0.2572712	0.055	0.005	2.66E-54	2.3	BMPR1B-A	11
PCDH17	1.29E-58	0.1308951	0.024	0.001	3.11E-54	2.3	PCDH17	24
RHOB1	3.71E-58	0.9823923	0.289	0.106	8.95E-54	2.3	RHOB	2.726415094
MSRB31	3.88E-58	0.408123	0.1	0.016	9.36E-54	2.3	MSRB3	6.25
SLC24A11	4.36E-58	0.4019894	0.101	0.017	1.05E-53	2.3	SLC24A1	5.941176471
CTA-392E5.12	1.24E-56	0.6963881	0.138	0.03	2.98E-52	2.3	CTA-392E5	4.6
CPEB22	4.85E-56	0.6935611	0.573	0.302	1.17E-51	2.3	CPEB2	1.897350993
RP11-432B6.3	5.75E-56	0.2239818	0.056	0.005	1.39E-51	2.3	RP11-432B	11.2
C14orf22	1.09E-55	0.8194254	0.81	0.705	2.63E-51	2.3	C14orf2	1.14893617
SLC38A21	1.36E-55	0.9456842	0.617	0.375	3.28E-51	2.3	SLC38A2	1.645333333
TXN4	1.92E-55	0.8318881	0.801	0.672	4.64E-51	2.3	TXN	1.191964286
RP11-727A23.10	2.17E-55	0.5200391	0.101	0.017	5.22E-51	2.3	RP11-727A	5.941176471
CENPL	3.24E-55	0.3595124	0.1	0.017	7.81E-51	2.3	CENPL	5.882352941
DYSF1	4.84E-55	0.2610415	0.063	0.007	1.17E-50	2.3	DYSF	9
NDUFA43	7.81E-55	0.7485499	0.834	0.747	1.88E-50	2.3	NDUFA4	1.116465863
SNX141	9.16E-55	0.7512843	0.294	0.113	2.21E-50	2.3	SNX14	2.601769912
Jun-01	1.09E-54	1.059256	0.444	0.23	2.62E-50	2.3	JUN	1.930434783
PRCP2	1.40E-54	0.5564958	0.173	0.046	3.38E-50	2.3	PRCP	3.760869565
SIRT11	1.41E-54	0.6908733	0.274	0.098	3.41E-50	2.3	SIRT1	2.795918367
KMT2E2	3.67E-54	0.9599966	0.646	0.461	8.85E-50	2.3	KMT2E	1.401301518
HORMAD2	5.22E-54	0.2652122	0.067	0.008	1.26E-49	2.3	HORMAD2	8.375
RP11-588H23.3	6.34E-54	0.1354132	0.032	0.002	1.53E-49	2.3	RP11-588H	16
ADAM321	1.09E-53	0.9551901	0.444	0.224	2.63E-49	2.3	ADAM32	1.982142857
RP11-455F5.3	1.45E-53	0.1505874	0.031	0.001	3.50E-49	2.3	RP11-455F5	31
PEAK12	3.45E-53	1.124885	0.482	0.261	8.33E-49	2.3	PEAK1	1.846743295

FAM105A2	4.55E-53	0.4310681	0.117	0.023	1.10E-48	2.3	FAM105A	5.086956522
PPP1R13B1	6.75E-53	0.8901293	0.395	0.186	1.63E-48	2.3	PPP1R13B	2.123655914
UBB3	7.59E-53	0.9944271	0.815	0.719	1.83E-48	2.3	UBB	1.133518776
ERBB42	8.82E-53	0.6604628	0.765	0.485	2.13E-48	2.3	ERBB4	1.577319588
UQCRB3	9.17E-53	0.7438607	0.865	0.804	2.21E-48	2.3	UQCRB	1.075870647
BASP11	3.26E-52	0.570506	0.178	0.049	7.86E-48	2.3	BASP1	3.632653061
RP11-324D17.1	9.00E-52	0.1225872	0.028	0.001	2.17E-47	2.3	RP11-324D	28
RP11-429J17.7	9.25E-52	0.1374517	0.027	0.001	2.23E-47	2.3	RP11-429J1	27
WLS2	2.01E-51	0.7744056	0.372	0.17	4.84E-47	2.3	WLS	2.188235294
POLG21	2.21E-51	0.6610124	0.233	0.079	5.33E-47	2.3	POLG2	2.949367089
GNAS2	2.82E-51	0.6414777	0.858	0.75	6.80E-47	2.3	GNAS	1.144
TMPRSS12	2.83E-51	0.2607166	0.069	0.009	6.82E-47	2.3	TMPRSS12	7.666666667
ACTG11	3.48E-51	1.00293	0.855	0.721	8.38E-47	2.3	ACTG1	1.185852982
AMD12	5.85E-51	0.8611865	0.462	0.246	1.41E-46	2.3	AMD1	1.87804878
WWP2	7.64E-51	0.7794651	0.205	0.065	1.84E-46	2.3	WWP2	3.153846154
LINC01033	1.57E-50	0.3441012	0.066	0.008	3.79E-46	2.3	LINC01033	8.25
EIF5	2.07E-50	0.9100624	0.427	0.222	5.00E-46	2.3	EIF5	1.923423423
DDX3X	3.49E-50	0.8424186	0.566	0.358	8.41E-46	2.3	DDX3X	1.581005587
NPHS1	4.42E-50	0.2583131	0.067	0.009	1.07E-45	2.3	NPHS1	7.444444444
CCNE1	6.59E-50	0.2290601	0.041	0.003	1.59E-45	2.3	CCNE1	13.66666667
AC007879.1	7.19E-50	0.402097	0.087	0.014	1.73E-45	2.3	AC007879.	6.214285714
NDUFA13	1.76E-49	0.7412618	0.739	0.603	4.25E-45	2.3	NDUFA1	1.225538972
MEPE2	2.71E-49	0.3399525	0.08	0.012	6.53E-45	2.3	MEPE	6.666666667
RP11-396C23.2	3.51E-49	0.1632833	0.041	0.003	8.46E-45	2.3	RP11-396C	13.66666667
JUND2	4.21E-49	0.7635926	0.367	0.169	1.01E-44	2.3	JUND	2.171597633
MAGI32	6.70E-49	0.8411513	0.593	0.378	1.62E-44	2.3	MAGI3	1.568783069
C6orf62	6.78E-49	0.8050146	0.4	0.203	1.63E-44	2.3	C6orf62	1.97044335
PYGM1	9.61E-49	0.1675257	0.035	0.002	2.32E-44	2.3	PYGM	17.5
RP11-178C3.1	1.66E-48	0.2882928	0.076	0.011	4.00E-44	2.3	RP11-178C	6.909090909
IER31	1.74E-48	1.021517	0.531	0.313	4.19E-44	2.3	IER3	1.696485623
HCLS11	4.81E-48	0.1646315	0.039	0.003	1.16E-43	2.3	HCLS1	13
RND11	4.92E-48	0.736098	0.229	0.079	1.19E-43	2.3	RND1	2.898734177
RP11-321N4.5	7.67E-48	0.2840419	0.072	0.01	1.85E-43	2.3	RP11-321N	7.2
ATP5J2	2.25E-47	0.7724368	0.673	0.494	5.42E-43	2.3	ATP5J	1.362348178
LINC015881	2.92E-47	0.770668	0.257	0.097	7.05E-43	2.3	LINC01588	2.649484536
COX6B12	6.10E-47	0.673875	0.829	0.759	1.47E-42	2.3	COX6B1	1.092226614
NELFB	6.61E-47	0.309657	0.072	0.011	1.59E-42	2.3	NELFB	6.545454545
LPAR32	1.35E-46	0.5317861	0.146	0.038	3.26E-42	2.3	LPAR3	3.842105263
CD4	1.92E-46	0.2190771	0.048	0.005	4.63E-42	2.3	CD4	9.6
GSPT12	2.02E-46	0.734867	0.572	0.363	4.88E-42	2.3	GSPT1	1.575757576
UQCR101	3.70E-46	0.8090582	0.667	0.504	8.93E-42	2.3	UQCR10	1.323412698
PGR2	4.93E-46	0.6102015	0.226	0.078	1.19E-41	2.3	PGR	2.897435897
ATP5I2	5.34E-46	0.7443581	0.316	0.139	1.29E-41	2.3	ATP5I	2.273381295
SRP142	5.85E-46	0.5057089	0.933	0.872	1.41E-41	2.3	SRP14	1.069954128
ARMC4	1.02E-45	0.2452231	0.046	0.005	2.45E-41	2.3	ARMC4	9.2
VCL4	2.30E-45	0.8260529	0.508	0.305	5.54E-41	2.3	VCL	1.66557377
C6orf991	2.93E-45	0.2824331	0.066	0.009	7.06E-41	2.3	C6orf99	7.333333333
IDI11	3.91E-45	0.8690019	0.41	0.216	9.44E-41	2.3	IDI1	1.898148148
TSC22D23	6.96E-45	0.5966276	0.687	0.461	1.68E-40	2.3	TSC22D2	1.490238612
RND32	1.41E-44	0.7762252	0.549	0.345	3.39E-40	2.3	RND3	1.591304348
DDR23	1.56E-44	0.6731367	0.216	0.076	3.77E-40	2.3	DDR2	2.842105263
TMSB4X	2.14E-44	0.3812511	0.994	0.935	5.15E-40	2.3	TMSB4X	1.063101604

PRPS22	3.38E-44	0.5594161	0.219	0.077	8.15E-40	2.3	PRPS2	2.844155844
FAM222A-AS12	3.45E-44	0.4293838	0.121	0.028	8.32E-40	2.3	FAM222A-/	4.321428571
LINC01504	4.16E-44	0.1953979	0.042	0.004	1.00E-39	2.3	LINC01504	10.5
GALNT62	6.10E-44	0.5814835	0.187	0.06	1.47E-39	2.3	GALNT6	3.116666667
DARS2	7.28E-44	0.5470721	0.206	0.07	1.76E-39	2.3	DARS2	2.942857143
APC21	1.11E-43	0.1284952	0.032	0.002	2.68E-39	2.3	APC2	16
NEDD4L3	1.23E-43	0.6775326	0.757	0.594	2.97E-39	2.3	NEDD4L	1.274410774
C9orf1521	1.61E-43	0.4946974	0.156	0.044	3.88E-39	2.3	C9orf152	3.545454545
ABHD5	1.63E-43	0.7460584	0.402	0.209	3.93E-39	2.3	ABHD5	1.923444976
RP11-173M1.4	1.69E-43	0.116377	0.024	0.001	4.09E-39	2.3	RP11-173M	24
P4HA32	2.66E-43	0.3924165	0.129	0.032	6.42E-39	2.3	P4HA3	4.03125
CDKL21	3.69E-43	0.3395343	0.084	0.015	8.89E-39	2.3	CDKL2	5.6
SEC623	1.00E-42	0.7903397	0.706	0.574	2.42E-38	2.3	SEC62	1.229965157
CAPRIN2	1.14E-42	0.2609447	0.058	0.008	2.74E-38	2.3	CAPRIN2	7.25
POU2F2	1.28E-42	0.4088588	0.08	0.014	3.08E-38	2.3	POU2F2	5.714285714
DNAJC3-AS12	1.89E-42	0.3325145	0.083	0.015	4.57E-38	2.3	DNAJC3-AS	5.533333333
ADAMTS122	3.00E-42	0.2657815	0.048	0.005	7.24E-38	2.3	ADAMTS12	9.6
HIST1H2BD2	3.39E-42	0.9968888	0.489	0.295	8.18E-38	2.3	HIST1H2BD	1.657627119
TFRC	3.62E-42	0.550643	0.159	0.047	8.74E-38	2.3	TFRC	3.382978723
DOCK112	3.65E-42	0.3940331	0.108	0.024	8.81E-38	2.3	DOCK11	4.5
AC073333.8	4.74E-42	0.2977693	0.073	0.012	1.14E-37	2.3	AC073333.8	6.083333333
RP11-440G9.1	5.13E-42	0.1504229	0.022	0.001	1.24E-37	2.3	RP11-440G	22
NME51	6.57E-42	0.4142018	0.107	0.024	1.58E-37	2.3	NME5	4.458333333
TMA72	7.08E-42	0.7278113	0.722	0.599	1.71E-37	2.3	TMA7	1.205342237
EFNB22	9.03E-42	0.660633	0.25	0.1	2.18E-37	2.3	EFNB2	2.5
MILR11	9.37E-42	0.2160696	0.039	0.004	2.26E-37	2.3	MILR1	9.75
IL1RN2	1.18E-41	0.5707909	0.16	0.048	2.85E-37	2.3	IL1RN	3.333333333
CPE2	1.58E-41	0.7580653	0.396	0.199	3.81E-37	2.3	CPE	1.989949749
CD632	1.80E-41	0.754658	0.577	0.377	4.35E-37	2.3	CD63	1.530503979
AP000318.2	1.85E-41	0.3562465	0.098	0.021	4.46E-37	2.3	AP000318.2	4.666666667
AC018890.62	1.92E-41	0.7739898	0.426	0.234	4.63E-37	2.3	AC018890.6	1.820512821
ABHD101	2.89E-41	0.2755297	0.076	0.013	6.98E-37	2.3	ABHD10	5.846153846
SPDYA1	2.99E-41	0.618326	0.251	0.099	7.22E-37	2.3	SPDYA	2.535353535
KIF17	3.77E-41	0.1594179	0.039	0.004	9.08E-37	2.3	KIF17	9.75
EPAS1	4.63E-41	0.656947	0.319	0.147	1.12E-36	2.3	EPAS1	2.170068027
AC004257.1	5.74E-41	0.1284222	0.035	0.003	1.38E-36	2.3	AC004257.	11.66666667
ANXA24	8.73E-41	0.4829203	0.971	0.925	2.10E-36	2.3	ANXA2	1.04972973
HIP12	9.72E-41	0.7412861	0.229	0.088	2.34E-36	2.3	HIP1	2.602272727
SLC27A42	1.07E-40	0.5589047	0.857	0.699	2.57E-36	2.3	SLC27A4	1.226037196
DBI4	1.86E-40	0.6694378	0.792	0.65	4.49E-36	2.3	DBI	1.218461538
CD1642	2.33E-40	0.763941	0.463	0.278	5.62E-36	2.3	CD164	1.665467626
MPHOSPH61	2.33E-40	0.7714149	0.281	0.124	5.63E-36	2.3	MPHOSPH6	2.266129032
UNQ6494	6.45E-40	0.1262718	0.024	0.001	1.56E-35	2.3	UNQ6494	24
RASL11B1	1.15E-39	0.1682543	0.039	0.004	2.76E-35	2.3	RASL11B	9.75
ARID5B2	2.64E-39	0.8408644	0.853	0.73	6.38E-35	2.3	ARID5B	1.168493151
PRKCG	7.40E-39	0.3208037	0.07	0.012	1.78E-34	2.3	PRKCG	5.833333333
GCM12	7.82E-39	0.2653968	0.049	0.006	1.89E-34	2.3	GCM1	8.166666667
ACVR1C1	7.95E-39	0.3701934	0.103	0.024	1.92E-34	2.3	ACVR1C	4.291666667
RP11-221J22.11	9.22E-39	0.1947122	0.045	0.005	2.22E-34	2.3	RP11-221J2	9
UQCRQ2	1.48E-38	0.6255795	0.261	0.111	3.56E-34	2.3	UQCRQ	2.351351351
PYY	1.57E-38	0.3522458	0.044	0.005	3.78E-34	2.3	PYY	8.8
PRLR2	4.07E-38	0.736428	0.419	0.232	9.82E-34	2.3	PRLR	1.806034483

AP3B2	4.15E-38	0.3325232	0.055	0.008	1.00E-33	2.3	AP3B2	6.875
USMG52	5.48E-38	0.6378486	0.749	0.626	1.32E-33	2.3	USMG5	1.196485623
SKIDA11	5.65E-38	0.1275316	0.021	0.001	1.36E-33	2.3	SKIDA1	21
CDKN2A	8.85E-38	0.1395788	0.028	0.002	2.13E-33	2.3	CDKN2A	14
RP11-718O11.1	1.06E-37	0.3155545	0.076	0.014	2.56E-33	2.3	RP11-718O	5.428571429
PHTF22	1.19E-37	0.5331811	0.213	0.081	2.86E-33	2.3	PHTF2	2.62962963
FAM179A1	1.44E-37	0.1982052	0.035	0.003	3.47E-33	2.3	FAM179A	11.66666667
HSPB81	1.69E-37	0.4959921	0.199	0.072	4.08E-33	2.3	HSPB8	2.763888889
AC002463.3	1.93E-37	0.2431822	0.039	0.004	4.66E-33	2.3	AC002463.:	9.75
RP11-26J3.1	1.94E-37	0.2676065	0.042	0.005	4.68E-33	2.3	RP11-26J3.	8.4
KCNK61	3.15E-37	0.4891177	0.142	0.042	7.59E-33	2.3	KCNK6	3.380952381
PANK32	3.16E-37	0.7208788	0.34	0.174	7.62E-33	2.3	PANK3	1.954022989
NDUFB32	4.03E-37	0.7343645	0.376	0.211	9.73E-33	2.3	NDUFB3	1.781990521
REC1141	5.08E-37	0.2466805	0.063	0.01	1.22E-32	2.3	REC114	6.3
EML61	6.64E-37	0.3708416	0.115	0.03	1.60E-32	2.3	EML6	3.833333333
RP11-517O13.31	8.68E-37	0.343891	0.081	0.016	2.09E-32	2.3	RP11-517O	5.0625
COX7B3	1.02E-36	0.6296462	0.749	0.63	2.47E-32	2.3	COX7B	1.188888889
ANKFY11	1.09E-36	0.5728657	0.204	0.077	2.64E-32	2.3	ANKFY1	2.649350649
PCAT292	1.18E-36	0.1824112	0.038	0.004	2.86E-32	2.3	PCAT29	9.5
FBP12	1.23E-36	0.3698531	0.125	0.034	2.98E-32	2.3	FBP1	3.676470588
KIAA13242	1.58E-36	0.6436288	0.406	0.223	3.81E-32	2.3	KIAA1324	1.820627803
NXF1	1.67E-36	0.5931971	0.218	0.086	4.03E-32	2.3	NXF1	2.534883721
ADGRF41	2.18E-36	0.1295257	0.032	0.003	5.26E-32	2.3	ADGRF4	10.66666667
AP000695.61	2.27E-36	0.2280916	0.052	0.007	5.48E-32	2.3	AP000695.(	7.428571429
RP11-48B3.41	2.31E-36	0.3125815	0.077	0.015	5.56E-32	2.3	RP11-48B3	5.133333333
AC106900.61	2.72E-36	0.2504982	0.067	0.012	6.56E-32	2.3	AC106900.(	5.583333333
SYTL31	4.53E-36	0.5592276	0.169	0.057	1.09E-31	2.3	SYTL3	2.964912281
IGF2BP31	5.27E-36	0.3973904	0.111	0.028	1.27E-31	2.3	IGF2BP3	3.964285714
HMGN52	5.44E-36	0.5606612	0.183	0.064	1.31E-31	2.3	HMGN5	2.859375
DLL4	6.21E-36	0.1682015	0.037	0.004	1.50E-31	2.3	DLL4	9.25
TLL21	6.57E-36	0.2635747	0.077	0.015	1.59E-31	2.3	TLL2	5.133333333
CTD-2540M10.1	6.99E-36	0.1143172	0.025	0.002	1.69E-31	2.3	CTD-2540N	12.5
NDUFA51	7.81E-36	0.7114211	0.476	0.306	1.88E-31	2.3	NDUFA5	1.555555556
TMEM150B	8.00E-36	0.2000985	0.041	0.005	1.93E-31	2.3	TMEM150I	8.2
ERRFI13	1.11E-35	0.6948846	0.702	0.536	2.68E-31	2.3	ERRFI1	1.309701493
EMCN1	1.99E-35	0.382516	0.112	0.029	4.80E-31	2.3	EMCN	3.862068966
TFPI2	2.15E-35	0.550582	0.544	0.32	5.17E-31	2.3	TFPI	1.7
ACRC2	2.74E-35	0.5527982	0.237	0.097	6.60E-31	2.3	ACRC	2.443298969
KRT86	3.21E-35	0.316673	0.07	0.013	7.73E-31	2.3	KRT86	5.384615385
COX7A24	6.69E-35	0.5956415	0.831	0.761	1.61E-30	2.3	COX7A2	1.091984231
PDSS12	8.07E-35	0.5398683	0.132	0.039	1.95E-30	2.3	PDSS1	3.384615385
TTC271	9.18E-35	0.5001869	0.163	0.055	2.21E-30	2.3	TTC27	2.963636364
FAM46A1	1.73E-34	0.7564834	0.329	0.166	4.16E-30	2.3	FAM46A	1.981927711
AP000695.4	1.75E-34	0.1574283	0.038	0.004	4.21E-30	2.3	AP000695.4	9.5
LINC010311	1.90E-34	0.2716469	0.074	0.015	4.58E-30	2.3	LINC01031	4.933333333
ZNF1651	2.22E-34	0.7284812	0.232	0.098	5.36E-30	2.3	ZNF165	2.367346939
PCF111	3.14E-34	0.5955995	0.267	0.121	7.57E-30	2.3	PCF11	2.20661157
ASB151	3.21E-34	0.1929342	0.046	0.006	7.75E-30	2.3	ASB15	7.666666667
RP11-202G18.11	3.51E-34	0.1487922	0.041	0.005	8.47E-30	2.3	RP11-202G	8.2
CTD-2308B18.31	3.75E-34	0.2275385	0.062	0.011	9.05E-30	2.3	CTD-2308B	5.636363636
TMEM22	3.93E-34	0.5063629	0.539	0.342	9.47E-30	2.3	TMEM2	1.576023392
RP11-507B12.22	4.31E-34	0.5399325	0.237	0.098	1.04E-29	2.3	RP11-507B	2.418367347

PPP1R1C	4.99E-34	0.3995657	0.07	0.013	1.20E-29	2.3	PPP1R1C	5.384615385
RP1-272L16.1	5.10E-34	0.1000364	0.02	0.001	1.23E-29	2.3	RP1-272L1(	20
HSP90AA14	5.98E-34	0.4511104	0.926	0.852	1.44E-29	2.3	HSP90AA1	1.08685446
BTG11	7.52E-34	0.5751732	0.735	0.577	1.81E-29	2.3	BTG1	1.273830156
LINGO13	1.64E-33	0.5177053	0.926	0.888	3.95E-29	2.3	LINGO1	1.042792793
CPNE31	2.71E-33	0.6906834	0.338	0.182	6.53E-29	2.3	CPNE3	1.857142857
PPM1K2	2.90E-33	0.6097572	0.299	0.146	7.00E-29	2.3	PPM1K	2.047945205
CHD12	2.98E-33	0.7125005	0.434	0.266	7.19E-29	2.3	CHD1	1.631578947
ADGRF21	6.21E-33	0.137693	0.038	0.004	1.50E-28	2.3	ADGRF2	9.5
RP11-174G6.11	6.41E-33	0.5732084	0.171	0.062	1.54E-28	2.3	RP11-174G	2.758064516
EGLN31	1.15E-32	0.6551821	0.292	0.142	2.76E-28	2.3	EGLN3	2.056338028
MYO5A1	1.41E-32	0.7015197	0.235	0.104	3.40E-28	2.3	MYO5A	2.259615385
L1TD11	1.54E-32	0.121378	0.027	0.002	3.72E-28	2.3	L1TD1	13.5
CCDC832	1.66E-32	0.2511397	0.084	0.019	4.01E-28	2.3	CCDC83	4.421052632
SLC17A81	1.69E-32	0.2173972	0.077	0.016	4.08E-28	2.3	SLC17A8	4.8125
PARK72	2.47E-32	0.6567891	0.59	0.452	5.96E-28	2.3	PARK7	1.305309735
RTKN21	2.48E-32	0.4242369	0.152	0.05	5.98E-28	2.3	RTKN2	3.04
HAP1	4.17E-32	0.1187101	0.034	0.003	1.00E-27	2.3	HAP1	11.33333333
LEKR1	5.00E-32	0.3748243	0.087	0.021	1.20E-27	2.3	LEKR1	4.142857143
LINC00877	7.30E-32	0.130769	0.028	0.002	1.76E-27	2.3	LINC00877	14
CALCRL	7.62E-32	0.1038177	0.027	0.002	1.84E-27	2.3	CALCRL	13.5
SLC9A1	8.86E-32	0.6117676	0.256	0.119	2.14E-27	2.3	SLC9A1	2.151260504
NUDT41	9.48E-32	0.4993529	0.184	0.07	2.29E-27	2.3	NUDT4	2.628571429
FLOT11	1.79E-31	0.8941231	0.25	0.119	4.31E-27	2.3	FLOT1	2.100840336
ERO1B2	2.36E-31	0.5593344	0.222	0.095	5.70E-27	2.3	ERO1B	2.336842105
POLN1	2.54E-31	0.4589513	0.132	0.042	6.12E-27	2.3	POLN	3.142857143
DYNLL13	3.60E-31	0.6624716	0.4	0.241	8.69E-27	2.3	DYNLL1	1.659751037
CYTIP1	5.18E-31	0.215397	0.052	0.008	1.25E-26	2.3	CYTIP	6.5
AIG11	5.53E-31	0.6393183	0.358	0.203	1.33E-26	2.3	AIG1	1.763546798
RP5-1085F17.3	6.54E-31	0.3265827	0.088	0.022	1.58E-26	2.3	RP5-1085F:	4
VTCN11	7.13E-31	0.5456415	0.237	0.105	1.72E-26	2.3	VTCN1	2.257142857
GLA2	8.56E-31	0.656991	0.218	0.094	2.06E-26	2.3	GLA	2.319148936
RP11-340E6.1	9.95E-31	0.1352002	0.027	0.002	2.40E-26	2.3	RP11-340E(	13.5
RP4-633O19--A.13	1.80E-30	0.2772929	0.079	0.018	4.33E-26	2.3	RP4-633O1	4.388888889
C9orf153	1.88E-30	0.3106082	0.069	0.014	4.54E-26	2.3	C9orf153	4.928571429
MDC1	2.00E-30	0.3853852	0.088	0.022	4.81E-26	2.3	MDC1	4
RP5-857K21.41	2.56E-30	0.8405848	0.579	0.43	6.18E-26	2.3	RP5-857K2	1.346511628
AC008074.12	2.72E-30	0.3698214	0.126	0.039	6.56E-26	2.3	AC008074.	3.230769231
ZSCAN16-AS12	2.94E-30	0.4705383	0.209	0.088	7.09E-26	2.3	ZSCAN16-A	2.375
BCL2L151	3.44E-30	0.1630321	0.038	0.005	8.29E-26	2.3	BCL2L15	7.6
EZR1	3.63E-30	0.6604263	0.541	0.373	8.74E-26	2.3	EZR	1.450402145
MAFB1	3.89E-30	0.4414951	0.139	0.046	9.38E-26	2.3	MAFB	3.02173913
DACT3-AS1	4.14E-30	0.1684625	0.032	0.003	9.97E-26	2.3	DACT3-AS1	10.66666667
FASN2	4.58E-30	0.4241625	0.145	0.049	1.10E-25	2.3	FASN	2.959183673
SNX27	5.21E-30	0.637762	0.292	0.15	1.26E-25	2.3	SNX27	1.946666667
HSP90B13	6.74E-30	0.5053211	0.669	0.503	1.62E-25	2.3	HSP90B1	1.330019881
PEBP42	6.76E-30	0.5180302	0.218	0.092	1.63E-25	2.3	PEBP4	2.369565217
CA23	7.25E-30	0.5330108	0.209	0.088	1.75E-25	2.3	CA2	2.375
ASB11	9.75E-30	0.1487658	0.034	0.004	2.35E-25	2.3	ASB11	8.5
SLCO2B11	1.08E-29	0.1200431	0.041	0.005	2.60E-25	2.3	SLCO2B1	8.2
RP11-567N4.21	1.13E-29	0.1057409	0.031	0.003	2.73E-25	2.3	RP11-567N	10.33333333
TCEB12	1.18E-29	0.7960696	0.458	0.303	2.86E-25	2.3	TCEB1	1.511551155

TMCO13	1.19E-29	0.6829474	0.492	0.342	2.88E-25	2.3	TMCO1	1.438596491
CLDN113	2.09E-29	0.4119202	0.162	0.059	5.03E-25	2.3	CLDN11	2.745762712
MAST1	2.32E-29	0.201423	0.032	0.004	5.59E-25	2.3	MAST1	8
STOM2	2.65E-29	0.5890209	0.426	0.258	6.40E-25	2.3	STOM	1.651162791
SFPQ2	2.73E-29	0.6734346	0.378	0.226	6.59E-25	2.3	SFPQ	1.672566372
LINC005042	2.83E-29	0.4690308	0.208	0.087	6.82E-25	2.3	LINC00504	2.390804598
NRCAM	3.01E-29	0.1714639	0.045	0.007	7.27E-25	2.3	NRCAM	6.428571429
TCEANC21	3.17E-29	0.6998025	0.39	0.235	7.63E-25	2.3	TCEANC2	1.659574468
ENPP12	3.44E-29	0.4975375	0.272	0.131	8.30E-25	2.3	ENPP1	2.076335878
ATP5G13	4.96E-29	0.6579065	0.323	0.179	1.20E-24	2.3	ATP5G1	1.804469274
RPN22	5.06E-29	0.5955103	0.424	0.264	1.22E-24	2.3	RPN2	1.606060606
TUFT13	5.26E-29	0.6149699	0.327	0.179	1.27E-24	2.3	TUFT1	1.826815642
IL20RA1	5.50E-29	0.493055	0.146	0.051	1.33E-24	2.3	IL20RA	2.862745098
ST8SIA5	6.07E-29	0.1110828	0.025	0.002	1.46E-24	2.3	ST8SIA5	12.5
CLSTN22	6.61E-29	0.4657695	0.177	0.068	1.59E-24	2.3	CLSTN2	2.602941176
RNF103-CHMP3	7.64E-29	0.3867693	0.108	0.032	1.84E-24	2.3	RNF103-CH	3.375
HBEGF1	8.32E-29	0.3056202	0.1	0.028	2.01E-24	2.3	HBEGF	3.571428571
SMIM142	1.00E-28	0.6312331	0.562	0.395	2.42E-24	2.3	SMIM14	1.42278481
CDKN1A	1.20E-28	0.4962215	0.164	0.063	2.89E-24	2.3	CDKN1A	2.603174603
SLC16A9	1.26E-28	0.1408267	0.034	0.004	3.03E-24	2.3	SLC16A9	8.5
SYT161	1.36E-28	0.1528776	0.039	0.005	3.27E-24	2.3	SYT16	7.8
ATP1A12	1.66E-28	0.6272283	0.633	0.502	4.00E-24	2.3	ATP1A1	1.260956175
CNBD21	1.85E-28	0.204299	0.049	0.008	4.47E-24	2.3	CNBD2	6.125
CYCS4	2.63E-28	0.7041324	0.66	0.517	6.35E-24	2.3	CYCS	1.276595745
IDI2	3.15E-28	0.1072751	0.018	0.001	7.60E-24	2.3	IDI2	18
RP13-131K19.1	3.49E-28	0.1113401	0.02	0.001	8.42E-24	2.3	RP13-131K	20
BAG31	4.01E-28	0.4956331	0.173	0.068	9.66E-24	2.3	BAG3	2.544117647
FAM71F11	4.36E-28	0.1108148	0.029	0.003	1.05E-23	2.3	FAM71F1	9.666666667
CRACR2A2	4.42E-28	0.2879973	0.083	0.021	1.07E-23	2.3	CRACR2A	3.952380952
RP11-53O19.11	4.59E-28	0.3580039	0.069	0.015	1.11E-23	2.3	RP11-53O1	4.6
ATXN11	5.36E-28	0.6952041	0.639	0.512	1.29E-23	2.3	ATXN1	1.248046875
CH17-437K3.11	5.81E-28	0.1815328	0.044	0.007	1.40E-23	2.3	CH17-437K	6.285714286
NEK112	6.04E-28	0.4620866	0.16	0.06	1.46E-23	2.3	NEK11	2.666666667
ZC3HAV11	6.53E-28	0.6624815	0.331	0.185	1.57E-23	2.3	ZC3HAV1	1.789189189
SLC9C11	8.76E-28	0.4497437	0.152	0.055	2.11E-23	2.3	SLC9C1	2.763636364
CTC-490E21.122	1.30E-27	0.434142	0.15	0.055	3.14E-23	2.3	CTC-490E2	2.727272727
PKD1L31	1.31E-27	0.348213	0.107	0.032	3.17E-23	2.3	PKD1L3	3.34375
AC015971.2	1.53E-27	0.4266602	0.124	0.041	3.69E-23	2.3	AC015971.	3.024390244
TDP2	1.58E-27	0.4009271	0.126	0.042	3.80E-23	2.3	TDP2	3
SLC26A33	1.71E-27	0.4392626	0.886	0.786	4.13E-23	2.3	SLC26A3	1.127226463
BAMBI1	1.80E-27	0.552981	0.219	0.1	4.33E-23	2.3	BAMBI	2.19
ASAH12	1.86E-27	0.5061019	0.218	0.099	4.48E-23	2.3	ASAH1	2.202020202
MATN1	2.50E-27	0.1609399	0.022	0.002	6.03E-23	2.3	MATN1	11
ACTB2	2.92E-27	0.751623	0.754	0.64	7.04E-23	2.3	ACTB	1.178125
RP13-143G15.4	4.11E-27	0.2157525	0.044	0.007	9.90E-23	2.3	RP13-143G	6.285714286
H2AFV2	4.80E-27	0.4693818	0.201	0.087	1.16E-22	2.3	H2AFV	2.310344828
BZW2	5.93E-27	0.636055	0.267	0.137	1.43E-22	2.3	BZW2	1.948905109
C18orf82	8.57E-27	0.4877156	0.17	0.068	2.07E-22	2.3	C18orf8	2.5
USP501	1.17E-26	0.1840744	0.052	0.01	2.81E-22	2.3	USP50	5.2
SLIRP3	1.27E-26	0.8909785	0.435	0.306	3.05E-22	2.3	SLIRP	1.421568627
TMEM39B1	1.45E-26	0.2426789	0.065	0.014	3.51E-22	2.3	TMEM39B	4.642857143
NPNT	1.49E-26	0.4132187	0.094	0.027	3.60E-22	2.3	NPNT	3.481481481

LGALS4	1.57E-26	0.1417635	0.035	0.005	3.78E-22	2.3	LGALS4	7
SPSB2	2.10E-26	0.4189406	0.032	0.004	5.07E-22	2.3	SPSB2	8
EREG2	2.31E-26	0.4632083	0.444	0.256	5.57E-22	2.3	EREG	1.734375
TNIK2	2.40E-26	0.5847092	0.299	0.158	5.79E-22	2.3	TNIK	1.892405063
SLC2A101	2.45E-26	0.2511255	0.063	0.014	5.90E-22	2.3	SLC2A10	4.5
SH3KBP11	2.47E-26	0.6715169	0.216	0.101	5.94E-22	2.3	SH3KBP1	2.138613861
CTD-2054N24.2	2.69E-26	0.2077345	0.055	0.011	6.50E-22	2.3	CTD-2054N	5
DUSP51	2.84E-26	0.5969799	0.389	0.234	6.85E-22	2.3	DUSP5	1.662393162
SIX42	3.98E-26	0.4937804	0.183	0.077	9.60E-22	2.3	SIX4	2.376623377
VSIG101	4.16E-26	0.4294779	0.11	0.035	1.00E-21	2.3	VSIG10	3.142857143
CKS22	5.34E-26	0.5659771	0.361	0.214	1.29E-21	2.3	CKS2	1.686915888
GATB	6.74E-26	0.191907	0.049	0.009	1.62E-21	2.3	GATB	5.444444444
CTD-2587M2.11	8.01E-26	0.3323917	0.093	0.026	1.93E-21	2.3	CTD-2587N	3.576923077
NR4A31	8.86E-26	0.3431542	0.076	0.019	2.14E-21	2.3	NR4A3	4
RP4-655J12.4	1.01E-25	0.2303719	0.045	0.008	2.43E-21	2.3	RP4-655J12	5.625
KIF24	1.29E-25	0.1704452	0.044	0.007	3.11E-21	2.3	KIF24	6.285714286
NEFM	1.34E-25	0.1007626	0.017	0.001	3.23E-21	2.3	NEFM	17
RIIAD11	1.49E-25	0.1316476	0.031	0.004	3.59E-21	2.3	RIIAD1	7.75
ATF7IP21	1.50E-25	0.4382196	0.122	0.042	3.62E-21	2.3	ATF7IP2	2.904761905
SLC40A11	1.87E-25	0.2727465	0.08	0.021	4.51E-21	2.3	SLC40A1	3.80952381
NDUFB41	2.76E-25	0.7626952	0.546	0.433	6.66E-21	2.3	NDUFB4	1.260969977
ANKRD46	3.09E-25	0.366113	0.112	0.037	7.45E-21	2.3	ANKRD46	3.027027027
MYRFL1	3.31E-25	0.5466308	0.171	0.071	7.98E-21	2.3	MYRFL	2.408450704
RP11-571L19.7	3.48E-25	0.221325	0.038	0.006	8.38E-21	2.3	RP11-571L1	6.333333333
CCDC343	3.68E-25	0.3323405	0.079	0.02	8.87E-21	2.3	CCDC34	3.95
RP11-1035H13.2	3.90E-25	0.2098189	0.049	0.009	9.40E-21	2.3	RP11-1035	5.444444444
GADD45B2	4.06E-25	0.7341445	0.225	0.106	9.78E-21	2.3	GADD45B	2.122641509
GPS22	5.23E-25	0.6164694	0.299	0.167	1.26E-20	2.3	GPS2	1.790419162
DPH1	5.26E-25	0.1493519	0.029	0.003	1.27E-20	2.3	DPH1	9.666666667
HSPA53	6.79E-25	0.3625364	0.61	0.446	1.64E-20	2.3	HSPA5	1.367713004
SLC7A22	6.90E-25	0.4698736	0.475	0.296	1.66E-20	2.3	SLC7A2	1.60472973
C18orf321	7.31E-25	0.5656618	0.281	0.151	1.76E-20	2.3	C18orf32	1.860927152
ZBTB102	7.88E-25	0.6090618	0.402	0.253	1.90E-20	2.3	ZBTB10	1.588932806
PLK21	8.34E-25	0.6513324	0.254	0.131	2.01E-20	2.3	PLK2	1.938931298
DNAH7	8.48E-25	0.3952671	0.097	0.029	2.05E-20	2.3	DNAH7	3.344827586
AFF32	8.88E-25	0.3101498	0.527	0.334	2.14E-20	2.3	AFF3	1.577844311
RP11-90K6.11	8.98E-25	0.2006578	0.053	0.011	2.17E-20	2.3	RP11-90K6	4.818181818
RP11-405A12.2	1.29E-24	0.2849516	0.072	0.018	3.11E-20	2.3	RP11-405A	4
TRIM242	1.42E-24	0.5342428	0.184	0.081	3.44E-20	2.3	TRIM24	2.271604938
EGOT2	1.61E-24	0.558397	0.23	0.113	3.89E-20	2.3	EGOT	2.03539823
EMP21	1.69E-24	0.4802115	0.242	0.119	4.07E-20	2.3	EMP2	2.033613445
RP5-940J5.61	1.79E-24	0.2482277	0.067	0.016	4.33E-20	2.3	RP5-940J5.	4.1875
PLEKHB22	1.89E-24	0.5188421	0.294	0.16	4.56E-20	2.3	PLEKHB2	1.8375
TTC39A2	2.16E-24	0.4633536	0.132	0.048	5.21E-20	2.3	TTC39A	2.75
PBDC11	2.65E-24	0.6474123	0.25	0.131	6.38E-20	2.3	PBDC1	1.908396947
EIF4E4	2.84E-24	0.6569817	0.511	0.379	6.85E-20	2.3	EIF4E	1.34828496
TSPAN132	3.34E-24	0.4951463	0.226	0.11	8.05E-20	2.3	TSPAN13	2.054545455
EIF1AX2	3.90E-24	0.545238	0.378	0.237	9.40E-20	2.3	EIF1AX	1.594936709
SERF22	3.90E-24	0.5097324	0.539	0.391	9.41E-20	2.3	SERF2	1.378516624
IQUB1	4.00E-24	0.2729558	0.058	0.012	9.63E-20	2.3	IQUB	4.833333333
TRNP1	4.10E-24	0.236676	0.053	0.011	9.88E-20	2.3	TRNP1	4.818181818
ELF12	4.94E-24	0.5018722	0.663	0.526	1.19E-19	2.3	ELF1	1.260456274

AHNAK	5.18E-24	0.5934921	0.489	0.353	1.25E-19	2.3	AHNAK	1.385269122
SON2	5.62E-24	0.5213918	0.765	0.677	1.36E-19	2.3	SON	1.129985229
EFNA12	7.21E-24	0.7009848	0.375	0.236	1.74E-19	2.3	EFNA1	1.588983051
SAP182	8.16E-24	0.551816	0.663	0.569	1.97E-19	2.3	SAP18	1.165202109
GYS1	8.21E-24	0.4667847	0.143	0.056	1.98E-19	2.3	GYS1	2.553571429
AC002117.12	8.65E-24	0.2689941	0.072	0.018	2.09E-19	2.3	AC002117.	4
ZFAND52	9.86E-24	0.6521568	0.555	0.432	2.38E-19	2.3	ZFAND5	1.284722222
PHTF1	1.06E-23	0.4569307	0.143	0.056	2.57E-19	2.3	PHTF1	2.553571429
CPEB41	1.35E-23	0.6546083	0.395	0.258	3.26E-19	2.3	CPEB4	1.531007752
BCOR2	1.39E-23	0.5145685	0.657	0.509	3.34E-19	2.3	BCOR	1.290766208
SRF1	1.46E-23	0.2496129	0.077	0.021	3.52E-19	2.3	SRF	3.666666667
CTD-2336O2.12	1.63E-23	0.1595494	0.046	0.009	3.92E-19	2.3	CTD-2336C	5.111111111
ATP1A3	1.75E-23	0.2281623	0.028	0.003	4.21E-19	2.3	ATP1A3	9.333333333
RP1-288H2.2	1.89E-23	0.1566762	0.018	0.001	4.55E-19	2.3	RP1-288H2	18
SLC8A2	2.13E-23	0.1021331	0.028	0.003	5.14E-19	2.3	SLC8A2	9.333333333
NEDD93	2.25E-23	0.5247005	0.514	0.368	5.43E-19	2.3	NEDD9	1.39673913
SOWAHC	2.27E-23	0.3869773	0.118	0.041	5.47E-19	2.3	SOWAHC	2.87804878
SELPLG1	2.52E-23	0.1857398	0.035	0.005	6.09E-19	2.3	SELPLG	7
TMSB102	2.62E-23	0.4428887	0.815	0.731	6.32E-19	2.3	TMSB10	1.114911081
TRPM3	2.67E-23	0.5069227	0.105	0.035	6.44E-19	2.3	TRPM3	3
IRX31	2.77E-23	0.4665126	0.192	0.088	6.67E-19	2.3	IRX3	2.181818182
VIT1	2.81E-23	0.1691028	0.044	0.008	6.78E-19	2.3	VIT	5.5
GIPC21	2.94E-23	0.4203972	0.104	0.034	7.09E-19	2.3	GIPC2	3.058823529
NRIP3	3.07E-23	0.3526784	0.119	0.042	7.40E-19	2.3	NRIP3	2.833333333
GSR1	3.21E-23	0.336601	0.098	0.031	7.75E-19	2.3	GSR	3.161290323
OST42	4.25E-23	0.5958194	0.532	0.412	1.02E-18	2.3	OST4	1.291262136
FOS2	4.50E-23	1.257253	0.355	0.228	1.09E-18	2.3	FOS	1.557017544
FNDC71	4.78E-23	0.1292188	0.035	0.005	1.15E-18	2.3	FNDC7	7
OTOA1	5.13E-23	0.1299226	0.029	0.004	1.24E-18	2.3	OTOA	7.25
MORN23	6.08E-23	0.4222378	0.147	0.059	1.47E-18	2.3	MORN2	2.491525424
MORC4	6.31E-23	0.3265924	0.086	0.025	1.52E-18	2.3	MORC4	3.44
SNU132	6.36E-23	0.496867	0.601	0.473	1.53E-18	2.3	SNU13	1.270613108
TRANK11	6.75E-23	0.2314667	0.053	0.011	1.63E-18	2.3	TRANK1	4.818181818
MTFR22	7.00E-23	0.1582625	0.049	0.01	1.69E-18	2.3	MTFR2	4.9
SKP14	7.44E-23	0.4275895	0.857	0.805	1.79E-18	2.3	SKP1	1.064596273
POLR2A	8.34E-23	0.7576959	0.288	0.164	2.01E-18	2.3	POLR2A	1.756097561
DAND5	8.57E-23	0.1138784	0.018	0.001	2.07E-18	2.3	DAND5	18
SEPP13	9.69E-23	0.6142014	0.449	0.309	2.34E-18	2.3	SEPP1	1.453074434
ATP13A42	1.00E-22	0.3309685	0.101	0.033	2.41E-18	2.3	ATP13A4	3.060606061
RP11-252E2.21	1.55E-22	0.1753554	0.052	0.011	3.75E-18	2.3	RP11-252E2	4.727272727
NDUFA122	1.68E-22	0.5687805	0.334	0.208	4.06E-18	2.3	NDUFA12	1.605769231
RHOH1	1.71E-22	0.3364968	0.097	0.031	4.12E-18	2.3	RHOH	3.129032258
KRT181	1.93E-22	0.4449128	0.218	0.106	4.65E-18	2.3	KRT18	2.056603774
PABPC15	2.12E-22	0.4833467	0.795	0.728	5.11E-18	2.3	PABPC1	1.092032967
ARL4A2	2.21E-22	0.6023991	0.226	0.115	5.32E-18	2.3	ARL4A	1.965217391
AC016723.4	2.23E-22	0.148067	0.041	0.007	5.38E-18	2.3	AC016723.4	5.857142857
ANKRD261	3.06E-22	0.4976582	0.169	0.075	7.37E-18	2.3	ANKRD26	2.253333333
AC008686.1	3.68E-22	0.1515907	0.029	0.004	8.88E-18	2.3	AC008686.	7.25
HGD2	4.11E-22	0.2116874	0.063	0.015	9.91E-18	2.3	HGD	4.2
LDHAL6A	4.15E-22	0.1089794	0.025	0.003	1.00E-17	2.3	LDHAL6A	8.333333333
SLC9C21	4.27E-22	0.2127852	0.042	0.008	1.03E-17	2.3	SLC9C2	5.25
CDC423	4.31E-22	0.480661	0.785	0.714	1.04E-17	2.3	CDC42	1.099439776

SCGB2A22	4.32E-22	1.273157	0.426	0.27	1.04E-17	2.3	SCGB2A2	1.577777778
GPLD1	5.01E-22	0.1434754	0.041	0.007	1.21E-17	2.3	GPLD1	5.857142857
MRPL242	5.32E-22	0.3869664	0.152	0.063	1.28E-17	2.3	MRPL24	2.412698413
C19orf38	5.99E-22	0.1790203	0.035	0.006	1.44E-17	2.3	C19orf38	5.833333333
RASEF2	6.32E-22	0.4825827	0.42	0.269	1.52E-17	2.3	RASEF	1.56133829
NFS1	7.54E-22	0.1448901	0.028	0.004	1.82E-17	2.3	NFS1	7
BTRC2	9.31E-22	0.6261262	0.396	0.26	2.24E-17	2.3	BTRC	1.523076923
C11orf651	1.01E-21	0.284135	0.067	0.018	2.44E-17	2.3	C11orf65	3.722222222
AFMID	1.32E-21	0.7105576	0.229	0.122	3.18E-17	2.3	AFMID	1.87704918
RP11-428J1.4	1.34E-21	0.1008521	0.027	0.003	3.23E-17	2.3	RP11-428J1	9
METTL7A2	1.40E-21	0.3915667	0.131	0.051	3.38E-17	2.3	METTL7A	2.568627451
ZNF34	1.45E-21	0.1098262	0.029	0.004	3.50E-17	2.3	ZNF34	7.25
FAM117B	1.63E-21	0.3733859	0.115	0.042	3.93E-17	2.3	FAM117B	2.738095238
PRR15L1	1.70E-21	0.3944687	0.098	0.033	4.09E-17	2.3	PRR15L	2.96969697
MUC41	1.75E-21	0.1360824	0.041	0.007	4.21E-17	2.3	MUC4	5.857142857
NDUFB91	1.86E-21	0.5467791	0.346	0.219	4.49E-17	2.3	NDUFB9	1.579908676
TFF12	2.30E-21	0.6183353	0.213	0.104	5.54E-17	2.3	TFF1	2.048076923
TBX32	2.35E-21	0.4297608	0.424	0.267	5.66E-17	2.3	TBX3	1.588014981
PGRMC12	2.48E-21	0.366187	0.181	0.083	5.99E-17	2.3	PGRMC1	2.180722892
PTGER4	2.93E-21	0.1988849	0.042	0.008	7.06E-17	2.3	PTGER4	5.25
LINC003931	2.95E-21	0.2501848	0.063	0.016	7.11E-17	2.3	LINC00393	3.9375
XG1	3.03E-21	0.2376929	0.052	0.011	7.32E-17	2.3	XG	4.727272727
ITGA102	3.05E-21	0.354878	0.132	0.052	7.36E-17	2.3	ITGA10	2.538461538
RP11-21G20.3	3.17E-21	0.1142407	0.01	0	7.65E-17	2.3	RP11-21G2	#DIV/0!
CDKL12	3.89E-21	0.4101716	0.133	0.053	9.39E-17	2.3	CDKL1	2.509433962
CYB5D1	4.31E-21	0.1781427	0.041	0.008	1.04E-16	2.3	CYB5D1	5.125
FOXA12	4.61E-21	0.4646012	0.226	0.116	1.11E-16	2.3	FOXA1	1.948275862
RP11-392P7.6	4.93E-21	0.4119623	0.125	0.048	1.19E-16	2.3	RP11-392P	2.604166667
TSPAN61	5.58E-21	0.5505874	0.294	0.174	1.35E-16	2.3	TSPAN6	1.689655172
MRC21	5.98E-21	0.1715122	0.049	0.01	1.44E-16	2.3	MRC2	4.9
SUGCT1	6.21E-21	0.448445	0.115	0.043	1.50E-16	2.3	SUGCT	2.674418605
NRXN32	6.81E-21	0.4895419	0.237	0.125	1.64E-16	2.3	NRXN3	1.896
PIGK1	7.16E-21	0.4268925	0.176	0.081	1.73E-16	2.3	PIGK	2.172839506
THUMPD3-AS13	7.27E-21	0.867822	0.336	0.222	1.75E-16	2.3	THUMPD3-	1.513513514
CUL9	7.85E-21	0.1984684	0.049	0.011	1.89E-16	2.3	CUL9	4.454545455
LINC00935	7.87E-21	0.2750717	0.039	0.007	1.90E-16	2.3	LINC00935	5.571428571
TPT13	8.12E-21	0.252837	0.914	0.85	1.96E-16	2.3	TPT1	1.075294118
PPP1R101	1.01E-20	0.6241642	0.215	0.112	2.44E-16	2.3	PPP1R10	1.919642857
CSRNP11	1.18E-20	0.3619894	0.118	0.044	2.86E-16	2.3	CSRNP1	2.681818182
RP11-863P13.51	1.20E-20	0.1425345	0.031	0.005	2.89E-16	2.3	RP11-863P	6.2
MYH10	1.24E-20	0.4741973	0.152	0.065	2.98E-16	2.3	MYH10	2.338461538
PPIA4	1.37E-20	0.4049292	0.857	0.803	3.29E-16	2.3	PPIA	1.067247821
IER2	1.37E-20	0.4688982	0.174	0.081	3.30E-16	2.3	IER2	2.148148148
IL1R11	1.76E-20	0.4060011	0.159	0.07	4.25E-16	2.3	IL1R1	2.271428571
MLPH2	2.35E-20	0.4242237	0.16	0.071	5.65E-16	2.3	MLPH	2.253521127
ZNF788	2.53E-20	0.1816836	0.052	0.012	6.11E-16	2.3	ZNF788	4.333333333
ATCAY	2.83E-20	0.22064	0.029	0.004	6.81E-16	2.3	ATCAY	7.25
RP11-486P11.1	2.91E-20	0.1571211	0.042	0.008	7.02E-16	2.3	RP11-486P	5.25
NEK8	3.01E-20	0.2075223	0.052	0.012	7.25E-16	2.3	NEK8	4.333333333
NPFFR22	3.56E-20	0.2633768	0.08	0.024	8.59E-16	2.3	NPFFR2	3.333333333
ATP5L5	4.02E-20	0.3824179	0.847	0.809	9.69E-16	2.3	ATP5L	1.04697157
RP11-353N4.5	4.35E-20	0.1317395	0.025	0.003	1.05E-15	2.3	RP11-353N	8.333333333

EIF4G22	4.51E-20	0.5796021	0.51	0.39	1.09E-15	2.3	EIF4G2	1.307692308
Sep-42	4.54E-20	0.2323604	0.065	0.017	1.09E-15	2.3	Sep-04	3.823529412
RAP2A	4.69E-20	0.2906502	0.098	0.034	1.13E-15	2.3	RAP2A	2.882352941
DAD13	5.22E-20	0.5068956	0.601	0.494	1.26E-15	2.3	DAD1	1.21659919
RP11-18H7.1	5.24E-20	0.3061024	0.09	0.03	1.26E-15	2.3	RP11-18H7	3
GNG4	5.65E-20	0.1359432	0.037	0.006	1.36E-15	2.3	GNG4	6.166666667
HSBP11	5.78E-20	0.4823714	0.646	0.553	1.39E-15	2.3	HSBP1	1.168173599
HSPB111	6.46E-20	0.4057061	0.201	0.099	1.56E-15	2.3	HSPB11	2.03030303
PER3	6.79E-20	0.3410915	0.103	0.037	1.64E-15	2.3	PER3	2.783783784
GTF2H53	7.13E-20	0.6278991	0.329	0.213	1.72E-15	2.3	GTF2H5	1.544600939
CATSPERD	7.19E-20	0.2864409	0.066	0.018	1.73E-15	2.3	CATSPERD	3.666666667
EIF5AL12	7.20E-20	0.3243672	0.119	0.046	1.74E-15	2.3	EIF5AL1	2.586956522
KCNB1	8.32E-20	0.1516343	0.027	0.004	2.01E-15	2.3	KCNB1	6.75
RP11-589M4.3	8.88E-20	0.1215577	0.027	0.004	2.14E-15	2.3	RP11-589M	6.75
RP3-393E18.21	9.89E-20	0.1144572	0.029	0.004	2.38E-15	2.3	RP3-393E1	7.25
WDR611	1.08E-19	0.4540903	0.215	0.111	2.60E-15	2.3	WDR61	1.936936937
TMEM2583	1.13E-19	0.442258	0.687	0.597	2.73E-15	2.3	TMEM258	1.150753769
RASGEF1B3	1.24E-19	0.3814708	0.903	0.892	3.00E-15	2.3	RASGEF1B	1.012331839
RP11-554F20.11	1.38E-19	0.389622	0.131	0.052	3.33E-15	2.3	RP11-554F	2.519230769
TKTL1	1.54E-19	0.1624343	0.032	0.005	3.72E-15	2.3	TKTL1	6.4
ELOVL21	1.62E-19	0.2021723	0.045	0.01	3.91E-15	2.3	ELOVL2	4.5
ACADM1	1.75E-19	0.4222012	0.173	0.081	4.21E-15	2.3	ACADM	2.135802469
MAPRE12	1.77E-19	0.5543106	0.272	0.159	4.28E-15	2.3	MAPRE1	1.710691824
JUNB1	1.78E-19	0.6649096	0.105	0.038	4.29E-15	2.3	JUNB	2.763157895
RBFOX24	1.95E-19	0.3690892	0.954	0.952	4.71E-15	2.3	RBFOX2	1.00210084
HNRNPK3	2.32E-19	0.5348702	0.555	0.447	5.59E-15	2.3	HNRNPK	1.241610738
RP5-1085F17.4	2.41E-19	0.3267485	0.093	0.032	5.82E-15	2.3	RP5-1085F	2.90625
C3orf141	2.69E-19	0.4756773	0.213	0.111	6.48E-15	2.3	C3orf14	1.918918919
RP11-60A24.3	2.71E-19	0.1385563	0.037	0.007	6.53E-15	2.3	RP11-60A2	5.285714286
KLRD11	3.14E-19	0.449617	0.281	0.16	7.57E-15	2.3	KLRD1	1.75625
TCEAL42	3.19E-19	0.606394	0.341	0.223	7.70E-15	2.3	TCEAL4	1.529147982
AC092620.32	3.67E-19	0.1419807	0.041	0.008	8.86E-15	2.3	AC092620.	5.125
TMEM2302	4.01E-19	0.4221971	0.178	0.086	9.67E-15	2.3	TMEM230	2.069767442
MEP1B2	4.09E-19	0.1845979	0.034	0.006	9.86E-15	2.3	MEP1B	5.666666667
S100A142	4.18E-19	0.3329778	0.815	0.732	1.01E-14	2.3	S100A14	1.113387978
GSTM42	4.18E-19	0.1447805	0.041	0.008	1.01E-14	2.3	GSTM4	5.125
CDH16	4.65E-19	0.4086551	0.701	0.604	1.12E-14	2.3	CDH1	1.160596026
ACAA21	5.10E-19	0.2426158	0.096	0.033	1.23E-14	2.3	ACAA2	2.909090909
NR1H41	5.15E-19	0.1828423	0.052	0.012	1.24E-14	2.3	NR1H4	4.333333333
RPS295	5.45E-19	0.3155827	0.92	0.925	1.32E-14	2.3	RPS29	0.994594595
LMOD21	6.93E-19	0.1287079	0.025	0.003	1.67E-14	2.3	LMOD2	8.333333333
RP11-434D9.1	7.12E-19	0.3378913	0.084	0.028	1.72E-14	2.3	RP11-434D	3
USP201	7.86E-19	0.2582374	0.067	0.019	1.89E-14	2.3	USP20	3.526315789
CTNS1	8.02E-19	0.1766381	0.059	0.016	1.93E-14	2.3	CTNS	3.6875
RCL12	9.71E-19	0.5036224	0.15	0.068	2.34E-14	2.3	RCL1	2.205882353
SERPINA112	1.04E-18	0.2626695	0.096	0.034	2.52E-14	2.3	SERPINA11	2.823529412
YWHAE2	1.05E-18	0.4201154	0.721	0.626	2.53E-14	2.3	YWHAE	1.151757188
RPL37A6	1.11E-18	0.2757272	0.98	0.975	2.67E-14	2.3	RPL37A	1.005128205
MIR4458HG1	1.13E-18	0.3576634	0.149	0.066	2.71E-14	2.3	MIR4458H(	2.257575758
DENND2C1	1.13E-18	0.3376979	0.097	0.034	2.73E-14	2.3	DENND2C	2.852941176
SYT2	1.22E-18	0.1338111	0.045	0.01	2.94E-14	2.3	SYT2	4.5
RP11-227G15.121	1.27E-18	0.1445773	0.037	0.007	3.07E-14	2.3	RP11-227G	5.285714286

NIT22	1.34E-18	0.4529449	0.201	0.104	3.23E-14	2.3	NIT2	1.932692308
UGDH-AS12	1.39E-18	0.6476879	0.206	0.108	3.36E-14	2.3	UGDH-AS1	1.907407407
C12orf652	1.51E-18	0.498326	0.125	0.052	3.63E-14	2.3	C12orf65	2.403846154
SHOC21	1.97E-18	0.5480205	0.376	0.257	4.76E-14	2.3	SHOC2	1.463035019
MGST24	1.99E-18	0.488598	0.263	0.152	4.81E-14	2.3	MGST2	1.730263158
PDK42	2.05E-18	0.4712234	0.166	0.078	4.94E-14	2.3	PDK4	2.128205128
MMRN2	2.14E-18	0.2232081	0.059	0.016	5.16E-14	2.3	MMRN2	3.6875
ADK1	2.28E-18	0.660445	0.574	0.48	5.49E-14	2.3	ADK	1.195833333
C2CD4A2	2.35E-18	0.3587916	0.118	0.047	5.66E-14	2.3	C2CD4A	2.510638298
COQ31	2.38E-18	0.2432242	0.073	0.022	5.74E-14	2.3	COQ3	3.318181818
REL2	2.40E-18	0.4830421	0.43	0.297	5.78E-14	2.3	REL	1.447811448
RP11-115N4.1	2.44E-18	0.1233677	0.024	0.003	5.90E-14	2.3	RP11-115N	8
CCR61	2.75E-18	0.1414045	0.024	0.003	6.62E-14	2.3	CCR6	8
LDLR3	3.05E-18	0.5419086	0.278	0.168	7.36E-14	2.3	LDLR	1.654761905
H2AFZ	3.33E-18	0.6110856	0.733	0.669	8.02E-14	2.3	H2AFZ	1.095665172
METTL24	3.64E-18	0.1515163	0.021	0.003	8.77E-14	2.3	METTL24	7
RSRC23	4.01E-18	0.5027215	0.688	0.624	9.66E-14	2.3	RSRC2	1.102564103
CLDN83	4.72E-18	0.4367518	0.129	0.055	1.14E-13	2.3	CLDN8	2.345454545
SLC45A1	4.95E-18	0.1040443	0.022	0.003	1.19E-13	2.3	SLC45A1	7.333333333
SPAG93	4.97E-18	0.5852058	0.459	0.343	1.20E-13	2.3	SPAG9	1.33819242
DECR12	5.32E-18	0.4764218	0.265	0.156	1.28E-13	2.3	DECR1	1.698717949
WDR351	5.76E-18	0.2811861	0.076	0.024	1.39E-13	2.3	WDR35	3.166666667
EIF11	6.57E-18	0.4332291	0.596	0.478	1.58E-13	2.3	EIF1	1.246861925
AC005355.11	7.69E-18	0.1521099	0.041	0.009	1.85E-13	2.3	AC005355.	4.555555556
SCGB1D22	7.86E-18	0.5233435	0.222	0.117	1.90E-13	2.3	SCGB1D2	1.897435897
PILRA	8.67E-18	0.2584481	0.072	0.022	2.09E-13	2.3	PILRA	3.272727273
LINC006071	9.21E-18	0.3719765	0.1	0.037	2.22E-13	2.3	LINC00607	2.702702703
HIST1H2BE	1.14E-17	0.230235	0.069	0.021	2.75E-13	2.3	HIST1H2BE	3.285714286
NEK10	1.22E-17	0.5016687	0.256	0.149	2.93E-13	2.3	NEK10	1.718120805
TESK21	1.27E-17	0.5149772	0.249	0.143	3.07E-13	2.3	TESK2	1.741258741
ATP5G24	1.29E-17	0.3650837	0.801	0.736	3.11E-13	2.3	ATP5G2	1.088315217
ADAMTS41	1.35E-17	0.3280724	0.121	0.05	3.25E-13	2.3	ADAMTS4	2.42
BZW1	1.39E-17	0.5150333	0.393	0.275	3.35E-13	2.3	BZW1	1.429090909
CTD-2192J16.26	1.41E-17	0.1257277	0.025	0.004	3.39E-13	2.3	CTD-2192J:	6.25
HSH2D	1.64E-17	0.1060034	0.027	0.004	3.95E-13	2.3	HSH2D	6.75
ZBTB182	1.70E-17	0.4021287	0.153	0.071	4.10E-13	2.3	ZBTB18	2.154929577
ITGB1BP1	1.71E-17	0.2388184	0.081	0.027	4.12E-13	2.3	ITGB1BP1	3
CDK191	1.71E-17	0.5684297	0.428	0.317	4.13E-13	2.3	CDK19	1.350157729
CDH171	1.96E-17	0.1142018	0.022	0.003	4.72E-13	2.3	CDH17	7.333333333
RP4-593C16.41	2.10E-17	0.1299285	0.035	0.007	5.07E-13	2.3	RP4-593C1	5
SPA171	2.20E-17	0.2879891	0.097	0.036	5.32E-13	2.3	SPA17	2.694444444
B3GALNT12	2.67E-17	0.1508946	0.049	0.012	6.44E-13	2.3	B3GALNT1	4.083333333
ATL1	3.49E-17	0.3290417	0.133	0.058	8.41E-13	2.3	ATL1	2.293103448
SIK11	3.82E-17	0.1479719	0.035	0.007	9.20E-13	2.3	SIK1	5
KLF54	3.95E-17	0.5229222	0.272	0.162	9.53E-13	2.3	KLF5	1.679012346
MAN1A11	3.96E-17	0.5215461	0.343	0.226	9.56E-13	2.3	MAN1A1	1.517699115
MYL12A1	4.30E-17	0.3037548	0.909	0.866	1.04E-12	2.3	MYL12A	1.04965358
FTO2	5.23E-17	0.5302789	0.368	0.252	1.26E-12	2.3	FTO	1.46031746
RPL385	5.54E-17	0.29913	0.921	0.891	1.34E-12	2.3	RPL38	1.033670034
TMPRSS9	5.68E-17	0.2626671	0.056	0.015	1.37E-12	2.3	TMPRSS9	3.733333333
ERLIN21	5.76E-17	0.3320187	0.111	0.045	1.39E-12	2.3	ERLIN2	2.466666667
EID11	5.91E-17	0.4260415	0.162	0.079	1.42E-12	2.3	EID1	2.050632911

TGFB31	6.10E-17	0.2359715	0.097	0.036	1.47E-12	2.3	TGFB3	2.694444444
PKD2L2	6.21E-17	0.1978402	0.072	0.023	1.50E-12	2.3	PKD2L2	3.130434783
ATP6V1G12	6.71E-17	0.532171	0.483	0.388	1.62E-12	2.3	ATP6V1G1	1.244845361
ARID4A2	6.72E-17	0.6383759	0.289	0.185	1.62E-12	2.3	ARID4A	1.562162162
PEBP12	7.32E-17	0.4583273	0.278	0.171	1.77E-12	2.3	PEBP1	1.625730994
CHMP31	7.78E-17	0.3935542	0.16	0.078	1.88E-12	2.3	CHMP3	2.051282051
ZFP361	8.16E-17	0.7129153	0.209	0.115	1.97E-12	2.3	ZFP36	1.817391304
RP11-761B3.1	8.45E-17	0.1641272	0.059	0.017	2.04E-12	2.3	RP11-761B	3.470588235
LINC012712	8.88E-17	0.3129256	0.053	0.014	2.14E-12	2.3	LINC01271	3.785714286
SYT11	9.29E-17	0.2331808	0.074	0.024	2.24E-12	2.3	SYT1	3.083333333
MAP4K2	9.74E-17	0.137514	0.035	0.007	2.35E-12	2.3	MAP4K2	5
RP11-364L4.31	1.12E-16	0.2599243	0.034	0.007	2.70E-12	2.3	RP11-364L4	4.857142857
SMARCA22	1.13E-16	0.6043701	0.412	0.308	2.71E-12	2.3	SMARCA2	1.337662338
MN11	1.16E-16	0.1859973	0.051	0.013	2.80E-12	2.3	MN1	3.923076923
ACOT132	1.17E-16	0.3798678	0.091	0.034	2.82E-12	2.3	ACOT13	2.676470588
SLC9A3R11	1.51E-16	0.4107873	0.181	0.094	3.63E-12	2.3	SLC9A3R1	1.925531915
ST3GAL3	1.57E-16	0.2941783	0.096	0.036	3.79E-12	2.3	ST3GAL3	2.666666667
AC017060.11	1.60E-16	0.15561	0.038	0.008	3.85E-12	2.3	AC017060.	4.75
ALG81	1.63E-16	0.2927156	0.108	0.044	3.94E-12	2.3	ALG8	2.454545455
LSMEM11	1.65E-16	0.4125558	0.149	0.069	3.98E-12	2.3	LSMEM1	2.15942029
KLF4	1.73E-16	0.3841075	0.205	0.11	4.17E-12	2.3	KLF4	1.863636364
NCALD3	1.76E-16	0.4014549	0.226	0.126	4.24E-12	2.3	NCALD	1.793650794
RHEBL11	2.21E-16	0.1301871	0.031	0.006	5.32E-12	2.3	RHEBL1	5.166666667
DYX1C11	2.26E-16	0.2273482	0.074	0.025	5.46E-12	2.3	DYX1C1	2.96
AEBP21	2.39E-16	0.5581955	0.362	0.255	5.75E-12	2.3	AEBP2	1.419607843
CTB-12O2.11	2.46E-16	0.2845231	0.067	0.021	5.93E-12	2.3	CTB-12O2.1	3.19047619
C4orf331	3.44E-16	0.1663243	0.052	0.014	8.30E-12	2.3	C4orf33	3.714285714
MPDU1	3.63E-16	0.2107948	0.056	0.016	8.75E-12	2.3	MPDU1	3.5
COL12A11	4.20E-16	0.3757026	0.115	0.048	1.01E-11	2.3	COL12A1	2.395833333
CHAC2	4.95E-16	0.1240357	0.034	0.007	1.19E-11	2.3	CHAC2	4.857142857
LARP1B2	5.63E-16	0.4534414	0.331	0.215	1.36E-11	2.3	LARP1B	1.539534884
TWSG11	5.65E-16	0.4177935	0.16	0.08	1.36E-11	2.3	TWSG1	2
AUNIP	5.78E-16	0.1035512	0.021	0.003	1.39E-11	2.3	AUNIP	7
SLAIN12	6.52E-16	0.2329	0.059	0.017	1.57E-11	2.3	SLAIN1	3.470588235
FLT1	7.11E-16	0.1613542	0.046	0.012	1.71E-11	2.3	FLT1	3.833333333
GABARAP2	7.41E-16	0.4702915	0.476	0.359	1.79E-11	2.3	GABARAP	1.325905292
FYB1	7.47E-16	0.1860247	0.035	0.007	1.80E-11	2.3	FYB	5
CTD-3088G3.83	7.50E-16	0.374468	0.121	0.052	1.81E-11	2.3	CTD-3088G	2.326923077
RP11-739N20.2	8.80E-16	0.2272314	0.072	0.024	2.12E-11	2.3	RP11-739N	3
CASC12	9.69E-16	0.2728621	0.086	0.031	2.34E-11	2.3	CASC1	2.774193548
AHCY1	1.08E-15	0.3259362	0.128	0.058	2.61E-11	2.3	AHCY	2.206896552
ANKRD501	1.14E-15	0.4134079	0.162	0.081	2.76E-11	2.3	ANKRD50	2
ZNF6891	1.22E-15	0.2510063	0.052	0.014	2.94E-11	2.3	ZNF689	3.714285714
RNF112	1.27E-15	0.4120032	0.357	0.244	3.07E-11	2.3	RNF11	1.463114754
CAPN12	1.38E-15	0.1201825	0.027	0.005	3.32E-11	2.3	CAPN12	5.4
BCAS4	1.39E-15	0.2460434	0.083	0.03	3.34E-11	2.3	BCAS4	2.766666667
NCAPG2	1.51E-15	0.1950933	0.062	0.019	3.63E-11	2.3	NCAPG2	3.263157895
AC142293.32	1.53E-15	0.1511902	0.038	0.008	3.69E-11	2.3	AC142293.3	4.75
POLR2K1	1.62E-15	0.4484021	0.475	0.367	3.91E-11	2.3	POLR2K	1.294277929
TGIF11	1.71E-15	0.4562052	0.329	0.218	4.12E-11	2.3	TGIF1	1.509174312
MT1A2	1.71E-15	0.2377779	0.056	0.016	4.12E-11	2.3	MT1A	3.5
TMEM2542	2.12E-15	0.2550905	0.104	0.042	5.10E-11	2.3	TMEM254	2.476190476

RP11-813I20.21	2.12E-15	0.2034212	0.069	0.023	5.12E-11	2.3	RP11-813I2	3
ORMDL22	2.30E-15	0.4394311	0.258	0.159	5.54E-11	2.3	ORMDL2	1.622641509
MUC61	2.30E-15	0.1045582	0.021	0.003	5.55E-11	2.3	MUC6	7
CD832	2.40E-15	0.2866576	0.142	0.067	5.79E-11	2.3	CD83	2.119402985
MED132	2.65E-15	0.5519343	0.493	0.397	6.39E-11	2.3	MED13	1.241813602
AZIN1-AS1	2.84E-15	0.3623357	0.117	0.051	6.84E-11	2.3	AZIN1-AS1	2.294117647
WNT9A1	3.00E-15	0.3658341	0.081	0.03	7.24E-11	2.3	WNT9A	2.7
S100A161	3.08E-15	0.4315764	0.254	0.153	7.42E-11	2.3	S100A16	1.660130719
ELAVL2	3.22E-15	0.1182622	0.027	0.005	7.77E-11	2.3	ELAVL2	5.4
ANKRD35	3.28E-15	0.107843	0.031	0.006	7.91E-11	2.3	ANKRD35	5.166666667
LMCD1-AS11	3.47E-15	0.3243269	0.107	0.045	8.37E-11	2.3	LMCD1-AS1	2.377777778
RP11-60I3.42	4.24E-15	0.2155203	0.07	0.024	1.02E-10	2.3	RP11-60I3.4	2.916666667
PECAM1	4.37E-15	0.1553045	0.041	0.01	1.05E-10	2.3	PECAM1	4.1
VEZF1	4.56E-15	0.3954702	0.178	0.095	1.10E-10	2.3	VEZF1	1.873684211
SPTAN11	5.00E-15	0.5229136	0.326	0.22	1.21E-10	2.3	SPTAN1	1.481818182
HPGD2	5.71E-15	0.1391264	0.044	0.011	1.38E-10	2.3	HPGD	4
RP11-879F14.1	6.01E-15	0.1447344	0.038	0.009	1.45E-10	2.3	RP11-879F1	4.222222222
LNP11	6.09E-15	0.1084209	0.031	0.006	1.47E-10	2.3	LNP1	5.166666667
IDH13	6.87E-15	0.4212596	0.156	0.08	1.66E-10	2.3	IDH1	1.95
RAN3	6.91E-15	0.4280162	0.614	0.527	1.67E-10	2.3	RAN	1.165085389
NR2C11	7.82E-15	0.2977539	0.129	0.059	1.88E-10	2.3	NR2C1	2.186440678
BTG21	7.95E-15	0.6320477	0.128	0.06	1.92E-10	2.3	BTG2	2.133333333
VWA9	7.98E-15	0.2450848	0.072	0.025	1.93E-10	2.3	VWA9	2.88
SH2D4B	8.33E-15	0.1062908	0.024	0.004	2.01E-10	2.3	SH2D4B	6
FLT32	8.86E-15	0.4585626	0.09	0.035	2.14E-10	2.3	FLT3	2.571428571
ARPC32	9.97E-15	0.4002704	0.712	0.66	2.40E-10	2.3	ARPC3	1.078787879
SCOC3	1.00E-14	0.4853133	0.337	0.238	2.42E-10	2.3	SCOC	1.415966387
UBXN82	1.07E-14	0.2682917	0.069	0.023	2.57E-10	2.3	UBXN8	3
ATOX12	1.15E-14	0.3940237	0.17	0.09	2.78E-10	2.3	ATOX1	1.888888889
RP1-313I6.121	1.24E-14	0.3786798	0.178	0.095	2.99E-10	2.3	RP1-313I6.	1.873684211
TXNDC171	1.29E-14	0.3476048	0.14	0.068	3.10E-10	2.3	TXNDC17	2.058823529
RAB302	1.30E-14	0.4204451	0.212	0.12	3.14E-10	2.3	RAB30	1.766666667
RP11-894P9.2	1.50E-14	0.1174156	0.017	0.002	3.62E-10	2.3	RP11-894P9	8.5
LINC007042	1.60E-14	0.2646373	0.076	0.027	3.87E-10	2.3	LINC00704	2.814814815
FAT1	1.82E-14	0.5230212	0.302	0.2	4.40E-10	2.3	FAT1	1.51
COX18	1.86E-14	0.2083951	0.056	0.017	4.48E-10	2.3	COX18	3.294117647
NUCB11	2.01E-14	0.2444398	0.073	0.026	4.86E-10	2.3	NUCB1	2.807692308
RABEPK	2.04E-14	0.1996672	0.056	0.017	4.91E-10	2.3	RABEPK	3.294117647
FGF121	2.12E-14	0.2870495	0.077	0.028	5.12E-10	2.3	FGF12	2.75
PTP4A1	2.13E-14	0.531019	0.386	0.282	5.14E-10	2.3	PTP4A1	1.368794326
RP11-277P12.203	2.20E-14	0.383283	0.139	0.067	5.30E-10	2.3	RP11-277P	2.074626866
MSMO12	2.23E-14	0.5329002	0.445	0.331	5.37E-10	2.3	MSMO1	1.344410876
RP11-613M10.91	2.28E-14	0.3799895	0.145	0.071	5.49E-10	2.3	RP11-613M	2.042253521
C9orf241	2.31E-14	0.1963506	0.059	0.018	5.57E-10	2.3	C9orf24	3.277777778
LAPTM4A2	2.50E-14	0.4232367	0.435	0.326	6.04E-10	2.3	LAPTM4A	1.334355828
PFDN13	2.54E-14	0.5279077	0.317	0.22	6.13E-10	2.3	PFDN1	1.440909091
PSMA42	2.72E-14	0.469397	0.528	0.438	6.56E-10	2.3	PSMA4	1.205479452
ARL6IP53	2.77E-14	0.4599626	0.567	0.487	6.69E-10	2.3	ARL6IP5	1.164271047
DNAJB92	2.79E-14	0.3750163	0.169	0.089	6.74E-10	2.3	DNAJB9	1.898876404
CRKL1	2.89E-14	0.3416148	0.152	0.076	6.96E-10	2.3	CRKL	2
ERBB2IP2	2.95E-14	0.3833666	0.531	0.426	7.11E-10	2.3	ERBB2IP	1.246478873
SLC28A12	3.18E-14	0.2086643	0.058	0.018	7.67E-10	2.3	SLC28A1	3.222222222

SKA3	3.22E-14	0.1253799	0.017	0.002	7.76E-10	2.3	SKA3	8.5
LFNG1	3.33E-14	0.1017913	0.024	0.004	8.02E-10	2.3	LFNG	6
ARSD1	3.90E-14	0.2951336	0.11	0.048	9.40E-10	2.3	ARSD	2.291666667
HYPK	3.97E-14	0.1663801	0.044	0.011	9.57E-10	2.3	HYPK	4
CUEDC1	4.01E-14	0.3498324	0.135	0.066	9.67E-10	2.3	CUEDC1	2.045454545
C5AR1	4.06E-14	0.1076182	0.027	0.005	9.78E-10	2.3	C5AR1	5.4
DNAJB11	4.44E-14	0.4638263	0.204	0.118	1.07E-09	2.3	DNAJB1	1.728813559
EIF5A2	4.59E-14	0.3552478	0.17	0.09	1.11E-09	2.3	EIF5A	1.888888889
RTN43	5.09E-14	0.3506365	0.711	0.653	1.23E-09	2.3	RTN4	1.088820827
TAOK31	5.46E-14	0.5176721	0.552	0.472	1.32E-09	2.3	TAOK3	1.169491525
RGMB	5.91E-14	0.2886696	0.094	0.039	1.43E-09	2.3	RGMB	2.41025641
RERE2	6.06E-14	0.4811884	0.64	0.569	1.46E-09	2.3	RERE	1.124780316
AC108938.5	6.70E-14	0.3819964	0.035	0.008	1.61E-09	2.3	AC108938.[illegible]	4.375
IGFBP22	8.13E-14	0.2733673	0.11	0.048	1.96E-09	2.3	IGFBP2	2.291666667
CD592	8.83E-14	0.3228735	0.921	0.881	2.13E-09	2.3	CD59	1.045402951
AC116366.61	9.49E-14	0.1552022	0.035	0.008	2.29E-09	2.3	AC116366.[illegible]	4.375
NCKAP5L	9.89E-14	0.1495145	0.039	0.01	2.38E-09	2.3	NCKAP5L	3.9
CBX4	1.02E-13	0.2110163	0.053	0.016	2.47E-09	2.3	CBX4	3.3125
PARD6B1	1.06E-13	0.488609	0.274	0.179	2.56E-09	2.3	PARD6B	1.530726257
IKZF4	1.06E-13	0.1747266	0.046	0.013	2.56E-09	2.3	IKZF4	3.538461538
GPR1371	1.07E-13	0.2490666	0.032	0.007	2.58E-09	2.3	GPR137	4.571428571
IMPG11	1.08E-13	0.1370627	0.031	0.007	2.60E-09	2.3	IMPG1	4.428571429
ADAM18	1.09E-13	0.1241652	0.032	0.007	2.64E-09	2.3	ADAM18	4.571428571
LINC012851	1.12E-13	0.1444553	0.029	0.006	2.69E-09	2.3	LINC01285	4.833333333
LINC01088	1.12E-13	0.1272365	0.027	0.005	2.70E-09	2.3	LINC01088	5.4
CAT2	1.15E-13	0.3050261	0.112	0.051	2.78E-09	2.3	CAT	2.196078431
SH3BP41	1.18E-13	0.4149467	0.253	0.157	2.84E-09	2.3	SH3BP4	1.611464968
PXDNL1	1.43E-13	0.2782895	0.088	0.036	3.45E-09	2.3	PXDNL	2.444444444
MRPL512	1.44E-13	0.4583188	0.51	0.433	3.48E-09	2.3	MRPL51	1.177829099
PHYHD1	1.47E-13	0.1650022	0.037	0.009	3.55E-09	2.3	PHYHD1	4.111111111
CMBL2	1.50E-13	0.1901266	0.066	0.023	3.61E-09	2.3	CMBL	2.869565217
MALAT12	1.59E-13	0.1333854	1	1	3.83E-09	2.3	MALAT1	1
PAK1IP12	1.59E-13	0.2226849	0.072	0.026	3.84E-09	2.3	PAK1IP1	2.769230769
TMEM14A2	1.61E-13	0.3344795	0.122	0.058	3.89E-09	2.3	TMEM14A	2.103448276
FER1L6-AS21	1.68E-13	0.1220067	0.027	0.005	4.05E-09	2.3	FER1L6-AS[illegible]	5.4
RUVBL2	1.79E-13	0.2857027	0.105	0.046	4.31E-09	2.3	RUVBL2	2.282608696
RP11-479G22.8	1.81E-13	0.1328509	0.025	0.005	4.37E-09	2.3	RP11-479G	5
F11-AS1	1.91E-13	0.1314479	0.028	0.006	4.59E-09	2.3	F11-AS1	4.666666667
UQCC22	2.03E-13	0.2931093	0.115	0.053	4.90E-09	2.3	UQCC2	2.169811321
COX171	2.05E-13	0.2834971	0.129	0.062	4.95E-09	2.3	COX17	2.080645161
RP11-114H23.12	2.21E-13	0.344995	0.187	0.104	5.32E-09	2.3	RP11-114H	1.798076923
GLB1L	2.54E-13	0.2006763	0.044	0.012	6.11E-09	2.3	GLB1L	3.666666667
AC104820.2	2.70E-13	0.1251527	0.028	0.006	6.51E-09	2.3	AC104820.[illegible]	4.666666667
ATP6AP22	3.15E-13	0.4090038	0.232	0.142	7.61E-09	2.3	ATP6AP2	1.633802817
SYNCRIP1	3.17E-13	0.4451139	0.183	0.105	7.64E-09	2.3	SYNCRIP	1.742857143
ASF1B1	3.24E-13	0.2129888	0.041	0.011	7.81E-09	2.3	ASF1B	3.727272727
BAG12	3.28E-13	0.3204026	0.178	0.097	7.90E-09	2.3	BAG1	1.835051546
IRS12	3.32E-13	0.3594341	0.173	0.094	8.00E-09	2.3	IRS1	1.840425532
MARCKSL11	3.35E-13	0.3861481	0.244	0.151	8.08E-09	2.3	MARCKSL1	1.61589404
USP9X2	3.37E-13	0.4309768	0.496	0.404	8.11E-09	2.3	USP9X	1.227722772
XYLB1	3.37E-13	0.1671732	0.044	0.012	8.12E-09	2.3	XYLB	3.666666667
STIP12	3.41E-13	0.3135618	0.124	0.059	8.22E-09	2.3	STIP1	2.101694915

GTF3C62	3.52E-13	0.292177	0.124	0.059	8.48E-09	2.3	GTF3C6	2.101694915
MZF1-AS1	3.80E-13	0.2549529	0.083	0.033	9.17E-09	2.3	MZF1-AS1	2.515151515
NAIP	3.92E-13	0.3907659	0.183	0.103	9.46E-09	2.3	NAIP	1.776699029
PVRL31	4.02E-13	0.1691692	0.046	0.013	9.70E-09	2.3	PVRL3	3.538461538
C4orf321	4.02E-13	0.4501566	0.181	0.102	9.70E-09	2.3	C4orf32	1.774509804
RP3-325F22.5	4.29E-13	0.1776473	0.052	0.016	1.03E-08	2.3	RP3-325F2:	3.25
GRK71	4.47E-13	0.159663	0.042	0.011	1.08E-08	2.3	GRK7	3.818181818
PKN1	4.67E-13	0.2158944	0.055	0.018	1.13E-08	2.3	PKN1	3.055555556
GDE11	4.96E-13	0.4049591	0.233	0.143	1.20E-08	2.3	GDE1	1.629370629
MRFAP11	4.98E-13	0.4720728	0.265	0.174	1.20E-08	2.3	MRFAP1	1.522988506
ZBED5-AS13	5.26E-13	0.2181588	0.077	0.03	1.27E-08	2.3	ZBED5-AS1	2.566666667
SIAE2	5.48E-13	0.2439915	0.103	0.045	1.32E-08	2.3	SIAE	2.288888889
KLF7	5.60E-13	0.4396438	0.209	0.125	1.35E-08	2.3	KLF7	1.672
MAOA2	5.63E-13	0.3080556	0.121	0.057	1.36E-08	2.3	MAOA	2.122807018
AK51	5.65E-13	0.2012174	0.073	0.027	1.36E-08	2.3	AK5	2.703703704
DNAJA12	5.75E-13	0.4613355	0.355	0.26	1.39E-08	2.3	DNAJA1	1.365384615
AC007386.41	6.73E-13	0.1409493	0.027	0.005	1.62E-08	2.3	AC007386.4	5.4
P2RX73	6.96E-13	0.2129514	0.052	0.016	1.68E-08	2.3	P2RX7	3.25
C20orf962	8.16E-13	0.249034	0.091	0.039	1.97E-08	2.3	C20orf96	2.333333333
SUMF21	8.42E-13	0.3270078	0.167	0.091	2.03E-08	2.3	SUMF2	1.835164835
TSPAN81	8.50E-13	0.1629621	0.059	0.02	2.05E-08	2.3	TSPAN8	2.95
TMEM104	8.59E-13	0.1334322	0.029	0.006	2.07E-08	2.3	TMEM104	4.833333333
CHST122	8.66E-13	0.2266482	0.074	0.028	2.09E-08	2.3	CHST12	2.642857143
QPRT2	8.71E-13	0.1298441	0.041	0.011	2.10E-08	2.3	QPRT	3.727272727
SPATA9	9.32E-13	0.1069498	0.024	0.004	2.25E-08	2.3	SPATA9	6
LINC014761	9.58E-13	0.1873231	0.069	0.025	2.31E-08	2.3	LINC01476	2.76
RP3-404K8.21	9.69E-13	0.1470099	0.038	0.01	2.34E-08	2.3	RP3-404K8	3.8
RBM35	1.02E-12	0.4134841	0.501	0.405	2.47E-08	2.3	RBM3	1.237037037
PPM1N	1.10E-12	0.1870625	0.039	0.011	2.66E-08	2.3	PPM1N	3.545454545
LMLN1	1.11E-12	0.1309439	0.045	0.013	2.69E-08	2.3	LMLN	3.461538462
RP11-507B12.12	1.13E-12	0.2277936	0.073	0.028	2.72E-08	2.3	RP11-507B	2.607142857
ADIRF2	1.16E-12	0.3944383	0.133	0.066	2.79E-08	2.3	ADIRF	2.015151515
FLNB3	1.20E-12	0.4064892	0.552	0.456	2.89E-08	2.3	FLNB	1.210526316
KLF10	1.20E-12	0.4758021	0.225	0.141	2.90E-08	2.3	KLF10	1.595744681
NOP101	1.29E-12	0.4193119	0.617	0.556	3.10E-08	2.3	NOP10	1.10971223
LSM32	1.29E-12	0.4746111	0.417	0.328	3.12E-08	2.3	LSM3	1.271341463
TMCO31	1.44E-12	0.4443208	0.221	0.136	3.47E-08	2.3	TMCO3	1.625
TMEM1441	1.49E-12	0.1693641	0.052	0.017	3.60E-08	2.3	TMEM144	3.058823529
PDZD82	1.63E-12	0.3568172	0.334	0.229	3.93E-08	2.3	PDZD8	1.458515284
IL12RB21	1.65E-12	0.1591957	0.062	0.022	3.97E-08	2.3	IL12RB2	2.818181818
MXRA81	1.65E-12	0.1057677	0.032	0.008	3.99E-08	2.3	MXRA8	4
HACL1	1.67E-12	0.3223176	0.143	0.075	4.02E-08	2.3	HACL1	1.906666667
TM4SF13	1.69E-12	0.5109679	0.923	0.906	4.07E-08	2.3	TM4SF1	1.018763797
TRAF5	1.74E-12	0.1989889	0.051	0.016	4.19E-08	2.3	TRAF5	3.1875
UGT2B15	1.76E-12	0.1435415	0.031	0.007	4.24E-08	2.3	UGT2B15	4.428571429
CTD-3222D19.21	1.87E-12	0.1379606	0.041	0.011	4.51E-08	2.3	CTD-3222D	3.727272727
RMND5A1	1.87E-12	0.3537201	0.181	0.103	4.51E-08	2.3	RMND5A	1.757281553
QSOX12	1.88E-12	0.3529376	0.24	0.15	4.54E-08	2.3	QSOX1	1.6
ARL5A2	2.03E-12	0.3974352	0.18	0.103	4.89E-08	2.3	ARL5A	1.747572816
POLE21	2.05E-12	0.4907783	0.33	0.236	4.94E-08	2.3	POLE2	1.398305085
DUSP162	2.34E-12	0.3830537	0.529	0.419	5.65E-08	2.3	DUSP16	1.262529833
TRAP13	2.47E-12	0.2130184	0.083	0.034	5.96E-08	2.3	TRAP1	2.441176471

DCXR2	2.53E-12	0.1453893	0.052	0.017	6.11E-08	2.3	DCXR	3.058823529
GGT11	2.57E-12	0.1431352	0.028	0.006	6.20E-08	2.3	GGT1	4.666666667
ITGB3BP4	2.60E-12	0.3111302	0.147	0.078	6.27E-08	2.3	ITGB3BP	1.884615385
TOB21	2.62E-12	0.3794978	0.098	0.044	6.31E-08	2.3	TOB2	2.227272727
BACH12	2.67E-12	0.4923088	0.397	0.306	6.43E-08	2.3	BACH1	1.297385621
SPTSSB1	2.73E-12	0.3423073	0.164	0.089	6.59E-08	2.3	SPTSSB	1.842696629
AASS1	2.83E-12	0.1644408	0.044	0.013	6.83E-08	2.3	AASS	3.384615385
VPS294	2.93E-12	0.4076729	0.414	0.318	7.07E-08	2.3	VPS29	1.301886792
EPCAM1	3.06E-12	0.4558258	0.299	0.211	7.38E-08	2.3	EPCAM	1.417061611
LINC001602	3.12E-12	0.1401727	0.048	0.015	7.54E-08	2.3	LINC00160	3.2
SPCS12	3.15E-12	0.3394657	0.156	0.085	7.58E-08	2.3	SPCS1	1.835294118
Sep-08	3.20E-12	0.132069	0.038	0.01	7.71E-08	2.3	Sep-08	3.8
KCTD19	3.27E-12	0.1033152	0.028	0.006	7.88E-08	2.3	KCTD19	4.666666667
ST7	3.52E-12	0.4383882	0.235	0.148	8.49E-08	2.3	ST7	1.587837838
RP11-140K17.31	3.61E-12	0.2149261	0.096	0.042	8.69E-08	2.3	RP11-140K	2.285714286
AR2	3.62E-12	0.2412184	0.256	0.158	8.72E-08	2.3	AR	1.620253165
EMC31	3.81E-12	0.3973739	0.327	0.235	9.20E-08	2.3	EMC3	1.391489362
YWHAB1	3.83E-12	0.4626878	0.397	0.314	9.23E-08	2.3	YWHAB	1.26433121
SUPT5H	3.84E-12	0.3494176	0.157	0.086	9.27E-08	2.3	SUPT5H	1.825581395
RP11-178C3.2	3.98E-12	0.1508833	0.037	0.01	9.61E-08	2.3	RP11-178C	3.7
TM4SF181	4.08E-12	0.3714396	0.162	0.09	9.84E-08	2.3	TM4SF18	1.8
CFAP1612	4.14E-12	0.2707836	0.088	0.038	9.97E-08	2.3	CFAP161	2.315789474
ZNF2512	4.31E-12	0.2612933	0.086	0.036	1.04E-07	2.3	ZNF251	2.388888889
RP11-384F7.22	4.36E-12	0.2258359	0.126	0.062	1.05E-07	2.3	RP11-384F	2.032258065
KLC4	4.70E-12	0.224223	0.07	0.027	1.13E-07	2.3	KLC4	2.592592593
ATP2A21	5.08E-12	0.4772852	0.354	0.263	1.23E-07	2.3	ATP2A2	1.346007605
GLS3	5.60E-12	0.452473	0.288	0.197	1.35E-07	2.3	GLS	1.461928934
RPF11	6.52E-12	0.3736828	0.14	0.075	1.57E-07	2.3	RPF1	1.866666667
SERTAD1	7.30E-12	0.1795816	0.048	0.015	1.76E-07	2.3	SERTAD1	3.2
PLAT2	7.31E-12	0.3656977	0.159	0.086	1.76E-07	2.3	PLAT	1.848837209
IRX51	7.51E-12	0.2538838	0.079	0.032	1.81E-07	2.3	IRX5	2.46875
GMNN2	7.94E-12	0.2304696	0.053	0.018	1.92E-07	2.3	GMNN	2.944444444
RP11-69E11.41	8.07E-12	0.1275047	0.032	0.008	1.95E-07	2.3	RP11-69E1	4
SCN9A2	8.12E-12	0.1262238	0.038	0.01	1.96E-07	2.3	SCN9A	3.8
ITM2B2	8.14E-12	0.4204872	0.435	0.349	1.96E-07	2.3	ITM2B	1.246418338
DDR11	8.25E-12	0.3896075	0.306	0.214	1.99E-07	2.3	DDR1	1.429906542
NUDT31	8.31E-12	0.3187742	0.143	0.076	2.00E-07	2.3	NUDT3	1.881578947
CCDC102B1	8.47E-12	0.1832536	0.076	0.03	2.04E-07	2.3	CCDC102B	2.533333333
TMOD31	8.84E-12	0.5319789	0.478	0.402	2.13E-07	2.3	TMOD3	1.189054726
SLC25A34	9.15E-12	0.4627957	0.306	0.221	2.21E-07	2.3	SLC25A3	1.384615385
IKBIP	9.43E-12	0.1518181	0.031	0.007	2.27E-07	2.3	IKBIP	4.428571429
MFSD111	9.84E-12	0.3377873	0.166	0.094	2.37E-07	2.3	MFSD11	1.765957447
RP11-421L21.31	1.02E-11	0.2347018	0.052	0.017	2.47E-07	2.3	RP11-421L2	3.058823529
LRRC592	1.04E-11	0.2564906	0.132	0.068	2.51E-07	2.3	LRRC59	1.941176471
RP11-798M19.3	1.05E-11	0.1023367	0.027	0.006	2.54E-07	2.3	RP11-798M	4.5
LRRIQ11	1.14E-11	0.1505543	0.044	0.013	2.74E-07	2.3	LRRIQ1	3.384615385
NOP561	1.15E-11	0.2872877	0.1	0.046	2.77E-07	2.3	NOP56	2.173913043
RP11-108M9.42	1.19E-11	0.2133876	0.07	0.027	2.87E-07	2.3	RP11-108M	2.592592593
RTF11	1.22E-11	0.4184315	0.16	0.09	2.94E-07	2.3	RTF1	1.777777778
NCSTN2	1.25E-11	0.2394602	0.11	0.052	3.01E-07	2.3	NCSTN	2.115384615
DCST1	1.29E-11	0.1372892	0.034	0.009	3.11E-07	2.3	DCST1	3.777777778
RP11-452K12.7	1.33E-11	0.1813417	0.056	0.02	3.22E-07	2.3	RP11-452K	2.8

RNF139-AS11	1.41E-11	0.1205747	0.038	0.011	3.41E-07	2.3	RNF139-AS	3.454545455
NPSR11	1.55E-11	0.126111	0.042	0.012	3.73E-07	2.3	NPSR1	3.5
TAT-AS11	1.56E-11	0.1066612	0.034	0.009	3.77E-07	2.3	TAT-AS1	3.777777778
MT1E1	1.63E-11	0.189681	0.104	0.048	3.93E-07	2.3	MT1E	2.166666667
PCSK61	1.75E-11	0.243587	0.091	0.04	4.22E-07	2.3	PCSK6	2.275
DNAJC191	1.87E-11	0.1870238	0.07	0.028	4.52E-07	2.3	DNAJC19	2.5
FKBP42	1.89E-11	0.3469674	0.135	0.071	4.57E-07	2.3	FKBP4	1.901408451
ZNF341-AS1	1.92E-11	0.1251015	0.032	0.008	4.62E-07	2.3	ZNF341-AS	4
DALRD3	2.00E-11	0.226752	0.056	0.02	4.81E-07	2.3	DALRD3	2.8
BANP1	2.03E-11	0.3257738	0.152	0.083	4.90E-07	2.3	BANP	1.831325301
DEPDC1B2	2.04E-11	0.206054	0.041	0.012	4.92E-07	2.3	DEPDC1B	3.416666667
SOX41	2.06E-11	0.1180354	0.933	0.836	4.97E-07	2.3	SOX4	1.116028708
SRP92	2.12E-11	0.5047177	0.485	0.422	5.12E-07	2.3	SRP9	1.1492891
SLC35E32	2.16E-11	0.3623354	0.142	0.077	5.20E-07	2.3	SLC35E3	1.844155844
ANKRD30B2	2.26E-11	0.1497386	0.055	0.019	5.44E-07	2.3	ANKRD30B	2.894736842
RNF1391	2.26E-11	0.2930049	0.124	0.063	5.45E-07	2.3	RNF139	1.968253968
CHMP51	2.35E-11	0.3606079	0.524	0.435	5.66E-07	2.3	CHMP5	1.204597701
TMEM1562	2.64E-11	0.7512478	0.104	0.049	6.36E-07	2.3	TMEM156	2.12244898
MOCS23	2.65E-11	0.3920983	0.126	0.067	6.39E-07	2.3	MOCS2	1.880597015
CTA-204B4.2	2.67E-11	0.1021589	0.022	0.004	6.45E-07	2.3	CTA-204B4	5.5
TMEM982	2.76E-11	0.1127712	0.034	0.009	6.64E-07	2.3	TMEM98	3.777777778
SEMA3E2	2.97E-11	0.3651014	0.223	0.14	7.16E-07	2.3	SEMA3E	1.592857143
WNT41	2.97E-11	0.1562122	0.041	0.012	7.16E-07	2.3	WNT4	3.416666667
RP11-96O20.21	2.99E-11	0.1197526	0.038	0.011	7.22E-07	2.3	RP11-96O2	3.454545455
ENPP52	3.06E-11	0.2159109	0.079	0.033	7.37E-07	2.3	ENPP5	2.393939394
AOAH	3.06E-11	0.1014607	0.025	0.005	7.38E-07	2.3	AOAH	5
IER52	3.10E-11	0.2983203	0.188	0.111	7.47E-07	2.3	IER5	1.693693694
OAT3	3.23E-11	0.4442996	0.364	0.275	7.80E-07	2.3	OAT	1.323636364
ULK4	3.26E-11	0.3832214	0.188	0.113	7.86E-07	2.3	ULK4	1.663716814
INPP4B1	3.55E-11	0.337685	0.447	0.327	8.55E-07	2.3	INPP4B	1.366972477
RP3-331H24.51	3.57E-11	0.1931228	0.076	0.031	8.62E-07	2.3	RP3-331H2	2.451612903
ENPP31	3.75E-11	0.1142328	0.039	0.011	9.04E-07	2.3	ENPP3	3.545454545
CNDP22	3.81E-11	0.3237297	0.201	0.123	9.18E-07	2.3	CNDP2	1.634146341
DNER2	3.84E-11	0.2313002	0.096	0.044	9.27E-07	2.3	DNER	2.181818182
MORF4L24	4.16E-11	0.3489177	0.756	0.742	1.00E-06	2.3	MORF4L2	1.018867925
SORD2	4.33E-11	0.2028092	0.077	0.032	1.04E-06	2.3	SORD	2.40625
RP1-221C16.81	4.42E-11	0.3731759	0.117	0.059	1.07E-06	2.3	RP1-221C1	1.983050847
PTH	4.54E-11	0.1333312	0.046	0.015	1.10E-06	2.3	PTH	3.066666667
DYNC1I21	4.65E-11	0.5368032	0.441	0.382	1.12E-06	2.3	DYNC1I2	1.154450262
RP11-96O20.42	4.65E-11	0.1186212	0.037	0.01	1.12E-06	2.3	RP11-96O2	3.7
GPBP13	4.68E-11	0.3630597	0.721	0.688	1.13E-06	2.3	GPBP1	1.047965116
BRAT1	4.91E-11	0.1127758	0.025	0.006	1.18E-06	2.3	BRAT1	4.166666667
ADGRA2	5.00E-11	0.1920874	0.017	0.003	1.21E-06	2.3	ADGRA2	5.666666667
FMOD2	5.09E-11	0.1371198	0.031	0.008	1.23E-06	2.3	FMOD	3.875
RBM20	5.21E-11	0.1252693	0.031	0.008	1.26E-06	2.3	RBM20	3.875
RGL22	5.23E-11	0.2203669	0.093	0.042	1.26E-06	2.3	RGL2	2.214285714
TSC22D1	5.66E-11	0.4181791	0.452	0.365	1.37E-06	2.3	TSC22D1	1.238356164
PNPLA41	6.01E-11	0.2162457	0.065	0.025	1.45E-06	2.3	PNPLA4	2.6
SBF2-AS11	6.21E-11	0.2458106	0.081	0.035	1.50E-06	2.3	SBF2-AS1	2.314285714
DDIT32	6.21E-11	0.3688975	0.177	0.105	1.50E-06	2.3	DDIT3	1.685714286
ACTN1-AS12	6.22E-11	0.1286002	0.031	0.008	1.50E-06	2.3	ACTN1-AS1	3.875
RP21	6.29E-11	0.2581861	0.076	0.032	1.52E-06	2.3	RP2	2.375

RP11-277A4.41	6.61E-11	0.1146755	0.046	0.015	1.59E-06	2.3	RP11-277A	3.066666667
LY75-CD302	7.65E-11	0.1037738	0.028	0.007	1.85E-06	2.3	LY75-CD30:	4
SREK1IP11	7.83E-11	0.4134738	0.243	0.166	1.89E-06	2.3	SREK1IP1	1.463855422
PPP1R72	7.85E-11	0.292641	0.1	0.048	1.89E-06	2.3	PPP1R7	2.083333333
STON21	8.06E-11	0.3101004	0.1	0.048	1.94E-06	2.3	STON2	2.083333333
ERBB22	8.23E-11	0.4088935	0.19	0.116	1.98E-06	2.3	ERBB2	1.637931034
KANSL1L1	8.28E-11	0.423363	0.298	0.213	2.00E-06	2.3	KANSL1L	1.399061033
SLC20A11	8.50E-11	0.3455434	0.124	0.064	2.05E-06	2.3	SLC20A1	1.9375
PRSS23	8.71E-11	0.3184035	0.32	0.22	2.10E-06	2.3	PRSS23	1.454545455
C11orf84	8.76E-11	0.146887	0.041	0.012	2.11E-06	2.3	C11orf84	3.416666667
PNPO1	9.36E-11	0.1005466	0.025	0.006	2.26E-06	2.3	PNPO	4.166666667
CXCL82	9.81E-11	0.534776	0.548	0.444	2.37E-06	2.3	CXCL8	1.234234234
EHD11	9.95E-11	0.189447	0.062	0.024	2.40E-06	2.3	EHD1	2.583333333
RP11-665G4.11	1.01E-10	0.3623487	0.156	0.088	2.45E-06	2.3	RP11-665G	1.772727273
FGF131	1.02E-10	0.345263	0.565	0.451	2.45E-06	2.3	FGF13	1.252771619
RP11-371F15.31	1.02E-10	0.3128933	0.153	0.085	2.46E-06	2.3	RP11-371F:	1.8
DNAJC91	1.03E-10	0.3275334	0.128	0.068	2.49E-06	2.3	DNAJC9	1.882352941
TCF7L22	1.06E-10	0.4697285	0.461	0.381	2.54E-06	2.3	TCF7L2	1.209973753
RASSF61	1.09E-10	0.3557891	0.129	0.069	2.63E-06	2.3	RASSF6	1.869565217
H3F3B2	1.15E-10	0.4734196	0.463	0.392	2.76E-06	2.3	H3F3B	1.181122449
SCD53	1.19E-10	0.242743	0.086	0.038	2.87E-06	2.3	SCD5	2.263157895
MT-ND61	1.20E-10	0.4859299	0.257	0.177	2.90E-06	2.3	MT-ND6	1.451977401
TBC1D10A2	1.24E-10	0.7883084	0.233	0.154	3.00E-06	2.3	TBC1D10A	1.512987013
DPCD1	1.25E-10	0.319771	0.147	0.082	3.01E-06	2.3	DPCD	1.792682927
TIMP3	1.29E-10	0.1403653	0.045	0.015	3.10E-06	2.3	TIMP3	3
RP5-823G15.51	1.32E-10	0.1065702	0.027	0.006	3.18E-06	2.3	RP5-823G1	4.5
RP11-696N14.1	1.36E-10	0.1516719	0.034	0.009	3.27E-06	2.3	RP11-696N	3.777777778
FUT82	1.37E-10	0.4676738	0.358	0.259	3.30E-06	2.3	FUT8	1.382239382
CETN21	1.37E-10	0.3834708	0.264	0.181	3.31E-06	2.3	CETN2	1.458563536
ADPRHL11	1.39E-10	0.1056093	0.031	0.008	3.34E-06	2.3	ADPRHL1	3.875
PLCG1	1.40E-10	0.1183649	0.035	0.01	3.38E-06	2.3	PLCG1	3.5
LGALS3BP	1.47E-10	0.2159079	0.069	0.028	3.54E-06	2.3	LGALS3BP	2.464285714
COL4A61	1.53E-10	0.2593934	0.118	0.06	3.68E-06	2.3	COL4A6	1.966666667
CCDC731	1.58E-10	0.4248282	0.178	0.106	3.81E-06	2.3	CCDC73	1.679245283
MYB2	1.66E-10	0.1818678	0.058	0.022	3.99E-06	2.3	MYB	2.636363636
ATG1011	1.70E-10	0.224616	0.073	0.031	4.09E-06	2.3	ATG101	2.35483871
FMO42	1.75E-10	0.234904	0.11	0.055	4.22E-06	2.3	FMO4	2
FSIP12	1.80E-10	0.2486449	0.105	0.051	4.33E-06	2.3	FSIP1	2.058823529
MAN2B21	1.80E-10	0.1442035	0.042	0.013	4.35E-06	2.3	MAN2B2	3.230769231
FBXL31	1.88E-10	0.3084064	0.174	0.104	4.54E-06	2.3	FBXL3	1.673076923
RNF1281	1.93E-10	0.3594874	0.201	0.126	4.64E-06	2.3	RNF128	1.595238095
RP11-659O3.12	2.11E-10	0.3231073	0.244	0.164	5.09E-06	2.3	RP11-659O	1.487804878
HTT-AS	2.11E-10	0.1003011	0.011	0.001	5.09E-06	2.3	HTT-AS	11
ALOX15B1	2.16E-10	0.291062	0.11	0.054	5.21E-06	2.3	ALOX15B	2.037037037
ANO4	2.17E-10	0.1160971	0.027	0.006	5.24E-06	2.3	ANO4	4.5
C12orf73	2.17E-10	0.1874429	0.051	0.018	5.24E-06	2.3	C12orf73	2.833333333
CTD-2666L21.1	2.20E-10	0.2163877	0.048	0.016	5.30E-06	2.3	CTD-2666L:	3
OVOL11	2.46E-10	0.1055361	0.032	0.009	5.93E-06	2.3	OVOL1	3.555555556
ASXL11	2.56E-10	0.4802029	0.333	0.255	6.18E-06	2.3	ASXL1	1.305882353
ERI2	2.56E-10	0.2329086	0.072	0.03	6.18E-06	2.3	ERI2	2.4
ATP5H3	2.66E-10	0.4797666	0.416	0.349	6.40E-06	2.3	ATP5H	1.191977077
CRNDE2	2.68E-10	0.3554859	0.17	0.102	6.45E-06	2.3	CRNDE	1.666666667

SLC25A44	2.69E-10	0.1496504	0.045	0.015	6.48E-06	2.3	SLC25A44	3
ECI21	2.72E-10	0.3365739	0.136	0.076	6.55E-06	2.3	ECI2	1.789473684
CXCR42	2.83E-10	0.4356528	0.302	0.216	6.82E-06	2.3	CXCR4	1.398148148
SLC13A12	2.85E-10	0.1231765	0.044	0.014	6.86E-06	2.3	SLC13A1	3.142857143
YY11	2.88E-10	0.392099	0.302	0.22	6.94E-06	2.3	YY1	1.372727273
LYPD3	2.99E-10	0.2302223	0.121	0.063	7.20E-06	2.3	LYPD3	1.920634921
TAF9B2	3.19E-10	0.1480657	0.045	0.015	7.69E-06	2.3	TAF9B	3
GAPLINC1	3.32E-10	0.1245973	0.035	0.01	8.01E-06	2.3	GAPLINC	3.5
CAPN132	3.36E-10	0.189953	0.083	0.037	8.11E-06	2.3	CAPN13	2.243243243
POLR3GL1	3.93E-10	0.1218815	0.037	0.011	9.46E-06	2.3	POLR3GL	3.363636364
SECISBP21	4.02E-10	0.3768541	0.163	0.098	9.69E-06	2.3	SECISBP2	1.663265306
HSP90AB15	4.04E-10	0.2446269	0.909	0.837	9.74E-06	2.3	HSP90AB1	1.086021505
TXLNB	4.19E-10	0.1009851	0.024	0.005	1.01E-05	2.3	TXLNB	4.8
KIZ-AS15	4.21E-10	0.2076876	0.868	0.843	1.02E-05	2.3	KIZ-AS1	1.029655991
AGA2	4.45E-10	0.1717927	0.074	0.032	1.07E-05	2.3	AGA	2.3125
RP11-21L19.1	4.98E-10	0.1638234	0.059	0.023	1.20E-05	2.3	RP11-21L19	2.565217391
FAM46B3	5.66E-10	0.2916921	0.105	0.053	1.37E-05	2.3	FAM46B	1.981132075
TTC61	6.06E-10	0.3445824	0.258	0.173	1.46E-05	2.3	TTC6	1.49132948
COPZ13	6.15E-10	0.448919	0.39	0.32	1.48E-05	2.3	COPZ1	1.21875
HIVEP22	6.18E-10	0.3065264	0.538	0.448	1.49E-05	2.3	HIVEP2	1.200892857
KLHL25	6.23E-10	0.108106	0.034	0.01	1.50E-05	2.3	KLHL25	3.4
TMEM1161	6.29E-10	0.3141712	0.133	0.074	1.52E-05	2.3	TMEM116	1.797297297
BRI3BP1	6.34E-10	0.1673653	0.039	0.012	1.53E-05	2.3	BRI3BP	3.25
ARHGEF372	6.35E-10	0.2464678	0.103	0.051	1.53E-05	2.3	ARHGEF37	2.019607843
PAFAH21	6.38E-10	0.1742078	0.055	0.021	1.54E-05	2.3	PAFAH2	2.619047619
MB21D1	6.69E-10	0.1507608	0.037	0.011	1.61E-05	2.3	MB21D1	3.363636364
FN12	6.95E-10	0.1834468	0.067	0.028	1.67E-05	2.3	FN1	2.392857143
TGFBR11	6.95E-10	0.3973977	0.183	0.114	1.68E-05	2.3	TGFBR1	1.605263158
STX32	7.07E-10	0.3596277	0.212	0.138	1.70E-05	2.3	STX3	1.536231884
XXYLT11	7.08E-10	0.133993	0.037	0.011	1.71E-05	2.3	XXYLT1	3.363636364
GATM1	7.53E-10	0.1670284	0.052	0.019	1.81E-05	2.3	GATM	2.736842105
PEMT2	7.98E-10	0.5779148	0.083	0.038	1.92E-05	2.3	PEMT	2.184210526
CTD-2201I18.1	8.02E-10	0.100223	0.029	0.008	1.93E-05	2.3	CTD-2201I1	3.625
PPP1R1B3	8.03E-10	0.1916675	0.074	0.033	1.94E-05	2.3	PPP1R1B	2.242424242
KLF63	8.07E-10	0.5178711	0.619	0.57	1.95E-05	2.3	KLF6	1.085964912
EPB41L51	8.27E-10	0.3946971	0.379	0.295	1.99E-05	2.3	EPB41L5	1.284745763
VDAC11	8.28E-10	0.4110249	0.287	0.211	2.00E-05	2.3	VDAC1	1.360189573
LINC01094	8.28E-10	0.1014663	0.022	0.005	2.00E-05	2.3	LINC01094	4.4
CYP51A1-AS1	8.37E-10	0.1118401	0.035	0.01	2.02E-05	2.3	CYP51A1-A	3.5
VAMP8	9.05E-10	0.2162582	0.806	0.759	2.18E-05	2.3	VAMP8	1.061923584
ASPH1	9.17E-10	0.3215474	0.379	0.283	2.21E-05	2.3	ASPH	1.339222615
DNASE1	9.89E-10	0.245036	0.103	0.052	2.39E-05	2.3	DNASE1	1.980769231
RP11-317J19.1	9.97E-10	0.2270949	0.066	0.028	2.40E-05	2.3	RP11-317J1	2.357142857
HNRNPA01	1.03E-09	0.3286991	0.162	0.096	2.48E-05	2.3	HNRNPA0	1.6875
AZIN12	1.06E-09	0.3935822	0.284	0.203	2.56E-05	2.3	AZIN1	1.399014778
GPN3	1.07E-09	0.1656623	0.059	0.023	2.59E-05	2.3	GPN3	2.565217391
KTN12	1.08E-09	0.3441645	0.597	0.542	2.61E-05	2.3	KTN1	1.101476015
CLIC61	1.14E-09	0.4828541	0.296	0.217	2.75E-05	2.3	CLIC6	1.3640553
PAPLN	1.15E-09	0.1137095	0.039	0.013	2.77E-05	2.3	PAPLN	3
GLRX52	1.21E-09	0.2223617	0.076	0.034	2.91E-05	2.3	GLRX5	2.235294118
PROM22	1.22E-09	0.2190318	0.098	0.049	2.94E-05	2.3	PROM2	2
ARPP191	1.27E-09	0.3699258	0.503	0.429	3.06E-05	2.3	ARPP19	1.172494172

MATN32	1.31E-09	0.1696525	0.07	0.03	3.17E-05	2.3	MATN3	2.333333333
SETD91	1.35E-09	0.2139041	0.056	0.022	3.26E-05	2.3	SETD9	2.545454545
WNK11	1.40E-09	0.3874173	0.317	0.241	3.38E-05	2.3	WNK1	1.315352697
MRPS211	1.42E-09	0.3828364	0.562	0.517	3.42E-05	2.3	MRPS21	1.087040619
GDPD42	1.43E-09	0.1266732	0.035	0.011	3.46E-05	2.3	GDPD4	3.181818182
GCLC	1.49E-09	0.3366439	0.126	0.07	3.60E-05	2.3	GCLC	1.8
PRPF40A1	1.56E-09	0.3621731	0.25	0.173	3.77E-05	2.3	PRPF40A	1.445086705
SYK1	1.56E-09	0.1561608	0.044	0.015	3.77E-05	2.3	SYK	2.933333333
TMEM9B1	1.61E-09	0.2413322	0.09	0.043	3.87E-05	2.3	TMEM9B	2.093023256
HMGN23	1.61E-09	0.295758	0.107	0.056	3.88E-05	2.3	HMGN2	1.910714286
ENY23	1.72E-09	0.4283167	0.454	0.389	4.15E-05	2.3	ENY2	1.167095116
DNAL11	1.73E-09	0.3491029	0.088	0.042	4.18E-05	2.3	DNAL1	2.095238095
ALDH3B22	1.73E-09	0.1053987	0.035	0.011	4.18E-05	2.3	ALDH3B2	3.181818182
LMO72	1.74E-09	0.5013815	0.27	0.184	4.20E-05	2.3	LMO7	1.467391304
CHD3	1.74E-09	0.2743544	0.107	0.055	4.20E-05	2.3	CHD3	1.945454545
RP11-13A1.31	1.81E-09	0.1042476	0.031	0.009	4.37E-05	2.3	RP11-13A1	3.444444444
CYB5A1	1.86E-09	0.3451411	0.347	0.257	4.49E-05	2.3	CYB5A	1.350194553
MMP10	1.98E-09	0.1607413	0.025	0.006	4.78E-05	2.3	MMP10	4.166666667
SNX51	2.00E-09	0.3315686	0.119	0.066	4.82E-05	2.3	SNX5	1.803030303
C16orf722	2.05E-09	0.376969	0.212	0.14	4.93E-05	2.3	C16orf72	1.514285714
SLC22A141	2.07E-09	0.1133635	0.032	0.009	4.99E-05	2.3	SLC22A14	3.555555556
DAB11	2.38E-09	0.3175627	0.108	0.056	5.74E-05	2.3	DAB1	1.928571429
LAMTOR53	2.44E-09	0.3129948	0.624	0.586	5.89E-05	2.3	LAMTOR5	1.064846416
SEP152	2.54E-09	0.3998194	0.478	0.412	6.13E-05	2.3	Sep-15	1.160194175
DPY302	2.57E-09	0.3736453	0.251	0.179	6.19E-05	2.3	DPY30	1.402234637
SLC2A5	2.59E-09	0.1085419	0.024	0.006	6.23E-05	2.3	SLC2A5	4
LMNA	2.60E-09	0.4109012	0.312	0.231	6.27E-05	2.3	LMNA	1.350649351
LYRM52	2.64E-09	0.3329693	0.173	0.108	6.36E-05	2.3	LYRM5	1.601851852
ERVK3-11	2.70E-09	0.1891443	0.077	0.035	6.50E-05	2.3	ERVK3-1	2.2
ZFAND2A2	2.72E-09	0.2926948	0.081	0.038	6.55E-05	2.3	ZFAND2A	2.131578947
FAM234B2	2.77E-09	0.2387983	0.094	0.047	6.68E-05	2.3	FAM234B	2
PHEX1	2.86E-09	0.1555386	0.055	0.021	6.90E-05	2.3	PHEX	2.619047619
RP4-724E16.21	2.88E-09	0.1438497	0.053	0.021	6.96E-05	2.3	RP4-724E1(	2.523809524
CYP2A62	2.91E-09	0.1034335	0.025	0.006	7.01E-05	2.3	CYP2A6	4.166666667
MSL11	2.93E-09	0.3090435	0.225	0.151	7.06E-05	2.3	MSL1	1.490066225
AL591893.11	2.97E-09	0.5583054	0.076	0.035	7.17E-05	2.3	AL591893.:	2.171428571
GPR158	3.17E-09	0.2681384	0.093	0.046	7.64E-05	2.3	GPR158	2.02173913
DAPK32	3.32E-09	0.2293682	0.104	0.054	8.01E-05	2.3	DAPK3	1.925925926
MRPL403	3.43E-09	0.3345884	0.154	0.093	8.27E-05	2.3	MRPL40	1.655913978
FEM1B2	3.46E-09	0.3364578	0.246	0.168	8.34E-05	2.3	FEM1B	1.464285714
TOP12	3.52E-09	0.3021348	0.528	0.445	8.50E-05	2.3	TOP1	1.186516854
LINC01060	3.64E-09	0.1671125	0.038	0.012	8.79E-05	2.3	LINC01060	3.166666667
BHLHE402	3.70E-09	0.5516981	0.26	0.187	8.91E-05	2.3	BHLHE40	1.390374332
VAV1	3.97E-09	0.1101277	0.024	0.006	9.57E-05	2.3	VAV1	4
HNRNPA2B13	4.10E-09	0.3849857	0.478	0.412	9.88E-05	2.3	HNRNPA2E	1.160194175
SLC35F41	4.21E-09	0.12199	0.032	0.01	0.000101448	2.3	SLC35F4	3.2
GNG51	4.21E-09	0.2779529	0.187	0.118	0.00010147	2.3	GNG5	1.584745763
TSPYL1	4.27E-09	0.2774208	0.104	0.055	0.00010296	2.3	TSPYL1	1.890909091
MEX3C1	4.35E-09	0.314129	0.121	0.066	0.000104893	2.3	MEX3C	1.833333333
SRRM3	4.37E-09	0.1386279	0.025	0.006	0.000105284	2.3	SRRM3	4.166666667
RNF81	4.43E-09	0.1476356	0.051	0.019	0.000106704	2.3	RNF8	2.684210526
CADPS22	4.51E-09	0.5455629	0.466	0.403	0.000108757	2.3	CADPS2	1.156327543

AMIGO22	4.57E-09	0.1885769	0.058	0.023	0.000110279	2.3	AMIGO2	2.52173913
RP11-37N22.11	4.73E-09	0.1961049	0.074	0.034	0.000113957	2.3	RP11-37N2	2.176470588
CFL11	4.75E-09	0.3751131	0.399	0.316	0.000114511	2.3	CFL1	1.262658228
RP11-439A17.41	4.83E-09	0.276681	0.121	0.066	0.000116363	2.3	RP11-439A	1.833333333
DUSP102	4.92E-09	0.3331502	0.308	0.223	0.000118522	2.3	DUSP10	1.381165919
RP11-189B4.71	5.12E-09	0.244359	0.107	0.056	0.000123516	2.3	RP11-189B	1.910714286
PFN11	5.32E-09	0.305999	0.26	0.185	0.000128287	2.3	PFN1	1.405405405
ZNF724P1	5.35E-09	0.120114	0.035	0.011	0.000128944	2.3	ZNF724P	3.181818182
MIR99AHG2	5.37E-09	0.2641068	0.119	0.065	0.000129384	2.3	MIR99AHG	1.830769231
MAPKAPK22	5.43E-09	0.4100312	0.215	0.145	0.000130835	2.3	MAPKAPK2	1.482758621
CTBS	5.59E-09	0.1803131	0.072	0.032	0.00013475	2.3	CTBS	2.25
TNC2	5.76E-09	0.191848	0.438	0.33	0.00013901	2.3	TNC	1.327272727
FAM84A	5.81E-09	0.202786	0.052	0.02	0.000140193	2.3	FAM84A	2.6
RP11-84A19.43	5.87E-09	0.2086404	0.104	0.054	0.000141552	2.3	RP11-84A1	1.925925926
FUS1	6.11E-09	0.3824091	0.358	0.283	0.000147386	2.3	FUS	1.265017668
PLIN3	6.41E-09	0.3636404	0.229	0.157	0.000154541	2.3	PLIN3	1.458598726
SOD11	6.41E-09	0.3160006	0.188	0.121	0.000154623	2.3	SOD1	1.553719008
ATP8A2	6.50E-09	0.1339796	0.032	0.01	0.00015664	2.3	ATP8A2	3.2
THSD4-AS13	6.57E-09	0.4143611	0.521	0.457	0.000158486	2.3	THSD4-AS1	1.140043764
STK35	6.98E-09	0.2012959	0.077	0.036	0.000168232	2.3	STK35	2.138888889
ABCC112	6.99E-09	0.1397857	0.041	0.014	0.000168591	2.3	ABCC11	2.928571429
PRKAR1A2	7.25E-09	0.3063155	0.322	0.238	0.000174902	2.3	PRKAR1A	1.352941176
C14orf371	7.38E-09	0.3217303	0.167	0.104	0.000177988	2.3	C14orf37	1.605769231
RAB11FIP4	7.44E-09	0.2311648	0.087	0.043	0.000179458	2.3	RAB11FIP4	2.023255814
AQP3	7.62E-09	0.4693544	0.104	0.055	0.000183723	2.3	AQP3	1.890909091
DGUOK-AS11	7.95E-09	0.1010965	0.031	0.009	0.000191708	2.3	DGUOK-AS	3.444444444
AC010149.41	7.99E-09	0.280649	0.114	0.062	0.00019267	2.3	AC010149.4	1.838709677
NOSTRIN1	8.00E-09	0.1938058	0.074	0.034	0.00019289	2.3	NOSTRIN	2.176470588
RIC8B	8.00E-09	0.2349432	0.083	0.04	0.000193024	2.3	RIC8B	2.075
DDX23	8.20E-09	0.2882155	0.11	0.059	0.000197664	2.3	DDX23	1.86440678
C5orf451	8.42E-09	0.3323195	0.076	0.036	0.000203003	2.3	C5orf45	2.111111111
MLH1	8.61E-09	0.2041744	0.087	0.043	0.000207668	2.3	MLH1	2.023255814
SSUH21	8.85E-09	0.1528763	0.042	0.015	0.000213388	2.3	SSUH2	2.8
RAB8A	9.27E-09	0.1811143	0.06	0.026	0.000223511	2.3	RAB8A	2.307692308
SKIL1	1.01E-08	0.339158	0.166	0.104	0.000243473	2.3	SKIL	1.596153846
GFPT22	1.04E-08	0.1386091	0.038	0.013	0.000250703	2.3	GFPT2	2.923076923
FSIP21	1.04E-08	0.1204636	0.034	0.011	0.00025077	2.3	FSIP2	3.090909091
GNPNAT11	1.12E-08	0.1956625	0.063	0.027	0.00026978	2.3	GNPNAT1	2.333333333
GJC32	1.14E-08	0.3084209	0.153	0.093	0.000274963	2.3	GJC3	1.64516129
ITPKA	1.33E-08	0.1011849	0.02	0.004	0.000319634	2.3	ITPKA	5
NR4A21	1.49E-08	0.3576706	0.173	0.109	0.000358803	2.3	NR4A2	1.587155963
MAGED21	1.55E-08	0.2016846	0.063	0.027	0.000374131	2.3	MAGED2	2.333333333
LONRF3	1.57E-08	0.2057503	0.058	0.024	0.000377911	2.3	LONRF3	2.416666667
KIF91	1.60E-08	0.640139	0.131	0.075	0.000386028	2.3	KIF9	1.746666667
FHAD1	1.66E-08	0.2130495	0.055	0.022	0.000399518	2.3	FHAD1	2.5
RPL376	1.66E-08	0.1693269	0.951	0.945	0.000400555	2.3	RPL37	1.006349206
PPCS1	1.70E-08	0.2142776	0.087	0.043	0.000410768	2.3	PPCS	2.023255814
ARAP21	1.76E-08	0.4177204	0.239	0.167	0.000423377	2.3	ARAP2	1.431137725
SARAF2	1.79E-08	0.3376864	0.426	0.352	0.000430695	2.3	SARAF	1.210227273
SDHC3	1.79E-08	0.3520249	0.229	0.162	0.000432751	2.3	SDHC	1.413580247
UBE2N3	1.80E-08	0.3861514	0.272	0.203	0.000434787	2.3	UBE2N	1.339901478
NFIL32	1.83E-08	0.3270154	0.26	0.187	0.000442421	2.3	NFIL3	1.390374332

ZCWPW11	1.86E-08	0.1155258	0.028	0.008	0.000449151	2.3	ZCWPW1	3.5
ADGRV11	1.93E-08	0.4129951	0.233	0.164	0.000464853	2.3	ADGRV1	1.420731707
RAB3D1	1.96E-08	0.203761	0.07	0.032	0.000473769	2.3	RAB3D	2.1875
MALRD12	2.00E-08	0.2711673	0.088	0.044	0.000483137	2.3	MALRD1	2
MARCH2	2.01E-08	0.1954577	0.086	0.043	0.000483897	2.3	MARCH2	2
TFF32	2.09E-08	0.3969653	0.108	0.058	0.000503292	2.3	TFF3	1.862068966
RPGRIP1L1	2.10E-08	0.1155534	0.031	0.01	0.000506364	2.3	RPGRIP1L	3.1
KHDRBS31	2.20E-08	0.3479829	0.192	0.126	0.000529665	2.3	KHDRBS3	1.523809524
KIAA0586	2.22E-08	0.3215452	0.143	0.087	0.000534219	2.3	KIAA0586	1.643678161
C1GALT1C11	2.27E-08	0.2490793	0.096	0.05	0.000546946	2.3	C1GALT1C1	1.92
AC007365.1	2.27E-08	0.1112687	0.021	0.005	0.000547722	2.3	AC007365.	4.2
RP11-420A23.11	2.32E-08	0.2649353	0.088	0.045	0.00056044	2.3	RP11-420A	1.955555556
POLR2G2	2.50E-08	0.3614404	0.24	0.173	0.000602149	2.3	POLR2G	1.387283237
ZC3H12A1	2.52E-08	0.3142133	0.119	0.067	0.000608084	2.3	ZC3H12A	1.776119403
PTPRN2	2.60E-08	0.1862315	0.037	0.012	0.000627207	2.3	PTPRN2	3.083333333
ZNF844	2.62E-08	0.2348469	0.098	0.052	0.000632702	2.3	ZNF844	1.884615385
C2orf743	2.63E-08	0.2183983	0.073	0.034	0.00063345	2.3	C2orf74	2.147058824
ANKRD281	2.68E-08	0.4596929	0.392	0.327	0.000645842	2.3	ANKRD28	1.198776758
DLC12	2.83E-08	0.2945626	0.115	0.064	0.000683554	2.3	DLC1	1.796875
RP11-689B22.21	2.99E-08	0.2443879	0.02	0.005	0.000721947	2.3	RP11-689B	4
FGD5-AS11	3.03E-08	0.2724612	0.103	0.055	0.00073107	2.3	FGD5-AS1	1.872727273
IKZF3	3.14E-08	0.1027923	0.027	0.008	0.000756523	2.3	IKZF3	3.375
PLBD1	3.24E-08	0.1104065	0.032	0.01	0.000782255	2.3	PLBD1	3.2
INTS123	3.52E-08	0.3964921	0.281	0.209	0.000849128	2.3	INTS12	1.344497608
ARL32	3.69E-08	0.3456897	0.337	0.257	0.000890788	2.3	ARL3	1.311284047
SNRPE5	3.76E-08	0.4199144	0.563	0.521	0.000907171	2.3	SNRPE	1.080614203
RP11-596C23.6	3.89E-08	0.4476442	0.046	0.018	0.000938675	2.3	RP11-596C	2.555555556
WBSCR22	4.07E-08	0.1628359	0.072	0.034	0.000981491	2.3	WBSCR22	2.117647059
PDIA33	4.08E-08	0.2824419	0.381	0.306	0.000984259	2.3	PDIA3	1.245098039
RP11-1035H13.3	4.13E-08	0.1016646	0.025	0.007	0.000994737	2.3	RP11-1035	3.571428571
CARS2	4.16E-08	0.3020122	0.104	0.057	0.001002727	2.3	CARS2	1.824561404
LURAP1L-AS12	4.18E-08	0.381806	0.145	0.09	0.001008163	2.3	LURAP1L-A	1.611111111
FAM177A11	4.30E-08	0.2710154	0.188	0.124	0.001037053	2.3	FAM177A1	1.516129032
GUSB2	4.52E-08	0.180973	0.088	0.045	0.001090447	2.3	GUSB	1.955555556
DNAH11	4.53E-08	0.2047867	0.094	0.049	0.001092179	2.3	DNAH11	1.918367347
GFM11	4.66E-08	0.2437889	0.098	0.053	0.001124486	2.3	GFM1	1.849056604
PTPN62	4.88E-08	0.233915	0.086	0.044	0.001175572	2.3	PTPN6	1.954545455
CADM2	4.88E-08	0.2028779	0.062	0.027	0.001176282	2.3	CADM2	2.296296296
SELT1	4.90E-08	0.2702572	0.129	0.076	0.001182126	2.3	SELT	1.697368421
C3orf522	5.01E-08	0.2179186	0.236	0.16	0.001206972	2.3	C3orf52	1.475
MAPK61	5.11E-08	0.3534517	0.344	0.272	0.001232837	2.3	MAPK6	1.264705882
MAP1LC3B	5.15E-08	0.3312281	0.221	0.153	0.001241089	2.3	MAP1LC3B	1.444444444
RP11-779O18.32	5.53E-08	0.3558866	0.316	0.243	0.001332741	2.3	RP11-779O	1.300411523
ICE21	5.54E-08	0.2686971	0.094	0.05	0.001335968	2.3	ICE2	1.88
ITPR12	5.74E-08	0.3044873	0.16	0.101	0.001382894	2.3	ITPR1	1.584158416
PSAP1	5.89E-08	0.3680449	0.184	0.121	0.001421288	2.3	PSAP	1.520661157
TIMM17A3	6.12E-08	0.3565167	0.287	0.219	0.00147546	2.3	TIMM17A	1.310502283
INO80D1	6.16E-08	0.2205391	0.654	0.566	0.001485057	2.3	INO80D	1.155477032
MCL11	6.22E-08	0.405461	0.388	0.325	0.001499332	2.3	MCL1	1.193846154
CTB-161M19.4	6.36E-08	0.1482194	0.038	0.014	0.001534157	2.3	CTB-161M1	2.714285714
GKAP1	6.43E-08	0.2161417	0.058	0.025	0.001549723	2.3	GKAP1	2.32
CTD-2410N18.5	6.46E-08	0.1659196	0.062	0.028	0.001558762	2.3	CTD-2410N	2.214285714

HADHB2	6.47E-08	0.3750518	0.338	0.273	0.001559306	2.3	HADHB	1.238095238
SPOCK1	6.58E-08	0.1667122	0.044	0.017	0.001586259	2.3	SPOCK1	2.588235294
SLC35A13	6.89E-08	0.177857	0.066	0.031	0.001660214	2.3	SLC35A1	2.129032258
GSG1L1	7.11E-08	0.3144978	0.044	0.017	0.001715233	2.3	GSG1L	2.588235294
YME1L11	7.12E-08	0.3644689	0.337	0.269	0.001717934	2.3	YME1L1	1.252788104
CABYR	7.18E-08	0.2068732	0.044	0.017	0.001731369	2.3	CABYR	2.588235294
FAM216A	7.42E-08	0.1009366	0.035	0.012	0.001789703	2.3	FAM216A	2.916666667
MRPS301	7.51E-08	0.1670137	0.052	0.022	0.001812004	2.3	MRPS30	2.363636364
ERMP1	7.56E-08	0.2077436	0.097	0.052	0.001823574	2.3	ERMP1	1.865384615
STAG22	7.60E-08	0.329682	0.423	0.355	0.001832294	2.3	STAG2	1.191549296
RTN31	7.72E-08	0.3537818	0.278	0.211	0.001860331	2.3	RTN3	1.317535545
FLJ37453	7.77E-08	0.1191716	0.034	0.011	0.00187441	2.3	FLJ37453	3.090909091
NHSL23	8.74E-08	0.326344	0.346	0.278	0.002106802	2.3	NHSL2	1.244604317
JTB2	8.79E-08	0.2384897	0.094	0.051	0.002119363	2.3	JTB	1.843137255
RP11-91P24.71	9.26E-08	0.2628589	0.084	0.044	0.002232271	2.3	RP11-91P2	1.909090909
CPM2	9.63E-08	0.1922795	0.063	0.029	0.002322152	2.3	CPM	2.172413793
ZFP2	9.71E-08	0.1852001	0.083	0.042	0.002340215	2.3	ZFP2	1.976190476
MYOM11	9.76E-08	0.2778127	0.091	0.048	0.002352946	2.3	MYOM1	1.895833333
TMED22	9.88E-08	0.3473255	0.261	0.195	0.002381198	2.3	TMED2	1.338461538
ELMOD21	9.96E-08	0.2836395	0.111	0.064	0.00240071	2.3	ELMOD2	1.734375
LRGUK	1.01E-07	0.1834999	0.051	0.021	0.002427009	2.3	LRGUK	2.428571429
DPH6-AS11	1.04E-07	0.188624	0.055	0.024	0.002507131	2.3	DPH6-AS1	2.291666667
SLC25A172	1.04E-07	0.2441162	0.098	0.053	0.002512048	2.3	SLC25A17	1.849056604
ISCA1	1.05E-07	0.1859443	0.083	0.042	0.002533505	2.3	ISCA1	1.976190476
HSPA131	1.05E-07	0.2088939	0.097	0.052	0.002534748	2.3	HSPA13	1.865384615
DLG51	1.06E-07	0.2907353	0.343	0.262	0.002559401	2.3	DLG5	1.309160305
NOLC11	1.09E-07	0.2591205	0.125	0.074	0.002634618	2.3	NOLC1	1.689189189
ABRACL2	1.11E-07	0.399021	0.471	0.422	0.00267015	2.3	ABRACL	1.116113744
FAM81B2	1.12E-07	0.1358353	0.042	0.016	0.002693331	2.3	FAM81B	2.625
CYTH32	1.13E-07	0.3539103	0.177	0.115	0.002735742	2.3	CYTH3	1.539130435
TDRD9	1.17E-07	0.1456622	0.044	0.017	0.002827687	2.3	TDRD9	2.588235294
GATAD2A2	1.23E-07	0.2963498	0.218	0.152	0.002964368	2.3	GATAD2A	1.434210526
EIF2B32	1.27E-07	0.2756968	0.107	0.061	0.003065898	2.3	EIF2B3	1.754098361
AC019117.2	1.31E-07	0.1092493	0.018	0.004	0.003147858	2.3	AC019117.	4.5
GSTA43	1.31E-07	0.249789	0.103	0.057	0.00315316	2.3	GSTA4	1.807017544
HSPA1B2	1.35E-07	0.2641155	0.114	0.065	0.003253582	2.3	HSPA1B	1.753846154
IRS21	1.39E-07	0.4169235	0.386	0.321	0.003360323	2.3	IRS2	1.202492212
FAM126A2	1.41E-07	0.1730931	0.063	0.029	0.003394979	2.3	FAM126A	2.172413793
HSPA83	1.45E-07	0.4345906	0.513	0.457	0.00350233	2.3	HSPA8	1.122538293
CASK1	1.52E-07	0.3279102	0.441	0.371	0.003661409	2.3	CASK	1.188679245
NRP11	1.53E-07	0.199196	0.107	0.059	0.00369738	2.3	NRP1	1.813559322
SELENBP11	1.65E-07	0.1344769	0.041	0.015	0.003969192	2.3	SELENBP1	2.733333333
CATSPERB2	1.77E-07	0.3398958	0.272	0.203	0.004260424	2.3	CATSPERB	1.339901478
ADIPOR2	1.81E-07	0.3324401	0.126	0.077	0.004357367	2.3	ADIPOR2	1.636363636
ZMPSTE241	1.81E-07	0.2070035	0.124	0.073	0.004375785	2.3	ZMPSTE24	1.698630137
G3BP12	1.83E-07	0.3224568	0.228	0.164	0.004422657	2.3	G3BP1	1.390243902
SNRPG3	1.86E-07	0.3249104	0.562	0.517	0.004495622	2.3	SNRPG	1.087040619
ASMTL	1.88E-07	0.1937504	0.053	0.023	0.004521741	2.3	ASMTL	2.304347826
RUFY1	1.94E-07	0.3302561	0.16	0.104	0.004673899	2.3	RUFY1	1.538461538
ZNF983	1.99E-07	0.3328401	0.146	0.093	0.004799826	2.3	ZNF98	1.569892473
LINC011701	2.27E-07	0.1712292	0.066	0.031	0.00547776	2.3	LINC01170	2.129032258
CAP11	2.36E-07	0.3757609	0.337	0.276	0.005693599	2.3	CAP1	1.221014493

TGM32	2.51E-07	0.4305657	0.053	0.023	0.006041945	2.3	TGM3	2.304347826
BAIAP2	2.54E-07	0.1448837	0.046	0.019	0.006128177	2.3	BAIAP2	2.421052632
LRRFIP13	2.55E-07	0.2748227	0.678	0.63	0.006144709	2.3	LRRFIP1	1.076190476
CANX2	2.56E-07	0.3644249	0.397	0.338	0.006184326	2.3	CANX	1.174556213
TPP1	2.64E-07	0.1741585	0.077	0.039	0.006373864	2.3	TPP1	1.974358974
MEGF92	2.78E-07	0.3393448	0.194	0.132	0.006697156	2.3	MEGF9	1.46969697
LAMTOR31	2.86E-07	0.3088332	0.218	0.156	0.006887359	2.3	LAMTOR3	1.397435897
KLF92	2.87E-07	0.3342723	0.213	0.152	0.006911093	2.3	KLF9	1.401315789
SERPINI11	2.87E-07	0.1012828	0.027	0.008	0.006921506	2.3	SERPINI1	3.375
NSUN72	2.89E-07	0.293878	0.107	0.062	0.00697254	2.3	NSUN7	1.725806452
CREM	3.09E-07	0.3499684	0.154	0.099	0.007441231	2.3	CREM	1.555555556
GOLM11	3.12E-07	0.3093592	0.306	0.233	0.007524383	2.3	GOLM1	1.313304721
GLI32	3.14E-07	0.2801895	0.198	0.135	0.007577674	2.3	GLI3	1.466666667
DNASE1L1	3.15E-07	0.1132835	0.035	0.013	0.00759743	2.3	DNASE1L1	2.692307692
SLC45A2	3.23E-07	0.1003412	0.035	0.013	0.007779144	2.3	SLC45A2	2.692307692
DCD1	3.40E-07	1.238073	0.015	0.003	0.008196803	2.3	DCD	5
MRPS281	3.61E-07	0.1531464	0.059	0.027	0.008716486	2.3	MRPS28	2.185185185
RP11-462L8.1	3.63E-07	0.1013259	0.02	0.005	0.008762209	2.3	RP11-462L8	4
FRZB1	3.71E-07	0.1007727	0.037	0.014	0.008950051	2.3	FRZB	2.642857143
RP11-654A16.3	3.73E-07	0.1618918	0.053	0.024	0.00900264	2.3	RP11-654A	2.208333333
ZC3H42	3.96E-07	0.2054807	0.066	0.032	0.009553773	2.3	ZC3H4	2.0625
CORIN1	4.15E-07	0.1226993	0.049	0.021	0.01001172	2.3	CORIN	2.333333333
MMD	4.35E-07	0.1626653	0.058	0.027	0.01049655	2.3	MMD	2.148148148
TP53TG11	4.39E-07	0.1099986	0.038	0.014	0.01058743	2.3	TP53TG1	2.714285714
KIAA08251	4.50E-07	0.2301548	0.147	0.094	0.01084365	2.3	KIAA0825	1.563829787
EI242	4.57E-07	0.256221	0.126	0.078	0.01102546	2.3	EI24	1.615384615
ADGRL32	4.58E-07	0.1695238	0.167	0.107	0.01104488	2.3	ADGRL3	1.560747664
ZSWIM51	4.60E-07	0.1691078	0.058	0.026	0.01109054	2.3	ZSWIM5	2.230769231
TRIM291	4.82E-07	0.250522	0.098	0.055	0.01161178	2.3	TRIM29	1.781818182
UBL31	5.04E-07	0.3683881	0.265	0.201	0.01214443	2.3	UBL3	1.31840796
WDSUB12	5.15E-07	0.2024277	0.097	0.054	0.01242305	2.3	WDSUB1	1.796296296
AP1M21	5.39E-07	0.2035814	0.128	0.078	0.01300231	2.3	AP1M2	1.641025641
CLIC11	5.43E-07	0.2865216	0.614	0.594	0.01309927	2.3	CLIC1	1.033670034
TIAM23	5.64E-07	0.3799511	0.289	0.228	0.01358955	2.3	TIAM2	1.26754386
ST6GALNAC22	5.88E-07	0.1568653	0.056	0.026	0.01417449	2.3	ST6GALNA(	2.153846154
C21	6.25E-07	0.212425	0.051	0.022	0.01506128	2.3	C2	2.318181818
MYO102	6.28E-07	0.3448723	0.315	0.253	0.01513264	2.3	MYO10	1.245059289
BAZ1A2	6.31E-07	0.4106888	0.437	0.384	0.01520903	2.3	BAZ1A	1.138020833
MRPS231	6.41E-07	0.1901909	0.076	0.039	0.01544833	2.3	MRPS23	1.948717949
DSP1	6.73E-07	0.3228693	0.417	0.354	0.01622221	2.3	DSP	1.177966102
NFKB13	6.77E-07	0.3256743	0.506	0.45	0.0163195	2.3	NFKB1	1.124444444
SRSF124	7.14E-07	0.2094062	0.079	0.041	0.01720972	2.3	SRSF12	1.926829268
RHOBTB32	7.20E-07	0.26719	0.192	0.132	0.01736169	2.3	RHOBTB3	1.454545455
ZCCHC14	7.25E-07	0.3343464	0.114	0.069	0.01749259	2.3	ZCCHC14	1.652173913
SF3B64	7.54E-07	0.2566292	0.638	0.608	0.01817614	2.3	SF3B6	1.049342105
NUP1071	7.88E-07	0.4549244	0.23	0.174	0.01900541	2.3	NUP107	1.32183908
NDUFV21	8.17E-07	0.3534967	0.336	0.271	0.01970249	2.3	NDUFV2	1.239852399
RP11-372K14.21	8.44E-07	0.1505972	0.058	0.027	0.02035882	2.3	RP11-372K	2.148148148
SNRPD32	8.47E-07	0.3636246	0.381	0.324	0.02043294	2.3	SNRPD3	1.175925926
RER1	8.67E-07	0.142564	0.045	0.019	0.02091047	2.3	RER1	2.368421053
RHOA1	8.79E-07	0.2943153	0.469	0.42	0.02118336	2.3	RHOA	1.116666667
C7orf50	8.80E-07	0.1953665	0.032	0.012	0.02121806	2.3	C7orf50	2.666666667

RP11-539L10.32	8.90E-07	0.1602873	0.063	0.031	0.02144906	2.3	RP11-539L1	2.032258065
SYDE21	9.90E-07	0.2011274	0.051	0.023	0.02386084	2.3	SYDE2	2.217391304
ZNF1011	9.95E-07	0.1157599	0.042	0.017	0.02399137	2.3	ZNF101	2.470588235
OSTC4	1.00E-06	0.2912286	0.507	0.458	0.02410943	2.3	OSTC	1.1069869
KCTD2	1.00E-06	0.1344455	0.046	0.02	0.02410957	2.3	KCTD2	2.3
MEGF81	1.00E-06	0.1211548	0.032	0.012	0.02419852	2.3	MEGF8	2.666666667
KIAA1841	1.02E-06	0.2000483	0.083	0.045	0.02455087	2.3	KIAA1841	1.844444444
H3F3A	1.04E-06	0.1458233	0.072	0.037	0.02504649	2.3	H3F3A	1.945945946
PPP1R15A2	1.04E-06	0.2127961	0.063	0.031	0.02516522	2.3	PPP1R15A	2.032258065
TEAD12	1.05E-06	0.3847916	0.507	0.452	0.02525333	2.3	TEAD1	1.121681416
ITGAV2	1.05E-06	0.2202683	0.478	0.399	0.02536046	2.3	ITGAV	1.197994987
LINC003242	1.07E-06	0.1696406	0.056	0.026	0.02570374	2.3	LINC00324	2.153846154
ATF41	1.07E-06	0.3975574	0.438	0.396	0.025742	2.3	ATF4	1.106060606
FCGRT	1.11E-06	0.1066687	0.041	0.017	0.02673321	2.3	FCGRT	2.411764706
GMPR3	1.12E-06	0.1239942	0.028	0.01	0.02708763	2.3	GMPR	2.8
GSTO23	1.13E-06	0.2683884	0.146	0.095	0.02723568	2.3	GSTO2	1.536842105
PTPRK1	1.16E-06	0.4501858	0.751	0.773	0.02789268	2.3	PTPRK	0.971539457
TCTA1	1.17E-06	0.1926186	0.028	0.01	0.02825501	2.3	TCTA	2.8
HNRNPF	1.17E-06	0.3050899	0.216	0.159	0.02831499	2.3	HNRNPF	1.358490566
MAF	1.21E-06	0.1072123	0.027	0.009	0.02911086	2.3	MAF	3
ENTPD3-AS11	1.21E-06	0.1695183	0.053	0.025	0.02916221	2.3	ENTPD3-AS	2.12
TMEM30B	1.21E-06	0.2798288	0.104	0.061	0.02926886	2.3	TMEM30B	1.704918033
NGFRAP13	1.22E-06	0.2069517	0.08	0.043	0.02936713	2.3	NGFRAP1	1.860465116
CDKN2AIP1	1.22E-06	0.1871077	0.056	0.026	0.02945466	2.3	CDKN2AIP	2.153846154
CMTM62	1.24E-06	0.3489326	0.36	0.302	0.02978065	2.3	CMTM6	1.19205298
SEMA3C1	1.25E-06	0.2272723	0.358	0.279	0.03024392	2.3	SEMA3C	1.283154122
EAF21	1.29E-06	0.1416282	0.046	0.02	0.03103326	2.3	EAF2	2.3
SSBP3	1.37E-06	0.2775033	0.181	0.125	0.03298198	2.3	SSBP3	1.448
CLIP1-AS11	1.38E-06	0.1114871	0.039	0.016	0.03334788	2.3	CLIP1-AS1	2.4375
HS6ST1	1.40E-06	0.1538394	0.056	0.027	0.03379044	2.3	HS6ST1	2.074074074
CDH41	1.44E-06	0.1494289	0.041	0.017	0.03477552	2.3	CDH4	2.411764706
MLEC2	1.47E-06	0.305907	0.128	0.081	0.035538	2.3	MLEC	1.580246914
IRF2BP22	1.52E-06	0.3659781	0.185	0.131	0.03666976	2.3	IRF2BP2	1.41221374
CACNA1B1	1.66E-06	0.2129149	0.028	0.01	0.04000637	2.3	CACNA1B	2.8
MAP91	1.66E-06	0.1521589	0.053	0.025	0.04002426	2.3	MAP9	2.12
FAM210B2	1.70E-06	0.2181738	0.104	0.062	0.04094615	2.3	FAM210B	1.677419355
TMEM602	1.73E-06	0.3193922	0.152	0.103	0.04163196	2.3	TMEM60	1.475728155
BCAP31	1.73E-06	0.2599539	0.145	0.095	0.04164001	2.3	BCAP31	1.526315789
GGCX1	1.75E-06	0.2081535	0.06	0.029	0.04225333	2.3	GGCX	2.068965517
MFSD2A1	1.76E-06	0.1021907	0.045	0.019	0.04246027	2.3	MFSD2A	2.368421053
POMP3	1.76E-06	0.2930015	0.653	0.632	0.04246166	2.3	POMP	1.033227848
GLO11	1.78E-06	0.3579383	0.23	0.171	0.04281638	2.3	GLO1	1.34502924
SH2B3	1.82E-06	0.1072531	0.035	0.014	0.04394405	2.3	SH2B3	2.5
TAT1	1.90E-06	0.1716851	0.031	0.011	0.04569935	2.3	TAT	2.818181818
PDCL31	1.90E-06	0.1973437	0.065	0.033	0.04578805	2.3	PDCL3	1.96969697
PAAF11	1.91E-06	0.1121211	0.055	0.026	0.04604536	2.3	PAAF1	2.115384615
EMC101	1.94E-06	0.2240686	0.101	0.06	0.04676301	2.3	EMC10	1.683333333
HNRNPH23	2.06E-06	0.379949	0.282	0.225	0.04955543	2.3	HNRNPH2	1.253333333
RPA31	2.06E-06	0.3031491	0.194	0.139	0.04960514	2.3	RPA3	1.395683453
RP11-12M5.41	2.06E-06	0.1020419	0.041	0.017	0.04967719	2.3	RP11-12M5	2.411764706
LINC009983	2.06E-06	0.4015914	0.18	0.129	0.04973329	2.3	LINC00998	1.395348837
GLCCI12	0	1.611337	0.689	0.236	0	2.4	GLCCI1	2.919491525

SYTL21	0	1.482382	0.857	0.383	0	2.4	SYTL2	2.237597911
FGF132	0	1.331415	0.787	0.396	0	2.4	FGF13	1.987373737
INPP4B2	0	1.252113	0.678	0.27	0	2.4	INPP4B	2.511111111
SLC38A22	0	1.235389	0.678	0.332	0	2.4	SLC38A2	2.042168675
RAB11FIP12	0	1.229003	0.96	0.696	0	2.4	RAB11FIP1	1.379310345
CPEB23	0	1.188119	0.611	0.259	0	2.4	CPEB2	2.359073359
TFPI3	0	1.104141	0.641	0.273	0	2.4	TFPI	2.347985348
OSBPL31	0	1.076053	0.52	0.183	0	2.4	OSBPL3	2.841530055
SAT1	0	1.060056	0.995	0.978	0	2.4	SAT1	1.017382413
MYL12B3	0	1.054367	0.935	0.801	0	2.4	MYL12B	1.167290886
BCAR31	0	1.048912	0.564	0.232	0	2.4	BCAR3	2.431034483
ATP1B1	0	1.047076	0.928	0.734	0	2.4	ATP1B1	1.264305177
EIF4G31	0	1.041572	0.803	0.462	0	2.4	EIF4G3	1.738095238
TMSB4X1	0	1.037931	0.976	0.93	0	2.4	TMSB4X	1.049462366
AREG3	0	1.029026	0.784	0.396	0	2.4	AREG	1.97979798
GOLM12	0	1.027068	0.52	0.186	0	2.4	GOLM1	2.795698925
MALAT13	0	0.7688858	1	1	0	2.4	MALAT1	1
TANC21	2.69E-300	0.8873912	0.751	0.431	6.48E-296	2.4	TANC2	1.742459397
NEBL1	1.94E-289	0.8810169	0.869	0.629	4.68E-285	2.4	NEBL	1.381558029
PRSS231	6.08E-277	0.9352703	0.465	0.181	1.47E-272	2.4	PRSS23	2.569060773
S100A6	1.29E-276	1.494481	0.792	0.537	3.10E-272	2.4	S100A6	1.474860335
THRB1	5.17E-273	0.9804703	0.706	0.419	1.25E-268	2.4	THRB	1.6849642
ITGAV3	2.25E-272	0.8765742	0.668	0.355	5.42E-268	2.4	ITGAV	1.881690141
PPARG1	4.20E-271	0.8905059	0.364	0.121	1.01E-266	2.4	PPARG	3.008264463
STK391	1.77E-267	0.7997393	0.486	0.198	4.26E-263	2.4	STK39	2.454545455
AIM11	4.56E-263	0.9552681	0.69	0.405	1.10E-258	2.4	AIM1	1.703703704
ERBB43	1.54E-258	0.76665	0.792	0.443	3.72E-254	2.4	ERBB4	1.787810384
ALCAM3	2.93E-256	0.8053862	0.796	0.465	7.07E-252	2.4	ALCAM	1.711827957
PTPRK2	2.07E-254	0.7676051	0.914	0.747	4.99E-250	2.4	PTPRK	1.22356091
LRBA2	5.75E-254	0.8962122	0.841	0.605	1.39E-249	2.4	LRBA	1.390082645
C8orf43	6.66E-250	0.554947	0.817	0.47	1.61E-245	2.4	C8orf4	1.738297872
MRPS6	4.50E-243	1.170024	0.509	0.243	1.08E-238	2.4	MRPS6	2.094650206
MAML3	7.33E-242	1.054122	0.586	0.312	1.77E-237	2.4	MAML3	1.878205128
SOX42	3.34E-240	0.8315687	0.945	0.821	8.05E-236	2.4	SOX4	1.151035323
FUT83	1.16E-239	0.9501374	0.492	0.222	2.79E-235	2.4	FUT8	2.216216216
TACSTD21	3.54E-239	1.034005	0.865	0.708	8.54E-235	2.4	TACSTD2	1.221751412
SMIM143	7.36E-239	0.80862	0.647	0.358	1.77E-234	2.4	SMIM14	1.80726257
GNAS3	1.27E-238	0.7083435	0.896	0.729	3.06E-234	2.4	GNAS	1.229080933
DGKH1	2.02E-238	0.8649242	0.547	0.269	4.87E-234	2.4	DGKH	2.033457249
ANO61	4.98E-238	0.7932984	0.748	0.481	1.20E-233	2.4	ANO6	1.555093555
LPP1	1.74E-237	0.6596374	0.974	0.905	4.20E-233	2.4	LPP	1.076243094
MGLL1	3.00E-227	0.7631453	0.39	0.15	7.24E-223	2.4	MGLL	2.6
C16orf451	3.36E-211	0.8086169	0.442	0.193	8.11E-207	2.4	C16orf45	2.29015544
TMEM45B2	2.69E-210	0.6902295	0.359	0.133	6.49E-206	2.4	TMEM45B	2.69924812
TEAD13	5.00E-204	0.8237895	0.668	0.417	1.21E-199	2.4	TEAD1	1.601918465
KIF13A1	5.03E-201	0.9330868	0.612	0.359	1.21E-196	2.4	KIF13A	1.704735376
CACNA2D13	6.37E-197	0.8447212	0.336	0.128	1.54E-192	2.4	CACNA2D1	2.625
SEMA3C2	7.71E-197	0.6964658	0.505	0.243	1.86E-192	2.4	SEMA3C	2.0781893
MAL22	2.11E-196	0.8535018	0.561	0.32	5.09E-192	2.4	MAL2	1.753125
SLC27A43	3.66E-194	0.9188747	0.851	0.68	8.84E-190	2.4	SLC27A4	1.251470588
LIMCH11	1.29E-192	0.6961227	0.725	0.448	3.11E-188	2.4	LIMCH1	1.618303571
TPBG1	4.97E-190	0.7275479	0.369	0.153	1.20E-185	2.4	TPBG	2.411764706

SMAD32	6.97E-188	0.7886643	0.478	0.237	1.68E-183	2.4	SMAD3	2.016877637
TMEM1361	1.12E-186	0.6474136	0.231	0.067	2.70E-182	2.4	TMEM136	3.447761194
ZNF704	4.66E-186	0.7832699	0.338	0.136	1.12E-181	2.4	ZNF704	2.485294118
CPE3	1.01E-184	0.69414	0.403	0.172	2.45E-180	2.4	CPE	2.343023256
CD9	3.66E-184	0.8197529	0.591	0.353	8.82E-180	2.4	CD9	1.674220963
ARHGEF122	7.05E-184	0.7600926	0.763	0.546	1.70E-179	2.4	ARHGEF12	1.397435897
FRYL1	6.73E-183	0.7167201	0.608	0.362	1.62E-178	2.4	FRYL	1.679558011
CAPN82	1.13E-182	0.7392298	0.418	0.186	2.72E-178	2.4	CAPN8	2.247311828
HERC41	1.21E-182	0.7731217	0.716	0.496	2.91E-178	2.4	HERC4	1.443548387
DUSP52	1.67E-179	0.6604512	0.441	0.205	4.04E-175	2.4	DUSP5	2.151219512
SLC39A10	6.42E-178	0.8501405	0.345	0.145	1.55E-173	2.4	SLC39A10	2.379310345
ZNF6441	1.45E-177	0.7923768	0.667	0.438	3.49E-173	2.4	ZNF644	1.52283105
NCEH12	7.73E-177	0.7289132	0.635	0.377	1.86E-172	2.4	NCEH1	1.684350133
TMSB103	3.78E-175	0.8832206	0.855	0.712	9.12E-171	2.4	TMSB10	1.200842697
PGM2L12	6.36E-175	0.7112209	0.425	0.202	1.53E-170	2.4	PGM2L1	2.103960396
TBX33	8.19E-175	0.6474263	0.484	0.235	1.98E-170	2.4	TBX3	2.059574468
TBL1XR11	1.58E-174	0.7820608	0.759	0.552	3.80E-170	2.4	TBL1XR1	1.375
MYO63	8.55E-174	0.543759	0.897	0.737	2.06E-169	2.4	MYO6	1.217096336
MAN2A11	1.51E-169	0.6884817	0.521	0.287	3.65E-165	2.4	MAN2A1	1.81533101
TSPAN52	1.68E-167	0.6214909	0.53	0.272	4.05E-163	2.4	TSPAN5	1.948529412
LSAMP3	5.61E-167	0.6610596	0.683	0.436	1.35E-162	2.4	LSAMP	1.566513761
ANKRD30A3	7.07E-167	0.1942501	0.775	0.421	1.70E-162	2.4	ANKRD30A	1.840855107
PTHLH3	2.70E-166	0.603881	0.44	0.202	6.51E-162	2.4	PTHLH	2.178217822
TNC3	8.32E-166	0.7458328	0.548	0.296	2.01E-161	2.4	TNC	1.851351351
TOM1L21	3.17E-164	0.7095166	0.495	0.269	7.64E-160	2.4	TOM1L2	1.840148699
H2AFZ1	9.15E-164	0.7374091	0.811	0.647	2.21E-159	2.4	H2AFZ	1.253477589
CLSTN23	6.44E-163	0.6287318	0.189	0.052	1.55E-158	2.4	CLSTN2	3.634615385
CDC42BPA1	3.80E-162	0.6936782	0.577	0.35	9.15E-158	2.4	CDC42BPA	1.648571429
THSD41	4.41E-162	0.7545505	0.744	0.533	1.06E-157	2.4	THSD4	1.39587242
LAMA31	7.83E-161	0.6610726	0.373	0.164	1.89E-156	2.4	LAMA3	2.274390244
CXCL133	4.42E-157	0.5274571	0.643	0.374	1.07E-152	2.4	CXCL13	1.719251337
ARHGAP321	1.51E-155	0.7361569	0.607	0.384	3.64E-151	2.4	ARHGAP32	1.580729167
TMOD32	5.71E-155	0.6839924	0.592	0.372	1.38E-150	2.4	TMOD3	1.591397849
OPHN1	1.75E-154	0.9308632	0.511	0.299	4.23E-150	2.4	OPHN1	1.7090301
MYOF1	1.79E-153	0.6814126	0.758	0.573	4.33E-149	2.4	MYOF	1.322862129
SMURF11	4.27E-153	0.6974287	0.559	0.335	1.03E-148	2.4	SMURF1	1.668656716
ANKS1B1	8.84E-153	0.6576097	0.662	0.427	2.13E-148	2.4	ANKS1B	1.550351288
NTN4	2.56E-152	0.7123185	0.495	0.269	6.17E-148	2.4	NTN4	1.840148699
MAST21	9.71E-152	0.6762153	0.403	0.194	2.34E-147	2.4	MAST2	2.077319588
RALGPS22	8.35E-151	0.6843825	0.486	0.264	2.01E-146	2.4	RALGPS2	1.840909091
CD991	2.72E-150	0.5770509	0.287	0.112	6.55E-146	2.4	CD99	2.5625
MACF12	4.64E-148	0.6101751	0.844	0.701	1.12E-143	2.4	MACF1	1.203994294
MTSS11	2.75E-147	0.6915896	0.311	0.129	6.64E-143	2.4	MTSS1	2.410852713
NOVA1	6.52E-146	0.5474875	0.178	0.05	1.57E-141	2.4	NOVA1	3.56
LMO73	1.89E-145	0.7473264	0.349	0.159	4.57E-141	2.4	LMO7	2.194968553
SYTL41	2.34E-142	0.5873845	0.266	0.103	5.65E-138	2.4	SYTL4	2.582524272
GLUD12	7.29E-141	0.6321078	0.459	0.247	1.76E-136	2.4	GLUD1	1.858299595
TMC5	1.54E-140	0.7096018	0.394	0.192	3.72E-136	2.4	TMC5	2.052083333
CACNB42	2.51E-140	0.696535	0.257	0.097	6.04E-136	2.4	CACNB4	2.649484536
TRERF1	5.84E-140	0.6532564	0.316	0.136	1.41E-135	2.4	TRERF1	2.323529412
ECT23	3.96E-139	0.5783851	0.375	0.178	9.54E-135	2.4	ECT2	2.106741573
COL4A53	4.48E-139	0.5930461	0.396	0.191	1.08E-134	2.4	COL4A5	2.073298429

CUX1	2.00E-138	0.6671876	0.528	0.32	4.82E-134	2.4	CUX1	1.65
SERHL21	5.56E-138	0.6376923	0.285	0.115	1.34E-133	2.4	SERHL2	2.47826087
PERP2	5.20E-137	0.6399353	0.626	0.426	1.25E-132	2.4	PERP	1.469483568
BZW11	1.62E-134	0.6588463	0.45	0.249	3.90E-130	2.4	BZW1	1.807228916
GPRC5A1	1.91E-134	0.6982164	0.572	0.361	4.61E-130	2.4	GPRC5A	1.584487535
MAP3K16	1.17E-133	0.6466955	0.648	0.427	2.83E-129	2.4	MAP3K1	1.517564403
TSC22D31	2.16E-132	0.7471349	0.346	0.162	5.20E-128	2.4	TSC22D3	2.135802469
UGCG1	4.37E-132	0.6310074	0.664	0.461	1.05E-127	2.4	UGCG	1.440347072
EPB41L4A	1.73E-128	0.6061622	0.306	0.137	4.17E-124	2.4	EPB41L4A	2.233576642
ACTB3	5.05E-128	0.9150039	0.75	0.626	1.22E-123	2.4	ACTB	1.198083067
ANXA51	6.35E-128	0.6102821	0.544	0.336	1.53E-123	2.4	ANXA5	1.619047619
DHX32	1.47E-127	0.5966784	0.466	0.27	3.55E-123	2.4	DHX32	1.725925926
SDR16C5	2.76E-127	0.479721	0.205	0.07	6.65E-123	2.4	SDR16C5	2.928571429
TM4SF14	2.23E-125	0.6715032	0.935	0.902	5.38E-121	2.4	TM4SF1	1.036585366
STAT5B1	3.97E-124	0.5802355	0.507	0.304	9.58E-120	2.4	STAT5B	1.667763158
ARHGAP421	2.67E-121	0.4840515	0.175	0.056	6.45E-117	2.4	ARHGAP42	3.125
HPX3	4.39E-121	0.604011	0.383	0.188	1.06E-116	2.4	HPX	2.037234043
ASPH2	3.05E-120	0.573279	0.455	0.257	7.35E-116	2.4	ASPH	1.770428016
FBXL21	1.27E-119	0.6075368	0.287	0.128	3.05E-115	2.4	FBXL2	2.2421875
AFF33	2.04E-118	0.3787259	0.537	0.307	4.91E-114	2.4	AFF3	1.749185668
TTTY14	4.43E-117	0.8252419	0.576	0.397	1.07E-112	2.4	TTTY14	1.450881612
DSTN1	6.15E-117	0.6957808	0.714	0.563	1.48E-112	2.4	DSTN	1.268206039
TOX32	1.71E-116	0.5268822	0.318	0.147	4.13E-112	2.4	TOX3	2.163265306
EREG3	2.80E-116	0.5689346	0.439	0.232	6.75E-112	2.4	EREG	1.892241379
JUP1	5.17E-116	0.630102	0.42	0.24	1.25E-111	2.4	JUP	1.75
COX6C3	1.22E-115	0.4160239	0.859	0.747	2.94E-111	2.4	COX6C	1.149933066
B3GLCT	4.18E-115	0.4394558	0.168	0.054	1.01E-110	2.4	B3GLCT	3.111111111
LYPD6B	2.49E-114	0.5447967	0.237	0.095	6.01E-110	2.4	LYPD6B	2.494736842
CASC152	2.17E-113	0.4600797	0.7	0.493	5.23E-109	2.4	CASC15	1.419878296
MIPOL12	1.05E-112	0.5738525	0.33	0.16	2.54E-108	2.4	MIPOL1	2.0625
ANKRD502	4.58E-112	0.4296789	0.187	0.065	1.10E-107	2.4	ANKRD50	2.876923077
S100A102	2.03E-111	0.686687	0.549	0.36	4.88E-107	2.4	S100A10	1.525
OPN32	3.53E-111	0.4303106	0.206	0.077	8.52E-107	2.4	OPN3	2.675324675
ATP2B42	5.51E-110	0.5766816	0.533	0.329	1.33E-105	2.4	ATP2B4	1.62006079
ITGA52	2.12E-109	0.4996826	0.213	0.082	5.11E-105	2.4	ITGA5	2.597560976
KCNMA13	5.31E-109	0.7193211	0.28	0.127	1.28E-104	2.4	KCNMA1	2.204724409
MSI22	1.67E-107	0.6009302	0.503	0.318	4.03E-103	2.4	MSI2	1.581761006
CD633	2.78E-107	0.5842203	0.545	0.356	6.71E-103	2.4	CD63	1.530898876
PGRMC21	3.01E-107	0.4835958	0.251	0.109	7.27E-103	2.4	PGRMC2	2.302752294
STMN1	8.22E-107	0.6369577	0.395	0.223	1.98E-102	2.4	STMN1	1.771300448
TANC11	1.24E-106	0.6467016	0.502	0.324	3.00E-102	2.4	TANC1	1.549382716
ZFAND32	6.19E-106	0.3803638	0.888	0.739	1.49E-101	2.4	ZFAND3	1.201623816
BCL2L1	1.29E-105	0.549427	0.294	0.14	3.10E-101	2.4	BCL2L1	2.1
EXOC6B1	1.44E-105	0.5911088	0.423	0.248	3.48E-101	2.4	EXOC6B	1.705645161
ADAM101	2.40E-105	0.5272254	0.537	0.348	5.78E-101	2.4	ADAM10	1.543103448
SLK1	2.47E-105	0.5378808	0.385	0.211	5.96E-101	2.4	SLK	1.82464455
ZNRF11	3.72E-105	0.5409111	0.3	0.146	8.97E-101	2.4	ZNRF1	2.054794521
DUSP42	2.32E-104	0.5055805	0.259	0.115	5.60E-100	2.4	DUSP4	2.252173913
PSD32	3.84E-104	0.6053244	0.506	0.321	9.25E-100	2.4	PSD3	1.576323988
LRRFIP14	5.48E-104	0.4857056	0.761	0.609	1.32E-99	2.4	LRRFIP1	1.249589491
CNN33	1.77E-103	0.5300883	0.658	0.492	4.26E-99	2.4	CNN3	1.337398374
INADL1	1.45E-102	0.4268169	0.912	0.805	3.50E-98	2.4	INADL	1.132919255

PHLDB22	1.67E-102	0.5783519	0.587	0.392	4.02E-98	2.4	PHLDB2	1.49744898
AKAP132	1.88E-102	0.4988561	0.75	0.589	4.53E-98	2.4	AKAP13	1.273344652
SHROOM32	2.49E-102	0.5303771	0.762	0.61	6.00E-98	2.4	SHROOM3	1.249180328
PKM1	2.16E-100	0.5835033	0.505	0.328	5.22E-96	2.4	PKM	1.539634146
FOXA13	2.32E-100	0.4118232	0.236	0.099	5.59E-96	2.4	FOXA1	2.383838384
ACTG12	3.93E-100	0.7101516	0.813	0.71	9.48E-96	2.4	ACTG1	1.145070423
CYB5A2	3.76E-99	0.5937837	0.411	0.234	9.06E-95	2.4	CYB5A	1.756410256
RTN44	9.15E-99	0.4589579	0.777	0.633	2.21E-94	2.4	RTN4	1.227488152
TMBIM63	9.81E-99	0.4318975	0.919	0.851	2.37E-94	2.4	TMBIM6	1.079905993
AZGP13	1.11E-98	0.5639337	0.702	0.523	2.68E-94	2.4	AZGP1	1.342256214
FAM46A2	2.46E-98	0.570134	0.301	0.149	5.93E-94	2.4	FAM46A	2.020134228
LCOR1	1.81E-97	0.5337089	0.415	0.242	4.36E-93	2.4	LCOR	1.714876033
NDUFV22	1.95E-97	0.5124395	0.421	0.247	4.71E-93	2.4	NDUFV2	1.704453441
CDH19	3.70E-97	0.4976122	0.738	0.584	8.92E-93	2.4	CDH1	1.26369863
MAGI33	4.05E-97	0.4277351	0.555	0.355	9.77E-93	2.4	MAGI3	1.563380282
UGDH3	5.77E-97	0.4371304	0.391	0.211	1.39E-92	2.4	UGDH	1.853080569
FAM107B2	1.16E-96	0.5803484	0.426	0.255	2.80E-92	2.4	FAM107B	1.670588235
KAT6B2	1.53E-96	0.5108635	0.405	0.233	3.70E-92	2.4	KAT6B	1.738197425
BTRC3	2.10E-96	0.4942517	0.414	0.239	5.07E-92	2.4	BTRC	1.732217573
PLAU	2.58E-96	0.4975631	0.191	0.074	6.23E-92	2.4	PLAU	2.581081081
HSPB82	3.93E-96	0.3859446	0.17	0.06	9.48E-92	2.4	HSPB8	2.833333333
DST3	4.90E-96	0.5702801	0.688	0.548	1.18E-91	2.4	DST	1.255474453
TJP12	7.36E-96	0.5157714	0.657	0.483	1.78E-91	2.4	TJP1	1.360248447
PPP3CA3	1.05E-95	0.4773976	0.71	0.54	2.53E-91	2.4	PPP3CA	1.314814815
CCDC732	2.11E-95	0.5438049	0.215	0.089	5.09E-91	2.4	CCDC73	2.415730337
NPEPPS2	8.02E-95	0.4356577	0.646	0.471	1.93E-90	2.4	NPEPPS	1.371549894
RNF1151	2.85E-94	0.5304155	0.407	0.242	6.88E-90	2.4	RNF115	1.681818182
TTC62	7.68E-94	0.456667	0.308	0.153	1.85E-89	2.4	TTC6	2.013071895
ATXN12	1.09E-93	0.4349483	0.671	0.49	2.63E-89	2.4	ATXN1	1.369387755
IER32	6.31E-93	0.609525	0.471	0.295	1.52E-88	2.4	IER3	1.596610169
FGD41	2.15E-92	0.6206386	0.407	0.248	5.18E-88	2.4	FGD4	1.641129032
TCF121	2.23E-91	0.4829314	0.732	0.571	5.37E-87	2.4	TCF12	1.281961471
CBLB1	5.81E-91	0.5572266	0.468	0.298	1.40E-86	2.4	CBLB	1.570469799
PTPRM1	2.10E-90	0.6044638	0.38	0.219	5.07E-86	2.4	PTPRM	1.735159817
LNX22	2.73E-89	0.4585969	0.265	0.128	6.58E-85	2.4	LNX2	2.0703125
VEGFC	5.93E-89	0.3774169	0.137	0.045	1.43E-84	2.4	VEGFC	3.044444444
SH3BP42	2.01E-88	0.4713533	0.281	0.139	4.84E-84	2.4	SH3BP4	2.021582734
VAMP81	3.24E-88	0.4864372	0.833	0.748	7.81E-84	2.4	VAMP8	1.113636364
MTURN1	3.52E-88	0.4493314	0.283	0.143	8.50E-84	2.4	MTURN	1.979020979
RBMS11	6.23E-88	0.5205899	0.489	0.321	1.50E-83	2.4	RBMS1	1.523364486
ZNF675	8.16E-88	0.438693	0.207	0.088	1.97E-83	2.4	ZNF675	2.352272727
PAWR2	1.47E-87	0.5737418	0.67	0.53	3.56E-83	2.4	PAWR	1.264150943
F2RL12	1.82E-87	0.3163901	0.13	0.042	4.38E-83	2.4	F2RL1	3.095238095
NAALADL22	1.85E-87	0.5723281	0.643	0.48	4.45E-83	2.4	NAALADL2	1.339583333
TNFRSF12A1	2.17E-87	0.3520335	0.157	0.057	5.23E-83	2.4	TNFRSF12A	2.754385965
ARID1B2	2.62E-87	0.5124993	0.631	0.473	6.32E-83	2.4	ARID1B	1.334038055
HS6ST22	3.20E-87	0.5345532	0.266	0.128	7.71E-83	2.4	HS6ST2	2.078125
TRAFD11	1.67E-86	0.5442238	0.308	0.164	4.02E-82	2.4	TRAFD1	1.87804878
RRAS22	6.26E-86	0.5170914	0.37	0.218	1.51E-81	2.4	RRAS2	1.697247706
GS1-114I9.31	8.70E-85	0.5263128	0.33	0.183	2.10E-80	2.4	GS1-114I9.	1.803278689
ARPP192	9.87E-85	0.4891279	0.574	0.407	2.38E-80	2.4	ARPP19	1.41031941
TRIO4	1.75E-84	0.3300726	0.904	0.794	4.22E-80	2.4	TRIO	1.138539043

ITSN11	7.55E-84	0.4828752	0.323	0.176	1.82E-79	2.4	ITSN1	1.835227273
PLPP1	8.38E-84	0.4787804	0.224	0.103	2.02E-79	2.4	PLPP1	2.174757282
DUSP103	1.87E-83	0.4466923	0.361	0.202	4.50E-79	2.4	DUSP10	1.787128713
PRKG11	1.96E-83	0.5617139	0.265	0.13	4.74E-79	2.4	PRKG1	2.038461538
TBC1D92	3.32E-83	0.4254155	0.607	0.423	8.00E-79	2.4	TBC1D9	1.43498818
TGFBR12	5.08E-83	0.5354343	0.217	0.098	1.22E-78	2.4	TGFBR1	2.214285714
DOPEY2	2.29E-82	0.3919833	0.178	0.072	5.52E-78	2.4	DOPEY2	2.472222222
TBL1X	4.82E-82	0.5141977	0.342	0.197	1.16E-77	2.4	TBL1X	1.736040609
BTBD9	9.38E-82	0.5797404	0.391	0.239	2.26E-77	2.4	BTBD9	1.635983264
RICTOR2	1.69E-81	0.4172889	0.75	0.602	4.07E-77	2.4	RICTOR	1.245847176
S100A4	2.40E-81	0.5448245	0.263	0.133	5.79E-77	2.4	S100A4	1.977443609
TFAP2A	5.55E-81	0.4413688	0.269	0.137	1.34E-76	2.4	TFAP2A	1.96350365
ESYT22	6.16E-81	0.4619682	0.645	0.491	1.48E-76	2.4	ESYT2	1.313645621
ADAM322	6.41E-81	0.4686652	0.364	0.209	1.55E-76	2.4	ADAM32	1.741626794
PTK21	8.16E-81	0.4049161	0.763	0.622	1.97E-76	2.4	PTK2	1.226688103
DIAPH11	4.21E-80	0.4712236	0.434	0.276	1.02E-75	2.4	DIAPH1	1.572463768
FAT11	4.48E-80	0.5344019	0.324	0.183	1.08E-75	2.4	FAT1	1.770491803
GJB31	4.94E-80	0.2648815	0.091	0.024	1.19E-75	2.4	GJB3	3.791666667
S100A143	4.97E-80	0.5387528	0.818	0.721	1.20E-75	2.4	S100A14	1.134535368
GSK3B2	1.21E-79	0.4689839	0.537	0.377	2.93E-75	2.4	GSK3B	1.424403183
APP1	1.33E-79	0.3546608	0.872	0.759	3.20E-75	2.4	APP	1.148880105
SORT11	4.19E-79	0.4110093	0.256	0.127	1.01E-74	2.4	SORT1	2.015748031
UBE2E2	3.12E-78	0.4800305	0.583	0.429	7.52E-74	2.4	UBE2E2	1.358974359
MBNL22	6.06E-78	0.5156607	0.666	0.523	1.46E-73	2.4	MBNL2	1.273422562
APLP23	1.48E-77	0.454419	0.413	0.256	3.58E-73	2.4	APLP2	1.61328125
FHL22	1.87E-77	0.4687512	0.289	0.153	4.52E-73	2.4	FHL2	1.888888889
BRWD11	5.16E-77	0.4546104	0.508	0.353	1.25E-72	2.4	BRWD1	1.439093484
ZNF91	5.64E-77	0.4784251	0.334	0.194	1.36E-72	2.4	ZNF91	1.721649485
GAN1	8.78E-77	0.4885819	0.309	0.174	2.12E-72	2.4	GAN	1.775862069
AKAP122	1.16E-76	0.6935672	0.112	0.035	2.80E-72	2.4	AKAP12	3.2
PPP2R2A4	1.46E-76	0.4087292	0.666	0.509	3.52E-72	2.4	PPP2R2A	1.308447937
CACNG41	1.63E-76	0.3180558	0.135	0.048	3.92E-72	2.4	CACNG4	2.8125
SLC7A23	2.65E-76	0.3900232	0.453	0.276	6.39E-72	2.4	SLC7A2	1.641304348
MYO5B3	3.03E-76	0.4596402	0.726	0.583	7.31E-72	2.4	MYO5B	1.245283019
STC1	3.51E-76	0.7073073	0.115	0.037	8.47E-72	2.4	STC1	3.108108108
AFF11	7.70E-76	0.5048265	0.509	0.353	1.86E-71	2.4	AFF1	1.441926346
SYBU	9.20E-76	0.4207275	0.168	0.069	2.22E-71	2.4	SYBU	2.434782609
ANGPTL11	1.67E-75	0.4239347	0.254	0.127	4.03E-71	2.4	ANGPTL1	2
SMYD3	4.47E-75	0.4762171	0.478	0.318	1.08E-70	2.4	SMYD3	1.503144654
PTPN133	6.01E-75	0.4081361	0.301	0.16	1.45E-70	2.4	PTPN13	1.88125
SFN1	1.99E-74	0.5833072	0.081	0.021	4.80E-70	2.4	SFN	3.857142857
GSPT13	2.95E-74	0.4502227	0.504	0.347	7.11E-70	2.4	GSPT1	1.452449568
FTX2	3.10E-74	0.4186766	0.693	0.535	7.47E-70	2.4	FTX	1.295327103
CD441	3.18E-74	0.3971907	0.724	0.568	7.66E-70	2.4	CD44	1.274647887
HLA-C	1.18E-73	0.4661306	0.443	0.292	2.86E-69	2.4	HLA-C	1.517123288
TIMP2	1.20E-73	0.3251547	0.141	0.053	2.91E-69	2.4	TIMP2	2.660377358
ZSWIM63	1.59E-73	0.3940226	0.636	0.476	3.83E-69	2.4	ZSWIM6	1.336134454
PARD3B	2.63E-73	0.5553163	0.316	0.184	6.34E-69	2.4	PARD3B	1.717391304
SDK11	5.17E-73	0.4900731	0.257	0.133	1.25E-68	2.4	SDK1	1.932330827
P4HA11	9.83E-73	0.4696269	0.523	0.363	2.37E-68	2.4	P4HA1	1.44077135
UMAD11	1.48E-72	0.4609541	0.327	0.188	3.57E-68	2.4	UMAD1	1.739361702
SPINT1	2.15E-72	0.430428	0.297	0.166	5.20E-68	2.4	SPINT1	1.789156627

TFF13	2.52E-72	0.6269086	0.2	0.091	6.08E-68	2.4	TFF1	2.197802198
TMEM65	1.43E-71	0.4886628	0.322	0.189	3.46E-67	2.4	TMEM65	1.703703704
ADGRL33	4.50E-71	0.4294361	0.202	0.093	1.08E-66	2.4	ADGRL3	2.172043011
ABHD181	6.36E-71	0.4236559	0.482	0.323	1.53E-66	2.4	ABHD18	1.492260062
PACS12	8.67E-71	0.5438114	0.459	0.316	2.09E-66	2.4	PACS1	1.452531646
WWP11	3.09E-70	0.4503409	0.439	0.291	7.45E-66	2.4	WWP1	1.508591065
USP252	7.64E-70	0.4249323	0.385	0.24	1.84E-65	2.4	USP25	1.604166667
PRKAA21	7.64E-70	0.4143146	0.228	0.113	1.84E-65	2.4	PRKAA2	2.017699115
MTCL12	1.35E-69	0.2881644	0.123	0.044	3.27E-65	2.4	MTCL1	2.795454545
AGO4	2.04E-69	0.450436	0.285	0.159	4.93E-65	2.4	AGO4	1.79245283
RUNX11	6.38E-69	0.3437986	0.76	0.625	1.54E-64	2.4	RUNX1	1.216
GDF15	1.40E-68	0.3837746	0.088	0.026	3.39E-64	2.4	GDF15	3.384615385
PARM11	4.21E-68	0.3420407	0.146	0.058	1.02E-63	2.4	PARM1	2.517241379
TNS4	4.29E-68	0.3026725	0.1	0.031	1.03E-63	2.4	TNS4	3.225806452
KLHL2	4.82E-68	0.4036681	0.239	0.122	1.16E-63	2.4	KLHL2	1.959016393
OTUD7B3	5.90E-68	0.4157027	0.424	0.274	1.42E-63	2.4	OTUD7B	1.547445255
KIAA15221	8.38E-68	0.4446235	0.345	0.211	2.02E-63	2.4	KIAA1522	1.63507109
DOCK9	8.60E-68	0.4651528	0.334	0.202	2.07E-63	2.4	DOCK9	1.653465347
MYO1D2	9.15E-68	0.4461228	0.442	0.298	2.21E-63	2.4	MYO1D	1.483221477
GDE12	1.12E-67	0.3773563	0.246	0.129	2.71E-63	2.4	GDE1	1.906976744
AAK11	1.14E-67	0.3990206	0.275	0.15	2.75E-63	2.4	AAK1	1.833333333
RAPH13	1.57E-67	0.4590707	0.468	0.316	3.78E-63	2.4	RAPH1	1.481012658
SAMD4A2	2.57E-67	0.4207269	0.74	0.604	6.20E-63	2.4	SAMD4A	1.225165563
MIR4435-2HG2	1.94E-66	0.5391843	0.617	0.485	4.68E-62	2.4	MIR4435-2	1.272164948
TMEM106B	3.23E-66	0.4151792	0.253	0.136	7.79E-62	2.4	TMEM106E	1.860294118
ZNF217	4.86E-66	0.4813829	0.289	0.168	1.17E-61	2.4	ZNF217	1.720238095
SSBP22	5.18E-66	0.4930346	0.558	0.417	1.25E-61	2.4	SSBP2	1.338129496
ABLIM31	5.74E-66	0.3123119	0.121	0.044	1.38E-61	2.4	ABLIM3	2.75
LASP11	7.71E-66	0.3755628	0.234	0.121	1.86E-61	2.4	LASP1	1.933884298
PDS5B	7.26E-65	0.4908688	0.463	0.326	1.75E-60	2.4	PDS5B	1.420245399
SLC44A42	1.10E-64	0.3543209	0.21	0.103	2.65E-60	2.4	SLC44A4	2.038834951
LYST1	7.61E-64	0.4342227	0.395	0.254	1.83E-59	2.4	LYST	1.55511811
RANBP92	9.28E-64	0.423301	0.422	0.279	2.24E-59	2.4	RANBP9	1.512544803
C1QTNF3-AMACR	1.52E-63	0.4829946	0.438	0.3	3.68E-59	2.4	C1QTNF3-A	1.46
HSPB13	2.00E-63	0.4488903	0.131	0.051	4.82E-59	2.4	HSPB1	2.568627451
SH3D191	2.40E-63	0.4481807	0.478	0.339	5.79E-59	2.4	SH3D19	1.410029499
ASAP11	2.66E-63	0.4370775	0.415	0.273	6.41E-59	2.4	ASAP1	1.52014652
CAB39L	2.71E-63	0.4581683	0.266	0.149	6.53E-59	2.4	CAB39L	1.785234899
PLOD22	3.05E-63	0.490406	0.356	0.221	7.36E-59	2.4	PLOD2	1.610859729
USP341	3.07E-63	0.3748436	0.759	0.634	7.41E-59	2.4	USP34	1.197160883
OGT2	3.44E-63	0.4132378	0.527	0.383	8.31E-59	2.4	OGT	1.375979112
COL12A12	5.42E-63	0.3806134	0.112	0.04	1.31E-58	2.4	COL12A1	2.8
RNF152	9.37E-63	0.3524782	0.175	0.08	2.26E-58	2.4	RNF152	2.1875
ENOX12	1.10E-62	0.4115281	0.238	0.124	2.66E-58	2.4	ENOX1	1.919354839
KLHL241	1.29E-62	0.4225111	0.471	0.332	3.11E-58	2.4	KLHL24	1.418674699
NBEA	1.29E-62	0.409666	0.373	0.232	3.11E-58	2.4	NBEA	1.607758621
ARHGAP292	1.35E-62	0.2916118	0.705	0.552	3.24E-58	2.4	ARHGAP29	1.277173913
KLF123	1.75E-62	0.4608698	0.246	0.132	4.23E-58	2.4	KLF12	1.863636364
GLI33	2.58E-62	0.3793924	0.233	0.121	6.22E-58	2.4	GLI3	1.925619835
NF11	2.71E-62	0.4193028	0.622	0.485	6.54E-58	2.4	NF1	1.282474227
JUND3	2.74E-62	0.3904854	0.281	0.157	6.61E-58	2.4	JUND	1.789808917
KPNA71	3.42E-62	0.2112501	0.064	0.015	8.25E-58	2.4	KPNA7	4.266666667

FGD61	4.55E-62	0.4601248	0.463	0.325	1.10E-57	2.4	FGD6	1.424615385
MAP42	4.81E-62	0.4325107	0.593	0.463	1.16E-57	2.4	MAP4	1.280777538
PKHD1	7.99E-62	0.3055677	0.108	0.038	1.93E-57	2.4	PKHD1	2.842105263
KLHL5	8.99E-62	0.4133756	0.402	0.267	2.17E-57	2.4	KLHL5	1.505617978
LINC007043	2.48E-61	0.2425433	0.076	0.021	5.97E-57	2.4	LINC00704	3.619047619
DUSP163	6.00E-61	0.4119505	0.546	0.401	1.45E-56	2.4	DUSP16	1.36159601
TP53INP1	6.01E-61	0.3271501	0.157	0.069	1.45E-56	2.4	TP53INP1	2.275362319
GLO12	1.56E-60	0.3846063	0.273	0.156	3.77E-56	2.4	GLO1	1.75
ABHD2	1.65E-60	0.3939075	0.243	0.131	3.99E-56	2.4	ABHD2	1.854961832
KLRD12	1.85E-60	0.4232948	0.264	0.147	4.47E-56	2.4	KLRD1	1.795918367
ALOX15B2	2.90E-60	0.2969624	0.12	0.045	7.00E-56	2.4	ALOX15B	2.666666667
PTP4A11	4.85E-60	0.4970024	0.399	0.266	1.17E-55	2.4	PTP4A1	1.5
ARID3B	7.92E-60	0.2681705	0.086	0.027	1.91E-55	2.4	ARID3B	3.185185185
CD241	1.52E-59	0.4905998	0.682	0.564	3.66E-55	2.4	CD24	1.209219858
RELL1	2.00E-59	0.355987	0.208	0.105	4.81E-55	2.4	RELL1	1.980952381
PRSS3	5.19E-59	0.2005737	0.059	0.014	1.25E-54	2.4	PRSS3	4.214285714
ITGA23	7.38E-59	0.3257631	0.522	0.367	1.78E-54	2.4	ITGA2	1.422343324
ANKIB1	2.54E-58	0.4144326	0.474	0.341	6.13E-54	2.4	ANKIB1	1.390029326
EPB41L52	3.12E-58	0.3840041	0.414	0.278	7.53E-54	2.4	EPB41L5	1.489208633
ZNRF21	5.10E-58	0.4484274	0.376	0.245	1.23E-53	2.4	ZNRF2	1.534693878
ERBB2IP3	5.76E-58	0.3911942	0.549	0.409	1.39E-53	2.4	ERBB2IP	1.342298289
LYPD61	2.21E-57	0.291797	0.112	0.042	5.34E-53	2.4	LYPD6	2.666666667
TUBA1A2	2.25E-57	0.7409239	0.357	0.239	5.42E-53	2.4	TUBA1A	1.493723849
MAN1A12	1.35E-56	0.3922631	0.337	0.211	3.25E-52	2.4	MAN1A1	1.597156398
PLEKHA81	1.42E-56	0.3395962	0.205	0.105	3.42E-52	2.4	PLEKHA8	1.952380952
SYNE21	3.05E-56	0.3880352	0.654	0.529	7.37E-52	2.4	SYNE2	1.236294896
RASEF3	3.16E-56	0.3072709	0.398	0.253	7.63E-52	2.4	RASEF	1.57312253
CTNNA1	3.84E-56	0.351312	0.647	0.521	9.26E-52	2.4	CTNNA1	1.24184261
TGIF12	8.04E-56	0.4365976	0.322	0.204	1.94E-51	2.4	TGIF1	1.578431373
CDKN1A1	9.04E-56	0.2747719	0.132	0.055	2.18E-51	2.4	CDKN1A	2.4
OCLN2	1.22E-55	0.4469897	0.449	0.32	2.93E-51	2.4	OCLN	1.403125
DCP21	2.85E-55	0.3491662	0.277	0.161	6.87E-51	2.4	DCP2	1.720496894
GPD21	3.13E-55	0.4796702	0.441	0.314	7.55E-51	2.4	GPD2	1.404458599
PPP2R2D1	3.63E-55	0.3638235	0.279	0.164	8.74E-51	2.4	PPP2R2D	1.701219512
MYL12A2	6.11E-55	0.3657972	0.895	0.863	1.47E-50	2.4	MYL12A	1.037079954
SUN11	1.05E-54	0.3492819	0.219	0.118	2.54E-50	2.4	SUN1	1.855932203
KLF64	1.75E-54	0.3976417	0.669	0.555	4.22E-50	2.4	KLF6	1.205405405
CRABP2	2.22E-54	0.3562114	0.207	0.109	5.36E-50	2.4	CRABP2	1.899082569
DLGAP12	7.06E-54	0.3743737	0.311	0.187	1.70E-49	2.4	DLGAP1	1.663101604
NEAT12	7.14E-54	0.3324352	0.98	0.942	1.72E-49	2.4	NEAT1	1.040339703
N4BP2L22	1.00E-53	0.3340939	0.827	0.709	2.42E-49	2.4	N4BP2L2	1.166431594
MYL62	1.33E-53	0.3226322	0.935	0.934	3.22E-49	2.4	MYL6	1.001070664
MPRIP2	2.42E-53	0.4063894	0.369	0.244	5.84E-49	2.4	MPRIP	1.512295082
WDR1	5.45E-53	0.3950111	0.273	0.163	1.31E-48	2.4	WDR1	1.674846626
EXPH5	7.32E-53	0.3968685	0.275	0.165	1.76E-48	2.4	EXPH5	1.666666667
HIF1A	1.20E-52	0.4667544	0.546	0.425	2.89E-48	2.4	HIF1A	1.284705882
TMCO32	1.37E-52	0.34381	0.226	0.124	3.31E-48	2.4	TMCO3	1.822580645
ANKRD123	1.42E-52	0.291001	0.77	0.658	3.42E-48	2.4	ANKRD12	1.170212766
EXOC21	1.95E-52	0.3797655	0.226	0.126	4.69E-48	2.4	EXOC2	1.793650794
MYO1B1	2.13E-52	0.3686212	0.566	0.429	5.14E-48	2.4	MYO1B	1.319347319
TINCR	2.15E-52	0.1552059	0.056	0.014	5.19E-48	2.4	TINCR	4
NR2C21	3.44E-52	0.3608271	0.275	0.165	8.29E-48	2.4	NR2C2	1.666666667

UBN22	3.58E-52	0.3695279	0.385	0.257	8.63E-48	2.4	UBN2	1.498054475
KIAA0232	6.31E-52	0.3973883	0.292	0.181	1.52E-47	2.4	KIAA0232	1.613259669
ITGA3	1.12E-51	0.3875115	0.274	0.165	2.70E-47	2.4	ITGA3	1.660606061
ARHGEF3	2.11E-51	0.4075083	0.495	0.366	5.08E-47	2.4	ARHGEF3	1.352459016
MARK31	2.52E-51	0.3893752	0.568	0.454	6.08E-47	2.4	MARK3	1.251101322
PRRG4	2.65E-51	0.3815091	0.369	0.248	6.40E-47	2.4	PRRG4	1.487903226
RND33	4.88E-51	0.3495516	0.465	0.333	1.18E-46	2.4	RND3	1.396396396
SARAF3	6.41E-51	0.366553	0.461	0.336	1.55E-46	2.4	SARAF	1.37202381
DRAM11	6.53E-51	0.3594895	0.409	0.279	1.57E-46	2.4	DRAM1	1.465949821
WHSC11	7.84E-51	0.3634639	0.273	0.164	1.89E-46	2.4	WHSC1	1.664634146
ZFP36L1	9.73E-51	0.3940988	0.682	0.578	2.35E-46	2.4	ZFP36L1	1.179930796
DNAJC123	1.29E-50	0.1530445	0.551	0.365	3.12E-46	2.4	DNAJC12	1.509589041
RUFY3	1.34E-50	0.3634802	0.342	0.223	3.23E-46	2.4	RUFY3	1.533632287
CAB392	2.27E-50	0.3745252	0.471	0.344	5.46E-46	2.4	CAB39	1.369186047
MAST42	3.87E-50	0.3463474	0.648	0.509	9.33E-46	2.4	MAST4	1.273084479
SCARB21	5.33E-50	0.3593577	0.274	0.165	1.29E-45	2.4	SCARB2	1.660606061
WDFY21	5.47E-50	0.4304608	0.369	0.25	1.32E-45	2.4	WDFY2	1.476
HIPK23	5.84E-50	0.3932885	0.481	0.356	1.41E-45	2.4	HIPK2	1.351123596
SLC39A63	7.47E-50	0.3458766	0.421	0.284	1.80E-45	2.4	SLC39A6	1.482394366
RAB27B2	7.94E-50	0.4524358	0.242	0.139	1.92E-45	2.4	RAB27B	1.741007194
UBL53	8.26E-50	0.2975824	0.621	0.491	1.99E-45	2.4	UBL5	1.264765784
TTC39C	8.91E-50	0.434526	0.304	0.194	2.15E-45	2.4	TTC39C	1.567010309
SLC38A12	1.25E-49	0.3471507	0.506	0.375	3.01E-45	2.4	SLC38A1	1.349333333
MOCOS	1.75E-49	0.3300807	0.185	0.096	4.22E-45	2.4	MOCOS	1.927083333
MYBL1	1.81E-49	0.3623121	0.138	0.063	4.37E-45	2.4	MYBL1	2.19047619
TBC1D1	2.07E-49	0.3520093	0.319	0.203	5.00E-45	2.4	TBC1D1	1.571428571
BASP12	2.20E-49	0.2587701	0.11	0.044	5.31E-45	2.4	BASP1	2.5
IDS1	2.24E-49	0.2774952	0.148	0.07	5.41E-45	2.4	IDS	2.114285714
C3orf523	3.48E-49	0.3266366	0.254	0.147	8.40E-45	2.4	C3orf52	1.727891156
SMAD22	7.09E-49	0.4021821	0.406	0.288	1.71E-44	2.4	SMAD2	1.409722222
WWC21	8.76E-49	0.3640196	0.294	0.183	2.11E-44	2.4	WWC2	1.606557377
S1PR31	1.19E-48	0.1957102	0.071	0.022	2.88E-44	2.4	S1PR3	3.227272727
FAM63B	2.41E-48	0.3617858	0.286	0.179	5.81E-44	2.4	FAM63B	1.597765363
SPTAN12	3.78E-48	0.3548562	0.322	0.206	9.11E-44	2.4	SPTAN1	1.563106796
ERO1A1	5.28E-48	0.350978	0.551	0.422	1.27E-43	2.4	ERO1A	1.305687204
DDX3X1	5.56E-48	0.359441	0.47	0.347	1.34E-43	2.4	DDX3X	1.354466859
HSPH13	6.47E-48	0.2644691	0.397	0.267	1.56E-43	2.4	HSPH1	1.486891386
TSPAN16	9.40E-48	0.3253036	0.333	0.211	2.27E-43	2.4	TSPAN1	1.578199052
HIF1A-AS22	1.26E-47	0.5738544	0.224	0.131	3.03E-43	2.4	HIF1A-AS2	1.709923664
ABCC3	1.40E-47	0.2914357	0.151	0.072	3.38E-43	2.4	ABCC3	2.097222222
SIPA1L21	1.51E-47	0.3811004	0.222	0.126	3.65E-43	2.4	SIPA1L2	1.761904762
STK38	1.55E-47	0.433784	0.349	0.24	3.75E-43	2.4	STK38	1.454166667
PDGFB1	1.61E-47	0.2523091	0.089	0.032	3.88E-43	2.4	PDGFB	2.78125
FBXL201	2.22E-47	0.369701	0.419	0.3	5.35E-43	2.4	FBXL20	1.396666667
TMEM87B1	2.40E-47	0.3350172	0.2	0.11	5.78E-43	2.4	TMEM87B	1.818181818
INTS63	3.41E-47	0.395911	0.386	0.266	8.23E-43	2.4	INTS6	1.45112782
WASL3	4.90E-47	0.3593348	0.33	0.215	1.18E-42	2.4	WASL	1.534883721
TAOK32	6.58E-47	0.417933	0.574	0.458	1.59E-42	2.4	TAOK3	1.253275109
PARD31	6.96E-47	0.3508392	0.731	0.627	1.68E-42	2.4	PARD3	1.165869219
LSMEM12	7.59E-47	0.3061311	0.134	0.061	1.83E-42	2.4	LSMEM1	2.196721311
SSH21	1.31E-46	0.3890883	0.497	0.374	3.15E-42	2.4	SSH2	1.328877005
RP11-665G4.12	1.36E-46	0.2815919	0.159	0.078	3.27E-42	2.4	RP11-665G	2.038461538

IGF1R2	1.36E-46	0.3597944	0.603	0.488	3.28E-42	2.4	IGF1R	1.235655738
ARHGAP211	1.52E-46	0.4272732	0.346	0.235	3.66E-42	2.4	ARHGAP21	1.472340426
YWHAZ2	1.52E-46	0.3392183	0.682	0.592	3.67E-42	2.4	YWHAZ	1.152027027
FER	1.66E-46	0.3774231	0.335	0.223	3.99E-42	2.4	FER	1.502242152
SETBP1	1.72E-46	0.329411	0.172	0.088	4.15E-42	2.4	SETBP1	1.954545455
ANKRD44	2.75E-46	0.2784111	0.142	0.067	6.64E-42	2.4	ANKRD44	2.119402985
COL4A62	3.37E-46	0.2541699	0.12	0.052	8.12E-42	2.4	COL4A6	2.307692308
CCDC501	3.54E-46	0.3860708	0.258	0.158	8.54E-42	2.4	CCDC50	1.632911392
TCP11L21	3.87E-46	0.3296287	0.178	0.094	9.32E-42	2.4	TCP11L2	1.893617021
SRGAP11	4.00E-46	0.353525	0.47	0.35	9.64E-42	2.4	SRGAP1	1.342857143
STX2	9.25E-46	0.1396246	0.047	0.011	2.23E-41	2.4	STX2	4.272727273
FOXP11	9.96E-46	0.2512658	0.74	0.618	2.40E-41	2.4	FOXP1	1.197411003
ZBTB382	1.15E-45	0.3600248	0.427	0.308	2.78E-41	2.4	ZBTB38	1.386363636
EZR2	1.25E-45	0.3515085	0.485	0.361	3.01E-41	2.4	EZR	1.343490305
CFL12	2.42E-45	0.4442462	0.416	0.302	5.84E-41	2.4	CFL1	1.377483444
MICAL2	2.95E-45	0.3436826	0.221	0.126	7.12E-41	2.4	MICAL2	1.753968254
RPS6KA33	4.40E-45	0.2663147	0.577	0.446	1.06E-40	2.4	RPS6KA3	1.293721973
RERE3	7.51E-45	0.2949649	0.676	0.554	1.81E-40	2.4	RERE	1.220216606
MIB12	8.03E-45	0.3614407	0.49	0.367	1.94E-40	2.4	MIB1	1.335149864
NRF1	8.05E-45	0.3374111	0.258	0.157	1.94E-40	2.4	NRF1	1.643312102
RP11-21L19.11	1.19E-44	0.2227937	0.06	0.018	2.88E-40	2.4	RP11-21L1!	3.333333333
LINC-PINT2	1.40E-44	0.4686788	0.655	0.563	3.38E-40	2.4	LINC-PINT	1.163410302
NRIP31	1.72E-44	0.2365752	0.093	0.036	4.15E-40	2.4	NRIP3	2.583333333
RIC11	1.89E-44	0.3125661	0.265	0.163	4.56E-40	2.4	RIC1	1.625766871
ADCY9	3.33E-44	0.2798823	0.116	0.051	8.03E-40	2.4	ADCY9	2.274509804
VAT11	5.21E-44	0.2977853	0.218	0.127	1.26E-39	2.4	VAT1	1.716535433
ANKS1A1	5.67E-44	0.3182843	0.286	0.182	1.37E-39	2.4	ANKS1A	1.571428571
SRGAP22	6.63E-44	0.3367812	0.227	0.134	1.60E-39	2.4	SRGAP2	1.694029851
LURAP1L2	8.69E-44	0.3637258	0.344	0.233	2.09E-39	2.4	LURAP1L	1.47639485
ACRC3	2.59E-43	0.3143402	0.172	0.09	6.24E-39	2.4	ACRC	1.911111111
SLC20A12	2.77E-43	0.2703779	0.124	0.056	6.68E-39	2.4	SLC20A1	2.214285714
GAREM1	3.24E-43	0.3905243	0.418	0.305	7.80E-39	2.4	GAREM1	1.370491803
MATN33	4.15E-43	0.1885851	0.072	0.024	1.00E-38	2.4	MATN3	3
PRKD12	5.12E-43	0.4053663	0.278	0.178	1.23E-38	2.4	PRKD1	1.561797753
LAMA12	6.96E-43	0.5597917	0.175	0.094	1.68E-38	2.4	LAMA1	1.861702128
SIPA1L12	1.09E-42	0.3937341	0.479	0.37	2.63E-38	2.4	SIPA1L1	1.294594595
UTRN1	1.46E-42	0.3884796	0.373	0.264	3.52E-38	2.4	UTRN	1.412878788
C5orf17	1.67E-42	0.3765996	0.19	0.105	4.03E-38	2.4	C5orf17	1.80952381
MSL12	2.17E-42	0.3193997	0.233	0.14	5.24E-38	2.4	MSL1	1.664285714
FAR2	2.39E-42	0.2512948	0.099	0.041	5.77E-38	2.4	FAR2	2.414634146
SP32	2.90E-42	0.3596141	0.304	0.2	6.98E-38	2.4	SP3	1.52
STK38L1	3.47E-42	0.3252346	0.286	0.18	8.36E-38	2.4	STK38L	1.588888889
KIF3C	4.00E-42	0.2051562	0.082	0.03	9.64E-38	2.4	KIF3C	2.733333333
UBL32	4.51E-42	0.293307	0.295	0.188	1.09E-37	2.4	UBL3	1.569148936
NCOR11	5.69E-42	0.3659963	0.514	0.409	1.37E-37	2.4	NCOR1	1.256723716
QSOX13	7.03E-42	0.3053304	0.235	0.139	1.70E-37	2.4	QSOX1	1.690647482
SLC17A51	1.01E-41	0.2912969	0.164	0.086	2.43E-37	2.4	SLC17A5	1.906976744
FSTL4	1.01E-41	0.1761637	0.047	0.012	2.44E-37	2.4	FSTL4	3.916666667
RNF113	1.03E-41	0.3146145	0.342	0.232	2.49E-37	2.4	RNF11	1.474137931
NRP12	1.60E-41	0.2513688	0.115	0.052	3.86E-37	2.4	NRP1	2.211538462
AC017101.102	1.69E-41	0.2805729	0.159	0.082	4.08E-37	2.4	AC017101.	1.93902439
BNIP3L2	2.02E-41	0.2932816	0.522	0.403	4.86E-37	2.4	BNIP3L	1.29528536

KIAA04302	3.14E-41	0.2909063	0.207	0.119	7.57E-37	2.4	KIAA0430	1.739495798
CKS23	3.14E-41	0.3369646	0.307	0.204	7.57E-37	2.4	CKS2	1.504901961
YAP11	3.37E-41	0.3435683	0.626	0.52	8.12E-37	2.4	YAP1	1.203846154
MKL21	6.98E-41	0.3584642	0.41	0.303	1.68E-36	2.4	MKL2	1.353135314
SRGAP2B	7.36E-41	0.2683514	0.147	0.075	1.78E-36	2.4	SRGAP2B	1.96
ZMYND81	7.95E-41	0.3946498	0.43	0.321	1.92E-36	2.4	ZMYND8	1.339563863
BTG12	8.71E-41	0.342188	0.671	0.567	2.10E-36	2.4	BTG1	1.183421517
RHOBTB1	9.05E-41	0.2377447	0.084	0.032	2.18E-36	2.4	RHOBTB1	2.625
NOP102	1.04E-40	0.3308446	0.64	0.543	2.52E-36	2.4	NOP10	1.178637201
KLF41	1.35E-40	0.3197284	0.183	0.101	3.26E-36	2.4	KLF4	1.811881188
OTUD7A	1.56E-40	0.2908049	0.168	0.09	3.77E-36	2.4	OTUD7A	1.866666667
CDK192	1.60E-40	0.3175155	0.419	0.304	3.86E-36	2.4	CDK19	1.378289474
LYPD31	2.20E-40	0.2339895	0.12	0.055	5.30E-36	2.4	LYPD3	2.181818182
TTLL51	2.53E-40	0.3384	0.378	0.268	6.10E-36	2.4	TTLL5	1.410447761
INHBB	3.65E-40	0.1945363	0.083	0.032	8.79E-36	2.4	INHBB	2.59375
PVRL22	4.21E-40	0.2985522	0.381	0.269	1.01E-35	2.4	PVRL2	1.416356877
MIR34AHG	4.36E-40	0.2614041	0.123	0.058	1.05E-35	2.4	MIR34AHG	2.120689655
ABHD17C1	4.93E-40	0.3289838	0.165	0.089	1.19E-35	2.4	ABHD17C	1.853932584
SPOPL2	4.97E-40	0.3140305	0.357	0.245	1.20E-35	2.4	SPOPL	1.457142857
DIXDC13	5.22E-40	0.4606266	0.187	0.105	1.26E-35	2.4	DIXDC1	1.780952381
DHRSX1	5.61E-40	0.388186	0.368	0.263	1.35E-35	2.4	DHRSX	1.399239544
MYO1E1	9.10E-40	0.3298252	0.568	0.461	2.19E-35	2.4	MYO1E	1.232104121
ADNP	1.13E-39	0.3264925	0.458	0.347	2.73E-35	2.4	ADNP	1.319884726
SRGAP2C1	1.26E-39	0.2615661	0.138	0.069	3.03E-35	2.4	SRGAP2C	2
TSPAN141	1.74E-39	0.2618437	0.159	0.084	4.20E-35	2.4	TSPAN14	1.892857143
PPP1R21	3.03E-39	0.3005858	0.187	0.106	7.31E-35	2.4	PPP1R21	1.764150943
ADIRF3	3.72E-39	0.2663433	0.125	0.059	8.98E-35	2.4	ADIRF	2.118644068
OBFC1	3.73E-39	0.2304782	0.129	0.063	8.99E-35	2.4	OBFC1	2.047619048
DPY19L11	3.75E-39	0.2571057	0.163	0.086	9.03E-35	2.4	DPY19L1	1.895348837
CAP12	4.37E-39	0.3416218	0.367	0.262	1.05E-34	2.4	CAP1	1.400763359
NKAIN3	4.93E-39	0.2565002	0.087	0.035	1.19E-34	2.4	NKAIN3	2.485714286
RAD23B2	5.14E-39	0.2673449	0.586	0.469	1.24E-34	2.4	RAD23B	1.249466951
NUPR1	5.14E-39	0.3951343	0.246	0.154	1.24E-34	2.4	NUPR1	1.597402597
S100A112	5.30E-39	0.3650121	0.791	0.713	1.28E-34	2.4	S100A11	1.109396914
STAT5A3	5.47E-39	0.2670131	0.268	0.167	1.32E-34	2.4	STAT5A	1.604790419
AK31	5.64E-39	0.3353068	0.292	0.194	1.36E-34	2.4	AK3	1.505154639
MAP4K31	1.07E-38	0.3237216	0.35	0.244	2.58E-34	2.4	MAP4K3	1.43442623
STXBP51	1.08E-38	0.2990756	0.198	0.115	2.60E-34	2.4	STXBP5	1.72173913
NOS1AP2	1.40E-38	0.2867751	0.306	0.204	3.37E-34	2.4	NOS1AP	1.5
LDHA2	1.88E-38	0.2854053	0.744	0.655	4.54E-34	2.4	LDHA	1.135877863
LGALS3	2.07E-38	0.3665748	0.291	0.193	5.00E-34	2.4	LGALS3	1.507772021
TFF33	2.07E-38	0.3340246	0.112	0.051	5.00E-34	2.4	TFF3	2.196078431
PRKCH1	2.25E-38	0.3950533	0.325	0.227	5.42E-34	2.4	PRKCH	1.431718062
CDYL1	2.47E-38	0.3063622	0.409	0.297	5.95E-34	2.4	CDYL	1.377104377
MTM11	2.74E-38	0.3671928	0.265	0.172	6.62E-34	2.4	MTM1	1.540697674
ELL22	3.97E-38	0.3218366	0.764	0.686	9.58E-34	2.4	ELL2	1.113702624
MYO5C	4.05E-38	0.3258563	0.228	0.14	9.76E-34	2.4	MYO5C	1.628571429
TWSG12	4.31E-38	0.2370306	0.142	0.072	1.04E-33	2.4	TWSG1	1.972222222
LIN7A1	4.32E-38	0.2142596	0.094	0.04	1.04E-33	2.4	LIN7A	2.35
STK24	8.96E-38	0.3438857	0.261	0.169	2.16E-33	2.4	STK24	1.544378698
GTDC1	8.98E-38	0.3055377	0.172	0.095	2.16E-33	2.4	GTDC1	1.810526316
OTUD13	9.90E-38	0.2070592	0.101	0.044	2.39E-33	2.4	OTUD1	2.295454545

PTPRE	1.00E-37	0.3049588	0.36	0.25	2.41E-33	2.4	PTPRE	1.44
BCL2L11	1.14E-37	0.2881775	0.199	0.116	2.75E-33	2.4	BCL2L11	1.715517241
CFAP692	1.65E-37	0.2878095	0.172	0.095	3.97E-33	2.4	CFAP69	1.810526316
SMCHD12	3.31E-37	0.3110804	0.505	0.392	7.97E-33	2.4	SMCHD1	1.288265306
RGS12	3.72E-37	0.2273797	0.12	0.058	8.97E-33	2.4	RGS12	2.068965517
TSPAN153	3.97E-37	0.2660937	0.171	0.093	9.56E-33	2.4	TSPAN15	1.838709677
FAM102A	4.96E-37	0.2809177	0.212	0.127	1.20E-32	2.4	FAM102A	1.669291339
CHMP52	6.97E-37	0.3281061	0.527	0.423	1.68E-32	2.4	CHMP5	1.245862884
UBE2H2	8.06E-37	0.2516125	0.823	0.745	1.94E-32	2.4	UBE2H	1.104697987
HIST1H2BJ	8.76E-37	0.3283632	0.202	0.121	2.11E-32	2.4	HIST1H2BJ	1.669421488
ANO101	9.17E-37	0.398204	0.357	0.261	2.21E-32	2.4	ANO10	1.367816092
PPM1A1	9.51E-37	0.3019813	0.252	0.162	2.29E-32	2.4	PPM1A	1.555555556
PLIN31	1.60E-36	0.3523282	0.232	0.147	3.85E-32	2.4	PLIN3	1.578231293
EP3002	1.64E-36	0.3040797	0.306	0.207	3.95E-32	2.4	EP300	1.47826087
FSIP13	2.92E-36	0.2025282	0.101	0.045	7.05E-32	2.4	FSIP1	2.244444444
MEGF93	4.07E-36	0.2693031	0.206	0.122	9.81E-32	2.4	MEGF9	1.68852459
TRIM581	4.49E-36	0.1196014	0.031	0.006	1.08E-31	2.4	TRIM58	5.166666667
CBL1	4.77E-36	0.2899535	0.184	0.106	1.15E-31	2.4	CBL	1.735849057
SEPT101	5.03E-36	0.3286851	0.23	0.146	1.21E-31	2.4	Sep-10	1.575342466
BRAF1	5.28E-36	0.3061997	0.512	0.406	1.27E-31	2.4	BRAF	1.261083744
IL13RA11	6.41E-36	0.2866125	0.193	0.113	1.55E-31	2.4	IL13RA1	1.707964602
RPS6KA2	6.42E-36	0.2746361	0.248	0.156	1.55E-31	2.4	RPS6KA2	1.58974359
SQLE2	6.77E-36	0.2963794	0.147	0.08	1.63E-31	2.4	SQLE	1.8375
OSBPL83	1.29E-35	0.2876642	0.326	0.226	3.11E-31	2.4	OSBPL8	1.442477876
ARRB1	1.29E-35	0.2205734	0.118	0.057	3.11E-31	2.4	ARRB1	2.070175439
TSC22D11	1.48E-35	0.3031605	0.462	0.351	3.58E-31	2.4	TSC22D1	1.316239316
PLAUR1	1.67E-35	0.3665141	0.272	0.179	4.03E-31	2.4	PLAUR	1.519553073
FAM219A1	1.78E-35	0.2593887	0.172	0.097	4.28E-31	2.4	FAM219A	1.773195876
RHOBTB33	1.80E-35	0.2719618	0.205	0.122	4.34E-31	2.4	RHOBTB3	1.680327869
WIPF22	2.16E-35	0.3137479	0.225	0.142	5.22E-31	2.4	WIPF2	1.584507042
USP35	2.25E-35	0.3153659	0.389	0.286	5.44E-31	2.4	USP3	1.36013986
ZNF365	2.97E-35	0.1220725	0.035	0.008	7.17E-31	2.4	ZNF365	4.375
ACER2	3.53E-35	0.2130014	0.093	0.041	8.51E-31	2.4	ACER2	2.268292683
UACA2	3.65E-35	0.3089994	0.281	0.187	8.79E-31	2.4	UACA	1.502673797
KTN13	3.72E-35	0.2719421	0.629	0.529	8.96E-31	2.4	KTN1	1.189035917
CCDC92	4.87E-35	0.2148493	0.107	0.05	1.17E-30	2.4	CCDC92	2.14
RP11-255H23.4	5.24E-35	0.2395599	0.117	0.057	1.26E-30	2.4	RP11-255H	2.052631579
LRP11	5.30E-35	0.276397	0.148	0.08	1.28E-30	2.4	LRP11	1.85
MBTPS11	6.93E-35	0.259565	0.18	0.103	1.67E-30	2.4	MBTPS1	1.747572816
RHOB2	9.17E-35	0.2676211	0.178	0.102	2.21E-30	2.4	RHOB	1.745098039
FBXL7	9.68E-35	0.3552008	0.077	0.031	2.33E-30	2.4	FBXL7	2.483870968
FOSL2	1.01E-34	0.3271365	0.323	0.23	2.43E-30	2.4	FOSL2	1.404347826
CDR2L	1.39E-34	0.1783317	0.083	0.035	3.35E-30	2.4	CDR2L	2.371428571
EMP3	1.41E-34	0.1739199	0.048	0.015	3.40E-30	2.4	EMP3	3.2
RAP2B3	1.68E-34	0.3244315	0.256	0.169	4.06E-30	2.4	RAP2B	1.514792899
FANK12	4.25E-34	0.3079176	0.222	0.139	1.02E-29	2.4	FANK1	1.597122302
NR1D2	4.87E-34	0.3009785	0.32	0.222	1.17E-29	2.4	NR1D2	1.441441441
RAB22A1	5.25E-34	0.264544	0.21	0.129	1.27E-29	2.4	RAB22A	1.627906977
UBE2K1	5.80E-34	0.337661	0.469	0.368	1.40E-29	2.4	UBE2K	1.274456522
ATRX1	6.37E-34	0.3205074	0.563	0.469	1.54E-29	2.4	ATRX	1.200426439
TSC22D24	6.98E-34	0.1599147	0.573	0.45	1.68E-29	2.4	TSC22D2	1.273333333
TMEM30A	9.60E-34	0.2973676	0.261	0.174	2.32E-29	2.4	TMEM30A	1.5

MID2	9.76E-34	0.2316743	0.11	0.054	2.35E-29	2.4	MID2	2.037037037
RP1-78O14.13	1.08E-33	0.2442159	0.292	0.191	2.61E-29	2.4	RP1-78O14	1.528795812
FLNB4	1.84E-33	0.3602089	0.55	0.443	4.44E-29	2.4	FLNB	1.241534989
TMEM593	2.11E-33	0.2770417	0.543	0.439	5.08E-29	2.4	TMEM59	1.23690205
GTF2IRD12	2.25E-33	0.2992384	0.24	0.155	5.43E-29	2.4	GTF2IRD1	1.548387097
EIF4G23	2.69E-33	0.320843	0.476	0.38	6.47E-29	2.4	EIF4G2	1.252631579
UBE2R21	2.76E-33	0.3143566	0.453	0.357	6.66E-29	2.4	UBE2R2	1.268907563
MBD22	2.84E-33	0.32291	0.391	0.295	6.84E-29	2.4	MBD2	1.325423729
EMP22	1.06E-32	0.2774528	0.189	0.112	2.55E-28	2.4	EMP2	1.6875
PMAIP12	1.07E-32	0.2988705	0.193	0.117	2.57E-28	2.4	PMAIP1	1.64957265
DDAH1	1.08E-32	0.2755768	0.195	0.119	2.60E-28	2.4	DDAH1	1.638655462
RASAL24	1.33E-32	0.2212152	0.635	0.527	3.20E-28	2.4	RASAL2	1.204933586
BCKDHB	1.55E-32	0.2762624	0.184	0.109	3.75E-28	2.4	BCKDHB	1.688073394
WHSC1L11	2.48E-32	0.3000923	0.343	0.248	5.98E-28	2.4	WHSC1L1	1.383064516
REEP3	3.40E-32	0.2868443	0.35	0.257	8.20E-28	2.4	REEP3	1.361867704
EPB41L4B1	3.53E-32	0.3168052	0.244	0.159	8.52E-28	2.4	EPB41L4B	1.534591195
TMCC32	3.94E-32	0.1744786	0.078	0.032	9.51E-28	2.4	TMCC3	2.4375
CMTM4	4.09E-32	0.2878703	0.22	0.14	9.87E-28	2.4	CMTM4	1.571428571
PLEKHA52	6.19E-32	0.3854001	0.47	0.38	1.49E-27	2.4	PLEKHA5	1.236842105
DACH12	7.78E-32	0.2592733	0.157	0.088	1.88E-27	2.4	DACH1	1.784090909
ATP11C	9.29E-32	0.2725182	0.162	0.094	2.24E-27	2.4	ATP11C	1.723404255
NR6A12	1.21E-31	0.1836178	0.45	0.336	2.91E-27	2.4	NR6A1	1.339285714
TMPRSS2	1.25E-31	0.2881792	0.208	0.131	3.01E-27	2.4	TMPRSS2	1.58778626
LIMD1	2.17E-31	0.2636514	0.125	0.066	5.22E-27	2.4	LIMD1	1.893939394
ARHGAP51	2.25E-31	0.3309748	0.48	0.387	5.44E-27	2.4	ARHGAP5	1.240310078
CDC14A3	2.58E-31	0.2599529	0.233	0.15	6.22E-27	2.4	CDC14A	1.553333333
AC007319.13	2.79E-31	0.2220305	0.147	0.081	6.74E-27	2.4	AC007319.	1.814814815
LLGL2	4.32E-31	0.2468937	0.159	0.092	1.04E-26	2.4	LLGL2	1.72826087
APBB21	4.36E-31	0.2676655	0.344	0.244	1.05E-26	2.4	APBB2	1.409836066
PROSER22	4.87E-31	0.1508641	0.118	0.06	1.17E-26	2.4	PROSER2	1.966666667
NINL	5.30E-31	0.1630064	0.068	0.027	1.28E-26	2.4	NINL	2.518518519
SLMAP2	5.42E-31	0.2383961	0.744	0.663	1.31E-26	2.4	SLMAP	1.122171946
CTSZ3	5.95E-31	0.1911363	0.114	0.057	1.43E-26	2.4	CTSZ	2
ATF42	7.35E-31	0.3153802	0.477	0.384	1.77E-26	2.4	ATF4	1.2421875
PANK33	8.51E-31	0.2588107	0.252	0.167	2.05E-26	2.4	PANK3	1.508982036
SHANK2	9.28E-31	0.2895071	0.31	0.22	2.24E-26	2.4	SHANK2	1.409090909
FAM155A2	1.07E-30	0.4740877	0.104	0.051	2.58E-26	2.4	FAM155A	2.039215686
TOM1L11	1.44E-30	0.3169819	0.343	0.254	3.46E-26	2.4	TOM1L1	1.350393701
MISP	1.46E-30	0.132356	0.045	0.014	3.53E-26	2.4	MISP	3.214285714
HSDL1	1.53E-30	0.1631816	0.074	0.031	3.68E-26	2.4	HSDL1	2.387096774
PPP1R13B2	1.85E-30	0.2922411	0.265	0.18	4.46E-26	2.4	PPP1R13B	1.472222222
SLC37A1	1.91E-30	0.2290249	0.139	0.077	4.61E-26	2.4	SLC37A1	1.805194805
PDK43	1.91E-30	0.2656548	0.133	0.072	4.61E-26	2.4	PDK4	1.847222222
GSN	2.36E-30	0.3151058	0.265	0.183	5.69E-26	2.4	GSN	1.448087432
SLC1A4	4.64E-30	0.1795418	0.075	0.032	1.12E-25	2.4	SLC1A4	2.34375
FAM214B1	4.76E-30	0.1014956	0.039	0.011	1.15E-25	2.4	FAM214B	3.545454545
DDR12	4.83E-30	0.2806896	0.292	0.204	1.16E-25	2.4	DDR1	1.431372549
STIM12	6.88E-30	0.3462224	0.305	0.222	1.66E-25	2.4	STIM1	1.373873874
PTPN121	7.18E-30	0.2907151	0.514	0.419	1.73E-25	2.4	PTPN12	1.22673031
MAPK82	7.66E-30	0.2540295	0.422	0.32	1.85E-25	2.4	MAPK8	1.31875
MCU2	8.63E-30	0.2565374	0.189	0.116	2.08E-25	2.4	MCU	1.629310345
AGR23	8.99E-30	0.1554145	0.337	0.23	2.17E-25	2.4	AGR2	1.465217391

SSFA2	9.11E-30	0.2677233	0.168	0.1	2.20E-25	2.4	SSFA2	1.68
SFMBT22	9.45E-30	0.1936866	0.244	0.158	2.28E-25	2.4	SFMBT2	1.544303797
SLC36A4	9.78E-30	0.2297156	0.148	0.084	2.36E-25	2.4	SLC36A4	1.761904762
SC5D3	1.00E-29	0.3155007	0.255	0.173	2.42E-25	2.4	SC5D	1.473988439
CLIP1	1.05E-29	0.2908854	0.481	0.387	2.53E-25	2.4	CLIP1	1.242894057
CD1091	1.16E-29	0.1515752	0.062	0.024	2.79E-25	2.4	CD109	2.583333333
TES2	1.21E-29	0.2768719	0.391	0.298	2.91E-25	2.4	TES	1.312080537
MT-ND31	1.28E-29	0.2390581	0.993	0.986	3.08E-25	2.4	MT-ND3	1.007099391
ENAH3	1.33E-29	0.1919636	0.642	0.539	3.20E-25	2.4	ENAH	1.19109462
EFHD13	1.49E-29	0.167935	0.401	0.285	3.60E-25	2.4	EFHD1	1.407017544
TMEM2221	1.50E-29	0.1866942	0.095	0.046	3.62E-25	2.4	TMEM222	2.065217391
AMN1	1.51E-29	0.3185129	0.224	0.147	3.64E-25	2.4	AMN1	1.523809524
FARP1	1.60E-29	0.2781373	0.461	0.368	3.87E-25	2.4	FARP1	1.252717391
SSBP31	1.94E-29	0.2520376	0.188	0.116	4.67E-25	2.4	SSBP3	1.620689655
RP11-486O13.41	1.96E-29	0.2237217	0.163	0.095	4.72E-25	2.4	RP11-486O	1.715789474
CDS11	2.21E-29	0.2453109	0.189	0.117	5.34E-25	2.4	CDS1	1.615384615
PIP4K2A2	2.80E-29	0.2511037	0.18	0.11	6.75E-25	2.4	PIP4K2A	1.636363636
SLC10A61	2.88E-29	0.1298091	0.056	0.021	6.94E-25	2.4	SLC10A6	2.666666667
PBX31	5.13E-29	0.2597421	0.143	0.082	1.24E-24	2.4	PBX3	1.743902439
CLDN43	5.14E-29	0.356145	0.268	0.187	1.24E-24	2.4	CLDN4	1.43315508
LAMP13	6.05E-29	0.2061171	0.154	0.089	1.46E-24	2.4	LAMP1	1.730337079
RERG1	6.37E-29	0.3807522	0.268	0.186	1.54E-24	2.4	RERG	1.440860215
SYT17	6.85E-29	0.1904929	0.105	0.053	1.65E-24	2.4	SYT17	1.981132075
PEMT3	7.16E-29	0.3492314	0.076	0.033	1.73E-24	2.4	PEMT	2.303030303
HN1L	7.59E-29	0.219763	0.128	0.07	1.83E-24	2.4	HN1L	1.828571429
FBXL171	8.34E-29	0.2820189	0.348	0.254	2.01E-24	2.4	FBXL17	1.37007874
SDHA	8.41E-29	0.2093072	0.145	0.082	2.03E-24	2.4	SDHA	1.768292683
DAZAP21	8.84E-29	0.2772919	0.502	0.413	2.13E-24	2.4	DAZAP2	1.215496368
BAMBI2	8.89E-29	0.3063982	0.158	0.095	2.14E-24	2.4	BAMBI	1.663157895
RB1CC11	1.13E-28	0.225307	0.567	0.469	2.73E-24	2.4	RB1CC1	1.208955224
NCKAP51	1.46E-28	0.294342	0.25	0.167	3.52E-24	2.4	NCKAP5	1.497005988
FEM1C1	1.73E-28	0.202316	0.131	0.072	4.16E-24	2.4	FEM1C	1.819444444
LAPTM4A3	2.30E-28	0.258699	0.411	0.316	5.56E-24	2.4	LAPTM4A	1.300632911
KRT801	2.34E-28	0.1867508	0.097	0.048	5.64E-24	2.4	KRT80	2.020833333
MAP2K43	2.92E-28	0.2650271	0.528	0.43	7.03E-24	2.4	MAP2K4	1.227906977
AP1S32	3.54E-28	0.2387025	0.184	0.113	8.53E-24	2.4	AP1S3	1.628318584
MPP73	3.60E-28	0.2519682	0.178	0.108	8.68E-24	2.4	MPP7	1.648148148
ARHGEF262	3.93E-28	0.1955314	0.163	0.095	9.47E-24	2.4	ARHGEF26	1.715789474
AMFR2	4.54E-28	0.2489695	0.173	0.105	1.09E-23	2.4	AMFR	1.647619048
DLG52	4.68E-28	0.3559904	0.339	0.252	1.13E-23	2.4	DLG5	1.345238095
ZBTB162	4.78E-28	0.3697908	0.35	0.265	1.15E-23	2.4	ZBTB16	1.320754717
CAMTA12	6.43E-28	0.2290555	0.659	0.57	1.55E-23	2.4	CAMTA1	1.156140351
TSPAN3	7.30E-28	0.2142512	0.124	0.067	1.76E-23	2.4	TSPAN3	1.850746269
FAM188A2	7.34E-28	0.2089934	0.157	0.092	1.77E-23	2.4	FAM188A	1.706521739
SDC3	7.38E-28	0.1463295	0.065	0.027	1.78E-23	2.4	SDC3	2.407407407
HIST1H2AC2	7.95E-28	0.28423	0.45	0.353	1.92E-23	2.4	HIST1H2AC	1.274787535
SLAIN22	8.01E-28	0.2618434	0.204	0.132	1.93E-23	2.4	SLAIN2	1.545454545
C2CD31	1.00E-27	0.2127436	0.115	0.061	2.42E-23	2.4	C2CD3	1.885245902
GPBP1L11	1.54E-27	0.2594928	0.423	0.332	3.71E-23	2.4	GPBP1L1	1.274096386
RNF1491	1.54E-27	0.2996045	0.424	0.337	3.72E-23	2.4	RNF149	1.258160237
MXD12	1.54E-27	0.3349667	0.286	0.201	3.72E-23	2.4	MXD1	1.422885572
BAZ2B1	1.62E-27	0.2588069	0.556	0.468	3.90E-23	2.4	BAZ2B	1.188034188

RP11-371F15.32	1.63E-27	0.2732841	0.138	0.078	3.93E-23	2.4	RP11-371F:	1.769230769
PCDH1	2.01E-27	0.1433123	0.062	0.025	4.83E-23	2.4	PCDH1	2.48
BMPR21	2.56E-27	0.2890796	0.344	0.256	6.18E-23	2.4	BMPR2	1.34375
SLC6A62	4.11E-27	0.2072473	0.108	0.057	9.91E-23	2.4	SLC6A6	1.894736842
KRT72	5.00E-27	0.3267864	0.338	0.251	1.21E-22	2.4	KRT7	1.346613546
BAG13	5.81E-27	0.2086587	0.154	0.091	1.40E-22	2.4	BAG1	1.692307692
DEAF1	9.41E-27	0.2122738	0.125	0.069	2.27E-22	2.4	DEAF1	1.811594203
ZFAND53	1.14E-26	0.2859713	0.508	0.424	2.76E-22	2.4	ZFAND5	1.198113208
PTGER32	1.24E-26	0.2106642	0.076	0.034	2.99E-22	2.4	PTGER3	2.235294118
ZBTB202	1.26E-26	0.2455785	0.606	0.509	3.05E-22	2.4	ZBTB20	1.190569745
VPS8	1.28E-26	0.2460369	0.187	0.118	3.08E-22	2.4	VPS8	1.584745763
FILIP1L2	1.50E-26	0.1999096	0.171	0.104	3.61E-22	2.4	FILIP1L	1.644230769
CDK131	1.56E-26	0.2840222	0.498	0.413	3.77E-22	2.4	CDK13	1.205811138
CASK2	1.74E-26	0.2471517	0.455	0.36	4.19E-22	2.4	CASK	1.263888889
SH2D4A	1.78E-26	0.1963301	0.096	0.049	4.29E-22	2.4	SH2D4A	1.959183673
SRI	1.80E-26	0.2374917	0.147	0.087	4.35E-22	2.4	SRI	1.689655172
LMTK22	1.87E-26	0.3083893	0.261	0.182	4.50E-22	2.4	LMTK2	1.434065934
TBC1D82	1.89E-26	0.323663	0.502	0.419	4.57E-22	2.4	TBC1D8	1.198090692
MYPN1	2.56E-26	0.1098908	0.036	0.011	6.18E-22	2.4	MYPN	3.272727273
ME3	3.03E-26	0.1946547	0.086	0.042	7.32E-22	2.4	ME3	2.047619048
JADE1	3.17E-26	0.2065552	0.088	0.044	7.64E-22	2.4	JADE1	2
NR3C11	3.24E-26	0.2844655	0.305	0.224	7.82E-22	2.4	NR3C1	1.361607143
CARS	4.46E-26	0.2381844	0.169	0.105	1.08E-21	2.4	CARS	1.60952381
GAB12	4.59E-26	0.2118431	0.467	0.373	1.11E-21	2.4	GAB1	1.252010724
RTKN22	5.07E-26	0.1937555	0.094	0.047	1.22E-21	2.4	RTKN2	2
SIN3B	5.24E-26	0.1437472	0.068	0.03	1.26E-21	2.4	SIN3B	2.266666667
LEPR	5.68E-26	0.2672468	0.152	0.092	1.37E-21	2.4	LEPR	1.652173913
PAN32	5.91E-26	0.188026	0.71	0.627	1.42E-21	2.4	PAN3	1.132376396
FGF142	7.25E-26	0.3395236	0.138	0.079	1.75E-21	2.4	FGF14	1.746835443
AMPH3	8.18E-26	0.2517889	0.155	0.093	1.97E-21	2.4	AMPH	1.666666667
ABCA41	8.86E-26	0.1914673	0.084	0.041	2.14E-21	2.4	ABCA4	2.048780488
FEM1B3	1.14E-25	0.2528643	0.235	0.16	2.76E-21	2.4	FEM1B	1.46875
PTBP3	1.22E-25	0.2720496	0.436	0.348	2.93E-21	2.4	PTBP3	1.252873563
CDKN2B1	1.40E-25	0.1057922	0.037	0.012	3.38E-21	2.4	CDKN2B	3.083333333
PDZD83	1.43E-25	0.2392019	0.304	0.221	3.44E-21	2.4	PDZD8	1.375565611
PIK3CB1	1.70E-25	0.2558216	0.29	0.21	4.10E-21	2.4	PIK3CB	1.380952381
ANXA32	1.80E-25	0.303095	0.436	0.349	4.34E-21	2.4	ANXA3	1.249283668
FAM214A1	1.81E-25	0.297558	0.303	0.224	4.37E-21	2.4	FAM214A	1.352678571
SOCS61	2.03E-25	0.2376066	0.187	0.121	4.90E-21	2.4	SOCS6	1.545454545
ATP1B32	2.22E-25	0.320874	0.323	0.245	5.36E-21	2.4	ATP1B3	1.318367347
SMAD5	2.39E-25	0.2284181	0.178	0.113	5.76E-21	2.4	SMAD5	1.575221239
VAV33	2.52E-25	0.2660874	0.365	0.274	6.08E-21	2.4	VAV3	1.332116788
SYT91	2.79E-25	0.1549658	0.081	0.038	6.74E-21	2.4	SYT9	2.131578947
PPM1E1	3.18E-25	0.1925215	0.079	0.038	7.67E-21	2.4	PPM1E	2.078947368
RP11-596C23.61	3.75E-25	0.1808016	0.043	0.015	9.03E-21	2.4	RP11-596C	2.866666667
RAB322	4.17E-25	0.2092334	0.146	0.087	1.01E-20	2.4	RAB32	1.67816092
KDM4B1	4.91E-25	0.1855397	0.138	0.08	1.18E-20	2.4	KDM4B	1.725
LARP1B3	5.15E-25	0.202274	0.291	0.206	1.24E-20	2.4	LARP1B	1.412621359
TUBB4B1	5.49E-25	0.2610431	0.106	0.057	1.32E-20	2.4	TUBB4B	1.859649123
NETO2	7.29E-25	0.1802737	0.094	0.048	1.76E-20	2.4	NETO2	1.958333333
COL28A1	7.42E-25	0.1477111	0.068	0.031	1.79E-20	2.4	COL28A1	2.193548387
DENND1A1	8.22E-25	0.2570668	0.335	0.253	1.98E-20	2.4	DENND1A	1.324110672

RP11-148B18.11	8.89E-25	0.2054893	0.084	0.041	2.14E-20	2.4	RP11-148B	2.048780488
TMEM50A1	9.24E-25	0.2451967	0.269	0.192	2.23E-20	2.4	TMEM50A	1.401041667
PDLIM1	9.96E-25	0.2478213	0.176	0.113	2.40E-20	2.4	PDLIM1	1.557522124
XIAP	1.15E-24	0.2454848	0.195	0.128	2.78E-20	2.4	XIAP	1.5234375
KIAA20261	1.56E-24	0.2448578	0.254	0.181	3.76E-20	2.4	KIAA2026	1.403314917
CREBBP1	1.67E-24	0.2571415	0.373	0.289	4.02E-20	2.4	CREBBP	1.290657439
ATAD2B1	1.73E-24	0.2801908	0.265	0.192	4.17E-20	2.4	ATAD2B	1.380208333
RALGAPA11	1.78E-24	0.2910486	0.374	0.295	4.28E-20	2.4	RALGAPA1	1.26779661
NEDD94	2.05E-24	0.2450916	0.452	0.36	4.94E-20	2.4	NEDD9	1.255555556
TMPRSS11E2	2.17E-24	0.1595433	0.094	0.048	5.23E-20	2.4	TMPRSS11I	1.958333333
PTGR1	2.30E-24	0.6372072	0.341	0.274	5.56E-20	2.4	PTGR1	1.244525547
GALNT2	2.51E-24	0.2543875	0.206	0.138	6.05E-20	2.4	GALNT2	1.492753623
ISCU	2.67E-24	0.1591326	0.103	0.055	6.44E-20	2.4	ISCU	1.872727273
CDA	2.79E-24	0.3105731	0.051	0.02	6.72E-20	2.4	CDA	2.55
TRIB11	2.82E-24	0.2490425	0.292	0.213	6.80E-20	2.4	TRIB1	1.370892019
LARGE2	3.00E-24	0.2539864	0.404	0.316	7.24E-20	2.4	LARGE	1.278481013
KATNBL11	3.52E-24	0.3115887	0.199	0.134	8.48E-20	2.4	KATNBL1	1.485074627
HNRNPLL	3.92E-24	0.3277785	0.184	0.121	9.45E-20	2.4	HNRNPLL	1.520661157
AHNAK1	4.03E-24	0.2725668	0.43	0.345	9.73E-20	2.4	AHNAK	1.246376812
SCNN1A	4.08E-24	0.2151098	0.176	0.112	9.83E-20	2.4	SCNN1A	1.571428571
TM9SF22	4.10E-24	0.2185291	0.333	0.247	9.89E-20	2.4	TM9SF2	1.348178138
SCMH12	4.14E-24	0.2811621	0.306	0.229	9.98E-20	2.4	SCMH1	1.336244541
ST141	4.27E-24	0.2833795	0.239	0.167	1.03E-19	2.4	ST14	1.431137725
ULK1	4.89E-24	0.156187	0.083	0.041	1.18E-19	2.4	ULK1	2.024390244
PTTG1IP	5.92E-24	0.2269721	0.228	0.156	1.43E-19	2.4	PTTG1IP	1.461538462
C9orf84	7.30E-24	0.1949567	0.11	0.061	1.76E-19	2.4	C9orf84	1.803278689
C1GALT1	7.64E-24	0.1941275	0.137	0.081	1.84E-19	2.4	C1GALT1	1.691358025
AP000304.12	1.05E-23	0.1846375	0.125	0.072	2.53E-19	2.4	AP000304.1	1.736111111
ALDH3A21	1.07E-23	0.1567952	0.081	0.04	2.59E-19	2.4	ALDH3A2	2.025
SAMD8	1.45E-23	0.2049645	0.151	0.093	3.49E-19	2.4	SAMD8	1.623655914
PDCD6IP	1.62E-23	0.2268249	0.249	0.176	3.92E-19	2.4	PDCD6IP	1.414772727
TBC1D22A1	1.84E-23	0.2010649	0.214	0.144	4.45E-19	2.4	TBC1D22A	1.486111111
GNB12	2.28E-23	0.2736353	0.413	0.335	5.49E-19	2.4	GNB1	1.232835821
WSB2	2.44E-23	0.1932537	0.136	0.081	5.89E-19	2.4	WSB2	1.679012346
SLC35D2	2.63E-23	0.1923474	0.127	0.074	6.33E-19	2.4	SLC35D2	1.716216216
CERS62	2.79E-23	0.1930994	0.23	0.156	6.73E-19	2.4	CERS6	1.474358974
NUTM2B-AS1	2.79E-23	0.2670676	0.388	0.312	6.74E-19	2.4	NUTM2B-A	1.243589744
RP11-382A20.32	2.82E-23	0.2159686	0.169	0.107	6.80E-19	2.4	RP11-382A	1.579439252
RC3H11	2.88E-23	0.2351979	0.265	0.191	6.95E-19	2.4	RC3H1	1.387434555
KAZN	2.90E-23	0.287454	0.283	0.208	6.99E-19	2.4	KAZN	1.360576923
CPPED1	2.96E-23	0.1165049	0.055	0.023	7.15E-19	2.4	CPPED1	2.391304348
CDR22	3.33E-23	0.2356157	0.236	0.165	8.04E-19	2.4	CDR2	1.43030303
DENND5B	3.36E-23	0.2313608	0.144	0.088	8.09E-19	2.4	DENND5B	1.636363636
SLC25A24	4.00E-23	0.1909801	0.132	0.078	9.63E-19	2.4	SLC25A24	1.692307692
HPS3	4.30E-23	0.1615383	0.079	0.039	1.04E-18	2.4	HPS3	2.025641026
AXL	4.55E-23	0.1470068	0.044	0.017	1.10E-18	2.4	AXL	2.588235294
PCED1B	5.43E-23	0.2087801	0.165	0.104	1.31E-18	2.4	PCED1B	1.586538462
RP11-282I1.11	6.18E-23	0.1470231	0.063	0.028	1.49E-18	2.4	RP11-282I1	2.25
WLS3	6.67E-23	0.191935	0.241	0.166	1.61E-18	2.4	WLS	1.451807229
DLG12	8.72E-23	0.2050632	0.581	0.494	2.10E-18	2.4	DLG1	1.17611336
MLLT31	9.20E-23	0.2806581	0.289	0.212	2.22E-18	2.4	MLLT3	1.363207547
ZNF680	9.37E-23	0.2241757	0.18	0.118	2.26E-18	2.4	ZNF680	1.525423729

CAPZB1	9.82E-23	0.2299629	0.252	0.179	2.37E-18	2.4	CAPZB	1.407821229
TRIM292	1.05E-22	0.1900773	0.094	0.05	2.54E-18	2.4	TRIM29	1.88
DYNLRB12	1.06E-22	0.2248047	0.383	0.302	2.56E-18	2.4	DYNLRB1	1.268211921
CTNND23	1.07E-22	0.2169614	0.216	0.145	2.57E-18	2.4	CTNND2	1.489655172
CKAP4	1.09E-22	0.2549378	0.268	0.197	2.63E-18	2.4	CKAP4	1.360406091
ZDHHC21	1.19E-22	0.175615	0.108	0.06	2.88E-18	2.4	ZDHHC21	1.8
OXSR12	1.29E-22	0.2740603	0.312	0.237	3.10E-18	2.4	OXSR1	1.316455696
TP53INP2	1.37E-22	0.1553801	0.083	0.042	3.31E-18	2.4	TP53INP2	1.976190476
GULP11	1.38E-22	0.3112257	0.269	0.198	3.32E-18	2.4	GULP1	1.358585859
ITGB61	1.40E-22	0.2299639	0.46	0.372	3.37E-18	2.4	ITGB6	1.23655914
RAPGEF23	1.43E-22	0.2206007	0.493	0.411	3.44E-18	2.4	RAPGEF2	1.199513382
ANLN2	1.44E-22	0.1505171	0.075	0.036	3.48E-18	2.4	ANLN	2.083333333
EXTL3	1.55E-22	0.2272125	0.142	0.087	3.75E-18	2.4	EXTL3	1.632183908
FAM114A12	1.67E-22	0.2464984	0.216	0.15	4.02E-18	2.4	FAM114A1	1.44
MCF22	1.75E-22	0.117956	0.051	0.021	4.21E-18	2.4	MCF2	2.428571429
RP11-289H16.1	1.76E-22	0.1795648	0.102	0.056	4.23E-18	2.4	RP11-289H	1.821428571
RP11-455P21.3	1.85E-22	0.1526907	0.063	0.029	4.46E-18	2.4	RP11-455P.	2.172413793
ACTN13	2.50E-22	0.2036132	0.419	0.331	6.02E-18	2.4	ACTN1	1.265861027
RP11-774D14.1	2.89E-22	0.2128802	0.155	0.098	6.97E-18	2.4	RP11-774D	1.581632653
WDR492	3.43E-22	0.147516	0.063	0.028	8.28E-18	2.4	WDR49	2.25
EMC32	3.74E-22	0.2295831	0.303	0.227	9.03E-18	2.4	EMC3	1.334801762
FAM171B3	3.80E-22	0.1705925	0.069	0.032	9.16E-18	2.4	FAM171B	2.15625
MS4A141	4.11E-22	0.116173	0.046	0.018	9.92E-18	2.4	MS4A14	2.555555556
ACADSB3	4.38E-22	0.167109	0.275	0.193	1.06E-17	2.4	ACADSB	1.424870466
TRIM35	4.66E-22	0.1176472	0.046	0.018	1.12E-17	2.4	TRIM35	2.555555556
NFYA1	5.10E-22	0.1745916	0.121	0.07	1.23E-17	2.4	NFYA	1.728571429
VTI1A1	5.12E-22	0.2337398	0.265	0.192	1.24E-17	2.4	VTI1A	1.380208333
AC011288.2	5.42E-22	0.2134301	0.102	0.056	1.31E-17	2.4	AC011288.:	1.821428571
KIF5C1	5.68E-22	0.1479478	0.062	0.028	1.37E-17	2.4	KIF5C	2.214285714
VOPP12	5.90E-22	0.1949584	0.114	0.065	1.42E-17	2.4	VOPP1	1.753846154
SOWAHC1	6.34E-22	0.1702832	0.077	0.038	1.53E-17	2.4	SOWAHC	2.026315789
ZCCHC7	6.46E-22	0.3197727	0.486	0.415	1.56E-17	2.4	ZCCHC7	1.171084337
ZNF4623	9.40E-22	0.2786427	0.372	0.298	2.27E-17	2.4	ZNF462	1.248322148
TMC7	9.78E-22	0.1127273	0.055	0.024	2.36E-17	2.4	TMC7	2.291666667
MITF1	9.97E-22	0.1609033	0.063	0.029	2.41E-17	2.4	MITF	2.172413793
UFD1L	1.01E-21	0.1989943	0.133	0.08	2.43E-17	2.4	UFD1L	1.6625
NR4A22	1.14E-21	0.2127138	0.162	0.103	2.75E-17	2.4	NR4A2	1.572815534
RP11-96H19.1	1.18E-21	0.2742901	0.261	0.188	2.84E-17	2.4	RP11-96H1	1.388297872
ABAT1	1.19E-21	0.1780059	0.096	0.052	2.86E-17	2.4	ABAT	1.846153846
RNF19A3	1.29E-21	0.3067218	0.637	0.564	3.12E-17	2.4	RNF19A	1.129432624
YY12	1.33E-21	0.216812	0.286	0.211	3.21E-17	2.4	YY1	1.355450237
CLIC62	1.35E-21	0.2342075	0.283	0.208	3.26E-17	2.4	CLIC6	1.360576923
LINC001522	1.50E-21	0.262597	0.395	0.312	3.62E-17	2.4	LINC00152	1.266025641
AHCYL2	1.74E-21	0.2445689	0.162	0.105	4.20E-17	2.4	AHCYL2	1.542857143
ATP8B11	1.78E-21	0.2451668	0.472	0.395	4.30E-17	2.4	ATP8B1	1.194936709
CYTH11	1.93E-21	0.2067589	0.211	0.144	4.67E-17	2.4	CYTH1	1.465277778
PGAM1	1.98E-21	0.2152428	0.178	0.118	4.77E-17	2.4	PGAM1	1.508474576
DYNLT31	2.02E-21	0.2374473	0.338	0.26	4.88E-17	2.4	DYNLT3	1.3
RHOD	2.09E-21	0.1020896	0.039	0.014	5.04E-17	2.4	RHOD	2.785714286
MAFK	2.16E-21	0.1073065	0.028	0.009	5.21E-17	2.4	MAFK	3.111111111
TM2D1	2.19E-21	0.2294085	0.164	0.107	5.28E-17	2.4	TM2D1	1.53271028
NCOA61	2.21E-21	0.2417779	0.228	0.162	5.33E-17	2.4	NCOA6	1.407407407

ASXL2	2.31E-21	0.1989424	0.153	0.097	5.57E-17	2.4	ASXL2	1.577319588
LRRC62	2.32E-21	0.1391781	0.078	0.039	5.60E-17	2.4	LRRC6	2
CYB5R4	2.38E-21	0.1646682	0.1	0.055	5.74E-17	2.4	CYB5R4	1.818181818
AC037445.1	2.47E-21	0.1467333	0.093	0.05	5.96E-17	2.4	AC037445.	1.86
GALNT32	2.76E-21	0.2131933	0.263	0.191	6.66E-17	2.4	GALNT3	1.376963351
S100A162	2.91E-21	0.3062369	0.212	0.147	7.02E-17	2.4	S100A16	1.442176871
KPNA31	2.94E-21	0.2431672	0.18	0.12	7.08E-17	2.4	KPNA3	1.5
JDP21	3.34E-21	0.1850981	0.085	0.044	8.04E-17	2.4	JDP2	1.931818182
MALT1	4.29E-21	0.2413397	0.176	0.117	1.03E-16	2.4	MALT1	1.504273504
EBF4	4.35E-21	0.18477	0.1	0.056	1.05E-16	2.4	EBF4	1.785714286
FNBP1L2	4.88E-21	0.2143975	0.486	0.409	1.18E-16	2.4	FNBP1L	1.188264059
PICALM1	5.97E-21	0.2390777	0.513	0.438	1.44E-16	2.4	PICALM	1.171232877
REPS11	6.00E-21	0.3077915	0.368	0.3	1.45E-16	2.4	REPS1	1.226666667
FNBP12	6.13E-21	0.2002101	0.592	0.517	1.48E-16	2.4	FNBP1	1.145067698
FOXO3	7.05E-21	0.2620791	0.426	0.353	1.70E-16	2.4	FOXO3	1.206798867
UGDH-AS13	7.64E-21	0.2001804	0.16	0.103	1.84E-16	2.4	UGDH-AS1	1.553398058
RYBP3	8.33E-21	0.1894839	0.242	0.171	2.01E-16	2.4	RYBP	1.415204678
TEX2	9.25E-21	0.2356757	0.15	0.095	2.23E-16	2.4	TEX2	1.578947368
EHMT1	9.68E-21	0.2387645	0.259	0.19	2.33E-16	2.4	EHMT1	1.363157895
TMEM1652	1.09E-20	0.2576711	0.584	0.512	2.64E-16	2.4	TMEM165	1.140625
ROCK23	1.10E-20	0.2366728	0.356	0.28	2.66E-16	2.4	ROCK2	1.271428571
RAD18	1.16E-20	0.1728881	0.119	0.07	2.80E-16	2.4	RAD18	1.7
CYSTM1	1.49E-20	0.2153537	0.269	0.198	3.59E-16	2.4	CYSTM1	1.358585859
MBNL12	1.54E-20	0.2583947	0.469	0.399	3.72E-16	2.4	MBNL1	1.175438596
MTHFD22	2.11E-20	0.2071009	0.184	0.123	5.10E-16	2.4	MTHFD2	1.495934959
TMTC22	2.18E-20	0.2073625	0.449	0.367	5.26E-16	2.4	TMTC2	1.223433243
ACTR21	2.55E-20	0.2153252	0.311	0.239	6.15E-16	2.4	ACTR2	1.30125523
HTT1	2.58E-20	0.202427	0.226	0.159	6.22E-16	2.4	HTT	1.421383648
PHF201	2.65E-20	0.2715468	0.421	0.347	6.40E-16	2.4	PHF20	1.213256484
ARFGEF32	2.80E-20	0.2192793	0.343	0.266	6.75E-16	2.4	ARFGEF3	1.289473684
ARIH12	3.43E-20	0.2135567	0.572	0.499	8.26E-16	2.4	ARIH1	1.146292585
CTNND12	4.78E-20	0.2143955	0.463	0.386	1.15E-15	2.4	CTNND1	1.199481865
USP532	5.05E-20	0.103531	0.7	0.611	1.22E-15	2.4	USP53	1.145662848
MICU2	5.10E-20	0.2167532	0.228	0.163	1.23E-15	2.4	MICU2	1.398773006
AP000769.11	5.52E-20	0.2760865	0.186	0.127	1.33E-15	2.4	AP000769.:	1.464566929
ZC3HAV12	5.67E-20	0.2119643	0.249	0.18	1.37E-15	2.4	ZC3HAV1	1.383333333
XPR12	5.93E-20	0.2000929	0.237	0.169	1.43E-15	2.4	XPR1	1.402366864
SCCPDH	6.21E-20	0.2008939	0.143	0.09	1.50E-15	2.4	SCCPDH	1.588888889
ZFAT1	6.30E-20	0.1477043	0.092	0.051	1.52E-15	2.4	ZFAT	1.803921569
MAP1LC3B1	8.18E-20	0.2261327	0.207	0.146	1.97E-15	2.4	MAP1LC3B	1.417808219
USP124	1.06E-19	0.2193852	0.25	0.181	2.56E-15	2.4	USP12	1.38121547
CCNYL12	1.09E-19	0.1928646	0.128	0.079	2.63E-15	2.4	CCNYL1	1.620253165
GRB102	1.15E-19	0.2176022	0.241	0.173	2.78E-15	2.4	GRB10	1.393063584
C1orf132	1.18E-19	0.1953864	0.154	0.1	2.84E-15	2.4	C1orf132	1.54
ABHD51	1.29E-19	0.2318093	0.275	0.205	3.11E-15	2.4	ABHD5	1.341463415
PINK11	1.39E-19	0.1605048	0.099	0.056	3.34E-15	2.4	PINK1	1.767857143
SDC1	1.44E-19	0.1078695	0.045	0.018	3.47E-15	2.4	SDC1	2.5
SERF23	1.71E-19	0.230727	0.465	0.384	4.11E-15	2.4	SERF2	1.2109375
ASAP2	1.78E-19	0.1882507	0.117	0.071	4.30E-15	2.4	ASAP2	1.647887324
MEX3C2	1.84E-19	0.1504966	0.106	0.062	4.43E-15	2.4	MEX3C	1.709677419
FAM193A	1.86E-19	0.2202854	0.235	0.171	4.49E-15	2.4	FAM193A	1.374269006
ULK41	1.87E-19	0.2092709	0.163	0.107	4.52E-15	2.4	ULK4	1.523364486

KANSL11	1.89E-19	0.2253253	0.471	0.403	4.56E-15	2.4	KANSL1	1.168734491
RP11-56B16.51	1.94E-19	0.1242282	0.069	0.034	4.68E-15	2.4	RP11-56B1	2.029411765
PHIP1	1.99E-19	0.237117	0.502	0.432	4.79E-15	2.4	PHIP	1.162037037
MARCKSL12	2.13E-19	0.2376981	0.206	0.145	5.13E-15	2.4	MARCKSL1	1.420689655
PIK3C2B	2.21E-19	0.1842156	0.13	0.08	5.33E-15	2.4	PIK3C2B	1.625
PLEKHA1	2.30E-19	0.2338351	0.209	0.149	5.55E-15	2.4	PLEKHA1	1.402684564
CASZ12	2.52E-19	0.1965562	0.142	0.091	6.09E-15	2.4	CASZ1	1.56043956
UBR21	2.56E-19	0.2162309	0.457	0.38	6.17E-15	2.4	UBR2	1.202631579
TTC281	2.56E-19	0.2625395	0.217	0.155	6.17E-15	2.4	TTC28	1.4
PIGT3	2.62E-19	0.1091509	0.061	0.029	6.32E-15	2.4	PIGT	2.103448276
EFTUD1	2.66E-19	0.1954245	0.212	0.147	6.41E-15	2.4	EFTUD1	1.442176871
KLF81	2.82E-19	0.1007725	0.042	0.017	6.81E-15	2.4	KLF8	2.470588235
IRS13	2.86E-19	0.2151575	0.14	0.089	6.90E-15	2.4	IRS1	1.573033708
ROCK1	2.93E-19	0.2417599	0.293	0.227	7.07E-15	2.4	ROCK1	1.290748899
PPP6R32	3.05E-19	0.1907873	0.573	0.499	7.36E-15	2.4	PPP6R3	1.148296593
VLDLR	3.08E-19	0.1680918	0.091	0.051	7.44E-15	2.4	VLDLR	1.784313725
PRDM101	3.36E-19	0.1812096	0.138	0.087	8.10E-15	2.4	PRDM10	1.586206897
ADK2	3.62E-19	0.2244151	0.543	0.473	8.73E-15	2.4	ADK	1.147991543
SH3RF21	3.83E-19	0.1966884	0.05	0.022	9.22E-15	2.4	SH3RF2	2.272727273
OCIAD2	3.87E-19	0.1678966	0.117	0.07	9.32E-15	2.4	OCIAD2	1.671428571
SEC14L12	4.26E-19	0.2346928	0.214	0.153	1.03E-14	2.4	SEC14L1	1.39869281
KIAA1958	5.04E-19	0.2270046	0.192	0.133	1.22E-14	2.4	KIAA1958	1.443609023
KLF31	7.32E-19	0.1959246	0.159	0.106	1.76E-14	2.4	KLF3	1.5
SMG6	7.93E-19	0.2122554	0.24	0.176	1.91E-14	2.4	SMG6	1.363636364
NUP160	8.27E-19	0.2023632	0.185	0.128	2.00E-14	2.4	NUP160	1.4453125
CCNI1	9.19E-19	0.1960644	0.687	0.619	2.21E-14	2.4	CCNI	1.109854604
STAG11	1.08E-18	0.2371424	0.569	0.504	2.61E-14	2.4	STAG1	1.128968254
DUSP1	1.14E-18	0.2752393	0.264	0.198	2.74E-14	2.4	DUSP1	1.333333333
SPATS2L2	1.33E-18	0.2413157	0.297	0.229	3.21E-14	2.4	SPATS2L	1.296943231
ANKRD172	1.44E-18	0.2194241	0.568	0.5	3.47E-14	2.4	ANKRD17	1.136
SH3KBP12	1.45E-18	0.1828778	0.15	0.097	3.50E-14	2.4	SH3KBP1	1.546391753
ETS21	1.54E-18	0.1867666	0.244	0.177	3.72E-14	2.4	ETS2	1.378531073
BCAS41	1.65E-18	0.1075164	0.058	0.027	3.97E-14	2.4	BCAS4	2.148148148
NUDC1	1.72E-18	0.19946	0.129	0.08	4.15E-14	2.4	NUDC	1.6125
GALNT72	1.80E-18	0.2144024	0.215	0.154	4.34E-14	2.4	GALNT7	1.396103896
ATP6AP23	1.83E-18	0.1722244	0.197	0.136	4.41E-14	2.4	ATP6AP2	1.448529412
ETAA1	2.00E-18	0.1181581	0.064	0.032	4.81E-14	2.4	ETAA1	2
PIK3CA1	2.05E-18	0.2096786	0.349	0.277	4.94E-14	2.4	PIK3CA	1.259927798
DOCK52	2.23E-18	0.2236439	0.265	0.2	5.38E-14	2.4	DOCK5	1.325
CAMSAP21	2.45E-18	0.2213396	0.21	0.151	5.90E-14	2.4	CAMSAP2	1.390728477
RP11-4K16.2	2.56E-18	0.1155701	0.039	0.016	6.16E-14	2.4	RP11-4K16	2.4375
NBPF12	2.59E-18	0.181343	0.13	0.082	6.25E-14	2.4	NBPF12	1.585365854
TFE32	2.67E-18	0.1545754	0.095	0.055	6.43E-14	2.4	TFE3	1.727272727
PANX1	2.76E-18	0.102858	0.053	0.024	6.66E-14	2.4	PANX1	2.208333333
IRF2BPL2	2.92E-18	0.2624707	0.214	0.156	7.05E-14	2.4	IRF2BPL	1.371794872
SCYL21	3.23E-18	0.222021	0.341	0.27	7.79E-14	2.4	SCYL2	1.262962963
RBBP62	3.71E-18	0.2167527	0.343	0.272	8.94E-14	2.4	RBBP6	1.261029412
TNFSF13B1	4.18E-18	0.145885	0.084	0.046	1.01E-13	2.4	TNFSF13B	1.826086957
ARF64	4.44E-18	0.2021411	0.264	0.196	1.07E-13	2.4	ARF6	1.346938776
MICALCL1	4.58E-18	0.1462791	0.07	0.036	1.10E-13	2.4	MICALCL	1.944444444
IBTK2	5.08E-18	0.1648435	0.282	0.213	1.23E-13	2.4	IBTK	1.323943662
GNG121	5.25E-18	0.1836052	0.394	0.318	1.27E-13	2.4	GNG12	1.238993711

MLLT43	5.43E-18	0.1455445	0.712	0.634	1.31E-13	2.4	MLLT4	1.123028391
SESN11	5.56E-18	0.1845738	0.169	0.113	1.34E-13	2.4	SESN1	1.495575221
STRN2	5.77E-18	0.203038	0.462	0.388	1.39E-13	2.4	STRN	1.190721649
USP222	6.10E-18	0.1850076	0.169	0.114	1.47E-13	2.4	USP22	1.48245614
BMP2K3	6.70E-18	0.1815822	0.145	0.094	1.62E-13	2.4	BMP2K	1.542553191
MBD51	6.79E-18	0.2270207	0.366	0.297	1.64E-13	2.4	MBD5	1.232323232
DNMBP	6.79E-18	0.1746835	0.108	0.065	1.64E-13	2.4	DNMBP	1.661538462
ZNF264	7.05E-18	0.176596	0.115	0.071	1.70E-13	2.4	ZNF264	1.61971831
RAB12	7.13E-18	0.1933028	0.325	0.252	1.72E-13	2.4	RAB12	1.28968254
NPTN2	7.26E-18	0.1732963	0.255	0.187	1.75E-13	2.4	NPTN	1.363636364
RUBCN2	7.57E-18	0.1728617	0.12	0.074	1.83E-13	2.4	RUBCN	1.621621622
ZNF407	7.70E-18	0.2064334	0.192	0.135	1.86E-13	2.4	ZNF407	1.422222222
DLEU21	9.02E-18	0.2662644	0.278	0.214	2.18E-13	2.4	DLEU2	1.299065421
CREB51	9.36E-18	0.2435377	0.424	0.35	2.26E-13	2.4	CREB5	1.211428571
HMGCLL11	9.42E-18	0.1254497	0.072	0.038	2.27E-13	2.4	HMGCLL1	1.894736842
MARC2	1.07E-17	0.1153336	0.056	0.027	2.57E-13	2.4	MARC2	2.074074074
ATF1	1.19E-17	0.2208343	0.17	0.117	2.88E-13	2.4	ATF1	1.452991453
LOXL2	1.22E-17	0.1315028	0.063	0.032	2.94E-13	2.4	LOXL2	1.96875
CCNY2	1.41E-17	0.1411905	0.322	0.252	3.41E-13	2.4	CCNY	1.277777778
RPGR	1.58E-17	0.186463	0.131	0.084	3.82E-13	2.4	RPGR	1.55952381
RNF1303	1.70E-17	0.2349981	0.227	0.169	4.09E-13	2.4	RNF130	1.343195266
EPHA21	1.86E-17	0.103145	0.05	0.023	4.49E-13	2.4	EPHA2	2.173913043
SHROOM2	2.02E-17	0.1256218	0.085	0.048	4.87E-13	2.4	SHROOM2	1.770833333
NAA252	2.07E-17	0.1855071	0.416	0.341	5.00E-13	2.4	NAA25	1.219941349
MPP3	2.20E-17	0.1049555	0.05	0.023	5.30E-13	2.4	MPP3	2.173913043
CTCF	2.20E-17	0.1737453	0.143	0.094	5.30E-13	2.4	CTCF	1.521276596
NRBP11	2.26E-17	0.1838038	0.12	0.075	5.45E-13	2.4	NRBP1	1.6
GTPBP10	2.35E-17	0.1963421	0.165	0.113	5.67E-13	2.4	GTPBP10	1.460176991
ZDHHC73	2.36E-17	0.1474742	0.102	0.06	5.68E-13	2.4	ZDHHC7	1.7
UBE2G13	3.00E-17	0.186061	0.318	0.248	7.22E-13	2.4	UBE2G1	1.282258065
REPS21	3.00E-17	0.2150627	0.148	0.099	7.24E-13	2.4	REPS2	1.494949495
RP11-189B4.72	3.04E-17	0.1490058	0.09	0.052	7.32E-13	2.4	RP11-189B	1.730769231
EIF12	3.07E-17	0.1417521	0.542	0.472	7.41E-13	2.4	EIF1	1.148305085
ACVR1C2	3.18E-17	0.1114416	0.049	0.022	7.66E-13	2.4	ACVR1C	2.227272727
SLC41A21	3.24E-17	0.2121334	0.166	0.113	7.82E-13	2.4	SLC41A2	1.469026549
ZNF7302	3.76E-17	0.1547849	0.124	0.078	9.08E-13	2.4	ZNF730	1.58974359
RTTN1	3.94E-17	0.1815101	0.105	0.064	9.49E-13	2.4	RTTN	1.640625
FNDC3B1	4.16E-17	0.2356886	0.808	0.767	1.00E-12	2.4	FNDC3B	1.05345502
MEX3D2	4.26E-17	0.1274259	0.061	0.031	1.03E-12	2.4	MEX3D	1.967741935
CMTM3	4.63E-17	0.1261562	0.071	0.038	1.12E-12	2.4	CMTM3	1.868421053
AHI1	4.88E-17	0.2147203	0.32	0.254	1.18E-12	2.4	AHI1	1.25984252
F11R3	5.17E-17	0.1943259	0.256	0.192	1.25E-12	2.4	F11R	1.333333333
SIRT12	5.24E-17	0.2003368	0.147	0.097	1.26E-12	2.4	SIRT1	1.515463918
NBAT11	5.79E-17	0.1894222	0.165	0.112	1.40E-12	2.4	NBAT1	1.473214286
MCL12	5.83E-17	0.1985409	0.387	0.316	1.41E-12	2.4	MCL1	1.224683544
ITM2B3	6.02E-17	0.1962709	0.415	0.341	1.45E-12	2.4	ITM2B	1.217008798
MKNK1	6.27E-17	0.1566333	0.106	0.064	1.51E-12	2.4	MKNK1	1.65625
HILPDA2	7.26E-17	0.2412708	0.338	0.27	1.75E-12	2.4	HILPDA	1.251851852
SETDB21	8.24E-17	0.1356929	0.057	0.028	1.99E-12	2.4	SETDB2	2.035714286
PLS31	8.56E-17	0.2265405	0.142	0.094	2.06E-12	2.4	PLS3	1.510638298
KLC11	9.07E-17	0.1201214	0.074	0.04	2.19E-12	2.4	KLC1	1.85
EPAS11	9.14E-17	0.1900252	0.203	0.145	2.20E-12	2.4	EPAS1	1.4

KIAA13281	9.91E-17	0.2281308	0.172	0.119	2.39E-12	2.4	KIAA1328	1.445378151
ADAM93	1.01E-16	0.1105962	0.634	0.558	2.44E-12	2.4	ADAM9	1.136200717
RP11-1038A11.3	1.17E-16	0.1189154	0.043	0.019	2.83E-12	2.4	RP11-1038.	2.263157895
NHS1	1.30E-16	0.3717414	0.216	0.162	3.12E-12	2.4	NHS	1.333333333
CEACAM6	1.43E-16	0.1279827	0.051	0.024	3.45E-12	2.4	CEACAM6	2.125
CSGALNACT1	1.45E-16	0.186438	0.24	0.178	3.50E-12	2.4	CSGALNAC	1.348314607
WDR37	1.46E-16	0.1933649	0.161	0.11	3.51E-12	2.4	WDR37	1.463636364
CASC3	1.53E-16	0.1923929	0.2	0.144	3.70E-12	2.4	CASC3	1.388888889
C4orf322	1.61E-16	0.1929899	0.146	0.097	3.88E-12	2.4	C4orf32	1.505154639
NPLOC4	1.83E-16	0.2050465	0.201	0.147	4.42E-12	2.4	NPLOC4	1.367346939
RP11-350J20.5	1.85E-16	0.1134354	0.053	0.025	4.46E-12	2.4	RP11-350J2	2.12
ARSJ2	1.87E-16	0.1538473	0.087	0.05	4.51E-12	2.4	ARSJ	1.74
SLC12A6	1.88E-16	0.1395859	0.075	0.041	4.54E-12	2.4	SLC12A6	1.829268293
SP41	1.88E-16	0.1319478	0.061	0.031	4.54E-12	2.4	SP4	1.967741935
CARHSP11	2.23E-16	0.2264105	0.329	0.264	5.37E-12	2.4	CARHSP1	1.246212121
PARD6B2	2.34E-16	0.1831056	0.234	0.174	5.64E-12	2.4	PARD6B	1.344827586
SLC35A32	2.42E-16	0.1857844	0.134	0.088	5.83E-12	2.4	SLC35A3	1.522727273
DGKD2	2.45E-16	0.1814333	0.171	0.118	5.90E-12	2.4	DGKD	1.449152542
KCMF13	2.46E-16	0.1889778	0.402	0.328	5.93E-12	2.4	KCMF1	1.225609756
GATA3	2.47E-16	0.2135414	0.245	0.186	5.95E-12	2.4	GATA3	1.317204301
SEMA3E3	2.48E-16	0.1798999	0.192	0.135	5.97E-12	2.4	SEMA3E	1.422222222
TBRG1	2.55E-16	0.1784939	0.145	0.097	6.14E-12	2.4	TBRG1	1.494845361
LGALS1	2.63E-16	0.3640781	0.118	0.076	6.34E-12	2.4	LGALS1	1.552631579
TMEM1642	2.65E-16	0.1659379	0.108	0.067	6.39E-12	2.4	TMEM164	1.611940299
RP11-174G6.12	2.69E-16	0.1763447	0.1	0.06	6.48E-12	2.4	RP11-174G	1.666666667
SLC30A9	2.75E-16	0.1946974	0.202	0.146	6.64E-12	2.4	SLC30A9	1.383561644
ZFAND61	2.85E-16	0.1926043	0.462	0.393	6.88E-12	2.4	ZFAND6	1.175572519
SPON12	2.86E-16	0.1181143	0.062	0.032	6.90E-12	2.4	SPON1	1.9375
AQP31	2.95E-16	0.1808812	0.088	0.051	7.12E-12	2.4	AQP3	1.725490196
WNT9A2	3.53E-16	0.100095	0.056	0.028	8.50E-12	2.4	WNT9A	2
CREBZF2	3.81E-16	0.1821042	0.103	0.062	9.19E-12	2.4	CREBZF	1.661290323
RHBDD11	3.82E-16	0.2006298	0.187	0.133	9.22E-12	2.4	RHBDD1	1.406015038
CMYA51	3.87E-16	0.1664345	0.149	0.1	9.32E-12	2.4	CMYA5	1.49
MPZL31	4.20E-16	0.2306303	0.368	0.308	1.01E-11	2.4	MPZL3	1.194805195
SEPW12	4.62E-16	0.1869474	0.21	0.154	1.11E-11	2.4	SEPW1	1.363636364
CHMP2B1	4.68E-16	0.1728698	0.203	0.146	1.13E-11	2.4	CHMP2B	1.390410959
TNRC18	5.56E-16	0.1677189	0.12	0.076	1.34E-11	2.4	TNRC18	1.578947368
MFSD112	5.77E-16	0.204393	0.135	0.09	1.39E-11	2.4	MFSD11	1.5
BUB3	5.99E-16	0.1735121	0.13	0.085	1.45E-11	2.4	BUB3	1.529411765
MAP71	6.78E-16	0.2319089	0.415	0.352	1.63E-11	2.4	MAP7	1.178977273
KITLG	6.80E-16	0.1975081	0.076	0.043	1.64E-11	2.4	KITLG	1.76744186
OTUD31	7.09E-16	0.1144917	0.077	0.043	1.71E-11	2.4	OTUD3	1.790697674
LINC012071	7.23E-16	0.1054279	0.06	0.03	1.74E-11	2.4	LINC01207	2
ATP2B11	7.71E-16	0.1434504	0.134	0.087	1.86E-11	2.4	ATP2B1	1.540229885
RP11-15H20.7	7.75E-16	0.1457436	0.094	0.056	1.87E-11	2.4	RP11-15H2	1.678571429
SCD1	9.79E-16	0.2036244	0.169	0.119	2.36E-11	2.4	SCD	1.420168067
RPS6KC1	1.01E-15	0.195358	0.15	0.102	2.44E-11	2.4	RPS6KC1	1.470588235
HECA	1.18E-15	0.1760301	0.177	0.125	2.84E-11	2.4	HECA	1.416
TUBB2	1.19E-15	0.2824335	0.386	0.33	2.86E-11	2.4	TUBB	1.16969697
MIDN	1.30E-15	0.1563747	0.098	0.059	3.14E-11	2.4	MIDN	1.661016949
RCN13	1.32E-15	0.1736888	0.203	0.147	3.17E-11	2.4	RCN1	1.380952381
FCHO2	1.57E-15	0.2180747	0.223	0.167	3.78E-11	2.4	FCHO2	1.335329341

ZCCHC141	1.91E-15	0.1606832	0.104	0.064	4.61E-11	2.4	ZCCHC14	1.625
HIVEP12	1.96E-15	0.1804153	0.245	0.185	4.72E-11	2.4	HIVEP1	1.324324324
DCUN1D21	2.01E-15	0.1248145	0.107	0.066	4.84E-11	2.4	DCUN1D2	1.621212121
SUSD11	2.04E-15	0.1749515	0.143	0.096	4.92E-11	2.4	SUSD1	1.489583333
YWHAG1	2.17E-15	0.1859818	0.309	0.246	5.23E-11	2.4	YWHAG	1.256097561
CTNNAL12	2.35E-15	0.1652352	0.173	0.122	5.67E-11	2.4	CTNNAL1	1.418032787
INPP4A	2.46E-15	0.1484174	0.084	0.049	5.93E-11	2.4	INPP4A	1.714285714
TMEM40	2.61E-15	0.1468899	0.066	0.036	6.29E-11	2.4	TMEM40	1.833333333
RP11-65I12.11	2.89E-15	0.1554716	0.084	0.049	6.97E-11	2.4	RP11-65I12	1.714285714
HOMER13	3.26E-15	0.1291713	0.159	0.109	7.87E-11	2.4	HOMER1	1.458715596
MGEA51	3.44E-15	0.2121711	0.459	0.401	8.30E-11	2.4	MGEA5	1.144638404
TMEM30B1	3.46E-15	0.1437596	0.094	0.057	8.33E-11	2.4	TMEM30B	1.649122807
RFWD23	3.49E-15	0.199289	0.356	0.293	8.41E-11	2.4	RFWD2	1.215017065
ATP6V0B	3.63E-15	0.1445587	0.057	0.029	8.76E-11	2.4	ATP6V0B	1.965517241
DLGAP4	3.78E-15	0.1960036	0.174	0.124	9.11E-11	2.4	DLGAP4	1.403225806
CAST1	3.83E-15	0.1529816	0.57	0.503	9.23E-11	2.4	CAST	1.133200795
WSB11	3.85E-15	0.1869071	0.448	0.382	9.28E-11	2.4	WSB1	1.172774869
DLG3	4.03E-15	0.1573419	0.113	0.073	9.72E-11	2.4	DLG3	1.547945205
MEF2D	4.14E-15	0.146734	0.101	0.062	9.98E-11	2.4	MEF2D	1.629032258
MEIS1	4.33E-15	0.1886279	0.104	0.065	1.04E-10	2.4	MEIS1	1.6
PEBP43	4.35E-15	0.1343911	0.137	0.09	1.05E-10	2.4	PEBP4	1.522222222
XPO1	5.25E-15	0.1922238	0.242	0.185	1.27E-10	2.4	XPO1	1.308108108
ST8SIA61	5.31E-15	0.205521	0.175	0.123	1.28E-10	2.4	ST8SIA6	1.422764228
GOLPH31	5.93E-15	0.1959974	0.316	0.256	1.43E-10	2.4	GOLPH3	1.234375
C20orf1942	6.39E-15	0.1985688	0.221	0.166	1.54E-10	2.4	C20orf194	1.331325301
ELF13	6.81E-15	0.1583701	0.592	0.52	1.64E-10	2.4	ELF1	1.138461538
FLVCR2	6.93E-15	0.1122546	0.067	0.037	1.67E-10	2.4	FLVCR2	1.810810811
FBXO7	7.40E-15	0.1947787	0.19	0.139	1.78E-10	2.4	FBXO7	1.366906475
MAPK62	7.63E-15	0.1925055	0.328	0.265	1.84E-10	2.4	MAPK6	1.237735849
C1orf228	7.80E-15	0.2054555	0.074	0.042	1.88E-10	2.4	C1orf228	1.761904762
TRIB3	8.11E-15	0.1078211	0.042	0.02	1.96E-10	2.4	TRIB3	2.1
RP11-368L12.11	8.45E-15	0.2066102	0.143	0.097	2.04E-10	2.4	RP11-368L1	1.474226804
ITGB5	8.46E-15	0.1502625	0.096	0.059	2.04E-10	2.4	ITGB5	1.627118644
RNASE4	9.22E-15	0.1738079	0.147	0.101	2.22E-10	2.4	RNASE4	1.455445545
ANXA6	9.30E-15	0.1647981	0.141	0.096	2.24E-10	2.4	ANXA6	1.46875
EXOC42	9.33E-15	0.1828383	0.394	0.329	2.25E-10	2.4	EXOC4	1.197568389
TRIP121	9.86E-15	0.1946136	0.385	0.32	2.38E-10	2.4	TRIP12	1.203125
GABPA1	9.98E-15	0.1266863	0.081	0.047	2.41E-10	2.4	GABPA	1.723404255
UNC13B1	1.01E-14	0.1647027	0.115	0.074	2.42E-10	2.4	UNC13B	1.554054054
EIF2AK13	1.02E-14	0.1773158	0.154	0.107	2.45E-10	2.4	EIF2AK1	1.439252336
KDM2A1	1.03E-14	0.1631555	0.493	0.429	2.47E-10	2.4	KDM2A	1.149184149
MTMR9	1.04E-14	0.1409922	0.073	0.042	2.51E-10	2.4	MTMR9	1.738095238
AF127936.92	1.05E-14	0.1469585	0.194	0.139	2.52E-10	2.4	AF127936.9	1.395683453
ZC2HC1A	1.07E-14	0.1386722	0.073	0.041	2.59E-10	2.4	ZC2HC1A	1.780487805
IGFBP23	1.08E-14	0.1063349	0.079	0.045	2.60E-10	2.4	IGFBP2	1.755555556
YPEL52	1.12E-14	0.1909186	0.451	0.392	2.70E-10	2.4	YPEL5	1.150510204
RNF41	1.13E-14	0.1899126	0.214	0.16	2.73E-10	2.4	RNF4	1.3375
FAM208B1	1.25E-14	0.1951826	0.288	0.228	3.01E-10	2.4	FAM208B	1.263157895
TARSL2	1.31E-14	0.1199253	0.05	0.025	3.15E-10	2.4	TARSL2	2
TIPARP	1.38E-14	0.1652194	0.165	0.116	3.32E-10	2.4	TIPARP	1.422413793
CDS2	1.38E-14	0.1481187	0.121	0.079	3.34E-10	2.4	CDS2	1.53164557
SERINC1	1.45E-14	0.167473	0.393	0.327	3.50E-10	2.4	SERINC1	1.201834862

ZNF5321	1.46E-14	0.1633017	0.095	0.059	3.52E-10	2.4	ZNF532	1.610169492
EFCAB14	1.54E-14	0.1511672	0.151	0.104	3.71E-10	2.4	EFCAB14	1.451923077
AP2A2	1.55E-14	0.1847342	0.17	0.121	3.75E-10	2.4	AP2A2	1.404958678
ERBB3	1.57E-14	0.1915356	0.175	0.127	3.79E-10	2.4	ERBB3	1.377952756
PTPN211	1.59E-14	0.1325241	0.09	0.055	3.83E-10	2.4	PTPN21	1.636363636
CLMN3	1.76E-14	0.1477432	0.593	0.523	4.24E-10	2.4	CLMN	1.133843212
SRPK21	1.83E-14	0.1605052	0.472	0.405	4.42E-10	2.4	SRPK2	1.165432099
MGAT4A	1.91E-14	0.1471285	0.129	0.086	4.60E-10	2.4	MGAT4A	1.5
GPATCH2	1.92E-14	0.1747808	0.14	0.096	4.62E-10	2.4	GPATCH2	1.458333333
RNF38	1.96E-14	0.1882987	0.286	0.226	4.73E-10	2.4	RNF38	1.265486726
TRAK1	2.17E-14	0.1919734	0.247	0.19	5.23E-10	2.4	TRAK1	1.3
RAP1B1	2.39E-14	0.171305	0.532	0.466	5.76E-10	2.4	RAP1B	1.141630901
AC058791.11	2.68E-14	0.1649729	0.304	0.241	6.45E-10	2.4	AC058791.	1.261410788
ZBTB103	3.09E-14	0.18391	0.312	0.249	7.44E-10	2.4	ZBTB10	1.253012048
PPP4R1	3.12E-14	0.1776215	0.286	0.226	7.52E-10	2.4	PPP4R1	1.265486726
TRAPPC101	3.38E-14	0.1862453	0.155	0.11	8.14E-10	2.4	TRAPPC10	1.409090909
ARGLU11	3.53E-14	0.1819796	0.233	0.179	8.52E-10	2.4	ARGLU1	1.301675978
MAGI13	3.60E-14	0.1402605	0.809	0.744	8.68E-10	2.4	MAGI1	1.087365591
SLC22A231	3.70E-14	0.1519203	0.303	0.241	8.91E-10	2.4	SLC22A23	1.257261411
PPP1R12A1	3.77E-14	0.2023442	0.285	0.229	9.09E-10	2.4	PPP1R12A	1.244541485
SCRN12	3.86E-14	0.1338654	0.096	0.06	9.30E-10	2.4	SCRN1	1.6
ZNF4301	4.12E-14	0.1574937	0.111	0.072	9.93E-10	2.4	ZNF430	1.541666667
STX182	4.22E-14	0.1753716	0.224	0.168	1.02E-09	2.4	STX18	1.333333333
PIP4K2C2	4.49E-14	0.1287792	0.113	0.073	1.08E-09	2.4	PIP4K2C	1.547945205
NEDD4	4.52E-14	0.2060004	0.162	0.116	1.09E-09	2.4	NEDD4	1.396551724
RARB2	4.66E-14	0.1713261	0.101	0.064	1.12E-09	2.4	RARB	1.578125
AC008937.21	5.16E-14	0.1302822	0.079	0.047	1.24E-09	2.4	AC008937.:	1.680851064
S100P1	5.38E-14	0.1596162	0.083	0.05	1.30E-09	2.4	S100P	1.66
TRAPPC92	6.05E-14	0.1980866	0.257	0.201	1.46E-09	2.4	TRAPPC9	1.278606965
LCORL	6.13E-14	0.1821856	0.189	0.139	1.48E-09	2.4	LCORL	1.35971223
PMEPA12	6.17E-14	0.1468047	0.092	0.056	1.49E-09	2.4	PMEPA1	1.642857143
L3MBTL3	6.83E-14	0.1752719	0.11	0.072	1.65E-09	2.4	L3MBTL3	1.527777778
UBE2E11	7.14E-14	0.229814	0.395	0.338	1.72E-09	2.4	UBE2E1	1.168639053
TREM1	7.17E-14	0.1074285	0.038	0.017	1.73E-09	2.4	TREM1	2.235294118
PLXND1	7.52E-14	0.1066157	0.056	0.029	1.81E-09	2.4	PLXND1	1.931034483
MAP3K41	7.58E-14	0.1587588	0.17	0.121	1.83E-09	2.4	MAP3K4	1.404958678
RBM331	8.07E-14	0.1590827	0.19	0.139	1.95E-09	2.4	RBM33	1.366906475
C5orf24	8.87E-14	0.1152748	0.081	0.048	2.14E-09	2.4	C5orf24	1.6875
UBE2Z	8.89E-14	0.1975633	0.257	0.202	2.14E-09	2.4	UBE2Z	1.272277228
UBE2D1	9.06E-14	0.1464354	0.104	0.066	2.18E-09	2.4	UBE2D1	1.575757576
CUL4A1	9.08E-14	0.201662	0.227	0.174	2.19E-09	2.4	CUL4A	1.304597701
ABCC13	9.53E-14	0.1367476	0.143	0.098	2.30E-09	2.4	ABCC1	1.459183673
CAPG	9.67E-14	0.1073279	0.049	0.025	2.33E-09	2.4	CAPG	1.96
NUTM2A-AS12	9.81E-14	0.1836229	0.354	0.295	2.37E-09	2.4	NUTM2A-A	1.2
CST33	1.10E-13	0.1065416	0.1	0.062	2.65E-09	2.4	CST3	1.612903226
KIF27	1.11E-13	0.1444348	0.103	0.066	2.68E-09	2.4	KIF27	1.560606061
RALGAPB	1.25E-13	0.1961489	0.195	0.146	3.02E-09	2.4	RALGAPB	1.335616438
YWHAH2	1.26E-13	0.1589438	0.26	0.203	3.03E-09	2.4	YWHAH	1.280788177
ARID1A	1.29E-13	0.1859006	0.288	0.234	3.11E-09	2.4	ARID1A	1.230769231
SRP143	1.36E-13	0.1406008	0.888	0.872	3.27E-09	2.4	SRP14	1.018348624
TDRD3	1.40E-13	0.1835259	0.143	0.101	3.37E-09	2.4	TDRD3	1.415841584
CHFR	1.40E-13	0.152477	0.125	0.084	3.38E-09	2.4	CHFR	1.488095238

EHD21	1.49E-13	0.1346757	0.087	0.054	3.59E-09	2.4	EHD2	1.611111111
UBE2I1	2.03E-13	0.1491569	0.165	0.118	4.90E-09	2.4	UBE2I	1.398305085
PRRC2B	2.22E-13	0.1703792	0.268	0.213	5.36E-09	2.4	PRRC2B	1.258215962
DNAJC1	2.30E-13	0.1918891	0.313	0.255	5.55E-09	2.4	DNAJC1	1.22745098
GM2A	2.32E-13	0.1311667	0.064	0.036	5.61E-09	2.4	GM2A	1.777777778
WASF21	2.38E-13	0.1587242	0.303	0.243	5.75E-09	2.4	WASF2	1.24691358
CIB11	2.39E-13	0.1341886	0.045	0.022	5.77E-09	2.4	CIB1	2.045454545
ROBO21	2.41E-13	0.1489514	0.106	0.069	5.80E-09	2.4	ROBO2	1.536231884
PLSCR22	2.69E-13	0.1329397	0.089	0.055	6.49E-09	2.4	PLSCR2	1.618181818
C2CD4A3	2.85E-13	0.1249025	0.076	0.045	6.87E-09	2.4	C2CD4A	1.688888889
LATS22	3.13E-13	0.1570995	0.149	0.105	7.54E-09	2.4	LATS2	1.419047619
KCNK12	3.32E-13	0.1964519	0.201	0.152	8.00E-09	2.4	KCNK1	1.322368421
RALGPS1	3.34E-13	0.1385046	0.091	0.057	8.05E-09	2.4	RALGPS1	1.596491228
CD1643	3.53E-13	0.1169209	0.341	0.274	8.52E-09	2.4	CD164	1.244525547
MBOAT1	3.81E-13	0.187029	0.178	0.13	9.18E-09	2.4	MBOAT1	1.369230769
HMGCR1	3.96E-13	0.1689849	0.125	0.086	9.55E-09	2.4	HMGCR	1.453488372
ASNS2	4.03E-13	0.1753643	0.12	0.081	9.73E-09	2.4	ASNS	1.481481481
DYNC1LI2	4.59E-13	0.174975	0.279	0.224	1.11E-08	2.4	DYNC1LI2	1.245535714
IL4R1	4.88E-13	0.1799473	0.182	0.136	1.18E-08	2.4	IL4R	1.338235294
GARS2	5.33E-13	0.1846586	0.224	0.173	1.29E-08	2.4	GARS	1.294797688
COG53	5.56E-13	0.1910939	0.496	0.439	1.34E-08	2.4	COG5	1.129840547
CEP1281	5.93E-13	0.1621589	0.092	0.058	1.43E-08	2.4	CEP128	1.586206897
DDAH2	6.01E-13	0.1358084	0.065	0.037	1.45E-08	2.4	DDAH2	1.756756757
GRB7	6.18E-13	0.1741582	0.166	0.12	1.49E-08	2.4	GRB7	1.383333333
VEZT1	6.25E-13	0.1838877	0.425	0.364	1.51E-08	2.4	VEZT	1.167582418
RASD1	6.32E-13	0.1423324	0.085	0.053	1.52E-08	2.4	RASD1	1.603773585
PLP22	6.41E-13	0.1193509	0.103	0.067	1.54E-08	2.4	PLP2	1.537313433
PTPRR1	6.72E-13	0.1245041	0.066	0.037	1.62E-08	2.4	PTPRR	1.783783784
PDE5A	7.23E-13	0.1884318	0.13	0.091	1.74E-08	2.4	PDE5A	1.428571429
RALA1	7.27E-13	0.1825654	0.264	0.208	1.75E-08	2.4	RALA	1.269230769
ARL33	7.55E-13	0.1724924	0.311	0.251	1.82E-08	2.4	ARL3	1.239043825
LRP10	7.58E-13	0.1544218	0.176	0.13	1.83E-08	2.4	LRP10	1.353846154
REEP53	8.35E-13	0.1376827	0.306	0.246	2.01E-08	2.4	REEP5	1.243902439
ITPR31	9.07E-13	0.1254485	0.112	0.074	2.19E-08	2.4	ITPR3	1.513513514
ATE12	9.71E-13	0.1588792	0.166	0.121	2.34E-08	2.4	ATE1	1.371900826
LIMA1	9.76E-13	0.2106851	0.45	0.393	2.35E-08	2.4	LIMA1	1.145038168
KIAA08953	1.14E-12	0.1203881	0.08	0.048	2.74E-08	2.4	KIAA0895	1.666666667
GBE14	1.15E-12	0.1305373	0.416	0.35	2.77E-08	2.4	GBE1	1.188571429
VASP2	1.19E-12	0.1188604	0.094	0.06	2.86E-08	2.4	VASP	1.566666667
NDFIP1	1.29E-12	0.1739391	0.24	0.188	3.12E-08	2.4	NDFIP1	1.276595745
EMC102	1.32E-12	0.1053373	0.089	0.056	3.19E-08	2.4	EMC10	1.589285714
ELF22	1.42E-12	0.1747016	0.439	0.382	3.42E-08	2.4	ELF2	1.14921466
FAM35A	1.58E-12	0.1477626	0.178	0.132	3.81E-08	2.4	FAM35A	1.348484848
GADD45B3	1.64E-12	0.1856862	0.148	0.104	3.95E-08	2.4	GADD45B	1.423076923
YPEL21	1.64E-12	0.1566882	0.153	0.11	3.97E-08	2.4	YPEL2	1.390909091
PSAP2	1.68E-12	0.1329709	0.162	0.117	4.04E-08	2.4	PSAP	1.384615385
SS18L1	2.00E-12	0.102264	0.057	0.032	4.82E-08	2.4	SS18L1	1.78125
LIFR2	2.34E-12	0.1625215	0.194	0.146	5.63E-08	2.4	LIFR	1.328767123
SOS12	2.36E-12	0.1800413	0.424	0.362	5.69E-08	2.4	SOS1	1.171270718
ISG20	2.45E-12	0.1172798	0.099	0.064	5.91E-08	2.4	ISG20	1.546875
SLC7A12	2.56E-12	0.1431229	0.16	0.116	6.18E-08	2.4	SLC7A1	1.379310345
TCF251	2.87E-12	0.1464774	0.168	0.124	6.91E-08	2.4	TCF25	1.35483871

FAM177A12	3.02E-12	0.1277427	0.165	0.12	7.29E-08	2.4	FAM177A1	1.375
POC1B1	3.58E-12	0.1798617	0.148	0.107	8.62E-08	2.4	POC1B	1.38317757
WNK12	3.77E-12	0.1635744	0.291	0.235	9.10E-08	2.4	WNK1	1.238297872
BCL102	3.81E-12	0.1533652	0.187	0.139	9.19E-08	2.4	BCL10	1.345323741
MXI11	4.07E-12	0.1729764	0.236	0.186	9.81E-08	2.4	MXI1	1.268817204
RAB11FIP41	4.21E-12	0.1035991	0.068	0.04	1.02E-07	2.4	RAB11FIP4	1.7
RP11-83A24.23	4.31E-12	0.1967119	0.169	0.127	1.04E-07	2.4	RP11-83A2	1.330708661
CROT	4.46E-12	0.1094673	0.07	0.042	1.08E-07	2.4	CROT	1.666666667
HACD2	4.70E-12	0.1375537	0.127	0.088	1.13E-07	2.4	HACD2	1.443181818
TTC93	4.73E-12	0.1776372	0.208	0.161	1.14E-07	2.4	TTC9	1.291925466
PI4KA	5.09E-12	0.1578724	0.159	0.116	1.23E-07	2.4	PI4KA	1.370689655
LINC005043	5.14E-12	0.1371794	0.124	0.085	1.24E-07	2.4	LINC00504	1.458823529
ABI21	5.16E-12	0.1794674	0.231	0.182	1.25E-07	2.4	ABI2	1.269230769
TRIQK1	5.48E-12	0.1021821	0.113	0.076	1.32E-07	2.4	TRIQK	1.486842105
AVL92	5.49E-12	0.1848461	0.244	0.195	1.32E-07	2.4	AVL9	1.251282051
WDR201	5.56E-12	0.1394474	0.15	0.108	1.34E-07	2.4	WDR20	1.388888889
MYH93	5.81E-12	0.1553247	0.561	0.509	1.40E-07	2.4	MYH9	1.1021611
STAU11	7.01E-12	0.1573917	0.284	0.229	1.69E-07	2.4	STAU1	1.240174672
SHOC22	7.81E-12	0.1875515	0.304	0.254	1.88E-07	2.4	SHOC2	1.196850394
GTF2H2C2	7.86E-12	0.1729399	0.106	0.072	1.90E-07	2.4	GTF2H2C	1.472222222
MYCBP21	7.99E-12	0.1547458	0.28	0.228	1.93E-07	2.4	MYCBP2	1.228070175
CA121	8.02E-12	0.1358509	0.353	0.289	1.93E-07	2.4	CA12	1.221453287
AP3S11	8.32E-12	0.1570762	0.147	0.106	2.01E-07	2.4	AP3S1	1.386792453
CORO2A	8.49E-12	0.1110488	0.09	0.058	2.05E-07	2.4	CORO2A	1.551724138
CUL32	8.54E-12	0.1321316	0.457	0.396	2.06E-07	2.4	CUL3	1.154040404
FAM83E	9.18E-12	0.1007161	0.044	0.023	2.21E-07	2.4	FAM83E	1.913043478
CPEB42	9.59E-12	0.1484303	0.311	0.254	2.31E-07	2.4	CPEB4	1.224409449
IMMP1L2	9.81E-12	0.1634002	0.178	0.134	2.37E-07	2.4	IMMP1L	1.328358209
SLC3A2	1.14E-11	0.1213707	0.09	0.058	2.74E-07	2.4	SLC3A2	1.551724138
PAK21	1.14E-11	0.156125	0.234	0.185	2.75E-07	2.4	PAK2	1.264864865
RORA1	1.16E-11	0.405714	0.608	0.569	2.81E-07	2.4	RORA	1.068541301
MYLIP2	1.22E-11	0.1191277	0.127	0.087	2.94E-07	2.4	MYLIP	1.459770115
VWDE1	1.22E-11	0.1037337	0.051	0.028	2.95E-07	2.4	VWDE	1.821428571
SNAP29	1.27E-11	0.11741	0.069	0.041	3.05E-07	2.4	SNAP29	1.682926829
ODF2L1	1.48E-11	0.2020276	0.197	0.153	3.56E-07	2.4	ODF2L	1.287581699
RTN32	1.69E-11	0.1763778	0.255	0.206	4.08E-07	2.4	RTN3	1.237864078
CERS21	1.76E-11	0.1105089	0.084	0.054	4.24E-07	2.4	CERS2	1.555555556
PTMA	1.95E-11	0.2596942	0.646	0.618	4.69E-07	2.4	PTMA	1.045307443
FBXO211	1.95E-11	0.1571264	0.141	0.102	4.71E-07	2.4	FBXO21	1.382352941
JAK12	2.03E-11	0.1294622	0.459	0.4	4.90E-07	2.4	JAK1	1.1475
DHX153	2.38E-11	0.1509194	0.307	0.255	5.75E-07	2.4	DHX15	1.203921569
PGRMC13	2.60E-11	0.1287774	0.117	0.081	6.26E-07	2.4	PGRMC1	1.444444444
HERPUD2	2.61E-11	0.125244	0.094	0.062	6.30E-07	2.4	HERPUD2	1.516129032
KCNE41	2.74E-11	0.1080804	0.051	0.028	6.61E-07	2.4	KCNE4	1.821428571
RAP1GDS12	2.77E-11	0.1658564	0.163	0.121	6.67E-07	2.4	RAP1GDS1	1.347107438
CLASP21	2.90E-11	0.1893399	0.319	0.267	7.00E-07	2.4	CLASP2	1.194756554
LINC002903	3.06E-11	0.1037831	0.05	0.027	7.37E-07	2.4	LINC00290	1.851851852
BNIP2	3.11E-11	0.1453087	0.165	0.123	7.51E-07	2.4	BNIP2	1.341463415
FGFR12	3.21E-11	0.1513539	0.239	0.187	7.74E-07	2.4	FGFR1	1.278074866
GRHL11	3.24E-11	0.2341652	0.312	0.261	7.80E-07	2.4	GRHL1	1.195402299
CAPN22	3.83E-11	0.155641	0.34	0.286	9.23E-07	2.4	CAPN2	1.188811189
APPL2	3.84E-11	0.1377039	0.144	0.105	9.25E-07	2.4	APPL2	1.371428571

ZNF2811	3.91E-11	0.1592272	0.152	0.112	9.44E-07	2.4	ZNF281	1.357142857
PDPK1	3.97E-11	0.1135026	0.091	0.059	9.56E-07	2.4	PDPK1	1.542372881
KRAS1	5.05E-11	0.15674	0.192	0.147	1.22E-06	2.4	KRAS	1.306122449
ZFC3H12	5.49E-11	0.1357338	0.308	0.252	1.32E-06	2.4	ZFC3H1	1.222222222
MTUS12	5.54E-11	0.1508754	0.425	0.371	1.34E-06	2.4	MTUS1	1.145552561
CEBPG	5.55E-11	0.1002056	0.071	0.044	1.34E-06	2.4	CEBPG	1.613636364
ASAH13	5.97E-11	0.1101226	0.136	0.097	1.44E-06	2.4	ASAH1	1.402061856
GEM	5.99E-11	0.1241449	0.075	0.047	1.44E-06	2.4	GEM	1.595744681
MALRD13	6.51E-11	0.1378901	0.069	0.042	1.57E-06	2.4	MALRD1	1.642857143
AC010149.42	6.65E-11	0.118073	0.09	0.059	1.60E-06	2.4	AC010149.4	1.525423729
DNAH53	6.76E-11	0.1101673	0.097	0.064	1.63E-06	2.4	DNAH5	1.515625
SH3BGRL31	6.86E-11	0.2060368	0.083	0.053	1.65E-06	2.4	SH3BGRL3	1.566037736
C2orf69	6.92E-11	0.1013058	0.077	0.048	1.67E-06	2.4	C2orf69	1.604166667
CDC424	7.41E-11	0.1668262	0.741	0.713	1.79E-06	2.4	CDC42	1.039270687
STC2	7.44E-11	0.2099372	0.129	0.092	1.79E-06	2.4	STC2	1.402173913
CTTN1	7.57E-11	0.1837352	0.226	0.181	1.83E-06	2.4	CTTN	1.248618785
GNG12-AS1	7.94E-11	0.1134013	0.11	0.075	1.91E-06	2.4	GNG12-AS1	1.466666667
CCDC64	8.09E-11	0.1154729	0.081	0.052	1.95E-06	2.4	CCDC64	1.557692308
BLOC1S5-TXNDC5	8.80E-11	0.1142676	0.032	0.015	2.12E-06	2.4	BLOC1S5-T	2.133333333
ATP6AP11	8.97E-11	0.1021251	0.08	0.051	2.16E-06	2.4	ATP6AP1	1.568627451
NFE2L1	9.10E-11	0.1295823	0.123	0.087	2.19E-06	2.4	NFE2L1	1.413793103
BOD1L11	9.11E-11	0.1591372	0.179	0.137	2.20E-06	2.4	BOD1L1	1.306569343
CDC42BPB1	9.41E-11	0.1287079	0.101	0.069	2.27E-06	2.4	CDC42BPB	1.463768116
TPRG12	9.64E-11	0.1642926	0.153	0.113	2.33E-06	2.4	TPRG1	1.353982301
SEZ6L2	9.86E-11	0.1040279	0.083	0.053	2.38E-06	2.4	SEZ6L2	1.566037736
STAM2	1.08E-10	0.1380078	0.119	0.084	2.60E-06	2.4	STAM2	1.416666667
PPP2R5A2	1.15E-10	0.173781	0.198	0.155	2.78E-06	2.4	PPP2R5A	1.277419355
STXBP41	1.23E-10	0.1302086	0.068	0.042	2.97E-06	2.4	STXBP4	1.619047619
VDAC2	1.43E-10	0.1642188	0.167	0.126	3.44E-06	2.4	VDAC2	1.325396825
RSRC11	1.45E-10	0.1801476	0.33	0.281	3.50E-06	2.4	RSRC1	1.174377224
CD2AP4	1.47E-10	0.1148244	0.478	0.421	3.55E-06	2.4	CD2AP	1.135391924
APMAP	1.50E-10	0.1098292	0.079	0.05	3.61E-06	2.4	APMAP	1.58
ATP9A	1.61E-10	0.1575284	0.14	0.102	3.87E-06	2.4	ATP9A	1.37254902
APLF1	1.62E-10	0.1540587	0.139	0.102	3.91E-06	2.4	APLF	1.362745098
BLCAP	1.66E-10	0.1167496	0.076	0.048	4.01E-06	2.4	BLCAP	1.583333333
MOB1B1	1.67E-10	0.1323663	0.093	0.062	4.03E-06	2.4	MOB1B	1.5
DHRS71	1.70E-10	0.1329859	0.123	0.087	4.11E-06	2.4	DHRS7	1.413793103
AGO22	1.76E-10	0.1448866	0.255	0.205	4.23E-06	2.4	AGO2	1.243902439
RALBP1	1.85E-10	0.1371257	0.145	0.107	4.47E-06	2.4	RALBP1	1.355140187
STX33	1.91E-10	0.1438588	0.177	0.135	4.61E-06	2.4	STX3	1.311111111
DDX171	1.92E-10	0.1461216	0.471	0.42	4.62E-06	2.4	DDX17	1.121428571
CDK81	1.93E-10	0.1671849	0.286	0.235	4.66E-06	2.4	CDK8	1.217021277
KRT171	2.00E-10	0.1456983	0.044	0.024	4.81E-06	2.4	KRT17	1.833333333
CBFA2T2	2.09E-10	0.1816306	0.206	0.163	5.04E-06	2.4	CBFA2T2	1.263803681
KLF101	2.49E-10	0.1375244	0.181	0.138	6.00E-06	2.4	KLF10	1.311594203
FAM83F	2.66E-10	0.1056581	0.062	0.037	6.40E-06	2.4	FAM83F	1.675675676
SRSF4	2.68E-10	0.1707529	0.338	0.288	6.46E-06	2.4	SRSF4	1.173611111
AMOT	2.77E-10	0.1114791	0.113	0.079	6.67E-06	2.4	AMOT	1.430379747
TET3	2.96E-10	0.1300955	0.165	0.124	7.14E-06	2.4	TET3	1.330645161
TOP13	3.00E-10	0.1045939	0.498	0.439	7.24E-06	2.4	TOP1	1.134396355
PCM12	3.10E-10	0.1773383	0.3	0.253	7.48E-06	2.4	PCM1	1.185770751
TBC1D52	3.17E-10	0.1736811	0.397	0.347	7.64E-06	2.4	TBC1D5	1.144092219

SLC44A1	3.19E-10	0.1817098	0.182	0.14	7.69E-06	2.4	SLC44A1	1.3
ITGB12	3.32E-10	0.1180175	0.384	0.33	8.01E-06	2.4	ITGB1	1.163636364
NDUFAF61	3.46E-10	0.136376	0.135	0.098	8.34E-06	2.4	NDUFAF6	1.37755102
MAP7D12	3.52E-10	0.1369904	0.191	0.148	8.49E-06	2.4	MAP7D1	1.290540541
P2RX4	3.62E-10	0.1216139	0.109	0.076	8.73E-06	2.4	P2RX4	1.434210526
ARHGAP35	3.91E-10	0.1440897	0.118	0.084	9.44E-06	2.4	ARHGAP35	1.404761905
AC018816.33	4.27E-10	0.1145366	0.132	0.094	1.03E-05	2.4	AC018816.	1.404255319
SLC9C12	4.28E-10	0.1131022	0.083	0.054	1.03E-05	2.4	SLC9C1	1.537037037
ZNF2542	4.41E-10	0.1756248	0.197	0.157	1.06E-05	2.4	ZNF254	1.25477707
ZNF6381	4.88E-10	0.1637131	0.484	0.438	1.18E-05	2.4	ZNF638	1.105022831
PVRL41	4.99E-10	0.1555044	0.231	0.187	1.20E-05	2.4	PVRL4	1.235294118
PBRM12	5.01E-10	0.1756634	0.265	0.22	1.21E-05	2.4	PBRM1	1.204545455
PUM23	5.06E-10	0.1186644	0.463	0.407	1.22E-05	2.4	PUM2	1.137592138
MT-ND62	5.45E-10	0.1957281	0.214	0.174	1.31E-05	2.4	MT-ND6	1.229885057
CA24	5.49E-10	0.124966	0.123	0.087	1.32E-05	2.4	CA2	1.413793103
PAIP21	5.79E-10	0.1635431	0.197	0.155	1.40E-05	2.4	PAIP2	1.270967742
SUMO21	6.32E-10	0.162289	0.14	0.105	1.52E-05	2.4	SUMO2	1.333333333
SERINC51	6.37E-10	0.1513949	0.285	0.239	1.54E-05	2.4	SERINC5	1.192468619
CREM1	7.00E-10	0.1278836	0.132	0.096	1.69E-05	2.4	CREM	1.375
PLEKHF21	7.18E-10	0.1289	0.134	0.097	1.73E-05	2.4	PLEKHF2	1.381443299
IQGAP12	7.44E-10	0.1531033	0.48	0.432	1.79E-05	2.4	IQGAP1	1.111111111
AGPAT3	7.64E-10	0.1519929	0.119	0.085	1.84E-05	2.4	AGPAT3	1.4
EIF2S22	7.66E-10	0.2087757	0.298	0.255	1.85E-05	2.4	EIF2S2	1.168627451
RSF11	7.75E-10	0.1492799	0.415	0.369	1.87E-05	2.4	RSF1	1.124661247
DYNC1H11	8.18E-10	0.1789689	0.222	0.179	1.97E-05	2.4	DYNC1H1	1.240223464
WWC32	8.21E-10	0.1078388	0.107	0.075	1.98E-05	2.4	WWC3	1.426666667
SERPINB15	9.33E-10	0.1096417	0.139	0.102	2.25E-05	2.4	SERPINB1	1.362745098
IRF62	9.42E-10	0.1362053	0.274	0.227	2.27E-05	2.4	IRF6	1.207048458
TMEM1563	9.95E-10	0.1724952	0.074	0.047	2.40E-05	2.4	TMEM156	1.574468085
BARD11	1.08E-09	0.1425986	0.121	0.088	2.60E-05	2.4	BARD1	1.375
HIST1H2BB2	1.10E-09	0.1065202	0.208	0.164	2.65E-05	2.4	HIST1H2BB	1.268292683
MAFB2	1.13E-09	0.1116513	0.072	0.046	2.73E-05	2.4	MAFB	1.565217391
HAVCR2	1.14E-09	0.1051273	0.058	0.035	2.76E-05	2.4	HAVCR2	1.657142857
RP11-123O10.42	1.25E-09	0.1825017	0.251	0.207	3.00E-05	2.4	RP11-123O	1.212560386
RP11-701H24.9	1.26E-09	0.1173624	0.097	0.067	3.04E-05	2.4	RP11-701H	1.447761194
MYO5A2	1.29E-09	0.1301064	0.139	0.103	3.10E-05	2.4	MYO5A	1.349514563
GAB22	1.31E-09	0.1534672	0.238	0.194	3.16E-05	2.4	GAB2	1.226804124
PPP3CB1	1.36E-09	0.1305771	0.134	0.099	3.29E-05	2.4	PPP3CB	1.353535354
CAPNS1	1.57E-09	0.1301827	0.109	0.077	3.78E-05	2.4	CAPNS1	1.415584416
SREBF22	1.60E-09	0.2090152	0.414	0.373	3.87E-05	2.4	SREBF2	1.109919571
SPATA6L1	1.75E-09	0.124526	0.087	0.059	4.22E-05	2.4	SPATA6L	1.474576271
CELSR1	1.80E-09	0.1170954	0.077	0.05	4.34E-05	2.4	CELSR1	1.54
ABCD33	1.81E-09	0.1032734	0.221	0.174	4.37E-05	2.4	ABCD3	1.270114943
MCUR11	1.85E-09	0.1126787	0.086	0.058	4.46E-05	2.4	MCUR1	1.482758621
HSBP12	1.89E-09	0.1628888	0.591	0.55	4.56E-05	2.4	HSBP1	1.074545455
SLC39A1	1.90E-09	0.106607	0.136	0.1	4.59E-05	2.4	SLC39A1	1.36
NRDC	1.91E-09	0.1595071	0.253	0.209	4.61E-05	2.4	NRDC	1.210526316
TAOK12	1.99E-09	0.1562615	0.289	0.244	4.80E-05	2.4	TAOK1	1.18442623
PLEKHB23	2.04E-09	0.1035504	0.203	0.158	4.92E-05	2.4	PLEKHB2	1.284810127
USP9X3	2.12E-09	0.114242	0.453	0.399	5.12E-05	2.4	USP9X	1.135338346
ARPC51	2.51E-09	0.1488082	0.141	0.106	6.05E-05	2.4	ARPC5	1.330188679
ZNF487	2.53E-09	0.1051548	0.08	0.053	6.10E-05	2.4	ZNF487	1.509433962

C6orf1061	2.57E-09	0.1524404	0.19	0.15	6.19E-05	2.4	C6orf106	1.266666667
ZNF800	2.58E-09	0.1318949	0.139	0.104	6.22E-05	2.4	ZNF800	1.336538462
NFRKB1	2.65E-09	0.1182888	0.111	0.079	6.39E-05	2.4	NFRKB	1.405063291
ZBTB183	3.00E-09	0.1001007	0.1	0.069	7.25E-05	2.4	ZBTB18	1.449275362
ZNF516	3.03E-09	0.1251462	0.12	0.087	7.32E-05	2.4	ZNF516	1.379310345
SGPP11	3.29E-09	0.1359523	0.109	0.078	7.94E-05	2.4	SGPP1	1.397435897
HNRNPU-AS12	3.30E-09	0.127143	0.123	0.09	7.95E-05	2.4	HNRNPU-A	1.366666667
SLTM	3.52E-09	0.1441221	0.335	0.29	8.50E-05	2.4	SLTM	1.155172414
KIF16B2	3.69E-09	0.1163272	0.226	0.181	8.90E-05	2.4	KIF16B	1.248618785
SBNO11	3.69E-09	0.1293731	0.187	0.147	8.90E-05	2.4	SBNO1	1.272108844
ATXN21	3.99E-09	0.1452306	0.378	0.329	9.61E-05	2.4	ATXN2	1.14893617
ZNF4313	4.18E-09	0.1605227	0.161	0.125	0.000100909	2.4	ZNF431	1.288
DDX54	4.28E-09	0.1439164	0.537	0.493	0.000103165	2.4	DDX5	1.089249493
ZNF24	4.58E-09	0.1525784	0.303	0.259	0.000110437	2.4	ZNF24	1.16988417
DNAH111	4.65E-09	0.1216875	0.072	0.047	0.000112068	2.4	DNAH11	1.531914894
DNM1L2	4.73E-09	0.1233493	0.151	0.115	0.000114129	2.4	DNM1L	1.313043478
KLF71	5.12E-09	0.1280286	0.159	0.122	0.000123564	2.4	KLF7	1.303278689
IST11	5.39E-09	0.1250725	0.373	0.325	0.000130034	2.4	IST1	1.147692308
SH3RF11	5.49E-09	0.1821479	0.262	0.219	0.000132362	2.4	SH3RF1	1.196347032
WDR442	5.59E-09	0.1224478	0.201	0.159	0.000134756	2.4	WDR44	1.264150943
EPC21	5.76E-09	0.1154176	0.097	0.068	0.00013885	2.4	EPC2	1.426470588
CSGALNACT21	6.01E-09	0.1056055	0.16	0.122	0.00014483	2.4	CSGALNAC	1.31147541
MROH1	6.23E-09	0.1138086	0.091	0.063	0.000150167	2.4	MROH1	1.444444444
CUEDC11	6.30E-09	0.1397453	0.092	0.064	0.000151892	2.4	CUEDC1	1.4375
RAB6A1	6.91E-09	0.154302	0.344	0.301	0.000166614	2.4	RAB6A	1.142857143
ACOT91	7.00E-09	0.1100172	0.122	0.089	0.000168873	2.4	ACOT9	1.370786517
MSRA1	7.21E-09	0.1576845	0.111	0.08	0.000173757	2.4	MSRA	1.3875
SDCBP2-AS12	7.33E-09	0.1381091	0.09	0.062	0.00017679	2.4	SDCBP2-AS	1.451612903
EFR3A1	7.34E-09	0.1126035	0.249	0.204	0.000177044	2.4	EFR3A	1.220588235
KLHL18	7.97E-09	0.1147906	0.107	0.076	0.000192136	2.4	KLHL18	1.407894737
ATL31	8.49E-09	0.1326458	0.156	0.121	0.000204602	2.4	ATL3	1.289256198
RREB12	8.50E-09	0.210043	0.3	0.26	0.000205016	2.4	RREB1	1.153846154
ABHD15-AS1	8.88E-09	0.1018373	0.059	0.037	0.00021418	2.4	ABHD15-AS	1.594594595
ANXA111	9.18E-09	0.1250618	0.283	0.237	0.000221369	2.4	ANXA11	1.194092827
DDX581	9.67E-09	0.1167838	0.124	0.092	0.000233081	2.4	DDX58	1.347826087
PAFAH1B23	9.88E-09	0.1250494	0.306	0.259	0.000238137	2.4	PAFAH1B2	1.181467181
NDUFA61	1.01E-08	0.1297978	0.111	0.081	0.000242881	2.4	NDUFA6	1.37037037
RYR21	1.02E-08	0.1292191	0.151	0.114	0.000245784	2.4	RYR2	1.324561404
PRMT22	1.05E-08	0.1378549	0.163	0.127	0.000253581	2.4	PRMT2	1.283464567
HMBOX11	1.06E-08	0.1705395	0.27	0.228	0.00025562	2.4	HMBOX1	1.184210526
RP1-313I6.122	1.07E-08	0.1097519	0.126	0.093	0.000258484	2.4	RP1-313I6.	1.35483871
UBR41	1.09E-08	0.1040431	0.273	0.225	0.000263485	2.4	UBR4	1.213333333
KIAA18411	1.11E-08	0.1049961	0.066	0.043	0.000268011	2.4	KIAA1841	1.534883721
R3HCC1L2	1.14E-08	0.136283	0.162	0.126	0.000274528	2.4	R3HCC1L	1.285714286
SOX91	1.29E-08	0.1746808	0.226	0.187	0.00031058	2.4	SOX9	1.20855615
NCOR2	1.29E-08	0.114387	0.098	0.069	0.000311579	2.4	NCOR2	1.420289855
RP11-347C12.1	1.31E-08	0.1078204	0.066	0.043	0.000315419	2.4	RP11-347C	1.534883721
SRPX2	1.32E-08	0.1278654	0.099	0.07	0.000317222	2.4	SRPX2	1.414285714
SIAH1	1.47E-08	0.1152512	0.146	0.111	0.000354506	2.4	SIAH1	1.315315315
RMND5A2	1.49E-08	0.1105045	0.134	0.101	0.000358149	2.4	RMND5A	1.326732673
POLR2A1	1.52E-08	0.102773	0.203	0.162	0.00036743	2.4	POLR2A	1.25308642
PLAT3	1.54E-08	0.1142504	0.116	0.084	0.000371867	2.4	PLAT	1.380952381

RP11-624L4.11	1.59E-08	0.1448137	0.088	0.061	0.000384281	2.4	RP11-624L4	1.442622951
UBE2Q2	1.77E-08	0.1274974	0.112	0.082	0.000426411	2.4	UBE2Q2	1.365853659
ANKRD111	1.81E-08	0.1038142	0.364	0.315	0.000436185	2.4	ANKRD11	1.155555556
ZNF611	1.82E-08	0.1325763	0.084	0.058	0.000438313	2.4	ZNF611	1.448275862
TROVE2	1.94E-08	0.1191257	0.169	0.132	0.000467145	2.4	TROVE2	1.28030303
SAP1301	1.95E-08	0.1080472	0.114	0.083	0.000469735	2.4	SAP130	1.373493976
BRD22	1.99E-08	0.1447489	0.307	0.265	0.000479661	2.4	BRD2	1.158490566
RP11-238K6.12	2.01E-08	0.1111588	0.155	0.118	0.00048568	2.4	RP11-238K	1.313559322
CAP21	2.10E-08	0.1378579	0.153	0.119	0.000505359	2.4	CAP2	1.285714286
SPTBN1	2.13E-08	0.1579978	0.255	0.215	0.000513424	2.4	SPTBN1	1.186046512
RAB7A2	2.16E-08	0.1100429	0.495	0.446	0.000520088	2.4	RAB7A	1.109865471
USP473	2.17E-08	0.1010826	0.465	0.416	0.00052348	2.4	USP47	1.117788462
KMT2E3	2.18E-08	0.1644069	0.501	0.461	0.000524964	2.4	KMT2E	1.086767896
PKP21	2.26E-08	0.1090881	0.123	0.091	0.000544869	2.4	PKP2	1.351648352
SDF2	2.33E-08	0.1098714	0.151	0.117	0.00056091	2.4	SDF2	1.290598291
MAP3K91	2.36E-08	0.1597948	0.196	0.16	0.000569813	2.4	MAP3K9	1.225
WDFY31	2.42E-08	0.1514793	0.29	0.25	0.000583537	2.4	WDFY3	1.16
PPP2R3C	2.44E-08	0.1317775	0.137	0.105	0.000589516	2.4	PPP2R3C	1.304761905
SERINC2	2.93E-08	0.1155853	0.129	0.097	0.000707668	2.4	SERINC2	1.329896907
CCDC1261	3.02E-08	0.1179267	0.099	0.071	0.000729054	2.4	CCDC126	1.394366197
CHMP2A1	3.26E-08	0.111735	0.119	0.088	0.000786461	2.4	CHMP2A	1.352272727
VTCN12	3.30E-08	0.1141387	0.139	0.105	0.00079552	2.4	VTCN1	1.323809524
CD61	3.32E-08	0.1505829	0.019	0.008	0.000801063	2.4	CD6	2.375
MSMO13	3.51E-08	0.2797199	0.366	0.33	0.000847462	2.4	MSMO1	1.109090909
CLOCK1	3.71E-08	0.1289337	0.245	0.203	0.000895729	2.4	CLOCK	1.206896552
DENND1B1	3.76E-08	0.1436222	0.179	0.143	0.0009068	2.4	DENND1B	1.251748252
TAF8	3.80E-08	0.105316	0.09	0.064	0.000915212	2.4	TAF8	1.40625
BAZ1B1	3.80E-08	0.1273809	0.206	0.168	0.000915726	2.4	BAZ1B	1.226190476
MOB1A1	3.93E-08	0.1290533	0.136	0.104	0.000947964	2.4	MOB1A	1.307692308
SPDYA2	4.36E-08	0.1136437	0.133	0.1	0.001050926	2.4	SPDYA	1.33
SLC6A161	4.36E-08	0.1020676	0.067	0.044	0.001052065	2.4	SLC6A16	1.522727273
MDGA23	4.38E-08	0.100686	0.095	0.067	0.001055208	2.4	MDGA2	1.417910448
ZMYM21	4.60E-08	0.1515589	0.4	0.359	0.001108595	2.4	ZMYM2	1.114206128
TAF3	4.64E-08	0.1122637	0.115	0.085	0.001119918	2.4	TAF3	1.352941176
GNG52	4.66E-08	0.1270289	0.149	0.115	0.001123202	2.4	GNG5	1.295652174
TLE11	4.68E-08	0.1673382	0.253	0.214	0.001127989	2.4	TLE1	1.182242991
REV3L	5.04E-08	0.1562135	0.318	0.275	0.001214493	2.4	REV3L	1.156363636
TBC1D121	5.21E-08	0.1076973	0.105	0.076	0.00125569	2.4	TBC1D12	1.381578947
SLC9A11	5.35E-08	0.1147031	0.153	0.118	0.001289053	2.4	SLC9A1	1.296610169
MTMR33	5.42E-08	0.125054	0.236	0.195	0.00130596	2.4	MTMR3	1.21025641
SQRDL1	6.01E-08	0.1291848	0.221	0.183	0.00144902	2.4	SQRDL	1.207650273
RCAN34	6.48E-08	0.1480255	0.225	0.185	0.001561518	2.4	RCAN3	1.216216216
CLDND12	6.49E-08	0.1198365	0.237	0.197	0.001565117	2.4	CLDND1	1.203045685
DNM21	6.62E-08	0.1004945	0.122	0.092	0.001597086	2.4	DNM2	1.326086957
BRD41	6.91E-08	0.1169838	0.239	0.198	0.001667087	2.4	BRD4	1.207070707
USP242	7.59E-08	0.1386807	0.203	0.166	0.00182945	2.4	USP24	1.222891566
RAB303	8.34E-08	0.1584348	0.153	0.118	0.002009924	2.4	RAB30	1.296610169
PRKAR2A2	8.76E-08	0.1490796	0.2	0.162	0.002111697	2.4	PRKAR2A	1.234567901
PHF112	8.78E-08	0.1345577	0.11	0.082	0.002116127	2.4	PHF11	1.341463415
DYNC1LI1	9.39E-08	0.1022966	0.098	0.071	0.002265075	2.4	DYNC1LI1	1.38028169
FOXK11	9.52E-08	0.1217135	0.217	0.179	0.002295294	2.4	FOXK1	1.212290503
DDX6	1.01E-07	0.128975	0.303	0.261	0.002437865	2.4	DDX6	1.16091954

ARNTL21	1.04E-07	0.13463	0.176	0.141	0.002510584	2.4	ARNTL2	1.24822695
RHOC	1.04E-07	0.1275305	0.089	0.063	0.002512433	2.4	RHOC	1.412698413
PRELID23	1.10E-07	0.1584523	0.094	0.068	0.00265788	2.4	PRELID2	1.382352941
ACAP21	1.12E-07	0.1435288	0.217	0.18	0.002707364	2.4	ACAP2	1.205555556
CYFIP11	1.25E-07	0.1091956	0.169	0.134	0.003006496	2.4	CYFIP1	1.26119403
DSP2	1.27E-07	0.1788504	0.388	0.351	0.003059162	2.4	DSP	1.105413105
ATP6V0E12	1.29E-07	0.1072782	0.726	0.689	0.003120634	2.4	ATP6V0E1	1.053701016
OSBPL1A3	1.33E-07	0.1338488	0.205	0.168	0.003200965	2.4	OSBPL1A	1.220238095
ARPC1A	1.34E-07	0.1041837	0.089	0.064	0.003240463	2.4	ARPC1A	1.390625
GLRX32	1.35E-07	0.1261319	0.152	0.119	0.003260949	2.4	GLRX3	1.277310924
MIPEP2	1.39E-07	0.1261881	0.077	0.054	0.003345854	2.4	MIPEP	1.425925926
NET12	1.47E-07	0.1319577	0.225	0.187	0.003533602	2.4	NET1	1.203208556
GNA132	1.47E-07	0.1019539	0.244	0.204	0.003550755	2.4	GNA13	1.196078431
NCKAP12	1.59E-07	0.1144051	0.471	0.429	0.003826107	2.4	NCKAP1	1.097902098
STK17B2	1.60E-07	0.1088406	0.192	0.155	0.003859475	2.4	STK17B	1.238709677
PAPD41	1.72E-07	0.1363095	0.351	0.308	0.004154169	2.4	PAPD4	1.13961039
HN11	1.83E-07	0.1151126	0.085	0.061	0.00441192	2.4	HN1	1.393442623
PDS5A1	1.89E-07	0.1891191	0.391	0.359	0.004567456	2.4	PDS5A	1.08913649
RP11-39M21.11	1.98E-07	0.1481646	0.082	0.058	0.004778525	2.4	RP11-39M2	1.413793103
IGF2R1	2.08E-07	0.1388272	0.223	0.186	0.005024952	2.4	IGF2R	1.198924731
UBE2E32	2.11E-07	0.1650751	0.202	0.167	0.005099868	2.4	UBE2E3	1.209580838
KIAA15512	2.26E-07	0.1370665	0.153	0.121	0.005459475	2.4	KIAA1551	1.26446281
TAB3	2.27E-07	0.1214914	0.116	0.088	0.005478347	2.4	TAB3	1.318181818
ESR22	2.51E-07	0.1322249	0.133	0.103	0.00605045	2.4	ESR2	1.291262136
CXCL6	2.56E-07	0.2297792	0.09	0.065	0.006168244	2.4	CXCL6	1.384615385
IKBKB1	2.61E-07	0.1050455	0.198	0.161	0.006292529	2.4	IKBKB	1.229813665
DSC22	2.66E-07	0.1250605	0.243	0.204	0.00642574	2.4	DSC2	1.191176471
DENND6A	2.75E-07	0.1203525	0.175	0.141	0.006621539	2.4	DENND6A	1.241134752
GCLC1	2.75E-07	0.11427	0.094	0.068	0.006631081	2.4	GCLC	1.382352941
CDKL51	2.77E-07	0.1183561	0.115	0.087	0.00667285	2.4	CDKL5	1.32183908
TULP31	2.86E-07	0.1499821	0.114	0.086	0.006900047	2.4	TULP3	1.325581395
CREB12	2.91E-07	0.1443955	0.221	0.186	0.007018956	2.4	CREB1	1.188172043
ZNF440	3.18E-07	0.1027165	0.068	0.047	0.007665389	2.4	ZNF440	1.446808511
STRN31	3.25E-07	0.1328287	0.353	0.314	0.007836902	2.4	STRN3	1.124203822
NKTR	3.30E-07	0.147385	0.354	0.317	0.007952162	2.4	NKTR	1.116719243
THOC21	3.77E-07	0.1075434	0.455	0.409	0.009088538	2.4	THOC2	1.112469438
LAMC22	3.89E-07	0.1216803	0.3	0.259	0.009371216	2.4	LAMC2	1.158301158
SERPINB61	3.98E-07	0.103379	0.076	0.053	0.009600278	2.4	SERPINB6	1.433962264
EP4001	4.00E-07	0.1164113	0.12	0.092	0.009649069	2.4	EP400	1.304347826
FDPS2	4.02E-07	0.2741613	0.487	0.45	0.0096902	2.4	FDPS	1.082222222
ABHD31	4.12E-07	0.1350105	0.206	0.171	0.00994644	2.4	ABHD3	1.204678363
PLD11	4.41E-07	0.1338519	0.157	0.126	0.01062374	2.4	PLD1	1.246031746
BTBD31	4.41E-07	0.1356185	0.143	0.114	0.01063615	2.4	BTBD3	1.254385965
KIAA0040	4.44E-07	0.1097822	0.105	0.078	0.01070932	2.4	KIAA0040	1.346153846
GIPC1	4.61E-07	0.1215285	0.16	0.129	0.0111249	2.4	GIPC1	1.240310078
ILKAP	4.63E-07	0.10116	0.089	0.065	0.01116683	2.4	ILKAP	1.369230769
HBP12	4.74E-07	0.1243351	0.297	0.258	0.01142149	2.4	HBP1	1.151162791
DDX10	4.74E-07	0.1445451	0.107	0.081	0.01144107	2.4	DDX10	1.320987654
ABLIM12	4.76E-07	0.1270912	0.305	0.265	0.01148845	2.4	ABLIM1	1.150943396
SESN21	5.28E-07	0.1390093	0.139	0.109	0.01271978	2.4	SESN2	1.275229358
CMC2	5.60E-07	0.108638	0.097	0.072	0.01350164	2.4	CMC2	1.347222222
COL4A3BP	5.70E-07	0.1252459	0.166	0.134	0.01375536	2.4	COL4A3BP	1.23880597

VPS501	5.85E-07	0.123566	0.111	0.085	0.01409522	2.4	VPS50	1.305882353
IRS22	6.00E-07	0.1984619	0.351	0.318	0.01446373	2.4	IRS2	1.103773585
AHR1	6.37E-07	0.152724	0.235	0.201	0.01536744	2.4	AHR	1.169154229
GNS3	6.84E-07	0.1046304	0.117	0.089	0.01650467	2.4	GNS	1.314606742
NAPG2	7.02E-07	0.1366653	0.21	0.177	0.01692466	2.4	NAPG	1.186440678
C6orf621	7.07E-07	0.1045146	0.241	0.205	0.01705322	2.4	C6orf62	1.175609756
SPSB1	7.53E-07	0.109421	0.109	0.083	0.01815563	2.4	SPSB1	1.313253012
PIP5K1B	7.57E-07	0.1107888	0.154	0.122	0.01825958	2.4	PIP5K1B	1.262295082
DHCR242	7.66E-07	0.1397277	0.218	0.184	0.01847612	2.4	DHCR24	1.184782609
SLC35F51	7.76E-07	0.1170682	0.145	0.115	0.01871989	2.4	SLC35F5	1.260869565
LRCH31	7.81E-07	0.1232018	0.379	0.343	0.01884062	2.4	LRCH3	1.104956268
EPS15L1	7.91E-07	0.1116943	0.105	0.08	0.01907462	2.4	EPS15L1	1.3125
SIPA1L31	8.07E-07	0.1774815	0.245	0.21	0.01946023	2.4	SIPA1L3	1.166666667
KPNA4	9.44E-07	0.1150516	0.198	0.165	0.02276697	2.4	KPNA4	1.2
MTX21	9.57E-07	0.1051325	0.108	0.082	0.0230711	2.4	MTX2	1.317073171
PSPC11	9.61E-07	0.1455604	0.221	0.188	0.0231715	2.4	PSPC1	1.175531915
GNAI31	9.69E-07	0.1020355	0.219	0.182	0.02336491	2.4	GNAI3	1.203296703
GPCPD1	9.88E-07	0.103547	0.181	0.147	0.02381645	2.4	GPCPD1	1.231292517
MARS1	1.02E-06	0.1262421	0.175	0.143	0.02453961	2.4	MARS	1.223776224
MED151	1.02E-06	0.1568895	0.203	0.168	0.02467267	2.4	MED15	1.208333333
VEGFA1	1.10E-06	0.1737663	0.261	0.226	0.02663768	2.4	VEGFA	1.154867257
PPM1K3	1.11E-06	0.1359779	0.179	0.147	0.0266766	2.4	PPM1K	1.217687075
CREBRF	1.15E-06	0.1328038	0.298	0.261	0.02766443	2.4	CREBRF	1.141762452
RABGAP1L2	1.18E-06	0.167471	0.259	0.225	0.02845546	2.4	RABGAP1L	1.151111111
KRT182	1.19E-06	0.2320414	0.134	0.106	0.02864498	2.4	KRT18	1.264150943
CITED21	1.23E-06	0.1021047	0.085	0.062	0.02974966	2.4	CITED2	1.370967742
TLDC12	1.31E-06	0.1290201	0.119	0.093	0.03159053	2.4	TLDC1	1.279569892
MTIF32	1.48E-06	0.1411078	0.303	0.268	0.03568191	2.4	MTIF3	1.130597015
LURAP1L-AS13	1.53E-06	0.1403678	0.114	0.088	0.03685437	2.4	LURAP1L-A	1.295454545
IL1RAP	1.56E-06	0.1035909	0.072	0.051	0.0376889	2.4	IL1RAP	1.411764706
POU2F11	1.89E-06	0.1164864	0.195	0.162	0.04550727	2.4	POU2F1	1.203703704
MYO9A1	1.91E-06	0.1176866	0.351	0.314	0.04617451	2.4	MYO9A	1.117834395
NDUFS1	1.97E-06	0.1332914	0.175	0.145	0.04748737	2.4	NDUFS1	1.206896552
NRXN33	2.05E-06	0.1131877	0.155	0.124	0.04931568	2.4	NRXN3	1.25
HDGF2	2.07E-06	0.1300834	0.296	0.263	0.04985295	2.4	HDGF	1.125475285
TNFSF102	0	1.479071	0.787	0.426	0	2.5	TNFSF10	1.84741784
TMC51	0	1.47386	0.63	0.161	0	2.5	TMC5	3.913043478
S100A61	0	1.469818	0.878	0.529	0	2.5	S100A6	1.65973535
TSPAN18	0	1.368976	0.546	0.181	0	2.5	TSPAN1	3.016574586
ANKS1B2	0	1.328653	0.851	0.404	0	2.5	ANKS1B	2.106435644
PTPRE1	0	1.298511	0.639	0.211	0	2.5	PTPRE	3.028436019
SAT11	0	1.280269	0.998	0.977	0	2.5	SAT1	1.021494371
AFF34	0	1.27643	0.744	0.28	0	2.5	AFF3	2.657142857
SLC7A24	0	1.271685	0.724	0.239	0	2.5	SLC7A2	3.029288703
ERBB44	0	1.257394	0.918	0.431	0	2.5	ERBB4	2.129930394
CSGALNACT11	0	1.234559	0.522	0.136	0	2.5	CSGALNAC	3.838235294
SMYD31	0	1.230147	0.695	0.289	0	2.5	SMYD3	2.404844291
MAP3K17	0	1.227693	0.84	0.402	0	2.5	MAP3K1	2.089552239
NAALADL23	0	1.224861	0.846	0.453	0	2.5	NAALADL2	1.867549669
KCCAT211	0	1.192615	0.37	0.075	0	2.5	KCCAT211	4.933333333
ALCAM4	0	1.189142	0.88	0.458	0	2.5	ALCAM	1.92139738
LIMCH12	0	1.098424	0.867	0.432	0	2.5	LIMCH1	2.006944444

FOXP12	0	1.091106	0.931	0.591	0	2.5	FOXP1	1.575296108
TBX34	0	1.063704	0.638	0.217	0	2.5	TBX3	2.940092166
TSC22D32	0	1.060541	0.472	0.146	0	2.5	TSC22D3	3.232876712
C1QTNF3-AMACR1	0	1.045794	0.617	0.275	0	2.5	C1QTNF3-A	2.243636364
PCED1B1	0	1.014991	0.369	0.074	0	2.5	PCED1B	4.986486486
ZMYND82	0	1.012139	0.637	0.292	0	2.5	ZMYND8	2.181506849
NBEA1	0	0.9924619	0.577	0.204	0	2.5	NBEA	2.828431373
ZFP36L11	0	0.9778988	0.857	0.554	0	2.5	ZFP36L1	1.546931408
DPYD2	0	0.9664844	0.825	0.475	0	2.5	DPYD	1.736842105
TFPI4	0	0.9502498	0.692	0.273	0	2.5	TFPI	2.534798535
COL4A54	0	0.9471422	0.551	0.172	0	2.5	COL4A5	3.203488372
CAPN83	0	0.9448668	0.547	0.171	0	2.5	CAPN8	3.198830409
FRY	0	0.9293445	0.353	0.066	0	2.5	FRY	5.348484848
TMSB4X2	0	0.927827	0.984	0.93	0	2.5	TMSB4X	1.058064516
TTC63	0	0.8815035	0.466	0.132	0	2.5	TTC6	3.53030303
EIF4G32	0	0.8743323	0.838	0.464	0	2.5	EIF4G3	1.806034483
SPTSSB3	0	0.8638142	0.3	0.06	0	2.5	SPTSSB	5
ST8SIA62	0	0.8277039	0.36	0.096	0	2.5	ST8SIA6	3.75
INPP4B3	0	0.7691871	0.682	0.278	0	2.5	INPP4B	2.45323741
SDR16C51	0	0.7297625	0.294	0.06	0	2.5	SDR16C5	4.9
DNAJC124	0	0.7080874	0.756	0.338	0	2.5	DNAJC12	2.236686391
MALAT14	0	0.6716096	1	1	0	2.5	MALAT1	1
ANKRD30A4	7.49E-308	0.8350091	0.834	0.419	1.81E-303	2.5	ANKRD30A	1.990453461
PRSS232	3.49E-304	0.8938996	0.505	0.181	8.43E-300	2.5	PRSS23	2.790055249
C8orf44	7.31E-303	0.5780276	0.87	0.469	1.76E-298	2.5	C8orf4	1.855010661
SYTL22	1.66E-301	0.826966	0.791	0.402	4.00E-297	2.5	SYTL2	1.967661692
SCCPDH1	1.84E-297	0.6922905	0.295	0.068	4.44E-293	2.5	SCCPDH	4.338235294
TBC1D93	6.10E-295	0.8523987	0.763	0.404	1.47E-290	2.5	TBC1D9	1.888613861
TNC4	6.08E-292	0.8872298	0.65	0.286	1.47E-287	2.5	TNC	2.272727273
TMEM45B3	2.15E-281	0.7481701	0.412	0.13	5.19E-277	2.5	TMEM45B	3.169230769
ATP1B11	1.17E-277	0.899192	0.948	0.735	2.82E-273	2.5	ATP1B1	1.289795918
ACADSB4	6.09E-276	0.8128398	0.468	0.165	1.47E-271	2.5	ACADSB	2.836363636
VAMP82	3.21E-272	0.8582402	0.908	0.738	7.73E-268	2.5	VAMP8	1.230352304
NCKAP52	3.44E-271	0.9056031	0.423	0.143	8.28E-267	2.5	NCKAP5	2.958041958
RP11-96H19.11	7.96E-267	0.9303898	0.442	0.162	1.92E-262	2.5	RP11-96H1	2.728395062
FARP11	2.11E-263	0.9471035	0.653	0.341	5.09E-259	2.5	FARP1	1.914956012
RORA2	3.52E-256	0.9166008	0.824	0.537	8.50E-252	2.5	RORA	1.534450652
DLGAP13	8.48E-256	0.7899208	0.456	0.167	2.05E-251	2.5	DLGAP1	2.730538922
AGR34	3.67E-252	0.7356591	0.482	0.179	8.84E-248	2.5	AGR3	2.69273743
ASPH3	1.05E-248	0.7601155	0.561	0.245	2.52E-244	2.5	ASPH	2.289795918
VAV34	4.53E-246	0.8415737	0.557	0.247	1.09E-241	2.5	VAV3	2.255060729
LYPD6B1	9.71E-246	0.6931654	0.311	0.086	2.34E-241	2.5	LYPD6B	3.61627907
ENPP13	3.47E-240	0.6893637	0.344	0.104	8.37E-236	2.5	ENPP1	3.307692308
SERHL22	1.77E-239	0.8295259	0.347	0.11	4.26E-235	2.5	SERHL2	3.154545455
S100A113	2.18E-238	0.7636028	0.911	0.697	5.25E-234	2.5	S100A11	1.307030129
C16orf452	1.01E-233	0.7293115	0.484	0.192	2.43E-229	2.5	C16orf45	2.520833333
NTN41	1.21E-233	0.8470543	0.569	0.262	2.92E-229	2.5	NTN4	2.171755725
TBL1XR12	2.43E-232	0.7754595	0.825	0.547	5.86E-228	2.5	TBL1XR1	1.508226691
TFAP2B2	8.61E-232	0.6074217	0.215	0.045	2.08E-227	2.5	TFAP2B	4.777777778
CPE4	1.21E-229	0.7540153	0.446	0.17	2.92E-225	2.5	CPE	2.623529412
MAML31	1.55E-226	0.7893322	0.623	0.312	3.73E-222	2.5	MAML3	1.996794872
EPB41L4A1	2.46E-223	0.6577095	0.378	0.13	5.92E-219	2.5	EPB41L4A	2.907692308

BCAR32	3.83E-223	0.7831407	0.543	0.242	9.23E-219	2.5	BCAR3	2.243801653
NEBL2	5.52E-221	0.6802555	0.899	0.629	1.33E-216	2.5	NEBL	1.429252782
CFB1	3.56E-220	0.9767366	0.356	0.125	8.59E-216	2.5	CFB	2.848
S100A41	3.48E-219	0.818512	0.354	0.122	8.39E-215	2.5	S100A4	2.901639344
GLCCI13	1.95E-217	0.6576225	0.578	0.262	4.71E-213	2.5	GLCCI1	2.20610687
APBB22	1.87E-216	0.7776505	0.507	0.222	4.50E-212	2.5	APBB2	2.283783784
TRERF11	6.72E-216	0.6719563	0.376	0.131	1.62E-211	2.5	TRERF1	2.870229008
NFIA1	1.51E-213	0.7591702	0.41	0.158	3.63E-209	2.5	NFIA	2.594936709
TPCN13	4.76E-212	0.6661746	0.373	0.135	1.15E-207	2.5	TPCN1	2.762962963
CENPP	1.85E-207	0.6674842	0.194	0.041	4.46E-203	2.5	CENPP	4.731707317
LINC005782	2.11E-206	0.6289211	0.222	0.053	5.09E-202	2.5	LINC00578	4.188679245
BCKDHB1	8.62E-206	0.6314963	0.3	0.093	2.08E-201	2.5	BCKDHB	3.225806452
IFITM2	4.43E-202	0.6128945	0.235	0.062	1.07E-197	2.5	IFITM2	3.790322581
ABCC31	2.55E-198	0.5443292	0.235	0.061	6.14E-194	2.5	ABCC3	3.852459016
AC018359.11	7.00E-197	0.6761579	0.143	0.023	1.69E-192	2.5	AC018359.	6.217391304
RAB11FIP13	9.44E-197	0.5981151	0.943	0.703	2.28E-192	2.5	RAB11FIP1	1.341394026
SLC38A23	1.08E-196	0.6877661	0.645	0.344	2.61E-192	2.5	SLC38A2	1.875
SAMD122	5.61E-194	0.7007213	0.479	0.217	1.35E-189	2.5	SAMD12	2.207373272
OSBPL32	2.40E-193	0.6532704	0.464	0.198	5.79E-189	2.5	OSBPL3	2.343434343
CYB5A3	4.67E-193	0.695371	0.497	0.225	1.13E-188	2.5	CYB5A	2.208888889
PIP5K1B1	3.67E-192	0.7172597	0.304	0.1	8.86E-188	2.5	PIP5K1B	3.04
SETBP11	5.68E-191	0.6216314	0.261	0.077	1.37E-186	2.5	SETBP1	3.38961039
SLC27A44	1.28E-190	0.9223354	0.878	0.679	3.09E-186	2.5	SLC27A4	1.293078056
CTNND24	6.33E-189	0.648051	0.35	0.126	1.53E-184	2.5	CTNND2	2.777777778
TANC22	1.05E-188	0.6410187	0.739	0.439	2.54E-184	2.5	TANC2	1.683371298
MAP3K53	3.66E-187	0.747424	0.613	0.33	8.82E-183	2.5	MAP3K5	1.857575758
ESR12	2.41E-184	0.6758758	0.389	0.156	5.82E-180	2.5	ESR1	2.493589744
TTC39C1	6.52E-181	0.7178507	0.418	0.179	1.57E-176	2.5	TTC39C	2.335195531
DACH13	2.85E-177	0.532952	0.254	0.075	6.87E-173	2.5	DACH1	3.386666667
SEMA3C3	3.26E-177	0.6346741	0.522	0.245	7.86E-173	2.5	SEMA3C	2.130612245
MPP74	3.81E-176	0.5143546	0.286	0.093	9.18E-172	2.5	MPP7	3.075268817
ARHGAP322	4.04E-176	0.6937716	0.656	0.381	9.75E-172	2.5	ARHGAP32	1.721784777
SPIDR3	1.71E-175	0.6379427	0.821	0.569	4.11E-171	2.5	SPIDR	1.44288225
PBX11	4.36E-175	0.7366639	0.483	0.231	1.05E-170	2.5	PBX1	2.090909091
CYP4Z1	5.28E-175	0.3009836	0.073	0.005	1.27E-170	2.5	CYP4Z1	14.6
MAGI34	3.10E-174	0.6111166	0.635	0.347	7.46E-170	2.5	MAGI3	1.829971182
TP53INP11	1.49E-173	0.4679766	0.223	0.061	3.60E-169	2.5	TP53INP1	3.655737705
DRAM12	4.13E-173	0.645434	0.532	0.263	9.97E-169	2.5	DRAM1	2.022813688
SERPINA32	4.27E-173	0.8792486	0.48	0.234	1.03E-168	2.5	SERPINA3	2.051282051
UTRN2	9.49E-173	0.6738027	0.504	0.247	2.29E-168	2.5	UTRN	2.04048583
SFMBT23	1.13E-172	0.6040404	0.366	0.141	2.74E-168	2.5	SFMBT2	2.595744681
PRLR3	1.92E-171	0.6829572	0.453	0.206	4.62E-167	2.5	PRLR	2.199029126
AZGP14	1.93E-171	0.8090948	0.775	0.515	4.65E-167	2.5	AZGP1	1.504854369
DUSP43	9.64E-170	0.5435319	0.31	0.11	2.33E-165	2.5	DUSP4	2.818181818
IRF2BPL3	3.48E-169	0.6067323	0.349	0.137	8.38E-165	2.5	IRF2BPL	2.547445255
EFHD14	5.76E-169	0.5065805	0.556	0.264	1.39E-164	2.5	EFHD1	2.106060606
PLAT4	1.10E-168	0.6972723	0.231	0.067	2.64E-164	2.5	PLAT	3.447761194
RUNX12	3.92E-166	0.5954793	0.855	0.614	9.45E-162	2.5	RUNX1	1.392508143
PTPRM2	2.96E-165	0.6257088	0.461	0.21	7.14E-161	2.5	PTPRM	2.195238095
SMIM144	4.04E-164	0.5246246	0.661	0.362	9.74E-160	2.5	SMIM14	1.825966851
FBXL172	1.25E-163	0.6785535	0.483	0.235	3.01E-159	2.5	FBXL17	2.055319149
NEAT13	2.65E-163	0.6258352	0.996	0.94	6.39E-159	2.5	NEAT1	1.059574468

KIF16B3	8.17E-163	0.585015	0.383	0.159	1.97E-158	2.5	KIF16B	2.408805031
DIO24	2.95E-162	0.7292798	0.437	0.193	7.11E-158	2.5	DIO2	2.264248705
CD91	8.03E-162	0.6717441	0.621	0.354	1.94E-157	2.5	CD9	1.754237288
MSI23	4.33E-160	0.6000809	0.578	0.31	1.04E-155	2.5	MSI2	1.864516129
SLC44A43	4.76E-159	0.5199197	0.278	0.095	1.15E-154	2.5	SLC44A4	2.926315789
EREG4	8.29E-159	0.7725099	0.483	0.23	2.00E-154	2.5	EREG	2.1
PTPN134	9.72E-159	0.5573398	0.373	0.152	2.34E-154	2.5	PTPN13	2.453947368
TSC22D12	3.88E-158	0.6474633	0.599	0.333	9.36E-154	2.5	TSC22D1	1.798798799
FGF133	1.45E-157	0.5046491	0.713	0.416	3.50E-153	2.5	FGF13	1.713942308
MTSS12	1.48E-157	0.6338678	0.335	0.129	3.57E-153	2.5	MTSS1	2.596899225
LRBA3	1.61E-156	0.3570232	0.872	0.605	3.87E-152	2.5	LRBA	1.441322314
MOCOS1	6.48E-156	0.5476253	0.258	0.086	1.56E-151	2.5	MOCOS	3
FTX3	2.60E-154	0.5906421	0.791	0.523	6.27E-150	2.5	FTX	1.512428298
MYCBP22	4.27E-154	0.6161688	0.436	0.206	1.03E-149	2.5	MYCBP2	2.116504854
THSD42	3.42E-153	0.6165288	0.786	0.531	8.24E-149	2.5	THSD4	1.480225989
SEMA4B	1.53E-152	0.5862785	0.421	0.196	3.68E-148	2.5	SEMA4B	2.147959184
SUMF12	2.06E-152	0.6035206	0.337	0.136	4.98E-148	2.5	SUMF1	2.477941176
MYBPC13	4.37E-152	0.7182112	0.44	0.203	1.05E-147	2.5	MYBPC1	2.167487685
SIDT11	1.22E-151	0.4033366	0.141	0.029	2.95E-147	2.5	SIDT1	4.862068966
CERS63	1.56E-151	0.5022639	0.35	0.14	3.75E-147	2.5	CERS6	2.5
TMBIM64	6.42E-150	0.538718	0.946	0.848	1.55E-145	2.5	TMBIM6	1.115566038
GLUL1	9.20E-149	0.5791993	0.599	0.345	2.22E-144	2.5	GLUL	1.736231884
BCL2L111	3.04E-148	0.5483928	0.285	0.105	7.34E-144	2.5	BCL2L11	2.714285714
KLHL21	6.03E-148	0.5097396	0.304	0.115	1.45E-143	2.5	KLHL2	2.643478261
ITGAV4	1.43E-147	0.5348664	0.643	0.365	3.46E-143	2.5	ITGAV	1.761643836
IER33	1.81E-147	0.5660957	0.544	0.287	4.36E-143	2.5	IER3	1.895470383
MEIS11	1.01E-146	0.4743201	0.192	0.053	2.45E-142	2.5	MEIS1	3.622641509
MBOAT11	2.65E-146	0.5490085	0.299	0.112	6.38E-142	2.5	MBOAT1	2.669642857
MICAL21	4.18E-146	0.5685406	0.302	0.116	1.01E-141	2.5	MICAL2	2.603448276
CADPS23	1.16E-145	0.4212723	0.637	0.371	2.81E-141	2.5	CADPS2	1.716981132
WWP12	3.70E-145	0.5605005	0.531	0.28	8.92E-141	2.5	WWP1	1.896428571
PDCD41	5.38E-145	0.67127	0.432	0.213	1.30E-140	2.5	PDCD4	2.028169014
CD634	2.36E-144	0.5734397	0.612	0.349	5.70E-140	2.5	CD63	1.753581662
TSPAN53	7.86E-144	0.4898268	0.55	0.274	1.90E-139	2.5	TSPAN5	2.00729927
CCDC912	8.04E-144	0.6025564	0.563	0.317	1.94E-139	2.5	CCDC91	1.776025237
BRE2	2.14E-143	0.5903064	0.398	0.182	5.15E-139	2.5	BRE	2.186813187
MAST43	4.13E-143	0.5573245	0.76	0.495	9.97E-139	2.5	MAST4	1.535353535
CLIC63	6.28E-143	0.5702089	0.412	0.19	1.51E-138	2.5	CLIC6	2.168421053
TFAP2A1	1.02E-142	0.4994633	0.326	0.131	2.46E-138	2.5	TFAP2A	2.488549618
EXOC43	3.02E-141	0.5832219	0.55	0.307	7.28E-137	2.5	EXOC4	1.791530945
RAB27B3	4.14E-141	0.5457113	0.322	0.129	9.97E-137	2.5	RAB27B	2.496124031
GLI34	1.05E-140	0.4723797	0.299	0.113	2.53E-136	2.5	GLI3	2.646017699
RALGAPA2	1.89E-140	0.574572	0.459	0.23	4.55E-136	2.5	RALGAPA2	1.995652174
PARD3B1	2.42E-140	0.565095	0.388	0.176	5.84E-136	2.5	PARD3B	2.204545455
ENOX13	2.51E-140	0.595569	0.3	0.116	6.04E-136	2.5	ENOX1	2.586206897
LSAMP4	3.38E-140	0.5739959	0.705	0.438	8.16E-136	2.5	LSAMP	1.609589041
C1orf1321	1.76E-139	0.4905142	0.248	0.086	4.25E-135	2.5	C1orf132	2.88372093
CD442	3.00E-139	0.6168511	0.788	0.561	7.22E-135	2.5	CD44	1.404634581
SOX43	4.96E-139	0.4406607	0.966	0.82	1.20E-134	2.5	SOX4	1.17804878
SERPINA1	7.51E-139	0.3935188	0.196	0.057	1.81E-134	2.5	SERPINA1	3.438596491
ITFG13	2.65E-138	0.5783815	0.471	0.244	6.39E-134	2.5	ITFG1	1.930327869
CUX11	2.93E-138	0.5509483	0.567	0.319	7.06E-134	2.5	CUX1	1.777429467

RAPH14	6.49E-138	0.5736625	0.554	0.306	1.56E-133	2.5	RAPH1	1.810457516
OPHN11	9.09E-136	0.5725906	0.541	0.298	2.19E-131	2.5	OPHN1	1.815436242
UMAD12	2.56E-135	0.5374922	0.393	0.18	6.17E-131	2.5	UMAD1	2.183333333
ARHGEF31	3.04E-134	0.5341274	0.601	0.352	7.34E-130	2.5	ARHGEF3	1.707386364
WWOX2	1.44E-133	0.683638	0.422	0.209	3.48E-129	2.5	WWOX	2.019138756
ADAM102	2.10E-133	0.5261247	0.593	0.343	5.06E-129	2.5	ADAM10	1.728862974
ITGA31	2.35E-132	0.5437734	0.351	0.155	5.68E-128	2.5	ITGA3	2.264516129
RASEF4	3.45E-132	0.4772277	0.486	0.242	8.31E-128	2.5	RASEF	2.008264463
ERMP12	1.67E-131	0.3413629	0.153	0.038	4.02E-127	2.5	ERMP1	4.026315789
ZBTB203	3.91E-131	0.539361	0.734	0.491	9.43E-127	2.5	ZBTB20	1.49490835
KLHL51	3.08E-130	0.588158	0.482	0.257	7.42E-126	2.5	KLHL5	1.875486381
PARK21	1.11E-129	0.5402603	0.352	0.157	2.67E-125	2.5	PARK2	2.242038217
TRPS13	1.57E-129	0.5397433	0.903	0.713	3.78E-125	2.5	TRPS1	1.266479663
AGO41	4.59E-129	0.5206867	0.345	0.152	1.11E-124	2.5	AGO4	2.269736842
FKBP53	7.43E-129	0.6948966	0.536	0.308	1.79E-124	2.5	FKBP5	1.74025974
MIPOL13	2.02E-127	0.4611143	0.364	0.159	4.87E-123	2.5	MIPOL1	2.289308176
TRIQK2	5.20E-127	0.3820212	0.201	0.063	1.25E-122	2.5	TRIQK	3.19047619
EMP23	6.81E-127	0.458815	0.269	0.101	1.64E-122	2.5	EMP2	2.663366337
PTGR11	3.08E-123	0.7264649	0.464	0.257	7.43E-119	2.5	PTGR1	1.805447471
RP11-774D14.11	6.71E-121	0.4609495	0.237	0.087	1.62E-116	2.5	RP11-774D	2.724137931
PTGS11	9.37E-121	0.239404	0.07	0.008	2.26E-116	2.5	PTGS1	8.75
PERP3	1.13E-120	0.5263032	0.661	0.425	2.73E-116	2.5	PERP	1.555294118
ANK32	3.19E-120	0.504737	0.502	0.276	7.70E-116	2.5	ANK3	1.81884058
TNIK4	8.69E-120	0.4577357	0.324	0.138	2.09E-115	2.5	TNIK	2.347826087
AC018816.34	1.62E-119	0.4838205	0.227	0.081	3.91E-115	2.5	AC018816.3	2.802469136
TPBG2	1.72E-119	0.4663969	0.354	0.16	4.16E-115	2.5	TPBG	2.2125
DSTN2	3.27E-119	0.5419875	0.762	0.559	7.88E-115	2.5	DSTN	1.363148479
DUSP164	3.57E-119	0.5604934	0.626	0.392	8.61E-115	2.5	DUSP16	1.596938776
DUSP104	3.64E-119	0.4786957	0.412	0.198	8.78E-115	2.5	DUSP10	2.080808081
VWA82	4.07E-119	0.4550589	0.23	0.083	9.81E-115	2.5	VWA8	2.771084337
TP53INP21	3.16E-118	0.3062001	0.138	0.035	7.62E-114	2.5	TP53INP2	3.942857143
XBP12	3.16E-118	0.4943014	0.591	0.363	7.62E-114	2.5	XBP1	1.628099174
TMSB104	6.21E-118	0.6308952	0.865	0.714	1.50E-113	2.5	TMSB10	1.211484594
MBD52	2.89E-117	0.5346163	0.497	0.279	6.97E-113	2.5	MBD5	1.781362007
TOX33	7.59E-117	0.530528	0.333	0.148	1.83E-112	2.5	TOX3	2.25
STEAP41	2.41E-116	0.2876816	0.11	0.023	5.81E-112	2.5	STEAP4	4.782608696
DLG53	4.11E-116	0.6456548	0.442	0.238	9.92E-112	2.5	DLG5	1.857142857
CD992	5.52E-115	0.4079228	0.286	0.116	1.33E-110	2.5	CD99	2.465517241
SRPX21	6.37E-115	0.3918603	0.183	0.058	1.54E-110	2.5	SRPX2	3.155172414
STC21	1.87E-114	0.4641089	0.22	0.079	4.51E-110	2.5	STC2	2.784810127
LINC-PINT3	9.88E-114	0.4909471	0.772	0.547	2.38E-109	2.5	LINC-PINT	1.411334552
CPEB24	3.53E-113	0.4746124	0.518	0.28	8.50E-109	2.5	CPEB2	1.85
FAAH22	4.30E-112	0.5242255	0.366	0.182	1.04E-107	2.5	FAAH2	2.010989011
HIST1H2AC3	5.18E-112	0.5870304	0.555	0.339	1.25E-107	2.5	HIST1H2AC	1.637168142
VPS13B2	5.24E-112	0.4738355	0.383	0.19	1.26E-107	2.5	VPS13B	2.015789474
FNIP23	1.82E-111	0.4419374	0.237	0.089	4.38E-107	2.5	FNIP2	2.662921348
TTLL52	6.07E-111	0.4805838	0.469	0.257	1.46E-106	2.5	TTLL5	1.824902724
PLD12	6.08E-111	0.4959671	0.266	0.11	1.47E-106	2.5	PLD1	2.418181818
NEDD41	8.29E-110	0.4655132	0.255	0.103	2.00E-105	2.5	NEDD4	2.475728155
RPS6KA52	8.80E-110	0.5047209	0.387	0.196	2.12E-105	2.5	RPS6KA5	1.974489796
WNT5A1	4.30E-108	0.2957879	0.118	0.028	1.04E-103	2.5	WNT5A	4.214285714
NCEH13	8.92E-108	0.4902243	0.62	0.384	2.15E-103	2.5	NCEH1	1.614583333

FHDC11	4.24E-107	0.4252307	0.255	0.103	1.02E-102	2.5	FHDC1	2.475728155
GRHL22	5.62E-107	0.5209617	0.46	0.258	1.36E-102	2.5	GRHL2	1.782945736
GOLM13	6.47E-107	0.3942214	0.416	0.208	1.56E-102	2.5	GOLM1	2
DHX321	8.03E-107	0.492352	0.48	0.272	1.94E-102	2.5	DHX32	1.764705882
NRIP13	1.62E-106	0.437659	0.457	0.242	3.90E-102	2.5	NRIP1	1.888429752
DGKD3	3.42E-106	0.4107695	0.26	0.105	8.24E-102	2.5	DGKD	2.476190476
NOVA11	7.70E-106	0.3876768	0.17	0.054	1.86E-101	2.5	NOVA1	3.148148148
CRADD1	7.88E-106	0.4720901	0.293	0.13	1.90E-101	2.5	CRADD	2.253846154
FOXA14	1.03E-105	0.3701799	0.252	0.1	2.49E-101	2.5	FOXA1	2.52
TBC1D53	4.45E-105	0.4673021	0.547	0.326	1.07E-100	2.5	TBC1D5	1.67791411
UBAC22	1.52E-104	0.4706544	0.394	0.207	3.67E-100	2.5	UBAC2	1.903381643
ABCA42	2.08E-104	0.3253378	0.131	0.035	5.00E-100	2.5	ABCA4	3.742857143
CASC153	5.28E-104	0.2385112	0.75	0.49	1.27E-99	2.5	CASC15	1.530612245
ARRB11	2.60E-103	0.346208	0.164	0.052	6.27E-99	2.5	ARRB1	3.153846154
MLLT32	4.24E-103	0.4671974	0.39	0.199	1.02E-98	2.5	MLLT3	1.959798995
PSD33	3.86E-102	0.4515268	0.544	0.319	9.30E-98	2.5	PSD3	1.705329154
TTTY141	1.32E-101	0.5850023	0.61	0.395	3.19E-97	2.5	TTTY14	1.544303797
CTD-2015H6.31	1.97E-101	0.3489848	0.16	0.051	4.74E-97	2.5	CTD-2015H	3.137254902
HIST1H2BB3	9.28E-101	0.4874878	0.317	0.149	2.24E-96	2.5	HIST1H2BB	2.127516779
HS6ST23	9.99E-101	0.4478554	0.29	0.127	2.41E-96	2.5	HS6ST2	2.283464567
CXCL134	1.53E-100	0.2824525	0.624	0.382	3.68E-96	2.5	CXCL13	1.633507853
SYNE22	5.19E-100	0.4414687	0.736	0.519	1.25E-95	2.5	SYNE2	1.418111753
SCNN1A1	7.08E-100	0.4081595	0.248	0.103	1.71E-95	2.5	SCNN1A	2.40776699
ABCD34	1.23E-99	0.4063383	0.333	0.158	2.98E-95	2.5	ABCD3	2.107594937
IL13RA12	1.37E-99	0.376032	0.254	0.105	3.30E-95	2.5	IL13RA1	2.419047619
C9orf841	2.42E-99	0.3370685	0.165	0.054	5.83E-95	2.5	C9orf84	3.055555556
DGKH2	6.46E-99	0.408871	0.498	0.282	1.56E-94	2.5	DGKH	1.765957447
PARP82	1.29E-98	0.4516596	0.301	0.14	3.10E-94	2.5	PARP8	2.15
WLS4	9.03E-98	0.4183243	0.327	0.154	2.18E-93	2.5	WLS	2.123376623
SPRED21	1.45E-97	0.4623997	0.372	0.19	3.50E-93	2.5	SPRED2	1.957894737
PLPP31	2.86E-97	0.5280178	0.417	0.229	6.90E-93	2.5	PLPP3	1.820960699
DMXL2	1.11E-96	0.3038703	0.123	0.033	2.67E-92	2.5	DMXL2	3.727272727
SLC44A11	2.79E-96	0.4292842	0.28	0.126	6.73E-92	2.5	SLC44A1	2.222222222
PRKG12	5.91E-96	0.4340116	0.29	0.129	1.43E-91	2.5	PRKG1	2.248062016
COL4A63	2.66E-95	0.3521445	0.154	0.049	6.41E-91	2.5	COL4A6	3.142857143
SH2D4A1	3.73E-95	0.3389544	0.142	0.043	9.00E-91	2.5	SH2D4A	3.302325581
PRKAA22	7.01E-95	0.3916648	0.258	0.111	1.69E-90	2.5	PRKAA2	2.324324324
CDC14A4	9.68E-95	0.4165246	0.303	0.141	2.33E-90	2.5	CDC14A	2.14893617
PTMA1	1.74E-94	0.4751223	0.786	0.598	4.21E-90	2.5	PTMA	1.314381271
HNMT2	7.26E-94	0.5639041	0.459	0.273	1.75E-89	2.5	HNMT	1.681318681
LMCD14	7.51E-94	0.5131694	0.227	0.092	1.81E-89	2.5	LMCD1	2.467391304
UGCG2	1.23E-93	0.4072437	0.691	0.461	2.96E-89	2.5	UGCG	1.498915401
PLXND11	2.99E-93	0.2510312	0.1	0.023	7.21E-89	2.5	PLXND1	4.347826087
SLC22A232	3.41E-93	0.4702261	0.411	0.226	8.21E-89	2.5	SLC22A23	1.818584071
PIK3C2B1	4.55E-93	0.3719676	0.193	0.072	1.10E-88	2.5	PIK3C2B	2.680555556
TFF14	5.23E-93	0.5177294	0.223	0.09	1.26E-88	2.5	TFF1	2.477777778
CDC42BPA2	1.30E-92	0.427174	0.568	0.356	3.14E-88	2.5	CDC42BPA	1.595505618
NRP13	2.18E-92	0.3488184	0.15	0.048	5.26E-88	2.5	NRP1	3.125
MPHOSPH62	1.62E-91	0.350802	0.257	0.111	3.91E-87	2.5	MPHOSPH6	2.315315315
CCDC102B2	1.70E-91	0.346149	0.096	0.022	4.10E-87	2.5	CCDC102B	4.363636364
ACER21	2.92E-91	0.3007216	0.128	0.037	7.04E-87	2.5	ACER2	3.459459459
TMTC23	3.22E-91	0.4130517	0.564	0.352	7.76E-87	2.5	TMTC2	1.602272727

DOCK81	2.00E-90	0.2873621	0.151	0.049	4.82E-86	2.5	DOCK8	3.081632653
REPS12	2.22E-90	0.5008048	0.474	0.285	5.36E-86	2.5	REPS1	1.663157895
ATAD2B2	2.59E-90	0.4170882	0.349	0.181	6.24E-86	2.5	ATAD2B	1.928176796
SSBP23	3.36E-90	0.4968109	0.612	0.412	8.10E-86	2.5	SSBP2	1.485436893
LIMA11	3.50E-90	0.4584294	0.575	0.375	8.43E-86	2.5	LIMA1	1.533333333
RP11-382A20.33	8.33E-90	0.3687951	0.234	0.098	2.01E-85	2.5	RP11-382A	2.387755102
MYL12B4	9.60E-90	0.3038784	0.925	0.806	2.32E-85	2.5	MYL12B	1.14764268
PER21	2.01E-89	0.398764	0.273	0.125	4.84E-85	2.5	PER2	2.184
HMGCLL12	2.45E-89	0.3078945	0.116	0.032	5.92E-85	2.5	HMGCLL1	3.625
ADGRL34	3.29E-89	0.4180559	0.224	0.092	7.93E-85	2.5	ADGRL3	2.434782609
CAMKMT2	5.11E-89	0.4668388	0.311	0.153	1.23E-84	2.5	CAMKMT	2.032679739
N4BP2L23	6.03E-89	0.3682074	0.883	0.703	1.45E-84	2.5	N4BP2L2	1.256045519
TEX21	9.66E-89	0.392282	0.214	0.087	2.33E-84	2.5	TEX2	2.459770115
PPP3CA4	1.42E-88	0.3966575	0.751	0.537	3.42E-84	2.5	PPP3CA	1.398510242
ZNF911	1.68E-88	0.4550431	0.362	0.193	4.05E-84	2.5	ZNF91	1.875647668
ITPR13	2.01E-88	0.3685346	0.214	0.086	4.86E-84	2.5	ITPR1	2.488372093
TRAPPC93	2.60E-88	0.4129673	0.357	0.187	6.28E-84	2.5	TRAPPC9	1.909090909
HSBP13	3.43E-88	0.4688045	0.709	0.533	8.28E-84	2.5	HSBP1	1.330206379
ASCC32	6.25E-88	0.45737	0.4	0.222	1.51E-83	2.5	ASCC3	1.801801802
RP11-624L4.12	6.64E-88	0.4218804	0.153	0.051	1.60E-83	2.5	RP11-624L4	3
FXYD34	7.01E-88	0.5213624	0.775	0.614	1.69E-83	2.5	FXYD3	1.262214984
TNS41	1.71E-87	0.3110346	0.113	0.031	4.12E-83	2.5	TNS4	3.64516129
DDX55	1.80E-87	0.4260242	0.684	0.472	4.34E-83	2.5	DDX5	1.449152542
C15orf482	3.06E-87	0.2813489	0.697	0.47	7.39E-83	2.5	C15orf48	1.482978723
TBL1X1	1.23E-86	0.4473905	0.365	0.196	2.97E-82	2.5	TBL1X	1.862244898
CD594	4.49E-86	0.4557284	0.958	0.871	1.08E-81	2.5	CD59	1.099885189
SCMH13	4.85E-86	0.4352407	0.393	0.217	1.17E-81	2.5	SCMH1	1.811059908
SESN12	6.14E-86	0.4006443	0.239	0.104	1.48E-81	2.5	SESN1	2.298076923
HLA-C1	7.38E-86	0.463782	0.48	0.29	1.78E-81	2.5	HLA-C	1.655172414
C5orf171	1.51E-85	0.4700135	0.232	0.1	3.63E-81	2.5	C5orf17	2.32
HES12	1.52E-85	0.4227268	0.513	0.319	3.67E-81	2.5	HES1	1.60815047
AMPH4	2.36E-85	0.3275356	0.213	0.085	5.69E-81	2.5	AMPH	2.505882353
DHRS31	6.26E-85	0.3929307	0.229	0.099	1.51E-80	2.5	DHRS3	2.313131313
FAM102A1	2.55E-84	0.3733726	0.263	0.121	6.14E-80	2.5	FAM102A	2.173553719
FAM214A2	6.88E-84	0.3753309	0.391	0.212	1.66E-79	2.5	FAM214A	1.844339623
ABHD17C2	7.99E-84	0.3830766	0.205	0.084	1.93E-79	2.5	ABHD17C	2.44047619
RAB121	1.92E-83	0.4814321	0.416	0.24	4.63E-79	2.5	RAB12	1.733333333
EFTUD11	1.99E-83	0.4162683	0.285	0.138	4.81E-79	2.5	EFTUD1	2.065217391
FBXL71	3.43E-83	0.3729309	0.104	0.028	8.28E-79	2.5	FBXL7	3.714285714
PON21	3.73E-83	0.3318769	0.196	0.078	9.00E-79	2.5	PON2	2.512820513
TGFBI1	4.34E-83	0.2423314	0.085	0.019	1.05E-78	2.5	TGFBI	4.473684211
RAD51B2	1.47E-82	0.5372536	0.376	0.211	3.54E-78	2.5	RAD51B	1.781990521
TGM22	1.94E-82	0.4143169	0.2	0.083	4.68E-78	2.5	TGM2	2.409638554
ESRRG	2.31E-82	0.3640667	0.136	0.044	5.58E-78	2.5	ESRRG	3.090909091
HPX4	3.14E-82	0.457305	0.369	0.194	7.58E-78	2.5	HPX	1.902061856
GPD22	3.61E-82	0.3757943	0.504	0.307	8.70E-78	2.5	GPD2	1.641693811
KIAA13244	6.47E-82	0.4297811	0.38	0.207	1.56E-77	2.5	KIAA1324	1.835748792
FGD62	7.18E-82	0.4241196	0.516	0.32	1.73E-77	2.5	FGD6	1.6125
TMEM106B1	7.20E-82	0.3881751	0.281	0.134	1.74E-77	2.5	TMEM106B	2.097014925
TMEM178B1	1.82E-81	0.3567493	0.169	0.063	4.39E-77	2.5	TMEM178B	2.682539683
KAZN1	1.99E-81	0.4044632	0.367	0.197	4.80E-77	2.5	KAZN	1.862944162
NOP103	5.23E-81	0.4099864	0.718	0.534	1.26E-76	2.5	NOP10	1.344569288

NDUFAF62	7.42E-81	0.3531483	0.21	0.088	1.79E-76	2.5	NDUFAF6	2.386363636
IFNGR11	1.60E-80	0.437649	0.426	0.25	3.86E-76	2.5	IFNGR1	1.704
FAM63B1	1.78E-80	0.4076659	0.33	0.174	4.29E-76	2.5	FAM63B	1.896551724
RASSF62	1.94E-80	0.2819849	0.16	0.057	4.67E-76	2.5	RASSF6	2.807017544
TREM11	2.17E-80	0.2385808	0.068	0.013	5.23E-76	2.5	TREM1	5.230769231
LYPD62	2.26E-80	0.279117	0.13	0.041	5.44E-76	2.5	LYPD6	3.170731707
CROT1	2.98E-80	0.2633804	0.118	0.035	7.18E-76	2.5	CROT	3.371428571
RSRC12	4.93E-80	0.4066453	0.445	0.264	1.19E-75	2.5	RSRC1	1.685606061
STYK14	6.45E-80	0.2762539	0.155	0.054	1.56E-75	2.5	STYK1	2.87037037
RP11-141M1.3	6.56E-80	0.4404248	0.198	0.082	1.58E-75	2.5	RP11-141M	2.414634146
VPS13C2	8.71E-80	0.4058551	0.394	0.222	2.10E-75	2.5	VPS13C	1.774774775
ZIM21	1.04E-79	0.2067575	0.068	0.013	2.51E-75	2.5	ZIM2	5.230769231
MAP4K32	1.26E-79	0.3985498	0.413	0.236	3.05E-75	2.5	MAP4K3	1.75
RELL11	2.48E-79	0.346109	0.233	0.103	5.97E-75	2.5	RELL1	2.262135922
PTPRG4	4.12E-79	0.4371975	0.435	0.254	9.93E-75	2.5	PTPRG	1.712598425
PAPSS2	6.37E-79	0.2766996	0.092	0.023	1.53E-74	2.5	PAPSS2	4
CMTM41	1.44E-78	0.3936253	0.275	0.134	3.47E-74	2.5	CMTM4	2.052238806
BATF4	2.30E-78	0.3197267	0.166	0.061	5.56E-74	2.5	BATF	2.721311475
AC011288.21	2.98E-78	0.4278892	0.145	0.05	7.20E-74	2.5	AC011288.2	2.9
NRXN34	5.16E-78	0.3430345	0.246	0.111	1.24E-73	2.5	NRXN3	2.216216216
MTIF33	7.01E-78	0.4151332	0.425	0.251	1.69E-73	2.5	MTIF3	1.693227092
VTI1A2	1.64E-77	0.3623326	0.341	0.182	3.95E-73	2.5	VTI1A	1.873626374
RP11-1028N23.4	2.15E-77	0.1486408	0.042	0.005	5.19E-73	2.5	RP11-1028	8.4
TCF122	2.24E-77	0.360534	0.761	0.57	5.41E-73	2.5	TCF12	1.335087719
CEBPB2	2.52E-77	0.4017072	0.298	0.152	6.09E-73	2.5	CEBPB	1.960526316
UQCR103	6.63E-77	0.3366453	0.681	0.484	1.60E-72	2.5	UQCR10	1.407024793
STC11	1.78E-76	0.3670207	0.121	0.038	4.28E-72	2.5	STC1	3.184210526
TNRC6B2	1.85E-76	0.3769906	0.663	0.463	4.45E-72	2.5	TNRC6B	1.431965443
KCTD15	2.21E-76	0.3246221	0.154	0.056	5.32E-72	2.5	KCTD1	2.75
DYNLT32	3.87E-76	0.393217	0.424	0.249	9.33E-72	2.5	DYNLT3	1.702811245
LNX11	7.66E-76	0.3866584	0.248	0.117	1.85E-71	2.5	LNX1	2.11965812
STPG21	1.22E-75	0.3727281	0.171	0.067	2.94E-71	2.5	STPG2	2.552238806
ANO102	1.68E-75	0.4188436	0.425	0.253	4.05E-71	2.5	ANO10	1.679841897
LINC005044	1.86E-75	0.3805138	0.188	0.076	4.48E-71	2.5	LINC00504	2.473684211
KAT6B3	2.50E-75	0.3874975	0.411	0.235	6.02E-71	2.5	KAT6B	1.74893617
MLPH3	4.18E-75	0.2573982	0.163	0.061	1.01E-70	2.5	MLPH	2.672131148
PLGRKT2	5.55E-75	0.2888382	0.172	0.067	1.34E-70	2.5	PLGRKT	2.567164179
SCARB22	6.20E-75	0.3554537	0.312	0.162	1.49E-70	2.5	SCARB2	1.925925926
RNF1521	1.27E-74	0.3375719	0.191	0.079	3.06E-70	2.5	RNF152	2.417721519
ITM2B4	1.96E-74	0.4138529	0.509	0.328	4.73E-70	2.5	ITM2B	1.551829268
PKM2	1.99E-74	0.4270485	0.517	0.33	4.80E-70	2.5	PKM	1.566666667
BZW21	2.36E-74	0.3447782	0.258	0.124	5.69E-70	2.5	BZW2	2.080645161
MEGF94	2.47E-74	0.3161091	0.251	0.117	5.95E-70	2.5	MEGF9	2.145299145
APPL21	2.73E-74	0.3428079	0.215	0.095	6.59E-70	2.5	APPL2	2.263157895
SLC38A13	3.99E-74	0.3925924	0.562	0.369	9.63E-70	2.5	SLC38A1	1.52303523
PREX14	8.01E-74	0.2878991	0.13	0.043	1.93E-69	2.5	PREX1	3.023255814
ACTR3C2	8.11E-74	0.2846217	0.127	0.043	1.96E-69	2.5	ACTR3C	2.953488372
ARHGEF123	1.09E-73	0.3529063	0.747	0.553	2.64E-69	2.5	ARHGEF12	1.350813743
MAP2K52	1.57E-73	0.3815204	0.24	0.114	3.79E-69	2.5	MAP2K5	2.105263158
WDR703	3.05E-73	0.3632503	0.264	0.129	7.34E-69	2.5	WDR70	2.046511628
ST3GAL41	3.86E-73	0.2882114	0.153	0.056	9.32E-69	2.5	ST3GAL4	2.732142857
BZW12	5.21E-73	0.3772398	0.43	0.256	1.26E-68	2.5	BZW1	1.6796875

ATP10A1	1.50E-72	0.2725561	0.111	0.034	3.62E-68	2.5	ATP10A	3.264705882
EMC33	2.26E-72	0.3797471	0.38	0.217	5.44E-68	2.5	EMC3	1.751152074
PLEKHF22	2.92E-72	0.3542665	0.202	0.088	7.03E-68	2.5	PLEKHF2	2.295454545
ARID1B3	3.69E-72	0.3327797	0.672	0.47	8.91E-68	2.5	ARID1B	1.429787234
FAIM2	4.17E-72	0.1603715	0.045	0.006	1.01E-67	2.5	FAIM2	7.5
PTHLH4	5.35E-72	0.2850031	0.393	0.214	1.29E-67	2.5	PTHLH	1.836448598
CA122	6.53E-72	0.4444224	0.451	0.276	1.57E-67	2.5	CA12	1.634057971
FUT84	8.55E-72	0.2560386	0.421	0.238	2.06E-67	2.5	FUT8	1.768907563
ELOVL53	1.25E-71	0.2363268	0.465	0.272	3.02E-67	2.5	ELOVL5	1.709558824
DNAJC110	1.45E-71	0.4051167	0.409	0.242	3.51E-67	2.5	DNAJC1	1.690082645
CGNL13	1.53E-71	0.4779518	0.319	0.173	3.69E-67	2.5	CGNL1	1.843930636
SPINT11	1.67E-71	0.3951059	0.311	0.166	4.03E-67	2.5	SPINT1	1.873493976
FGF143	1.96E-71	0.5580867	0.18	0.074	4.72E-67	2.5	FGF14	2.432432432
CRY2	2.15E-71	0.3456496	0.202	0.089	5.18E-67	2.5	CRY2	2.269662921
AGR24	3.29E-71	0.3507331	0.404	0.222	7.94E-67	2.5	AGR2	1.81981982
ULK42	4.67E-71	0.3116915	0.221	0.1	1.13E-66	2.5	ULK4	2.21
PCSK62	7.68E-71	0.2548111	0.107	0.032	1.85E-66	2.5	PCSK6	3.34375
MKL22	7.93E-71	0.3848266	0.471	0.296	1.91E-66	2.5	MKL2	1.591216216
TFF34	8.09E-71	0.3206347	0.137	0.048	1.95E-66	2.5	TFF3	2.854166667
FHL23	9.53E-71	0.373408	0.298	0.155	2.30E-66	2.5	FHL2	1.922580645
USP310	1.29E-70	0.3902148	0.45	0.279	3.12E-66	2.5	USP3	1.612903226
RERE4	1.45E-70	0.2863371	0.744	0.546	3.51E-66	2.5	RERE	1.362637363
SP1001	3.21E-70	0.350224	0.275	0.14	7.73E-66	2.5	SP100	1.964285714
MGAT4A1	4.19E-70	0.3365549	0.184	0.078	1.01E-65	2.5	MGAT4A	2.358974359
LARGE3	1.44E-68	0.3492872	0.488	0.305	3.48E-64	2.5	LARGE	1.6
MYOF2	2.22E-68	0.3653376	0.751	0.578	5.35E-64	2.5	MYOF	1.299307958
EPAS12	2.51E-68	0.3529322	0.27	0.136	6.05E-64	2.5	EPAS1	1.985294118
ZCCHC71	6.98E-68	0.3998243	0.582	0.402	1.68E-63	2.5	ZCCHC7	1.447761194
EXOC22	8.89E-68	0.3230874	0.252	0.124	2.14E-63	2.5	EXOC2	2.032258065
KIAA08253	1.03E-67	0.2982771	0.19	0.082	2.48E-63	2.5	KIAA0825	2.317073171
AC007319.14	1.09E-67	0.2894762	0.183	0.077	2.63E-63	2.5	AC007319.	2.376623377
BAZ2B2	2.26E-67	0.382085	0.638	0.458	5.45E-63	2.5	BAZ2B	1.3930131
PCM13	5.06E-67	0.3763482	0.399	0.239	1.22E-62	2.5	PCM1	1.669456067
USP402	5.23E-67	0.2951622	0.159	0.064	1.26E-62	2.5	USP40	2.484375
HACD33	6.89E-67	0.3193472	0.236	0.114	1.66E-62	2.5	HACD3	2.070175439
RP13-726E6.2	7.06E-67	0.2892258	0.149	0.058	1.70E-62	2.5	RP13-726E(	2.568965517
ADCY91	2.20E-66	0.2892344	0.135	0.049	5.30E-62	2.5	ADCY9	2.755102041
CACNA2D14	4.72E-66	0.3118515	0.278	0.141	1.14E-61	2.5	CACNA2D1	1.971631206
MS4A142	6.77E-66	0.1788168	0.068	0.015	1.63E-61	2.5	MS4A14	4.533333333
ZNF420	7.14E-66	0.2353524	0.107	0.034	1.72E-61	2.5	ZNF420	3.147058824
TACSTD22	7.68E-66	0.3037749	0.87	0.71	1.85E-61	2.5	TACSTD2	1.225352113
AHI11	1.56E-65	0.3773205	0.404	0.242	3.75E-61	2.5	AHI1	1.669421488
SLC39A64	1.99E-65	0.2939498	0.461	0.281	4.80E-61	2.5	SLC39A6	1.640569395
MRPL271	2.47E-65	0.3553577	0.305	0.168	5.95E-61	2.5	MRPL27	1.81547619
MBTPS12	2.88E-65	0.290718	0.215	0.1	6.96E-61	2.5	MBTPS1	2.15
PARP91	6.10E-65	0.2810865	0.15	0.059	1.47E-60	2.5	PARP9	2.542372881
VEGFC1	9.82E-65	0.2407786	0.132	0.047	2.37E-60	2.5	VEGFC	2.808510638
MRPS61	1.08E-64	0.3673357	0.427	0.261	2.60E-60	2.5	MRPS6	1.636015326
RBL21	1.41E-64	0.3359262	0.231	0.113	3.41E-60	2.5	RBL2	2.044247788
RABEP11	3.20E-64	0.3248737	0.277	0.144	7.73E-60	2.5	RABEP1	1.923611111
MBNL23	4.33E-64	0.3136655	0.702	0.521	1.04E-59	2.5	MBNL2	1.347408829
C2CD4A4	5.13E-64	0.2291778	0.116	0.039	1.24E-59	2.5	C2CD4A	2.974358974

PROM24	5.33E-64	0.2489796	0.118	0.04	1.28E-59	2.5	PROM2	2.95
NRF11	9.36E-64	0.3471576	0.288	0.155	2.26E-59	2.5	NRF1	1.858064516
FHIT1	1.09E-63	0.4342891	0.501	0.337	2.63E-59	2.5	FHIT	1.486646884
RERG2	1.26E-63	0.4205108	0.321	0.18	3.04E-59	2.5	RERG	1.783333333
CPEB43	1.43E-63	0.3616588	0.401	0.242	3.46E-59	2.5	CPEB4	1.657024793
BRWD12	1.44E-63	0.3502537	0.53	0.352	3.48E-59	2.5	BRWD1	1.505681818
SRI1	1.48E-63	0.3457597	0.186	0.082	3.56E-59	2.5	SRI	2.268292683
MT-ND32	1.59E-63	0.3022077	0.998	0.985	3.82E-59	2.5	MT-ND3	1.01319797
UVRAG3	1.69E-63	0.3559064	0.627	0.442	4.08E-59	2.5	UVRAG	1.418552036
MYO9A2	2.26E-63	0.3494705	0.466	0.297	5.44E-59	2.5	MYO9A	1.569023569
RHOBTB34	2.78E-63	0.3084284	0.24	0.118	6.70E-59	2.5	RHOBTB3	2.033898305
AC008937.22	3.75E-63	0.2420104	0.119	0.041	9.03E-59	2.5	AC008937.	2.902439024
LAMA32	3.85E-63	0.2969959	0.325	0.176	9.29E-59	2.5	LAMA3	1.846590909
SRGAP2B1	5.65E-63	0.2757286	0.171	0.072	1.36E-58	2.5	SRGAP2B	2.375
SLC44A5	5.98E-63	0.2102701	0.072	0.018	1.44E-58	2.5	SLC44A5	4
CERS22	6.16E-63	0.2372385	0.129	0.047	1.49E-58	2.5	CERS2	2.744680851
UBR22	8.02E-63	0.3991405	0.541	0.368	1.94E-58	2.5	UBR2	1.470108696
ARFGEF33	8.29E-63	0.3795809	0.417	0.256	2.00E-58	2.5	ARFGEF3	1.62890625
TANC12	1.03E-62	0.3432995	0.498	0.328	2.48E-58	2.5	TANC1	1.518292683
GNAS4	1.34E-62	0.2620738	0.883	0.734	3.22E-58	2.5	GNAS	1.202997275
DNAH54	1.58E-62	0.2460568	0.147	0.057	3.81E-58	2.5	DNAH5	2.578947368
SLC39A111	1.69E-62	0.3070602	0.204	0.095	4.08E-58	2.5	SLC39A11	2.147368421
PKP43	2.33E-62	0.3890596	0.655	0.482	5.61E-58	2.5	PKP4	1.358921162
DENND1B2	3.00E-62	0.3436736	0.256	0.132	7.23E-58	2.5	DENND1B	1.939393939
EFCAB6	3.84E-62	0.1922636	0.081	0.022	9.25E-58	2.5	EFCAB6	3.681818182
FOSL21	4.08E-62	0.3778275	0.374	0.224	9.85E-58	2.5	FOSL2	1.669642857
RALGAPA12	4.67E-62	0.3814018	0.449	0.285	1.13E-57	2.5	RALGAPA1	1.575438596
FANK13	6.71E-62	0.2858011	0.264	0.135	1.62E-57	2.5	FANK1	1.955555556
MICU21	1.84E-61	0.3345702	0.287	0.155	4.45E-57	2.5	MICU2	1.851612903
TRAFD12	3.57E-61	0.2546822	0.308	0.167	8.61E-57	2.5	TRAFD1	1.844311377
TTC282	3.83E-61	0.3353591	0.278	0.147	9.23E-57	2.5	TTC28	1.891156463
ZNF6082	5.88E-61	0.3656801	0.295	0.164	1.42E-56	2.5	ZNF608	1.798780488
INTS101	6.00E-61	0.3674701	0.234	0.118	1.45E-56	2.5	INTS10	1.983050847
FAM213A3	6.39E-61	0.3095925	0.22	0.107	1.54E-56	2.5	FAM213A	2.056074766
CAB39L1	1.70E-60	0.3211451	0.279	0.149	4.10E-56	2.5	CAB39L	1.872483221
DLEU22	2.06E-60	0.4443721	0.345	0.205	4.96E-56	2.5	DLEU2	1.682926829
AC007246.31	2.06E-60	0.252863	0.109	0.037	4.97E-56	2.5	AC007246.	2.945945946
CXCL61	2.17E-60	0.5829778	0.143	0.057	5.23E-56	2.5	CXCL6	2.50877193
RPS6KA21	3.42E-60	0.300148	0.285	0.153	8.24E-56	2.5	RPS6KA2	1.862745098
ANKRD441	4.05E-60	0.253751	0.159	0.066	9.76E-56	2.5	ANKRD44	2.409090909
DMGDH1	4.96E-60	0.2662154	0.111	0.038	1.20E-55	2.5	DMGDH	2.921052632
TMEM594	8.04E-60	0.3252763	0.609	0.431	1.94E-55	2.5	TMEM59	1.412993039
STX81	1.08E-59	0.3728805	0.357	0.214	2.59E-55	2.5	STX8	1.668224299
CDON1	1.14E-59	0.2586804	0.113	0.04	2.76E-55	2.5	CDON	2.825
NAMPT2	1.48E-59	0.3812887	0.775	0.631	3.57E-55	2.5	NAMPT	1.228209192
ARSG	1.85E-59	0.2283481	0.108	0.037	4.46E-55	2.5	ARSG	2.918918919
UCP23	2.52E-59	0.1948789	0.089	0.026	6.09E-55	2.5	UCP2	3.423076923
TUBA1A3	2.62E-59	0.6267349	0.374	0.238	6.33E-55	2.5	TUBA1A	1.571428571
WHSC1L12	3.19E-59	0.3279821	0.394	0.242	7.68E-55	2.5	WHSC1L1	1.628099174
SIPA1L32	3.88E-59	0.2597471	0.341	0.196	9.36E-55	2.5	SIPA1L3	1.739795918
SLTM1	5.12E-59	0.3689356	0.431	0.276	1.23E-54	2.5	SLTM	1.561594203
STAT5B2	5.50E-59	0.3205547	0.481	0.312	1.33E-54	2.5	STAT5B	1.541666667

PGM2L13	5.94E-59	0.2901254	0.369	0.215	1.43E-54	2.5	PGM2L1	1.71627907
CHMP53	7.48E-59	0.3649955	0.585	0.416	1.80E-54	2.5	CHMP5	1.40625
LYPD32	7.56E-59	0.2253116	0.138	0.054	1.82E-54	2.5	LYPD3	2.555555556
LCOR2	8.36E-59	0.3116494	0.404	0.247	2.02E-54	2.5	LCOR	1.63562753
SLC30A91	1.16E-58	0.3333241	0.261	0.138	2.79E-54	2.5	SLC30A9	1.891304348
NNMT1	1.25E-58	0.3375478	0.202	0.095	3.02E-54	2.5	NNMT	2.126315789
PLAU1	1.92E-58	0.3277796	0.176	0.078	4.62E-54	2.5	PLAU	2.256410256
ITGB62	2.00E-58	0.3651469	0.532	0.363	4.82E-54	2.5	ITGB6	1.465564738
RP4-601P9.22	2.82E-58	0.1631553	0.052	0.01	6.80E-54	2.5	RP4-601P9	5.2
NFIC2	3.68E-58	0.3553994	0.3	0.17	8.87E-54	2.5	NFIC	1.764705882
OBFC11	4.98E-58	0.2541078	0.149	0.061	1.20E-53	2.5	OBFC1	2.442622951
USP392	7.07E-58	0.3922567	0.476	0.318	1.71E-53	2.5	USP39	1.496855346
P4HA12	9.16E-58	0.2894426	0.541	0.364	2.21E-53	2.5	P4HA1	1.486263736
NR1D21	9.46E-58	0.3435702	0.362	0.218	2.28E-53	2.5	NR1D2	1.660550459
BCAM2	9.64E-58	0.1918576	0.081	0.023	2.32E-53	2.5	BCAM	3.52173913
ZBTB163	1.03E-57	0.4340345	0.404	0.259	2.48E-53	2.5	ZBTB16	1.55984556
HPS31	1.44E-57	0.2118341	0.104	0.036	3.48E-53	2.5	HPS3	2.888888889
LYST2	1.89E-57	0.3012625	0.414	0.254	4.56E-53	2.5	LYST	1.62992126
CTC-236F12.41	2.06E-57	0.1561953	0.056	0.012	4.97E-53	2.5	CTC-236F1:	4.666666667
SP1101	2.25E-57	0.1772113	0.074	0.02	5.44E-53	2.5	SP110	3.7
BTG22	2.43E-57	0.2272903	0.133	0.052	5.85E-53	2.5	BTG2	2.557692308
PTPRF1	2.52E-57	0.2886611	0.219	0.109	6.07E-53	2.5	PTPRF	2.009174312
CFAP693	3.04E-57	0.2832423	0.198	0.093	7.34E-53	2.5	CFAP69	2.129032258
SMCHD13	3.46E-57	0.3241373	0.561	0.386	8.33E-53	2.5	SMCHD1	1.453367876
CST34	4.85E-57	0.2593346	0.141	0.057	1.17E-52	2.5	CST3	2.473684211
UBR11	4.86E-57	0.315917	0.252	0.134	1.17E-52	2.5	UBR1	1.880597015
FAM151B1	5.26E-57	0.2349602	0.139	0.056	1.27E-52	2.5	FAM151B	2.482142857
EFCAB11	5.44E-57	0.2252927	0.096	0.031	1.31E-52	2.5	EFCAB11	3.096774194
ACBD62	7.46E-57	0.2706943	0.16	0.069	1.80E-52	2.5	ACBD6	2.31884058
ERO1A2	7.49E-57	0.2956588	0.596	0.418	1.81E-52	2.5	ERO1A	1.425837321
USP253	1.05E-56	0.2850423	0.396	0.241	2.53E-52	2.5	USP25	1.643153527
RP11-434D9.11	1.86E-56	0.2417156	0.078	0.022	4.48E-52	2.5	RP11-434D	3.545454545
SARAF4	2.21E-56	0.3319684	0.497	0.333	5.32E-52	2.5	SARAF	1.492492492
STK392	2.33E-56	0.1243899	0.379	0.22	5.61E-52	2.5	STK39	1.722727273
ZNF7041	3.45E-56	0.2931811	0.276	0.149	8.32E-52	2.5	ZNF704	1.852348993
DNAH141	3.54E-56	0.2922832	0.183	0.085	8.54E-52	2.5	DNAH14	2.152941176
SPATA62	5.14E-56	0.2384318	0.125	0.048	1.24E-51	2.5	SPATA6	2.604166667
RP11-776H12.11	6.77E-56	0.1123589	0.032	0.004	1.63E-51	2.5	RP11-776H	8
MYO5C1	9.20E-56	0.2843741	0.258	0.138	2.22E-51	2.5	MYO5C	1.869565217
FLVCR21	1.03E-55	0.2302478	0.098	0.033	2.49E-51	2.5	FLVCR2	2.96969697
IQCB11	1.08E-55	0.2825306	0.216	0.107	2.61E-51	2.5	IQCB1	2.018691589
PHLDB23	1.32E-55	0.2716201	0.579	0.397	3.19E-51	2.5	PHLDB2	1.458438287
RP11-701H24.91	1.54E-55	0.2667517	0.145	0.061	3.70E-51	2.5	RP11-701H	2.37704918
VPS81	1.58E-55	0.2749375	0.225	0.114	3.82E-51	2.5	VPS8	1.973684211
KTN14	1.67E-55	0.2955472	0.693	0.522	4.02E-51	2.5	KTN1	1.327586207
PARM12	2.88E-55	0.2532419	0.145	0.06	6.96E-51	2.5	PARM1	2.416666667
CAP22	3.07E-55	0.3190136	0.217	0.11	7.40E-51	2.5	CAP2	1.972727273
PRKAR2A3	3.33E-55	0.2389409	0.276	0.152	8.02E-51	2.5	PRKAR2A	1.815789474
KITLG1	3.79E-55	0.2374623	0.108	0.039	9.14E-51	2.5	KITLG	2.769230769
UBA6-AS1	7.88E-55	0.3145639	0.211	0.104	1.90E-50	2.5	UBA6-AS1	2.028846154
CLASP22	1.05E-54	0.3320315	0.402	0.256	2.53E-50	2.5	CLASP2	1.5703125
DNMBP1	1.09E-54	0.2396268	0.144	0.06	2.62E-50	2.5	DNMBP	2.4

TIMP21	1.26E-54	0.2276935	0.137	0.056	3.05E-50	2.5	TIMP2	2.446428571
ANKIB11	1.31E-54	0.3295214	0.504	0.339	3.17E-50	2.5	ANKIB1	1.486725664
SLC16A43	1.73E-54	0.2564313	0.185	0.086	4.18E-50	2.5	SLC16A4	2.151162791
CSPP12	1.98E-54	0.2886562	0.291	0.162	4.79E-50	2.5	CSPP1	1.796296296
PPA22	2.13E-54	0.3230876	0.308	0.179	5.14E-50	2.5	PPA2	1.720670391
LPP2	5.84E-54	0.2520923	0.97	0.907	1.41E-49	2.5	LPP	1.069459757
SLC37A11	6.62E-54	0.2618807	0.164	0.074	1.60E-49	2.5	SLC37A1	2.216216216
PPP4R41	1.12E-53	0.3923495	0.173	0.08	2.69E-49	2.5	PPP4R4	2.1625
SHANK21	1.12E-53	0.3256354	0.353	0.216	2.71E-49	2.5	SHANK2	1.634259259
ZBTB383	1.14E-53	0.3045028	0.462	0.305	2.75E-49	2.5	ZBTB38	1.514754098
IL20RA2	1.51E-53	0.2550541	0.118	0.045	3.64E-49	2.5	IL20RA	2.622222222
HIST2H2BE2	1.95E-53	0.3107478	0.2	0.099	4.71E-49	2.5	HIST2H2BE	2.02020202
TXNIP2	2.09E-53	0.3583968	0.526	0.362	5.04E-49	2.5	TXNIP	1.453038674
PI4KA1	2.24E-53	0.2788402	0.215	0.109	5.40E-49	2.5	PI4KA	1.972477064
NRDC1	2.84E-53	0.3164898	0.332	0.198	6.85E-49	2.5	NRDC	1.676767677
GULP12	3.16E-53	0.3403766	0.321	0.192	7.61E-49	2.5	GULP1	1.671875
ZNF6751	3.56E-53	0.2900554	0.193	0.093	8.58E-49	2.5	ZNF675	2.075268817
USP342	4.69E-53	0.3101019	0.782	0.633	1.13E-48	2.5	USP34	1.235387046
STMN11	5.04E-53	0.3557825	0.37	0.23	1.22E-48	2.5	STMN1	1.608695652
ARHGAP351	5.88E-53	0.2617886	0.169	0.077	1.42E-48	2.5	ARHGAP35	2.194805195
RANBP93	9.63E-53	0.3148624	0.435	0.28	2.32E-48	2.5	RANBP9	1.553571429
KIAA16712	1.76E-52	0.2877836	0.311	0.181	4.26E-48	2.5	KIAA1671	1.718232044
ATP6V0E13	1.93E-52	0.2886048	0.812	0.677	4.66E-48	2.5	ATP6V0E1	1.199409158
KIAA02321	3.84E-52	0.3141077	0.308	0.18	9.25E-48	2.5	KIAA0232	1.711111111
TCP11L22	3.92E-52	0.2634951	0.192	0.094	9.45E-48	2.5	TCP11L2	2.042553191
DIO2-AS13	8.40E-52	0.2589066	0.163	0.073	2.03E-47	2.5	DIO2-AS1	2.232876712
KCNE42	1.03E-51	0.1883931	0.079	0.024	2.47E-47	2.5	KCNE4	3.291666667
SMAD13	1.04E-51	0.2708238	0.186	0.09	2.51E-47	2.5	SMAD1	2.066666667
SUCLG22	1.30E-51	0.3022324	0.232	0.123	3.14E-47	2.5	SUCLG2	1.886178862
CTD-2561J22.32	1.32E-51	0.198308	0.12	0.047	3.18E-47	2.5	CTD-2561J2	2.553191489
BRAF2	1.43E-51	0.3264675	0.558	0.401	3.44E-47	2.5	BRAF	1.391521197
NFE2L22	1.44E-51	0.288957	0.525	0.361	3.47E-47	2.5	NFE2L2	1.454293629
SNX252	1.46E-51	0.3254119	0.296	0.172	3.51E-47	2.5	SNX25	1.720930233
AC009950.21	1.51E-51	0.1430966	0.053	0.012	3.64E-47	2.5	AC009950.:	4.416666667
LNX23	1.90E-51	0.2539276	0.25	0.133	4.58E-47	2.5	LNX2	1.879699248
AKAP91	2.03E-51	0.3069486	0.543	0.382	4.91E-47	2.5	AKAP9	1.421465969
AIM12	2.07E-51	0.2718794	0.595	0.425	4.98E-47	2.5	AIM1	1.4
TOM1L22	3.24E-51	0.2766059	0.44	0.282	7.81E-47	2.5	TOM1L2	1.560283688
AK9	3.74E-51	0.2539534	0.155	0.069	9.03E-47	2.5	AK9	2.246376812
ABHD21	4.62E-51	0.2968121	0.244	0.132	1.12E-46	2.5	ABHD2	1.848484848
CAPS2	4.86E-51	0.187799	0.084	0.027	1.17E-46	2.5	CAPS2	3.111111111
AIG12	5.22E-51	0.3107574	0.323	0.191	1.26E-46	2.5	AIG1	1.691099476
TBC1D11	5.69E-51	0.3527684	0.332	0.203	1.37E-46	2.5	TBC1D1	1.63546798
KIAA19581	5.84E-51	0.2778239	0.238	0.127	1.41E-46	2.5	KIAA1958	1.874015748
FCHO21	6.92E-51	0.2989164	0.28	0.16	1.67E-46	2.5	FCHO2	1.75
PPARG2	9.16E-51	0.2913054	0.261	0.142	2.21E-46	2.5	PPARG	1.838028169
NBPF1	9.24E-51	0.2040426	0.092	0.031	2.23E-46	2.5	NBPF1	2.967741935
SLC38A92	1.24E-50	0.2451361	0.142	0.061	2.98E-46	2.5	SLC38A9	2.327868852
PRIM22	1.24E-50	0.2518387	0.173	0.082	2.99E-46	2.5	PRIM2	2.109756098
FAM188A3	1.37E-50	0.2378629	0.186	0.089	3.31E-46	2.5	FAM188A	2.08988764
ECE12	1.39E-50	0.2862143	0.301	0.172	3.34E-46	2.5	ECE1	1.75
RAB17	1.40E-50	0.1590646	0.07	0.02	3.37E-46	2.5	RAB17	3.5

DYNLT12	1.44E-50	0.3730145	0.714	0.577	3.47E-46	2.5	DYNLT1	1.237435009
BMF	2.75E-50	0.1093386	0.038	0.006	6.64E-46	2.5	BMF	6.333333333
NAIP2	3.02E-50	0.2533127	0.19	0.093	7.28E-46	2.5	NAIP	2.043010753
ADGRV12	3.04E-50	0.2825954	0.27	0.151	7.34E-46	2.5	ADGRV1	1.78807947
LMNA2	3.29E-50	0.3182357	0.35	0.216	7.93E-46	2.5	LMNA	1.62037037
PAPD42	5.12E-50	0.3217155	0.442	0.296	1.23E-45	2.5	PAPD4	1.493243243
GM2A1	5.34E-50	0.1748859	0.093	0.032	1.29E-45	2.5	GM2A	2.90625
SPICE11	6.60E-50	0.2476779	0.136	0.058	1.59E-45	2.5	SPICE1	2.344827586
CFAP44	9.43E-50	0.2105143	0.1	0.036	2.27E-45	2.5	CFAP44	2.777777778
KIAA14561	1.01E-49	0.1524079	0.066	0.018	2.44E-45	2.5	KIAA1456	3.666666667
EBF41	1.21E-49	0.2367248	0.128	0.053	2.92E-45	2.5	EBF4	2.41509434
XRN23	1.61E-49	0.3026747	0.321	0.194	3.88E-45	2.5	XRN2	1.654639175
SEPW13	1.85E-49	0.3128328	0.262	0.147	4.46E-45	2.5	SEPW1	1.782312925
CSMD22	2.46E-49	0.1686136	0.068	0.019	5.93E-45	2.5	CSMD2	3.578947368
ANKS1A2	2.90E-49	0.2969523	0.307	0.181	6.98E-45	2.5	ANKS1A	1.696132597
RPGR1	3.04E-49	0.2462462	0.168	0.079	7.32E-45	2.5	RPGR	2.126582278
SERF24	3.63E-49	0.3998651	0.531	0.376	8.76E-45	2.5	SERF2	1.412234043
RAB312	4.69E-49	0.346229	0.298	0.176	1.13E-44	2.5	RAB31	1.693181818
ZC2HC1A1	5.79E-49	0.1911947	0.102	0.038	1.40E-44	2.5	ZC2HC1A	2.684210526
PHF21A2	7.66E-49	0.3222667	0.355	0.222	1.85E-44	2.5	PHF21A	1.599099099
HDAC82	7.77E-49	0.3180708	0.364	0.228	1.87E-44	2.5	HDAC8	1.596491228
PTPRK3	9.43E-49	0.1793713	0.901	0.753	2.27E-44	2.5	PTPRK	1.196547145
PLPP11	1.07E-48	0.2891549	0.21	0.108	2.57E-44	2.5	PLPP1	1.944444444
HIST1H2BJ1	1.33E-48	0.3002742	0.224	0.119	3.21E-44	2.5	HIST1H2BJ	1.882352941
MSH32	1.84E-48	0.2445709	0.181	0.089	4.43E-44	2.5	MSH3	2.033707865
RNF2132	2.58E-48	0.3125993	0.283	0.166	6.22E-44	2.5	RNF213	1.704819277
PDZD84	3.46E-48	0.2776263	0.353	0.215	8.34E-44	2.5	PDZD8	1.641860465
GMDS-AS12	3.58E-48	0.3220246	0.195	0.1	8.63E-44	2.5	GMDS-AS1	1.95
ARL152	3.63E-48	0.3137803	0.319	0.193	8.75E-44	2.5	ARL15	1.652849741
NINJ1	3.78E-48	0.1883048	0.068	0.019	9.12E-44	2.5	NINJ1	3.578947368
TMEM1351	4.68E-48	0.2403794	0.194	0.097	1.13E-43	2.5	TMEM135	2
KIAA13282	5.12E-48	0.2919808	0.216	0.113	1.23E-43	2.5	KIAA1328	1.911504425
PLXNA22	5.14E-48	0.2212293	0.099	0.036	1.24E-43	2.5	PLXNA2	2.75
HIF1A1	8.68E-48	0.4043593	0.566	0.425	2.09E-43	2.5	HIF1A	1.331764706
FAM46C2	9.95E-48	0.1786626	0.088	0.03	2.40E-43	2.5	FAM46C	2.933333333
AMFR3	1.00E-47	0.2660185	0.201	0.102	2.41E-43	2.5	AMFR	1.970588235
EXPH51	1.01E-47	0.2972338	0.285	0.166	2.43E-43	2.5	EXPH5	1.71686747
NR6A13	1.01E-47	0.1500869	0.501	0.331	2.45E-43	2.5	NR6A1	1.513595166
SLK2	1.02E-47	0.2578791	0.357	0.219	2.45E-43	2.5	SLK	1.630136986
FBXL202	1.25E-47	0.2934314	0.447	0.299	3.00E-43	2.5	FBXL20	1.494983278
AC026188.1	1.30E-47	0.1985298	0.076	0.024	3.13E-43	2.5	AC026188.	3.166666667
ETS22	1.40E-47	0.2966014	0.294	0.171	3.37E-43	2.5	ETS2	1.719298246
IGFBP44	1.43E-47	0.1752192	0.241	0.126	3.44E-43	2.5	IGFBP4	1.912698413
SRD5A31	1.51E-47	0.2372569	0.139	0.061	3.63E-43	2.5	SRD5A3	2.278688525
TTC8	1.56E-47	0.1812787	0.089	0.031	3.75E-43	2.5	TTC8	2.870967742
MIR34AHG1	1.80E-47	0.231233	0.134	0.058	4.35E-43	2.5	MIR34AHG	2.310344828
STAM21	2.44E-47	0.2564761	0.163	0.078	5.87E-43	2.5	STAM2	2.08974359
CSF3R1	2.73E-47	0.2134814	0.087	0.03	6.59E-43	2.5	CSF3R	2.9
ZMAT3	2.85E-47	0.2155912	0.118	0.048	6.88E-43	2.5	ZMAT3	2.458333333
ZKSCAN12	3.83E-47	0.3051147	0.373	0.238	9.23E-43	2.5	ZKSCAN1	1.567226891
SPG112	3.83E-47	0.2786471	0.421	0.278	9.24E-43	2.5	SPG11	1.514388489
SCD2	4.40E-47	0.3754871	0.211	0.113	1.06E-42	2.5	SCD	1.867256637

VPS252	4.62E-47	0.2915368	0.23	0.126	1.11E-42	2.5	VPS25	1.825396825
GALNT21	4.64E-47	0.2744757	0.243	0.134	1.12E-42	2.5	GALNT2	1.813432836
HDAC4	4.73E-47	0.1870633	0.116	0.047	1.14E-42	2.5	HDAC4	2.468085106
MAN1A13	5.11E-47	0.2668611	0.346	0.212	1.23E-42	2.5	MAN1A1	1.632075472
WDR72	1.03E-46	0.2041036	0.112	0.044	2.49E-42	2.5	WDR7	2.545454545
CAMK2N12	1.09E-46	0.1813517	0.102	0.038	2.62E-42	2.5	CAMK2N1	2.684210526
CP1	1.13E-46	0.8385063	0.125	0.054	2.73E-42	2.5	CP	2.314814815
PBRM13	1.32E-46	0.268621	0.339	0.21	3.18E-42	2.5	PBRM1	1.614285714
VPS502	1.61E-46	0.2813533	0.163	0.077	3.87E-42	2.5	VPS50	2.116883117
GIGYF21	1.77E-46	0.2814343	0.419	0.277	4.26E-42	2.5	GIGYF2	1.512635379
ZFAND54	1.79E-46	0.263376	0.578	0.415	4.32E-42	2.5	ZFAND5	1.392771084
LAMA13	2.22E-46	0.3878027	0.186	0.094	5.36E-42	2.5	LAMA1	1.978723404
EFNA13	2.44E-46	0.2740541	0.359	0.223	5.89E-42	2.5	EFNA1	1.609865471
DCLK11	3.00E-46	0.3070746	0.105	0.041	7.24E-42	2.5	DCLK1	2.56097561
ZFAND33	3.12E-46	0.1873591	0.883	0.743	7.53E-42	2.5	ZFAND3	1.188425303
CEACAM61	3.14E-46	0.1741729	0.071	0.022	7.58E-42	2.5	CEACAM6	3.227272727
WDFY22	3.90E-46	0.2950697	0.386	0.25	9.40E-42	2.5	WDFY2	1.544
CARHSP12	4.62E-46	0.315842	0.39	0.256	1.12E-41	2.5	CARHSP1	1.5234375
DNAH72	5.32E-46	0.1673506	0.077	0.025	1.28E-41	2.5	DNAH7	3.08
AAK12	7.71E-46	0.2245774	0.271	0.153	1.86E-41	2.5	AAK1	1.77124183
ABHD182	7.75E-46	0.252401	0.485	0.325	1.87E-41	2.5	ABHD18	1.492307692
ZNF2082	8.97E-46	0.169514	0.096	0.035	2.16E-41	2.5	ZNF208	2.742857143
KLHL423	1.05E-45	0.2266678	0.141	0.064	2.54E-41	2.5	KLHL42	2.203125
FGFR1OP2	1.15E-45	0.2014518	0.118	0.048	2.77E-41	2.5	FGFR1OP	2.458333333
B3GLCT1	1.40E-45	0.2164726	0.138	0.061	3.37E-41	2.5	B3GLCT	2.262295082
INTS81	1.55E-45	0.2081901	0.106	0.042	3.73E-41	2.5	INTS8	2.523809524
FOXO31	1.81E-45	0.341634	0.493	0.344	4.37E-41	2.5	FOXO3	1.433139535
MTX22	2.24E-45	0.2391466	0.157	0.075	5.41E-41	2.5	MTX2	2.093333333
LRP111	2.55E-45	0.2610124	0.164	0.079	6.14E-41	2.5	LRP11	2.075949367
TAPBP2	3.38E-45	0.2962886	0.235	0.131	8.14E-41	2.5	TAPBP	1.79389313
AMN11	3.43E-45	0.2721088	0.253	0.144	8.26E-41	2.5	AMN1	1.756944444
FNDC3B2	3.48E-45	0.2778688	0.881	0.757	8.38E-41	2.5	FNDC3B	1.163804491
MRPS101	5.55E-45	0.2019546	0.131	0.057	1.34E-40	2.5	MRPS10	2.298245614
ZBTB13	6.44E-45	0.2661685	0.18	0.092	1.55E-40	2.5	ZBTB1	1.956521739
GPCPD11	6.60E-45	0.3092987	0.244	0.139	1.59E-40	2.5	GPCPD1	1.755395683
ZNRF12	6.74E-45	0.2668612	0.268	0.154	1.63E-40	2.5	ZNRF1	1.74025974
SECISBP23	7.21E-45	0.234733	0.178	0.089	1.74E-40	2.5	SECISBP2	2
RPH3AL1	9.00E-45	0.133113	0.057	0.015	2.17E-40	2.5	RPH3AL	3.8
ZFYVE162	9.47E-45	0.2544032	0.186	0.095	2.28E-40	2.5	ZFYVE16	1.957894737
AC007682.11	1.08E-44	0.2475816	0.062	0.018	2.60E-40	2.5	AC007682.	3.444444444
BTG13	1.15E-44	0.3011827	0.709	0.564	2.77E-40	2.5	BTG1	1.257092199
MICALCL2	1.18E-44	0.1840713	0.092	0.034	2.84E-40	2.5	MICALCL	2.705882353
CD1644	1.19E-44	0.246774	0.408	0.265	2.87E-40	2.5	CD164	1.539622642
SIK21	1.31E-44	0.3778898	0.448	0.308	3.17E-40	2.5	SIK2	1.454545455
HSPH14	1.68E-44	0.1228141	0.421	0.266	4.06E-40	2.5	HSPH1	1.582706767
ADAR1	1.71E-44	0.2636544	0.212	0.115	4.13E-40	2.5	ADAR	1.843478261
TMCC12	1.89E-44	0.2981279	0.364	0.238	4.57E-40	2.5	TMCC1	1.529411765
SNX101	1.98E-44	0.2364655	0.136	0.061	4.78E-40	2.5	SNX10	2.229508197
RPRD22	2.75E-44	0.2846003	0.24	0.136	6.64E-40	2.5	RPRD2	1.764705882
EFHC12	4.09E-44	0.2010094	0.134	0.059	9.86E-40	2.5	EFHC1	2.271186441
TDRD31	4.13E-44	0.2478959	0.185	0.095	9.95E-40	2.5	TDRD3	1.947368421
OPN33	4.28E-44	0.2069943	0.172	0.085	1.03E-39	2.5	OPN3	2.023529412

PDS5B1	4.37E-44	0.2690324	0.474	0.328	1.05E-39	2.5	PDS5B	1.445121951
CASK3	4.85E-44	0.2505246	0.51	0.353	1.17E-39	2.5	CASK	1.444759207
TMEM2411	5.55E-44	0.2491856	0.161	0.078	1.34E-39	2.5	TMEM241	2.064102564
RPS27L3	5.91E-44	0.4590448	0.64	0.5	1.42E-39	2.5	RPS27L	1.28
RCN14	6.40E-44	0.2514324	0.249	0.141	1.54E-39	2.5	RCN1	1.765957447
ZNF1072	7.90E-44	0.2693807	0.166	0.083	1.90E-39	2.5	ZNF107	2
TMEM1401	1.05E-43	0.1047379	0.049	0.012	2.53E-39	2.5	TMEM140	4.083333333
MCPH12	1.06E-43	0.2924296	0.273	0.162	2.56E-39	2.5	MCPH1	1.685185185
RNASE41	1.10E-43	0.2608397	0.186	0.096	2.66E-39	2.5	RNASE4	1.9375
CORO2A1	1.15E-43	0.2265819	0.123	0.053	2.78E-39	2.5	CORO2A	2.320754717
GSTCD3	2.12E-43	0.1870889	0.192	0.099	5.11E-39	2.5	GSTCD	1.939393939
NFATC32	2.23E-43	0.2541601	0.269	0.158	5.37E-39	2.5	NFATC3	1.702531646
DDB21	2.37E-43	0.1862965	0.086	0.031	5.72E-39	2.5	DDB2	2.774193548
RBX11	2.95E-43	0.3056223	0.565	0.421	7.11E-39	2.5	RBX1	1.342042755
EXOC6B2	2.95E-43	0.2264642	0.396	0.256	7.12E-39	2.5	EXOC6B	1.546875
S100P2	3.48E-43	0.3020892	0.111	0.046	8.38E-39	2.5	S100P	2.413043478
CTD-2337A12.11	3.95E-43	0.2924538	0.304	0.185	9.52E-39	2.5	CTD-2337A	1.643243243
STAT11	4.10E-43	0.2461251	0.145	0.068	9.90E-39	2.5	STAT1	2.132352941
KDM4B2	4.54E-43	0.2211936	0.161	0.078	1.09E-38	2.5	KDM4B	2.064102564
RBM473	5.56E-43	0.2598955	0.774	0.617	1.34E-38	2.5	RBM47	1.25445705
DUSP53	6.62E-43	0.1595316	0.36	0.222	1.60E-38	2.5	DUSP5	1.621621622
TNRC6C1	6.89E-43	0.1820188	0.094	0.035	1.66E-38	2.5	TNRC6C	2.685714286
BCL2L16	7.33E-43	0.2924476	0.256	0.149	1.77E-38	2.5	BCL2L1	1.718120805
LDLRAD43	7.74E-43	0.2777631	0.267	0.157	1.87E-38	2.5	LDLRAD4	1.700636943
ZNF4401	8.19E-43	0.2028151	0.104	0.042	1.97E-38	2.5	ZNF440	2.476190476
NSF2	9.12E-43	0.2460689	0.243	0.138	2.20E-38	2.5	NSF	1.760869565
RFK2	9.38E-43	0.1640733	0.101	0.039	2.26E-38	2.5	RFK	2.58974359
REEP54	1.10E-42	0.2562899	0.37	0.237	2.65E-38	2.5	REEP5	1.561181435
ZDHHC202	1.20E-42	0.2577522	0.276	0.164	2.88E-38	2.5	ZDHHC20	1.682926829
SIPA1L22	1.42E-42	0.3259614	0.227	0.127	3.43E-38	2.5	SIPA1L2	1.787401575
AFF12	1.43E-42	0.2640107	0.505	0.356	3.45E-38	2.5	AFF1	1.418539326
FDPS3	1.61E-42	0.4776936	0.573	0.437	3.87E-38	2.5	FDPS	1.311212815
SLX4IP2	2.74E-42	0.2013753	0.116	0.049	6.62E-38	2.5	SLX4IP	2.367346939
DDI22	3.06E-42	0.2481623	0.172	0.087	7.38E-38	2.5	DDI2	1.977011494
DHCR243	3.30E-42	0.3138179	0.286	0.175	7.95E-38	2.5	DHCR24	1.634285714
SLC41A22	4.02E-42	0.2315384	0.204	0.108	9.69E-38	2.5	SLC41A2	1.888888889
ST3GAL13	4.06E-42	0.2970493	0.3	0.188	9.78E-38	2.5	ST3GAL1	1.595744681
CCPG11	4.95E-42	0.219416	0.173	0.087	1.19E-37	2.5	CCPG1	1.988505747
NDUFV23	5.88E-42	0.2198593	0.395	0.254	1.42E-37	2.5	NDUFV2	1.55511811
STIM13	8.16E-42	0.2991398	0.34	0.218	1.97E-37	2.5	STIM1	1.559633028
PPP1R211	1.05E-41	0.2626602	0.198	0.107	2.52E-37	2.5	PPP1R21	1.85046729
SLC35A33	1.10E-41	0.2168683	0.167	0.084	2.66E-37	2.5	SLC35A3	1.988095238
ACACA1	1.13E-41	0.2926579	0.253	0.148	2.72E-37	2.5	ACACA	1.709459459
LOXL21	1.47E-41	0.2070079	0.082	0.029	3.55E-37	2.5	LOXL2	2.827586207
ZZEF12	1.78E-41	0.2312111	0.179	0.092	4.29E-37	2.5	ZZEF1	1.945652174
BPTF3	2.19E-41	0.2757215	0.456	0.32	5.28E-37	2.5	BPTF	1.425
LASP12	2.35E-41	0.2407367	0.225	0.125	5.66E-37	2.5	LASP1	1.8
INADL2	2.75E-41	0.220884	0.927	0.805	6.63E-37	2.5	INADL	1.151552795
ANO62	4.19E-41	0.1751117	0.674	0.498	1.01E-36	2.5	ANO6	1.353413655
ZNF6382	4.22E-41	0.2657546	0.574	0.425	1.02E-36	2.5	ZNF638	1.350588235
LINC012072	5.03E-41	0.1483866	0.08	0.028	1.21E-36	2.5	LINC01207	2.857142857
SLMAP3	5.56E-41	0.2646269	0.799	0.656	1.34E-36	2.5	SLMAP	1.217987805

FARS22	6.18E-41	0.2118049	0.154	0.075	1.49E-36	2.5	FARS2	2.053333333
IMMP2L2	6.47E-41	0.3367092	0.406	0.279	1.56E-36	2.5	IMMP2L	1.455197133
RHBDD12	6.60E-41	0.2728267	0.225	0.128	1.59E-36	2.5	RHBDD1	1.7578125
SDC31	7.56E-41	0.1669387	0.076	0.026	1.82E-36	2.5	SDC3	2.923076923
PHC21	7.77E-41	0.2247064	0.19	0.101	1.87E-36	2.5	PHC2	1.881188119
TOM1L12	7.81E-41	0.2656646	0.38	0.25	1.88E-36	2.5	TOM1L1	1.52
FAM120B2	8.95E-41	0.2266396	0.201	0.109	2.16E-36	2.5	FAM120B	1.844036697
CTD-2047H16.41	1.17E-40	0.1767423	0.099	0.039	2.82E-36	2.5	CTD-2047H	2.538461538
VPS13A1	1.54E-40	0.2348352	0.245	0.141	3.70E-36	2.5	VPS13A	1.737588652
DCDC22	1.79E-40	0.1543278	0.062	0.019	4.33E-36	2.5	DCDC2	3.263157895
SLC1A41	2.07E-40	0.1643863	0.085	0.032	5.00E-36	2.5	SLC1A4	2.65625
CSNK1A13	2.21E-40	0.2429934	0.772	0.639	5.32E-36	2.5	CSNK1A1	1.208137715
KLHL181	2.36E-40	0.2291629	0.147	0.071	5.70E-36	2.5	KLHL18	2.070422535
ANKRD491	2.53E-40	0.2005906	0.129	0.059	6.10E-36	2.5	ANKRD49	2.186440678
IBTK3	2.81E-40	0.1657028	0.333	0.206	6.79E-36	2.5	IBTK	1.616504854
ATP9B1	2.92E-40	0.2281515	0.152	0.075	7.05E-36	2.5	ATP9B	2.026666667
TBCK2	3.90E-40	0.2565958	0.202	0.111	9.41E-36	2.5	TBCK	1.81981982
KIAA20262	3.91E-40	0.2546432	0.29	0.177	9.44E-36	2.5	KIAA2026	1.638418079
AZIN1-AS11	4.75E-40	0.2138751	0.107	0.045	1.15E-35	2.5	AZIN1-AS1	2.377777778
SNTB1	5.18E-40	0.2631628	0.136	0.064	1.25E-35	2.5	SNTB1	2.125
OTUD14	5.18E-40	0.1809377	0.107	0.045	1.25E-35	2.5	OTUD1	2.377777778
SHROOM33	5.37E-40	0.2433711	0.756	0.614	1.30E-35	2.5	SHROOM3	1.231270358
RP11-507B12.23	5.67E-40	0.2326729	0.178	0.091	1.37E-35	2.5	RP11-507B	1.956043956
RBM63	6.02E-40	0.2972355	0.457	0.324	1.45E-35	2.5	RBM6	1.410493827
CRABP21	6.08E-40	0.255853	0.204	0.111	1.47E-35	2.5	CRABP2	1.837837838
BTBD91	6.94E-40	0.2686666	0.372	0.245	1.67E-35	2.5	BTBD9	1.518367347
SSBP32	7.22E-40	0.2422416	0.208	0.114	1.74E-35	2.5	SSBP3	1.824561404
BAG14	9.02E-40	0.2010136	0.174	0.089	2.18E-35	2.5	BAG1	1.95505618
NKAIN31	9.12E-40	0.2047659	0.091	0.035	2.20E-35	2.5	NKAIN3	2.6
TTC39B2	9.96E-40	0.2688129	0.161	0.083	2.40E-35	2.5	TTC39B	1.939759036
BMPR22	1.02E-39	0.2798827	0.378	0.253	2.47E-35	2.5	BMPR2	1.494071146
SNRK1	1.22E-39	0.1998979	0.128	0.058	2.95E-35	2.5	SNRK	2.206896552
PAK11	1.22E-39	0.2565214	0.332	0.212	2.95E-35	2.5	PAK1	1.566037736
SIPA1L13	1.25E-39	0.2376356	0.514	0.367	3.02E-35	2.5	SIPA1L1	1.400544959
FYCO11	1.29E-39	0.1689946	0.098	0.039	3.12E-35	2.5	FYCO1	2.512820513
LPGAT11	1.29E-39	0.2390417	0.157	0.078	3.12E-35	2.5	LPGAT1	2.012820513
CDKAL13	1.43E-39	0.2539282	0.348	0.225	3.45E-35	2.5	CDKAL1	1.546666667
CYP1B11	1.63E-39	0.5310119	0.189	0.105	3.92E-35	2.5	CYP1B1	1.8
ADIRF4	2.28E-39	0.3241533	0.129	0.06	5.50E-35	2.5	ADIRF	2.15
RP11-486O13.42	2.34E-39	0.2542886	0.178	0.094	5.64E-35	2.5	RP11-486O	1.893617021
ARFGEF22	2.67E-39	0.2684264	0.493	0.356	6.43E-35	2.5	ARFGEF2	1.384831461
PRKD13	2.83E-39	0.2406333	0.291	0.178	6.82E-35	2.5	PRKD1	1.634831461
SYTL42	4.49E-39	0.2166477	0.21	0.115	1.08E-34	2.5	SYTL4	1.826086957
APOL11	5.02E-39	0.1751848	0.078	0.028	1.21E-34	2.5	APOL1	2.785714286
ZNF191	6.09E-39	0.1549709	0.066	0.022	1.47E-34	2.5	ZNF19	3
IGF1R3	7.75E-39	0.2080744	0.644	0.484	1.87E-34	2.5	IGF1R	1.330578512
HIPK24	8.03E-39	0.2600616	0.5	0.356	1.94E-34	2.5	HIPK2	1.404494382
NAT11	1.07E-38	0.1141821	0.051	0.014	2.59E-34	2.5	NAT1	3.642857143
MOCS11	1.10E-38	0.1243608	0.056	0.016	2.66E-34	2.5	MOCS1	3.5
TBC1D302	1.33E-38	0.1814307	0.085	0.032	3.21E-34	2.5	TBC1D30	2.65625
TAF31	1.36E-38	0.2163487	0.157	0.079	3.28E-34	2.5	TAF3	1.987341772
ANKRD124	1.42E-38	0.1780281	0.794	0.656	3.43E-34	2.5	ANKRD12	1.210365854

WDR111	1.48E-38	0.2226442	0.153	0.077	3.57E-34	2.5	WDR11	1.987012987
ME31	1.68E-38	0.183928	0.1	0.041	4.04E-34	2.5	ME3	2.43902439
DIS3L22	2.36E-38	0.232669	0.167	0.087	5.69E-34	2.5	DIS3L2	1.91954023
RREB13	2.60E-38	0.3074858	0.37	0.25	6.26E-34	2.5	RREB1	1.48
TRAF3IP11	3.09E-38	0.1776676	0.098	0.04	7.46E-34	2.5	TRAF3IP1	2.45
SMC1A3	3.43E-38	0.2265255	0.179	0.095	8.28E-34	2.5	SMC1A	1.884210526
KIAA1211	3.52E-38	0.1296846	0.048	0.013	8.50E-34	2.5	KIAA1211	3.692307692
SETD72	4.03E-38	0.2288044	0.21	0.117	9.71E-34	2.5	SETD7	1.794871795
KLF102	5.03E-38	0.2502295	0.227	0.132	1.21E-33	2.5	KLF10	1.71969697
STX183	5.71E-38	0.2265468	0.27	0.162	1.38E-33	2.5	STX18	1.666666667
S100A144	6.49E-38	0.2161573	0.852	0.718	1.56E-33	2.5	S100A14	1.186629526
SDK12	7.13E-38	0.2433141	0.238	0.138	1.72E-33	2.5	SDK1	1.724637681
LINC006571	7.26E-38	0.2268755	0.276	0.169	1.75E-33	2.5	LINC00657	1.633136095
PRRG41	9.08E-38	0.292999	0.37	0.25	2.19E-33	2.5	PRRG4	1.48
NT5DC3	9.26E-38	0.1865417	0.091	0.036	2.23E-33	2.5	NT5DC3	2.527777778
FAM114A13	9.31E-38	0.2476551	0.246	0.147	2.24E-33	2.5	FAM114A1	1.673469388
RGS121	1.08E-37	0.1949896	0.125	0.058	2.60E-33	2.5	RGS12	2.155172414
ARIH13	1.16E-37	0.2589048	0.634	0.491	2.79E-33	2.5	ARIH1	1.291242363
LINC009701	1.17E-37	0.1735739	0.081	0.031	2.83E-33	2.5	LINC00970	2.612903226
PGR3	1.22E-37	0.1838615	0.15	0.073	2.93E-33	2.5	PGR	2.054794521
DOCK13	1.24E-37	0.2523957	0.404	0.276	2.99E-33	2.5	DOCK1	1.463768116
RP11-371F15.33	1.42E-37	0.1932175	0.154	0.077	3.43E-33	2.5	RP11-371F:	2
GPRC5C1	1.56E-37	0.1399184	0.062	0.02	3.77E-33	2.5	GPRC5C	3.1
IL6ST3	1.73E-37	0.2358956	0.454	0.317	4.18E-33	2.5	IL6ST	1.432176656
TRIM442	1.88E-37	0.2350333	0.25	0.15	4.52E-33	2.5	TRIM44	1.666666667
DUSP15	2.17E-37	0.2341067	0.305	0.193	5.24E-33	2.5	DUSP1	1.580310881
LPCAT32	2.40E-37	0.1936467	0.127	0.059	5.79E-33	2.5	LPCAT3	2.152542373
CREBRF1	2.40E-37	0.2621738	0.372	0.251	5.80E-33	2.5	CREBRF	1.482071713
PKHD11	2.48E-37	0.1834268	0.099	0.041	5.97E-33	2.5	PKHD1	2.414634146
TTC142	2.58E-37	0.2048983	0.133	0.064	6.23E-33	2.5	TTC14	2.078125
NARS22	3.26E-37	0.1996127	0.127	0.06	7.85E-33	2.5	NARS2	2.116666667
SNX272	3.29E-37	0.2316521	0.241	0.142	7.92E-33	2.5	SNX27	1.697183099
APP2	4.27E-37	0.1857227	0.885	0.759	1.03E-32	2.5	APP	1.166007905
HK11	4.34E-37	0.2262703	0.222	0.128	1.05E-32	2.5	HK1	1.734375
CEP2902	4.74E-37	0.2145325	0.134	0.065	1.14E-32	2.5	CEP290	2.061538462
MLF12	4.75E-37	0.2701199	0.352	0.232	1.14E-32	2.5	MLF1	1.517241379
NUDT121	5.20E-37	0.147965	0.087	0.034	1.25E-32	2.5	NUDT12	2.558823529
TBRG11	5.37E-37	0.1997122	0.177	0.094	1.30E-32	2.5	TBRG1	1.882978723
Sep-92	5.55E-37	0.2106082	0.215	0.122	1.34E-32	2.5	Sep-09	1.762295082
R3HDM12	5.70E-37	0.2057926	0.12	0.055	1.37E-32	2.5	R3HDM1	2.181818182
STON23	6.49E-37	0.1445892	0.1	0.042	1.57E-32	2.5	STON2	2.380952381
UBE2L61	8.82E-37	0.2173722	0.145	0.073	2.13E-32	2.5	UBE2L6	1.98630137
PATZ11	8.87E-37	0.1544089	0.076	0.028	2.14E-32	2.5	PATZ1	2.714285714
SMAD33	1.10E-36	0.2182712	0.386	0.256	2.66E-32	2.5	SMAD3	1.5078125
KCNC23	1.11E-36	0.1916301	0.079	0.03	2.68E-32	2.5	KCNC2	2.633333333
ACAP22	1.12E-36	0.2335937	0.278	0.171	2.70E-32	2.5	ACAP2	1.625730994
LAMA51	1.12E-36	0.1172225	0.045	0.012	2.70E-32	2.5	LAMA5	3.75
ASAH14	1.26E-36	0.1830586	0.175	0.092	3.04E-32	2.5	ASAH1	1.902173913
ZDHHC211	1.52E-36	0.1814956	0.126	0.059	3.67E-32	2.5	ZDHHC21	2.13559322
PINK12	1.53E-36	0.1833399	0.118	0.054	3.69E-32	2.5	PINK1	2.185185185
TBC1D21	2.52E-36	0.1321672	0.051	0.015	6.07E-32	2.5	TBC1D2	3.4
EHMT11	2.71E-36	0.2276662	0.296	0.186	6.52E-32	2.5	EHMT1	1.591397849

MTHFR1	3.29E-36	0.1435685	0.068	0.024	7.92E-32	2.5	MTHFR	2.833333333
EIF4E31	3.29E-36	0.1637383	0.099	0.042	7.94E-32	2.5	EIF4E3	2.357142857
C10orf762	5.11E-36	0.2517007	0.21	0.122	1.23E-31	2.5	C10orf76	1.721311475
RP11-206M11.71	7.03E-36	0.1611698	0.146	0.074	1.70E-31	2.5	RP11-206M	1.972972973
NFE2L11	8.03E-36	0.2240548	0.158	0.083	1.94E-31	2.5	NFE2L1	1.903614458
APLP24	9.11E-36	0.2098381	0.393	0.262	2.20E-31	2.5	APLP2	1.5
GDE13	9.48E-36	0.2300254	0.228	0.134	2.29E-31	2.5	GDE1	1.701492537
FBXL33	1.02E-35	0.1971893	0.178	0.096	2.45E-31	2.5	FBXL3	1.854166667
CDK121	1.14E-35	0.2133285	0.193	0.108	2.76E-31	2.5	CDK12	1.787037037
POF1B3	1.18E-35	0.1032637	0.045	0.012	2.84E-31	2.5	POF1B	3.75
SSH22	1.33E-35	0.2452578	0.514	0.374	3.21E-31	2.5	SSH2	1.374331551
AZIN14	1.55E-35	0.2271896	0.303	0.192	3.74E-31	2.5	AZIN1	1.578125
PTPRA1	1.70E-35	0.2385986	0.273	0.17	4.11E-31	2.5	PTPRA	1.605882353
LDAH1	1.96E-35	0.144838	0.077	0.029	4.72E-31	2.5	LDAH	2.655172414
GATA31	2.09E-35	0.2857847	0.286	0.181	5.04E-31	2.5	GATA3	1.580110497
SORT12	2.13E-35	0.1962222	0.229	0.134	5.14E-31	2.5	SORT1	1.708955224
NBPF121	2.23E-35	0.1939474	0.154	0.079	5.37E-31	2.5	NBPF12	1.949367089
PRR133	2.25E-35	0.2097059	0.201	0.113	5.42E-31	2.5	PRR13	1.778761062
TMEM1643	2.28E-35	0.2137895	0.132	0.064	5.49E-31	2.5	TMEM164	2.0625
GALNT73	2.30E-35	0.2225109	0.249	0.15	5.56E-31	2.5	GALNT7	1.66
CEP1622	2.38E-35	0.1754526	0.104	0.046	5.73E-31	2.5	CEP162	2.260869565
PCNA	2.65E-35	0.1270916	0.065	0.022	6.38E-31	2.5	PCNA	2.954545455
VTCN13	3.34E-35	0.2647104	0.181	0.099	8.06E-31	2.5	VTCN1	1.828282828
RBM231	3.42E-35	0.2139405	0.167	0.089	8.25E-31	2.5	RBM23	1.876404494
GAN2	3.55E-35	0.2337095	0.287	0.18	8.56E-31	2.5	GAN	1.594444444
KMO	3.57E-35	0.149525	0.063	0.022	8.62E-31	2.5	KMO	2.863636364
KIAA12172	4.40E-35	0.1245249	0.922	0.784	1.06E-30	2.5	KIAA1217	1.176020408
ANXA52	4.55E-35	0.2079939	0.491	0.348	1.10E-30	2.5	ANXA5	1.41091954
TINCR1	5.30E-35	0.1285877	0.051	0.015	1.28E-30	2.5	TINCR	3.4
NLK	5.60E-35	0.2072192	0.196	0.11	1.35E-30	2.5	NLK	1.781818182
HP1BP32	5.70E-35	0.2626383	0.309	0.203	1.37E-30	2.5	HP1BP3	1.522167488
NUP1601	5.91E-35	0.2311843	0.214	0.124	1.42E-30	2.5	NUP160	1.725806452
C14orf1192	6.65E-35	0.2493449	0.292	0.188	1.60E-30	2.5	C14orf119	1.553191489
TSPAN32	6.98E-35	0.1602772	0.136	0.067	1.68E-30	2.5	TSPAN3	2.029850746
PARN2	8.15E-35	0.2117162	0.146	0.075	1.97E-30	2.5	PARN	1.946666667
PTPRR2	8.35E-35	0.1781593	0.086	0.035	2.01E-30	2.5	PTPRR	2.457142857
ERBB23	9.07E-35	0.198117	0.193	0.108	2.19E-30	2.5	ERBB2	1.787037037
SYT171	1.03E-34	0.1766199	0.114	0.052	2.48E-30	2.5	SYT17	2.192307692
CD1511	1.12E-34	0.2393521	0.096	0.041	2.70E-30	2.5	CD151	2.341463415
GNAQ3	1.13E-34	0.231643	0.419	0.296	2.73E-30	2.5	GNAQ	1.415540541
NUPR11	1.14E-34	0.3095126	0.252	0.155	2.75E-30	2.5	NUPR1	1.625806452
GDF151	1.20E-34	0.1790971	0.076	0.029	2.88E-30	2.5	GDF15	2.620689655
FRYL2	1.27E-34	0.1805349	0.525	0.38	3.06E-30	2.5	FRYL	1.381578947
ISCU1	1.46E-34	0.1732068	0.116	0.054	3.52E-30	2.5	ISCU	2.148148148
DNAJC152	1.62E-34	0.207621	0.127	0.062	3.90E-30	2.5	DNAJC15	2.048387097
RAB11FIP42	1.62E-34	0.1480183	0.09	0.037	3.90E-30	2.5	RAB11FIP4	2.432432432
FAM83E1	1.76E-34	0.1483913	0.062	0.021	4.25E-30	2.5	FAM83E	2.952380952
CLNS1A2	2.69E-34	0.213182	0.157	0.083	6.49E-30	2.5	CLNS1A	1.891566265
ZNF2543	3.31E-34	0.2347489	0.245	0.15	7.98E-30	2.5	ZNF254	1.633333333
TTBK23	3.45E-34	0.2412508	0.243	0.147	8.32E-30	2.5	TTBK2	1.653061224
CPPED11	3.70E-34	0.1416355	0.063	0.022	8.93E-30	2.5	CPPED1	2.863636364
TSPAN154	3.91E-34	0.1687931	0.176	0.094	9.42E-30	2.5	TSPAN15	1.872340426

NAALADL2-AS21	4.16E-34	0.111935	0.049	0.014	1.00E-29	2.5	NAALADL2-	3.5
TRIB12	4.81E-34	0.2207092	0.321	0.21	1.16E-29	2.5	TRIB1	1.528571429
RRP12	5.39E-34	0.2284943	0.151	0.079	1.30E-29	2.5	RRP12	1.911392405
ATP2B12	5.56E-34	0.1736569	0.16	0.084	1.34E-29	2.5	ATP2B1	1.904761905
ADNP1	6.46E-34	0.2333691	0.479	0.346	1.56E-29	2.5	ADNP	1.384393064
ALDH3A22	6.74E-34	0.1517906	0.092	0.039	1.62E-29	2.5	ALDH3A2	2.358974359
SCAF111	7.40E-34	0.2700263	0.412	0.293	1.78E-29	2.5	SCAF11	1.406143345
SPPL2A2	7.83E-34	0.2646599	0.366	0.25	1.89E-29	2.5	SPPL2A	1.464
PEBP13	7.93E-34	0.2328538	0.261	0.161	1.91E-29	2.5	PEBP1	1.621118012
RP11-455P21.31	8.12E-34	0.1554634	0.073	0.028	1.96E-29	2.5	RP11-455P.	2.607142857
ARL34	8.38E-34	0.217377	0.366	0.244	2.02E-29	2.5	ARL3	1.5
RRM2B1	8.40E-34	0.1491013	0.084	0.034	2.03E-29	2.5	RRM2B	2.470588235
HNRNPF2	9.39E-34	0.2283278	0.243	0.149	2.26E-29	2.5	HNRNPF	1.630872483
SMG61	9.46E-34	0.2175781	0.273	0.172	2.28E-29	2.5	SMG6	1.587209302
CAPN23	9.49E-34	0.1959584	0.405	0.277	2.29E-29	2.5	CAPN2	1.462093863
ZMYM22	9.89E-34	0.2279092	0.479	0.348	2.38E-29	2.5	ZMYM2	1.376436782
CCDC301	1.06E-33	0.1568089	0.077	0.03	2.56E-29	2.5	CCDC30	2.566666667
FAF12	1.19E-33	0.2453215	0.326	0.219	2.88E-29	2.5	FAF1	1.488584475
ALDH1A21	1.23E-33	0.1709908	0.096	0.042	2.95E-29	2.5	ALDH1A2	2.285714286
HOMER14	1.27E-33	0.1659409	0.19	0.105	3.07E-29	2.5	HOMER1	1.80952381
KIAA15222	1.34E-33	0.2386435	0.326	0.216	3.24E-29	2.5	KIAA1522	1.509259259
CASP42	1.36E-33	0.2596719	0.335	0.226	3.28E-29	2.5	CASP4	1.482300885
ATP6AP24	1.36E-33	0.1974648	0.226	0.133	3.29E-29	2.5	ATP6AP2	1.69924812
GTDC11	1.42E-33	0.2156327	0.175	0.096	3.41E-29	2.5	GTDC1	1.822916667
ABHD52	1.47E-33	0.2222564	0.31	0.201	3.54E-29	2.5	ABHD5	1.542288557
YBX31	1.75E-33	0.2643805	0.388	0.273	4.22E-29	2.5	YBX3	1.421245421
RHOBTB11	1.77E-33	0.1341607	0.083	0.034	4.26E-29	2.5	RHOBTB1	2.441176471
TACC22	2.16E-33	0.250122	0.244	0.152	5.20E-29	2.5	TACC2	1.605263158
ANKFN1	2.35E-33	0.1275153	0.042	0.011	5.67E-29	2.5	ANKFN1	3.818181818
CDK82	2.62E-33	0.2656914	0.339	0.228	6.33E-29	2.5	CDK8	1.486842105
CAB393	2.70E-33	0.2664916	0.472	0.347	6.52E-29	2.5	CAB39	1.360230548
PLIN32	2.88E-33	0.2262124	0.241	0.147	6.96E-29	2.5	PLIN3	1.639455782
SUSD63	2.97E-33	0.2459694	0.386	0.265	7.17E-29	2.5	SUSD6	1.456603774
SLC12A61	3.23E-33	0.1628575	0.092	0.039	7.79E-29	2.5	SLC12A6	2.358974359
MED13L2	3.26E-33	0.2054678	0.8	0.673	7.86E-29	2.5	MED13L	1.188707281
EZR3	3.37E-33	0.2500582	0.494	0.362	8.14E-29	2.5	EZR	1.364640884
DHRS72	3.66E-33	0.2036064	0.156	0.083	8.81E-29	2.5	DHRS7	1.879518072
GPATCH21	3.67E-33	0.1894475	0.17	0.092	8.86E-29	2.5	GPATCH2	1.847826087
KIF3C1	3.96E-33	0.1637751	0.08	0.032	9.55E-29	2.5	KIF3C	2.5
GTPBP101	4.63E-33	0.2074351	0.193	0.11	1.12E-28	2.5	GTPBP10	1.754545455
RAP1GDS13	4.75E-33	0.2518302	0.2	0.116	1.15E-28	2.5	RAP1GDS1	1.724137931
INHBB1	5.08E-33	0.1855469	0.082	0.034	1.22E-28	2.5	INHBB	2.411764706
PBX32	5.23E-33	0.1978512	0.154	0.081	1.26E-28	2.5	PBX3	1.901234568
CBFA2T21	5.41E-33	0.2454357	0.252	0.157	1.30E-28	2.5	CBFA2T2	1.605095541
C1orf2281	5.41E-33	0.2633371	0.092	0.04	1.30E-28	2.5	C1orf228	2.3
CDC25B2	5.53E-33	0.1397696	0.072	0.028	1.33E-28	2.5	CDC25B	2.571428571
IKBKB2	5.76E-33	0.190625	0.251	0.154	1.39E-28	2.5	IKBKB	1.62987013
CEP1922	6.08E-33	0.1959575	0.186	0.105	1.47E-28	2.5	CEP192	1.771428571
PTPN42	6.08E-33	0.2083442	0.148	0.078	1.47E-28	2.5	PTPN4	1.897435897
FANCI1	6.82E-33	0.1455556	0.072	0.028	1.64E-28	2.5	FANCI	2.571428571
ATRX2	8.52E-33	0.2125763	0.603	0.465	2.05E-28	2.5	ATRX	1.296774194
CEP1282	1.34E-32	0.1797025	0.116	0.055	3.24E-28	2.5	CEP128	2.109090909

MMS22L1	1.58E-32	0.1354224	0.066	0.024	3.80E-28	2.5	MMS22L	2.75
SNX132	2.06E-32	0.1923864	0.184	0.104	4.96E-28	2.5	SNX13	1.769230769
RP11-16D22.21	2.15E-32	0.1607422	0.068	0.025	5.18E-28	2.5	RP11-16D2	2.72
TAOK33	2.25E-32	0.2298005	0.587	0.458	5.42E-28	2.5	TAOK3	1.281659389
ZZZ32	2.31E-32	0.2118621	0.256	0.161	5.58E-28	2.5	ZZZ3	1.590062112
PDLIM11	2.54E-32	0.2051824	0.194	0.111	6.12E-28	2.5	PDLIM1	1.747747748
AC097721.22	2.58E-32	0.1505566	0.068	0.025	6.22E-28	2.5	AC097721.:	2.72
CKAP41	2.59E-32	0.2202306	0.297	0.194	6.24E-28	2.5	CKAP4	1.530927835
TRAK11	2.88E-32	0.2272188	0.287	0.185	6.94E-28	2.5	TRAK1	1.551351351
XKR62	3.13E-32	0.2284506	0.237	0.146	7.55E-28	2.5	XKR6	1.623287671
TTC173	3.25E-32	0.2434216	0.399	0.282	7.84E-28	2.5	TTC17	1.414893617
TIPARP1	3.28E-32	0.2379334	0.194	0.112	7.91E-28	2.5	TIPARP	1.732142857
COMMD102	3.64E-32	0.214905	0.293	0.19	8.77E-28	2.5	COMMD10	1.542105263
DIXDC14	4.14E-32	0.2133638	0.188	0.107	9.98E-28	2.5	DIXDC1	1.757009346
TBC1D22A2	4.74E-32	0.2131901	0.234	0.143	1.14E-27	2.5	TBC1D22A	1.636363636
ITGA53	4.85E-32	0.1700373	0.169	0.091	1.17E-27	2.5	ITGA5	1.857142857
LAPTM4A4	4.88E-32	0.2025828	0.442	0.313	1.18E-27	2.5	LAPTM4A	1.412140575
NT5DC11	5.69E-32	0.1919345	0.144	0.075	1.37E-27	2.5	NT5DC1	1.92
RAD9A3	6.01E-32	0.2266952	0.176	0.099	1.45E-27	2.5	RAD9A	1.777777778
PIGN1	6.16E-32	0.1818688	0.197	0.114	1.49E-27	2.5	PIGN	1.728070175
CASP73	6.34E-32	0.2241386	0.241	0.149	1.53E-27	2.5	CASP7	1.617449664
LINC004703	6.47E-32	0.1276019	0.046	0.013	1.56E-27	2.5	LINC00470	3.538461538
PLEKHA11	6.69E-32	0.2085597	0.238	0.146	1.61E-27	2.5	PLEKHA1	1.630136986
ST52	7.29E-32	0.3876855	0.44	0.327	1.76E-27	2.5	ST5	1.345565749
FUCA1	7.44E-32	0.1051173	0.04	0.011	1.79E-27	2.5	FUCA1	3.636363636
CCDC148	7.94E-32	0.1864384	0.095	0.042	1.92E-27	2.5	CCDC148	2.261904762
AKAP113	8.03E-32	0.151708	0.098	0.044	1.94E-27	2.5	AKAP11	2.227272727
GSPT14	9.04E-32	0.1777513	0.496	0.351	2.18E-27	2.5	GSPT1	1.413105413
STRN32	9.12E-32	0.2252986	0.426	0.303	2.20E-27	2.5	STRN3	1.405940594
ELP22	9.86E-32	0.2199065	0.309	0.202	2.38E-27	2.5	ELP2	1.52970297
XRCC43	9.86E-32	0.1950144	0.113	0.054	2.38E-27	2.5	XRCC4	2.092592593
PHC32	1.14E-31	0.2428693	0.25	0.159	2.76E-27	2.5	PHC3	1.572327044
CLASP12	1.14E-31	0.2098242	0.352	0.24	2.76E-27	2.5	CLASP1	1.466666667
ZNF6801	1.15E-31	0.2040179	0.2	0.116	2.78E-27	2.5	ZNF680	1.724137931
STAT31	1.18E-31	0.2468466	0.651	0.523	2.84E-27	2.5	STAT3	1.244741874
NBAT12	1.27E-31	0.171911	0.192	0.109	3.06E-27	2.5	NBAT1	1.76146789
CEP3501	1.34E-31	0.2516141	0.308	0.206	3.22E-27	2.5	CEP350	1.495145631
TBK1	1.51E-31	0.2423717	0.397	0.28	3.64E-27	2.5	TBK1	1.417857143
ECHDC22	2.03E-31	0.214355	0.192	0.112	4.90E-27	2.5	ECHDC2	1.714285714
MCM91	2.10E-31	0.1988165	0.113	0.054	5.05E-27	2.5	MCM9	2.092592593
MCM32	2.38E-31	0.1280684	0.068	0.026	5.73E-27	2.5	MCM3	2.615384615
LONP21	2.38E-31	0.2004973	0.319	0.212	5.74E-27	2.5	LONP2	1.504716981
MLLT102	2.71E-31	0.2290599	0.237	0.147	6.54E-27	2.5	MLLT10	1.612244898
CELSR11	2.84E-31	0.1604111	0.102	0.047	6.84E-27	2.5	CELSR1	2.170212766
EVL1	2.99E-31	0.1769278	0.122	0.061	7.21E-27	2.5	EVL	2
FAM182B	3.37E-31	0.1470118	0.08	0.033	8.12E-27	2.5	FAM182B	2.424242424
HSPB14	3.40E-31	0.1816979	0.115	0.055	8.19E-27	2.5	HSPB1	2.090909091
ATG72	3.41E-31	0.2109494	0.302	0.2	8.22E-27	2.5	ATG7	1.51
HKR1	3.44E-31	0.1642406	0.127	0.064	8.31E-27	2.5	HKR1	1.984375
HSD17B111	3.66E-31	0.132284	0.08	0.033	8.83E-27	2.5	HSD17B11	2.424242424
MAFB3	3.80E-31	0.1310036	0.096	0.043	9.17E-27	2.5	MAFB	2.23255814
DLG13	4.18E-31	0.1973415	0.628	0.489	1.01E-26	2.5	DLG1	1.284253579

PPP2R5A3	4.24E-31	0.2311498	0.239	0.15	1.02E-26	2.5	PPP2R5A	1.593333333
SMURF12	4.36E-31	0.2281187	0.48	0.352	1.05E-26	2.5	SMURF1	1.363636364
CTCF1	4.56E-31	0.2141855	0.165	0.092	1.10E-26	2.5	CTCF	1.793478261
BBS43	4.71E-31	0.1559528	0.111	0.053	1.14E-26	2.5	BBS4	2.094339623
PHF202	5.75E-31	0.2247809	0.469	0.341	1.39E-26	2.5	PHF20	1.375366569
C5orf422	6.02E-31	0.1612776	0.098	0.044	1.45E-26	2.5	C5orf42	2.227272727
NKTR1	6.76E-31	0.2286623	0.427	0.307	1.63E-26	2.5	NKTR	1.390879479
RALGPS11	8.16E-31	0.1528941	0.113	0.054	1.97E-26	2.5	RALGPS1	2.092592593
TMEM87B2	8.19E-31	0.2097179	0.193	0.113	1.98E-26	2.5	TMEM87B	1.707964602
FAM208B2	8.90E-31	0.2309006	0.33	0.223	2.15E-26	2.5	FAM208B	1.479820628
STK33	9.03E-31	0.206218	0.49	0.363	2.18E-26	2.5	STK3	1.349862259
LIN7A2	1.00E-30	0.1463853	0.093	0.041	2.42E-26	2.5	LIN7A	2.268292683
NEO11	1.08E-30	0.1937848	0.162	0.089	2.61E-26	2.5	NEO1	1.820224719
S1PR32	1.28E-30	0.1158029	0.065	0.024	3.08E-26	2.5	S1PR3	2.708333333
CDK132	1.32E-30	0.2824725	0.53	0.41	3.19E-26	2.5	CDK13	1.292682927
BAALC-AS11	1.41E-30	0.1447049	0.063	0.023	3.41E-26	2.5	BAALC-AS1	2.739130435
NDUFS2	1.43E-30	0.1389103	0.122	0.06	3.44E-26	2.5	NDUFS2	2.033333333
CEP1522	1.70E-30	0.1286913	0.076	0.031	4.10E-26	2.5	CEP152	2.451612903
PCCA2	1.73E-30	0.1838105	0.29	0.188	4.16E-26	2.5	PCCA	1.542553191
TBC1D2B1	1.82E-30	0.1464293	0.078	0.032	4.39E-26	2.5	TBC1D2B	2.4375
NSUN61	1.86E-30	0.2004668	0.169	0.095	4.48E-26	2.5	NSUN6	1.778947368
SEMA5A	1.87E-30	0.1334844	0.053	0.018	4.52E-26	2.5	SEMA5A	2.944444444
MARC21	2.29E-30	0.1332183	0.067	0.025	5.53E-26	2.5	MARC2	2.68
IQCE1	2.37E-30	0.1118912	0.054	0.018	5.72E-26	2.5	IQCE	3
RHOT11	2.63E-30	0.2077256	0.163	0.091	6.33E-26	2.5	RHOT1	1.791208791
SERINC21	3.22E-30	0.213836	0.165	0.092	7.75E-26	2.5	SERINC2	1.793478261
USP491	3.36E-30	0.1388337	0.081	0.034	8.11E-26	2.5	USP49	2.382352941
MR11	3.94E-30	0.1591537	0.095	0.043	9.50E-26	2.5	MR1	2.209302326
PTP4A12	3.95E-30	0.2242475	0.386	0.271	9.53E-26	2.5	PTP4A1	1.424354244
STOM3	3.98E-30	0.3391881	0.353	0.25	9.59E-26	2.5	STOM	1.412
BRD8	3.99E-30	0.1663369	0.15	0.081	9.63E-26	2.5	BRD8	1.851851852
STK241	4.18E-30	0.2231057	0.264	0.17	1.01E-25	2.5	STK24	1.552941176
TRIB22	4.82E-30	0.1740687	0.102	0.048	1.16E-25	2.5	TRIB2	2.125
AUTS23	4.92E-30	0.2215925	0.641	0.513	1.19E-25	2.5	AUTS2	1.249512671
ZNF2352	4.97E-30	0.1910215	0.131	0.068	1.20E-25	2.5	ZNF235	1.926470588
CYP20A12	5.08E-30	0.1958674	0.14	0.074	1.22E-25	2.5	CYP20A1	1.891891892
UBE2E21	5.15E-30	0.2294362	0.567	0.435	1.24E-25	2.5	UBE2E2	1.303448276
MIRLET7BHG1	5.77E-30	0.1571482	0.089	0.039	1.39E-25	2.5	MIRLET7BH	2.282051282
MARK2	5.84E-30	0.1896832	0.197	0.116	1.41E-25	2.5	MARK2	1.698275862
JAK13	5.97E-30	0.2389318	0.52	0.392	1.44E-25	2.5	JAK1	1.326530612
ZFAND62	6.27E-30	0.1878907	0.52	0.386	1.51E-25	2.5	ZFAND6	1.347150259
XPR13	6.43E-30	0.1957985	0.261	0.167	1.55E-25	2.5	XPR1	1.562874251
PDSS22	6.98E-30	0.2062928	0.196	0.117	1.68E-25	2.5	PDSS2	1.675213675
SRP144	7.62E-30	0.1909616	0.924	0.867	1.84E-25	2.5	SRP14	1.065743945
SASS6	7.89E-30	0.1457962	0.076	0.032	1.90E-25	2.5	SASS6	2.375
TMEM2451	8.23E-30	0.2073042	0.193	0.114	1.98E-25	2.5	TMEM245	1.692982456
LRRC64	8.35E-30	0.1388729	0.088	0.039	2.01E-25	2.5	LRRC6	2.256410256
PVRL23	8.37E-30	0.2596516	0.382	0.271	2.02E-25	2.5	PVRL2	1.409594096
IFT881	1.06E-29	0.1664412	0.111	0.054	2.56E-25	2.5	IFT88	2.055555556
NUP1072	1.10E-29	0.1931547	0.256	0.164	2.64E-25	2.5	NUP107	1.56097561
CCAR12	1.32E-29	0.217568	0.332	0.229	3.18E-25	2.5	CCAR1	1.449781659
DTX3L1	1.38E-29	0.1387882	0.092	0.041	3.32E-25	2.5	DTX3L	2.243902439

BBS92	1.46E-29	0.1817565	0.124	0.063	3.52E-25	2.5	BBS9	1.968253968
DPY19L12	1.47E-29	0.1828864	0.16	0.088	3.56E-25	2.5	DPY19L1	1.818181818
SC5D4	1.57E-29	0.2449029	0.267	0.173	3.78E-25	2.5	SC5D	1.543352601
CASC42	1.64E-29	0.2072837	0.272	0.177	3.95E-25	2.5	CASC4	1.536723164
NUDT43	1.65E-29	0.1617642	0.129	0.066	3.97E-25	2.5	NUDT4	1.954545455
FILIP1L3	1.69E-29	0.161048	0.182	0.104	4.08E-25	2.5	FILIP1L	1.75
CYP3A51	1.73E-29	0.2109733	0.066	0.026	4.18E-25	2.5	CYP3A5	2.538461538
GRTP1	1.78E-29	0.1012931	0.038	0.01	4.28E-25	2.5	GRTP1	3.8
MTSS1L3	1.98E-29	0.172619	0.107	0.052	4.77E-25	2.5	MTSS1L	2.057692308
PHF142	2.04E-29	0.2137328	0.194	0.116	4.92E-25	2.5	PHF14	1.672413793
PIK3R31	2.07E-29	0.1441135	0.09	0.04	5.00E-25	2.5	PIK3R3	2.25
CDK5RAP21	2.09E-29	0.1679498	0.134	0.071	5.05E-25	2.5	CDK5RAP2	1.887323944
ZNF33A2	2.43E-29	0.2167013	0.303	0.204	5.86E-25	2.5	ZNF33A	1.485294118
TBC1D321	2.51E-29	0.1553539	0.105	0.05	6.05E-25	2.5	TBC1D32	2.1
METTL153	2.85E-29	0.2143676	0.196	0.118	6.88E-25	2.5	METTL15	1.661016949
S100A103	3.00E-29	0.1965039	0.497	0.372	7.23E-25	2.5	S100A10	1.336021505
BTD2	3.23E-29	0.1637369	0.12	0.06	7.79E-25	2.5	BTD	2
HNF4G1	3.35E-29	0.1232348	0.054	0.019	8.07E-25	2.5	HNF4G	2.842105263
DDR13	3.38E-29	0.1994971	0.306	0.204	8.14E-25	2.5	DDR1	1.5
KLF93	3.63E-29	0.2179171	0.23	0.143	8.75E-25	2.5	KLF9	1.608391608
APLF2	3.66E-29	0.1896261	0.172	0.098	8.81E-25	2.5	APLF	1.755102041
HS2ST12	3.77E-29	0.144516	0.079	0.033	9.10E-25	2.5	HS2ST1	2.393939394
DOPEY21	3.94E-29	0.1468015	0.147	0.079	9.49E-25	2.5	DOPEY2	1.860759494
EPB41L53	4.29E-29	0.1549548	0.406	0.282	1.03E-24	2.5	EPB41L5	1.439716312
TUBGCP32	4.57E-29	0.1273006	0.066	0.026	1.10E-24	2.5	TUBGCP3	2.538461538
ECT24	4.71E-29	0.1628796	0.3	0.193	1.14E-24	2.5	ECT2	1.554404145
CD242	4.88E-29	0.3768893	0.68	0.566	1.18E-24	2.5	CD24	1.201413428
RHOQ1	5.51E-29	0.2271462	0.406	0.293	1.33E-24	2.5	RHOQ	1.385665529
WBP1L1	5.55E-29	0.1856377	0.143	0.077	1.34E-24	2.5	WBP1L	1.857142857
SRGAP2C2	5.69E-29	0.1604407	0.134	0.071	1.37E-24	2.5	SRGAP2C	1.887323944
GEM1	5.70E-29	0.1711755	0.096	0.044	1.37E-24	2.5	GEM	2.181818182
ASH1L2	6.69E-29	0.2367221	0.656	0.537	1.61E-24	2.5	ASH1L	1.22160149
DNM3	7.33E-29	0.1617749	0.086	0.038	1.77E-24	2.5	DNM3	2.263157895
REV3L1	7.85E-29	0.2056793	0.379	0.266	1.89E-24	2.5	REV3L	1.42481203
POLA11	8.94E-29	0.1485986	0.096	0.045	2.15E-24	2.5	POLA1	2.133333333
DMXL12	9.52E-29	0.1928888	0.248	0.156	2.30E-24	2.5	DMXL1	1.58974359
OVOL22	9.93E-29	0.1775392	0.114	0.057	2.39E-24	2.5	OVOL2	2
GLUD13	1.01E-28	0.1937803	0.378	0.263	2.43E-24	2.5	GLUD1	1.437262357
MTMR6	1.02E-28	0.1617314	0.112	0.055	2.45E-24	2.5	MTMR6	2.036363636
CMTR22	1.07E-28	0.1463387	0.085	0.038	2.58E-24	2.5	CMTR2	2.236842105
PCNXL43	1.07E-28	0.1783318	0.17	0.098	2.59E-24	2.5	PCNXL4	1.734693878
PRPF31	1.28E-28	0.2114479	0.201	0.122	3.09E-24	2.5	PRPF3	1.647540984
UBL54	1.39E-28	0.1422675	0.634	0.492	3.35E-24	2.5	UBL5	1.288617886
KIF271	1.42E-28	0.1756707	0.124	0.064	3.42E-24	2.5	KIF27	1.9375
MYL63	1.52E-28	0.1898991	0.956	0.931	3.66E-24	2.5	MYL6	1.026852846
CASD11	1.65E-28	0.1681855	0.118	0.06	3.98E-24	2.5	CASD1	1.966666667
TP53I11	1.67E-28	0.1048578	0.053	0.018	4.04E-24	2.5	TP53I11	2.944444444
WSB12	1.80E-28	0.165461	0.502	0.375	4.35E-24	2.5	WSB1	1.338666667
LIMD11	1.92E-28	0.1671424	0.129	0.067	4.64E-24	2.5	LIMD1	1.925373134
ZNF4382	2.10E-28	0.1901148	0.124	0.065	5.07E-24	2.5	ZNF438	1.907692308
PGM21	2.23E-28	0.1808949	0.133	0.07	5.37E-24	2.5	PGM2	1.9
NEK114	2.24E-28	0.136716	0.114	0.056	5.39E-24	2.5	NEK11	2.035714286

LRRC37A32	2.24E-28	0.1981382	0.175	0.102	5.41E-24	2.5	LRRC37A3	1.715686275
TULP41	2.63E-28	0.1356268	0.63	0.483	6.35E-24	2.5	TULP4	1.304347826
SIRT13	2.69E-28	0.1935939	0.167	0.095	6.49E-24	2.5	SIRT1	1.757894737
TSPAN62	2.93E-28	0.2131595	0.257	0.167	7.06E-24	2.5	TSPAN6	1.538922156
KIAA00401	2.96E-28	0.1704599	0.138	0.074	7.13E-24	2.5	KIAA0040	1.864864865
GOLPH32	2.97E-28	0.2075673	0.359	0.251	7.16E-24	2.5	GOLPH3	1.430278884
MAPK83	3.63E-28	0.1109051	0.446	0.319	8.76E-24	2.5	MAPK8	1.398119122
EFCAB141	4.32E-28	0.176953	0.175	0.101	1.04E-23	2.5	EFCAB14	1.732673267
KIAA14681	4.51E-28	0.1981186	0.201	0.122	1.09E-23	2.5	KIAA1468	1.647540984
TTC39A3	4.51E-28	0.134697	0.096	0.045	1.09E-23	2.5	TTC39A	2.133333333
ANAPC133	4.56E-28	0.2115553	0.219	0.137	1.10E-23	2.5	ANAPC13	1.598540146
TNFSF13B2	4.63E-28	0.1569697	0.096	0.045	1.12E-23	2.5	TNFSF13B	2.133333333
RP11-384F7.24	5.09E-28	0.2357774	0.113	0.056	1.23E-23	2.5	RP11-384F	2.017857143
PPP1R9A2	5.14E-28	0.2493959	0.228	0.146	1.24E-23	2.5	PPP1R9A	1.561643836
TMEM50A2	5.45E-28	0.201229	0.287	0.191	1.31E-23	2.5	TMEM50A	1.502617801
EVI52	5.46E-28	0.2014768	0.232	0.146	1.32E-23	2.5	EVI5	1.589041096
ABRACL3	6.29E-28	0.2091664	0.532	0.407	1.52E-23	2.5	ABRACL	1.307125307
DENND1A2	6.98E-28	0.1894476	0.36	0.251	1.68E-23	2.5	DENND1A	1.434262948
TRIM66	7.13E-28	0.1058401	0.053	0.019	1.72E-23	2.5	TRIM66	2.789473684
ATE13	7.61E-28	0.196906	0.195	0.118	1.84E-23	2.5	ATE1	1.652542373
ZBTB184	8.58E-28	0.1361022	0.127	0.066	2.07E-23	2.5	ZBTB18	1.924242424
ETFA1	8.90E-28	0.1837818	0.201	0.122	2.15E-23	2.5	ETFA	1.647540984
HHAT1	9.89E-28	0.1133539	0.053	0.019	2.39E-23	2.5	HHAT	2.789473684
TMEM161B-AS12	1.07E-27	0.215495	0.172	0.101	2.58E-23	2.5	TMEM161E	1.702970297
BUB31	1.08E-27	0.1598379	0.15	0.083	2.61E-23	2.5	BUB3	1.807228916
CCDC931	1.17E-27	0.2026382	0.219	0.137	2.83E-23	2.5	CCDC93	1.598540146
TMEM1653	1.22E-27	0.1681743	0.647	0.504	2.94E-23	2.5	TMEM165	1.283730159
PRICKLE2	1.24E-27	0.1076252	0.066	0.026	3.00E-23	2.5	PRICKLE2	2.538461538
RP11-382A20.41	1.50E-27	0.1245891	0.078	0.034	3.61E-23	2.5	RP11-382A	2.294117647
KRIT12	1.62E-27	0.1839675	0.179	0.106	3.89E-23	2.5	KRIT1	1.688679245
COPG21	1.67E-27	0.1367271	0.084	0.038	4.03E-23	2.5	COPG2	2.210526316
TNRC181	1.69E-27	0.1590928	0.137	0.075	4.09E-23	2.5	TNRC18	1.826666667
RTN4RL1	1.90E-27	0.1218081	0.054	0.019	4.59E-23	2.5	RTN4RL1	2.842105263
RASD11	1.93E-27	0.1736172	0.103	0.051	4.66E-23	2.5	RASD1	2.019607843
PSMD15	2.03E-27	0.1902579	0.224	0.141	4.89E-23	2.5	PSMD1	1.588652482
PPT12	2.05E-27	0.1251873	0.079	0.034	4.94E-23	2.5	PPT1	2.323529412
RP11-39M21.12	2.14E-27	0.1527828	0.11	0.055	5.16E-23	2.5	RP11-39M2	2
UBL33	2.16E-27	0.2282115	0.285	0.191	5.22E-23	2.5	UBL3	1.492146597
ZNF4871	2.33E-27	0.1591551	0.103	0.05	5.61E-23	2.5	ZNF487	2.06
FCHSD22	2.35E-27	0.2000205	0.2	0.122	5.66E-23	2.5	FCHSD2	1.639344262
EP3003	2.66E-27	0.2092844	0.306	0.209	6.41E-23	2.5	EP300	1.464114833
LCORL1	2.72E-27	0.2012652	0.217	0.136	6.56E-23	2.5	LCORL	1.595588235
AHCYL13	2.88E-27	0.2126031	0.4	0.29	6.95E-23	2.5	AHCYL1	1.379310345
RUFY31	3.25E-27	0.1912988	0.329	0.227	7.83E-23	2.5	RUFY3	1.449339207
ELMSAN1	3.59E-27	0.1693615	0.147	0.081	8.66E-23	2.5	ELMSAN1	1.814814815
ETAA11	3.77E-27	0.1298329	0.073	0.031	9.09E-23	2.5	ETAA1	2.35483871
CACNG42	3.87E-27	0.1565422	0.108	0.053	9.33E-23	2.5	CACNG4	2.037735849
SYNPO1	3.93E-27	0.1192373	0.06	0.023	9.48E-23	2.5	SYNPO	2.608695652
BNIPL2	4.44E-27	0.1314986	0.087	0.04	1.07E-22	2.5	BNIPL	2.175
ZNF4071	4.65E-27	0.1912911	0.214	0.133	1.12E-22	2.5	ZNF407	1.609022556
SDF21	4.73E-27	0.2006064	0.186	0.112	1.14E-22	2.5	SDF2	1.660714286
TMPRSS11E3	5.08E-27	0.160667	0.1	0.048	1.23E-22	2.5	TMPRSS11I	2.083333333

TCF252	5.35E-27	0.1768095	0.198	0.12	1.29E-22	2.5	TCF25	1.65
LLGL21	5.36E-27	0.1849857	0.162	0.093	1.29E-22	2.5	LLGL2	1.741935484
CHCHD73	5.67E-27	0.166588	0.142	0.079	1.37E-22	2.5	CHCHD7	1.797468354
TAP11	6.06E-27	0.1400658	0.083	0.037	1.46E-22	2.5	TAP1	2.243243243
PCDH11	6.48E-27	0.1243593	0.065	0.026	1.56E-22	2.5	PCDH1	2.5
AHCYL21	7.04E-27	0.1744577	0.176	0.104	1.70E-22	2.5	AHCYL2	1.692307692
AVL93	7.17E-27	0.1961596	0.283	0.19	1.73E-22	2.5	AVL9	1.489473684
PTK22	7.55E-27	0.1639094	0.758	0.626	1.82E-22	2.5	PTK2	1.21086262
PARP142	7.72E-27	0.1944648	0.204	0.126	1.86E-22	2.5	PARP14	1.619047619
APOL62	8.00E-27	0.1956294	0.183	0.11	1.93E-22	2.5	APOL6	1.663636364
IFT1221	8.54E-27	0.1396463	0.069	0.029	2.06E-22	2.5	IFT122	2.379310345
SRP93	8.73E-27	0.1883688	0.531	0.408	2.10E-22	2.5	SRP9	1.301470588
MAOA3	9.05E-27	0.1355836	0.106	0.052	2.18E-22	2.5	MAOA	2.038461538
ZNF5682	9.77E-27	0.1383347	0.08	0.035	2.36E-22	2.5	ZNF568	2.285714286
AGL3	1.01E-26	0.1565292	0.086	0.039	2.44E-22	2.5	AGL	2.205128205
ATG16L13	1.02E-26	0.1210705	0.09	0.042	2.46E-22	2.5	ATG16L1	2.142857143
ZNF2262	1.12E-26	0.1766657	0.149	0.084	2.69E-22	2.5	ZNF226	1.773809524
METTL162	1.17E-26	0.2090081	0.255	0.168	2.82E-22	2.5	METTL16	1.517857143
ACSS31	1.23E-26	0.1015456	0.05	0.017	2.96E-22	2.5	ACSS3	2.941176471
CD2741	1.23E-26	0.1366644	0.072	0.03	2.97E-22	2.5	CD274	2.4
ARFIP12	1.46E-26	0.1839845	0.222	0.14	3.51E-22	2.5	ARFIP1	1.585714286
ADAMTS171	1.55E-26	0.111506	0.061	0.024	3.74E-22	2.5	ADAMTS17	2.541666667
PSPC12	1.58E-26	0.2014092	0.272	0.181	3.80E-22	2.5	PSPC1	1.502762431
ZDHHC142	1.61E-26	0.1743234	0.187	0.111	3.89E-22	2.5	ZDHHC14	1.684684685
SAXO23	1.64E-26	0.1230286	0.069	0.029	3.96E-22	2.5	SAXO2	2.379310345
TEAD31	1.74E-26	0.138902	0.106	0.052	4.19E-22	2.5	TEAD3	2.038461538
PIP4K2A3	1.80E-26	0.122522	0.186	0.11	4.35E-22	2.5	PIP4K2A	1.690909091
SDHA1	1.81E-26	0.1533269	0.149	0.083	4.37E-22	2.5	SDHA	1.795180723
IFT432	2.05E-26	0.1778693	0.152	0.086	4.93E-22	2.5	IFT43	1.76744186
CTSZ4	2.07E-26	0.140334	0.115	0.059	4.99E-22	2.5	CTSZ	1.949152542
SUPT20H1	2.26E-26	0.1584223	0.134	0.073	5.44E-22	2.5	SUPT20H	1.835616438
RPAP22	2.27E-26	0.19755	0.285	0.189	5.47E-22	2.5	RPAP2	1.507936508
TBC1D122	2.46E-26	0.2032013	0.134	0.072	5.92E-22	2.5	TBC1D12	1.861111111
DHRSX2	2.80E-26	0.2029947	0.371	0.265	6.76E-22	2.5	DHRSX	1.4
ANKMY2	2.90E-26	0.1402043	0.083	0.038	6.99E-22	2.5	ANKMY2	2.184210526
AP1M22	2.98E-26	0.1526626	0.133	0.072	7.20E-22	2.5	AP1M2	1.847222222
SLC16A21	3.15E-26	0.127281	0.069	0.029	7.60E-22	2.5	SLC16A2	2.379310345
SCGB2B22	3.20E-26	0.1601765	0.1	0.049	7.72E-22	2.5	SCGB2B2	2.040816327
CCBL22	3.46E-26	0.1833787	0.184	0.111	8.35E-22	2.5	CCBL2	1.657657658
ANKRA21	3.65E-26	0.1762086	0.119	0.063	8.79E-22	2.5	ANKRA2	1.888888889
AHNAK3	3.90E-26	0.2011008	0.456	0.343	9.41E-22	2.5	AHNAK	1.329446064
C2orf691	4.53E-26	0.1475582	0.095	0.046	1.09E-21	2.5	C2orf69	2.065217391
KCNS31	4.54E-26	0.1264722	0.063	0.026	1.09E-21	2.5	KCNS3	2.423076923
ATP1A13	4.69E-26	0.1999119	0.613	0.491	1.13E-21	2.5	ATP1A1	1.248472505
WWC22	4.95E-26	0.15744	0.284	0.187	1.19E-21	2.5	WWC2	1.518716578
TXNDC121	5.08E-26	0.1603029	0.119	0.063	1.23E-21	2.5	TXNDC12	1.888888889
KIAA03681	5.38E-26	0.2098671	0.331	0.234	1.30E-21	2.5	KIAA0368	1.414529915
PARP13	5.42E-26	0.1780126	0.169	0.1	1.31E-21	2.5	PARP1	1.69
HIST1H4H2	5.96E-26	0.1765968	0.129	0.07	1.44E-21	2.5	HIST1H4H	1.842857143
MACROD22	6.21E-26	0.2573599	0.326	0.231	1.50E-21	2.5	MACROD2	1.411255411
PGRMC22	6.32E-26	0.1537881	0.198	0.12	1.52E-21	2.5	PGRMC2	1.65
TBC1D43	6.65E-26	0.217487	0.276	0.185	1.60E-21	2.5	TBC1D4	1.491891892

CCDC641	7.27E-26	0.1717917	0.1	0.05	1.75E-21	2.5	CCDC64	2
AF127936.93	7.32E-26	0.1493673	0.22	0.136	1.76E-21	2.5	AF127936.9	1.617647059
PTER1	7.57E-26	0.1306101	0.094	0.045	1.82E-21	2.5	PTER	2.088888889
ZC3H62	8.99E-26	0.1670377	0.126	0.068	2.17E-21	2.5	ZC3H6	1.852941176
RPA32	9.09E-26	0.1744474	0.208	0.131	2.19E-21	2.5	RPA3	1.58778626
SLC20A13	9.16E-26	0.1656247	0.115	0.059	2.21E-21	2.5	SLC20A1	1.949152542
ALMS11	9.75E-26	0.1111388	0.059	0.023	2.35E-21	2.5	ALMS1	2.565217391
LRRC691	1.01E-25	0.1701593	0.096	0.047	2.44E-21	2.5	LRRC69	2.042553191
DPH63	1.07E-25	0.1778727	0.115	0.06	2.57E-21	2.5	DPH6	1.916666667
UNC13B2	1.15E-25	0.1419109	0.133	0.072	2.77E-21	2.5	UNC13B	1.847222222
RB1CC12	1.18E-25	0.1825134	0.6	0.466	2.83E-21	2.5	RB1CC1	1.287553648
ATM2	1.18E-25	0.1528996	0.115	0.06	2.85E-21	2.5	ATM	1.916666667
AK32	1.28E-25	0.1923479	0.29	0.196	3.08E-21	2.5	AK3	1.479591837
TOB11	1.33E-25	0.1842431	0.182	0.11	3.21E-21	2.5	TOB1	1.654545455
RAI142	1.39E-25	0.1669749	0.247	0.161	3.35E-21	2.5	RAI14	1.534161491
RP11-418J17.11	1.45E-25	0.1545026	0.087	0.041	3.50E-21	2.5	RP11-418J1	2.12195122
ATIC	1.66E-25	0.1184881	0.068	0.028	4.00E-21	2.5	ATIC	2.428571429
HSDL11	1.68E-25	0.108395	0.074	0.032	4.06E-21	2.5	HSDL1	2.3125
MCCC23	1.89E-25	0.1696133	0.232	0.149	4.55E-21	2.5	MCCC2	1.55704698
DDX3X2	2.10E-25	0.1748975	0.467	0.35	5.06E-21	2.5	DDX3X	1.334285714
CTB-171A8.11	2.40E-25	0.1681708	0.148	0.085	5.79E-21	2.5	CTB-171A8	1.741176471
HAUS11	2.49E-25	0.1482827	0.117	0.062	6.01E-21	2.5	HAUS1	1.887096774
SMARCC13	2.80E-25	0.1957213	0.383	0.278	6.76E-21	2.5	SMARCC1	1.377697842
CETN22	2.86E-25	0.1641296	0.262	0.172	6.90E-21	2.5	CETN2	1.523255814
LGALS31	2.90E-25	0.1931627	0.287	0.195	6.99E-21	2.5	LGALS3	1.471794872
WDR781	2.94E-25	0.1424625	0.075	0.034	7.10E-21	2.5	WDR78	2.205882353
SETDB22	2.99E-25	0.1247252	0.066	0.028	7.22E-21	2.5	SETDB2	2.357142857
LRP101	3.03E-25	0.1746027	0.203	0.127	7.31E-21	2.5	LRP10	1.598425197
MCUR12	3.05E-25	0.1516773	0.108	0.055	7.35E-21	2.5	MCUR1	1.963636364
KLF124	3.48E-25	0.1374366	0.22	0.138	8.39E-21	2.5	KLF12	1.594202899
XIAP1	3.49E-25	0.1843448	0.204	0.128	8.42E-21	2.5	XIAP	1.59375
GNS4	3.55E-25	0.1607241	0.15	0.085	8.57E-21	2.5	GNS	1.764705882
ADSS	3.63E-25	0.1220787	0.075	0.033	8.76E-21	2.5	ADSS	2.272727273
SRBD11	3.75E-25	0.1684221	0.111	0.057	9.05E-21	2.5	SRBD1	1.947368421
FAM179B2	3.87E-25	0.1802125	0.139	0.078	9.34E-21	2.5	FAM179B	1.782051282
FAM32A2	3.88E-25	0.160376	0.136	0.076	9.35E-21	2.5	FAM32A	1.789473684
B4GALT11	3.94E-25	0.2308072	0.542	0.426	9.50E-21	2.5	B4GALT1	1.272300469
CDYL3	4.70E-25	0.1668043	0.413	0.299	1.13E-20	2.5	CDYL	1.381270903
ZNF2481	4.74E-25	0.1523917	0.098	0.049	1.14E-20	2.5	ZNF248	2
NOSTRIN3	5.00E-25	0.1088258	0.071	0.03	1.21E-20	2.5	NOSTRIN	2.366666667
GNG12-AS11	5.02E-25	0.1669116	0.131	0.073	1.21E-20	2.5	GNG12-AS1	1.794520548
CHMP2A2	5.40E-25	0.1573862	0.148	0.084	1.30E-20	2.5	CHMP2A	1.761904762
PCAT11	5.61E-25	0.1167154	0.055	0.021	1.35E-20	2.5	PCAT1	2.619047619
USP542	5.93E-25	0.2203876	0.608	0.481	1.43E-20	2.5	USP54	1.264033264
NSD12	6.92E-25	0.1852355	0.189	0.118	1.67E-20	2.5	NSD1	1.601694915
AAGAB	7.63E-25	0.1875366	0.217	0.139	1.84E-20	2.5	AAGAB	1.561151079
SLC9A12	7.81E-25	0.1854346	0.186	0.114	1.88E-20	2.5	SLC9A1	1.631578947
ZC3HAV13	7.92E-25	0.1837877	0.268	0.179	1.91E-20	2.5	ZC3HAV1	1.497206704
MARCH21	8.01E-25	0.1333447	0.083	0.038	1.93E-20	2.5	MARCH2	2.184210526
JMY1	8.68E-25	0.1923491	0.196	0.123	2.09E-20	2.5	JMY	1.593495935
SPPL32	8.86E-25	0.1693859	0.356	0.253	2.14E-20	2.5	SPPL3	1.407114625
KANSL31	9.05E-25	0.142682	0.135	0.075	2.18E-20	2.5	KANSL3	1.8

TNS32	9.48E-25	0.1515648	0.119	0.064	2.29E-20	2.5	TNS3	1.859375
MFSD113	9.55E-25	0.1705355	0.151	0.088	2.30E-20	2.5	MFSD11	1.715909091
PAK41	1.04E-24	0.133071	0.084	0.04	2.50E-20	2.5	PAK4	2.1
GLB1	1.09E-24	0.1222962	0.084	0.039	2.64E-20	2.5	GLB1	2.153846154
ARHGDIB2	1.21E-24	0.2232131	0.225	0.146	2.92E-20	2.5	ARHGDIB	1.54109589
NR4A23	1.23E-24	0.1961948	0.171	0.103	2.96E-20	2.5	NR4A2	1.660194175
PPP2R3C1	1.31E-24	0.1694524	0.168	0.101	3.17E-20	2.5	PPP2R3C	1.663366337
CHFR1	1.56E-24	0.1578179	0.144	0.082	3.75E-20	2.5	CHFR	1.756097561
TEAD23	1.64E-24	0.1574847	0.125	0.069	3.95E-20	2.5	TEAD2	1.811594203
ACSL12	1.65E-24	0.2142016	0.281	0.194	3.98E-20	2.5	ACSL1	1.448453608
ZMYND111	2.19E-24	0.1710322	0.21	0.133	5.27E-20	2.5	ZMYND11	1.578947368
UEVLD2	2.29E-24	0.1475384	0.12	0.065	5.53E-20	2.5	UEVLD	1.846153846
C3orf142	2.38E-24	0.1338528	0.176	0.106	5.74E-20	2.5	C3orf14	1.660377358
EPB41L4B2	2.47E-24	0.1676511	0.244	0.161	5.96E-20	2.5	EPB41L4B	1.51552795
DPYD-AS12	2.63E-24	0.1495747	0.126	0.069	6.34E-20	2.5	DPYD-AS1	1.826086957
PANK34	2.75E-24	0.1336523	0.257	0.168	6.64E-20	2.5	PANK3	1.529761905
DAZAP22	2.76E-24	0.1816196	0.529	0.411	6.66E-20	2.5	DAZAP2	1.287104623
C4orf323	3.08E-24	0.1484723	0.163	0.096	7.43E-20	2.5	C4orf32	1.697916667
RP5-1101C3.12	3.15E-24	0.1350272	0.104	0.054	7.60E-20	2.5	RP5-1101C	1.925925926
ATP6AP12	3.35E-24	0.1373755	0.097	0.049	8.08E-20	2.5	ATP6AP1	1.979591837
ATP6V0D11	3.55E-24	0.1263665	0.096	0.048	8.55E-20	2.5	ATP6V0D1	2
WNK13	3.82E-24	0.1794851	0.327	0.231	9.22E-20	2.5	WNK1	1.415584416
SENP21	4.00E-24	0.1923414	0.184	0.114	9.63E-20	2.5	SENP2	1.614035088
IQCD1	4.09E-24	0.1105465	0.051	0.019	9.87E-20	2.5	IQCD	2.684210526
SHPRH2	4.14E-24	0.1626431	0.143	0.083	9.98E-20	2.5	SHPRH	1.722891566
C16orf461	4.67E-24	0.1121614	0.058	0.023	1.13E-19	2.5	C16orf46	2.52173913
CTNND13	4.70E-24	0.1720801	0.501	0.382	1.13E-19	2.5	CTNND1	1.311518325
TMEM87A2	5.44E-24	0.1746351	0.489	0.371	1.31E-19	2.5	TMEM87A	1.318059299
EMC103	5.46E-24	0.1486581	0.105	0.054	1.32E-19	2.5	EMC10	1.944444444
NCOR12	6.08E-24	0.1604601	0.529	0.408	1.47E-19	2.5	NCOR1	1.296568627
SLC39A12	6.10E-24	0.1844546	0.162	0.097	1.47E-19	2.5	SLC39A1	1.670103093
RPS6KC11	6.24E-24	0.1785218	0.167	0.101	1.50E-19	2.5	RPS6KC1	1.653465347
SVIP2	6.29E-24	0.125596	0.087	0.042	1.52E-19	2.5	SVIP	2.071428571
PSD4	6.43E-24	0.1063638	0.051	0.019	1.55E-19	2.5	PSD4	2.684210526
NPC23	6.76E-24	0.1895077	0.589	0.47	1.63E-19	2.5	NPC2	1.253191489
TRIM383	7.22E-24	0.1484937	0.154	0.091	1.74E-19	2.5	TRIM38	1.692307692
SPATA6L2	7.40E-24	0.1241561	0.108	0.056	1.78E-19	2.5	SPATA6L	1.928571429
NIN3	7.44E-24	0.157486	0.161	0.096	1.79E-19	2.5	NIN	1.677083333
NBR12	7.70E-24	0.1959487	0.245	0.164	1.86E-19	2.5	NBR1	1.493902439
AASDH2	7.70E-24	0.132071	0.097	0.049	1.86E-19	2.5	AASDH	1.979591837
IGFBP24	8.57E-24	0.13561	0.091	0.044	2.07E-19	2.5	IGFBP2	2.068181818
SAMD81	9.85E-24	0.1435602	0.158	0.093	2.38E-19	2.5	SAMD8	1.698924731
ANKMY1	9.99E-24	0.1072016	0.05	0.018	2.41E-19	2.5	ANKMY1	2.777777778
GDAP21	1.02E-23	0.1437506	0.106	0.055	2.46E-19	2.5	GDAP2	1.927272727
GPR137C1	1.03E-23	0.1840599	0.132	0.074	2.49E-19	2.5	GPR137C	1.783783784
CYB5R12	1.11E-23	0.1160771	0.065	0.027	2.68E-19	2.5	CYB5R1	2.407407407
ODF2L2	1.15E-23	0.1666809	0.228	0.149	2.78E-19	2.5	ODF2L	1.530201342
RHOH2	1.23E-23	0.1063854	0.066	0.028	2.96E-19	2.5	RHOH	2.357142857
DNAJC161	1.23E-23	0.1229568	0.113	0.06	2.98E-19	2.5	DNAJC16	1.883333333
ATG2B	1.25E-23	0.1162558	0.077	0.036	3.03E-19	2.5	ATG2B	2.138888889
PPM1K4	1.25E-23	0.1887509	0.219	0.141	3.03E-19	2.5	PPM1K	1.553191489
NINL1	1.36E-23	0.1302806	0.066	0.029	3.29E-19	2.5	NINL	2.275862069

PEBP44	1.42E-23	0.1594209	0.152	0.088	3.43E-19	2.5	PEBP4	1.727272727
INIP	1.63E-23	0.1487874	0.127	0.07	3.94E-19	2.5	INIP	1.814285714
RHOB3	1.64E-23	0.1159465	0.173	0.104	3.95E-19	2.5	RHOB	1.663461538
ERBB31	1.68E-23	0.1620587	0.197	0.124	4.05E-19	2.5	ERBB3	1.588709677
HOOK22	1.69E-23	0.1636585	0.225	0.147	4.07E-19	2.5	HOOK2	1.530612245
MAPKAP12	1.73E-23	0.1707617	0.171	0.104	4.17E-19	2.5	MAPKAP1	1.644230769
PVRL42	1.89E-23	0.1839968	0.268	0.182	4.55E-19	2.5	PVRL4	1.472527473
ALG131	1.91E-23	0.1593863	0.177	0.108	4.60E-19	2.5	ALG13	1.638888889
AC016831.72	1.97E-23	0.1423334	0.346	0.245	4.75E-19	2.5	AC016831.7	1.412244898
CFL13	1.99E-23	0.217755	0.409	0.305	4.81E-19	2.5	CFL1	1.340983607
C11orf491	2.07E-23	0.1798596	0.225	0.147	4.99E-19	2.5	C11orf49	1.530612245
STAU21	2.23E-23	0.1599176	0.166	0.1	5.38E-19	2.5	STAU2	1.66
SBF22	2.27E-23	0.1550268	0.752	0.621	5.47E-19	2.5	SBF2	1.210950081
ZNF273	2.34E-23	0.1216674	0.071	0.032	5.65E-19	2.5	ZNF273	2.21875
YPEL22	2.48E-23	0.1882981	0.176	0.107	5.97E-19	2.5	YPEL2	1.644859813
PDE11A1	2.72E-23	0.1120951	0.069	0.031	6.56E-19	2.5	PDE11A	2.225806452
NUP62CL1	2.75E-23	0.1097132	0.049	0.018	6.64E-19	2.5	NUP62CL	2.722222222
RNF1492	3.03E-23	0.1597321	0.446	0.335	7.31E-19	2.5	RNF149	1.331343284
WWC13	3.09E-23	0.2781542	0.509	0.407	7.45E-19	2.5	WWC1	1.250614251
RP11-437B10.11	3.16E-23	0.1339765	0.508	0.388	7.61E-19	2.5	RP11-437B	1.309278351
NSMCE22	3.37E-23	0.1852029	0.473	0.369	8.13E-19	2.5	NSMCE2	1.281842818
CAPN133	3.52E-23	0.1253712	0.074	0.033	8.49E-19	2.5	CAPN13	2.242424242
SYCP22	3.57E-23	0.1247796	0.076	0.035	8.62E-19	2.5	SYCP2	2.171428571
GALNT101	4.14E-23	0.1420811	0.104	0.054	9.99E-19	2.5	GALNT10	1.925925926
GBP32	4.33E-23	0.185482	0.205	0.132	1.04E-18	2.5	GBP3	1.553030303
ATR2	4.44E-23	0.1920398	0.202	0.13	1.07E-18	2.5	ATR	1.553846154
ZNF7182	4.50E-23	0.15348	0.111	0.059	1.08E-18	2.5	ZNF718	1.881355932
RP11-6N13.13	4.61E-23	0.2693872	0.083	0.04	1.11E-18	2.5	RP11-6N13	2.075
MED312	4.63E-23	0.1944876	0.248	0.168	1.12E-18	2.5	MED31	1.476190476
LGALS11	4.75E-23	0.2839212	0.131	0.074	1.14E-18	2.5	LGALS1	1.77027027
TIGAR1	4.88E-23	0.1142585	0.063	0.027	1.18E-18	2.5	TIGAR	2.333333333
LRRFIP15	4.89E-23	0.1399879	0.745	0.614	1.18E-18	2.5	LRRFIP1	1.213355049
DUSP61	4.94E-23	0.1305892	0.103	0.054	1.19E-18	2.5	DUSP6	1.907407407
FAT12	5.30E-23	0.1270715	0.283	0.192	1.28E-18	2.5	FAT1	1.473958333
ZNF5401	5.84E-23	0.1321721	0.071	0.032	1.41E-18	2.5	ZNF540	2.21875
PRKRA1	5.93E-23	0.1465952	0.137	0.079	1.43E-18	2.5	PRKRA	1.734177215
SGK2231	5.96E-23	0.159803	0.131	0.074	1.44E-18	2.5	SGK223	1.77027027
PHF113	7.65E-23	0.1631047	0.136	0.079	1.84E-18	2.5	PHF11	1.721518987
SEMA3E4	7.86E-23	0.1209443	0.211	0.133	1.90E-18	2.5	SEMA3E	1.586466165
CNST1	8.58E-23	0.1343954	0.115	0.062	2.07E-18	2.5	CNST	1.85483871
HAT12	8.63E-23	0.1603534	0.271	0.183	2.08E-18	2.5	HAT1	1.480874317
TMCO33	9.18E-23	0.1523388	0.204	0.129	2.21E-18	2.5	TMCO3	1.581395349
KLHL132	1.03E-22	0.2288455	0.167	0.102	2.48E-18	2.5	KLHL13	1.637254902
TAF152	1.15E-22	0.1825628	0.379	0.282	2.77E-18	2.5	TAF15	1.343971631
IMMP1L3	1.19E-22	0.142815	0.205	0.13	2.86E-18	2.5	IMMP1L	1.576923077
FAM208A3	1.34E-22	0.1472316	0.137	0.079	3.22E-18	2.5	FAM208A	1.734177215
FBXW42	1.53E-22	0.155797	0.139	0.08	3.69E-18	2.5	FBXW4	1.7375
ACYP11	1.54E-22	0.1330768	0.135	0.077	3.72E-18	2.5	ACYP1	1.753246753
MREG2	1.65E-22	0.1589601	0.208	0.133	3.99E-18	2.5	MREG	1.563909774
CARS1	1.67E-22	0.1485376	0.172	0.106	4.03E-18	2.5	CARS	1.622641509
AP000769.12	1.68E-22	0.203927	0.198	0.126	4.06E-18	2.5	AP000769.1	1.571428571
CTC-260E6.61	1.79E-22	0.1195063	0.095	0.049	4.33E-18	2.5	CTC-260E6.	1.93877551

CSGALNACT22	1.90E-22	0.146346	0.19	0.118	4.59E-18	2.5	CSGALNAC	1.610169492
MVP2	2.01E-22	0.1647506	0.106	0.057	4.83E-18	2.5	MVP	1.859649123
UBE4B2	2.23E-22	0.1373569	0.321	0.228	5.37E-18	2.5	UBE4B	1.407894737
NOS1AP3	2.41E-22	0.1507857	0.295	0.207	5.81E-18	2.5	NOS1AP	1.425120773
STEAP12	2.55E-22	0.1327568	0.093	0.047	6.14E-18	2.5	STEAP1	1.978723404
SYT71	2.58E-22	0.1584284	0.09	0.045	6.22E-18	2.5	SYT7	2
MTOR2	2.60E-22	0.1324438	0.098	0.051	6.26E-18	2.5	MTOR	1.921568627
CITED22	2.60E-22	0.1543603	0.11	0.059	6.27E-18	2.5	CITED2	1.86440678
NPTN3	2.68E-22	0.1607268	0.273	0.186	6.47E-18	2.5	NPTN	1.467741935
RFWD24	2.71E-22	0.1589981	0.393	0.288	6.54E-18	2.5	RFWD2	1.364583333
HEATR5A2	2.94E-22	0.1477062	0.108	0.058	7.09E-18	2.5	HEATR5A	1.862068966
TLK12	2.97E-22	0.1610554	0.269	0.185	7.16E-18	2.5	TLK1	1.454054054
MAP1LC3B3	3.10E-22	0.1598892	0.222	0.145	7.48E-18	2.5	MAP1LC3B	1.531034483
PIK3CA2	3.39E-22	0.1787497	0.376	0.275	8.17E-18	2.5	PIK3CA	1.367272727
PRKDC2	3.84E-22	0.1867245	0.165	0.101	9.25E-18	2.5	PRKDC	1.633663366
HIVEP33	3.90E-22	0.1727018	0.404	0.305	9.39E-18	2.5	HIVEP3	1.324590164
ARID21	4.03E-22	0.1833106	0.4	0.298	9.71E-18	2.5	ARID2	1.342281879
HMGB14	4.15E-22	0.168122	0.655	0.529	1.00E-17	2.5	HMGB1	1.238185255
CREBBP2	4.16E-22	0.1528732	0.392	0.288	1.00E-17	2.5	CREBBP	1.361111111
OLA12	4.20E-22	0.1586233	0.419	0.312	1.01E-17	2.5	OLA1	1.342948718
NUMA12	4.40E-22	0.1319873	0.116	0.063	1.06E-17	2.5	NUMA1	1.841269841
EMC71	4.41E-22	0.1256718	0.092	0.047	1.06E-17	2.5	EMC7	1.957446809
UBXN43	4.81E-22	0.1721251	0.4	0.299	1.16E-17	2.5	UBXN4	1.337792642
BORCS5	5.21E-22	0.1265752	0.11	0.06	1.26E-17	2.5	BORCS5	1.833333333
JAK22	5.43E-22	0.1223926	0.159	0.096	1.31E-17	2.5	JAK2	1.65625
PDHX2	6.05E-22	0.1315113	0.124	0.07	1.46E-17	2.5	PDHX	1.771428571
DYNC1LI21	6.28E-22	0.1683855	0.31	0.221	1.52E-17	2.5	DYNC1LI2	1.402714932
C11orf542	6.56E-22	0.1583888	0.158	0.097	1.58E-17	2.5	C11orf54	1.628865979
APMAP1	6.61E-22	0.139214	0.094	0.048	1.59E-17	2.5	APMAP	1.958333333
GUSB4	6.88E-22	0.1173346	0.084	0.041	1.66E-17	2.5	GUSB	2.048780488
ZBTB8OS2	7.66E-22	0.166333	0.217	0.143	1.85E-17	2.5	ZBTB8OS	1.517482517
UBE2B1	7.75E-22	0.1789989	0.352	0.258	1.87E-17	2.5	UBE2B	1.364341085
MAP2K44	8.55E-22	0.1914667	0.542	0.43	2.06E-17	2.5	MAP2K4	1.260465116
SLC35B21	9.30E-22	0.1044503	0.053	0.021	2.24E-17	2.5	SLC35B2	2.523809524
FIG41	9.30E-22	0.1032554	0.061	0.026	2.24E-17	2.5	FIG4	2.346153846
PIKFYVE2	9.35E-22	0.1570686	0.178	0.111	2.26E-17	2.5	PIKFYVE	1.603603604
PHIP2	9.81E-22	0.1776326	0.538	0.428	2.37E-17	2.5	PHIP	1.257009346
ATG102	1.09E-21	0.1710814	0.208	0.136	2.64E-17	2.5	ATG10	1.529411765
CDKN1B1	1.27E-21	0.1551856	0.13	0.075	3.07E-17	2.5	CDKN1B	1.733333333
YTHDC21	1.34E-21	0.1216111	0.084	0.042	3.22E-17	2.5	YTHDC2	2
GRN2	1.51E-21	0.1086882	0.071	0.033	3.64E-17	2.5	GRN	2.151515152
ABCB71	1.54E-21	0.1357677	0.124	0.07	3.72E-17	2.5	ABCB7	1.771428571
FNDC3A1	1.59E-21	0.2108293	0.46	0.358	3.84E-17	2.5	FNDC3A	1.284916201
ZNF2743	1.76E-21	0.1571299	0.138	0.082	4.23E-17	2.5	ZNF274	1.682926829
NFYC1	1.78E-21	0.1137969	0.075	0.035	4.30E-17	2.5	NFYC	2.142857143
ICE22	1.80E-21	0.1326626	0.09	0.046	4.33E-17	2.5	ICE2	1.956521739
SLC1A2	1.90E-21	0.1023425	0.042	0.015	4.58E-17	2.5	SLC1A2	2.8
C2CD32	2.10E-21	0.1405017	0.113	0.062	5.06E-17	2.5	C2CD3	1.822580645
FAM134C1	2.44E-21	0.1302242	0.092	0.047	5.89E-17	2.5	FAM134C	1.957446809
CCDC1701	2.90E-21	0.1371989	0.098	0.051	7.00E-17	2.5	CCDC170	1.921568627
BBX2	3.08E-21	0.1615371	0.499	0.387	7.42E-17	2.5	BBX	1.289405685
C14orf12	3.22E-21	0.1648027	0.124	0.071	7.76E-17	2.5	C14orf1	1.746478873

DOCK91	3.46E-21	0.1422621	0.299	0.21	8.34E-17	2.5	DOCK9	1.423809524
POLR2G3	3.68E-21	0.1691819	0.242	0.165	8.89E-17	2.5	POLR2G	1.466666667
NANS1	3.70E-21	0.1475756	0.081	0.04	8.91E-17	2.5	NANS	2.025
ZNF280D2	3.71E-21	0.1580359	0.14	0.083	8.94E-17	2.5	ZNF280D	1.686746988
KPNA32	3.98E-21	0.1453244	0.189	0.12	9.59E-17	2.5	KPNA3	1.575
CNNM22	4.05E-21	0.155947	0.112	0.062	9.78E-17	2.5	CNNM2	1.806451613
POLR2K2	4.43E-21	0.1495915	0.466	0.356	1.07E-16	2.5	POLR2K	1.308988764
LAMP14	4.74E-21	0.1378661	0.151	0.09	1.14E-16	2.5	LAMP1	1.677777778
GTF3C22	4.75E-21	0.1487635	0.119	0.068	1.15E-16	2.5	GTF3C2	1.75
NEDD95	5.04E-21	0.1502095	0.469	0.359	1.21E-16	2.5	NEDD9	1.306406685
FANCC2	5.22E-21	0.1695598	0.189	0.121	1.26E-16	2.5	FANCC	1.561983471
EXTL31	5.81E-21	0.1670151	0.145	0.088	1.40E-16	2.5	EXTL3	1.647727273
SNX292	5.92E-21	0.1296999	0.151	0.091	1.43E-16	2.5	SNX29	1.659340659
MAPK142	5.98E-21	0.1771095	0.218	0.147	1.44E-16	2.5	MAPK14	1.482993197
EPS153	6.40E-21	0.1733651	0.276	0.194	1.54E-16	2.5	EPS15	1.422680412
MESDC2	6.50E-21	0.1315666	0.105	0.057	1.57E-16	2.5	MESDC2	1.842105263
HMGXB41	6.79E-21	0.165513	0.302	0.216	1.64E-16	2.5	HMGXB4	1.398148148
DCTN41	7.12E-21	0.1759748	0.249	0.171	1.72E-16	2.5	DCTN4	1.456140351
ACSF23	7.82E-21	0.1129924	0.07	0.033	1.89E-16	2.5	ACSF2	2.121212121
UBR31	8.14E-21	0.1393533	0.27	0.186	1.96E-16	2.5	UBR3	1.451612903
NFYA2	8.32E-21	0.1254248	0.124	0.071	2.01E-16	2.5	NFYA	1.746478873
MGME11	8.67E-21	0.1383268	0.124	0.071	2.09E-16	2.5	MGME1	1.746478873
NEK93	8.77E-21	0.1332049	0.109	0.06	2.11E-16	2.5	NEK9	1.816666667
SLC36A41	8.77E-21	0.1366782	0.145	0.086	2.11E-16	2.5	SLC36A4	1.686046512
DCP22	8.78E-21	0.1222081	0.249	0.168	2.12E-16	2.5	DCP2	1.482142857
SLC6A63	8.96E-21	0.1279325	0.106	0.058	2.16E-16	2.5	SLC6A6	1.827586207
DNM1L3	9.70E-21	0.1445826	0.177	0.112	2.34E-16	2.5	DNM1L	1.580357143
ZNF694	9.73E-21	0.1309419	0.1	0.054	2.35E-16	2.5	ZNF69	1.851851852
YAP12	9.85E-21	0.1644219	0.64	0.52	2.38E-16	2.5	YAP1	1.230769231
UGGT1	1.02E-20	0.1134124	0.101	0.054	2.45E-16	2.5	UGGT1	1.87037037
SLC25A132	1.08E-20	0.1469001	0.115	0.065	2.60E-16	2.5	SLC25A13	1.769230769
MID21	1.10E-20	0.1198001	0.103	0.056	2.65E-16	2.5	MID2	1.839285714
NETO21	1.12E-20	0.1252275	0.094	0.049	2.71E-16	2.5	NETO2	1.918367347
LRRC4C1	1.19E-20	0.1379339	0.097	0.051	2.86E-16	2.5	LRRC4C	1.901960784
FCF12	1.27E-20	0.1388859	0.11	0.061	3.07E-16	2.5	FCF1	1.803278689
ZMPSTE243	1.28E-20	0.1173209	0.121	0.068	3.08E-16	2.5	ZMPSTE24	1.779411765
HPS41	1.44E-20	0.1304846	0.079	0.039	3.47E-16	2.5	HPS4	2.025641026
MEF2D1	1.44E-20	0.1380441	0.111	0.062	3.48E-16	2.5	MEF2D	1.790322581
PIK3IP12	1.50E-20	0.1356017	0.104	0.057	3.62E-16	2.5	PIK3IP1	1.824561404
CYSTM11	1.52E-20	0.1708122	0.281	0.198	3.66E-16	2.5	CYSTM1	1.419191919
AMMECR11	1.71E-20	0.1135165	0.071	0.034	4.12E-16	2.5	AMMECR1	2.088235294
CACNA1D	1.82E-20	0.1079173	0.043	0.016	4.38E-16	2.5	CACNA1D	2.6875
ELF23	1.92E-20	0.1570351	0.484	0.376	4.62E-16	2.5	ELF2	1.287234043
ARHGEF112	1.95E-20	0.1497215	0.094	0.05	4.69E-16	2.5	ARHGEF11	1.88
RPTOR1	2.01E-20	0.1236308	0.104	0.056	4.86E-16	2.5	RPTOR	1.857142857
HOXA9	2.02E-20	0.112669	0.056	0.024	4.87E-16	2.5	HOXA9	2.333333333
CLTC1	2.06E-20	0.1529315	0.319	0.229	4.96E-16	2.5	CLTC	1.3930131
BLVRA	2.24E-20	0.1623999	0.155	0.096	5.41E-16	2.5	BLVRA	1.614583333
APH1B1	2.29E-20	0.1127686	0.07	0.033	5.53E-16	2.5	APH1B	2.121212121
CCDC1731	2.31E-20	0.1444069	0.112	0.063	5.57E-16	2.5	CCDC173	1.777777778
RFX33	2.37E-20	0.183643	0.233	0.16	5.70E-16	2.5	RFX3	1.45625
ADD33	2.43E-20	0.1733102	0.266	0.187	5.85E-16	2.5	ADD3	1.422459893

MARCKSL13	2.43E-20	0.1060159	0.219	0.144	5.87E-16	2.5	MARCKSL1	1.520833333
CFAP432	2.48E-20	0.1096272	0.07	0.033	5.98E-16	2.5	CFAP43	2.121212121
SNX143	2.51E-20	0.1681593	0.175	0.111	6.05E-16	2.5	SNX14	1.576576577
ACP61	2.52E-20	0.1038137	0.059	0.026	6.08E-16	2.5	ACP6	2.269230769
EEF2K1	2.95E-20	0.1303338	0.079	0.039	7.12E-16	2.5	EEF2K	2.025641026
RALGAPB1	2.98E-20	0.1504761	0.216	0.144	7.20E-16	2.5	RALGAPB	1.5
CUL33	3.10E-20	0.1677638	0.498	0.391	7.47E-16	2.5	CUL3	1.273657289
RPN24	3.11E-20	0.1352325	0.355	0.257	7.50E-16	2.5	RPN2	1.381322957
ASTN22	3.20E-20	0.1421297	0.364	0.262	7.73E-16	2.5	ASTN2	1.389312977
MGLL2	3.58E-20	0.1430976	0.257	0.175	8.64E-16	2.5	MGLL	1.468571429
C2CD51	3.76E-20	0.1042683	0.057	0.025	9.06E-16	2.5	C2CD5	2.28
STIM2	3.99E-20	0.1126112	0.068	0.032	9.62E-16	2.5	STIM2	2.125
RP11-123O10.43	4.20E-20	0.1667236	0.285	0.203	1.01E-15	2.5	RP11-123O	1.403940887
NPLOC41	4.77E-20	0.169798	0.216	0.146	1.15E-15	2.5	NPLOC4	1.479452055
ZNF7303	4.87E-20	0.1297502	0.133	0.078	1.17E-15	2.5	ZNF730	1.705128205
MAP3K42	5.05E-20	0.1534444	0.186	0.12	1.22E-15	2.5	MAP3K4	1.55
RIC12	5.18E-20	0.1359347	0.245	0.168	1.25E-15	2.5	RIC1	1.458333333
RRAGB1	5.19E-20	0.1410468	0.137	0.082	1.25E-15	2.5	RRAGB	1.670731707
MRPS212	5.48E-20	0.1620488	0.619	0.504	1.32E-15	2.5	MRPS21	1.228174603
C20orf1943	5.48E-20	0.1567166	0.238	0.164	1.32E-15	2.5	C20orf194	1.451219512
C5orf241	5.56E-20	0.1198997	0.09	0.047	1.34E-15	2.5	C5orf24	1.914893617
DTNBP11	5.57E-20	0.1288189	0.111	0.062	1.34E-15	2.5	DTNBP1	1.790322581
C5orf151	5.63E-20	0.1268863	0.128	0.075	1.36E-15	2.5	C5orf15	1.706666667
CHDH2	5.89E-20	0.139202	0.088	0.046	1.42E-15	2.5	CHDH	1.913043478
APOL21	6.72E-20	0.1200668	0.071	0.035	1.62E-15	2.5	APOL2	2.028571429
MTCH22	7.40E-20	0.1397779	0.121	0.07	1.78E-15	2.5	MTCH2	1.728571429
DAXX2	7.65E-20	0.1292417	0.092	0.049	1.84E-15	2.5	DAXX	1.87755102
HSBP1L1	8.28E-20	0.1084126	0.057	0.025	2.00E-15	2.5	HSBP1L1	2.28
HIPK33	8.47E-20	0.1549343	0.261	0.183	2.04E-15	2.5	HIPK3	1.426229508
RND12	8.52E-20	0.1507537	0.131	0.077	2.06E-15	2.5	RND1	1.701298701
MARCH64	8.68E-20	0.1485237	0.499	0.388	2.09E-15	2.5	MARCH6	1.286082474
PURA1	8.93E-20	0.1452957	0.11	0.063	2.15E-15	2.5	PURA	1.746031746
TMEM331	9.17E-20	0.1407603	0.163	0.102	2.21E-15	2.5	TMEM33	1.598039216
RNF1112	9.64E-20	0.1640235	0.345	0.254	2.33E-15	2.5	RNF111	1.358267717
PEMT4	9.64E-20	0.1771256	0.072	0.035	2.33E-15	2.5	PEMT	2.057142857
WWC33	1.09E-19	0.128521	0.125	0.073	2.63E-15	2.5	WWC3	1.712328767
PSMD131	1.09E-19	0.1407688	0.139	0.084	2.64E-15	2.5	PSMD13	1.654761905
GPBP1L12	1.13E-19	0.1535692	0.434	0.332	2.73E-15	2.5	GPBP1L1	1.307228916
SLC10A72	1.26E-19	0.152207	0.093	0.05	3.05E-15	2.5	SLC10A7	1.86
ZNF1381	1.44E-19	0.1476917	0.115	0.066	3.48E-15	2.5	ZNF138	1.742424242
SLC22A53	1.47E-19	0.1264858	0.084	0.043	3.54E-15	2.5	SLC22A5	1.953488372
SPTBN11	1.47E-19	0.152865	0.294	0.21	3.54E-15	2.5	SPTBN1	1.4
DPY19L3	1.55E-19	0.115928	0.071	0.035	3.73E-15	2.5	DPY19L3	2.028571429
AATF2	1.72E-19	0.129257	0.082	0.042	4.16E-15	2.5	AATF	1.952380952
FAM174A2	1.73E-19	0.1089379	0.061	0.028	4.17E-15	2.5	FAM174A	2.178571429
ATXN7L12	1.74E-19	0.1236376	0.105	0.059	4.20E-15	2.5	ATXN7L1	1.779661017
HLA-A2	1.86E-19	0.1557964	0.404	0.307	4.50E-15	2.5	HLA-A	1.315960912
STXBP52	1.89E-19	0.1464073	0.183	0.119	4.57E-15	2.5	STXBP5	1.537815126
SSR1	1.92E-19	0.1691212	0.273	0.194	4.63E-15	2.5	SSR1	1.407216495
UBXN2B1	1.98E-19	0.1168551	0.089	0.047	4.78E-15	2.5	UBXN2B	1.893617021
RP11-420A23.13	2.06E-19	0.1146287	0.081	0.041	4.96E-15	2.5	RP11-420A	1.975609756
PTTG1IP1	2.33E-19	0.1423471	0.231	0.157	5.62E-15	2.5	PTTG1IP	1.47133758

DDX46	2.58E-19	0.1323669	0.165	0.105	6.22E-15	2.5	DDX46	1.571428571
SNAP291	2.62E-19	0.1064253	0.08	0.04	6.31E-15	2.5	SNAP29	2
MDM42	2.68E-19	0.1841559	0.323	0.239	6.46E-15	2.5	MDM4	1.351464435
PPM1L2	2.68E-19	0.139675	0.118	0.069	6.47E-15	2.5	PPM1L	1.710144928
RNF135	2.93E-19	0.1060213	0.06	0.027	7.07E-15	2.5	RNF135	2.222222222
ANAPC53	2.93E-19	0.130634	0.161	0.101	7.07E-15	2.5	ANAPC5	1.594059406
PSAP3	3.03E-19	0.1460522	0.178	0.115	7.31E-15	2.5	PSAP	1.547826087
NUP581	3.09E-19	0.1367573	0.196	0.128	7.44E-15	2.5	NUP58	1.53125
ABCF1	3.32E-19	0.1225683	0.128	0.076	8.00E-15	2.5	ABCF1	1.684210526
SS18L11	3.39E-19	0.1131925	0.066	0.031	8.18E-15	2.5	SS18L1	2.129032258
SRGAP23	3.39E-19	0.1429465	0.208	0.139	8.18E-15	2.5	SRGAP2	1.496402878
BIRC62	3.39E-19	0.1443632	0.671	0.55	8.18E-15	2.5	BIRC6	1.22
TUBG21	3.45E-19	0.1187249	0.071	0.035	8.33E-15	2.5	TUBG2	2.028571429
EML21	3.49E-19	0.1351035	0.093	0.05	8.41E-15	2.5	EML2	1.86
TMEM1812	3.52E-19	0.1590602	0.311	0.227	8.49E-15	2.5	TMEM181	1.370044053
WSB21	3.60E-19	0.1319414	0.137	0.082	8.68E-15	2.5	WSB2	1.670731707
TSPAN91	3.64E-19	0.1071123	0.145	0.088	8.78E-15	2.5	TSPAN9	1.647727273
IL1R12	3.81E-19	0.106454	0.116	0.066	9.18E-15	2.5	IL1R1	1.757575758
PEX23	3.83E-19	0.1536401	0.157	0.099	9.23E-15	2.5	PEX2	1.585858586
ATP6V1E11	3.91E-19	0.1482472	0.378	0.282	9.42E-15	2.5	ATP6V1E1	1.340425532
TMEM62	3.91E-19	0.1111555	0.08	0.041	9.43E-15	2.5	TMEM62	1.951219512
VPS411	3.95E-19	0.1507658	0.147	0.091	9.53E-15	2.5	VPS41	1.615384615
MIPEP3	4.62E-19	0.1184241	0.095	0.052	1.11E-14	2.5	MIPEP	1.826923077
MLLT62	4.70E-19	0.1306689	0.139	0.084	1.13E-14	2.5	MLLT6	1.654761905
ZNRF22	4.71E-19	0.1190436	0.346	0.252	1.13E-14	2.5	ZNRF2	1.373015873
POLB	4.71E-19	0.1484354	0.128	0.076	1.14E-14	2.5	POLB	1.684210526
FOCAD2	4.81E-19	0.1718173	0.226	0.156	1.16E-14	2.5	FOCAD	1.448717949
S100A163	4.92E-19	0.172285	0.218	0.147	1.19E-14	2.5	S100A16	1.482993197
ACBD51	5.26E-19	0.1011825	0.06	0.028	1.27E-14	2.5	ACBD5	2.142857143
IRS14	5.28E-19	0.1274877	0.146	0.089	1.27E-14	2.5	IRS1	1.640449438
NIF3L12	5.28E-19	0.1151048	0.096	0.052	1.27E-14	2.5	NIF3L1	1.846153846
VTA11	5.38E-19	0.1561736	0.16	0.101	1.30E-14	2.5	VTA1	1.584158416
ELMOD32	5.43E-19	0.1515693	0.134	0.082	1.31E-14	2.5	ELMOD3	1.634146341
SUMF22	5.72E-19	0.1429005	0.142	0.087	1.38E-14	2.5	SUMF2	1.632183908
ID22	5.80E-19	0.1096707	0.158	0.099	1.40E-14	2.5	ID2	1.595959596
NFX12	6.39E-19	0.1615723	0.211	0.144	1.54E-14	2.5	NFX1	1.465277778
PTPRT1	6.43E-19	0.1390635	0.089	0.047	1.55E-14	2.5	PTPRT	1.893617021
MAPK104	6.64E-19	0.1212938	0.08	0.041	1.60E-14	2.5	MAPK10	1.951219512
RABL3	6.91E-19	0.1075948	0.089	0.047	1.67E-14	2.5	RABL3	1.893617021
ZBTB443	7.16E-19	0.1357956	0.203	0.135	1.73E-14	2.5	ZBTB44	1.503703704
TTF21	7.25E-19	0.1113433	0.083	0.043	1.75E-14	2.5	TTF2	1.930232558
MYH143	7.26E-19	0.16562	0.221	0.153	1.75E-14	2.5	MYH14	1.444444444
ZC3H132	7.93E-19	0.14778	0.268	0.19	1.91E-14	2.5	ZC3H13	1.410526316
ZSCAN18	8.38E-19	0.1067008	0.069	0.034	2.02E-14	2.5	ZSCAN18	2.029411765
PDCD6IP1	8.69E-19	0.1333576	0.253	0.177	2.10E-14	2.5	PDCD6IP	1.429378531
AQR1	9.17E-19	0.1485113	0.153	0.096	2.21E-14	2.5	AQR	1.59375
FBXO361	9.35E-19	0.1165877	0.092	0.049	2.25E-14	2.5	FBXO36	1.87755102
SHISA62	9.49E-19	0.1019993	0.048	0.02	2.29E-14	2.5	SHISA6	2.4
HDGF3	9.84E-19	0.1872036	0.341	0.257	2.37E-14	2.5	HDGF	1.326848249
KLF42	9.88E-19	0.1337542	0.166	0.105	2.38E-14	2.5	KLF4	1.580952381
ZBTB40	1.00E-18	0.1057772	0.05	0.021	2.42E-14	2.5	ZBTB40	2.380952381
DYNLRB13	1.07E-18	0.181954	0.394	0.302	2.57E-14	2.5	DYNLRB1	1.304635762

RP11-1379J22.21	1.13E-18	0.1349471	0.107	0.06	2.73E-14	2.5	RP11-1379.	1.783333333
CUL11	1.15E-18	0.1549009	0.178	0.116	2.78E-14	2.5	CUL1	1.534482759
FAM155A3	1.16E-18	0.1556815	0.097	0.054	2.79E-14	2.5	FAM155A	1.796296296
COMMD13	1.24E-18	0.1746196	0.132	0.08	3.00E-14	2.5	COMMD1	1.65
HNRNPUL1	1.37E-18	0.140262	0.175	0.114	3.31E-14	2.5	HNRNPUL1	1.535087719
SNRNP352	1.44E-18	0.1368317	0.119	0.07	3.48E-14	2.5	SNRNP35	1.7
CCDC733	1.45E-18	0.1459909	0.16	0.1	3.51E-14	2.5	CCDC73	1.6
DUSP3	1.47E-18	0.1301201	0.115	0.067	3.54E-14	2.5	DUSP3	1.71641791
P2RX41	1.56E-18	0.1315686	0.124	0.074	3.77E-14	2.5	P2RX4	1.675675676
HN1L1	1.63E-18	0.1294281	0.122	0.072	3.92E-14	2.5	HN1L	1.694444444
PIAS12	1.69E-18	0.1172515	0.583	0.472	4.06E-14	2.5	PIAS1	1.235169492
MKNK11	1.72E-18	0.1366033	0.111	0.064	4.16E-14	2.5	MKNK1	1.734375
EP4002	1.83E-18	0.1405409	0.144	0.089	4.42E-14	2.5	EP400	1.617977528
ZNF4802	1.93E-18	0.1056775	0.09	0.049	4.66E-14	2.5	ZNF480	1.836734694
NEK61	2.11E-18	0.1076843	0.066	0.032	5.10E-14	2.5	NEK6	2.0625
ITGA103	2.29E-18	0.1339511	0.091	0.049	5.51E-14	2.5	ITGA10	1.857142857
ARHGEF263	2.29E-18	0.1023442	0.156	0.098	5.53E-14	2.5	ARHGEF26	1.591836735
CWF19L22	2.57E-18	0.148398	0.208	0.141	6.19E-14	2.5	CWF19L2	1.475177305
STXBP31	2.67E-18	0.1297505	0.16	0.102	6.43E-14	2.5	STXBP3	1.568627451
CMSS11	2.74E-18	0.1283519	0.168	0.108	6.60E-14	2.5	CMSS1	1.555555556
CNDP23	2.79E-18	0.1187258	0.181	0.118	6.72E-14	2.5	CNDP2	1.533898305
UPF22	2.93E-18	0.1646332	0.374	0.282	7.07E-14	2.5	UPF2	1.326241135
MED23	2.94E-18	0.1387145	0.136	0.084	7.10E-14	2.5	MED23	1.619047619
DOCK53	2.97E-18	0.1300616	0.278	0.199	7.16E-14	2.5	DOCK5	1.396984925
BCLAF1	3.00E-18	0.157537	0.467	0.367	7.24E-14	2.5	BCLAF1	1.272479564
SLC19A22	3.40E-18	0.1619647	0.174	0.114	8.20E-14	2.5	SLC19A2	1.526315789
GPR1604	3.48E-18	0.132985	0.144	0.089	8.39E-14	2.5	GPR160	1.617977528
TMEM38B1	3.60E-18	0.1151554	0.082	0.043	8.68E-14	2.5	TMEM38B	1.906976744
TRIP122	3.97E-18	0.1467746	0.413	0.317	9.56E-14	2.5	TRIP12	1.302839117
MTMR91	4.11E-18	0.1219021	0.079	0.041	9.92E-14	2.5	MTMR9	1.926829268
RBM412	4.19E-18	0.1337136	0.115	0.068	1.01E-13	2.5	RBM41	1.691176471
OSTF11	4.87E-18	0.1044753	0.078	0.04	1.18E-13	2.5	OSTF1	1.95
RIPK11	5.06E-18	0.1038745	0.095	0.053	1.22E-13	2.5	RIPK1	1.79245283
CLK41	5.09E-18	0.1315015	0.113	0.066	1.23E-13	2.5	CLK4	1.712121212
DDX172	5.29E-18	0.1708541	0.512	0.415	1.28E-13	2.5	DDX17	1.23373494
SELT3	5.33E-18	0.1116233	0.121	0.072	1.29E-13	2.5	SELT	1.680555556
INTU1	5.72E-18	0.107247	0.09	0.048	1.38E-13	2.5	INTU	1.875
STK38L2	6.11E-18	0.1184534	0.264	0.186	1.47E-13	2.5	STK38L	1.419354839
GON4L1	6.21E-18	0.1533534	0.211	0.145	1.50E-13	2.5	GON4L	1.455172414
DDX101	6.28E-18	0.1390414	0.128	0.078	1.51E-13	2.5	DDX10	1.641025641
HERPUD21	6.38E-18	0.116281	0.106	0.061	1.54E-13	2.5	HERPUD2	1.737704918
RAB7A3	6.80E-18	0.1116513	0.548	0.439	1.64E-13	2.5	RAB7A	1.248291572
RSPH31	6.82E-18	0.1379229	0.119	0.071	1.64E-13	2.5	RSPH3	1.676056338
TSG1012	7.00E-18	0.155162	0.234	0.164	1.69E-13	2.5	TSG101	1.426829268
STAT62	7.09E-18	0.1051443	0.129	0.079	1.71E-13	2.5	STAT6	1.632911392
RUFY12	7.38E-18	0.1357024	0.155	0.099	1.78E-13	2.5	RUFY1	1.565656566
TXNDC162	8.57E-18	0.1212687	0.094	0.052	2.07E-13	2.5	TXNDC16	1.807692308
SENP61	9.53E-18	0.1398379	0.392	0.3	2.30E-13	2.5	SENP6	1.306666667
ERC21	9.59E-18	0.1443736	0.158	0.101	2.31E-13	2.5	ERC2	1.564356436
RAB282	9.73E-18	0.1188548	0.094	0.052	2.35E-13	2.5	RAB28	1.807692308
ATL11	9.84E-18	0.1120235	0.098	0.055	2.37E-13	2.5	ATL1	1.781818182
C1orf43	1.04E-17	0.1507873	0.189	0.127	2.51E-13	2.5	C1orf43	1.488188976

RP11-289H16.11	1.05E-17	0.1153118	0.101	0.057	2.54E-13	2.5	RP11-289H	1.771929825
MAP3K21	1.06E-17	0.1689107	0.253	0.182	2.55E-13	2.5	MAP3K2	1.39010989
NDUFS11	1.07E-17	0.139175	0.207	0.141	2.57E-13	2.5	NDUFS1	1.468085106
NBPF111	1.09E-17	0.1360384	0.112	0.066	2.64E-13	2.5	NBPF11	1.696969697
RP11-665G4.13	1.22E-17	0.1024339	0.136	0.083	2.93E-13	2.5	RP11-665G	1.638554217
SERPINB62	1.22E-17	0.1124929	0.093	0.051	2.94E-13	2.5	SERPINB6	1.823529412
MYEF22	1.31E-17	0.1424629	0.137	0.085	3.15E-13	2.5	MYEF2	1.611764706
POLR2A2	1.34E-17	0.1226641	0.229	0.159	3.23E-13	2.5	POLR2A	1.440251572
PRPF4B2	1.35E-17	0.1349232	0.249	0.176	3.26E-13	2.5	PRPF4B	1.414772727
YPEL53	1.35E-17	0.1525011	0.49	0.387	3.26E-13	2.5	YPEL5	1.266149871
IRAK41	1.38E-17	0.1129906	0.067	0.033	3.32E-13	2.5	IRAK4	2.03030303
MGA2	1.38E-17	0.1223694	0.107	0.062	3.33E-13	2.5	MGA	1.725806452
RPRD1B	1.49E-17	0.1039306	0.094	0.052	3.60E-13	2.5	RPRD1B	1.807692308
RNF1413	1.52E-17	0.1099095	0.099	0.055	3.66E-13	2.5	RNF141	1.8
SP33	1.72E-17	0.1215972	0.284	0.205	4.14E-13	2.5	SP3	1.385365854
TPP21	1.74E-17	0.119239	0.151	0.096	4.19E-13	2.5	TPP2	1.572916667
UBE4A1	1.84E-17	0.1513655	0.175	0.115	4.43E-13	2.5	UBE4A	1.52173913
FAM83F1	1.99E-17	0.1084088	0.072	0.036	4.81E-13	2.5	FAM83F	2
LMCD1-AS12	2.01E-17	0.1268813	0.08	0.042	4.85E-13	2.5	LMCD1-AS1	1.904761905
KIAA05562	2.06E-17	0.1169533	0.154	0.099	4.98E-13	2.5	KIAA0556	1.555555556
ARL6IP54	2.07E-17	0.143946	0.585	0.475	5.00E-13	2.5	ARL6IP5	1.231578947
FAM21A1	2.09E-17	0.1012972	0.061	0.029	5.04E-13	2.5	FAM21A	2.103448276
PIAS32	2.24E-17	0.1060483	0.07	0.036	5.41E-13	2.5	PIAS3	1.944444444
DENND5B1	2.40E-17	0.1436581	0.143	0.09	5.79E-13	2.5	DENND5B	1.588888889
CLSTN12	2.41E-17	0.1317174	0.133	0.082	5.81E-13	2.5	CLSTN1	1.62195122
HTATIP22	2.58E-17	0.1260621	0.14	0.088	6.21E-13	2.5	HTATIP2	1.590909091
NVL2	2.62E-17	0.1152147	0.103	0.059	6.32E-13	2.5	NVL	1.745762712
MTERF11	2.80E-17	0.1292759	0.13	0.079	6.75E-13	2.5	MTERF1	1.64556962
GTF2IRD2B1	2.83E-17	0.1271223	0.124	0.075	6.81E-13	2.5	GTF2IRD2B	1.653333333
TMEM2602	3.00E-17	0.1098572	0.101	0.058	7.24E-13	2.5	TMEM260	1.74137931
ATRN1	3.10E-17	0.1134518	0.124	0.075	7.48E-13	2.5	ATRN	1.653333333
LINC004753	3.23E-17	0.1350133	0.13	0.08	7.80E-13	2.5	LINC00475	1.625
AC037445.11	3.33E-17	0.1022928	0.092	0.051	8.02E-13	2.5	AC037445.	1.803921569
DNAJC27	3.34E-17	0.1246859	0.071	0.036	8.06E-13	2.5	DNAJC27	1.972222222
MAPK63	3.37E-17	0.1334465	0.351	0.263	8.12E-13	2.5	MAPK6	1.33460076
SLC9A82	3.52E-17	0.1368479	0.132	0.082	8.48E-13	2.5	SLC9A8	1.609756098
STX172	3.54E-17	0.1499145	0.169	0.113	8.54E-13	2.5	STX17	1.495575221
PKN22	3.56E-17	0.137254	0.337	0.255	8.59E-13	2.5	PKN2	1.321568627
SMARCAD12	3.82E-17	0.1374989	0.098	0.056	9.21E-13	2.5	SMARCAD1	1.75
COQ10B	4.13E-17	0.1298847	0.188	0.125	9.95E-13	2.5	COQ10B	1.504
CDK193	4.15E-17	0.1414536	0.401	0.309	1.00E-12	2.5	CDK19	1.297734628
FDFT12	4.91E-17	0.1868205	0.393	0.304	1.18E-12	2.5	FDFT1	1.292763158
TAGLN22	4.91E-17	0.1575355	0.287	0.211	1.18E-12	2.5	TAGLN2	1.360189573
ROCK11	5.41E-17	0.1506663	0.305	0.226	1.31E-12	2.5	ROCK1	1.349557522
SH3BP43	5.43E-17	0.1316577	0.22	0.151	1.31E-12	2.5	SH3BP4	1.456953642
DNM22	6.11E-17	0.1289365	0.142	0.089	1.47E-12	2.5	DNM2	1.595505618
MTA32	6.32E-17	0.1520629	0.2	0.137	1.52E-12	2.5	MTA3	1.459854015
ACOT71	6.70E-17	0.1180952	0.047	0.02	1.61E-12	2.5	ACOT7	2.35
RHOC1	6.87E-17	0.1184777	0.106	0.061	1.66E-12	2.5	RHOC	1.737704918
RFWD32	7.00E-17	0.1180561	0.087	0.048	1.69E-12	2.5	RFWD3	1.8125
CLCN32	7.14E-17	0.1531663	0.226	0.16	1.72E-12	2.5	CLCN3	1.4125
LINC015721	7.16E-17	0.1114753	0.075	0.039	1.73E-12	2.5	LINC01572	1.923076923

CNOT6L2	7.23E-17	0.1033133	0.162	0.105	1.74E-12	2.5	CNOT6L	1.542857143
RP11-368L12.12	7.41E-17	0.1546462	0.151	0.097	1.79E-12	2.5	RP11-368L:	1.556701031
LUZP12	7.46E-17	0.1253271	0.169	0.111	1.80E-12	2.5	LUZP1	1.522522523
OLMALINC1	7.65E-17	0.1078015	0.075	0.04	1.84E-12	2.5	OLMALINC	1.875
ZNF434	7.72E-17	0.1390345	0.186	0.126	1.86E-12	2.5	ZNF43	1.476190476
MDK2	7.79E-17	0.1076111	0.059	0.028	1.88E-12	2.5	MDK	2.107142857
ABAT2	8.00E-17	0.1157664	0.095	0.053	1.93E-12	2.5	ABAT	1.79245283
MZF1-AS11	8.10E-17	0.1062759	0.062	0.031	1.95E-12	2.5	MZF1-AS1	2
NRIP32	8.19E-17	0.1173324	0.076	0.04	1.97E-12	2.5	NRIP3	1.9
SAP1302	9.39E-17	0.131331	0.131	0.081	2.26E-12	2.5	SAP130	1.617283951
RAB132	9.58E-17	0.1303626	0.199	0.136	2.31E-12	2.5	RAB13	1.463235294
RABGAP1L3	9.61E-17	0.1135674	0.299	0.219	2.32E-12	2.5	RABGAP1L	1.365296804
MPV171	9.65E-17	0.1102255	0.082	0.044	2.33E-12	2.5	MPV17	1.863636364
ALOX15B3	1.01E-16	0.1210574	0.092	0.051	2.44E-12	2.5	ALOX15B	1.803921569
ZFX1	1.01E-16	0.1354137	0.168	0.112	2.44E-12	2.5	ZFX	1.5
PSMB32	1.04E-16	0.1023732	0.137	0.085	2.51E-12	2.5	PSMB3	1.611764706
GLG12	1.04E-16	0.1299153	0.256	0.184	2.51E-12	2.5	GLG1	1.391304348
ZNF5442	1.05E-16	0.1342081	0.129	0.08	2.52E-12	2.5	ZNF544	1.6125
ZDHHC31	1.07E-16	0.1091548	0.088	0.049	2.57E-12	2.5	ZDHHC3	1.795918367
NUCB23	1.10E-16	0.1138533	0.184	0.122	2.64E-12	2.5	NUCB2	1.508196721
JKAMP3	1.10E-16	0.1214975	0.134	0.083	2.65E-12	2.5	JKAMP	1.614457831
MYH101	1.10E-16	0.1148291	0.107	0.062	2.66E-12	2.5	MYH10	1.725806452
CAPN71	1.13E-16	0.1282621	0.199	0.137	2.72E-12	2.5	CAPN7	1.452554745
ZCCHC142	1.14E-16	0.1224324	0.109	0.064	2.75E-12	2.5	ZCCHC14	1.703125
MTMR123	1.16E-16	0.1238774	0.138	0.087	2.80E-12	2.5	MTMR12	1.586206897
GCC24	1.17E-16	0.1445853	0.384	0.296	2.82E-12	2.5	GCC2	1.297297297
ACTR101	1.18E-16	0.1370965	0.198	0.135	2.85E-12	2.5	ACTR10	1.466666667
SLF22	1.21E-16	0.1041358	0.072	0.037	2.92E-12	2.5	SLF2	1.945945946
PPP1R13B3	1.23E-16	0.1622464	0.253	0.184	2.97E-12	2.5	PPP1R13B	1.375
SLC25A461	1.33E-16	0.1036535	0.076	0.04	3.20E-12	2.5	SLC25A46	1.9
ZFHX33	1.34E-16	0.1432473	0.169	0.113	3.23E-12	2.5	ZFHX3	1.495575221
GLO13	1.35E-16	0.1047531	0.235	0.164	3.26E-12	2.5	GLO1	1.432926829
CBX32	1.37E-16	0.1727768	0.295	0.221	3.29E-12	2.5	CBX3	1.334841629
IKZF22	1.39E-16	0.1072295	0.073	0.038	3.35E-12	2.5	IKZF2	1.921052632
NCAPD21	1.45E-16	0.1126275	0.078	0.042	3.51E-12	2.5	NCAPD2	1.857142857
CTNNA11	1.46E-16	0.1120569	0.632	0.526	3.53E-12	2.5	CTNNA1	1.201520913
CASP21	1.52E-16	0.1300008	0.08	0.044	3.66E-12	2.5	CASP2	1.818181818
H1F02	1.60E-16	0.1718424	0.215	0.152	3.87E-12	2.5	H1F0	1.414473684
HSD17B43	1.62E-16	0.1215971	0.237	0.168	3.92E-12	2.5	HSD17B4	1.410714286
ASAP12	1.64E-16	0.1235755	0.373	0.283	3.96E-12	2.5	ASAP1	1.318021201
CTPS21	1.69E-16	0.1351187	0.11	0.066	4.08E-12	2.5	CTPS2	1.666666667
ALS21	1.71E-16	0.103668	0.066	0.033	4.13E-12	2.5	ALS2	2
NDUFB42	1.84E-16	0.1187386	0.526	0.423	4.43E-12	2.5	NDUFB4	1.243498818
CHST152	1.89E-16	0.1031039	0.069	0.036	4.56E-12	2.5	CHST15	1.916666667
RCOR31	1.93E-16	0.1278559	0.141	0.09	4.65E-12	2.5	RCOR3	1.566666667
SLC35D21	1.93E-16	0.1432924	0.122	0.075	4.65E-12	2.5	SLC35D2	1.626666667
C18orf323	2.08E-16	0.1228315	0.214	0.147	5.01E-12	2.5	C18orf32	1.455782313
HINT31	2.13E-16	0.1172919	0.103	0.06	5.15E-12	2.5	HINT3	1.716666667
RGL31	2.30E-16	0.1015744	0.06	0.03	5.54E-12	2.5	RGL3	2
NUP1332	2.32E-16	0.1028408	0.09	0.05	5.59E-12	2.5	NUP133	1.8
CCDC1742	2.65E-16	0.1484924	0.234	0.168	6.39E-12	2.5	CCDC174	1.392857143
ATP9A1	2.88E-16	0.1276859	0.155	0.101	6.94E-12	2.5	ATP9A	1.534653465

SELM1	2.99E-16	0.1700659	0.063	0.031	7.20E-12	2.5	SELM	2.032258065
ARFGEF11	3.00E-16	0.1622762	0.379	0.295	7.24E-12	2.5	ARFGEF1	1.284745763
PRR14L3	3.12E-16	0.1224976	0.142	0.091	7.52E-12	2.5	PRR14L	1.56043956
AP5M11	3.18E-16	0.1163268	0.112	0.068	7.67E-12	2.5	AP5M1	1.647058824
RNF1811	3.28E-16	0.1506373	0.469	0.376	7.91E-12	2.5	RNF181	1.247340426
NOL102	3.39E-16	0.1112333	0.164	0.108	8.16E-12	2.5	NOL10	1.518518519
TMEM1271	3.48E-16	0.1075206	0.088	0.049	8.40E-12	2.5	TMEM127	1.795918367
DENND4C1	3.56E-16	0.1745524	0.282	0.211	8.57E-12	2.5	DENND4C	1.336492891
REEP31	3.63E-16	0.1514347	0.341	0.261	8.75E-12	2.5	REEP3	1.30651341
KLHL23	3.65E-16	0.1270907	0.123	0.075	8.80E-12	2.5	KLHL23	1.64
ESCO12	3.66E-16	0.1355551	0.142	0.091	8.81E-12	2.5	ESCO1	1.56043956
LEPR1	3.74E-16	0.1141238	0.147	0.094	9.01E-12	2.5	LEPR	1.563829787
CHPT12	3.78E-16	0.129353	0.385	0.301	9.11E-12	2.5	CHPT1	1.279069767
TMOD33	3.95E-16	0.1127557	0.492	0.392	9.53E-12	2.5	TMOD3	1.255102041
PHF8	3.97E-16	0.1192757	0.128	0.08	9.56E-12	2.5	PHF8	1.6
SREBF23	4.17E-16	0.1387438	0.462	0.367	1.01E-11	2.5	SREBF2	1.258855586
PLD32	4.27E-16	0.1154467	0.081	0.045	1.03E-11	2.5	PLD3	1.8
AC018890.64	4.67E-16	0.1096859	0.312	0.23	1.13E-11	2.5	AC018890.(	1.356521739
ZHX22	4.86E-16	0.1146988	0.421	0.33	1.17E-11	2.5	ZHX2	1.275757576
RARB3	6.20E-16	0.1260879	0.107	0.064	1.49E-11	2.5	RARB	1.671875
ATAD21	6.44E-16	0.1268817	0.11	0.066	1.55E-11	2.5	ATAD2	1.666666667
SMAD23	6.65E-16	0.1329852	0.381	0.294	1.60E-11	2.5	SMAD2	1.295918367
ATRAID2	6.91E-16	0.1002627	0.082	0.045	1.67E-11	2.5	ATRAID	1.822222222
KDELR12	7.10E-16	0.1283016	0.158	0.104	1.71E-11	2.5	KDELR1	1.519230769
VAT12	7.59E-16	0.1428843	0.192	0.133	1.83E-11	2.5	VAT1	1.443609023
MYBL11	7.73E-16	0.1392903	0.113	0.068	1.86E-11	2.5	MYBL1	1.661764706
ZNF7141	7.88E-16	0.119232	0.073	0.039	1.90E-11	2.5	ZNF714	1.871794872
HTT2	7.92E-16	0.1078893	0.228	0.16	1.91E-11	2.5	HTT	1.425
MRPL421	8.72E-16	0.1442259	0.255	0.185	2.10E-11	2.5	MRPL42	1.378378378
CASC31	9.83E-16	0.1369623	0.207	0.145	2.37E-11	2.5	CASC3	1.427586207
IFNGR21	9.87E-16	0.1419531	0.377	0.293	2.38E-11	2.5	IFNGR2	1.28668942
TGFBR13	1.06E-15	0.1116621	0.164	0.109	2.55E-11	2.5	TGFBR1	1.504587156
CDS12	1.08E-15	0.1185926	0.178	0.12	2.60E-11	2.5	CDS1	1.483333333
ZNF241	1.08E-15	0.1528653	0.336	0.255	2.60E-11	2.5	ZNF24	1.317647059
SNRNP200	1.20E-15	0.1374	0.177	0.12	2.90E-11	2.5	SNRNP200	1.475
TYW11	1.22E-15	0.144736	0.134	0.085	2.95E-11	2.5	TYW1	1.576470588
R3HCC1L3	1.27E-15	0.1419049	0.181	0.124	3.06E-11	2.5	R3HCC1L	1.459677419
IFT742	1.36E-15	0.101917	0.077	0.042	3.27E-11	2.5	IFT74	1.833333333
GABARAP3	1.37E-15	0.1455953	0.442	0.352	3.30E-11	2.5	GABARAP	1.255681818
H2AFJ2	1.45E-15	0.2013477	0.068	0.036	3.49E-11	2.5	H2AFJ	1.888888889
DSTYK1	1.51E-15	0.1139585	0.097	0.057	3.64E-11	2.5	DSTYK	1.701754386
IRAK22	1.52E-15	0.1598825	0.178	0.122	3.67E-11	2.5	IRAK2	1.459016393
ZFPM21	1.59E-15	0.1168074	0.165	0.109	3.84E-11	2.5	ZFPM2	1.513761468
TNFRSF12A2	1.64E-15	0.1013506	0.11	0.066	3.94E-11	2.5	TNFRSF12A	1.666666667
MAK1	1.74E-15	0.1337974	0.134	0.086	4.20E-11	2.5	MAK	1.558139535
SRSF54	1.76E-15	0.110547	0.381	0.294	4.26E-11	2.5	SRSF5	1.295918367
MAPK91	1.79E-15	0.1178689	0.1	0.059	4.32E-11	2.5	MAPK9	1.694915254
NMRK12	1.80E-15	0.1094314	0.057	0.028	4.33E-11	2.5	NMRK1	2.035714286
MRPL501	1.84E-15	0.1194239	0.183	0.125	4.44E-11	2.5	MRPL50	1.464
CYB5R41	1.90E-15	0.1061915	0.097	0.057	4.57E-11	2.5	CYB5R4	1.701754386
SLC39A92	1.94E-15	0.1220768	0.143	0.092	4.68E-11	2.5	SLC39A9	1.554347826
MECP2	2.05E-15	0.1023436	0.123	0.076	4.95E-11	2.5	MECP2	1.618421053

THOC22	2.10E-15	0.1446633	0.499	0.403	5.07E-11	2.5	THOC2	1.2382134
RP1-167A14.21	2.15E-15	0.1345225	0.085	0.048	5.19E-11	2.5	RP1-167A1	1.770833333
ATP5A12	2.19E-15	0.1223945	0.25	0.182	5.28E-11	2.5	ATP5A1	1.373626374
ELP4	2.41E-15	0.1078701	0.08	0.044	5.81E-11	2.5	ELP4	1.818181818
TAF81	2.48E-15	0.1254623	0.104	0.062	5.97E-11	2.5	TAF8	1.677419355
SREK11	2.53E-15	0.1144363	0.222	0.158	6.11E-11	2.5	SREK1	1.405063291
MXI12	2.62E-15	0.1476709	0.252	0.184	6.31E-11	2.5	MXI1	1.369565217
CAPNS11	2.66E-15	0.1523797	0.121	0.076	6.41E-11	2.5	CAPNS1	1.592105263
CDK5RAP12	2.78E-15	0.1006982	0.079	0.043	6.69E-11	2.5	CDK5RAP1	1.837209302
ANKRD13C2	2.99E-15	0.13345	0.16	0.107	7.20E-11	2.5	ANKRD13C	1.495327103
SMC62	3.04E-15	0.1143013	0.113	0.069	7.32E-11	2.5	SMC6	1.637681159
NR2C22	3.05E-15	0.1260347	0.24	0.173	7.35E-11	2.5	NR2C2	1.387283237
TSC21	3.16E-15	0.1083454	0.056	0.028	7.62E-11	2.5	TSC2	2
ARID1A1	3.47E-15	0.1369543	0.307	0.232	8.36E-11	2.5	ARID1A	1.323275862
CUL53	3.49E-15	0.1295551	0.167	0.114	8.42E-11	2.5	CUL5	1.464912281
OPA12	3.65E-15	0.1231624	0.151	0.099	8.80E-11	2.5	OPA1	1.525252525
COA12	3.81E-15	0.1583759	0.279	0.209	9.18E-11	2.5	COA1	1.33492823
CASP8AP22	3.84E-15	0.101593	0.081	0.045	9.25E-11	2.5	CASP8AP2	1.8
CENPC1	3.93E-15	0.1030301	0.106	0.064	9.48E-11	2.5	CENPC	1.65625
TAF111	3.95E-15	0.1207184	0.093	0.054	9.53E-11	2.5	TAF11	1.722222222
MIDN1	4.11E-15	0.1074375	0.101	0.06	9.92E-11	2.5	MIDN	1.683333333
EPRS2	4.48E-15	0.1417452	0.192	0.134	1.08E-10	2.5	EPRS	1.432835821
ILKAP1	4.74E-15	0.1367821	0.105	0.063	1.14E-10	2.5	ILKAP	1.666666667
MRFAP13	4.80E-15	0.104664	0.236	0.169	1.16E-10	2.5	MRFAP1	1.396449704
EXOC12	4.90E-15	0.119169	0.157	0.104	1.18E-10	2.5	EXOC1	1.509615385
LSM14A2	5.13E-15	0.1513579	0.428	0.341	1.24E-10	2.5	LSM14A	1.255131965
WIPF23	5.21E-15	0.1143401	0.209	0.146	1.26E-10	2.5	WIPF2	1.431506849
VDR2	5.83E-15	0.1216563	0.122	0.077	1.40E-10	2.5	VDR	1.584415584
MRFAP1L12	6.19E-15	0.1001421	0.082	0.046	1.49E-10	2.5	MRFAP1L1	1.782608696
ASNS3	6.37E-15	0.1082991	0.128	0.081	1.54E-10	2.5	ASNS	1.580246914
SPG201	6.51E-15	0.1112983	0.179	0.123	1.57E-10	2.5	SPG20	1.455284553
TSPAN134	6.63E-15	0.1000924	0.161	0.107	1.60E-10	2.5	TSPAN13	1.504672897
RPRD1A	7.04E-15	0.1303511	0.177	0.122	1.70E-10	2.5	RPRD1A	1.450819672
XRN11	7.11E-15	0.1171833	0.386	0.301	1.71E-10	2.5	XRN1	1.282392027
GAREM11	8.00E-15	0.1342491	0.395	0.311	1.93E-10	2.5	GAREM1	1.270096463
HSPB112	8.23E-15	0.103554	0.147	0.096	1.98E-10	2.5	HSPB11	1.53125
TRAPPC102	8.34E-15	0.1162556	0.163	0.11	2.01E-10	2.5	TRAPPC10	1.481818182
PLAA2	8.43E-15	0.1027073	0.105	0.064	2.03E-10	2.5	PLAA	1.640625
ARHGAP52	8.44E-15	0.1206513	0.486	0.388	2.04E-10	2.5	ARHGAP5	1.25257732
LINC008622	9.91E-15	0.1005511	0.069	0.037	2.39E-10	2.5	LINC00862	1.864864865
ASH2L1	1.02E-14	0.1124013	0.099	0.059	2.46E-10	2.5	ASH2L	1.677966102
INPP4A1	1.04E-14	0.1009446	0.086	0.049	2.51E-10	2.5	INPP4A	1.755102041
FADS32	1.05E-14	0.1081184	0.167	0.111	2.54E-10	2.5	FADS3	1.504504505
DDX582	1.17E-14	0.1062585	0.139	0.09	2.82E-10	2.5	DDX58	1.544444444
C18orf251	1.21E-14	0.1127344	0.103	0.062	2.92E-10	2.5	C18orf25	1.661290323
FAM193A1	1.21E-14	0.1330671	0.236	0.172	2.92E-10	2.5	FAM193A	1.372093023
SYBU1	1.23E-14	0.1203302	0.123	0.078	2.96E-10	2.5	SYBU	1.576923077
ALPK12	1.25E-14	0.1048347	0.282	0.21	3.01E-10	2.5	ALPK1	1.342857143
SYNRG1	1.31E-14	0.1180737	0.101	0.061	3.17E-10	2.5	SYNRG	1.655737705
KLHL282	1.33E-14	0.1137577	0.152	0.101	3.21E-10	2.5	KLHL28	1.504950495
MED271	1.34E-14	0.1150455	0.092	0.054	3.22E-10	2.5	MED27	1.703703704
FAM174B2	1.42E-14	0.1206282	0.111	0.069	3.42E-10	2.5	FAM174B	1.608695652

ATF7IP3	1.43E-14	0.123515	0.232	0.168	3.44E-10	2.5	ATF7IP	1.380952381
TMED31	1.45E-14	0.1136476	0.121	0.076	3.50E-10	2.5	TMED3	1.592105263
SH3BGRL32	1.47E-14	0.1367042	0.09	0.052	3.54E-10	2.5	SH3BGRL3	1.730769231
TCAIM2	1.48E-14	0.108358	0.096	0.057	3.57E-10	2.5	TCAIM	1.684210526
KIF26B2	1.49E-14	0.1185929	0.055	0.027	3.58E-10	2.5	KIF26B	2.037037037
CUL4A2	1.61E-14	0.1206098	0.239	0.173	3.89E-10	2.5	CUL4A	1.38150289
LUC7L5	1.67E-14	0.1284282	0.137	0.09	4.03E-10	2.5	LUC7L	1.522222222
SMARCA23	1.70E-14	0.1086256	0.388	0.301	4.11E-10	2.5	SMARCA2	1.289036545
ATXN22	1.73E-14	0.1122856	0.415	0.325	4.18E-10	2.5	ATXN2	1.276923077
KAT6A1	1.77E-14	0.1052662	0.32	0.244	4.26E-10	2.5	KAT6A	1.31147541
C2orf683	1.78E-14	0.1030477	0.097	0.058	4.30E-10	2.5	C2orf68	1.672413793
IMMT1	1.89E-14	0.1153608	0.119	0.075	4.55E-10	2.5	IMMT	1.586666667
KIAA04303	1.89E-14	0.1055418	0.181	0.124	4.56E-10	2.5	KIAA0430	1.459677419
KMT5B3	1.99E-14	0.1257242	0.216	0.155	4.79E-10	2.5	KMT5B	1.393548387
CBWD52	2.03E-14	0.1173077	0.235	0.17	4.89E-10	2.5	CBWD5	1.382352941
MED42	2.09E-14	0.1143663	0.21	0.149	5.05E-10	2.5	MED4	1.409395973
R3HDM22	2.26E-14	0.1323659	0.164	0.112	5.46E-10	2.5	R3HDM2	1.464285714
USP3-AS11	2.35E-14	0.1166566	0.112	0.069	5.67E-10	2.5	USP3-AS1	1.623188406
C4orf47	2.35E-14	0.1013672	0.084	0.049	5.67E-10	2.5	C4orf47	1.714285714
PDK44	2.44E-14	0.1040118	0.12	0.075	5.89E-10	2.5	PDK4	1.6
SUGP21	2.46E-14	0.1036995	0.091	0.053	5.92E-10	2.5	SUGP2	1.716981132
VPRBP1	2.52E-14	0.1053427	0.086	0.05	6.07E-10	2.5	VPRBP	1.72
ERP293	2.60E-14	0.1183054	0.172	0.117	6.28E-10	2.5	ERP29	1.47008547
FAF22	2.74E-14	0.1531107	0.213	0.154	6.60E-10	2.5	FAF2	1.383116883
WARS21	2.83E-14	0.1054001	0.103	0.062	6.84E-10	2.5	WARS2	1.661290323
SS182	3.26E-14	0.1335	0.187	0.131	7.86E-10	2.5	SS18	1.427480916
ZNF447	3.36E-14	0.1056354	0.1	0.061	8.11E-10	2.5	ZNF44	1.639344262
TET31	3.38E-14	0.1175877	0.178	0.123	8.16E-10	2.5	TET3	1.447154472
EAPP2	3.42E-14	0.1382764	0.345	0.27	8.25E-10	2.5	EAPP	1.277777778
SMARCA5	3.95E-14	0.1229549	0.166	0.114	9.52E-10	2.5	SMARCA5	1.456140351
NEK102	4.05E-14	0.1271804	0.205	0.145	9.76E-10	2.5	NEK10	1.413793103
RAD502	4.13E-14	0.1225867	0.125	0.081	9.97E-10	2.5	RAD50	1.543209877
SIAH11	4.21E-14	0.103754	0.162	0.11	1.01E-09	2.5	SIAH1	1.472727273
GTF2I1	4.83E-14	0.1009243	0.534	0.433	1.16E-09	2.5	GTF2I	1.233256351
CDC42SE12	5.71E-14	0.1284627	0.201	0.143	1.38E-09	2.5	CDC42SE1	1.405594406
CCDC1251	6.29E-14	0.1194838	0.1	0.061	1.52E-09	2.5	CCDC125	1.639344262
UBE2E12	6.37E-14	0.1077291	0.424	0.335	1.54E-09	2.5	UBE2E1	1.265671642
SQLE3	6.46E-14	0.1273319	0.129	0.084	1.56E-09	2.5	SQLE	1.535714286
PRPF81	6.72E-14	0.1130677	0.124	0.079	1.62E-09	2.5	PRPF8	1.569620253
CHURC1-FNTB1	6.73E-14	0.1027141	0.071	0.039	1.62E-09	2.5	CHURC1-FN	1.820512821
TNKS3	6.89E-14	0.1205662	0.356	0.277	1.66E-09	2.5	TNKS	1.285198556
KIF3B1	8.08E-14	0.1076124	0.124	0.079	1.95E-09	2.5	KIF3B	1.569620253
C9orf32	8.18E-14	0.1045552	0.187	0.132	1.97E-09	2.5	C9orf3	1.416666667
ZNF7201	8.52E-14	0.1098601	0.125	0.081	2.05E-09	2.5	ZNF720	1.543209877
SPOP4	8.75E-14	0.1211874	0.124	0.08	2.11E-09	2.5	SPOP	1.55
MSRA2	8.85E-14	0.1043644	0.123	0.079	2.13E-09	2.5	MSRA	1.556962025
PLP23	9.55E-14	0.1078841	0.108	0.067	2.30E-09	2.5	PLP2	1.611940299
ZNF7362	1.05E-13	0.1133293	0.122	0.079	2.53E-09	2.5	ZNF736	1.544303797
ABI22	1.05E-13	0.1116363	0.246	0.181	2.54E-09	2.5	ABI2	1.359116022
POGZ2	1.10E-13	0.1402973	0.328	0.257	2.66E-09	2.5	POGZ	1.276264591
FAM219A2	1.15E-13	0.1087071	0.152	0.102	2.78E-09	2.5	FAM219A	1.490196078
KCTD73	1.16E-13	0.1211913	0.155	0.105	2.79E-09	2.5	KCTD7	1.476190476

WHSC12	1.24E-13	0.1016784	0.236	0.172	2.98E-09	2.5	WHSC1	1.372093023
TRAPPC132	1.25E-13	0.105031	0.095	0.057	3.00E-09	2.5	TRAPPC13	1.666666667
UQCC12	1.53E-13	0.1137195	0.107	0.067	3.70E-09	2.5	UQCC1	1.597014925
CSNK1D1	1.58E-13	0.1077992	0.11	0.069	3.81E-09	2.5	CSNK1D	1.594202899
SHROOM21	1.58E-13	0.1050193	0.084	0.049	3.82E-09	2.5	SHROOM2	1.714285714
PSMB52	1.99E-13	0.133302	0.304	0.233	4.79E-09	2.5	PSMB5	1.30472103
EID12	2.01E-13	0.1011457	0.119	0.076	4.85E-09	2.5	EID1	1.565789474
SLC35F52	2.09E-13	0.1047629	0.164	0.113	5.03E-09	2.5	SLC35F5	1.451327434
VLDLR1	2.33E-13	0.108443	0.087	0.052	5.62E-09	2.5	VLDLR	1.673076923
ANXA73	2.37E-13	0.1419446	0.355	0.282	5.72E-09	2.5	ANXA7	1.258865248
CLK13	2.39E-13	0.1151248	0.493	0.407	5.77E-09	2.5	CLK1	1.211302211
EPC22	2.85E-13	0.1001325	0.107	0.067	6.88E-09	2.5	EPC2	1.597014925
AC058791.12	2.99E-13	0.1397852	0.311	0.241	7.21E-09	2.5	AC058791.	1.290456432
XRCC51	2.99E-13	0.134893	0.425	0.346	7.21E-09	2.5	XRCC5	1.228323699
ARGLU12	3.00E-13	0.1187047	0.241	0.179	7.24E-09	2.5	ARGLU1	1.346368715
GRIP12	3.07E-13	0.1108658	0.246	0.182	7.41E-09	2.5	GRIP1	1.351648352
PAXBP11	3.10E-13	0.1104477	0.134	0.089	7.47E-09	2.5	PAXBP1	1.505617978
MROH11	3.17E-13	0.1037448	0.101	0.063	7.65E-09	2.5	MROH1	1.603174603
URI1	3.22E-13	0.1138626	0.203	0.145	7.75E-09	2.5	URI1	1.4
FBXO71	3.52E-13	0.1048187	0.195	0.139	8.49E-09	2.5	FBXO7	1.402877698
TBC1D142	4.28E-13	0.1032222	0.106	0.067	1.03E-08	2.5	TBC1D14	1.582089552
HMBOX12	4.91E-13	0.1115559	0.295	0.225	1.18E-08	2.5	HMBOX1	1.311111111
BLVRB2	5.30E-13	0.1193583	0.103	0.064	1.28E-08	2.5	BLVRB	1.609375
MITD12	5.62E-13	0.1191138	0.143	0.097	1.35E-08	2.5	MITD1	1.474226804
DECR13	5.67E-13	0.1221469	0.21	0.153	1.37E-08	2.5	DECR1	1.37254902
BAZ1B2	6.75E-13	0.1191149	0.225	0.166	1.63E-08	2.5	BAZ1B	1.355421687
TUBB3	6.85E-13	0.1697637	0.4	0.329	1.65E-08	2.5	TUBB	1.215805471
IST12	7.06E-13	0.1192953	0.4	0.321	1.70E-08	2.5	IST1	1.246105919
FBXO331	7.24E-13	0.1063884	0.141	0.094	1.75E-08	2.5	FBXO33	1.5
SOS13	7.51E-13	0.1059606	0.445	0.36	1.81E-08	2.5	SOS1	1.236111111
VPS363	7.63E-13	0.110292	0.145	0.098	1.84E-08	2.5	VPS36	1.479591837
DEAF11	7.68E-13	0.1075245	0.113	0.072	1.85E-08	2.5	DEAF1	1.569444444
TNPO33	9.23E-13	0.1111414	0.198	0.142	2.22E-08	2.5	TNPO3	1.394366197
IL4R2	9.25E-13	0.1024176	0.19	0.136	2.23E-08	2.5	IL4R	1.397058824
FIGN2	9.96E-13	0.1425999	0.143	0.098	2.40E-08	2.5	FIGN	1.459183673
DLG31	1.11E-12	0.1021561	0.114	0.073	2.67E-08	2.5	DLG3	1.561643836
PSMD52	1.11E-12	0.1315447	0.162	0.113	2.68E-08	2.5	PSMD5	1.433628319
GNPAT2	1.13E-12	0.1038455	0.094	0.058	2.73E-08	2.5	GNPAT	1.620689655
TMF1	1.27E-12	0.1030114	0.238	0.177	3.06E-08	2.5	TMF1	1.344632768
RPA12	1.33E-12	0.1015649	0.101	0.063	3.22E-08	2.5	RPA1	1.603174603
PARG2	1.38E-12	0.105386	0.123	0.081	3.34E-08	2.5	PARG	1.518518519
MRPL333	1.79E-12	0.1159271	0.498	0.412	4.30E-08	2.5	MRPL33	1.208737864
USP141	1.82E-12	0.110922	0.137	0.093	4.40E-08	2.5	USP14	1.47311828
NAPG3	1.87E-12	0.123734	0.232	0.174	4.51E-08	2.5	NAPG	1.333333333
PLEKHA53	1.89E-12	0.1169842	0.467	0.383	4.55E-08	2.5	PLEKHA5	1.219321149
C1D3	1.90E-12	0.1094128	0.196	0.142	4.59E-08	2.5	C1D	1.38028169
AAMDC2	2.18E-12	0.1027076	0.218	0.16	5.26E-08	2.5	AAMDC	1.3625
DCAF102	2.26E-12	0.1297365	0.24	0.18	5.44E-08	2.5	DCAF10	1.333333333
HSPA92	2.26E-12	0.1120297	0.275	0.209	5.44E-08	2.5	HSPA9	1.315789474
UFD1L1	2.29E-12	0.1107877	0.125	0.083	5.52E-08	2.5	UFD1L	1.506024096
SQRDL2	2.55E-12	0.1264426	0.24	0.181	6.14E-08	2.5	SQRDL	1.325966851
NCOA62	2.56E-12	0.1239347	0.222	0.164	6.17E-08	2.5	NCOA6	1.353658537

HEATR5B1	3.03E-12	0.1130451	0.169	0.119	7.31E-08	2.5	HEATR5B	1.420168067
RBM262	3.06E-12	0.1127291	0.247	0.187	7.39E-08	2.5	RBM26	1.320855615
NUDCD33	3.28E-12	0.1041075	0.148	0.102	7.91E-08	2.5	NUDCD3	1.450980392
PDS5A2	3.30E-12	0.1171134	0.434	0.353	7.97E-08	2.5	PDS5A	1.229461756
PIBF12	3.64E-12	0.111735	0.228	0.169	8.77E-08	2.5	PIBF1	1.349112426
SORL13	3.79E-12	0.1019046	0.1	0.063	9.13E-08	2.5	SORL1	1.587301587
PPP4R3A1	3.83E-12	0.1282273	0.236	0.178	9.23E-08	2.5	PPP4R3A	1.325842697
IQCJ-SCHIP12	4.15E-12	0.2639326	0.137	0.094	1.00E-07	2.5	IQCJ-SCHIP	1.457446809
RPL7L13	4.21E-12	0.1089047	0.166	0.117	1.02E-07	2.5	RPL7L1	1.418803419
IFITM32	5.18E-12	0.1610704	0.232	0.178	1.25E-07	2.5	IFITM3	1.303370787
STAT21	5.41E-12	0.1091762	0.122	0.082	1.30E-07	2.5	STAT2	1.487804878
LARP72	5.58E-12	0.1107333	0.202	0.148	1.35E-07	2.5	LARP7	1.364864865
XPO42	5.98E-12	0.1208543	0.154	0.108	1.44E-07	2.5	XPO4	1.425925926
FAM168B1	6.12E-12	0.1131248	0.128	0.087	1.48E-07	2.5	FAM168B	1.471264368
BSDC11	6.22E-12	0.1196902	0.24	0.182	1.50E-07	2.5	BSDC1	1.318681319
RBM14-RBM41	6.35E-12	0.1050992	0.084	0.051	1.53E-07	2.5	RBM14-RBI	1.647058824
OXR12	6.85E-12	0.1359305	0.243	0.185	1.65E-07	2.5	OXR1	1.313513514
TTF13	7.47E-12	0.103867	0.158	0.111	1.80E-07	2.5	TTF1	1.423423423
PPP4R3B1	7.70E-12	0.1094058	0.224	0.167	1.86E-07	2.5	PPP4R3B	1.341317365
CCDC533	9.91E-12	0.1017878	0.128	0.086	2.39E-07	2.5	CCDC53	1.488372093
FBXL53	1.19E-11	0.119619	0.175	0.126	2.88E-07	2.5	FBXL5	1.388888889
PSMA43	1.28E-11	0.1390656	0.509	0.43	3.08E-07	2.5	PSMA4	1.18372093
UBE2D33	1.38E-11	0.1046557	0.672	0.579	3.33E-07	2.5	UBE2D3	1.160621762
GCH11	1.53E-11	0.1225568	0.145	0.1	3.68E-07	2.5	GCH1	1.45
ATXN102	1.53E-11	0.1241594	0.192	0.141	3.70E-07	2.5	ATXN10	1.361702128
ZFP142	1.63E-11	0.1069101	0.095	0.061	3.93E-07	2.5	ZFP14	1.557377049
RSF12	1.67E-11	0.100136	0.448	0.365	4.04E-07	2.5	RSF1	1.22739726
CEP571	1.78E-11	0.1126992	0.135	0.093	4.29E-07	2.5	CEP57	1.451612903
MPZL32	1.86E-11	0.133944	0.378	0.307	4.48E-07	2.5	MPZL3	1.231270358
S100PBP2	1.98E-11	0.1022201	0.148	0.104	4.77E-07	2.5	S100PBP	1.423076923
C16orf621	2.04E-11	0.1028235	0.101	0.065	4.91E-07	2.5	C16orf62	1.553846154
MON21	2.17E-11	0.1153634	0.289	0.227	5.23E-07	2.5	MON2	1.273127753
ARHGAP442	2.36E-11	0.1259155	0.227	0.172	5.68E-07	2.5	ARHGAP44	1.319767442
MCC2	2.51E-11	0.1322588	0.143	0.1	6.05E-07	2.5	MCC	1.43
BCAP312	2.84E-11	0.1030307	0.133	0.091	6.85E-07	2.5	BCAP31	1.461538462
ENSA2	2.97E-11	0.1089964	0.439	0.359	7.17E-07	2.5	ENSA	1.222841226
DYNC1H12	3.12E-11	0.1031633	0.235	0.178	7.52E-07	2.5	DYNC1H1	1.320224719
STRAP1	3.13E-11	0.1027903	0.19	0.138	7.56E-07	2.5	STRAP	1.376811594
RAF11	3.79E-11	0.1143369	0.252	0.194	9.13E-07	2.5	RAF1	1.298969072
NDFIP11	4.05E-11	0.1181128	0.247	0.189	9.75E-07	2.5	NDFIP1	1.306878307
ORMDL23	4.08E-11	0.137618	0.207	0.155	9.84E-07	2.5	ORMDL2	1.335483871
CIR12	4.17E-11	0.1104579	0.315	0.249	1.01E-06	2.5	CIR1	1.265060241
ZNF7102	4.58E-11	0.1018226	0.127	0.086	1.10E-06	2.5	ZNF710	1.476744186
DCTN22	4.69E-11	0.1030409	0.173	0.125	1.13E-06	2.5	DCTN2	1.384
DPP82	5.03E-11	0.1149815	0.127	0.087	1.21E-06	2.5	DPP8	1.459770115
ATXN32	5.35E-11	0.1069048	0.187	0.138	1.29E-06	2.5	ATXN3	1.355072464
TASP12	5.71E-11	0.1031062	0.172	0.125	1.38E-06	2.5	TASP1	1.376
ABCA51	6.54E-11	0.120757	0.164	0.118	1.58E-06	2.5	ABCA5	1.389830508
CNTN42	6.64E-11	0.1863616	0.178	0.131	1.60E-06	2.5	CNTN4	1.358778626
CHKA2	6.95E-11	0.1048702	0.145	0.102	1.67E-06	2.5	CHKA	1.421568627
SESN22	7.80E-11	0.1004344	0.152	0.108	1.88E-06	2.5	SESN2	1.407407407
CCDC90B2	9.50E-11	0.1034123	0.121	0.083	2.29E-06	2.5	CCDC90B	1.457831325

PDE4D1	1.00E-10	0.1228906	0.14	0.099	2.42E-06	2.5	PDE4D	1.414141414
STK17B3	1.14E-10	0.1208352	0.206	0.154	2.74E-06	2.5	STK17B	1.337662338
RFX3-AS12	1.14E-10	0.1073249	0.09	0.058	2.76E-06	2.5	RFX3-AS1	1.551724138
METTL92	1.32E-10	0.1049167	0.142	0.1	3.18E-06	2.5	METTL9	1.42
SH3GLB1	1.58E-10	0.1080718	0.196	0.147	3.80E-06	2.5	SH3GLB1	1.333333333
SYNGR21	1.60E-10	0.100887	0.061	0.035	3.86E-06	2.5	SYNGR2	1.742857143
TRPM72	1.77E-10	0.1066257	0.273	0.212	4.26E-06	2.5	TRPM7	1.287735849
FBXO212	1.94E-10	0.1104919	0.144	0.103	4.67E-06	2.5	FBXO21	1.398058252
PIN44	2.18E-10	0.1008091	0.324	0.26	5.26E-06	2.5	PIN4	1.246153846
TP532	2.68E-10	0.114355	0.09	0.059	6.47E-06	2.5	TP53	1.525423729
GRAMD31	3.43E-10	0.1493714	0.406	0.34	8.28E-06	2.5	GRAMD3	1.194117647
IDH14	4.49E-10	0.1030537	0.113	0.078	1.08E-05	2.5	IDH1	1.448717949
VPS452	4.57E-10	0.1011767	0.109	0.074	1.10E-05	2.5	VPS45	1.472972973
YES1	4.61E-10	0.1077603	0.273	0.214	1.11E-05	2.5	YES1	1.275700935
RAB5B	5.05E-10	0.1080307	0.24	0.187	1.22E-05	2.5	RAB5B	1.28342246
ANXA42	5.16E-10	0.1012228	0.145	0.104	1.24E-05	2.5	ANXA4	1.394230769
PRKAG12	5.50E-10	0.1041767	0.181	0.135	1.33E-05	2.5	PRKAG1	1.340740741
CBR43	7.22E-10	0.1160218	0.285	0.228	1.74E-05	2.5	CBR4	1.25
WDFY11	9.38E-10	0.1032384	0.118	0.081	2.26E-05	2.5	WDFY1	1.456790123
ACLY2	1.04E-09	0.1089952	0.15	0.109	2.50E-05	2.5	ACLY	1.376146789
USP82	1.08E-09	0.1057114	0.22	0.169	2.61E-05	2.5	USP8	1.301775148
BCL31	1.17E-09	0.1094764	0.138	0.099	2.82E-05	2.5	BCL3	1.393939394
PRDX53	1.18E-09	0.1078084	0.092	0.061	2.84E-05	2.5	PRDX5	1.508196721
RBBP82	1.58E-09	0.114351	0.136	0.097	3.81E-05	2.5	RBBP8	1.402061856
MARK12	1.60E-09	0.1080087	0.092	0.061	3.87E-05	2.5	MARK1	1.508196721
MDM22	2.51E-09	0.106928	0.178	0.135	6.06E-05	2.5	MDM2	1.318518519
DTWD12	2.76E-09	0.1023547	0.09	0.06	6.67E-05	2.5	DTWD1	1.5
MT-ND12	2.95E-09	0.1254759	0.995	0.977	7.11E-05	2.5	MT-ND1	1.018423746
ARNTL22	3.16E-09	0.1005649	0.185	0.14	7.63E-05	2.5	ARNTL2	1.321428571
ICA12	3.96E-09	0.108219	0.321	0.263	9.54E-05	2.5	ICA1	1.220532319
EIF2S23	4.39E-09	0.1013249	0.309	0.254	0.000105754	2.5	EIF2S2	1.216535433
NUDC2	4.67E-09	0.1077947	0.118	0.083	0.000112541	2.5	NUDC	1.421686747
ARPC33	7.51E-09	0.1508435	0.713	0.655	0.000181096	2.5	ARPC3	1.088549618
PIGL3	8.24E-09	0.1029139	0.1	0.069	0.000198591	2.5	PIGL	1.449275362
ST6GAL13	1.74E-08	0.1176285	0.3	0.245	0.000420249	2.5	ST6GAL1	1.224489796
DNAJB111	1.83E-08	0.127238	0.224	0.18	0.000441154	2.5	DNAJB11	1.244444444
PPP4R11	1.95E-08	0.1093788	0.28	0.228	0.000471334	2.5	PPP4R1	1.228070175
ANAPC112	2.30E-08	0.1063309	0.056	0.034	0.000553741	2.5	ANAPC11	1.647058824
EBP3	2.50E-08	0.105939	0.15	0.112	0.000602861	2.5	EBP	1.339285714
POLR2L1	1.03E-07	0.109483	0.056	0.035	0.002478322	2.5	POLR2L	1.6
RFX72	3.39E-07	0.1026112	0.15	0.115	0.008179052	2.5	RFX7	1.304347826
IDI12	3.49E-07	0.1012792	0.264	0.216	0.008422281	2.5	IDI1	1.222222222
SH3PXD2B4	4.38E-07	0.1081003	0.154	0.119	0.01056008	2.5	SH3PXD2B	1.294117647
PLAU2	3.22E-261	1.308846	0.396	0.076	7.77E-257	2.6	PLAU	5.210526316
CXCL15	2.47E-236	2.298766	0.669	0.252	5.96E-232	2.6	CXCL1	2.654761905
ST53	5.15E-185	1.399599	0.723	0.323	1.24E-180	2.6	ST5	2.238390093
TNFAIP22	2.54E-173	1.140457	0.63	0.252	6.12E-169	2.6	TNFAIP2	2.5
CXCL83	6.78E-165	1.795199	0.764	0.431	1.64E-160	2.6	CXCL8	1.77262181
ST3GAL42	1.43E-163	0.8069561	0.284	0.058	3.44E-159	2.6	ST3GAL4	4.896551724
PLPP32	2.51E-160	1.305688	0.592	0.236	6.04E-156	2.6	PLPP3	2.508474576
EFNA14	3.10E-160	1.312399	0.572	0.224	7.48E-156	2.6	EFNA1	2.553571429
NCEH14	3.07E-151	1.104449	0.778	0.397	7.40E-147	2.6	NCEH1	1.959697733

PPP4R42	1.33E-150	1.050186	0.325	0.081	3.21E-146	2.6	PPP4R4	4.012345679
RAB11FIP14	4.77E-150	1.072728	0.982	0.723	1.15E-145	2.6	RAB11FIP1	1.358229599
ABTB23	1.55E-149	1.330014	0.568	0.232	3.74E-145	2.6	ABTB2	2.448275862
TRERF12	1.33E-142	0.9995201	0.451	0.148	3.20E-138	2.6	TRERF1	3.047297297
EREG5	5.21E-141	1.288767	0.594	0.246	1.26E-136	2.6	EREG	2.414634146
ATP1B12	3.15E-137	1.105463	0.963	0.753	7.58E-133	2.6	ATP1B1	1.278884462
ELOVL72	4.26E-137	1.172385	0.339	0.095	1.03E-132	2.6	ELOVL7	3.568421053
SAT12	2.30E-133	0.9544101	1	0.979	5.55E-129	2.6	SAT1	1.02145046
CXCL22	2.52E-131	1.413183	0.513	0.207	6.08E-127	2.6	CXCL2	2.47826087
KLHL133	3.76E-129	0.9273221	0.346	0.099	9.07E-125	2.6	KLHL13	3.494949495
IRAK23	2.50E-124	0.9126978	0.372	0.117	6.03E-120	2.6	IRAK2	3.179487179
ALCAM5	2.93E-122	0.8311393	0.876	0.496	7.08E-118	2.6	ALCAM	1.766129032
TNC5	4.55E-122	1.090961	0.655	0.318	1.10E-117	2.6	TNC	2.059748428
RP11-519G16.32	1.42E-119	1.054704	0.821	0.494	3.42E-115	2.6	RP11-519G	1.66194332
LIMCH13	8.09E-116	0.9399839	0.805	0.473	1.95E-111	2.6	LIMCH1	1.701902748
CSGALNACT12	9.07E-116	0.9646894	0.46	0.173	2.19E-111	2.6	CSGALNAC	2.658959538
CXCL62	6.20E-114	1.481212	0.246	0.06	1.49E-109	2.6	CXCL6	4.1
PTPRE2	8.58E-114	0.8880764	0.572	0.251	2.07E-109	2.6	PTPRE	2.278884462
USP543	4.74E-109	1.204501	0.788	0.483	1.14E-104	2.6	USP54	1.631469979
TBL1XR13	1.34E-108	0.9077485	0.865	0.569	3.22E-104	2.6	TBL1XR1	1.520210896
CDK133	1.68E-107	0.9134213	0.732	0.41	4.04E-103	2.6	CDK13	1.785365854
DENND4A2	3.77E-105	0.9644898	0.854	0.583	9.10E-101	2.6	DENND4A	1.46483705
ENOX14	5.49E-104	0.775109	0.378	0.129	1.32E-99	2.6	ENOX1	2.930232558
DIO25	6.43E-102	0.6987131	0.52	0.211	1.55E-97	2.6	DIO2	2.464454976
TNFAIP33	1.91E-101	0.9972699	0.557	0.267	4.60E-97	2.6	TNFAIP3	2.086142322
BBC31	8.81E-100	0.6444015	0.265	0.074	2.12E-95	2.6	BBC3	3.581081081
ACADSB5	1.67E-99	0.7957388	0.475	0.191	4.03E-95	2.6	ACADSB	2.486910995
HIVEP23	1.77E-98	1.164538	0.713	0.438	4.26E-94	2.6	HIVEP2	1.627853881
PIP5K1B2	1.44E-97	0.8099777	0.34	0.116	3.46E-93	2.6	PIP5K1B	2.931034483
STAT5A5	5.96E-97	0.8347606	0.431	0.17	1.44E-92	2.6	STAT5A	2.535294118
SCGB2A23	6.58E-96	1.424819	0.54	0.262	1.59E-91	2.6	SCGB2A2	2.061068702
KLHL52	1.47E-95	0.8467055	0.566	0.273	3.54E-91	2.6	KLHL5	2.073260073
GCNT24	1.08E-94	0.7885927	0.753	0.431	2.60E-90	2.6	GCNT2	1.747099768
MYO1B3	1.52E-92	0.9052258	0.752	0.434	3.66E-88	2.6	MYO1B	1.732718894
CX3CL13	1.93E-89	0.7076136	0.495	0.219	4.66E-85	2.6	CX3CL1	2.260273973
FGF144	1.46E-88	1.003888	0.263	0.079	3.51E-84	2.6	FGF14	3.329113924
PLEKHF23	2.78E-88	0.6203097	0.292	0.093	6.71E-84	2.6	PLEKHF2	3.139784946
MGLL3	6.73E-88	0.6682217	0.427	0.173	1.62E-83	2.6	MGLL	2.468208092
SLC22A233	2.61E-87	0.7359518	0.519	0.236	6.29E-83	2.6	SLC22A23	2.199152542
CASC154	3.47E-86	0.7642741	0.828	0.509	8.36E-82	2.6	CASC15	1.626719057
RND13	5.36E-86	0.6890149	0.254	0.076	1.29E-81	2.6	RND1	3.342105263
SEMA3C4	7.55E-86	0.8095027	0.562	0.268	1.82E-81	2.6	SEMA3C	2.097014925
MTSS13	3.77E-85	0.7840587	0.379	0.145	9.10E-81	2.6	MTSS1	2.613793103
FOXP13	9.33E-82	0.7677145	0.887	0.623	2.25E-77	2.6	FOXP1	1.423756019
PIK3C2B2	2.01E-81	0.7086638	0.254	0.079	4.84E-77	2.6	PIK3C2B	3.215189873
PHLDB24	5.29E-81	0.7232725	0.703	0.407	1.28E-76	2.6	PHLDB2	1.727272727
TFPI5	1.68E-80	0.5664111	0.638	0.312	4.05E-76	2.6	TFPI	2.044871795
DRAM13	1.84E-80	0.7228338	0.569	0.284	4.43E-76	2.6	DRAM1	2.003521127
HPX5	4.27E-78	0.7056576	0.465	0.204	1.03E-73	2.6	HPX	2.279411765
CPE5	7.45E-78	0.6802779	0.449	0.194	1.80E-73	2.6	CPE	2.31443299
GADD45A4	1.85E-77	0.9318567	0.515	0.256	4.47E-73	2.6	GADD45A	2.01171875
BCAR33	3.24E-77	0.807451	0.538	0.268	7.81E-73	2.6	BCAR3	2.007462687

ECE13	2.68E-76	0.6721384	0.415	0.178	6.46E-72	2.6	ECE1	2.331460674
BCL2L112	5.91E-74	0.7148119	0.316	0.119	1.42E-69	2.6	BCL2L11	2.655462185
IFNGR22	9.57E-74	0.6519862	0.562	0.291	2.31E-69	2.6	IFNGR2	1.931271478
RAB122	7.04E-72	0.7151163	0.506	0.251	1.70E-67	2.6	RAB12	2.015936255
ANKS1B3	2.29E-71	0.678558	0.735	0.449	5.53E-67	2.6	ANKS1B	1.636971047
TNIP13	5.83E-71	0.7016808	0.437	0.202	1.41E-66	2.6	TNIP1	2.163366337
TBX35	8.21E-71	0.6148347	0.533	0.259	1.98E-66	2.6	TBX3	2.057915058
ITGB63	2.30E-69	0.753133	0.635	0.373	5.56E-65	2.6	ITGB6	1.702412869
NFKBIA4	5.05E-69	0.6189083	0.85	0.618	1.22E-64	2.6	NFKBIA	1.375404531
PRSS233	2.91E-68	0.5346873	0.47	0.211	7.02E-64	2.6	PRSS23	2.227488152
C16orf453	5.44E-68	0.6436396	0.462	0.219	1.31E-63	2.6	C16orf45	2.109589041
BTG14	8.76E-68	0.6443365	0.826	0.57	2.11E-63	2.6	BTG1	1.449122807
GRAMD32	4.43E-67	0.7732486	0.591	0.337	1.07E-62	2.6	GRAMD3	1.753709199
RAB313	6.21E-67	0.7505669	0.398	0.182	1.50E-62	2.6	RAB31	2.186813187
ITGAV5	2.82E-66	0.5994121	0.672	0.388	6.80E-62	2.6	ITGAV	1.731958763
TSPAN54	3.70E-66	0.684291	0.569	0.298	8.93E-62	2.6	TSPAN5	1.909395973
ICAM13	3.71E-65	0.6159699	0.392	0.175	8.96E-61	2.6	ICAM1	2.24
AMOT2	5.85E-65	0.589768	0.23	0.077	1.41E-60	2.6	AMOT	2.987012987
HIVEP34	3.88E-64	0.7123744	0.563	0.306	9.36E-60	2.6	HIVEP3	1.839869281
FHDC12	2.00E-63	0.5500417	0.296	0.114	4.83E-59	2.6	FHDC1	2.596491228
PKP44	9.93E-63	0.6387482	0.749	0.492	2.40E-58	2.6	PKP4	1.522357724
FGF134	1.94E-61	0.4789186	0.744	0.44	4.69E-57	2.6	FGF13	1.690909091
MACC13	2.04E-61	0.7180081	0.665	0.402	4.93E-57	2.6	MACC1	1.654228856
CXCL135	5.15E-61	0.3167545	0.687	0.4	1.24E-56	2.6	CXCL13	1.7175
RAP1B2	6.09E-61	0.6951002	0.714	0.464	1.47E-56	2.6	RAP1B	1.538793103
ZFAND34	8.97E-61	0.5422943	0.937	0.753	2.16E-56	2.6	ZFAND3	1.24435591
ANKRD33B2	9.09E-61	0.4978335	0.194	0.06	2.19E-56	2.6	ANKRD33B	3.233333333
SMAD34	5.76E-60	0.6367938	0.502	0.261	1.39E-55	2.6	SMAD3	1.923371648
IRF14	7.36E-60	0.6195529	0.385	0.177	1.77E-55	2.6	IRF1	2.175141243
ANO63	8.19E-60	0.5511497	0.772	0.508	1.97E-55	2.6	ANO6	1.519685039
ZBTB104	1.23E-59	0.6714481	0.482	0.247	2.96E-55	2.6	ZBTB10	1.951417004
LRRC13	5.39E-59	0.4984055	0.33	0.137	1.30E-54	2.6	LRRC1	2.408759124
LSAMP5	6.18E-59	0.7732892	0.699	0.461	1.49E-54	2.6	LSAMP	1.51626898
ABLIM14	7.34E-59	0.6572499	0.491	0.26	1.77E-54	2.6	ABLIM1	1.888461538
CD2742	9.63E-58	0.3539822	0.128	0.031	2.32E-53	2.6	CD274	4.129032258
CDYL4	3.64E-57	0.6215097	0.545	0.302	8.77E-53	2.6	CDYL	1.804635762
ASAP13	5.61E-57	0.7223046	0.51	0.284	1.35E-52	2.6	ASAP1	1.795774648
NFKB14	9.82E-57	0.6007917	0.695	0.44	2.37E-52	2.6	NFKB1	1.579545455
ADAMTS42	1.15E-56	0.4079768	0.162	0.046	2.76E-52	2.6	ADAMTS4	3.52173913
GPD23	1.72E-56	0.7022596	0.558	0.321	4.15E-52	2.6	GPD2	1.738317757
CUX12	1.75E-56	0.517204	0.596	0.339	4.21E-52	2.6	CUX1	1.758112094
UGCG3	1.95E-56	0.5405294	0.745	0.479	4.70E-52	2.6	UGCG	1.555323591
PRRG42	3.09E-56	0.5868824	0.484	0.255	7.45E-52	2.6	PRRG4	1.898039216
MALAT15	4.39E-56	0.3017946	1	1	1.06E-51	2.6	MALAT1	1
BAZ1A4	2.03E-55	0.6061866	0.62	0.374	4.90E-51	2.6	BAZ1A	1.657754011
DUSP165	5.68E-55	0.5494287	0.672	0.41	1.37E-50	2.6	DUSP16	1.63902439
CPEB25	2.54E-54	0.4597798	0.564	0.299	6.11E-50	2.6	CPEB2	1.886287625
TMSB105	2.74E-54	0.3317931	0.928	0.724	6.61E-50	2.6	TMSB10	1.281767956
C1QTNF3-AMACR2	4.39E-54	0.5738996	0.556	0.308	1.06E-49	2.6	C1QTNF3-A	1.805194805
TNFAIP84	4.87E-54	0.5568468	0.623	0.375	1.18E-49	2.6	TNFAIP8	1.661333333
B4GALT12	1.17E-53	0.6036236	0.672	0.43	2.82E-49	2.6	B4GALT1	1.562790698
TTTY142	1.01E-52	0.7156291	0.636	0.413	2.43E-48	2.6	TTTY14	1.539951574

GNAS5	1.05E-52	0.4282965	0.929	0.745	2.54E-48	2.6	GNAS	1.246979866
NBEA2	1.80E-52	0.5335963	0.472	0.242	4.34E-48	2.6	NBEA	1.950413223
RAB214	1.88E-52	0.5773197	0.473	0.254	4.54E-48	2.6	RAB21	1.862204724
PTHLH5	2.41E-52	0.4380404	0.457	0.227	5.81E-48	2.6	PTHLH	2.013215859
BCL25	4.70E-52	0.565335	0.497	0.264	1.13E-47	2.6	BCL2	1.882575758
APBB23	4.91E-52	0.6479143	0.474	0.248	1.18E-47	2.6	APBB2	1.911290323
FNDC3B3	1.53E-51	0.5051216	0.934	0.765	3.69E-47	2.6	FNDC3B	1.220915033
SPRED22	1.98E-51	0.5457966	0.41	0.204	4.78E-47	2.6	SPRED2	2.009803922
TTC39C2	1.45E-50	0.5151419	0.406	0.201	3.51E-46	2.6	TTC39C	2.019900498
THSD43	4.50E-50	0.5205631	0.806	0.552	1.09E-45	2.6	THSD4	1.460144928
JAG14	6.18E-50	0.3394182	0.138	0.038	1.49E-45	2.6	JAG1	3.631578947
CD443	6.45E-50	0.5497541	0.814	0.58	1.56E-45	2.6	CD44	1.403448276
TJP13	1.28E-49	0.5451577	0.755	0.497	3.09E-45	2.6	TJP1	1.519114688
PTPRK4	2.16E-49	0.4501307	0.927	0.764	5.20E-45	2.6	PTPRK	1.213350785
SLMAP4	1.49E-48	0.5447992	0.857	0.666	3.60E-44	2.6	SLMAP	1.286786787
ARHGEF383	1.96E-48	0.5753759	0.7	0.475	4.72E-44	2.6	ARHGEF38	1.473684211
KAZN2	2.38E-48	0.6005927	0.413	0.209	5.74E-44	2.6	KAZN	1.976076555
STC22	2.70E-48	0.3980546	0.234	0.09	6.51E-44	2.6	STC2	2.6
FAM222A4	2.78E-48	0.3688619	0.138	0.039	6.70E-44	2.6	FAM222A	3.538461538
B4GALT52	2.84E-48	0.5031043	0.523	0.296	6.84E-44	2.6	B4GALT5	1.766891892
RALGAPA13	5.93E-48	0.575679	0.519	0.296	1.43E-43	2.6	RALGAPA1	1.753378378
C8orf45	8.91E-48	0.1589267	0.829	0.506	2.15E-43	2.6	C8orf4	1.638339921
ARNTL23	1.35E-47	0.5380056	0.306	0.138	3.26E-43	2.6	ARNTL2	2.217391304
STAT5B3	1.38E-47	0.4661245	0.564	0.322	3.33E-43	2.6	STAT5B	1.751552795
PVRL24	1.81E-47	0.4447591	0.51	0.274	4.36E-43	2.6	PVRL2	1.861313869
TACSTD23	2.61E-47	0.471903	0.894	0.723	6.30E-43	2.6	TACSTD2	1.236514523
ACSL34	2.96E-47	0.6833622	0.548	0.327	7.13E-43	2.6	ACSL3	1.675840979
ARHGAP422	3.03E-47	0.5315024	0.19	0.067	7.30E-43	2.6	ARHGAP42	2.835820896
DNAJC125	4.09E-47	0.3291678	0.665	0.379	9.87E-43	2.6	DNAJC12	1.754617414
ASH1L3	4.29E-47	0.5053677	0.773	0.541	1.04E-42	2.6	ASH1L	1.42883549
MYO104	6.40E-47	0.5614433	0.457	0.245	1.54E-42	2.6	MYO10	1.865306122
RASEF5	1.05E-46	0.4250346	0.494	0.263	2.54E-42	2.6	RASEF	1.878326996
RASSF51	1.25E-46	0.3706494	0.144	0.043	3.01E-42	2.6	RASSF5	3.348837209
CCBE14	2.16E-46	0.3517261	0.149	0.045	5.22E-42	2.6	CCBE1	3.311111111
MAST44	2.48E-46	0.5633684	0.758	0.518	5.99E-42	2.6	MAST4	1.463320463
SPPL2A3	4.80E-46	0.5803432	0.458	0.255	1.16E-41	2.6	SPPL2A	1.796078431
MAP3K82	4.84E-46	0.5338992	0.505	0.292	1.17E-41	2.6	MAP3K8	1.729452055
RELL12	7.86E-46	0.4322549	0.266	0.113	1.90E-41	2.6	RELL1	2.353982301
AMPH5	1.91E-45	0.4795568	0.238	0.095	4.61E-41	2.6	AMPH	2.505263158
NEDD42	2.01E-45	0.5102543	0.266	0.115	4.84E-41	2.6	NEDD4	2.313043478
UVRAG4	2.12E-45	0.524155	0.694	0.455	5.12E-41	2.6	UVRAG	1.525274725
MAP4K33	2.69E-45	0.4333373	0.464	0.249	6.49E-41	2.6	MAP4K3	1.863453815
FTH14	5.32E-45	0.3413238	0.945	0.839	1.28E-40	2.6	FTH1	1.126340882
CREB53	6.50E-45	0.5012212	0.586	0.349	1.57E-40	2.6	CREB5	1.679083095
BATF5	7.24E-45	0.4078104	0.192	0.069	1.75E-40	2.6	BATF	2.782608696
FXYD35	7.95E-45	0.4779362	0.83	0.625	1.92E-40	2.6	FXYD3	1.328
SGMS12	9.80E-45	0.6243018	0.509	0.303	2.36E-40	2.6	SGMS1	1.679867987
RUNX13	1.44E-44	0.4138013	0.854	0.635	3.47E-40	2.6	RUNX1	1.34488189
RAB27B4	1.86E-44	0.6042319	0.312	0.146	4.48E-40	2.6	RAB27B	2.136986301
UBE2E22	3.82E-44	0.5489916	0.674	0.441	9.20E-40	2.6	UBE2E2	1.528344671
NEDD4L4	5.12E-44	0.4465971	0.798	0.59	1.23E-39	2.6	NEDD4L	1.352542373
PEBP45	8.43E-44	0.4189663	0.227	0.09	2.03E-39	2.6	PEBP4	2.522222222

HS6ST24	1.46E-43	0.5225753	0.308	0.14	3.52E-39	2.6	HS6ST2	2.2
PAN33	1.51E-43	0.5179973	0.831	0.63	3.65E-39	2.6	PAN3	1.319047619
ARHGEF32	1.79E-43	0.484875	0.604	0.374	4.33E-39	2.6	ARHGEF3	1.614973262
PLAUR2	1.88E-43	0.5892129	0.367	0.184	4.54E-39	2.6	PLAUR	1.994565217
TBC1D18	2.45E-43	0.5656069	0.399	0.211	5.90E-39	2.6	TBC1D1	1.890995261
SLC44A44	3.54E-43	0.4086411	0.261	0.112	8.54E-39	2.6	SLC44A4	2.330357143
RAB11A3	7.89E-43	0.5066749	0.831	0.633	1.90E-38	2.6	RAB11A	1.312796209
PLXND12	2.06E-42	0.288438	0.11	0.029	4.96E-38	2.6	PLXND1	3.793103448
ABCC32	2.73E-42	0.4356399	0.202	0.078	6.57E-38	2.6	ABCC3	2.58974359
TMEM1564	3.56E-42	0.2902294	0.146	0.046	8.59E-38	2.6	TMEM156	3.173913043
ERBB45	4.17E-42	0.1174918	0.806	0.479	1.01E-37	2.6	ERBB4	1.682672234
NR6A14	5.56E-42	0.4487391	0.573	0.342	1.34E-37	2.6	NR6A1	1.675438596
TTLL53	6.58E-42	0.4764613	0.485	0.275	1.59E-37	2.6	TTLL5	1.763636364
SOX44	7.31E-42	0.4294463	0.966	0.833	1.76E-37	2.6	SOX4	1.159663866
ETS23	1.11E-41	0.4544403	0.354	0.179	2.69E-37	2.6	ETS2	1.977653631
DDR14	2.63E-41	0.4484847	0.394	0.209	6.35E-37	2.6	DDR1	1.885167464
NRIP14	2.64E-41	0.4211611	0.473	0.26	6.37E-37	2.6	NRIP1	1.819230769
LYST3	2.82E-41	0.5163165	0.467	0.265	6.80E-37	2.6	LYST	1.762264151
ECT25	3.71E-41	0.4116735	0.385	0.198	8.94E-37	2.6	ECT2	1.944444444
PTPRJ3	3.73E-41	0.4806544	0.516	0.298	9.01E-37	2.6	PTPRJ	1.731543624
DPYD3	4.91E-41	0.5216663	0.726	0.51	1.18E-36	2.6	DPYD	1.423529412
TRIM443	1.07E-40	0.4525626	0.32	0.156	2.58E-36	2.6	TRIM44	2.051282051
SIK22	1.81E-40	0.5037875	0.527	0.316	4.35E-36	2.6	SIK2	1.667721519
INADL3	3.34E-40	0.4227098	0.951	0.815	8.06E-36	2.6	INADL	1.166871166
STAT32	3.56E-40	0.4981172	0.75	0.529	8.58E-36	2.6	STAT3	1.417769376
LAMC23	6.63E-40	0.4495997	0.451	0.256	1.60E-35	2.6	LAMC2	1.76171875
GCH12	1.01E-39	0.336201	0.236	0.099	2.43E-35	2.6	GCH1	2.383838384
ABCA52	1.02E-39	0.4564713	0.26	0.117	2.45E-35	2.6	ABCA5	2.222222222
IER34	2.86E-39	0.3900862	0.53	0.31	6.90E-35	2.6	IER3	1.709677419
CAMTA13	3.21E-39	0.5068609	0.768	0.574	7.74E-35	2.6	CAMTA1	1.337979094
CD92	3.61E-39	0.4589085	0.598	0.378	8.70E-35	2.6	CD9	1.582010582
HSPB84	3.87E-39	0.348738	0.187	0.071	9.34E-35	2.6	HSPB8	2.633802817
MYO1E2	7.16E-39	0.5598616	0.677	0.467	1.73E-34	2.6	MYO1E	1.449678801
OPHN12	1.99E-38	0.3797018	0.534	0.32	4.79E-34	2.6	OPHN1	1.66875
DUSP105	3.43E-38	0.3987376	0.41	0.217	8.27E-34	2.6	DUSP10	1.889400922
RELB3	4.15E-38	0.3954872	0.276	0.129	1.00E-33	2.6	RELB	2.139534884
GPBP14	4.42E-38	0.4009415	0.866	0.68	1.07E-33	2.6	GPBP1	1.273529412
COX6C5	4.93E-38	0.4082429	0.903	0.756	1.19E-33	2.6	COX6C	1.194444444
RNF19B2	5.05E-38	0.486892	0.285	0.136	1.22E-33	2.6	RNF19B	2.095588235
GSK3B4	9.13E-38	0.4848232	0.615	0.39	2.20E-33	2.6	GSK3B	1.576923077
FBXO323	9.38E-38	0.3498797	0.582	0.36	2.26E-33	2.6	FBXO32	1.616666667
FLVCR22	1.15E-37	0.2706929	0.121	0.037	2.78E-33	2.6	FLVCR2	3.27027027
THRB2	1.55E-37	0.3416203	0.688	0.451	3.75E-33	2.6	THRB	1.525498891
RPS6KA34	1.88E-37	0.4491407	0.69	0.454	4.54E-33	2.6	RPS6KA3	1.519823789
USP343	2.26E-37	0.3988232	0.854	0.643	5.46E-33	2.6	USP34	1.3281493
RAB3IP1	2.99E-37	0.4139319	0.286	0.135	7.20E-33	2.6	RAB3IP	2.118518519
MT-ND33	3.14E-37	0.3303211	0.995	0.987	7.57E-33	2.6	MT-ND3	1.00810537
RP11-131L23.12	4.82E-37	0.4704216	0.222	0.096	1.16E-32	2.6	RP11-131L2	2.3125
ARFGEF23	1.07E-36	0.4231909	0.584	0.364	2.58E-32	2.6	ARFGEF2	1.604395604
ATP2B43	1.17E-36	0.4915576	0.553	0.349	2.83E-32	2.6	ATP2B4	1.584527221
LMCD15	1.40E-36	0.5072616	0.233	0.103	3.38E-32	2.6	LMCD1	2.262135922
HIST1H2AC4	1.43E-36	0.4243885	0.573	0.357	3.46E-32	2.6	HIST1H2AC	1.605042017

CCDC932	1.57E-36	0.3859063	0.29	0.141	3.80E-32	2.6	CCDC93	2.056737589
MIR4435-2HG3	1.80E-36	0.4755024	0.711	0.494	4.34E-32	2.6	MIR4435-2	1.439271255
TBC1D94	1.88E-36	0.3282088	0.681	0.439	4.54E-32	2.6	TBC1D9	1.551252847
ABCA43	1.94E-36	0.3167763	0.132	0.043	4.67E-32	2.6	ABCA4	3.069767442
JAK14	2.83E-36	0.4948111	0.601	0.399	6.82E-32	2.6	JAK1	1.506265664
NAALADL24	2.99E-36	0.437747	0.718	0.493	7.22E-32	2.6	NAALADL2	1.456389452
CAB394	5.85E-36	0.4672492	0.554	0.353	1.41E-31	2.6	CAB39	1.569405099
FGD63	6.37E-36	0.4621382	0.541	0.336	1.54E-31	2.6	FGD6	1.610119048
AF127936.94	6.46E-36	0.4192802	0.289	0.14	1.56E-31	2.6	AF127936.9	2.064285714
CEBPB3	7.02E-36	0.3936181	0.323	0.164	1.69E-31	2.6	CEBPB	1.969512195
LAMA14	7.13E-36	0.6898104	0.226	0.1	1.72E-31	2.6	LAMA1	2.26
RB1CC13	7.52E-36	0.4545791	0.678	0.474	1.81E-31	2.6	RB1CC1	1.430379747
TSPAN155	1.64E-35	0.338873	0.227	0.099	3.96E-31	2.6	TSPAN15	2.292929293
FCHSD23	3.15E-35	0.4204083	0.265	0.125	7.60E-31	2.6	FCHSD2	2.12
TPBG3	3.39E-35	0.3639624	0.342	0.177	8.17E-31	2.6	TPBG	1.93220339
AC018359.12	3.82E-35	0.3419656	0.114	0.035	9.21E-31	2.6	AC018359.	3.257142857
ANKRD125	5.33E-35	0.3728644	0.854	0.665	1.29E-30	2.6	ANKRD12	1.284210526
USP431	5.70E-35	0.3932001	0.216	0.094	1.37E-30	2.6	USP43	2.29787234
ZNF385C	6.10E-35	0.2956505	0.121	0.039	1.47E-30	2.6	ZNF385C	3.102564103
PTPRG5	1.29E-34	0.4998128	0.453	0.269	3.11E-30	2.6	PTPRG	1.68401487
IGFBP25	3.03E-34	0.2783527	0.135	0.046	7.31E-30	2.6	IGFBP2	2.934782609
MICAL22	5.58E-34	0.3598139	0.277	0.133	1.35E-29	2.6	MICAL2	2.082706767
KLF43	5.59E-34	0.377681	0.233	0.107	1.35E-29	2.6	KLF4	2.177570093
NBPF13	6.35E-34	0.2894434	0.113	0.036	1.53E-29	2.6	NBPF1	3.138888889
ANXA53	1.19E-33	0.4239176	0.561	0.357	2.86E-29	2.6	ANXA5	1.571428571
SIPA1L33	1.45E-33	0.3393162	0.376	0.207	3.49E-29	2.6	SIPA1L3	1.816425121
ITPR33	1.65E-33	0.3541637	0.182	0.074	3.97E-29	2.6	ITPR3	2.459459459
CD473	1.85E-33	0.3930523	0.608	0.407	4.46E-29	2.6	CD47	1.493857494
HERC43	2.38E-33	0.3704134	0.739	0.518	5.73E-29	2.6	HERC4	1.426640927
MYBL12	2.54E-33	0.4070424	0.173	0.069	6.12E-29	2.6	MYBL1	2.507246377
TRAK12	3.02E-33	0.4356264	0.351	0.191	7.29E-29	2.6	TRAK1	1.837696335
SEMA5A1	3.32E-33	0.2367054	0.078	0.02	8.01E-29	2.6	SEMA5A	3.9
NPAS23	3.38E-33	0.3835355	0.569	0.363	8.16E-29	2.6	NPAS2	1.567493113
KIAA12173	4.32E-33	0.303226	0.952	0.795	1.04E-28	2.6	KIAA1217	1.197484277
PARD33	4.46E-33	0.4477481	0.816	0.634	1.07E-28	2.6	PARD3	1.287066246
MT-ND13	5.18E-33	0.2207442	0.994	0.979	1.25E-28	2.6	MT-ND1	1.015321757
SPINT12	5.25E-33	0.3569223	0.336	0.178	1.26E-28	2.6	SPINT1	1.887640449
CDK83	5.28E-33	0.4588129	0.407	0.234	1.27E-28	2.6	CDK8	1.739316239
GTDC12	6.08E-33	0.4199517	0.222	0.101	1.47E-28	2.6	GTDC1	2.198019802
CFB2	6.09E-33	0.4845979	0.292	0.149	1.47E-28	2.6	CFB	1.959731544
SMCHD14	6.73E-33	0.4389742	0.596	0.4	1.62E-28	2.6	SMCHD1	1.49
SCGB1D23	7.91E-33	0.4781226	0.245	0.114	1.91E-28	2.6	SCGB1D2	2.149122807
ADAM323	1.06E-32	0.3320398	0.4	0.223	2.55E-28	2.6	ADAM32	1.793721973
DNAJB64	2.85E-32	0.4718959	0.569	0.375	6.88E-28	2.6	DNAJB6	1.517333333
PSD34	3.22E-32	0.4544577	0.535	0.339	7.77E-28	2.6	PSD3	1.578171091
PDGFB3	3.63E-32	0.2998957	0.114	0.037	8.76E-28	2.6	PDGFB	3.081081081
GULP13	4.74E-32	0.3801292	0.365	0.201	1.14E-27	2.6	GULP1	1.815920398
ST3GAL14	4.84E-32	0.3548398	0.356	0.195	1.17E-27	2.6	ST3GAL1	1.825641026
ARHGEF124	4.87E-32	0.2957851	0.803	0.567	1.17E-27	2.6	ARHGEF12	1.41622575
PLEKHA12	6.79E-32	0.3367995	0.296	0.151	1.64E-27	2.6	PLEKHA1	1.960264901
SSUH22	6.81E-32	0.2022167	0.062	0.014	1.64E-27	2.6	SSUH2	4.428571429
OTUD7A2	8.39E-32	0.3219611	0.216	0.096	2.02E-27	2.6	OTUD7A	2.25

LARGE4	8.75E-32	0.4189446	0.513	0.32	2.11E-27	2.6	LARGE	1.603125
KTN15	1.19E-31	0.3674666	0.754	0.533	2.86E-27	2.6	KTN1	1.414634146
MAP3K54	1.46E-31	0.490913	0.551	0.358	3.51E-27	2.6	MAP3K5	1.539106145
AREG5	1.65E-31	0.1023473	0.714	0.44	3.97E-27	2.6	AREG	1.622727273
OXTR1	1.71E-31	0.1428824	0.045	0.008	4.12E-27	2.6	OXTR	5.625
DMXL21	1.72E-31	0.3391725	0.12	0.041	4.15E-27	2.6	DMXL2	2.926829268
NAMPT3	2.46E-31	0.4057575	0.813	0.642	5.94E-27	2.6	NAMPT	1.26635514
AHNAK4	2.69E-31	0.3985958	0.548	0.348	6.47E-27	2.6	AHNAK	1.574712644
RALGAPA21	4.71E-31	0.4441666	0.424	0.252	1.14E-26	2.6	RALGAPA2	1.682539683
FBXL173	4.86E-31	0.3831724	0.436	0.259	1.17E-26	2.6	FBXL17	1.683397683
IER55	5.66E-31	0.3431318	0.23	0.108	1.36E-26	2.6	IER5	2.12962963
ACSL13	5.92E-31	0.4222488	0.355	0.198	1.43E-26	2.6	ACSL1	1.792929293
TNF3	7.02E-31	0.353892	0.187	0.081	1.69E-26	2.6	TNF	2.308641975
MAFF2	7.10E-31	0.3909982	0.382	0.219	1.71E-26	2.6	MAFF	1.744292237
MAP3K18	1.04E-30	0.2826594	0.687	0.448	2.51E-26	2.6	MAP3K1	1.533482143
MED153	1.15E-30	0.4032294	0.318	0.166	2.77E-26	2.6	MED15	1.915662651
GAREM12	1.38E-30	0.3912262	0.498	0.313	3.33E-26	2.6	GAREM1	1.591054313
TTC64	1.60E-30	0.2629642	0.329	0.168	3.86E-26	2.6	TTC6	1.958333333
CYB5A4	3.20E-30	0.3104382	0.435	0.252	7.71E-26	2.6	CYB5A	1.726190476
ARHGAP293	3.51E-30	0.2210336	0.782	0.564	8.45E-26	2.6	ARHGAP29	1.386524823
GPRC5A2	6.66E-30	0.328486	0.584	0.383	1.61E-25	2.6	GPRC5A	1.524804178
ASCC33	6.85E-30	0.3823666	0.404	0.237	1.65E-25	2.6	ASCC3	1.70464135
MIPOL14	7.89E-30	0.3452324	0.332	0.178	1.90E-25	2.6	MIPOL1	1.865168539
NETO22	8.01E-30	0.3296761	0.136	0.051	1.93E-25	2.6	NETO2	2.666666667
TBC1D2B2	8.08E-30	0.2729344	0.107	0.035	1.95E-25	2.6	TBC1D2B	3.057142857
NCOA74	1.14E-29	0.2944245	0.652	0.431	2.74E-25	2.6	NCOA7	1.512761021
RICTOR3	1.33E-29	0.2936323	0.814	0.614	3.21E-25	2.6	RICTOR	1.325732899
SLC7A25	1.53E-29	0.2182103	0.51	0.292	3.69E-25	2.6	SLC7A2	1.746575342
PTAFR2	2.16E-29	0.1766598	0.064	0.015	5.21E-25	2.6	PTAFR	4.266666667
MT-CYB4	3.53E-29	0.2249344	0.995	0.991	8.52E-25	2.6	MT-CYB	1.004036327
CHD23	5.09E-29	0.2634023	0.747	0.539	1.23E-24	2.6	CHD2	1.385899814
CRADD2	5.91E-29	0.4240414	0.28	0.145	1.42E-24	2.6	CRADD	1.931034483
PHF203	6.71E-29	0.3603556	0.546	0.349	1.62E-24	2.6	PHF20	1.564469914
MT-CO34	6.93E-29	0.2108861	0.997	0.995	1.67E-24	2.6	MT-CO3	1.00201005
PARD3B2	7.36E-29	0.3853174	0.349	0.196	1.78E-24	2.6	PARD3B	1.780612245
TMOD34	1.01E-28	0.363855	0.598	0.395	2.43E-24	2.6	TMOD3	1.513924051
HIPK25	1.28E-28	0.3308878	0.569	0.365	3.08E-24	2.6	HIPK2	1.55890411
DLEU23	2.10E-28	0.3395205	0.373	0.216	5.06E-24	2.6	DLEU2	1.726851852
LAMB33	2.15E-28	0.2577318	0.396	0.233	5.18E-24	2.6	LAMB3	1.699570815
TNFRSF10B3	2.29E-28	0.3419871	0.355	0.199	5.53E-24	2.6	TNFRSF10E	1.783919598
SAMD4A3	3.15E-28	0.3756415	0.802	0.615	7.59E-24	2.6	SAMD4A	1.304065041
COL4A55	4.79E-28	0.2802801	0.383	0.213	1.15E-23	2.6	COL4A5	1.798122066
ITSN24	5.22E-28	0.3995895	0.444	0.273	1.26E-23	2.6	ITSN2	1.626373626
FAM160A13	6.83E-28	0.3609737	0.808	0.643	1.65E-23	2.6	FAM160A1	1.256609642
FAM177B3	7.15E-28	0.3078956	0.68	0.484	1.72E-23	2.6	FAM177B	1.404958678
PRKG13	7.85E-28	0.4239295	0.273	0.144	1.89E-23	2.6	PRKG1	1.895833333
ZC3HAV14	1.20E-27	0.2958303	0.332	0.183	2.90E-23	2.6	ZC3HAV1	1.81420765
CTNND25	2.01E-27	0.3194041	0.285	0.149	4.85E-23	2.6	CTNND2	1.912751678
RP1-78O14.14	2.25E-27	0.2939279	0.354	0.198	5.42E-23	2.6	RP1-78O14	1.787878788
NAB11	3.52E-27	0.2645863	0.155	0.064	8.49E-23	2.6	NAB1	2.421875
CYLD4	3.89E-27	0.3699207	0.358	0.206	9.39E-23	2.6	CYLD	1.737864078
CSGALNACT23	4.47E-27	0.3495146	0.244	0.122	1.08E-22	2.6	CSGALNAC	2

AGO23	4.90E-27	0.3739997	0.354	0.206	1.18E-22	2.6	AGO2	1.718446602
PTPRM3	8.61E-27	0.2623643	0.402	0.235	2.08E-22	2.6	PTPRM	1.710638298
SIX43	1.03E-26	0.2582328	0.174	0.076	2.49E-22	2.6	SIX4	2.289473684
ATAD2B3	1.08E-26	0.3782693	0.344	0.196	2.59E-22	2.6	ATAD2B	1.755102041
NDUFV24	1.23E-26	0.3736231	0.428	0.265	2.95E-22	2.6	NDUFV2	1.61509434
MYO64	1.30E-26	0.2170048	0.924	0.752	3.14E-22	2.6	MYO6	1.228723404
NEAT14	1.51E-26	0.2190558	0.998	0.945	3.64E-22	2.6	NEAT1	1.056084656
KLRD13	1.53E-26	0.3508119	0.294	0.157	3.70E-22	2.6	KLRD1	1.872611465
RCN15	1.73E-26	0.2965692	0.282	0.149	4.17E-22	2.6	RCN1	1.89261745
PLD13	1.76E-26	0.3528194	0.246	0.125	4.23E-22	2.6	PLD1	1.968
PERP4	1.81E-26	0.3344689	0.629	0.447	4.37E-22	2.6	PERP	1.407158837
TMEM28	1.82E-26	0.3356029	0.531	0.34	4.39E-22	2.6	TMEM2	1.561764706
YTHDC16	2.08E-26	0.3880636	0.551	0.378	5.01E-22	2.6	YTHDC1	1.457671958
PVRL43	2.09E-26	0.3351315	0.33	0.187	5.04E-22	2.6	PVRL4	1.764705882
RBMS13	2.20E-26	0.4536426	0.515	0.337	5.29E-22	2.6	RBMS1	1.528189911
NCKAP53	2.36E-26	0.3013054	0.316	0.173	5.69E-22	2.6	NCKAP5	1.826589595
STYK15	2.75E-26	0.2878012	0.152	0.063	6.64E-22	2.6	STYK1	2.412698413
NPTN4	3.15E-26	0.3008966	0.337	0.19	7.60E-22	2.6	NPTN	1.773684211
ZEB13	3.21E-26	0.2791373	0.132	0.052	7.75E-22	2.6	ZEB1	2.538461538
TBK11	3.23E-26	0.3688101	0.458	0.287	7.78E-22	2.6	TBK1	1.595818815
TAB22	4.84E-26	0.3269568	0.444	0.277	1.17E-21	2.6	TAB2	1.602888087
SPG113	4.94E-26	0.3175146	0.461	0.288	1.19E-21	2.6	SPG11	1.600694444
UBR23	5.57E-26	0.4095274	0.557	0.383	1.34E-21	2.6	UBR2	1.454308094
SERF25	6.70E-26	0.1733388	0.592	0.386	1.62E-21	2.6	SERF2	1.533678756
DIO2-AS14	7.12E-26	0.2610941	0.179	0.08	1.72E-21	2.6	DIO2-AS1	2.2375
TNS13	1.31E-25	0.2452328	0.114	0.042	3.16E-21	2.6	TNS1	2.714285714
RGS161	1.35E-25	0.1539388	0.041	0.008	3.25E-21	2.6	RGS16	5.125
ITGA54	1.41E-25	0.3071609	0.202	0.096	3.39E-21	2.6	ITGA5	2.104166667
C6orf1321	2.23E-25	0.3176974	0.275	0.148	5.39E-21	2.6	C6orf132	1.858108108
CNOT42	2.27E-25	0.3484499	0.576	0.393	5.48E-21	2.6	CNOT4	1.465648855
MT-ATP62	2.77E-25	0.1860069	0.995	0.991	6.68E-21	2.6	MT-ATP6	1.004036327
TOP15	2.92E-25	0.3193349	0.624	0.439	7.03E-21	2.6	TOP1	1.421412301
SMIM145	3.29E-25	0.2642747	0.596	0.391	7.94E-21	2.6	SMIM14	1.524296675
SHANK22	4.80E-25	0.3123721	0.38	0.226	1.16E-20	2.6	SHANK2	1.681415929
CD993	5.31E-25	0.3235196	0.253	0.132	1.28E-20	2.6	CD99	1.916666667
AIM13	5.55E-25	0.315122	0.634	0.438	1.34E-20	2.6	AIM1	1.447488584
MSN4	9.33E-25	0.360121	0.584	0.413	2.25E-20	2.6	MSN	1.414043584
MAML32	1.01E-24	0.2978731	0.52	0.344	2.43E-20	2.6	MAML3	1.511627907
SERHL23	1.17E-24	0.3071297	0.256	0.135	2.82E-20	2.6	SERHL2	1.896296296
CUL4A3	1.46E-24	0.2925796	0.312	0.175	3.52E-20	2.6	CUL4A	1.782857143
PATL11	1.82E-24	0.2664525	0.261	0.138	4.39E-20	2.6	PATL1	1.891304348
AZIN15	1.98E-24	0.2859091	0.345	0.199	4.79E-20	2.6	AZIN1	1.733668342
NBAT13	2.10E-24	0.3103	0.226	0.114	5.06E-20	2.6	NBAT1	1.98245614
TBC1D84	2.15E-24	0.382912	0.607	0.422	5.18E-20	2.6	TBC1D8	1.438388626
ANKRD442	2.22E-24	0.3384605	0.163	0.074	5.35E-20	2.6	ANKRD44	2.202702703
CLIC64	2.25E-24	0.2615762	0.366	0.212	5.44E-20	2.6	CLIC6	1.726415094
ARL5B2	2.29E-24	0.3085555	0.211	0.104	5.52E-20	2.6	ARL5B	2.028846154
REPS13	2.58E-24	0.3203506	0.472	0.302	6.22E-20	2.6	REPS1	1.562913907
RALA3	2.60E-24	0.3219252	0.355	0.209	6.28E-20	2.6	RALA	1.698564593
BID2	2.74E-24	0.2788269	0.247	0.128	6.61E-20	2.6	BID	1.9296875
CHMP4B4	2.89E-24	0.2851475	0.329	0.186	6.96E-20	2.6	CHMP4B	1.768817204
NFAT54	3.36E-24	0.2576899	0.797	0.622	8.10E-20	2.6	NFAT5	1.281350482

KMT2A3	4.11E-24	0.2989166	0.608	0.413	9.92E-20	2.6	KMT2A	1.472154964
UXS14	4.48E-24	0.2590708	0.187	0.088	1.08E-19	2.6	UXS1	2.125
NFKBIE2	6.31E-24	0.190918	0.084	0.027	1.52E-19	2.6	NFKBIE	3.111111111
OPN34	6.56E-24	0.2856015	0.19	0.092	1.58E-19	2.6	OPN3	2.065217391
SMYD32	8.01E-24	0.2818516	0.509	0.334	1.93E-19	2.6	SMYD3	1.523952096
WWC14	9.03E-24	0.3455323	0.586	0.412	2.18E-19	2.6	WWC1	1.422330097
FNDC3A2	1.11E-23	0.4097233	0.526	0.363	2.67E-19	2.6	FNDC3A	1.449035813
EXPH52	1.23E-23	0.3255618	0.309	0.175	2.96E-19	2.6	EXPH5	1.765714286
PSTPIP22	1.46E-23	0.2937396	0.367	0.219	3.53E-19	2.6	PSTPIP2	1.675799087
PGR4	1.52E-23	0.3061902	0.172	0.079	3.66E-19	2.6	PGR	2.17721519
TMEM45B4	1.69E-23	0.2868815	0.29	0.161	4.07E-19	2.6	TMEM45B	1.801242236
SOCS23	1.69E-23	0.180762	0.074	0.023	4.08E-19	2.6	SOCS2	3.217391304
TOM1L23	1.88E-23	0.3017569	0.467	0.294	4.54E-19	2.6	TOM1L2	1.588435374
IL4R3	2.16E-23	0.2660491	0.256	0.137	5.22E-19	2.6	IL4R	1.868613139
BRAF3	2.22E-23	0.3477562	0.6	0.413	5.36E-19	2.6	BRAF	1.452784504
TLE13	2.30E-23	0.3081741	0.356	0.213	5.56E-19	2.6	TLE1	1.671361502
WNT5A2	2.65E-23	0.2446171	0.101	0.037	6.39E-19	2.6	WNT5A	2.72972973
ANKRD112	2.75E-23	0.3333761	0.477	0.315	6.63E-19	2.6	ANKRD11	1.514285714
ARID1A2	3.97E-23	0.2805135	0.387	0.235	9.57E-19	2.6	ARID1A	1.646808511
MYO1D4	4.15E-23	0.3362886	0.479	0.311	1.00E-18	2.6	MYO1D	1.540192926
RP3-523E19.22	4.42E-23	0.2022919	0.107	0.04	1.07E-18	2.6	RP3-523E1!	2.675
KIAA13245	4.52E-23	0.2867299	0.374	0.223	1.09E-18	2.6	KIAA1324	1.677130045
F33	5.20E-23	0.2710963	0.214	0.107	1.25E-18	2.6	F3	2
SNX102	6.46E-23	0.2667053	0.151	0.067	1.56E-18	2.6	SNX10	2.253731343
FNBP13	6.65E-23	0.2995723	0.712	0.519	1.60E-18	2.6	FNBP1	1.371868979
TIMP22	6.96E-23	0.2005541	0.145	0.062	1.68E-18	2.6	TIMP2	2.338709677
MB21D22	7.62E-23	0.2788354	0.19	0.092	1.84E-18	2.6	MB21D2	2.065217391
ATP6AP13	7.89E-23	0.1972122	0.126	0.051	1.90E-18	2.6	ATP6AP1	2.470588235
LTBP14	8.59E-23	0.2638835	0.331	0.195	2.07E-18	2.6	LTBP1	1.697435897
ABHD183	8.76E-23	0.3370934	0.508	0.338	2.11E-18	2.6	ABHD18	1.50295858
SPIDR4	9.82E-23	0.2476622	0.784	0.593	2.37E-18	2.6	SPIDR	1.322091062
EIF4G33	1.07E-22	0.2469889	0.701	0.504	2.57E-18	2.6	EIF4G3	1.390873016
URGCP1	1.12E-22	0.2088548	0.106	0.04	2.71E-18	2.6	URGCP	2.65
MYH94	1.13E-22	0.2134079	0.704	0.507	2.73E-18	2.6	MYH9	1.388560158
CNN34	1.35E-22	0.2993734	0.692	0.508	3.26E-18	2.6	CNN3	1.362204724
CREBBP3	1.38E-22	0.2936005	0.452	0.294	3.33E-18	2.6	CREBBP	1.537414966
CRY21	1.59E-22	0.3165393	0.197	0.099	3.83E-18	2.6	CRY2	1.98989899
USP475	1.72E-22	0.2701662	0.601	0.414	4.16E-18	2.6	USP47	1.451690821
AR5	2.04E-22	0.2959425	0.284	0.155	4.93E-18	2.6	AR	1.832258065
ABAT3	2.10E-22	0.2851896	0.131	0.055	5.06E-18	2.6	ABAT	2.381818182
ATXN13	2.14E-22	0.2058881	0.704	0.507	5.17E-18	2.6	ATXN1	1.388560158
SSH23	2.17E-22	0.3516935	0.553	0.384	5.24E-18	2.6	SSH2	1.440104167
WWC23	2.29E-22	0.2892167	0.33	0.193	5.52E-18	2.6	WWC2	1.70984456
SRGAP24	2.60E-22	0.3341853	0.26	0.143	6.26E-18	2.6	SRGAP2	1.818181818
EFTUD12	2.63E-22	0.2890062	0.272	0.151	6.35E-18	2.6	EFTUD1	1.801324503
IFNGR12	2.78E-22	0.3452442	0.414	0.266	6.70E-18	2.6	IFNGR1	1.556390977
IL6ST4	3.01E-22	0.3112083	0.491	0.327	7.27E-18	2.6	IL6ST	1.501529052
CDKL44	3.26E-22	0.1833131	0.074	0.023	7.85E-18	2.6	CDKL4	3.217391304
EPB41L54	3.39E-22	0.329284	0.453	0.29	8.17E-18	2.6	EPB41L5	1.562068966
TSC22D33	4.08E-22	0.2460809	0.318	0.182	9.84E-18	2.6	TSC22D3	1.747252747
AC007319.15	4.11E-22	0.2402792	0.18	0.086	9.92E-18	2.6	AC007319.	2.093023256
CMYA53	4.43E-22	0.3313562	0.201	0.102	1.07E-17	2.6	CMYA5	1.970588235

RANBP94	5.17E-22	0.3014886	0.452	0.293	1.25E-17	2.6	RANBP9	1.542662116
TP53INP12	6.94E-22	0.2627135	0.166	0.078	1.67E-17	2.6	TP53INP1	2.128205128
YAP13	7.13E-22	0.265796	0.712	0.527	1.72E-17	2.6	YAP1	1.351043643
GEM2	8.15E-22	0.2537894	0.117	0.048	1.97E-17	2.6	GEM	2.4375
FMNL3	1.00E-21	0.1545608	0.058	0.016	2.42E-17	2.6	FMNL3	3.625
UBL34	1.01E-21	0.3045274	0.331	0.197	2.44E-17	2.6	UBL3	1.680203046
ZNF7042	1.23E-21	0.2893711	0.282	0.16	2.97E-17	2.6	ZNF704	1.7625
FOSL22	1.35E-21	0.3207127	0.381	0.237	3.25E-17	2.6	FOSL2	1.607594937
TNFSF13B3	1.69E-21	0.1881795	0.119	0.048	4.07E-17	2.6	TNFSF13B	2.479166667
NEO12	2.05E-21	0.2693236	0.189	0.094	4.93E-17	2.6	NEO1	2.010638298
RASSF63	2.17E-21	0.2172189	0.149	0.067	5.22E-17	2.6	RASSF6	2.223880597
DGKH3	2.28E-21	0.2567085	0.468	0.302	5.50E-17	2.6	DGKH	1.549668874
LITAF3	2.29E-21	0.3029507	0.606	0.431	5.51E-17	2.6	LITAF	1.406032483
ERBB2IP4	3.05E-21	0.2121755	0.606	0.421	7.36E-17	2.6	ERBB2IP	1.439429929
REL3	3.25E-21	0.3987566	0.44	0.295	7.84E-17	2.6	REL	1.491525424
NR4A24	3.26E-21	0.2679105	0.208	0.107	7.87E-17	2.6	NR4A2	1.943925234
DSTN3	3.33E-21	0.2188526	0.756	0.577	8.02E-17	2.6	DSTN	1.310225303
HIST1H2BJ2	3.35E-21	0.3348792	0.234	0.128	8.07E-17	2.6	HIST1H2BJ	1.828125
LSM14A3	3.76E-21	0.2751666	0.517	0.344	9.05E-17	2.6	LSM14A	1.502906977
CLIC44	3.87E-21	0.2748602	0.731	0.554	9.33E-17	2.6	CLIC4	1.319494585
DUSP54	4.44E-21	0.2552355	0.383	0.233	1.07E-16	2.6	DUSP5	1.643776824
FEM1B4	5.28E-21	0.2696349	0.289	0.165	1.27E-16	2.6	FEM1B	1.751515152
MYCBP23	5.51E-21	0.2624816	0.374	0.229	1.33E-16	2.6	MYCBP2	1.633187773
TEAD14	6.44E-21	0.3302745	0.619	0.446	1.55E-16	2.6	TEAD1	1.387892377
COL12A14	6.47E-21	0.2000408	0.116	0.047	1.56E-16	2.6	COL12A1	2.468085106
CEP3502	7.30E-21	0.273955	0.346	0.213	1.76E-16	2.6	CEP350	1.624413146
ABHD32	8.45E-21	0.2716041	0.294	0.17	2.04E-16	2.6	ABHD3	1.729411765
PRKAG23	8.72E-21	0.2929359	0.26	0.146	2.10E-16	2.6	PRKAG2	1.780821918
NR1D22	1.21E-20	0.2807362	0.369	0.23	2.93E-16	2.6	NR1D2	1.604347826
RP11-486O13.43	1.33E-20	0.2657254	0.196	0.1	3.20E-16	2.6	RP11-486O	1.96
C3orf524	1.48E-20	0.2399983	0.28	0.157	3.56E-16	2.6	C3orf52	1.78343949
NFE2L23	1.67E-20	0.3142722	0.54	0.374	4.04E-16	2.6	NFE2L2	1.443850267
CYTH13	1.87E-20	0.2394658	0.266	0.149	4.52E-16	2.6	CYTH1	1.785234899
MED13L3	1.97E-20	0.3388873	0.823	0.683	4.74E-16	2.6	MED13L	1.204978038
KCCAT2111	2.05E-20	0.263139	0.208	0.108	4.95E-16	2.6	KCCAT211	1.925925926
STK38L3	2.10E-20	0.2422697	0.32	0.19	5.06E-16	2.6	STK38L	1.684210526
EFHD15	2.19E-20	0.1166776	0.474	0.293	5.28E-16	2.6	EFHD1	1.61774744
FARP12	2.33E-20	0.2150966	0.557	0.373	5.61E-16	2.6	FARP1	1.493297587
WLS5	2.95E-20	0.2292385	0.296	0.171	7.10E-16	2.6	WLS	1.730994152
LINC002652	3.15E-20	0.1878074	0.104	0.041	7.60E-16	2.6	LINC00265	2.536585366
DHRSX3	3.15E-20	0.2946015	0.418	0.272	7.60E-16	2.6	DHRSX	1.536764706
SARAF5	4.41E-20	0.2393751	0.52	0.346	1.06E-15	2.6	SARAF	1.502890173
LINC009702	4.65E-20	0.2288568	0.091	0.034	1.12E-15	2.6	LINC00970	2.676470588
SLC41A23	6.60E-20	0.2776689	0.218	0.116	1.59E-15	2.6	SLC41A2	1.879310345
ISG202	7.07E-20	0.2246116	0.143	0.065	1.71E-15	2.6	ISG20	2.2
BTRC4	7.51E-20	0.2362505	0.408	0.258	1.81E-15	2.6	BTRC	1.581395349
RBM474	7.66E-20	0.3211579	0.776	0.631	1.85E-15	2.6	RBM47	1.229793978
IFIH11	7.67E-20	0.2682622	0.182	0.093	1.85E-15	2.6	IFIH1	1.956989247
PIK3CA3	8.63E-20	0.269822	0.434	0.281	2.08E-15	2.6	PIK3CA	1.544483986
EHF3	8.65E-20	0.1996342	0.658	0.465	2.09E-15	2.6	EHF	1.415053763
ZFC3H13	9.03E-20	0.2791206	0.396	0.253	2.18E-15	2.6	ZFC3H1	1.565217391
ACRC4	9.07E-20	0.2306457	0.191	0.097	2.19E-15	2.6	ACRC	1.969072165

FAM46A3	9.90E-20	0.223155	0.287	0.166	2.39E-15	2.6	FAM46A	1.728915663
OSBPL33	1.15E-19	0.2090756	0.37	0.226	2.78E-15	2.6	OSBPL3	1.637168142
CREBRF2	1.22E-19	0.2992256	0.402	0.26	2.95E-15	2.6	CREBRF	1.546153846
ZFP362	1.24E-19	0.1958746	0.214	0.114	2.99E-15	2.6	ZFP36	1.877192982
RP11-318C2.12	1.28E-19	0.1845697	0.086	0.032	3.10E-15	2.6	RP11-318C	2.6875
ZNRF23	1.38E-19	0.2394414	0.407	0.257	3.32E-15	2.6	ZNRF2	1.583657588
IDS3	1.41E-19	0.2147023	0.16	0.077	3.40E-15	2.6	IDS	2.077922078
DIRC31	1.61E-19	0.215712	0.066	0.021	3.89E-15	2.6	DIRC3	3.142857143
AC018816.35	1.63E-19	0.2409735	0.188	0.095	3.93E-15	2.6	AC018816.	1.978947368
CREB3L13	1.63E-19	0.1685894	0.072	0.024	3.93E-15	2.6	CREB3L1	3
SSBP33	1.72E-19	0.1869799	0.227	0.121	4.15E-15	2.6	SSBP3	1.876033058
WASL5	1.91E-19	0.2426944	0.364	0.226	4.59E-15	2.6	WASL	1.610619469
CMTM64	2.12E-19	0.2613734	0.45	0.297	5.12E-15	2.6	CMTM6	1.515151515
TMSB4X3	2.18E-19	0.1182312	0.974	0.935	5.25E-15	2.6	TMSB4X	1.04171123
RP11-290O12.22	2.49E-19	0.2822841	0.137	0.063	6.00E-15	2.6	RP11-290O	2.174603175
RORA3	2.51E-19	0.3516728	0.726	0.567	6.04E-15	2.6	RORA	1.28042328
LCOR3	2.56E-19	0.270536	0.406	0.26	6.18E-15	2.6	LCOR	1.561538462
ARIH14	2.90E-19	0.2838234	0.674	0.502	6.99E-15	2.6	ARIH1	1.342629482
SUSD64	3.20E-19	0.2590657	0.421	0.274	7.73E-15	2.6	SUSD6	1.53649635
AGO42	3.48E-19	0.249645	0.293	0.172	8.39E-15	2.6	AGO4	1.703488372
FAM188A4	3.64E-19	0.3111097	0.186	0.097	8.78E-15	2.6	FAM188A	1.917525773
ARAP22	3.67E-19	0.2617473	0.281	0.164	8.84E-15	2.6	ARAP2	1.713414634
BCL32	4.64E-19	0.2393267	0.19	0.1	1.12E-14	2.6	BCL3	1.9
LHFP2	5.32E-19	0.2063376	0.081	0.03	1.28E-14	2.6	LHFP	2.7
MBOAT12	5.87E-19	0.2891078	0.234	0.132	1.42E-14	2.6	MBOAT1	1.772727273
CCNI3	6.86E-19	0.2719709	0.776	0.621	1.65E-14	2.6	CCNI	1.249597424
PTMA2	6.88E-19	0.1738199	0.777	0.615	1.66E-14	2.6	PTMA	1.263414634
MARK21	7.76E-19	0.2681959	0.221	0.122	1.87E-14	2.6	MARK2	1.81147541
BAALC-AS12	8.98E-19	0.1690222	0.075	0.026	2.17E-14	2.6	BAALC-AS1	2.884615385
ABCD35	9.55E-19	0.256981	0.296	0.175	2.30E-14	2.6	ABCD3	1.691428571
TNFSF103	9.78E-19	0.2138598	0.633	0.466	2.36E-14	2.6	TNFSF10	1.358369099
UBN23	9.94E-19	0.2622767	0.413	0.269	2.40E-14	2.6	UBN2	1.535315985
TLE42	1.17E-18	0.2729635	0.498	0.333	2.82E-14	2.6	TLE4	1.495495495
EPB41L4B3	1.29E-18	0.2454356	0.284	0.166	3.12E-14	2.6	EPB41L4B	1.710843373
MAPK84	1.31E-18	0.2053215	0.486	0.328	3.16E-14	2.6	MAPK8	1.481707317
NNMT2	1.41E-18	0.2093854	0.197	0.105	3.39E-14	2.6	NNMT	1.876190476
CCDC1732	1.67E-18	0.2138738	0.14	0.066	4.03E-14	2.6	CCDC173	2.121212121
CSRNP13	1.75E-18	0.1756821	0.106	0.044	4.21E-14	2.6	CSRNP1	2.409090909
RP11-1038A11.32	1.83E-18	0.2048292	0.063	0.02	4.41E-14	2.6	RP11-1038.	3.15
MARK33	1.86E-18	0.255762	0.637	0.462	4.48E-14	2.6	MARK3	1.378787879
TULP42	1.99E-18	0.2455578	0.663	0.494	4.79E-14	2.6	TULP4	1.342105263
ACTN14	2.08E-18	0.1755679	0.501	0.337	5.02E-14	2.6	ACTN1	1.486646884
TNFRSF12A3	2.21E-18	0.2286433	0.143	0.068	5.32E-14	2.6	TNFRSF12A	2.102941176
SLC11A23	2.34E-18	0.2384304	0.498	0.341	5.64E-14	2.6	SLC11A2	1.460410557
LAMA33	2.61E-18	0.2397084	0.318	0.189	6.30E-14	2.6	LAMA3	1.682539683
TSC22D13	2.87E-18	0.2301492	0.524	0.36	6.92E-14	2.6	TSC22D1	1.455555556
ISCU2	2.90E-18	0.1860675	0.128	0.058	7.00E-14	2.6	ISCU	2.206896552
IGF2R3	3.02E-18	0.2821094	0.306	0.186	7.28E-14	2.6	IGF2R	1.64516129
SLCO3A13	3.38E-18	0.2704455	0.297	0.181	8.15E-14	2.6	SLCO3A1	1.640883978
IKBKB3	3.39E-18	0.229893	0.274	0.161	8.16E-14	2.6	IKBKB	1.701863354
FAM177A14	3.85E-18	0.2011468	0.223	0.121	9.29E-14	2.6	FAM177A1	1.842975207
PTK23	3.91E-18	0.2317309	0.793	0.635	9.43E-14	2.6	PTK2	1.248818898

RP11-1105O14.12	4.27E-18	0.1111522	0.036	0.008	1.03E-13	2.6	RP11-1105(	4.5
MGAT4A2	4.58E-18	0.2022403	0.174	0.088	1.10E-13	2.6	MGAT4A	1.977272727
RP11-779O18.34	4.73E-18	0.3262851	0.369	0.239	1.14E-13	2.6	RP11-779O	1.543933054
ZBTB433	4.82E-18	0.2032361	0.294	0.175	1.16E-13	2.6	ZBTB43	1.68
ZBTB23	5.23E-18	0.188574	0.108	0.046	1.26E-13	2.6	ZBTB2	2.347826087
KIAA15223	5.24E-18	0.2435431	0.356	0.224	1.26E-13	2.6	KIAA1522	1.589285714
NIPBL4	5.33E-18	0.2303716	0.702	0.542	1.28E-13	2.6	NIPBL	1.295202952
RALY3	5.68E-18	0.3010952	0.291	0.177	1.37E-13	2.6	RALY	1.644067797
QKI3	6.59E-18	0.1902254	0.713	0.533	1.59E-13	2.6	QKI	1.337711069
RP13-726E6.21	7.03E-18	0.2175155	0.139	0.066	1.69E-13	2.6	RP13-726E(	2.106060606
AC013461.11	7.52E-18	0.2912387	0.319	0.199	1.81E-13	2.6	AC013461.	1.603015075
ITM2B5	8.53E-18	0.2968096	0.495	0.344	2.06E-13	2.6	ITM2B	1.438953488
MEF2A3	8.71E-18	0.205103	0.582	0.415	2.10E-13	2.6	MEF2A	1.402409639
ZSWIM45	9.32E-18	0.1979206	0.302	0.183	2.25E-13	2.6	ZSWIM4	1.650273224
SMG12	1.13E-17	0.1908065	0.482	0.321	2.73E-13	2.6	SMG1	1.501557632
MCF24	1.19E-17	0.1787549	0.068	0.023	2.87E-13	2.6	MCF2	2.956521739
SYTL43	1.25E-17	0.2579579	0.22	0.123	3.02E-13	2.6	SYTL4	1.788617886
HES13	1.36E-17	0.1517765	0.491	0.337	3.29E-13	2.6	HES1	1.456973294
CCNL13	1.39E-17	0.2213947	0.572	0.405	3.35E-13	2.6	CCNL1	1.412345679
LINC005981	1.65E-17	0.195169	0.089	0.036	3.97E-13	2.6	LINC00598	2.472222222
RP11-774D14.12	2.15E-17	0.2272933	0.19	0.102	5.19E-13	2.6	RP11-774D	1.862745098
PRKCE4	2.19E-17	0.2446993	0.31	0.192	5.29E-13	2.6	PRKCE	1.614583333
SLC30A92	2.23E-17	0.2235275	0.254	0.149	5.38E-13	2.6	SLC30A9	1.704697987
NOVA12	2.23E-17	0.1832382	0.139	0.066	5.38E-13	2.6	NOVA1	2.106060606
RAF12	2.33E-17	0.2408817	0.316	0.196	5.62E-13	2.6	RAF1	1.612244898
CSF12	2.49E-17	0.2103613	0.121	0.056	6.00E-13	2.6	CSF1	2.160714286
SLC7A15	2.50E-17	0.2263591	0.212	0.118	6.02E-13	2.6	SLC7A1	1.796610169
RAPH15	2.51E-17	0.2762593	0.479	0.331	6.04E-13	2.6	RAPH1	1.447129909
NUP1602	3.12E-17	0.2242058	0.232	0.131	7.51E-13	2.6	NUP160	1.770992366
RAC12	4.10E-17	0.2477761	0.672	0.504	9.89E-13	2.6	RAC1	1.333333333
CD635	4.28E-17	0.219988	0.535	0.376	1.03E-12	2.6	CD63	1.42287234
TAPBP3	4.83E-17	0.2382809	0.242	0.14	1.17E-12	2.6	TAPBP	1.728571429
KIF1B4	5.00E-17	0.2536131	0.55	0.399	1.20E-12	2.6	KIF1B	1.378446115
IST13	5.08E-17	0.3060558	0.467	0.325	1.22E-12	2.6	IST1	1.436923077
RNF117	5.48E-17	0.2354609	0.375	0.242	1.32E-12	2.6	RNF11	1.549586777
RP11-1114A5.42	5.67E-17	0.1796334	0.151	0.075	1.37E-12	2.6	RP11-1114.	2.013333333
SMC41	6.38E-17	0.2065588	0.156	0.079	1.54E-12	2.6	SMC4	1.974683544
TFAP2A2	6.99E-17	0.1867722	0.259	0.151	1.69E-12	2.6	TFAP2A	1.715231788
YPEL23	7.04E-17	0.2662076	0.202	0.112	1.70E-12	2.6	YPEL2	1.803571429
TMEM63B	8.64E-17	0.1472186	0.071	0.026	2.08E-12	2.6	TMEM63B	2.730769231
CYFIP13	9.45E-17	0.2443284	0.231	0.135	2.28E-12	2.6	CYFIP1	1.711111111
DLGAP14	1.21E-16	0.1164912	0.323	0.199	2.92E-12	2.6	DLGAP1	1.623115578
ZHX23	1.24E-16	0.271866	0.477	0.335	2.98E-12	2.6	ZHX2	1.423880597
STK40	1.29E-16	0.1829488	0.23	0.13	3.10E-12	2.6	STK40	1.769230769
ERO1A3	1.35E-16	0.1164913	0.602	0.433	3.24E-12	2.6	ERO1A	1.390300231
WBP1L2	1.51E-16	0.2490405	0.158	0.082	3.63E-12	2.6	WBP1L	1.926829268
SMCO22	1.51E-16	0.1241855	0.049	0.015	3.64E-12	2.6	SMCO2	3.266666667
RBM14-RBM42	1.51E-16	0.1315792	0.116	0.052	3.64E-12	2.6	RBM14-RBI	2.230769231
TRAF33	1.51E-16	0.2396307	0.21	0.119	3.64E-12	2.6	TRAF3	1.764705882
FOXA15	1.54E-16	0.2338414	0.207	0.115	3.72E-12	2.6	FOXA1	1.8
TMEM106A2	1.56E-16	0.1917622	0.12	0.056	3.76E-12	2.6	TMEM106/	2.142857143
OLA13	1.61E-16	0.2538724	0.467	0.318	3.89E-12	2.6	OLA1	1.468553459

MIR34AHG2	2.04E-16	0.1820708	0.134	0.064	4.91E-12	2.6	MIR34AHG	2.09375
PLCD33	2.14E-16	0.1007078	0.048	0.014	5.17E-12	2.6	PLCD3	3.428571429
HSPG23	2.26E-16	0.2092661	0.154	0.079	5.46E-12	2.6	HSPG2	1.949367089
GS1-114I9.33	2.34E-16	0.1860847	0.321	0.199	5.65E-12	2.6	GS1-114I9.	1.613065327
ELF24	2.45E-16	0.2217929	0.54	0.383	5.91E-12	2.6	ELF2	1.409921671
DDX102	2.67E-16	0.279171	0.157	0.081	6.45E-12	2.6	DDX10	1.938271605
SLC39A65	3.03E-16	0.1136847	0.456	0.297	7.31E-12	2.6	SLC39A6	1.535353535
FOXO13	3.05E-16	0.3960803	0.294	0.189	7.35E-12	2.6	FOXO1	1.555555556
UMAD13	3.52E-16	0.312495	0.314	0.203	8.48E-12	2.6	UMAD1	1.54679803
DUSP62	3.91E-16	0.1725647	0.122	0.057	9.42E-12	2.6	DUSP6	2.140350877
BAG15	4.18E-16	0.2181575	0.179	0.096	1.01E-11	2.6	BAG1	1.864583333
FRYL3	4.21E-16	0.2187512	0.548	0.391	1.01E-11	2.6	FRYL	1.401534527
HDGF4	4.31E-16	0.2488279	0.391	0.262	1.04E-11	2.6	HDGF	1.492366412
BACH22	4.36E-16	0.2670765	0.474	0.331	1.05E-11	2.6	BACH2	1.432024169
FAM63B2	4.43E-16	0.2150065	0.305	0.189	1.07E-11	2.6	FAM63B	1.613756614
RP11-141M1.31	4.50E-16	0.2656279	0.174	0.093	1.08E-11	2.6	RP11-141M	1.870967742
MICALCL3	4.56E-16	0.185166	0.092	0.039	1.10E-11	2.6	MICALCL	2.358974359
SHC32	4.73E-16	0.1532904	0.041	0.011	1.14E-11	2.6	SHC3	3.727272727
ATF35	5.07E-16	0.1516195	0.566	0.4	1.22E-11	2.6	ATF3	1.415
PPM1K5	5.10E-16	0.2938947	0.245	0.147	1.23E-11	2.6	PPM1K	1.666666667
NTN13	6.34E-16	0.2452034	0.199	0.112	1.53E-11	2.6	NTN1	1.776785714
KIF16B4	6.52E-16	0.1861913	0.298	0.183	1.57E-11	2.6	KIF16B	1.628415301
TAGLN23	6.55E-16	0.1888711	0.337	0.215	1.58E-11	2.6	TAGLN2	1.56744186
PMAIP14	6.59E-16	0.1969485	0.216	0.123	1.59E-11	2.6	PMAIP1	1.756097561
GLI35	7.25E-16	0.2340182	0.229	0.133	1.75E-11	2.6	GLI3	1.721804511
TOX34	7.41E-16	0.2154188	0.277	0.167	1.79E-11	2.6	TOX3	1.658682635
CAPN84	7.47E-16	0.1549546	0.343	0.214	1.80E-11	2.6	CAPN8	1.602803738
MLLT63	7.48E-16	0.1677192	0.168	0.087	1.80E-11	2.6	MLLT6	1.931034483
FAM155A4	8.44E-16	0.3149119	0.119	0.056	2.04E-11	2.6	FAM155A	2.125
ZNF6383	9.14E-16	0.2212886	0.6	0.437	2.20E-11	2.6	ZNF638	1.372997712
S100A42	9.98E-16	0.1626138	0.249	0.148	2.41E-11	2.6	S100A4	1.682432432
GSPT15	1.08E-15	0.1934127	0.521	0.363	2.59E-11	2.6	GSPT1	1.435261708
NR2C23	1.16E-15	0.2204112	0.286	0.176	2.80E-11	2.6	NR2C2	1.625
RP11-371F15.34	1.17E-15	0.268367	0.158	0.084	2.82E-11	2.6	RP11-371F:	1.880952381
MTURN3	1.18E-15	0.2023183	0.264	0.158	2.85E-11	2.6	MTURN	1.670886076
MAN1A14	1.28E-15	0.232824	0.343	0.224	3.08E-11	2.6	MAN1A1	1.53125
LPP3	1.30E-15	0.1431476	0.972	0.913	3.14E-11	2.6	LPP	1.064622125
EGOT3	1.34E-15	0.1853988	0.202	0.113	3.23E-11	2.6	EGOT	1.787610619
PCMTD13	1.36E-15	0.217247	0.487	0.336	3.28E-11	2.6	PCMTD1	1.449404762
MEIS12	1.36E-15	0.2078936	0.137	0.068	3.29E-11	2.6	MEIS1	2.014705882
EIF13	1.41E-15	0.1015895	0.635	0.474	3.40E-11	2.6	EIF1	1.339662447
USP125	1.46E-15	0.2570406	0.296	0.186	3.51E-11	2.6	USP12	1.591397849
GPCPD12	1.53E-15	0.2748682	0.244	0.148	3.68E-11	2.6	GPCPD1	1.648648649
ARID4B2	1.59E-15	0.2034126	0.715	0.55	3.84E-11	2.6	ARID4B	1.3
USP244	1.59E-15	0.2004208	0.271	0.166	3.85E-11	2.6	USP24	1.63253012
TACC23	1.72E-15	0.1749939	0.267	0.159	4.15E-11	2.6	TACC2	1.679245283
USP312	1.93E-15	0.17215	0.2	0.112	4.64E-11	2.6	USP31	1.785714286
LEPR2	2.07E-15	0.2165701	0.178	0.097	5.00E-11	2.6	LEPR	1.835051546
PER22	2.12E-15	0.2104625	0.236	0.14	5.12E-11	2.6	PER2	1.685714286
INTS102	2.21E-15	0.2192364	0.221	0.129	5.34E-11	2.6	INTS10	1.713178295
GOLM14	2.31E-15	0.1277755	0.363	0.229	5.57E-11	2.6	GOLM1	1.585152838
UBE2B2	2.36E-15	0.2013432	0.398	0.264	5.69E-11	2.6	UBE2B	1.507575758

CCDC734	2.76E-15	0.2552052	0.187	0.104	6.66E-11	2.6	CCDC73	1.798076923
SHROOM34	2.94E-15	0.1454628	0.785	0.625	7.09E-11	2.6	SHROOM3	1.256
FAM102A2	3.05E-15	0.2139497	0.228	0.135	7.35E-11	2.6	FAM102A	1.688888889
STEAP42	3.53E-15	0.1198576	0.08	0.032	8.51E-11	2.6	STEAP4	2.5
SIRT14	3.68E-15	0.2204005	0.182	0.101	8.87E-11	2.6	SIRT1	1.801980198
DOCK14	3.74E-15	0.1798841	0.422	0.286	9.02E-11	2.6	DOCK1	1.475524476
RAB7A4	4.16E-15	0.238383	0.594	0.446	1.00E-10	2.6	RAB7A	1.331838565
ZNF4624	4.34E-15	0.2323145	0.439	0.303	1.05E-10	2.6	ZNF462	1.448844884
C1orf1322	4.54E-15	0.1739827	0.187	0.104	1.09E-10	2.6	C1orf132	1.798076923
ITFG14	4.75E-15	0.1945738	0.4	0.267	1.14E-10	2.6	ITFG1	1.498127341
METTL93	4.82E-15	0.1839285	0.184	0.101	1.16E-10	2.6	METTL9	1.821782178
MAP7D14	5.64E-15	0.2190739	0.247	0.149	1.36E-10	2.6	MAP7D1	1.657718121
WHSC13	6.47E-15	0.2031252	0.285	0.175	1.56E-10	2.6	WHSC1	1.628571429
NTN42	6.77E-15	0.1536075	0.438	0.295	1.63E-10	2.6	NTN4	1.484745763
ZNF242	8.47E-15	0.2090388	0.386	0.26	2.04E-10	2.6	ZNF24	1.484615385
ATF7IP4	8.47E-15	0.2450733	0.274	0.171	2.04E-10	2.6	ATF7IP	1.602339181
SOX92	8.63E-15	0.2353137	0.293	0.187	2.08E-10	2.6	SOX9	1.56684492
FHOD32	9.15E-15	0.1458378	0.071	0.028	2.21E-10	2.6	FHOD3	2.535714286
NRIP33	9.43E-15	0.1730429	0.096	0.042	2.27E-10	2.6	NRIP3	2.285714286
SRGAP2C3	1.02E-14	0.1888373	0.146	0.076	2.47E-10	2.6	SRGAP2C	1.921052632
ATP2B13	1.04E-14	0.2328133	0.166	0.091	2.51E-10	2.6	ATP2B1	1.824175824
TRPM73	1.19E-14	0.2323652	0.331	0.215	2.86E-10	2.6	TRPM7	1.539534884
NEDD96	1.20E-14	0.1436569	0.516	0.366	2.89E-10	2.6	NEDD9	1.409836066
TAP12	1.29E-14	0.1454038	0.092	0.041	3.11E-10	2.6	TAP1	2.243902439
CASC32	1.29E-14	0.2208053	0.244	0.148	3.11E-10	2.6	CASC3	1.648648649
CDK122	1.30E-14	0.2129846	0.2	0.115	3.13E-10	2.6	CDK12	1.739130435
CEP1923	1.46E-14	0.1899182	0.196	0.112	3.51E-10	2.6	CEP192	1.75
OGFRL13	1.59E-14	0.1618949	0.292	0.181	3.83E-10	2.6	OGFRL1	1.613259669
ARID22	1.75E-14	0.2272654	0.439	0.305	4.22E-10	2.6	ARID2	1.439344262
ANKRD102	1.80E-14	0.2306635	0.633	0.48	4.33E-10	2.6	ANKRD10	1.31875
HIST2H2BE3	1.84E-14	0.19479	0.19	0.108	4.43E-10	2.6	HIST2H2BE	1.759259259
ANKIB12	1.97E-14	0.2089934	0.493	0.354	4.74E-10	2.6	ANKIB1	1.392655367
FAM53C3	2.12E-14	0.2064566	0.332	0.218	5.10E-10	2.6	FAM53C	1.52293578
RP11-123O10.44	2.39E-14	0.2431212	0.319	0.208	5.76E-10	2.6	RP11-123O	1.533653846
TUFT14	2.40E-14	0.126801	0.288	0.179	5.78E-10	2.6	TUFT1	1.608938547
BTG31	2.43E-14	0.1960015	0.281	0.176	5.87E-10	2.6	BTG3	1.596590909
FOXK13	2.81E-14	0.1557744	0.289	0.179	6.78E-10	2.6	FOXK1	1.61452514
EPG52	2.96E-14	0.182789	0.186	0.105	7.14E-10	2.6	EPG5	1.771428571
PIM33	3.21E-14	0.1776116	0.136	0.07	7.75E-10	2.6	PIM3	1.942857143
SLC22A54	3.45E-14	0.1844807	0.1	0.046	8.32E-10	2.6	SLC22A5	2.173913043
SKIL2	4.13E-14	0.1739926	0.182	0.102	9.97E-10	2.6	SKIL	1.784313725
RGS122	4.36E-14	0.1553911	0.127	0.064	1.05E-09	2.6	RGS12	1.984375
ARNTL1	5.13E-14	0.1618951	0.121	0.06	1.24E-09	2.6	ARNTL	2.016666667
OSMR-AS13	5.85E-14	0.1947701	0.268	0.168	1.41E-09	2.6	OSMR-AS1	1.595238095
MT-ND23	6.35E-14	0.1487341	0.994	0.976	1.53E-09	2.6	MT-ND2	1.018442623
AC017101.104	6.37E-14	0.1503765	0.165	0.09	1.54E-09	2.6	AC017101.	1.833333333
USP9X4	6.98E-14	0.2198524	0.545	0.401	1.68E-09	2.6	USP9X	1.359102244
MBTPS13	7.06E-14	0.2228128	0.191	0.111	1.70E-09	2.6	MBTPS1	1.720720721
ARHGAP323	7.41E-14	0.2144739	0.558	0.41	1.79E-09	2.6	ARHGAP32	1.36097561
MOCOS2	7.71E-14	0.2168931	0.184	0.105	1.86E-09	2.6	MOCOS	1.752380952
CST35	7.77E-14	0.1818193	0.127	0.065	1.87E-09	2.6	CST3	1.953846154
MPZL33	8.48E-14	0.2251139	0.441	0.31	2.04E-09	2.6	MPZL3	1.422580645

ERN13	8.70E-14	0.1994048	0.251	0.155	2.10E-09	2.6	ERN1	1.619354839
GRIP13	9.08E-14	0.2258668	0.288	0.186	2.19E-09	2.6	GRIP1	1.548387097
CDC42BPA3	9.19E-14	0.1731481	0.525	0.377	2.21E-09	2.6	CDC42BPA	1.392572944
PLIN33	9.63E-14	0.178345	0.251	0.155	2.32E-09	2.6	PLIN3	1.619354839
TSPAN110	1.01E-13	0.1126416	0.343	0.223	2.45E-09	2.6	TSPAN1	1.538116592
CLASP23	1.36E-13	0.206291	0.391	0.269	3.29E-09	2.6	CLASP2	1.453531599
APPL22	1.37E-13	0.1760144	0.187	0.107	3.31E-09	2.6	APPL2	1.747663551
ITPR23	1.42E-13	0.1010358	0.627	0.468	3.42E-09	2.6	ITPR2	1.33974359
S100A104	1.46E-13	0.1180712	0.523	0.381	3.52E-09	2.6	S100A10	1.372703412
RBBP63	1.54E-13	0.1727161	0.407	0.277	3.71E-09	2.6	RBBP6	1.469314079
MYRFL2	1.55E-13	0.2047544	0.136	0.071	3.75E-09	2.6	MYRFL	1.915492958
NUTM2B-AS12	1.65E-13	0.2002103	0.448	0.317	3.97E-09	2.6	NUTM2B-A	1.413249211
CDKN1A3	1.69E-13	0.15793	0.125	0.063	4.08E-09	2.6	CDKN1A	1.984126984
LIF3	1.72E-13	0.130706	0.053	0.019	4.15E-09	2.6	LIF	2.789473684
HINT32	1.75E-13	0.1555325	0.123	0.063	4.23E-09	2.6	HINT3	1.952380952
ADORA2A-AS12	1.80E-13	0.1159381	0.048	0.016	4.34E-09	2.6	ADORA2A-/	3
KLHL182	1.85E-13	0.1594911	0.146	0.078	4.47E-09	2.6	KLHL18	1.871794872
SIPA1L14	1.86E-13	0.1836976	0.523	0.379	4.48E-09	2.6	SIPA1L1	1.37994723
BPTF4	1.86E-13	0.193771	0.466	0.331	4.48E-09	2.6	BPTF	1.407854985
KAT6B4	1.87E-13	0.2305355	0.37	0.253	4.50E-09	2.6	KAT6B	1.462450593
AHI12	1.92E-13	0.1875587	0.38	0.258	4.63E-09	2.6	AHI1	1.472868217
SLC17A83	1.95E-13	0.1360346	0.049	0.017	4.69E-09	2.6	SLC17A8	2.882352941
LRP102	1.97E-13	0.1618727	0.222	0.133	4.75E-09	2.6	LRP10	1.669172932
VEGFC2	1.98E-13	0.1291312	0.114	0.056	4.77E-09	2.6	VEGFC	2.035714286
SMURF13	1.98E-13	0.1515406	0.509	0.361	4.77E-09	2.6	SMURF1	1.409972299
HIVEP14	2.07E-13	0.2060676	0.293	0.189	4.98E-09	2.6	HIVEP1	1.55026455
TLR23	2.32E-13	0.1879908	0.264	0.166	5.60E-09	2.6	TLR2	1.590361446
ZCCHC72	2.47E-13	0.1874327	0.567	0.418	5.95E-09	2.6	ZCCHC7	1.35645933
ATP9A2	2.47E-13	0.1736126	0.181	0.104	5.96E-09	2.6	ATP9A	1.740384615
SMAD24	2.54E-13	0.2158138	0.424	0.299	6.13E-09	2.6	SMAD2	1.418060201
XIAP2	2.54E-13	0.1920065	0.221	0.134	6.13E-09	2.6	XIAP	1.649253731
COL28A12	2.59E-13	0.1510071	0.079	0.034	6.24E-09	2.6	COL28A1	2.323529412
ZDHHC143	2.81E-13	0.2356937	0.197	0.117	6.77E-09	2.6	ZDHHC14	1.683760684
MAATS11	2.83E-13	0.1191936	0.046	0.015	6.81E-09	2.6	MAATS1	3.066666667
DDX62	2.91E-13	0.20036	0.382	0.261	7.02E-09	2.6	DDX6	1.463601533
TCF7L23	2.93E-13	0.1966744	0.522	0.377	7.06E-09	2.6	TCF7L2	1.384615385
TRIM293	3.12E-13	0.1604007	0.11	0.054	7.52E-09	2.6	TRIM29	2.037037037
FRY1	3.35E-13	0.2460064	0.175	0.1	8.07E-09	2.6	FRY	1.75
KLF103	3.57E-13	0.2024032	0.228	0.14	8.62E-09	2.6	KLF10	1.628571429
NFKBIB2	3.73E-13	0.1601439	0.081	0.036	9.00E-09	2.6	NFKBIB	2.25
WDR203	3.95E-13	0.1536281	0.191	0.11	9.52E-09	2.6	WDR20	1.736363636
SESN13	4.34E-13	0.2543226	0.197	0.118	1.05E-08	2.6	SESN1	1.669491525
ZC3H7B1	4.35E-13	0.1562046	0.117	0.059	1.05E-08	2.6	ZC3H7B	1.983050847
SMAD52	4.42E-13	0.1731183	0.201	0.118	1.07E-08	2.6	SMAD5	1.703389831
BCKDHB2	4.50E-13	0.1782815	0.198	0.116	1.08E-08	2.6	BCKDHB	1.706896552
NR4A33	4.68E-13	0.1421905	0.053	0.019	1.13E-08	2.6	NR4A3	2.789473684
AMOTL21	4.74E-13	0.161553	0.126	0.065	1.14E-08	2.6	AMOTL2	1.938461538
MTCL14	4.79E-13	0.113879	0.109	0.053	1.16E-08	2.6	MTCL1	2.056603774
TYMP3	5.00E-13	0.1255848	0.078	0.034	1.21E-08	2.6	TYMP	2.294117647
APLP25	5.27E-13	0.1543318	0.402	0.273	1.27E-08	2.6	APLP2	1.472527473
ZC3H12A2	5.50E-13	0.1622321	0.127	0.066	1.33E-08	2.6	ZC3H12A	1.924242424
ATP8B13	5.70E-13	0.1863881	0.533	0.4	1.38E-08	2.6	ATP8B1	1.3325

RP11-350J20.52	5.75E-13	0.1199728	0.068	0.027	1.39E-08	2.6	RP11-350J2	2.518518519
TMBIM13	6.04E-13	0.1642569	0.22	0.133	1.46E-08	2.6	TMBIM1	1.654135338
PPP2R3C2	6.10E-13	0.1791953	0.183	0.106	1.47E-08	2.6	PPP2R3C	1.726415094
CDSN2	6.33E-13	0.1092552	0.055	0.021	1.53E-08	2.6	CDSN	2.619047619
NDUFAF63	6.43E-13	0.2224638	0.175	0.1	1.55E-08	2.6	NDUFAF6	1.75
DHX322	7.03E-13	0.175697	0.42	0.293	1.70E-08	2.6	DHX32	1.433447099
TBL1X2	7.96E-13	0.180904	0.323	0.213	1.92E-08	2.6	TBL1X	1.516431925
ZFAND55	8.16E-13	0.2217876	0.564	0.43	1.97E-08	2.6	ZFAND5	1.311627907
EP3004	8.79E-13	0.1684476	0.328	0.216	2.12E-08	2.6	EP300	1.518518519
AGAP33	8.98E-13	0.1373692	0.11	0.054	2.17E-08	2.6	AGAP3	2.037037037
CKAP42	9.29E-13	0.1696205	0.308	0.203	2.24E-08	2.6	CKAP4	1.517241379
ZSWIM64	9.39E-13	0.1294331	0.652	0.492	2.26E-08	2.6	ZSWIM6	1.325203252
ANKRD174	9.46E-13	0.1987463	0.657	0.503	2.28E-08	2.6	ANKRD17	1.306163022
CGN2	1.01E-12	0.1857486	0.155	0.087	2.44E-08	2.6	CGN	1.781609195
SCNN1A2	1.05E-12	0.1878807	0.197	0.118	2.54E-08	2.6	SCNN1A	1.669491525
STAG13	1.13E-12	0.1673509	0.659	0.506	2.72E-08	2.6	STAG1	1.302371542
PCF113	1.13E-12	0.1851277	0.202	0.122	2.74E-08	2.6	PCF11	1.655737705
TP53INP22	1.16E-12	0.1434316	0.097	0.046	2.79E-08	2.6	TP53INP2	2.108695652
FILIP1L4	1.18E-12	0.167818	0.189	0.11	2.85E-08	2.6	FILIP1L	1.718181818
C2CD4A5	1.24E-12	0.1890101	0.098	0.047	2.99E-08	2.6	C2CD4A	2.085106383
ZBTB444	1.26E-12	0.1956107	0.227	0.139	3.05E-08	2.6	ZBTB44	1.633093525
MPRIP3	1.29E-12	0.1795041	0.375	0.257	3.10E-08	2.6	MPRIP	1.459143969
CFAP694	1.36E-12	0.1368152	0.18	0.103	3.29E-08	2.6	CFAP69	1.747572816
SSBP24	1.38E-12	0.1682176	0.585	0.431	3.33E-08	2.6	SSBP2	1.357308585
HNRNPM4	1.42E-12	0.1723043	0.378	0.258	3.44E-08	2.6	HNRNPM	1.465116279
UNK3	1.54E-12	0.1175448	0.105	0.051	3.70E-08	2.6	UNK	2.058823529
LAMP15	1.60E-12	0.1749451	0.166	0.095	3.85E-08	2.6	LAMP1	1.747368421
PRKCH2	1.60E-12	0.1644013	0.346	0.236	3.86E-08	2.6	PRKCH	1.466101695
NDFIP12	1.89E-12	0.2108898	0.29	0.191	4.55E-08	2.6	NDFIP1	1.518324607
LIMD12	1.99E-12	0.1250452	0.136	0.072	4.80E-08	2.6	LIMD1	1.888888889
WNK14	2.07E-12	0.1710166	0.35	0.238	4.99E-08	2.6	WNK1	1.470588235
ITPKC2	2.12E-12	0.1581337	0.191	0.114	5.11E-08	2.6	ITPKC	1.675438596
CCDC1481	2.15E-12	0.1477558	0.098	0.047	5.19E-08	2.6	CCDC148	2.085106383
ZFP36L12	2.42E-12	0.1623221	0.735	0.587	5.84E-08	2.6	ZFP36L1	1.252129472
DOCK92	2.69E-12	0.1692718	0.324	0.217	6.48E-08	2.6	DOCK9	1.493087558
BRE3	2.72E-12	0.2341055	0.303	0.205	6.56E-08	2.6	BRE	1.47804878
PPP3CA5	2.90E-12	0.1621862	0.711	0.558	6.99E-08	2.6	PPP3CA	1.274193548
PDZD85	2.90E-12	0.1521352	0.34	0.228	7.00E-08	2.6	PDZD8	1.49122807
RP11-834C11.41	2.94E-12	0.114539	0.077	0.034	7.09E-08	2.6	RP11-834C	2.264705882
SRPK13	3.17E-12	0.1603844	0.624	0.481	7.65E-08	2.6	SRPK1	1.297297297
PEA152	3.55E-12	0.2224769	0.29	0.193	8.56E-08	2.6	PEA15	1.502590674
DLG14	3.57E-12	0.2054156	0.632	0.501	8.61E-08	2.6	DLG1	1.261477046
KIAA03682	3.62E-12	0.2039232	0.353	0.241	8.74E-08	2.6	KIAA0368	1.46473029
RAD182	3.72E-12	0.191411	0.137	0.075	8.96E-08	2.6	RAD18	1.826666667
PSEN12	4.14E-12	0.1745652	0.341	0.229	9.99E-08	2.6	PSEN1	1.489082969
HMGCS12	4.18E-12	0.1626749	0.351	0.236	1.01E-07	2.6	HMGCS1	1.487288136
SRPX22	4.56E-12	0.1352886	0.134	0.072	1.10E-07	2.6	SRPX2	1.861111111
PSPC13	4.81E-12	0.1597612	0.29	0.188	1.16E-07	2.6	PSPC1	1.542553191
MAP2K13	4.83E-12	0.1593592	0.365	0.249	1.16E-07	2.6	MAP2K1	1.465863454
DMXL13	5.51E-12	0.152572	0.257	0.164	1.33E-07	2.6	DMXL1	1.567073171
AC026188.11	5.53E-12	0.2211052	0.068	0.029	1.33E-07	2.6	AC026188.	2.344827586
GM2A2	5.76E-12	0.1585809	0.082	0.038	1.39E-07	2.6	GM2A	2.157894737

CUL12	5.78E-12	0.1949509	0.197	0.12	1.39E-07	2.6	CUL1	1.641666667
SUSD13	5.79E-12	0.1906405	0.172	0.1	1.40E-07	2.6	SUSD1	1.72
SLC37A12	5.81E-12	0.1632304	0.148	0.083	1.40E-07	2.6	SLC37A1	1.78313253
UBL55	5.88E-12	0.1675836	0.645	0.504	1.42E-07	2.6	UBL5	1.279761905
KDM2A3	5.92E-12	0.1720506	0.577	0.431	1.43E-07	2.6	KDM2A	1.3387471
GOLPH33	6.06E-12	0.1529484	0.379	0.259	1.46E-07	2.6	GOLPH3	1.463320463
DLG54	6.07E-12	0.1746074	0.371	0.259	1.46E-07	2.6	DLG5	1.432432432
QSER12	6.80E-12	0.1515593	0.245	0.154	1.64E-07	2.6	QSER1	1.590909091
MAPK64	6.88E-12	0.1641184	0.387	0.269	1.66E-07	2.6	MAPK6	1.43866171
OTUD7B4	7.06E-12	0.1209876	0.42	0.29	1.70E-07	2.6	OTUD7B	1.448275862
ZNF2642	7.11E-12	0.123604	0.137	0.074	1.71E-07	2.6	ZNF264	1.851351351
SGPP23	7.51E-12	0.1953211	0.181	0.108	1.81E-07	2.6	SGPP2	1.675925926
FCHO22	7.79E-12	0.1806505	0.264	0.171	1.88E-07	2.6	FCHO2	1.543859649
MOCS12	8.17E-12	0.10224	0.052	0.02	1.97E-07	2.6	MOCS1	2.6
RP11-83A24.25	8.63E-12	0.1649837	0.211	0.129	2.08E-07	2.6	RP11-83A2	1.635658915
APLF3	9.28E-12	0.1766163	0.177	0.104	2.24E-07	2.6	APLF	1.701923077
AP1S33	9.35E-12	0.1418173	0.199	0.12	2.25E-07	2.6	AP1S3	1.658333333
N4BP2L12	1.05E-11	0.1725459	0.181	0.107	2.53E-07	2.6	N4BP2L1	1.691588785
PTP4A13	1.07E-11	0.1918552	0.394	0.281	2.59E-07	2.6	PTP4A1	1.402135231
KMT2E4	1.09E-11	0.1564413	0.593	0.461	2.63E-07	2.6	KMT2E	1.286334056
ZMAT31	1.09E-11	0.1668296	0.107	0.055	2.64E-07	2.6	ZMAT3	1.945454545
ST71	1.12E-11	0.1774411	0.233	0.147	2.70E-07	2.6	ST7	1.585034014
AK33	1.23E-11	0.159117	0.306	0.204	2.96E-07	2.6	AK3	1.5
ZNF4402	1.26E-11	0.120897	0.097	0.047	3.05E-07	2.6	ZNF440	2.063829787
VAMP83	1.27E-11	0.1511297	0.856	0.756	3.06E-07	2.6	VAMP8	1.132275132
EPB41L4A2	1.33E-11	0.1415305	0.25	0.158	3.20E-07	2.6	EPB41L4A	1.582278481
RASD12	1.43E-11	0.1177528	0.108	0.055	3.45E-07	2.6	RASD1	1.963636364
KLHL424	1.49E-11	0.1533876	0.131	0.071	3.60E-07	2.6	KLHL42	1.845070423
RHBDD13	1.49E-11	0.208535	0.217	0.137	3.60E-07	2.6	RHBDD1	1.583941606
UBE2Z1	1.61E-11	0.16948	0.306	0.206	3.88E-07	2.6	UBE2Z	1.485436893
NBPF122	1.71E-11	0.162596	0.15	0.086	4.13E-07	2.6	NBPF12	1.744186047
AVL94	1.77E-11	0.1772876	0.295	0.198	4.27E-07	2.6	AVL9	1.48989899
TMTC24	1.77E-11	0.1882927	0.504	0.373	4.27E-07	2.6	TMTC2	1.351206434
PRKAR2A4	1.83E-11	0.2334443	0.253	0.164	4.41E-07	2.6	PRKAR2A	1.542682927
ARHGAP53	1.88E-11	0.1663036	0.53	0.395	4.52E-07	2.6	ARHGAP5	1.341772152
UHRF1BP1L2	1.95E-11	0.1691957	0.301	0.202	4.70E-07	2.6	UHRF1BP1	1.49009901
RHEB3	1.97E-11	0.1172568	0.376	0.259	4.74E-07	2.6	RHEB	1.451737452
FAM222A-AS13	1.98E-11	0.1083989	0.069	0.03	4.78E-07	2.6	FAM222A-/	2.3
TLE31	2.09E-11	0.129051	0.072	0.032	5.03E-07	2.6	TLE3	2.25
YME1L13	2.13E-11	0.2073762	0.376	0.266	5.12E-07	2.6	YME1L1	1.413533835
PIAS13	2.14E-11	0.1721287	0.627	0.48	5.16E-07	2.6	PIAS1	1.30625
GRIN2A2	2.15E-11	0.1349843	0.102	0.051	5.18E-07	2.6	GRIN2A	2
BZW13	2.15E-11	0.157972	0.392	0.273	5.19E-07	2.6	BZW1	1.435897436
RFK3	2.17E-11	0.1355455	0.092	0.045	5.24E-07	2.6	RFK	2.044444444
WDR193	2.20E-11	0.1604876	0.171	0.101	5.30E-07	2.6	WDR19	1.693069307
PINK13	2.34E-11	0.1277776	0.114	0.06	5.63E-07	2.6	PINK1	1.9
CD243	2.41E-11	0.1148379	0.708	0.575	5.81E-07	2.6	CD24	1.231304348
MED134	2.47E-11	0.1437587	0.528	0.394	5.96E-07	2.6	MED13	1.340101523
PGAP12	2.52E-11	0.1675094	0.139	0.078	6.07E-07	2.6	PGAP1	1.782051282
IFT571	2.62E-11	0.233155	0.217	0.138	6.33E-07	2.6	IFT57	1.572463768
HNRNPUL11	2.84E-11	0.1932835	0.194	0.119	6.85E-07	2.6	HNRNPUL1	1.630252101
APOL63	2.91E-11	0.2082835	0.188	0.116	7.02E-07	2.6	APOL6	1.620689655

AVIL4	3.11E-11	0.1694942	0.113	0.06	7.49E-07	2.6	AVIL	1.883333333
INTS64	3.28E-11	0.1297903	0.396	0.278	7.91E-07	2.6	INTS6	1.424460432
RP11-382A20.34	3.36E-11	0.1577998	0.188	0.113	8.11E-07	2.6	RP11-382A	1.663716814
HLA-C2	3.36E-11	0.1567077	0.43	0.309	8.11E-07	2.6	HLA-C	1.391585761
EXOC23	3.38E-11	0.1586988	0.218	0.137	8.16E-07	2.6	EXOC2	1.591240876
PPP1R212	3.51E-11	0.1385124	0.19	0.115	8.45E-07	2.6	PPP1R21	1.652173913
PPP6R33	3.78E-11	0.1550191	0.639	0.503	9.11E-07	2.6	PPP6R3	1.270377734
ANXA62	3.97E-11	0.1478294	0.169	0.099	9.56E-07	2.6	ANXA6	1.707070707
GBF11	4.16E-11	0.1591091	0.325	0.222	1.00E-06	2.6	GBF1	1.463963964
SBNO13	4.30E-11	0.1587139	0.233	0.149	1.04E-06	2.6	SBNO1	1.563758389
MICU22	4.95E-11	0.1866764	0.255	0.168	1.19E-06	2.6	MICU2	1.517857143
CCSER12	4.98E-11	0.1858364	0.563	0.437	1.20E-06	2.6	CCSER1	1.288329519
PMEPA13	5.11E-11	0.142083	0.112	0.059	1.23E-06	2.6	PMEPA1	1.898305085
CTSB3	5.17E-11	0.1122481	0.403	0.287	1.25E-06	2.6	CTSB	1.404181185
MIR181A2HG4	5.25E-11	0.1556521	0.147	0.084	1.27E-06	2.6	MIR181A2I	1.75
BCL2L17	5.42E-11	0.1510259	0.246	0.159	1.31E-06	2.6	BCL2L1	1.547169811
OST44	5.51E-11	0.1803117	0.548	0.41	1.33E-06	2.6	OST4	1.336585366
TMEM2172	5.88E-11	0.1652667	0.116	0.062	1.42E-06	2.6	TMEM217	1.870967742
KDM6B	5.97E-11	0.1176407	0.054	0.022	1.44E-06	2.6	KDM6B	2.454545455
PPARA4	5.98E-11	0.1586389	0.141	0.08	1.44E-06	2.6	PPARA	1.7625
ATP5E5	6.04E-11	0.1063338	0.7	0.55	1.46E-06	2.6	ATP5E	1.272727273
ZNF6752	6.21E-11	0.1768392	0.172	0.103	1.50E-06	2.6	ZNF675	1.669902913
RP11-65M17.32	6.29E-11	0.1136495	0.054	0.022	1.52E-06	2.6	RP11-65M1	2.454545455
SCAF83	6.43E-11	0.1326969	0.344	0.238	1.55E-06	2.6	SCAF8	1.445378151
FBXL72	6.64E-11	0.1107214	0.077	0.036	1.60E-06	2.6	FBXL7	2.138888889
DNAJC113	6.88E-11	0.1808217	0.366	0.259	1.66E-06	2.6	DNAJC1	1.413127413
SLC9A72	7.06E-11	0.1446728	0.395	0.278	1.70E-06	2.6	SLC9A7	1.420863309
SLC44A51	7.27E-11	0.1089072	0.057	0.023	1.75E-06	2.6	SLC44A5	2.47826087
TMEM106B2	7.45E-11	0.1591011	0.234	0.149	1.80E-06	2.6	TMEM106I	1.570469799
LMNA3	7.57E-11	0.154938	0.33	0.229	1.83E-06	2.6	LMNA	1.441048035
RP11-624L4.13	7.97E-11	0.1267217	0.116	0.062	1.92E-06	2.6	RP11-624L	1.870967742
GJB33	8.39E-11	0.1152069	0.071	0.032	2.02E-06	2.6	GJB3	2.21875
PHC22	8.69E-11	0.1595573	0.179	0.109	2.10E-06	2.6	PHC2	1.642201835
RANGAP12	8.86E-11	0.1226328	0.101	0.052	2.14E-06	2.6	RANGAP1	1.942307692
MIB14	9.15E-11	0.1862137	0.511	0.379	2.21E-06	2.6	MIB1	1.34828496
LNX24	9.45E-11	0.1108599	0.229	0.145	2.28E-06	2.6	LNX2	1.579310345
USP323	9.48E-11	0.1563441	0.331	0.228	2.29E-06	2.6	USP32	1.451754386
DYNLRB14	9.51E-11	0.1517711	0.433	0.308	2.29E-06	2.6	DYNLRB1	1.405844156
SLC9A13	9.78E-11	0.1336194	0.195	0.12	2.36E-06	2.6	SLC9A1	1.625
EHD23	9.87E-11	0.1084915	0.108	0.056	2.38E-06	2.6	EHD2	1.928571429
CUL34	1.01E-10	0.1618806	0.526	0.399	2.43E-06	2.6	CUL3	1.318295739
NRF12	1.03E-10	0.1442429	0.259	0.168	2.48E-06	2.6	NRF1	1.541666667
ANO103	1.03E-10	0.2171605	0.378	0.27	2.49E-06	2.6	ANO10	1.4
IBTK4	1.06E-10	0.1363418	0.321	0.218	2.54E-06	2.6	IBTK	1.472477064
FOSB2	1.09E-10	0.1177483	0.525	0.39	2.62E-06	2.6	FOSB	1.346153846
CTD-2060L22.12	1.09E-10	0.154467	0.052	0.021	2.62E-06	2.6	CTD-2060L	2.476190476
SEMA4B1	1.18E-10	0.1006529	0.326	0.22	2.84E-06	2.6	SEMA4B	1.481818182
JUND5	1.18E-10	0.1504032	0.259	0.171	2.85E-06	2.6	JUND	1.514619883
PHIP3	1.31E-10	0.2020777	0.565	0.436	3.17E-06	2.6	PHIP	1.29587156
NPLOC42	1.33E-10	0.1307507	0.234	0.151	3.20E-06	2.6	NPLOC4	1.549668874
DIAPH13	1.34E-10	0.1256999	0.414	0.293	3.23E-06	2.6	DIAPH1	1.412969283
TMEM595	1.34E-10	0.1886903	0.579	0.448	3.23E-06	2.6	TMEM59	1.292410714

C3orf351	1.34E-10	0.1182283	0.11	0.058	3.24E-06	2.6	C3orf35	1.896551724
TNRC6C2	1.39E-10	0.1316505	0.084	0.041	3.36E-06	2.6	TNRC6C	2.048780488
SBF23	1.43E-10	0.168219	0.756	0.633	3.45E-06	2.6	SBF2	1.194312796
SLC35F2	1.49E-10	0.1732151	0.104	0.054	3.59E-06	2.6	SLC35F2	1.925925926
VPS13A2	1.54E-10	0.1833124	0.231	0.151	3.71E-06	2.6	VPS13A	1.529801325
ADAM103	1.68E-10	0.1181192	0.51	0.369	4.06E-06	2.6	ADAM10	1.382113821
ERO1B4	1.69E-10	0.1256928	0.162	0.096	4.07E-06	2.6	ERO1B	1.6875
TTC174	1.84E-10	0.1784379	0.405	0.292	4.43E-06	2.6	TTC17	1.386986301
CLEC16A2	2.02E-10	0.1500168	0.237	0.155	4.87E-06	2.6	CLEC16A	1.529032258
SCCPDH2	2.10E-10	0.1264733	0.16	0.095	5.06E-06	2.6	SCCPDH	1.684210526
RPAP23	2.22E-10	0.197201	0.288	0.197	5.36E-06	2.6	RPAP2	1.461928934
SBDS1	2.24E-10	0.1068891	0.155	0.091	5.40E-06	2.6	SBDS	1.703296703
TGM23	2.29E-10	0.1409788	0.16	0.095	5.51E-06	2.6	TGM2	1.684210526
OGT3	2.36E-10	0.1695669	0.527	0.398	5.70E-06	2.6	OGT	1.324120603
TJP24	2.37E-10	0.1250779	0.44	0.316	5.71E-06	2.6	TJP2	1.392405063
GUSB5	2.39E-10	0.1204145	0.089	0.045	5.76E-06	2.6	GUSB	1.977777778
FAM208A4	2.40E-10	0.1612575	0.144	0.084	5.79E-06	2.6	FAM208A	1.714285714
RUFY13	2.45E-10	0.1636369	0.17	0.103	5.91E-06	2.6	RUFY1	1.650485437
LAMC12	2.55E-10	0.1228077	0.365	0.257	6.15E-06	2.6	LAMC1	1.420233463
HNF4G2	2.57E-10	0.1181789	0.053	0.022	6.21E-06	2.6	HNF4G	2.409090909
SLC16A44	2.66E-10	0.1094777	0.162	0.096	6.40E-06	2.6	SLC16A4	1.6875
MAP2K31	2.69E-10	0.104008	0.11	0.059	6.48E-06	2.6	MAP2K3	1.86440678
CASP74	2.71E-10	0.1567962	0.239	0.158	6.54E-06	2.6	CASP7	1.512658228
MAL24	2.76E-10	0.1008195	0.481	0.349	6.66E-06	2.6	MAL2	1.378223496
PPP1R15B5	2.83E-10	0.1281556	0.126	0.07	6.83E-06	2.6	PPP1R15B	1.8
IL1RN4	2.83E-10	0.1385724	0.097	0.05	6.84E-06	2.6	IL1RN	1.94
LINC010364	2.88E-10	0.1723319	0.155	0.093	6.94E-06	2.6	LINC01036	1.666666667
GNB14	2.92E-10	0.1195363	0.467	0.34	7.04E-06	2.6	GNB1	1.373529412
PARM13	2.97E-10	0.1176715	0.124	0.069	7.16E-06	2.6	PARM1	1.797101449
YY1AP13	3.07E-10	0.1385808	0.255	0.169	7.40E-06	2.6	YY1AP1	1.50887574
NFIL34	3.25E-10	0.1401213	0.277	0.185	7.84E-06	2.6	NFIL3	1.497297297
TET32	3.28E-10	0.1598701	0.2	0.127	7.92E-06	2.6	TET3	1.57480315
ZNF2673	3.35E-10	0.114477	0.245	0.159	8.08E-06	2.6	ZNF267	1.540880503
ARID1B4	3.45E-10	0.135028	0.632	0.489	8.31E-06	2.6	ARID1B	1.292433538
PPP2R2D3	3.46E-10	0.1268782	0.266	0.177	8.35E-06	2.6	PPP2R2D	1.502824859
MAK2	3.65E-10	0.138843	0.151	0.089	8.80E-06	2.6	MAK	1.696629213
PEBP14	3.70E-10	0.113556	0.26	0.17	8.92E-06	2.6	PEBP1	1.529411765
ING13	3.84E-10	0.1038455	0.065	0.029	9.25E-06	2.6	ING1	2.24137931
ERBB32	3.85E-10	0.1196061	0.207	0.13	9.28E-06	2.6	ERBB3	1.592307692
DNAAF21	3.92E-10	0.1307639	0.059	0.025	9.45E-06	2.6	DNAAF2	2.36
POGZ3	4.00E-10	0.1821953	0.368	0.262	9.64E-06	2.6	POGZ	1.404580153
RP11-212D19.43	4.08E-10	0.1077882	0.073	0.034	9.84E-06	2.6	RP11-212D	2.147058824
CTNNA12	4.21E-10	0.1312035	0.675	0.533	1.01E-05	2.6	CTNNA1	1.26641651
NPFFR24	4.21E-10	0.1308003	0.058	0.025	1.02E-05	2.6	NPFFR2	2.32
CKS25	4.63E-10	0.1396756	0.31	0.215	1.12E-05	2.6	CKS2	1.441860465
CPD3	4.66E-10	0.1969906	0.333	0.237	1.12E-05	2.6	CPD	1.405063291
SUN13	4.73E-10	0.1320854	0.205	0.129	1.14E-05	2.6	SUN1	1.589147287
SFMBT11	4.86E-10	0.1348878	0.108	0.058	1.17E-05	2.6	SFMBT1	1.862068966
BRD23	5.08E-10	0.1492512	0.369	0.266	1.22E-05	2.6	BRD2	1.387218045
GRB104	5.20E-10	0.1486537	0.266	0.179	1.25E-05	2.6	GRB10	1.48603352
SULF23	5.24E-10	0.1107692	0.175	0.107	1.26E-05	2.6	SULF2	1.635514019
RNF1283	5.39E-10	0.1784693	0.196	0.125	1.30E-05	2.6	RNF128	1.568

MXD13	5.41E-10	0.1442034	0.302	0.209	1.30E-05	2.6	MXD1	1.444976077
NR3C13	5.46E-10	0.1340319	0.333	0.231	1.32E-05	2.6	NR3C1	1.441558442
HERPUD22	5.55E-10	0.1352496	0.116	0.064	1.34E-05	2.6	HERPUD2	1.8125
CMTM42	5.60E-10	0.13992	0.227	0.148	1.35E-05	2.6	CMTM4	1.533783784
HS3ST3B12	5.70E-10	0.1299293	0.06	0.026	1.37E-05	2.6	HS3ST3B1	2.307692308
FAM110C3	5.75E-10	0.1089067	0.081	0.04	1.39E-05	2.6	FAM110C	2.025
PARK22	5.91E-10	0.1939105	0.263	0.179	1.42E-05	2.6	PARK2	1.469273743
SRGAP2B2	6.07E-10	0.1498139	0.141	0.083	1.46E-05	2.6	SRGAP2B	1.698795181
KIAA02322	6.15E-10	0.119887	0.286	0.193	1.48E-05	2.6	KIAA0232	1.481865285
ZMIZ13	6.17E-10	0.1002946	0.158	0.094	1.49E-05	2.6	ZMIZ1	1.680851064
SDHA2	6.19E-10	0.2047567	0.148	0.089	1.49E-05	2.6	SDHA	1.662921348
COG72	6.40E-10	0.1428548	0.145	0.085	1.54E-05	2.6	COG7	1.705882353
PRPF32	6.43E-10	0.1409788	0.202	0.129	1.55E-05	2.6	PRPF3	1.565891473
AKAP133	6.62E-10	0.1010536	0.745	0.606	1.60E-05	2.6	AKAP13	1.229372937
TNS33	6.67E-10	0.1284916	0.121	0.069	1.61E-05	2.6	TNS3	1.753623188
SUZ122	6.72E-10	0.1137419	0.27	0.18	1.62E-05	2.6	SUZ12	1.5
IQSEC11	6.74E-10	0.1135954	0.044	0.017	1.63E-05	2.6	IQSEC1	2.588235294
RAD23B3	6.77E-10	0.1746167	0.606	0.48	1.63E-05	2.6	RAD23B	1.2625
PGM22	7.24E-10	0.1295772	0.133	0.076	1.75E-05	2.6	PGM2	1.75
ELP23	7.27E-10	0.1185506	0.311	0.212	1.75E-05	2.6	ELP2	1.466981132
PREX15	7.55E-10	0.1046936	0.1	0.052	1.82E-05	2.6	PREX1	1.923076923
NSUN21	7.81E-10	0.1032982	0.071	0.033	1.88E-05	2.6	NSUN2	2.151515152
KLF94	7.84E-10	0.1528236	0.229	0.151	1.89E-05	2.6	KLF9	1.516556291
BAZ2B3	8.34E-10	0.2107819	0.596	0.476	2.01E-05	2.6	BAZ2B	1.25210084
RPS10-NUDT32	8.79E-10	0.116175	0.089	0.046	2.12E-05	2.6	RPS10-NUI	1.934782609
MEF2D2	9.13E-10	0.1037547	0.118	0.066	2.20E-05	2.6	MEF2D	1.787878788
KLF65	9.19E-10	0.1212368	0.705	0.565	2.21E-05	2.6	KLF6	1.247787611
IRF2BPL4	9.47E-10	0.1446759	0.244	0.161	2.28E-05	2.6	IRF2BPL	1.51552795
PGRMC23	9.74E-10	0.1137383	0.2	0.126	2.35E-05	2.6	PGRMC2	1.587301587
SPTSSB4	9.87E-10	0.1600588	0.148	0.089	2.38E-05	2.6	SPTSSB	1.662921348
LINC005783	9.89E-10	0.184292	0.126	0.073	2.38E-05	2.6	LINC00578	1.726027397
ZSCAN16-AS13	1.07E-09	0.1534616	0.149	0.089	2.58E-05	2.6	ZSCAN16-A	1.674157303
CTD-2369P2.52	1.16E-09	0.1380902	0.061	0.027	2.80E-05	2.6	CTD-2369P	2.259259259
NT5DC31	1.17E-09	0.1603928	0.082	0.041	2.81E-05	2.6	NT5DC3	2
TTBK24	1.17E-09	0.1146131	0.237	0.156	2.82E-05	2.6	TTBK2	1.519230769
NUDT34	1.21E-09	0.1068112	0.133	0.076	2.92E-05	2.6	NUDT3	1.75
FADS33	1.26E-09	0.1590067	0.182	0.115	3.05E-05	2.6	FADS3	1.582608696
CDH110	1.42E-09	0.1360497	0.742	0.6	3.43E-05	2.6	CDH1	1.236666667
INTS124	1.51E-09	0.1616912	0.298	0.207	3.65E-05	2.6	INTS12	1.439613527
CARS23	1.52E-09	0.1008945	0.105	0.057	3.66E-05	2.6	CARS2	1.842105263
CNST2	1.58E-09	0.1452827	0.117	0.066	3.81E-05	2.6	CNST	1.772727273
SH2D4A2	1.59E-09	0.180104	0.1	0.054	3.83E-05	2.6	SH2D4A	1.851851852
HSDL12	1.63E-09	0.1617719	0.074	0.036	3.93E-05	2.6	HSDL1	2.055555556
LINC011372	1.63E-09	0.1357379	0.1	0.054	3.94E-05	2.6	LINC01137	1.851851852
ZCCHC143	1.71E-09	0.1356654	0.119	0.068	4.12E-05	2.6	ZCCHC14	1.75
TMPRSS11E4	1.74E-09	0.1177645	0.099	0.053	4.20E-05	2.6	TMPRSS11I	1.867924528
AZIN1-AS12	1.75E-09	0.1085651	0.097	0.051	4.21E-05	2.6	AZIN1-AS1	1.901960784
RPS6KA22	1.76E-09	0.135513	0.248	0.166	4.23E-05	2.6	RPS6KA2	1.493975904
WDR112	1.76E-09	0.1639039	0.141	0.084	4.25E-05	2.6	WDR11	1.678571429
TOM1L13	1.78E-09	0.1496815	0.366	0.262	4.28E-05	2.6	TOM1L1	1.396946565
UBAC23	1.98E-09	0.1764875	0.319	0.227	4.77E-05	2.6	UBAC2	1.405286344
GNS5	1.99E-09	0.1402723	0.151	0.091	4.80E-05	2.6	GNS	1.659340659

RAB305	2.00E-09	0.139623	0.19	0.12	4.82E-05	2.6	RAB30	1.583333333
GDE14	2.04E-09	0.1313803	0.218	0.142	4.92E-05	2.6	GDE1	1.535211268
FAM189A23	2.05E-09	0.1210411	0.117	0.066	4.95E-05	2.6	FAM189A2	1.772727273
CD823	2.11E-09	0.121775	0.095	0.05	5.10E-05	2.6	CD82	1.9
DLGAP42	2.12E-09	0.1043715	0.2	0.128	5.12E-05	2.6	DLGAP4	1.5625
HTT3	2.13E-09	0.1287794	0.246	0.165	5.13E-05	2.6	HTT	1.490909091
TCF123	2.20E-09	0.1089951	0.731	0.588	5.30E-05	2.6	TCF12	1.243197279
TRAF3IP12	2.21E-09	0.1130403	0.088	0.046	5.32E-05	2.6	TRAF3IP1	1.913043478
FAM193A2	2.22E-09	0.1196218	0.261	0.176	5.35E-05	2.6	FAM193A	1.482954545
MAP3K92	2.22E-09	0.1067027	0.243	0.161	5.35E-05	2.6	MAP3K9	1.50931677
GPS23	2.34E-09	0.1650157	0.249	0.168	5.64E-05	2.6	GPS2	1.482142857
TERF2IP2	2.42E-09	0.1434987	0.233	0.156	5.84E-05	2.6	TERF2IP	1.493589744
PARP83	2.58E-09	0.1268137	0.237	0.157	6.23E-05	2.6	PARP8	1.50955414
PAFAH1B14	2.59E-09	0.1329473	0.488	0.365	6.26E-05	2.6	PAFAH1B1	1.336986301
TBC1D44	2.61E-09	0.1618126	0.279	0.193	6.29E-05	2.6	TBC1D4	1.445595855
MSL14	2.78E-09	0.1527019	0.225	0.15	6.71E-05	2.6	MSL1	1.5
OCLN3	2.89E-09	0.1404019	0.449	0.333	6.97E-05	2.6	OCLN	1.348348348
GATA32	2.92E-09	0.1654409	0.274	0.191	7.05E-05	2.6	GATA3	1.434554974
PPP1R181	2.96E-09	0.1275859	0.102	0.055	7.13E-05	2.6	PPP1R18	1.854545455
BRWD13	2.96E-09	0.1237953	0.502	0.369	7.15E-05	2.6	BRWD1	1.360433604
ATP2C13	2.99E-09	0.1452723	0.318	0.225	7.20E-05	2.6	ATP2C1	1.413333333
TDRD32	3.05E-09	0.114467	0.169	0.104	7.36E-05	2.6	TDRD3	1.625
ZNF5162	3.09E-09	0.114013	0.149	0.089	7.45E-05	2.6	ZNF516	1.674157303
MBD53	3.24E-09	0.1183745	0.414	0.302	7.82E-05	2.6	MBD5	1.370860927
STK382	3.42E-09	0.1202092	0.356	0.251	8.24E-05	2.6	STK38	1.418326693
DENND1A3	3.50E-09	0.1416359	0.361	0.261	8.43E-05	2.6	DENND1A	1.383141762
PPP1CB3	3.65E-09	0.1331776	0.467	0.35	8.81E-05	2.6	PPP1CB	1.334285714
KMT2C1	3.80E-09	0.1381797	0.724	0.594	9.16E-05	2.6	KMT2C	1.218855219
OLFM23	3.86E-09	0.104122	0.063	0.029	9.32E-05	2.6	OLFM2	2.172413793
AUTS24	3.93E-09	0.1281256	0.654	0.523	9.48E-05	2.6	AUTS2	1.250478011
DGKD4	4.02E-09	0.1330841	0.191	0.122	9.70E-05	2.6	DGKD	1.56557377
PPARG3	4.19E-09	0.177999	0.228	0.154	0.000101145	2.6	PPARG	1.480519481
SPG202	4.27E-09	0.1209656	0.197	0.127	0.000102846	2.6	SPG20	1.551181102
H2AFZ3	4.37E-09	0.1052393	0.774	0.666	0.000105299	2.6	H2AFZ	1.162162162
DIXDC15	4.68E-09	0.2680609	0.178	0.114	0.000112843	2.6	DIXDC1	1.561403509
SPG74	4.72E-09	0.1542738	0.113	0.064	0.000113787	2.6	SPG7	1.765625
SLC9C13	4.92E-09	0.1358687	0.103	0.056	0.000118689	2.6	SLC9C1	1.839285714
KDM7A4	5.27E-09	0.1006885	0.381	0.27	0.000127008	2.6	KDM7A	1.411111111
FAM151B2	5.37E-09	0.1308795	0.113	0.064	0.00012937	2.6	FAM151B	1.765625
LMCD1-AS13	5.52E-09	0.1081098	0.086	0.045	0.000133018	2.6	LMCD1-AS1	1.911111111
SMARCA24	5.53E-09	0.1207552	0.414	0.307	0.000133311	2.6	SMARCA2	1.348534202
COL4A3BP2	5.55E-09	0.1536409	0.206	0.136	0.000133833	2.6	COL4A3BP	1.514705882
MARCKSL14	5.64E-09	0.1555091	0.228	0.15	0.000136078	2.6	MARCKSL1	1.52
KSR13	5.70E-09	0.1332517	0.078	0.04	0.000137407	2.6	KSR1	1.95
SNRNP2001	5.78E-09	0.1120504	0.193	0.124	0.000139278	2.6	SNRNP200	1.556451613
MYLIP4	5.83E-09	0.146263	0.148	0.091	0.000140628	2.6	MYLIP	1.626373626
LRCH32	6.17E-09	0.1602246	0.452	0.343	0.000148661	2.6	LRCH3	1.317784257
AHCTF12	6.24E-09	0.1395031	0.221	0.147	0.000150454	2.6	AHCTF1	1.503401361
GRHL23	6.25E-09	0.1989661	0.381	0.279	0.000150636	2.6	GRHL2	1.365591398
PRKAA23	6.33E-09	0.108154	0.197	0.127	0.000152597	2.6	PRKAA2	1.551181102
DNAH55	6.38E-09	0.1082459	0.117	0.067	0.000153869	2.6	DNAH5	1.746268657
ATXN23	6.61E-09	0.1293972	0.443	0.331	0.000159288	2.6	ATXN2	1.33836858

HNRNPC3	6.61E-09	0.1224286	0.873	0.78	0.000159502	2.6	HNRNPC	1.119230769
ZZEF13	7.15E-09	0.1340853	0.161	0.101	0.000172492	2.6	ZZEF1	1.594059406
MGME12	7.70E-09	0.1116215	0.128	0.076	0.000185659	2.6	MGME1	1.684210526
MAP3K73	7.71E-09	0.1293235	0.156	0.096	0.000185803	2.6	MAP3K7	1.625
MYOF3	7.78E-09	0.1314534	0.735	0.594	0.000187618	2.6	MYOF	1.237373737
VAT13	7.85E-09	0.1139233	0.209	0.137	0.000189314	2.6	VAT1	1.525547445
MLF13	8.25E-09	0.1144009	0.34	0.243	0.000198918	2.6	MLF1	1.399176955
PPP4R3B2	8.36E-09	0.1221809	0.25	0.171	0.000201642	2.6	PPP4R3B	1.461988304
TRIB13	8.54E-09	0.1010554	0.314	0.22	0.000205947	2.6	TRIB1	1.427272727
SLC39A13	8.72E-09	0.126279	0.163	0.103	0.000210355	2.6	SLC39A1	1.582524272
PPTC72	9.74E-09	0.1011449	0.275	0.191	0.000234768	2.6	PPTC7	1.439790576
GABPB12	1.06E-08	0.1477953	0.18	0.116	0.000256384	2.6	GABPB1	1.551724138
SNRPA12	1.22E-08	0.1392121	0.155	0.097	0.000294014	2.6	SNRPA1	1.597938144
BCOR5	1.23E-08	0.1603647	0.627	0.508	0.000296423	2.6	BCOR	1.234251969
SAFB23	1.27E-08	0.1024679	0.11	0.062	0.000305099	2.6	SAFB2	1.774193548
CLIP12	1.36E-08	0.1178438	0.511	0.396	0.000326747	2.6	CLIP1	1.29040404
DNMBP2	1.43E-08	0.1091034	0.118	0.069	0.000343684	2.6	DNMBP	1.710144928
ZNRF13	1.46E-08	0.1051473	0.244	0.165	0.000351015	2.6	ZNRF1	1.478787879
LPGAT12	1.47E-08	0.1319438	0.141	0.086	0.000354555	2.6	LPGAT1	1.639534884
ZBTB114	1.83E-08	0.1094574	0.14	0.085	0.000442471	2.6	ZBTB11	1.647058824
SREBF24	1.91E-08	0.119654	0.488	0.374	0.000460845	2.6	SREBF2	1.304812834
SLTM2	1.95E-08	0.162479	0.394	0.292	0.000469476	2.6	SLTM	1.349315068
PBX33	1.98E-08	0.1330507	0.144	0.088	0.000478087	2.6	PBX3	1.636363636
CMTM32	2.02E-08	0.1014106	0.079	0.041	0.000486043	2.6	CMTM3	1.926829268
PLGRKT3	2.06E-08	0.1202698	0.131	0.078	0.000496686	2.6	PLGRKT	1.679487179
C9orf842	2.07E-08	0.1258371	0.114	0.066	0.000499292	2.6	C9orf84	1.727272727
COQ10B1	2.12E-08	0.1215404	0.197	0.13	0.000510363	2.6	COQ10B	1.515384615
ZXDC4	2.17E-08	0.1027026	0.141	0.086	0.000523762	2.6	ZXDC	1.639534884
RP1-34B20.212	2.25E-08	0.1084727	0.088	0.048	0.000542359	2.6	RP1-34B20	1.833333333
PTTG1IP2	2.28E-08	0.1592632	0.236	0.164	0.00055054	2.6	PTTG1IP	1.43902439
TM9SF24	2.33E-08	0.1195887	0.351	0.255	0.00056286	2.6	TM9SF2	1.376470588
ZNF4872	2.34E-08	0.1282117	0.099	0.055	0.000565089	2.6	ZNF487	1.8
FAM83F2	2.43E-08	0.1110677	0.076	0.039	0.00058557	2.6	FAM83F	1.948717949
IRF63	2.46E-08	0.1038672	0.321	0.23	0.000593947	2.6	IRF6	1.395652174
TPCN14	2.48E-08	0.1244014	0.237	0.163	0.000598095	2.6	TPCN1	1.45398773
VPS13C3	2.52E-08	0.1208824	0.333	0.24	0.000608266	2.6	VPS13C	1.3875
LYPD63	2.54E-08	0.1132537	0.092	0.051	0.00061174	2.6	LYPD6	1.803921569
GLO14	2.58E-08	0.1370758	0.246	0.169	0.000621624	2.6	GLO1	1.455621302
ARHGEF63	2.66E-08	0.112192	0.126	0.076	0.000641777	2.6	ARHGEF6	1.657894737
WDTC12	2.69E-08	0.1289573	0.134	0.081	0.000649043	2.6	WDTC1	1.654320988
TAF16	2.70E-08	0.1182349	0.211	0.141	0.000651431	2.6	TAF1	1.496453901
RNF2133	2.83E-08	0.1091855	0.257	0.177	0.000683064	2.6	RNF213	1.451977401
RAB22A3	2.97E-08	0.1138126	0.208	0.138	0.000715899	2.6	RAB22A	1.507246377
YBX32	3.00E-08	0.1117008	0.382	0.283	0.000723684	2.6	YBX3	1.349823322
WDFY23	3.01E-08	0.1252161	0.361	0.263	0.000725747	2.6	WDFY2	1.372623574
ATRX3	3.04E-08	0.1423099	0.607	0.477	0.000731906	2.6	ATRX	1.272536688
BIRC63	3.12E-08	0.1529391	0.676	0.56	0.000751573	2.6	BIRC6	1.207142857
TRIP123	3.22E-08	0.1139098	0.434	0.324	0.000776722	2.6	TRIP12	1.339506173
FAM120B3	3.24E-08	0.1407754	0.18	0.118	0.000780811	2.6	FAM120B	1.525423729
LYPD33	3.24E-08	0.1260778	0.108	0.063	0.000781045	2.6	LYPD3	1.714285714
DNM31	3.34E-08	0.1370132	0.08	0.043	0.000805207	2.6	DNM3	1.860465116
MCC3	3.46E-08	0.1218001	0.161	0.103	0.000834216	2.6	MCC	1.563106796

ARIH21	3.51E-08	0.1196035	0.211	0.141	0.000845237	2.6	ARIH2	1.496453901
BBOF14	3.74E-08	0.1017783	0.091	0.05	0.000902222	2.6	BBOF1	1.82
CNOT23	3.89E-08	0.1501749	0.517	0.4	0.000937359	2.6	CNOT2	1.2925
NOP104	4.05E-08	0.1158529	0.678	0.552	0.000975473	2.6	NOP10	1.22826087
MREG3	4.33E-08	0.1311589	0.208	0.139	0.001044048	2.6	MREG	1.496402878
YWHAB3	4.37E-08	0.1153655	0.42	0.311	0.001054373	2.6	YWHAB	1.350482315
CTNNAL13	4.45E-08	0.1246253	0.191	0.126	0.001073456	2.6	CTNNAL1	1.515873016
PPM1A3	4.54E-08	0.1618781	0.246	0.172	0.001093716	2.6	PPM1A	1.430232558
NUP982	4.57E-08	0.1243718	0.291	0.205	0.001102078	2.6	NUP98	1.419512195
TRIM272	4.60E-08	0.1345858	0.142	0.088	0.001109396	2.6	TRIM27	1.613636364
SLC22A41	4.64E-08	0.1023187	0.048	0.021	0.001119004	2.6	SLC22A4	2.285714286
DPY19L13	4.71E-08	0.1477123	0.151	0.095	0.001135691	2.6	DPY19L1	1.589473684
RPS6KC12	4.73E-08	0.1075036	0.166	0.107	0.001141322	2.6	RPS6KC1	1.551401869
DOT1L2	4.81E-08	0.1008864	0.054	0.025	0.00115922	2.6	DOT1L	2.16
FLJ128253	4.90E-08	0.1265171	0.121	0.073	0.001182216	2.6	FLJ12825	1.657534247
IL13RA13	4.99E-08	0.1075094	0.186	0.122	0.001203371	2.6	IL13RA1	1.524590164
SENP62	5.13E-08	0.1267581	0.404	0.307	0.001236076	2.6	SENP6	1.315960912
MARK13	5.17E-08	0.1068711	0.109	0.063	0.001247237	2.6	MARK1	1.73015873
SPSB12	5.18E-08	0.110144	0.137	0.084	0.001248433	2.6	SPSB1	1.630952381
ABHD22	5.21E-08	0.1311308	0.213	0.144	0.0012573	2.6	ABHD2	1.479166667
CHD13	5.36E-08	0.1042713	0.365	0.267	0.001293648	2.6	CHD1	1.367041199
SYT93	5.40E-08	0.1299921	0.08	0.043	0.00130176	2.6	SYT9	1.860465116
UBXN44	5.42E-08	0.1181557	0.41	0.307	0.001306433	2.6	UBXN4	1.335504886
GNG12-AS12	5.68E-08	0.103029	0.128	0.078	0.001368908	2.6	GNG12-AS1	1.641025641
SQRDL3	5.72E-08	0.1257288	0.262	0.185	0.001378348	2.6	SQRDL	1.416216216
CCSER21	6.13E-08	0.115001	0.261	0.183	0.001479207	2.6	CCSER2	1.426229508
OTUD15	6.36E-08	0.1269476	0.091	0.051	0.001533658	2.6	OTUD1	1.784313725
GNG72	6.37E-08	0.2397701	0.1	0.057	0.001535732	2.6	GNG7	1.754385965
WIPF24	6.52E-08	0.1163166	0.221	0.151	0.001571007	2.6	WIPF2	1.463576159
INO80D4	6.58E-08	0.1291887	0.684	0.563	0.001585452	2.6	INO80D	1.214920071
NRDC2	6.92E-08	0.1715323	0.292	0.212	0.001667914	2.6	NRDC	1.377358491
RPGR2	6.98E-08	0.1060328	0.142	0.088	0.001683384	2.6	RPGR	1.613636364
EPAS13	7.06E-08	0.1021689	0.221	0.15	0.001701637	2.6	EPAS1	1.473333333
HNRNPH11	7.54E-08	0.1595111	0.527	0.413	0.00181853	2.6	HNRNPH1	1.276029056
NCKAP13	7.91E-08	0.105952	0.553	0.429	0.001906992	2.6	NCKAP1	1.289044289
PLSCR23	7.94E-08	0.1007487	0.101	0.058	0.001914287	2.6	PLSCR2	1.74137931
CCDC502	8.29E-08	0.1692018	0.24	0.169	0.001998332	2.6	CCDC50	1.420118343
MCPH13	8.29E-08	0.1350029	0.248	0.173	0.001998677	2.6	MCPH1	1.433526012
UBR42	8.93E-08	0.12031	0.314	0.228	0.002153832	2.6	UBR4	1.377192982
PML3	9.00E-08	0.1157548	0.127	0.079	0.002168997	2.6	PML	1.607594937
UBR54	9.07E-08	0.1704676	0.581	0.466	0.002186038	2.6	UBR5	1.246781116
BTBD92	9.17E-08	0.1405256	0.348	0.257	0.002211226	2.6	BTBD9	1.354085603
DLG1-AS13	9.21E-08	0.1171293	0.078	0.042	0.002219995	2.6	DLG1-AS1	1.857142857
SIAE4	1.02E-07	0.1013257	0.083	0.046	0.002463784	2.6	SIAE	1.804347826
PTPRA2	1.03E-07	0.1141751	0.256	0.18	0.002491436	2.6	PTPRA	1.422222222
MYO5C2	1.05E-07	0.1015466	0.219	0.15	0.002532914	2.6	MYO5C	1.46
ZNFX12	1.10E-07	0.1377257	0.19	0.128	0.002645346	2.6	ZNFX1	1.484375
PPP4R12	1.10E-07	0.1017743	0.32	0.231	0.002659832	2.6	PPP4R1	1.385281385
DACH14	1.11E-07	0.105424	0.151	0.096	0.002684405	2.6	DACH1	1.572916667
HACD22	1.13E-07	0.1253637	0.144	0.092	0.002713083	2.6	HACD2	1.565217391
ZNF4383	1.13E-07	0.1218727	0.116	0.07	0.002713139	2.6	ZNF438	1.657142857
PNPLA83	1.17E-07	0.124568	0.366	0.276	0.002816157	2.6	PNPLA8	1.326086957

RP11-507B12.24	1.19E-07	0.1717964	0.156	0.1	0.00286269	2.6	RP11-507B	1.56
HELLS4	1.24E-07	0.1025192	0.055	0.027	0.002988934	2.6	HELLS	2.037037037
HIST1H2BB4	1.34E-07	0.1325528	0.239	0.168	0.003240491	2.6	HIST1H2BB	1.422619048
CYSTM12	1.39E-07	0.1206374	0.284	0.205	0.003357448	2.6	CYSTM1	1.385365854
FBXO213	1.45E-07	0.1014926	0.162	0.105	0.003498542	2.6	FBXO21	1.542857143
MCU4	1.52E-07	0.1021911	0.186	0.124	0.003667304	2.6	MCU	1.5
VPS13B3	1.53E-07	0.1139945	0.294	0.211	0.003683285	2.6	VPS13B	1.393364929
WDR14	1.54E-07	0.1160752	0.251	0.176	0.003704052	2.6	WDR1	1.426136364
WDR444	1.63E-07	0.1019384	0.234	0.162	0.003932518	2.6	WDR44	1.444444444
SLC9A83	1.78E-07	0.1157045	0.137	0.086	0.0042812	2.6	SLC9A8	1.593023256
SUCLG23	1.80E-07	0.111753	0.199	0.134	0.004328898	2.6	SUCLG2	1.485074627
TRA2A2	1.80E-07	0.129128	0.503	0.398	0.004334182	2.6	TRA2A	1.263819095
ABHD17C3	1.82E-07	0.1232726	0.152	0.098	0.004392519	2.6	ABHD17C	1.551020408
CR1L1	1.88E-07	0.1168758	0.072	0.038	0.004526126	2.6	CR1L	1.894736842
DDX3X3	2.32E-07	0.111771	0.472	0.36	0.005592221	2.6	DDX3X	1.311111111
ZFX2	2.42E-07	0.1188871	0.175	0.116	0.005823894	2.6	ZFX	1.50862069
DYNLT33	2.46E-07	0.1198493	0.359	0.267	0.00594295	2.6	DYNLT3	1.344569288
DAZAP23	2.51E-07	0.1145848	0.535	0.421	0.006062327	2.6	DAZAP2	1.270783848
SRRM23	2.60E-07	0.1165011	0.294	0.214	0.006271669	2.6	SRRM2	1.373831776
SLK3	2.62E-07	0.1139384	0.319	0.233	0.006316214	2.6	SLK	1.369098712
RP11-6N13.14	2.76E-07	0.1290309	0.08	0.044	0.006646583	2.6	RP11-6N13	1.818181818
ELF14	2.91E-07	0.1157751	0.652	0.524	0.007014521	2.6	ELF1	1.244274809
KPNA63	3.02E-07	0.105429	0.379	0.282	0.007283728	2.6	KPNA6	1.343971631
NHS3	3.23E-07	0.2458699	0.23	0.167	0.007790992	2.6	NHS	1.377245509
MARS3	3.33E-07	0.1130299	0.21	0.145	0.008022258	2.6	MARS	1.448275862
UBE2E13	3.61E-07	0.1000836	0.446	0.342	0.008698588	2.6	UBE2E1	1.304093567
TMEM161B-AS13	3.86E-07	0.1714456	0.161	0.108	0.009308201	2.6	TMEM161E	1.490740741
IWS12	4.04E-07	0.1159331	0.213	0.147	0.009736621	2.6	IWS1	1.448979592
TMEM178B2	4.21E-07	0.1408117	0.12	0.075	0.01014418	2.6	TMEM178E	1.6
RND34	4.44E-07	0.101442	0.451	0.347	0.01069929	2.6	RND3	1.299711816
KLHL26	4.46E-07	0.1354289	0.197	0.136	0.01075919	2.6	KLHL2	1.448529412
JMY2	4.57E-07	0.1489986	0.188	0.13	0.01102229	2.6	JMY	1.446153846
CTC-490E21.123	4.60E-07	0.1290041	0.096	0.056	0.01109763	2.6	CTC-490E2	1.714285714
PPP4R3A2	4.89E-07	0.1072623	0.256	0.182	0.01179263	2.6	PPP4R3A	1.406593407
PIGA2	4.95E-07	0.102206	0.146	0.094	0.01193987	2.6	PIGA	1.553191489
RP11-238K6.14	5.00E-07	0.1557472	0.177	0.121	0.01205341	2.6	RP11-238K	1.462809917
PRPF82	5.04E-07	0.1129763	0.131	0.083	0.01216078	2.6	PRPF8	1.578313253
SPTBN12	5.09E-07	0.1028125	0.297	0.217	0.01227398	2.6	SPTBN1	1.368663594
AC011288.22	5.11E-07	0.1322637	0.102	0.061	0.01231199	2.6	AC011288.:	1.672131148
CFAP702	5.34E-07	0.1018842	0.075	0.041	0.01288693	2.6	CFAP70	1.829268293
ATP6AP25	5.54E-07	0.1074504	0.205	0.142	0.0133504	2.6	ATP6AP2	1.443661972
SMC63	5.74E-07	0.1011858	0.117	0.073	0.01384224	2.6	SMC6	1.602739726
TRAF42	5.94E-07	0.1119344	0.082	0.047	0.01432211	2.6	TRAF4	1.744680851
TRAF3IP23	5.96E-07	0.1094486	0.182	0.124	0.01437305	2.6	TRAF3IP2	1.467741935
PRKD33	6.08E-07	0.1066789	0.166	0.111	0.01466516	2.6	PRKD3	1.495495495
LINC006572	6.19E-07	0.1158685	0.252	0.179	0.01492228	2.6	LINC00657	1.407821229
ZNF6083	6.97E-07	0.1304245	0.249	0.177	0.01680015	2.6	ZNF608	1.406779661
KANSL1L3	7.80E-07	0.1273396	0.286	0.213	0.01880022	2.6	KANSL1L	1.342723005
ADCY23	7.87E-07	0.1049961	0.109	0.067	0.01897981	2.6	ADCY2	1.626865672
GGNBP23	8.55E-07	0.12296	0.304	0.227	0.02062659	2.6	GGNBP2	1.339207048
SUMF13	9.18E-07	0.1358622	0.225	0.16	0.02212982	2.6	SUMF1	1.40625
GLTSCR1L4	9.22E-07	0.1246662	0.199	0.14	0.02223415	2.6	GLTSCR1L	1.421428571

C4orf192	9.60E-07	0.1003564	0.205	0.143	0.02313721	2.6	C4orf19	1.433566434
PEX132	9.91E-07	0.1080542	0.176	0.119	0.02390017	2.6	PEX13	1.478991597
AC073218.14	1.06E-06	0.1125953	0.136	0.088	0.0255773	2.6	AC073218.	1.545454545
FAM168B2	1.09E-06	0.1059362	0.138	0.09	0.02635445	2.6	FAM168B	1.533333333
LINC013174	1.11E-06	0.1084235	0.127	0.082	0.02674937	2.6	LINC01317	1.548780488
CCDC663	1.12E-06	0.1064317	0.194	0.135	0.0269769	2.6	CCDC66	1.437037037
LUZP13	1.17E-06	0.1100408	0.172	0.116	0.02821326	2.6	LUZP1	1.482758621
FANK14	1.41E-06	0.1119251	0.212	0.149	0.0341015	2.6	FANK1	1.422818792
UACA4	1.47E-06	0.1381725	0.267	0.198	0.0354919	2.6	UACA	1.348484848
GNG122	1.47E-06	0.1097978	0.42	0.325	0.03554419	2.6	GNG12	1.292307692
TYW12	1.59E-06	0.1573573	0.138	0.089	0.03842668	2.6	TYW1	1.550561798
ST142	1.63E-06	0.1111753	0.242	0.174	0.0393948	2.6	ST14	1.390804598
C5orf172	1.91E-06	0.1589513	0.168	0.115	0.04609596	2.6	C5orf17	1.460869565
RHOV4	1.93E-06	0.1198773	0.082	0.048	0.04655867	2.6	RHOV	1.708333333
RP11-1379J22.22	1.93E-06	0.1173403	0.105	0.065	0.04661793	2.6	RP11-1379.	1.615384615
RP11-206M11.72	0	4.495255	0.588	0.071	0	3	RP11-206M	8.281690141
PIP1	0	4.261205	0.533	0.061	0	3	PIP	8.737704918
SERPINA12	0	2.12721	0.517	0.064	0	3	SERPINA1	8.078125
TFPI21	0	1.490705	0.339	0.005	0	3	TFPI2	67.8
TAT2	0	1.318974	0.222	0.007	0	3	TAT	31.71428571
MSMB	0	1.297772	0.157	0.003	0	3	MSMB	52.33333333
IGSF1	0	0.7730943	0.182	0.005	0	3	IGSF1	36.4
GLRA3	0	0.6432149	0.13	0.002	0	3	GLRA3	65
PI15	3.35E-304	2.131779	0.262	0.015	8.09E-300	3	PI15	17.46666667
HMGB3	3.21E-267	1.704935	0.381	0.041	7.73E-263	3	HMGB3	9.292682927
APOD2	7.57E-262	3.30857	0.404	0.048	1.83E-257	3	APOD	8.416666667
MUCL11	2.64E-261	6.700421	0.81	0.262	6.37E-257	3	MUCL1	3.091603053
TAT-AS12	1.17E-158	0.648582	0.128	0.007	2.82E-154	3	TAT-AS1	18.28571429
PCAT18	9.24E-149	0.3058334	0.092	0.003	2.23E-144	3	PCAT18	30.66666667
FGG	1.86E-147	1.079728	0.044	0	4.48E-143	3	FGG	#DIV/0!
CP2	9.37E-147	2.187467	0.341	0.057	2.26E-142	3	CP	5.98245614
PPP4R43	3.17E-139	1.297753	0.421	0.085	7.64E-135	3	PPP4R4	4.952941176
MIR2052HG	1.45E-130	0.5914698	0.096	0.005	3.50E-126	3	MIR2052H(	19.2
CYP4X11	7.90E-127	0.3168795	0.092	0.004	1.91E-122	3	CYP4X1	23
UBXN10-AS1	1.89E-124	0.2539088	0.063	0.002	4.57E-120	3	UBXN10-AS	31.5
TANC23	5.57E-109	1.71995	0.82	0.47	1.34E-104	3	TANC2	1.744680851
NPY1R	2.17E-104	0.3447553	0.096	0.006	5.23E-100	3	NPY1R	16
RP11-136K7.2	2.38E-103	0.2065293	0.048	0.001	5.73E-99	3	RP11-136K	48
HCAR3	5.65E-98	0.3679659	0.092	0.006	1.36E-93	3	HCAR3	15.33333333
MAOA4	1.36E-90	0.7870239	0.272	0.054	3.28E-86	3	MAOA	5.037037037
SLC1A12	9.94E-88	0.4672721	0.134	0.014	2.40E-83	3	SLC1A1	9.571428571
CFTR1	1.57E-86	0.630668	0.119	0.012	3.79E-82	3	CFTR	9.916666667
FGB	3.42E-85	0.387811	0.023	0	8.24E-81	3	FGB	#DIV/0!
GLUL2	2.11E-82	1.906775	0.705	0.371	5.08E-78	3	GLUL	1.900269542
CTB-33O18.12	1.57E-78	0.2296905	0.059	0.003	3.79E-74	3	CTB-33O18	19.66666667
AQP34	3.65E-73	0.6758135	0.245	0.052	8.80E-69	3	AQP3	4.711538462
HMGCS21	2.81E-68	0.297846	0.059	0.003	6.78E-64	3	HMGCS2	19.66666667
AZGP15	3.90E-61	1.243417	0.86	0.542	9.41E-57	3	AZGP1	1.586715867
IRAK32	8.30E-60	0.4282204	0.126	0.018	2.00E-55	3	IRAK3	7
SAT13	2.79E-57	0.9729307	1	0.98	6.73E-53	3	SAT1	1.020408163
CECR2	1.49E-54	0.4313121	0.142	0.025	3.59E-50	3	CECR2	5.68
MGST14	3.41E-54	0.8867759	0.952	0.858	8.23E-50	3	MGST1	1.10955711

TMC53	3.73E-54	1.053194	0.498	0.216	9.00E-50	3	TMC5	2.305555556
LEF1	6.69E-50	0.2012244	0.04	0.002	1.61E-45	3	LEF1	20
INPP4B4	1.19E-46	0.8951315	0.621	0.324	2.88E-42	3	INPP4B	1.916666667
MGP3	2.53E-44	0.6297929	0.956	0.816	6.11E-40	3	MGP	1.171568627
MALAT16	1.88E-43	0.4632744	1	1	4.54E-39	3	MALAT1	1
KCCAT2112	2.11E-43	1.005045	0.303	0.109	5.10E-39	3	KCCAT211	2.779816514
SUB16	1.27E-42	1.369992	0.81	0.635	3.07E-38	3	SUB1	1.275590551
ORM1	8.14E-42	0.1071661	0.013	0	1.96E-37	3	ORM1	#DIV/0!
RP11-146N18.1	1.90E-41	0.1768769	0.038	0.002	4.59E-37	3	RP11-146N	19
PTGR12	3.54E-41	1.049687	0.531	0.278	8.53E-37	3	PTGR1	1.910071942
DKK11	1.43E-40	0.1723964	0.021	0.001	3.46E-36	3	DKK1	21
TMEM1655	2.50E-40	0.9146241	0.728	0.518	6.04E-36	3	TMEM165	1.405405405
HNMT4	3.50E-40	1.144468	0.531	0.292	8.43E-36	3	HNMT	1.818493151
RP11-556O9.22	4.14E-40	0.1996466	0.075	0.01	9.99E-36	3	RP11-556O	7.5
CERS3-AS11	5.61E-40	0.1688889	0.046	0.004	1.35E-35	3	CERS3-AS1	11.5
SRD5A32	7.96E-40	0.6729288	0.222	0.068	1.92E-35	3	SRD5A3	3.264705882
RP11-355F16.12	9.20E-40	0.2711386	0.054	0.005	2.22E-35	3	RP11-355F:	10.8
S100P3	3.69E-39	0.6088557	0.188	0.051	8.91E-35	3	S100P	3.68627451
KCNE43	1.03E-38	0.4004439	0.134	0.029	2.49E-34	3	KCNE4	4.620689655
LINC013442	1.22E-38	0.7633071	0.14	0.032	2.94E-34	3	LINC01344	4.375
TNFSF104	2.06E-38	1.361422	0.665	0.469	4.97E-34	3	TNFSF10	1.417910448
SLC4A74	4.26E-38	1.085329	0.51	0.276	1.03E-33	3	SLC4A7	1.847826087
CSGALNACT13	7.93E-38	1.029001	0.4	0.182	1.91E-33	3	CSGALNAC	2.197802198
CYP1B13	1.30E-37	1.7428	0.287	0.112	3.15E-33	3	CYP1B1	2.5625
C15orf483	2.17E-37	1.476702	0.701	0.495	5.24E-33	3	C15orf48	1.416161616
RUNX22	4.76E-37	0.2897939	0.113	0.022	1.15E-32	3	RUNX2	5.136363636
ANKRD30B3	6.41E-37	1.070137	0.1	0.018	1.55E-32	3	ANKRD30B	5.555555556
PRDX13	2.51E-35	0.6840862	0.845	0.681	6.06E-31	3	PRDX1	1.24082232
TMEM1565	1.69E-34	0.4752228	0.174	0.048	4.07E-30	3	TMEM156	3.625
AC037445.12	4.68E-33	0.39927	0.182	0.053	1.13E-28	3	AC037445.	3.433962264
TRAFD14	4.56E-32	0.8582206	0.383	0.181	1.10E-27	3	TRAFD1	2.116022099
NEAT15	8.17E-32	0.7865034	0.962	0.947	1.97E-27	3	NEAT1	1.015839493
RHOBTB36	1.19E-30	0.578199	0.312	0.13	2.88E-26	3	RHOBTB3	2.4
KIAA15514	7.94E-30	0.9343813	0.287	0.122	1.91E-25	3	KIAA1551	2.352459016
HGD4	1.17E-29	0.2345114	0.084	0.016	2.81E-25	3	HGD	5.25
CYP4Z11	1.55E-29	0.3748405	0.073	0.012	3.74E-25	3	CYP4Z1	6.083333333
ORM2	3.31E-29	0.1374151	0.01	0	7.98E-25	3	ORM2	#DIV/0!
NEK103	5.37E-29	0.834497	0.326	0.148	1.29E-24	3	NEK10	2.202702703
AP004372.13	1.68E-28	0.194273	0.065	0.01	4.05E-24	3	AP004372.:	6.5
PIEZO2	1.84E-28	0.2463131	0.077	0.014	4.43E-24	3	PIEZO2	5.5
BCAP293	8.13E-28	0.5354097	0.328	0.148	1.96E-23	3	BCAP29	2.216216216
RP11-693J15.6	9.25E-28	0.1067084	0.015	0	2.23E-23	3	RP11-693J1	#DIV/0!
FRY2	2.72E-27	0.6973089	0.249	0.1	6.55E-23	3	FRY	2.49
CCDC109B1	5.58E-26	0.3755785	0.197	0.07	1.35E-21	3	CCDC109B	2.814285714
LINC008624	6.24E-26	0.3568159	0.136	0.039	1.50E-21	3	LINC00862	3.487179487
ANKRD36C3	2.09E-25	1.019846	0.636	0.455	5.04E-21	3	ANKRD36C	1.397802198
PON31	2.84E-25	0.1122715	0.04	0.005	6.85E-21	3	PON3	8
PDLIM12	1.15E-24	0.4690102	0.274	0.119	2.77E-20	3	PDLIM1	2.302521008
ZNF7371	2.70E-24	0.2356684	0.092	0.021	6.51E-20	3	ZNF737	4.380952381
NFIA2	2.71E-24	0.6132635	0.366	0.187	6.53E-20	3	NFIA	1.957219251
SORD4	4.10E-24	0.3724734	0.117	0.032	9.89E-20	3	SORD	3.65625
UGT2B111	4.31E-24	0.2378829	0.029	0.003	1.04E-19	3	UGT2B11	9.666666667

HILPDA3	5.41E-24	1.188445	0.462	0.275	1.30E-19	3	HILPDA	1.68
AMN12	1.91E-23	0.5379475	0.318	0.155	4.61E-19	3	AMN1	2.051612903
MGAT4A3	2.14E-23	0.4189303	0.224	0.089	5.17E-19	3	MGAT4A	2.516853933
LIN7A3	2.30E-23	0.3197067	0.144	0.045	5.55E-19	3	LIN7A	3.2
SLC39A66	7.28E-23	0.7024435	0.496	0.3	1.76E-18	3	SLC39A6	1.653333333
PLCL1	1.95E-22	0.378033	0.107	0.029	4.71E-18	3	PLCL1	3.689655172
XBP13	2.29E-22	0.830628	0.577	0.388	5.53E-18	3	XBP1	1.487113402
TENM31	5.74E-22	0.168854	0.084	0.019	1.38E-17	3	TENM3	4.421052632
ABCA124	7.54E-22	0.2268355	0.09	0.022	1.82E-17	3	ABCA12	4.090909091
NTS	9.76E-22	0.2636045	0.006	0	2.35E-17	3	NTS	#DIV/0!
TIMP13	9.78E-22	0.6280829	0.178	0.066	2.36E-17	3	TIMP1	2.696969697
FGA	9.79E-22	0.1023182	0.006	0	2.36E-17	3	FGA	#DIV/0!
LURAP1L3	1.63E-21	0.5729697	0.429	0.245	3.93E-17	3	LURAP1L	1.751020408
SC5D5	1.75E-21	0.5633439	0.347	0.181	4.22E-17	3	SC5D	1.917127072
FSIP15	1.96E-21	0.3497944	0.151	0.051	4.72E-17	3	FSIP1	2.960784314
ESRRG1	2.31E-21	0.4603827	0.155	0.054	5.57E-17	3	ESRRG	2.87037037
SYTL24	2.38E-21	0.5497975	0.663	0.448	5.74E-17	3	SYTL2	1.479910714
AR6	5.35E-21	0.3972877	0.324	0.158	1.29E-16	3	AR	2.050632911
PNPLA33	1.09E-20	0.3209721	0.105	0.03	2.63E-16	3	PNPLA3	3.5
TTC65	1.13E-20	0.4552144	0.339	0.172	2.73E-16	3	TTC6	1.970930233
DHCR244	1.65E-20	0.5356308	0.343	0.185	3.97E-16	3	DHCR24	1.854054054
RP11-15H20.72	4.16E-20	0.3065525	0.163	0.06	1.00E-15	3	RP11-15H2	2.716666667
ZNF385B4	1.50E-19	0.2148306	0.077	0.019	3.62E-15	3	ZNF385B	4.052631579
PRKAR2B2	1.51E-18	0.3771282	0.146	0.053	3.64E-14	3	PRKAR2B	2.754716981
TCN12	3.23E-18	0.3905358	0.075	0.019	7.80E-14	3	TCN1	3.947368421
SRI2	4.22E-18	0.7248583	0.209	0.093	1.02E-13	3	SRI	2.247311828
IL24	4.27E-18	0.2252089	0.013	0.001	1.03E-13	3	IL24	13
CERS65	4.36E-18	0.3730667	0.322	0.164	1.05E-13	3	CERS6	1.963414634
HMOX11	4.36E-18	0.27079	0.14	0.05	1.05E-13	3	HMOX1	2.8
PAPSS21	6.55E-18	0.4318091	0.1	0.03	1.58E-13	3	PAPSS2	3.333333333
MARCH101	7.84E-18	0.1349184	0.046	0.008	1.89E-13	3	MARCH10	5.75
STC23	2.29E-17	0.6138156	0.209	0.094	5.51E-13	3	STC2	2.223404255
ADD35	2.66E-17	0.5075058	0.343	0.194	6.43E-13	3	ADD3	1.768041237
GCLM3	2.98E-17	0.5683655	0.278	0.146	7.19E-13	3	GCLM	1.904109589
MITF3	3.94E-17	0.2962434	0.103	0.032	9.50E-13	3	MITF	3.21875
LINC012074	3.99E-17	0.2642216	0.105	0.033	9.61E-13	3	LINC01207	3.181818182
ZNF3501	5.25E-17	0.2364924	0.115	0.039	1.27E-12	3	ZNF350	2.948717949
ABCC114	8.08E-17	0.2158704	0.061	0.014	1.95E-12	3	ABCC11	4.357142857
TSPAN55	1.52E-16	0.5121	0.49	0.306	3.66E-12	3	TSPAN5	1.60130719
CA123	1.62E-16	0.5080086	0.464	0.295	3.91E-12	3	CA12	1.572881356
ST8SIA64	1.67E-16	0.3587103	0.259	0.128	4.03E-12	3	ST8SIA6	2.0234375
SEPP14	2.35E-16	0.7212309	0.464	0.31	5.66E-12	3	SEPP1	1.496774194
OSGIN21	3.53E-16	0.3527267	0.151	0.06	8.52E-12	3	OSGIN2	2.516666667
S100A63	6.36E-16	0.7378059	0.383	0.58	1.53E-11	3	S100A6	0.660344828
COPZ14	9.67E-16	0.4259755	0.487	0.319	2.33E-11	3	COPZ1	1.526645768
CERS43	2.00E-15	0.1974934	0.098	0.032	4.82E-11	3	CERS4	3.0625
GK1	2.45E-15	0.2443442	0.132	0.05	5.91E-11	3	GK	2.64
PDZK12	6.52E-15	0.2806536	0.079	0.024	1.57E-10	3	PDZK1	3.291666667
TMSB106	8.79E-15	0.1864976	0.594	0.737	2.12E-10	3	TMSB10	0.805970149
TSPAN82	1.19E-14	0.3567186	0.071	0.02	2.88E-10	3	TSPAN8	3.55
SLC40A13	1.54E-14	0.1915443	0.075	0.022	3.72E-10	3	SLC40A1	3.409090909
CTA-392E5.13	1.67E-14	0.307288	0.096	0.032	4.03E-10	3	CTA-392E5	3

SHTN12	3.87E-14	0.3625179	0.245	0.128	9.34E-10	3	SHTN1	1.9140625
CAPN85	4.00E-14	0.3927491	0.368	0.217	9.65E-10	3	CAPN8	1.695852535
DGKD5	4.97E-14	0.2943235	0.243	0.123	1.20E-09	3	DGKD	1.975609756
SLC41A24	8.91E-14	0.3366756	0.232	0.118	2.15E-09	3	SLC41A2	1.966101695
TGFB23	9.70E-14	1.058376	0.305	0.187	2.34E-09	3	TGFB2	1.631016043
LETM21	1.10E-13	0.1243214	0.036	0.007	2.66E-09	3	LETM2	5.142857143
ZNF2172	1.20E-13	0.4674831	0.316	0.183	2.88E-09	3	ZNF217	1.726775956
KMO2	1.25E-13	0.2218273	0.082	0.026	3.02E-09	3	KMO	3.153846154
ERN14	1.34E-13	0.4565947	0.28	0.157	3.22E-09	3	ERN1	1.78343949
BLVRA1	1.96E-13	0.3466633	0.203	0.101	4.73E-09	3	BLVRA	2.00990099
RP11-563J2.23	2.19E-13	0.1405012	0.054	0.014	5.27E-09	3	RP11-563J2	3.857142857
RALGAPA22	2.44E-13	0.4146743	0.4	0.257	5.88E-09	3	RALGAPA2	1.556420233
VMP13	2.84E-13	0.3474678	0.841	0.746	6.84E-09	3	VMP1	1.127345845
CYB5A5	5.39E-13	0.5407299	0.406	0.257	1.30E-08	3	CYB5A	1.579766537
PAPD53	6.27E-13	0.3034328	0.142	0.061	1.51E-08	3	PAPD5	2.327868852
ZNF662	7.45E-13	0.2116383	0.09	0.031	1.80E-08	3	ZNF66	2.903225806
IDH22	1.07E-12	0.2717167	0.165	0.077	2.57E-08	3	IDH2	2.142857143
CHCHD74	1.57E-12	0.2564564	0.178	0.085	3.79E-08	3	CHCHD7	2.094117647
FN16	1.68E-12	0.1926512	0.084	0.028	4.05E-08	3	FN1	3
AKR1C13	1.86E-12	0.9209508	0.155	0.072	4.49E-08	3	AKR1C1	2.152777778
UGDH4	1.91E-12	0.3025128	0.389	0.235	4.60E-08	3	UGDH	1.655319149
DIRAS31	1.93E-12	0.1153532	0.023	0.003	4.66E-08	3	DIRAS3	7.666666667
ZNF2814	2.02E-12	0.3364141	0.222	0.116	4.88E-08	3	ZNF281	1.913793103
TMEM167A2	2.14E-12	0.3911209	0.404	0.262	5.16E-08	3	TMEM167A	1.541984733
GNB42	3.00E-12	0.1192135	0.059	0.016	7.23E-08	3	GNB4	3.6875
STEAP43	3.15E-12	0.233968	0.092	0.033	7.59E-08	3	STEAP4	2.787878788
ABCC33	4.34E-12	0.3025956	0.172	0.082	1.05E-07	3	ABCC3	2.097560976
RP11-356O9.12	4.42E-12	0.137145	0.04	0.009	1.07E-07	3	RP11-356O	4.444444444
LMO74	4.67E-12	0.4259014	0.308	0.184	1.13E-07	3	LMO7	1.673913043
PLAT6	4.71E-12	0.3205251	0.178	0.086	1.14E-07	3	PLAT	2.069767442
OLMALINC2	5.45E-12	0.2124355	0.109	0.043	1.31E-07	3	OLMALINC	2.534883721
NPR32	1.44E-11	0.2182098	0.048	0.012	3.48E-07	3	NPR3	4
AFMID2	1.47E-11	0.392612	0.224	0.123	3.55E-07	3	AFMID	1.821138211
ECHDC23	2.30E-11	0.2822248	0.222	0.12	5.54E-07	3	ECHDC2	1.85
DUSP166	2.83E-11	0.3250692	0.567	0.42	6.83E-07	3	DUSP16	1.35
KIAA19583	3.44E-11	0.2730511	0.249	0.139	8.30E-07	3	KIAA1958	1.791366906
TMEM596	3.73E-11	0.3500831	0.596	0.451	9.00E-07	3	TMEM59	1.321507761
ZRSR21	5.00E-11	0.1793943	0.142	0.064	1.20E-06	3	ZRSR2	2.21875
FNIP25	5.33E-11	0.2891466	0.203	0.107	1.29E-06	3	FNIP2	1.897196262
CHMP55	7.92E-11	0.3629082	0.569	0.435	1.91E-06	3	CHMP5	1.308045977
ATP6V1G13	9.79E-11	0.4229153	0.527	0.388	2.36E-06	3	ATP6V1G1	1.358247423
RP11-545E17.32	1.18E-10	0.1254908	0.054	0.016	2.84E-06	3	RP11-545E	3.375
FGFR22	1.19E-10	0.1155226	0.069	0.023	2.86E-06	3	FGFR2	3
MXD14	2.74E-10	0.3829187	0.331	0.211	6.60E-06	3	MXD1	1.568720379
PLAG11	2.93E-10	0.1097436	0.042	0.011	7.06E-06	3	PLAG1	3.818181818
ANGPTL42	3.22E-10	0.1911312	0.071	0.025	7.76E-06	3	ANGPTL4	2.84
SDR16C52	3.68E-10	0.2957549	0.174	0.088	8.88E-06	3	SDR16C5	1.977272727
BORCS51	3.97E-10	0.221145	0.138	0.065	9.58E-06	3	BORCS5	2.123076923
SNRPG4	4.19E-10	0.4532118	0.632	0.516	1.01E-05	3	SNRPG	1.224806202
MRPL272	4.48E-10	0.2988864	0.297	0.183	1.08E-05	3	MRPL27	1.62295082
NUCB24	5.50E-10	0.3096356	0.226	0.128	1.33E-05	3	NUCB2	1.765625
CTPS23	5.74E-10	0.2990546	0.142	0.07	1.38E-05	3	CTPS2	2.028571429

UBE2L34	5.80E-10	0.3383107	0.567	0.437	1.40E-05	3	UBE2L3	1.297482838
ZNF1073	6.75E-10	0.2421717	0.176	0.092	1.63E-05	3	ZNF107	1.913043478
ADAMTS172	7.31E-10	0.1494898	0.075	0.027	1.76E-05	3	ADAMTS17	2.777777778
SLC7A26	7.83E-10	0.2927154	0.446	0.299	1.89E-05	3	SLC7A2	1.491638796
HAT13	8.82E-10	0.2823891	0.31	0.192	2.13E-05	3	HAT1	1.614583333
MORF4L25	8.88E-10	0.3287782	0.814	0.741	2.14E-05	3	MORF4L2	1.09851552
DDHD12	1.16E-09	0.1593418	0.096	0.04	2.80E-05	3	DDHD1	2.4
HMGCS13	1.26E-09	0.6308706	0.345	0.239	3.03E-05	3	HMGCS1	1.443514644
C20orf2031	1.31E-09	0.4031852	0.04	0.01	3.17E-05	3	C20orf203	4
C9orf843	1.51E-09	0.2932654	0.136	0.067	3.65E-05	3	C9orf84	2.029850746
FHL25	1.68E-09	0.3178681	0.276	0.171	4.05E-05	3	FHL2	1.614035088
RPL36AL3	1.72E-09	0.250821	0.916	0.886	4.15E-05	3	RPL36AL	1.033860045
CTD-2626G11.24	1.82E-09	0.2449353	0.105	0.046	4.38E-05	3	CTD-2626G	2.282608696
C4orf34	1.87E-09	0.3893091	0.644	0.534	4.51E-05	3	C4orf3	1.205992509
FAM69A3	1.96E-09	0.4061201	0.172	0.091	4.72E-05	3	FAM69A	1.89010989
PBX34	2.19E-09	0.298715	0.169	0.089	5.28E-05	3	PBX3	1.898876404
MTHFD25	2.49E-09	0.2813255	0.224	0.13	5.99E-05	3	MTHFD2	1.723076923
MMP102	2.63E-09	0.1980465	0.029	0.006	6.35E-05	3	MMP10	4.833333333
RALGPS23	3.05E-09	0.2742559	0.427	0.294	7.35E-05	3	RALGPS2	1.452380952
CTD-2015H6.33	3.06E-09	0.2015472	0.132	0.064	7.37E-05	3	CTD-2015H	2.0625
YIPF62	3.08E-09	0.3122507	0.293	0.185	7.43E-05	3	YIPF6	1.583783784
IRX33	3.64E-09	0.2605301	0.169	0.09	8.79E-05	3	IRX3	1.877777778
EPS15L13	3.76E-09	0.244213	0.157	0.082	9.06E-05	3	EPS15L1	1.914634146
ATP6V1E12	3.91E-09	0.3177275	0.414	0.292	9.42E-05	3	ATP6V1E1	1.417808219
EMC35	4.02E-09	0.2616403	0.356	0.235	9.68E-05	3	EMC3	1.514893617
LRRC67	4.83E-09	0.194421	0.1	0.044	0.000116561	3	LRRC6	2.272727273
SLC31A12	4.93E-09	0.215938	0.218	0.125	0.00011888	3	SLC31A1	1.744
IGBP13	5.01E-09	0.2988923	0.523	0.394	0.000120892	3	IGBP1	1.327411168
TAF123	6.49E-09	0.2166702	0.188	0.104	0.000156423	3	TAF12	1.807692308
RUFY33	6.95E-09	0.2782766	0.362	0.238	0.000167521	3	RUFY3	1.521008403
NBN2	7.31E-09	0.2166614	0.111	0.051	0.000176171	3	NBN	2.176470588
PON23	7.92E-09	0.2022653	0.172	0.092	0.000191051	3	PON2	1.869565217
MREG4	8.24E-09	0.2442213	0.238	0.14	0.000198598	3	MREG	1.7
LLPH4	8.33E-09	0.2286305	0.213	0.124	0.000200869	3	LLPH	1.717741935
ELP24	8.43E-09	0.3176384	0.322	0.214	0.000203251	3	ELP2	1.504672897
RP11-519G16.33	8.73E-09	0.3213714	0.649	0.507	0.000210424	3	RP11-519G	1.280078895
ACADSB6	9.83E-09	0.3148348	0.312	0.202	0.000236985	3	ACADSB	1.544554455
MIPOL15	1.07E-08	0.183207	0.297	0.183	0.000257358	3	MIPOL1	1.62295082
HES14	1.30E-08	0.4210744	0.458	0.341	0.000313338	3	HES1	1.343108504
PLIN23	1.41E-08	0.662403	0.169	0.094	0.0003388	3	PLIN2	1.79787234
LIMA12	1.73E-08	0.3467739	0.519	0.398	0.000417065	3	LIMA1	1.304020101
SCD3	1.84E-08	0.2587055	0.211	0.124	0.000443064	3	SCD	1.701612903
SRP145	1.99E-08	0.2562712	0.923	0.873	0.000480929	3	SRP14	1.057273769
GPR137B3	2.05E-08	0.1349222	0.098	0.043	0.000495361	3	GPR137B	2.279069767
MPHOSPH63	2.35E-08	0.2646944	0.215	0.128	0.000566478	3	MPHOSPH6	1.6796875
TTF14	2.42E-08	0.2109285	0.201	0.115	0.000584585	3	TTF1	1.747826087
TXNRD13	2.64E-08	0.3322427	0.506	0.376	0.00063741	3	TXNRD1	1.345744681
ASPH5	3.11E-08	0.308407	0.4	0.284	0.000748742	3	ASPH	1.408450704
CLOCK3	3.33E-08	0.2912256	0.31	0.207	0.000802528	3	CLOCK	1.497584541
CPB13	3.53E-08	0.3880019	0.029	0.007	0.000851774	3	CPB1	4.142857143
RP11-597D13.92	3.73E-08	0.102392	0.025	0.006	0.000899323	3	RP11-597D	4.166666667
IFT882	3.95E-08	0.236081	0.121	0.06	0.000951606	3	IFT88	2.016666667

MTIF34	4.21E-08	0.2776721	0.381	0.271	0.001014185	3	MTIF3	1.405904059
ADCY11	4.25E-08	0.1220474	0.05	0.017	0.00102577	3	ADCY1	2.941176471
PRKAA13	4.33E-08	0.253753	0.314	0.207	0.001043963	3	PRKAA1	1.516908213
CHD1L1	5.91E-08	0.2218937	0.103	0.048	0.001425577	3	CHD1L	2.145833333
AIM14	7.56E-08	0.2656703	0.575	0.445	0.001822251	3	AIM1	1.292134831
FBXO363	8.21E-08	0.1552675	0.111	0.053	0.00198005	3	FBXO36	2.094339623
DNAJB112	8.69E-08	0.2953088	0.278	0.183	0.002095114	3	DNAJB11	1.519125683
MAML33	9.14E-08	0.2387307	0.469	0.35	0.002203055	3	MAML3	1.34
B4GALNT31	9.58E-08	0.1559269	0.092	0.042	0.002309871	3	B4GALNT3	2.19047619
ZMYND83	1.08E-07	0.3981114	0.441	0.335	0.002594122	3	ZMYND8	1.31641791
LIMCH14	1.27E-07	0.2837365	0.623	0.486	0.003065457	3	LIMCH1	1.281893004
CH17-189H20.12	1.30E-07	0.2157256	0.163	0.092	0.003137489	3	CH17-189H	1.77173913
AFF13	1.35E-07	0.255353	0.494	0.373	0.00326073	3	AFF1	1.324396783
STARD134	1.46E-07	0.3342088	0.299	0.201	0.003508881	3	STARD13	1.487562189
MPP75	1.56E-07	0.2335357	0.199	0.116	0.003758346	3	MPP7	1.715517241
CCDC643	1.60E-07	0.1971247	0.111	0.055	0.00384897	3	CCDC64	2.018181818
SRRM12	1.70E-07	0.3193697	0.634	0.535	0.004104553	3	SRRM1	1.185046729
CROT3	1.73E-07	0.1386174	0.096	0.045	0.004172859	3	CROT	2.133333333
PLOD23	1.78E-07	0.4598724	0.335	0.238	0.004289988	3	PLOD2	1.407563025
POLR2K3	1.83E-07	0.243487	0.498	0.368	0.004405182	3	POLR2K	1.35326087
LYRM13	2.04E-07	0.1995339	0.161	0.091	0.004910945	3	LYRM1	1.769230769
HOXA91	2.04E-07	0.1193376	0.067	0.027	0.004926931	3	HOXA9	2.481481481
ALB1	2.18E-07	0.2393225	0.017	0.003	0.005248672	3	ALB	5.666666667
SLC7A62	2.36E-07	0.1431426	0.073	0.031	0.005682682	3	SLC7A6	2.35483871
GOLT1B2	2.68E-07	0.223523	0.228	0.143	0.006451798	3	GOLT1B	1.594405594
SBNO14	3.15E-07	0.2340174	0.238	0.151	0.007586937	3	SBNO1	1.57615894
COX203	3.32E-07	0.2397159	0.218	0.135	0.00800063	3	COX20	1.614814815
HPS33	3.98E-07	0.1562586	0.092	0.043	0.009588421	3	HPS3	2.139534884
HIST1H4H4	4.05E-07	0.2802326	0.138	0.076	0.009766715	3	HIST1H4H	1.815789474
EIF4G34	4.14E-07	0.1846084	0.64	0.51	0.009977274	3	EIF4G3	1.254901961
COPB21	4.43E-07	0.2524084	0.285	0.189	0.01069101	3	COPB2	1.507936508
DLC13	4.47E-07	0.2198815	0.123	0.065	0.01079054	3	DLC1	1.892307692
TTC39B4	4.53E-07	0.2133965	0.159	0.091	0.01093414	3	TTC39B	1.747252747
PLD14	4.66E-07	0.3305363	0.207	0.129	0.01122779	3	PLD1	1.604651163
ACER23	5.01E-07	0.1951554	0.098	0.048	0.01207061	3	ACER2	2.041666667
ZG16B3	5.29E-07	0.1255303	0.061	0.024	0.01276448	3	ZG16B	2.541666667
C9orf1524	5.94E-07	0.256523	0.096	0.047	0.01433341	3	C9orf152	2.042553191
BZW14	6.13E-07	0.1711991	0.395	0.276	0.01479285	3	BZW1	1.43115942
FGFR13	6.36E-07	0.2832075	0.285	0.192	0.01533646	3	FGFR1	1.484375
DPCD2	6.38E-07	0.1823098	0.149	0.083	0.01538693	3	DPCD	1.795180723
HN1L2	6.42E-07	0.1738847	0.14	0.077	0.01547631	3	HN1L	1.818181818
ARPC34	6.53E-07	0.2681822	0.745	0.66	0.01575377	3	ARPC3	1.128787879
CFB3	6.93E-07	0.4967496	0.232	0.154	0.01671426	3	CFB	1.506493506
IFITM22	7.57E-07	0.4678666	0.146	0.083	0.01825645	3	IFITM2	1.759036145
EFHD16	7.98E-07	0.1908389	0.423	0.299	0.01923913	3	EFHD1	1.414715719
SS18L23	9.43E-07	0.1836037	0.228	0.144	0.02273373	3	SS18L2	1.583333333
SHFM13	9.48E-07	0.2908737	0.713	0.626	0.02286485	3	SHFM1	1.138977636
CPD4	1.06E-06	0.1748376	0.349	0.239	0.02554034	3	CPD	1.460251046
POMP4	1.11E-06	0.2149592	0.738	0.63	0.02673693	3	POMP	1.171428571
CAPN134	1.22E-06	0.1256999	0.082	0.038	0.02953169	3	CAPN13	2.157894737
ZNF1002	1.24E-06	0.1199986	0.077	0.035	0.02983723	3	ZNF100	2.2
CA25	1.43E-06	0.2225823	0.157	0.091	0.03452048	3	CA2	1.725274725

PRELID3B2	1.44E-06	0.2546262	0.335	0.239	0.03467763	3	PRELID3B	1.40167364
CENPP1	1.52E-06	0.2412666	0.113	0.06	0.03662691	3	CENPP	1.883333333
ACTL6A3	1.64E-06	0.154887	0.126	0.068	0.03963308	3	ACTL6A	1.852941176
TNIK5	1.69E-06	0.1340554	0.249	0.161	0.04073089	3	TNIK	1.546583851
DUSP55	1.91E-06	0.1804408	0.343	0.237	0.04605012	3	DUSP5	1.447257384
SRGN1	0	4.623162	0.589	0.023	0	0	SRGN	25.60869565
IL1B	0	3.137399	0.212	0.002	0	0	IL1B	106
FCER1G	0	3.01144	0.356	0.002	0	0	FCER1G	178
RGS1	0	2.727623	0.288	0.004	0	0	RGS1	72
CYTIP2	0	2.439028	0.356	0.007	0	0	CYTIP	50.85714286
TCF4	0	2.38423	0.384	0.007	0	0	TCF4	54.85714286
ZEB2	0	2.296144	0.315	0.001	0	0	ZEB2	315
HLA-DQA11	0	2.181689	0.329	0.005	0	0	HLA-DQA1	65.8
GPR183	0	1.753111	0.26	0.002	0	0	GPR183	130
FGL2	0	1.744689	0.199	0	0	0	FGL2	#DIV/0!
PTPRC	0	1.601916	0.199	0.001	0	0	PTPRC	199
CD53	0	1.481391	0.226	0.001	0	0	CD53	226
HLA-DPB1	0	1.37406	0.253	0.004	0	0	HLA-DPB1	63.25
HLA-DPA1	0	1.269555	0.205	0.002	0	0	HLA-DPA1	102.5
NRP2	0	1.26448	0.226	0.002	0	0	NRP2	113
PLEK	0	1.040968	0.116	0	0	0	PLEK	#DIV/0!
OLR1	0	0.6824914	0.096	0	0	0	OLR1	#DIV/0!
GIMAP4	0	0.6468897	0.082	0	0	0	GIMAP4	#DIV/0!
MS4A6A	0	0.5735986	0.123	0	0	0	MS4A6A	#DIV/0!
CD93	0	0.4492098	0.075	0	0	0	CD93	#DIV/0!
MMP19	0	0.3495196	0.082	0	0	0	MMP19	#DIV/0!
PIK3R5	1.04E-303	1.52811	0.199	0.003	2.50E-299	0	PIK3R5	66.33333333
A2M	3.25E-292	1.3512	0.205	0.003	7.83E-288	0	A2M	68.33333333
CD163	2.57E-280	0.7423169	0.096	0	6.20E-276	0	CD163	#DIV/0!
SLC2A31	1.74E-276	1.532577	0.205	0.003	4.21E-272	0	SLC2A3	68.33333333
CLECL1	6.57E-271	0.449916	0.082	0	1.58E-266	0	CLECL1	#DIV/0!
HLA-DRB51	7.77E-265	1.40195	0.267	0.006	1.87E-260	0	HLA-DRB5	44.5
CHST111	8.57E-253	1.574951	0.308	0.009	2.07E-248	0	CHST11	34.22222222
ENTPD1	8.75E-253	1.15992	0.219	0.004	2.11E-248	0	ENTPD1	54.75
LAPTM5	1.46E-240	0.5412252	0.082	0	3.52E-236	0	LAPTM5	#DIV/0!
CXorf21	6.40E-232	0.3053523	0.068	0	1.54E-227	0	CXorf21	#DIV/0!
C5AR11	5.41E-227	1.105747	0.205	0.004	1.31E-222	0	C5AR1	51.25
RP11-556E13.1	9.35E-226	1.683701	0.185	0.003	2.26E-221	0	RP11-556E	61.66666667
TLR4	4.23E-202	0.5944297	0.075	0	1.02E-197	0	TLR4	#DIV/0!
IL2RG	5.34E-202	0.4313662	0.075	0	1.29E-197	0	IL2RG	#DIV/0!
ATP8B42	3.60E-194	2.082921	0.267	0.009	8.67E-190	0	ATP8B4	29.66666667
CSF2RA3	1.33E-188	2.208554	0.336	0.016	3.21E-184	0	CSF2RA	21
FCGR2A	1.94E-186	0.7806454	0.171	0.004	4.68E-182	0	FCGR2A	42.75
GIMAP7	4.73E-182	0.369346	0.041	0	1.14E-177	0	GIMAP7	#DIV/0!
EVI2B	4.73E-182	0.2082456	0.041	0	1.14E-177	0	EVI2B	#DIV/0!
CD80	1.71E-180	0.6324722	0.089	0.001	4.13E-176	0	CD80	89
HLA-DRA2	4.21E-169	4.784841	0.671	0.078	1.02E-164	0	HLA-DRA	8.602564103
ALOX5AP	1.93E-168	1.039507	0.164	0.004	4.64E-164	0	ALOX5AP	41
EPB41L31	2.60E-168	2.089122	0.315	0.015	6.26E-164	0	EPB41L3	21
IL7R	5.68E-167	1.112889	0.068	0	1.37E-162	0	IL7R	#DIV/0!
CD300E	4.45E-158	0.5417141	0.068	0	1.07E-153	0	CD300E	#DIV/0!
TYROBP	5.29E-158	0.4838393	0.068	0	1.27E-153	0	TYROBP	#DIV/0!

HLA-DRB12	5.44E-155	2.480785	0.514	0.046	1.31E-150	0	HLA-DRB1	11.17391304
SAMSN13	1.75E-154	2.617716	0.363	0.023	4.21E-150	0	SAMSN1	15.7826087
SLAMF7	4.82E-152	0.3180138	0.034	0	1.16E-147	0	SLAMF7	#DIV/0!
NLRP3	1.75E-148	0.3379132	0.055	0	4.21E-144	0	NLRP3	#DIV/0!
GNG11	1.17E-134	0.4452416	0.048	0	2.83E-130	0	GNG11	#DIV/0!
FAM49A4	1.64E-133	1.716737	0.384	0.029	3.95E-129	0	FAM49A	13.24137931
CD744	2.11E-130	2.797706	0.616	0.084	5.08E-126	0	CD74	7.333333333
MSR1	7.12E-128	1.293163	0.219	0.01	1.72E-123	0	MSR1	21.9
FCGR3A	1.09E-126	0.2472296	0.034	0	2.63E-122	0	FCGR3A	#DIV/0!
NCKAP1L	1.56E-121	0.3432529	0.062	0.001	3.77E-117	0	NCKAP1L	62
PLA2G7	1.18E-115	0.3352033	0.062	0.001	2.85E-111	0	PLA2G7	62
LAIR1	3.15E-113	0.2477091	0.055	0	7.59E-109	0	LAIR1	#DIV/0!
LUM	4.72E-109	0.7593702	0.041	0	1.14E-104	0	LUM	#DIV/0!
LILRB1	4.92E-109	0.234808	0.041	0	1.19E-104	0	LILRB1	#DIV/0!
CD86	7.08E-107	0.6932559	0.13	0.004	1.71E-102	0	CD86	32.5
DCN	1.28E-105	0.9658774	0.048	0	3.09E-101	0	DCN	#DIV/0!
IL2RA1	1.32E-103	1.050096	0.178	0.008	3.19E-99	0	IL2RA	22.25
SLC1A32	2.97E-101	1.946289	0.322	0.027	7.17E-97	0	SLC1A3	11.92592593
CCL4L21	3.31E-101	0.8632973	0.055	0.001	7.99E-97	0	CCL4L2	55
LST11	4.85E-101	0.3254388	0.062	0.001	1.17E-96	0	LST1	62
IRAK33	8.65E-100	1.524381	0.267	0.019	2.09E-95	0	IRAK3	14.05263158
MMP9	1.12E-97	0.1653317	0.027	0	2.71E-93	0	MMP9	#DIV/0!
WIPF12	5.60E-97	0.9587705	0.171	0.008	1.35E-92	0	WIPF1	21.375
SPARC	5.92E-97	0.8562066	0.123	0.004	1.43E-92	0	SPARC	30.75
LYZ1	8.75E-97	0.5478086	0.082	0.002	2.11E-92	0	LYZ	41
CCL32	1.16E-95	1.307506	0.089	0.002	2.79E-91	0	CCL3	44.5
NPL	2.50E-94	0.751061	0.144	0.006	6.04E-90	0	NPL	24
MPP1	3.85E-93	0.56235	0.11	0.003	9.28E-89	0	MPP1	36.66666667
COL1A2	5.29E-92	0.4325637	0.021	0	1.28E-87	0	COL1A2	#DIV/0!
C1QA	5.29E-92	0.2307629	0.021	0	1.28E-87	0	C1QA	#DIV/0!
FLI1	1.92E-88	0.4243697	0.082	0.002	4.64E-84	0	FLI1	41
PAG1	5.10E-88	0.9056181	0.13	0.005	1.23E-83	0	PAG1	26
SPARCL14	1.16E-85	3.087791	0.349	0.038	2.81E-81	0	SPARCL1	9.184210526
HLA-DQA2	7.55E-83	0.5083902	0.096	0.003	1.82E-78	0	HLA-DQA2	32
BCAT1	2.38E-82	0.6643369	0.11	0.004	5.73E-78	0	BCAT1	27.5
GPNMB2	3.78E-82	0.769718	0.137	0.006	9.11E-78	0	GPNMB	22.83333333
IGLC3	1.95E-81	2.48868	0.027	0	4.70E-77	0	IGLC3	#DIV/0!
MNDA	1.98E-81	0.2989229	0.027	0	4.78E-77	0	MNDA	#DIV/0!
CCR1	2.04E-81	0.1482718	0.027	0	4.92E-77	0	CCR1	#DIV/0!
SLAMF9	2.06E-81	0.1015458	0.027	0	4.96E-77	0	SLAMF9	#DIV/0!
LCP13	6.63E-76	0.9990032	0.205	0.015	1.60E-71	0	LCP1	13.66666667
ERG	9.10E-76	1.052224	0.144	0.007	2.19E-71	0	ERG	20.57142857
KCNK13	1.22E-72	0.3901474	0.082	0.002	2.93E-68	0	KCNK13	41
ITGAX1	8.52E-71	0.3192039	0.055	0.001	2.05E-66	0	ITGAX	55
IGSF6	9.63E-71	0.3127929	0.062	0.001	2.32E-66	0	IGSF6	62
COL3A1	7.76E-70	1.122259	0.027	0	1.87E-65	0	COL3A1	#DIV/0!
CCR7	3.71E-69	0.1613686	0.021	0	8.94E-65	0	CCR7	#DIV/0!
AIF1	6.19E-68	0.4836825	0.041	0	1.49E-63	0	AIF1	#DIV/0!
SERPINE1	3.77E-66	1.55742	0.144	0.008	9.08E-62	0	SERPINE1	18
PDE3B	4.32E-66	1.373634	0.158	0.01	1.04E-61	0	PDE3B	15.8
MS4A4A2	7.75E-66	0.545981	0.096	0.004	1.87E-61	0	MS4A4A	24
RHOH4	1.69E-65	1.735738	0.281	0.031	4.07E-61	0	RHOH	9.064516129

CTSL1	1.88E-64	1.508454	0.178	0.013	4.53E-60	0	CTSL	13.69230769
CCDC88A3	3.94E-64	1.438688	0.233	0.022	9.50E-60	0	CCDC88A	10.59090909
PRRX1	6.38E-64	0.3299639	0.048	0.001	1.54E-59	0	PRRX1	48
TIE1	6.87E-64	0.4100222	0.048	0.001	1.66E-59	0	TIE1	48
IL10RA	7.30E-64	0.246591	0.041	0.001	1.76E-59	0	IL10RA	41
ADAM283	3.91E-62	1.733708	0.212	0.019	9.43E-58	0	ADAM28	11.15789474
F2RL3	5.90E-62	0.1842318	0.014	0	1.42E-57	0	F2RL3	#DIV/0!
C1QB	5.90E-62	0.1414277	0.014	0	1.42E-57	0	C1QB	#DIV/0!
NPR1	5.90E-62	0.1200528	0.014	0	1.42E-57	0	NPR1	#DIV/0!
GPR132	1.06E-61	0.393378	0.089	0.003	2.55E-57	0	GPR132	29.66666667
RP11-325F22.21	2.56E-61	0.7983626	0.158	0.011	6.17E-57	0	RP11-325F2	14.36363636
PRKCB	2.83E-59	1.166009	0.137	0.008	6.82E-55	0	PRKCB	17.125
PLXNC1	4.75E-59	1.024141	0.144	0.009	1.15E-54	0	PLXNC1	16
RP11-624C23.12	5.70E-59	1.379504	0.205	0.019	1.37E-54	0	RP11-624C	10.78947368
CD834	2.70E-57	1.832541	0.39	0.067	6.52E-53	0	CD83	5.820895522
ARHGAP242	3.45E-57	1.403555	0.267	0.032	8.33E-53	0	ARHGAP24	8.34375
ELMO11	4.69E-57	1.200932	0.24	0.026	1.13E-52	0	ELMO1	9.230769231
ST8SIA4	1.98E-56	1.068268	0.185	0.016	4.77E-52	0	ST8SIA4	11.5625
LCP2	1.06E-55	0.5797818	0.13	0.008	2.56E-51	0	LCP2	16.25
MEG8	2.00E-55	0.1137132	0.021	0	4.83E-51	0	MEG8	#DIV/0!
PECAM12	2.51E-55	1.477854	0.144	0.01	6.05E-51	0	PECAM1	14.4
ABCA16	4.73E-55	1.084243	0.24	0.027	1.14E-50	0	ABCA1	8.888888889
RASSF21	6.59E-55	0.5176581	0.082	0.003	1.59E-50	0	RASSF2	27.33333333
DOCK82	2.07E-54	2.266053	0.356	0.06	5.00E-50	0	DOCK8	5.933333333
TCHH1	3.12E-54	0.3442945	0.041	0.001	7.53E-50	0	TCHH	41
RBMS3-AS32	3.94E-54	0.6623967	0.116	0.007	9.50E-50	0	RBMS3-AS3	16.57142857
CRLF21	3.99E-54	0.3386669	0.075	0.003	9.62E-50	0	CRLF2	25
ANKDD1A	7.20E-54	0.3707436	0.089	0.004	1.74E-49	0	ANKDD1A	22.25
CLIC2	2.52E-53	0.5191014	0.089	0.004	6.09E-49	0	CLIC2	22.25
PFKFB33	2.70E-53	1.412486	0.363	0.061	6.50E-49	0	PFKFB3	5.950819672
APBB1IP	9.41E-52	0.6504618	0.096	0.005	2.27E-47	0	APBB1IP	19.2
BMP6	1.02E-51	0.5114406	0.075	0.003	2.47E-47	0	BMP6	25
PTGIR	1.45E-51	0.1652682	0.041	0.001	3.50E-47	0	PTGIR	41
RAPGEF13	1.55E-51	1.759384	0.459	0.1	3.73E-47	0	RAPGEF1	4.59
VASH11	1.50E-50	0.1604921	0.034	0	3.62E-46	0	VASH1	#DIV/0!
SMAP23	1.63E-50	1.051325	0.233	0.028	3.94E-46	0	SMAP2	8.321428571
PIK3AP1	1.49E-49	0.6563428	0.137	0.01	3.58E-45	0	PIK3AP1	13.7
NR2F1	6.47E-49	0.1915636	0.027	0	1.56E-44	0	NR2F1	#DIV/0!
SLC16A101	9.24E-49	1.260486	0.219	0.025	2.23E-44	0	SLC16A10	8.76
ARHGAP312	9.67E-49	0.845516	0.164	0.015	2.33E-44	0	ARHGAP31	10.93333333
CALCRL1	8.02E-48	0.6765846	0.068	0.003	1.93E-43	0	CALCRL	22.66666667
SSH12	8.79E-48	0.8027503	0.226	0.027	2.12E-43	0	SSH1	8.37037037
SPP12	1.74E-47	2.698775	0.151	0.013	4.19E-43	0	SPP1	11.61538462
HCK1	1.79E-47	0.5815042	0.123	0.008	4.32E-43	0	HCK	15.375
SLC11A1	3.77E-47	0.3728033	0.048	0.001	9.08E-43	0	SLC11A1	48
RP11-286N3.21	4.45E-47	0.3938052	0.041	0.001	1.07E-42	0	RP11-286N	41
COL4A24	1.23E-46	1.093665	0.158	0.014	2.96E-42	0	COL4A2	11.28571429
RP11-426C22.5	1.28E-46	0.4166895	0.062	0.002	3.09E-42	0	RP11-426C	31
RBMS32	1.30E-46	1.560221	0.329	0.058	3.13E-42	0	RBMS3	5.672413793
TBXAS12	1.32E-46	1.042661	0.219	0.026	3.19E-42	0	TBXAS1	8.423076923
SLA	4.66E-46	0.8106108	0.11	0.007	1.12E-41	0	SLA	15.71428571
C3AR11	2.12E-45	0.59769	0.11	0.007	5.10E-41	0	C3AR1	15.71428571

MIR181A1HG1	4.25E-45	0.9033524	0.144	0.012	1.03E-40	0	MIR181A1H	12
RNASE61	5.08E-45	0.4847616	0.055	0.002	1.22E-40	0	RNASE6	27.5
KLHL6	1.18E-44	0.448682	0.068	0.003	2.84E-40	0	KLHL6	22.66666667
RP11-452H21.4	1.74E-44	0.1800327	0.027	0	4.20E-40	0	RP11-452H	#DIV/0!
HLA-DQB12	3.48E-44	0.5842193	0.123	0.009	8.39E-40	0	HLA-DQB1	13.66666667
IGLC2	1.30E-41	0.2862544	0.014	0	3.13E-37	0	IGLC2	#DIV/0!
RASGRP31	1.91E-41	0.8762162	0.192	0.023	4.61E-37	0	RASGRP3	8.347826087
CTD-2282P23.21	6.62E-41	0.2323998	0.055	0.002	1.60E-36	0	CTD-2282P	27.5
ACKR3	8.75E-41	0.3075007	0.027	0	2.11E-36	0	ACKR3	#DIV/0!
KCNQ32	1.69E-40	1.270045	0.219	0.03	4.08E-36	0	KCNQ3	7.3
ADGRF5	4.06E-40	0.7230778	0.062	0.002	9.80E-36	0	ADGRF5	31
MEF2C2	5.31E-40	0.8776707	0.158	0.016	1.28E-35	0	MEF2C	9.875
WIF1	5.32E-40	0.317968	0.041	0.001	1.28E-35	0	WIF1	41
SLC7A7	2.13E-39	0.5315748	0.123	0.01	5.14E-35	0	SLC7A7	12.3
COL4A11	2.16E-39	0.6898138	0.082	0.005	5.20E-35	0	COL4A1	16.4
DOCK43	2.70E-39	2.845021	0.692	0.311	6.51E-35	0	DOCK4	2.225080386
RFTN1	3.02E-39	0.3985855	0.096	0.006	7.29E-35	0	RFTN1	16
GK2	8.16E-38	1.187664	0.281	0.05	1.97E-33	0	GK	5.62
GMFG	1.86E-37	0.3457359	0.075	0.004	4.48E-33	0	GMFG	18.75
PLEKHO1	3.40E-37	0.3365602	0.082	0.005	8.19E-33	0	PLEKHO1	16.4
PTGS22	3.78E-37	1.005435	0.13	0.012	9.12E-33	0	PTGS2	10.83333333
ARHGEF151	5.44E-36	0.2562457	0.034	0.001	1.31E-31	0	ARHGEF15	34
MMP3	2.01E-35	2.516542	0.048	0.002	4.84E-31	0	MMP3	24
MCOLN21	2.18E-35	0.4101515	0.048	0.002	5.25E-31	0	MCOLN2	24
CD2002	5.10E-35	0.4536468	0.062	0.003	1.23E-30	0	CD200	20.66666667
FMNL31	1.47E-34	0.6573433	0.151	0.017	3.54E-30	0	FMNL3	8.882352941
ARHGAP25	4.13E-34	0.4076188	0.068	0.004	9.96E-30	0	ARHGAP25	17
GPSM31	4.90E-34	0.3077186	0.055	0.002	1.18E-29	0	GPSM3	27.5
S1PR11	1.20E-33	0.2302718	0.041	0.001	2.90E-29	0	S1PR1	41
LPAR62	1.53E-33	1.166356	0.322	0.071	3.70E-29	0	LPAR6	4.535211268
MYLK	1.88E-33	2.122999	0.151	0.018	4.53E-29	0	MYLK	8.388888889
FYN3	6.03E-33	1.162095	0.185	0.026	1.45E-28	0	FYN	7.115384615
CNRIP1	7.67E-33	0.1591383	0.034	0.001	1.85E-28	0	CNRIP1	34
SLAMF1	1.27E-32	0.1503137	0.027	0.001	3.06E-28	0	SLAMF1	27
INHBA2	1.51E-32	0.5897411	0.089	0.007	3.64E-28	0	INHBA	12.71428571
TFEC1	1.66E-32	1.000492	0.171	0.023	4.00E-28	0	TFEC	7.434782609
PKD22	4.18E-32	1.533046	0.274	0.056	1.01E-27	0	PKD2	4.892857143
FAM78A	7.54E-32	0.169909	0.007	0	1.82E-27	0	FAM78A	#DIV/0!
CORO1A	7.54E-32	0.169909	0.007	0	1.82E-27	0	CORO1A	#DIV/0!
CD2	7.54E-32	0.1203927	0.007	0	1.82E-27	0	CD2	#DIV/0!
GIMAP6	7.54E-32	0.1203927	0.007	0	1.82E-27	0	GIMAP6	#DIV/0!
RAB202	2.35E-31	0.9055876	0.164	0.022	5.68E-27	0	RAB20	7.454545455
AMPD32	2.96E-31	1.202187	0.219	0.037	7.15E-27	0	AMPD3	5.918918919
PIK3CG	5.30E-31	0.1546785	0.021	0	1.28E-26	0	PIK3CG	#DIV/0!
PDE4A2	7.82E-31	0.5465017	0.116	0.011	1.88E-26	0	PDE4A	10.54545455
DOCK10	9.39E-31	0.8163895	0.144	0.017	2.26E-26	0	DOCK10	8.470588235
TNFRSF1B3	1.30E-30	0.9852007	0.212	0.036	3.15E-26	0	TNFRSF1B	5.888888889
POSTN	1.32E-30	0.458529	0.027	0.001	3.18E-26	0	POSTN	27
ESAM	1.33E-30	0.2711338	0.027	0.001	3.20E-26	0	ESAM	27
VCAN	1.37E-30	0.196875	0.027	0.001	3.30E-26	0	VCAN	27
RIN33	1.48E-30	0.8756253	0.164	0.022	3.56E-26	0	RIN3	7.454545455
FTL4	4.35E-30	2.259114	0.801	0.53	1.05E-25	0	FTL	1.511320755

NR5A2	7.11E-30	0.3450051	0.062	0.003	1.71E-25	0	NR5A2	20.66666667
NEAT16	1.40E-29	1.147334	0.986	0.947	3.38E-25	0	NEAT1	1.041182682
MGAT11	1.79E-29	0.4784487	0.116	0.012	4.32E-25	0	MGAT1	9.666666667
EPDR1	8.51E-29	0.146679	0.027	0.001	2.05E-24	0	EPDR1	27
ITGA4	8.64E-29	0.4081528	0.027	0.001	2.08E-24	0	ITGA4	27
LMOD1	1.41E-28	0.4435113	0.055	0.003	3.40E-24	0	LMOD1	18.33333333
ACSL44	2.69E-28	1.251873	0.253	0.052	6.49E-24	0	ACSL4	4.865384615
PLXDC22	3.46E-28	1.986135	0.397	0.122	8.35E-24	0	PLXDC2	3.254098361
WNT2B3	5.01E-28	1.211219	0.219	0.041	1.21E-23	0	WNT2B	5.341463415
CD841	6.13E-28	0.1049725	0.021	0	1.48E-23	0	CD84	#DIV/0!
ACTA2	1.68E-27	2.407696	0.11	0.012	4.05E-23	0	ACTA2	9.166666667
ARHGAP61	3.97E-27	0.5340699	0.082	0.007	9.58E-23	0	ARHGAP6	11.71428571
PILRA1	5.45E-27	0.6871539	0.158	0.023	1.31E-22	0	PILRA	6.869565217
ADGRB31	1.04E-26	0.7626263	0.13	0.016	2.52E-22	0	ADGRB3	8.125
CEP1702	2.20E-26	1.074999	0.288	0.071	5.30E-22	0	CEP170	4.056338028
FMNL24	2.50E-25	1.798102	0.658	0.351	6.03E-21	0	FMNL2	1.874643875
CLEC2B4	2.58E-25	1.750883	0.267	0.063	6.22E-21	0	CLEC2B	4.238095238
MIR155HG2	1.11E-24	0.853199	0.096	0.01	2.67E-20	0	MIR155HG	9.6
MME	1.55E-24	0.266956	0.055	0.003	3.74E-20	0	MME	18.33333333
ARHGAP201	2.96E-24	0.1441005	0.034	0.001	7.13E-20	0	ARHGAP20	34
COL5A2	2.98E-24	0.6521107	0.041	0.002	7.20E-20	0	COL5A2	20.5
C10orf112	3.06E-24	0.9259259	0.199	0.038	7.39E-20	0	C10orf11	5.236842105
CXCL102	7.33E-24	1.008745	0.062	0.004	1.77E-19	0	CXCL10	15.5
JAM3	1.04E-23	0.3152521	0.041	0.002	2.52E-19	0	JAM3	20.5
GPX31	1.16E-23	0.1776373	0.041	0.002	2.80E-19	0	GPX3	20.5
AXL1	1.17E-23	0.7671336	0.137	0.02	2.83E-19	0	AXL	6.85
TCF4-AS1	1.79E-23	0.1307058	0.034	0.001	4.30E-19	0	TCF4-AS1	34
NID1	2.06E-23	0.4109503	0.048	0.003	4.96E-19	0	NID1	16
SPRY1	4.09E-23	0.5715788	0.068	0.006	9.87E-19	0	SPRY1	11.33333333
CIITA1	5.72E-23	0.3954786	0.068	0.006	1.38E-18	0	CIITA	11.33333333
SOBP	8.48E-23	0.2855423	0.034	0.001	2.05E-18	0	SOBP	34
HAVCR23	8.74E-23	0.8509713	0.192	0.038	2.11E-18	0	HAVCR2	5.052631579
RUNX1T1	2.99E-22	0.2729286	0.027	0.001	7.21E-18	0	RUNX1T1	27
MERTK3	3.86E-22	0.5116217	0.089	0.009	9.31E-18	0	MERTK	9.888888889
PCAT192	4.39E-22	0.5697776	0.055	0.004	1.06E-17	0	PCAT19	13.75
DSE2	1.30E-21	1.017214	0.192	0.04	3.14E-17	0	DSE	4.8
ADGRE53	1.43E-21	0.4602911	0.116	0.016	3.45E-17	0	ADGRE5	7.25
LINC009961	1.83E-21	0.2175198	0.034	0.001	4.41E-17	0	LINC00996	34
VIM4	2.60E-21	1.177039	0.363	0.123	6.28E-17	0	VIM	2.951219512
CAV12	2.77E-21	1.6507	0.164	0.031	6.69E-17	0	CAV1	5.290322581
COL15A11	2.85E-21	0.5733443	0.041	0.002	6.88E-17	0	COL15A1	20.5
NID2	2.86E-21	0.17244	0.027	0.001	6.90E-17	0	NID2	27
ACTG22	3.76E-21	1.685114	0.089	0.01	9.06E-17	0	ACTG2	8.9
AFAP1L1	6.37E-21	0.383483	0.048	0.003	1.54E-16	0	AFAP1L1	16
JCHAIN	7.21E-21	4.236802	0.034	0.002	1.74E-16	0	JCHAIN	17
OSCAR	8.41E-21	0.1418919	0.034	0.002	2.03E-16	0	OSCAR	17
PTK2B3	9.38E-21	1.231925	0.178	0.036	2.26E-16	0	PTK2B	4.944444444
FABP51	1.80E-20	0.585327	0.062	0.005	4.34E-16	0	FABP5	12.4
SH3BP53	2.17E-20	0.8166075	0.226	0.055	5.24E-16	0	SH3BP5	4.109090909
TWIST1	2.21E-20	0.1406694	0.027	0.001	5.32E-16	0	TWIST1	27
ARHGAP9	2.23E-20	0.1443453	0.027	0.001	5.38E-16	0	ARHGAP9	27
TGFBI3	2.37E-20	1.207253	0.151	0.027	5.72E-16	0	TGFBI	5.592592593

HLA-DMB1	3.16E-20	0.1655348	0.034	0.002	7.62E-16	0	HLA-DMB	17
P2RX74	4.02E-20	0.5296575	0.116	0.017	9.69E-16	0	P2RX7	6.823529412
FCHSD24	4.85E-20	1.357641	0.363	0.13	1.17E-15	0	FCHSD2	2.792307692
C1orf54	6.98E-20	0.4423489	0.103	0.014	1.68E-15	0	C1orf54	7.357142857
PTPN15	7.98E-20	0.962842	0.205	0.047	1.92E-15	0	PTPN1	4.361702128
JAM2	1.13E-19	0.2998796	0.048	0.003	2.73E-15	0	JAM2	16
BASP13	1.15E-19	1.033946	0.219	0.053	2.78E-15	0	BASP1	4.132075472
ST6GALNAC31	1.71E-19	1.036849	0.11	0.016	4.13E-15	0	ST6GALNA(	6.875
ABCA62	2.42E-19	0.2951862	0.048	0.003	5.84E-15	0	ABCA6	16
RP11-44K6.31	2.64E-19	0.2878058	0.082	0.009	6.37E-15	0	RP11-44K6	9.111111111
NAV13	2.78E-19	1.014246	0.205	0.049	6.69E-15	0	NAV1	4.183673469
PHACTR1	3.32E-19	0.7734954	0.144	0.026	8.02E-15	0	PHACTR1	5.538461538
ARMC93	3.71E-19	1.399129	0.322	0.105	8.95E-15	0	ARMC9	3.066666667
PDPN	8.27E-19	0.1664752	0.027	0.001	1.99E-14	0	PDPN	27
PELI14	9.34E-19	1.954974	0.596	0.328	2.25E-14	0	PELI1	1.817073171
IL62	1.48E-18	0.4364153	0.041	0.003	3.57E-14	0	IL6	13.66666667
RNASE12	1.72E-18	0.8372194	0.123	0.02	4.14E-14	0	RNASE1	6.15
GK-AS11	2.10E-18	0.4437532	0.075	0.008	5.07E-14	0	GK-AS1	9.375
RASSF83	2.63E-18	0.9533654	0.315	0.105	6.34E-14	0	RASSF8	3
FAM126A3	2.89E-18	1.053189	0.151	0.029	6.97E-14	0	FAM126A	5.206896552
IL4I11	3.16E-18	0.2536986	0.055	0.005	7.61E-14	0	IL4I1	11
IGF11	4.04E-18	0.3003353	0.027	0.001	9.73E-14	0	IGF1	27
SLC8A11	4.11E-18	0.9271982	0.103	0.015	9.91E-14	0	SLC8A1	6.866666667
CCDC168	4.17E-18	0.1196551	0.027	0.001	1.00E-13	0	CCDC168	27
EBF12	4.34E-18	0.750418	0.11	0.017	1.05E-13	0	EBF1	6.470588235
GYPC3	4.37E-18	0.4730404	0.075	0.008	1.05E-13	0	GYPC	9.375
RUNX33	4.77E-18	0.3074035	0.055	0.005	1.15E-13	0	RUNX3	11
AC069368.3	6.84E-18	0.4939217	0.082	0.01	1.65E-13	0	AC069368.:	8.2
LAMA41	6.99E-18	0.5672103	0.055	0.005	1.68E-13	0	LAMA4	11
CREG13	1.10E-17	0.9497088	0.205	0.051	2.64E-13	0	CREG1	4.019607843
VOPP14	3.16E-17	0.8304441	0.247	0.071	7.61E-13	0	VOPP1	3.478873239
RUNX23	3.37E-17	0.733262	0.13	0.024	8.13E-13	0	RUNX2	5.416666667
CCL203	3.96E-17	2.986833	0.411	0.183	9.54E-13	0	CCL20	2.245901639
ARHGAP182	5.39E-17	0.986316	0.253	0.075	1.30E-12	0	ARHGAP18	3.373333333
CPVL1	6.97E-17	0.2686143	0.055	0.005	1.68E-12	0	CPVL	11
GNG2	7.23E-17	0.4636938	0.068	0.007	1.74E-12	0	GNG2	9.714285714
AFF14	9.57E-17	1.150782	0.616	0.374	2.31E-12	0	AFF1	1.647058824
PDE4DIP2	1.02E-16	0.8652836	0.233	0.066	2.45E-12	0	PDE4DIP	3.53030303
ANKRD443	1.15E-16	1.247018	0.253	0.077	2.78E-12	0	ANKRD44	3.285714286
MB21D23	1.41E-16	0.9548548	0.295	0.096	3.41E-12	0	MB21D2	3.072916667
TNFRSF91	1.43E-16	0.148222	0.034	0.002	3.45E-12	0	TNFRSF9	17
IGHA1	1.65E-16	3.323306	0.007	0	3.98E-12	0	IGHA1	#DIV/0!
CEP682	1.83E-16	0.5871631	0.164	0.037	4.42E-12	0	CEP68	4.432432432
RP11-290H9.22	2.67E-16	0.4366704	0.068	0.008	6.44E-12	0	RP11-290H	8.5
CCL8	2.71E-16	0.4854161	0.027	0.001	6.53E-12	0	CCL8	27
SCARF1	2.75E-16	0.1207059	0.027	0.001	6.64E-12	0	SCARF1	27
SLCO2B12	2.92E-16	0.4458755	0.062	0.006	7.04E-12	0	SLCO2B1	10.33333333
KB-1507C5.42	3.07E-16	0.4640759	0.062	0.006	7.40E-12	0	KB-1507C5	10.33333333
SDCCAG83	3.08E-16	0.9576543	0.37	0.149	7.41E-12	0	SDCCAG8	2.483221477
CLMP	3.92E-16	1.05427	0.103	0.016	9.46E-12	0	CLMP	6.4375
THBD1	4.00E-16	0.2886436	0.041	0.003	9.64E-12	0	THBD	13.66666667
ADGRL4	4.14E-16	0.5666408	0.048	0.004	9.99E-12	0	ADGRL4	12

ROBO12	4.45E-16	0.8706276	0.233	0.068	1.07E-11	0	ROBO1	3.426470588
RGL12	4.54E-16	0.8896788	0.199	0.052	1.09E-11	0	RGL1	3.826923077
MCTP12	4.73E-16	1.275663	0.288	0.099	1.14E-11	0	MCTP1	2.909090909
IL15RA3	5.76E-16	0.6046198	0.151	0.032	1.39E-11	0	IL15RA	4.71875
PPP1R16B	6.79E-16	0.5584443	0.041	0.003	1.64E-11	0	PPP1R16B	13.66666667
FGF71	7.39E-16	0.3153315	0.034	0.002	1.78E-11	0	FGF7	17
PREX2	8.13E-16	0.5396571	0.055	0.005	1.96E-11	0	PREX2	11
CFH	9.01E-16	0.1652038	0.027	0.001	2.17E-11	0	CFH	27
SLC7A113	9.30E-16	0.9556161	0.192	0.05	2.24E-11	0	SLC7A11	3.84
ETS24	1.23E-15	1.204083	0.418	0.186	2.98E-11	0	ETS2	2.247311828
WDFY4	1.39E-15	0.1682885	0.041	0.003	3.35E-11	0	WDFY4	13.66666667
SH3TC1	1.55E-15	0.2681532	0.034	0.002	3.74E-11	0	SH3TC1	17
TIMP32	2.26E-15	1.012076	0.096	0.015	5.45E-11	0	TIMP3	6.4
ATG74	2.39E-15	1.153626	0.438	0.212	5.75E-11	0	ATG7	2.066037736
SYTL32	2.42E-15	1.13553	0.212	0.06	5.82E-11	0	SYTL3	3.533333333
LAMB1	2.60E-15	0.6541339	0.103	0.017	6.26E-11	0	LAMB1	6.058823529
IL1A1	2.82E-15	0.3175162	0.027	0.001	6.79E-11	0	IL1A	27
RILPL24	3.03E-15	0.6898938	0.171	0.042	7.32E-11	0	RILPL2	4.071428571
SLC8A1-AS1	3.88E-15	0.108647	0.034	0.002	9.35E-11	0	SLC8A1-AS1	17
ZEB14	4.03E-15	1.512844	0.199	0.055	9.72E-11	0	ZEB1	3.618181818
NUAK11	4.30E-15	0.3384628	0.075	0.01	1.04E-10	0	NUAK1	7.5
GJA13	6.02E-15	0.7372903	0.137	0.029	1.45E-10	0	GJA1	4.724137931
IDO11	7.38E-15	0.7875531	0.13	0.026	1.78E-10	0	IDO1	5
NOTCH42	7.41E-15	0.2069985	0.021	0.001	1.79E-10	0	NOTCH4	21
STK42	8.11E-15	1.390413	0.199	0.055	1.96E-10	0	STK4	3.618181818
MYO1F2	9.01E-15	0.3648884	0.075	0.01	2.17E-10	0	MYO1F	7.5
HMOX12	9.90E-15	0.962334	0.192	0.051	2.39E-10	0	HMOX1	3.764705882
COL6A3	1.23E-14	0.4568273	0.055	0.006	2.96E-10	0	COL6A3	9.166666667
MANBA2	1.26E-14	0.7058459	0.219	0.064	3.05E-10	0	MANBA	3.421875
SH3PXD2B6	1.39E-14	0.9045182	0.315	0.122	3.36E-10	0	SH3PXD2B	2.581967213
GPAT33	1.47E-14	1.251543	0.178	0.046	3.55E-10	0	GPAT3	3.869565217
PIK3R6	1.52E-14	0.1374409	0.034	0.002	3.67E-10	0	PIK3R6	17
ATP13A33	1.96E-14	1.167704	0.479	0.261	4.72E-10	0	ATP13A3	1.835249042
APBB3	2.23E-14	0.223026	0.048	0.004	5.38E-10	0	APBB3	12
SLC17A7	2.29E-14	0.1134875	0.014	0	5.51E-10	0	SLC17A7	#DIV/0!
DNAJB5-AS1	2.29E-14	0.1079904	0.014	0	5.53E-10	0	DNAJB5-AS	#DIV/0!
MEG31	2.30E-14	0.3257796	0.027	0.001	5.54E-10	0	MEG3	27
IL18R12	2.31E-14	0.2790083	0.027	0.001	5.58E-10	0	IL18R1	27
LPXN2	2.75E-14	0.8472959	0.192	0.054	6.62E-10	0	LPXN	3.555555556
HTRA11	2.89E-14	0.1834331	0.041	0.003	6.97E-10	0	HTRA1	13.66666667
RCBTB2	3.03E-14	0.2527609	0.048	0.004	7.31E-10	0	RCBTB2	12
MAP4K43	3.71E-14	1.077707	0.459	0.235	8.95E-10	0	MAP4K4	1.953191489
GPRIN32	3.94E-14	0.4689955	0.096	0.016	9.50E-10	0	GPRIN3	6
RP11-563J2.24	4.34E-14	0.3251751	0.089	0.014	1.05E-09	0	RP11-563J2	6.357142857
AOAH1	4.39E-14	0.6776556	0.055	0.006	1.06E-09	0	AOAH	9.166666667
ENG3	7.99E-14	0.5249621	0.13	0.028	1.93E-09	0	ENG	4.642857143
ADA	9.98E-14	0.2602224	0.034	0.002	2.41E-09	0	ADA	17
RP11-428F8.21	1.06E-13	0.1604855	0.034	0.002	2.55E-09	0	RP11-428F8	17
ITPKB3	1.25E-13	0.8723755	0.164	0.043	3.01E-09	0	ITPKB	3.813953488
SEMA6B	1.44E-13	0.2487523	0.027	0.002	3.47E-09	0	SEMA6B	13.5
CPNE82	2.09E-13	0.3222228	0.068	0.009	5.05E-09	0	CPNE8	7.555555556
IFI163	2.15E-13	1.064711	0.322	0.134	5.18E-09	0	IFI16	2.402985075

PIM23	2.17E-13	0.5195104	0.089	0.015	5.24E-09	0	PIM2	5.933333333
B2M4	2.41E-13	0.8223764	0.993	0.971	5.82E-09	0	B2M	1.022657055
TFRC1	2.54E-13	1.089154	0.178	0.05	6.13E-09	0	TFRC	3.56
SV2B1	2.57E-13	0.3910795	0.055	0.006	6.21E-09	0	SV2B	9.166666667
CACNA1C2	2.63E-13	0.9252285	0.096	0.017	6.35E-09	0	CACNA1C	5.647058824
ITGA12	3.29E-13	0.438133	0.068	0.009	7.93E-09	0	ITGA1	7.555555556
SLC43A21	3.37E-13	0.3894298	0.096	0.017	8.13E-09	0	SLC43A2	5.647058824
TMEM176B2	3.49E-13	0.4659003	0.123	0.026	8.42E-09	0	TMEM176E	4.730769231
C1orf162	3.67E-13	0.1119592	0.027	0.002	8.84E-09	0	C1orf162	13.5
RP11-248J23.72	4.03E-13	0.2742628	0.062	0.008	9.71E-09	0	RP11-248J2	7.75
MAML24	4.94E-13	0.765047	0.692	0.415	1.19E-08	0	MAML2	1.66746988
ST8SIA2	5.25E-13	0.4580012	0.034	0.003	1.27E-08	0	ST8SIA2	11.33333333
CLEC4F	5.31E-13	0.2231435	0.014	0	1.28E-08	0	CLEC4F	#DIV/0!
CD79B1	5.32E-13	0.1087797	0.014	0	1.28E-08	0	CD79B	#DIV/0!
LRP1	5.86E-13	0.2335446	0.041	0.004	1.41E-08	0	LRP1	10.25
FAM49B4	6.33E-13	1.043967	0.411	0.203	1.53E-08	0	FAM49B	2.024630542
SULF11	7.86E-13	0.2351433	0.055	0.006	1.90E-08	0	SULF1	9.166666667
HMCN1	8.23E-13	0.2486884	0.048	0.005	1.98E-08	0	HMCN1	9.6
SASH14	9.86E-13	0.9789287	0.356	0.158	2.38E-08	0	SASH1	2.253164557
CDKN2A1	1.07E-12	0.1031908	0.034	0.003	2.57E-08	0	CDKN2A	11.33333333
FGD52	1.10E-12	0.2575185	0.048	0.005	2.64E-08	0	FGD5	9.6
RP11-1148O4.2	1.47E-12	0.1800098	0.041	0.004	3.56E-08	0	RP11-1148	10.25
CAMK2D4	2.46E-12	0.8724833	0.377	0.174	5.94E-08	0	CAMK2D	2.166666667
RAB8B3	2.83E-12	0.9067886	0.342	0.146	6.82E-08	0	RAB8B	2.342465753
SH2B31	3.70E-12	0.3276958	0.082	0.014	8.93E-08	0	SH2B3	5.857142857
B3GNTL11	4.27E-12	0.3015808	0.048	0.005	1.03E-07	0	B3GNTL1	9.6
SDS	4.47E-12	0.154015	0.034	0.003	1.08E-07	0	SDS	11.33333333
ACER32	5.00E-12	0.6409378	0.164	0.046	1.21E-07	0	ACER3	3.565217391
SOAT13	5.12E-12	0.7822845	0.144	0.037	1.23E-07	0	SOAT1	3.891891892
CDH114	6.37E-12	0.251401	0.034	0.003	1.54E-07	0	CDH11	11.33333333
CCDC803	7.06E-12	0.2726512	0.075	0.012	1.70E-07	0	CCDC80	6.25
CRTAM1	7.13E-12	0.1151511	0.014	0	1.72E-07	0	CRTAM	#DIV/0!
DOCK23	7.93E-12	0.370114	0.062	0.009	1.91E-07	0	DOCK2	6.888888889
CDC42SE22	8.20E-12	1.147186	0.404	0.208	1.98E-07	0	CDC42SE2	1.942307692
KCNE13	8.86E-12	0.4554572	0.062	0.009	2.14E-07	0	KCNE1	6.888888889
IKZF12	1.10E-11	0.2045748	0.041	0.004	2.66E-07	0	IKZF1	10.25
FLT11	1.26E-11	0.5010066	0.075	0.012	3.03E-07	0	FLT1	6.25
MMP15	1.30E-11	1.034873	0.027	0.002	3.12E-07	0	MMP1	13.5
PTGER42	1.40E-11	0.5161084	0.062	0.009	3.37E-07	0	PTGER4	6.888888889
CTSH1	1.54E-11	0.1862058	0.062	0.009	3.71E-07	0	CTSH	6.888888889
CTSS3	1.61E-11	0.8358815	0.315	0.137	3.88E-07	0	CTSS	2.299270073
PRDM13	1.90E-11	0.5078836	0.13	0.032	4.57E-07	0	PRDM1	4.0625
ME11	2.07E-11	0.7981365	0.144	0.038	4.99E-07	0	ME1	3.789473684
CTTNBP2NL3	2.16E-11	1.115494	0.397	0.21	5.22E-07	0	CTTNBP2N	1.89047619
GBP51	2.59E-11	0.1441044	0.034	0.003	6.25E-07	0	GBP5	11.33333333
FNIP26	2.98E-11	1.271707	0.267	0.108	7.18E-07	0	FNIP2	2.472222222
ITM2A2	3.26E-11	0.3309048	0.048	0.006	7.86E-07	0	ITM2A	8
MMP21	3.87E-11	0.166622	0.034	0.003	9.34E-07	0	MMP2	11.33333333
ENTPD1-AS12	3.97E-11	0.6800069	0.171	0.052	9.56E-07	0	ENTPD1-AS	3.288461538
CD141	3.97E-11	0.1829687	0.041	0.004	9.57E-07	0	CD14	10.25
GAB25	4.01E-11	1.157528	0.384	0.199	9.67E-07	0	GAB2	1.929648241
SIPA1L15	4.33E-11	1.391033	0.568	0.385	1.04E-06	0	SIPA1L1	1.475324675

RCSD11	5.33E-11	0.5179485	0.075	0.013	1.28E-06	0	RCSD1	5.769230769
MAF4	5.41E-11	0.2409609	0.062	0.009	1.30E-06	0	MAF	6.888888889
PROS11	5.57E-11	0.1744668	0.034	0.003	1.34E-06	0	PROS1	11.33333333
MITF4	5.76E-11	0.6273356	0.13	0.033	1.39E-06	0	MITF	3.939393939
C5AR21	7.44E-11	0.4616675	0.062	0.009	1.79E-06	0	C5AR2	6.888888889
ST182	7.46E-11	0.3526382	0.055	0.007	1.80E-06	0	ST18	7.857142857
GIMAP8	8.25E-11	0.2092078	0.027	0.002	1.99E-06	0	GIMAP8	13.5
B3GNT54	9.64E-11	0.8391226	0.178	0.057	2.32E-06	0	B3GNT5	3.122807018
LRRK21	9.66E-11	0.3216103	0.055	0.007	2.33E-06	0	LRRK2	7.857142857
AKR1B152	1.25E-10	0.2377062	0.048	0.006	3.00E-06	0	AKR1B15	8
RP11-452H21.13	1.26E-10	0.7445613	0.199	0.069	3.05E-06	0	RP11-452H	2.884057971
OLFML2B1	1.41E-10	0.2067502	0.027	0.002	3.41E-06	0	OLFML2B	13.5
TEX26-AS1	1.61E-10	0.1672718	0.021	0.001	3.89E-06	0	TEX26-AS1	21
GSTO13	1.79E-10	0.5836388	0.13	0.034	4.31E-06	0	GSTO1	3.823529412
PCBP31	2.07E-10	0.2763946	0.041	0.005	4.99E-06	0	PCBP3	8.2
CYTH14	2.26E-10	0.8262812	0.322	0.153	5.45E-06	0	CYTH1	2.104575163
RHOJ2	2.69E-10	0.4880662	0.096	0.021	6.48E-06	0	RHOJ	4.571428571
RP11-779O18.12	3.74E-10	0.2788231	0.055	0.008	9.03E-06	0	RP11-779O	6.875
PDGFRA2	3.81E-10	0.224861	0.027	0.002	9.18E-06	0	PDGFRA	13.5
RP11-46C24.7	3.86E-10	0.1106439	0.014	0.001	9.31E-06	0	RP11-46C2	14
FYB2	4.49E-10	0.5874593	0.055	0.008	1.08E-05	0	FYB	6.875
MSRB32	4.56E-10	0.4941127	0.089	0.019	1.10E-05	0	MSRB3	4.684210526
ATP1B33	4.88E-10	0.9442478	0.438	0.255	1.18E-05	0	ATP1B3	1.717647059
CFLAR3	5.01E-10	0.7146374	0.541	0.34	1.21E-05	0	CFLAR	1.591176471
KIAA1462	5.02E-10	0.2820317	0.034	0.003	1.21E-05	0	KIAA1462	11.33333333
BHLHE414	5.76E-10	0.5064523	0.151	0.045	1.39E-05	0	BHLHE41	3.355555556
CREM4	5.99E-10	0.8018861	0.247	0.1	1.44E-05	0	CREM	2.47
JAZF13	6.67E-10	0.9221405	0.226	0.087	1.61E-05	0	JAZF1	2.597701149
RP11-307P5.13	7.60E-10	0.4701908	0.096	0.022	1.83E-05	0	RP11-307P	4.363636364
GLS4	7.71E-10	0.6704323	0.384	0.199	1.86E-05	0	GLS	1.929648241
PRKCA2	7.91E-10	0.7140154	0.192	0.066	1.91E-05	0	PRKCA	2.909090909
IFI27	8.12E-10	0.2744459	0.021	0.001	1.96E-05	0	IFI27	21
ADAMTS123	8.43E-10	0.8562494	0.048	0.006	2.03E-05	0	ADAMTS12	8
C8orf58	1.00E-09	0.1401616	0.034	0.003	2.41E-05	0	C8orf58	11.33333333
MCAM3	1.07E-09	0.5802187	0.082	0.017	2.59E-05	0	MCAM	4.823529412
FAM135A2	1.14E-09	0.717743	0.185	0.065	2.75E-05	0	FAM135A	2.846153846
VWF	1.21E-09	0.4139631	0.048	0.007	2.92E-05	0	VWF	6.857142857
RBP14	1.38E-09	0.4869849	0.151	0.046	3.32E-05	0	RBP1	3.282608696
RP11-758H9.21	1.57E-09	0.4128957	0.082	0.017	3.78E-05	0	RP11-758H	4.823529412
CASP101	1.66E-09	0.1188529	0.027	0.002	4.00E-05	0	CASP10	13.5
PLVAP1	1.70E-09	0.5486782	0.034	0.004	4.10E-05	0	PLVAP	8.5
EPB41L24	1.73E-09	0.8026054	0.26	0.112	4.18E-05	0	EPB41L2	2.321428571
CD343	1.81E-09	0.2468308	0.048	0.007	4.35E-05	0	CD34	6.857142857
IGFBP71	1.85E-09	0.7485851	0.068	0.013	4.45E-05	0	IGFBP7	5.230769231
SLFN111	1.85E-09	0.1757233	0.041	0.005	4.46E-05	0	SLFN11	8.2
MZB11	1.86E-09	0.1783911	0.014	0.001	4.48E-05	0	MZB1	14
TRAC	1.86E-09	0.1179751	0.014	0.001	4.50E-05	0	TRAC	14
CD5	1.86E-09	0.1007478	0.014	0.001	4.50E-05	0	CD5	14
FILIP1	2.04E-09	0.3832197	0.048	0.007	4.91E-05	0	FILIP1	6.857142857
SAMD93	2.19E-09	0.2919981	0.075	0.015	5.27E-05	0	SAMD9	5
HLA-A3	2.21E-09	1.093307	0.507	0.318	5.34E-05	0	HLA-A	1.594339623
LIX1L2	2.25E-09	0.2587064	0.062	0.01	5.43E-05	0	LIX1L	6.2

IRAK24	2.36E-09	0.707854	0.288	0.128	5.68E-05	0	IRAK2	2.25
REL4	2.39E-09	0.9180156	0.486	0.301	5.77E-05	0	REL	1.61461794
PLEKHM32	2.44E-09	0.4186275	0.116	0.031	5.88E-05	0	PLEKHM3	3.741935484
EMILIN21	2.50E-09	0.1120371	0.027	0.002	6.03E-05	0	EMILIN2	13.5
PCNX4	2.62E-09	1.049154	0.37	0.209	6.32E-05	0	PCNX	1.770334928
LINC010601	3.05E-09	0.689216	0.068	0.013	7.34E-05	0	LINC01060	5.230769231
LUCAT13	3.06E-09	0.9643381	0.384	0.202	7.39E-05	0	LUCAT1	1.900990099
HLA-B4	3.43E-09	1.044751	0.671	0.542	8.27E-05	0	HLA-B	1.23800738
GPRASP21	3.46E-09	0.1420646	0.021	0.001	8.35E-05	0	GPRASP2	21
PSIP13	3.49E-09	0.3151953	0.082	0.017	8.41E-05	0	PSIP1	4.823529412
LRFN51	3.61E-09	0.4889055	0.027	0.002	8.70E-05	0	LRFN5	13.5
LYN3	4.02E-09	0.9916718	0.575	0.408	9.68E-05	0	LYN	1.409313725
FMN12	4.06E-09	1.253294	0.247	0.106	9.80E-05	0	FMN1	2.330188679
TXNDC151	4.78E-09	0.2647033	0.075	0.015	0.000115353	0	TXNDC15	5
GNA133	4.85E-09	0.8863222	0.384	0.209	0.000116891	0	GNA13	1.837320574
RHBDF23	5.05E-09	0.3997736	0.13	0.038	0.000121716	0	RHBDF2	3.421052632
CCL51	5.38E-09	0.3180968	0.068	0.013	0.00012966	0	CCL5	5.230769231
SYK4	5.42E-09	0.4326827	0.075	0.015	0.000130703	0	SYK	5
LBR3	5.46E-09	0.3529956	0.082	0.018	0.000131564	0	LBR	4.555555556
ALDH23	5.60E-09	0.3680826	0.11	0.029	0.000135043	0	ALDH2	3.793103448
ZSCAN311	5.66E-09	0.1563786	0.041	0.005	0.000136568	0	ZSCAN31	8.2
NRP14	6.46E-09	0.9972713	0.171	0.06	0.000155819	0	NRP1	2.85
GAB3	6.56E-09	0.1011756	0.021	0.001	0.000158167	0	GAB3	21
KLRB1	7.25E-09	0.3278001	0.014	0.001	0.000174778	0	KLRB1	14
PARVG	7.25E-09	0.1121931	0.014	0.001	0.000174778	0	PARVG	14
JPH4	7.27E-09	0.11898	0.014	0.001	0.000175406	0	JPH4	14
NR4A34	7.33E-09	0.3936063	0.089	0.021	0.000176714	0	NR4A3	4.238095238
SDCBP4	7.34E-09	0.7239315	0.733	0.617	0.00017707	0	SDCBP	1.188006483
EBI31	7.57E-09	0.1476966	0.034	0.004	0.000182504	0	EBI3	8.5
SGK13	8.65E-09	0.9691857	0.24	0.105	0.0002085	0	SGK1	2.285714286
BCL2A12	1.03E-08	0.6426655	0.212	0.082	0.000248532	0	BCL2A1	2.585365854
DOCK113	1.11E-08	0.6241602	0.103	0.027	0.000267877	0	DOCK11	3.814814815
IGDCC42	1.20E-08	0.1064955	0.021	0.001	0.000290479	0	IGDCC4	21
FRMD4A3	1.22E-08	0.8566129	0.466	0.277	0.000294972	0	FRMD4A	1.682310469
NLRP14	1.60E-08	0.4220388	0.068	0.014	0.000385652	0	NLRP1	4.857142857
SORCS32	1.62E-08	0.2304574	0.048	0.007	0.000390029	0	SORCS3	6.857142857
SH3RF32	1.70E-08	0.3759927	0.075	0.016	0.000409652	0	SH3RF3	4.6875
RP5-1024G6.2	1.70E-08	0.1261731	0.027	0.003	0.000410599	0	RP5-1024G	9
SP140	1.84E-08	0.3998931	0.048	0.007	0.000442958	0	SP140	6.857142857
FTH16	1.95E-08	1.265882	0.89	0.844	0.000470781	0	FTH1	1.05450237
CEP85L3	2.01E-08	0.3447449	0.13	0.039	0.000483943	0	CEP85L	3.333333333
FBXL73	2.19E-08	0.943098	0.123	0.037	0.00052924	0	FBXL7	3.324324324
TVP23A	2.21E-08	0.1083423	0.034	0.004	0.000533165	0	TVP23A	8.5
RASA33	2.25E-08	0.4481249	0.062	0.012	0.000542984	0	RASA3	5.166666667
AKR1B12	2.44E-08	0.6029598	0.144	0.047	0.000587534	0	AKR1B1	3.063829787
HCLS12	2.50E-08	0.2149648	0.034	0.004	0.000602998	0	HCLS1	8.5
CTSK1	3.22E-08	0.4832361	0.048	0.008	0.000777027	0	CTSK	6
CAPZB2	3.31E-08	0.6524982	0.356	0.189	0.000797828	0	CAPZB	1.883597884
HIP13	3.32E-08	0.6486067	0.219	0.092	0.00079982	0	HIP1	2.380434783
EPSTI11	3.61E-08	0.2335525	0.048	0.008	0.000869737	0	EPSTI1	6
NPC25	3.64E-08	1.122563	0.568	0.485	0.000876807	0	NPC2	1.171134021
RP11-6J21.21	3.70E-08	0.1162231	0.021	0.002	0.000891624	0	RP11-6J21.	10.5

FAM102B2	3.73E-08	0.4203327	0.096	0.025	0.000898604	0	FAM102B	3.84
RP11-326G21.11	3.74E-08	0.1307142	0.021	0.002	0.000901664	0	RP11-326G	10.5
MYO15B1	3.87E-08	0.2929517	0.062	0.012	0.000932765	0	MYO15B	5.166666667
OAF1	4.01E-08	0.1479233	0.034	0.004	0.000966743	0	OAF	8.5
PALD11	4.44E-08	0.2331634	0.027	0.003	0.001070938	0	PALD1	9
DMXL22	4.56E-08	0.5882584	0.137	0.044	0.001098683	0	DMXL2	3.113636364
ZFYVE281	4.73E-08	0.398236	0.082	0.02	0.001140483	0	ZFYVE28	4.1
ATP2B14	4.81E-08	0.7723644	0.219	0.093	0.001160869	0	ATP2B1	2.35483871
G0S23	4.86E-08	0.3460877	0.089	0.022	0.001171995	0	G0S2	4.045454545
GRB23	5.20E-08	0.9029263	0.308	0.162	0.001253315	0	GRB2	1.901234568
TSHZ32	5.38E-08	0.3676485	0.048	0.008	0.001297211	0	TSHZ3	6
ADAP21	5.94E-08	0.1554793	0.041	0.006	0.001431369	0	ADAP2	6.833333333
CARD164	6.30E-08	0.4556881	0.137	0.045	0.001517968	0	CARD16	3.044444444
AC104532.4	6.32E-08	0.1286685	0.021	0.002	0.001524229	0	AC104532.4	10.5
AHRR	6.32E-08	0.217551	0.034	0.004	0.001524328	0	AHRR	8.5
ZNF3312	6.49E-08	0.5573799	0.151	0.053	0.001564132	0	ZNF331	2.849056604
PLCB13	6.89E-08	1.091984	0.384	0.224	0.001661091	0	PLCB1	1.714285714
IL1RAP2	6.90E-08	0.6546566	0.151	0.053	0.001664665	0	IL1RAP	2.849056604
CSF2RB1	6.96E-08	0.1193545	0.014	0.001	0.001678012	0	CSF2RB	14
DLGAP1-AS2	8.01E-08	0.1525022	0.041	0.006	0.001931305	0	DLGAP1-AS	6.833333333
HIF1A3	8.31E-08	1.025621	0.582	0.442	0.002003448	0	HIF1A	1.316742081
CD41	8.35E-08	0.1872575	0.041	0.006	0.002014095	0	CD4	6.833333333
RP11-360L9.42	9.04E-08	0.5425385	0.13	0.041	0.002180712	0	RP11-360L9	3.170731707
SH2D3C1	9.38E-08	0.203066	0.034	0.004	0.002262723	0	SH2D3C	8.5
IL23A	1.08E-07	0.4509677	0.041	0.006	0.002593615	0	IL23A	6.833333333
NEURL1B1	1.20E-07	0.1273404	0.034	0.004	0.002897782	0	NEURL1B	8.5
ARRB2	1.35E-07	0.5035253	0.055	0.01	0.003265903	0	ARRB2	5.5
ADAM82	1.51E-07	0.1623529	0.027	0.003	0.003640889	0	ADAM8	9
RAB9B1	1.55E-07	0.121915	0.027	0.003	0.003725543	0	RAB9B	9
TMEM233	1.56E-07	0.19192	0.021	0.002	0.003753038	0	TMEM233	10.5
LINC003092	1.56E-07	0.1413945	0.027	0.003	0.003767088	0	LINC00309	9
RAB39A	1.57E-07	0.118441	0.027	0.003	0.003788029	0	RAB39A	9
KCNQ1OT14	1.60E-07	0.7049528	0.205	0.087	0.003862888	0	KCNQ1OT1	2.356321839
TBC1D22A4	1.60E-07	0.9176596	0.295	0.154	0.003863347	0	TBC1D22A	1.915584416
GOLIM43	1.62E-07	0.2408732	0.075	0.018	0.003895721	0	GOLIM4	4.166666667
LGALS12	1.74E-07	1.222879	0.192	0.081	0.004185576	0	LGALS1	2.37037037
CPM3	1.76E-07	0.3958174	0.103	0.03	0.004237936	0	CPM	3.433333333
GRAMD1A4	1.80E-07	0.7746197	0.116	0.037	0.004339253	0	GRAMD1A	3.135135135
TG1	1.93E-07	0.1822499	0.055	0.01	0.004654074	0	TG	5.5
GPR137B4	1.98E-07	0.5871715	0.13	0.044	0.004786316	0	GPR137B	2.954545455
ENPP21	2.00E-07	0.7546225	0.062	0.013	0.004826693	0	ENPP2	4.769230769
RASGRF22	2.01E-07	0.3550028	0.048	0.008	0.00485344	0	RASGRF2	6
MIR22HG2	2.04E-07	0.223661	0.055	0.01	0.004926051	0	MIR22HG	5.5
IL3RA3	2.05E-07	0.3303698	0.068	0.015	0.004934681	0	IL3RA	4.533333333
ARAP11	2.17E-07	0.3086267	0.055	0.011	0.005221974	0	ARAP1	5
PLIN24	2.34E-07	0.7656603	0.212	0.095	0.005642272	0	PLIN2	2.231578947
FLRT21	2.41E-07	0.2086384	0.021	0.002	0.005814695	0	FLRT2	10.5
TMEM178A1	2.53E-07	0.1172128	0.021	0.002	0.006094087	0	TMEM178A	10.5
WARS3	2.54E-07	0.5179927	0.171	0.068	0.006123657	0	WARS	2.514705882
EDA1	2.56E-07	0.6562212	0.164	0.063	0.006166845	0	EDA	2.603174603
HIST1H4C5	2.89E-07	0.738801	0.301	0.167	0.006958642	0	HIST1H4C	1.80239521
AKT34	2.95E-07	0.6842761	0.514	0.346	0.007117252	0	AKT3	1.485549133

FTX5	3.06E-07	0.7002046	0.671	0.558	0.007381603	0	FTX	1.202508961
RP11-38L15.31	3.12E-07	0.1390236	0.041	0.006	0.007525166	0	RP11-38L1!	6.833333333
STC13	3.17E-07	1.186148	0.137	0.048	0.007642391	0	STC1	2.854166667
HIF1A-AS23	3.82E-07	0.9711974	0.281	0.144	0.009201539	0	HIF1A-AS2	1.951388889
MIR3142HG2	4.11E-07	0.2309142	0.055	0.011	0.009916852	0	MIR3142H(	5
LAG31	4.11E-07	0.2027976	0.014	0.001	0.009921494	0	LAG3	14
UCHL1-AS1	4.13E-07	0.1083689	0.014	0.001	0.009963394	0	UCHL1-AS1	14
PTPN93	4.20E-07	0.4775197	0.199	0.085	0.0101324	0	PTPN9	2.341176471
DIRC23	4.31E-07	0.7102818	0.151	0.058	0.01039711	0	DIRC2	2.603448276
HLA-E3	4.74E-07	1.127787	0.205	0.092	0.01144151	0	HLA-E	2.22826087
CCL42	5.44E-07	0.9577712	0.137	0.049	0.01311332	0	CCL4	2.795918367
FOXN33	5.86E-07	0.7573997	0.322	0.18	0.01413513	0	FOXN3	1.788888889
MT2A2	6.15E-07	1.288889	0.158	0.06	0.0148278	0	MT2A	2.633333333
MBNL15	6.70E-07	1.06172	0.534	0.408	0.01615513	0	MBNL1	1.308823529
SNX293	6.82E-07	0.6021097	0.212	0.098	0.01645555	0	SNX29	2.163265306
RASSF8-AS11	6.94E-07	0.2747395	0.055	0.011	0.01673845	0	RASSF8-AS	5
RP11-557H15.41	7.10E-07	0.1741802	0.034	0.005	0.01712344	0	RP11-557H	6.8
CD402	7.34E-07	0.1273997	0.034	0.005	0.01769838	0	CD40	6.8
HYAL22	7.51E-07	0.2017935	0.034	0.005	0.01811506	0	HYAL2	6.8
RP11-796E2.42	8.34E-07	0.3414026	0.075	0.019	0.02010925	0	RP11-796E	3.947368421
PAPSS22	8.42E-07	0.4146648	0.103	0.032	0.02029712	0	PAPSS2	3.21875
MDFIC1	8.71E-07	0.2747782	0.048	0.009	0.02100997	0	MDFIC	5.333333333
MMP14	9.14E-07	0.2779284	0.055	0.011	0.02204985	0	MMP14	5
CTD-2020K17.12	9.89E-07	0.2255136	0.055	0.011	0.02383782	0	CTD-2020K	5
ARL4A3	1.08E-06	0.555926	0.24	0.118	0.02612056	0	ARL4A	2.033898305
CLEC1A1	1.09E-06	0.3630177	0.075	0.02	0.02630893	0	CLEC1A	3.75
IL6R2	1.12E-06	0.4494859	0.164	0.066	0.02695353	0	IL6R	2.484848485
TWIST2	1.13E-06	0.1515608	0.021	0.002	0.02726431	0	TWIST2	10.5
TIMP14	1.13E-06	1.045582	0.164	0.068	0.02731865	0	TIMP1	2.411764706
CTB-113P19.1	1.14E-06	0.1171075	0.021	0.002	0.02741202	0	CTB-113P1	10.5
TPM32	1.17E-06	0.5938092	0.233	0.112	0.02827146	0	TPM3	2.080357143
STX111	1.19E-06	0.3870501	0.082	0.023	0.0286433	0	STX11	3.565217391
ATG16L22	1.31E-06	0.1872815	0.048	0.009	0.03155972	0	ATG16L2	5.333333333
MYO9B3	1.33E-06	0.5855463	0.329	0.187	0.03209222	0	MYO9B	1.759358289
PLCL21	1.35E-06	0.3901164	0.075	0.02	0.0325183	0	PLCL2	3.75
LAMP31	1.35E-06	0.1309526	0.027	0.003	0.03267257	0	LAMP3	9
SAMHD11	1.56E-06	0.2001121	0.048	0.009	0.03754433	0	SAMHD1	5.333333333
AC079305.11	1.62E-06	0.1213192	0.021	0.002	0.03900175	0	AC079305.	10.5
CALCB	1.63E-06	0.1401379	0.021	0.002	0.03924224	0	CALCB	10.5
FAM101B	1.76E-06	0.1024705	0.014	0.001	0.04237754	0	FAM101B	14
SYN11	1.76E-06	0.1016498	0.014	0.001	0.04248501	0	SYN1	14
CXCL111	1.80E-06	0.286181	0.014	0.001	0.04329942	0	CXCL11	14
DPYSL22	1.83E-06	0.6189635	0.24	0.12	0.04408233	0	DPYSL2	2
PTAFR3	1.85E-06	0.3387296	0.068	0.017	0.04453327	0	PTAFR	4
GIMAP22	1.86E-06	0.1569134	0.041	0.007	0.04487087	0	GIMAP2	5.857142857
