# Supplementary material for: Ductal keratin 15+ luminal progenitors in normal breast exhibit a basal-like breast cancer transcriptomic signature
Source: NPJ Breast Cancer. 2022 Jul 12;8:81. doi: 10.1038/s41523-022-00444-8 (PMC9276673; doi:10.1038/s41523-022-00444-8)
Supplement: Supplementary file 4 — Dataset2 [file 41523_2022_444_MOESM4_ESM.pdf]

Supplementary Data 2.

DEGs encoding cell-surface proteins.
Date include DEGs from Supplementary Data 1, identified in the in silico human surfaceome list of all predicted membrane proteins.

Cluster	Cell-surface protein encoding genes
1.1	PIGR
1.1	KIAA0922
1.1	CXCR4
1.1	LRP6
1.1	GLIPR1
1.1	HCAR2
1.1	FGFR1
1.1	CLDN1
1.1	SLC20A2
1.1	GABRP
1.1	MET
1.1	SLC9A7
1.1	ATP13A3
1.1	DSG3
1.1	HLA-DRA
1.1	PTPRJ
1.1	TGFBR3
1.1	NTRK2
1.1	TNFRSF11A
1.1	FAS
1.2	CLDN1
1.2	GLIPR1
1.2	ITGA2
1.2	GABRP
1.2	ZPLD1
1.2	DSG3
1.2	ITGB8
1.2	MET
1.2	HCAR2
1.2	CDCP1
1.2	PLAUR
1.2	PIGR
1.2	EGFR
1.2	BACE2
1.2	SLC20A2
1.2	CD55
1.2	SLC28A3
1.2	DSG2
1.2	PROM1
1.2	SLC6A14
1.2	VNN1
1.2	EFNA5
1.2	IFNAR2
1.2	SLC39A14
1.2	GPM6B
1.3	SLC12A2

1.3	LRP1B
1.3	EFNA5
1.3	VNN1
1.3	EGFR
1.3	SEMA6A
1.3	PROM1
1.3	SLC34A2
1.3	VNN3
1.3	KIT
1.3	PIGR
1.3	EVA1C
1.3	ITGB8
1.3	SLC28A3
1.3	LINGO2
1.3	CHPT1
1.3	SLC26A2
1.3	SLC6A14
1.3	PLXDC2
1.3	TGFBR3
1.3	SLC39A14
1.3	BACE2
1.4	EVA1C
1.4	VNN3
1.4	CHODL
1.4	SLC34A2
1.4	PROM1
1.4	KIT
1.4	DCHS2
1.4	EFNA5
1.4	CHPT1
1.4	SEMA6A
1.4	CNTN4
1.4	LRP1B
1.4	SLC28A3
1.4	GABRP
1.4	SLC26A2
1.4	PLXDC2
1.4	SLC39A8
1.4	VNN1
1.4	PODXL
1.4	TLR2
1.4	GHR
1.4	OSMR
1.4	PIGR
1.4	SLC12A2
1.4	LRIG1
1.4	TRABD2B

1.4	EGFR
1.4	SLC39A14
1.4	NALCN
1.4	RNF150
1.4	PLA2R1
1.4	SLC11A2
1.4	CLEC7A
1.4	ITGB8
1.4	SGCZ
1.4	SLC5A6
1.4	CX3CL1
1.4	SLC24A3
1.4	IFNAR2
2.1	AREG
2.1	TSPAN5
2.1	ADAM32
2.1	EREG
2.1	ERBB4
2.1	ALCAM
2.1	ITGAV
2.1	EFNA1
2.1	SLC2A1
2.1	SLC39A6
2.1	TFPI
2.1	SDC2
2.1	ECE1
2.2	TNFSF11
2.2	F3
2.2	SLC39A6
2.2	PRLR
2.2	AREG
2.2	ERBB4
2.2	HEPACAM2
2.2	SDC2
2.2	TSPAN5
2.2	ENPP1
2.2	SLC7A2
2.2	KIAA1324
2.2	LPAR3
2.2	IL20RA
2.2	NRXN3
2.2	ERBB2
2.2	VTCN1
2.2	MEGF9
2.2	TSPAN1
2.2	ECE1
2.3	AREG

2.3	PTGER3
2.3	SLC39A6
2.3	SDC2
2.3	TNFSF11
2.3	SEMA4A
2.3	ADAM32
2.3	SLC38A2
2.3	TSPAN1
2.3	F3
2.3	APLP2
2.3	KIAA0319
2.3	GLRA1
2.3	LSAMP
2.3	CD164
2.3	CNNM4
2.3	CD63
2.3	PRLR
2.3	SLC10A1
2.3	EFNA1
2.3	TMPRSS11E
2.3	GRIK4
2.3	DDR2
2.3	EFNB2
2.3	ERBB4
2.3	KIAA1324
2.3	NUP210L
2.3	HEPACAM2
2.3	FAM171B
2.3	SLC9A1
2.3	BAMBI
2.3	TSPAN6
2.3	TFPI
2.3	VTCN1
2.3	LDLR
2.3	LPAR3
2.3	GUCY2C
2.3	FAT1
2.4	SLC38A2
2.4	TFPI
2.4	AREG
2.4	ITGAV
2.4	SLC39A10
2.4	CD9
2.4	ALCAM
2.4	ANO6
2.4	ERBB4
2.4	TPBG

2.4	GPRC5A
2.4	LSAMP
2.4	CLSTN2
2.4	TSPAN5
2.4	PTPRM
2.4	CD63
2.4	ATP2B4
2.4	EREG
2.4	LYPD6B
2.4	TGFBR1
2.4	FAT1
2.4	ADAM10
2.5	TSPAN1
2.5	SLC7A2
2.5	ERBB4
2.5	ALCAM
2.5	TFPI
2.5	EREG
2.5	LYPD6B
2.5	ENPP1
2.5	SLC38A2
2.5	PRLR
2.5	CD9
2.5	TPCN1
2.5	PTPRM
2.5	SEMA4B
2.5	ITFG1
2.5	LSAMP
2.5	CD63
2.5	ABCC3
2.5	ITGA3
2.5	ITGAV
2.5	ADAM10
2.5	SLC44A4
2.6	EFNA1
2.6	EREG
2.6	ALCAM
2.6	LSAMP
2.6	ITGB6
2.6	SLC22A23
2.6	CX3CL1
2.6	TSPAN5
2.6	ECE1
2.6	IFNGR2
2.6	ICAM1
2.6	ITGAV
2.6	PLAUR

2.6	SPPL2A
2.6	TFPI
2.6	ANO6
3	SLC4A7
3	IGSF1
3	SLC39A6
3	GLRA3
3	TSPAN5
3	CA12
0	HLA-DRA
0	CD74
0	HLA-DRB1
0	CSF2RA
0	HLA-DQA1
0	PLXDC2
0	SLC1A3
0	CD83
0	GPR183
0	ADAM28
0	PTPRC
0	PKD2
0	SLC2A3
0	CD53
0	PECAM1
0	HLA-DRB5
0	HLA-DPB1
0	MSR1
0	HLA-DPA1
0	NRP2
0	ATP13A3
0	LPAR6
0	ENTPD1
0	HLA-E
0	C5AR1
0	HLA-A
0	ABCA1
0	CLMP
0	IL2RA
0	PCNX
0	PLXNC1
0	NRP1
0	TNFRSF1B
0	ATP1B3
0	SLC8A1
0	CACNA1C
0	ROBO1
0	HAVCR2

0	FCGR2A
0	GPNMB
0	AXL
0	GJA1
0	DIRC2
0	CD86
0	PILRA
0	CALCRL
0	EDA
0	IL1RAP
0	CD80
0	IL15RA
0	C3AR1
0	GPR137B
0	HLA-DQB1
0	MCAM
0	PLVAP
0	P2RX7
0	ENG
0	PTGER4
0	MERTK
0	HLA-DQA2
0	FLT1
