# Supplementary material for: Ductal keratin 15+ luminal progenitors in normal breast exhibit a basal-like breast cancer transcriptomic signature
Source: NPJ Breast Cancer. 2022 Jul 12;8:81. doi: 10.1038/s41523-022-00444-8 (PMC9276673; doi:10.1038/s41523-022-00444-8)
Supplement: Supplementary file 5 — Dataset 3 [file 41523_2022_444_MOESM5_ESM.pdf]

Supplementary Data 3.

DEGs between ductal and TDLU derived luminal cells.
Data include DEGs between duct and TDLU-derived luminal cells with log2FC threshold of 0.25, cutoff of 25% for relative number of cells expressing a given gene in ducts or TDLUs and a threshold of 0.01 for the adjusted p-value.

Abbreviations:
- p_val = p-value
- avg_log2FC = average log2(fold change)
- pct.1 = percentage of cells in which the gene is detected in a given cluster
- pct.2 = percentage of cell in which the gene is detected in the rest of cells
- p_val_adj = adjusted p-value

	p_val	avg_log2FC	pct.1	pct.2	p_val_adj	cluster	gene
RPL10	0	1.819908231	0.949	0.912	0	lobular	RPL10
RPS3	0	1.81604144	0.884	0.843	0	lobular	RPS3
RPS11	0	1.653818756	0.894	0.564	0	lobular	RPS11
TMSB10	0	1.579126235	0.878	0.491	0	lobular	TMSB10
RPS12	0	1.41506119	0.737	0.503	0	lobular	RPS12
DIO2	0	1.371056782	0.31	0.083	0	lobular	DIO2
RPL19	0	1.369488293	0.93	0.855	0	lobular	RPL19
RPLP0	0	1.33754745	0.91	0.794	0	lobular	RPLP0
HPX	0	1.22775497	0.304	0.07	0	lobular	HPX
ANKRD30A	0	1.20772945	0.6	0.263	0	lobular	ANKRD30A
PTHLH	0	1.178957482	0.331	0.081	0	lobular	PTHLH
EIF2B5	0	1.151714614	0.55	0.315	0	lobular	EIF2B5
ERBB4	0	1.137604686	0.605	0.309	0	lobular	ERBB4
RPL10A	0	1.129483999	0.799	0.582	0	lobular	RPL10A
FTL	0	1.113596813	0.655	0.326	0	lobular	FTL
NBEAL1	0	1.055255649	0.828	0.529	0	lobular	NBEAL1
AREG	0	1.04445651	0.561	0.272	0	lobular	AREG
RPS18	0	0.988999102	0.756	0.436	0	lobular	RPS18
GNAS	0	0.977566622	0.839	0.61	0	lobular	GNAS
SERF2	0	0.956735644	0.527	0.176	0	lobular	SERF2
AMBRA1	0	0.955761452	0.787	0.494	0	lobular	AMBRA1
RPL15	0	0.949047938	0.867	0.631	0	lobular	RPL15
ACTG1	0	0.910288446	0.8	0.601	0	lobular	ACTG1
GNB2L1	0	0.905572278	0.772	0.506	0	lobular	GNB2L1
COX4I1	0	0.871950883	0.839	0.666	0	lobular	COX4I1
RPS8	0	0.845433614	0.828	0.587	0	lobular	RPS8
OST4	0	0.832455471	0.519	0.244	0	lobular	OST4
MT-ND1	0	0.577968263	0.991	0.961	0	lobular	MT-ND1
RPS14	0	0.558013124	0.519	0.234	0	lobular	RPS14
TPT1	0	0.540946218	0.907	0.761	0	lobular	TPT1
MT-ATP6	0	0.534050557	0.995	0.984	0	lobular	MT-ATP6
RPL37A	0	-0.406225757	0.971	0.98	0	lobular	RPL37A
EEF1A1	0	-0.481011136	0.97	0.983	0	lobular	EEF1A1
RPL39	0	-0.509772286	0.871	0.944	0	lobular	RPL39
RPL34	0	-0.592745866	0.975	0.983	0	lobular	RPL34
KIZ-AS1	0	-0.596703007	0.802	0.914	0	lobular	KIZ-AS1
LINC00486	0	-0.61251405	0.984	0.993	0	lobular	LINC00486
SLC47A1	0	-0.641508596	0.863	0.952	0	lobular	SLC47A1
RPL24	0	-0.676667684	0.894	0.954	0	lobular	RPL24
PLEKHA6	0	-0.716527974	0.787	0.91	0	lobular	PLEKHA6
SKP1	0	-0.718890034	0.767	0.875	0	lobular	SKP1
ADGRL3-AS	0	-0.729782258	0.521	0.729	0	lobular	ADGRL3-AS1

RBFOX2	0	-0.732646875	0.94	0.971	0	lobular	RBFOX2
NPM1	0	-0.749023387	0.642	0.836	0	lobular	NPM1
ANXA2	0	-0.774807154	0.917	0.943	0	lobular	ANXA2
SON	0	-0.813488169	0.623	0.777	0	lobular	SON
SEC61G	0	-0.862317764	0.524	0.727	0	lobular	SEC61G
RPS3A	0	-0.890992547	0.869	0.95	0	lobular	RPS3A
RP11-356C	0	-0.908074306	0.078	0.27	0	lobular	RP11-356C4.5
MET	0	-0.917931571	0.146	0.365	0	lobular	MET
RP11-795H	0	-1.002727826	0.273	0.512	0	lobular	RP11-795H16.3
UBC	0	-1.014091205	0.861	0.963	0	lobular	UBC
SOD2	0	-1.030661906	0.714	0.885	0	lobular	SOD2
GRB14	0	-1.067678487	0.323	0.556	0	lobular	GRB14
RPL21	0	-1.069539434	0.822	0.951	0	lobular	RPL21
PTEN	0	-1.072595202	0.759	0.89	0	lobular	PTEN
TSHZ2	0	-1.077660036	0.163	0.397	0	lobular	TSHZ2
PSME2	0	-1.089003029	0.198	0.456	0	lobular	PSME2
EPB42	0	-1.099491879	0.869	0.94	0	lobular	EPB42
PCNXL2	0	-1.117886167	0.705	0.864	0	lobular	PCNXL2
FAM172A	0	-1.129217956	0.541	0.749	0	lobular	FAM172A
PGK1	0	-1.153963297	0.465	0.709	0	lobular	PGK1
LTF	0	-1.189813971	0.244	0.513	0	lobular	LTF
ALDH1A3	0	-1.206707653	0.18	0.441	0	lobular	ALDH1A3
ANKRD36C	0	-1.213598799	0.356	0.633	0	lobular	ANKRD36C
STEAP1B	0	-1.243422608	0.922	0.954	0	lobular	STEAP1B
SDCBP	0	-1.275730213	0.528	0.77	0	lobular	SDCBP
RCAN1	0	-1.29291809	0.311	0.54	0	lobular	RCAN1
AKT3	0	-1.347825087	0.231	0.543	0	lobular	AKT3
B2M	0	-1.35009724	0.963	0.986	0	lobular	B2M
PDE4B	0	-1.521512584	0.411	0.698	0	lobular	PDE4B
FAM177B	0	-1.657631728	0.375	0.693	0	lobular	FAM177B
SLPI	0	-1.787375225	0.414	0.781	0	lobular	SLPI
ANXA1	0	-1.812768301	0.514	0.771	0	lobular	ANXA1
PTN	0	-2.024538586	0.018	0.275	0	lobular	PTN
TNFAIP6	0	-2.151492186	0.114	0.357	0	lobular	TNFAIP6
MGP	0	-2.262830937	0.74	0.952	0	lobular	MGP
CCL2	0	-2.725122155	0.148	0.447	0	lobular	CCL2
HINT1	7.47E-306	0.728819844	0.68	0.472	1.80E-301	lobular	HINT1
EIF4A2	5.87E-304	-0.785508174	0.644	0.787	1.41E-299	lobular	EIF4A2
PI3	7.87E-300	-1.72874024	0.078	0.264	1.90E-295	lobular	PI3
GCNT2	2.02E-296	1.073512747	0.537	0.294	4.87E-292	lobular	GCNT2
PSMA4	2.62E-296	-0.906113528	0.363	0.571	6.33E-292	lobular	PSMA4
MT-ND2	3.00E-296	0.425266432	0.988	0.959	7.23E-292	lobular	MT-ND2
SLC25A37	7.42E-296	-1.012555721	0.298	0.533	1.79E-291	lobular	SLC25A37
EREG	1.91E-295	1.129437377	0.349	0.119	4.60E-291	lobular	EREG
RPS20	2.44E-295	0.931541542	0.924	0.925	5.89E-291	lobular	RPS20
PRSS23	8.79E-295	0.927162491	0.304	0.087	2.12E-290	lobular	PRSS23
RPL35	2.23E-292	0.547413043	0.371	0.12	5.38E-288	lobular	RPL35

PTMA	9.27E-285	0.70340638	0.703	0.487	2.23E-280	lobular	PTMA
C8orf4	2.34E-284	0.70153973	0.635	0.332	5.64E-280	lobular	C8orf4
ALCAM	1.91E-279	1.006126082	0.597	0.375	4.62E-275	lobular	ALCAM
MT-CO3	4.71E-276	0.388237544	0.997	0.992	1.13E-271	lobular	MT-CO3
CPEB2	5.00E-274	0.918324418	0.397	0.167	1.21E-269	lobular	CPEB2
PRDX1	5.92E-274	-0.869905189	0.637	0.765	1.43E-269	lobular	PRDX1
FAM129A	7.40E-272	-1.19688608	0.197	0.402	1.79E-267	lobular	FAM129A
SLC26A3	2.34E-264	-0.696763643	0.746	0.864	5.65E-260	lobular	SLC26A3
EFHD1	6.92E-264	0.853468492	0.386	0.162	1.67E-259	lobular	EFHD1
AFF3	3.93E-261	1.109166937	0.426	0.199	9.49E-257	lobular	AFF3
ARRDC3	2.60E-256	-0.864813579	0.305	0.519	6.27E-252	lobular	ARRDC3
SEC62	5.01E-255	-0.643444507	0.511	0.693	1.21E-250	lobular	SEC62
POMP	2.82E-252	-0.688236785	0.579	0.723	6.80E-248	lobular	POMP
TNC	6.45E-252	0.980565083	0.415	0.197	1.55E-247	lobular	TNC
HSP90AB1	1.51E-249	-0.582403055	0.807	0.895	3.65E-245	lobular	HSP90AB1
RPS7	1.32E-247	-0.339920161	0.889	0.963	3.18E-243	lobular	RPS7
H2AFZ	4.11E-246	0.742461407	0.733	0.568	9.92E-242	lobular	H2AFZ
EFNA1	5.17E-246	0.902050068	0.316	0.115	1.25E-241	lobular	EFNA1
MYBPC1	1.35E-245	1.038135522	0.311	0.106	3.25E-241	lobular	MYBPC1
BIRC3	4.91E-244	-0.74761515	0.543	0.73	1.18E-239	lobular	BIRC3
RAB11FIP1	1.68E-243	0.930657497	0.781	0.658	4.04E-239	lobular	RAB11FIP1
CXCL13	5.97E-243	0.648141877	0.5	0.269	1.44E-238	lobular	CXCL13
KIAA1324	1.14E-238	0.702053588	0.304	0.106	2.75E-234	lobular	KIAA1324
RPL9	8.72E-237	-0.666902878	0.508	0.675	2.10E-232	lobular	RPL9
SMIM14	1.82E-236	0.781861995	0.48	0.269	4.39E-232	lobular	SMIM14
TBX3	2.11E-236	0.798212627	0.348	0.145	5.09E-232	lobular	TBX3
RPS6	4.18E-235	-0.40453666	0.974	0.985	1.01E-230	lobular	RPS6
MAML2	4.28E-234	-0.787481138	0.336	0.554	1.03E-229	lobular	MAML2
GLIS3	2.35E-231	-0.801683136	0.099	0.265	5.66E-227	lobular	GLIS3
SLC28A3	4.17E-231	-0.719107526	0.113	0.286	1.01E-226	lobular	SLC28A3
BTG1	1.39E-230	0.69733317	0.655	0.461	3.35E-226	lobular	BTG1
TSPAN5	2.32E-230	0.859026918	0.389	0.178	5.61E-226	lobular	TSPAN5
RASGEF1B	1.14E-229	-0.541126374	0.859	0.947	2.76E-225	lobular	RASGEF1B
ADAMTS9	1.36E-229	-1.003982756	0.144	0.329	3.29E-225	lobular	ADAMTS9
ST6GALNA(	5.46E-228	-0.975553795	0.122	0.297	1.32E-223	lobular	ST6GALNAC5
RNF181	6.48E-228	-0.702127179	0.315	0.51	1.56E-223	lobular	RNF181
FXYD3	1.44E-225	0.739965568	0.704	0.518	3.47E-221	lobular	FXYD3
SLC7A2	6.79E-225	0.771745132	0.38	0.173	1.64E-220	lobular	SLC7A2
C15orf48	1.16E-224	-1.303795184	0.436	0.606	2.80E-220	lobular	C15orf48
GABRP	5.48E-223	-0.873785178	0.195	0.385	1.32E-218	lobular	GABRP
SNU13	3.13E-222	0.64135365	0.556	0.347	7.55E-218	lobular	SNU13
UGCG	3.04E-220	0.679530662	0.569	0.36	7.34E-216	lobular	UGCG
HNRNPA1	4.60E-219	-0.665213457	0.713	0.836	1.11E-214	lobular	HNRNPA1
CPE	3.62E-211	0.738412275	0.273	0.094	8.73E-207	lobular	CPE
FGF13	7.94E-208	0.860223233	0.529	0.33	1.91E-203	lobular	FGF13
RPL11	2.21E-207	-0.309488245	0.947	0.98	5.32E-203	lobular	RPL11
DYNLRB1	7.63E-205	0.557545789	0.391	0.186	1.84E-200	lobular	DYNLRB1

MAP3K13	3.14E-204	-0.780528994	0.566	0.715	7.58E-200	lobular	MAP3K13
SOX4	4.06E-203	0.454278113	0.897	0.743	9.78E-199	lobular	SOX4
RPS17	9.15E-202	-0.384511078	0.927	0.964	2.21E-197	lobular	RPS17
RPS27A	3.48E-201	-0.273840503	0.962	0.985	8.39E-197	lobular	RPS27A
RPL37	4.54E-200	-0.333427488	0.933	0.967	1.09E-195	lobular	RPL37
ANKS1B	1.05E-199	0.768393564	0.536	0.338	2.52E-195	lobular	ANKS1B
SELK	9.39E-199	-0.601320794	0.553	0.711	2.26E-194	lobular	SELK
PSMA3	2.78E-198	-0.672356934	0.272	0.452	6.71E-194	lobular	PSMA3
STAT5B	4.42E-196	0.675069398	0.407	0.211	1.06E-191	lobular	STAT5B
APBB2	3.66E-191	0.786350288	0.326	0.147	8.81E-187	lobular	APBB2
LINGO1	1.31E-190	-0.48281975	0.86	0.938	3.16E-186	lobular	LINGO1
DAPP1	6.51E-188	-0.770088616	0.181	0.347	1.57E-183	lobular	DAPP1
FBLN5	3.24E-184	-1.165776883	0.112	0.259	7.82E-180	lobular	FBLN5
NDRG1	2.62E-183	-0.749438558	0.286	0.465	6.32E-179	lobular	NDRG1
MAP3K1	3.19E-183	0.754777275	0.53	0.342	7.69E-179	lobular	MAP3K1
EPS8	4.78E-183	-0.78619943	0.257	0.428	1.15E-178	lobular	EPS8
RPL31	2.16E-181	-0.39557886	0.905	0.958	5.22E-177	lobular	RPL31
ITGAV	6.23E-179	0.672675221	0.472	0.284	1.50E-174	lobular	ITGAV
COMMD6	3.98E-178	0.543039763	0.428	0.236	9.60E-174	lobular	COMMD6
ACTN1	1.59E-177	0.65542962	0.415	0.226	3.83E-173	lobular	ACTN1
SAA1	9.97E-177	0.30023426	0.681	0.816	2.40E-172	lobular	SAA1
CCL20	3.93E-176	-2.082814658	0.129	0.277	9.48E-172	lobular	CCL20
CALD1	1.76E-175	-0.902221841	0.233	0.409	4.23E-171	lobular	CALD1
PSMB7	2.46E-175	-0.639444538	0.394	0.554	5.92E-171	lobular	PSMB7
PSMC1	8.40E-175	-0.596301598	0.218	0.384	2.03E-170	lobular	PSMC1
GNA12	2.83E-174	-0.652016769	0.239	0.404	6.83E-170	lobular	GNA12
TAGLN2	2.90E-173	0.480140323	0.284	0.114	6.99E-169	lobular	TAGLN2
MYL12A	1.24E-172	-0.53777542	0.851	0.895	3.00E-168	lobular	MYL12A
CTSB	8.21E-172	0.626395151	0.357	0.183	1.98E-167	lobular	CTSB
RP1-78O14	5.39E-171	0.876106024	0.265	0.106	1.30E-166	lobular	RP1-78O14.1
CD59	1.01E-169	-0.686050045	0.874	0.897	2.44E-165	lobular	CD59
ELOVL5	1.02E-169	0.810120108	0.362	0.188	2.46E-165	lobular	ELOVL5
RP11-608O	1.06E-169	-0.631946125	0.476	0.645	2.55E-165	lobular	RP11-608O21.1
ARHGAP26	4.21E-169	-0.595104939	0.611	0.761	1.01E-164	lobular	ARHGAP26
CA8	1.04E-168	-0.791629386	0.177	0.336	2.50E-164	lobular	CA8
COX7C	1.01E-167	-0.346659587	0.856	0.902	2.43E-163	lobular	COX7C
RPL30	1.82E-166	-0.297807521	0.941	0.974	4.39E-162	lobular	RPL30
SEMA3C	4.42E-166	0.751627584	0.346	0.174	1.07E-161	lobular	SEMA3C
LYN	7.63E-166	-0.783722607	0.347	0.515	1.84E-161	lobular	LYN
NEBL	2.08E-165	0.654932969	0.72	0.571	5.01E-161	lobular	NEBL
DUSP16	7.36E-165	0.624347037	0.491	0.31	1.77E-160	lobular	DUSP16
RPLP1	6.91E-164	0.278187617	0.359	0.174	1.67E-159	lobular	RPLP1
RPL23	4.93E-163	-0.350625652	0.899	0.945	1.19E-158	lobular	RPL23
GSTM3	4.67E-162	0.885069579	0.394	0.222	1.13E-157	lobular	GSTM3
DNAJC12	1.51E-161	0.551734973	0.467	0.268	3.63E-157	lobular	DNAJC12
EFNA5	1.61E-161	-0.728056884	0.221	0.385	3.88E-157	lobular	EFNA5
RPL12	2.38E-161	0.540548362	0.352	0.18	5.75E-157	lobular	RPL12

MT-ND4	1.01E-160	0.324124623	0.995	0.991	2.43E-156	lobular	MT-ND4
SLC39A6	1.82E-159	0.577589293	0.369	0.195	4.39E-155	lobular	SLC39A6
RPS9	2.00E-158	0.300883311	0.295	0.123	4.83E-154	lobular	RPS9
RSL24D1	2.40E-158	-0.486832265	0.499	0.663	5.79E-154	lobular	RSL24D1
SLC27A4	2.82E-156	-0.840966834	0.656	0.787	6.80E-152	lobular	SLC27A4
PSD3	1.83E-154	0.701994758	0.412	0.242	4.42E-150	lobular	PSD3
CYP7B1	2.76E-154	-1.000354873	0.177	0.323	6.66E-150	lobular	CYP7B1
NCEH1	6.38E-154	0.746132164	0.476	0.313	1.54E-149	lobular	NCEH1
SUB1	1.02E-152	-0.503317303	0.591	0.718	2.47E-148	lobular	SUB1
NACA	1.25E-152	-0.268387352	0.887	0.945	3.01E-148	lobular	NACA
GAPDH	3.79E-152	0.44928616	0.632	0.457	9.14E-148	lobular	GAPDH
ERGIC3	3.93E-152	0.530297915	0.39	0.221	9.48E-148	lobular	ERGIC3
INSIG2	5.38E-152	-0.61660419	0.158	0.303	1.30E-147	lobular	INSIG2
SSR3	4.93E-151	-0.58985524	0.387	0.542	1.19E-146	lobular	SSR3
GLCCI1	7.04E-151	0.728450167	0.365	0.2	1.70E-146	lobular	GLCCI1
LUCAT1	3.73E-149	-0.637170112	0.149	0.296	8.99E-145	lobular	LUCAT1
LIPH	3.78E-149	-1.104610882	0.257	0.401	9.12E-145	lobular	LIPH
RPL35A	1.09E-148	-0.282018205	0.942	0.977	2.64E-144	lobular	RPL35A
XBP1	2.05E-148	0.746720838	0.455	0.289	4.95E-144	lobular	XBP1
PHLDB2	4.67E-148	0.647152125	0.484	0.315	1.13E-143	lobular	PHLDB2
ZFP36L2	1.06E-147	0.580034539	0.441	0.26	2.56E-143	lobular	ZFP36L2
TBC1D9	9.49E-147	0.565678292	0.517	0.34	2.29E-142	lobular	TBC1D9
DSTN	1.48E-146	0.571387199	0.645	0.486	3.57E-142	lobular	DSTN
SYPL1	2.81E-146	-0.545858312	0.315	0.469	6.79E-142	lobular	SYPL1
TMEM2	6.22E-146	0.705502468	0.412	0.244	1.50E-141	lobular	TMEM2
PIGR	9.70E-146	-0.798067409	0.193	0.345	2.34E-141	lobular	PIGR
HSP90AA1	1.69E-144	-0.428916187	0.829	0.898	4.07E-140	lobular	HSP90AA1
PVRL2	6.34E-144	0.594161244	0.346	0.185	1.53E-139	lobular	PVRL2
SNRPB2	7.90E-144	-0.536304124	0.288	0.441	1.90E-139	lobular	SNRPB2
SRRM1	1.61E-143	-0.508936276	0.483	0.629	3.89E-139	lobular	SRRM1
GLRX	2.14E-143	-0.720974518	0.232	0.384	5.15E-139	lobular	GLRX
MORF4L1	4.26E-143	-0.4640838	0.589	0.703	1.03E-138	lobular	MORF4L1
MDH1	5.42E-143	-0.551516358	0.22	0.372	1.31E-138	lobular	MDH1
TJP1	5.77E-143	0.594108106	0.57	0.407	1.39E-138	lobular	TJP1
TPK1	6.84E-143	-0.546156919	0.157	0.297	1.65E-138	lobular	TPK1
CFLAR	1.22E-142	-0.760128352	0.289	0.43	2.95E-138	lobular	CFLAR
ITGA2	1.44E-141	-0.787615698	0.335	0.483	3.48E-137	lobular	ITGA2
UGDH	1.95E-141	0.67422724	0.295	0.144	4.71E-137	lobular	UGDH
SHROOM3	4.02E-141	0.455570914	0.693	0.531	9.68E-137	lobular	SHROOM3
AIMP1	4.83E-140	-0.579739894	0.247	0.397	1.16E-135	lobular	AIMP1
ERBB2IP	2.34E-139	0.638782125	0.49	0.329	5.64E-135	lobular	ERBB2IP
TCF12	6.08E-139	0.464128449	0.656	0.493	1.47E-134	lobular	TCF12
RPS24	4.56E-138	-0.264749132	0.958	0.98	1.10E-133	lobular	RPS24
ZNF644	3.58E-137	0.620950011	0.533	0.371	8.62E-133	lobular	ZNF644
JUP	7.12E-137	0.505519355	0.325	0.17	1.72E-132	lobular	JUP
PAWR	1.70E-136	0.552275913	0.611	0.45	4.10E-132	lobular	PAWR
SARNP	4.17E-136	-0.521306309	0.149	0.285	1.00E-131	lobular	SARNP

NR6A1	6.70E-136	0.722521824	0.413	0.253	1.61E-131	lobular	NR6A1
SVIL	3.67E-135	-0.555515178	0.536	0.669	8.85E-131	lobular	SVIL
HIPK2	5.11E-135	0.507886587	0.436	0.273	1.23E-130	lobular	HIPK2
RPL36AL	6.20E-135	-0.339714751	0.869	0.917	1.50E-130	lobular	RPL36AL
INPP4B	1.12E-134	0.574756628	0.395	0.224	2.69E-130	lobular	INPP4B
ZBTB10	4.89E-134	0.563426585	0.315	0.164	1.18E-129	lobular	ZBTB10
USMG5	2.45E-133	-0.388347795	0.577	0.718	5.91E-129	lobular	USMG5
SYTL2	4.96E-133	0.494628622	0.523	0.337	1.20E-128	lobular	SYTL2
TULP4	5.88E-133	0.496578411	0.564	0.399	1.42E-128	lobular	TULP4
GADD45A	2.88E-132	0.591891956	0.325	0.173	6.95E-128	lobular	GADD45A
TM4SF1	3.02E-132	-0.820894879	0.901	0.917	7.27E-128	lobular	TM4SF1
DRAM1	1.10E-131	0.573174817	0.356	0.201	2.65E-127	lobular	DRAM1
MT-CYB	2.91E-131	0.250290392	0.993	0.988	7.02E-127	lobular	MT-CYB
TBL1XR1	1.27E-130	0.63412192	0.633	0.499	3.07E-126	lobular	TBL1XR1
LARP1B	6.62E-129	0.596218661	0.271	0.132	1.60E-124	lobular	LARP1B
DYNLL1	3.97E-128	0.51672953	0.301	0.154	9.57E-124	lobular	DYNLL1
MYL6	6.18E-128	-0.43021124	0.925	0.95	1.49E-123	lobular	MYL6
BCAR3	1.29E-126	0.670881335	0.335	0.191	3.11E-122	lobular	BCAR3
SERPINA3	3.28E-126	-0.719029256	0.216	0.35	7.90E-122	lobular	SERPINA3
BRK1	5.18E-126	-0.43935	0.563	0.683	1.25E-121	lobular	BRK1
ATP5G2	8.93E-126	0.551158505	0.767	0.691	2.15E-121	lobular	ATP5G2
MBNL2	1.59E-125	0.492649112	0.603	0.446	3.84E-121	lobular	MBNL2
RPL13	2.89E-125	0.47869586	0.482	0.316	6.97E-121	lobular	RPL13
TOM1L2	3.21E-125	0.552212901	0.358	0.209	7.74E-121	lobular	TOM1L2
RERE	4.49E-125	0.481728123	0.628	0.478	1.08E-120	lobular	RERE
DDX24	1.23E-123	-0.530661633	0.46	0.586	2.96E-119	lobular	DDX24
FRMD4A	1.68E-123	-0.691605137	0.226	0.365	4.05E-119	lobular	FRMD4A
RPL13A	3.21E-123	0.341798005	0.546	0.369	7.74E-119	lobular	RPL13A
RPL36	8.14E-123	0.43168049	0.254	0.112	1.96E-118	lobular	RPL36
ELF3	1.76E-122	0.452200548	0.574	0.415	4.25E-118	lobular	ELF3
WTAP	1.93E-121	-0.766635443	0.469	0.592	4.64E-117	lobular	WTAP
TNFAIP8	7.17E-120	-0.624425884	0.334	0.476	1.73E-115	lobular	TNFAIP8
DLGAP1	2.04E-118	0.65102927	0.255	0.123	4.92E-114	lobular	DLGAP1
CCNI	2.97E-118	0.376346244	0.684	0.536	7.15E-114	lobular	CCNI
NEDD9	5.73E-118	0.476799396	0.433	0.274	1.38E-113	lobular	NEDD9
GOLM1	3.39E-117	0.515295912	0.287	0.15	8.17E-113	lobular	GOLM1
CCL28	5.25E-116	-0.535635641	0.308	0.464	1.27E-111	lobular	CCL28
C8orf37-AS	7.76E-116	-0.455944106	0.196	0.332	1.87E-111	lobular	C8orf37-AS1
NUB1	1.54E-115	-0.669692942	0.148	0.269	3.71E-111	lobular	NUB1
PCBP2	2.40E-115	0.396421323	0.628	0.478	5.79E-111	lobular	PCBP2
FUT8	2.82E-115	0.614195043	0.314	0.175	6.80E-111	lobular	FUT8
IGF2BP2	4.09E-115	-0.678612181	0.223	0.355	9.87E-111	lobular	IGF2BP2
UQCR10	1.14E-114	0.486704002	0.564	0.417	2.76E-110	lobular	UQCR10
BCL2	1.20E-114	0.752650286	0.326	0.189	2.90E-110	lobular	BCL2
APLP2	2.11E-114	0.488435287	0.332	0.192	5.08E-110	lobular	APLP2
7-Sep	3.16E-114	-0.479972235	0.324	0.461	7.62E-110	lobular	7-Sep
OPHN1	5.42E-114	0.615259305	0.385	0.238	1.31E-109	lobular	OPHN1

PAN3	7.51E-114	0.448872024	0.691	0.552	1.81E-109	lobular	PAN3
CHCHD3	2.19E-113	0.424683539	0.855	0.807	5.28E-109	lobular	CHCHD3
TCEANC2	2.38E-113	-0.474153791	0.191	0.324	5.75E-109	lobular	TCEANC2
RAPH1	6.09E-113	0.588253214	0.391	0.25	1.47E-108	lobular	RAPH1
ARHGEF12	1.14E-112	0.409701619	0.637	0.481	2.76E-108	lobular	ARHGEF12
THRB	2.48E-111	0.632787913	0.513	0.377	5.99E-107	lobular	THRB
COX7A2	3.31E-111	-0.359896281	0.736	0.81	7.98E-107	lobular	COX7A2
PDCD10	6.96E-111	-0.485007289	0.205	0.334	1.68E-106	lobular	PDCD10
PCBP1	2.69E-110	0.405904835	0.563	0.411	6.48E-106	lobular	PCBP1
MT-ND3	5.06E-110	0.363454835	0.993	0.978	1.22E-105	lobular	MT-ND3
TMEM258	1.28E-109	-0.388372324	0.56	0.667	3.09E-105	lobular	TMEM258
CCT5	2.48E-109	-0.545364241	0.249	0.377	5.98E-105	lobular	CCT5
EIF3E	5.84E-109	-0.336318686	0.517	0.675	1.41E-104	lobular	EIF3E
DAD1	2.53E-108	-0.438874664	0.452	0.574	6.10E-104	lobular	DAD1
NFKBIZ	3.46E-108	-0.567429197	0.521	0.626	8.35E-104	lobular	NFKBIZ
CLDN1	8.19E-108	-0.86016884	0.184	0.304	1.98E-103	lobular	CLDN1
RORA	4.86E-107	0.629302975	0.622	0.494	1.17E-102	lobular	RORA
ILF2	5.15E-107	-0.516245772	0.383	0.512	1.24E-102	lobular	ILF2
NEDD4L	5.29E-107	0.534585889	0.651	0.514	1.27E-102	lobular	NEDD4L
PSMC2	4.61E-106	-0.408126065	0.173	0.299	1.11E-101	lobular	PSMC2
MAGI3	5.35E-106	0.494083714	0.439	0.294	1.29E-101	lobular	MAGI3
VPS29	1.19E-105	-0.479581926	0.273	0.403	2.86E-101	lobular	VPS29
WFDC2	8.07E-104	-1.256736964	0.277	0.407	1.95E-99	lobular	WFDC2
IK	1.37E-102	-0.403866628	0.143	0.261	3.31E-98	lobular	IK
UBB	6.19E-102	-0.313924224	0.685	0.784	1.49E-97	lobular	UBB
RPL29	2.18E-101	0.450430826	0.288	0.152	5.26E-97	lobular	RPL29
KTN1	6.26E-101	0.45900334	0.593	0.461	1.51E-96	lobular	KTN1
RARRES3	2.16E-100	-0.551553656	0.176	0.294	5.22E-96	lobular	RARRES3
KIAA1522	2.52E-100	0.404204919	0.279	0.15	6.08E-96	lobular	KIAA1522
TAOK3	6.55E-100	0.453811172	0.527	0.389	1.58E-95	lobular	TAOK3
GSPT1	8.14E-100	0.471803867	0.421	0.286	1.96E-95	lobular	GSPT1
TANK	1.43E-99	-0.469647042	0.429	0.552	3.45E-95	lobular	TANK
HIST1H2AC	2.27E-99	0.404669815	0.422	0.274	5.47E-95	lobular	HIST1H2AC
RANBP9	2.45E-99	0.445225884	0.35	0.217	5.91E-95	lobular	RANBP9
BCL6	2.47E-99	0.475456504	0.412	0.272	5.96E-95	lobular	BCL6
GBP2	7.89E-99	-0.521539881	0.578	0.679	1.90E-94	lobular	GBP2
DEFB1	3.33E-98	-0.324502309	0.382	0.538	8.03E-94	lobular	DEFB1
MYH9	3.38E-98	0.429572375	0.571	0.425	8.16E-94	lobular	MYH9
FLNB	4.39E-98	0.441549362	0.515	0.365	1.06E-93	lobular	FLNB
EGFR	1.29E-97	-0.501522331	0.138	0.25	3.12E-93	lobular	EGFR
PRLR	1.56E-97	0.50541528	0.285	0.16	3.75E-93	lobular	PRLR
DGKH	6.04E-97	0.535177422	0.359	0.228	1.46E-92	lobular	DGKH
OOEP	1.05E-96	-0.26003172	0.958	0.978	2.53E-92	lobular	OOEP
C4orf3	1.21E-96	-0.422379174	0.493	0.61	2.91E-92	lobular	C4orf3
SLIRP	2.50E-96	-0.386596175	0.261	0.394	6.03E-92	lobular	SLIRP
COL4A5	5.63E-96	0.476551275	0.267	0.145	1.36E-91	lobular	COL4A5
KCTD9	5.71E-96	-0.569250056	0.192	0.306	1.38E-91	lobular	KCTD9

SSBP1	7.12E-96	-0.491813825	0.317	0.436	1.72E-91	lobular	SSBP1
UBL5	1.12E-95	0.527060584	0.554	0.439	2.71E-91	lobular	UBL5
LRBA	1.72E-95	0.419550399	0.689	0.559	4.15E-91	lobular	LRBA
PKP4	2.02E-95	0.447764536	0.555	0.419	4.87E-91	lobular	PKP4
FARP1	2.55E-95	0.44578121	0.434	0.294	6.16E-91	lobular	FARP1
AGR2	2.57E-95	0.30205311	0.296	0.161	6.20E-91	lobular	AGR2
CDCP1	3.00E-95	-0.480003646	0.234	0.355	7.24E-91	lobular	CDCP1
SSBP2	1.39E-94	0.548998443	0.486	0.358	3.36E-90	lobular	SSBP2
ENAH	1.94E-94	0.382229916	0.606	0.467	4.67E-90	lobular	ENAH
ATP8B1	2.53E-94	0.563727092	0.454	0.325	6.10E-90	lobular	ATP8B1
ACTB	1.36E-93	0.504181829	0.691	0.566	3.29E-89	lobular	ACTB
NCOA7	1.69E-93	-0.528021603	0.392	0.523	4.07E-89	lobular	NCOA7
TAX1BP1	7.84E-93	-0.387708739	0.591	0.683	1.89E-88	lobular	TAX1BP1
KLHL5	1.35E-92	0.49024559	0.333	0.21	3.26E-88	lobular	KLHL5
INO80D	1.47E-92	-0.433569653	0.525	0.643	3.55E-88	lobular	INO80D
INTS6	2.42E-92	0.482099005	0.331	0.205	5.83E-88	lobular	INTS6
RBM8A	2.64E-92	-0.451991592	0.388	0.504	6.37E-88	lobular	RBM8A
UGP2	1.13E-91	-0.445413039	0.393	0.51	2.72E-87	lobular	UGP2
RASEF	1.14E-91	0.443556131	0.322	0.195	2.74E-87	lobular	RASEF
RNF145	2.85E-91	-0.510498639	0.293	0.416	6.88E-87	lobular	RNF145
ERH	1.18E-90	-0.431215201	0.467	0.572	2.83E-86	lobular	ERH
MTHFD2L	3.89E-90	-0.686735803	0.359	0.476	9.38E-86	lobular	MTHFD2L
KAZN	7.31E-90	0.478279517	0.263	0.146	1.76E-85	lobular	KAZN
GAB1	1.03E-89	0.405442753	0.437	0.302	2.48E-85	lobular	GAB1
CUX1	1.40E-89	0.453569364	0.399	0.271	3.38E-85	lobular	CUX1
CD24	1.60E-89	0.490042078	0.628	0.502	3.87E-85	lobular	CD24
REEP5	3.74E-89	0.419704702	0.3	0.179	9.03E-85	lobular	REEP5
C16orf45	1.21E-88	0.490665897	0.274	0.157	2.91E-84	lobular	C16orf45
LDHA	1.48E-88	-0.40430431	0.631	0.732	3.57E-84	lobular	LDHA
NAALADL2	2.03E-88	0.519389727	0.55	0.428	4.90E-84	lobular	NAALADL2
LPP	2.62E-88	0.283221099	0.933	0.886	6.33E-84	lobular	LPP
ATP6V0E1	4.02E-88	0.336563524	0.734	0.628	9.69E-84	lobular	ATP6V0E1
UBN2	9.17E-88	0.422434202	0.323	0.197	2.21E-83	lobular	UBN2
WWC1	3.70E-87	0.425853786	0.47	0.337	8.91E-83	lobular	WWC1
GNB1	5.04E-87	0.376568774	0.395	0.266	1.22E-82	lobular	GNB1
RPLP2	9.97E-87	0.272581828	0.273	0.149	2.40E-82	lobular	RPLP2
RPS16	1.19E-86	0.269740605	0.361	0.225	2.87E-82	lobular	RPS16
OSBPL3	1.76E-86	0.430694264	0.277	0.159	4.25E-82	lobular	OSBPL3
DDX18	2.43E-86	-0.452548244	0.247	0.362	5.86E-82	lobular	DDX18
BTRC	2.85E-86	0.418013619	0.311	0.188	6.87E-82	lobular	BTRC
DCTN6	6.92E-86	-0.410005218	0.236	0.353	1.67E-81	lobular	DCTN6
MTRNR2L1	1.45E-85	-0.445570476	0.182	0.296	3.49E-81	lobular	MTRNR2L12
ATP6V1D	5.48E-85	-0.447507412	0.234	0.348	1.32E-80	lobular	ATP6V1D
NOS1AP	7.35E-85	0.392018069	0.262	0.146	1.77E-80	lobular	NOS1AP
ARIH1	7.94E-85	0.358342719	0.558	0.428	1.91E-80	lobular	ARIH1
CADPS2	2.76E-84	0.812529508	0.45	0.33	6.65E-80	lobular	CADPS2
ACSL3	5.42E-84	0.502507057	0.385	0.259	1.31E-79	lobular	ACSL3

DLG5	1.56E-83	0.507795065	0.308	0.191	3.77E-79	lobular	DLG5
PSMB5	1.88E-83	0.342252129	0.286	0.168	4.53E-79	lobular	PSMB5
DNAJB6	2.07E-83	0.410855487	0.432	0.304	5.00E-79	lobular	DNAJB6
ARF4	3.74E-83	-0.381664297	0.587	0.675	9.03E-79	lobular	ARF4
CDH1	6.26E-83	0.381034973	0.651	0.533	1.51E-78	lobular	CDH1
STOM	1.00E-82	0.483360414	0.307	0.191	2.42E-78	lobular	STOM
CDK13	1.37E-82	0.469812038	0.472	0.348	3.31E-78	lobular	CDK13
ESD	1.40E-82	-0.409925162	0.271	0.39	3.37E-78	lobular	ESD
EIF3H	2.27E-82	0.356386075	0.662	0.566	5.47E-78	lobular	EIF3H
PROM1	4.06E-82	-0.482184222	0.187	0.298	9.79E-78	lobular	PROM1
FNBP1	9.27E-82	0.358976005	0.579	0.443	2.24E-77	lobular	FNBP1
ZNF292	1.22E-81	-0.380201177	0.526	0.631	2.94E-77	lobular	ZNF292
MAP4	1.45E-81	0.382585727	0.53	0.402	3.51E-77	lobular	MAP4
MSI2	1.88E-81	0.421525241	0.391	0.268	4.54E-77	lobular	MSI2
BARX2	3.33E-81	-0.576248021	0.212	0.318	8.03E-77	lobular	BARX2
SLC38A2	4.41E-81	0.448864111	0.432	0.302	1.06E-76	lobular	SLC38A2
SLC4A7	4.57E-81	-0.460169691	0.239	0.353	1.10E-76	lobular	SLC4A7
FEZ2	5.04E-81	-0.524341362	0.301	0.409	1.22E-76	lobular	FEZ2
HERC4	6.71E-81	0.4540561	0.574	0.454	1.62E-76	lobular	HERC4
GSK3B	8.18E-81	0.404604487	0.448	0.322	1.97E-76	lobular	GSK3B
SLCO3A1	1.91E-80	-0.456573255	0.148	0.25	4.60E-76	lobular	SLCO3A1
SPRED2	6.08E-80	0.403690653	0.255	0.145	1.47E-75	lobular	SPRED2
RUFY3	6.68E-80	0.359411244	0.284	0.168	1.61E-75	lobular	RUFY3
ZCCHC17	7.01E-80	-0.442164769	0.219	0.327	1.69E-75	lobular	ZCCHC17
ZFAND3	1.14E-79	0.379621661	0.79	0.714	2.76E-75	lobular	ZFAND3
C1QTNF3-A	3.19E-79	0.470015208	0.366	0.244	7.70E-75	lobular	C1QTNF3-AMACR
EIF2S1	3.21E-79	-0.410920679	0.162	0.264	7.74E-75	lobular	EIF2S1
UBE2V2	7.87E-79	-0.396879293	0.211	0.319	1.90E-74	lobular	UBE2V2
AC072062.	1.10E-78	0.377281525	0.523	0.391	2.66E-74	lobular	AC072062.1
PPP6R3	2.58E-78	0.37005294	0.555	0.434	6.22E-74	lobular	PPP6R3
MKL1	6.95E-78	0.355053666	0.546	0.417	1.68E-73	lobular	MKL1
DYNLT1	8.66E-78	-0.42050235	0.567	0.644	2.09E-73	lobular	DYNLT1
DLEU2	1.56E-77	0.402673056	0.264	0.155	3.75E-73	lobular	DLEU2
SRSF3	3.37E-77	-0.565243137	0.396	0.496	8.13E-73	lobular	SRSF3
TTLL5	4.53E-77	0.43249548	0.328	0.212	1.09E-72	lobular	TTLL5
TSC22D2	5.79E-77	0.356861176	0.518	0.386	1.40E-72	lobular	TSC22D2
SH3BGRL	4.25E-76	0.447215606	0.448	0.329	1.02E-71	lobular	SH3BGRL
FOXP1	6.62E-76	0.424250935	0.673	0.574	1.60E-71	lobular	FOXP1
CTNND1	9.54E-76	0.345144908	0.444	0.319	2.30E-71	lobular	CTNND1
MAST4	1.00E-75	0.434557449	0.575	0.454	2.42E-71	lobular	MAST4
RBM47	1.29E-75	0.349722532	0.681	0.566	3.11E-71	lobular	RBM47
TBL1X	2.32E-75	0.340851443	0.259	0.149	5.60E-71	lobular	TBL1X
SUSD6	3.32E-75	0.386732495	0.324	0.208	8.01E-71	lobular	SUSD6
RP1-292B1	6.50E-75	0.301038952	0.257	0.146	1.57E-70	lobular	RP1-292B18.4
YTHDC1	8.49E-75	0.37700193	0.432	0.309	2.05E-70	lobular	YTHDC1
ANKRD11	9.31E-75	0.33368852	0.368	0.246	2.24E-70	lobular	ANKRD11
EIF4A1	1.36E-73	-0.428739541	0.364	0.472	3.27E-69	lobular	EIF4A1

NHSL2	1.57E-73	-0.378770636	0.24	0.349	3.80E-69	lobular	NHSL2
COX7B	9.38E-73	-0.336622942	0.605	0.682	2.26E-68	lobular	COX7B
DUSP10	1.28E-72	0.400883575	0.265	0.16	3.09E-68	lobular	DUSP10
PRRG4	2.48E-72	0.363336491	0.308	0.195	5.99E-68	lobular	PRRG4
ATP6V1G1	2.70E-72	0.328154719	0.437	0.315	6.50E-68	lobular	ATP6V1G1
ADAM32	1.00E-71	0.424609956	0.272	0.164	2.42E-67	lobular	ADAM32
EIF3K	1.21E-71	0.550467978	0.367	0.274	2.92E-67	lobular	EIF3K
GPBP1	3.55E-71	0.392882748	0.722	0.635	8.56E-67	lobular	GPBP1
KAT6B	5.29E-71	0.37503208	0.3	0.189	1.28E-66	lobular	KAT6B
CTC-471J1.	7.00E-71	-0.67169315	0.624	0.658	1.69E-66	lobular	CTC-471J1.9
ARPC3	7.82E-71	-0.360661854	0.64	0.7	1.89E-66	lobular	ARPC3
ARFGEF2	1.20E-70	0.364197836	0.419	0.299	2.90E-66	lobular	ARFGEF2
OSBPL8	2.18E-70	0.376032448	0.281	0.174	5.26E-66	lobular	OSBPL8
KLHL24	2.24E-70	0.316633558	0.398	0.276	5.39E-66	lobular	KLHL24
ATF4	3.34E-70	0.351201588	0.442	0.323	8.05E-66	lobular	ATF4
CNOT4	9.94E-70	0.329247608	0.447	0.325	2.40E-65	lobular	CNOT4
CREBBP	2.05E-69	0.330113735	0.345	0.23	4.95E-65	lobular	CREBBP
CDC42BPA	3.63E-69	0.377587903	0.428	0.31	8.75E-65	lobular	CDC42BPA
KCMF1	4.18E-69	0.348701844	0.383	0.265	1.01E-64	lobular	KCMF1
HNRNPM	8.59E-69	0.332364696	0.305	0.195	2.07E-64	lobular	HNRNPM
YAP1	1.09E-68	0.337252193	0.579	0.462	2.63E-64	lobular	YAP1
RTCB	1.74E-68	-0.386129845	0.371	0.474	4.20E-64	lobular	RTCB
UFM1	2.73E-68	-0.384581761	0.287	0.391	6.59E-64	lobular	UFM1
ITGB8	3.82E-68	-0.440922639	0.461	0.559	9.20E-64	lobular	ITGB8
ZFAS1	4.18E-68	-0.267848021	0.915	0.951	1.01E-63	lobular	ZFAS1
SMARCC1	6.15E-68	0.335814414	0.334	0.222	1.48E-63	lobular	SMARCC1
RP5-857K2	6.73E-68	0.328233908	0.481	0.359	1.62E-63	lobular	RP5-857K21.4
RPL41	8.17E-68	-0.679637482	0.882	0.938	1.97E-63	lobular	RPL41
BACE2	1.25E-67	-0.43387141	0.275	0.377	3.03E-63	lobular	BACE2
CFL1	1.42E-67	0.411913007	0.359	0.251	3.42E-63	lobular	CFL1
EEF2	3.38E-67	0.41170816	0.458	0.333	8.15E-63	lobular	EEF2
POLR1D	3.41E-67	0.319631971	0.265	0.163	8.22E-63	lobular	POLR1D
TOP1	6.79E-67	0.333556748	0.491	0.375	1.64E-62	lobular	TOP1
DPM1	7.65E-67	-0.36594229	0.207	0.307	1.84E-62	lobular	DPM1
TIAM1	9.15E-67	-0.549401867	0.207	0.304	2.21E-62	lobular	TIAM1
CD63	1.28E-66	0.394953942	0.425	0.315	3.08E-62	lobular	CD63
UBE2G1	1.31E-66	0.326361973	0.299	0.191	3.17E-62	lobular	UBE2G1
BRD2	1.94E-66	0.338173464	0.311	0.204	4.69E-62	lobular	BRD2
SDC4	2.14E-66	-0.612065958	0.295	0.388	5.15E-62	lobular	SDC4
UBR5	2.45E-66	0.322630938	0.516	0.398	5.92E-62	lobular	UBR5
OTUD7B	2.59E-66	0.360230716	0.338	0.227	6.24E-62	lobular	OTUD7B
FGD6	2.72E-66	0.371667258	0.388	0.276	6.55E-62	lobular	FGD6
GBE1	2.78E-66	-0.512521194	0.322	0.424	6.69E-62	lobular	GBE1
COX6C	3.26E-66	0.689053859	0.769	0.754	7.87E-62	lobular	COX6C
USP47	3.64E-66	0.366079634	0.466	0.35	8.78E-62	lobular	USP47
WNK1	4.52E-66	0.31773578	0.282	0.178	1.09E-61	lobular	WNK1
SNRPD1	5.07E-66	-0.396256697	0.249	0.35	1.22E-61	lobular	SNRPD1

EIF3L	5.16E-66	-0.27701116	0.547	0.662	1.25E-61	lobular	EIF3L
KIF13A	6.49E-66	0.437381135	0.44	0.326	1.56E-61	lobular	KIF13A
CNKSR3	7.26E-66	-0.693051529	0.274	0.366	1.75E-61	lobular	CNKSR3
TMEM150(	2.56E-65	-0.357798725	0.173	0.265	6.17E-61	lobular	TMEM150C
CCT2	9.07E-65	-0.391327021	0.267	0.366	2.19E-60	lobular	CCT2
SLC38A1	1.28E-64	0.348888963	0.437	0.325	3.10E-60	lobular	SLC38A1
PRDX3	1.71E-64	0.345133623	0.264	0.164	4.12E-60	lobular	PRDX3
RPL7	3.97E-64	0.345036864	0.835	0.946	9.57E-60	lobular	RPL7
PDZD8	5.26E-64	0.382455811	0.27	0.171	1.27E-59	lobular	PDZD8
S100A6	1.22E-63	0.828898269	0.601	0.532	2.93E-59	lobular	S100A6
SSR2	1.59E-63	-0.285113826	0.658	0.733	3.84E-59	lobular	SSR2
VAV3	3.74E-63	0.390812032	0.327	0.222	9.03E-59	lobular	VAV3
15-Sep	3.81E-63	-0.370064982	0.38	0.473	9.19E-59	lobular	15-Sep
RBX1	4.11E-63	-0.365587557	0.406	0.497	9.92E-59	lobular	RBX1
SARAF	1.10E-62	0.291143604	0.398	0.282	2.64E-58	lobular	SARAF
ZSWIM6	1.24E-62	0.367812514	0.54	0.432	2.98E-58	lobular	ZSWIM6
ASH1L	1.94E-62	0.3261521	0.593	0.484	4.67E-58	lobular	ASH1L
EIF1	7.46E-62	0.296656839	0.526	0.408	1.80E-57	lobular	EIF1
C8orf59	1.37E-61	-0.336580474	0.25	0.35	3.31E-57	lobular	C8orf59
IGF1R	1.75E-61	0.316766544	0.548	0.433	4.22E-57	lobular	IGF1R
KYNU	2.49E-61	-0.772374108	0.208	0.301	6.01E-57	lobular	KYNU
HNRNPH2	2.50E-61	-0.332533779	0.191	0.286	6.04E-57	lobular	HNRNPH2
SLC12A2	2.58E-61	-0.590868554	0.377	0.466	6.23E-57	lobular	SLC12A2
SEMA6A	2.60E-61	-0.570973372	0.19	0.28	6.27E-57	lobular	SEMA6A
ZRANB2	2.91E-61	-0.359761058	0.198	0.291	7.02E-57	lobular	ZRANB2
ELF2	3.10E-61	0.353852976	0.431	0.322	7.48E-57	lobular	ELF2
EP300	3.18E-61	0.321342561	0.258	0.161	7.68E-57	lobular	EP300
PAM	5.20E-61	-0.376534008	0.433	0.527	1.25E-56	lobular	PAM
AHNAK	1.30E-60	0.333898468	0.399	0.29	3.14E-56	lobular	AHNAK
RUNX1	1.34E-60	0.282112912	0.685	0.579	3.23E-56	lobular	RUNX1
DNAJC8	2.44E-60	-0.337943968	0.186	0.28	5.89E-56	lobular	DNAJC8
SRPK1	2.94E-60	-0.407612754	0.455	0.542	7.09E-56	lobular	SRPK1
CD164	4.37E-60	0.340335703	0.322	0.221	1.05E-55	lobular	CD164
USP53	4.83E-60	-0.61077	0.603	0.661	1.16E-55	lobular	USP53
LRRFIP1	6.86E-60	0.254577817	0.675	0.559	1.65E-55	lobular	LRRFIP1
CRY1	8.29E-60	0.384597211	0.428	0.319	2.00E-55	lobular	CRY1
RBBP6	1.51E-59	0.307506286	0.322	0.217	3.63E-55	lobular	RBBP6
UBE2K	1.55E-59	0.315285432	0.424	0.312	3.73E-55	lobular	UBE2K
FBXO32	2.09E-59	-0.57727673	0.336	0.428	5.04E-55	lobular	FBXO32
ZFAND5	2.28E-59	0.318694219	0.477	0.367	5.50E-55	lobular	ZFAND5
PERP	2.57E-59	0.288542672	0.498	0.385	6.19E-55	lobular	PERP
RARS	2.58E-59	-0.339006279	0.189	0.28	6.23E-55	lobular	RARS
C2orf88	3.29E-59	-0.396925484	0.315	0.41	7.93E-55	lobular	C2orf88
BCAS3	3.95E-59	-0.264959421	0.716	0.777	9.52E-55	lobular	BCAS3
SLTM	5.57E-59	0.303155991	0.335	0.231	1.34E-54	lobular	SLTM
HSPH1	7.38E-59	0.426455969	0.324	0.223	1.78E-54	lobular	HSPH1
SREBF2	7.91E-59	0.31370641	0.421	0.309	1.91E-54	lobular	SREBF2

SNRPG	9.45E-59	-0.358911885	0.49	0.568	2.28E-54	lobular	SNRPG
TGIF1	1.03E-58	0.342257951	0.256	0.163	2.48E-54	lobular	TGIF1
ATP5E	1.49E-58	0.457504292	0.59	0.502	3.59E-54	lobular	ATP5E
RNMT	1.84E-58	-0.359417608	0.293	0.393	4.43E-54	lobular	RNMT
PTPRE	3.71E-58	0.464966454	0.302	0.208	8.95E-54	lobular	PTPRE
PPP2CB	3.77E-58	0.294101567	0.381	0.272	9.08E-54	lobular	PPP2CB
LIMCH1	3.87E-58	0.415757283	0.525	0.428	9.33E-54	lobular	LIMCH1
MYO1B	9.41E-58	0.405637755	0.488	0.383	2.27E-53	lobular	MYO1B
ATXN2	1.15E-57	0.302182501	0.376	0.269	2.77E-53	lobular	ATXN2
CD46	1.21E-57	0.299237454	0.446	0.333	2.92E-53	lobular	CD46
SMCHD1	1.67E-57	0.375203505	0.447	0.346	4.02E-53	lobular	SMCHD1
MAN1A1	2.60E-57	0.316360125	0.265	0.17	6.28E-53	lobular	MAN1A1
AC018890.	2.69E-57	0.319757162	0.277	0.18	6.49E-53	lobular	AC018890.6
GRAMD3	2.71E-57	0.351329783	0.389	0.282	6.54E-53	lobular	GRAMD3
DMD	3.97E-57	-0.4739159	0.228	0.318	9.56E-53	lobular	DMD
CD9	4.09E-57	0.371834893	0.427	0.324	9.85E-53	lobular	CD9
TMEM87A	4.71E-57	0.274517159	0.428	0.317	1.14E-52	lobular	TMEM87A
ATP6V1E1	7.23E-57	-0.340171141	0.26	0.353	1.74E-52	lobular	ATP6V1E1
ATF3	1.50E-56	0.339961322	0.449	0.34	3.62E-52	lobular	ATF3
CTNNA1	1.91E-56	0.28831696	0.578	0.475	4.59E-52	lobular	CTNNA1
PLCB1	2.89E-56	-0.489759923	0.192	0.28	6.98E-52	lobular	PLCB1
DTNB	3.14E-56	-0.378518831	0.392	0.489	7.58E-52	lobular	DTNB
KLF6	3.90E-56	0.314009513	0.611	0.507	9.41E-52	lobular	KLF6
SRSF4	4.20E-56	0.277307052	0.334	0.232	1.01E-51	lobular	SRSF4
RB1CC1	6.14E-56	0.305585423	0.524	0.416	1.48E-51	lobular	RB1CC1
CSNK1A1	7.96E-56	0.288447235	0.693	0.595	1.92E-51	lobular	CSNK1A1
RNF11	9.05E-56	0.290266083	0.284	0.188	2.18E-51	lobular	RNF11
GABARAP	1.11E-55	-0.359018421	0.327	0.424	2.68E-51	lobular	GABARAP
ARHGEF28	1.34E-55	-0.492235725	0.241	0.332	3.24E-51	lobular	ARHGEF28
CCT3	1.75E-55	0.298709587	0.4	0.298	4.23E-51	lobular	CCT3
SLC22A23	1.79E-55	0.308593537	0.286	0.189	4.31E-51	lobular	SLC22A23
BAZ1A	2.97E-55	0.372713575	0.424	0.322	7.17E-51	lobular	BAZ1A
UBE2R2	3.19E-55	0.285745494	0.411	0.305	7.69E-51	lobular	UBE2R2
EIF3I	3.54E-55	-0.303688986	0.567	0.643	8.53E-51	lobular	EIF3I
PTPRK	8.31E-55	0.293830099	0.794	0.735	2.00E-50	lobular	PTPRK
CAB39	9.01E-55	0.343557757	0.4	0.301	2.17E-50	lobular	CAB39
LAMC2	1.18E-54	-0.369437658	0.23	0.324	2.84E-50	lobular	LAMC2
TMBIM6	1.23E-54	-0.328525313	0.854	0.873	2.96E-50	lobular	TMBIM6
RNF144B	1.72E-54	-0.489476468	0.256	0.343	4.14E-50	lobular	RNF144B
RAB21	1.92E-54	0.31337929	0.301	0.203	4.62E-50	lobular	RAB21
YY1	2.15E-54	0.274179961	0.257	0.165	5.17E-50	lobular	YY1
POLE2	2.50E-54	-0.314658256	0.205	0.297	6.02E-50	lobular	POLE2
MAP2K4	2.60E-54	-0.363322234	0.412	0.498	6.27E-50	lobular	MAP2K4
GPBP1L1	2.72E-54	0.292503517	0.384	0.281	6.56E-50	lobular	GPBP1L1
CTTNBP2NI	3.27E-54	-0.346776759	0.18	0.265	7.87E-50	lobular	CTTNBP2NL
SCGB2A2	3.32E-54	0.506063487	0.314	0.21	8.02E-50	lobular	SCGB2A2
CLIC6	4.39E-54	0.374053549	0.252	0.164	1.06E-49	lobular	CLIC6

SEMA4B	5.32E-54	0.272551279	0.26	0.167	1.28E-49	lobular	SEMA4B
ITPR2	6.63E-54	0.3540992	0.513	0.412	1.60E-49	lobular	ITPR2
ANO6	9.35E-54	0.342447654	0.556	0.463	2.25E-49	lobular	ANO6
CIR1	1.33E-53	-0.327803467	0.224	0.314	3.20E-49	lobular	CIR1
NAV2	1.74E-53	-0.44988989	0.383	0.473	4.20E-49	lobular	NAV2
MAN2A1	5.95E-53	0.291929364	0.359	0.258	1.44E-48	lobular	MAN2A1
PDK3	7.56E-53	-0.454648192	0.198	0.282	1.82E-48	lobular	PDK3
MAP4K4	1.02E-52	-0.429673366	0.205	0.289	2.47E-48	lobular	MAP4K4
TBC1D3P1-	1.18E-52	-0.436448065	0.56	0.638	2.85E-48	lobular	TBC1D3P1-DHX40P1
PIP5K1A	1.31E-52	0.294625731	0.341	0.242	3.15E-48	lobular	PIP5K1A
RALGAPA1	1.41E-52	0.334701928	0.342	0.247	3.39E-48	lobular	RALGAPA1
CXCR4	2.07E-52	-0.399549607	0.187	0.272	5.00E-48	lobular	CXCR4
CTBP2	2.76E-52	0.264724044	0.386	0.28	6.65E-48	lobular	CTBP2
RNF10	3.35E-52	0.283929868	0.26	0.171	8.07E-48	lobular	RNF10
ZBTB16	4.34E-52	0.318670367	0.314	0.216	1.05E-47	lobular	ZBTB16
PTPN14	4.44E-52	-0.462102745	0.271	0.356	1.07E-47	lobular	PTPN14
YBX3	5.27E-52	0.281849781	0.324	0.227	1.27E-47	lobular	YBX3
CREBRF	6.71E-52	0.272659336	0.302	0.207	1.62E-47	lobular	CREBRF
AC090498.	1.29E-51	-0.33552784	0.275	0.363	3.11E-47	lobular	AC090498.1
CYCS	1.34E-51	-0.332060535	0.493	0.569	3.23E-47	lobular	CYCS
ATP5J	2.98E-51	-0.298744467	0.47	0.551	7.19E-47	lobular	ATP5J
RAB7A	3.49E-51	0.277165542	0.491	0.39	8.41E-47	lobular	RAB7A
CHCHD2	4.14E-51	0.346104827	0.291	0.201	9.97E-47	lobular	CHCHD2
LAMB3	5.67E-51	-0.4646258	0.209	0.294	1.37E-46	lobular	LAMB3
SRRM2	7.84E-51	0.266948921	0.251	0.163	1.89E-46	lobular	SRRM2
EEF1B2	1.92E-50	0.641554188	0.726	0.736	4.64E-46	lobular	EEF1B2
CBLB	2.98E-50	0.312418465	0.359	0.262	7.19E-46	lobular	CBLB
RARRES1	3.20E-50	-0.453293524	0.181	0.266	7.72E-46	lobular	RARRES1
AKAP13	4.75E-50	0.329719185	0.646	0.557	1.14E-45	lobular	AKAP13
GNG12	5.68E-50	0.30933461	0.366	0.269	1.37E-45	lobular	GNG12
MYO10	1.25E-49	0.329137703	0.289	0.199	3.01E-45	lobular	MYO10
UBE2L3	2.04E-49	-0.329044089	0.41	0.49	4.92E-45	lobular	UBE2L3
ARID1B	2.22E-49	0.296933387	0.535	0.432	5.35E-45	lobular	ARID1B
GHITM	3.52E-49	-0.334659664	0.416	0.494	8.49E-45	lobular	GHITM
MAST2	4.59E-49	0.324746735	0.258	0.171	1.11E-44	lobular	MAST2
ASPH	6.09E-49	0.299175248	0.321	0.228	1.47E-44	lobular	ASPH
EIF4G3	7.88E-49	0.314135699	0.55	0.452	1.90E-44	lobular	EIF4G3
DIAPH1	8.09E-49	0.276265823	0.335	0.239	1.95E-44	lobular	DIAPH1
CALR	8.48E-49	0.304341632	0.261	0.173	2.05E-44	lobular	CALR
ARID1A	9.10E-49	0.269520475	0.275	0.186	2.20E-44	lobular	ARID1A
LCOR	1.21E-48	0.330287554	0.3	0.213	2.93E-44	lobular	LCOR
EMP1	1.76E-48	-0.613970935	0.675	0.709	4.25E-44	lobular	EMP1
ARL1	6.13E-48	-0.303928599	0.219	0.304	1.48E-43	lobular	ARL1
ATP5H	6.19E-48	-0.278771447	0.318	0.407	1.49E-43	lobular	ATP5H
HNRNPC	6.22E-48	-0.287189649	0.773	0.803	1.50E-43	lobular	HNRNPC
CCT8	1.57E-47	-0.343966016	0.253	0.333	3.78E-43	lobular	CCT8
CLIP1	1.69E-47	0.307567124	0.438	0.34	4.07E-43	lobular	CLIP1

BCOR	1.99E-47	-0.252453411	0.474	0.581	4.80E-43	lobular	BCOR
CLINT1	2.15E-47	-0.347825223	0.218	0.301	5.17E-43	lobular	CLINT1
NFAT5	2.28E-47	0.301446279	0.664	0.572	5.51E-43	lobular	NFAT5
OLA1	3.77E-47	0.312406198	0.36	0.268	9.09E-43	lobular	OLA1
SIK3	4.21E-47	-0.511055578	0.97	0.977	1.01E-42	lobular	SIK3
CALCOCO2	4.46E-47	-0.328517216	0.222	0.304	1.07E-42	lobular	CALCOCO2
TMEM41B	6.37E-47	0.269266122	0.284	0.195	1.54E-42	lobular	TMEM41B
CHD2	7.47E-47	0.262455812	0.589	0.483	1.80E-42	lobular	CHD2
CYB5A	2.13E-46	0.356672836	0.292	0.207	5.14E-42	lobular	CYB5A
ATXN1	2.39E-46	0.297095111	0.554	0.455	5.76E-42	lobular	ATXN1
CYR61	2.83E-46	0.328001609	0.308	0.211	6.82E-42	lobular	CYR61
CAPN8	3.98E-46	0.265820394	0.253	0.166	9.60E-42	lobular	CAPN8
HIST1H2BD	4.00E-46	0.286588045	0.337	0.243	9.64E-42	lobular	HIST1H2BD
KMT2E	4.46E-46	0.315545915	0.503	0.408	1.08E-41	lobular	KMT2E
PFDN4	4.87E-46	-0.294232307	0.304	0.392	1.17E-41	lobular	PFDN4
NDUFV2	8.60E-46	0.28590179	0.306	0.217	2.07E-41	lobular	NDUFV2
THSD4-AS1	1.31E-45	-0.30611415	0.43	0.508	3.15E-41	lobular	THSD4-AS1
PNPLA8	1.40E-45	-0.362287384	0.249	0.332	3.38E-41	lobular	PNPLA8
PPM1H	3.34E-45	-0.451313773	0.286	0.363	8.06E-41	lobular	PPM1H
CWC15	3.52E-45	-0.289046914	0.254	0.339	8.48E-41	lobular	CWC15
SF3B6	4.47E-45	-0.278539803	0.584	0.65	1.08E-40	lobular	SF3B6
RP11-511B	5.62E-45	-0.287720169	0.48	0.555	1.35E-40	lobular	RP11-511B23.2
PAPSS1	5.65E-45	-0.385441547	0.201	0.277	1.36E-40	lobular	PAPSS1
FAM185A	1.22E-44	-0.315805045	0.183	0.259	2.93E-40	lobular	FAM185A
SMC5	1.77E-44	-0.384267412	0.277	0.355	4.27E-40	lobular	SMC5
OCLN	1.92E-44	0.2770575	0.374	0.28	4.63E-40	lobular	OCLN
OSMR	2.18E-44	-0.336069944	0.277	0.359	5.26E-40	lobular	OSMR
NCOR1	3.80E-44	0.2618251	0.459	0.366	9.17E-40	lobular	NCOR1
LAMTOR5	4.05E-44	-0.270361724	0.563	0.63	9.78E-40	lobular	LAMTOR5
TTC1	1.73E-43	-0.315139147	0.244	0.323	4.17E-39	lobular	TTC1
HLA-C	2.23E-43	0.337330652	0.346	0.262	5.37E-39	lobular	HLA-C
NUP153	2.23E-43	0.265996505	0.318	0.228	5.37E-39	lobular	NUP153
TEAD1	2.45E-43	0.278661253	0.491	0.394	5.91E-39	lobular	TEAD1
RBPMS	3.21E-43	-0.357114316	0.639	0.697	7.74E-39	lobular	RBPMS
ARID4B	3.32E-43	0.283983033	0.593	0.498	7.99E-39	lobular	ARID4B
CASC15	6.54E-43	0.580943484	0.547	0.485	1.58E-38	lobular	CASC15
USP25	1.59E-42	0.296751993	0.292	0.209	3.83E-38	lobular	USP25
TNRC6B	1.78E-42	0.266207546	0.524	0.43	4.28E-38	lobular	TNRC6B
B4GALT1	1.80E-42	0.252210358	0.477	0.381	4.34E-38	lobular	B4GALT1
ASTN2	3.64E-42	0.302774418	0.307	0.222	8.79E-38	lobular	ASTN2
NEAT1	4.87E-42	-0.27713743	0.938	0.964	1.18E-37	lobular	NEAT1
PPP3CA	5.09E-42	0.312091652	0.596	0.514	1.23E-37	lobular	PPP3CA
SOS1	1.56E-41	0.2746161	0.405	0.315	3.76E-37	lobular	SOS1
MARCKS	1.65E-41	-0.365769703	0.322	0.4	3.99E-37	lobular	MARCKS
CCDC6	2.26E-41	0.30086367	0.351	0.266	5.46E-37	lobular	CCDC6
SMAD2	1.04E-40	0.274099557	0.337	0.253	2.50E-36	lobular	SMAD2
CXCL1	1.60E-40	0.826750129	0.3	0.224	3.86E-36	lobular	CXCL1

ANKRD12	1.68E-40	0.254682037	0.702	0.629	4.06E-36	lobular	ANKRD12
OVOS2	2.06E-40	-0.432405593	0.238	0.324	4.97E-36	lobular	OVOS2
WWP1	2.25E-40	0.284636007	0.344	0.262	5.42E-36	lobular	WWP1
SCOC	2.86E-40	-0.258124411	0.213	0.291	6.89E-36	lobular	SCOC
NSRP1	3.20E-40	-0.279907825	0.184	0.257	7.73E-36	lobular	NSRP1
CXCL8	3.86E-40	-0.868712617	0.416	0.499	9.31E-36	lobular	CXCL8
CLASP2	4.88E-40	0.273200685	0.306	0.223	1.18E-35	lobular	CLASP2
IER3	1.17E-39	0.283325216	0.353	0.267	2.83E-35	lobular	IER3
CAPZA2	1.39E-39	-0.292726264	0.28	0.357	3.36E-35	lobular	CAPZA2
GNL3	1.78E-39	-0.297840904	0.219	0.295	4.30E-35	lobular	GNL3
COPZ1	1.88E-39	-0.259683395	0.292	0.375	4.53E-35	lobular	COPZ1
JUN	2.73E-39	0.301053023	0.266	0.189	6.57E-35	lobular	JUN
PCM1	3.13E-39	0.258072852	0.29	0.209	7.56E-35	lobular	PCM1
DPYD	5.21E-39	-0.297142955	0.492	0.569	1.26E-34	lobular	DPYD
WDFY2	8.76E-39	0.261963912	0.298	0.216	2.11E-34	lobular	WDFY2
MRPL22	9.39E-39	-0.279325672	0.19	0.263	2.26E-34	lobular	MRPL22
CDK8	1.70E-38	0.319654541	0.271	0.195	4.10E-34	lobular	CDK8
PELI1	1.93E-38	-0.365238139	0.302	0.377	4.65E-34	lobular	PELI1
KDM6A	2.04E-38	0.250534289	0.402	0.312	4.93E-34	lobular	KDM6A
LRRFIP2	2.73E-38	-0.563876559	0.756	0.775	6.59E-34	lobular	LRRFIP2
MACF1	4.72E-38	0.280333596	0.748	0.678	1.14E-33	lobular	MACF1
TXN	6.51E-38	-0.326192722	0.664	0.697	1.57E-33	lobular	TXN
EPB41L5	1.31E-37	0.263229608	0.329	0.246	3.16E-33	lobular	EPB41L5
LMNA	1.86E-37	0.264249999	0.263	0.184	4.48E-33	lobular	LMNA
TMA7	2.23E-37	0.308481366	0.631	0.557	5.37E-33	lobular	TMA7
STAT3	2.57E-37	0.273013662	0.568	0.492	6.19E-33	lobular	STAT3
SFRP1	3.33E-37	-0.271330429	0.199	0.272	8.03E-33	lobular	SFRP1
ANKRD17	5.43E-37	0.253500126	0.542	0.456	1.31E-32	lobular	ANKRD17
GAREM1	6.56E-37	0.278391206	0.352	0.271	1.58E-32	lobular	GAREM1
TBC1D8	9.73E-37	0.257102421	0.464	0.376	2.35E-32	lobular	TBC1D8
DHRSX	1.27E-36	0.27633184	0.309	0.228	3.05E-32	lobular	DHRSX
CLMN	1.30E-36	-0.384779269	0.512	0.571	3.13E-32	lobular	CLMN
RBMS1	1.83E-36	0.307301629	0.376	0.296	4.41E-32	lobular	RBMS1
NTN4	9.01E-36	0.272032556	0.332	0.252	2.17E-31	lobular	NTN4
ATP5F1	9.32E-36	-0.260841886	0.346	0.422	2.25E-31	lobular	ATP5F1
FAM19A2	1.05E-35	0.413235572	0.626	0.545	2.54E-31	lobular	FAM19A2
PBX1	1.13E-35	0.253940805	0.294	0.215	2.72E-31	lobular	PBX1
TMOD3	1.52E-35	0.279685197	0.434	0.356	3.68E-31	lobular	TMOD3
S100A11	1.83E-35	-0.330233016	0.709	0.751	4.42E-31	lobular	S100A11
DENND4A	2.75E-35	0.388182565	0.619	0.556	6.62E-31	lobular	DENND4A
BTF3	4.10E-35	0.450977285	0.878	0.907	9.89E-31	lobular	BTF3
FMNL2	6.46E-35	-0.426506093	0.329	0.395	1.56E-30	lobular	FMNL2
PTGES3	1.15E-34	0.26034502	0.272	0.199	2.76E-30	lobular	PTGES3
TCEAL8	2.77E-34	-0.265647044	0.209	0.278	6.68E-30	lobular	TCEAL8
SESTD1	2.95E-34	-0.304518118	0.465	0.53	7.12E-30	lobular	SESTD1
NRIP1	3.07E-34	0.273717191	0.299	0.223	7.41E-30	lobular	NRIP1
RNF24	1.35E-33	-0.306152292	0.307	0.377	3.26E-29	lobular	RNF24

CHD9	1.69E-33	-0.2984741	0.378	0.445	4.06E-29	lobular	CHD9
TMTC2	1.75E-33	0.255035282	0.411	0.327	4.23E-29	lobular	TMTC2
FDFT1	2.36E-33	0.262290527	0.344	0.268	5.69E-29	lobular	FDFT1
SMAD3	4.17E-33	0.314881433	0.298	0.23	1.01E-28	lobular	SMAD3
GPD2	9.53E-33	0.311547071	0.36	0.287	2.30E-28	lobular	GPD2
STK39	9.67E-33	0.388839003	0.267	0.196	2.33E-28	lobular	STK39
MAML3	1.21E-32	0.313901227	0.381	0.305	2.92E-28	lobular	MAML3
FRYL	1.69E-32	0.261671492	0.428	0.351	4.07E-28	lobular	FRYL
RAPGEF5	2.20E-32	-0.31105566	0.312	0.385	5.30E-28	lobular	RAPGEF5
PSMC6	2.34E-32	-0.275365565	0.291	0.359	5.64E-28	lobular	PSMC6
MMADHC	5.74E-32	-0.265487469	0.284	0.351	1.38E-27	lobular	MMADHC
PIN4	7.61E-32	-0.257863758	0.243	0.311	1.84E-27	lobular	PIN4
REL	1.38E-31	0.255724162	0.33	0.255	3.33E-27	lobular	REL
SEC11A	2.34E-31	-0.250782165	0.461	0.524	5.63E-27	lobular	SEC11A
EIF4E2	4.14E-31	-0.25400183	0.3	0.365	9.98E-27	lobular	EIF4E2
IRF2	6.59E-31	-0.258086817	0.209	0.274	1.59E-26	lobular	IRF2
CX3CL1	9.76E-31	0.262721801	0.259	0.189	2.35E-26	lobular	CX3CL1
SPATS2L	1.32E-30	-0.322681896	0.216	0.279	3.19E-26	lobular	SPATS2L
NDUFB4	1.43E-30	0.262547702	0.467	0.386	3.44E-26	lobular	NDUFB4
PSTPIP2	1.59E-30	-0.362478257	0.203	0.265	3.83E-26	lobular	PSTPIP2
ARHGEF38	5.28E-30	0.288343551	0.513	0.44	1.27E-25	lobular	ARHGEF38
SORBS2	6.82E-30	-0.44267285	0.356	0.423	1.64E-25	lobular	SORBS2
DAPK2	7.57E-30	-0.281008812	0.21	0.275	1.82E-25	lobular	DAPK2
PRDM2	1.96E-29	0.318579717	0.325	0.252	4.73E-25	lobular	PRDM2
GPRC5A	2.12E-29	0.27820975	0.42	0.346	5.11E-25	lobular	GPRC5A
HES1	2.24E-29	0.409825389	0.371	0.3	5.40E-25	lobular	HES1
PALLD	3.53E-29	-0.349049516	0.381	0.439	8.51E-25	lobular	PALLD
PSMD11	8.70E-29	-0.267004151	0.235	0.297	2.10E-24	lobular	PSMD11
CAPN2	1.24E-28	-0.259521803	0.27	0.335	2.98E-24	lobular	CAPN2
PNRC1	3.44E-28	-0.279586325	0.397	0.454	8.29E-24	lobular	PNRC1
TJP2	3.65E-28	-0.329644375	0.301	0.358	8.79E-24	lobular	TJP2
RPS2	5.54E-28	0.278728476	0.303	0.231	1.34E-23	lobular	RPS2
ABHD18	9.51E-28	0.271354829	0.372	0.303	2.29E-23	lobular	ABHD18
RASA2	2.73E-27	-0.336641372	0.521	0.573	6.58E-23	lobular	RASA2
YPEL5	2.81E-27	-0.288254474	0.38	0.436	6.76E-23	lobular	YPEL5
RIPK2	3.78E-27	-0.362925506	0.258	0.318	9.12E-23	lobular	RIPK2
HNRNPA3	3.99E-27	-0.257383514	0.242	0.302	9.63E-23	lobular	HNRNPA3
CAST	1.21E-26	-0.254268522	0.494	0.546	2.93E-22	lobular	CAST
TPT1-AS1	1.83E-26	-0.27866689	0.214	0.272	4.42E-22	lobular	TPT1-AS1
GLUL	2.01E-26	0.250815655	0.407	0.331	4.84E-22	lobular	GLUL
AC016831.	3.90E-26	-0.306951453	0.236	0.296	9.40E-22	lobular	AC016831.7
MAP4K5	4.73E-26	-0.302383327	0.265	0.325	1.14E-21	lobular	MAP4K5
ARHGEF10	1.51E-25	-0.338956013	0.2	0.254	3.65E-21	lobular	ARHGEF10
SEPP1	1.53E-25	0.262267563	0.339	0.272	3.69E-21	lobular	SEPP1
S100A14	4.09E-25	-0.267454518	0.724	0.754	9.86E-21	lobular	S100A14
CD55	4.50E-25	-0.281018936	0.269	0.326	1.08E-20	lobular	CD55
PTBP2	9.05E-25	-0.265052147	0.324	0.381	2.18E-20	lobular	PTBP2

CMIP	1.24E-24	-0.268505439	0.246	0.303	2.99E-20	lobular	CMIP
JARID2	3.11E-24	0.378592599	0.277	0.215	7.49E-20	lobular	JARID2
MPZL1	3.51E-24	-0.267344084	0.355	0.41	8.46E-20	lobular	MPZL1
RPSA	5.14E-24	0.431356109	0.798	0.831	1.24E-19	lobular	RPSA
EXT1	9.46E-24	-0.303487422	0.478	0.531	2.28E-19	lobular	EXT1
CRIM1	1.25E-23	-0.372928769	0.424	0.469	3.02E-19	lobular	CRIM1
LSAMP	2.35E-23	0.44204311	0.487	0.449	5.67E-19	lobular	LSAMP
PDE8A	6.30E-23	-0.275173431	0.308	0.363	1.52E-18	lobular	PDE8A
CLK1	4.02E-22	-0.275417545	0.399	0.45	9.69E-18	lobular	CLK1
KDM7A	4.64E-21	-0.253560606	0.257	0.308	1.12E-16	lobular	KDM7A
TACSTD2	7.05E-21	0.31891673	0.739	0.717	1.70E-16	lobular	TACSTD2
ATP13A3	3.57E-19	-0.286541461	0.245	0.293	8.62E-15	lobular	ATP13A3
RPS27	4.59E-19	-1.065799586	0.631	0.648	1.11E-14	lobular	RPS27
ERO1A	3.66E-18	-0.355366203	0.426	0.467	8.84E-14	lobular	ERO1A
ADAM17	3.69E-18	-0.267581125	0.368	0.41	8.90E-14	lobular	ADAM17
HIVEP2	1.18E-17	0.379451804	0.465	0.429	2.85E-13	lobular	HIVEP2
DHFR	9.13E-15	-0.262560868	0.794	0.786	2.20E-10	lobular	DHFR
DAAM1	7.74E-13	-0.299100891	0.501	0.524	1.87E-08	lobular	DAAM1
RP11-37B2	2.45E-12	-0.259501697	0.214	0.25	5.90E-08	lobular	RP11-37B2.1
TNFSF10	9.84E-12	-0.395674103	0.466	0.487	2.37E-07	lobular	TNFSF10
FOSB	1.53E-11	-0.262509169	0.386	0.413	3.68E-07	lobular	FOSB
IFRD1	2.64E-11	-0.253683866	0.342	0.37	6.38E-07	lobular	IFRD1
ATP5EP2	2.93E-11	0.265058578	0.317	0.287	7.06E-07	lobular	ATP5EP2
BAIAP2L1	2.14E-10	0.355432154	0.911	0.953	5.17E-06	lobular	BAIAP2L1
DAPK1	3.37E-10	-0.364604726	0.577	0.462	8.13E-06	lobular	DAPK1
ADAM9	4.98E-10	-0.266710572	0.563	0.579	1.20E-05	lobular	ADAM9
CCL2.1	0	2.725122155	0.447	0.148	0	ductal	CCL2
MGP.1	0	2.262830937	0.952	0.74	0	ductal	MGP
TNFAIP6.1	0	2.151492186	0.357	0.114	0	ductal	TNFAIP6
PTN.1	0	2.024538586	0.275	0.018	0	ductal	PTN
ANXA1.1	0	1.812768301	0.771	0.514	0	ductal	ANXA1
SLPI.1	0	1.787375225	0.781	0.414	0	ductal	SLPI
FAM177B.1	0	1.657631728	0.693	0.375	0	ductal	FAM177B
PDE4B.1	0	1.521512584	0.698	0.411	0	ductal	PDE4B
B2M.1	0	1.35009724	0.986	0.963	0	ductal	B2M
AKT3.1	0	1.347825087	0.543	0.231	0	ductal	AKT3
RCAN1.1	0	1.29291809	0.54	0.311	0	ductal	RCAN1
SDCBP.1	0	1.275730213	0.77	0.528	0	ductal	SDCBP
STEAP1B.1	0	1.243422608	0.954	0.922	0	ductal	STEAP1B
ANKRD36C	0	1.213598799	0.633	0.356	0	ductal	ANKRD36C
ALDH1A3.1	0	1.206707653	0.441	0.18	0	ductal	ALDH1A3
LTF.1	0	1.189813971	0.513	0.244	0	ductal	LTF
PGK1.1	0	1.153963297	0.709	0.465	0	ductal	PGK1
FAM172A.1	0	1.129217956	0.749	0.541	0	ductal	FAM172A
PCNXL2.1	0	1.117886167	0.864	0.705	0	ductal	PCNXL2
EPB42.1	0	1.099491879	0.94	0.869	0	ductal	EPB42
PSME2.1	0	1.089003029	0.456	0.198	0	ductal	PSME2

TSHZ2.1	0	1.077660036	0.397	0.163	0	ductal	TSHZ2
PTEN.1	0	1.072595202	0.89	0.759	0	ductal	PTEN
RPL21.1	0	1.069539434	0.951	0.822	0	ductal	RPL21
GRB14.1	0	1.067678487	0.556	0.323	0	ductal	GRB14
SOD2.1	0	1.030661906	0.885	0.714	0	ductal	SOD2
UBC.1	0	1.014091205	0.963	0.861	0	ductal	UBC
RP11-795H	0	1.002727826	0.512	0.273	0	ductal	RP11-795H16.3
MET.1	0	0.917931571	0.365	0.146	0	ductal	MET
RP11-356C	0	0.908074306	0.27	0.078	0	ductal	RP11-356C4.5
RPS3A.1	0	0.890992547	0.95	0.869	0	ductal	RPS3A
SEC61G.1	0	0.862317764	0.727	0.524	0	ductal	SEC61G
SON.1	0	0.813488169	0.777	0.623	0	ductal	SON
ANXA2.1	0	0.774807154	0.943	0.917	0	ductal	ANXA2
NPM1.1	0	0.749023387	0.836	0.642	0	ductal	NPM1
RBFOX2.1	0	0.732646875	0.971	0.94	0	ductal	RBFOX2
ADGRL3-AS	0	0.729782258	0.729	0.521	0	ductal	ADGRL3-AS1
SKP1.1	0	0.718890034	0.875	0.767	0	ductal	SKP1
PLEKHA6.1	0	0.716527974	0.91	0.787	0	ductal	PLEKHA6
RPL24.1	0	0.676667684	0.954	0.894	0	ductal	RPL24
SLC47A1.1	0	0.641508596	0.952	0.863	0	ductal	SLC47A1
LINC00486	0	0.61251405	0.993	0.984	0	ductal	LINC00486
KIZ-AS1.1	0	0.596703007	0.914	0.802	0	ductal	KIZ-AS1
RPL34.1	0	0.592745866	0.983	0.975	0	ductal	RPL34
RPL39.1	0	0.509772286	0.944	0.871	0	ductal	RPL39
EEF1A1.1	0	0.481011136	0.983	0.97	0	ductal	EEF1A1
RPL37A.1	0	0.406225757	0.98	0.971	0	ductal	RPL37A
MT-ATP6.1	0	-0.534050557	0.984	0.995	0	ductal	MT-ATP6
TPT1.1	0	-0.540946218	0.761	0.907	0	ductal	TPT1
RPS14.1	0	-0.558013124	0.234	0.519	0	ductal	RPS14
MT-ND1.1	0	-0.577968263	0.961	0.991	0	ductal	MT-ND1
OST4.1	0	-0.832455471	0.244	0.519	0	ductal	OST4
RPS8.1	0	-0.845433614	0.587	0.828	0	ductal	RPS8
COX4I1.1	0	-0.871950883	0.666	0.839	0	ductal	COX4I1
GNB2L1.1	0	-0.905572278	0.506	0.772	0	ductal	GNB2L1
ACTG1.1	0	-0.910288446	0.601	0.8	0	ductal	ACTG1
RPL15.1	0	-0.949047938	0.631	0.867	0	ductal	RPL15
AMBRA1.1	0	-0.955761452	0.494	0.787	0	ductal	AMBRA1
SERF2.1	0	-0.956735644	0.176	0.527	0	ductal	SERF2
GNAS.1	0	-0.977566622	0.61	0.839	0	ductal	GNAS
RPS18.1	0	-0.988999102	0.436	0.756	0	ductal	RPS18
AREG.1	0	-1.04445651	0.272	0.561	0	ductal	AREG
NBEAL1.1	0	-1.055255649	0.529	0.828	0	ductal	NBEAL1
FTL.1	0	-1.113596813	0.326	0.655	0	ductal	FTL
RPL10A.1	0	-1.129483999	0.582	0.799	0	ductal	RPL10A
ERBB4.1	0	-1.137604686	0.309	0.605	0	ductal	ERBB4
EIF2B5.1	0	-1.151714614	0.315	0.55	0	ductal	EIF2B5
PTHLH.1	0	-1.178957482	0.081	0.331	0	ductal	PTHLH

ANKRD30A	0	-1.20772945	0.263	0.6	0	ductal	ANKRD30A
HPX.1	0	-1.22775497	0.07	0.304	0	ductal	HPX
RPLP0.1	0	-1.33754745	0.794	0.91	0	ductal	RPLP0
RPL19.1	0	-1.369488293	0.855	0.93	0	ductal	RPL19
DIO2.1	0	-1.371056782	0.083	0.31	0	ductal	DIO2
RPS12.1	0	-1.41506119	0.503	0.737	0	ductal	RPS12
TMSB10.1	0	-1.579126235	0.491	0.878	0	ductal	TMSB10
RPS11.1	0	-1.653818756	0.564	0.894	0	ductal	RPS11
RPS3.1	0	-1.81604144	0.843	0.884	0	ductal	RPS3
RPL10.1	0	-1.819908231	0.912	0.949	0	ductal	RPL10
HINT1.1	7.47E-306	-0.728819844	0.472	0.68	1.80E-301	ductal	HINT1
EIF4A2.1	5.87E-304	0.785508174	0.787	0.644	1.41E-299	ductal	EIF4A2
PI3.1	7.87E-300	1.72874024	0.264	0.078	1.90E-295	ductal	PI3
GCNT2.1	2.02E-296	-1.073512747	0.294	0.537	4.87E-292	ductal	GCNT2
PSMA4.1	2.62E-296	0.906113528	0.571	0.363	6.33E-292	ductal	PSMA4
MT-ND2.1	3.00E-296	-0.425266432	0.959	0.988	7.23E-292	ductal	MT-ND2
SLC25A37.:	7.42E-296	1.012555721	0.533	0.298	1.79E-291	ductal	SLC25A37
EREG.1	1.91E-295	-1.129437377	0.119	0.349	4.60E-291	ductal	EREG
RPS20.1	2.44E-295	-0.931541542	0.925	0.924	5.89E-291	ductal	RPS20
PRSS23.1	8.79E-295	-0.927162491	0.087	0.304	2.12E-290	ductal	PRSS23
RPL35.1	2.23E-292	-0.547413043	0.12	0.371	5.38E-288	ductal	RPL35
PTMA.1	9.27E-285	-0.70340638	0.487	0.703	2.23E-280	ductal	PTMA
C8orf4.1	2.34E-284	-0.70153973	0.332	0.635	5.64E-280	ductal	C8orf4
ALCAM.1	1.91E-279	-1.006126082	0.375	0.597	4.62E-275	ductal	ALCAM
MT-CO3.1	4.71E-276	-0.388237544	0.992	0.997	1.13E-271	ductal	MT-CO3
CPEB2.1	5.00E-274	-0.918324418	0.167	0.397	1.21E-269	ductal	CPEB2
PRDX1.1	5.92E-274	0.869905189	0.765	0.637	1.43E-269	ductal	PRDX1
FAM129A.:	7.40E-272	1.19688608	0.402	0.197	1.79E-267	ductal	FAM129A
SLC26A3.1	2.34E-264	0.696763643	0.864	0.746	5.65E-260	ductal	SLC26A3
EFHD1.1	6.92E-264	-0.853468492	0.162	0.386	1.67E-259	ductal	EFHD1
AFF3.1	3.93E-261	-1.109166937	0.199	0.426	9.49E-257	ductal	AFF3
ARRDC3.1	2.60E-256	0.864813579	0.519	0.305	6.27E-252	ductal	ARRDC3
SEC62.1	5.01E-255	0.643444507	0.693	0.511	1.21E-250	ductal	SEC62
POMP.1	2.82E-252	0.688236785	0.723	0.579	6.80E-248	ductal	POMP
TNC.1	6.45E-252	-0.980565083	0.197	0.415	1.55E-247	ductal	TNC
HSP90AB1.	1.51E-249	0.582403055	0.895	0.807	3.65E-245	ductal	HSP90AB1
RPS7.1	1.32E-247	0.339920161	0.963	0.889	3.18E-243	ductal	RPS7
H2AFZ.1	4.11E-246	-0.742461407	0.568	0.733	9.92E-242	ductal	H2AFZ
EFNA1.1	5.17E-246	-0.902050068	0.115	0.316	1.25E-241	ductal	EFNA1
MYBPC1.1	1.35E-245	-1.038135522	0.106	0.311	3.25E-241	ductal	MYBPC1
BIRC3.1	4.91E-244	0.74761515	0.73	0.543	1.18E-239	ductal	BIRC3
RAB11FIP1	1.68E-243	-0.930657497	0.658	0.781	4.04E-239	ductal	RAB11FIP1
CXCL13.1	5.97E-243	-0.648141877	0.269	0.5	1.44E-238	ductal	CXCL13
KIAA1324.:	1.14E-238	-0.702053588	0.106	0.304	2.75E-234	ductal	KIAA1324
RPL9.1	8.72E-237	0.666902878	0.675	0.508	2.10E-232	ductal	RPL9
SMIM14.1	1.82E-236	-0.781861995	0.269	0.48	4.39E-232	ductal	SMIM14
TBX3.1	2.11E-236	-0.798212627	0.145	0.348	5.09E-232	ductal	TBX3

RPS6.1	4.18E-235	0.40453666	0.985	0.974	1.01E-230	ductal	RPS6
MAML2.1	4.28E-234	0.787481138	0.554	0.336	1.03E-229	ductal	MAML2
GLIS3.1	2.35E-231	0.801683136	0.265	0.099	5.66E-227	ductal	GLIS3
SLC28A3.1	4.17E-231	0.719107526	0.286	0.113	1.01E-226	ductal	SLC28A3
BTG1.1	1.39E-230	-0.69733317	0.461	0.655	3.35E-226	ductal	BTG1
TSPAN5.1	2.32E-230	-0.859026918	0.178	0.389	5.61E-226	ductal	TSPAN5
RASGEF1B.	1.14E-229	0.541126374	0.947	0.859	2.76E-225	ductal	RASGEF1B
ADAMTS9.:	1.36E-229	1.003982756	0.329	0.144	3.29E-225	ductal	ADAMTS9
ST6GALNA(	5.46E-228	0.975553795	0.297	0.122	1.32E-223	ductal	ST6GALNAC5
RNF181.1	6.48E-228	0.702127179	0.51	0.315	1.56E-223	ductal	RNF181
FXYD3.1	1.44E-225	-0.739965568	0.518	0.704	3.47E-221	ductal	FXYD3
SLC7A2.1	6.79E-225	-0.771745132	0.173	0.38	1.64E-220	ductal	SLC7A2
C15orf48.1	1.16E-224	1.303795184	0.606	0.436	2.80E-220	ductal	C15orf48
GABRP.1	5.48E-223	0.873785178	0.385	0.195	1.32E-218	ductal	GABRP
SNU13.1	3.13E-222	-0.64135365	0.347	0.556	7.55E-218	ductal	SNU13
UGCG.1	3.04E-220	-0.679530662	0.36	0.569	7.34E-216	ductal	UGCG
HNRNPA1.:	4.60E-219	0.665213457	0.836	0.713	1.11E-214	ductal	HNRNPA1
CPE.1	3.62E-211	-0.738412275	0.094	0.273	8.73E-207	ductal	CPE
FGF13.1	7.94E-208	-0.860223233	0.33	0.529	1.91E-203	ductal	FGF13
RPL11.1	2.21E-207	0.309488245	0.98	0.947	5.32E-203	ductal	RPL11
DYNLRB1.1	7.63E-205	-0.557545789	0.186	0.391	1.84E-200	ductal	DYNLRB1
MAP3K13.:	3.14E-204	0.780528994	0.715	0.566	7.58E-200	ductal	MAP3K13
SOX4.1	4.06E-203	-0.454278113	0.743	0.897	9.78E-199	ductal	SOX4
RPS17.1	9.15E-202	0.384511078	0.964	0.927	2.21E-197	ductal	RPS17
RPS27A.1	3.48E-201	0.273840503	0.985	0.962	8.39E-197	ductal	RPS27A
RPL37.1	4.54E-200	0.333427488	0.967	0.933	1.09E-195	ductal	RPL37
ANKS1B.1	1.05E-199	-0.768393564	0.338	0.536	2.52E-195	ductal	ANKS1B
SELK.1	9.39E-199	0.601320794	0.711	0.553	2.26E-194	ductal	SELK
PSMA3.1	2.78E-198	0.672356934	0.452	0.272	6.71E-194	ductal	PSMA3
STAT5B.1	4.42E-196	-0.675069398	0.211	0.407	1.06E-191	ductal	STAT5B
APBB2.1	3.66E-191	-0.786350288	0.147	0.326	8.81E-187	ductal	APBB2
LINGO1.1	1.31E-190	0.48281975	0.938	0.86	3.16E-186	ductal	LINGO1
DAPP1.1	6.51E-188	0.770088616	0.347	0.181	1.57E-183	ductal	DAPP1
FBLN5.1	3.24E-184	1.165776883	0.259	0.112	7.82E-180	ductal	FBLN5
NDRG1.1	2.62E-183	0.749438558	0.465	0.286	6.32E-179	ductal	NDRG1
MAP3K1.1	3.19E-183	-0.754777275	0.342	0.53	7.69E-179	ductal	MAP3K1
EPS8.1	4.78E-183	0.78619943	0.428	0.257	1.15E-178	ductal	EPS8
RPL31.1	2.16E-181	0.39557886	0.958	0.905	5.22E-177	ductal	RPL31
ITGAV.1	6.23E-179	-0.672675221	0.284	0.472	1.50E-174	ductal	ITGAV
COMMD6.:	3.98E-178	-0.543039763	0.236	0.428	9.60E-174	ductal	COMMD6
ACTN1.1	1.59E-177	-0.65542962	0.226	0.415	3.83E-173	ductal	ACTN1
SAA1.1	9.97E-177	-0.30023426	0.816	0.681	2.40E-172	ductal	SAA1
CCL20.1	3.93E-176	2.082814658	0.277	0.129	9.48E-172	ductal	CCL20
CALD1.1	1.76E-175	0.902221841	0.409	0.233	4.23E-171	ductal	CALD1
PSMB7.1	2.46E-175	0.639444538	0.554	0.394	5.92E-171	ductal	PSMB7
PSMC1.1	8.40E-175	0.596301598	0.384	0.218	2.03E-170	ductal	PSMC1
GNA12.1	2.83E-174	0.652016769	0.404	0.239	6.83E-170	ductal	GNA12

TAGLN2.1	2.90E-173	-0.480140323	0.114	0.284	6.99E-169	ductal	TAGLN2
MYL12A.1	1.24E-172	0.53777542	0.895	0.851	3.00E-168	ductal	MYL12A
CTSB.1	8.21E-172	-0.626395151	0.183	0.357	1.98E-167	ductal	CTSB
RP1-78O14	5.39E-171	-0.876106024	0.106	0.265	1.30E-166	ductal	RP1-78O14.1
CD59.1	1.01E-169	0.686050045	0.897	0.874	2.44E-165	ductal	CD59
ELOVL5.1	1.02E-169	-0.810120108	0.188	0.362	2.46E-165	ductal	ELOVL5
RP11-608O	1.06E-169	0.631946125	0.645	0.476	2.55E-165	ductal	RP11-608O21.1
ARHGAP26	4.21E-169	0.595104939	0.761	0.611	1.01E-164	ductal	ARHGAP26
CA8.1	1.04E-168	0.791629386	0.336	0.177	2.50E-164	ductal	CA8
COX7C.1	1.01E-167	0.346659587	0.902	0.856	2.43E-163	ductal	COX7C
RPL30.1	1.82E-166	0.297807521	0.974	0.941	4.39E-162	ductal	RPL30
SEMA3C.1	4.42E-166	-0.751627584	0.174	0.346	1.07E-161	ductal	SEMA3C
LYN.1	7.63E-166	0.783722607	0.515	0.347	1.84E-161	ductal	LYN
NEBL.1	2.08E-165	-0.654932969	0.571	0.72	5.01E-161	ductal	NEBL
DUSP16.1	7.36E-165	-0.624347037	0.31	0.491	1.77E-160	ductal	DUSP16
RPLP1.1	6.91E-164	-0.278187617	0.174	0.359	1.67E-159	ductal	RPLP1
RPL23.1	4.93E-163	0.350625652	0.945	0.899	1.19E-158	ductal	RPL23
GSTM3.1	4.67E-162	-0.885069579	0.222	0.394	1.13E-157	ductal	GSTM3
DNAJC12.1	1.51E-161	-0.551734973	0.268	0.467	3.63E-157	ductal	DNAJC12
EFNA5.1	1.61E-161	0.728056884	0.385	0.221	3.88E-157	ductal	EFNA5
RPL12.1	2.38E-161	-0.540548362	0.18	0.352	5.75E-157	ductal	RPL12
MT-ND4.1	1.01E-160	-0.324124623	0.991	0.995	2.43E-156	ductal	MT-ND4
SLC39A6.1	1.82E-159	-0.577589293	0.195	0.369	4.39E-155	ductal	SLC39A6
RPS9.1	2.00E-158	-0.300883311	0.123	0.295	4.83E-154	ductal	RPS9
RSL24D1.1	2.40E-158	0.486832265	0.663	0.499	5.79E-154	ductal	RSL24D1
SLC27A4.1	2.82E-156	0.840966834	0.787	0.656	6.80E-152	ductal	SLC27A4
PSD3.1	1.83E-154	-0.701994758	0.242	0.412	4.42E-150	ductal	PSD3
CYP7B1.1	2.76E-154	1.000354873	0.323	0.177	6.66E-150	ductal	CYP7B1
NCEH1.1	6.38E-154	-0.746132164	0.313	0.476	1.54E-149	ductal	NCEH1
SUB1.1	1.02E-152	0.503317303	0.718	0.591	2.47E-148	ductal	SUB1
NACA.1	1.25E-152	0.268387352	0.945	0.887	3.01E-148	ductal	NACA
GAPDH.1	3.79E-152	-0.44928616	0.457	0.632	9.14E-148	ductal	GAPDH
ERGIC3.1	3.93E-152	-0.530297915	0.221	0.39	9.48E-148	ductal	ERGIC3
INSIG2.1	5.38E-152	0.61660419	0.303	0.158	1.30E-147	ductal	INSIG2
SSR3.1	4.93E-151	0.58985524	0.542	0.387	1.19E-146	ductal	SSR3
GLCCI1.1	7.04E-151	-0.728450167	0.2	0.365	1.70E-146	ductal	GLCCI1
LUCAT1.1	3.73E-149	0.637170112	0.296	0.149	8.99E-145	ductal	LUCAT1
LIPH.1	3.78E-149	1.104610882	0.401	0.257	9.12E-145	ductal	LIPH
RPL35A.1	1.09E-148	0.282018205	0.977	0.942	2.64E-144	ductal	RPL35A
XBP1.1	2.05E-148	-0.746720838	0.289	0.455	4.95E-144	ductal	XBP1
PHLDB2.1	4.67E-148	-0.647152125	0.315	0.484	1.13E-143	ductal	PHLDB2
ZFP36L2.1	1.06E-147	-0.580034539	0.26	0.441	2.56E-143	ductal	ZFP36L2
TBC1D9.1	9.49E-147	-0.565678292	0.34	0.517	2.29E-142	ductal	TBC1D9
DSTN.1	1.48E-146	-0.571387199	0.486	0.645	3.57E-142	ductal	DSTN
SYPL1.1	2.81E-146	0.545858312	0.469	0.315	6.79E-142	ductal	SYPL1
TMEM2.1	6.22E-146	-0.705502468	0.244	0.412	1.50E-141	ductal	TMEM2
PIGR.1	9.70E-146	0.798067409	0.345	0.193	2.34E-141	ductal	PIGR

HSP90AA1.	1.69E-144	0.428916187	0.898	0.829	4.07E-140	ductal	HSP90AA1
PVRL2.1	6.34E-144	-0.594161244	0.185	0.346	1.53E-139	ductal	PVRL2
SNRPB2.1	7.90E-144	0.536304124	0.441	0.288	1.90E-139	ductal	SNRPB2
SRRM1.1	1.61E-143	0.508936276	0.629	0.483	3.89E-139	ductal	SRRM1
GLRX.1	2.14E-143	0.720974518	0.384	0.232	5.15E-139	ductal	GLRX
MORF4L1.1	4.26E-143	0.4640838	0.703	0.589	1.03E-138	ductal	MORF4L1
MDH1.1	5.42E-143	0.551516358	0.372	0.22	1.31E-138	ductal	MDH1
TJP1.1	5.77E-143	-0.594108106	0.407	0.57	1.39E-138	ductal	TJP1
TPK1.1	6.84E-143	0.546156919	0.297	0.157	1.65E-138	ductal	TPK1
CFLAR.1	1.22E-142	0.760128352	0.43	0.289	2.95E-138	ductal	CFLAR
ITGA2.1	1.44E-141	0.787615698	0.483	0.335	3.48E-137	ductal	ITGA2
UGDH.1	1.95E-141	-0.67422724	0.144	0.295	4.71E-137	ductal	UGDH
SHROOM3.	4.02E-141	-0.455570914	0.531	0.693	9.68E-137	ductal	SHROOM3
AIMP1.1	4.83E-140	0.579739894	0.397	0.247	1.16E-135	ductal	AIMP1
ERBB2IP.1	2.34E-139	-0.638782125	0.329	0.49	5.64E-135	ductal	ERBB2IP
TCF12.1	6.08E-139	-0.464128449	0.493	0.656	1.47E-134	ductal	TCF12
RPS24.1	4.56E-138	0.264749132	0.98	0.958	1.10E-133	ductal	RPS24
ZNF644.1	3.58E-137	-0.620950011	0.371	0.533	8.62E-133	ductal	ZNF644
JUP.1	7.12E-137	-0.505519355	0.17	0.325	1.72E-132	ductal	JUP
PAWR.1	1.70E-136	-0.552275913	0.45	0.611	4.10E-132	ductal	PAWR
SARNP.1	4.17E-136	0.521306309	0.285	0.149	1.00E-131	ductal	SARNP
NR6A1.1	6.70E-136	-0.722521824	0.253	0.413	1.61E-131	ductal	NR6A1
SVIL.1	3.67E-135	0.555515178	0.669	0.536	8.85E-131	ductal	SVIL
HIPK2.1	5.11E-135	-0.507886587	0.273	0.436	1.23E-130	ductal	HIPK2
RPL36AL.1	6.20E-135	0.339714751	0.917	0.869	1.50E-130	ductal	RPL36AL
INPP4B.1	1.12E-134	-0.574756628	0.224	0.395	2.69E-130	ductal	INPP4B
ZBTB10.1	4.89E-134	-0.563426585	0.164	0.315	1.18E-129	ductal	ZBTB10
USMG5.1	2.45E-133	0.388347795	0.718	0.577	5.91E-129	ductal	USMG5
SYTL2.1	4.96E-133	-0.494628622	0.337	0.523	1.20E-128	ductal	SYTL2
TULP4.1	5.88E-133	-0.496578411	0.399	0.564	1.42E-128	ductal	TULP4
GADD45A.1	2.88E-132	-0.591891956	0.173	0.325	6.95E-128	ductal	GADD45A
TM4SF1.1	3.02E-132	0.820894879	0.917	0.901	7.27E-128	ductal	TM4SF1
DRAM1.1	1.10E-131	-0.573174817	0.201	0.356	2.65E-127	ductal	DRAM1
MT-CYB.1	2.91E-131	-0.250290392	0.988	0.993	7.02E-127	ductal	MT-CYB
TBL1XR1.1	1.27E-130	-0.63412192	0.499	0.633	3.07E-126	ductal	TBL1XR1
LARP1B.1	6.62E-129	-0.596218661	0.132	0.271	1.60E-124	ductal	LARP1B
DYNLL1.1	3.97E-128	-0.51672953	0.154	0.301	9.57E-124	ductal	DYNLL1
MYL6.1	6.18E-128	0.43021124	0.95	0.925	1.49E-123	ductal	MYL6
BCAR3.1	1.29E-126	-0.670881335	0.191	0.335	3.11E-122	ductal	BCAR3
SERPINA3.1	3.28E-126	0.719029256	0.35	0.216	7.90E-122	ductal	SERPINA3
BRK1.1	5.18E-126	0.43935	0.683	0.563	1.25E-121	ductal	BRK1
ATP5G2.1	8.93E-126	-0.551158505	0.691	0.767	2.15E-121	ductal	ATP5G2
MBNL2.1	1.59E-125	-0.492649112	0.446	0.603	3.84E-121	ductal	MBNL2
RPL13.1	2.89E-125	-0.47869586	0.316	0.482	6.97E-121	ductal	RPL13
TOM1L2.1	3.21E-125	-0.552212901	0.209	0.358	7.74E-121	ductal	TOM1L2
RERE.1	4.49E-125	-0.481728123	0.478	0.628	1.08E-120	ductal	RERE
DDX24.1	1.23E-123	0.530661633	0.586	0.46	2.96E-119	ductal	DDX24

FRMD4A.1	1.68E-123	0.691605137	0.365	0.226	4.05E-119	ductal	FRMD4A
RPL13A.1	3.21E-123	-0.341798005	0.369	0.546	7.74E-119	ductal	RPL13A
RPL36.1	8.14E-123	-0.43168049	0.112	0.254	1.96E-118	ductal	RPL36
ELF3.1	1.76E-122	-0.452200548	0.415	0.574	4.25E-118	ductal	ELF3
WTAP.1	1.93E-121	0.766635443	0.592	0.469	4.64E-117	ductal	WTAP
TNFAIP8.1	7.17E-120	0.624425884	0.476	0.334	1.73E-115	ductal	TNFAIP8
DLGAP1.1	2.04E-118	-0.65102927	0.123	0.255	4.92E-114	ductal	DLGAP1
CCNI.1	2.97E-118	-0.376346244	0.536	0.684	7.15E-114	ductal	CCNI
NEDD9.1	5.73E-118	-0.476799396	0.274	0.433	1.38E-113	ductal	NEDD9
GOLM1.1	3.39E-117	-0.515295912	0.15	0.287	8.17E-113	ductal	GOLM1
CCL28.1	5.25E-116	0.535635641	0.464	0.308	1.27E-111	ductal	CCL28
C8orf37-AS	7.76E-116	0.455944106	0.332	0.196	1.87E-111	ductal	C8orf37-AS1
NUB1.1	1.54E-115	0.669692942	0.269	0.148	3.71E-111	ductal	NUB1
PCBP2.1	2.40E-115	-0.396421323	0.478	0.628	5.79E-111	ductal	PCBP2
FUT8.1	2.82E-115	-0.614195043	0.175	0.314	6.80E-111	ductal	FUT8
IGF2BP2.1	4.09E-115	0.678612181	0.355	0.223	9.87E-111	ductal	IGF2BP2
UQCR10.1	1.14E-114	-0.486704002	0.417	0.564	2.76E-110	ductal	UQCR10
BCL2.1	1.20E-114	-0.752650286	0.189	0.326	2.90E-110	ductal	BCL2
APLP2.1	2.11E-114	-0.488435287	0.192	0.332	5.08E-110	ductal	APLP2
SEPT7.1	3.16E-114	0.479972235	0.461	0.324	7.62E-110	ductal	7-Sep
OPHN1.1	5.42E-114	-0.615259305	0.238	0.385	1.31E-109	ductal	OPHN1
PAN3.1	7.51E-114	-0.448872024	0.552	0.691	1.81E-109	ductal	PAN3
CHCHD3.1	2.19E-113	-0.424683539	0.807	0.855	5.28E-109	ductal	CHCHD3
TCEANC2.1	2.38E-113	0.474153791	0.324	0.191	5.75E-109	ductal	TCEANC2
RAPH1.1	6.09E-113	-0.588253214	0.25	0.391	1.47E-108	ductal	RAPH1
ARHGEF12.	1.14E-112	-0.409701619	0.481	0.637	2.76E-108	ductal	ARHGEF12
THRB.1	2.48E-111	-0.632787913	0.377	0.513	5.99E-107	ductal	THRB
COX7A2.1	3.31E-111	0.359896281	0.81	0.736	7.98E-107	ductal	COX7A2
PDCD10.1	6.96E-111	0.485007289	0.334	0.205	1.68E-106	ductal	PDCD10
PCBP1.1	2.69E-110	-0.405904835	0.411	0.563	6.48E-106	ductal	PCBP1
MT-ND3.1	5.06E-110	-0.363454835	0.978	0.993	1.22E-105	ductal	MT-ND3
TMEM258.	1.28E-109	0.388372324	0.667	0.56	3.09E-105	ductal	TMEM258
CCT5.1	2.48E-109	0.545364241	0.377	0.249	5.98E-105	ductal	CCT5
EIF3E.1	5.84E-109	0.336318686	0.675	0.517	1.41E-104	ductal	EIF3E
DAD1.1	2.53E-108	0.438874664	0.574	0.452	6.10E-104	ductal	DAD1
NFKBIZ.1	3.46E-108	0.567429197	0.626	0.521	8.35E-104	ductal	NFKBIZ
CLDN1.1	8.19E-108	0.86016884	0.304	0.184	1.98E-103	ductal	CLDN1
RORA.1	4.86E-107	-0.629302975	0.494	0.622	1.17E-102	ductal	RORA
ILF2.1	5.15E-107	0.516245772	0.512	0.383	1.24E-102	ductal	ILF2
NEDD4L.1	5.29E-107	-0.534585889	0.514	0.651	1.27E-102	ductal	NEDD4L
PSMC2.1	4.61E-106	0.408126065	0.299	0.173	1.11E-101	ductal	PSMC2
MAGI3.1	5.35E-106	-0.494083714	0.294	0.439	1.29E-101	ductal	MAGI3
VPS29.1	1.19E-105	0.479581926	0.403	0.273	2.86E-101	ductal	VPS29
WFDC2.1	8.07E-104	1.256736964	0.407	0.277	1.95E-99	ductal	WFDC2
IK.1	1.37E-102	0.403866628	0.261	0.143	3.31E-98	ductal	IK
UBB.1	6.19E-102	0.313924224	0.784	0.685	1.49E-97	ductal	UBB
RPL29.1	2.18E-101	-0.450430826	0.152	0.288	5.26E-97	ductal	RPL29

KTN1.1	6.26E-101	-0.45900334	0.461	0.593	1.51E-96	ductal	KTN1
RARRES3.1	2.16E-100	0.551553656	0.294	0.176	5.22E-96	ductal	RARRES3
KIAA1522.1	2.52E-100	-0.404204919	0.15	0.279	6.08E-96	ductal	KIAA1522
TAOK3.1	6.55E-100	-0.453811172	0.389	0.527	1.58E-95	ductal	TAOK3
GSPT1.1	8.14E-100	-0.471803867	0.286	0.421	1.96E-95	ductal	GSPT1
TANK.1	1.43E-99	0.469647042	0.552	0.429	3.45E-95	ductal	TANK
HIST1H2AC	2.27E-99	-0.404669815	0.274	0.422	5.47E-95	ductal	HIST1H2AC
RANBP9.1	2.45E-99	-0.445225884	0.217	0.35	5.91E-95	ductal	RANBP9
BCL6.1	2.47E-99	-0.475456504	0.272	0.412	5.96E-95	ductal	BCL6
GBP2.1	7.89E-99	0.521539881	0.679	0.578	1.90E-94	ductal	GBP2
DEFB1.1	3.33E-98	0.324502309	0.538	0.382	8.03E-94	ductal	DEFB1
MYH9.1	3.38E-98	-0.429572375	0.425	0.571	8.16E-94	ductal	MYH9
FLNB.1	4.39E-98	-0.441549362	0.365	0.515	1.06E-93	ductal	FLNB
EGFR.1	1.29E-97	0.501522331	0.25	0.138	3.12E-93	ductal	EGFR
PRLR.1	1.56E-97	-0.50541528	0.16	0.285	3.75E-93	ductal	PRLR
DGKH.1	6.04E-97	-0.535177422	0.228	0.359	1.46E-92	ductal	DGKH
OOEP.1	1.05E-96	0.26003172	0.978	0.958	2.53E-92	ductal	OOEP
C4orf3.1	1.21E-96	0.422379174	0.61	0.493	2.91E-92	ductal	C4orf3
SLIRP.1	2.50E-96	0.386596175	0.394	0.261	6.03E-92	ductal	SLIRP
COL4A5.1	5.63E-96	-0.476551275	0.145	0.267	1.36E-91	ductal	COL4A5
KCTD9.1	5.71E-96	0.569250056	0.306	0.192	1.38E-91	ductal	KCTD9
SSBP1.1	7.12E-96	0.491813825	0.436	0.317	1.72E-91	ductal	SSBP1
UBL5.1	1.12E-95	-0.527060584	0.439	0.554	2.71E-91	ductal	UBL5
LRBA.1	1.72E-95	-0.419550399	0.559	0.689	4.15E-91	ductal	LRBA
PKP4.1	2.02E-95	-0.447764536	0.419	0.555	4.87E-91	ductal	PKP4
FARP1.1	2.55E-95	-0.44578121	0.294	0.434	6.16E-91	ductal	FARP1
AGR2.1	2.57E-95	-0.30205311	0.161	0.296	6.20E-91	ductal	AGR2
CDCP1.1	3.00E-95	0.480003646	0.355	0.234	7.24E-91	ductal	CDCP1
SSBP2.1	1.39E-94	-0.548998443	0.358	0.486	3.36E-90	ductal	SSBP2
ENAH.1	1.94E-94	-0.382229916	0.467	0.606	4.67E-90	ductal	ENAH
ATP8B1.1	2.53E-94	-0.563727092	0.325	0.454	6.10E-90	ductal	ATP8B1
ACTB.1	1.36E-93	-0.504181829	0.566	0.691	3.29E-89	ductal	ACTB
NCOA7.1	1.69E-93	0.528021603	0.523	0.392	4.07E-89	ductal	NCOA7
TAX1BP1.1	7.84E-93	0.387708739	0.683	0.591	1.89E-88	ductal	TAX1BP1
KLHL5.1	1.35E-92	-0.49024559	0.21	0.333	3.26E-88	ductal	KLHL5
INO80D.1	1.47E-92	0.433569653	0.643	0.525	3.55E-88	ductal	INO80D
INTS6.1	2.42E-92	-0.482099005	0.205	0.331	5.83E-88	ductal	INTS6
RBM8A.1	2.64E-92	0.451991592	0.504	0.388	6.37E-88	ductal	RBM8A
UGP2.1	1.13E-91	0.445413039	0.51	0.393	2.72E-87	ductal	UGP2
RASEF.1	1.14E-91	-0.443556131	0.195	0.322	2.74E-87	ductal	RASEF
RNF145.1	2.85E-91	0.510498639	0.416	0.293	6.88E-87	ductal	RNF145
ERH.1	1.18E-90	0.431215201	0.572	0.467	2.83E-86	ductal	ERH
MTHFD2L.1	3.89E-90	0.686735803	0.476	0.359	9.38E-86	ductal	MTHFD2L
KAZN.1	7.31E-90	-0.478279517	0.146	0.263	1.76E-85	ductal	KAZN
GAB1.1	1.03E-89	-0.405442753	0.302	0.437	2.48E-85	ductal	GAB1
CUX1.1	1.40E-89	-0.453569364	0.271	0.399	3.38E-85	ductal	CUX1
CD24.1	1.60E-89	-0.490042078	0.502	0.628	3.87E-85	ductal	CD24

REEP5.1	3.74E-89	-0.419704702	0.179	0.3	9.03E-85	ductal	REEP5
C16orf45.1	1.21E-88	-0.490665897	0.157	0.274	2.91E-84	ductal	C16orf45
LDHA.1	1.48E-88	0.40430431	0.732	0.631	3.57E-84	ductal	LDHA
NAALADL2.	2.03E-88	-0.519389727	0.428	0.55	4.90E-84	ductal	NAALADL2
LPP.1	2.62E-88	-0.283221099	0.886	0.933	6.33E-84	ductal	LPP
ATP6V0E1.	4.02E-88	-0.336563524	0.628	0.734	9.69E-84	ductal	ATP6V0E1
UBN2.1	9.17E-88	-0.422434202	0.197	0.323	2.21E-83	ductal	UBN2
WWC1.1	3.70E-87	-0.425853786	0.337	0.47	8.91E-83	ductal	WWC1
GNB1.1	5.04E-87	-0.376568774	0.266	0.395	1.22E-82	ductal	GNB1
RPLP2.1	9.97E-87	-0.272581828	0.149	0.273	2.40E-82	ductal	RPLP2
RPS16.1	1.19E-86	-0.269740605	0.225	0.361	2.87E-82	ductal	RPS16
OSBPL3.1	1.76E-86	-0.430694264	0.159	0.277	4.25E-82	ductal	OSBPL3
DDX18.1	2.43E-86	0.452548244	0.362	0.247	5.86E-82	ductal	DDX18
BTRC.1	2.85E-86	-0.418013619	0.188	0.311	6.87E-82	ductal	BTRC
DCTN6.1	6.92E-86	0.410005218	0.353	0.236	1.67E-81	ductal	DCTN6
MTRNR2L1	1.45E-85	0.445570476	0.296	0.182	3.49E-81	ductal	MTRNR2L12
ATP6V1D.1	5.48E-85	0.447507412	0.348	0.234	1.32E-80	ductal	ATP6V1D
NOS1AP.1	7.35E-85	-0.392018069	0.146	0.262	1.77E-80	ductal	NOS1AP
ARIH1.1	7.94E-85	-0.358342719	0.428	0.558	1.91E-80	ductal	ARIH1
CADPS2.1	2.76E-84	-0.812529508	0.33	0.45	6.65E-80	ductal	CADPS2
ACSL3.1	5.42E-84	-0.502507057	0.259	0.385	1.31E-79	ductal	ACSL3
DLG5.1	1.56E-83	-0.507795065	0.191	0.308	3.77E-79	ductal	DLG5
PSMB5.1	1.88E-83	-0.342252129	0.168	0.286	4.53E-79	ductal	PSMB5
DNAJB6.1	2.07E-83	-0.410855487	0.304	0.432	5.00E-79	ductal	DNAJB6
ARF4.1	3.74E-83	0.381664297	0.675	0.587	9.03E-79	ductal	ARF4
CDH1.1	6.26E-83	-0.381034973	0.533	0.651	1.51E-78	ductal	CDH1
STOM.1	1.00E-82	-0.483360414	0.191	0.307	2.42E-78	ductal	STOM
CDK13.1	1.37E-82	-0.469812038	0.348	0.472	3.31E-78	ductal	CDK13
ESD.1	1.40E-82	0.409925162	0.39	0.271	3.37E-78	ductal	ESD
EIF3H.1	2.27E-82	-0.356386075	0.566	0.662	5.47E-78	ductal	EIF3H
PROM1.1	4.06E-82	0.482184222	0.298	0.187	9.79E-78	ductal	PROM1
FNBP1.1	9.27E-82	-0.358976005	0.443	0.579	2.24E-77	ductal	FNBP1
ZNF292.1	1.22E-81	0.380201177	0.631	0.526	2.94E-77	ductal	ZNF292
MAP4.1	1.45E-81	-0.382585727	0.402	0.53	3.51E-77	ductal	MAP4
MSI2.1	1.88E-81	-0.421525241	0.268	0.391	4.54E-77	ductal	MSI2
BARX2.1	3.33E-81	0.576248021	0.318	0.212	8.03E-77	ductal	BARX2
SLC38A2.1	4.41E-81	-0.448864111	0.302	0.432	1.06E-76	ductal	SLC38A2
SLC4A7.1	4.57E-81	0.460169691	0.353	0.239	1.10E-76	ductal	SLC4A7
FEZ2.1	5.04E-81	0.524341362	0.409	0.301	1.22E-76	ductal	FEZ2
HERC4.1	6.71E-81	-0.4540561	0.454	0.574	1.62E-76	ductal	HERC4
GSK3B.1	8.18E-81	-0.404604487	0.322	0.448	1.97E-76	ductal	GSK3B
SLCO3A1.1	1.91E-80	0.456573255	0.25	0.148	4.60E-76	ductal	SLCO3A1
SPRED2.1	6.08E-80	-0.403690653	0.145	0.255	1.47E-75	ductal	SPRED2
RUFY3.1	6.68E-80	-0.359411244	0.168	0.284	1.61E-75	ductal	RUFY3
ZCCHC17.1	7.01E-80	0.442164769	0.327	0.219	1.69E-75	ductal	ZCCHC17
ZFAND3.1	1.14E-79	-0.379621661	0.714	0.79	2.76E-75	ductal	ZFAND3
C1QTNF3-A	3.19E-79	-0.470015208	0.244	0.366	7.70E-75	ductal	C1QTNF3-AMACR

EIF2S1.1	3.21E-79	0.410920679	0.264	0.162	7.74E-75	ductal	EIF2S1
UBE2V2.1	7.87E-79	0.396879293	0.319	0.211	1.90E-74	ductal	UBE2V2
AC072062.	1.10E-78	-0.377281525	0.391	0.523	2.66E-74	ductal	AC072062.1
PPP6R3.1	2.58E-78	-0.37005294	0.434	0.555	6.22E-74	ductal	PPP6R3
MKL1.1	6.95E-78	-0.355053666	0.417	0.546	1.68E-73	ductal	MKL1
DYNLT1.1	8.66E-78	0.42050235	0.644	0.567	2.09E-73	ductal	DYNLT1
DLEU2.1	1.56E-77	-0.402673056	0.155	0.264	3.75E-73	ductal	DLEU2
SRSF3.1	3.37E-77	0.565243137	0.496	0.396	8.13E-73	ductal	SRSF3
TTLL5.1	4.53E-77	-0.43249548	0.212	0.328	1.09E-72	ductal	TTLL5
TSC22D2.1	5.79E-77	-0.356861176	0.386	0.518	1.40E-72	ductal	TSC22D2
SH3BGRL.1	4.25E-76	-0.447215606	0.329	0.448	1.02E-71	ductal	SH3BGRL
FOXP1.1	6.62E-76	-0.424250935	0.574	0.673	1.60E-71	ductal	FOXP1
CTNND1.1	9.54E-76	-0.345144908	0.319	0.444	2.30E-71	ductal	CTNND1
MAST4.1	1.00E-75	-0.434557449	0.454	0.575	2.42E-71	ductal	MAST4
RBM47.1	1.29E-75	-0.349722532	0.566	0.681	3.11E-71	ductal	RBM47
TBL1X.1	2.32E-75	-0.340851443	0.149	0.259	5.60E-71	ductal	TBL1X
SUSD6.1	3.32E-75	-0.386732495	0.208	0.324	8.01E-71	ductal	SUSD6
RP1-292B1	6.50E-75	-0.301038952	0.146	0.257	1.57E-70	ductal	RP1-292B18.4
YTHDC1.1	8.49E-75	-0.37700193	0.309	0.432	2.05E-70	ductal	YTHDC1
ANKRD11.1	9.31E-75	-0.33368852	0.246	0.368	2.24E-70	ductal	ANKRD11
EIF4A1.1	1.36E-73	0.428739541	0.472	0.364	3.27E-69	ductal	EIF4A1
NHSL2.1	1.57E-73	0.378770636	0.349	0.24	3.80E-69	ductal	NHSL2
COX7B.1	9.38E-73	0.336622942	0.682	0.605	2.26E-68	ductal	COX7B
DUSP10.1	1.28E-72	-0.400883575	0.16	0.265	3.09E-68	ductal	DUSP10
PRRG4.1	2.48E-72	-0.363336491	0.195	0.308	5.99E-68	ductal	PRRG4
ATP6V1G1.	2.70E-72	-0.328154719	0.315	0.437	6.50E-68	ductal	ATP6V1G1
ADAM32.1	1.00E-71	-0.424609956	0.164	0.272	2.42E-67	ductal	ADAM32
EIF3K.1	1.21E-71	-0.550467978	0.274	0.367	2.92E-67	ductal	EIF3K
GPBP1.1	3.55E-71	-0.392882748	0.635	0.722	8.56E-67	ductal	GPBP1
KAT6B.1	5.29E-71	-0.37503208	0.189	0.3	1.28E-66	ductal	KAT6B
CTC-471J1.	7.00E-71	0.67169315	0.658	0.624	1.69E-66	ductal	CTC-471J1.9
ARPC3.1	7.82E-71	0.360661854	0.7	0.64	1.89E-66	ductal	ARPC3
ARFGEF2.1	1.20E-70	-0.364197836	0.299	0.419	2.90E-66	ductal	ARFGEF2
OSBPL8.1	2.18E-70	-0.376032448	0.174	0.281	5.26E-66	ductal	OSBPL8
KLHL24.1	2.24E-70	-0.316633558	0.276	0.398	5.39E-66	ductal	KLHL24
ATF4.1	3.34E-70	-0.351201588	0.323	0.442	8.05E-66	ductal	ATF4
CNOT4.1	9.94E-70	-0.329247608	0.325	0.447	2.40E-65	ductal	CNOT4
CREBBP.1	2.05E-69	-0.330113735	0.23	0.345	4.95E-65	ductal	CREBBP
CDC42BPA.	3.63E-69	-0.377587903	0.31	0.428	8.75E-65	ductal	CDC42BPA
KCMF1.1	4.18E-69	-0.348701844	0.265	0.383	1.01E-64	ductal	KCMF1
HNRNPM.1	8.59E-69	-0.332364696	0.195	0.305	2.07E-64	ductal	HNRNPM
YAP1.1	1.09E-68	-0.337252193	0.462	0.579	2.63E-64	ductal	YAP1
RTCB.1	1.74E-68	0.386129845	0.474	0.371	4.20E-64	ductal	RTCB
UFM1.1	2.73E-68	0.384581761	0.391	0.287	6.59E-64	ductal	UFM1
ITGB8.1	3.82E-68	0.440922639	0.559	0.461	9.20E-64	ductal	ITGB8
ZFAS1.1	4.18E-68	0.267848021	0.951	0.915	1.01E-63	ductal	ZFAS1
SMARCC1.1	6.15E-68	-0.335814414	0.222	0.334	1.48E-63	ductal	SMARCC1

RP5-857K2	6.73E-68	-0.328233908	0.359	0.481	1.62E-63	ductal	RP5-857K21.4
RPL41.1	8.17E-68	0.679637482	0.938	0.882	1.97E-63	ductal	RPL41
BACE2.1	1.25E-67	0.43387141	0.377	0.275	3.03E-63	ductal	BACE2
CFL1.1	1.42E-67	-0.411913007	0.251	0.359	3.42E-63	ductal	CFL1
EEF2.1	3.38E-67	-0.41170816	0.333	0.458	8.15E-63	ductal	EEF2
POLR1D.1	3.41E-67	-0.319631971	0.163	0.265	8.22E-63	ductal	POLR1D
TOP1.1	6.79E-67	-0.333556748	0.375	0.491	1.64E-62	ductal	TOP1
DPM1.1	7.65E-67	0.36594229	0.307	0.207	1.84E-62	ductal	DPM1
TIAM1.1	9.15E-67	0.549401867	0.304	0.207	2.21E-62	ductal	TIAM1
CD63.1	1.28E-66	-0.394953942	0.315	0.425	3.08E-62	ductal	CD63
UBE2G1.1	1.31E-66	-0.326361973	0.191	0.299	3.17E-62	ductal	UBE2G1
BRD2.1	1.94E-66	-0.338173464	0.204	0.311	4.69E-62	ductal	BRD2
SDC4.1	2.14E-66	0.612065958	0.388	0.295	5.15E-62	ductal	SDC4
UBR5.1	2.45E-66	-0.322630938	0.398	0.516	5.92E-62	ductal	UBR5
OTUD7B.1	2.59E-66	-0.360230716	0.227	0.338	6.24E-62	ductal	OTUD7B
FGD6.1	2.72E-66	-0.371667258	0.276	0.388	6.55E-62	ductal	FGD6
GBE1.1	2.78E-66	0.512521194	0.424	0.322	6.69E-62	ductal	GBE1
COX6C.1	3.26E-66	-0.689053859	0.754	0.769	7.87E-62	ductal	COX6C
USP47.1	3.64E-66	-0.366079634	0.35	0.466	8.78E-62	ductal	USP47
WNK1.1	4.52E-66	-0.31773578	0.178	0.282	1.09E-61	ductal	WNK1
SNRPD1.1	5.07E-66	0.396256697	0.35	0.249	1.22E-61	ductal	SNRPD1
EIF3L.1	5.16E-66	0.27701116	0.662	0.547	1.25E-61	ductal	EIF3L
KIF13A.1	6.49E-66	-0.437381135	0.326	0.44	1.56E-61	ductal	KIF13A
CNKSR3.1	7.26E-66	0.693051529	0.366	0.274	1.75E-61	ductal	CNKSR3
TMEM150(	2.56E-65	0.357798725	0.265	0.173	6.17E-61	ductal	TMEM150C
CCT2.1	9.07E-65	0.391327021	0.366	0.267	2.19E-60	ductal	CCT2
SLC38A1.1	1.28E-64	-0.348888963	0.325	0.437	3.10E-60	ductal	SLC38A1
PRDX3.1	1.71E-64	-0.345133623	0.164	0.264	4.12E-60	ductal	PRDX3
RPL7.1	3.97E-64	-0.345036864	0.946	0.835	9.57E-60	ductal	RPL7
PDZD8.1	5.26E-64	-0.382455811	0.171	0.27	1.27E-59	ductal	PDZD8
S100A6.1	1.22E-63	-0.828898269	0.532	0.601	2.93E-59	ductal	S100A6
SSR2.1	1.59E-63	0.285113826	0.733	0.658	3.84E-59	ductal	SSR2
VAV3.1	3.74E-63	-0.390812032	0.222	0.327	9.03E-59	ductal	VAV3
SEP15.1	3.81E-63	0.370064982	0.473	0.38	9.19E-59	ductal	15-Sep
RBX1.1	4.11E-63	0.365587557	0.497	0.406	9.92E-59	ductal	RBX1
SARAF.1	1.10E-62	-0.291143604	0.282	0.398	2.64E-58	ductal	SARAF
ZSWIM6.1	1.24E-62	-0.367812514	0.432	0.54	2.98E-58	ductal	ZSWIM6
ASH1L.1	1.94E-62	-0.3261521	0.484	0.593	4.67E-58	ductal	ASH1L
EIF1.1	7.46E-62	-0.296656839	0.408	0.526	1.80E-57	ductal	EIF1
C8orf59.1	1.37E-61	0.336580474	0.35	0.25	3.31E-57	ductal	C8orf59
IGF1R.1	1.75E-61	-0.316766544	0.433	0.548	4.22E-57	ductal	IGF1R
KYNU.1	2.49E-61	0.772374108	0.301	0.208	6.01E-57	ductal	KYNU
HNRNPH2.:	2.50E-61	0.332533779	0.286	0.191	6.04E-57	ductal	HNRNPH2
SLC12A2.1	2.58E-61	0.590868554	0.466	0.377	6.23E-57	ductal	SLC12A2
SEMA6A.1	2.60E-61	0.570973372	0.28	0.19	6.27E-57	ductal	SEMA6A
ZRANB2.1	2.91E-61	0.359761058	0.291	0.198	7.02E-57	ductal	ZRANB2
ELF2.1	3.10E-61	-0.353852976	0.322	0.431	7.48E-57	ductal	ELF2

EP300.1	3.18E-61	-0.321342561	0.161	0.258	7.68E-57	ductal	EP300
PAM.1	5.20E-61	0.376534008	0.527	0.433	1.25E-56	ductal	PAM
AHNAK.1	1.30E-60	-0.333898468	0.29	0.399	3.14E-56	ductal	AHNAK
RUNX1.1	1.34E-60	-0.282112912	0.579	0.685	3.23E-56	ductal	RUNX1
DNAJC8.1	2.44E-60	0.337943968	0.28	0.186	5.89E-56	ductal	DNAJC8
SRPK1.1	2.94E-60	0.407612754	0.542	0.455	7.09E-56	ductal	SRPK1
CD164.1	4.37E-60	-0.340335703	0.221	0.322	1.05E-55	ductal	CD164
USP53.1	4.83E-60	0.61077	0.661	0.603	1.16E-55	ductal	USP53
LRRFIP1.1	6.86E-60	-0.254577817	0.559	0.675	1.65E-55	ductal	LRRFIP1
CRY1.1	8.29E-60	-0.384597211	0.319	0.428	2.00E-55	ductal	CRY1
RBBP6.1	1.51E-59	-0.307506286	0.217	0.322	3.63E-55	ductal	RBBP6
UBE2K.1	1.55E-59	-0.315285432	0.312	0.424	3.73E-55	ductal	UBE2K
FBXO32.1	2.09E-59	0.57727673	0.428	0.336	5.04E-55	ductal	FBXO32
ZFAND5.1	2.28E-59	-0.318694219	0.367	0.477	5.50E-55	ductal	ZFAND5
PERP.1	2.57E-59	-0.288542672	0.385	0.498	6.19E-55	ductal	PERP
RARS.1	2.58E-59	0.339006279	0.28	0.189	6.23E-55	ductal	RARS
C2orf88.1	3.29E-59	0.396925484	0.41	0.315	7.93E-55	ductal	C2orf88
BCAS3.1	3.95E-59	0.264959421	0.777	0.716	9.52E-55	ductal	BCAS3
SLTM.1	5.57E-59	-0.303155991	0.231	0.335	1.34E-54	ductal	SLTM
HSPH1.1	7.38E-59	-0.426455969	0.223	0.324	1.78E-54	ductal	HSPH1
SREBF2.1	7.91E-59	-0.31370641	0.309	0.421	1.91E-54	ductal	SREBF2
SNRPG.1	9.45E-59	0.358911885	0.568	0.49	2.28E-54	ductal	SNRPG
TGIF1.1	1.03E-58	-0.342257951	0.163	0.256	2.48E-54	ductal	TGIF1
ATP5E.1	1.49E-58	-0.457504292	0.502	0.59	3.59E-54	ductal	ATP5E
RNMT.1	1.84E-58	0.359417608	0.393	0.293	4.43E-54	ductal	RNMT
PTPRE.1	3.71E-58	-0.464966454	0.208	0.302	8.95E-54	ductal	PTPRE
PPP2CB.1	3.77E-58	-0.294101567	0.272	0.381	9.08E-54	ductal	PPP2CB
LIMCH1.1	3.87E-58	-0.415757283	0.428	0.525	9.33E-54	ductal	LIMCH1
MYO1B.1	9.41E-58	-0.405637755	0.383	0.488	2.27E-53	ductal	MYO1B
ATXN2.1	1.15E-57	-0.302182501	0.269	0.376	2.77E-53	ductal	ATXN2
CD46.1	1.21E-57	-0.299237454	0.333	0.446	2.92E-53	ductal	CD46
SMCHD1.1	1.67E-57	-0.375203505	0.346	0.447	4.02E-53	ductal	SMCHD1
MAN1A1.1	2.60E-57	-0.316360125	0.17	0.265	6.28E-53	ductal	MAN1A1
AC018890.	2.69E-57	-0.319757162	0.18	0.277	6.49E-53	ductal	AC018890.6
GRAMD3.1	2.71E-57	-0.351329783	0.282	0.389	6.54E-53	ductal	GRAMD3
DMD.1	3.97E-57	0.4739159	0.318	0.228	9.56E-53	ductal	DMD
CD9.1	4.09E-57	-0.371834893	0.324	0.427	9.85E-53	ductal	CD9
TMEM87A.	4.71E-57	-0.274517159	0.317	0.428	1.14E-52	ductal	TMEM87A
ATP6V1E1.	7.23E-57	0.340171141	0.353	0.26	1.74E-52	ductal	ATP6V1E1
ATF3.1	1.50E-56	-0.339961322	0.34	0.449	3.62E-52	ductal	ATF3
CTNNA1.1	1.91E-56	-0.28831696	0.475	0.578	4.59E-52	ductal	CTNNA1
PLCB1.1	2.89E-56	0.489759923	0.28	0.192	6.98E-52	ductal	PLCB1
DTNB.1	3.14E-56	0.378518831	0.489	0.392	7.58E-52	ductal	DTNB
KLF6.1	3.90E-56	-0.314009513	0.507	0.611	9.41E-52	ductal	KLF6
SRSF4.1	4.20E-56	-0.277307052	0.232	0.334	1.01E-51	ductal	SRSF4
RB1CC1.1	6.14E-56	-0.305585423	0.416	0.524	1.48E-51	ductal	RB1CC1
CSNK1A1.1	7.96E-56	-0.288447235	0.595	0.693	1.92E-51	ductal	CSNK1A1

RNF11.1	9.05E-56	-0.290266083	0.188	0.284	2.18E-51	ductal	RNF11
GABARAP.1	1.11E-55	0.359018421	0.424	0.327	2.68E-51	ductal	GABARAP
ARHGEF28.	1.34E-55	0.492235725	0.332	0.241	3.24E-51	ductal	ARHGEF28
CCT3.1	1.75E-55	-0.298709587	0.298	0.4	4.23E-51	ductal	CCT3
SLC22A23.1	1.79E-55	-0.308593537	0.189	0.286	4.31E-51	ductal	SLC22A23
BAZ1A.1	2.97E-55	-0.372713575	0.322	0.424	7.17E-51	ductal	BAZ1A
UBE2R2.1	3.19E-55	-0.285745494	0.305	0.411	7.69E-51	ductal	UBE2R2
EIF3I.1	3.54E-55	0.303688986	0.643	0.567	8.53E-51	ductal	EIF3I
PTPRK.1	8.31E-55	-0.293830099	0.735	0.794	2.00E-50	ductal	PTPRK
CAB39.1	9.01E-55	-0.343557757	0.301	0.4	2.17E-50	ductal	CAB39
LAMC2.1	1.18E-54	0.369437658	0.324	0.23	2.84E-50	ductal	LAMC2
TMBIM6.1	1.23E-54	0.328525313	0.873	0.854	2.96E-50	ductal	TMBIM6
RNF144B.1	1.72E-54	0.489476468	0.343	0.256	4.14E-50	ductal	RNF144B
RAB21.1	1.92E-54	-0.31337929	0.203	0.301	4.62E-50	ductal	RAB21
YY1.1	2.15E-54	-0.274179961	0.165	0.257	5.17E-50	ductal	YY1
POLE2.1	2.50E-54	0.314658256	0.297	0.205	6.02E-50	ductal	POLE2
MAP2K4.1	2.60E-54	0.363322234	0.498	0.412	6.27E-50	ductal	MAP2K4
GPBP1L1.1	2.72E-54	-0.292503517	0.281	0.384	6.56E-50	ductal	GPBP1L1
CTTNBP2NI	3.27E-54	0.346776759	0.265	0.18	7.87E-50	ductal	CTTNBP2NL
SCGB2A2.1	3.32E-54	-0.506063487	0.21	0.314	8.02E-50	ductal	SCGB2A2
CLIC6.1	4.39E-54	-0.374053549	0.164	0.252	1.06E-49	ductal	CLIC6
SEMA4B.1	5.32E-54	-0.272551279	0.167	0.26	1.28E-49	ductal	SEMA4B
ITPR2.1	6.63E-54	-0.3540992	0.412	0.513	1.60E-49	ductal	ITPR2
ANO6.1	9.35E-54	-0.342447654	0.463	0.556	2.25E-49	ductal	ANO6
CIR1.1	1.33E-53	0.327803467	0.314	0.224	3.20E-49	ductal	CIR1
NAV2.1	1.74E-53	0.44988989	0.473	0.383	4.20E-49	ductal	NAV2
MAN2A1.1	5.95E-53	-0.291929364	0.258	0.359	1.44E-48	ductal	MAN2A1
PDK3.1	7.56E-53	0.454648192	0.282	0.198	1.82E-48	ductal	PDK3
MAP4K4.1	1.02E-52	0.429673366	0.289	0.205	2.47E-48	ductal	MAP4K4
TBC1D3P1-	1.18E-52	0.436448065	0.638	0.56	2.85E-48	ductal	TBC1D3P1-DHX40P1
PIP5K1A.1	1.31E-52	-0.294625731	0.242	0.341	3.15E-48	ductal	PIP5K1A
RALGAPA1.	1.41E-52	-0.334701928	0.247	0.342	3.39E-48	ductal	RALGAPA1
CXCR4.1	2.07E-52	0.399549607	0.272	0.187	5.00E-48	ductal	CXCR4
CTBP2.1	2.76E-52	-0.264724044	0.28	0.386	6.65E-48	ductal	CTBP2
RNF10.1	3.35E-52	-0.283929868	0.171	0.26	8.07E-48	ductal	RNF10
ZBTB16.1	4.34E-52	-0.318670367	0.216	0.314	1.05E-47	ductal	ZBTB16
PTPN14.1	4.44E-52	0.462102745	0.356	0.271	1.07E-47	ductal	PTPN14
YBX3.1	5.27E-52	-0.281849781	0.227	0.324	1.27E-47	ductal	YBX3
CREBRF.1	6.71E-52	-0.272659336	0.207	0.302	1.62E-47	ductal	CREBRF
AC090498.	1.29E-51	0.33552784	0.363	0.275	3.11E-47	ductal	AC090498.1
CYCS.1	1.34E-51	0.332060535	0.569	0.493	3.23E-47	ductal	CYCS
ATP5J.1	2.98E-51	0.298744467	0.551	0.47	7.19E-47	ductal	ATP5J
RAB7A.1	3.49E-51	-0.277165542	0.39	0.491	8.41E-47	ductal	RAB7A
CHCHD2.1	4.14E-51	-0.346104827	0.201	0.291	9.97E-47	ductal	CHCHD2
LAMB3.1	5.67E-51	0.4646258	0.294	0.209	1.37E-46	ductal	LAMB3
SRRM2.1	7.84E-51	-0.266948921	0.163	0.251	1.89E-46	ductal	SRRM2
EEF1B2.1	1.92E-50	-0.641554188	0.736	0.726	4.64E-46	ductal	EEF1B2

CBLB.1	2.98E-50	-0.312418465	0.262	0.359	7.19E-46	ductal	CBLB
RARRES1.1	3.20E-50	0.453293524	0.266	0.181	7.72E-46	ductal	RARRES1
AKAP13.1	4.75E-50	-0.329719185	0.557	0.646	1.14E-45	ductal	AKAP13
GNG12.1	5.68E-50	-0.30933461	0.269	0.366	1.37E-45	ductal	GNG12
MYO10.1	1.25E-49	-0.329137703	0.199	0.289	3.01E-45	ductal	MYO10
UBE2L3.1	2.04E-49	0.329044089	0.49	0.41	4.92E-45	ductal	UBE2L3
ARID1B.1	2.22E-49	-0.296933387	0.432	0.535	5.35E-45	ductal	ARID1B
GHITM.1	3.52E-49	0.334659664	0.494	0.416	8.49E-45	ductal	GHITM
MAST2.1	4.59E-49	-0.324746735	0.171	0.258	1.11E-44	ductal	MAST2
ASPH.1	6.09E-49	-0.299175248	0.228	0.321	1.47E-44	ductal	ASPH
EIF4G3.1	7.88E-49	-0.314135699	0.452	0.55	1.90E-44	ductal	EIF4G3
DIAPH1.1	8.09E-49	-0.276265823	0.239	0.335	1.95E-44	ductal	DIAPH1
CALR.1	8.48E-49	-0.304341632	0.173	0.261	2.05E-44	ductal	CALR
ARID1A.1	9.10E-49	-0.269520475	0.186	0.275	2.20E-44	ductal	ARID1A
LCOR.1	1.21E-48	-0.330287554	0.213	0.3	2.93E-44	ductal	LCOR
EMP1.1	1.76E-48	0.613970935	0.709	0.675	4.25E-44	ductal	EMP1
ARL1.1	6.13E-48	0.303928599	0.304	0.219	1.48E-43	ductal	ARL1
ATP5H.1	6.19E-48	0.278771447	0.407	0.318	1.49E-43	ductal	ATP5H
HNRNPC.1	6.22E-48	0.287189649	0.803	0.773	1.50E-43	ductal	HNRNPC
CCT8.1	1.57E-47	0.343966016	0.333	0.253	3.78E-43	ductal	CCT8
CLIP1.1	1.69E-47	-0.307567124	0.34	0.438	4.07E-43	ductal	CLIP1
BCOR.1	1.99E-47	0.252453411	0.581	0.474	4.80E-43	ductal	BCOR
CLINT1.1	2.15E-47	0.347825223	0.301	0.218	5.17E-43	ductal	CLINT1
NFAT5.1	2.28E-47	-0.301446279	0.572	0.664	5.51E-43	ductal	NFAT5
OLA1.1	3.77E-47	-0.312406198	0.268	0.36	9.09E-43	ductal	OLA1
SIK3.1	4.21E-47	0.511055578	0.977	0.97	1.01E-42	ductal	SIK3
CALCOCO2	4.46E-47	0.328517216	0.304	0.222	1.07E-42	ductal	CALCOCO2
TMEM41B.	6.37E-47	-0.269266122	0.195	0.284	1.54E-42	ductal	TMEM41B
CHD2.1	7.47E-47	-0.262455812	0.483	0.589	1.80E-42	ductal	CHD2
CYB5A.1	2.13E-46	-0.356672836	0.207	0.292	5.14E-42	ductal	CYB5A
ATXN1.1	2.39E-46	-0.297095111	0.455	0.554	5.76E-42	ductal	ATXN1
CYR61.1	2.83E-46	-0.328001609	0.211	0.308	6.82E-42	ductal	CYR61
CAPN8.1	3.98E-46	-0.265820394	0.166	0.253	9.60E-42	ductal	CAPN8
HIST1H2BD	4.00E-46	-0.286588045	0.243	0.337	9.64E-42	ductal	HIST1H2BD
KMT2E.1	4.46E-46	-0.315545915	0.408	0.503	1.08E-41	ductal	KMT2E
PFDN4.1	4.87E-46	0.294232307	0.392	0.304	1.17E-41	ductal	PFDN4
NDUFV2.1	8.60E-46	-0.28590179	0.217	0.306	2.07E-41	ductal	NDUFV2
THSD4-AS1	1.31E-45	0.30611415	0.508	0.43	3.15E-41	ductal	THSD4-AS1
PNPLA8.1	1.40E-45	0.362287384	0.332	0.249	3.38E-41	ductal	PNPLA8
PPM1H.1	3.34E-45	0.451313773	0.363	0.286	8.06E-41	ductal	PPM1H
CWC15.1	3.52E-45	0.289046914	0.339	0.254	8.48E-41	ductal	CWC15
SF3B6.1	4.47E-45	0.278539803	0.65	0.584	1.08E-40	ductal	SF3B6
RP11-511B	5.62E-45	0.287720169	0.555	0.48	1.35E-40	ductal	RP11-511B23.2
PAPSS1.1	5.65E-45	0.385441547	0.277	0.201	1.36E-40	ductal	PAPSS1
FAM185A.1	1.22E-44	0.315805045	0.259	0.183	2.93E-40	ductal	FAM185A
SMC5.1	1.77E-44	0.384267412	0.355	0.277	4.27E-40	ductal	SMC5
OCLN.1	1.92E-44	-0.2770575	0.28	0.374	4.63E-40	ductal	OCLN

OSMR.1	2.18E-44	0.336069944	0.359	0.277	5.26E-40	ductal	OSMR
NCOR1.1	3.80E-44	-0.2618251	0.366	0.459	9.17E-40	ductal	NCOR1
LAMTOR5.1	4.05E-44	0.270361724	0.63	0.563	9.78E-40	ductal	LAMTOR5
TTC1.1	1.73E-43	0.315139147	0.323	0.244	4.17E-39	ductal	TTC1
HLA-C.1	2.23E-43	-0.337330652	0.262	0.346	5.37E-39	ductal	HLA-C
NUP153.1	2.23E-43	-0.265996505	0.228	0.318	5.37E-39	ductal	NUP153
TEAD1.1	2.45E-43	-0.278661253	0.394	0.491	5.91E-39	ductal	TEAD1
RBPMS.1	3.21E-43	0.357114316	0.697	0.639	7.74E-39	ductal	RBPMS
ARID4B.1	3.32E-43	-0.283983033	0.498	0.593	7.99E-39	ductal	ARID4B
CASC15.1	6.54E-43	-0.580943484	0.485	0.547	1.58E-38	ductal	CASC15
USP25.1	1.59E-42	-0.296751993	0.209	0.292	3.83E-38	ductal	USP25
TNRC6B.1	1.78E-42	-0.266207546	0.43	0.524	4.28E-38	ductal	TNRC6B
B4GALT1.1	1.80E-42	-0.252210358	0.381	0.477	4.34E-38	ductal	B4GALT1
ASTN2.1	3.64E-42	-0.302774418	0.222	0.307	8.79E-38	ductal	ASTN2
NEAT1.1	4.87E-42	0.27713743	0.964	0.938	1.18E-37	ductal	NEAT1
PPP3CA.1	5.09E-42	-0.312091652	0.514	0.596	1.23E-37	ductal	PPP3CA
SOS1.1	1.56E-41	-0.2746161	0.315	0.405	3.76E-37	ductal	SOS1
MARCKS.1	1.65E-41	0.365769703	0.4	0.322	3.99E-37	ductal	MARCKS
CCDC6.1	2.26E-41	-0.30086367	0.266	0.351	5.46E-37	ductal	CCDC6
SMAD2.1	1.04E-40	-0.274099557	0.253	0.337	2.50E-36	ductal	SMAD2
CXCL1.1	1.60E-40	-0.826750129	0.224	0.3	3.86E-36	ductal	CXCL1
ANKRD12.1	1.68E-40	-0.254682037	0.629	0.702	4.06E-36	ductal	ANKRD12
OVOS2.1	2.06E-40	0.432405593	0.324	0.238	4.97E-36	ductal	OVOS2
WWP1.1	2.25E-40	-0.284636007	0.262	0.344	5.42E-36	ductal	WWP1
SCOC.1	2.86E-40	0.258124411	0.291	0.213	6.89E-36	ductal	SCOC
NSRP1.1	3.20E-40	0.279907825	0.257	0.184	7.73E-36	ductal	NSRP1
CXCL8.1	3.86E-40	0.868712617	0.499	0.416	9.31E-36	ductal	CXCL8
CLASP2.1	4.88E-40	-0.273200685	0.223	0.306	1.18E-35	ductal	CLASP2
IER3.1	1.17E-39	-0.283325216	0.267	0.353	2.83E-35	ductal	IER3
CAPZA2.1	1.39E-39	0.292726264	0.357	0.28	3.36E-35	ductal	CAPZA2
GNL3.1	1.78E-39	0.297840904	0.295	0.219	4.30E-35	ductal	GNL3
COPZ1.1	1.88E-39	0.259683395	0.375	0.292	4.53E-35	ductal	COPZ1
JUN.1	2.73E-39	-0.301053023	0.189	0.266	6.57E-35	ductal	JUN
PCM1.1	3.13E-39	-0.258072852	0.209	0.29	7.56E-35	ductal	PCM1
DPYD.1	5.21E-39	0.297142955	0.569	0.492	1.26E-34	ductal	DPYD
WDFY2.1	8.76E-39	-0.261963912	0.216	0.298	2.11E-34	ductal	WDFY2
MRPL22.1	9.39E-39	0.279325672	0.263	0.19	2.26E-34	ductal	MRPL22
CDK8.1	1.70E-38	-0.319654541	0.195	0.271	4.10E-34	ductal	CDK8
PELI1.1	1.93E-38	0.365238139	0.377	0.302	4.65E-34	ductal	PELI1
KDM6A.1	2.04E-38	-0.250534289	0.312	0.402	4.93E-34	ductal	KDM6A
LRRFIP2.1	2.73E-38	0.563876559	0.775	0.756	6.59E-34	ductal	LRRFIP2
MACF1.1	4.72E-38	-0.280333596	0.678	0.748	1.14E-33	ductal	MACF1
TXN.1	6.51E-38	0.326192722	0.697	0.664	1.57E-33	ductal	TXN
EPB41L5.1	1.31E-37	-0.263229608	0.246	0.329	3.16E-33	ductal	EPB41L5
LMNA.1	1.86E-37	-0.264249999	0.184	0.263	4.48E-33	ductal	LMNA
TMA7.1	2.23E-37	-0.308481366	0.557	0.631	5.37E-33	ductal	TMA7
STAT3.1	2.57E-37	-0.273013662	0.492	0.568	6.19E-33	ductal	STAT3

SFRP1.1	3.33E-37	0.271330429	0.272	0.199	8.03E-33	ductal	SFRP1
ANKRD17.1	5.43E-37	-0.253500126	0.456	0.542	1.31E-32	ductal	ANKRD17
GAREM1.1	6.56E-37	-0.278391206	0.271	0.352	1.58E-32	ductal	GAREM1
TBC1D8.1	9.73E-37	-0.257102421	0.376	0.464	2.35E-32	ductal	TBC1D8
DHRSX.1	1.27E-36	-0.27633184	0.228	0.309	3.05E-32	ductal	DHRSX
CLMN.1	1.30E-36	0.384779269	0.571	0.512	3.13E-32	ductal	CLMN
RBMS1.1	1.83E-36	-0.307301629	0.296	0.376	4.41E-32	ductal	RBMS1
NTN4.1	9.01E-36	-0.272032556	0.252	0.332	2.17E-31	ductal	NTN4
ATP5F1.1	9.32E-36	0.260841886	0.422	0.346	2.25E-31	ductal	ATP5F1
FAM19A2.1	1.05E-35	-0.413235572	0.545	0.626	2.54E-31	ductal	FAM19A2
PBX1.1	1.13E-35	-0.253940805	0.215	0.294	2.72E-31	ductal	PBX1
TMOD3.1	1.52E-35	-0.279685197	0.356	0.434	3.68E-31	ductal	TMOD3
S100A11.1	1.83E-35	0.330233016	0.751	0.709	4.42E-31	ductal	S100A11
DENND4A.1	2.75E-35	-0.388182565	0.556	0.619	6.62E-31	ductal	DENND4A
BTF3.1	4.10E-35	-0.450977285	0.907	0.878	9.89E-31	ductal	BTF3
FMNL2.1	6.46E-35	0.426506093	0.395	0.329	1.56E-30	ductal	FMNL2
PTGES3.1	1.15E-34	-0.26034502	0.199	0.272	2.76E-30	ductal	PTGES3
TCEAL8.1	2.77E-34	0.265647044	0.278	0.209	6.68E-30	ductal	TCEAL8
SESTD1.1	2.95E-34	0.304518118	0.53	0.465	7.12E-30	ductal	SESTD1
NRIP1.1	3.07E-34	-0.273717191	0.223	0.299	7.41E-30	ductal	NRIP1
RNF24.1	1.35E-33	0.306152292	0.377	0.307	3.26E-29	ductal	RNF24
CHD9.1	1.69E-33	0.2984741	0.445	0.378	4.06E-29	ductal	CHD9
TMTC2.1	1.75E-33	-0.255035282	0.327	0.411	4.23E-29	ductal	TMTC2
FDFT1.1	2.36E-33	-0.262290527	0.268	0.344	5.69E-29	ductal	FDFT1
SMAD3.1	4.17E-33	-0.314881433	0.23	0.298	1.01E-28	ductal	SMAD3
GPD2.1	9.53E-33	-0.311547071	0.287	0.36	2.30E-28	ductal	GPD2
STK39.1	9.67E-33	-0.388839003	0.196	0.267	2.33E-28	ductal	STK39
MAML3.1	1.21E-32	-0.313901227	0.305	0.381	2.92E-28	ductal	MAML3
FRYL.1	1.69E-32	-0.261671492	0.351	0.428	4.07E-28	ductal	FRYL
RAPGEF5.1	2.20E-32	0.31105566	0.385	0.312	5.30E-28	ductal	RAPGEF5
PSMC6.1	2.34E-32	0.275365565	0.359	0.291	5.64E-28	ductal	PSMC6
MMADHC.1	5.74E-32	0.265487469	0.351	0.284	1.38E-27	ductal	MMADHC
PIN4.1	7.61E-32	0.257863758	0.311	0.243	1.84E-27	ductal	PIN4
REL.1	1.38E-31	-0.255724162	0.255	0.33	3.33E-27	ductal	REL
SEC11A.1	2.34E-31	0.250782165	0.524	0.461	5.63E-27	ductal	SEC11A
EIF4E2.1	4.14E-31	0.25400183	0.365	0.3	9.98E-27	ductal	EIF4E2
IRF2.1	6.59E-31	0.258086817	0.274	0.209	1.59E-26	ductal	IRF2
CX3CL1.1	9.76E-31	-0.262721801	0.189	0.259	2.35E-26	ductal	CX3CL1
SPATS2L.1	1.32E-30	0.322681896	0.279	0.216	3.19E-26	ductal	SPATS2L
NDUFB4.1	1.43E-30	-0.262547702	0.386	0.467	3.44E-26	ductal	NDUFB4
PSTPIP2.1	1.59E-30	0.362478257	0.265	0.203	3.83E-26	ductal	PSTPIP2
ARHGEF38.1	5.28E-30	-0.288343551	0.44	0.513	1.27E-25	ductal	ARHGEF38
SORBS2.1	6.82E-30	0.44267285	0.423	0.356	1.64E-25	ductal	SORBS2
DAPK2.1	7.57E-30	0.281008812	0.275	0.21	1.82E-25	ductal	DAPK2
PRDM2.1	1.96E-29	-0.318579717	0.252	0.325	4.73E-25	ductal	PRDM2
GPRC5A.1	2.12E-29	-0.27820975	0.346	0.42	5.11E-25	ductal	GPRC5A
HES1.1	2.24E-29	-0.409825389	0.3	0.371	5.40E-25	ductal	HES1

PALLD.1	3.53E-29	0.349049516	0.439	0.381	8.51E-25	ductal	PALLD
PSMD11.1	8.70E-29	0.267004151	0.297	0.235	2.10E-24	ductal	PSMD11
CAPN2.1	1.24E-28	0.259521803	0.335	0.27	2.98E-24	ductal	CAPN2
PNRC1.1	3.44E-28	0.279586325	0.454	0.397	8.29E-24	ductal	PNRC1
TJP2.1	3.65E-28	0.329644375	0.358	0.301	8.79E-24	ductal	TJP2
RPS2.1	5.54E-28	-0.278728476	0.231	0.303	1.34E-23	ductal	RPS2
ABHD18.1	9.51E-28	-0.271354829	0.303	0.372	2.29E-23	ductal	ABHD18
RASA2.1	2.73E-27	0.336641372	0.573	0.521	6.58E-23	ductal	RASA2
YPEL5.1	2.81E-27	0.288254474	0.436	0.38	6.76E-23	ductal	YPEL5
RIPK2.1	3.78E-27	0.362925506	0.318	0.258	9.12E-23	ductal	RIPK2
HNRNPA3.1	3.99E-27	0.257383514	0.302	0.242	9.63E-23	ductal	HNRNPA3
CAST.1	1.21E-26	0.254268522	0.546	0.494	2.93E-22	ductal	CAST
TPT1-AS1.1	1.83E-26	0.27866689	0.272	0.214	4.42E-22	ductal	TPT1-AS1
GLUL.1	2.01E-26	-0.250815655	0.331	0.407	4.84E-22	ductal	GLUL
AC016831.	3.90E-26	0.306951453	0.296	0.236	9.40E-22	ductal	AC016831.7
MAP4K5.1	4.73E-26	0.302383327	0.325	0.265	1.14E-21	ductal	MAP4K5
ARHGEF10.	1.51E-25	0.338956013	0.254	0.2	3.65E-21	ductal	ARHGEF10
SEPP1.1	1.53E-25	-0.262267563	0.272	0.339	3.69E-21	ductal	SEPP1
S100A14.1	4.09E-25	0.267454518	0.754	0.724	9.86E-21	ductal	S100A14
CD55.1	4.50E-25	0.281018936	0.326	0.269	1.08E-20	ductal	CD55
PTBP2.1	9.05E-25	0.265052147	0.381	0.324	2.18E-20	ductal	PTBP2
CMIP.1	1.24E-24	0.268505439	0.303	0.246	2.99E-20	ductal	CMIP
JARID2.1	3.11E-24	-0.378592599	0.215	0.277	7.49E-20	ductal	JARID2
MPZL1.1	3.51E-24	0.267344084	0.41	0.355	8.46E-20	ductal	MPZL1
RPSA.1	5.14E-24	-0.431356109	0.831	0.798	1.24E-19	ductal	RPSA
EXT1.1	9.46E-24	0.303487422	0.531	0.478	2.28E-19	ductal	EXT1
CRIM1.1	1.25E-23	0.372928769	0.469	0.424	3.02E-19	ductal	CRIM1
LSAMP.1	2.35E-23	-0.44204311	0.449	0.487	5.67E-19	ductal	LSAMP
PDE8A.1	6.30E-23	0.275173431	0.363	0.308	1.52E-18	ductal	PDE8A
CLK1.1	4.02E-22	0.275417545	0.45	0.399	9.69E-18	ductal	CLK1
KDM7A.1	4.64E-21	0.253560606	0.308	0.257	1.12E-16	ductal	KDM7A
TACSTD2.1	7.05E-21	-0.31891673	0.717	0.739	1.70E-16	ductal	TACSTD2
ATP13A3.1	3.57E-19	0.286541461	0.293	0.245	8.62E-15	ductal	ATP13A3
RPS27.1	4.59E-19	1.065799586	0.648	0.631	1.11E-14	ductal	RPS27
ERO1A.1	3.66E-18	0.355366203	0.467	0.426	8.84E-14	ductal	ERO1A
ADAM17.1	3.69E-18	0.267581125	0.41	0.368	8.90E-14	ductal	ADAM17
HIVEP2.1	1.18E-17	-0.379451804	0.429	0.465	2.85E-13	ductal	HIVEP2
DHFR.1	9.13E-15	0.262560868	0.786	0.794	2.20E-10	ductal	DHFR
DAAM1.1	7.74E-13	0.299100891	0.524	0.501	1.87E-08	ductal	DAAM1
RP11-37B2	2.45E-12	0.259501697	0.25	0.214	5.90E-08	ductal	RP11-37B2.1
TNFSF10.1	9.84E-12	0.395674103	0.487	0.466	2.37E-07	ductal	TNFSF10
FOSB.1	1.53E-11	0.262509169	0.413	0.386	3.68E-07	ductal	FOSB
IFRD1.1	2.64E-11	0.253683866	0.37	0.342	6.38E-07	ductal	IFRD1
ATP5EP2.1	2.93E-11	-0.265058578	0.287	0.317	7.06E-07	ductal	ATP5EP2
BAIAP2L1.1	2.14E-10	-0.355432154	0.953	0.911	5.17E-06	ductal	BAIAP2L1
DAPK1.1	3.37E-10	0.364604726	0.462	0.577	8.13E-06	ductal	DAPK1
ADAM9.1	4.98E-10	0.266710572	0.579	0.563	1.20E-05	ductal	ADAM9
