# Supplementary material for: Ductal keratin 15+ luminal progenitors in normal breast exhibit a basal-like breast cancer transcriptomic signature
Source: NPJ Breast Cancer. 2022 Jul 12;8:81. doi: 10.1038/s41523-022-00444-8 (PMC9276673; doi:10.1038/s41523-022-00444-8)
Supplement: Supplementary file 6 — Dataset 4 [file 41523_2022_444_MOESM6_ESM.pdf]

Supplementary Data 4.

DEGs among clusters 1.1, 1.2, 1.3 and 1.4.
A cutoff of 0.1 for the log2FC and 1% for the relative number of cells expressing the gene in the given cluster were applied to obtain the list of DEGs.

Abbreviations:	p_val = p-value
	avg_log2FC = average log2(fold change)
	pct.1 = percentage of cells in which the gene is detected in a given cluster
	pct.2 = percentage of cell in which the gene is detected in the rest of cells
	p_val_adj = adjusted p-value

gene	p_val	avg_log2FC	pct.1	pct.2	p_val_adj	cluster
OSBPL9	0	1.506227788	0.712	0.373	0	1.1
PSME4	0	1.314285145	0.846	0.648	0	1.1
EIF2AK3	0	1.811317759	0.603	0.191	0	1.1
LRRFIP2	0	1.470910615	0.94	0.769	0	1.1
TSC22D2	0	1.653394461	0.614	0.219	0	1.1
RARRES1	0	-2.025698144	0.073	0.518	0	1.1
TRIO	0	1.448304337	0.905	0.686	0	1.1
SOD2	0	-1.259023531	0.837	0.984	0	1.1
SAT1	0	-1.494038621	0.924	0.976	0	1.1
FTX	0	-1.586062931	0.159	0.681	0	1.1
HSPA5	0	2.109216515	0.703	0.285	0	1.1
SAA2-SAA4	0	-1.618097305	0.044	0.495	0	1.1
SAA1	0	-3.450793334	0.88	0.981	0	1.1
CD59	0	-1.680100701	0.619	0.953	0	1.1
NEAT1	0	-0.957630284	0.89	0.991	0	1.1
FRMD4A	0	-1.992699665	0.122	0.606	0	1.1
HNRNPA1	0	0.954124183	0.956	0.872	0	1.1
UBC	0	1.982467159	0.988	0.936	0	1.1
RP11-519G	0	-1.853625347	0.137	0.629	0	1.1
DTNA	0	1.459515075	0.882	0.664	0	1.1
OSER1	0	2.087743858	0.857	0.439	0	1.1
MLLT4	6.42E-308	1.259209192	0.845	0.597	1.55E-303	1.1
CYR61	5.97E-307	2.290999488	0.546	0.191	1.44E-302	1.1
SLPI	2.60E-299	-2.423934879	0.636	0.878	6.27E-295	1.1
EVA1C	2.67E-284	-1.539105662	0.133	0.563	6.44E-280	1.1
N4BP2L2	3.86E-280	-1.057833391	0.448	0.823	9.30E-276	1.1
S100A11	1.00E-278	-1.358509701	0.424	0.807	2.42E-274	1.1
DEFB1	1.82E-278	-1.824381852	0.302	0.704	4.40E-274	1.1
TXNIP	5.82E-277	-1.561210396	0.152	0.576	1.40E-272	1.1
LTF	2.27E-274	-1.665019812	0.393	0.764	5.48E-270	1.1
OVOS2	2.99E-270	-2.495592344	0.219	0.602	7.20E-266	1.1
FOXP1	3.42E-268	-1.304143375	0.244	0.661	8.25E-264	1.1
C15orf48	1.21E-265	-2.513414065	0.2	0.59	2.93E-261	1.1
KRT15	6.81E-264	1.433175153	0.601	0.248	1.64E-259	1.1
KYNU	1.03E-259	-2.298366339	0.091	0.484	2.48E-255	1.1
MTHFD2L	1.25E-257	-1.362973863	0.333	0.717	3.01E-253	1.1
WFDC2	6.78E-253	-1.989638039	0.191	0.61	1.64E-248	1.1
MGST1	7.82E-250	-1.095468082	0.794	0.916	1.89E-245	1.1
RNF145	4.14E-249	1.285247173	0.744	0.498	9.98E-245	1.1
DPYD	6.14E-240	-1.276351146	0.231	0.64	1.48E-235	1.1
RPS4X	4.67E-239	0.517097604	0.994	0.98	1.13E-234	1.1
HSP90B1	1.08E-236	1.233899888	0.684	0.39	2.61E-232	1.1
CYP7B1	1.08E-235	-1.614077921	0.093	0.477	2.60E-231	1.1
LUCAT1	1.81E-235	-1.462967965	0.116	0.501	4.37E-231	1.1
LYN	7.75E-235	-1.033594927	0.408	0.789	1.87E-230	1.1
OSMR	1.00E-233	-1.185668188	0.09	0.476	2.42E-229	1.1

RASA2	6.91E-232	1.214856484	0.792	0.573	1.67E-227	1.1
CCL2	2.11E-225	-3.505392221	0.173	0.525	5.09E-221	1.1
FLNB	1.08E-215	1.167282833	0.596	0.301	2.60E-211	1.1
TNFAIP6	1.09E-210	-2.013162728	0.149	0.516	2.62E-206	1.1
FBLN5	1.50E-210	-1.766184265	0.08	0.427	3.62E-206	1.1
ST5	6.88E-205	-1.252735877	0.128	0.486	1.66E-200	1.1
PSTPIP2	4.50E-204	-1.262867877	0.1	0.45	1.08E-199	1.1
WAC	3.64E-203	0.999582807	0.769	0.577	8.79E-199	1.1
SLC34A2	1.00E-202	-1.179437098	0.043	0.373	2.42E-198	1.1
TBC1D5	3.72E-200	-1.136453626	0.101	0.447	8.98E-196	1.1
ANKRD36C	6.64E-199	-1.409189278	0.312	0.649	1.60E-194	1.1
PPP2R3A	3.93E-193	-1.059524102	0.148	0.504	9.49E-189	1.1
LHFPL2	2.87E-191	1.153741689	0.597	0.33	6.92E-187	1.1
AUH	1.67E-188	1.182383151	0.536	0.267	4.02E-184	1.1
RAD23B	2.55E-188	1.090957159	0.611	0.351	6.15E-184	1.1
ARHGAP26	1.09E-185	-1.053953436	0.691	0.886	2.64E-181	1.1
VNN3	3.22E-185	-1.400132274	0.044	0.353	7.77E-181	1.1
ZBTB20	6.34E-185	-1.058972666	0.223	0.575	1.53E-180	1.1
FGFR1	3.19E-182	1.145160623	0.345	0.103	7.69E-178	1.1
SORBS1	3.26E-181	-1.10976181	0.103	0.431	7.86E-177	1.1
CCND3	5.36E-180	-1.13748311	0.113	0.446	1.29E-175	1.1
SPIDR	1.19E-177	-0.982674003	0.343	0.681	2.86E-173	1.1
EEF1A1	1.57E-177	0.439450107	0.993	0.98	3.79E-173	1.1
PI3	2.29E-177	-2.852196142	0.072	0.377	5.53E-173	1.1
RLF	4.44E-177	1.207979504	0.538	0.281	1.07E-172	1.1
AGAP1	1.42E-175	-1.084214837	0.386	0.702	3.43E-171	1.1
ENAH	5.56E-175	1.13468292	0.655	0.427	1.34E-170	1.1
NPAS2	7.20E-175	-1.025689849	0.23	0.577	1.74E-170	1.1
ALPK1	8.91E-174	-1.033233947	0.073	0.381	2.15E-169	1.1
VNN1	8.12E-173	-1.133844156	0.041	0.337	1.96E-168	1.1
MAST4	3.00E-171	-1.225781677	0.146	0.468	7.24E-167	1.1
COBL	4.76E-171	1.064650669	0.672	0.434	1.15E-166	1.1
MGAM2	9.95E-171	-1.30601262	0.108	0.421	2.40E-166	1.1
INADL	1.79E-169	-0.741090598	0.591	0.874	4.31E-165	1.1
TNFAIP2	4.77E-168	-1.36356761	0.075	0.378	1.15E-163	1.1
ABI1	2.13E-167	0.874450884	0.831	0.691	5.13E-163	1.1
FDPS	2.33E-166	-1.235649207	0.24	0.542	5.62E-162	1.1
TMTC2	2.12E-164	-1.038179898	0.087	0.392	5.12E-160	1.1
C3	4.52E-164	-0.94091377	0.081	0.384	1.09E-159	1.1
SLFN5	1.64E-162	-0.779911725	0.1	0.416	3.95E-158	1.1
RPL30	1.76E-162	0.446785432	0.99	0.976	4.24E-158	1.1
5-Mar	2.59E-162	1.004396042	0.417	0.167	6.25E-158	1.1
RPS6	1.06E-161	0.402635978	0.994	0.981	2.55E-157	1.1
TM4SF1	8.17E-161	1.199608063	0.936	0.884	1.97E-156	1.1
USP53	1.16E-160	1.064815132	0.798	0.622	2.80E-156	1.1
MYO5B	7.71E-159	1.162086285	0.729	0.551	1.86E-154	1.1
PTPN2	1.34E-157	-0.940716272	0.147	0.455	3.22E-153	1.1

SOS1	1.33E-154	-0.964444956	0.155	0.465	3.21E-150	1.1
DAAM1	1.59E-153	1.029126956	0.672	0.459	3.83E-149	1.1
SLC28A3	1.47E-152	-0.874549268	0.128	0.444	3.55E-148	1.1
CRY1	1.83E-152	1.117955983	0.523	0.279	4.42E-148	1.1
SLC25A37	3.44E-152	-0.777678652	0.474	0.787	8.29E-148	1.1
USP39	9.73E-151	-0.819648329	0.128	0.433	2.35E-146	1.1
HILPDA	1.68E-150	1.276193529	0.428	0.182	4.04E-146	1.1
ATP5G2	1.74E-150	0.824781499	0.835	0.767	4.20E-146	1.1
RAPGEF5	2.94E-150	-0.906107631	0.243	0.568	7.09E-146	1.1
SRRM1	6.58E-150	0.808664412	0.766	0.62	1.59E-145	1.1
TMEM165	1.81E-149	-0.857606783	0.24	0.562	4.35E-145	1.1
ARHGEF28	2.25E-149	1.283188262	0.551	0.324	5.42E-145	1.1
ROPN1B	2.30E-149	0.803415155	0.281	0.079	5.55E-145	1.1
DTNB	6.40E-149	-0.853039325	0.348	0.662	1.54E-144	1.1
GOLGA4	6.73E-149	0.841160315	0.764	0.609	1.62E-144	1.1
EFNA5	1.79E-148	-0.897392137	0.253	0.585	4.31E-144	1.1
AKR1C3	2.45E-148	-0.907421189	0.012	0.256	5.90E-144	1.1
SLC12A2	7.98E-148	-1.127826054	0.318	0.619	1.92E-143	1.1
DOCK4	9.34E-147	-1.141266626	0.142	0.435	2.25E-142	1.1
DYNLT1	1.11E-146	-0.822182275	0.401	0.695	2.68E-142	1.1
FAM177B	1.64E-146	-1.06811054	0.405	0.727	3.95E-142	1.1
ANK3	2.66E-146	-1.000381457	0.128	0.421	6.42E-142	1.1
MYO6	1.50E-142	0.977688696	0.748	0.61	3.62E-138	1.1
RBM47	3.79E-142	-0.821303352	0.365	0.671	9.13E-138	1.1
PRDX1	5.29E-142	-1.107720437	0.576	0.769	1.28E-137	1.1
DHFR	6.69E-142	0.664141048	0.914	0.825	1.61E-137	1.1
NPM1	1.12E-141	0.58252759	0.938	0.902	2.69E-137	1.1
RPL11	1.52E-141	0.461562281	0.991	0.977	3.67E-137	1.1
ADAM9	3.55E-140	0.992108684	0.705	0.533	8.57E-136	1.1
EIF4G3	7.94E-139	-0.885789472	0.115	0.399	1.91E-134	1.1
CHODL	9.18E-139	-1.086180951	0.025	0.265	2.21E-134	1.1
CADPS2	1.10E-138	-0.867522518	0.103	0.386	2.66E-134	1.1
RIN2	1.00E-137	-0.897607657	0.106	0.385	2.41E-133	1.1
PROM1	2.05E-137	-0.847470313	0.232	0.538	4.95E-133	1.1
TLR2	3.16E-137	-0.82848565	0.063	0.327	7.63E-133	1.1
PLEKHA5	4.95E-137	-0.827259275	0.198	0.501	1.19E-132	1.1
EGFR	6.59E-137	-0.943605303	0.126	0.411	1.59E-132	1.1
PPM1H	1.62E-136	-0.908113351	0.165	0.457	3.90E-132	1.1
DDX21	2.44E-136	0.809480812	0.742	0.584	5.89E-132	1.1
RUNX1	3.13E-135	-0.87895928	0.341	0.644	7.55E-131	1.1
CTSB	5.44E-135	-0.991996462	0.107	0.375	1.31E-130	1.1
CPEB3	6.27E-135	1.060290516	0.499	0.275	1.51E-130	1.1
RPS24	7.90E-135	0.40151675	0.993	0.979	1.90E-130	1.1
AFF1	1.45E-134	-0.813385511	0.095	0.37	3.50E-130	1.1
HSP90AB1	8.16E-134	0.512561813	0.96	0.928	1.97E-129	1.1
ST6GAL1	1.27E-133	-0.859178455	0.127	0.413	3.05E-129	1.1
GPBP1	3.30E-133	0.834527393	0.725	0.578	7.95E-129	1.1

QKI	3.53E-132	1.008684013	0.655	0.493	8.51E-128	1.1
HS3ST4	9.84E-132	-1.030108857	0.142	0.419	2.37E-127	1.1
PLSCR1	3.79E-131	-0.761165259	0.093	0.361	9.15E-127	1.1
MT-ND3	7.13E-131	-0.496583725	0.979	0.986	1.72E-126	1.1
THRB	3.26E-130	-0.823376687	0.095	0.37	7.85E-126	1.1
NAA25	5.07E-130	0.980382404	0.484	0.259	1.22E-125	1.1
L3MBTL4	1.35E-129	-0.849596997	0.133	0.414	3.26E-125	1.1
LINC-PINT	2.42E-129	-0.957508312	0.332	0.622	5.84E-125	1.1
OSMR-AS1	1.44E-128	-0.790650484	0.028	0.258	3.48E-124	1.1
THUMPD3-	2.17E-128	-0.77658634	0.109	0.376	5.23E-124	1.1
FAM13A	5.36E-128	1.259525704	0.524	0.324	1.29E-123	1.1
NEDD4L	1.71E-127	0.858905197	0.692	0.495	4.13E-123	1.1
PLCB1	2.34E-127	-0.980320976	0.19	0.473	5.65E-123	1.1
NACA	3.94E-127	0.464960263	0.979	0.957	9.49E-123	1.1
RICTOR	1.02E-126	0.897930234	0.66	0.477	2.45E-122	1.1
IFRD1	1.03E-126	0.935250454	0.542	0.321	2.47E-122	1.1
MKL1	2.60E-126	1.054886782	0.623	0.439	6.27E-122	1.1
KMT2C	1.98E-125	0.906946211	0.702	0.582	4.76E-121	1.1
CORO1C	2.76E-125	0.847332356	0.398	0.178	6.65E-121	1.1
RPL31	5.10E-125	0.454365655	0.983	0.958	1.23E-120	1.1
SLC11A2	6.36E-124	-0.75725814	0.217	0.506	1.53E-119	1.1
RPL32	7.26E-124	0.464269742	0.995	0.981	1.75E-119	1.1
SIK2	1.21E-123	-0.79163914	0.128	0.398	2.91E-119	1.1
SERPINB7	2.32E-123	-1.208284879	0.033	0.255	5.60E-119	1.1
UBE2H	9.16E-123	0.730249119	0.796	0.688	2.21E-118	1.1
GAB1	2.05E-122	-0.947414557	0.165	0.433	4.95E-118	1.1
ZFAS1	2.22E-122	0.432023602	0.979	0.956	5.36E-118	1.1
ITPR2	1.93E-121	-0.943525369	0.353	0.613	4.66E-117	1.1
MT-ATP6	3.39E-121	-0.530372613	0.982	0.989	8.18E-117	1.1
3-Mar	3.49E-121	-1.064914664	0.042	0.27	8.41E-117	1.1
RERE	8.92E-121	-0.717397056	0.221	0.506	2.15E-116	1.1
PPP2CB	2.19E-120	0.875770581	0.464	0.252	5.27E-116	1.1
CHI3L1	3.84E-120	-1.672470406	0.038	0.26	9.25E-116	1.1
RAD21	4.93E-120	0.876741423	0.512	0.305	1.19E-115	1.1
RPS23	1.38E-119	0.453765113	0.994	0.98	3.33E-115	1.1
PLA2R1	2.19E-119	-0.837610253	0.063	0.302	5.28E-115	1.1
CHI3L2	3.77E-119	-1.21347955	0.314	0.566	9.08E-115	1.1
ZMYND8	4.04E-119	-0.838147793	0.08	0.325	9.74E-115	1.1
CD44	4.70E-119	-0.738817131	0.263	0.56	1.13E-114	1.1
MAP3K5	7.00E-119	-0.868374531	0.13	0.391	1.69E-114	1.1
TRPS1	3.35E-118	-0.908744228	0.569	0.772	8.08E-114	1.1
PDIA6	4.05E-118	0.892173727	0.398	0.191	9.76E-114	1.1
KIAA0922	4.45E-118	1.288350738	0.409	0.208	1.07E-113	1.1
THADA	1.26E-116	-1.173831786	0.095	0.337	3.04E-112	1.1
RIPK2	1.47E-116	0.950865946	0.55	0.342	3.54E-112	1.1
ATF3	2.32E-116	1.063347876	0.493	0.29	5.60E-112	1.1
CXCR4	2.59E-116	1.312490662	0.449	0.245	6.24E-112	1.1

SELK	3.73E-116	0.771209167	0.767	0.642	9.00E-112	1.1
RSRC2	5.65E-116	0.825126551	0.708	0.566	1.36E-111	1.1
RPS15A	1.33E-115	0.421946799	0.984	0.965	3.20E-111	1.1
PIK3R1	2.65E-115	-0.961439705	0.09	0.331	6.40E-111	1.1
ITFG1	1.34E-114	-0.782091869	0.073	0.307	3.24E-110	1.1
RPL35A	2.55E-114	0.446906005	0.992	0.977	6.16E-110	1.1
GBP1	1.19E-113	0.993311621	0.336	0.139	2.86E-109	1.1
TOMM7	2.35E-113	0.535320675	0.929	0.899	5.68E-109	1.1
BPTF	2.07E-112	-0.710813414	0.146	0.403	5.00E-108	1.1
MXD1	2.18E-112	0.807093987	0.298	0.11	5.27E-108	1.1
EXOC4	2.22E-112	-0.772199366	0.113	0.359	5.35E-108	1.1
AUTS2	3.58E-112	-0.742523789	0.367	0.634	8.64E-108	1.1
RFWD2	6.94E-112	-0.696720725	0.109	0.36	1.67E-107	1.1
NAV2	7.27E-112	1.025821786	0.577	0.386	1.75E-107	1.1
RP5-1198O	1.22E-111	-0.824178284	0.015	0.21	2.94E-107	1.1
ANKS1B	1.78E-111	-0.819722769	0.093	0.336	4.30E-107	1.1
LINC00152	8.04E-111	0.98549599	0.49	0.291	1.94E-106	1.1
TAF1D	9.36E-111	0.823815698	0.478	0.267	2.26E-106	1.1
FAAH2	1.51E-110	-0.757513041	0.051	0.269	3.64E-106	1.1
CFLAR	3.13E-110	-0.774568689	0.266	0.543	7.56E-106	1.1
FMN1	8.44E-110	-0.739426533	0.022	0.221	2.04E-105	1.1
HNRNPC	1.33E-109	0.628470432	0.866	0.784	3.22E-105	1.1
PDE4B	2.46E-109	0.729710029	0.931	0.906	5.92E-105	1.1
DOCK1	2.57E-109	-0.723788123	0.117	0.367	6.20E-105	1.1
CRYAB	6.82E-109	0.987728477	0.647	0.465	1.64E-104	1.1
ARHGAP29	1.12E-108	1.26114021	0.605	0.439	2.71E-104	1.1
RPL14	1.98E-108	0.460041985	0.99	0.978	4.78E-104	1.1
RPL34	2.88E-108	0.335956714	0.996	0.98	6.94E-104	1.1
PLEKHA7	6.72E-108	0.317438083	0.995	0.981	1.62E-103	1.1
CYP24A1	5.71E-107	-1.226259883	0.07	0.287	1.38E-102	1.1
LRBA	6.59E-107	-0.695836197	0.256	0.536	1.59E-102	1.1
DDX5	2.08E-106	-0.650774889	0.3	0.58	5.03E-102	1.1
MYO1B	4.55E-106	-0.797188338	0.149	0.398	1.10E-101	1.1
PHLPP1	5.93E-106	0.938355177	0.586	0.414	1.43E-101	1.1
TNFAIP8	7.16E-106	-0.75191355	0.202	0.475	1.73E-101	1.1
TACC1	1.94E-105	-0.762177682	0.071	0.295	4.68E-101	1.1
DDIT3	3.32E-105	0.875704761	0.246	0.082	8.01E-101	1.1
C1QTNF3-A	4.46E-105	-0.69634858	0.056	0.275	1.07E-100	1.1
DCHS2	6.29E-105	-0.857517908	0.013	0.198	1.52E-100	1.1
ROPN1	6.80E-105	0.519828076	0.16	0.033	1.64E-100	1.1
RPL22	7.56E-105	0.580397737	0.843	0.777	1.82E-100	1.1
SLC26A2	3.68E-104	-0.843681469	0.021	0.211	8.88E-100	1.1
ATP1A1	5.90E-104	-0.662155645	0.312	0.583	1.42E-99	1.1
HSD11B1	7.38E-104	-0.849466733	0.047	0.251	1.78E-99	1.1
RBM3	9.53E-104	0.719155965	0.627	0.459	2.30E-99	1.1
CDC42BPA	4.62E-103	-0.708562797	0.114	0.348	1.11E-98	1.1
TNFSF10	7.84E-103	-0.816149336	0.31	0.558	1.89E-98	1.1

CCL20	2.86E-102	-1.634191418	0.133	0.364	6.89E-98	1.1
LINC01184	3.70E-102	-0.702019261	0.063	0.276	8.91E-98	1.1
PLEKHS1	5.89E-102	-0.768709833	0.093	0.322	1.42E-97	1.1
FNBP1	9.29E-102	0.895672707	0.606	0.444	2.24E-97	1.1
BICD1	1.10E-101	-0.745049149	0.045	0.248	2.66E-97	1.1
NUP153	1.83E-101	0.824229624	0.414	0.218	4.41E-97	1.1
MEAF6	5.98E-101	0.68354858	0.273	0.101	1.44E-96	1.1
CCDC146	8.07E-101	-0.7110387	0.04	0.239	1.95E-96	1.1
DHCR24	3.28E-100	-0.595305593	0.029	0.219	7.91E-96	1.1
EIF3E	7.62E-100	0.566149462	0.842	0.783	1.84E-95	1.1
PDZK1IP1	9.00E-100	-0.894865482	0.032	0.222	2.17E-95	1.1
PLPP3	2.59E-99	-0.796070947	0.07	0.283	6.25E-95	1.1
PPP2R2A	2.82E-99	0.778298977	0.546	0.361	6.81E-95	1.1
RPS3A	4.78E-99	0.394674936	0.972	0.944	1.15E-94	1.1
PNISR	1.08E-98	-0.564176508	0.607	0.822	2.61E-94	1.1
PTPRK	2.54E-98	-0.558345569	0.536	0.782	6.13E-94	1.1
ARID1B	2.80E-98	-0.678948978	0.221	0.475	6.75E-94	1.1
KCMF1	4.86E-98	0.857679561	0.441	0.253	1.17E-93	1.1
SSBP2	5.20E-98	1.04573241	0.481	0.296	1.25E-93	1.1
SKAP2	7.58E-98	-0.665937949	0.148	0.387	1.83E-93	1.1
RSL1D1	7.96E-98	0.696216634	0.705	0.603	1.92E-93	1.1
STAG1	1.22E-97	-0.628262313	0.282	0.553	2.95E-93	1.1
CA8	1.24E-97	-0.711410843	0.249	0.508	2.99E-93	1.1
SGPP2	1.43E-97	-0.690375745	0.047	0.244	3.44E-93	1.1
NPEPPS	1.51E-97	0.916686828	0.576	0.408	3.65E-93	1.1
MBD5	6.36E-97	-0.668715778	0.101	0.326	1.53E-92	1.1
S100A8	1.33E-96	-3.030387863	0.071	0.269	3.20E-92	1.1
SRSF5	1.64E-96	-0.574557711	0.141	0.384	3.95E-92	1.1
IFITM3	2.78E-96	-0.82401387	0.098	0.311	6.71E-92	1.1
DENND5A	8.51E-96	0.808957681	0.494	0.305	2.05E-91	1.1
HK2	2.26E-95	0.771382722	0.298	0.126	5.46E-91	1.1
EHBP1	1.13E-94	-0.746195802	0.17	0.406	2.72E-90	1.1
PLXDC2	1.34E-94	-0.711525422	0.083	0.295	3.24E-90	1.1
BACH2	1.78E-94	0.937482119	0.549	0.363	4.30E-90	1.1
C4orf19	2.14E-94	-0.694094252	0.047	0.239	5.15E-90	1.1
CNTN4	2.82E-93	-0.903979944	0.054	0.248	6.79E-89	1.1
LRP6	2.82E-93	0.837746182	0.428	0.242	6.81E-89	1.1
SAMD12	4.71E-93	-0.712740174	0.1	0.321	1.14E-88	1.1
B2M	6.38E-93	-0.521847647	0.995	0.982	1.54E-88	1.1
NOCT	2.03E-92	0.707585676	0.255	0.096	4.90E-88	1.1
LRP1B	2.73E-92	-1.448546833	0.062	0.257	6.58E-88	1.1
ZNF518A	2.90E-92	-0.631833301	0.047	0.237	6.99E-88	1.1
BTF3	3.79E-92	0.548261539	0.957	0.942	9.13E-88	1.1
FTH1	1.74E-91	-0.686513192	0.802	0.901	4.20E-87	1.1
RCAN1	2.48E-91	-0.787068105	0.51	0.718	5.99E-87	1.1
CTD-2015G	3.66E-91	-0.604957466	0.043	0.23	8.83E-87	1.1
C11orf80	7.15E-91	-0.62514377	0.07	0.272	1.72E-86	1.1

RALGAPA2	9.90E-91	-0.580911505	0.05	0.241	2.39E-86	1.1
ADAMTS9-/	1.15E-90	-0.691916978	0.152	0.381	2.77E-86	1.1
NFIL3	1.32E-90	0.68110606	0.275	0.113	3.19E-86	1.1
TPM1	2.61E-90	-0.686809593	0.447	0.662	6.29E-86	1.1
SIPA1L1	6.36E-90	-0.63430268	0.18	0.42	1.53E-85	1.1
SVIL	6.99E-90	-0.568175667	0.72	0.876	1.69E-85	1.1
FNBP1L	8.23E-90	0.8963115	0.55	0.393	1.98E-85	1.1
SDCBP	1.03E-89	-0.730197836	0.562	0.771	2.48E-85	1.1
TRIM56	1.26E-89	-0.62093413	0.06	0.255	3.03E-85	1.1
SERAC1	4.35E-89	0.581567638	0.172	0.046	1.05E-84	1.1
SRPK1	7.01E-89	-0.610639869	0.403	0.646	1.69E-84	1.1
ILF2	7.87E-89	0.691967661	0.664	0.525	1.90E-84	1.1
ST8SIA1	1.18E-88	-0.649721519	0.046	0.23	2.85E-84	1.1
VMP1	3.55E-88	-0.58545672	0.644	0.828	8.55E-84	1.1
HELZ	4.97E-88	0.757719354	0.457	0.278	1.20E-83	1.1
SLC9A7	1.31E-87	0.813467876	0.424	0.242	3.16E-83	1.1
RSRP1	1.48E-87	-0.588548818	0.094	0.306	3.57E-83	1.1
PDLIM5	2.48E-87	0.574440658	0.876	0.825	5.98E-83	1.1
RIOK3	2.72E-87	0.781327572	0.428	0.251	6.56E-83	1.1
HSPA8	4.37E-87	-0.808213549	0.401	0.608	1.05E-82	1.1
SPDYE2	4.79E-87	-0.535792555	0.053	0.239	1.15E-82	1.1
VPS13D	7.44E-87	-0.609766822	0.155	0.387	1.79E-82	1.1
AKR1C1	8.25E-87	-0.931526349	0.01	0.164	1.99E-82	1.1
METTL17	5.06E-86	0.49042923	0.148	0.035	1.22E-81	1.1
TSHZ2	5.17E-86	-0.722811123	0.288	0.529	1.25E-81	1.1
NFE2L2	1.75E-85	-0.626271234	0.188	0.417	4.21E-81	1.1
ANO10	9.98E-85	-0.579462753	0.086	0.289	2.41E-80	1.1
BTAF1	1.02E-84	0.745045412	0.376	0.2	2.47E-80	1.1
THSD4	1.94E-84	-0.724544153	0.242	0.479	4.68E-80	1.1
BBX	2.52E-84	-0.580476971	0.217	0.455	6.09E-80	1.1
CMPK1	3.25E-84	-0.599433396	0.152	0.379	7.84E-80	1.1
RPL5	3.86E-84	0.382343448	0.986	0.97	9.30E-80	1.1
ATG7	6.30E-84	-0.573538756	0.078	0.274	1.52E-79	1.1
PFDN5	7.14E-84	0.453689755	0.912	0.892	1.72E-79	1.1
SORBS2	7.31E-84	-0.738204496	0.41	0.639	1.76E-79	1.1
RNF213	1.42E-83	-0.555919858	0.049	0.229	3.43E-79	1.1
ZNF521	1.74E-83	-0.652496042	0.053	0.232	4.20E-79	1.1
PTPRG	1.98E-83	-0.704278451	0.084	0.281	4.77E-79	1.1
SPG11	2.46E-83	-0.581395106	0.126	0.343	5.93E-79	1.1
MAP4K4	2.53E-83	-0.632010616	0.183	0.414	6.10E-79	1.1
SLC39A8	2.60E-83	-0.722458218	0.029	0.193	6.28E-79	1.1
ENOSF1	3.01E-83	-0.610505285	0.08	0.272	7.26E-79	1.1
RASGRP1	3.08E-83	-0.566627924	0.021	0.181	7.42E-79	1.1
APOO	2.09E-82	0.371372107	0.991	0.978	5.03E-78	1.1
DENND2D	5.79E-82	-0.490589432	0.02	0.176	1.40E-77	1.1
YTHDC1	6.72E-82	0.704195846	0.411	0.236	1.62E-77	1.1
WEE1	1.09E-81	0.686715374	0.397	0.218	2.63E-77	1.1

LTBP1	1.09E-81	-0.721567221	0.084	0.276	2.63E-77	1.1
KDM5B	1.39E-81	0.729782045	0.635	0.531	3.36E-77	1.1
RPSA	1.69E-81	0.533489316	0.932	0.917	4.07E-77	1.1
NEDD9	3.14E-81	0.821004963	0.395	0.226	7.57E-77	1.1
FBXL17	3.71E-81	-0.641494172	0.055	0.234	8.94E-77	1.1
CFB	1.46E-80	-0.557027962	0.021	0.177	3.53E-76	1.1
GRHL2	1.47E-80	-0.586474118	0.123	0.33	3.56E-76	1.1
FDFT1	3.84E-80	-0.702700938	0.182	0.395	9.27E-76	1.1
MAP3K13	4.30E-80	-0.4644371	0.7	0.876	1.04E-75	1.1
ZBTB38	7.53E-80	-0.621288407	0.163	0.377	1.81E-75	1.1
ELL2	8.94E-80	0.684159157	0.757	0.654	2.16E-75	1.1
ESYT2	1.32E-79	0.78017528	0.614	0.474	3.19E-75	1.1
ZNF638	1.79E-79	-0.564233143	0.252	0.485	4.32E-75	1.1
NFAT5	4.38E-79	0.640371016	0.705	0.598	1.06E-74	1.1
DIAPH2	8.37E-79	-0.646367311	0.124	0.328	2.02E-74	1.1
ATF7IP	1.05E-78	-0.556677521	0.048	0.22	2.52E-74	1.1
ARHGAP17	1.30E-78	0.76559444	0.338	0.175	3.14E-74	1.1
PARP4	1.33E-78	-0.547581188	0.064	0.243	3.21E-74	1.1
CXCL1	1.39E-78	-0.809398936	0.108	0.303	3.35E-74	1.1
HGSNAT	1.78E-78	-0.555595973	0.045	0.212	4.30E-74	1.1
SEC24D	1.94E-78	0.813029175	0.423	0.255	4.68E-74	1.1
BTBD9	2.15E-78	-0.623467532	0.066	0.245	5.19E-74	1.1
CXCL8	5.44E-78	-2.250741358	0.212	0.41	1.31E-73	1.1
ARHGAP44	5.85E-78	-0.556100822	0.097	0.295	1.41E-73	1.1
ST6GALNA(	6.25E-78	-0.739366174	0.224	0.447	1.51E-73	1.1
GPRC5A	7.47E-78	1.119328789	0.46	0.307	1.80E-73	1.1
STRN	8.86E-78	0.759125192	0.513	0.356	2.14E-73	1.1
CXCL17	8.88E-78	-0.864524842	0.058	0.229	2.14E-73	1.1
CHPT1	2.51E-77	-0.9041861	0.235	0.442	6.05E-73	1.1
RPS27A	2.54E-77	0.315548306	0.994	0.982	6.13E-73	1.1
PDZRN3	2.62E-77	-0.656951403	0.059	0.232	6.33E-73	1.1
SEMA6A	2.73E-77	-0.685973544	0.245	0.475	6.59E-73	1.1
HIVEP3	3.18E-77	-0.762149781	0.163	0.375	7.68E-73	1.1
SHROOM3	3.51E-77	0.76446001	0.619	0.488	8.47E-73	1.1
GABPB1-AS	3.98E-77	-0.499955238	0.069	0.251	9.60E-73	1.1
SEMA6A-A!	5.13E-77	-0.62753119	0.106	0.306	1.24E-72	1.1
NR2F2-AS1	5.40E-77	-0.588025374	0.028	0.182	1.30E-72	1.1
RTN4	1.45E-76	0.630271395	0.711	0.597	3.50E-72	1.1
CRIM1	1.95E-76	0.732195333	0.611	0.464	4.71E-72	1.1
RPS27L	1.45E-75	-0.662139179	0.374	0.585	3.50E-71	1.1
ANKIB1	1.73E-75	-0.52085071	0.163	0.375	4.17E-71	1.1
LDHB	3.12E-75	0.563556878	0.788	0.736	7.52E-71	1.1
RPL4	3.70E-75	0.428459616	0.967	0.954	8.93E-71	1.1
NFIB	4.02E-75	-0.474019899	0.755	0.886	9.70E-71	1.1
STIM1	6.86E-75	-0.560361488	0.076	0.255	1.65E-70	1.1
TCF12	1.46E-74	-0.502018425	0.324	0.56	3.53E-70	1.1
ARL15	2.25E-74	-0.55998098	0.076	0.257	5.41E-70	1.1

TRABD2B	6.49E-74	-0.632328855	0.007	0.14	1.57E-69	1.1
USP54	1.01E-73	0.872483269	0.607	0.48	2.43E-69	1.1
RPL7A	1.46E-73	0.405728136	0.986	0.972	3.52E-69	1.1
TXNRD1	1.47E-73	-0.80633819	0.246	0.463	3.54E-69	1.1
IRF2BPL	1.94E-73	-0.470899948	0.022	0.167	4.68E-69	1.1
PRKD1	2.26E-73	-0.613624907	0.045	0.205	5.44E-69	1.1
IFI16	4.11E-73	-0.54572801	0.13	0.328	9.90E-69	1.1
XKR6	4.48E-73	-0.568183239	0.053	0.217	1.08E-68	1.1
OTUD7B	1.31E-72	0.653672522	0.279	0.129	3.16E-68	1.1
ZNF33A	1.42E-72	-0.508277744	0.109	0.3	3.42E-68	1.1
RPL21	1.59E-72	0.34032329	0.976	0.952	3.84E-68	1.1
UGGT2	2.24E-72	-0.584878959	0.052	0.215	5.40E-68	1.1
JMJD1C	2.94E-72	0.652393098	0.736	0.658	7.10E-68	1.1
MAPK14	3.66E-72	-0.49885966	0.056	0.222	8.84E-68	1.1
LDHA	4.88E-72	0.820185197	0.761	0.704	1.18E-67	1.1
TRAPPC9	6.87E-72	-0.559478184	0.056	0.222	1.66E-67	1.1
KLHL5	1.31E-71	-0.514939986	0.056	0.221	3.17E-67	1.1
PLEKHA6	4.86E-71	0.37610727	0.942	0.917	1.17E-66	1.1
SRSF3	4.93E-71	0.553829016	0.703	0.562	1.19E-66	1.1
PRKCE	8.77E-71	-0.638222635	0.078	0.251	2.12E-66	1.1
ATR	1.92E-70	-0.492215798	0.05	0.211	4.63E-66	1.1
LAMC2	2.42E-70	-0.69248635	0.151	0.346	5.83E-66	1.1
KPNA1	4.92E-70	0.728124628	0.387	0.23	1.19E-65	1.1
TULP4	5.70E-70	0.734763635	0.59	0.462	1.37E-65	1.1
SLC47A1	1.10E-69	0.329557239	0.972	0.937	2.66E-65	1.1
PTPRJ	1.47E-69	0.728557716	0.438	0.28	3.54E-65	1.1
COA1	1.72E-69	-0.516804443	0.132	0.324	4.15E-65	1.1
ARID5B	1.82E-69	0.626688867	0.791	0.729	4.39E-65	1.1
CCSER1	1.95E-69	-0.493080944	0.357	0.588	4.69E-65	1.1
MAML3	2.54E-69	-0.614820484	0.108	0.29	6.12E-65	1.1
GCNT2	3.92E-69	-0.579859405	0.209	0.42	9.45E-65	1.1
MCPH1	7.96E-69	-0.485194244	0.052	0.21	1.92E-64	1.1
ATP1B1	9.13E-69	-0.926251089	0.598	0.752	2.20E-64	1.1
GLRX	9.28E-69	-0.506137996	0.33	0.559	2.24E-64	1.1
NSD1	9.72E-69	-0.473383042	0.038	0.187	2.34E-64	1.1
CDH1	1.10E-68	0.641853139	0.618	0.48	2.66E-64	1.1
EIF4A2	1.14E-68	0.460312962	0.862	0.796	2.75E-64	1.1
SIK3	1.61E-68	0.325678358	0.99	0.982	3.88E-64	1.1
DYRK1A	2.10E-68	0.732700786	0.468	0.319	5.05E-64	1.1
SLC30A4	2.67E-68	-0.489816844	0.026	0.167	6.43E-64	1.1
ZC3H12C	3.46E-68	-0.54168729	0.088	0.263	8.35E-64	1.1
GPATCH8	5.63E-68	-0.50601932	0.12	0.305	1.36E-63	1.1
SMYD3	1.29E-67	-0.65883353	0.102	0.277	3.11E-63	1.1
SMS	1.45E-67	0.871126034	0.493	0.353	3.50E-63	1.1
CAMK1D	2.82E-67	0.709375964	0.252	0.113	6.79E-63	1.1
EIF4E2	2.96E-67	-0.48650042	0.243	0.454	7.14E-63	1.1
GLIPR2	4.45E-67	-0.476093848	0.026	0.163	1.07E-62	1.1

ALOX5	4.93E-67	-0.48336964	0.03	0.17	1.19E-62	1.1
JARID2	6.81E-67	-0.617778183	0.121	0.303	1.64E-62	1.1
CDK13	8.24E-67	-0.502148234	0.197	0.404	1.99E-62	1.1
FAM49B	1.48E-66	-0.500783134	0.107	0.285	3.58E-62	1.1
CEPT1	2.01E-66	-0.501981513	0.144	0.337	4.86E-62	1.1
CX3CL1	2.57E-66	-0.664115325	0.13	0.315	6.19E-62	1.1
SLCO3A1	2.67E-66	-0.530410201	0.117	0.303	6.44E-62	1.1
PCBP1	3.25E-66	0.674794657	0.56	0.45	7.85E-62	1.1
NOS1AP	3.73E-66	-0.6362128	0.049	0.2	9.00E-62	1.1
NT5C2	4.08E-66	-0.487063214	0.219	0.431	9.84E-62	1.1
BRE	4.69E-66	-0.573324038	0.06	0.214	1.13E-61	1.1
ZFP36L2	5.87E-66	0.906669383	0.476	0.322	1.42E-61	1.1
BACH1	6.60E-66	-0.538560993	0.161	0.358	1.59E-61	1.1
RP11-123O	7.04E-66	-0.526745096	0.09	0.261	1.70E-61	1.1
C4orf3	7.76E-66	0.676367301	0.694	0.601	1.87E-61	1.1
THBS1	1.37E-65	0.595013303	0.151	0.046	3.30E-61	1.1
PRELID1	1.52E-65	-0.452273018	0.036	0.179	3.66E-61	1.1
CLEC7A	2.85E-65	-0.461236774	0.028	0.164	6.86E-61	1.1
JPX	3.20E-65	-0.548234349	0.129	0.306	7.70E-61	1.1
MORF4L2	3.22E-65	0.48211467	0.795	0.728	7.77E-61	1.1
GSAP	8.56E-65	-0.479002715	0.045	0.191	2.06E-60	1.1
CCDC91	8.59E-65	-0.462931647	0.203	0.411	2.07E-60	1.1
CCAR1	9.37E-65	-0.497603953	0.124	0.303	2.26E-60	1.1
PACS1	1.06E-64	-0.505009372	0.16	0.352	2.55E-60	1.1
PBX1	1.46E-64	-0.643872713	0.1	0.268	3.53E-60	1.1
NAPG	1.67E-64	-0.445504891	0.073	0.234	4.04E-60	1.1
CNN3	2.05E-64	0.726809516	0.557	0.428	4.95E-60	1.1
SRGAP2	3.27E-64	-0.430633488	0.022	0.151	7.89E-60	1.1
GLUL	3.67E-64	-0.626938031	0.182	0.376	8.84E-60	1.1
LINC00969	3.92E-64	-0.409915695	0.135	0.32	9.45E-60	1.1
CBR4	5.01E-64	-0.456205422	0.129	0.315	1.21E-59	1.1
ASS1	5.52E-64	-0.511251008	0.051	0.199	1.33E-59	1.1
MEF2A	6.75E-64	0.788694927	0.488	0.352	1.63E-59	1.1
NUTM2A-A	7.00E-64	-0.455160512	0.137	0.328	1.69E-59	1.1
HDAC8	8.02E-64	-0.477946592	0.099	0.273	1.93E-59	1.1
CCDC6	2.36E-63	0.717148056	0.449	0.302	5.68E-59	1.1
SERPINB4	7.09E-63	-1.33316587	0.01	0.127	1.71E-58	1.1
CUX1	1.80E-62	-0.484648738	0.103	0.273	4.33E-58	1.1
RANBP17	2.58E-62	-0.538738872	0.063	0.216	6.22E-58	1.1
SLC25A3	5.30E-62	-0.468272451	0.078	0.239	1.28E-57	1.1
S100B	5.49E-62	0.411926887	0.117	0.029	1.32E-57	1.1
BAIAP2L1	7.26E-62	0.563416759	0.978	0.963	1.75E-57	1.1
NDUFS5	8.54E-62	0.409626689	0.866	0.823	2.06E-57	1.1
CPD	9.80E-62	-0.529022759	0.098	0.265	2.36E-57	1.1
FKBP5	1.07E-61	-0.584171088	0.145	0.331	2.59E-57	1.1
LINC01138	1.15E-61	-0.47749882	0.105	0.276	2.78E-57	1.1
GNE	1.21E-61	-0.490983417	0.065	0.218	2.92E-57	1.1

ZKSCAN1	1.88E-61	-0.485624789	0.133	0.312	4.54E-57	1.1
RABGAP1L	3.65E-61	-0.524808061	0.103	0.272	8.81E-57	1.1
SSH2	4.19E-61	-0.543191962	0.197	0.389	1.01E-56	1.1
KIAA0319L	5.29E-61	-0.487945551	0.056	0.203	1.28E-56	1.1
MGST3	8.78E-61	-0.524485142	0.346	0.546	2.12E-56	1.1
HERC1	8.81E-61	0.749747391	0.487	0.359	2.12E-56	1.1
TPCN1	1.06E-60	-0.492568779	0.027	0.154	2.56E-56	1.1
DISC1	1.44E-60	-0.427236276	0.015	0.134	3.47E-56	1.1
EEF1B2	1.44E-60	0.539453161	0.897	0.878	3.48E-56	1.1
ST3GAL1	1.68E-60	-0.427583982	0.078	0.235	4.04E-56	1.1
UVRAG	1.75E-60	-0.461595465	0.261	0.473	4.21E-56	1.1
ADAMTS9	1.90E-60	-0.858766936	0.304	0.485	4.59E-56	1.1
S100A6	2.20E-60	-0.912806508	0.313	0.521	5.30E-56	1.1
CDR2	2.60E-60	-0.457240817	0.039	0.176	6.26E-56	1.1
LRRC49	9.78E-60	-0.480351703	0.03	0.158	2.36E-55	1.1
VTI1A	1.05E-59	-0.482674826	0.06	0.207	2.53E-55	1.1
ANKRD36	1.16E-59	-0.551211203	0.043	0.18	2.80E-55	1.1
RAPGEF2	1.33E-59	0.73888436	0.506	0.371	3.21E-55	1.1
PDIA3	1.48E-59	0.684309398	0.378	0.232	3.56E-55	1.1
SNX24	1.53E-59	-0.440960923	0.089	0.249	3.70E-55	1.1
IFNAR2	3.67E-59	-0.471509616	0.166	0.352	8.86E-55	1.1
SAA4	4.36E-59	-0.367812339	0.14	0.328	1.05E-54	1.1
SLC24A3	5.38E-59	-0.482542258	0.056	0.2	1.30E-54	1.1
OOEP	6.62E-59	0.270809186	0.994	0.976	1.60E-54	1.1
SCMH1	7.05E-59	-0.532280966	0.097	0.259	1.70E-54	1.1
BTG1	7.88E-59	-0.591600889	0.328	0.519	1.90E-54	1.1
ACTN1	1.27E-58	0.726584266	0.386	0.236	3.07E-54	1.1
MYO1D	1.66E-58	-0.494097191	0.137	0.314	4.00E-54	1.1
PODXL	2.25E-58	-0.462783945	0.076	0.229	5.43E-54	1.1
RP11-290O	3.54E-58	-0.565321772	0.013	0.126	8.54E-54	1.1
TGM2	4.01E-58	-0.35171901	0.01	0.12	9.67E-54	1.1
CBX1	5.21E-58	0.562124551	0.232	0.108	1.26E-53	1.1
PTPRE	6.54E-58	-0.427067947	0.035	0.165	1.58E-53	1.1
CLLU1OS	7.12E-58	-0.38924523	0.01	0.119	1.72E-53	1.1
RPL38	7.71E-58	0.322199934	0.935	0.925	1.86E-53	1.1
SERPINA3	7.98E-58	-0.671216058	0.099	0.261	1.92E-53	1.1
NSF	8.10E-58	-0.470046353	0.055	0.194	1.95E-53	1.1
AC159540.	9.83E-58	-0.628444057	0.068	0.213	2.37E-53	1.1
SERPINB3	1.47E-57	-1.175278711	0.007	0.112	3.56E-53	1.1
MORC3	1.90E-57	0.60523982	0.328	0.188	4.59E-53	1.1
MAP2K5	2.53E-57	-0.445327793	0.033	0.161	6.09E-53	1.1
CHD2	4.08E-57	0.62793837	0.533	0.395	9.84E-53	1.1
MTUS1	4.29E-57	0.670482159	0.473	0.333	1.04E-52	1.1
C10orf90	5.13E-57	-0.441179519	0.024	0.143	1.24E-52	1.1
RNF130	5.25E-57	-0.434085294	0.058	0.199	1.27E-52	1.1
PHLDA1	6.61E-57	-0.458249576	0.066	0.21	1.59E-52	1.1
EHF	9.29E-57	0.675505872	0.661	0.575	2.24E-52	1.1

HIST1H2AC	1.08E-56	-0.533253199	0.185	0.367	2.60E-52	1.1
VAMP8	1.38E-56	-0.441567729	0.59	0.739	3.34E-52	1.1
SAMD4A	1.45E-56	-0.489091904	0.503	0.691	3.50E-52	1.1
TTC17	1.46E-56	-0.48073184	0.154	0.331	3.53E-52	1.1
CAPZA1	1.56E-56	-0.429942649	0.297	0.5	3.76E-52	1.1
AFTPH	3.68E-56	0.609188939	0.419	0.277	8.87E-52	1.1
HELB	3.77E-56	-0.414504771	0.03	0.155	9.09E-52	1.1
RPL10	3.79E-56	0.566824728	0.977	0.96	9.13E-52	1.1
UQCRB	4.05E-56	0.413490443	0.853	0.818	9.76E-52	1.1
ATP1B3	4.14E-56	-0.467916146	0.143	0.313	9.99E-52	1.1
DENND1A	4.57E-56	-0.442741168	0.1	0.26	1.10E-51	1.1
C1S	4.87E-56	-0.376286009	0.027	0.148	1.17E-51	1.1
RAP1GAP2	4.95E-56	-0.515126303	0.063	0.205	1.19E-51	1.1
PDK1	5.22E-56	0.556359131	0.204	0.088	1.26E-51	1.1
C1RL	5.75E-56	-0.434484708	0.031	0.154	1.39E-51	1.1
FDCSP	7.00E-56	-3.106280471	0.1	0.243	1.69E-51	1.1
SERTAD2	7.11E-56	0.636730966	0.305	0.169	1.71E-51	1.1
SENP5	8.15E-56	-0.450547251	0.093	0.248	1.97E-51	1.1
MOB4	8.16E-56	-0.387125034	0.084	0.239	1.97E-51	1.1
IER3	1.34E-55	-0.55130499	0.049	0.182	3.22E-51	1.1
MYCBP2	1.35E-55	-0.459336385	0.068	0.21	3.26E-51	1.1
NUP107	2.59E-55	-0.405921007	0.053	0.19	6.26E-51	1.1
RNF13	2.65E-55	-0.450832844	0.168	0.347	6.38E-51	1.1
RPL27	3.15E-55	0.280082791	0.97	0.949	7.59E-51	1.1
TPST1	3.50E-55	-0.53118728	0.048	0.181	8.44E-51	1.1
ANKRD36B	3.92E-55	-0.492038074	0.03	0.149	9.44E-51	1.1
HDAC9	4.24E-55	-0.512417511	0.116	0.277	1.02E-50	1.1
PAM	5.43E-55	-0.374615162	0.42	0.643	1.31E-50	1.1
DLEU2	6.63E-55	-0.46898265	0.081	0.228	1.60E-50	1.1
PKP4	9.90E-55	-0.436186997	0.291	0.492	2.39E-50	1.1
BAZ2B	1.04E-54	-0.431633196	0.295	0.494	2.51E-50	1.1
CCL28	1.05E-54	-0.698372532	0.609	0.742	2.53E-50	1.1
SLC20A2	1.15E-54	0.633013673	0.489	0.349	2.78E-50	1.1
GBP3	1.65E-54	-0.450052924	0.06	0.197	3.97E-50	1.1
DAPL1	1.65E-54	0.382160929	0.108	0.029	3.97E-50	1.1
CSTB	2.08E-54	-0.586156022	0.082	0.226	5.03E-50	1.1
CACNB2	2.16E-54	-0.490078428	0.048	0.178	5.22E-50	1.1
NBEA	3.06E-54	-0.484356338	0.025	0.141	7.37E-50	1.1
R3HDM2	3.32E-54	-0.453659381	0.054	0.188	8.00E-50	1.1
SDC4	3.62E-54	-0.532521981	0.287	0.486	8.72E-50	1.1
ZNF254	4.03E-54	-0.395386032	0.06	0.197	9.72E-50	1.1
SEL1L	4.47E-54	0.500216261	0.167	0.064	1.08E-49	1.1
KIT	4.53E-54	-0.515765171	0.228	0.417	1.09E-49	1.1
SGMS2	8.48E-54	0.565464659	0.276	0.143	2.04E-49	1.1
MGP	1.05E-53	1.170616169	0.893	0.86	2.52E-49	1.1
GSK3B	1.13E-53	-0.453090215	0.167	0.343	2.71E-49	1.1
RAD51B	1.32E-53	-0.536406197	0.11	0.266	3.19E-49	1.1

RP11-142C	1.34E-53	-0.455378495	0.033	0.153	3.24E-49	1.1
EIF4E	1.42E-53	0.592555769	0.482	0.349	3.42E-49	1.1
SKP1	1.46E-53	0.408259732	0.874	0.825	3.51E-49	1.1
KIAA1551	2.26E-53	-0.386687085	0.041	0.166	5.44E-49	1.1
WDR70	4.29E-53	-0.41422324	0.04	0.164	1.03E-48	1.1
ZNF608	5.87E-53	-0.437834295	0.064	0.202	1.41E-48	1.1
C10orf10	6.64E-53	-0.443301923	0.087	0.236	1.60E-48	1.1
INVS	7.01E-53	-0.439623724	0.041	0.166	1.69E-48	1.1
C5orf28	9.62E-53	-0.425289771	0.076	0.218	2.32E-48	1.1
RP4-678D1	1.06E-52	-0.434889811	0.059	0.197	2.56E-48	1.1
SPINK5	2.37E-52	0.372394341	0.057	0.006	5.71E-48	1.1
PHF21A	2.61E-52	-0.444692769	0.126	0.287	6.29E-48	1.1
RP11-536O	4.00E-52	-0.425710107	0.033	0.15	9.64E-48	1.1
SLC2A1	4.37E-52	0.480636014	0.15	0.054	1.05E-47	1.1
UBAC2	7.34E-52	-0.436236973	0.103	0.253	1.77E-47	1.1
C14orf119	1.19E-51	-0.408415204	0.089	0.234	2.88E-47	1.1
RPL24	1.23E-51	0.282238399	0.975	0.946	2.97E-47	1.1
PPP1R9A	1.35E-51	-0.490893947	0.098	0.245	3.24E-47	1.1
SIPA1L3	1.74E-51	-0.484224945	0.076	0.217	4.19E-47	1.1
FBXO32	1.92E-51	-0.684720351	0.283	0.462	4.63E-47	1.1
RBM6	2.12E-51	-0.444357867	0.198	0.38	5.10E-47	1.1
GNG12	2.28E-51	0.635064534	0.4	0.27	5.51E-47	1.1
BRINP1	2.38E-51	-0.557259971	0.093	0.24	5.74E-47	1.1
TACSTD2	2.46E-51	-0.370538305	0.578	0.772	5.92E-47	1.1
MAGED1	5.15E-51	-0.365313449	0.026	0.139	1.24E-46	1.1
ARHGEF3	5.46E-51	-0.363911176	0.18	0.357	1.32E-46	1.1
CYP1B1	5.54E-51	-0.50587192	0.038	0.157	1.34E-46	1.1
ATP6V0E1	6.14E-51	-0.392817508	0.541	0.712	1.48E-46	1.1
FRMD6	6.92E-51	0.628835706	0.29	0.162	1.67E-46	1.1
RP11-114H	7.11E-51	-0.711269131	0.084	0.222	1.71E-46	1.1
OXSR1	9.95E-51	0.574234601	0.332	0.198	2.40E-46	1.1
FAF2	1.97E-50	-0.382459555	0.072	0.208	4.74E-46	1.1
CMTM8	2.10E-50	-0.454349489	0.09	0.234	5.07E-46	1.1
HSD17B7	2.60E-50	-0.412381076	0.032	0.146	6.27E-46	1.1
FRK	2.96E-50	-0.407694239	0.068	0.202	7.14E-46	1.1
KRT6B	2.97E-50	-0.577981428	0.035	0.151	7.16E-46	1.1
SMIM14	2.99E-50	-0.404144583	0.061	0.191	7.21E-46	1.1
ZNF143	3.97E-50	0.50195075	0.237	0.118	9.58E-46	1.1
KCTD3	4.63E-50	0.600546767	0.293	0.167	1.12E-45	1.1
CTSS	4.64E-50	-0.47900236	0.147	0.306	1.12E-45	1.1
UQCRH	4.85E-50	0.423285153	0.863	0.851	1.17E-45	1.1
BMPR1A	5.70E-50	-0.416411571	0.16	0.329	1.37E-45	1.1
LRIG1	5.76E-50	-0.502555105	0.13	0.287	1.39E-45	1.1
UBR5	6.17E-50	0.570813051	0.5	0.376	1.49E-45	1.1
MT-ND6	6.28E-50	-0.389951687	0.065	0.197	1.51E-45	1.1
FER	6.31E-50	-0.397344001	0.102	0.254	1.52E-45	1.1
ASCC1	7.32E-50	-0.38874652	0.072	0.208	1.76E-45	1.1

SPDYE16	1.19E-49	-0.348591762	0.021	0.126	2.88E-45	1.1
GMDS-AS1	1.80E-49	-0.472136973	0.033	0.147	4.35E-45	1.1
BCAP29	2.22E-49	-0.436239588	0.063	0.191	5.35E-45	1.1
ZNF544	2.41E-49	-0.339158172	0.022	0.128	5.80E-45	1.1
PDSS2	2.54E-49	-0.404375577	0.047	0.169	6.13E-45	1.1
TBC1D22A	2.64E-49	-0.412463256	0.043	0.163	6.37E-45	1.1
CASC4	2.97E-49	-0.380567103	0.082	0.222	7.17E-45	1.1
WIPI1	3.08E-49	-0.378654433	0.068	0.203	7.42E-45	1.1
EVI5	3.37E-49	-0.419062095	0.048	0.17	8.12E-45	1.1
LRRC37A3	3.81E-49	-0.357971701	0.038	0.153	9.18E-45	1.1
PHF14	3.90E-49	-0.423239584	0.054	0.178	9.41E-45	1.1
TPD52L1	4.42E-49	-0.469434209	0.116	0.265	1.06E-44	1.1
CLIC6	4.53E-49	-0.373238587	0.029	0.139	1.09E-44	1.1
MKL2	5.86E-49	-0.421494047	0.142	0.302	1.41E-44	1.1
ZBTB8OS	8.28E-49	-0.337336531	0.048	0.17	2.00E-44	1.1
UBAP1	1.30E-48	0.569956038	0.431	0.302	3.13E-44	1.1
GANC	1.32E-48	-0.367429406	0.026	0.133	3.18E-44	1.1
CRTC3	1.66E-48	-0.446125477	0.144	0.299	4.01E-44	1.1
AKR1C2	2.15E-48	-0.402365858	0.009	0.101	5.18E-44	1.1
MRPL13	2.27E-48	-0.385730317	0.08	0.216	5.46E-44	1.1
GCLM	2.42E-48	-0.433475934	0.062	0.19	5.83E-44	1.1
MED31	2.48E-48	-0.357152982	0.088	0.227	5.97E-44	1.1
BMPR2	2.69E-48	-0.398751407	0.122	0.275	6.49E-44	1.1
SNRPD2	2.79E-48	0.470502557	0.761	0.739	6.72E-44	1.1
HDGF	2.90E-48	-0.41152444	0.148	0.306	6.98E-44	1.1
RABGAP1	2.92E-48	-0.392595798	0.178	0.348	7.05E-44	1.1
FAF1	3.28E-48	-0.467497033	0.154	0.312	7.90E-44	1.1
NF1	4.35E-48	-0.388915085	0.314	0.508	1.05E-43	1.1
ZCCHC17	4.73E-48	-0.368954781	0.185	0.358	1.14E-43	1.1
DAPK2	6.22E-48	0.671648733	0.527	0.417	1.50E-43	1.1
TTC39C	6.31E-48	-0.394750651	0.046	0.166	1.52E-43	1.1
OTUD6B-AS	6.54E-48	-0.339627659	0.092	0.237	1.58E-43	1.1
HIVEP1	1.01E-47	-0.393507974	0.065	0.194	2.44E-43	1.1
FOXN3	1.03E-47	-0.463176303	0.125	0.272	2.47E-43	1.1
UBE2V2	1.42E-47	-0.349275055	0.165	0.334	3.42E-43	1.1
R3HCC1L	2.43E-47	-0.362639429	0.041	0.157	5.86E-43	1.1
UST	2.90E-47	-0.518437493	0.136	0.287	7.00E-43	1.1
JAK2	3.22E-47	-0.490419225	0.013	0.108	7.77E-43	1.1
MECOM	3.29E-47	0.347998708	0.904	0.883	7.92E-43	1.1
SYNJ2	3.50E-47	-0.449890109	0.189	0.356	8.45E-43	1.1
FAM114A1	5.31E-47	-0.365019289	0.058	0.182	1.28E-42	1.1
AC026202.	6.20E-47	0.605916394	0.286	0.164	1.50E-42	1.1
FAM63B	6.90E-47	-0.368807121	0.046	0.162	1.66E-42	1.1
RSRC1	7.42E-47	-0.388736571	0.143	0.298	1.79E-42	1.1
USP34	8.20E-47	-0.374827091	0.44	0.637	1.98E-42	1.1
SLC16A1-A	9.91E-47	-0.390879357	0.034	0.143	2.39E-42	1.1
VPS13C	1.03E-46	-0.406725948	0.112	0.259	2.47E-42	1.1

GJC3	1.08E-46	0.439747668	0.153	0.061	2.60E-42	1.1
EIF3L	1.43E-46	0.405206986	0.792	0.751	3.44E-42	1.1
OTUD3	1.43E-46	0.343651389	0.101	0.029	3.45E-42	1.1
RP11-83A2	1.56E-46	-0.349923785	0.037	0.149	3.75E-42	1.1
TMCC1	1.67E-46	-0.416143493	0.16	0.319	4.02E-42	1.1
SERPINB1	1.88E-46	-0.371662682	0.028	0.133	4.54E-42	1.1
GADD45A	1.96E-46	0.702369104	0.322	0.2	4.72E-42	1.1
SOX10	2.37E-46	-0.415528591	0.044	0.158	5.71E-42	1.1
LRRC16A	2.58E-46	-0.431152039	0.082	0.212	6.23E-42	1.1
SWAP70	3.01E-46	0.602571874	0.396	0.271	7.25E-42	1.1
PTPRM	3.41E-46	-0.592462011	0.079	0.208	8.23E-42	1.1
SUMF1	3.49E-46	-0.422115268	0.036	0.145	8.43E-42	1.1
GNAQ	3.65E-46	-0.41955525	0.191	0.357	8.80E-42	1.1
FOSL2	3.68E-46	-0.377720872	0.083	0.216	8.87E-42	1.1
RBX1	4.93E-46	-0.370121239	0.317	0.503	1.19E-41	1.1
PDXK	4.98E-46	-0.41333988	0.068	0.194	1.20E-41	1.1
ZNF43	5.09E-46	-0.373827493	0.054	0.173	1.23E-41	1.1
SUPT3H	5.57E-46	-0.408711809	0.066	0.193	1.34E-41	1.1
EYA2	5.71E-46	-0.441529048	0.102	0.241	1.38E-41	1.1
FAM20C	8.27E-46	-0.458452901	0.026	0.128	1.99E-41	1.1
PHKB	1.02E-45	-0.386641945	0.096	0.233	2.46E-41	1.1
ADAM10	1.03E-45	-0.382363428	0.13	0.281	2.49E-41	1.1
TTLL5	1.13E-45	-0.389447673	0.079	0.21	2.74E-41	1.1
LINC01152	1.36E-45	-0.511319496	0.043	0.155	3.28E-41	1.1
APEX1	1.53E-45	0.557506429	0.348	0.227	3.70E-41	1.1
CLIP2	1.62E-45	-0.436744292	0.089	0.223	3.91E-41	1.1
WFDC3	1.70E-45	-0.330264087	0.028	0.132	4.10E-41	1.1
C1R	1.78E-45	-0.342623554	0.01	0.1	4.30E-41	1.1
ADK	1.81E-45	-0.478896603	0.325	0.504	4.37E-41	1.1
SLMAP	2.20E-45	0.52813908	0.678	0.613	5.31E-41	1.1
MINK1	2.66E-45	-0.391821477	0.055	0.173	6.40E-41	1.1
DGKH	2.69E-45	-0.392170317	0.066	0.191	6.49E-41	1.1
CYP27A1	2.84E-45	-0.323466922	0.014	0.107	6.84E-41	1.1
UBE2E3	3.11E-45	-0.40139959	0.08	0.21	7.51E-41	1.1
RELB	3.13E-45	-0.411090665	0.062	0.182	7.54E-41	1.1
ADM	3.19E-45	0.251218537	0.063	0.011	7.70E-41	1.1
ABLIM1	3.20E-45	-0.437840949	0.152	0.303	7.71E-41	1.1
TMEM87B	4.74E-45	-0.336344494	0.018	0.112	1.14E-40	1.1
NNMT	5.34E-45	-0.4736973	0.015	0.108	1.29E-40	1.1
MMP7	6.00E-45	-0.560097557	0.196	0.354	1.45E-40	1.1
GALNT15	7.28E-45	-0.644294585	0.058	0.174	1.75E-40	1.1
RP11-449D	8.83E-45	-0.428430213	0.018	0.113	2.13E-40	1.1
C11orf54	9.12E-45	-0.308381174	0.026	0.127	2.20E-40	1.1
DAPP1	1.09E-44	-0.391506126	0.277	0.462	2.64E-40	1.1
CYCS	1.13E-44	0.527937208	0.624	0.515	2.72E-40	1.1
DOCK7	1.41E-44	-0.434886728	0.135	0.282	3.40E-40	1.1
STOX2	1.71E-44	-0.393721837	0.083	0.213	4.14E-40	1.1

TIMM9	1.86E-44	0.517022993	0.307	0.19	4.50E-40	1.1
GGPS1	1.98E-44	-0.361824078	0.101	0.239	4.78E-40	1.1
SMARCA2	2.37E-44	-0.415056635	0.184	0.344	5.71E-40	1.1
SEC62	3.63E-44	0.544021703	0.679	0.606	8.76E-40	1.1
GCC2	3.67E-44	-0.402911533	0.206	0.37	8.85E-40	1.1
TATDN1	4.31E-44	0.516726211	0.397	0.277	1.04E-39	1.1
NFATC2	5.10E-44	0.595031393	0.226	0.117	1.23E-39	1.1
HERPUD1	5.64E-44	0.551257348	0.284	0.164	1.36E-39	1.1
CLU	6.01E-44	-0.682620215	0.034	0.138	1.45E-39	1.1
FBXL20	6.70E-44	-0.378894974	0.143	0.295	1.61E-39	1.1
SFRP1	9.12E-44	-0.449194033	0.33	0.507	2.20E-39	1.1
ARPP19	9.26E-44	0.558217939	0.421	0.302	2.23E-39	1.1
GPD2	1.02E-43	-0.35372079	0.132	0.28	2.47E-39	1.1
FANCC	1.31E-43	-0.327806628	0.046	0.159	3.16E-39	1.1
TTLL4	1.60E-43	-0.360800263	0.077	0.204	3.85E-39	1.1
PPTC7	1.76E-43	0.61245078	0.306	0.185	4.24E-39	1.1
RARRES3	2.12E-43	0.587379791	0.406	0.284	5.11E-39	1.1
CD2AP	2.16E-43	0.694144422	0.466	0.355	5.20E-39	1.1
RPRD2	2.17E-43	-0.383598841	0.063	0.181	5.23E-39	1.1
ELF3	2.44E-43	0.638547488	0.605	0.513	5.88E-39	1.1
TBCK	2.86E-43	-0.376234022	0.045	0.155	6.89E-39	1.1
SLC35E3	3.09E-43	-0.325654725	0.025	0.123	7.44E-39	1.1
ZRANB2	3.19E-43	-0.387452226	0.218	0.383	7.70E-39	1.1
ARF4	3.36E-43	0.456737727	0.702	0.635	8.10E-39	1.1
MFGE8	3.61E-43	-0.714184039	0.094	0.221	8.72E-39	1.1
DYNLL1	3.99E-43	0.560368855	0.32	0.199	9.61E-39	1.1
RANBP2	4.23E-43	0.540422858	0.366	0.245	1.02E-38	1.1
FOXO1	4.57E-43	-0.37952572	0.143	0.29	1.10E-38	1.1
IRAK2	4.82E-43	-0.382623261	0.056	0.172	1.16E-38	1.1
TBL1X	6.47E-43	-0.374079671	0.074	0.198	1.56E-38	1.1
FCHSD2	6.71E-43	-0.345295661	0.032	0.134	1.62E-38	1.1
SREBF2	7.89E-43	-0.373014466	0.236	0.406	1.90E-38	1.1
PCCA	8.44E-43	-0.381598089	0.07	0.192	2.04E-38	1.1
CDK14	9.65E-43	-0.389492381	0.274	0.45	2.33E-38	1.1
GNA13	1.06E-42	0.520958832	0.286	0.167	2.55E-38	1.1
CPEB4	1.19E-42	-0.350516864	0.087	0.217	2.87E-38	1.1
PAPSS1	1.21E-42	-0.399599024	0.254	0.42	2.92E-38	1.1
PCNXL4	1.21E-42	-0.315767621	0.028	0.127	2.93E-38	1.1
S100A10	1.31E-42	-0.493181408	0.191	0.351	3.15E-38	1.1
CEBPB	1.36E-42	-0.345410909	0.06	0.177	3.27E-38	1.1
PKD2	1.61E-42	-0.335768441	0.018	0.109	3.88E-38	1.1
MARCO	1.73E-42	-0.394352998	0.028	0.125	4.17E-38	1.1
SCAPER	1.89E-42	-0.419305171	0.12	0.256	4.55E-38	1.1
UACA	2.15E-42	-0.391106308	0.076	0.199	5.18E-38	1.1
RAI14	2.31E-42	0.58525962	0.253	0.141	5.56E-38	1.1
ELOVL6	2.60E-42	-0.354604132	0.017	0.107	6.27E-38	1.1
NLK	2.98E-42	-0.341511872	0.03	0.129	7.20E-38	1.1

SUB1	3.45E-42	-0.39182556	0.67	0.784	8.32E-38	1.1
APP	4.25E-42	-0.410960702	0.671	0.806	1.02E-37	1.1
LDHC	4.63E-42	0.297782823	0.064	0.012	1.12E-37	1.1
STXBP5	4.99E-42	-0.316188627	0.026	0.122	1.20E-37	1.1
KRIT1	5.34E-42	-0.336348998	0.043	0.151	1.29E-37	1.1
CLASP2	5.50E-42	-0.363590249	0.128	0.27	1.33E-37	1.1
TMCO4	6.27E-42	-0.323296324	0.027	0.123	1.51E-37	1.1
TGOLN2	6.90E-42	-0.318811522	0.037	0.139	1.66E-37	1.1
PARD3	9.19E-42	-0.346649176	0.517	0.692	2.22E-37	1.1
LDLRAD4	1.00E-41	-0.458297317	0.082	0.207	2.42E-37	1.1
S100A9	1.01E-41	-0.908600574	0.008	0.089	2.44E-37	1.1
SS18	1.15E-41	-0.340148487	0.063	0.18	2.78E-37	1.1
BCL2L14	1.20E-41	0.444294519	0.226	0.119	2.89E-37	1.1
MYO1E	1.34E-41	-0.436428962	0.357	0.535	3.24E-37	1.1
PITPNA	1.37E-41	-0.354884487	0.059	0.173	3.31E-37	1.1
PLD1	1.47E-41	-0.32134449	0.028	0.123	3.55E-37	1.1
AGFG1	1.66E-41	0.578780671	0.486	0.381	4.01E-37	1.1
SH3GL1	1.68E-41	0.402666055	0.155	0.066	4.05E-37	1.1
CASP7	2.06E-41	-0.327755765	0.076	0.198	4.97E-37	1.1
MBP	2.09E-41	-0.361621389	0.084	0.209	5.03E-37	1.1
WWP1	2.49E-41	-0.335038217	0.094	0.224	6.00E-37	1.1
APLF	2.85E-41	-0.312253748	0.018	0.107	6.88E-37	1.1
NFIC	3.08E-41	-0.36114163	0.082	0.206	7.44E-37	1.1
S100A14	4.78E-41	-0.41438626	0.56	0.705	1.15E-36	1.1
CAMKMT	4.85E-41	-0.412658929	0.074	0.194	1.17E-36	1.1
HERC4	4.90E-41	-0.342858738	0.261	0.441	1.18E-36	1.1
GPHN	5.31E-41	-0.35283122	0.178	0.335	1.28E-36	1.1
ARNTL2	7.15E-41	-0.382228213	0.055	0.165	1.72E-36	1.1
EXOC6B	7.26E-41	-0.344828038	0.098	0.228	1.75E-36	1.1
METTL15	8.42E-41	-0.3652478	0.051	0.16	2.03E-36	1.1
CHD9	1.31E-40	-0.324648391	0.334	0.518	3.15E-36	1.1
RSU1	1.37E-40	-0.394783314	0.102	0.231	3.31E-36	1.1
SAP18	1.57E-40	0.435885525	0.652	0.586	3.80E-36	1.1
MOB3B	1.94E-40	-0.340047994	0.139	0.282	4.67E-36	1.1
PUM2	2.67E-40	0.545879639	0.437	0.327	6.44E-36	1.1
ZNF124	2.75E-40	-0.302922416	0.023	0.113	6.63E-36	1.1
ASH1L	2.92E-40	-0.351489679	0.368	0.546	7.04E-36	1.1
NR2C2	3.14E-40	-0.342286567	0.057	0.167	7.56E-36	1.1
EDN1	3.16E-40	0.590069192	0.299	0.182	7.61E-36	1.1
TAPBP	3.29E-40	-0.36095163	0.047	0.152	7.93E-36	1.1
FXR1	3.38E-40	0.511996916	0.501	0.401	8.16E-36	1.1
EIF2A	3.48E-40	0.499281019	0.502	0.409	8.39E-36	1.1
GTF2IRD2	3.78E-40	-0.308935253	0.024	0.115	9.12E-36	1.1
NFATC3	4.16E-40	-0.342745526	0.073	0.191	1.00E-35	1.1
RP1-28O10	4.36E-40	-0.272869955	0.008	0.086	1.05E-35	1.1
HSD17B12	4.52E-40	-0.383768072	0.113	0.245	1.09E-35	1.1
INTS10	5.65E-40	-0.319999899	0.038	0.138	1.36E-35	1.1

GS1-114I9.	9.38E-40	-0.303863796	0.048	0.155	2.26E-35	1.1
PTPRA	9.46E-40	-0.357052762	0.067	0.181	2.28E-35	1.1
ANXA7	1.06E-39	-0.35190467	0.184	0.334	2.57E-35	1.1
RGL1	1.21E-39	-0.335757073	0.025	0.116	2.93E-35	1.1
C6orf62	1.53E-39	-0.290705817	0.085	0.207	3.70E-35	1.1
TBC1D1	1.93E-39	-0.372662133	0.098	0.225	4.66E-35	1.1
EIF3K	2.17E-39	0.594208921	0.528	0.454	5.22E-35	1.1
RHBDD1	2.43E-39	-0.319577777	0.049	0.155	5.85E-35	1.1
SMG6	2.96E-39	-0.369685356	0.074	0.19	7.14E-35	1.1
INTS12	3.03E-39	0.519436491	0.274	0.164	7.30E-35	1.1
AVL9	3.37E-39	-0.345822542	0.09	0.214	8.13E-35	1.1
PARP14	4.62E-39	-0.341877294	0.071	0.185	1.11E-34	1.1
RP11-84A1	4.72E-39	0.282848559	0.086	0.025	1.14E-34	1.1
RPS17	5.97E-39	0.212607414	0.979	0.958	1.44E-34	1.1
CLOCK	6.15E-39	-0.338662613	0.095	0.221	1.48E-34	1.1
CUL5	6.94E-39	-0.327543369	0.056	0.162	1.67E-34	1.1
SYTL2	7.68E-39	-0.315319487	0.062	0.173	1.85E-34	1.1
TNIP3	8.46E-39	-0.307262361	0.017	0.1	2.04E-34	1.1
IDH2	8.76E-39	-0.308489592	0.023	0.111	2.11E-34	1.1
GLUD1	9.30E-39	0.564307012	0.241	0.135	2.24E-34	1.1
ENPP6	9.46E-39	-0.257252973	0.001	0.069	2.28E-34	1.1
AKR1B1	1.10E-38	-0.410125379	0.013	0.092	2.66E-34	1.1
CYFIP1	1.22E-38	-0.350075516	0.048	0.151	2.95E-34	1.1
SLC30A7	1.24E-38	-0.311053712	0.044	0.146	2.99E-34	1.1
ZNF429	1.33E-38	-0.27477491	0.019	0.104	3.21E-34	1.1
LRSAM1	1.35E-38	-0.323216576	0.043	0.142	3.25E-34	1.1
AHCYL1	1.70E-38	-0.336588745	0.175	0.322	4.09E-34	1.1
NALCN	2.20E-38	-0.37367041	0.071	0.184	5.31E-34	1.1
ZNF91	2.39E-38	-0.324999741	0.065	0.176	5.77E-34	1.1
PIP5K1A	2.79E-38	0.525296006	0.385	0.275	6.73E-34	1.1
ZC3HAV1	3.00E-38	-0.296150845	0.041	0.14	7.24E-34	1.1
ERBB2IP	3.06E-38	-0.370784877	0.167	0.31	7.39E-34	1.1
VPS13B	3.31E-38	-0.335310048	0.079	0.197	7.97E-34	1.1
ATRNL1	3.40E-38	-0.367526932	0.017	0.1	8.19E-34	1.1
ZNF397	3.58E-38	-0.306041272	0.043	0.141	8.62E-34	1.1
AEBP2	3.61E-38	-0.392268573	0.143	0.28	8.71E-34	1.1
NIN	3.65E-38	-0.284630467	0.033	0.128	8.81E-34	1.1
UMAD1	3.82E-38	-0.3408162	0.039	0.136	9.20E-34	1.1
TFCP2	4.15E-38	-0.317873832	0.058	0.165	1.00E-33	1.1
RPS3	4.21E-38	0.609112506	0.927	0.927	1.01E-33	1.1
CNIH4	4.31E-38	-0.340006239	0.265	0.424	1.04E-33	1.1
LITAF	4.83E-38	-0.329545787	0.368	0.544	1.16E-33	1.1
B4GALT1	4.95E-38	-0.383918875	0.285	0.447	1.19E-33	1.1
ZNF708	5.01E-38	-0.333065353	0.025	0.114	1.21E-33	1.1
DPYSL2	5.62E-38	-0.315545079	0.05	0.154	1.35E-33	1.1
ANKRD37	6.78E-38	0.330572436	0.12	0.046	1.63E-33	1.1
FAM120B	7.04E-38	-0.294129973	0.041	0.139	1.70E-33	1.1

TSG101	7.40E-38	-0.311528851	0.088	0.209	1.78E-33	1.1
RGS10	7.69E-38	-0.331684125	0.046	0.146	1.85E-33	1.1
AFF4	7.99E-38	0.516164062	0.499	0.405	1.93E-33	1.1
CEP350	8.51E-38	-0.342100033	0.104	0.228	2.05E-33	1.1
TFG	8.90E-38	0.53614745	0.391	0.283	2.15E-33	1.1
RFX3	9.08E-38	-0.358274292	0.077	0.191	2.19E-33	1.1
DNAJB6	1.08E-37	0.596787552	0.428	0.323	2.61E-33	1.1
MYRFL	1.10E-37	-0.299280602	0.015	0.095	2.65E-33	1.1
PUM1	1.24E-37	0.488775885	0.57	0.493	2.99E-33	1.1
CXCL16	1.32E-37	-0.310457455	0.106	0.233	3.17E-33	1.1
DMKN	1.63E-37	-0.296488833	0.039	0.135	3.93E-33	1.1
C16orf72	1.67E-37	0.415103536	0.218	0.117	4.03E-33	1.1
NIPAL3	1.79E-37	-0.305360225	0.018	0.101	4.32E-33	1.1
MCTP2	1.93E-37	-0.352199418	0.068	0.179	4.66E-33	1.1
PDE8A	1.96E-37	-0.380017257	0.351	0.52	4.73E-33	1.1
COPS2	2.11E-37	0.467495737	0.433	0.326	5.10E-33	1.1
LAMB3	2.51E-37	-0.332868052	0.177	0.329	6.06E-33	1.1
IPO7	2.68E-37	0.509626111	0.371	0.261	6.46E-33	1.1
NBR1	2.83E-37	-0.323687337	0.085	0.202	6.82E-33	1.1
IGF2BP2	3.06E-37	0.675947888	0.563	0.492	7.39E-33	1.1
CATSPERB	3.79E-37	-0.255003013	0.142	0.284	9.13E-33	1.1
LDLR	4.18E-37	-0.384428435	0.083	0.199	1.01E-32	1.1
PARD3B	4.44E-37	-0.330503031	0.055	0.159	1.07E-32	1.1
CDH3	5.09E-37	-0.350113574	0.068	0.176	1.23E-32	1.1
CALM1	5.13E-37	-0.375078129	0.133	0.264	1.24E-32	1.1
ANXA3	5.32E-37	-0.460894415	0.306	0.452	1.28E-32	1.1
TES	5.73E-37	0.496244026	0.404	0.292	1.38E-32	1.1
GTF2H5	5.91E-37	-0.333360637	0.13	0.259	1.42E-32	1.1
CD9	6.13E-37	-0.346708673	0.153	0.291	1.48E-32	1.1
TRAF3	6.44E-37	-0.328842429	0.059	0.164	1.55E-32	1.1
MPHOSPH8	9.00E-37	-0.308310755	0.083	0.199	2.17E-32	1.1
HNRNPM	9.27E-37	0.469367849	0.273	0.166	2.23E-32	1.1
SCARA3	9.51E-37	-0.322659221	0.011	0.086	2.29E-32	1.1
XPO4	9.77E-37	-0.298459854	0.059	0.164	2.35E-32	1.1
NUB1	1.01E-36	-0.634926761	0.194	0.327	2.45E-32	1.1
C10orf76	1.06E-36	-0.29540211	0.058	0.161	2.55E-32	1.1
RASAL2	1.42E-36	0.757446804	0.59	0.524	3.44E-32	1.1
TTC9	1.62E-36	-0.327974082	0.104	0.228	3.90E-32	1.1
DHRSX	1.66E-36	-0.369097483	0.129	0.258	4.00E-32	1.1
ZPLD1	1.78E-36	-0.537486646	0.053	0.153	4.28E-32	1.1
RGS2	1.92E-36	1.098757703	0.287	0.184	4.63E-32	1.1
COG5	2.00E-36	-0.333270619	0.333	0.504	4.82E-32	1.1
IL4R	2.02E-36	-0.337530812	0.06	0.163	4.88E-32	1.1
PARVA	3.16E-36	-0.326499425	0.065	0.172	7.63E-32	1.1
STEAP1B	3.23E-36	0.365637561	0.975	0.946	7.80E-32	1.1
EBP	3.42E-36	-0.308030718	0.037	0.129	8.25E-32	1.1
VDR	3.62E-36	-0.269116234	0.03	0.119	8.74E-32	1.1

RPL37	3.74E-36	0.229287369	0.987	0.969	9.03E-32	1.1
ADGRF1	3.88E-36	-0.29658722	0.009	0.08	9.36E-32	1.1
PTPN4	4.07E-36	-0.287706274	0.028	0.114	9.81E-32	1.1
ZNF235	4.11E-36	-0.281118386	0.018	0.097	9.92E-32	1.1
KIAA2026	5.41E-36	-0.321659194	0.068	0.174	1.30E-31	1.1
RPS6KC1	5.82E-36	-0.289760336	0.025	0.11	1.40E-31	1.1
NCEH1	7.87E-36	-0.427568873	0.126	0.255	1.90E-31	1.1
PPP2R5A	8.32E-36	-0.320338135	0.07	0.177	2.01E-31	1.1
ICAM1	8.43E-36	-0.334202045	0.075	0.188	2.03E-31	1.1
PPM1K	8.87E-36	-0.277890654	0.027	0.112	2.14E-31	1.1
TTC14	1.06E-35	-0.269184592	0.017	0.095	2.55E-31	1.1
ZNF592	1.09E-35	0.419435659	0.192	0.099	2.62E-31	1.1
BCL2L1	1.10E-35	-0.307352384	0.032	0.12	2.65E-31	1.1
RUFY3	1.21E-35	-0.349013668	0.09	0.206	2.93E-31	1.1
TANK	1.38E-35	-0.240331217	0.49	0.686	3.33E-31	1.1
ATXN3	1.41E-35	-0.306105448	0.083	0.197	3.39E-31	1.1
SON	1.47E-35	-0.381630508	0.579	0.73	3.54E-31	1.1
ATP2B4	1.55E-35	-0.417287526	0.169	0.31	3.73E-31	1.1
ESD	1.74E-35	0.477611617	0.519	0.433	4.20E-31	1.1
MYO9A	1.75E-35	-0.313947363	0.185	0.333	4.23E-31	1.1
SOS2	2.42E-35	-0.293062764	0.341	0.515	5.84E-31	1.1
NR3C2	2.48E-35	-0.350859022	0.049	0.146	5.97E-31	1.1
HIPK3	2.60E-35	-0.352962695	0.097	0.214	6.27E-31	1.1
NR4A1	2.97E-35	0.223001734	0.061	0.014	7.15E-31	1.1
LRIF1	3.18E-35	-0.240675504	0.014	0.089	7.66E-31	1.1
PRDM1	3.36E-35	-0.261226589	0.013	0.088	8.11E-31	1.1
NUMB	3.41E-35	-0.279736056	0.318	0.49	8.22E-31	1.1
SLC9A8	3.47E-35	-0.276216037	0.028	0.113	8.36E-31	1.1
ZNF710	3.77E-35	-0.297968879	0.036	0.125	9.10E-31	1.1
RND3	3.82E-35	0.561958567	0.381	0.272	9.21E-31	1.1
PRMT2	4.44E-35	-0.297419044	0.054	0.153	1.07E-30	1.1
TAF7	5.08E-35	-0.302874123	0.069	0.175	1.23E-30	1.1
EPS15	5.33E-35	-0.317740034	0.14	0.271	1.28E-30	1.1
CD58	5.44E-35	-0.284737331	0.028	0.112	1.31E-30	1.1
ATXN10	5.54E-35	-0.321616422	0.078	0.187	1.34E-30	1.1
TASP1	5.89E-35	-0.319210417	0.073	0.18	1.42E-30	1.1
MFSD6	6.91E-35	-0.27374066	0.027	0.112	1.67E-30	1.1
ARF6	7.04E-35	0.484588411	0.265	0.161	1.70E-30	1.1
RPL22L1	7.15E-35	0.424485637	0.615	0.543	1.73E-30	1.1
CNTNAP3B	7.25E-35	-0.271857985	0.043	0.138	1.75E-30	1.1
GLCCI1	7.26E-35	-0.258971485	0.022	0.103	1.75E-30	1.1
ROCK2	7.40E-35	-0.342456135	0.162	0.298	1.78E-30	1.1
WDR11	7.45E-35	-0.261674041	0.021	0.101	1.80E-30	1.1
DPP8	9.98E-35	-0.266753851	0.025	0.108	2.41E-30	1.1
UAP1	1.01E-34	-0.300725882	0.144	0.277	2.43E-30	1.1
SNX18	1.03E-34	0.276254962	0.11	0.042	2.49E-30	1.1
FOCAD	1.09E-34	-0.339917032	0.095	0.21	2.63E-30	1.1

FBXL4	1.17E-34	-0.268607076	0.025	0.107	2.83E-30	1.1
ATE1	1.18E-34	-0.297198122	0.041	0.133	2.85E-30	1.1
CRADD	1.25E-34	-0.323962842	0.036	0.124	3.02E-30	1.1
RBBP6	1.33E-34	-0.300565366	0.132	0.26	3.21E-30	1.1
MICU2	1.37E-34	-0.298648013	0.058	0.158	3.31E-30	1.1
LINGO2	1.37E-34	-0.556329055	0.013	0.086	3.32E-30	1.1
LIMCH1	1.45E-34	-0.297213215	0.164	0.306	3.49E-30	1.1
LARGE	1.55E-34	-0.33546211	0.139	0.268	3.75E-30	1.1
LARP7	1.66E-34	-0.288100202	0.09	0.205	4.01E-30	1.1
TKT	1.94E-34	-0.305748983	0.06	0.162	4.67E-30	1.1
RAB31	2.05E-34	-0.29514769	0.042	0.134	4.93E-30	1.1
MMP24	2.06E-34	-0.277327777	0.04	0.131	4.96E-30	1.1
FAM157C	2.07E-34	-0.288434806	0.075	0.185	4.99E-30	1.1
LPIN1	2.16E-34	0.557795166	0.419	0.316	5.21E-30	1.1
TGFB2	2.28E-34	0.546462268	0.325	0.214	5.50E-30	1.1
CAP1	2.32E-34	-0.310529375	0.135	0.262	5.60E-30	1.1
PPA2	2.46E-34	-0.311070991	0.113	0.234	5.92E-30	1.1
MAPKAPK2	2.75E-34	0.363380506	0.196	0.102	6.62E-30	1.1
SDCCAG8	2.78E-34	-0.353903321	0.1	0.214	6.71E-30	1.1
BIRC6-AS2	2.99E-34	-0.301050169	0.135	0.266	7.20E-30	1.1
MITD1	3.98E-34	-0.256913545	0.046	0.141	9.59E-30	1.1
RSBN1L	4.04E-34	0.473892884	0.189	0.099	9.75E-30	1.1
ZNF106	4.42E-34	-0.286462877	0.049	0.144	1.07E-29	1.1
PNRC1	4.51E-34	-0.267834133	0.409	0.583	1.09E-29	1.1
SP1	4.56E-34	-0.309408545	0.065	0.167	1.10E-29	1.1
UBR1	4.84E-34	-0.304222547	0.058	0.156	1.17E-29	1.1
MEST	5.10E-34	0.269715302	0.078	0.023	1.23E-29	1.1
SHPRH	5.57E-34	-0.309878516	0.04	0.128	1.34E-29	1.1
CDKAL1	5.70E-34	-0.318291839	0.149	0.281	1.37E-29	1.1
HERC3	6.24E-34	0.541617724	0.226	0.13	1.50E-29	1.1
C20orf194	6.42E-34	-0.330500378	0.081	0.19	1.55E-29	1.1
PPM1L	6.85E-34	-0.297153523	0.018	0.094	1.65E-29	1.1
TALDO1	8.00E-34	-0.302788449	0.024	0.103	1.93E-29	1.1
DIAPH3	8.03E-34	0.486622794	0.195	0.102	1.94E-29	1.1
PHLDB2	8.51E-34	-0.372136691	0.157	0.287	2.05E-29	1.1
MEIS2	9.07E-34	-0.363220988	0.158	0.291	2.19E-29	1.1
IMMP2L	9.38E-34	-0.471572775	0.218	0.352	2.26E-29	1.1
ZBED5	9.43E-34	-0.249338888	0.02	0.098	2.27E-29	1.1
ATP6V0A1	9.66E-34	0.425532111	0.247	0.145	2.33E-29	1.1
DIRC2	9.73E-34	-0.257685635	0.015	0.089	2.35E-29	1.1
CCNY	1.37E-33	0.825483378	0.331	0.235	3.31E-29	1.1
PDK3	1.62E-33	0.641542259	0.358	0.254	3.90E-29	1.1
TRIB2	1.64E-33	-0.21672585	0.008	0.074	3.97E-29	1.1
PPP1R21	1.67E-33	-0.275169651	0.03	0.114	4.02E-29	1.1
SMAD1	1.74E-33	-0.24410597	0.028	0.109	4.21E-29	1.1
MT-ND1	1.78E-33	-0.3232977	0.962	0.976	4.30E-29	1.1
NAIP	1.80E-33	-0.24204071	0.012	0.082	4.34E-29	1.1

ELMOD3	1.89E-33	-0.267069628	0.029	0.111	4.55E-29	1.1
GS1-24F4.2	2.04E-33	-0.328911029	0.042	0.132	4.92E-29	1.1
CRABP1	2.10E-33	0.112906848	0.04	0.006	5.06E-29	1.1
PSD3	2.12E-33	-0.363588559	0.119	0.238	5.11E-29	1.1
RFX3-AS1	2.15E-33	-0.28683106	0.018	0.092	5.19E-29	1.1
RPS6KA3	2.50E-33	0.60377484	0.416	0.319	6.03E-29	1.1
TOP2B	2.64E-33	0.433248777	0.251	0.151	6.36E-29	1.1
PTGR1	3.09E-33	-0.320330939	0.163	0.295	7.46E-29	1.1
UBA52	3.53E-33	0.248480152	0.957	0.955	8.52E-29	1.1
ST3GAL6	3.96E-33	-0.314493381	0.05	0.144	9.54E-29	1.1
NOP10	4.09E-33	-0.32428838	0.355	0.512	9.86E-29	1.1
RPL7	4.12E-33	0.417136652	0.971	0.956	9.94E-29	1.1
CH17-189H	4.17E-33	-0.333176255	0.06	0.157	1.00E-28	1.1
FAM208A	4.27E-33	-0.228104024	0.017	0.091	1.03E-28	1.1
PRR14L	4.49E-33	-0.26970365	0.028	0.11	1.08E-28	1.1
UBE2E2	4.55E-33	-0.343866269	0.287	0.443	1.10E-28	1.1
WNT2B	4.88E-33	-0.266961186	0.008	0.075	1.18E-28	1.1
HTATIP2	4.89E-33	-0.232712298	0.039	0.127	1.18E-28	1.1
SEL1L3	4.97E-33	-0.244481036	0.008	0.073	1.20E-28	1.1
ADRBK2	5.41E-33	-0.29003539	0.04	0.128	1.30E-28	1.1
RALGAPA1	5.66E-33	-0.337446306	0.144	0.27	1.36E-28	1.1
CTSC	5.71E-33	-0.240998649	0.008	0.073	1.38E-28	1.1
FAM134B	5.71E-33	-0.382008179	0.075	0.178	1.38E-28	1.1
RPS6KA5	6.44E-33	-0.308831513	0.121	0.241	1.55E-28	1.1
CDC26	6.73E-33	-0.276554538	0.131	0.254	1.62E-28	1.1
EIF3H	6.90E-33	0.3673732	0.719	0.691	1.66E-28	1.1
SMC6	7.20E-33	-0.252754958	0.02	0.096	1.74E-28	1.1
NPAS3	7.71E-33	-0.33158413	0.083	0.191	1.86E-28	1.1
TRIM38	8.95E-33	-0.280777719	0.041	0.128	2.16E-28	1.1
SH3PXD2B	9.10E-33	-0.272245128	0.06	0.158	2.19E-28	1.1
TXNDC9	9.46E-33	-0.292966714	0.072	0.175	2.28E-28	1.1
CEBPZOS	9.53E-33	-0.250168161	0.068	0.17	2.30E-28	1.1
LRP10	1.03E-32	-0.267003483	0.044	0.134	2.48E-28	1.1
ABHD3	1.15E-32	-0.253182531	0.068	0.17	2.77E-28	1.1
IFFO2	1.30E-32	0.414642683	0.213	0.117	3.14E-28	1.1
KREMEN1	1.34E-32	-0.230012795	0.019	0.094	3.23E-28	1.1
DHRS7B	1.39E-32	-0.245464714	0.015	0.086	3.35E-28	1.1
PDCD4	1.41E-32	-0.36050485	0.134	0.257	3.39E-28	1.1
GXYLT2	1.46E-32	-0.281256738	0.028	0.108	3.52E-28	1.1
PITPNC1	1.69E-32	-0.484264242	0.129	0.245	4.07E-28	1.1
TYW5	1.69E-32	-0.233262732	0.014	0.084	4.08E-28	1.1
NCOA1	1.70E-32	-0.353310587	0.291	0.443	4.10E-28	1.1
HUWE1	2.02E-32	-0.276684534	0.227	0.376	4.87E-28	1.1
FGGY	2.23E-32	-0.322817506	0.139	0.264	5.38E-28	1.1
HIST1H2BB	2.29E-32	-0.371147641	0.065	0.163	5.52E-28	1.1
ADNP	2.45E-32	-0.323950215	0.209	0.351	5.92E-28	1.1
IDH1	2.70E-32	-0.212555167	0.018	0.09	6.52E-28	1.1

COX7C	2.74E-32	0.29771071	0.905	0.881	6.60E-28	1.1
PADI2	2.77E-32	-0.390524855	0.1	0.209	6.69E-28	1.1
BTBD11	3.13E-32	-0.300766165	0.021	0.095	7.56E-28	1.1
IGF1R	3.27E-32	-0.364688104	0.299	0.455	7.88E-28	1.1
TTC39B	3.36E-32	-0.266922725	0.04	0.126	8.11E-28	1.1
CHCHD3	3.41E-32	0.278625182	0.897	0.878	8.22E-28	1.1
UQCRC2	4.00E-32	0.454631195	0.505	0.428	9.64E-28	1.1
PARN	4.78E-32	-0.259635476	0.027	0.105	1.15E-27	1.1
YARS	4.96E-32	0.495621918	0.356	0.253	1.20E-27	1.1
LINC01191	5.02E-32	-0.226063753	0.009	0.074	1.21E-27	1.1
MRPL33	5.05E-32	-0.284321486	0.311	0.472	1.22E-27	1.1
SC5D	5.26E-32	-0.25780017	0.032	0.114	1.27E-27	1.1
ZFP14	5.61E-32	-0.226386374	0.018	0.091	1.35E-27	1.1
MSH3	5.65E-32	-0.306470425	0.041	0.128	1.36E-27	1.1
MAMDC2	5.70E-32	0.312438916	0.944	0.938	1.37E-27	1.1
KATNBL1	6.89E-32	-0.285887722	0.072	0.172	1.66E-27	1.1
NEBL	7.07E-32	-0.315977945	0.386	0.544	1.71E-27	1.1
ECHDC1	7.12E-32	-0.286138404	0.178	0.31	1.72E-27	1.1
GUCY1A3	7.65E-32	-0.355185108	0.141	0.263	1.85E-27	1.1
WWOX	7.95E-32	-0.629754	0.14	0.254	1.92E-27	1.1
ATM	8.04E-32	-0.266296123	0.02	0.093	1.94E-27	1.1
KCTD7	8.29E-32	-0.306464669	0.059	0.153	2.00E-27	1.1
RPL19	8.45E-32	0.402507411	0.935	0.939	2.04E-27	1.1
KCNQ1	8.72E-32	-0.291358296	0.035	0.119	2.10E-27	1.1
SNX14	9.04E-32	-0.237983272	0.032	0.113	2.18E-27	1.1
TBC1D8	9.16E-32	0.529822765	0.474	0.381	2.21E-27	1.1
ZNRF2	1.00E-31	-0.289611903	0.082	0.187	2.42E-27	1.1
SGMS1	1.21E-31	-0.246848772	0.172	0.311	2.91E-27	1.1
ZNF37A	1.22E-31	-0.270813947	0.033	0.113	2.94E-27	1.1
TNS3	1.24E-31	-0.305720208	0.025	0.101	3.00E-27	1.1
ZNF718	1.25E-31	-0.225427489	0.011	0.077	3.02E-27	1.1
SMURF1	1.28E-31	-0.25918321	0.167	0.3	3.08E-27	1.1
AGO4	1.69E-31	-0.24951536	0.042	0.128	4.07E-27	1.1
RP11-711K	1.73E-31	-0.276102966	0.012	0.079	4.17E-27	1.1
YWHAB	1.98E-31	-0.298056279	0.182	0.315	4.76E-27	1.1
HLA-DRA	2.16E-31	0.636905883	0.225	0.132	5.22E-27	1.1
SPX	2.34E-31	-0.212327501	0.003	0.061	5.64E-27	1.1
KRT7	2.43E-31	0.466846146	0.357	0.249	5.85E-27	1.1
ZMYM4	2.52E-31	0.315435103	0.893	0.899	6.07E-27	1.1
H1F0	2.78E-31	-0.324119441	0.094	0.199	6.70E-27	1.1
FAM20A	2.87E-31	-0.262383234	0.018	0.089	6.91E-27	1.1
IKBKB	2.91E-31	-0.280841008	0.05	0.141	7.01E-27	1.1
ADARB1	2.96E-31	-0.221606195	0.01	0.075	7.14E-27	1.1
RBM39	2.97E-31	0.401962363	0.523	0.437	7.15E-27	1.1
FBN1	3.00E-31	-0.275145235	0.005	0.066	7.24E-27	1.1
10-Sep	3.11E-31	-0.282937362	0.076	0.176	7.49E-27	1.1
LINC01183	3.28E-31	-0.26049508	0.012	0.077	7.91E-27	1.1

NR2F2	3.38E-31	-0.22154601	0.034	0.115	8.16E-27	1.1
MBOAT1	3.39E-31	0.35164907	0.118	0.05	8.18E-27	1.1
GRIP1	3.69E-31	-0.298673508	0.115	0.23	8.89E-27	1.1
ADD3	4.49E-31	-0.315118084	0.084	0.186	1.08E-26	1.1
C2orf68	4.55E-31	-0.212813805	0.015	0.083	1.10E-26	1.1
MMADHC	4.95E-31	-0.300226566	0.268	0.414	1.19E-26	1.1
MIR4435-2	5.03E-31	0.55247666	0.544	0.464	1.21E-26	1.1
PARP8	5.27E-31	-0.310554423	0.057	0.15	1.27E-26	1.1
CSGALNAC	5.90E-31	-0.312141681	0.032	0.111	1.42E-26	1.1
SERPINE2	5.96E-31	-0.251078151	0.008	0.069	1.44E-26	1.1
ZDHHC13	6.05E-31	-0.253764327	0.043	0.128	1.46E-26	1.1
COL27A1	6.28E-31	-0.371183335	0.038	0.121	1.52E-26	1.1
UXS1	6.29E-31	-0.293340446	0.037	0.119	1.52E-26	1.1
SCGB2B2	6.60E-31	-0.231071384	0.012	0.077	1.59E-26	1.1
CSNK1E	7.29E-31	-0.317493128	0.165	0.294	1.76E-26	1.1
CATSPER2	9.45E-31	-0.236574532	0.03	0.108	2.28E-26	1.1
CD63	9.68E-31	-0.397165855	0.131	0.245	2.33E-26	1.1
STAT5B	1.00E-30	-0.332095209	0.105	0.214	2.42E-26	1.1
MRPS18C	1.03E-30	-0.235771354	0.108	0.222	2.47E-26	1.1
PHIP	1.04E-30	-0.300723085	0.307	0.462	2.51E-26	1.1
PXDN	1.12E-30	-0.249921585	0.051	0.141	2.70E-26	1.1
LINC01137	1.14E-30	-0.227577094	0.012	0.077	2.74E-26	1.1
PAWR	1.20E-30	0.530439485	0.529	0.463	2.91E-26	1.1
ZFAND6	1.25E-30	-0.274386198	0.257	0.407	3.01E-26	1.1
TCEB1	1.30E-30	-0.329821456	0.196	0.331	3.13E-26	1.1
RNF170	1.30E-30	-0.207378359	0.01	0.073	3.14E-26	1.1
OPHN1	1.38E-30	-0.317312805	0.136	0.256	3.33E-26	1.1
PSEN1	1.41E-30	-0.291561868	0.13	0.248	3.39E-26	1.1
SCNN1A	1.42E-30	-0.244655427	0.023	0.096	3.43E-26	1.1
UBE4B	1.77E-30	-0.259017223	0.125	0.244	4.27E-26	1.1
SEC22A	1.80E-30	-0.299269768	0.068	0.165	4.35E-26	1.1
PRRG4	1.83E-30	-0.290311493	0.118	0.229	4.40E-26	1.1
TMEM57	1.85E-30	0.448089007	0.24	0.147	4.45E-26	1.1
ZBTB1	1.89E-30	-0.25087922	0.032	0.11	4.56E-26	1.1
SOSTDC1	1.91E-30	0.230664722	0.053	0.012	4.61E-26	1.1
MICAL2	1.94E-30	-0.261974341	0.015	0.082	4.68E-26	1.1
SGPL1	2.04E-30	-0.245277903	0.053	0.143	4.91E-26	1.1
MCC	2.04E-30	-0.297220681	0.05	0.137	4.92E-26	1.1
KPNA3	2.04E-30	-0.269046671	0.048	0.136	4.93E-26	1.1
SPINT1	2.06E-30	-0.284024308	0.057	0.148	4.96E-26	1.1
CEP192	3.31E-30	-0.247898118	0.034	0.113	7.99E-26	1.1
DHX32	3.41E-30	-0.30782162	0.133	0.25	8.23E-26	1.1
ZNF791	3.50E-30	-0.25359242	0.048	0.135	8.45E-26	1.1
TRIM13	3.54E-30	-0.200803858	0.03	0.106	8.53E-26	1.1
ESRRA	3.56E-30	-0.208582089	0.017	0.085	8.57E-26	1.1
DPYD-AS1	3.91E-30	-0.284533919	0.033	0.112	9.42E-26	1.1
PSMB1	4.02E-30	-0.311033096	0.351	0.502	9.69E-26	1.1

CACHD1	4.20E-30	-0.263190183	0.033	0.111	1.01E-25	1.1
ZNF431	4.27E-30	-0.252169662	0.065	0.159	1.03E-25	1.1
SLC39A14	4.37E-30	-0.327934605	0.241	0.381	1.05E-25	1.1
MNAT1	4.90E-30	-0.304382029	0.224	0.361	1.18E-25	1.1
LPP	5.18E-30	-0.298861575	0.827	0.911	1.25E-25	1.1
EIF2S2	5.75E-30	-0.341941833	0.183	0.307	1.39E-25	1.1
ZNF721	6.29E-30	-0.304206904	0.13	0.246	1.52E-25	1.1
HSD17B4	6.75E-30	-0.298247898	0.085	0.188	1.63E-25	1.1
ZNF69	7.93E-30	-0.210980156	0.011	0.074	1.91E-25	1.1
HSP90AA1	7.98E-30	0.30446593	0.917	0.916	1.92E-25	1.1
SNX29	8.05E-30	-0.313807154	0.044	0.128	1.94E-25	1.1
CDC27	9.32E-30	0.543739746	0.31	0.216	2.25E-25	1.1
ZNF766	9.44E-30	-0.217382878	0.026	0.101	2.28E-25	1.1
PPP1CB	1.04E-29	0.496487367	0.478	0.401	2.50E-25	1.1
NRG2	1.06E-29	-0.278842045	0.013	0.077	2.56E-25	1.1
SGK1	1.11E-29	-0.278497856	0.099	0.206	2.67E-25	1.1
6-Mar	1.16E-29	0.480349819	0.409	0.32	2.81E-25	1.1
FAM160A1	1.24E-29	0.485442243	0.715	0.695	2.99E-25	1.1
BBIP1	1.38E-29	-0.227141609	0.044	0.128	3.33E-25	1.1
UBD	1.45E-29	-0.809028103	0.095	0.196	3.49E-25	1.1
TUFT1	1.45E-29	0.386212465	0.216	0.126	3.50E-25	1.1
PHC3	1.54E-29	-0.274162157	0.105	0.212	3.71E-25	1.1
ZFHX3	1.55E-29	-0.299206623	0.053	0.139	3.73E-25	1.1
TLR5	1.56E-29	-0.197244682	0.007	0.066	3.76E-25	1.1
PARK2	1.69E-29	-0.331867046	0.091	0.193	4.08E-25	1.1
ZNF24	1.74E-29	-0.258688688	0.145	0.264	4.20E-25	1.1
GBAS	1.77E-29	-0.25500191	0.11	0.221	4.26E-25	1.1
MRPL27	1.89E-29	-0.252264175	0.094	0.199	4.56E-25	1.1
ACACA	1.91E-29	-0.272523085	0.081	0.181	4.60E-25	1.1
TOP1	2.08E-29	0.497289613	0.408	0.319	5.02E-25	1.1
TMEM245	2.09E-29	-0.244142598	0.039	0.12	5.04E-25	1.1
SPRED2	2.34E-29	-0.261478394	0.053	0.141	5.65E-25	1.1
USP32	2.44E-29	-0.293675007	0.118	0.23	5.88E-25	1.1
TSFM	2.70E-29	-0.218177068	0.01	0.07	6.52E-25	1.1
FERMT2	2.72E-29	0.440312418	0.228	0.138	6.57E-25	1.1
RPL10A	2.77E-29	0.410991627	0.796	0.782	6.69E-25	1.1
VPS45	3.08E-29	-0.224505404	0.032	0.109	7.42E-25	1.1
ZNF274	3.09E-29	-0.234887568	0.03	0.106	7.45E-25	1.1
PSMA4	3.17E-29	-0.425703908	0.398	0.539	7.66E-25	1.1
MBD2	3.43E-29	0.54446471	0.35	0.26	8.27E-25	1.1
DTX2	3.58E-29	-0.274454137	0.043	0.125	8.64E-25	1.1
PTK2B	3.59E-29	-0.236482571	0.012	0.075	8.65E-25	1.1
MGA	3.79E-29	-0.24250988	0.02	0.088	9.14E-25	1.1
SRD5A3	4.06E-29	-0.196809168	0.01	0.072	9.80E-25	1.1
CAP2	4.20E-29	-0.284516099	0.039	0.118	1.01E-24	1.1
NEO1	4.35E-29	-0.245967405	0.03	0.105	1.05E-24	1.1
MRPL50	4.35E-29	-0.240175309	0.067	0.161	1.05E-24	1.1

KDM3B	5.41E-29	-0.29357183	0.1	0.205	1.31E-24	1.1
SPATA5	5.53E-29	-0.307405644	0.067	0.159	1.33E-24	1.1
STK3	5.56E-29	-0.211747497	0.259	0.415	1.34E-24	1.1
RBM41	5.67E-29	-0.227448483	0.032	0.108	1.37E-24	1.1
MYO5C	6.04E-29	-0.28912309	0.046	0.127	1.46E-24	1.1
PRKRIP1	6.62E-29	-0.253408509	0.071	0.166	1.60E-24	1.1
YWHAE	7.25E-29	0.390983187	0.652	0.606	1.75E-24	1.1
RP1-167A1	8.59E-29	-0.22859559	0.012	0.073	2.07E-24	1.1
PLIN2	8.97E-29	0.589228892	0.216	0.13	2.16E-24	1.1
PDCD10	9.00E-29	-0.319695586	0.193	0.321	2.17E-24	1.1
POC1B	9.67E-29	-0.241503047	0.039	0.119	2.33E-24	1.1
EPS8	1.04E-28	-0.231477193	0.326	0.493	2.51E-24	1.1
RPL41	1.07E-28	0.301067501	0.964	0.95	2.58E-24	1.1
RP5-945F2.	1.08E-28	0.602835085	0.128	0.059	2.60E-24	1.1
ASCC3	1.08E-28	-0.31506503	0.128	0.238	2.61E-24	1.1
STARD13	1.17E-28	-0.28057964	0.121	0.232	2.82E-24	1.1
HOOK2	1.20E-28	-0.289485517	0.09	0.19	2.88E-24	1.1
TRAF3IP2	1.23E-28	-0.303073659	0.099	0.202	2.96E-24	1.1
RP11-481C	1.24E-28	0.313560455	0.11	0.046	2.99E-24	1.1
NUTM2B-A	1.26E-28	-0.257200523	0.2	0.333	3.03E-24	1.1
ANGPT2	1.26E-28	-0.194319062	0.005	0.06	3.03E-24	1.1
URM1	1.29E-28	-0.217660828	0.02	0.087	3.12E-24	1.1
C1QTNF1	1.45E-28	-0.206526054	0.005	0.06	3.51E-24	1.1
DIS3L2	1.46E-28	-0.272941954	0.045	0.127	3.52E-24	1.1
SCD	1.46E-28	-0.225754321	0.03	0.105	3.53E-24	1.1
INO80	1.48E-28	-0.254261036	0.09	0.19	3.58E-24	1.1
AC016831.	1.49E-28	-0.306542826	0.164	0.289	3.59E-24	1.1
7-Mar	1.62E-28	0.464069932	0.35	0.257	3.90E-24	1.1
ATG14	1.62E-28	0.353034405	0.155	0.08	3.90E-24	1.1
RPS25	1.65E-28	0.369519357	0.862	0.867	3.97E-24	1.1
FANCL	1.87E-28	-0.227879584	0.028	0.1	4.50E-24	1.1
LINC00894	1.90E-28	-0.215185901	0.016	0.081	4.57E-24	1.1
XRRA1	1.91E-28	-0.247699723	0.05	0.134	4.62E-24	1.1
ATG5	1.95E-28	-0.261902888	0.05	0.133	4.70E-24	1.1
KDSR	1.96E-28	-0.243107922	0.048	0.13	4.72E-24	1.1
IWS1	2.19E-28	-0.286821028	0.08	0.175	5.29E-24	1.1
SLC5A6	2.22E-28	-0.274818998	0.066	0.157	5.35E-24	1.1
ZNF680	2.30E-28	-0.236184686	0.039	0.118	5.55E-24	1.1
NDUFA4	2.34E-28	0.341347552	0.807	0.799	5.64E-24	1.1
CCDC174	3.09E-28	-0.259615772	0.107	0.212	7.45E-24	1.1
ZNF586	3.10E-28	-0.246042604	0.042	0.121	7.48E-24	1.1
KCCAT211	3.11E-28	-0.289004716	0.011	0.071	7.50E-24	1.1
ACBD6	3.18E-28	-0.262183239	0.021	0.089	7.68E-24	1.1
ZBTB44	3.24E-28	-0.261331583	0.046	0.128	7.82E-24	1.1
H2AFZ	3.47E-28	-0.374611006	0.523	0.648	8.37E-24	1.1
GTF2IRD2B	3.53E-28	-0.227818032	0.031	0.105	8.50E-24	1.1
NRG1	3.55E-28	-0.357825448	0.061	0.15	8.56E-24	1.1

JKAMP	3.73E-28	-0.231187425	0.03	0.102	8.99E-24	1.1
ARPC3	3.74E-28	-0.33681907	0.58	0.699	9.02E-24	1.1
SPPL3	3.78E-28	-0.278617472	0.162	0.283	9.11E-24	1.1
ZNF260	3.82E-28	-0.192049412	0.035	0.111	9.20E-24	1.1
NMI	3.82E-28	-0.253844168	0.06	0.147	9.22E-24	1.1
PUS10	3.82E-28	-0.225865769	0.02	0.087	9.22E-24	1.1
ZNF98	4.12E-28	-0.263886015	0.035	0.111	9.94E-24	1.1
UBAP2	4.14E-28	-0.280302641	0.11	0.216	9.99E-24	1.1
PBRM1	4.24E-28	-0.253592573	0.117	0.226	1.02E-23	1.1
OSBPL1A	4.33E-28	-0.281344508	0.079	0.174	1.04E-23	1.1
SYNE2	4.34E-28	-0.285669702	0.397	0.551	1.05E-23	1.1
VWA5A	4.44E-28	-0.215719151	0.048	0.131	1.07E-23	1.1
ALG13	4.46E-28	-0.225867538	0.042	0.122	1.07E-23	1.1
CCL4	4.54E-28	-2.447361294	0.034	0.106	1.09E-23	1.1
FOXN2	4.68E-28	-0.296113121	0.044	0.123	1.13E-23	1.1
MTIF3	4.74E-28	-0.2743893	0.155	0.275	1.14E-23	1.1
GOSR1	4.92E-28	-0.239799366	0.096	0.199	1.19E-23	1.1
NEMF	5.11E-28	-0.25217451	0.222	0.359	1.23E-23	1.1
TRMT10B	5.29E-28	-0.225064219	0.051	0.136	1.28E-23	1.1
IKBKE	5.44E-28	-0.197412837	0.003	0.056	1.31E-23	1.1
MYO3B	5.77E-28	-0.374648227	0.126	0.235	1.39E-23	1.1
USP6NL	5.81E-28	0.464102831	0.276	0.182	1.40E-23	1.1
DDR2	5.99E-28	-0.314040646	0.055	0.14	1.44E-23	1.1
TEAD2	7.01E-28	-0.198279747	0.023	0.091	1.69E-23	1.1
ERO1A	7.08E-28	0.63133815	0.409	0.331	1.71E-23	1.1
ERN1	7.79E-28	-0.264580732	0.073	0.166	1.88E-23	1.1
DAZAP2	7.85E-28	-0.24649084	0.269	0.413	1.89E-23	1.1
MARK1	8.53E-28	-0.250729196	0.032	0.105	2.06E-23	1.1
RNF175	8.75E-28	-0.246048597	0.008	0.065	2.11E-23	1.1
THAP6	9.33E-28	-0.184228512	0.022	0.09	2.25E-23	1.1
NAA38	9.83E-28	-0.220291481	0.016	0.08	2.37E-23	1.1
RAB18	1.01E-27	0.449203695	0.353	0.262	2.43E-23	1.1
RP11-66B2	1.02E-27	-0.2926594	0.03	0.101	2.47E-23	1.1
CP	1.17E-27	-0.282036487	0.02	0.086	2.82E-23	1.1
CDCP1	1.18E-27	-0.248542408	0.265	0.417	2.84E-23	1.1
SSR3	1.29E-27	0.42898748	0.629	0.579	3.11E-23	1.1
NSUN4	1.32E-27	-0.165615771	0.028	0.098	3.18E-23	1.1
SHOC2	1.36E-27	-0.259024854	0.136	0.25	3.27E-23	1.1
AL109761.!	1.39E-27	-0.173882762	0.004	0.057	3.35E-23	1.1
SPAG9	1.44E-27	0.511275726	0.403	0.316	3.46E-23	1.1
LLPH	1.51E-27	-0.234998732	0.068	0.159	3.64E-23	1.1
TYW1B	1.57E-27	-0.274779412	0.081	0.177	3.78E-23	1.1
TMCO1	1.69E-27	-0.249513954	0.228	0.366	4.07E-23	1.1
RIC1	1.70E-27	-0.26375538	0.049	0.131	4.11E-23	1.1
SMG1	1.75E-27	0.509890403	0.324	0.235	4.22E-23	1.1
C21orf91-C	1.77E-27	-0.185255311	0.003	0.053	4.26E-23	1.1
COX7B	1.81E-27	0.349768024	0.682	0.648	4.37E-23	1.1

ATP11A	1.83E-27	-0.23735203	0.028	0.099	4.40E-23	1.1
ITGB4	1.83E-27	-0.252460289	0.01	0.069	4.41E-23	1.1
NUP214	1.83E-27	-0.223671874	0.027	0.096	4.42E-23	1.1
TMBIM6	2.00E-27	-0.41786464	0.738	0.822	4.83E-23	1.1
UBXN4	2.09E-27	-0.272333778	0.188	0.312	5.04E-23	1.1
PPP1R13B	2.12E-27	-0.276940204	0.068	0.157	5.11E-23	1.1
NSRP1	2.33E-27	-0.256348533	0.183	0.308	5.61E-23	1.1
CDC42SE1	2.34E-27	-0.263264282	0.085	0.181	5.65E-23	1.1
TNFRSF11E	2.50E-27	-0.356837017	0.047	0.126	6.04E-23	1.1
EMP1	2.67E-27	0.817672923	0.696	0.727	6.44E-23	1.1
ALOX12-AS	2.71E-27	-0.257640295	0.021	0.087	6.53E-23	1.1
FLOT2	2.95E-27	-0.219144701	0.013	0.073	7.11E-23	1.1
SNRPG	3.14E-27	-0.302802686	0.432	0.585	7.58E-23	1.1
SCAF4	3.34E-27	0.299127018	0.133	0.064	8.05E-23	1.1
UEVLD	3.34E-27	-0.201461679	0.017	0.08	8.05E-23	1.1
RP11-577H	3.49E-27	-0.190785017	0.021	0.087	8.42E-23	1.1
ERCC6L2	3.49E-27	-0.23855246	0.035	0.108	8.42E-23	1.1
CCNB1IP1	3.50E-27	0.42131508	0.431	0.346	8.44E-23	1.1
TRNAU1AP	3.52E-27	-0.253241006	0.183	0.306	8.48E-23	1.1
SNX10	3.64E-27	-0.213302227	0.011	0.069	8.79E-23	1.1
ZNF407	3.78E-27	-0.253427842	0.053	0.136	9.13E-23	1.1
NEURL3	3.79E-27	-0.243518452	0.016	0.078	9.14E-23	1.1
SS18L2	3.88E-27	-0.213159625	0.073	0.164	9.36E-23	1.1
HERC2	4.07E-27	-0.249368923	0.031	0.102	9.81E-23	1.1
VPS36	4.14E-27	-0.251070989	0.049	0.13	9.99E-23	1.1
ABHD17C	4.15E-27	-0.233138397	0.018	0.081	1.00E-22	1.1
HRSP12	4.17E-27	-0.221930689	0.035	0.108	1.01E-22	1.1
ARL4A	4.28E-27	0.419528628	0.194	0.111	1.03E-22	1.1
EXOC6	4.51E-27	-0.251914194	0.028	0.098	1.09E-22	1.1
PLOD2	4.64E-27	-0.325084492	0.089	0.185	1.12E-22	1.1
EIF2AK2	4.65E-27	-0.241785332	0.048	0.128	1.12E-22	1.1
DCLRE1C	4.67E-27	-0.200474394	0.023	0.09	1.13E-22	1.1
RP11-66B2	4.71E-27	-0.265874161	0.045	0.122	1.14E-22	1.1
GLIS3	4.83E-27	-0.236985904	0.189	0.322	1.17E-22	1.1
ZNF22	4.83E-27	-0.255514648	0.151	0.265	1.17E-22	1.1
TRIM44	5.31E-27	-0.235460538	0.08	0.173	1.28E-22	1.1
ADAM32	5.56E-27	-0.29326102	0.061	0.147	1.34E-22	1.1
WDR48	5.74E-27	0.410686159	0.251	0.163	1.39E-22	1.1
ATAD2B	5.90E-27	-0.261494663	0.083	0.177	1.42E-22	1.1
BPGM	6.22E-27	-0.263894014	0.093	0.191	1.50E-22	1.1
MSI2	6.82E-27	-0.305601152	0.12	0.226	1.65E-22	1.1
TTC28	7.47E-27	-0.283230311	0.039	0.114	1.80E-22	1.1
TMEM33	7.56E-27	-0.214490241	0.037	0.111	1.82E-22	1.1
EVL	7.95E-27	-0.245538204	0.016	0.078	1.92E-22	1.1
GABRE	8.01E-27	-0.201199303	0.018	0.08	1.93E-22	1.1
SLC38A9	8.57E-27	-0.224862401	0.016	0.077	2.07E-22	1.1
PRKDC	8.61E-27	-0.249192009	0.053	0.136	2.08E-22	1.1

ZNF131	8.67E-27	-0.276776984	0.075	0.165	2.09E-22	1.1
MDM4	9.04E-27	-0.311618483	0.184	0.303	2.18E-22	1.1
ATP6V1B2	9.67E-27	-0.28655132	0.083	0.176	2.33E-22	1.1
FCHO2	9.83E-27	-0.252148906	0.081	0.174	2.37E-22	1.1
MRPS14	1.02E-26	-0.218161745	0.088	0.185	2.47E-22	1.1
C5orf17	1.03E-26	-0.279772989	0.019	0.082	2.48E-22	1.1
MFAP1	1.07E-26	-0.222433818	0.088	0.184	2.58E-22	1.1
TTC9C	1.09E-26	-0.239378142	0.081	0.174	2.63E-22	1.1
MORN2	1.17E-26	0.242349869	0.095	0.038	2.82E-22	1.1
SLC18B1	1.18E-26	-0.273306695	0.122	0.23	2.86E-22	1.1
ATP5E	1.21E-26	-0.296222753	0.359	0.496	2.91E-22	1.1
TM4SF18	1.26E-26	-0.31312749	0.035	0.106	3.05E-22	1.1
MAP2K6	1.33E-26	0.19886971	0.064	0.02	3.20E-22	1.1
ARHGAP5	1.34E-26	-0.260381715	0.286	0.426	3.23E-22	1.1
TLN2	1.35E-26	-0.297457151	0.047	0.125	3.26E-22	1.1
TPRG1	1.43E-26	-0.273742127	0.038	0.113	3.44E-22	1.1
ODF2L	1.44E-26	-0.294926357	0.076	0.165	3.47E-22	1.1
PPARA	1.47E-26	-0.221012396	0.028	0.097	3.54E-22	1.1
LLGL2	1.49E-26	-0.219148854	0.029	0.099	3.60E-22	1.1
KLHL20	1.51E-26	-0.222251853	0.025	0.092	3.64E-22	1.1
LSAMP	1.58E-26	-0.284786055	0.163	0.28	3.80E-22	1.1
ACAP2	1.60E-26	-0.26027475	0.085	0.179	3.85E-22	1.1
RHBDF2	1.66E-26	-0.220349638	0.02	0.084	4.00E-22	1.1
DLG2	1.69E-26	-0.33364017	0.095	0.193	4.08E-22	1.1
ACTR3C	1.88E-26	-0.19912307	0.009	0.064	4.52E-22	1.1
IDE	2.03E-26	-0.215738549	0.027	0.094	4.90E-22	1.1
ACO1	2.46E-26	-0.198608309	0.015	0.074	5.93E-22	1.1
ATG10	2.47E-26	-0.292146969	0.075	0.164	5.96E-22	1.1
EIF3M	2.50E-26	0.414690542	0.526	0.473	6.03E-22	1.1
WDPCP	2.55E-26	-0.278694244	0.082	0.175	6.16E-22	1.1
AC008074.	2.75E-26	-0.221646525	0.03	0.1	6.64E-22	1.1
PDE1C	2.82E-26	-0.292211078	0.03	0.099	6.80E-22	1.1
KLHL2	2.94E-26	-0.247184785	0.02	0.084	7.10E-22	1.1
GRB14	3.06E-26	0.441632462	0.613	0.544	7.38E-22	1.1
HNRNPD	3.11E-26	0.42226845	0.354	0.266	7.50E-22	1.1
NLGN4X	3.29E-26	-0.248256164	0.028	0.097	7.92E-22	1.1
EPC2	3.33E-26	-0.194515641	0.018	0.081	8.03E-22	1.1
TAOK1	3.34E-26	-0.268222334	0.168	0.285	8.05E-22	1.1
EPB42	3.54E-26	0.27548417	0.961	0.915	8.53E-22	1.1
PGK1	3.56E-26	-0.379722317	0.564	0.683	8.58E-22	1.1
DOCK9	3.67E-26	-0.233824044	0.096	0.194	8.86E-22	1.1
HLCS	3.94E-26	-0.251634816	0.034	0.104	9.50E-22	1.1
KHDRBS1	4.19E-26	0.424893604	0.36	0.27	1.01E-21	1.1
CTDSP2	4.30E-26	-0.24444026	0.114	0.216	1.04E-21	1.1
USP13	4.38E-26	-0.230471256	0.038	0.111	1.06E-21	1.1
CNDP2	4.45E-26	-0.258146027	0.046	0.123	1.07E-21	1.1
NACA2	4.52E-26	0.417142642	0.662	0.628	1.09E-21	1.1

PM20D2	4.93E-26	-0.244135267	0.071	0.16	1.19E-21	1.1
MFSD14C	5.03E-26	-0.252242233	0.137	0.247	1.21E-21	1.1
RP11-631N	5.06E-26	-0.223858978	0.025	0.091	1.22E-21	1.1
ZNF480	5.08E-26	-0.184182606	0.011	0.067	1.23E-21	1.1
SMARCA1	5.33E-26	-0.276595441	0.08	0.169	1.29E-21	1.1
RAB10	5.67E-26	-0.2615679	0.189	0.31	1.37E-21	1.1
FBXL5	5.78E-26	-0.228176389	0.066	0.152	1.39E-21	1.1
HDAC7	6.00E-26	-0.282517079	0.068	0.154	1.45E-21	1.1
EXOC2	6.00E-26	-0.198599513	0.025	0.091	1.45E-21	1.1
FUT8	6.07E-26	-0.264849921	0.039	0.112	1.46E-21	1.1
MAP2K4	6.26E-26	-0.321110208	0.271	0.402	1.51E-21	1.1
SQLE	6.49E-26	-0.172529815	0.013	0.071	1.56E-21	1.1
CYP27B1	6.81E-26	-0.187431927	0.005	0.056	1.64E-21	1.1
COBLL1	7.11E-26	-0.306167966	0.068	0.152	1.71E-21	1.1
TLDC1	7.25E-26	-0.205785888	0.048	0.127	1.75E-21	1.1
ZNF438	7.32E-26	-0.222740131	0.023	0.087	1.77E-21	1.1
PPP2R3C	7.73E-26	-0.210994205	0.036	0.108	1.86E-21	1.1
FRMD5	7.79E-26	-0.314611457	0.098	0.195	1.88E-21	1.1
RP11-417F:	8.26E-26	-0.246074767	0.057	0.138	1.99E-21	1.1
SQRDL	8.85E-26	-0.267655156	0.11	0.208	2.13E-21	1.1
PIK3IP1	9.03E-26	-0.193047387	0.021	0.083	2.18E-21	1.1
ELF1	9.25E-26	0.441914363	0.499	0.425	2.23E-21	1.1
SRP9	9.50E-26	-0.23985579	0.305	0.446	2.29E-21	1.1
TBC1D3P1-	9.65E-26	0.357094579	0.757	0.749	2.33E-21	1.1
KIAA1671	9.81E-26	-0.29582688	0.109	0.21	2.37E-21	1.1
PALLD	9.82E-26	-0.29186913	0.408	0.553	2.37E-21	1.1
FAM196A	9.94E-26	-0.200763803	0.021	0.085	2.40E-21	1.1
EPB41L1	1.09E-25	-0.283209223	0.048	0.124	2.63E-21	1.1
SECISBP2	1.11E-25	-0.17887551	0.03	0.098	2.67E-21	1.1
GSDMC	1.11E-25	-0.160386352	0.003	0.05	2.67E-21	1.1
CKAP4	1.11E-25	-0.240183738	0.095	0.191	2.69E-21	1.1
CXCL3	1.17E-25	0.517347321	0.143	0.073	2.82E-21	1.1
CBWD7	1.25E-25	-0.20723817	0.026	0.092	3.02E-21	1.1
MAPKAP1	1.36E-25	-0.234359379	0.044	0.119	3.27E-21	1.1
RPS20	1.48E-25	0.365015261	0.962	0.955	3.57E-21	1.1
CHKA	1.48E-25	-0.24558513	0.063	0.147	3.57E-21	1.1
ALDH1A3	1.50E-25	-0.385164806	0.439	0.578	3.62E-21	1.1
H6PD	1.52E-25	-0.190967061	0.01	0.064	3.67E-21	1.1
STX17	1.53E-25	-0.239023646	0.077	0.166	3.68E-21	1.1
FGD6	1.56E-25	-0.315134697	0.17	0.282	3.76E-21	1.1
ARHGAP24	1.56E-25	-0.260584602	0.009	0.063	3.76E-21	1.1
USP40	1.60E-25	-0.215908694	0.023	0.087	3.86E-21	1.1
NFATC2IP	1.71E-25	-0.202742699	0.026	0.091	4.11E-21	1.1
IL22RA2	1.72E-25	0.131854709	0.043	0.01	4.14E-21	1.1
AGO3	1.79E-25	-0.265749227	0.164	0.278	4.32E-21	1.1
NFKBIZ	1.86E-25	0.337433527	0.796	0.797	4.48E-21	1.1
PTPN3	1.87E-25	-0.190417755	0.024	0.089	4.52E-21	1.1

LINC00998	1.88E-25	-0.247900104	0.098	0.194	4.54E-21	1.1
PIM1	2.12E-25	0.178494558	0.057	0.017	5.12E-21	1.1
ITGB1	2.16E-25	0.519735008	0.416	0.335	5.20E-21	1.1
TRAPPC10	2.17E-25	-0.206776947	0.035	0.106	5.24E-21	1.1
TCF20	2.24E-25	-0.23404335	0.053	0.132	5.39E-21	1.1
TMEM51	2.61E-25	-0.235459214	0.103	0.201	6.29E-21	1.1
KCNQ3	2.64E-25	-0.268139029	0.013	0.07	6.37E-21	1.1
CMTM4	2.72E-25	-0.242983595	0.046	0.122	6.56E-21	1.1
MFSD11	2.72E-25	-0.213965493	0.025	0.089	6.56E-21	1.1
AK3	2.76E-25	-0.228147525	0.07	0.155	6.65E-21	1.1
RP1-122P2	2.77E-25	-0.172369058	0.002	0.047	6.68E-21	1.1
TUBB	2.84E-25	-0.318379382	0.258	0.382	6.84E-21	1.1
ITCH	2.88E-25	-0.212190936	0.254	0.391	6.94E-21	1.1
TSC22D1	3.01E-25	-0.323555268	0.173	0.286	7.26E-21	1.1
CYP20A1	3.52E-25	-0.217568118	0.023	0.086	8.48E-21	1.1
MIR222HG	3.58E-25	0.358299555	0.15	0.079	8.64E-21	1.1
CCDC59	3.84E-25	0.364028614	0.244	0.158	9.25E-21	1.1
EMC3	3.95E-25	-0.249862838	0.09	0.181	9.52E-21	1.1
FAM135A	3.96E-25	-0.231768437	0.033	0.101	9.54E-21	1.1
LMBRD1	4.06E-25	0.389671942	0.305	0.215	9.78E-21	1.1
ERC1	4.16E-25	-0.247700418	0.206	0.328	1.00E-20	1.1
TNFRSF1B	4.20E-25	-0.211146773	0.026	0.091	1.01E-20	1.1
CD47	4.33E-25	-0.261158547	0.331	0.475	1.04E-20	1.1
CCDC66	4.35E-25	-0.236593136	0.085	0.176	1.05E-20	1.1
DNAJC1	4.81E-25	-0.275891028	0.13	0.235	1.16E-20	1.1
LYPLA1	5.04E-25	-0.241820349	0.051	0.128	1.21E-20	1.1
TMEM65	5.07E-25	-0.243465124	0.107	0.206	1.22E-20	1.1
ZFYVE9	5.28E-25	-0.243386268	0.053	0.13	1.27E-20	1.1
CNTNAP3	5.40E-25	-0.196573419	0.018	0.078	1.30E-20	1.1
MSRA	5.73E-25	-0.224714197	0.023	0.086	1.38E-20	1.1
FGFBP1	5.75E-25	-0.381423294	0.027	0.092	1.39E-20	1.1
CBWD5	5.93E-25	-0.227932295	0.107	0.206	1.43E-20	1.1
RARS	5.99E-25	-0.240255354	0.197	0.319	1.44E-20	1.1
RNF217	6.12E-25	-0.237354828	0.042	0.114	1.47E-20	1.1
RBFOX2	6.52E-25	0.307990503	0.978	0.959	1.57E-20	1.1
ZDHHC20	6.90E-25	-0.243944917	0.084	0.173	1.66E-20	1.1
TMEM161E	7.75E-25	-0.241535467	0.045	0.119	1.87E-20	1.1
ZFR	7.93E-25	-0.248373166	0.281	0.417	1.91E-20	1.1
CYB5R2	8.45E-25	-0.186542425	0.018	0.077	2.04E-20	1.1
B4GALT6	8.62E-25	-0.17066825	0.005	0.053	2.08E-20	1.1
XPR1	8.68E-25	-0.237314677	0.062	0.144	2.09E-20	1.1
ANXA2	8.79E-25	0.223815242	0.93	0.916	2.12E-20	1.1
SND1	8.92E-25	-0.272582491	0.224	0.353	2.15E-20	1.1
RUSC2	1.00E-24	0.367854557	0.176	0.101	2.42E-20	1.1
IREB2	1.01E-24	-0.210424138	0.027	0.092	2.43E-20	1.1
SLC6A16	1.04E-24	-0.179310137	0.009	0.061	2.52E-20	1.1
HSDL2	1.08E-24	-0.221774527	0.05	0.127	2.60E-20	1.1

MT2A	1.09E-24	-1.152123782	0.028	0.092	2.62E-20	1.1
DENND1B	1.20E-24	-0.260213799	0.07	0.155	2.90E-20	1.1
DDX58	1.21E-24	-0.222793676	0.029	0.095	2.92E-20	1.1
GLTP	1.25E-24	-0.23356107	0.106	0.203	3.00E-20	1.1
CARHSP1	1.29E-24	-0.350673641	0.189	0.303	3.10E-20	1.1
KIAA0556	1.32E-24	-0.214442533	0.036	0.107	3.18E-20	1.1
MRPS6	1.34E-24	-0.333304272	0.118	0.218	3.23E-20	1.1
TUG1	1.37E-24	-0.238357782	0.059	0.139	3.29E-20	1.1
CBFA2T2	1.37E-24	-0.242805767	0.078	0.163	3.30E-20	1.1
RP11-96H1	1.41E-24	-0.299119271	0.056	0.134	3.40E-20	1.1
PHF11	1.48E-24	-0.197629815	0.032	0.099	3.56E-20	1.1
CAAP1	1.54E-24	-0.19565186	0.018	0.077	3.71E-20	1.1
ZNF251	1.58E-24	-0.181870076	0.008	0.058	3.80E-20	1.1
WBP1L	1.75E-24	-0.210594827	0.02	0.079	4.22E-20	1.1
MFSD8	1.79E-24	-0.189294542	0.014	0.069	4.30E-20	1.1
MICU1	1.79E-24	-0.280464405	0.141	0.246	4.31E-20	1.1
TMED3	1.81E-24	-0.221123453	0.025	0.087	4.38E-20	1.1
DCAF10	1.90E-24	-0.225360424	0.114	0.213	4.57E-20	1.1
COMMD10	1.95E-24	-0.27380583	0.135	0.237	4.70E-20	1.1
FAM78B	1.97E-24	-0.261420647	0.027	0.09	4.75E-20	1.1
CSRP1	2.01E-24	0.306790347	0.196	0.116	4.85E-20	1.1
KIZ-AS1	2.01E-24	0.288021301	0.925	0.879	4.85E-20	1.1
ANKRD49	2.07E-24	-0.168589574	0.019	0.078	4.98E-20	1.1
HLA-A	2.10E-24	-0.300755305	0.239	0.363	5.06E-20	1.1
TEFM	2.13E-24	-0.179522424	0.011	0.064	5.13E-20	1.1
IRF2	2.25E-24	-0.251054727	0.209	0.329	5.44E-20	1.1
DDX52	2.29E-24	-0.205192604	0.056	0.134	5.53E-20	1.1
SLC35F5	2.31E-24	-0.247656936	0.047	0.121	5.56E-20	1.1
PDXDC1	2.33E-24	-0.213865445	0.208	0.331	5.61E-20	1.1
SEMA4B	2.37E-24	-0.303442582	0.097	0.188	5.72E-20	1.1
CDC42	2.54E-24	0.333560226	0.722	0.694	6.13E-20	1.1
NARS2	2.67E-24	-0.21924802	0.02	0.079	6.43E-20	1.1
MVP	2.68E-24	-0.20870217	0.023	0.085	6.46E-20	1.1
RP11-68E1	2.81E-24	0.255793802	0.094	0.04	6.77E-20	1.1
AHCYL2	2.81E-24	-0.23812614	0.032	0.099	6.78E-20	1.1
SMC1A	2.84E-24	-0.198183761	0.031	0.097	6.84E-20	1.1
ZYG11B	2.85E-24	-0.208471023	0.028	0.092	6.87E-20	1.1
CARD6	3.16E-24	-0.171171281	0.014	0.069	7.61E-20	1.1
ZEB1	3.22E-24	-0.222708546	0.011	0.063	7.75E-20	1.1
AC013461.	3.26E-24	-0.218945021	0.135	0.238	7.86E-20	1.1
RAD9A	3.31E-24	-0.236769666	0.05	0.126	7.99E-20	1.1
TTF1	3.48E-24	-0.205648108	0.063	0.144	8.38E-20	1.1
SP100	3.48E-24	-0.281180378	0.085	0.172	8.39E-20	1.1
CEP295	3.48E-24	-0.179793323	0.017	0.074	8.40E-20	1.1
SSR2	3.83E-24	0.310687068	0.765	0.764	9.23E-20	1.1
RNMT	4.01E-24	0.473630219	0.426	0.354	9.67E-20	1.1
GPATCH2L	4.18E-24	-0.239639137	0.064	0.144	1.01E-19	1.1

MFN1	4.18E-24	-0.222510031	0.086	0.175	1.01E-19	1.1
NCOA7	4.25E-24	-0.258604558	0.573	0.71	1.02E-19	1.1
PNPLA3	4.88E-24	-0.180830374	0.004	0.05	1.18E-19	1.1
AC005152.	4.99E-24	-0.456665242	0.103	0.193	1.20E-19	1.1
ENOX2	5.14E-24	-0.227721522	0.015	0.071	1.24E-19	1.1
TRIP11	5.30E-24	-0.221879718	0.087	0.176	1.28E-19	1.1
LCN2	5.45E-24	-0.311840256	0.003	0.048	1.32E-19	1.1
PSMA5	5.51E-24	-0.251864734	0.142	0.245	1.33E-19	1.1
DDX3X	5.52E-24	-0.245353337	0.207	0.326	1.33E-19	1.1
ARGLU1	6.34E-24	-0.258795844	0.107	0.202	1.53E-19	1.1
PPP6R2	6.49E-24	-0.22010808	0.05	0.126	1.57E-19	1.1
PARP1	6.51E-24	-0.202771178	0.038	0.107	1.57E-19	1.1
ACTN4	6.69E-24	0.397344941	0.399	0.312	1.61E-19	1.1
RAPH1	6.75E-24	-0.250944252	0.12	0.219	1.63E-19	1.1
CEBPZ	6.81E-24	-0.222789153	0.106	0.202	1.64E-19	1.1
WIPF2	7.30E-24	-0.205112676	0.05	0.125	1.76E-19	1.1
CBFB	8.10E-24	-0.226733228	0.058	0.135	1.95E-19	1.1
RHOQ	8.19E-24	-0.240327643	0.211	0.33	1.98E-19	1.1
LINC00342	8.28E-24	-0.204235024	0.021	0.08	2.00E-19	1.1
PRDX3	9.08E-24	0.34719541	0.209	0.13	2.19E-19	1.1
SYNM	9.51E-24	-0.345645015	0.093	0.182	2.29E-19	1.1
SLC36A4	9.83E-24	-0.200238423	0.025	0.086	2.37E-19	1.1
LINC01235	9.95E-24	-0.274444668	0.124	0.223	2.40E-19	1.1
FAM213A	1.03E-23	-0.195526187	0.031	0.096	2.49E-19	1.1
HP1BP3	1.10E-23	-0.21646513	0.123	0.223	2.64E-19	1.1
AZGP1	1.10E-23	-0.471925682	0.254	0.372	2.65E-19	1.1
YLPM1	1.10E-23	-0.208839339	0.068	0.148	2.66E-19	1.1
HSPG2	1.15E-23	-0.237508579	0.06	0.138	2.76E-19	1.1
SAV1	1.22E-23	-0.266673218	0.283	0.409	2.93E-19	1.1
SLC25A13	1.23E-23	-0.231499289	0.027	0.089	2.96E-19	1.1
MAN1A1	1.26E-23	-0.23905412	0.076	0.161	3.03E-19	1.1
ZNF33B	1.28E-23	-0.281250074	0.116	0.21	3.09E-19	1.1
SMIM8	1.36E-23	-0.20647248	0.039	0.108	3.29E-19	1.1
RPF2	1.39E-23	0.371395685	0.243	0.161	3.35E-19	1.1
CMC1	1.51E-23	-0.179122391	0.012	0.064	3.63E-19	1.1
FBXW11	1.54E-23	-0.196634076	0.233	0.356	3.71E-19	1.1
CSF1	1.57E-23	-0.259174224	0.039	0.107	3.80E-19	1.1
WDR7	1.72E-23	-0.185119914	0.013	0.066	4.15E-19	1.1
RAB12	1.78E-23	-0.232375778	0.124	0.223	4.30E-19	1.1
CCDC122	1.79E-23	-0.180032072	0.011	0.062	4.33E-19	1.1
NCOA6	1.94E-23	-0.245968896	0.089	0.177	4.68E-19	1.1
SETD7	1.96E-23	-0.215307524	0.053	0.127	4.72E-19	1.1
RBL2	2.02E-23	-0.210072106	0.045	0.116	4.87E-19	1.1
CLTA	2.10E-23	-0.216163321	0.093	0.184	5.06E-19	1.1
ANKRD6	2.12E-23	-0.2303936	0.038	0.106	5.12E-19	1.1
ARHGEF11	2.18E-23	-0.18451522	0.012	0.064	5.25E-19	1.1
FZD7	2.22E-23	-0.215382998	0.032	0.096	5.35E-19	1.1

ECHDC2	2.23E-23	-0.231519546	0.06	0.137	5.37E-19	1.1
ZNF567	2.38E-23	-0.161362802	0.016	0.072	5.75E-19	1.1
LINC01344	2.40E-23	-0.19709595	0.007	0.055	5.79E-19	1.1
IFT43	2.42E-23	-0.220814882	0.035	0.102	5.82E-19	1.1
ZCCHC10	2.44E-23	-0.193301222	0.048	0.121	5.87E-19	1.1
HSPA9	2.46E-23	0.386149085	0.272	0.189	5.93E-19	1.1
ARL17A	2.48E-23	-0.190400616	0.021	0.079	5.98E-19	1.1
LINC00475	2.55E-23	-0.219020512	0.036	0.104	6.16E-19	1.1
RP11-608O	2.56E-23	0.312778282	0.747	0.733	6.18E-19	1.1
IGSF3	2.57E-23	-0.177828866	0.03	0.093	6.20E-19	1.1
PGM1	2.63E-23	-0.251100013	0.063	0.142	6.34E-19	1.1
ANKRD13C	2.65E-23	-0.22677111	0.065	0.145	6.39E-19	1.1
ZNF720	2.76E-23	-0.193908534	0.035	0.102	6.66E-19	1.1
SLC30A6	2.82E-23	-0.176979216	0.017	0.073	6.80E-19	1.1
NMT1	3.05E-23	-0.204566556	0.119	0.217	7.35E-19	1.1
DOCK5	3.05E-23	-0.238943035	0.142	0.247	7.35E-19	1.1
ZNF529-AS	3.17E-23	-0.186410007	0.013	0.066	7.64E-19	1.1
ANKS1A	3.25E-23	-0.244884414	0.075	0.157	7.82E-19	1.1
CHORDC1	3.38E-23	-0.204582927	0.045	0.116	8.14E-19	1.1
RAB28	3.40E-23	-0.189235787	0.012	0.064	8.20E-19	1.1
ARL8B	3.51E-23	0.449059954	0.314	0.231	8.48E-19	1.1
FBXW4	3.56E-23	-0.225451459	0.031	0.095	8.57E-19	1.1
DCAF6	3.67E-23	-0.184252946	0.346	0.495	8.84E-19	1.1
RPL7L1	4.07E-23	-0.215453971	0.073	0.154	9.82E-19	1.1
FARP1	4.42E-23	-0.239116776	0.221	0.345	1.07E-18	1.1
CASP4	4.60E-23	-0.256604158	0.206	0.321	1.11E-18	1.1
IFITM2	5.09E-23	-0.180526583	0.01	0.06	1.23E-18	1.1
EIF2B5	5.54E-23	0.481046327	0.595	0.556	1.34E-18	1.1
HMGB1	5.71E-23	-0.268870393	0.445	0.574	1.38E-18	1.1
PSMB5	5.91E-23	-0.234746914	0.155	0.261	1.42E-18	1.1
RP11-286E	6.01E-23	0.281101581	0.103	0.047	1.45E-18	1.1
ZNF141	6.12E-23	-0.195992775	0.021	0.079	1.48E-18	1.1
ARL17B	6.18E-23	-0.273502389	0.031	0.095	1.49E-18	1.1
S100PBP	6.61E-23	-0.236961179	0.058	0.133	1.59E-18	1.1
SPCS2	6.78E-23	0.401357328	0.376	0.294	1.64E-18	1.1
PSPC1	6.87E-23	-0.22332175	0.098	0.189	1.66E-18	1.1
TMEM126E	7.08E-23	-0.1946044	0.064	0.142	1.71E-18	1.1
AIMP1	7.17E-23	0.414871983	0.476	0.402	1.73E-18	1.1
BCL2A1	7.20E-23	-0.489084548	0.095	0.181	1.74E-18	1.1
RRAS2	7.54E-23	-0.195932679	0.148	0.254	1.82E-18	1.1
ZZZ3	7.60E-23	-0.230181044	0.109	0.202	1.83E-18	1.1
NTN4	7.69E-23	-0.323599918	0.111	0.203	1.85E-18	1.1
SEC24A	7.75E-23	0.476285256	0.276	0.197	1.87E-18	1.1
BOC	7.97E-23	-0.185225463	0.006	0.051	1.92E-18	1.1
LRP11	8.00E-23	-0.17421131	0.02	0.077	1.93E-18	1.1
RPP38	8.16E-23	-0.177961361	0.041	0.109	1.97E-18	1.1
GALNT1	8.60E-23	0.398583611	0.206	0.131	2.07E-18	1.1

ZNF195	9.00E-23	-0.209147697	0.058	0.134	2.17E-18	1.1
PML	9.96E-23	-0.229091755	0.06	0.135	2.40E-18	1.1
FARS2	9.98E-23	-0.238060871	0.036	0.101	2.41E-18	1.1
AC007566.	1.04E-22	-0.155543843	0.007	0.053	2.50E-18	1.1
PDS5A	1.06E-22	-0.228074101	0.265	0.395	2.56E-18	1.1
MT-CO3	1.07E-22	-0.163537685	0.998	0.993	2.57E-18	1.1
UBB	1.07E-22	0.404375726	0.781	0.774	2.58E-18	1.1
MCTP1	1.14E-22	-0.286095964	0.052	0.125	2.75E-18	1.1
MAN2A1	1.25E-22	-0.230575143	0.127	0.224	3.02E-18	1.1
TANC1	1.28E-22	-0.245351011	0.178	0.287	3.10E-18	1.1
HIP1	1.32E-22	-0.239187928	0.042	0.11	3.18E-18	1.1
C21orf91	1.38E-22	-0.193867137	0.023	0.081	3.33E-18	1.1
CDC25B	1.41E-22	-0.192538494	0.003	0.045	3.39E-18	1.1
TNRC6A	1.50E-22	0.429213263	0.312	0.23	3.61E-18	1.1
ORC3	1.50E-22	-0.188463151	0.034	0.098	3.62E-18	1.1
SNED1	1.52E-22	-0.183171651	0.018	0.073	3.66E-18	1.1
MAT2A	1.56E-22	-0.206452872	0.021	0.077	3.76E-18	1.1
PSMB2	1.57E-22	-0.210968081	0.078	0.161	3.80E-18	1.1
KRT19	1.58E-22	0.561132734	0.376	0.296	3.80E-18	1.1
PURA	1.60E-22	-0.188602458	0.023	0.081	3.86E-18	1.1
KIAA1033	1.63E-22	-0.231293161	0.053	0.127	3.94E-18	1.1
NNT	1.69E-22	-0.247570664	0.05	0.122	4.06E-18	1.1
RAB27A	1.81E-22	-0.20773943	0.03	0.091	4.36E-18	1.1
VPS50	1.86E-22	-0.190695144	0.018	0.073	4.48E-18	1.1
MAP4K3	1.89E-22	-0.241021386	0.108	0.202	4.56E-18	1.1
POLE2	1.92E-22	-0.22718805	0.138	0.242	4.64E-18	1.1
ATP6V1E1	1.98E-22	-0.232418895	0.195	0.309	4.77E-18	1.1
HNRNPDL	2.03E-22	0.36131336	0.363	0.28	4.90E-18	1.1
FAM32A	2.08E-22	-0.161120433	0.027	0.086	5.01E-18	1.1
PSMD1	2.10E-22	-0.221385209	0.08	0.164	5.05E-18	1.1
TAF15	2.14E-22	-0.263486166	0.23	0.35	5.17E-18	1.1
KIAA1958	2.15E-22	-0.214632459	0.043	0.111	5.18E-18	1.1
LINC00511	2.17E-22	-0.280670446	0.064	0.141	5.23E-18	1.1
RHPN2	2.30E-22	-0.223077839	0.199	0.318	5.53E-18	1.1
KIAA0232	2.48E-22	-0.2222043	0.077	0.158	5.98E-18	1.1
SNRNP35	2.56E-22	-0.193044907	0.035	0.099	6.18E-18	1.1
ESCO1	2.61E-22	-0.191145397	0.047	0.117	6.29E-18	1.1
CDK12	2.62E-22	-0.211843969	0.05	0.121	6.31E-18	1.1
UHRF1BP1I	2.63E-22	0.383043429	0.253	0.173	6.33E-18	1.1
TNFRSF10E	2.75E-22	0.403593618	0.257	0.175	6.62E-18	1.1
DNAJB14	2.80E-22	-0.185191366	0.043	0.112	6.74E-18	1.1
ZNF146	2.86E-22	-0.219979516	0.063	0.138	6.90E-18	1.1
KRT23	3.05E-22	-0.35400033	0.167	0.27	7.36E-18	1.1
VAPA	3.18E-22	0.368595207	0.521	0.466	7.68E-18	1.1
PELI2	3.24E-22	-0.224064953	0.04	0.107	7.82E-18	1.1
C2orf88	3.34E-22	0.385604839	0.442	0.367	8.04E-18	1.1
PHC2	3.38E-22	-0.240248545	0.042	0.108	8.15E-18	1.1

TBC1D14	3.46E-22	-0.204334439	0.031	0.093	8.35E-18	1.1
RTTN	3.48E-22	-0.189416234	0.025	0.083	8.39E-18	1.1
ZNF292	3.52E-22	0.406029769	0.652	0.63	8.48E-18	1.1
NIFK-AS1	4.17E-22	-0.153540007	0.008	0.055	1.01E-17	1.1
LINC00536	4.21E-22	-0.231800812	0.033	0.095	1.02E-17	1.1
BRIX1	4.26E-22	0.37792226	0.175	0.105	1.03E-17	1.1
SLC19A2	4.79E-22	0.310960428	0.137	0.073	1.15E-17	1.1
ARL5B	4.79E-22	0.283791059	0.115	0.057	1.15E-17	1.1
CHMP5	4.84E-22	-0.229954837	0.252	0.375	1.17E-17	1.1
IL15RA	5.22E-22	-0.213557595	0.018	0.072	1.26E-17	1.1
TCF7L1	5.23E-22	0.502376979	0.39	0.316	1.26E-17	1.1
SCFD1	5.36E-22	-0.231780949	0.153	0.253	1.29E-17	1.1
SYNPO2	5.87E-22	0.398809833	0.268	0.18	1.42E-17	1.1
DARS	6.15E-22	-0.238611676	0.242	0.363	1.48E-17	1.1
ALDH1A2	6.28E-22	-0.196157023	0.007	0.052	1.52E-17	1.1
DBI	6.38E-22	0.426895346	0.709	0.689	1.54E-17	1.1
HIST1H2BJ	6.40E-22	-0.206067796	0.043	0.11	1.54E-17	1.1
ARFIP1	6.75E-22	-0.220838363	0.089	0.173	1.63E-17	1.1
PAFAH1B2	6.81E-22	0.389952329	0.291	0.212	1.64E-17	1.1
VRK2	6.84E-22	-0.206390728	0.113	0.205	1.65E-17	1.1
CLK4	6.86E-22	-0.156880532	0.024	0.082	1.66E-17	1.1
STAMBP	7.03E-22	-0.203728303	0.116	0.211	1.70E-17	1.1
C20orf24	7.04E-22	-0.162843947	0.023	0.08	1.70E-17	1.1
RPAP2	7.05E-22	-0.201862841	0.103	0.196	1.70E-17	1.1
MAK	7.09E-22	-0.19485125	0.04	0.107	1.71E-17	1.1
TNPO1	7.16E-22	-0.232990668	0.146	0.248	1.73E-17	1.1
AKAP6	8.34E-22	-0.1966527	0.015	0.066	2.01E-17	1.1
MAP7D1	8.66E-22	0.349628762	0.186	0.114	2.09E-17	1.1
WNK1	8.93E-22	-0.217056951	0.114	0.207	2.15E-17	1.1
TTC1	9.52E-22	-0.224266048	0.241	0.358	2.30E-17	1.1
RP11-511B	9.64E-22	0.33207771	0.617	0.58	2.32E-17	1.1
CCDC125	1.01E-21	-0.205245944	0.025	0.082	2.43E-17	1.1
CHSY1	1.04E-21	0.269189499	0.146	0.08	2.50E-17	1.1
EP300	1.09E-21	-0.222042615	0.083	0.165	2.63E-17	1.1
RAB29	1.11E-21	-0.1662184	0.035	0.098	2.68E-17	1.1
IQCG	1.15E-21	0.426191044	0.277	0.197	2.78E-17	1.1
STIL	1.18E-21	-0.183208678	0.037	0.101	2.84E-17	1.1
PLSCR2	1.20E-21	-0.196306225	0.01	0.058	2.88E-17	1.1
LCORL	1.23E-21	-0.206379282	0.063	0.139	2.97E-17	1.1
RP11-138A	1.24E-21	-0.170184703	0.065	0.144	2.99E-17	1.1
ABI2	1.25E-21	-0.216637408	0.089	0.173	3.01E-17	1.1
TNRC18	1.29E-21	-0.157945593	0.02	0.075	3.12E-17	1.1
RP4-605O3	1.59E-21	-0.21430133	0.048	0.117	3.84E-17	1.1
FAM217B	1.61E-21	-0.13634016	0.007	0.051	3.88E-17	1.1
GRB2	1.69E-21	-0.212421017	0.073	0.15	4.08E-17	1.1
PHYKPL	1.75E-21	-0.182752816	0.016	0.068	4.21E-17	1.1
NCK2	1.83E-21	-0.252639631	0.101	0.187	4.41E-17	1.1

ZNF248	1.89E-21	-0.185189004	0.014	0.063	4.55E-17	1.1
ATXN7L1	2.06E-21	-0.182244145	0.021	0.075	4.97E-17	1.1
RP11-739G	2.09E-21	-0.274450233	0.008	0.053	5.05E-17	1.1
TTBK2	2.10E-21	-0.209500651	0.063	0.138	5.06E-17	1.1
IFIT2	2.16E-21	0.41107972	0.072	0.028	5.20E-17	1.1
GON4L	2.23E-21	-0.199719073	0.083	0.164	5.39E-17	1.1
CEACAM1	2.25E-21	-0.201503337	0.063	0.136	5.42E-17	1.1
RALY	2.26E-21	-0.177724377	0.092	0.178	5.44E-17	1.1
SLF2	2.26E-21	-0.146440253	0.007	0.05	5.46E-17	1.1
TNPO3	2.28E-21	-0.195176805	0.076	0.156	5.50E-17	1.1
ENTPD1-AS	2.29E-21	-0.197188353	0.028	0.086	5.51E-17	1.1
HEATR5A	2.41E-21	-0.147134422	0.021	0.076	5.80E-17	1.1
CTD-3131K	2.51E-21	-0.149183548	0.014	0.063	6.05E-17	1.1
MYH14	2.54E-21	-0.222569705	0.105	0.193	6.11E-17	1.1
SPDYE1	2.58E-21	-0.151568486	0.013	0.062	6.22E-17	1.1
LBP	2.71E-21	-0.23111965	0.004	0.045	6.53E-17	1.1
SUCLG2	2.72E-21	-0.231910817	0.048	0.116	6.56E-17	1.1
LGALS1	2.79E-21	-0.27461598	0.032	0.092	6.74E-17	1.1
MGME1	2.93E-21	-0.170889702	0.025	0.081	7.06E-17	1.1
APPL2	3.03E-21	-0.207988538	0.03	0.089	7.30E-17	1.1
ASXL2	3.04E-21	-0.186361505	0.033	0.093	7.33E-17	1.1
AMY2B	3.08E-21	-0.146664511	0.008	0.053	7.42E-17	1.1
ADAM17	3.09E-21	0.42521546	0.469	0.404	7.45E-17	1.1
RABEP1	3.10E-21	-0.231675562	0.054	0.123	7.47E-17	1.1
EFHD1	3.20E-21	0.208324159	0.087	0.038	7.72E-17	1.1
ANKRD10	3.46E-21	-0.16690119	0.394	0.546	8.33E-17	1.1
KIAA1109	3.58E-21	-0.215365654	0.066	0.141	8.62E-17	1.1
RPS11	3.82E-21	0.374441661	0.78	0.769	9.22E-17	1.1
RASD1	4.11E-21	-0.141056905	0.003	0.042	9.90E-17	1.1
MLLT3	4.14E-21	-0.274785689	0.092	0.173	9.97E-17	1.1
TM2D1	4.29E-21	-0.191590152	0.045	0.112	1.04E-16	1.1
ZDHHC2	4.36E-21	-0.148453353	0.018	0.069	1.05E-16	1.1
STK38L	4.39E-21	0.394271385	0.195	0.123	1.06E-16	1.1
ALG14	4.46E-21	-0.205644349	0.041	0.105	1.08E-16	1.1
TAF3	4.48E-21	-0.186995786	0.023	0.079	1.08E-16	1.1
SNAP23	4.60E-21	-0.201575271	0.088	0.17	1.11E-16	1.1
APBB2	4.63E-21	-0.253697802	0.055	0.125	1.12E-16	1.1
TAGLN2	4.96E-21	-0.292056133	0.12	0.21	1.20E-16	1.1
BIRC3	5.01E-21	-0.162999904	0.738	0.856	1.21E-16	1.1
RBM23	5.04E-21	-0.183971435	0.041	0.105	1.21E-16	1.1
SULF2	5.27E-21	-0.226680406	0.05	0.119	1.27E-16	1.1
ATP9B	5.37E-21	-0.202420665	0.035	0.095	1.29E-16	1.1
FLOT1	5.46E-21	-0.210417733	0.06	0.132	1.32E-16	1.1
MLF1	5.53E-21	0.379319939	0.29	0.207	1.33E-16	1.1
RP11-65I12	5.54E-21	-0.182431139	0.016	0.067	1.34E-16	1.1
DTWD1	5.69E-21	-0.176770132	0.028	0.085	1.37E-16	1.1
GLS	5.73E-21	-0.287193342	0.182	0.286	1.38E-16	1.1

LAP3	5.76E-21	-0.266370318	0.115	0.201	1.39E-16	1.1
WBP2NL	5.92E-21	-0.17027338	0.016	0.066	1.43E-16	1.1
WDSUB1	6.22E-21	-0.18937767	0.02	0.072	1.50E-16	1.1
ARHGAP32	6.23E-21	-0.220904744	0.197	0.308	1.50E-16	1.1
BBS4	6.32E-21	-0.171265702	0.015	0.065	1.52E-16	1.1
GTPBP10	6.36E-21	-0.199087124	0.051	0.12	1.53E-16	1.1
RPS13	6.70E-21	0.391058638	0.829	0.855	1.62E-16	1.1
PICALM	6.72E-21	-0.195131112	0.313	0.449	1.62E-16	1.1
YBX3	6.78E-21	-0.29724863	0.202	0.305	1.64E-16	1.1
STRBP	7.33E-21	-0.228880494	0.124	0.215	1.77E-16	1.1
PRPF3	7.61E-21	-0.232456562	0.066	0.14	1.84E-16	1.1
TPBG	7.84E-21	-0.161175722	0.02	0.072	1.89E-16	1.1
TIMM10B.1	7.93E-21	-0.142913487	0.01	0.056	1.91E-16	1.1
ANAPC13	7.97E-21	-0.19399832	0.07	0.146	1.92E-16	1.1
KLF3	8.38E-21	-0.212115825	0.062	0.135	2.02E-16	1.1
LIPE-AS1	8.41E-21	-0.233678273	0.038	0.1	2.03E-16	1.1
MRFAP1L1	8.63E-21	-0.150378001	0.013	0.06	2.08E-16	1.1
LARS2	8.85E-21	-0.146940073	0.011	0.058	2.13E-16	1.1
CD164	9.20E-21	-0.198424189	0.114	0.205	2.22E-16	1.1
PTP4A2	9.23E-21	0.309686133	0.165	0.097	2.23E-16	1.1
CASK	9.65E-21	-0.187858027	0.206	0.32	2.33E-16	1.1
WDR43	1.01E-20	0.337994984	0.159	0.093	2.43E-16	1.1
PURB	1.01E-20	-0.182698974	0.025	0.08	2.44E-16	1.1
ZXDC	1.03E-20	-0.190186226	0.043	0.108	2.48E-16	1.1
EIF3J-AS1	1.03E-20	-0.157938307	0.023	0.078	2.49E-16	1.1
TAMM41	1.14E-20	-0.155256056	0.01	0.055	2.75E-16	1.1
RP11-350N	1.16E-20	0.115766353	0.032	0.006	2.79E-16	1.1
SLC30A9	1.20E-20	-0.203142014	0.063	0.136	2.90E-16	1.1
MAGI2	1.23E-20	-0.279470275	0.047	0.114	2.97E-16	1.1
PUDP	1.25E-20	-0.140583895	0.007	0.049	3.01E-16	1.1
MYH9	1.25E-20	0.296370864	0.547	0.467	3.01E-16	1.1
EYA3	1.31E-20	-0.219435143	0.049	0.116	3.15E-16	1.1
FOXO3	1.32E-20	-0.123840844	0.238	0.36	3.17E-16	1.1
MT-ND2	1.33E-20	-0.163742635	0.969	0.978	3.20E-16	1.1
GK5	1.34E-20	-0.18679798	0.027	0.083	3.24E-16	1.1
AP001439.	1.35E-20	0.321642537	0.136	0.075	3.26E-16	1.1
CTCF	1.39E-20	-0.184079505	0.032	0.09	3.34E-16	1.1
STEAP1	1.40E-20	-0.16264906	0.013	0.06	3.38E-16	1.1
CD82	1.41E-20	-0.199036162	0.03	0.088	3.41E-16	1.1
CHD6	1.44E-20	0.418341596	0.35	0.276	3.47E-16	1.1
ITSN2	1.49E-20	-0.267151285	0.241	0.354	3.60E-16	1.1
TRPC1	1.51E-20	-0.160540478	0.013	0.06	3.65E-16	1.1
HCFC2	1.59E-20	0.181573704	0.068	0.026	3.85E-16	1.1
MROH1	1.61E-20	-0.181556137	0.018	0.069	3.87E-16	1.1
LYRM2	1.61E-20	-0.181362793	0.058	0.129	3.87E-16	1.1
DANT2	1.73E-20	-0.215415454	0.039	0.101	4.17E-16	1.1
RNF168	1.73E-20	-0.165136608	0.035	0.095	4.17E-16	1.1

ZNF75A	1.74E-20	-0.144545453	0.006	0.048	4.20E-16	1.1
BCL11A	1.80E-20	-0.17371469	0.022	0.075	4.33E-16	1.1
LUC7L	1.84E-20	-0.202550717	0.048	0.113	4.44E-16	1.1
HNRNPU	1.89E-20	0.316496253	0.15	0.086	4.55E-16	1.1
ESR2	1.97E-20	-0.194322016	0.06	0.132	4.75E-16	1.1
RASGEF1A	1.98E-20	-0.160954889	0.008	0.051	4.78E-16	1.1
GCNT1	2.02E-20	0.39456856	0.136	0.076	4.86E-16	1.1
RP5-1101C	2.02E-20	-0.168099192	0.016	0.065	4.87E-16	1.1
ATP11C	2.08E-20	-0.132216834	0.03	0.089	5.01E-16	1.1
GGA2	2.09E-20	0.350784812	0.202	0.131	5.04E-16	1.1
RP11-277P	2.18E-20	-0.229734046	0.03	0.086	5.25E-16	1.1
C11orf49	2.20E-20	-0.24584896	0.084	0.163	5.31E-16	1.1
TCF25	2.29E-20	-0.253631909	0.06	0.131	5.53E-16	1.1
EZH1	2.34E-20	-0.187334612	0.049	0.116	5.63E-16	1.1
MTX2	2.38E-20	-0.18254062	0.028	0.085	5.74E-16	1.1
UFC1	2.40E-20	0.343489423	0.544	0.498	5.78E-16	1.1
SLC39A1	2.45E-20	-0.203559981	0.039	0.1	5.91E-16	1.1
LMBR1	2.54E-20	-0.217637297	0.068	0.141	6.13E-16	1.1
GNL2	2.56E-20	0.292972377	0.149	0.085	6.18E-16	1.1
THUMPD1	2.80E-20	-0.174323505	0.038	0.099	6.74E-16	1.1
SAR1B	2.81E-20	0.533620007	0.496	0.441	6.78E-16	1.1
MYO9B	3.09E-20	0.419512254	0.258	0.182	7.46E-16	1.1
ZC3H13	3.30E-20	-0.21153603	0.116	0.205	7.96E-16	1.1
ZFAND1	3.34E-20	0.358443208	0.334	0.258	8.06E-16	1.1
DAPK3	3.37E-20	0.213701634	0.091	0.042	8.12E-16	1.1
PPCDC	3.59E-20	-0.166198291	0.013	0.059	8.65E-16	1.1
RP5-894A1	3.79E-20	-0.132192946	0.003	0.04	9.14E-16	1.1
RAB13	3.84E-20	-0.227595379	0.086	0.164	9.25E-16	1.1
SOCS5	4.20E-20	-0.239153998	0.105	0.19	1.01E-15	1.1
CD83	4.24E-20	0.39433925	0.146	0.083	1.02E-15	1.1
DDX46	4.40E-20	-0.221156401	0.054	0.121	1.06E-15	1.1
CDC42SE2	4.43E-20	-0.192882877	0.152	0.251	1.07E-15	1.1
CSF3R	4.46E-20	-0.152305208	0.005	0.046	1.08E-15	1.1
VPS54	4.51E-20	-0.205466051	0.158	0.258	1.09E-15	1.1
CHCHD7	4.61E-20	-0.167386272	0.031	0.089	1.11E-15	1.1
DYM	4.73E-20	-0.205580587	0.169	0.274	1.14E-15	1.1
ZCCHC8	4.82E-20	0.337125006	0.183	0.114	1.16E-15	1.1
PTTG1IP	4.83E-20	-0.202953256	0.082	0.161	1.16E-15	1.1
ATL3	4.84E-20	-0.176302881	0.064	0.137	1.17E-15	1.1
DYNLT3	5.23E-20	-0.229578373	0.147	0.243	1.26E-15	1.1
SH2D3A	5.27E-20	-0.176051809	0.018	0.067	1.27E-15	1.1
AHI1	5.44E-20	-0.240805399	0.138	0.232	1.31E-15	1.1
ACTB	5.49E-20	0.352659787	0.658	0.601	1.32E-15	1.1
CXCL6	5.71E-20	-0.206712898	0.007	0.049	1.38E-15	1.1
YIPF4	5.95E-20	-0.188582444	0.048	0.113	1.43E-15	1.1
PAPD4	6.11E-20	-0.212971075	0.214	0.322	1.47E-15	1.1
NUDCD3	6.25E-20	-0.210575262	0.057	0.125	1.51E-15	1.1

ZFYVE16	6.27E-20	-0.175191907	0.039	0.101	1.51E-15	1.1
TRA2B	6.37E-20	0.422716218	0.252	0.179	1.54E-15	1.1
ACSS2	6.39E-20	-0.221049439	0.115	0.202	1.54E-15	1.1
DNHD1	6.48E-20	-0.171235794	0.03	0.086	1.56E-15	1.1
TUSC3	6.92E-20	-0.230172467	0.089	0.167	1.67E-15	1.1
MOCOS	7.15E-20	-0.167120291	0.015	0.063	1.72E-15	1.1
N4BP2L1	7.18E-20	-0.202376551	0.057	0.126	1.73E-15	1.1
WWTR1	7.81E-20	0.440751724	0.453	0.392	1.88E-15	1.1
RUFY1	7.90E-20	-0.204029772	0.035	0.093	1.90E-15	1.1
NEDD1	8.20E-20	-0.172605855	0.032	0.089	1.98E-15	1.1
HP	8.22E-20	-0.484836994	0.002	0.038	1.98E-15	1.1
C1orf198	8.70E-20	0.237307971	0.103	0.051	2.10E-15	1.1
TNKS	9.35E-20	-0.188541571	0.193	0.301	2.25E-15	1.1
SPOP	9.38E-20	-0.151897448	0.043	0.107	2.26E-15	1.1
C1orf132	9.96E-20	-0.218082279	0.028	0.082	2.40E-15	1.1
TGIF1	1.00E-19	-0.161530845	0.077	0.153	2.41E-15	1.1
ZNF765	1.06E-19	-0.142294847	0.006	0.047	2.55E-15	1.1
TLR1	1.14E-19	-0.175267691	0.021	0.072	2.75E-15	1.1
PIGL	1.17E-19	-0.200338244	0.033	0.09	2.81E-15	1.1
EBF4	1.17E-19	-0.154307329	0.009	0.051	2.82E-15	1.1
SEMA3B	1.19E-19	0.161718068	0.045	0.013	2.86E-15	1.1
C1orf147	1.20E-19	-0.143994478	0.005	0.043	2.90E-15	1.1
DDR1	1.23E-19	-0.196457076	0.083	0.16	2.96E-15	1.1
ITSN1	1.26E-19	-0.256653868	0.077	0.151	3.03E-15	1.1
PRIM2	1.31E-19	-0.177193977	0.035	0.093	3.16E-15	1.1
ERCC3	1.36E-19	-0.151027826	0.008	0.049	3.28E-15	1.1
RBP5	1.44E-19	-0.132873125	0.011	0.055	3.47E-15	1.1
RP11-141O	1.47E-19	-0.164859234	0.007	0.047	3.54E-15	1.1
HDAC1	1.49E-19	-0.19654425	0.061	0.131	3.59E-15	1.1
ZNHIT6	1.54E-19	-0.228540731	0.125	0.212	3.72E-15	1.1
MTMR3	1.57E-19	0.362218325	0.22	0.147	3.78E-15	1.1
SLC22A23	1.60E-19	-0.192197299	0.104	0.188	3.86E-15	1.1
RP11-473I1	1.66E-19	0.190526511	0.081	0.036	4.01E-15	1.1
NCALD	1.75E-19	0.54288781	0.269	0.198	4.22E-15	1.1
PPM1A	1.76E-19	-0.195503204	0.075	0.149	4.25E-15	1.1
TLK1	1.84E-19	-0.227568397	0.139	0.232	4.45E-15	1.1
RSPH3	1.85E-19	-0.18847978	0.027	0.08	4.45E-15	1.1
FABP3	1.88E-19	0.367249742	0.035	0.009	4.54E-15	1.1
PIBF1	2.01E-19	-0.269546489	0.145	0.236	4.86E-15	1.1
TRIP4	2.02E-19	-0.149666748	0.014	0.06	4.86E-15	1.1
SMARCD3	2.03E-19	-0.161519405	0.007	0.047	4.89E-15	1.1
MED13	2.05E-19	0.402917391	0.4	0.331	4.93E-15	1.1
C15orf41	2.16E-19	-0.188066161	0.032	0.088	5.21E-15	1.1
MAGI3	2.23E-19	-0.186049651	0.12	0.209	5.38E-15	1.1
EXOSC8	2.24E-19	0.325766056	0.201	0.13	5.39E-15	1.1
HNRNPF	2.24E-19	-0.197762962	0.078	0.153	5.41E-15	1.1
RAP1GDS1	2.46E-19	-0.191234	0.041	0.101	5.94E-15	1.1

ZNF44	2.49E-19	-0.148476706	0.018	0.067	6.00E-15	1.1
CHFR	2.51E-19	-0.16741742	0.029	0.084	6.05E-15	1.1
GDE1	2.55E-19	-0.152646024	0.029	0.084	6.16E-15	1.1
PEA15	2.60E-19	-0.199808756	0.129	0.218	6.28E-15	1.1
MLH3	2.69E-19	-0.189521466	0.053	0.118	6.48E-15	1.1
PMM2	2.97E-19	-0.189957866	0.046	0.11	7.15E-15	1.1
PPID	3.21E-19	-0.158495636	0.035	0.093	7.74E-15	1.1
PAK2	3.21E-19	-0.171358062	0.112	0.199	7.75E-15	1.1
DDX60L	3.49E-19	-0.144054243	0.014	0.06	8.41E-15	1.1
DNER	3.56E-19	-0.265838303	0.032	0.087	8.59E-15	1.1
TFB1M	3.67E-19	-0.154110772	0.024	0.076	8.85E-15	1.1
CHD8	3.75E-19	-0.196062684	0.047	0.109	9.04E-15	1.1
SDF2	3.80E-19	-0.163218794	0.05	0.114	9.15E-15	1.1
MB21D2	3.80E-19	0.286571903	0.113	0.059	9.17E-15	1.1
MIR646HG	3.97E-19	-0.227110649	0.049	0.113	9.58E-15	1.1
CTNND1	3.98E-19	0.362695049	0.372	0.3	9.60E-15	1.1
RP11-106M	4.07E-19	-0.171869877	0.011	0.055	9.82E-15	1.1
KBTBD3	4.19E-19	-0.132278464	0.01	0.053	1.01E-14	1.1
ZNF302	4.26E-19	-0.122635191	0.005	0.042	1.03E-14	1.1
WASF1	4.26E-19	-0.151728107	0.02	0.07	1.03E-14	1.1
DCUN1D1	4.53E-19	-0.181795794	0.087	0.165	1.09E-14	1.1
9-Sep	4.68E-19	-0.176426806	0.072	0.146	1.13E-14	1.1
EML4	4.74E-19	0.371522221	0.321	0.244	1.14E-14	1.1
CUL4A	4.78E-19	-0.193925002	0.083	0.159	1.15E-14	1.1
ZNF611	4.89E-19	-0.138518081	0.019	0.067	1.18E-14	1.1
CREB1	4.92E-19	-0.18282535	0.119	0.206	1.19E-14	1.1
TNC	5.02E-19	-0.444857158	0.065	0.132	1.21E-14	1.1
TRPM7	5.02E-19	-0.213837029	0.124	0.211	1.21E-14	1.1
SETD5	5.09E-19	0.400871594	0.546	0.509	1.23E-14	1.1
TRMT11	5.30E-19	-0.174640637	0.081	0.158	1.28E-14	1.1
RCOR3	5.39E-19	-0.222038424	0.05	0.113	1.30E-14	1.1
SNX3	5.81E-19	-0.178012377	0.126	0.214	1.40E-14	1.1
KIAA0196	5.85E-19	-0.145763763	0.017	0.064	1.41E-14	1.1
ITGB8	5.85E-19	-0.236420277	0.654	0.766	1.41E-14	1.1
WSB1	5.99E-19	0.523921374	0.385	0.32	1.44E-14	1.1
PRLR	6.18E-19	-0.215238677	0.078	0.152	1.49E-14	1.1
GDAP2	6.45E-19	-0.142278383	0.011	0.054	1.56E-14	1.1
RALGAPB	6.48E-19	-0.219323272	0.074	0.145	1.56E-14	1.1
PON2	6.72E-19	-0.157090778	0.021	0.071	1.62E-14	1.1
ERC2	6.76E-19	-0.207547172	0.046	0.108	1.63E-14	1.1
NSUN3	7.13E-19	-0.168453057	0.017	0.063	1.72E-14	1.1
RP11-475O	7.24E-19	-0.191786111	0.018	0.065	1.75E-14	1.1
BZW1	7.29E-19	-0.197479502	0.096	0.174	1.76E-14	1.1
PIWIL4	7.37E-19	-0.174718931	0.025	0.076	1.78E-14	1.1
GRIK1	7.45E-19	-0.17588298	0.019	0.067	1.80E-14	1.1
FAM179B	7.71E-19	-0.189042822	0.037	0.094	1.86E-14	1.1
ZNF100	7.74E-19	-0.142774523	0.01	0.052	1.87E-14	1.1

DRAM1	7.85E-19	-0.211169241	0.108	0.191	1.89E-14	1.1
AP4E1	8.00E-19	-0.136081457	0.014	0.059	1.93E-14	1.1
GULP1	8.13E-19	-0.207665745	0.125	0.213	1.96E-14	1.1
ACTR2	8.18E-19	-0.17408098	0.147	0.241	1.97E-14	1.1
AC058791.	8.31E-19	-0.209796598	0.142	0.233	2.00E-14	1.1
UBL5	8.44E-19	-0.185977535	0.289	0.411	2.03E-14	1.1
MFSD14B	8.67E-19	-0.173959853	0.052	0.116	2.09E-14	1.1
KLK5	8.75E-19	0.325641918	0.108	0.056	2.11E-14	1.1
RPL23	8.88E-19	0.14952844	0.968	0.961	2.14E-14	1.1
CREB5	8.89E-19	0.402983005	0.377	0.297	2.14E-14	1.1
PLIN3	9.03E-19	-0.169188937	0.06	0.128	2.18E-14	1.1
SUGT1	9.04E-19	-0.177280168	0.095	0.174	2.18E-14	1.1
ZC3H6	9.09E-19	-0.175018667	0.03	0.084	2.19E-14	1.1
DNAJC5	9.17E-19	-0.189336448	0.041	0.1	2.21E-14	1.1
FAM3B	9.28E-19	-0.184001781	0.034	0.089	2.24E-14	1.1
ROCK1	9.65E-19	-0.20387843	0.14	0.231	2.33E-14	1.1
PIGA	9.78E-19	0.272587617	0.125	0.069	2.36E-14	1.1
MIB1	9.79E-19	-0.179919851	0.22	0.334	2.36E-14	1.1
RFWD3	1.09E-18	-0.163389933	0.015	0.061	2.63E-14	1.1
NRF1	1.11E-18	-0.203970161	0.073	0.145	2.68E-14	1.1
SPG7	1.13E-18	-0.183613111	0.021	0.071	2.72E-14	1.1
IL34	1.20E-18	-0.186680105	0.103	0.186	2.89E-14	1.1
C11orf1	1.22E-18	0.340628025	0.231	0.161	2.94E-14	1.1
RP11-418J1	1.23E-18	-0.160263128	0.012	0.054	2.96E-14	1.1
ZSWIM4	1.24E-18	-0.136371951	0.131	0.22	2.99E-14	1.1
SMARCAD1	1.25E-18	-0.172715634	0.029	0.083	3.02E-14	1.1
FRY	1.29E-18	-0.167714966	0.01	0.051	3.12E-14	1.1
NBPF12	1.31E-18	-0.136632147	0.026	0.078	3.15E-14	1.1
RNF149	1.32E-18	0.395189853	0.351	0.279	3.19E-14	1.1
PRKX	1.39E-18	-0.180300967	0.114	0.198	3.36E-14	1.1
PERP	1.40E-18	-0.209856575	0.246	0.358	3.38E-14	1.1
KIF9-AS1	1.41E-18	-0.150350258	0.015	0.06	3.39E-14	1.1
SRSF4	1.44E-18	-0.219435554	0.21	0.313	3.48E-14	1.1
DNAJC7	1.47E-18	-0.19524057	0.145	0.236	3.55E-14	1.1
TMEM164	1.49E-18	-0.140760263	0.013	0.056	3.58E-14	1.1
C15orf52	1.56E-18	-0.144571294	0.014	0.058	3.77E-14	1.1
ZNF226	1.56E-18	-0.170593547	0.037	0.093	3.77E-14	1.1
STAT1	1.64E-18	-0.161734847	0.035	0.091	3.95E-14	1.1
HS2ST1	1.64E-18	-0.154298648	0.008	0.047	3.96E-14	1.1
ACTR6	1.76E-18	-0.161740465	0.111	0.195	4.23E-14	1.1
BPNT1	1.78E-18	-0.150017925	0.023	0.073	4.30E-14	1.1
DHRS7	1.90E-18	-0.142882409	0.021	0.069	4.59E-14	1.1
PKM	2.03E-18	-0.275578752	0.185	0.28	4.90E-14	1.1
CTD-2337A	2.05E-18	-0.23260363	0.126	0.214	4.94E-14	1.1
RP11-793A	2.17E-18	-0.182108244	0.011	0.053	5.23E-14	1.1
GOLGB1	2.20E-18	-0.198934745	0.291	0.409	5.30E-14	1.1
RAB30-AS1	2.23E-18	-0.156237456	0.052	0.116	5.37E-14	1.1

ODAM	2.30E-18	-0.267112873	0.001	0.034	5.53E-14	1.1
PILRB	2.34E-18	-0.184466383	0.02	0.068	5.65E-14	1.1
RPL39	2.37E-18	0.195833938	0.963	0.936	5.72E-14	1.1
PLGRKT	2.42E-18	-0.147330655	0.014	0.057	5.84E-14	1.1
CRLF3	2.46E-18	-0.178644333	0.073	0.145	5.94E-14	1.1
UBA6-AS1	2.48E-18	-0.22588051	0.045	0.105	5.98E-14	1.1
ESR1	2.59E-18	-0.263084681	0.083	0.155	6.25E-14	1.1
DICER1	2.79E-18	-0.200034739	0.108	0.189	6.74E-14	1.1
CTD-2561J:	2.80E-18	-0.137212888	0.012	0.054	6.75E-14	1.1
RP11-774D	2.89E-18	-0.139015594	0.024	0.074	6.96E-14	1.1
MTERF1	3.03E-18	-0.151776363	0.037	0.094	7.31E-14	1.1
FAM168A	3.09E-18	0.380619572	0.277	0.205	7.46E-14	1.1
BOD1L1	3.19E-18	-0.183243149	0.058	0.123	7.69E-14	1.1
NBAT1	3.20E-18	-0.182377422	0.039	0.097	7.72E-14	1.1
EPB41L4A	3.37E-18	-0.154248477	0.012	0.053	8.13E-14	1.1
CBX3	3.45E-18	-0.210360914	0.165	0.26	8.32E-14	1.1
RECQL	3.73E-18	-0.142020316	0.029	0.081	9.00E-14	1.1
ITPKB	3.79E-18	-0.161964476	0.025	0.076	9.15E-14	1.1
UGP2	3.81E-18	-0.153112579	0.421	0.562	9.18E-14	1.1
FAM102A	3.85E-18	-0.159391135	0.034	0.089	9.27E-14	1.1
CHD7	4.24E-18	-0.182310056	0.078	0.15	1.02E-13	1.1
ARHGEF38	4.27E-18	0.400210744	0.525	0.469	1.03E-13	1.1
SLC33A1	4.32E-18	-0.17230319	0.036	0.092	1.04E-13	1.1
KIAA1147	4.41E-18	-0.202563889	0.043	0.101	1.06E-13	1.1
RP11-544A	4.41E-18	-0.138328756	0.01	0.051	1.06E-13	1.1
FBXO38	4.42E-18	-0.138586733	0.02	0.068	1.07E-13	1.1
TECPR2	4.49E-18	-0.154875107	0.013	0.056	1.08E-13	1.1
GJA1	4.51E-18	0.296012243	0.093	0.046	1.09E-13	1.1
SF3B3	4.54E-18	-0.176486774	0.146	0.236	1.09E-13	1.1
SMAD5	4.54E-18	-0.161259667	0.044	0.104	1.09E-13	1.1
SPDYE5	4.62E-18	-0.169719332	0.038	0.095	1.11E-13	1.1
SLC7A11	4.68E-18	-0.207489146	0.016	0.061	1.13E-13	1.1
PWWP2A	4.69E-18	-0.175562853	0.031	0.084	1.13E-13	1.1
QDPR	4.73E-18	-0.164760895	0.026	0.076	1.14E-13	1.1
HDAC4	4.78E-18	-0.177317525	0.012	0.053	1.15E-13	1.1
AQR	4.85E-18	-0.182718769	0.041	0.099	1.17E-13	1.1
ACLY	4.89E-18	-0.113076962	0.061	0.127	1.18E-13	1.1
APOL1	4.94E-18	-0.131375519	0.006	0.044	1.19E-13	1.1
RP3-325F2:	4.99E-18	-0.132172407	0.007	0.044	1.20E-13	1.1
LINC01122	5.08E-18	-0.215523086	0.028	0.08	1.23E-13	1.1
RAD50	5.33E-18	-0.196797015	0.054	0.117	1.28E-13	1.1
TIMP1	5.39E-18	-0.231414389	0.023	0.072	1.30E-13	1.1
TMEM99	5.45E-18	0.244166386	0.118	0.065	1.31E-13	1.1
RNF144B	5.46E-18	-0.276170964	0.272	0.378	1.32E-13	1.1
NDUFS4	5.54E-18	0.353583526	0.494	0.451	1.34E-13	1.1
ABHD18	5.54E-18	-0.169214359	0.165	0.263	1.34E-13	1.1
CYYR1	5.64E-18	-0.192167872	0.037	0.092	1.36E-13	1.1

TPTEP1	5.66E-18	-0.202744864	0.163	0.257	1.37E-13	1.1
GLMN	6.29E-18	-0.166619677	0.021	0.068	1.52E-13	1.1
ABCC4	6.51E-18	-0.192502337	0.008	0.046	1.57E-13	1.1
ATP13A3	6.67E-18	0.466643576	0.387	0.327	1.61E-13	1.1
TMSB10	6.70E-18	-0.335194243	0.56	0.651	1.61E-13	1.1
ZNF562	6.76E-18	-0.20883328	0.169	0.262	1.63E-13	1.1
PIGR	6.99E-18	0.579617824	0.559	0.504	1.68E-13	1.1
KRCC1	7.07E-18	-0.160867596	0.037	0.093	1.70E-13	1.1
FAM107B	7.08E-18	-0.246692849	0.176	0.269	1.71E-13	1.1
C1GALT1C1	7.13E-18	-0.136107054	0.015	0.059	1.72E-13	1.1
KIAA1328	7.16E-18	-0.179336499	0.045	0.104	1.73E-13	1.1
LARP1	7.35E-18	-0.183884689	0.118	0.201	1.77E-13	1.1
PEX2	7.36E-18	-0.16890318	0.051	0.112	1.77E-13	1.1
CYP1B1-AS	7.45E-18	-0.140265114	0.01	0.049	1.80E-13	1.1
RP11-266O	7.71E-18	-0.278189452	0.018	0.062	1.86E-13	1.1
SMAD2	7.79E-18	-0.202523797	0.168	0.263	1.88E-13	1.1
CTPS2	7.92E-18	-0.162981487	0.03	0.082	1.91E-13	1.1
SEC61G	7.97E-18	-0.258962762	0.623	0.73	1.92E-13	1.1
ZNF430	8.02E-18	-0.159975869	0.03	0.081	1.93E-13	1.1
PRKAR2A	8.10E-18	-0.150902359	0.072	0.143	1.95E-13	1.1
SOX5	8.37E-18	-0.189660749	0.011	0.051	2.02E-13	1.1
GABRG3	8.77E-18	-0.16105229	0.01	0.05	2.12E-13	1.1
RNASEH2B	9.30E-18	-0.12552006	0.011	0.052	2.24E-13	1.1
NF2	9.93E-18	0.243334395	0.104	0.054	2.39E-13	1.1
NHS	1.01E-17	-0.235703763	0.129	0.212	2.44E-13	1.1
PIK3C2G	1.02E-17	0.279987249	0.15	0.089	2.46E-13	1.1
NUDT5	1.05E-17	-0.190543531	0.123	0.205	2.54E-13	1.1
RFX7	1.09E-17	-0.214000103	0.094	0.169	2.63E-13	1.1
SHROOM4	1.12E-17	-0.127311979	0.005	0.041	2.71E-13	1.1
HIST2H2BE	1.13E-17	-0.203438031	0.05	0.11	2.72E-13	1.1
BTF3L4	1.13E-17	-0.195997543	0.211	0.312	2.73E-13	1.1
TRAF1	1.13E-17	-0.207098889	0.029	0.08	2.73E-13	1.1
C16orf62	1.16E-17	-0.163859987	0.024	0.072	2.81E-13	1.1
FBXO9	1.20E-17	-0.175948736	0.033	0.085	2.90E-13	1.1
SH3RF1	1.20E-17	0.402694571	0.295	0.222	2.90E-13	1.1
FBXO22	1.20E-17	-0.150523473	0.018	0.063	2.90E-13	1.1
TRA2A	1.23E-17	0.357385587	0.455	0.397	2.95E-13	1.1
CYB5R3	1.25E-17	-0.181224964	0.036	0.091	3.01E-13	1.1
HS3ST3B1	1.26E-17	-0.127913297	0.003	0.035	3.04E-13	1.1
EEF2	1.37E-17	-0.368954295	0.368	0.474	3.30E-13	1.1
TIAM2	1.40E-17	-0.146972499	0.172	0.27	3.38E-13	1.1
RP11-142M	1.44E-17	-0.135145761	0.003	0.035	3.46E-13	1.1
UTRN	1.49E-17	-0.287081057	0.151	0.239	3.59E-13	1.1
CYP2U1	1.50E-17	0.127651713	0.051	0.018	3.61E-13	1.1
TAF8	1.52E-17	-0.135527956	0.018	0.063	3.66E-13	1.1
PBX3	1.54E-17	-0.165826628	0.016	0.059	3.71E-13	1.1
SCGB1D2	1.58E-17	1.052359251	0.136	0.079	3.82E-13	1.1

CDYL	1.60E-17	-0.158611098	0.158	0.252	3.85E-13	1.1
SRGAP2B	1.63E-17	-0.135414414	0.008	0.046	3.93E-13	1.1
MTRNR2L8	1.65E-17	0.329621867	0.254	0.183	3.99E-13	1.1
SLTM	1.72E-17	-0.225683351	0.19	0.285	4.15E-13	1.1
SMIM12	1.73E-17	-0.138929238	0.01	0.049	4.18E-13	1.1
RP11-514P	1.77E-17	-0.137020629	0.01	0.049	4.26E-13	1.1
SLC38A6	1.77E-17	-0.124202518	0.01	0.049	4.27E-13	1.1
GLTSCR1L	1.78E-17	-0.187117681	0.088	0.161	4.29E-13	1.1
PDCD5	1.78E-17	-0.140228642	0.012	0.052	4.30E-13	1.1
TCTN3	1.78E-17	-0.133025403	0.01	0.048	4.30E-13	1.1
MKLN1	1.80E-17	-0.2321764	0.546	0.665	4.33E-13	1.1
SLC39A11	1.85E-17	-0.153154591	0.036	0.091	4.46E-13	1.1
LINC00853	1.87E-17	-0.111763742	0.005	0.041	4.51E-13	1.1
CTNNAL1	1.92E-17	0.334793783	0.15	0.092	4.64E-13	1.1
RP11-287D	1.97E-17	-0.135832131	0.009	0.047	4.74E-13	1.1
ANKUB1	2.02E-17	-0.178130995	0.084	0.157	4.87E-13	1.1
PSMF1	2.02E-17	-0.171260675	0.049	0.109	4.88E-13	1.1
NTRK2	2.08E-17	0.314774502	0.24	0.169	5.02E-13	1.1
SFT2D2	2.10E-17	-0.201846046	0.159	0.25	5.08E-13	1.1
RASA1	2.14E-17	-0.196732322	0.138	0.225	5.15E-13	1.1
CA5B	2.18E-17	0.300091348	0.181	0.117	5.25E-13	1.1
INSIG1	2.19E-17	-0.189675835	0.06	0.124	5.27E-13	1.1
GLG1	2.23E-17	-0.189823032	0.138	0.225	5.37E-13	1.1
TGFBR2	2.23E-17	0.370928554	0.171	0.11	5.39E-13	1.1
PRCC	2.25E-17	-0.148225781	0.033	0.085	5.42E-13	1.1
NDUFAF7	2.28E-17	-0.144239738	0.028	0.079	5.50E-13	1.1
R3HDM1	2.34E-17	-0.180422398	0.025	0.074	5.63E-13	1.1
ARID4B	2.34E-17	0.376055369	0.531	0.493	5.65E-13	1.1
OGDH	2.39E-17	-0.194719865	0.062	0.126	5.76E-13	1.1
MLXIP	2.46E-17	-0.167961914	0.115	0.196	5.93E-13	1.1
RP11-20I2(	2.50E-17	-0.119385007	0.007	0.043	6.02E-13	1.1
RPS6KA2	2.60E-17	-0.128167551	0.059	0.123	6.26E-13	1.1
RP11-692D	2.67E-17	-0.14780446	0.006	0.042	6.43E-13	1.1
MSN	2.69E-17	-0.196465351	0.375	0.498	6.48E-13	1.1
UQCR10	2.80E-17	-0.190171943	0.314	0.432	6.76E-13	1.1
RHBDL2	2.89E-17	-0.131907302	0.013	0.054	6.96E-13	1.1
LYPLAL1	2.93E-17	-0.160578374	0.038	0.093	7.08E-13	1.1
IRX3	3.14E-17	-0.129239398	0.018	0.063	7.56E-13	1.1
ERGIC3	3.18E-17	0.394401425	0.404	0.35	7.68E-13	1.1
ARHGEF9	3.24E-17	-0.13841197	0.017	0.061	7.80E-13	1.1
AMN1	3.33E-17	-0.192579097	0.078	0.147	8.02E-13	1.1
SYMPK	3.38E-17	-0.171071146	0.03	0.081	8.14E-13	1.1
CISD1	3.42E-17	-0.188428333	0.158	0.248	8.26E-13	1.1
N4BP1	3.43E-17	-0.233879256	0.131	0.212	8.28E-13	1.1
ZNF609	3.46E-17	-0.244675201	0.444	0.565	8.35E-13	1.1
AATF	3.47E-17	-0.148362727	0.012	0.051	8.36E-13	1.1
HAGH	3.52E-17	-0.123685177	0.007	0.043	8.50E-13	1.1

RABGGTB	3.56E-17	0.265845504	0.135	0.079	8.58E-13	1.1
BMS1	3.57E-17	-0.161065365	0.057	0.12	8.62E-13	1.1
DNAH14	3.59E-17	-0.207141656	0.03	0.08	8.65E-13	1.1
SYNRG	3.59E-17	-0.155172672	0.023	0.07	8.67E-13	1.1
SPRED1	3.61E-17	-0.192170964	0.037	0.09	8.71E-13	1.1
EPB41L5	3.65E-17	-0.144617025	0.128	0.214	8.80E-13	1.1
VPS25	3.70E-17	-0.146185226	0.077	0.147	8.91E-13	1.1
ZFAND3	3.74E-17	-0.140323029	0.536	0.675	9.03E-13	1.1
DNAJC16	3.76E-17	-0.140516638	0.022	0.069	9.07E-13	1.1
ANKRD17	3.87E-17	-0.196540488	0.348	0.472	9.34E-13	1.1
RP11-115D	3.92E-17	-0.141691986	0.005	0.04	9.44E-13	1.1
FBXO42	3.93E-17	0.365049492	0.222	0.155	9.47E-13	1.1
NBPF11	3.96E-17	-0.137374185	0.025	0.074	9.55E-13	1.1
ZADH2	4.17E-17	-0.127290185	0.008	0.045	1.01E-12	1.1
SLC30A5	4.32E-17	-0.144399272	0.029	0.08	1.04E-12	1.1
UQCC1	4.40E-17	-0.151591794	0.026	0.075	1.06E-12	1.1
CCT6A	4.51E-17	-0.151940438	0.074	0.142	1.09E-12	1.1
MGAT4A	4.52E-17	-0.150242431	0.017	0.06	1.09E-12	1.1
AMOT	4.52E-17	-0.153559757	0.019	0.063	1.09E-12	1.1
RP11-286N	4.61E-17	-0.146969066	0.017	0.06	1.11E-12	1.1
MYC	4.68E-17	0.356343624	0.196	0.131	1.13E-12	1.1
FAM3C	4.69E-17	-0.194906715	0.101	0.177	1.13E-12	1.1
CLEC16A	4.74E-17	-0.205124219	0.103	0.179	1.14E-12	1.1
MRPS33	4.75E-17	0.378382576	0.354	0.292	1.15E-12	1.1
KIN	4.78E-17	-0.148248896	0.037	0.091	1.15E-12	1.1
HNRNPA2B	4.78E-17	-0.243034669	0.339	0.452	1.15E-12	1.1
GAPVD1	4.79E-17	0.316507841	0.261	0.19	1.16E-12	1.1
PPL	4.88E-17	-0.180782631	0.053	0.113	1.18E-12	1.1
FANK1	4.92E-17	-0.164724289	0.034	0.087	1.19E-12	1.1
PLAUR	4.97E-17	-0.285739671	0.127	0.206	1.20E-12	1.1
RP11-318C	5.09E-17	-0.143655991	0.015	0.058	1.23E-12	1.1
STAT2	5.35E-17	-0.204461355	0.051	0.11	1.29E-12	1.1
AP5M1	5.41E-17	-0.153187706	0.03	0.081	1.30E-12	1.1
MT-CO2	5.60E-17	-0.202439413	0.985	0.985	1.35E-12	1.1
AC004878.	5.67E-17	0.153551103	0.046	0.016	1.37E-12	1.1
RP11-508N	5.89E-17	-0.138336942	0.033	0.086	1.42E-12	1.1
ANKRD26	5.89E-17	-0.175295848	0.036	0.089	1.42E-12	1.1
SRPX2	5.90E-17	-0.132921214	0.013	0.054	1.42E-12	1.1
AC091153.	5.90E-17	0.104369818	0.039	0.012	1.42E-12	1.1
RPA1	5.98E-17	-0.154207852	0.027	0.076	1.44E-12	1.1
CBLB	6.36E-17	-0.194724215	0.164	0.255	1.53E-12	1.1
XRCC4	6.37E-17	-0.139435044	0.02	0.064	1.54E-12	1.1
ZBED5-AS1	6.39E-17	0.117562217	0.043	0.014	1.54E-12	1.1
PHF3	6.41E-17	0.345521815	0.393	0.326	1.55E-12	1.1
P3H2	6.47E-17	-0.210348755	0.082	0.152	1.56E-12	1.1
ZNF780B	6.52E-17	-0.143598153	0.013	0.052	1.57E-12	1.1
MLLT6	6.58E-17	-0.205014104	0.037	0.09	1.59E-12	1.1

CFAP161	6.62E-17	-0.153624704	0.008	0.044	1.60E-12	1.1
POLR2F	6.67E-17	-0.196779977	0.033	0.084	1.61E-12	1.1
MINA	6.80E-17	-0.137720459	0.037	0.09	1.64E-12	1.1
ZCCHC7	6.96E-17	-0.243426421	0.313	0.422	1.68E-12	1.1
FAM193A	6.98E-17	-0.205866876	0.095	0.167	1.68E-12	1.1
TMEM51-A	7.01E-17	-0.135440412	0.006	0.041	1.69E-12	1.1
C1orf56	7.15E-17	-0.17084633	0.104	0.181	1.72E-12	1.1
ZNF814	7.57E-17	-0.165143664	0.05	0.108	1.82E-12	1.1
TMEM63A	7.63E-17	-0.189407764	0.033	0.084	1.84E-12	1.1
ANTXR1	7.79E-17	-0.17626747	0.023	0.068	1.88E-12	1.1
TOR1AIP2	7.99E-17	0.373999352	0.288	0.222	1.93E-12	1.1
RP11-1H15	8.05E-17	-0.154317203	0.046	0.104	1.94E-12	1.1
MUC16	8.15E-17	-0.178919311	0.019	0.063	1.96E-12	1.1
TYW1	8.26E-17	-0.151776789	0.042	0.097	1.99E-12	1.1
KIAA0368	8.39E-17	-0.197751333	0.163	0.253	2.02E-12	1.1
INPP5B	8.52E-17	-0.130966808	0.022	0.068	2.06E-12	1.1
SEC24B	8.59E-17	0.372087278	0.348	0.282	2.07E-12	1.1
MGAM	8.91E-17	-0.123378277	0.002	0.033	2.15E-12	1.1
DCAF13	9.15E-17	-0.153481394	0.043	0.099	2.21E-12	1.1
FAM126B	9.30E-17	0.372127357	0.206	0.141	2.24E-12	1.1
C7orf73	9.31E-17	-0.167675696	0.068	0.133	2.25E-12	1.1
PTPN9	9.35E-17	-0.157313179	0.035	0.087	2.25E-12	1.1
LCOR	9.65E-17	-0.185801431	0.098	0.172	2.33E-12	1.1
KMT5B	9.78E-17	-0.188110084	0.106	0.182	2.36E-12	1.1
RNPC3	9.80E-17	-0.171456084	0.038	0.091	2.36E-12	1.1
EFCAB14	1.01E-16	-0.164506043	0.033	0.084	2.44E-12	1.1
CTC-340D7	1.01E-16	-0.11199855	0.004	0.037	2.44E-12	1.1
RP11-93K2	1.02E-16	-0.100354297	0.002	0.032	2.46E-12	1.1
TPI1	1.04E-16	-0.260128815	0.295	0.4	2.52E-12	1.1
GTF2H2	1.09E-16	-0.129265038	0.015	0.056	2.62E-12	1.1
SLC4A7	1.09E-16	-0.2082597	0.257	0.363	2.62E-12	1.1
TMEM260	1.10E-16	-0.135314813	0.015	0.056	2.64E-12	1.1
PTPN14	1.14E-16	0.592274176	0.478	0.45	2.74E-12	1.1
DRAM2	1.14E-16	-0.165639112	0.217	0.318	2.75E-12	1.1
POLR2L	1.17E-16	-0.19527648	0.009	0.045	2.81E-12	1.1
PPP4R4	1.18E-16	-0.193510834	0.015	0.057	2.85E-12	1.1
B4GALT5	1.19E-16	-0.159960322	0.251	0.364	2.86E-12	1.1
COL22A1	1.19E-16	-0.148431138	0.008	0.044	2.86E-12	1.1
LIMS1	1.19E-16	0.510802666	0.359	0.301	2.87E-12	1.1
ADCY3	1.19E-16	-0.132818668	0.011	0.049	2.87E-12	1.1
BAZ1A	1.20E-16	-0.192681673	0.243	0.346	2.88E-12	1.1
MBNL2	1.22E-16	0.349315888	0.451	0.391	2.94E-12	1.1
RPL37A	1.22E-16	0.150143334	0.995	0.978	2.94E-12	1.1
TBC1D9	1.22E-16	-0.203996982	0.159	0.248	2.94E-12	1.1
CCDC64	1.22E-16	-0.137590009	0.008	0.043	2.95E-12	1.1
TAPT1-AS1	1.22E-16	-0.157506453	0.029	0.079	2.95E-12	1.1
TDRD3	1.24E-16	-0.189240281	0.038	0.09	3.00E-12	1.1

OR2A1-AS1	1.24E-16	-0.14331828	0.02	0.065	3.00E-12	1.1
RNPEP	1.29E-16	-0.137214279	0.023	0.068	3.11E-12	1.1
ZBTB46	1.31E-16	-0.142645023	0.03	0.079	3.15E-12	1.1
TSEN2	1.33E-16	-0.123862663	0.004	0.037	3.21E-12	1.1
BBS9	1.36E-16	-0.171680092	0.029	0.078	3.29E-12	1.1
MTERF4	1.37E-16	-0.163877585	0.045	0.101	3.29E-12	1.1
ZNF135	1.40E-16	-0.104968579	0.005	0.038	3.37E-12	1.1
NEK9	1.42E-16	-0.150752929	0.033	0.083	3.42E-12	1.1
TNFRSF10A	1.44E-16	-0.171894038	0.038	0.09	3.48E-12	1.1
ZNF630	1.48E-16	-0.124414351	0.005	0.038	3.56E-12	1.1
SNX19	1.48E-16	-0.129880573	0.016	0.057	3.58E-12	1.1
LRRC69	1.52E-16	-0.13146503	0.017	0.059	3.65E-12	1.1
HLA-E	1.54E-16	-0.247182512	0.072	0.136	3.71E-12	1.1
TMEM2	1.55E-16	-0.179086333	0.109	0.186	3.75E-12	1.1
OXR1	1.56E-16	-0.162191843	0.128	0.209	3.77E-12	1.1
CCDC170	1.59E-16	-0.139188398	0.015	0.055	3.82E-12	1.1
NHLRC2	1.62E-16	-0.147861525	0.029	0.078	3.91E-12	1.1
CLTC	1.64E-16	-0.176013599	0.143	0.228	3.95E-12	1.1
NOL3	1.70E-16	-0.142242465	0.019	0.062	4.10E-12	1.1
SRGAP2C	1.70E-16	-0.112855307	0.01	0.047	4.10E-12	1.1
SLC5A1	1.73E-16	-0.212961761	0.063	0.124	4.16E-12	1.1
WFDC10B	1.78E-16	-0.109770149	0.004	0.036	4.29E-12	1.1
CCDC93	1.79E-16	-0.157077707	0.071	0.137	4.33E-12	1.1
C3orf38	1.88E-16	-0.126183961	0.018	0.06	4.54E-12	1.1
IL12RB1	1.89E-16	-0.141645419	0.008	0.044	4.56E-12	1.1
S100A4	1.89E-16	-0.211446346	0.055	0.113	4.57E-12	1.1
FAM151B	1.93E-16	-0.140765085	0.017	0.059	4.65E-12	1.1
PIK3IP1-AS	1.95E-16	-0.155111913	0.02	0.063	4.71E-12	1.1
PLD3	1.98E-16	-0.13988676	0.015	0.056	4.78E-12	1.1
CALD1	1.99E-16	0.328542691	0.633	0.586	4.79E-12	1.1
SSBP1	2.01E-16	-0.207377851	0.358	0.473	4.84E-12	1.1
ZNF761	2.02E-16	-0.120677167	0.009	0.045	4.88E-12	1.1
LIPG	2.07E-16	-0.13462549	0.006	0.04	4.99E-12	1.1
VKORC1L1	2.07E-16	-0.139379491	0.021	0.065	5.00E-12	1.1
TCAIM	2.07E-16	-0.163466604	0.029	0.077	5.00E-12	1.1
RBM12B	2.10E-16	-0.132115714	0.008	0.043	5.06E-12	1.1
WWC1	2.10E-16	-0.268204892	0.371	0.483	5.07E-12	1.1
IFT88	2.12E-16	-0.159547941	0.021	0.066	5.11E-12	1.1
AVIL	2.17E-16	-0.143970697	0.017	0.059	5.24E-12	1.1
FAM73A	2.27E-16	-0.150304264	0.058	0.119	5.47E-12	1.1
WDR60	2.27E-16	-0.196845638	0.075	0.142	5.48E-12	1.1
KIAA0040	2.33E-16	-0.166191616	0.028	0.075	5.62E-12	1.1
CDC42EP1	2.44E-16	-0.186396495	0.023	0.068	5.88E-12	1.1
NAA20	2.56E-16	-0.168596946	0.08	0.148	6.16E-12	1.1
NDUFAF6	2.70E-16	-0.134021245	0.019	0.062	6.50E-12	1.1
C2	2.77E-16	-0.11991344	0.006	0.039	6.68E-12	1.1
SLC25A5	2.93E-16	-0.22148307	0.101	0.172	7.07E-12	1.1

SLC1A5	2.94E-16	-0.128938554	0.016	0.057	7.09E-12	1.1
METAP1D	3.03E-16	0.111598905	0.042	0.014	7.31E-12	1.1
FADS1	3.07E-16	-0.124100357	0.005	0.038	7.39E-12	1.1
LINC00657	3.07E-16	-0.164100977	0.1	0.173	7.41E-12	1.1
KALRN	3.09E-16	-0.149416464	0.08	0.148	7.45E-12	1.1
NFIA	3.10E-16	-0.22906941	0.08	0.146	7.47E-12	1.1
STC2	3.29E-16	-0.154312186	0.011	0.049	7.94E-12	1.1
MPZL2	3.30E-16	-0.164986361	0.079	0.146	7.96E-12	1.1
RP11-499P	3.42E-16	-0.141629891	0.012	0.05	8.24E-12	1.1
UGDH-AS1	3.46E-16	-0.121538679	0.019	0.062	8.33E-12	1.1
OSBPL3	3.47E-16	-0.142553112	0.027	0.074	8.37E-12	1.1
CLSTN1	3.50E-16	-0.148570822	0.039	0.091	8.45E-12	1.1
TTN-AS1	3.51E-16	-0.146649031	0.024	0.069	8.46E-12	1.1
ATP8B1	3.52E-16	0.595879536	0.368	0.319	8.50E-12	1.1
NRBF2	3.58E-16	-0.137311581	0.034	0.084	8.63E-12	1.1
IL27RA	3.73E-16	-0.12622061	0.012	0.05	8.98E-12	1.1
CLASP1	3.77E-16	-0.208939915	0.19	0.28	9.09E-12	1.1
RB1CC1	3.86E-16	-0.132933299	0.314	0.436	9.31E-12	1.1
KCTD13	4.05E-16	-0.116698482	0.008	0.043	9.77E-12	1.1
VPS8	4.14E-16	-0.170022574	0.044	0.099	9.98E-12	1.1
UPF2	4.15E-16	-0.1524057	0.213	0.311	1.00E-11	1.1
CLCN6	4.18E-16	-0.116701275	0.008	0.043	1.01E-11	1.1
TGFA	4.21E-16	-0.161076155	0.05	0.107	1.02E-11	1.1
VPS39	4.27E-16	-0.129397142	0.012	0.05	1.03E-11	1.1
TLE4	4.29E-16	0.430851879	0.442	0.392	1.03E-11	1.1
LINC00662	4.43E-16	-0.16517976	0.045	0.099	1.07E-11	1.1
NCOA2	4.52E-16	-0.200261917	0.403	0.533	1.09E-11	1.1
ERGIC2	4.59E-16	-0.181380443	0.146	0.23	1.11E-11	1.1
TRAPPC13	4.62E-16	-0.143583076	0.025	0.071	1.11E-11	1.1
IDO1	4.65E-16	-0.325042697	0.011	0.048	1.12E-11	1.1
CERS5	4.85E-16	-0.15454554	0.03	0.078	1.17E-11	1.1
ALS2CL	5.12E-16	-0.118075962	0.001	0.03	1.23E-11	1.1
RNASE1	5.30E-16	-0.178510959	0.013	0.051	1.28E-11	1.1
NDRG1	5.34E-16	0.674485907	0.475	0.447	1.29E-11	1.1
FSTL1	5.34E-16	-0.181923242	0.069	0.131	1.29E-11	1.1
RP5-1180E	5.63E-16	-0.121636007	0.011	0.048	1.36E-11	1.1
SLC23A2	5.70E-16	-0.146986305	0.029	0.076	1.37E-11	1.1
NIF3L1	5.72E-16	-0.140205906	0.028	0.076	1.38E-11	1.1
RAB11FIP3	5.75E-16	-0.186176144	0.04	0.093	1.39E-11	1.1
ATXN7L3B	6.25E-16	-0.142859752	0.022	0.065	1.51E-11	1.1
GRPEL1	6.27E-16	-0.123430554	0.027	0.073	1.51E-11	1.1
WDR33	6.42E-16	0.337247952	0.359	0.296	1.55E-11	1.1
RP11-759A	6.65E-16	-0.121128934	0.008	0.043	1.60E-11	1.1
KANSL1L	6.71E-16	-0.16728455	0.126	0.207	1.62E-11	1.1
NTN1	6.90E-16	-0.152348278	0.101	0.174	1.66E-11	1.1
KLF9	6.95E-16	-0.161598157	0.08	0.147	1.68E-11	1.1
ABL1	7.13E-16	-0.214001381	0.084	0.15	1.72E-11	1.1

NRDC	7.15E-16	-0.138545709	0.118	0.197	1.73E-11	1.1
PJA2	7.25E-16	0.371532419	0.278	0.211	1.75E-11	1.1
DNMBP	7.32E-16	-0.123191565	0.011	0.048	1.77E-11	1.1
LINC00866	7.37E-16	0.282280013	0.038	0.012	1.78E-11	1.1
GTF3C2	7.47E-16	-0.152820835	0.034	0.083	1.80E-11	1.1
PAN3	7.71E-16	0.37117322	0.581	0.573	1.86E-11	1.1
RAB1A	7.80E-16	0.383179775	0.481	0.445	1.88E-11	1.1
TBC1D4	7.80E-16	-0.23673434	0.092	0.16	1.88E-11	1.1
SLC15A1	7.95E-16	-0.129282714	0.008	0.042	1.92E-11	1.1
MPP6	8.26E-16	-0.155707607	0.038	0.088	1.99E-11	1.1
IPO9	8.90E-16	-0.147244963	0.035	0.085	2.14E-11	1.1
CCDC71L	9.03E-16	-0.111822515	0.013	0.05	2.18E-11	1.1
NRDE2	9.39E-16	-0.121917177	0.012	0.05	2.26E-11	1.1
TUBA1C	9.65E-16	-0.187872456	0.283	0.385	2.33E-11	1.1
CASC8	9.82E-16	0.207684243	0.128	0.075	2.37E-11	1.1
SLC37A1	9.83E-16	-0.129536431	0.02	0.062	2.37E-11	1.1
ETS1	9.85E-16	-0.177484657	0.09	0.16	2.38E-11	1.1
CDK5RAP2	9.90E-16	-0.143239871	0.027	0.073	2.39E-11	1.1
KLHL36	9.90E-16	-0.114425816	0.009	0.044	2.39E-11	1.1
SCLT1	1.05E-15	-0.16381108	0.018	0.058	2.53E-11	1.1
PLEKHM3	1.07E-15	-0.136417257	0.018	0.059	2.59E-11	1.1
ATP2C1	1.08E-15	-0.144920474	0.14	0.223	2.62E-11	1.1
SPOPL	1.09E-15	0.285517413	0.197	0.134	2.62E-11	1.1
VWA8	1.09E-15	-0.17400716	0.031	0.079	2.63E-11	1.1
CLDN8	1.11E-15	-0.107284847	0.047	0.102	2.67E-11	1.1
TBC1D10A	1.11E-15	0.692922359	0.157	0.102	2.67E-11	1.1
DST	1.12E-15	-0.160499377	0.501	0.628	2.70E-11	1.1
STAT5A	1.12E-15	-0.171004601	0.035	0.084	2.71E-11	1.1
PTP4A1	1.16E-15	0.459355439	0.258	0.193	2.79E-11	1.1
USP45	1.17E-15	-0.115329794	0.02	0.063	2.81E-11	1.1
CLEC1A	1.18E-15	-0.105074895	0.005	0.037	2.84E-11	1.1
STAT4	1.21E-15	-0.113995893	0.002	0.03	2.91E-11	1.1
CECR7	1.22E-15	-0.147474307	0.047	0.101	2.94E-11	1.1
NSMAF	1.25E-15	-0.162541835	0.052	0.108	3.01E-11	1.1
USP8	1.31E-15	-0.14554847	0.127	0.206	3.17E-11	1.1
ULK2	1.33E-15	-0.126998241	0.012	0.049	3.20E-11	1.1
NFX1	1.39E-15	-0.127275753	0.108	0.184	3.35E-11	1.1
RTCB	1.40E-15	0.364680856	0.482	0.441	3.38E-11	1.1
APTX	1.40E-15	-0.112799631	0.091	0.161	3.39E-11	1.1
CPSF2	1.43E-15	-0.134026006	0.018	0.059	3.44E-11	1.1
CCL5	1.44E-15	-0.155580371	0.003	0.033	3.47E-11	1.1
PDE7A	1.48E-15	-0.203145361	0.196	0.288	3.56E-11	1.1
CCDC126	1.48E-15	0.224418746	0.103	0.056	3.57E-11	1.1
GSKIP	1.49E-15	-0.10478394	0.018	0.059	3.60E-11	1.1
MTMR9	1.54E-15	-0.107809433	0.005	0.037	3.71E-11	1.1
MCU	1.55E-15	-0.162291509	0.05	0.105	3.74E-11	1.1
AASDH	1.57E-15	-0.120834146	0.024	0.068	3.79E-11	1.1

STEAP3	1.57E-15	-0.143861855	0.015	0.055	3.80E-11	1.1
ERICH1	1.58E-15	-0.146634928	0.013	0.05	3.80E-11	1.1
CCDC90B	1.60E-15	-0.141992859	0.055	0.113	3.87E-11	1.1
PGD	1.63E-15	-0.150997325	0.031	0.079	3.92E-11	1.1
MLLT10	1.64E-15	-0.159332877	0.11	0.185	3.96E-11	1.1
CMIP	1.66E-15	-0.14903449	0.28	0.389	3.99E-11	1.1
DUSP5	1.66E-15	-0.154993541	0.034	0.082	4.01E-11	1.1
STAU2	1.71E-15	-0.166021681	0.054	0.111	4.13E-11	1.1
CXorf23	1.72E-15	-0.123681769	0.023	0.067	4.16E-11	1.1
RNF19B	1.76E-15	-0.193715911	0.117	0.191	4.24E-11	1.1
REL	1.76E-15	0.390424138	0.283	0.221	4.24E-11	1.1
CBWD2	1.77E-15	-0.130941844	0.022	0.065	4.26E-11	1.1
HLA-C	1.81E-15	-0.191376979	0.169	0.255	4.35E-11	1.1
PHF20L1	1.82E-15	-0.201601014	0.084	0.149	4.40E-11	1.1
DPH6	1.85E-15	-0.183453017	0.034	0.082	4.47E-11	1.1
BRD8	1.94E-15	-0.167065534	0.035	0.083	4.67E-11	1.1
ARFGAP3	1.96E-15	-0.137922572	0.125	0.202	4.74E-11	1.1
CFLAR-AS1	2.00E-15	-0.135791823	0.045	0.099	4.81E-11	1.1
RAB14	2.00E-15	0.302585154	0.164	0.106	4.81E-11	1.1
ACAT2	2.01E-15	-0.265163264	0.223	0.315	4.85E-11	1.1
ARMT1	2.01E-15	-0.148825645	0.049	0.104	4.85E-11	1.1
RHOJ	2.07E-15	-0.108653088	0.009	0.043	4.98E-11	1.1
IDI1	2.08E-15	-0.12052717	0.152	0.238	5.02E-11	1.1
FAM84B	2.11E-15	-0.145907881	0.029	0.075	5.10E-11	1.1
C9orf84	2.17E-15	-0.117584591	0.008	0.042	5.23E-11	1.1
USO1	2.17E-15	-0.162298373	0.126	0.204	5.23E-11	1.1
PPP6R3	2.21E-15	0.376411393	0.459	0.419	5.32E-11	1.1
ZNF644	2.23E-15	-0.195058124	0.261	0.368	5.37E-11	1.1
FBXO2	2.24E-15	-0.110272671	0.003	0.032	5.41E-11	1.1
ZFP36L1	2.26E-15	-0.195957364	0.444	0.554	5.44E-11	1.1
CHD1	2.28E-15	0.384562335	0.28	0.215	5.49E-11	1.1
CAB39	2.28E-15	-0.200768879	0.209	0.302	5.50E-11	1.1
SLC25A17	2.30E-15	-0.139210563	0.022	0.065	5.56E-11	1.1
AC023590.	2.34E-15	-0.112618149	0.013	0.05	5.64E-11	1.1
CCNC	2.52E-15	-0.180590026	0.198	0.288	6.09E-11	1.1
WDR41	2.55E-15	-0.168446911	0.059	0.116	6.15E-11	1.1
IFNGR1	2.64E-15	-0.216148957	0.193	0.28	6.35E-11	1.1
ACAD10	2.78E-15	-0.105338327	0.006	0.038	6.71E-11	1.1
RP11-530C	2.83E-15	-0.140799231	0.015	0.053	6.82E-11	1.1
EIF1AX	2.88E-15	0.360490466	0.284	0.221	6.95E-11	1.1
PLXNA2	2.95E-15	-0.150047603	0.013	0.05	7.12E-11	1.1
LCMT1	2.99E-15	-0.143062008	0.038	0.089	7.21E-11	1.1
TRAPPC11	3.05E-15	-0.1442021	0.03	0.077	7.35E-11	1.1
HNRNPU-A	3.10E-15	-0.165976335	0.041	0.092	7.46E-11	1.1
FRMD3	3.11E-15	-0.184016899	0.051	0.107	7.51E-11	1.1
CASP8AP2	3.19E-15	-0.125818956	0.018	0.059	7.70E-11	1.1
RBM8A	3.33E-15	-0.152092339	0.423	0.542	8.02E-11	1.1

TNFAIP1	3.36E-15	-0.139847407	0.041	0.093	8.09E-11	1.1
CETN3	3.43E-15	-0.108866841	0.018	0.057	8.26E-11	1.1
HSF2	3.47E-15	0.248344864	0.12	0.071	8.36E-11	1.1
PSMD13	3.47E-15	-0.143223649	0.041	0.093	8.37E-11	1.1
PSMA3	3.50E-15	-0.275879769	0.365	0.466	8.43E-11	1.1
BET1L	3.52E-15	-0.108505377	0.013	0.05	8.48E-11	1.1
PPP1R12A	3.55E-15	-0.176748298	0.151	0.233	8.56E-11	1.1
RP11-415J8	3.65E-15	-0.143587301	0.028	0.073	8.79E-11	1.1
ZNFX1	3.66E-15	-0.162291957	0.085	0.151	8.82E-11	1.1
ANO6	3.81E-15	-0.146615467	0.272	0.383	9.19E-11	1.1
DNAJC11	3.85E-15	-0.144198083	0.02	0.062	9.29E-11	1.1
DNAJC9	3.95E-15	-0.150273255	0.027	0.071	9.53E-11	1.1
RP11-745L1	3.99E-15	-0.153326968	0.031	0.078	9.63E-11	1.1
HSD17B2	4.07E-15	-0.174257808	0.032	0.079	9.81E-11	1.1
C9orf72	4.19E-15	-0.151712496	0.046	0.099	1.01E-10	1.1
LINC01481	4.24E-15	-0.170520538	0.023	0.066	1.02E-10	1.1
TUBA1A	4.25E-15	-0.27542017	0.193	0.276	1.03E-10	1.1
FRAS1	4.30E-15	-0.18106723	0.03	0.077	1.04E-10	1.1
LINC01482	4.37E-15	-0.133944131	0.006	0.038	1.05E-10	1.1
MMP24-AS	4.46E-15	-0.12869142	0.015	0.054	1.08E-10	1.1
KDM1B	4.52E-15	-0.123863443	0.032	0.079	1.09E-10	1.1
REST	4.65E-15	-0.148629937	0.037	0.086	1.12E-10	1.1
RNF10	4.84E-15	0.276178342	0.216	0.153	1.17E-10	1.1
RPS29	4.84E-15	0.137001896	0.976	0.962	1.17E-10	1.1
CEP290	4.87E-15	-0.159023573	0.035	0.083	1.17E-10	1.1
PKNOX1	4.89E-15	-0.136070717	0.014	0.051	1.18E-10	1.1
RP11-519G	4.91E-15	-0.26692035	0.053	0.107	1.18E-10	1.1
NMRK1	4.97E-15	-0.103471921	0.007	0.039	1.20E-10	1.1
MCM9	5.01E-15	-0.136595395	0.015	0.052	1.21E-10	1.1
ARPC1B	5.03E-15	-0.111881985	0.006	0.038	1.21E-10	1.1
PIP4K2A	5.13E-15	-0.126575777	0.022	0.063	1.24E-10	1.1
FAM171A1	5.15E-15	-0.186571819	0.036	0.084	1.24E-10	1.1
ZCCHC9	5.17E-15	-0.128880935	0.031	0.077	1.25E-10	1.1
ZBTB7C	5.31E-15	-0.154593159	0.013	0.049	1.28E-10	1.1
MICAL3	5.33E-15	0.385140249	0.349	0.285	1.29E-10	1.1
SDK1	5.47E-15	-0.220785803	0.05	0.103	1.32E-10	1.1
HNRNPA3	5.48E-15	0.317011816	0.386	0.325	1.32E-10	1.1
TOM1L1	5.56E-15	-0.222746565	0.152	0.23	1.34E-10	1.1
GGNBP2	5.59E-15	-0.154381373	0.146	0.229	1.35E-10	1.1
SP3	5.63E-15	-0.147916959	0.1	0.171	1.36E-10	1.1
LINC00623	5.79E-15	-0.133421492	0.015	0.053	1.40E-10	1.1
SYCP3	5.85E-15	-0.132064027	0.008	0.04	1.41E-10	1.1
MBIP	5.94E-15	0.283744616	0.208	0.146	1.43E-10	1.1
HERPUD2	5.97E-15	-0.119967713	0.014	0.051	1.44E-10	1.1
RAB9A	5.98E-15	0.358018578	0.254	0.19	1.44E-10	1.1
KIF13A	6.03E-15	0.516743784	0.314	0.256	1.45E-10	1.1
TUBA1B	6.24E-15	-0.211971515	0.206	0.295	1.50E-10	1.1

TAB2	6.24E-15	0.360716275	0.316	0.254	1.50E-10	1.1
FAM234B	6.36E-15	0.155377331	0.055	0.023	1.53E-10	1.1
SLC9A3R1	6.40E-15	-0.143422735	0.048	0.101	1.54E-10	1.1
EPRS	6.41E-15	-0.18341339	0.101	0.17	1.55E-10	1.1
TMEM154	6.50E-15	-0.141381262	0.025	0.069	1.57E-10	1.1
IGFBP7	6.51E-15	-0.152089293	0.003	0.032	1.57E-10	1.1
TRIP12	6.54E-15	-0.162377664	0.207	0.303	1.58E-10	1.1
CCM2	6.55E-15	-0.128097158	0.014	0.051	1.58E-10	1.1
KCND2	6.75E-15	-0.275503684	0.049	0.102	1.63E-10	1.1
APPBP2	6.77E-15	-0.17963048	0.066	0.125	1.63E-10	1.1
HADH	6.80E-15	0.44183017	0.251	0.189	1.64E-10	1.1
C5orf42	6.82E-15	-0.127179862	0.018	0.057	1.65E-10	1.1
SLC25A46	6.89E-15	-0.129613405	0.013	0.05	1.66E-10	1.1
PACRGL	6.98E-15	-0.111763475	0.012	0.047	1.68E-10	1.1
TMEM135	6.99E-15	-0.158686694	0.04	0.091	1.69E-10	1.1
EHMT1	7.26E-15	-0.153256001	0.116	0.192	1.75E-10	1.1
TCHP	7.41E-15	-0.163169065	0.014	0.05	1.79E-10	1.1
ZNF213	7.45E-15	-0.120309723	0.009	0.042	1.80E-10	1.1
EDA	7.45E-15	-0.160123455	0.03	0.075	1.80E-10	1.1
PIGN	7.49E-15	-0.158511488	0.067	0.126	1.81E-10	1.1
DNTTIP2	7.77E-15	-0.141768576	0.155	0.238	1.87E-10	1.1
KCNC4	7.92E-15	-0.124656865	0.01	0.043	1.91E-10	1.1
DTNBP1	8.10E-15	-0.15530343	0.025	0.069	1.95E-10	1.1
COX4I1	8.30E-15	0.292891753	0.77	0.789	2.00E-10	1.1
GRB10	8.48E-15	-0.20098892	0.089	0.154	2.04E-10	1.1
RGCC	8.77E-15	0.188310716	0.084	0.043	2.11E-10	1.1
TMEM167/	9.02E-15	-0.204199061	0.203	0.29	2.17E-10	1.1
ATL2	9.03E-15	-0.201885391	0.216	0.309	2.18E-10	1.1
C2CD2	9.12E-15	-0.13151852	0.021	0.062	2.20E-10	1.1
MED27	9.20E-15	-0.140424621	0.028	0.073	2.22E-10	1.1
MKNK1	9.22E-15	-0.113233169	0.019	0.059	2.22E-10	1.1
LIMK2	9.35E-15	-0.191661429	0.082	0.145	2.26E-10	1.1
MRPL24	9.65E-15	0.218975008	0.094	0.051	2.33E-10	1.1
CUTC	9.71E-15	-0.137757378	0.023	0.065	2.34E-10	1.1
PRMT3	1.05E-14	-0.13085622	0.009	0.042	2.53E-10	1.1
C14orf159	1.05E-14	-0.139744727	0.033	0.08	2.53E-10	1.1
HMBOX1	1.05E-14	-0.149987764	0.158	0.242	2.53E-10	1.1
CALU	1.08E-14	-0.164479489	0.224	0.318	2.60E-10	1.1
PRDM2	1.10E-14	0.324138501	0.358	0.297	2.66E-10	1.1
SLC44A1	1.12E-14	-0.118281296	0.051	0.106	2.70E-10	1.1
GNPDA2	1.15E-14	-0.104951376	0.012	0.048	2.77E-10	1.1
MACROD2	1.20E-14	-0.216612618	0.198	0.285	2.89E-10	1.1
RP11-77K1	1.22E-14	-0.10654807	0.008	0.041	2.94E-10	1.1
SFI1	1.22E-14	-0.124967265	0.023	0.065	2.95E-10	1.1
ITGB6	1.24E-14	0.408257747	0.36	0.299	3.00E-10	1.1
TMEM59	1.25E-14	-0.152586244	0.265	0.367	3.01E-10	1.1
RP11-37B2	1.28E-14	-0.18156598	0.225	0.317	3.08E-10	1.1

HSBP1	1.30E-14	-0.150844841	0.396	0.514	3.14E-10	1.1
LINC00324	1.31E-14	0.130848576	0.043	0.016	3.16E-10	1.1
VAT1	1.33E-14	-0.166090529	0.076	0.137	3.21E-10	1.1
NUP50-AS1	1.33E-14	-0.120096137	0.007	0.038	3.22E-10	1.1
AP1S3	1.35E-14	0.257878314	0.108	0.062	3.25E-10	1.1
LINC01549	1.35E-14	-0.230023197	0.011	0.045	3.25E-10	1.1
CH507-528	1.35E-14	-0.106146258	0.008	0.04	3.27E-10	1.1
RHOT1	1.37E-14	-0.1496559	0.053	0.107	3.32E-10	1.1
KCTD1	1.40E-14	-0.119977149	0.018	0.056	3.38E-10	1.1
KDM4C	1.41E-14	-0.12262602	0.034	0.081	3.40E-10	1.1
PDP1	1.41E-14	-0.156606515	0.05	0.102	3.41E-10	1.1
TUBD1	1.44E-14	-0.100359282	0.02	0.059	3.46E-10	1.1
DIAPH2-AS	1.48E-14	-0.116491666	0.013	0.049	3.57E-10	1.1
RP11-1069	1.52E-14	-0.114251071	0.008	0.04	3.66E-10	1.1
KLHL28	1.52E-14	-0.127861027	0.04	0.089	3.68E-10	1.1
ZNF621	1.53E-14	-0.136150856	0.025	0.068	3.70E-10	1.1
CYP4A22-A	1.54E-14	-0.100181811	0.003	0.03	3.72E-10	1.1
URI1	1.55E-14	-0.165286982	0.093	0.159	3.74E-10	1.1
SH3RF3	1.57E-14	-0.104082832	0.004	0.032	3.77E-10	1.1
RAB11FIP2	1.58E-14	-0.129450397	0.013	0.048	3.80E-10	1.1
EIF4EBP2	1.60E-14	-0.157423446	0.074	0.134	3.85E-10	1.1
ZNF26	1.60E-14	-0.115966653	0.014	0.05	3.85E-10	1.1
BEX5	1.63E-14	0.153073951	0.055	0.023	3.92E-10	1.1
CCDC34	1.65E-14	0.105113438	0.037	0.012	3.97E-10	1.1
CDK19	1.65E-14	-0.131868978	0.199	0.29	3.98E-10	1.1
BAMBI	1.70E-14	-0.138014121	0.042	0.092	4.09E-10	1.1
ADAMTS4	1.71E-14	-0.135799876	0.003	0.031	4.12E-10	1.1
MACF1	1.73E-14	0.377143032	0.639	0.632	4.17E-10	1.1
SPATA6	1.75E-14	-0.130618167	0.013	0.049	4.21E-10	1.1
C1RL-AS1	1.78E-14	-0.116232724	0.007	0.038	4.28E-10	1.1
PVT1	1.81E-14	0.307872505	0.43	0.375	4.36E-10	1.1
GHITM	1.81E-14	-0.246654679	0.409	0.507	4.37E-10	1.1
RP11-386I1	1.87E-14	0.157136162	0.055	0.023	4.51E-10	1.1
AL592183.1	1.88E-14	-0.152795293	0.049	0.101	4.54E-10	1.1
MRPL19	1.94E-14	-0.132056059	0.028	0.071	4.68E-10	1.1
SNTB2	1.96E-14	-0.104629781	0.005	0.033	4.72E-10	1.1
TBC1D32	1.98E-14	-0.179711826	0.024	0.066	4.78E-10	1.1
USP44	2.02E-14	-0.113280101	0.008	0.04	4.87E-10	1.1
ZNRF1	2.10E-14	-0.127263109	0.049	0.101	5.06E-10	1.1
ZMYND11	2.13E-14	-0.130559514	0.068	0.128	5.13E-10	1.1
CASZ1	2.17E-14	0.168002142	0.089	0.047	5.23E-10	1.1
FAM172A	2.20E-14	0.375654292	0.75	0.747	5.31E-10	1.1
MSL1	2.21E-14	-0.132621668	0.047	0.098	5.32E-10	1.1
PPP3CB	2.23E-14	-0.140033255	0.045	0.096	5.37E-10	1.1
ST7L	2.24E-14	-0.117987671	0.02	0.06	5.41E-10	1.1
CAMTA1	2.27E-14	0.379419054	0.548	0.514	5.48E-10	1.1
PRKD3	2.36E-14	-0.134604436	0.093	0.16	5.70E-10	1.1

ACOX1	2.37E-14	-0.120998741	0.018	0.056	5.72E-10	1.1
RP11-544A	2.44E-14	-0.134162586	0.022	0.062	5.87E-10	1.1
ZNF565	2.44E-14	-0.165672845	0.123	0.196	5.87E-10	1.1
PKN2	2.47E-14	-0.153849135	0.185	0.274	5.95E-10	1.1
NSA2	2.51E-14	0.331393537	0.451	0.407	6.06E-10	1.1
DCTN2	2.57E-14	-0.150471265	0.081	0.143	6.20E-10	1.1
SP140L	2.58E-14	-0.13988892	0.019	0.058	6.23E-10	1.1
MASTL	2.59E-14	-0.120698529	0.022	0.062	6.26E-10	1.1
CYB5B	2.60E-14	-0.132313078	0.112	0.182	6.28E-10	1.1
LNX1	2.67E-14	-0.174982095	0.055	0.108	6.45E-10	1.1
TM9SF3	2.68E-14	0.339317106	0.324	0.265	6.47E-10	1.1
TMOD3	2.71E-14	-0.159068859	0.218	0.312	6.54E-10	1.1
PDE6D	2.75E-14	-0.135186959	0.015	0.051	6.64E-10	1.1
MIS12	2.76E-14	-0.113143975	0.013	0.048	6.66E-10	1.1
PRPF40B	2.91E-14	-0.139033924	0.011	0.045	7.01E-10	1.1
SLC25A6	2.91E-14	-0.274598855	0.093	0.155	7.01E-10	1.1
MAT2B	2.99E-14	-0.12371155	0.023	0.064	7.21E-10	1.1
XRCC5	3.01E-14	-0.185312139	0.279	0.38	7.26E-10	1.1
WDR27	3.01E-14	-0.127961487	0.016	0.053	7.26E-10	1.1
GLYATL2	3.08E-14	-0.108265166	0.038	0.087	7.42E-10	1.1
PCLO	3.09E-14	-0.171469074	0.018	0.055	7.45E-10	1.1
ZC4H2	3.11E-14	-0.119592384	0.012	0.046	7.49E-10	1.1
TFAP2C	3.12E-14	-0.159436258	0.078	0.14	7.52E-10	1.1
GALNT10	3.12E-14	-0.125837978	0.013	0.048	7.53E-10	1.1
KDM2B	3.14E-14	-0.134624172	0.021	0.06	7.56E-10	1.1
SLC25A43	3.17E-14	-0.126947811	0.023	0.063	7.65E-10	1.1
DSTYK	3.18E-14	-0.116135943	0.03	0.074	7.67E-10	1.1
ZSCAN5A	3.23E-14	-0.11398196	0.01	0.042	7.78E-10	1.1
CNNM2	3.34E-14	-0.132571953	0.024	0.066	8.05E-10	1.1
VPS37A	3.37E-14	-0.148495818	0.029	0.073	8.13E-10	1.1
TAF1	3.56E-14	-0.151404365	0.083	0.146	8.58E-10	1.1
APLP2	3.65E-14	-0.133320127	0.083	0.147	8.79E-10	1.1
AC027119.	3.71E-14	-0.105649242	0.001	0.025	8.94E-10	1.1
EEPD1	3.71E-14	0.218077468	0.086	0.046	8.96E-10	1.1
RPS12	3.79E-14	0.459446833	0.717	0.742	9.13E-10	1.1
STXBP3	3.80E-14	-0.133156871	0.065	0.123	9.15E-10	1.1
TRIM47	3.89E-14	-0.104572289	0.003	0.03	9.38E-10	1.1
C21orf62-A	3.90E-14	-0.107470352	0.008	0.04	9.40E-10	1.1
ARHGEF7	3.90E-14	0.326028975	0.217	0.157	9.40E-10	1.1
SFT2D1	3.90E-14	-0.155583369	0.03	0.074	9.41E-10	1.1
SOCS6	4.03E-14	-0.146690554	0.078	0.139	9.72E-10	1.1
MAVS	4.05E-14	-0.133979423	0.028	0.071	9.76E-10	1.1
ASAP1	4.15E-14	-0.135285617	0.179	0.265	1.00E-09	1.1
BAZ1B	4.28E-14	-0.125471039	0.103	0.171	1.03E-09	1.1
TRAPPC12	4.29E-14	-0.109140662	0.008	0.038	1.04E-09	1.1
DFFA	4.31E-14	-0.128188309	0.03	0.074	1.04E-09	1.1
DENND4C	4.36E-14	-0.190542573	0.156	0.236	1.05E-09	1.1

TNFRSF21	4.42E-14	-0.163266733	0.035	0.08	1.07E-09	1.1
RNF14	4.53E-14	-0.106025538	0.015	0.051	1.09E-09	1.1
XDH	4.54E-14	-0.265038252	0.073	0.132	1.09E-09	1.1
CTDSPL	4.64E-14	-0.140536743	0.031	0.075	1.12E-09	1.1
PTGS2	4.66E-14	-0.259178707	0.005	0.033	1.12E-09	1.1
CSRNP1	4.71E-14	0.119874148	0.049	0.02	1.14E-09	1.1
USP4	4.81E-14	-0.151678853	0.053	0.106	1.16E-09	1.1
MT1E	4.88E-14	-0.437893531	0.015	0.05	1.18E-09	1.1
METTL3	4.88E-14	-0.13351993	0.022	0.062	1.18E-09	1.1
AKNA	4.96E-14	-0.107006354	0.007	0.037	1.20E-09	1.1
TRMT10C	5.03E-14	-0.142420208	0.082	0.144	1.21E-09	1.1
GNPTAB	5.07E-14	-0.144618289	0.068	0.127	1.22E-09	1.1
PARP9	5.15E-14	-0.116576322	0.024	0.065	1.24E-09	1.1
CCBL2	5.36E-14	-0.133412253	0.076	0.137	1.29E-09	1.1
TAP1	5.43E-14	-0.117979564	0.008	0.039	1.31E-09	1.1
ABHD5	5.47E-14	-0.106813344	0.11	0.181	1.32E-09	1.1
CD24	5.48E-14	-0.271309443	0.5	0.594	1.32E-09	1.1
HNRNPH2	5.49E-14	-0.119744279	0.164	0.248	1.32E-09	1.1
SERINC3	5.65E-14	-0.168323616	0.129	0.201	1.36E-09	1.1
KITLG	5.69E-14	-0.101650529	0.005	0.033	1.37E-09	1.1
CEBPD	5.75E-14	-0.167660804	0.162	0.242	1.39E-09	1.1
PIGP	5.79E-14	0.273344106	0.204	0.146	1.40E-09	1.1
CHDH	5.79E-14	-0.153695514	0.024	0.065	1.40E-09	1.1
PCMTD1	5.90E-14	-0.197870849	0.246	0.339	1.42E-09	1.1
SDAD1	5.96E-14	-0.161351907	0.05	0.102	1.44E-09	1.1
FMNL2	6.00E-14	-0.102934506	0.35	0.474	1.45E-09	1.1
SLC35F3	6.02E-14	-0.157766436	0.023	0.063	1.45E-09	1.1
ZNF800	6.06E-14	-0.128274524	0.046	0.096	1.46E-09	1.1
MRPL35	6.06E-14	-0.11129956	0.028	0.07	1.46E-09	1.1
RNF115	6.40E-14	0.485571422	0.28	0.225	1.54E-09	1.1
TBC1D16	6.41E-14	-0.12688874	0.015	0.05	1.54E-09	1.1
INTS8	6.45E-14	-0.104683056	0.01	0.042	1.56E-09	1.1
KCNJ2	6.46E-14	-0.101218169	0.004	0.031	1.56E-09	1.1
ZNF385D	6.47E-14	-0.176388236	0.033	0.078	1.56E-09	1.1
S100A7	6.71E-14	-0.602699932	0.007	0.037	1.62E-09	1.1
IQCB1	7.17E-14	-0.176060429	0.059	0.113	1.73E-09	1.1
TMEM126/	7.24E-14	0.237963057	0.164	0.11	1.75E-09	1.1
RIN3	7.27E-14	-0.119031116	0.013	0.047	1.75E-09	1.1
SERPINA1	7.33E-14	-0.149509821	0.011	0.044	1.77E-09	1.1
RAB7A	7.36E-14	0.353662538	0.424	0.377	1.78E-09	1.1
ZNF677	7.40E-14	-0.117993488	0.025	0.066	1.78E-09	1.1
ADPGK	7.52E-14	-0.138738906	0.024	0.065	1.81E-09	1.1
AP3B1	7.57E-14	-0.174805109	0.133	0.205	1.82E-09	1.1
ZNRF3	7.62E-14	-0.120690109	0.014	0.049	1.84E-09	1.1
UBQLN1	7.94E-14	0.286988071	0.295	0.233	1.91E-09	1.1
ABHD12	7.99E-14	-0.149828706	0.014	0.048	1.93E-09	1.1
HCG17	8.19E-14	-0.14276733	0.035	0.08	1.98E-09	1.1

PLAU	8.37E-14	-0.113844975	0.007	0.037	2.02E-09	1.1
FCF1	8.42E-14	-0.130892475	0.031	0.075	2.03E-09	1.1
GSTP1	8.50E-14	-0.280706751	0.043	0.09	2.05E-09	1.1
YWHAQ	8.51E-14	0.42041352	0.411	0.362	2.05E-09	1.1
ARL6IP6	8.53E-14	-0.122459688	0.024	0.064	2.06E-09	1.1
MPHOSPH9	8.56E-14	-0.112334178	0.014	0.048	2.07E-09	1.1
NUDT2	8.68E-14	0.264797101	0.184	0.129	2.09E-09	1.1
FOXK1	8.70E-14	0.256359817	0.19	0.132	2.10E-09	1.1
ELMOD2	9.12E-14	-0.132129704	0.026	0.068	2.20E-09	1.1
UCK2	9.13E-14	-0.217971591	0.12	0.189	2.20E-09	1.1
NKTR	9.26E-14	-0.173573899	0.235	0.328	2.23E-09	1.1
MTCH2	9.48E-14	-0.142498717	0.04	0.087	2.29E-09	1.1
ZNF169	9.49E-14	-0.104685751	0.007	0.036	2.29E-09	1.1
RPRD1A	9.56E-14	-0.157338537	0.08	0.14	2.31E-09	1.1
ATRX	9.64E-14	-0.117202476	0.352	0.461	2.32E-09	1.1
MBD4	9.68E-14	-0.118198391	0.02	0.058	2.33E-09	1.1
RBM5	9.72E-14	-0.18131719	0.108	0.176	2.34E-09	1.1
SRGAP3	1.00E-13	-0.154388374	0.033	0.078	2.41E-09	1.1
DNAJA1	1.00E-13	-0.118663233	0.185	0.27	2.42E-09	1.1
OLMALINC	1.01E-13	-0.111919659	0.015	0.051	2.43E-09	1.1
EFNA1	1.01E-13	-0.165712717	0.07	0.126	2.44E-09	1.1
RPGR	1.02E-13	-0.139548018	0.026	0.067	2.45E-09	1.1
INIP	1.02E-13	-0.104249034	0.029	0.072	2.45E-09	1.1
NABP1	1.02E-13	-0.169234179	0.035	0.08	2.46E-09	1.1
ETV6	1.04E-13	0.255891307	0.684	0.677	2.50E-09	1.1
RNF216	1.05E-13	-0.149283379	0.094	0.159	2.52E-09	1.1
IQGAP1	1.05E-13	0.314420164	0.469	0.421	2.54E-09	1.1
SERGEF	1.06E-13	-0.127346594	0.011	0.044	2.55E-09	1.1
AGPAT3	1.06E-13	-0.129622937	0.036	0.081	2.56E-09	1.1
OGT	1.07E-13	0.362799736	0.406	0.355	2.58E-09	1.1
SNIP1	1.07E-13	0.142057335	0.062	0.029	2.58E-09	1.1
ELMO1	1.07E-13	-0.106412024	0.007	0.036	2.58E-09	1.1
SUPT16H	1.08E-13	-0.121913838	0.04	0.087	2.59E-09	1.1
TMEM263	1.08E-13	0.213424469	0.145	0.092	2.61E-09	1.1
CDC42EP5	1.12E-13	-0.108297254	0.01	0.042	2.69E-09	1.1
KANSL1	1.14E-13	-0.186583509	0.307	0.409	2.74E-09	1.1
OVOL2	1.15E-13	-0.136175233	0.033	0.077	2.78E-09	1.1
ZNF133	1.15E-13	-0.10168333	0.011	0.044	2.78E-09	1.1
GLB1	1.18E-13	-0.102741249	0.012	0.045	2.85E-09	1.1
ZNF440	1.19E-13	-0.11177314	0.01	0.042	2.88E-09	1.1
DYRK2	1.19E-13	0.139198125	0.06	0.027	2.88E-09	1.1
METTL16	1.20E-13	-0.164748449	0.133	0.206	2.88E-09	1.1
FARP2	1.20E-13	-0.144470941	0.045	0.095	2.90E-09	1.1
BTRC	1.21E-13	-0.153271205	0.085	0.147	2.91E-09	1.1
XPNPEP1	1.22E-13	-0.147793019	0.085	0.146	2.94E-09	1.1
DPY30	1.25E-13	-0.14812019	0.114	0.184	3.01E-09	1.1
SLC25A24	1.25E-13	-0.113864969	0.03	0.073	3.01E-09	1.1

ANXA1	1.26E-13	0.110101667	0.889	0.913	3.03E-09	1.1
POLN	1.28E-13	-0.133457803	0.01	0.042	3.08E-09	1.1
KDM3A	1.28E-13	0.251227066	0.148	0.095	3.09E-09	1.1
DNMT3A	1.28E-13	-0.14556786	0.03	0.072	3.10E-09	1.1
H2AFJ	1.32E-13	-0.138906206	0.011	0.044	3.19E-09	1.1
SPATS2L	1.33E-13	-0.18874776	0.213	0.3	3.21E-09	1.1
NCOA5	1.33E-13	-0.132311006	0.016	0.052	3.21E-09	1.1
TMEM41B	1.37E-13	0.366480006	0.239	0.182	3.30E-09	1.1
NHSL1	1.37E-13	-0.104247	0.147	0.223	3.31E-09	1.1
KIFC3	1.39E-13	-0.117264573	0.017	0.053	3.35E-09	1.1
PCID2	1.41E-13	-0.141549151	0.073	0.13	3.39E-09	1.1
PC	1.42E-13	-0.102337701	0.007	0.037	3.42E-09	1.1
STX7	1.44E-13	-0.170275865	0.11	0.177	3.48E-09	1.1
TRIOBP	1.47E-13	-0.118071648	0.014	0.048	3.53E-09	1.1
SRSF6	1.47E-13	-0.147307092	0.049	0.099	3.55E-09	1.1
RP11-613M	1.48E-13	-0.132176367	0.033	0.077	3.56E-09	1.1
ANKRA2	1.52E-13	-0.125493625	0.034	0.079	3.65E-09	1.1
SDK2	1.53E-13	0.327728523	0.101	0.058	3.69E-09	1.1
SLC35D2	1.53E-13	-0.128400653	0.032	0.076	3.69E-09	1.1
RP11-779O	1.55E-13	0.396328474	0.259	0.199	3.75E-09	1.1
AP1B1	1.57E-13	-0.124104454	0.011	0.044	3.78E-09	1.1
TNRC6B	1.59E-13	-0.123046492	0.334	0.449	3.82E-09	1.1
TAF2	1.61E-13	-0.134876064	0.045	0.094	3.88E-09	1.1
IKZF2	1.69E-13	-0.146463442	0.02	0.057	4.07E-09	1.1
RERG	1.72E-13	-0.225594863	0.108	0.175	4.14E-09	1.1
IDH3A	1.72E-13	-0.120789419	0.042	0.089	4.14E-09	1.1
CTTN	1.72E-13	-0.184547671	0.123	0.194	4.16E-09	1.1
PSORS1C1	1.73E-13	-0.106440106	0.021	0.06	4.17E-09	1.1
DHRS3	1.73E-13	-0.1812003	0.054	0.105	4.17E-09	1.1
RP11-353M	1.75E-13	-0.115651035	0.003	0.029	4.22E-09	1.1
FOXJ3	1.75E-13	0.374816353	0.335	0.28	4.22E-09	1.1
PDE9A	1.77E-13	-0.142816877	0.041	0.088	4.26E-09	1.1
ELP2	1.84E-13	0.27758429	0.211	0.152	4.43E-09	1.1
RAP2B	1.85E-13	0.388409007	0.232	0.176	4.47E-09	1.1
TAF1B	1.87E-13	-0.118502502	0.056	0.108	4.50E-09	1.1
IGF2BP2-AS	1.87E-13	0.187507413	0.074	0.038	4.50E-09	1.1
LRRC37A2	1.87E-13	-0.103255215	0.013	0.047	4.51E-09	1.1
ICE2	1.88E-13	-0.113841219	0.023	0.061	4.54E-09	1.1
RBAK-RBAK	1.94E-13	-0.142649313	0.03	0.073	4.68E-09	1.1
GPATCH2	2.00E-13	-0.115159959	0.037	0.083	4.83E-09	1.1
C1orf21	2.01E-13	0.405214601	0.268	0.209	4.85E-09	1.1
MRPS5	2.04E-13	-0.113452495	0.027	0.068	4.93E-09	1.1
AK9	2.06E-13	-0.133465095	0.028	0.07	4.97E-09	1.1
RPL23A	2.13E-13	0.35854628	0.707	0.729	5.15E-09	1.1
MFSD14A	2.14E-13	0.275967742	0.176	0.122	5.16E-09	1.1
RP11-91P2	2.17E-13	-0.128500956	0.02	0.057	5.22E-09	1.1
SETX	2.18E-13	-0.118607144	0.112	0.182	5.26E-09	1.1

BTBD10	2.21E-13	-0.150631698	0.033	0.075	5.32E-09	1.1
AC097721.	2.25E-13	-0.102091333	0.005	0.033	5.42E-09	1.1
ACVR2A	2.26E-13	-0.135001127	0.022	0.06	5.44E-09	1.1
ATXN2	2.27E-13	-0.143362589	0.214	0.306	5.46E-09	1.1
IL10RB	2.28E-13	-0.113744178	0.011	0.043	5.49E-09	1.1
BROX	2.30E-13	-0.124842612	0.09	0.153	5.54E-09	1.1
PSMC1	2.33E-13	-0.187109291	0.291	0.388	5.61E-09	1.1
DNM2	2.37E-13	-0.112283385	0.037	0.083	5.71E-09	1.1
TIMM17A	2.37E-13	-0.124822928	0.162	0.243	5.72E-09	1.1
HMGCR	2.51E-13	-0.124340047	0.032	0.075	6.05E-09	1.1
TNFRSF11A	2.52E-13	0.340668822	0.172	0.118	6.07E-09	1.1
GSN	2.62E-13	-0.197569423	0.133	0.202	6.31E-09	1.1
VPS41	2.62E-13	-0.148940425	0.045	0.093	6.33E-09	1.1
PSMC2	2.62E-13	-0.155444205	0.221	0.309	6.33E-09	1.1
GPALPP1	2.63E-13	-0.103784608	0.015	0.05	6.33E-09	1.1
TMOD1	2.65E-13	-0.160131304	0.083	0.142	6.38E-09	1.1
NDUFAF1	2.68E-13	-0.116898166	0.025	0.064	6.47E-09	1.1
PANK3	2.69E-13	-0.13834429	0.053	0.104	6.49E-09	1.1
DDX23	2.71E-13	-0.120645761	0.02	0.057	6.53E-09	1.1
TIMMDC1	2.75E-13	-0.116357461	0.024	0.063	6.63E-09	1.1
RBMS1	2.77E-13	-0.113339333	0.201	0.29	6.67E-09	1.1
GTF2H2C	2.79E-13	-0.132274819	0.028	0.068	6.72E-09	1.1
GATAD1	2.81E-13	-0.11731198	0.015	0.049	6.78E-09	1.1
WDR89	2.82E-13	-0.106381128	0.011	0.042	6.80E-09	1.1
GATSL2	2.82E-13	0.100052968	0.044	0.017	6.81E-09	1.1
SMARCC1	2.92E-13	-0.163789385	0.19	0.275	7.03E-09	1.1
ARRB1	2.95E-13	-0.101653667	0.005	0.033	7.11E-09	1.1
ZNF264	2.95E-13	-0.122701291	0.028	0.068	7.12E-09	1.1
POU2F3	3.05E-13	-0.185694619	0.119	0.186	7.35E-09	1.1
PCGF5	3.06E-13	0.335462258	0.223	0.166	7.38E-09	1.1
SLC1A3	3.06E-13	-0.121793171	0.023	0.062	7.38E-09	1.1
CARS	3.10E-13	-0.159882476	0.054	0.105	7.47E-09	1.1
TRAPPC3	3.11E-13	-0.129552021	0.04	0.086	7.50E-09	1.1
ENTPD5	3.15E-13	-0.114709109	0.012	0.044	7.59E-09	1.1
SLC27A1	3.15E-13	-0.111858128	0.022	0.06	7.59E-09	1.1
CIR1	3.31E-13	-0.123812566	0.226	0.321	7.98E-09	1.1
LRRK1	3.40E-13	-0.139190687	0.048	0.096	8.19E-09	1.1
UGT2B7	3.41E-13	-0.110049269	0.022	0.06	8.22E-09	1.1
UFD1L	3.42E-13	-0.126340379	0.037	0.081	8.24E-09	1.1
SCRN1	3.52E-13	-0.142594194	0.033	0.076	8.48E-09	1.1
KAZN	3.72E-13	-0.135422193	0.1	0.166	8.98E-09	1.1
C15orf57	3.79E-13	-0.114232519	0.037	0.081	9.15E-09	1.1
ZMYM4-AS	3.87E-13	-0.104912925	0.018	0.054	9.34E-09	1.1
UBE3B	4.07E-13	-0.109767304	0.014	0.048	9.81E-09	1.1
PLA2G4A	4.08E-13	-0.14528851	0.022	0.059	9.84E-09	1.1
WBP2	4.10E-13	-0.144953216	0.025	0.063	9.89E-09	1.1
ZMYM1	4.17E-13	-0.114868146	0.027	0.067	1.00E-08	1.1

FOSB	4.22E-13	0.385708177	0.458	0.414	1.02E-08	1.1
ZCCHC6	4.43E-13	-0.145501192	0.274	0.371	1.07E-08	1.1
RP11-289H	4.47E-13	-0.105369019	0.019	0.055	1.08E-08	1.1
UBP1	4.57E-13	-0.125450505	0.039	0.084	1.10E-08	1.1
RALGPS1	4.65E-13	-0.1113465	0.019	0.055	1.12E-08	1.1
CAMSAP2	4.71E-13	-0.150328613	0.084	0.144	1.14E-08	1.1
CTD-3252C	4.72E-13	-0.145540771	0.053	0.103	1.14E-08	1.1
DLG3	4.75E-13	-0.119182137	0.027	0.067	1.15E-08	1.1
TCN1	4.79E-13	-0.194062315	0.012	0.044	1.15E-08	1.1
AC018890.	4.79E-13	-0.194550329	0.126	0.195	1.16E-08	1.1
RP11-306I1	4.84E-13	0.100223369	0.03	0.009	1.17E-08	1.1
SLC35B3	4.88E-13	-0.108897207	0.013	0.045	1.18E-08	1.1
FAM188A	4.96E-13	-0.10825947	0.011	0.043	1.20E-08	1.1
MCEE	4.99E-13	-0.104694714	0.011	0.042	1.20E-08	1.1
AES	5.08E-13	-0.107915611	0.013	0.045	1.23E-08	1.1
RP11-138A	5.10E-13	-0.13453166	0.035	0.079	1.23E-08	1.1
LINC00486	5.13E-13	-0.109801432	0.988	0.995	1.24E-08	1.1
CTGF	5.45E-13	0.295769768	0.058	0.026	1.31E-08	1.1
CEP162	5.51E-13	-0.110541888	0.015	0.049	1.33E-08	1.1
ZNF880	5.52E-13	-0.110500643	0.015	0.048	1.33E-08	1.1
HEY2	5.54E-13	0.143939647	0.058	0.026	1.34E-08	1.1
NDUFS2	5.56E-13	-0.129766784	0.026	0.066	1.34E-08	1.1
STK4	5.56E-13	-0.121685073	0.02	0.057	1.34E-08	1.1
MCFD2	5.58E-13	-0.123850652	0.108	0.173	1.35E-08	1.1
ALDOA	5.85E-13	-0.316997225	0.08	0.136	1.41E-08	1.1
SRGN	6.08E-13	-0.313299494	0.011	0.042	1.47E-08	1.1
C7orf49	6.30E-13	-0.100000795	0.008	0.038	1.52E-08	1.1
POGK	6.31E-13	-0.115369743	0.027	0.067	1.52E-08	1.1
SRBD1	6.37E-13	-0.123042389	0.031	0.072	1.54E-08	1.1
ZNF107	6.56E-13	-0.135728047	0.042	0.087	1.58E-08	1.1
RPLP0	6.58E-13	0.276278251	0.891	0.907	1.59E-08	1.1
FBXO34	6.59E-13	-0.15473903	0.137	0.209	1.59E-08	1.1
C12orf65	6.77E-13	-0.108769914	0.03	0.071	1.63E-08	1.1
NXN	6.91E-13	-0.156375283	0.116	0.183	1.67E-08	1.1
SIPA1L2	6.99E-13	-0.124392273	0.041	0.087	1.68E-08	1.1
ORMDL2	7.03E-13	-0.137839003	0.114	0.181	1.69E-08	1.1
HMG20A	7.06E-13	-0.124762234	0.028	0.068	1.70E-08	1.1
PSMD11	7.07E-13	-0.169767588	0.238	0.325	1.71E-08	1.1
PTEN	7.10E-13	0.261694864	0.862	0.846	1.71E-08	1.1
LRRC4C	7.26E-13	-0.14958706	0.028	0.068	1.75E-08	1.1
GLIPR1	7.36E-13	0.354237228	0.389	0.331	1.77E-08	1.1
SUPT20H	7.70E-13	-0.115156398	0.047	0.095	1.86E-08	1.1
GALNT18	7.71E-13	-0.18370292	0.024	0.062	1.86E-08	1.1
NUDT19	7.71E-13	-0.107122862	0.016	0.05	1.86E-08	1.1
OSBPL10	7.91E-13	-0.190632806	0.09	0.15	1.91E-08	1.1
GPCPD1	7.98E-13	-0.193782288	0.084	0.141	1.92E-08	1.1
UFL1	8.09E-13	-0.138112921	0.034	0.077	1.95E-08	1.1

CMTM6	8.16E-13	-0.100513073	0.193	0.281	1.97E-08	1.1
RP11-347C	8.29E-13	-0.112863598	0.01	0.041	2.00E-08	1.1
GRIN2B	8.31E-13	-0.129126243	0.011	0.042	2.00E-08	1.1
MMP23B	8.45E-13	-0.109508917	0.026	0.065	2.04E-08	1.1
SLC26A4	8.51E-13	-0.175198368	0.004	0.029	2.05E-08	1.1
PCMT1	8.67E-13	-0.131262484	0.068	0.123	2.09E-08	1.1
STK24	8.70E-13	-0.166189971	0.098	0.16	2.10E-08	1.1
EPS15L1	8.73E-13	-0.112957372	0.038	0.082	2.10E-08	1.1
REEP3	8.98E-13	-0.119451385	0.184	0.268	2.16E-08	1.1
PHLPP2	9.08E-13	-0.118992416	0.021	0.058	2.19E-08	1.1
SUGP2	9.14E-13	-0.129955219	0.025	0.064	2.20E-08	1.1
TJP3	9.17E-13	-0.102078331	0.012	0.044	2.21E-08	1.1
ZNF736	9.20E-13	-0.125406068	0.05	0.098	2.22E-08	1.1
SNRPD1	9.60E-13	0.327866321	0.434	0.396	2.31E-08	1.1
EIF4B	9.73E-13	0.289532266	0.511	0.476	2.35E-08	1.1
CARD14	1.00E-12	-0.12732217	0.01	0.041	2.42E-08	1.1
DROSHA	1.01E-12	-0.11788592	0.017	0.051	2.44E-08	1.1
ZNF345	1.01E-12	-0.112160366	0.026	0.065	2.45E-08	1.1
COMMD1	1.03E-12	-0.149340708	0.049	0.096	2.49E-08	1.1
IER2	1.04E-12	-0.171991186	0.035	0.078	2.52E-08	1.1
SBNO1	1.05E-12	-0.11461659	0.063	0.117	2.53E-08	1.1
EXTL3	1.05E-12	-0.136269582	0.049	0.097	2.54E-08	1.1
NRG3	1.06E-12	-0.180405801	0.061	0.112	2.56E-08	1.1
RSF1	1.08E-12	-0.152865168	0.258	0.348	2.61E-08	1.1
TRIM22	1.11E-12	-0.14677119	0.068	0.122	2.67E-08	1.1
FASTKD1	1.15E-12	-0.139703903	0.025	0.063	2.77E-08	1.1
UBLCP1	1.15E-12	-0.106420967	0.037	0.081	2.77E-08	1.1
NAA50	1.15E-12	0.313468774	0.258	0.2	2.77E-08	1.1
C4BPB	1.18E-12	-0.111423969	0.02	0.056	2.84E-08	1.1
SLX4IP	1.20E-12	-0.117747968	0.016	0.049	2.90E-08	1.1
RP11-230B	1.21E-12	-0.102950991	0.01	0.04	2.92E-08	1.1
RELA	1.22E-12	-0.129796621	0.055	0.104	2.94E-08	1.1
CFL1	1.23E-12	-0.265798762	0.187	0.264	2.97E-08	1.1
CYP3A5	1.25E-12	-0.106619148	0.004	0.029	3.02E-08	1.1
GLYR1	1.27E-12	-0.1383706	0.077	0.133	3.05E-08	1.1
BRAF	1.33E-12	-0.110846726	0.265	0.363	3.20E-08	1.1
CNKSR3	1.33E-12	0.217629598	0.554	0.499	3.21E-08	1.1
NECAP2	1.34E-12	-0.108364586	0.018	0.052	3.23E-08	1.1
KCNQ1OT1	1.34E-12	-0.155258901	0.061	0.113	3.24E-08	1.1
RP11-294J2	1.34E-12	-0.100659886	0.022	0.059	3.24E-08	1.1
DLD	1.35E-12	-0.149252609	0.09	0.149	3.26E-08	1.1
TRNT1	1.41E-12	-0.100857821	0.013	0.045	3.39E-08	1.1
PSME2	1.42E-12	-0.285867449	0.291	0.375	3.42E-08	1.1
DIS3	1.44E-12	-0.132273919	0.048	0.094	3.48E-08	1.1
CALR	1.47E-12	0.301201939	0.23	0.173	3.53E-08	1.1
HM13	1.50E-12	-0.15325005	0.11	0.173	3.61E-08	1.1
DLEU1	1.52E-12	-0.169117962	0.112	0.176	3.66E-08	1.1

HAVCR2	1.53E-12	-0.108501004	0.008	0.035	3.68E-08	1.1
LINC00907	1.55E-12	-0.110234708	0.007	0.035	3.74E-08	1.1
C14orf1	1.56E-12	-0.116293115	0.028	0.067	3.77E-08	1.1
TMEM258	1.59E-12	0.221474077	0.649	0.624	3.85E-08	1.1
TRIM4	1.60E-12	-0.132797226	0.033	0.074	3.87E-08	1.1
HIST1H4H	1.63E-12	-0.112578288	0.038	0.082	3.93E-08	1.1
RP13-188A	1.65E-12	-0.122502827	0.012	0.042	3.99E-08	1.1
RP5-864K1	1.66E-12	-0.108772954	0.015	0.048	4.00E-08	1.1
ZBTB11	1.67E-12	0.182752106	0.089	0.05	4.03E-08	1.1
ARPC5	1.68E-12	-0.149965976	0.064	0.116	4.05E-08	1.1
CARF	1.74E-12	-0.105806654	0.011	0.041	4.20E-08	1.1
TBCEL	1.76E-12	-0.103230317	0.006	0.033	4.25E-08	1.1
LEPR	1.79E-12	-0.115471085	0.044	0.089	4.31E-08	1.1
BCKDHB	1.80E-12	-0.142527832	0.025	0.062	4.34E-08	1.1
MED15	1.81E-12	0.262670205	0.18	0.127	4.35E-08	1.1
PIK3R3	1.83E-12	-0.105804055	0.012	0.043	4.42E-08	1.1
CCNDBP1	1.84E-12	-0.107180078	0.027	0.066	4.44E-08	1.1
SMC5-AS1	1.87E-12	-0.106291638	0.012	0.042	4.51E-08	1.1
ICA1	1.93E-12	-0.187787177	0.222	0.305	4.65E-08	1.1
PREP	1.96E-12	0.232241844	0.144	0.095	4.72E-08	1.1
FOS	1.97E-12	-0.244517151	0.187	0.265	4.76E-08	1.1
SPICE1	1.97E-12	-0.120957617	0.025	0.062	4.76E-08	1.1
NR2C1	1.98E-12	-0.117229505	0.03	0.071	4.78E-08	1.1
ASPH	1.98E-12	-0.140084835	0.075	0.13	4.79E-08	1.1
CNOT10	2.03E-12	-0.116110722	0.03	0.069	4.90E-08	1.1
EP400	2.04E-12	-0.123677431	0.048	0.095	4.91E-08	1.1
ZNF717	2.06E-12	-0.100236948	0.012	0.042	4.97E-08	1.1
MYOF	2.06E-12	0.315794943	0.542	0.511	4.98E-08	1.1
PRDX5	2.12E-12	-0.176129952	0.035	0.076	5.12E-08	1.1
PIK3C2B	2.13E-12	-0.13463683	0.014	0.045	5.14E-08	1.1
CFAP44	2.15E-12	-0.101429352	0.01	0.039	5.18E-08	1.1
TUBGCP3	2.15E-12	-0.100871179	0.008	0.035	5.18E-08	1.1
EPHA4	2.16E-12	0.231547377	0.076	0.04	5.20E-08	1.1
MOB1B	2.19E-12	-0.108651537	0.033	0.075	5.28E-08	1.1
RHEB	2.21E-12	0.314588562	0.291	0.236	5.33E-08	1.1
ATRN	2.26E-12	-0.135598683	0.041	0.085	5.46E-08	1.1
LRCH1	2.27E-12	-0.180432954	0.283	0.378	5.46E-08	1.1
TRMT112	2.27E-12	-0.137712784	0.04	0.084	5.48E-08	1.1
ARMC9	2.27E-12	-0.220256692	0.064	0.116	5.48E-08	1.1
GCA	2.29E-12	-0.101052616	0.035	0.078	5.51E-08	1.1
CTC-254B4	2.29E-12	-0.102394204	0.009	0.037	5.52E-08	1.1
LSM8	2.38E-12	-0.121727306	0.203	0.289	5.74E-08	1.1
WWP2	2.40E-12	-0.124063072	0.029	0.068	5.79E-08	1.1
MLF2	2.42E-12	-0.132711231	0.031	0.071	5.83E-08	1.1
SNRPE	2.43E-12	0.245768665	0.622	0.61	5.85E-08	1.1
BLVRB	2.51E-12	-0.132116644	0.038	0.081	6.05E-08	1.1
SFXN1	2.61E-12	-0.131023775	0.033	0.074	6.30E-08	1.1

CNOT7	2.63E-12	-0.12856136	0.065	0.116	6.35E-08	1.1
MIRLET7BH	2.67E-12	-0.149699577	0.013	0.043	6.43E-08	1.1
NUBPL	2.70E-12	-0.165852114	0.033	0.073	6.51E-08	1.1
XIAP	2.71E-12	-0.121092405	0.065	0.118	6.54E-08	1.1
PYROXD1	2.71E-12	-0.112255626	0.021	0.057	6.54E-08	1.1
ATF2	2.79E-12	0.296527598	0.17	0.12	6.72E-08	1.1
ROR1	2.86E-12	-0.184432633	0.015	0.046	6.88E-08	1.1
POLG2	2.92E-12	0.328252726	0.107	0.065	7.04E-08	1.1
POLR3B	2.97E-12	-0.108924776	0.007	0.033	7.17E-08	1.1
CEP63	2.99E-12	-0.135122882	0.049	0.095	7.22E-08	1.1
CNOT4	3.03E-12	-0.127729885	0.276	0.376	7.31E-08	1.1
LGALS3	3.04E-12	-0.189649299	0.132	0.199	7.34E-08	1.1
GAPDH	3.15E-12	-0.302405298	0.53	0.631	7.60E-08	1.1
MRPL14	3.16E-12	-0.122748338	0.078	0.133	7.61E-08	1.1
FAM118B	3.19E-12	-0.113787584	0.016	0.049	7.68E-08	1.1
LINC01389	3.23E-12	-0.119524375	0.014	0.046	7.78E-08	1.1
RPLP2	3.31E-12	-0.344832634	0.183	0.252	7.99E-08	1.1
RNF34	3.35E-12	-0.129266003	0.032	0.071	8.07E-08	1.1
SIMC1	3.39E-12	-0.107303019	0.017	0.05	8.18E-08	1.1
MT-ND4L	3.42E-12	0.259560615	0.554	0.527	8.24E-08	1.1
TRAFD1	3.47E-12	-0.12627252	0.058	0.107	8.36E-08	1.1
MAP2K1	3.58E-12	-0.135902741	0.156	0.231	8.62E-08	1.1
ADGRA3	3.58E-12	-0.142574261	0.09	0.149	8.63E-08	1.1
KLHL8	3.61E-12	-0.102201476	0.011	0.04	8.71E-08	1.1
HIBADH	3.62E-12	-0.132694772	0.14	0.211	8.72E-08	1.1
ITPR3	3.73E-12	-0.141939306	0.023	0.058	8.99E-08	1.1
RNASE4	3.74E-12	-0.123699028	0.04	0.083	9.03E-08	1.1
SZT2	3.77E-12	-0.100695176	0.008	0.034	9.08E-08	1.1
HIF1AN	3.82E-12	-0.108840751	0.013	0.044	9.22E-08	1.1
LINC00693	3.88E-12	-0.110709414	0.007	0.034	9.36E-08	1.1
ANGEL2	4.03E-12	-0.101406056	0.012	0.042	9.73E-08	1.1
SPCS3	4.08E-12	-0.143065014	0.031	0.07	9.83E-08	1.1
BIRC6	4.13E-12	-0.125181803	0.475	0.588	9.97E-08	1.1
TMEM117	4.20E-12	-0.122139515	0.016	0.049	1.01E-07	1.1
FAM19A2	4.23E-12	0.181591212	0.671	0.621	1.02E-07	1.1
KIF1C	4.25E-12	-0.114564713	0.017	0.05	1.02E-07	1.1
MED6	4.32E-12	-0.124802813	0.124	0.191	1.04E-07	1.1
CCT3	4.47E-12	-0.154400591	0.365	0.468	1.08E-07	1.1
GAN	4.48E-12	-0.11356437	0.096	0.157	1.08E-07	1.1
GS1-124K5	4.59E-12	-0.111481017	0.007	0.034	1.11E-07	1.1
ZER1	4.61E-12	-0.14193091	0.068	0.119	1.11E-07	1.1
RCL1	4.84E-12	-0.102077692	0.013	0.044	1.17E-07	1.1
VPRBP	4.91E-12	-0.126239681	0.025	0.061	1.18E-07	1.1
KLK10	4.93E-12	-0.142374459	0.03	0.07	1.19E-07	1.1
SFMBT1	4.97E-12	-0.119579792	0.02	0.055	1.20E-07	1.1
AMD1	5.33E-12	0.372590962	0.281	0.228	1.29E-07	1.1
RSL24D1	5.33E-12	0.186699162	0.754	0.742	1.29E-07	1.1

TRIM27	5.39E-12	-0.112013083	0.057	0.106	1.30E-07	1.1
ATAD2	5.43E-12	-0.129396092	0.038	0.079	1.31E-07	1.1
STAM2	5.44E-12	-0.1220179	0.032	0.071	1.31E-07	1.1
FBXO21	5.47E-12	-0.129256026	0.059	0.109	1.32E-07	1.1
HOOK3	5.47E-12	-0.116218323	0.086	0.144	1.32E-07	1.1
C9orf91	5.55E-12	-0.104870017	0.012	0.042	1.34E-07	1.1
LINC01572	5.57E-12	-0.105180132	0.017	0.05	1.34E-07	1.1
DEPDC5	5.60E-12	-0.12177832	0.019	0.052	1.35E-07	1.1
RC3H2	5.63E-12	-0.131509037	0.064	0.115	1.36E-07	1.1
ATP6AP1L	5.64E-12	-0.101330858	0.01	0.038	1.36E-07	1.1
PRKRA	5.65E-12	-0.13560212	0.047	0.092	1.36E-07	1.1
MAP1B	5.99E-12	-0.242569137	0.346	0.431	1.44E-07	1.1
ATXN7	6.15E-12	-0.14681657	0.103	0.163	1.48E-07	1.1
STPG2	6.17E-12	-0.14604287	0.029	0.067	1.49E-07	1.1
C5orf63	6.31E-12	-0.100673533	0.013	0.044	1.52E-07	1.1
CHM	6.33E-12	-0.120101243	0.044	0.088	1.53E-07	1.1
SLC26A8	6.42E-12	-0.117222707	0.011	0.04	1.55E-07	1.1
SIRPA	6.55E-12	-0.105867691	0.034	0.075	1.58E-07	1.1
NFYB	6.75E-12	-0.110503383	0.037	0.078	1.63E-07	1.1
MCCC1	6.75E-12	-0.109019093	0.068	0.12	1.63E-07	1.1
CORO2A	6.76E-12	-0.114207544	0.016	0.048	1.63E-07	1.1
ACER3	6.86E-12	-0.127013255	0.029	0.067	1.65E-07	1.1
NEK10	6.92E-12	-0.169811345	0.08	0.135	1.67E-07	1.1
ASXL1	7.03E-12	0.306246492	0.304	0.251	1.69E-07	1.1
KIAA1143	7.12E-12	-0.124146601	0.077	0.131	1.72E-07	1.1
SLC6A14	7.21E-12	-0.185299479	0.16	0.231	1.74E-07	1.1
ABRACL	7.56E-12	-0.121783745	0.315	0.411	1.82E-07	1.1
ARL4C	7.64E-12	0.317378148	0.257	0.202	1.84E-07	1.1
SARNP	7.77E-12	0.340952094	0.267	0.212	1.87E-07	1.1
TPGS2	7.80E-12	-0.10590416	0.027	0.064	1.88E-07	1.1
PARG	7.91E-12	-0.108287176	0.057	0.106	1.91E-07	1.1
LATS2	7.94E-12	0.230226702	0.157	0.107	1.92E-07	1.1
TMEM136	8.01E-12	0.150134948	0.06	0.03	1.93E-07	1.1
CYTH3	8.29E-12	0.276769125	0.116	0.074	2.00E-07	1.1
ANXA5	8.42E-12	-0.169601597	0.195	0.271	2.03E-07	1.1
CEP112	8.53E-12	-0.123708645	0.018	0.051	2.06E-07	1.1
L3MBTL1	8.69E-12	-0.100357599	0.015	0.046	2.10E-07	1.1
SPEN	8.70E-12	0.316260674	0.313	0.257	2.10E-07	1.1
AAGAB	8.72E-12	-0.126104746	0.095	0.153	2.10E-07	1.1
YIPF1	8.87E-12	-0.100967343	0.023	0.059	2.14E-07	1.1
SMYD4	8.87E-12	-0.114303038	0.01	0.039	2.14E-07	1.1
FAM192A	9.00E-12	-0.118501746	0.03	0.068	2.17E-07	1.1
GIT2	9.01E-12	-0.117944609	0.04	0.083	2.17E-07	1.1
BFAR	9.05E-12	0.26900021	0.236	0.182	2.18E-07	1.1
RP11-701H	9.23E-12	-0.117461577	0.024	0.059	2.23E-07	1.1
C6orf132	9.67E-12	-0.166984927	0.119	0.182	2.33E-07	1.1
DMXL1	9.68E-12	-0.151299437	0.081	0.136	2.33E-07	1.1

SHANK2	9.71E-12	-0.158853968	0.145	0.215	2.34E-07	1.1
ABCC3	9.74E-12	-0.118386325	0.006	0.032	2.35E-07	1.1
PSMB3	9.79E-12	-0.102926629	0.04	0.084	2.36E-07	1.1
BRK1	9.85E-12	-0.179156966	0.622	0.716	2.38E-07	1.1
CTNNBL1	9.91E-12	-0.11988625	0.05	0.096	2.39E-07	1.1
CCZ1B	9.99E-12	-0.104533968	0.02	0.054	2.41E-07	1.1
CREBBP	1.00E-11	-0.157399233	0.183	0.259	2.42E-07	1.1
TBC1D19	1.01E-11	-0.100845404	0.007	0.032	2.43E-07	1.1
CCNG1	1.04E-11	-0.12415338	0.083	0.14	2.50E-07	1.1
GALNT11	1.04E-11	-0.147249416	0.051	0.096	2.51E-07	1.1
PHTF1	1.04E-11	-0.113456175	0.017	0.049	2.51E-07	1.1
MAPRE2	1.04E-11	-0.153281236	0.095	0.154	2.52E-07	1.1
LRP5	1.05E-11	-0.112975097	0.017	0.049	2.54E-07	1.1
PAX8	1.07E-11	-0.117294379	0.01	0.038	2.57E-07	1.1
NUP58	1.08E-11	0.222421571	0.141	0.093	2.61E-07	1.1
GAS7	1.09E-11	-0.132623777	0.027	0.064	2.63E-07	1.1
TMEM181	1.11E-11	-0.131238108	0.185	0.263	2.68E-07	1.1
C16orf45	1.14E-11	-0.130814909	0.031	0.07	2.75E-07	1.1
NR6A1	1.15E-11	-0.123638161	0.13	0.198	2.76E-07	1.1
PGBD5	1.15E-11	-0.133943622	0.048	0.092	2.78E-07	1.1
SBNO2	1.15E-11	-0.114210755	0.013	0.043	2.78E-07	1.1
CCPG1	1.19E-11	-0.116063477	0.036	0.076	2.87E-07	1.1
NCK1	1.22E-11	-0.145172418	0.115	0.177	2.95E-07	1.1
LINC00869	1.27E-11	-0.107545049	0.026	0.063	3.06E-07	1.1
P4HA2	1.38E-11	-0.132948742	0.043	0.086	3.32E-07	1.1
CUL1	1.39E-11	-0.136064621	0.071	0.122	3.34E-07	1.1
PTRH2	1.42E-11	-0.15665242	0.12	0.183	3.42E-07	1.1
NGFRAP1	1.43E-11	0.145513668	0.068	0.035	3.45E-07	1.1
ARPC2	1.48E-11	-0.132134161	0.128	0.193	3.58E-07	1.1
TMEM50A	1.49E-11	-0.142504835	0.108	0.168	3.59E-07	1.1
ADIPOR2	1.49E-11	-0.111208012	0.026	0.063	3.60E-07	1.1
FAM35A	1.53E-11	-0.174674194	0.076	0.129	3.68E-07	1.1
CDK8	1.60E-11	-0.108209124	0.118	0.182	3.86E-07	1.1
SRPK2	1.66E-11	0.35910335	0.406	0.368	4.00E-07	1.1
RNF111	1.67E-11	-0.109732806	0.18	0.261	4.02E-07	1.1
BID	1.67E-11	-0.146500669	0.103	0.163	4.02E-07	1.1
GGA1	1.71E-11	-0.112571063	0.02	0.053	4.11E-07	1.1
CUEDC1	1.71E-11	-0.114733782	0.028	0.064	4.12E-07	1.1
PPP2R5C	1.71E-11	-0.109938533	0.156	0.23	4.13E-07	1.1
GLCE	1.75E-11	-0.125258824	0.024	0.059	4.21E-07	1.1
POLB	1.75E-11	-0.116236535	0.039	0.08	4.22E-07	1.1
NOP58	1.78E-11	0.283497744	0.258	0.203	4.30E-07	1.1
C12orf57	1.85E-11	-0.142088414	0.021	0.054	4.47E-07	1.1
TAF11	1.86E-11	-0.113426191	0.025	0.061	4.48E-07	1.1
MTR	1.88E-11	-0.123172944	0.04	0.081	4.54E-07	1.1
ANAPC4	1.88E-11	-0.101708027	0.007	0.032	4.54E-07	1.1
MFSD1	1.89E-11	-0.103226665	0.028	0.065	4.56E-07	1.1

KCTD9	1.90E-11	0.40426033	0.339	0.287	4.57E-07	1.1
FAM120C	1.92E-11	-0.129263988	0.028	0.064	4.62E-07	1.1
GRAMD1C	1.96E-11	-0.106549473	0.021	0.055	4.72E-07	1.1
PSMD7	1.96E-11	-0.133864949	0.106	0.165	4.73E-07	1.1
TXNDC12	2.08E-11	-0.117278715	0.023	0.058	5.01E-07	1.1
NDUFS1	2.08E-11	-0.104350602	0.088	0.145	5.02E-07	1.1
COX19	2.08E-11	-0.107726729	0.025	0.06	5.02E-07	1.1
NSMCE2	2.16E-11	-0.138911705	0.352	0.453	5.20E-07	1.1
PRKAA2	2.16E-11	-0.108146332	0.02	0.052	5.20E-07	1.1
ARHGAP31	2.19E-11	-0.10062471	0.006	0.03	5.27E-07	1.1
DLGAP4	2.21E-11	-0.142192576	0.073	0.124	5.33E-07	1.1
LINC00937	2.22E-11	-0.10033749	0.01	0.037	5.36E-07	1.1
SAMD12-A:	2.28E-11	-0.106645494	0.011	0.039	5.49E-07	1.1
EPG5	2.28E-11	-0.140715925	0.056	0.103	5.50E-07	1.1
MAPK8	2.35E-11	-0.117190582	0.162	0.235	5.66E-07	1.1
EPM2AIP1	2.44E-11	-0.119181599	0.03	0.068	5.89E-07	1.1
RGS12	2.46E-11	-0.121197692	0.011	0.039	5.92E-07	1.1
CAPNS1	2.46E-11	-0.164876253	0.035	0.074	5.94E-07	1.1
KIAA0430	2.47E-11	-0.123290478	0.042	0.084	5.96E-07	1.1
C9orf3	2.48E-11	-0.200806914	0.122	0.184	5.98E-07	1.1
TMEM132/	2.51E-11	-0.102208171	0.004	0.026	6.06E-07	1.1
C3orf35	2.53E-11	-0.116102747	0.02	0.052	6.10E-07	1.1
COG6	2.53E-11	-0.106318786	0.011	0.039	6.11E-07	1.1
MRPS31	2.55E-11	-0.108850873	0.078	0.131	6.16E-07	1.1
FGF13	2.59E-11	-0.164309859	0.166	0.236	6.23E-07	1.1
RP11-317G	2.61E-11	0.181313375	0.076	0.042	6.29E-07	1.1
KCNH8	2.62E-11	-0.126264908	0.018	0.05	6.31E-07	1.1
CTA-292E1	2.63E-11	-0.160806103	0.074	0.125	6.35E-07	1.1
SHFM1	2.64E-11	-0.15933874	0.552	0.656	6.36E-07	1.1
ZDHHC17	2.65E-11	-0.144955272	0.06	0.107	6.39E-07	1.1
MUC15	2.66E-11	-0.104743777	0.023	0.057	6.42E-07	1.1
TFAP2A	2.67E-11	-0.126718677	0.038	0.078	6.43E-07	1.1
WDR37	2.69E-11	-0.115532316	0.053	0.099	6.48E-07	1.1
AMBRA1	2.71E-11	0.178562957	0.737	0.729	6.53E-07	1.1
PIKFYVE	2.71E-11	-0.10937375	0.065	0.114	6.53E-07	1.1
CTC-444N2	2.72E-11	-0.117278686	0.05	0.095	6.56E-07	1.1
ATP5EP2	2.74E-11	-0.142400488	0.154	0.224	6.62E-07	1.1
CSPP1	2.75E-11	-0.136828466	0.086	0.141	6.63E-07	1.1
PIGF	2.76E-11	-0.11513379	0.074	0.126	6.66E-07	1.1
SMAGP	2.82E-11	-0.113472678	0.03	0.067	6.80E-07	1.1
CARNMT1	2.85E-11	-0.113472309	0.027	0.062	6.87E-07	1.1
DYNC2LI1	2.90E-11	0.21360039	0.125	0.082	6.99E-07	1.1
GPRIN3	2.99E-11	-0.103370214	0.011	0.039	7.20E-07	1.1
IL13RA1	3.03E-11	-0.106073638	0.023	0.056	7.31E-07	1.1
SLC38A2	3.07E-11	-0.148706757	0.13	0.193	7.40E-07	1.1
GBP2	3.11E-11	0.296493785	0.75	0.756	7.49E-07	1.1
CCDC109B	3.15E-11	-0.125662023	0.033	0.071	7.59E-07	1.1

MAGT1	3.16E-11	0.268480718	0.218	0.165	7.62E-07	1.1
RP11-420A	3.22E-11	-0.109756231	0.013	0.041	7.77E-07	1.1
VTA1	3.34E-11	-0.106745823	0.056	0.103	8.05E-07	1.1
PTK2	3.38E-11	-0.108517009	0.474	0.597	8.15E-07	1.1
MTOR	3.41E-11	-0.106917961	0.026	0.061	8.22E-07	1.1
TCP11L2	3.41E-11	-0.100839681	0.033	0.071	8.23E-07	1.1
C6orf89	3.45E-11	-0.111861342	0.048	0.092	8.32E-07	1.1
AGPAT5	3.47E-11	-0.114495653	0.021	0.054	8.36E-07	1.1
SSR1	3.47E-11	-0.122156911	0.131	0.196	8.38E-07	1.1
PTPN6	3.48E-11	-0.104662074	0.022	0.055	8.40E-07	1.1
SOCS7	3.49E-11	-0.119596799	0.018	0.049	8.41E-07	1.1
MIDN	3.54E-11	-0.127993082	0.023	0.057	8.55E-07	1.1
RP11-66B2	3.57E-11	-0.114386069	0.019	0.05	8.61E-07	1.1
HIST1H2BG	3.62E-11	-0.108635019	0.07	0.121	8.73E-07	1.1
WTAP	3.63E-11	0.562911347	0.66	0.665	8.74E-07	1.1
KANSL2	3.70E-11	-0.110920523	0.023	0.057	8.93E-07	1.1
NT5DC1	3.77E-11	-0.13711388	0.041	0.082	9.09E-07	1.1
DAP	3.90E-11	-0.118503418	0.046	0.089	9.39E-07	1.1
HKR1	3.96E-11	-0.102772988	0.032	0.069	9.55E-07	1.1
SAMD8	4.07E-11	-0.108900761	0.041	0.082	9.81E-07	1.1
MAP4K5	4.14E-11	0.411316898	0.391	0.355	9.97E-07	1.1
TLE2	4.17E-11	-0.112966559	0.028	0.064	1.01E-06	1.1
KMT2A	4.20E-11	0.266158523	0.433	0.39	1.01E-06	1.1
EXOSC1	4.21E-11	-0.110199267	0.026	0.061	1.01E-06	1.1
TM9SF4	4.52E-11	-0.134153507	0.07	0.12	1.09E-06	1.1
ZNF121	4.55E-11	-0.135402631	0.055	0.1	1.10E-06	1.1
VPS13A	4.58E-11	-0.109540326	0.076	0.129	1.10E-06	1.1
MED28	4.60E-11	-0.124908861	0.07	0.12	1.11E-06	1.1
RPLP1	4.72E-11	-0.389591081	0.218	0.286	1.14E-06	1.1
TTI2	4.77E-11	-0.107521714	0.029	0.065	1.15E-06	1.1
DGCR2	4.79E-11	-0.100404722	0.018	0.05	1.16E-06	1.1
PIR	4.84E-11	-0.100075277	0.01	0.037	1.17E-06	1.1
DCP2	4.85E-11	-0.13957447	0.061	0.108	1.17E-06	1.1
SREK1IP1	4.90E-11	-0.111514755	0.108	0.167	1.18E-06	1.1
EIF5B	5.02E-11	-0.132311206	0.17	0.241	1.21E-06	1.1
ZNF217	5.13E-11	-0.129116595	0.101	0.159	1.24E-06	1.1
PDCD6IP	5.23E-11	-0.129011968	0.1	0.157	1.26E-06	1.1
PTK7	5.26E-11	-0.125933808	0.058	0.104	1.27E-06	1.1
PISD	5.32E-11	0.292296926	0.214	0.163	1.28E-06	1.1
ZNF136	5.35E-11	-0.103801613	0.026	0.061	1.29E-06	1.1
TAF4B	5.41E-11	-0.106718537	0.038	0.078	1.31E-06	1.1
EXOSC6	5.43E-11	-0.101192659	0.01	0.037	1.31E-06	1.1
NFE2L1	5.54E-11	-0.122298951	0.033	0.07	1.33E-06	1.1
PDZD8	5.63E-11	-0.141134406	0.064	0.111	1.36E-06	1.1
LRIG3	5.76E-11	-0.117202205	0.03	0.066	1.39E-06	1.1
GTDC1	5.81E-11	-0.11125103	0.033	0.07	1.40E-06	1.1
PTGFRN	5.82E-11	-0.138044099	0.082	0.135	1.40E-06	1.1

HLA-B	5.87E-11	-0.202420906	0.566	0.663	1.41E-06	1.1
PMS1	5.87E-11	-0.117431601	0.024	0.057	1.42E-06	1.1
IFT22	5.94E-11	0.117880974	0.06	0.03	1.43E-06	1.1
NIT2	6.13E-11	0.234818049	0.135	0.092	1.48E-06	1.1
SPATA6L	6.22E-11	-0.103944875	0.015	0.045	1.50E-06	1.1
SETDB1	6.36E-11	-0.121979595	0.054	0.099	1.53E-06	1.1
CCT7	6.43E-11	-0.127786076	0.015	0.044	1.55E-06	1.1
PRPF4B	6.43E-11	-0.122823262	0.144	0.211	1.55E-06	1.1
STAG2	6.62E-11	-0.145315291	0.305	0.396	1.60E-06	1.1
CCDC92	6.69E-11	-0.10627008	0.015	0.045	1.61E-06	1.1
PLAA	6.71E-11	-0.132581916	0.039	0.078	1.62E-06	1.1
AC072062.	6.75E-11	0.250262539	0.548	0.513	1.63E-06	1.1
MFI2	6.79E-11	-0.108268777	0.009	0.034	1.64E-06	1.1
EGF	6.80E-11	-0.129141106	0.048	0.09	1.64E-06	1.1
PPP2R4	6.86E-11	-0.100275721	0.008	0.033	1.65E-06	1.1
NPLOC4	6.88E-11	-0.134390158	0.087	0.141	1.66E-06	1.1
RARS2	6.97E-11	-0.11669301	0.068	0.117	1.68E-06	1.1
PI4KA	6.98E-11	-0.141323409	0.059	0.105	1.68E-06	1.1
SPATS2	7.04E-11	-0.13455389	0.073	0.123	1.70E-06	1.1
BUB3	7.05E-11	-0.122971288	0.035	0.072	1.70E-06	1.1
ZNF280D	7.10E-11	-0.104165349	0.068	0.116	1.71E-06	1.1
TMEM106A	7.32E-11	-0.113477261	0.02	0.052	1.76E-06	1.1
HAX1	7.33E-11	-0.11551025	0.267	0.356	1.77E-06	1.1
GRAMD1A	7.47E-11	-0.131917709	0.017	0.047	1.80E-06	1.1
IPO11	7.57E-11	-0.114950927	0.017	0.047	1.83E-06	1.1
BRCC3	7.59E-11	-0.127575262	0.041	0.081	1.83E-06	1.1
KIF16B	7.75E-11	-0.1257957	0.053	0.097	1.87E-06	1.1
BCAS3	8.00E-11	0.188365557	0.824	0.847	1.93E-06	1.1
CDC123	8.09E-11	-0.106969869	0.021	0.053	1.95E-06	1.1
SOAT1	8.46E-11	-0.10808254	0.032	0.069	2.04E-06	1.1
AREG	8.66E-11	0.290528987	0.13	0.087	2.09E-06	1.1
FGFR2	8.66E-11	-0.129916392	0.018	0.048	2.09E-06	1.1
ELF5	8.67E-11	0.327586396	0.336	0.285	2.09E-06	1.1
GALK2	9.13E-11	-0.102663241	0.046	0.088	2.20E-06	1.1
CDK5RAP1	9.34E-11	-0.10622162	0.023	0.055	2.25E-06	1.1
GTF2A2	9.52E-11	-0.124204142	0.057	0.101	2.30E-06	1.1
KLHDC2	9.66E-11	-0.106372199	0.029	0.065	2.33E-06	1.1
CLNS1A	1.01E-10	-0.120665771	0.057	0.102	2.43E-06	1.1
BLZF1	1.01E-10	-0.121319781	0.058	0.104	2.43E-06	1.1
H2AFY	1.05E-10	-0.137888116	0.097	0.152	2.54E-06	1.1
KDM6A	1.06E-10	0.27948393	0.356	0.305	2.55E-06	1.1
HEBP2	1.06E-10	-0.126738531	0.129	0.192	2.56E-06	1.1
ZDHHC14	1.07E-10	-0.117829677	0.067	0.116	2.57E-06	1.1
FPGS	1.07E-10	-0.110464493	0.018	0.048	2.59E-06	1.1
CD81	1.09E-10	-0.131758643	0.014	0.042	2.63E-06	1.1
SFPQ	1.14E-10	-0.146885226	0.168	0.238	2.74E-06	1.1
SCP2	1.15E-10	0.290778502	0.267	0.217	2.76E-06	1.1

SORT1	1.20E-10	-0.109427169	0.046	0.087	2.89E-06	1.1
RP11-16D2	1.20E-10	-0.101326423	0.005	0.027	2.89E-06	1.1
CDC14A	1.20E-10	-0.131582541	0.038	0.077	2.90E-06	1.1
SRP54	1.21E-10	-0.104262167	0.217	0.298	2.91E-06	1.1
APPL1	1.23E-10	-0.103189714	0.048	0.091	2.97E-06	1.1
TRIM5	1.24E-10	-0.168602426	0.106	0.163	2.99E-06	1.1
COX20	1.26E-10	-0.111574711	0.075	0.125	3.04E-06	1.1
RNF38	1.26E-10	-0.141379222	0.172	0.243	3.04E-06	1.1
CROCC	1.29E-10	0.203783956	0.1	0.062	3.11E-06	1.1
ARID4A	1.31E-10	0.240464593	0.21	0.16	3.17E-06	1.1
TRAP1	1.35E-10	-0.125739191	0.023	0.055	3.26E-06	1.1
LASP1	1.42E-10	-0.11887398	0.042	0.082	3.42E-06	1.1
NARS	1.44E-10	-0.141956067	0.23	0.311	3.48E-06	1.1
KIFAP3	1.46E-10	-0.104194974	0.029	0.064	3.53E-06	1.1
GK	1.49E-10	-0.108438033	0.026	0.06	3.59E-06	1.1
SQSTM1	1.50E-10	0.297387305	0.321	0.267	3.61E-06	1.1
BDNF-AS	1.51E-10	0.299719873	0.667	0.68	3.65E-06	1.1
NDFIP1	1.58E-10	-0.110911352	0.113	0.172	3.81E-06	1.1
OLFM4	1.61E-10	-0.27297449	0.018	0.048	3.87E-06	1.1
WBP11	1.62E-10	0.248276678	0.162	0.116	3.91E-06	1.1
RPS10-NUI	1.62E-10	0.159449146	0.046	0.021	3.92E-06	1.1
NFATC1	1.63E-10	-0.115657499	0.015	0.043	3.92E-06	1.1
SGCZ	1.64E-10	-0.248738378	0.054	0.097	3.96E-06	1.1
RHOC	1.65E-10	-0.1344038	0.029	0.064	3.98E-06	1.1
PAFAH1B1	1.66E-10	0.34483098	0.396	0.359	3.99E-06	1.1
SNRPF	1.66E-10	0.287551528	0.285	0.236	3.99E-06	1.1
SPP1	1.66E-10	-0.410318498	0.005	0.027	4.00E-06	1.1
THOC1	1.67E-10	-0.152025482	0.068	0.114	4.02E-06	1.1
IL1R1	1.68E-10	-0.112197826	0.017	0.046	4.06E-06	1.1
PSMA7	1.70E-10	-0.122133214	0.138	0.201	4.11E-06	1.1
AGTPBP1	1.71E-10	-0.139731469	0.051	0.094	4.12E-06	1.1
PSMB8	1.71E-10	-0.121606703	0.077	0.127	4.13E-06	1.1
XPO6	1.72E-10	-0.121243397	0.042	0.081	4.16E-06	1.1
DNTTIP1	1.81E-10	-0.116763629	0.041	0.08	4.36E-06	1.1
MAPK1IP1I	1.81E-10	-0.121853281	0.153	0.219	4.36E-06	1.1
DNAJC10	1.82E-10	-0.111797213	0.029	0.063	4.39E-06	1.1
GATAD2A	1.83E-10	-0.13073197	0.068	0.116	4.41E-06	1.1
RNGTT	1.89E-10	-0.10148774	0.035	0.072	4.55E-06	1.1
ITGAV	1.93E-10	-0.109445113	0.114	0.174	4.66E-06	1.1
AGK	2.00E-10	-0.102017588	0.018	0.048	4.83E-06	1.1
ABHD2	2.05E-10	0.21029637	0.132	0.089	4.94E-06	1.1
SUN2	2.05E-10	-0.112232076	0.015	0.043	4.95E-06	1.1
S100P	2.06E-10	-0.141241191	0.013	0.04	4.97E-06	1.1
RALGDS	2.07E-10	-0.125892565	0.044	0.084	4.99E-06	1.1
BCAR3	2.10E-10	-0.103683354	0.059	0.105	5.06E-06	1.1
RGS6	2.14E-10	-0.199253616	0.105	0.16	5.17E-06	1.1
SNRPB2	2.20E-10	-0.123520544	0.378	0.476	5.31E-06	1.1

CREBL2	2.27E-10	-0.110721018	0.034	0.071	5.48E-06	1.1
KAT8	2.36E-10	-0.10467676	0.035	0.072	5.69E-06	1.1
FAM129B	2.37E-10	-0.15585606	0.074	0.122	5.72E-06	1.1
MBNL1	2.40E-10	-0.115973515	0.354	0.451	5.79E-06	1.1
PALMD	2.42E-10	0.109925371	0.05	0.024	5.83E-06	1.1
ITM2B	2.47E-10	-0.123623214	0.198	0.274	5.94E-06	1.1
PEX14	2.51E-10	-0.125293821	0.068	0.115	6.05E-06	1.1
DIAPH1	2.58E-10	-0.108714785	0.163	0.231	6.21E-06	1.1
DPP10	2.64E-10	-0.143956296	0.026	0.059	6.36E-06	1.1
RAD18	2.69E-10	-0.103340635	0.027	0.061	6.48E-06	1.1
WHSC1L1	2.75E-10	-0.148130791	0.145	0.207	6.64E-06	1.1
PLPP1	2.76E-10	-0.106924158	0.045	0.086	6.66E-06	1.1
PRRC2B	2.77E-10	-0.174829201	0.169	0.236	6.67E-06	1.1
SUFU	2.77E-10	-0.126625061	0.025	0.057	6.68E-06	1.1
MFF	2.81E-10	-0.107520216	0.055	0.098	6.79E-06	1.1
RNF7	2.85E-10	-0.116206485	0.035	0.071	6.86E-06	1.1
NEB	2.86E-10	-0.100267383	0.011	0.037	6.90E-06	1.1
EGLN1	2.87E-10	0.182229954	0.126	0.084	6.91E-06	1.1
APBA2	2.95E-10	-0.106034221	0.032	0.068	7.10E-06	1.1
ADGRV1	2.97E-10	-0.137438128	0.094	0.147	7.17E-06	1.1
FEM1C	2.97E-10	0.185825269	0.073	0.041	7.17E-06	1.1
AC073283.	3.01E-10	-0.145969887	0.078	0.127	7.25E-06	1.1
TRAPPC8	3.07E-10	-0.101153989	0.06	0.105	7.41E-06	1.1
RAP1A	3.09E-10	-0.122281237	0.163	0.229	7.44E-06	1.1
RHOU	3.21E-10	-0.101312736	0.018	0.047	7.73E-06	1.1
FAM53C	3.27E-10	0.288580132	0.249	0.198	7.88E-06	1.1
GALNT7	3.29E-10	-0.109967778	0.063	0.109	7.93E-06	1.1
SRSF7	3.36E-10	-0.140427158	0.228	0.307	8.11E-06	1.1
CYLD	3.42E-10	0.334692594	0.28	0.228	8.24E-06	1.1
PLA2G4C	3.45E-10	-0.113760343	0.032	0.067	8.32E-06	1.1
H3F3B	3.58E-10	-0.213974232	0.331	0.421	8.63E-06	1.1
RP11-428O	3.59E-10	-0.100493257	0.009	0.033	8.66E-06	1.1
DMGDH	3.61E-10	-0.109583456	0.018	0.048	8.71E-06	1.1
ARMCX4	3.62E-10	-0.100069027	0.012	0.037	8.73E-06	1.1
SOX4	3.63E-10	0.321163464	0.751	0.735	8.74E-06	1.1
PIAS1	3.65E-10	0.324493338	0.445	0.413	8.79E-06	1.1
CADM1	3.70E-10	-0.150823284	0.055	0.098	8.92E-06	1.1
POLA1	3.75E-10	-0.100122514	0.024	0.056	9.04E-06	1.1
MRAS	3.78E-10	-0.113494201	0.046	0.086	9.11E-06	1.1
ERGIC1	3.78E-10	-0.110115597	0.063	0.108	9.12E-06	1.1
KANK1	3.79E-10	-0.149341374	0.169	0.236	9.14E-06	1.1
XYLT1	3.89E-10	-0.125981682	0.08	0.129	9.37E-06	1.1
GOLPH3L	3.93E-10	-0.115491497	0.161	0.228	9.47E-06	1.1
SH3BP5	3.93E-10	0.196127056	0.129	0.087	9.48E-06	1.1
PFKFB4	3.98E-10	0.177851627	0.06	0.032	9.61E-06	1.1
SLC16A13	4.00E-10	0.468437022	0.076	0.044	9.63E-06	1.1
FMO4	4.17E-10	-0.101062705	0.02	0.05	1.01E-05	1.1

CAV1	4.19E-10	-0.143612124	0.034	0.07	1.01E-05	1.1
RCN1	4.24E-10	-0.136632154	0.059	0.102	1.02E-05	1.1
FAM174B	4.26E-10	-0.116042454	0.032	0.067	1.03E-05	1.1
SDHC	4.28E-10	-0.107054266	0.108	0.165	1.03E-05	1.1
NCBP2	4.51E-10	-0.102789204	0.066	0.112	1.09E-05	1.1
GABARAP	4.55E-10	0.381862821	0.334	0.294	1.10E-05	1.1
NCMAP	4.81E-10	-0.11467457	0.058	0.101	1.16E-05	1.1
CACUL1	4.85E-10	0.308043393	0.426	0.392	1.17E-05	1.1
RPS16	4.88E-10	-0.3057218	0.302	0.377	1.18E-05	1.1
CPNE4	5.01E-10	-0.153592158	0.023	0.053	1.21E-05	1.1
ZNF512	5.12E-10	-0.120906652	0.043	0.082	1.23E-05	1.1
ATF1	5.39E-10	-0.113346639	0.065	0.11	1.30E-05	1.1
EPN2	5.52E-10	-0.115710624	0.094	0.147	1.33E-05	1.1
PTPRF	5.67E-10	-0.11654515	0.058	0.102	1.37E-05	1.1
IMMP1L	5.77E-10	-0.108551569	0.051	0.093	1.39E-05	1.1
AKAP12	6.00E-10	0.212771433	0.058	0.03	1.45E-05	1.1
BTBD3	6.00E-10	-0.127575575	0.076	0.124	1.45E-05	1.1
ANKRD33B	6.13E-10	0.189053123	0.08	0.047	1.48E-05	1.1
ROBO1	6.16E-10	-0.1271536	0.046	0.086	1.48E-05	1.1
HBS1L	6.18E-10	0.278048866	0.231	0.183	1.49E-05	1.1
UBASH3B	6.51E-10	-0.135786407	0.037	0.073	1.57E-05	1.1
DNM3	6.55E-10	-0.100019284	0.015	0.042	1.58E-05	1.1
11-Sep	6.64E-10	-0.135757196	0.083	0.132	1.60E-05	1.1
CTB-91J4.1	6.72E-10	-0.118809383	0	0.017	1.62E-05	1.1
BCL2L11	6.90E-10	-0.11061083	0.032	0.066	1.66E-05	1.1
UBE3A	6.93E-10	0.328731704	0.357	0.315	1.67E-05	1.1
FZD6	6.99E-10	-0.105726239	0.029	0.062	1.69E-05	1.1
CCDC14	7.59E-10	-0.122001549	0.103	0.156	1.83E-05	1.1
ZNF432	7.62E-10	-0.100841852	0.018	0.046	1.84E-05	1.1
PLSCR4	8.02E-10	-0.12167587	0.051	0.091	1.93E-05	1.1
C18orf8	8.43E-10	-0.111888907	0.041	0.078	2.03E-05	1.1
PCGF3	8.47E-10	-0.111966739	0.041	0.079	2.04E-05	1.1
TMEM87A	8.51E-10	-0.115512758	0.298	0.385	2.05E-05	1.1
CAPN2	8.59E-10	-0.151884535	0.241	0.318	2.07E-05	1.1
PPP2R2D	8.83E-10	0.237340155	0.159	0.114	2.13E-05	1.1
PPIP5K1	9.16E-10	-0.104592168	0.023	0.054	2.21E-05	1.1
PRR13	9.33E-10	-0.120113777	0.057	0.098	2.25E-05	1.1
RBPMS	9.58E-10	-0.110635997	0.715	0.816	2.31E-05	1.1
RP11-241G	9.65E-10	0.135716868	0.063	0.035	2.33E-05	1.1
ACP1	9.77E-10	-0.127119074	0.338	0.428	2.35E-05	1.1
ZNHIT3	1.00E-09	-0.103799193	0.16	0.226	2.42E-05	1.1
HDAC5	1.01E-09	0.140653652	0.059	0.032	2.43E-05	1.1
SAP30BP	1.04E-09	-0.108055827	0.044	0.082	2.51E-05	1.1
SIN3A	1.05E-09	-0.110425503	0.063	0.106	2.53E-05	1.1
PKIG	1.06E-09	0.166182098	0.082	0.049	2.55E-05	1.1
AC097724.	1.09E-09	-0.102180708	0.055	0.097	2.62E-05	1.1
FOXP2	1.10E-09	-0.100818193	0.013	0.038	2.65E-05	1.1

MANF	1.17E-09	0.140600071	0.061	0.033	2.82E-05	1.1
ENO1	1.21E-09	-0.273448216	0.445	0.541	2.91E-05	1.1
MIS18BP1	1.23E-09	-0.118300811	0.093	0.143	2.96E-05	1.1
RPS27	1.26E-09	0.159325488	0.79	0.793	3.04E-05	1.1
LIF	1.27E-09	-0.119189353	0.014	0.04	3.06E-05	1.1
PAIP1	1.29E-09	-0.128857107	0.148	0.209	3.11E-05	1.1
EPB41L2	1.29E-09	0.519339598	0.223	0.181	3.11E-05	1.1
SERP1	1.30E-09	0.24709784	0.308	0.262	3.13E-05	1.1
THUMPD2	1.31E-09	-0.105962804	0.02	0.049	3.16E-05	1.1
SOX9	1.36E-09	-0.101341575	0.154	0.216	3.28E-05	1.1
CCNI	1.38E-09	-0.13389313	0.522	0.622	3.32E-05	1.1
WDR1	1.43E-09	-0.119309728	0.103	0.156	3.44E-05	1.1
TMEM60	1.43E-09	-0.106232271	0.07	0.116	3.46E-05	1.1
NBN	1.47E-09	-0.112937881	0.04	0.077	3.53E-05	1.1
ZNF90	1.47E-09	-0.197925132	0.084	0.131	3.54E-05	1.1
SLC10A7	1.48E-09	-0.122527136	0.035	0.069	3.57E-05	1.1
C5orf15	1.49E-09	-0.106758953	0.036	0.071	3.59E-05	1.1
CNN2	1.51E-09	-0.116147	0.015	0.041	3.64E-05	1.1
RP11-443B	1.51E-09	0.100009012	0.047	0.023	3.64E-05	1.1
LIFR	1.52E-09	-0.142913743	0.078	0.125	3.66E-05	1.1
ABR	1.53E-09	-0.123744239	0.026	0.057	3.70E-05	1.1
KIF2A	1.60E-09	-0.124375613	0.148	0.209	3.86E-05	1.1
MIER1	1.60E-09	0.30328971	0.218	0.173	3.87E-05	1.1
MARS	1.62E-09	-0.131293971	0.097	0.148	3.91E-05	1.1
TRAK2	1.65E-09	-0.138455032	0.018	0.046	3.97E-05	1.1
KPNA6	1.67E-09	0.313870219	0.309	0.266	4.03E-05	1.1
CCNG2	1.68E-09	0.256372394	0.186	0.14	4.05E-05	1.1
ANKH	1.71E-09	-0.165727816	0.045	0.083	4.12E-05	1.1
ARRDC3-AS	1.71E-09	-0.108902907	0.026	0.057	4.13E-05	1.1
GMEB1	1.74E-09	-0.104427224	0.038	0.073	4.19E-05	1.1
SCAF11	1.79E-09	-0.120989359	0.239	0.315	4.32E-05	1.1
PIK3C3	1.83E-09	-0.106280185	0.064	0.108	4.41E-05	1.1
RPL27A	1.92E-09	-0.194370773	0.405	0.488	4.62E-05	1.1
ABCA5	1.94E-09	-0.114200364	0.085	0.133	4.67E-05	1.1
ZC3H15	1.96E-09	0.275634316	0.301	0.256	4.73E-05	1.1
BDP1	2.00E-09	0.302008042	0.244	0.197	4.82E-05	1.1
RBBP8	2.04E-09	-0.113807381	0.073	0.117	4.91E-05	1.1
VPS37B	2.11E-09	0.191526455	0.191	0.142	5.08E-05	1.1
NDRG2	2.11E-09	-0.150773321	0.205	0.274	5.09E-05	1.1
VLDLR-AS1	2.21E-09	-0.107195842	0.01	0.033	5.33E-05	1.1
BMPR1B	2.22E-09	-0.175018178	0.014	0.04	5.35E-05	1.1
EFTUD1	2.29E-09	-0.148514179	0.071	0.116	5.51E-05	1.1
RAB8B	2.36E-09	0.272398009	0.2	0.152	5.69E-05	1.1
SH3BGRL3	2.42E-09	-0.186597794	0.028	0.06	5.82E-05	1.1
RP11-111H	2.43E-09	-0.102496953	0.014	0.039	5.85E-05	1.1
POLR2A	2.43E-09	-0.103686552	0.106	0.159	5.87E-05	1.1
RPTOR	2.52E-09	-0.126951935	0.036	0.071	6.07E-05	1.1

CWF19L2	2.53E-09	-0.105998558	0.101	0.154	6.10E-05	1.1
TMED5	2.58E-09	-0.127219628	0.073	0.117	6.22E-05	1.1
ENTPD4	2.63E-09	-0.115846742	0.032	0.065	6.35E-05	1.1
PSAP	2.71E-09	-0.150128472	0.05	0.088	6.53E-05	1.1
CDK6	2.71E-09	-0.115849838	0.18	0.247	6.55E-05	1.1
UNK	2.72E-09	-0.105643834	0.028	0.059	6.57E-05	1.1
C1orf43	2.74E-09	-0.11239518	0.082	0.129	6.62E-05	1.1
EPAS1	2.83E-09	-0.124377528	0.053	0.093	6.83E-05	1.1
PEG10	2.84E-09	0.215836694	0.088	0.055	6.86E-05	1.1
COX6B1	2.92E-09	0.210433675	0.723	0.743	7.04E-05	1.1
WBP5	3.08E-09	0.311223398	0.42	0.387	7.43E-05	1.1
RILPL2	3.09E-09	-0.102703977	0.025	0.056	7.45E-05	1.1
ADGRL3-AS	3.12E-09	0.21218394	0.692	0.656	7.53E-05	1.1
MBTD1	3.21E-09	-0.115067714	0.128	0.186	7.74E-05	1.1
ATP5G1	3.26E-09	0.288640585	0.239	0.195	7.87E-05	1.1
AC008074.	3.29E-09	0.146142046	0.062	0.035	7.92E-05	1.1
MIPOL1	3.46E-09	-0.100860709	0.016	0.042	8.34E-05	1.1
NEDD4	3.46E-09	-0.131448229	0.056	0.096	8.35E-05	1.1
RPS7	3.48E-09	0.157044183	0.976	0.966	8.40E-05	1.1
CARS2	3.49E-09	-0.116265287	0.028	0.059	8.41E-05	1.1
SGPP1	3.54E-09	-0.102328828	0.038	0.072	8.54E-05	1.1
STK17B	3.63E-09	-0.105011228	0.088	0.135	8.75E-05	1.1
RTN3	3.65E-09	-0.120154434	0.15	0.211	8.80E-05	1.1
RPS28	3.68E-09	-0.222325077	0.08	0.125	8.88E-05	1.1
CLCN3	3.72E-09	-0.10532145	0.144	0.204	8.96E-05	1.1
MBTPS1	3.75E-09	-0.106652531	0.032	0.064	9.04E-05	1.1
NDUFAF4	3.81E-09	0.246479681	0.141	0.101	9.18E-05	1.1
ERBB4	3.98E-09	-0.153733136	0.08	0.126	9.61E-05	1.1
LIAS	4.26E-09	0.185839658	0.089	0.056	0.0001027	1.1
CSNK1A1	4.37E-09	0.256858165	0.592	0.598	0.0001054	1.1
RP11-141M	4.38E-09	-0.120186751	0.041	0.076	0.0001056	1.1
LSS	4.45E-09	-0.106155437	0.027	0.058	0.0001072	1.1
RP11-274H	4.57E-09	-0.110398596	0.031	0.063	0.0001101	1.1
MYO19	4.81E-09	-0.104449772	0.021	0.049	0.0001159	1.1
DEGS1	4.83E-09	-0.110695241	0.06	0.101	0.0001165	1.1
POU2F1	4.90E-09	-0.125549641	0.101	0.151	0.0001182	1.1
CHMP4B	5.01E-09	0.266293911	0.154	0.113	0.0001209	1.1
PPP2R1A	5.03E-09	-0.107527071	0.032	0.064	0.0001212	1.1
PLA2G16	5.05E-09	-0.117842694	0.093	0.142	0.0001217	1.1
TMTC3	5.09E-09	-0.10154808	0.031	0.063	0.0001227	1.1
CCT2	5.19E-09	0.241989756	0.435	0.397	0.0001252	1.1
IRAK1BP1	5.38E-09	-0.110849261	0.046	0.083	0.0001297	1.1
SYNE1	5.46E-09	0.172462143	0.05	0.026	0.0001316	1.1
PLCB4	5.64E-09	-0.140037804	0.088	0.135	0.000136	1.1
MAPRE1	5.67E-09	0.268768052	0.19	0.147	0.0001368	1.1
GNG12-AS1	5.78E-09	-0.121051711	0.028	0.059	0.0001394	1.1
SNX6	5.78E-09	-0.105903145	0.303	0.387	0.0001394	1.1

RP5-968J1.	6.11E-09	0.101946782	0.047	0.024	0.0001474	1.1
ITPR1	6.12E-09	-0.108819397	0.021	0.049	0.0001476	1.1
RP11-539L:	6.25E-09	0.103965166	0.046	0.023	0.0001507	1.1
COMT	6.34E-09	-0.102146219	0.031	0.062	0.0001528	1.1
SCYL2	6.37E-09	0.295439841	0.288	0.244	0.0001536	1.1
MTRNR2L1	6.75E-09	0.219141093	0.216	0.168	0.0001629	1.1
SSU72	6.96E-09	-0.104424193	0.023	0.051	0.0001677	1.1
RREB1	7.48E-09	-0.130939754	0.216	0.282	0.0001805	1.1
PPM1B	7.74E-09	-0.107681595	0.136	0.194	0.0001866	1.1
GPR75-ASB	7.80E-09	-0.118219943	0.11	0.162	0.000188	1.1
ENSA	7.80E-09	-0.102313427	0.321	0.404	0.0001881	1.1
NEK1	7.80E-09	-0.124692191	0.058	0.097	0.0001882	1.1
ZNF385C	7.89E-09	-0.10006983	0.018	0.044	0.0001903	1.1
POLH	8.01E-09	0.120392824	0.06	0.034	0.0001931	1.1
S100A16	8.23E-09	-0.194834643	0.07	0.112	0.0001985	1.1
C1orf27	8.30E-09	-0.108712305	0.09	0.138	0.0002002	1.1
TCEAL4	8.39E-09	0.282569727	0.258	0.216	0.0002023	1.1
TMEM41A	8.43E-09	-0.119104293	0.167	0.227	0.0002032	1.1
NCKAP1	8.61E-09	0.29558443	0.431	0.402	0.0002075	1.1
EPB41	8.71E-09	-0.105966397	0.106	0.157	0.0002101	1.1
ACTG1	9.17E-09	-0.229054835	0.653	0.713	0.0002211	1.1
CDC37	9.27E-09	-0.112859061	0.018	0.043	0.0002236	1.1
ADAR	9.48E-09	-0.115549293	0.084	0.129	0.0002285	1.1
RP11-557C	9.74E-09	0.219288752	0.084	0.053	0.0002348	1.1
KIF27	1.02E-08	-0.101468569	0.028	0.057	0.0002453	1.1
UBE2Q2	1.05E-08	-0.110492775	0.046	0.081	0.0002524	1.1
ZMYM2	1.07E-08	-0.100159642	0.296	0.383	0.0002578	1.1
RUBCN	1.07E-08	0.177184514	0.091	0.058	0.0002584	1.1
RAC1	1.08E-08	0.258767099	0.5	0.484	0.0002615	1.1
PPM1D	1.10E-08	0.171171536	0.102	0.067	0.0002643	1.1
XPC	1.13E-08	-0.103938364	0.017	0.042	0.000273	1.1
TUBB4B	1.15E-08	-0.135147948	0.032	0.063	0.0002777	1.1
RP11-758H	1.16E-08	0.125850239	0.035	0.016	0.0002809	1.1
TMX4	1.18E-08	-0.112601899	0.026	0.055	0.0002844	1.1
SEPP1	1.20E-08	0.419245671	0.378	0.357	0.0002898	1.1
CASD1	1.32E-08	-0.102447079	0.037	0.069	0.000318	1.1
ASNS	1.33E-08	-0.125368624	0.055	0.092	0.0003195	1.1
ANXA4	1.36E-08	-0.108344475	0.076	0.12	0.0003288	1.1
TRIM26	1.41E-08	-0.10826016	0.044	0.079	0.00034	1.1
PLEKHG1	1.43E-08	-0.109326725	0.134	0.19	0.0003438	1.1
PTCHD1	1.43E-08	-0.102283762	0.056	0.094	0.0003451	1.1
RP11-30J2(	1.55E-08	-0.107588864	0.017	0.042	0.0003742	1.1
ZNF219	1.57E-08	0.128253033	0.08	0.049	0.0003786	1.1
ATP2B1	1.57E-08	-0.10826756	0.025	0.053	0.0003796	1.1
NPC2	1.59E-08	-0.1483505	0.397	0.484	0.0003831	1.1
TIAM1	1.59E-08	0.412289366	0.339	0.301	0.000384	1.1
MYBL1	1.62E-08	-0.103002978	0.023	0.05	0.0003894	1.1

TBC1D9B	1.63E-08	-0.104925057	0.02	0.046	0.000394	1.1
SKI	1.74E-08	-0.102730678	0.01	0.031	0.0004198	1.1
PTS	1.76E-08	-0.100832379	0.074	0.117	0.0004255	1.1
PRKCA	1.78E-08	0.25687659	0.116	0.081	0.0004296	1.1
GPX1	1.80E-08	-0.11428114	0.023	0.05	0.0004341	1.1
CSN3	1.84E-08	0.153028243	0.017	0.005	0.0004448	1.1
FMR1	1.86E-08	0.17706897	0.106	0.071	0.0004479	1.1
STAT3	1.89E-08	0.335446881	0.524	0.516	0.0004568	1.1
GSTO2	1.90E-08	0.18843632	0.147	0.107	0.0004578	1.1
ERI3	1.91E-08	-0.112852677	0.023	0.05	0.0004606	1.1
OPA1	1.96E-08	-0.117319623	0.08	0.123	0.0004734	1.1
RP11-685M	1.97E-08	0.162768308	0.073	0.044	0.0004746	1.1
LPL	1.97E-08	-0.110261978	0.034	0.066	0.0004749	1.1
DDI2	2.05E-08	-0.103296049	0.074	0.116	0.0004953	1.1
PDPR	2.10E-08	-0.106865267	0.024	0.051	0.000506	1.1
KLHDC10	2.14E-08	0.220745521	0.151	0.111	0.000516	1.1
IGFBP5	2.16E-08	-0.17662346	0.041	0.074	0.0005219	1.1
MTRNR2L1	2.17E-08	0.250107942	0.315	0.268	0.0005228	1.1
FTO	2.29E-08	-0.111657396	0.189	0.254	0.0005512	1.1
TSPAN9	2.32E-08	-0.102125498	0.044	0.078	0.0005585	1.1
RPL18	2.32E-08	-0.225771742	0.089	0.132	0.0005586	1.1
GNAS	2.35E-08	0.271596281	0.591	0.603	0.0005676	1.1
RP11-541P	2.42E-08	-0.112482444	0.048	0.084	0.0005826	1.1
KDM5C	2.44E-08	0.173292705	0.11	0.075	0.0005895	1.1
CPAMD8	2.59E-08	-0.148529593	0.085	0.129	0.0006246	1.1
SAR1A	2.63E-08	0.262150506	0.243	0.202	0.0006353	1.1
UBA2	2.64E-08	0.219716865	0.171	0.131	0.0006372	1.1
RBPJ	2.65E-08	0.273947426	0.422	0.395	0.0006395	1.1
P4HA1	2.71E-08	0.547079415	0.293	0.262	0.0006523	1.1
TRAM1	2.72E-08	0.225679585	0.247	0.204	0.0006567	1.1
KANSL3	2.72E-08	-0.1029884	0.039	0.072	0.0006569	1.1
LRP2	2.75E-08	0.298401231	0.264	0.221	0.0006636	1.1
MYEOV2	2.78E-08	-0.113711434	0.024	0.051	0.0006713	1.1
ZCWPW2	2.87E-08	-0.119639674	0.046	0.08	0.0006919	1.1
BET1	2.94E-08	-0.101452416	0.111	0.161	0.0007101	1.1
SPIN1	2.97E-08	0.26917881	0.216	0.176	0.0007159	1.1
MTURN	3.01E-08	-0.102567298	0.075	0.117	0.0007248	1.1
PATL1	3.03E-08	-0.108581446	0.114	0.163	0.0007303	1.1
C1orf52	3.12E-08	0.134937517	0.072	0.044	0.0007521	1.1
PSMB4	3.14E-08	-0.107267725	0.109	0.158	0.0007567	1.1
OPTN	3.14E-08	-0.101331159	0.141	0.197	0.0007578	1.1
TOMM20	3.24E-08	0.199173426	0.284	0.238	0.000782	1.1
SEC11A	3.28E-08	0.214647969	0.546	0.53	0.0007907	1.1
ELP4	3.39E-08	-0.101325954	0.02	0.045	0.000817	1.1
CBLC	3.40E-08	-0.10763222	0.045	0.08	0.0008193	1.1
INSR	3.41E-08	-0.171600608	0.586	0.674	0.0008221	1.1
RPL28	3.49E-08	-0.404738657	0.098	0.14	0.0008424	1.1

PAXBP1	3.61E-08	-0.103673417	0.066	0.105	0.0008705	1.1
SSR4	3.62E-08	-0.120190608	0.019	0.043	0.000874	1.1
SLK	3.67E-08	0.197905469	0.165	0.123	0.0008856	1.1
BRD4	4.01E-08	0.202631293	0.204	0.16	0.0009672	1.1
CUL3	4.03E-08	0.313587972	0.37	0.338	0.0009717	1.1
SMARCA4	4.39E-08	-0.118917746	0.107	0.155	0.0010593	1.1
PGAP1	4.40E-08	0.154002969	0.09	0.058	0.0010598	1.1
RASSF3	4.40E-08	0.214704114	0.181	0.138	0.0010621	1.1
SCAI	4.46E-08	-0.106157917	0.058	0.095	0.0010743	1.1
TPR	4.47E-08	-0.113599874	0.121	0.171	0.0010781	1.1
RRBP1	4.62E-08	-0.134941068	0.047	0.081	0.0011139	1.1
MRE11A	4.91E-08	-0.116581616	0.05	0.085	0.0011846	1.1
HDLBP	4.94E-08	-0.101759002	0.154	0.211	0.0011901	1.1
STX8	4.96E-08	-0.108546859	0.158	0.215	0.0011969	1.1
CCNL1	4.97E-08	0.288877821	0.418	0.389	0.0011986	1.1
TNKS1BP1	4.99E-08	-0.109191937	0.033	0.063	0.001203	1.1
BCAP31	5.04E-08	-0.105134425	0.045	0.078	0.0012157	1.1
CTC-471J1.	5.06E-08	0.195021103	0.742	0.762	0.0012211	1.1
DAB1	5.21E-08	-0.110709911	0.04	0.073	0.0012564	1.1
CHD4	5.33E-08	-0.100931571	0.067	0.107	0.001286	1.1
RP13-726E	5.34E-08	-0.107555744	0.028	0.055	0.0012871	1.1
DDX50	5.54E-08	-0.118811447	0.083	0.125	0.0013353	1.1
MBOAT2	5.99E-08	-0.114351776	0.085	0.128	0.0014455	1.1
PIP	6.20E-08	0.211083889	0.071	0.044	0.0014955	1.1
SEC63	6.36E-08	0.261726397	0.281	0.241	0.0015335	1.1
GIPC2	6.82E-08	0.165726479	0.057	0.033	0.0016438	1.1
ACADVL	6.87E-08	-0.128306622	0.024	0.05	0.0016569	1.1
RAB21	6.89E-08	0.222245122	0.228	0.185	0.0016605	1.1
RPS5	6.91E-08	-0.32379374	0.247	0.306	0.0016669	1.1
AP3D1	6.97E-08	-0.10428167	0.035	0.065	0.001681	1.1
MRPL20	7.16E-08	-0.107823376	0.045	0.078	0.0017271	1.1
FAM60A	7.22E-08	-0.145472582	0.338	0.415	0.0017413	1.1
EGR1	7.30E-08	-0.164977428	0.051	0.086	0.0017605	1.1
GSTM3	7.43E-08	-0.104991909	0.068	0.107	0.0017925	1.1
KMT2E	7.69E-08	0.311008646	0.418	0.398	0.0018533	1.1
SPG20	7.73E-08	-0.101512464	0.084	0.126	0.0018651	1.1
TIMM10	7.81E-08	0.244225075	0.281	0.242	0.0018821	1.1
CTC-425F1.	8.01E-08	0.22040676	0.107	0.073	0.0019324	1.1
TP53	8.29E-08	-0.110450751	0.044	0.076	0.001998	1.1
FBL	8.50E-08	0.204915053	0.248	0.206	0.0020489	1.1
TMEM30A	8.72E-08	-0.103946634	0.12	0.17	0.0021019	1.1
FNBP4	8.72E-08	0.266367249	0.471	0.451	0.0021037	1.1
CBL	8.75E-08	-0.102192706	0.065	0.102	0.002111	1.1
ETFA	9.02E-08	-0.103177461	0.072	0.112	0.0021739	1.1
TCEB2	9.37E-08	-0.126325498	0.023	0.048	0.0022601	1.1
GSTA1	9.39E-08	0.404766038	0.115	0.08	0.0022631	1.1
PSMD4	9.69E-08	-0.115931621	0.067	0.105	0.0023368	1.1

ELOVL7	9.89E-08	-0.135919259	0.058	0.093	0.002384	1.1
GPX4	1.00E-07	-0.121664693	0.02	0.045	0.0024204	1.1
HNRNPUL1	1.04E-07	-0.116154479	0.077	0.117	0.0025162	1.1
MTHFD1L	1.05E-07	-0.17381634	0.241	0.303	0.0025411	1.1
FOXK2	1.08E-07	0.290677107	0.326	0.293	0.0026049	1.1
RP11-371F	1.14E-07	-0.114254956	0.017	0.04	0.0027474	1.1
NFRKB	1.18E-07	0.127910461	0.078	0.05	0.0028468	1.1
LGALS8	1.19E-07	-0.113680948	0.091	0.134	0.0028584	1.1
ETS2	1.22E-07	0.193665213	0.155	0.115	0.0029531	1.1
CTD-2528L	1.24E-07	0.159645874	0.055	0.031	0.0029868	1.1
KLF5	1.24E-07	0.262651313	0.259	0.217	0.0029919	1.1
EML5	1.24E-07	-0.118647438	0.079	0.119	0.0029943	1.1
OSBPL8	1.27E-07	-0.113335861	0.125	0.176	0.0030732	1.1
CHMP2B	1.36E-07	0.189831301	0.142	0.105	0.0032913	1.1
PRMT1	1.38E-07	-0.110262424	0.015	0.037	0.0033165	1.1
MAFF	1.43E-07	0.236819269	0.233	0.191	0.0034411	1.1
REXO2	1.44E-07	-0.110621294	0.056	0.09	0.0034614	1.1
POMP	1.45E-07	-0.207117816	0.611	0.678	0.0035046	1.1
MAP4	1.47E-07	-0.119807907	0.374	0.452	0.0035416	1.1
EIF1	1.59E-07	-0.137171402	0.373	0.448	0.0038353	1.1
TULP3	1.66E-07	-0.102439148	0.058	0.093	0.0039995	1.1
ARHGDIB	1.70E-07	0.289698721	0.208	0.169	0.004088	1.1
IL32	1.86E-07	-0.132068921	0.048	0.08	0.0044756	1.1
EEF1D	1.93E-07	-0.152465796	0.093	0.134	0.0046567	1.1
METAP1	1.93E-07	0.186915815	0.115	0.082	0.0046653	1.1
ZMYM5	1.99E-07	0.240474963	0.201	0.162	0.0047901	1.1
KCNK5	2.09E-07	-0.100725273	0.019	0.042	0.0050447	1.1
ZRANB1	2.13E-07	0.227541942	0.186	0.147	0.0051394	1.1
YBX1	2.15E-07	-0.131467306	0.293	0.358	0.0051941	1.1
TPD52	2.24E-07	0.235961474	0.199	0.16	0.0054001	1.1
HADHA	2.29E-07	0.259953481	0.373	0.347	0.0055257	1.1
SIL1	2.30E-07	-0.101415099	0.078	0.119	0.0055572	1.1
IRF2BP2	2.34E-07	0.254154497	0.159	0.123	0.0056396	1.1
SMARCC2	2.40E-07	-0.115604716	0.068	0.105	0.0057819	1.1
EYS	2.41E-07	-0.10022182	0.046	0.078	0.0058066	1.1
OSTC	2.58E-07	0.226293092	0.512	0.502	0.0062331	1.1
OCLN	2.63E-07	0.310211614	0.335	0.307	0.0063433	1.1
PNPLA8	2.80E-07	0.370693806	0.312	0.281	0.0067531	1.1
UGGT1	2.97E-07	-0.103373403	0.033	0.061	0.0071698	1.1
KIAA1217	2.99E-07	-0.117239587	0.769	0.842	0.0072198	1.1
ARHGEF12	3.10E-07	0.29293275	0.448	0.427	0.0074813	1.1
RPS18	3.38E-07	0.121112547	0.694	0.671	0.0081569	1.1
GABRP	3.46E-07	-0.183478626	0.496	0.569	0.0083465	1.1
DSP	3.48E-07	-0.109754268	0.29	0.362	0.0084002	1.1
PRKCI	3.59E-07	0.270065426	0.266	0.228	0.0086447	1.1
WHSC1	3.62E-07	-0.100544643	0.089	0.13	0.0087207	1.1
ARIH1	3.68E-07	0.296638382	0.439	0.415	0.0088776	1.1

MYO10	3.68E-07	-0.104714567	0.15	0.2	0.0088784	1.1
COL6A1	3.93E-07	-0.132443894	0.027	0.052	0.0094733	1.1
AK2	4.67E-07	0.184525865	0.17	0.132	0.0112585	1.1
PTRF	4.81E-07	0.212406718	0.206	0.166	0.011594	1.1
MAN1A2	5.07E-07	-0.119358622	0.145	0.194	0.0122237	1.1
PAIP2	5.34E-07	-0.121297985	0.102	0.144	0.0128811	1.1
LAMP2	5.63E-07	0.304466759	0.278	0.243	0.0135826	1.1
TRAF6	5.65E-07	0.143494829	0.079	0.052	0.0136354	1.1
UQCRHL	5.77E-07	0.244554303	0.302	0.271	0.0139179	1.1
RBMX	6.29E-07	-0.10177201	0.203	0.261	0.0151576	1.1
NPC1	7.19E-07	0.343072143	0.339	0.318	0.0173285	1.1
LSM5	7.26E-07	0.237404791	0.444	0.434	0.0174993	1.1
HMGCS1	7.63E-07	-0.122602672	0.194	0.25	0.0183949	1.1
CDKN1B	7.67E-07	0.122568393	0.088	0.06	0.0185013	1.1
SPECC1	7.96E-07	-0.117396256	0.036	0.063	0.0191971	1.1
LATS1	7.99E-07	-0.112647012	0.079	0.116	0.0192609	1.1
LALBA	7.99E-07	0.715802396	0.023	0.01	0.0192751	1.1
GCH1	8.12E-07	-0.118605284	0.065	0.1	0.0195679	1.1
TPRKB	8.22E-07	0.248582706	0.24	0.204	0.0198207	1.1
FIGN	9.21E-07	-0.135285578	0.103	0.144	0.022203	1.1
PTN	9.21E-07	0.624743553	0.257	0.221	0.0222124	1.1
MPZL3	9.72E-07	0.282962587	0.328	0.299	0.0234339	1.1
PPP4R1	9.75E-07	0.222145723	0.232	0.194	0.0235129	1.1
LRRC1	9.86E-07	0.15051928	0.085	0.057	0.0237802	1.1
MECP2	9.89E-07	-0.103165256	0.05	0.08	0.0238536	1.1
HAPLN3	1.00E-06	-0.103164228	0.022	0.044	0.0241243	1.1
SEMA3C	1.01E-06	0.219233662	0.088	0.06	0.0242693	1.1
FXYD3	1.06E-06	0.235472181	0.532	0.52	0.0256236	1.1
IGFBP3	1.11E-06	0.267642396	0.143	0.108	0.0267934	1.1
ACTR3	1.24E-06	0.243240897	0.326	0.294	0.0297807	1.1
LOX	1.30E-06	-0.101797977	0.038	0.064	0.0314246	1.1
UBE2G2	1.41E-06	-0.10618206	0.045	0.073	0.0339363	1.1
PDGFRL	1.43E-06	-0.101891536	0.036	0.063	0.0344426	1.1
PRNP	1.50E-06	-0.109003175	0.105	0.145	0.0361134	1.1
RPS8	1.53E-06	0.12392114	0.758	0.78	0.0368226	1.1
INSIG2	1.53E-06	0.310279072	0.261	0.227	0.037013	1.1
TULP2	1.54E-06	0.106286816	0.048	0.028	0.0372269	1.1
EDF1	1.79E-06	-0.135433876	0.055	0.086	0.0432543	1.1
KIF13B	1.91E-06	0.33873057	0.314	0.288	0.0461031	1.1
AZIN1	1.96E-06	0.169276466	0.146	0.112	0.047181	1.1
RTKN2	2.01E-06	0.137083637	0.051	0.03	0.04837	1.1
DCTN6	2.03E-06	0.250202697	0.364	0.338	0.0489333	1.1
MREG	2.04E-06	0.191361234	0.118	0.087	0.0490934	1.1
TFDP2	2.06E-06	-0.10788646	0.19	0.242	0.0497506	1.1
IL20	2.11E-06	-0.127576246	0.004	0.017	0.0508488	1.1
GPC5	2.12E-06	-0.103175248	0.023	0.045	0.0511968	1.1
LIPH	2.17E-06	0.277758372	0.487	0.462	0.0522556	1.1

RP11-299J3	2.25E-06	0.126448139	0.079	0.053	0.0541945	1.1
AC004231.	2.26E-06	-0.121929143	0.043	0.071	0.0545396	1.1
EZR	2.36E-06	0.284187337	0.335	0.302	0.0568979	1.1
YWHAG	2.46E-06	0.253465047	0.254	0.223	0.0592211	1.1
SRSF2	2.48E-06	-0.103592396	0.063	0.095	0.0598143	1.1
WDR45B	2.52E-06	0.268836158	0.308	0.279	0.0607106	1.1
RNF19A	2.70E-06	0.302621128	0.619	0.627	0.0651434	1.1
SH3BGRL	2.83E-06	0.255145172	0.265	0.233	0.0682108	1.1
MAP3K1	3.21E-06	0.260059114	0.237	0.203	0.0774026	1.1
SRRM2	3.43E-06	0.179644157	0.227	0.19	0.0826229	1.1
YY1AP1	3.67E-06	0.190491945	0.193	0.159	0.0885757	1.1
DAPK1	3.99E-06	0.175698983	0.664	0.666	0.0961498	1.1
RPS15	4.33E-06	-0.298754063	0.14	0.182	0.1045211	1.1
STX3	4.36E-06	0.180487964	0.122	0.091	0.10515	1.1
RP1-117O3	4.58E-06	0.148725597	0.067	0.044	0.1104328	1.1
TCEB3	4.84E-06	0.129767115	0.093	0.065	0.1168001	1.1
PVRL2	4.89E-06	0.19671994	0.203	0.167	0.1179145	1.1
SMAP1	4.94E-06	-0.100027909	0.08	0.115	0.1190612	1.1
MGAT5	5.11E-06	0.269033558	0.329	0.303	0.1232093	1.1
RP11-496N	5.11E-06	0.254802919	0.027	0.013	0.1232464	1.1
LMO7	5.23E-06	-0.122568808	0.056	0.085	0.126044	1.1
ELMSAN1	5.33E-06	0.14669262	0.102	0.074	0.1284723	1.1
YWHAZ	5.37E-06	0.194529417	0.578	0.593	0.129497	1.1
CHIC2	5.70E-06	0.31591718	0.253	0.223	0.1375393	1.1
HMGA1	5.79E-06	-0.11899931	0.108	0.146	0.1395553	1.1
ARMCX3	6.22E-06	0.180064138	0.174	0.141	0.150094	1.1
RP11-817J1	6.39E-06	0.211038264	0.133	0.103	0.1541796	1.1
PREPL	6.44E-06	0.166667954	0.155	0.122	0.1552837	1.1
ACIN1	7.82E-06	0.147834723	0.173	0.139	0.1886407	1.1
TPT1	8.19E-06	-0.266383569	0.811	0.842	0.1974964	1.1
SAMD5	8.39E-06	0.1109856	0.079	0.054	0.2024175	1.1
CA2	8.78E-06	-0.105706895	0.048	0.075	0.2116791	1.1
LMTK2	9.66E-06	0.24785884	0.179	0.148	0.2328265	1.1
CTR9	9.76E-06	0.177700657	0.113	0.085	0.2354175	1.1
RP11-314N	1.03E-05	0.25852006	0.17	0.138	0.2490987	1.1
MRPL42	1.05E-05	0.212294663	0.208	0.175	0.2536335	1.1
USP38	1.08E-05	0.160424392	0.065	0.044	0.2613335	1.1
TEX14	1.16E-05	0.436259534	0.276	0.246	0.2802898	1.1
EPS8L2	1.18E-05	-0.10986375	0.024	0.044	0.2842522	1.1
SERF2	1.20E-05	-0.201732671	0.228	0.277	0.2904617	1.1
SEC23B	1.23E-05	-0.100599049	0.065	0.095	0.2964282	1.1
PAICS	1.25E-05	0.186841478	0.235	0.203	0.3010442	1.1
USP47	1.32E-05	0.257406048	0.331	0.307	0.3186457	1.1
SARS	1.38E-05	0.17871216	0.228	0.196	0.3320313	1.1
WBP4	1.38E-05	0.178714069	0.146	0.116	0.3330945	1.1
EIF5	1.46E-05	0.263297126	0.225	0.195	0.3523784	1.1
RFC1	1.51E-05	0.209999862	0.283	0.255	0.3630211	1.1

CCNK	1.53E-05	0.204615237	0.145	0.116	0.3700589	1.1
KLHL24	1.67E-05	0.241981444	0.297	0.271	0.4023469	1.1
SLU7	1.69E-05	0.180716036	0.163	0.131	0.4082606	1.1
RASGEF1C	1.74E-05	0.189416949	0.093	0.068	0.4184393	1.1
TOX3	2.05E-05	0.115715348	0.047	0.029	0.494698	1.1
SNHG8	2.22E-05	0.244577005	0.336	0.312	0.5348056	1.1
BARX2	2.24E-05	0.256481731	0.401	0.377	0.5404099	1.1
FAM133B	2.31E-05	0.153345489	0.141	0.111	0.5579377	1.1
FHIT	2.41E-05	-0.126727364	0.343	0.402	0.5822366	1.1
ZNF165	2.46E-05	0.17446793	0.08	0.057	0.5927461	1.1
DSC2	2.56E-05	0.250662395	0.26	0.233	0.6176003	1.1
PFDN1	2.76E-05	0.198575315	0.258	0.226	0.6665597	1.1
SBF2	2.76E-05	0.284576184	0.622	0.671	0.6665799	1.1
SEL1L2	2.80E-05	-0.124488562	0.008	0.02	0.6740151	1.1
CLDN1	2.90E-05	-0.119012818	0.367	0.429	0.7001915	1.1
C3orf52	2.97E-05	0.136468348	0.087	0.062	0.7167702	1.1
IFT57	3.01E-05	0.217066702	0.147	0.119	0.7247723	1.1
CAPZA2	3.03E-05	0.253425078	0.346	0.324	0.729449	1.1
KRT18	3.05E-05	0.125245044	0.104	0.077	0.7356417	1.1
TBC1D22B	3.06E-05	0.216576298	0.208	0.177	0.7370952	1.1
MTF2	3.07E-05	0.223414965	0.237	0.206	0.7406241	1.1
KRAS	3.40E-05	0.173520147	0.165	0.135	0.819016	1.1
COMMD6	3.53E-05	0.222297148	0.423	0.411	0.8522622	1.1
SESN2	3.54E-05	0.176129316	0.126	0.098	0.8547309	1.1
FGD4	3.71E-05	0.265404096	0.284	0.26	0.8954274	1.1
MPRIP-AS1	3.88E-05	0.140185548	0.072	0.05	0.9347512	1.1
HLA-DRB1	3.95E-05	0.134931396	0.081	0.058	0.9527708	1.1
NDUFV2	4.11E-05	0.185038942	0.176	0.146	0.9908862	1.1
KRT80	4.19E-05	0.10854784	0.055	0.036	1	1.1
AC016995.	4.20E-05	-0.154325308	0.169	0.208	1	1.1
FAM162A	4.27E-05	0.183218001	0.165	0.135	1	1.1
HMGN2	4.30E-05	0.116241721	0.08	0.057	1	1.1
ARMCX6	4.37E-05	0.122115214	0.09	0.067	1	1.1
EFNB2	4.65E-05	0.148640104	0.146	0.116	1	1.1
SF3B6	4.68E-05	0.132668335	0.649	0.67	1	1.1
RPL36	4.82E-05	-0.315255731	0.214	0.253	1	1.1
RPL3	4.83E-05	-0.142927047	0.323	0.377	1	1.1
RP11-437B	5.08E-05	0.247765665	0.404	0.391	1	1.1
TAGAP	5.12E-05	0.105601408	0.037	0.022	1	1.1
MAGI1	5.27E-05	0.181368934	0.783	0.819	1	1.1
IRF6	5.51E-05	0.215264849	0.242	0.214	1	1.1
RAB11A	5.59E-05	0.365748538	0.58	0.607	1	1.1
PIAS2	5.85E-05	0.155678044	0.115	0.089	1	1.1
OSBP	5.99E-05	0.194658076	0.164	0.136	1	1.1
UBE2G1	6.06E-05	0.240806528	0.207	0.179	1	1.1
GSTA4	6.14E-05	0.109899969	0.086	0.063	1	1.1
RPS9	6.37E-05	-0.358031455	0.266	0.311	1	1.1

FAM129A	6.41E-05	0.202694063	0.498	0.472	1	1.1
LMNA	6.64E-05	0.215348203	0.229	0.197	1	1.1
HIST1H4C	6.91E-05	0.262911394	0.246	0.219	1	1.1
AIM1	7.07E-05	0.362602809	0.325	0.31	1	1.1
TBCA	7.13E-05	-0.102698432	0.627	0.697	1	1.1
SERINC1	7.39E-05	0.213206065	0.323	0.301	1	1.1
IP6K2	7.51E-05	0.224368198	0.234	0.207	1	1.1
RPL12	7.89E-05	0.188971481	0.406	0.39	1	1.1
FABP6	8.96E-05	0.308038686	0.06	0.041	1	1.1
RPS6KB1	9.33E-05	0.111184355	0.102	0.077	1	1.1
KLF6	9.73E-05	0.244040689	0.526	0.526	1	1.1
PLK2	9.98E-05	0.177477624	0.129	0.103	1	1.1
FAU	0.000101	-0.176623145	0.183	0.22	1	1.1
LINC00887	0.000102	0.204148789	0.083	0.061	1	1.1
ADGRL2	0.000103	-0.123478425	0.102	0.133	1	1.1
DPCD	0.00011	0.154781204	0.094	0.071	1	1.1
PIWIL1	0.000112	0.188747227	0.073	0.053	1	1.1
MARK3	0.000128	0.20475683	0.438	0.431	1	1.1
SIAH2	0.00013	-0.119923959	0.043	0.065	1	1.1
HSPB11	0.000139	0.158270091	0.093	0.07	1	1.1
RUVBL1	0.00015	0.16744223	0.148	0.121	1	1.1
NXF1	0.000157	0.126103942	0.1	0.077	1	1.1
RPL8	0.00016	-0.333220996	0.163	0.197	1	1.1
PHKG1	0.000163	0.115936091	0.071	0.051	1	1.1
RSBN1	0.000166	0.115798251	0.078	0.057	1	1.1
NET1	0.000168	0.201277731	0.209	0.183	1	1.1
FEM1B	0.000171	0.125466626	0.147	0.118	1	1.1
DECR1	0.00018	0.16463098	0.165	0.138	1	1.1
BTG3	0.000186	0.189449768	0.213	0.187	1	1.1
PEX13	0.000197	0.153493426	0.139	0.113	1	1.1
CLCA4	0.000202	0.158520435	0.047	0.031	1	1.1
CANX	0.000209	0.172169457	0.352	0.331	1	1.1
SLC25A33	0.000221	0.310191366	0.153	0.128	1	1.1
DDX24	0.000225	0.212840694	0.607	0.633	1	1.1
LINC01198	0.00026	0.295504519	0.327	0.311	1	1.1
HBP1	0.000263	0.207108894	0.245	0.221	1	1.1
VCL	0.000266	0.182594646	0.34	0.317	1	1.1
PARD6B	0.000277	0.170686287	0.161	0.136	1	1.1
STRAP	0.000288	0.155501139	0.144	0.118	1	1.1
FBLIM1	0.000289	0.16486726	0.165	0.139	1	1.1
DDX47	0.000313	0.165475122	0.059	0.042	1	1.1
NBEAL1	0.000317	0.108239674	0.682	0.689	1	1.1
PPIH	0.000347	0.140836911	0.128	0.104	1	1.1
WASF2	0.000373	0.19921052	0.233	0.209	1	1.1
NFKB1	0.000375	0.423822225	0.399	0.402	1	1.1
RPL9	0.000378	0.12725286	0.668	0.673	1	1.1
RPL29	0.000379	-0.361228504	0.272	0.309	1	1.1

NIPBL	0.000394	0.263039847	0.47	0.48	1	1.1
ANP32B	0.000396	0.155819988	0.203	0.177	1	1.1
BCL10	0.000411	0.166767471	0.146	0.122	1	1.1
RP11-293M	0.000448	0.13469678	0.071	0.053	1	1.1
LEMD3	0.000458	0.126089418	0.081	0.062	1	1.1
DNAJB9	0.000489	0.128721662	0.075	0.056	1	1.1
CAMSAP1	0.000549	0.164517132	0.092	0.072	1	1.1
TGFBR3	0.00055	0.159621119	0.212	0.185	1	1.1
EIF4A3	0.000585	0.116855544	0.116	0.093	1	1.1
SNX1	0.00062	0.191590053	0.157	0.134	1	1.1
DUSP10	0.00062	0.119339636	0.075	0.056	1	1.1
NDUFA5	0.000651	0.210945118	0.309	0.295	1	1.1
RP11-659O	0.000656	0.217585525	0.205	0.182	1	1.1
AC195454.	0.000659	0.13027261	0.063	0.045	1	1.1
TANC2	0.000674	0.216333577	0.304	0.286	1	1.1
NR3C1	0.000724	0.211423146	0.263	0.244	1	1.1
GATA3	0.000739	0.189544916	0.182	0.158	1	1.1
LINGO1	0.000751	0.119273875	0.88	0.885	1	1.1
OAZ2	0.000767	0.143962755	0.099	0.079	1	1.1
RPS19	0.000778	-0.485398092	0.241	0.272	1	1.1
RNF11	0.000823	0.161719354	0.174	0.151	1	1.1
TCF7L2	0.000848	0.280037115	0.441	0.448	1	1.1
ZBTB16	0.000858	0.241389157	0.293	0.272	1	1.1
RAB5A	0.000867	0.184923779	0.237	0.215	1	1.1
NONO	0.000925	0.172144582	0.401	0.395	1	1.1
COPZ1	0.000949	0.210219032	0.355	0.348	1	1.1
TAGLN	0.000998	-0.272069324	0.016	0.028	1	1.1
CCT5	0.001003	-0.106761334	0.342	0.388	1	1.1
TTC33	0.001012	0.117028023	0.075	0.057	1	1.1
CCDC85C	0.001039	0.122700799	0.068	0.051	1	1.1
CTSV	0.001083	0.526596611	0.235	0.215	1	1.1
KLRD1	0.001179	0.142497767	0.086	0.068	1	1.1
C14orf2	0.00119	0.129661547	0.705	0.721	1	1.1
DDB1	0.001276	0.155298822	0.11	0.09	1	1.1
RP3-510L9.	0.001296	0.149932543	0.081	0.063	1	1.1
ACVR1	0.001353	0.172527948	0.137	0.116	1	1.1
FAM46B	0.001371	0.159665674	0.087	0.068	1	1.1
RP11-244M	0.001372	0.166323358	0.535	0.558	1	1.1
FAR1	0.001427	0.144196869	0.126	0.105	1	1.1
AKAP9	0.001443	0.209520268	0.369	0.36	1	1.1
BHLHE40	0.001477	0.138865286	0.118	0.097	1	1.1
PDZD11	0.001488	0.10125787	0.109	0.089	1	1.1
FNDC3A	0.00153	0.231760311	0.338	0.326	1	1.1
SYAP1	0.001647	0.112045838	0.125	0.103	1	1.1
ARFGEF3	0.001689	0.241022675	0.243	0.224	1	1.1
FNDC3B	0.001733	0.170722856	0.707	0.751	1	1.1
RP1-221C1	0.001751	0.118873395	0.075	0.058	1	1.1

PFKFB3	0.001937	0.117324313	0.089	0.071	1	1.1
FAM46A	0.002116	0.142416083	0.126	0.105	1	1.1
MACC1	0.002172	0.209699433	0.414	0.4	1	1.1
SNHG12	0.002172	0.115918244	0.08	0.063	1	1.1
LYRM4	0.002183	0.146323203	0.189	0.168	1	1.1
BCL6	0.002186	0.209447271	0.349	0.338	1	1.1
TXN	0.002237	-0.162414836	0.674	0.719	1	1.1
GALM	0.002257	0.117659148	0.087	0.069	1	1.1
USP9X	0.002357	0.20658819	0.334	0.326	1	1.1
TMEM123	0.002368	0.237704515	0.419	0.425	1	1.1
MAP3K8	0.00238	0.251087908	0.29	0.277	1	1.1
CTA-293F1	0.002396	0.253155538	0.139	0.121	1	1.1
ATP11B	0.002497	0.224596449	0.21	0.192	1	1.1
ZNF281	0.002498	0.112543383	0.105	0.086	1	1.1
MGEA5	0.002543	0.188466255	0.401	0.4	1	1.1
TM4SF1-AS	0.002573	0.125532493	0.11	0.091	1	1.1
RAPGEF1	0.00263	0.132175083	0.145	0.125	1	1.1
SASH1	0.002634	0.207086131	0.327	0.313	1	1.1
LARS	0.002708	0.174874704	0.256	0.238	1	1.1
RDX	0.002765	0.211279466	0.229	0.211	1	1.1
MRPL22	0.002833	0.168615303	0.271	0.255	1	1.1
RNF114	0.003161	0.161562159	0.163	0.143	1	1.1
ATP6V1H	0.003183	0.144318951	0.111	0.093	1	1.1
MCTS1	0.003266	0.175043975	0.298	0.287	1	1.1
ARHGAP10	0.003351	0.175633107	0.15	0.13	1	1.1
RP5-896L1	0.003465	0.173195114	0.355	0.348	1	1.1
CLIP1	0.003711	0.149789397	0.388	0.382	1	1.1
KAT6A	0.003768	0.196066337	0.262	0.244	1	1.1
FAXC	0.003813	0.110945543	0.043	0.031	1	1.1
MICA	0.00394	0.15859148	0.148	0.129	1	1.1
NPTN	0.003998	0.165803245	0.133	0.114	1	1.1
DNAJB1	0.004124	0.28865178	0.116	0.1	1	1.1
DHX15	0.004232	0.206459127	0.221	0.205	1	1.1
GRHL1	0.004285	0.181215087	0.267	0.25	1	1.1
CREBRF	0.004388	0.172101633	0.233	0.214	1	1.1
CSDE1	0.004469	0.127097928	0.569	0.59	1	1.1
NEK7	0.004576	0.204073254	0.215	0.197	1	1.1
RBMS2	0.004808	0.1777081	0.166	0.148	1	1.1
POGZ	0.004855	0.168571114	0.287	0.273	1	1.1
RCAN3	0.004859	0.226908876	0.163	0.197	1	1.1
SRSF10	0.004935	0.125590509	0.159	0.139	1	1.1
DYRK3	0.005561	0.138846646	0.087	0.071	1	1.1
CSNK2A1	0.005564	0.138732836	0.214	0.196	1	1.1
HIVEP2	0.00564	0.291553067	0.367	0.367	1	1.1
NDUFB1	0.006006	0.191499071	0.354	0.346	1	1.1
NMD3	0.006071	0.130861676	0.178	0.159	1	1.1
RP11-795H	0.00613	0.212246832	0.448	0.438	1	1.1

EGOT	0.006142	0.119475444	0.084	0.069	1	1.1
OTULIN	0.00665	0.130563371	0.075	0.06	1	1.1
CETN2	0.006717	0.176600018	0.148	0.13	1	1.1
FASTKD2	0.006831	0.158175717	0.115	0.099	1	1.1
MYNN	0.006919	0.115234141	0.098	0.082	1	1.1
MRPS21	0.00703	0.166032018	0.506	0.525	1	1.1
HIF1A-AS2	0.007229	0.165556119	0.158	0.139	1	1.1
KIF15	0.007434	0.10414174	0.024	0.015	1	1.1
MYSM1	0.007447	0.134177413	0.143	0.125	1	1.1
ITGB5-AS1	0.00748	0.210347598	0.049	0.037	1	1.1
LYRM5	0.007748	0.118416587	0.124	0.106	1	1.1
WDR26	0.007773	0.173460877	0.222	0.205	1	1.1
KIDINS220	0.007873	0.12804146	0.191	0.173	1	1.1
SESTD1	0.007874	0.152785298	0.603	0.631	1	1.1
RP1-313I6.	0.007986	0.137873477	0.074	0.06	1	1.1
IRF1	0.008071	0.151345962	0.162	0.143	1	1.1
PPP2R5E	0.008247	0.253569917	0.279	0.273	1	1.1
ELMO2	0.008387	0.124051336	0.075	0.06	1	1.1
YWHAH	0.009022	0.139775257	0.231	0.214	1	1.1
RPL35	0.00929	-0.199111195	0.292	0.321	1	1.1
EIF2S1	0.009342	0.150868241	0.272	0.257	1	1.1
EPB41L4B	0.009475	0.15560706	0.125	0.108	1	1.1
RALA	0.00956	0.173818726	0.187	0.17	1	1.1
ARHGEF26	0.009985	0.189958053	0.083	0.069	1	1.1
GRB7	0.010021	0.109262097	0.112	0.095	1	1.1
TMEM27	0.010334	0.101987518	0.055	0.043	1	1.1
MIR99AHG	0.010397	0.107019855	0.088	0.073	1	1.1
SERPINB9	0.010484	0.558157483	0.17	0.154	1	1.1
AC090498.	0.010734	0.182350323	0.384	0.392	1	1.1
PELI1	0.011079	0.231244301	0.447	0.457	1	1.1
SNU13	0.01134	0.166395746	0.475	0.485	1	1.1
TXLNG	0.011491	0.147071737	0.123	0.107	1	1.1
GTF2F2	0.011592	0.120574446	0.157	0.14	1	1.1
TCEA1	0.011774	0.133286079	0.115	0.099	1	1.1
NR1D2	0.012027	0.148363921	0.183	0.167	1	1.1
FAM69A	0.012172	0.106788811	0.071	0.058	1	1.1
SMR3B	0.012743	0.143444955	0.01	0.006	1	1.1
VEGFA	0.012876	0.179848624	0.232	0.217	1	1.1
SEMA3E	0.014102	0.111770944	0.082	0.068	1	1.1
ITGA6	0.014113	0.106638241	0.118	0.101	1	1.1
MPP5	0.014398	0.201779442	0.251	0.239	1	1.1
ALDH7A1	0.014569	0.125025541	0.11	0.095	1	1.1
RNF128	0.01466	0.163642129	0.108	0.094	1	1.1
MTDH	0.014838	0.167818823	0.331	0.324	1	1.1
RP1-292B1	0.014982	0.132715101	0.257	0.244	1	1.1
TPM4	0.015454	-0.106185451	0.459	0.511	1	1.1
SLC25A36	0.015614	0.114023222	0.187	0.17	1	1.1

CDC73	0.01566	0.137639367	0.187	0.172	1	1.1
RPL13A	0.01597	-0.170320983	0.506	0.549	1	1.1
SLAIN2	0.016135	0.106776561	0.105	0.09	1	1.1
WDR61	0.016534	0.14132134	0.115	0.101	1	1.1
HIST1H2BD	0.016633	0.164833737	0.249	0.293	1	1.1
HSPA1A	0.016726	0.172965669	0.041	0.054	1	1.1
TMEM167E	0.017565	0.105559421	0.078	0.065	1	1.1
NUS1	0.017759	0.149643613	0.129	0.115	1	1.1
AHDC1	0.018523	0.122953628	0.104	0.089	1	1.1
DHX9	0.019193	0.180841926	0.2	0.188	1	1.1
HINT1	0.019472	0.171173725	0.627	0.679	1	1.1
TRIM2	0.020175	0.208653392	0.307	0.302	1	1.1
PRRC1	0.020344	0.105897475	0.115	0.1	1	1.1
OTUD4	0.021795	0.130161118	0.142	0.127	1	1.1
RP11-840I1	0.022114	0.120499838	0.103	0.089	1	1.1
ATP5H	0.023601	0.163226238	0.374	0.378	1	1.1
GADD45B	0.024139	0.190099763	0.062	0.051	1	1.1
RLIM	0.024336	0.100234851	0.082	0.07	1	1.1
RCC1	0.024521	0.209313876	0.351	0.357	1	1.1
BANF1	0.024639	0.166389912	0.218	0.209	1	1.1
ZNF207	0.024874	0.154379096	0.474	0.491	1	1.1
URB1	0.025448	0.13300016	0.049	0.062	1	1.1
ACBD3	0.025642	0.147267325	0.162	0.147	1	1.1
PTBP2	0.026372	0.207578862	0.441	0.461	1	1.1
EWSR1	0.026768	0.102989379	0.133	0.118	1	1.1
GSTK1	0.02687	0.100810689	0.088	0.075	1	1.1
CAB39L	0.027432	0.24182439	0.123	0.11	1	1.1
WAPL	0.027686	0.164252995	0.182	0.17	1	1.1
MTMR1	0.029134	0.14309315	0.15	0.137	1	1.1
ETF1	0.03064	0.128503991	0.156	0.143	1	1.1
RAB3IP	0.03113	0.139342546	0.129	0.116	1	1.1
ZBTB43	0.032923	0.135841264	0.169	0.156	1	1.1
YY1	0.033141	0.135321335	0.18	0.167	1	1.1
TNFAIP3	0.034076	0.23565921	0.261	0.251	1	1.1
TMA7	0.035631	0.126711729	0.556	0.596	1	1.1
RP11-580I1	0.035795	0.112136338	0.069	0.058	1	1.1
RASGEF1B	0.036426	0.115767601	0.877	0.901	1	1.1
PPP3CA	0.037256	0.319040686	0.399	0.418	1	1.1
YTHDF3	0.039413	0.109804629	0.148	0.134	1	1.1
SNX9	0.039443	0.192882762	0.439	0.455	1	1.1
SET	0.039781	0.104646569	0.477	0.49	1	1.1
ANKRD28	0.041549	0.281520274	0.303	0.306	1	1.1
EFR3A	0.04207	0.176103232	0.173	0.162	1	1.1
DCP1A	0.042505	0.165168883	0.263	0.257	1	1.1
BAG4	0.042975	0.101209989	0.057	0.047	1	1.1
NCOR1	0.043444	0.176040014	0.356	0.365	1	1.1
CCT8	0.0439	0.146064786	0.376	0.381	1	1.1

GPS2	0.044068	0.120139825	0.123	0.111	1	1.1
CDK17	0.044093	0.11789244	0.148	0.135	1	1.1
SRP72	0.044699	0.124596898	0.192	0.182	1	1.1
TMSB4X	0.044993	0.166714307	0.886	0.89	1	1.1
SF3B1	0.050303	0.154429432	0.353	0.36	1	1.1
INTS6-AS1	0.050857	0.118978363	0.178	0.168	1	1.1
PUM3	0.051215	0.165855362	0.239	0.233	1	1.1
PSME1	0.051823	0.131515176	0.207	0.197	1	1.1
PGM3	0.052644	0.102010316	0.12	0.108	1	1.1
MAP3K14	0.057724	0.188070425	0.083	0.073	1	1.1
LSM14A	0.061121	0.183389515	0.345	0.352	1	1.1
WDFY2	0.061193	0.179767499	0.2	0.194	1	1.1
TP53BP2	0.063384	0.169813487	0.166	0.156	1	1.1
PEAK1	0.063554	0.247656783	0.187	0.178	1	1.1
MT-CO1	0.063628	-0.138981015	0.996	0.986	1	1.1
RIF1	0.064542	0.153043232	0.327	0.327	1	1.1
APTR	0.065632	0.129118155	0.089	0.079	1	1.1
CXCL2	0.06607	0.266243091	0.235	0.266	1	1.1
FBXO3	0.06653	0.124015113	0.107	0.096	1	1.1
STK38	0.067948	0.16707871	0.257	0.252	1	1.1
GPR107	0.068144	0.117324246	0.161	0.15	1	1.1
AC074391.	0.070327	0.197433322	0.048	0.058	1	1.1
TM9SF2	0.071987	0.19531634	0.194	0.187	1	1.1
PCBP2	0.07506	0.120753386	0.572	0.62	1	1.1
NT5C3A	0.075934	0.174109801	0.198	0.19	1	1.1
LRRFIP1	0.077152	0.162584953	0.501	0.521	1	1.1
COPB1	0.077455	0.143450763	0.218	0.211	1	1.1
LHFPL3	0.080404	0.13837629	0.309	0.311	1	1.1
SH2B1	0.084575	0.14254673	0.019	0.026	1	1.1
TNIP1	0.085074	0.122375424	0.185	0.175	1	1.1
FAM204A	0.086088	0.165461542	0.216	0.211	1	1.1
MDH1	0.086183	0.12690932	0.358	0.364	1	1.1
TMED10	0.089567	0.128611739	0.313	0.316	1	1.1
HDAC2	0.090604	0.13615563	0.213	0.208	1	1.1
VDAC1	0.092548	0.111882227	0.223	0.215	1	1.1
GPAT3	0.094549	0.119518221	0.092	0.082	1	1.1
TTC19	0.101878	0.159615011	0.215	0.21	1	1.1
SUMO1	0.105024	0.110490014	0.515	0.538	1	1.1
CDA	0.10919	0.135620244	0.018	0.013	1	1.1
TSPAN6	0.113085	0.113376145	0.144	0.135	1	1.1
MGST2	0.114161	0.147212618	0.177	0.17	1	1.1
BBC3	0.116355	0.121955601	0.074	0.066	1	1.1
FBXO11	0.116842	0.140293319	0.38	0.388	1	1.1
RHOA	0.117653	0.204625658	0.404	0.427	1	1.1
ANXA11	0.118417	0.131468233	0.248	0.245	1	1.1
NAP1L1	0.120352	0.111965808	0.434	0.447	1	1.1
SLC5A11	0.123499	0.112860209	0.025	0.031	1	1.1

TCERG1	0.123835	0.102527265	0.17	0.162	1	1.1
C12orf60	0.127502	0.130346315	0.102	0.093	1	1.1
FAS	0.131294	0.132353575	0.201	0.194	1	1.1
GRAMD3	0.133456	0.224070919	0.361	0.378	1	1.1
DAD1	0.135516	0.11601293	0.501	0.524	1	1.1
CTC1	0.138234	0.118147436	0.052	0.045	1	1.1
FAT1	0.140717	0.115946135	0.147	0.139	1	1.1
POLR2G	0.150146	0.128543843	0.178	0.172	1	1.1
VPS26A	0.151762	0.133664838	0.17	0.164	1	1.1
DDX6	0.152057	0.14966701	0.238	0.236	1	1.1
EIF3J	0.155953	0.129094891	0.156	0.15	1	1.1
MRPL45	0.169364	0.111653481	0.213	0.21	1	1.1
CA12	0.180431	0.163333354	0.237	0.231	1	1.1
GTF2I	0.18228	0.198631084	0.413	0.443	1	1.1
COPS4	0.183275	0.10673552	0.136	0.129	1	1.1
AP000487.	0.183595	0.159097999	0.208	0.203	1	1.1
NOTCH2	0.191895	0.109428956	0.174	0.196	1	1.1
VPS29	0.200534	0.136312981	0.381	0.392	1	1.1
CLEC2B	0.20174	0.100584412	0.106	0.119	1	1.1
HCAR2	0.20698	0.362379063	0.202	0.201	1	1.1
BNIP3L	0.209243	0.122277317	0.306	0.308	1	1.1
PPIL4	0.213015	0.122824496	0.162	0.157	1	1.1
TLE1	0.22556	0.145787438	0.229	0.227	1	1.1
LRRC23	0.227635	0.19367814	0.093	0.104	1	1.1
CLINT1	0.233901	0.204215591	0.353	0.374	1	1.1
STK39	0.23416	0.382342545	0.135	0.129	1	1.1
LMAN1	0.236251	0.1092495	0.205	0.202	1	1.1
BZW2	0.237847	0.13021341	0.118	0.111	1	1.1
TBC1D23	0.240125	0.125737113	0.16	0.156	1	1.1
USP3	0.240554	0.128821728	0.227	0.227	1	1.1
TOX4	0.245313	0.141742744	0.218	0.22	1	1.1
F11R	0.248248	0.102144651	0.178	0.175	1	1.1
PTPN12	0.270249	0.144105566	0.409	0.432	1	1.1
BCLAF1	0.280202	0.111765201	0.37	0.384	1	1.1
TMED2	0.28067	0.101610547	0.158	0.154	1	1.1
STMN1	0.280709	0.130795827	0.199	0.193	1	1.1
PRSS8	0.289335	0.307866109	0.065	0.06	1	1.1
RAP1B	0.291396	0.134551975	0.402	0.417	1	1.1
MED21	0.29258	0.1289671	0.219	0.245	1	1.1
YES1	0.294898	0.101188847	0.211	0.209	1	1.1
DUSP16	0.294901	0.139003986	0.252	0.258	1	1.1
HNRNPK	0.296121	0.102316988	0.455	0.48	1	1.1
ABTB2	0.296932	0.102300507	0.226	0.246	1	1.1
MUCL1	0.301653	0.148971271	0.155	0.152	1	1.1
PCNX	0.302399	0.103503324	0.178	0.197	1	1.1
KPNB1	0.306237	0.157792994	0.425	0.454	1	1.1
ITGA2	0.313868	0.128476244	0.453	0.465	1	1.1

ANKRD12	0.313909	0.224285492	0.52	0.579	1	1.1
BTBD7	0.314053	0.12357202	0.147	0.144	1	1.1
KRT8	0.316744	0.221270756	0.202	0.2	1	1.1
JAK1	0.326931	0.13962429	0.329	0.344	1	1.1
MON2	0.346021	0.107884599	0.238	0.264	1	1.1
PRPF40A	0.355481	0.113915892	0.176	0.175	1	1.1
SRGAP1	0.359249	0.123159347	0.301	0.33	1	1.1
TET2	0.36238	0.155569057	0.21	0.212	1	1.1
DIP2B	0.373224	0.156118927	0.313	0.327	1	1.1
NFKBIA	0.37434	0.212219402	0.563	0.645	1	1.1
EIF1B	0.378853	0.112309164	0.277	0.283	1	1.1
ALKBH1	0.381726	0.149609425	0.037	0.042	1	1.1
SNX8	0.384837	0.122414729	0.081	0.077	1	1.1
ELAVL1	0.386716	0.148405035	0.146	0.158	1	1.1
FABP7	0.388434	-0.869767756	0.222	0.221	1	1.1
CHCHD2	0.390908	0.102805155	0.235	0.233	1	1.1
MAPK6	0.390932	0.140698167	0.213	0.214	1	1.1
HNRNPH1	0.392667	0.132968097	0.403	0.419	1	1.1
BCAS2	0.400594	0.151341257	0.231	0.236	1	1.1
EPCAM	0.417878	0.119198	0.194	0.195	1	1.1
MRPL48	0.422371	0.101287172	0.118	0.116	1	1.1
NBPF14	0.435346	0.108487165	0.112	0.11	1	1.1
STX12	0.436243	0.148053539	0.414	0.461	1	1.1
CTTNBP2NI	0.456487	0.113975036	0.264	0.29	1	1.1
LAPTM4A	0.457037	0.1081942	0.237	0.238	1	1.1
C14orf166	0.463934	0.10255029	0.241	0.246	1	1.1
EIF3D	0.519563	0.102088516	0.393	0.416	1	1.1
DENND4A	0.521088	0.187162685	0.497	0.529	1	1.1
PPHLN1	0.521456	0.146509699	0.381	0.406	1	1.1
MAP3K2	0.52251	0.104132772	0.181	0.184	1	1.1
ZNF267	0.527283	0.120858842	0.175	0.177	1	1.1
ARRDC3	0.535787	0.192941632	0.596	0.669	1	1.1
TAOK3	0.536465	0.101272129	0.385	0.426	1	1.1
SLC27A4	0.541282	0.113742164	0.557	0.566	1	1.1
PCNP	0.543112	0.113611482	0.356	0.376	1	1.1
PHACTR4	0.546527	0.126759195	0.368	0.405	1	1.1
COL6A2	0.552858	0.124298228	0.142	0.141	1	1.1
TNKS2	0.558467	0.121255473	0.253	0.261	1	1.1
GBE1	0.561983	0.199589311	0.384	0.403	1	1.1
C5orf56	0.576679	0.11489465	0.125	0.125	1	1.1
GNL3	0.591599	0.139879974	0.27	0.279	1	1.1
TFCP2L1	0.607399	0.153468507	0.152	0.151	1	1.1
RP11-445F	0.612061	0.147180819	0.023	0.021	1	1.1
ATP5F1	0.619328	0.115968147	0.441	0.477	1	1.1
DSG3	0.620336	0.336819672	0.07	0.069	1	1.1
BBOX1	0.63138	0.110205126	0.251	0.27	1	1.1
WRN	0.650416	0.126125555	0.184	0.188	1	1.1

FBXW7	0.652792	0.105638223	0.303	0.329	1	1.1
SCGB2A2	0.668622	0.702690055	0.18	0.193	1	1.1
ACSS1	0.682044	0.155780295	0.015	0.016	1	1.1
UBA6	0.682724	0.11626789	0.314	0.343	1	1.1
EXT1	0.695604	0.19006375	0.531	0.574	1	1.1
COPA	0.695811	0.132977702	0.322	0.357	1	1.1
LSM3	0.710271	0.112415801	0.306	0.326	1	1.1
POLR2B	0.712998	0.104522605	0.204	0.21	1	1.1
LAMC1	0.721584	0.134234099	0.253	0.271	1	1.1
CLK1	0.721665	0.181325236	0.376	0.397	1	1.1
GNA12	0.730879	0.146253996	0.341	0.364	1	1.1
HIF1A	0.732749	0.233128781	0.371	0.399	1	1.1
ZNF462	0.737648	0.111320066	0.333	0.354	1	1.1
TMF1	0.738628	0.107415547	0.183	0.193	1	1.1
ZMAT2	0.769973	0.123068222	0.214	0.23	1	1.1
DPP6	0.782013	0.153030364	0.155	0.166	1	1.1
MPDZ	0.792176	0.118939761	0.176	0.181	1	1.1
PDE4D	0.806491	0.130882568	0.105	0.106	1	1.1
RIC3	0.811539	0.231431147	0.086	0.091	1	1.1
CELF1	0.816647	0.108603146	0.326	0.347	1	1.1
TRPC4AP	0.824163	0.128303628	0.218	0.233	1	1.1
MET	0.833323	0.250924781	0.326	0.362	1	1.1
AKT3	0.848736	0.253248967	0.432	0.492	1	1.1
SMCHD1	0.857323	0.158640267	0.276	0.295	1	1.1
PROSER2	0.885895	0.122026377	0.068	0.069	1	1.1
TJP1	0.909544	0.163594593	0.367	0.398	1	1.1
ERRFI1	0.918756	0.100973865	0.574	0.614	1	1.1
TCP1	0.955922	0.11772151	0.222	0.235	1	1.1
HECTD1	0.979761	0.222120912	0.25	0.269	1	1.1
UBE2B	0.981287	0.101425285	0.236	0.251	1	1.1
HADHB	0.985247	0.108625593	0.277	0.295	1	1.1
MYL12A	9.87E-259	1.035597972	0.945	0.834	2.38E-254	1.2
ITGA2	7.93E-242	1.248962197	0.737	0.384	1.91E-237	1.2
CLDN1	3.17E-207	1.399101597	0.658	0.344	7.64E-203	1.2
MYL6	2.17E-183	0.769170093	0.972	0.929	5.22E-179	1.2
ANXA1	9.24E-183	1.035134092	0.974	0.887	2.23E-178	1.2
ANXA2	2.17E-178	0.807391587	0.973	0.904	5.24E-174	1.2
TRPS1	1.82E-147	-1.034979721	0.523	0.773	4.39E-143	1.2
RARRES1	1.89E-146	-1.476701344	0.16	0.468	4.55E-142	1.2
GLIPR1	1.27E-145	1.015703399	0.557	0.287	3.06E-141	1.2
LIPH	1.00E-141	1.247614458	0.657	0.416	2.42E-137	1.2
RAB11FIP1	2.52E-137	0.927244858	0.705	0.463	6.07E-133	1.2
USP53	6.04E-137	0.831227697	0.826	0.624	1.46E-132	1.2
DBI	5.29E-135	-0.952759478	0.498	0.749	1.28E-130	1.2
CALD1	9.08E-134	0.990246892	0.77	0.55	2.19E-129	1.2
CHPT1	1.63E-128	-1.319686541	0.163	0.451	3.93E-124	1.2
MYL12B	5.28E-122	0.773230559	0.834	0.681	1.27E-117	1.2

PIK3R1	3.73E-120	-1.12839214	0.065	0.324	8.99E-116	1.2
ADAMTS9	4.10E-120	1.140898773	0.621	0.385	9.89E-116	1.2
MACC1	1.90E-115	0.933590867	0.595	0.35	4.59E-111	1.2
ELF5	2.72E-115	-0.935130349	0.093	0.356	6.55E-111	1.2
BCL2A1	4.07E-113	1.092037279	0.314	0.114	9.82E-109	1.2
MYO5B	2.39E-111	0.615299026	0.782	0.546	5.76E-107	1.2
PLAUR	2.76E-111	0.918281086	0.347	0.139	6.67E-107	1.2
TACSTD2	3.11E-111	0.821849502	0.832	0.689	7.51E-107	1.2
LTF	9.49E-111	-1.220727376	0.52	0.707	2.29E-106	1.2
KYNU	1.86E-107	-1.609979996	0.182	0.436	4.48E-103	1.2
EVA1C	4.59E-107	-1.034258478	0.248	0.506	1.11E-102	1.2
PLEKHS1	6.92E-105	-0.934399801	0.073	0.315	1.67E-100	1.2
ARHGAP26	3.79E-104	-0.875541814	0.748	0.859	9.14E-100	1.2
EMP1	1.79E-103	0.68597431	0.841	0.684	4.33E-99	1.2
ANKRD36C	4.21E-103	0.785989128	0.734	0.511	1.01E-98	1.2
ANK3	2.73E-102	-0.959297928	0.146	0.399	6.58E-98	1.2
RCAN1	1.06E-99	0.788912859	0.808	0.622	2.55E-95	1.2
MYO1E	6.80E-97	0.849207902	0.64	0.445	1.64E-92	1.2
ATP2B4	1.17E-95	0.926698619	0.435	0.227	2.83E-91	1.2
DOCK4	4.34E-92	-0.979738155	0.168	0.411	1.05E-87	1.2
LRRFIP1	2.23E-91	0.707872145	0.654	0.477	5.38E-87	1.2
CNKSR3	8.68E-91	-0.868551423	0.315	0.57	2.09E-86	1.2
ACTB	1.44E-89	0.747548404	0.737	0.582	3.48E-85	1.2
OVOS2	1.76E-88	-1.556829942	0.342	0.546	4.25E-84	1.2
MAP1B	4.53E-85	0.791465914	0.564	0.364	1.09E-80	1.2
PALLD	1.49E-82	0.68032946	0.654	0.476	3.59E-78	1.2
PGK1	4.65E-81	0.617772484	0.776	0.617	1.12E-76	1.2
EHF	6.60E-81	-0.738875005	0.415	0.65	1.59E-76	1.2
TEAD1	7.24E-80	0.70099231	0.524	0.326	1.75E-75	1.2
CHI3L2	8.01E-80	-0.954331686	0.32	0.55	1.93E-75	1.2
KRT23	5.19E-79	0.912419582	0.384	0.202	1.25E-74	1.2
TNFSF10	7.54E-79	-0.752314243	0.312	0.543	1.82E-74	1.2
GUCY1A3	1.92E-76	-0.740712953	0.076	0.274	4.63E-72	1.2
DAPP1	5.81E-76	0.769764982	0.55	0.374	1.40E-71	1.2
CAPN2	7.38E-76	0.660233263	0.448	0.255	1.78E-71	1.2
SFRP1	2.64E-75	-0.683332886	0.282	0.51	6.37E-71	1.2
FBLN5	4.16E-74	-1.055660901	0.169	0.382	1.00E-69	1.2
KIAA1217	9.55E-74	-0.538388985	0.697	0.858	2.30E-69	1.2
CD59	1.07E-73	0.611497254	0.939	0.844	2.59E-69	1.2
CXCL8	2.04E-73	1.774378938	0.501	0.317	4.91E-69	1.2
CAST	9.19E-72	0.599355778	0.663	0.51	2.22E-67	1.2
HIST1H2AC	6.37E-71	-0.745119815	0.154	0.365	1.54E-66	1.2
ELK3	1.11E-70	0.570744358	0.312	0.147	2.69E-66	1.2
SAA1	5.42E-70	-1.488416892	0.955	0.954	1.31E-65	1.2
CHODL	7.30E-67	-0.784217599	0.065	0.24	1.76E-62	1.2
S100A10	2.59E-66	0.730291709	0.45	0.269	6.24E-62	1.2
LRIG1	4.42E-66	-0.761018214	0.1	0.287	1.07E-61	1.2

AKR1C3	5.24E-66	-0.615262292	0.057	0.229	1.26E-61	1.2
TIAM1	1.55E-64	0.646728649	0.454	0.27	3.73E-60	1.2
TMSB4X	1.76E-64	0.53510384	0.941	0.874	4.23E-60	1.2
STX12	5.47E-64	0.659457116	0.574	0.414	1.32E-59	1.2
PADI2	1.77E-63	-0.607123551	0.052	0.216	4.26E-59	1.2
CYP1B1	4.86E-63	-0.618000873	0.016	0.156	1.17E-58	1.2
MAP2	1.95E-62	0.579912065	0.286	0.134	4.69E-58	1.2
LAMC2	4.72E-62	0.776544767	0.422	0.259	1.14E-57	1.2
HMGN3	5.73E-62	-0.512155404	0.045	0.203	1.38E-57	1.2
RNF145	7.84E-61	-0.737942859	0.411	0.606	1.89E-56	1.2
SLC26A2	1.14E-60	-0.713299684	0.042	0.194	2.75E-56	1.2
ZFP36L2	1.74E-60	-0.728787261	0.203	0.408	4.20E-56	1.2
RP11-114H	1.94E-60	-0.819129203	0.061	0.221	4.68E-56	1.2
ADAM32	4.20E-60	0.64067619	0.225	0.095	1.01E-55	1.2
FHIT	4.54E-60	-0.733767798	0.234	0.43	1.10E-55	1.2
ITPR2	1.09E-59	-0.614944169	0.389	0.588	2.62E-55	1.2
PLXDC2	1.25E-59	-0.579163627	0.101	0.278	3.00E-55	1.2
EZR	4.88E-59	0.55924245	0.445	0.273	1.18E-54	1.2
MEIS2	8.16E-59	-0.625090095	0.116	0.295	1.97E-54	1.2
SSBP2	1.08E-58	-0.73410393	0.194	0.388	2.60E-54	1.2
VNN3	3.64E-58	-0.867918081	0.134	0.31	8.77E-54	1.2
SAMD4A	4.59E-58	0.512136547	0.752	0.61	1.11E-53	1.2
RPL24	1.92E-57	0.339329988	0.963	0.951	4.63E-53	1.2
FGF13	2.04E-57	0.746248802	0.338	0.184	4.92E-53	1.2
ZPLD1	1.58E-56	0.831416245	0.226	0.099	3.82E-52	1.2
PBX1	2.17E-56	-0.590468008	0.092	0.261	5.23E-52	1.2
INADL	6.50E-56	0.421602193	0.876	0.778	1.57E-51	1.2
SMARCA2	6.95E-56	-0.607614329	0.162	0.341	1.68E-51	1.2
GHR	7.33E-56	-0.684218338	0.031	0.168	1.77E-51	1.2
SELK	1.08E-55	0.502205197	0.761	0.651	2.61E-51	1.2
RP5-1198O	1.88E-55	-0.632045358	0.045	0.19	4.54E-51	1.2
SEPP1	3.67E-55	-0.646300333	0.22	0.403	8.84E-51	1.2
YWHAH	4.75E-55	0.533704991	0.337	0.186	1.15E-50	1.2
TPM4	6.18E-55	0.565711332	0.615	0.464	1.49E-50	1.2
FXYD3	1.03E-53	-0.546307639	0.381	0.563	2.50E-49	1.2
CDH1	1.42E-53	0.503240665	0.636	0.483	3.42E-49	1.2
INSIG2	3.24E-53	0.487185854	0.358	0.202	7.80E-49	1.2
LDLRAD4	3.48E-53	-0.592432115	0.059	0.207	8.38E-49	1.2
BCL6	4.92E-53	-0.649175253	0.198	0.381	1.19E-48	1.2
GCNT2	5.62E-53	-0.605155404	0.219	0.405	1.36E-48	1.2
RARRES3	1.38E-52	-0.634921979	0.18	0.355	3.33E-48	1.2
PSTPIP2	4.70E-52	-0.624741395	0.215	0.397	1.13E-47	1.2
WWOX	1.57E-51	-0.797394272	0.101	0.258	3.79E-47	1.2
CDCP1	3.33E-51	0.565122653	0.496	0.343	8.03E-47	1.2
THSD4	3.71E-51	0.614047249	0.54	0.381	8.94E-47	1.2
GBE1	4.11E-51	0.714501779	0.519	0.364	9.90E-47	1.2
ST5	7.93E-51	-0.691372131	0.251	0.431	1.91E-46	1.2

KIT	1.49E-50	-0.512590931	0.218	0.409	3.59E-46	1.2
GALNT3	1.57E-50	0.546786019	0.325	0.181	3.79E-46	1.2
TRIO	1.79E-50	0.288491473	0.836	0.718	4.31E-46	1.2
ZNF521	2.02E-49	-0.555718664	0.071	0.216	4.87E-45	1.2
CHI3L1	3.08E-49	-1.10980202	0.085	0.234	7.42E-45	1.2
NEBL	4.60E-49	-0.566993738	0.359	0.542	1.11E-44	1.2
THADA	6.78E-49	-0.694548195	0.144	0.31	1.64E-44	1.2
ATXN1	8.58E-49	0.591522488	0.48	0.329	2.07E-44	1.2
FDPS	2.27E-48	-0.660849886	0.332	0.498	5.46E-44	1.2
MALAT1	6.78E-48	0.270988535	1	1	1.63E-43	1.2
ZNF518A	1.40E-47	-0.504968486	0.075	0.218	3.39E-43	1.2
ALDH1A3	1.64E-47	0.51783086	0.652	0.51	3.95E-43	1.2
BTG1	2.07E-47	-0.602048448	0.335	0.506	4.99E-43	1.2
FAM13A	3.72E-47	-0.759045272	0.244	0.415	8.97E-43	1.2
PPP1R9A	5.97E-47	-0.488572925	0.09	0.239	1.44E-42	1.2
NEAT1	1.01E-46	0.365305804	0.986	0.959	2.42E-42	1.2
ANKS1B	2.92E-46	-0.546046389	0.144	0.308	7.05E-42	1.2
C4orf3	9.44E-46	0.506013926	0.702	0.605	2.28E-41	1.2
SLC34A2	2.01E-45	-0.570593505	0.162	0.321	4.84E-41	1.2
PODXL	2.66E-45	-0.470745213	0.078	0.219	6.42E-41	1.2
PPP1CB	3.82E-45	-0.534302636	0.284	0.46	9.20E-41	1.2
USP54	4.56E-45	-0.576554547	0.372	0.554	1.10E-40	1.2
ESR1	1.22E-44	-0.469926243	0.04	0.163	2.94E-40	1.2
C10orf90	1.26E-44	-0.417210212	0.024	0.136	3.05E-40	1.2
FOSL1	2.65E-44	0.437084704	0.181	0.076	6.38E-40	1.2
PRNP	7.61E-44	0.454574691	0.224	0.109	1.83E-39	1.2
ARRDC3	8.77E-44	-0.481027336	0.525	0.685	2.12E-39	1.2
LDHA	1.02E-43	0.373594074	0.793	0.698	2.46E-39	1.2
GLUL	1.16E-43	-0.640799819	0.198	0.36	2.80E-39	1.2
MGAM2	1.38E-43	-0.633946369	0.208	0.375	3.32E-39	1.2
NOS1AP	2.11E-43	-0.551355545	0.06	0.188	5.08E-39	1.2
MAP4	3.13E-43	0.519213471	0.533	0.403	7.56E-39	1.2
ANO6	3.42E-43	0.517502861	0.464	0.322	8.25E-39	1.2
ANXA11	4.39E-43	0.511091261	0.353	0.215	1.06E-38	1.2
DHCR24	5.18E-43	-0.414816658	0.067	0.198	1.25E-38	1.2
ATP1B1	5.33E-43	0.450010977	0.783	0.691	1.28E-38	1.2
MICAL3	1.58E-42	0.541472735	0.415	0.27	3.82E-38	1.2
ANXA3	2.83E-42	0.536700652	0.522	0.383	6.83E-38	1.2
MET	3.83E-42	0.468820931	0.471	0.32	9.23E-38	1.2
DENND2C	5.03E-42	0.311999941	0.094	0.026	1.21E-37	1.2
PDK3	5.22E-42	-0.596899649	0.162	0.315	1.26E-37	1.2
FAAH2	9.52E-42	-0.484814469	0.102	0.242	2.30E-37	1.2
TXNIP	1.03E-41	-0.827065972	0.355	0.494	2.49E-37	1.2
ANXA5	1.07E-41	0.599915174	0.354	0.222	2.58E-37	1.2
LDHB	1.17E-41	-0.409537608	0.652	0.777	2.83E-37	1.2
RASA2	2.45E-41	0.333436514	0.729	0.604	5.91E-37	1.2
RPL36AL	2.48E-41	0.323812713	0.933	0.909	5.99E-37	1.2

FABP7	2.75E-41	-1.318413858	0.116	0.251	6.64E-37	1.2
ST8SIA1	3.06E-41	-0.453507927	0.078	0.211	7.38E-37	1.2
MAP4K4	4.43E-41	0.54406961	0.457	0.323	1.07E-36	1.2
ALPK1	4.72E-41	-0.546800831	0.182	0.333	1.14E-36	1.2
UTRN	6.55E-41	-0.484736379	0.104	0.247	1.58E-36	1.2
PDE7A	6.90E-41	-0.52922302	0.148	0.296	1.66E-36	1.2
TM2D2	6.96E-41	0.297477566	0.121	0.042	1.68E-36	1.2
MAP2K1	7.61E-41	0.488366982	0.311	0.183	1.84E-36	1.2
SEC61G	7.99E-41	0.443955948	0.764	0.684	1.93E-36	1.2
S100A14	1.07E-40	0.580857462	0.736	0.647	2.59E-36	1.2
MYO1D	1.20E-40	0.498819189	0.374	0.237	2.90E-36	1.2
SLC27A4	1.65E-40	0.561739597	0.667	0.535	3.97E-36	1.2
NR2F2-AS1	2.00E-40	-0.446259697	0.049	0.167	4.82E-36	1.2
CFLAR	2.67E-40	0.577897743	0.559	0.444	6.43E-36	1.2
TC2N	3.12E-40	-0.464766846	0.219	0.387	7.52E-36	1.2
SLC20A2	4.55E-40	0.531913269	0.493	0.356	1.10E-35	1.2
KRT6B	5.20E-40	0.757489267	0.201	0.097	1.25E-35	1.2
RP1-78O14	5.83E-40	0.501680708	0.132	0.05	1.41E-35	1.2
CYP7B1	6.51E-40	-0.646112983	0.248	0.411	1.57E-35	1.2
SAT1	7.20E-40	0.414345029	0.976	0.958	1.74E-35	1.2
SERPINB7	1.03E-39	-0.742710479	0.094	0.225	2.48E-35	1.2
RPLP0	1.22E-39	-0.449928759	0.883	0.908	2.94E-35	1.2
FIGN	2.13E-39	-0.408789522	0.044	0.158	5.13E-35	1.2
TMEM87A	2.76E-39	-0.441415573	0.236	0.398	6.66E-35	1.2
CDC42	5.98E-39	0.394499763	0.772	0.681	1.44E-34	1.2
MAML2	6.67E-39	-0.38268888	0.672	0.811	1.61E-34	1.2
SUB1	8.61E-39	0.41144116	0.795	0.742	2.08E-34	1.2
C6orf132	9.61E-39	0.415036556	0.256	0.139	2.32E-34	1.2
NALCN	1.36E-38	-0.409310333	0.06	0.18	3.29E-34	1.2
PTN	2.18E-38	0.638070894	0.335	0.202	5.25E-34	1.2
ARFGEF3	2.64E-38	0.42765975	0.331	0.2	6.36E-34	1.2
RP11-449D	2.78E-38	-0.420403654	0.016	0.108	6.70E-34	1.2
MT-CO3	5.36E-38	-0.240381559	0.996	0.993	1.29E-33	1.2
ARHGAP26	7.28E-38	-0.51218076	0.124	0.263	1.75E-33	1.2
AC016995.	7.93E-38	-0.474181517	0.096	0.226	1.91E-33	1.2
CNTN4	1.16E-37	-0.623416819	0.096	0.225	2.79E-33	1.2
MAP3K13	1.23E-37	0.454945288	0.872	0.817	2.97E-33	1.2
NAMPT	1.59E-37	-0.508373323	0.465	0.618	3.85E-33	1.2
SRPK1	1.61E-37	0.477665916	0.658	0.56	3.89E-33	1.2
DSG3	2.01E-37	0.691053381	0.133	0.051	4.85E-33	1.2
YWHAZ	2.09E-37	0.422537914	0.664	0.567	5.05E-33	1.2
CNN3	2.10E-37	0.3535146	0.57	0.432	5.05E-33	1.2
RPS3	9.64E-37	-0.567883406	0.914	0.931	2.32E-32	1.2
DCHS2	1.56E-36	-0.505703923	0.06	0.174	3.77E-32	1.2
CXCR4	2.59E-36	-0.721866628	0.188	0.33	6.25E-32	1.2
AGAP1	2.83E-36	-0.537878616	0.523	0.645	6.81E-32	1.2
FEM1B	3.46E-36	0.402829054	0.205	0.103	8.34E-32	1.2

S100A8	6.27E-36	-1.681492828	0.119	0.244	1.51E-31	1.2
TXNRD1	8.17E-36	0.431616856	0.507	0.377	1.97E-31	1.2
ZMYND8	2.01E-35	-0.440542518	0.151	0.291	4.84E-31	1.2
RASGRP1	2.17E-35	-0.418644226	0.054	0.163	5.24E-31	1.2
SLC39A8	2.52E-35	-0.461651568	0.062	0.174	6.07E-31	1.2
FDCSP	2.79E-35	-2.500981329	0.112	0.231	6.74E-31	1.2
CTD-2015G	3.16E-35	-0.368628272	0.085	0.208	7.62E-31	1.2
CLMN	3.83E-35	0.423910111	0.67	0.568	9.22E-31	1.2
MAP3K5	5.06E-35	-0.433910718	0.204	0.355	1.22E-30	1.2
RPS12	5.43E-35	-0.554529795	0.672	0.753	1.31E-30	1.2
CCL2	7.73E-35	-0.51293942	0.319	0.464	1.86E-30	1.2
AKR1C1	2.11E-34	-0.559097686	0.044	0.146	5.08E-30	1.2
CACNB2	2.40E-34	-0.417528396	0.058	0.167	5.79E-30	1.2
COG5	2.45E-34	0.476268473	0.543	0.435	5.91E-30	1.2
CHD2	2.84E-34	0.443424175	0.527	0.405	6.85E-30	1.2
SON	4.17E-34	0.485744199	0.755	0.671	1.00E-29	1.2
NCOA1	4.29E-34	-0.474770175	0.285	0.436	1.03E-29	1.2
SORBS1	4.54E-34	-0.392822987	0.23	0.376	1.10E-29	1.2
HIVEP2	6.89E-34	0.456703563	0.469	0.339	1.66E-29	1.2
CLLU1OS	1.57E-33	-0.299727539	0.022	0.11	3.78E-29	1.2
HRSP12	2.34E-33	-0.275259715	0.021	0.108	5.64E-29	1.2
RPS6KA5	2.59E-33	-0.409921539	0.111	0.237	6.24E-29	1.2
SERPINB4	3.02E-33	-1.168525682	0.027	0.116	7.29E-29	1.2
KIAA0922	5.38E-33	-0.723827589	0.163	0.289	1.30E-28	1.2
PHLDA1	6.84E-33	-0.364483418	0.081	0.198	1.65E-28	1.2
ZFAS1	7.21E-33	0.272716244	0.97	0.96	1.74E-28	1.2
SERPINB3	7.57E-33	-1.025264515	0.019	0.103	1.83E-28	1.2
TNFAIP2	7.86E-33	-0.721970378	0.191	0.328	1.89E-28	1.2
TMEM217	1.13E-32	0.310128908	0.129	0.053	2.73E-28	1.2
SAA2-SAA4	1.52E-32	-0.489763914	0.267	0.406	3.66E-28	1.2
RTN4	1.91E-32	0.353889514	0.698	0.607	4.62E-28	1.2
CAMTA1	2.17E-32	0.398027108	0.608	0.499	5.24E-28	1.2
NCALD	2.27E-32	-0.474004637	0.119	0.244	5.46E-28	1.2
BMPR1B	2.30E-32	0.478382561	0.074	0.021	5.54E-28	1.2
GNE	2.81E-32	-0.392191615	0.087	0.203	6.78E-28	1.2
RPL19	3.11E-32	-0.399620101	0.927	0.941	7.50E-28	1.2
MAMLD1	6.40E-32	0.330479173	0.111	0.043	1.54E-27	1.2
COL6A2	6.93E-32	0.514411872	0.219	0.12	1.67E-27	1.2
MLLT4	1.66E-31	0.222463523	0.755	0.636	4.01E-27	1.2
RORA	2.40E-31	-0.440529294	0.375	0.517	5.80E-27	1.2
PAPSS1	2.50E-31	-0.428436443	0.262	0.408	6.03E-27	1.2
RP11-142C	2.67E-31	-0.354494524	0.046	0.143	6.44E-27	1.2
MIR4435-2	3.13E-31	0.483296661	0.565	0.462	7.55E-27	1.2
HIST1H2BB	3.27E-31	-0.380996191	0.057	0.159	7.90E-27	1.2
MYO3B	3.29E-31	0.475239036	0.294	0.182	7.94E-27	1.2
NFIA	3.61E-31	-0.343851195	0.051	0.151	8.70E-27	1.2
TCF7L1	3.96E-31	-0.501566553	0.229	0.365	9.56E-27	1.2

HERC4	6.39E-31	0.466952495	0.483	0.368	1.54E-26	1.2
3-Mar	6.54E-31	-0.537471152	0.116	0.236	1.58E-26	1.2
SKP1	7.77E-31	0.350023423	0.863	0.831	1.87E-26	1.2
TES	8.05E-31	0.38187925	0.417	0.295	1.94E-26	1.2
GRIN2A	1.08E-30	0.425554626	0.109	0.042	2.61E-26	1.2
LRP2	1.09E-30	-0.473309853	0.136	0.259	2.63E-26	1.2
AUH	1.47E-30	-0.512449197	0.233	0.368	3.55E-26	1.2
CAP1	1.50E-30	0.388283323	0.315	0.204	3.63E-26	1.2
CLU	1.54E-30	-0.62776208	0.039	0.13	3.72E-26	1.2
FMN1	1.56E-30	-0.413303582	0.083	0.193	3.76E-26	1.2
SLC9C1	1.78E-30	0.211975731	0.066	0.018	4.29E-26	1.2
DSG2	2.03E-30	0.411968778	0.445	0.335	4.91E-26	1.2
MTM1	2.12E-30	0.440093685	0.277	0.173	5.12E-26	1.2
RPL10A	3.42E-30	-0.398624652	0.738	0.799	8.26E-26	1.2
MGST2	3.43E-30	-0.345499453	0.086	0.196	8.26E-26	1.2
GCLM	3.57E-30	0.513953613	0.231	0.135	8.61E-26	1.2
MPRIP	3.80E-30	0.419134044	0.314	0.204	9.17E-26	1.2
EEF1B2	4.28E-30	-0.356599139	0.853	0.892	1.03E-25	1.2
ST6GALNA(	5.58E-30	0.578949836	0.477	0.363	1.35E-25	1.2
UCK2	6.12E-30	-0.35876387	0.086	0.195	1.48E-25	1.2
SLC5A6	7.03E-30	-0.322678875	0.057	0.155	1.70E-25	1.2
SDCBP	7.13E-30	0.464594676	0.771	0.7	1.72E-25	1.2
CD55	8.00E-30	0.460086062	0.467	0.354	1.93E-25	1.2
PTRF	9.83E-30	0.386979193	0.257	0.154	2.37E-25	1.2
CD44	1.38E-29	0.477853536	0.562	0.458	3.33E-25	1.2
MYO6	1.53E-29	0.28020667	0.711	0.628	3.68E-25	1.2
CADPS2	6.42E-29	-0.403276594	0.205	0.34	1.55E-24	1.2
FOXN3	8.60E-29	-0.373782291	0.138	0.26	2.07E-24	1.2
EIF2AK3	1.50E-28	-0.695611588	0.209	0.325	3.63E-24	1.2
CLEC7A	1.67E-28	-0.335675057	0.055	0.149	4.02E-24	1.2
JUP	1.89E-28	0.443763154	0.301	0.197	4.56E-24	1.2
RALGPS2	2.04E-28	0.429375191	0.236	0.14	4.92E-24	1.2
ADAM9	3.08E-28	0.247624545	0.668	0.553	7.42E-24	1.2
FGF2	3.13E-28	0.396410301	0.15	0.072	7.54E-24	1.2
CRIM1	3.63E-28	0.332973604	0.596	0.477	8.76E-24	1.2
SERPINA3	4.60E-28	-0.485981028	0.126	0.244	1.11E-23	1.2
FAM168A	5.49E-28	-0.411983839	0.133	0.249	1.32E-23	1.2
S100A6	5.70E-28	0.709633279	0.546	0.443	1.37E-23	1.2
ATL2	5.85E-28	-0.375768275	0.184	0.312	1.41E-23	1.2
AMBRA1	7.56E-28	-0.29325773	0.648	0.755	1.82E-23	1.2
OCLN	1.17E-27	0.358931282	0.404	0.289	2.82E-23	1.2
SCP2	1.27E-27	-0.31003017	0.136	0.257	3.07E-23	1.2
RPL10	1.85E-27	-0.485763776	0.961	0.965	4.46E-23	1.2
DENND2D	1.96E-27	-0.292630858	0.061	0.155	4.72E-23	1.2
RGL1	2.04E-27	-0.279002066	0.03	0.11	4.91E-23	1.2
ITGB1	2.12E-27	0.378783386	0.448	0.331	5.10E-23	1.2
SEMA4B	2.14E-27	-0.358343888	0.085	0.187	5.16E-23	1.2

SCARA3	2.58E-27	-0.284563044	0.013	0.081	6.22E-23	1.2
PTGS2	2.65E-27	0.420038169	0.059	0.016	6.39E-23	1.2
SYNM	2.83E-27	-0.436755004	0.08	0.18	6.84E-23	1.2
FOXP1	3.43E-27	-0.433999191	0.454	0.578	8.26E-23	1.2
KRT16	3.47E-27	0.297001174	0.053	0.013	8.38E-23	1.2
TBC1D5	3.50E-27	-0.441619867	0.255	0.384	8.43E-23	1.2
ASAP1	3.78E-27	0.413587993	0.327	0.219	9.13E-23	1.2
DDX21	3.83E-27	0.384137742	0.679	0.611	9.23E-23	1.2
LRRFIP2	3.98E-27	0.162186234	0.856	0.802	9.59E-23	1.2
MAL2	4.43E-27	0.39077865	0.331	0.224	1.07E-22	1.2
ECHDC1	4.47E-27	-0.353986513	0.178	0.303	1.08E-22	1.2
SMS	4.84E-27	0.376988186	0.479	0.365	1.17E-22	1.2
ARL6IP5	5.99E-27	-0.300262242	0.373	0.521	1.45E-22	1.2
MT-CO2	6.41E-27	-0.235444952	0.987	0.985	1.55E-22	1.2
RPS23	6.43E-27	-0.218879697	0.99	0.982	1.55E-22	1.2
DPYD	7.20E-27	-0.455992127	0.431	0.56	1.74E-22	1.2
CTTNBP2NL	7.90E-27	0.370302048	0.366	0.26	1.91E-22	1.2
ZBTB16	8.06E-27	-0.415911202	0.181	0.305	1.94E-22	1.2
NUTM2A-A	8.56E-27	0.370495269	0.36	0.254	2.06E-22	1.2
HSD17B4	9.33E-27	-0.331194839	0.083	0.183	2.25E-22	1.2
SLC12A2	1.00E-26	-0.520460388	0.444	0.566	2.41E-22	1.2
TRABD2B	1.10E-26	-0.398081367	0.04	0.123	2.66E-22	1.2
PDLIM5	1.18E-26	0.313273739	0.864	0.831	2.86E-22	1.2
HSD17B7	1.28E-26	-0.325493352	0.048	0.135	3.08E-22	1.2
TPCN1	1.45E-26	-0.344443147	0.051	0.14	3.50E-22	1.2
EIF2B5	1.61E-26	-0.43123827	0.475	0.592	3.87E-22	1.2
KLF6	1.70E-26	0.363292627	0.601	0.505	4.11E-22	1.2
RCC1	1.77E-26	0.415111072	0.436	0.333	4.27E-22	1.2
EPB41L1	2.00E-26	-0.305922394	0.039	0.122	4.83E-22	1.2
AKT3	2.09E-26	0.387955637	0.562	0.452	5.04E-22	1.2
HMOX1	2.18E-26	0.462983313	0.113	0.049	5.25E-22	1.2
ZNHIT6	2.18E-26	-0.351800049	0.106	0.213	5.25E-22	1.2
FOXN2	2.87E-26	0.356113915	0.165	0.085	6.93E-22	1.2
RPS11	3.38E-26	-0.390807856	0.716	0.788	8.14E-22	1.2
MAML3	3.50E-26	-0.341154807	0.151	0.268	8.44E-22	1.2
GARS	3.51E-26	0.391142196	0.263	0.165	8.45E-22	1.2
FMO2	4.14E-26	-0.320753974	0.05	0.136	9.99E-22	1.2
SDC4	4.20E-26	0.558645587	0.508	0.412	1.01E-21	1.2
CCND3	5.79E-26	-0.405572712	0.257	0.386	1.40E-21	1.2
UFM1	6.28E-26	0.414721474	0.432	0.329	1.51E-21	1.2
WWC1	6.52E-26	-0.439125246	0.35	0.482	1.57E-21	1.2
ACSL1	7.92E-26	-0.347224501	0.143	0.258	1.91E-21	1.2
FBXO11	9.70E-26	0.425620482	0.467	0.363	2.34E-21	1.2
AHNAK	9.94E-26	0.376857022	0.369	0.265	2.40E-21	1.2
INSR	1.12E-25	0.444159568	0.7	0.637	2.71E-21	1.2
RPSA	1.23E-25	-0.315180217	0.9	0.927	2.96E-21	1.2
TMOD3	1.43E-25	0.413593651	0.367	0.264	3.44E-21	1.2

FTO	1.43E-25	-0.313730586	0.145	0.262	3.45E-21	1.2
ARID1B	1.67E-25	-0.322613461	0.301	0.438	4.03E-21	1.2
WTAP	1.77E-25	-0.472639809	0.57	0.69	4.26E-21	1.2
SORBS2	1.91E-25	-0.391796124	0.476	0.607	4.60E-21	1.2
LRP1B	2.34E-25	-0.671682996	0.122	0.228	5.64E-21	1.2
ITFG1	2.55E-25	-0.368010015	0.155	0.27	6.15E-21	1.2
IMMP2L	2.63E-25	-0.38838786	0.219	0.344	6.33E-21	1.2
COL27A1	2.95E-25	-0.355330345	0.038	0.116	7.12E-21	1.2
TNIP3	2.96E-25	-0.255446143	0.023	0.094	7.14E-21	1.2
IGF2BP2	4.21E-25	0.327827132	0.586	0.49	1.01E-20	1.2
FKBP5	4.51E-25	-0.445811966	0.19	0.308	1.09E-20	1.2
HOOK2	5.27E-25	-0.309163847	0.087	0.185	1.27E-20	1.2
C1QTNF3-A	7.61E-25	-0.362133106	0.132	0.241	1.83E-20	1.2
DOCK7	8.22E-25	-0.338951233	0.154	0.269	1.98E-20	1.2
RAD51B	1.05E-24	-0.352371248	0.138	0.249	2.53E-20	1.2
C1S	1.06E-24	-0.261446169	0.05	0.134	2.55E-20	1.2
CMIP	1.14E-24	0.374706979	0.441	0.337	2.76E-20	1.2
C3orf52	1.15E-24	0.266320264	0.119	0.054	2.78E-20	1.2
CFB	1.22E-24	-0.316688743	0.066	0.156	2.95E-20	1.2
TMBIM6	1.75E-24	0.287838045	0.837	0.789	4.23E-20	1.2
TPT1-AS1	1.95E-24	0.418521977	0.479	0.387	4.70E-20	1.2
PPM1L	2.00E-24	-0.274114586	0.021	0.089	4.83E-20	1.2
B2M	2.20E-24	0.211132081	0.988	0.985	5.31E-20	1.2
TMEM41A	2.50E-24	0.386910178	0.284	0.191	6.03E-20	1.2
AC005152.	2.54E-24	-0.38179486	0.093	0.19	6.12E-20	1.2
SORL1	3.05E-24	-0.229234935	0.021	0.088	7.37E-20	1.2
KPNB1	3.61E-24	0.371938381	0.515	0.427	8.71E-20	1.2
GPC5	4.49E-24	0.335157462	0.078	0.029	1.08E-19	1.2
IFNGR1	4.58E-24	-0.34843185	0.167	0.282	1.10E-19	1.2
CCL28	5.26E-24	0.53005668	0.735	0.699	1.27E-19	1.2
OSBPL1A	5.57E-24	-0.302249819	0.078	0.169	1.34E-19	1.2
FEZ2	5.87E-24	0.424653823	0.462	0.364	1.42E-19	1.2
ALDH9A1	5.93E-24	-0.239508127	0.03	0.101	1.43E-19	1.2
PDCD4	6.02E-24	-0.348897732	0.14	0.248	1.45E-19	1.2
CDK14	6.08E-24	0.393175327	0.481	0.381	1.47E-19	1.2
RPS20	6.12E-24	-0.335343862	0.955	0.957	1.47E-19	1.2
SASH1	6.50E-24	-0.315344294	0.217	0.345	1.57E-19	1.2
CRY1	7.74E-24	-0.499310974	0.256	0.369	1.87E-19	1.2
PLCB1	1.18E-23	0.542640044	0.474	0.377	2.84E-19	1.2
LINC01184	1.33E-23	-0.309128337	0.134	0.243	3.20E-19	1.2
RASSF3	1.46E-23	0.326015102	0.218	0.13	3.51E-19	1.2
SLPI	1.57E-23	0.4227497	0.864	0.8	3.79E-19	1.2
PLSCR2	1.74E-23	0.255731918	0.086	0.034	4.21E-19	1.2
LYN	1.75E-23	-0.38238229	0.62	0.708	4.22E-19	1.2
PITPNB	1.95E-23	0.374628053	0.392	0.294	4.70E-19	1.2
H2AFZ	2.04E-23	0.484430205	0.657	0.603	4.93E-19	1.2
EIF3K	2.16E-23	-0.369629227	0.378	0.501	5.22E-19	1.2

LAMB3	2.18E-23	0.820007993	0.353	0.271	5.24E-19	1.2
KRT17	2.49E-23	0.31248492	0.063	0.02	6.01E-19	1.2
PLA2R1	2.88E-23	-0.333735856	0.152	0.263	6.95E-19	1.2
RPL32	3.00E-23	-0.205120024	0.991	0.983	7.24E-19	1.2
PARK2	3.52E-23	-0.340662055	0.092	0.187	8.50E-19	1.2
ADAMTS9-/	3.57E-23	0.449712433	0.398	0.298	8.61E-19	1.2
PRKCE	4.05E-23	-0.395629194	0.125	0.228	9.78E-19	1.2
DYRK1A	4.10E-23	-0.397411857	0.264	0.385	9.90E-19	1.2
PRDX3	4.79E-23	-0.273491564	0.08	0.171	1.15E-18	1.2
C10orf10	5.35E-23	-0.291815942	0.115	0.219	1.29E-18	1.2
PARD3B	6.56E-23	-0.305504322	0.066	0.15	1.58E-18	1.2
MCTS1	7.53E-23	-0.284347561	0.197	0.316	1.82E-18	1.2
ASS1	8.24E-23	-0.300953929	0.087	0.18	1.99E-18	1.2
BBOX1	8.43E-23	-0.438289554	0.179	0.289	2.03E-18	1.2
NPAS3	1.14E-22	-0.362604247	0.091	0.182	2.74E-18	1.2
COA1	1.54E-22	-0.27313683	0.182	0.299	3.70E-18	1.2
HIST2H2BE	1.59E-22	-0.261215098	0.037	0.11	3.83E-18	1.2
ARHGAP44	1.69E-22	-0.328231667	0.158	0.267	4.07E-18	1.2
ABCA5	2.11E-22	-0.249939223	0.056	0.138	5.09E-18	1.2
BRE	2.23E-22	-0.275046197	0.099	0.194	5.39E-18	1.2
ZBTB38	2.28E-22	0.360747044	0.396	0.299	5.49E-18	1.2
IRF2BPL	2.51E-22	-0.237118405	0.063	0.147	6.06E-18	1.2
ECHDC2	3.19E-22	-0.264721774	0.054	0.134	7.70E-18	1.2
BMPR1A	3.37E-22	-0.325777513	0.195	0.31	8.11E-18	1.2
CBLC	3.56E-22	0.262739624	0.119	0.057	8.59E-18	1.2
ARFGEF2	3.61E-22	-0.314396099	0.237	0.359	8.70E-18	1.2
SPX	3.84E-22	-0.188296562	0.006	0.057	9.26E-18	1.2
CGNL1	3.91E-22	-0.267454797	0.067	0.152	9.44E-18	1.2
HSD17B2	4.00E-22	-0.202841506	0.019	0.08	9.63E-18	1.2
NNMT	4.01E-22	-0.408031998	0.031	0.098	9.68E-18	1.2
CTSV	4.47E-22	0.443865583	0.294	0.199	1.08E-17	1.2
WNT2B	6.37E-22	0.287362834	0.1	0.045	1.54E-17	1.2
CCDC25	7.57E-22	-0.224597661	0.053	0.131	1.82E-17	1.2
JPX	9.68E-22	-0.272087098	0.171	0.284	2.33E-17	1.2
INTS10	1.02E-21	-0.247845878	0.052	0.129	2.47E-17	1.2
RP11-266O	1.29E-21	-0.310833742	0.009	0.062	3.11E-17	1.2
FBN1	1.40E-21	-0.228471509	0.009	0.061	3.36E-17	1.2
MRPS21	1.46E-21	-0.292388023	0.422	0.548	3.51E-17	1.2
LINC01198	1.71E-21	-0.351698587	0.225	0.34	4.13E-17	1.2
ENOSF1	1.77E-21	-0.311004699	0.141	0.244	4.28E-17	1.2
VWA8	1.81E-21	-0.236641753	0.02	0.08	4.37E-17	1.2
AKR1C2	2.29E-21	-0.266434781	0.027	0.091	5.52E-17	1.2
KNOP1	2.38E-21	-0.240954094	0.093	0.185	5.75E-17	1.2
TCEB1	2.42E-21	0.434573429	0.366	0.276	5.84E-17	1.2
CDKAL1	2.65E-21	-0.337949199	0.164	0.269	6.40E-17	1.2
HINT1	2.75E-21	-0.306695325	0.594	0.686	6.64E-17	1.2
GTF2IRD1	3.14E-21	0.328295627	0.21	0.129	7.57E-17	1.2

NTRK2	3.21E-21	-0.287659023	0.112	0.209	7.73E-17	1.2
LSM5	3.23E-21	-0.289517566	0.339	0.464	7.79E-17	1.2
GAB1	3.65E-21	-0.450715729	0.274	0.387	8.79E-17	1.2
LPP	4.19E-21	0.255073425	0.904	0.885	1.01E-16	1.2
RGS2	5.14E-21	-0.70671513	0.137	0.232	1.24E-16	1.2
ROPN1B	5.82E-21	-0.309769039	0.07	0.15	1.40E-16	1.2
HELB	6.30E-21	-0.274676004	0.061	0.139	1.52E-16	1.2
TMTC2	6.37E-21	-0.334941336	0.221	0.336	1.54E-16	1.2
STAC2	7.53E-21	-0.241306598	0.009	0.06	1.82E-16	1.2
COMMD10	8.35E-21	-0.273906315	0.132	0.232	2.01E-16	1.2
FMNL2	9.91E-21	0.376087344	0.508	0.423	2.39E-16	1.2
RHOA	1.12E-20	0.361176496	0.484	0.403	2.70E-16	1.2
RP11-739G	1.32E-20	-0.313937144	0.005	0.051	3.19E-16	1.2
RIN2	1.33E-20	-0.331970909	0.225	0.336	3.20E-16	1.2
METAP2	1.36E-20	-0.266355741	0.263	0.383	3.29E-16	1.2
TANC2	1.54E-20	0.379518665	0.364	0.27	3.70E-16	1.2
PIK3C2G	1.59E-20	-0.322148305	0.049	0.121	3.84E-16	1.2
CCDC14	1.82E-20	-0.255106899	0.077	0.16	4.38E-16	1.2
SPIDR	2.34E-20	-0.32865584	0.502	0.617	5.64E-16	1.2
BLZF1	2.41E-20	0.289639701	0.142	0.077	5.80E-16	1.2
UBA52	2.68E-20	-0.187347246	0.952	0.956	6.45E-16	1.2
UBB	2.81E-20	-0.240972153	0.7	0.797	6.78E-16	1.2
OAT	2.84E-20	-0.307950293	0.285	0.398	6.85E-16	1.2
IDH2	2.84E-20	-0.22283648	0.036	0.102	6.86E-16	1.2
CD46	3.00E-20	-0.326346688	0.332	0.455	7.25E-16	1.2
CDKN1A	3.03E-20	0.164411145	0.058	0.02	7.31E-16	1.2
FAM107B	3.07E-20	0.373020875	0.314	0.225	7.40E-16	1.2
DNAJC6	3.58E-20	0.320831737	0.153	0.086	8.63E-16	1.2
BTF3	3.83E-20	-0.265013884	0.943	0.947	9.24E-16	1.2
SMYD3	4.00E-20	-0.379390263	0.155	0.252	9.65E-16	1.2
KRT15	4.11E-20	0.242071792	0.428	0.317	9.92E-16	1.2
IFITM3	4.38E-20	-0.346671373	0.176	0.277	1.06E-15	1.2
PDZK1IP1	4.44E-20	-0.305702265	0.101	0.192	1.07E-15	1.2
SAMD12	5.63E-20	-0.317095378	0.181	0.286	1.36E-15	1.2
RPL6	5.70E-20	-0.197622009	0.976	0.966	1.37E-15	1.2
PDZRN3	6.30E-20	-0.313125772	0.115	0.206	1.52E-15	1.2
TNFRSF11E	6.40E-20	0.347200771	0.159	0.09	1.54E-15	1.2
DARS	6.45E-20	-0.273255057	0.241	0.356	1.56E-15	1.2
GLS	6.46E-20	0.435749452	0.325	0.239	1.56E-15	1.2
LAP3	6.74E-20	-0.279204997	0.107	0.198	1.63E-15	1.2
ZNF33A	6.74E-20	-0.254089124	0.168	0.272	1.63E-15	1.2
DIAPH1	6.83E-20	0.338851172	0.28	0.194	1.65E-15	1.2
HES1	7.31E-20	-0.335836272	0.168	0.271	1.76E-15	1.2
LNX1	7.55E-20	-0.240102924	0.041	0.109	1.82E-15	1.2
PRKAG2	7.88E-20	-0.313640074	0.098	0.185	1.90E-15	1.2
POLR2G	8.33E-20	-0.238894323	0.103	0.193	2.01E-15	1.2
RP11-314N	8.36E-20	0.373244165	0.208	0.129	2.02E-15	1.2

TUBB6	8.60E-20	0.206208965	0.095	0.043	2.07E-15	1.2
PTGR1	9.30E-20	-0.238176002	0.176	0.283	2.24E-15	1.2
KCTD3	9.47E-20	-0.305078203	0.127	0.221	2.28E-15	1.2
C3	1.03E-19	-0.351171346	0.219	0.328	2.49E-15	1.2
RNF150	1.25E-19	-0.326331948	0.074	0.154	3.02E-15	1.2
RELB	1.39E-19	-0.246417742	0.085	0.169	3.35E-15	1.2
RBFOX2	1.94E-19	0.257473541	0.969	0.962	4.68E-15	1.2
CAB39L	2.33E-19	-0.283415489	0.056	0.129	5.61E-15	1.2
MARCO	2.85E-19	-0.245400426	0.046	0.114	6.88E-15	1.2
CSGALNAC	3.08E-19	-0.23862514	0.039	0.104	7.44E-15	1.2
NOCT	3.38E-19	-0.3288855	0.078	0.156	8.16E-15	1.2
CA2	3.39E-19	0.289862797	0.112	0.056	8.19E-15	1.2
ZNF608	3.55E-19	-0.294260967	0.099	0.184	8.55E-15	1.2
GABRP	3.69E-19	0.430944571	0.594	0.537	8.91E-15	1.2
EIF3L	3.77E-19	-0.248388375	0.7	0.779	9.10E-15	1.2
LINC-PINT	3.84E-19	-0.403430867	0.461	0.569	9.26E-15	1.2
RPS4X	3.90E-19	-0.160184176	0.992	0.981	9.40E-15	1.2
UQCRC2	3.92E-19	-0.286283225	0.36	0.473	9.45E-15	1.2
SLC24A3	4.02E-19	-0.267724503	0.095	0.181	9.68E-15	1.2
HIST1H2BD	4.29E-19	-0.378434812	0.202	0.304	1.03E-14	1.2
C4orf19	4.61E-19	-0.311889819	0.118	0.208	1.11E-14	1.2
DYNLT3	5.81E-19	0.352438122	0.28	0.2	1.40E-14	1.2
ZFAND3	6.93E-19	0.306387265	0.68	0.626	1.67E-14	1.2
ADK	7.24E-19	0.354616746	0.517	0.439	1.75E-14	1.2
CASC15	7.35E-19	0.470700267	0.421	0.334	1.77E-14	1.2
ADGRL2	8.57E-19	-0.30042136	0.067	0.141	2.07E-14	1.2
F11R	8.66E-19	0.294350637	0.238	0.158	2.09E-14	1.2
MIR646HG	8.96E-19	-0.257820601	0.044	0.11	2.16E-14	1.2
ADGRF1	9.40E-19	-0.172287092	0.019	0.073	2.27E-14	1.2
GPHN	9.81E-19	-0.25042617	0.208	0.317	2.36E-14	1.2
HNMT	9.89E-19	-0.278195185	0.152	0.248	2.38E-14	1.2
CACHD1	1.06E-18	-0.238020341	0.04	0.105	2.55E-14	1.2
CAV1	1.17E-18	0.284814638	0.101	0.049	2.83E-14	1.2
R3HDM2	1.24E-18	-0.28484848	0.089	0.171	2.98E-14	1.2
ANGPTL1	1.34E-18	0.289783072	0.119	0.063	3.22E-14	1.2
RAP2B	1.42E-18	0.270548707	0.255	0.172	3.41E-14	1.2
RP11-244M	1.42E-18	-0.301077504	0.473	0.574	3.42E-14	1.2
SYNE2	1.43E-18	-0.261734957	0.415	0.536	3.45E-14	1.2
CLCN3	1.55E-18	-0.262028814	0.119	0.207	3.74E-14	1.2
SOX10	1.71E-18	-0.282506544	0.07	0.144	4.11E-14	1.2
PLS3	1.78E-18	0.274200749	0.164	0.096	4.29E-14	1.2
TM4SF1	1.83E-18	0.242369625	0.917	0.892	4.41E-14	1.2
TNFAIP3	1.87E-18	0.337283464	0.322	0.234	4.50E-14	1.2
ENTPD1-AS	1.93E-18	-0.212747536	0.026	0.083	4.64E-14	1.2
HIBCH	1.94E-18	-0.232201953	0.084	0.164	4.69E-14	1.2
RAB8B	2.01E-18	0.31543428	0.225	0.148	4.84E-14	1.2
MYRFL	2.02E-18	0.300664357	0.117	0.061	4.88E-14	1.2

KRT14	2.10E-18	0.133876008	0.03	0.006	5.05E-14	1.2
TMCO1	2.17E-18	-0.241132048	0.246	0.353	5.23E-14	1.2
RP11-519G	2.23E-18	-0.302939384	0.043	0.107	5.37E-14	1.2
ATP5F1	2.38E-18	-0.280094717	0.378	0.493	5.73E-14	1.2
LIMCH1	2.50E-18	-0.247048898	0.186	0.291	6.04E-14	1.2
NKX3-1	2.77E-18	0.277746191	0.137	0.075	6.69E-14	1.2
EBP	2.97E-18	-0.210920503	0.051	0.12	7.15E-14	1.2
NR2F2	3.14E-18	-0.209138591	0.043	0.108	7.58E-14	1.2
ARPC3	3.51E-18	-0.226977662	0.581	0.692	8.46E-14	1.2
MAP3K1	3.79E-18	-0.290097938	0.14	0.232	9.15E-14	1.2
SGCZ	4.74E-18	-0.312722116	0.038	0.099	1.14E-13	1.2
TEX14	4.97E-18	-0.429746389	0.181	0.274	1.20E-13	1.2
CCDC50	5.48E-18	0.288340169	0.215	0.139	1.32E-13	1.2
AC159540.	5.69E-18	0.404949749	0.233	0.158	1.37E-13	1.2
MRPL40	6.06E-18	-0.197614141	0.051	0.117	1.46E-13	1.2
CD2AP	6.87E-18	0.25385777	0.455	0.364	1.66E-13	1.2
AKAP6	6.94E-18	-0.184739512	0.014	0.063	1.67E-13	1.2
PTPN2	8.12E-18	-0.27318219	0.288	0.397	1.96E-13	1.2
FANCL	8.35E-18	-0.174164842	0.034	0.094	2.01E-13	1.2
KIAA1324	8.49E-18	-0.227906435	0.055	0.123	2.05E-13	1.2
RP11-141O	8.67E-18	-0.168737277	0.005	0.045	2.09E-13	1.2
TACC2	8.79E-18	-0.233066678	0.088	0.166	2.12E-13	1.2
ERC1	9.69E-18	0.339116139	0.361	0.278	2.34E-13	1.2
ATF7IP	1.06E-17	-0.290144986	0.108	0.193	2.56E-13	1.2
ARID5B	1.09E-17	-0.263572257	0.672	0.766	2.63E-13	1.2
NFE2L3	1.13E-17	0.3806515	0.139	0.079	2.72E-13	1.2
SMARCD3	1.16E-17	-0.1584747	0.005	0.045	2.80E-13	1.2
AL109761.!	1.21E-17	-0.147983159	0.009	0.052	2.92E-13	1.2
PRDM2	1.22E-17	-0.236020597	0.229	0.337	2.93E-13	1.2
RPL7A	1.25E-17	-0.192567983	0.985	0.973	3.02E-13	1.2
GMDS-AS1	1.27E-17	-0.315806938	0.063	0.132	3.05E-13	1.2
EDN1	1.30E-17	0.309878238	0.277	0.194	3.12E-13	1.2
ANKRD36B	1.32E-17	0.311391844	0.17	0.103	3.18E-13	1.2
MGP	1.33E-17	0.130633485	0.896	0.861	3.20E-13	1.2
SERPINB5	1.35E-17	0.174959861	0.052	0.018	3.26E-13	1.2
LINC01122	1.38E-17	-0.251248063	0.024	0.078	3.32E-13	1.2
DANT2	1.66E-17	-0.201364134	0.037	0.097	4.01E-13	1.2
TUBA1C	1.67E-17	0.356245971	0.416	0.342	4.02E-13	1.2
MBP	1.68E-17	0.3345353	0.234	0.159	4.04E-13	1.2
IQGAP2	1.96E-17	-0.205478505	0.031	0.088	4.72E-13	1.2
CADM1	1.97E-17	-0.240976885	0.039	0.1	4.75E-13	1.2
TMEM135	2.02E-17	-0.195399433	0.032	0.09	4.87E-13	1.2
RPL4	2.05E-17	-0.211505546	0.951	0.959	4.95E-13	1.2
LAMC1	2.08E-17	0.321793897	0.33	0.248	5.02E-13	1.2
TBC1D4	2.27E-17	-0.22382396	0.082	0.159	5.47E-13	1.2
GLYATL2	2.28E-17	-0.157425018	0.03	0.087	5.50E-13	1.2
PI3	2.31E-17	0.966924933	0.358	0.278	5.58E-13	1.2

MAGED1	2.50E-17	-0.228052458	0.056	0.124	6.03E-13	1.2
PSD3	2.64E-17	0.394286424	0.266	0.19	6.37E-13	1.2
UBE2E3	2.65E-17	-0.246516758	0.11	0.194	6.39E-13	1.2
DECR1	2.85E-17	-0.228138692	0.085	0.162	6.86E-13	1.2
RP11-96H1	3.07E-17	-0.279004899	0.06	0.128	7.41E-13	1.2
CYP1B1-AS	3.14E-17	-0.152326166	0.007	0.048	7.57E-13	1.2
ANAPC16	3.28E-17	-0.226560215	0.208	0.309	7.90E-13	1.2
RPL7	3.54E-17	-0.385859402	0.958	0.96	8.53E-13	1.2
NMT1	4.39E-17	-0.25995515	0.125	0.21	1.06E-12	1.2
C1orf21	4.46E-17	0.30169524	0.289	0.206	1.07E-12	1.2
FNBP1L	4.51E-17	-0.399535073	0.357	0.456	1.09E-12	1.2
PRLR	4.55E-17	-0.226797107	0.075	0.149	1.10E-12	1.2
GALNT15	5.40E-17	-0.336277375	0.085	0.16	1.30E-12	1.2
LINC01235	5.45E-17	-0.230261839	0.128	0.216	1.32E-12	1.2
SH3RF2	5.47E-17	0.169398101	0.047	0.016	1.32E-12	1.2
HCAR2	5.60E-17	0.440085248	0.261	0.184	1.35E-12	1.2
TMCO4	5.62E-17	-0.187643502	0.048	0.112	1.36E-12	1.2
VMP1	5.91E-17	0.270192678	0.801	0.773	1.42E-12	1.2
GRHL1	6.12E-17	-0.26970121	0.179	0.276	1.48E-12	1.2
TM4SF18	6.38E-17	-0.257606216	0.041	0.1	1.54E-12	1.2
MRPS33	6.49E-17	-0.261063762	0.23	0.33	1.57E-12	1.2
MTHFD2	6.68E-17	0.2354505	0.132	0.074	1.61E-12	1.2
SPOCD1	7.09E-17	0.111074909	0.036	0.01	1.71E-12	1.2
SUCLG2	7.35E-17	-0.2101504	0.049	0.112	1.77E-12	1.2
IDI1	7.63E-17	-0.26582514	0.147	0.235	1.84E-12	1.2
BAIAP2L1	8.16E-17	-0.389958931	0.971	0.966	1.97E-12	1.2
JAK2	8.22E-17	-0.312282979	0.038	0.096	1.98E-12	1.2
GPRC5B	8.23E-17	-0.187843535	0.025	0.077	1.99E-12	1.2
AGO2	8.30E-17	0.291749647	0.238	0.162	2.00E-12	1.2
LIMK2	8.56E-17	-0.218322428	0.073	0.144	2.06E-12	1.2
USMG5	9.47E-17	0.288757848	0.642	0.572	2.28E-12	1.2
MT-CYB	1.14E-16	-0.137844461	0.992	0.988	2.75E-12	1.2
TPI1	1.66E-16	0.357981965	0.433	0.355	4.00E-12	1.2
GXYLT2	1.71E-16	-0.217247802	0.041	0.1	4.12E-12	1.2
IRF6	1.79E-16	0.350745857	0.281	0.204	4.32E-12	1.2
KRT80	1.94E-16	0.168374588	0.074	0.032	4.67E-12	1.2
NFIL3	1.94E-16	-0.284453414	0.097	0.172	4.68E-12	1.2
TPST1	2.11E-16	-0.312176836	0.089	0.162	5.09E-12	1.2
IFRD1	2.24E-16	-0.352487788	0.304	0.4	5.41E-12	1.2
NUDT5	2.32E-16	-0.20456931	0.118	0.202	5.59E-12	1.2
ARF4	2.37E-16	0.2750106	0.694	0.641	5.71E-12	1.2
SCGB2A2	2.52E-16	0.776573845	0.249	0.173	6.08E-12	1.2
WDPCP	2.60E-16	-0.250449578	0.091	0.167	6.26E-12	1.2
NMB	2.61E-16	0.150046766	0.055	0.021	6.28E-12	1.2
CPEB3	2.64E-16	-0.33891013	0.257	0.356	6.37E-12	1.2
PLD1	2.77E-16	-0.196534874	0.049	0.112	6.67E-12	1.2
TLK1	2.82E-16	-0.231523706	0.139	0.226	6.80E-12	1.2

NSMCE1	2.98E-16	-0.181921405	0.063	0.13	7.18E-12	1.2
LBP	3.26E-16	-0.222772286	0.005	0.043	7.86E-12	1.2
ATP5G2	3.67E-16	-0.276993407	0.733	0.8	8.85E-12	1.2
KCCAT211	3.80E-16	-0.239304126	0.018	0.065	9.17E-12	1.2
LINC00998	3.82E-16	-0.189591305	0.105	0.187	9.21E-12	1.2
HIBADH	3.82E-16	-0.256755639	0.128	0.211	9.22E-12	1.2
CCDC53	4.47E-16	-0.168064502	0.04	0.098	1.08E-11	1.2
GNB1	4.53E-16	0.286998291	0.35	0.272	1.09E-11	1.2
PNPLA8	6.12E-16	0.288201516	0.355	0.271	1.48E-11	1.2
QKI	6.23E-16	-0.358972746	0.462	0.557	1.50E-11	1.2
DSP	6.56E-16	0.343446983	0.399	0.327	1.58E-11	1.2
SLC38A1	6.72E-16	0.299244366	0.349	0.268	1.62E-11	1.2
EXOC4	6.73E-16	-0.277494924	0.217	0.316	1.62E-11	1.2
MINA	6.93E-16	-0.169208516	0.034	0.088	1.67E-11	1.2
THRB	7.02E-16	-0.238423011	0.221	0.318	1.69E-11	1.2
ZMYM4	7.11E-16	-0.218485909	0.876	0.904	1.71E-11	1.2
ZNF429	7.22E-16	-0.183552143	0.037	0.094	1.74E-11	1.2
IDS	7.23E-16	0.145063399	0.077	0.035	1.74E-11	1.2
VAPA	7.31E-16	-0.260012599	0.394	0.505	1.76E-11	1.2
VAV3	7.37E-16	-0.271318433	0.09	0.164	1.78E-11	1.2
ATP13A5	7.51E-16	-0.182813105	0.013	0.056	1.81E-11	1.2
PCCA	7.96E-16	-0.261379784	0.1	0.177	1.92E-11	1.2
C15orf48	8.03E-16	-0.747762585	0.431	0.503	1.94E-11	1.2
GANC	8.24E-16	-0.185548094	0.055	0.118	1.99E-11	1.2
ARNTL2	8.32E-16	0.269218256	0.189	0.121	2.01E-11	1.2
KREMEN1	8.96E-16	-0.190807158	0.032	0.086	2.16E-11	1.2
RPL11	9.60E-16	-0.164951599	0.987	0.979	2.32E-11	1.2
KIAA0319L	9.90E-16	-0.255735269	0.104	0.181	2.39E-11	1.2
ESD	9.96E-16	-0.268017429	0.374	0.479	2.40E-11	1.2
CLDN8	1.04E-15	-0.201557344	0.042	0.1	2.50E-11	1.2
PAM	1.11E-15	0.270292909	0.626	0.572	2.67E-11	1.2
OGFRL1	1.13E-15	-0.223331672	0.173	0.264	2.72E-11	1.2
SDHC	1.15E-15	-0.184873594	0.091	0.166	2.77E-11	1.2
KRT81	1.15E-15	0.322474748	0.062	0.026	2.78E-11	1.2
BICD1	1.28E-15	-0.305176548	0.131	0.213	3.09E-11	1.2
OCIAD1	1.31E-15	-0.22739874	0.202	0.298	3.16E-11	1.2
NDUFA4	1.32E-15	-0.209467609	0.748	0.816	3.19E-11	1.2
TRAF1	1.44E-15	0.27505257	0.105	0.056	3.48E-11	1.2
LINC01344	1.48E-15	-0.177395921	0.011	0.051	3.56E-11	1.2
ARL4A	1.49E-15	-0.227491045	0.079	0.149	3.58E-11	1.2
LINC00969	1.53E-15	0.304201985	0.33	0.254	3.68E-11	1.2
FAM20C	1.63E-15	-0.263026614	0.053	0.115	3.92E-11	1.2
PLPP3	1.74E-15	-0.348640077	0.161	0.245	4.19E-11	1.2
MAST4	1.85E-15	-0.395094338	0.311	0.404	4.47E-11	1.2
TPM1	1.97E-15	0.275088206	0.646	0.594	4.75E-11	1.2
H1F0	1.98E-15	-0.224791069	0.111	0.189	4.77E-11	1.2
NDUFA5	2.26E-15	-0.241518421	0.222	0.321	5.46E-11	1.2

C1RL	2.30E-15	-0.238553342	0.07	0.136	5.56E-11	1.2
NFIX	2.37E-15	-0.262652336	0.068	0.133	5.71E-11	1.2
C21orf91-C	2.39E-15	-0.149393409	0.009	0.048	5.77E-11	1.2
NBR1	2.56E-15	-0.161268311	0.109	0.189	6.18E-11	1.2
EPPK1	2.56E-15	0.224128198	0.098	0.051	6.18E-11	1.2
GSTA4	2.64E-15	-0.161724144	0.029	0.08	6.36E-11	1.2
SUMF1	2.65E-15	-0.223966646	0.065	0.13	6.40E-11	1.2
RAP1B	2.73E-15	0.332016203	0.467	0.398	6.57E-11	1.2
CXCL17	2.76E-15	-0.444862283	0.125	0.201	6.65E-11	1.2
ABTB2	2.76E-15	0.336625359	0.301	0.223	6.66E-11	1.2
LINGO1	3.25E-15	0.273994274	0.909	0.877	7.83E-11	1.2
COX4I1	3.50E-15	-0.245964568	0.733	0.798	8.44E-11	1.2
TNRC6B	3.78E-15	-0.26691378	0.334	0.442	9.11E-11	1.2
MYADM	3.82E-15	0.153815318	0.077	0.036	9.21E-11	1.2
BOC	3.91E-15	-0.149704959	0.009	0.048	9.43E-11	1.2
KLK5	4.21E-15	0.190036674	0.109	0.058	1.01E-10	1.2
WDR1	4.23E-15	0.260656298	0.193	0.128	1.02E-10	1.2
IQCK	4.30E-15	-0.148199402	0.019	0.064	1.04E-10	1.2
ZFAND6	4.58E-15	0.318643192	0.425	0.351	1.10E-10	1.2
PLAC9	4.59E-15	0.13626628	0.044	0.015	1.11E-10	1.2
LURAP1L	5.31E-15	0.314073869	0.279	0.206	1.28E-10	1.2
SVIL	5.41E-15	0.218045826	0.86	0.827	1.30E-10	1.2
TIMM23B	5.64E-15	-0.231855605	0.141	0.224	1.36E-10	1.2
CYCS	5.75E-15	0.334669806	0.586	0.532	1.39E-10	1.2
CCAR1	5.83E-15	-0.202535008	0.183	0.276	1.41E-10	1.2
SAMD5	5.90E-15	-0.172765956	0.024	0.071	1.42E-10	1.2
C4BPB	6.16E-15	-0.147048843	0.014	0.055	1.49E-10	1.2
ENPP6	6.85E-15	-0.153582195	0.017	0.061	1.65E-10	1.2
SLC7A1	7.06E-15	0.22553998	0.142	0.086	1.70E-10	1.2
LINC01183	7.21E-15	-0.169586428	0.023	0.07	1.74E-10	1.2
SLCO1A2	7.49E-15	-0.135346052	0.003	0.036	1.81E-10	1.2
NUDCD1	7.99E-15	0.24800889	0.168	0.106	1.93E-10	1.2
ACTG1	9.03E-15	0.441322522	0.731	0.688	2.18E-10	1.2
CCNYL1	9.19E-15	0.192350079	0.113	0.063	2.22E-10	1.2
SUN1	9.71E-15	0.234639408	0.155	0.096	2.34E-10	1.2
ABHD17C	9.72E-15	-0.17182646	0.027	0.075	2.34E-10	1.2
COPZ1	9.84E-15	-0.21227134	0.271	0.372	2.37E-10	1.2
FRMD6	9.97E-15	-0.314743474	0.135	0.213	2.40E-10	1.2
UBTD2	1.03E-14	0.269270694	0.183	0.12	2.49E-10	1.2
RP11-486O	1.12E-14	0.22549621	0.089	0.045	2.71E-10	1.2
LOX	1.13E-14	0.245278967	0.092	0.048	2.72E-10	1.2
SESN2	1.19E-14	0.247246802	0.151	0.093	2.86E-10	1.2
APEX1	1.20E-14	-0.246787612	0.19	0.279	2.90E-10	1.2
CTC-340D7	1.25E-14	-0.123808753	0.003	0.036	3.01E-10	1.2
OGT	1.26E-14	0.271540006	0.427	0.352	3.04E-10	1.2
GRHL2	1.27E-14	-0.239990538	0.203	0.295	3.05E-10	1.2
BBS4	1.33E-14	-0.142957136	0.018	0.061	3.20E-10	1.2

MCCC2	1.37E-14	-0.187953452	0.067	0.13	3.31E-10	1.2
STX17	1.39E-14	-0.17304159	0.088	0.158	3.36E-10	1.2
CELF2	1.41E-14	0.310625992	0.308	0.233	3.40E-10	1.2
ZNF280D	1.46E-14	-0.210740457	0.057	0.116	3.53E-10	1.2
NUDT7	1.55E-14	-0.123598418	0.014	0.054	3.73E-10	1.2
GGA2	1.69E-14	-0.2651789	0.097	0.165	4.07E-10	1.2
DDX3X	1.70E-14	0.310343736	0.352	0.278	4.11E-10	1.2
RBBP6	1.78E-14	0.311018379	0.28	0.211	4.30E-10	1.2
HBEGF	1.79E-14	0.135809571	0.049	0.019	4.33E-10	1.2
PEA15	1.84E-14	0.299442036	0.246	0.18	4.43E-10	1.2
CREB1	1.86E-14	0.250888768	0.238	0.167	4.49E-10	1.2
RPS8	1.91E-14	-0.184431526	0.727	0.788	4.61E-10	1.2
SH3BGRL3	1.92E-14	0.372812091	0.084	0.042	4.62E-10	1.2
SEMA6A	1.96E-14	-0.268049406	0.336	0.436	4.74E-10	1.2
GTPBP4	2.51E-14	0.277607226	0.186	0.123	6.06E-10	1.2
HILPDA	2.80E-14	-0.556628599	0.189	0.264	6.74E-10	1.2
CBL	3.20E-14	0.235397829	0.134	0.081	7.71E-10	1.2
NFIB	3.22E-14	-0.15464069	0.788	0.87	7.78E-10	1.2
KMT2C	3.42E-14	-0.30649648	0.535	0.636	8.25E-10	1.2
LINC01191	3.48E-14	-0.138477753	0.022	0.066	8.39E-10	1.2
SRD5A3	3.50E-14	-0.145533875	0.021	0.065	8.44E-10	1.2
AC009313.	3.59E-14	-0.234358068	0.079	0.144	8.65E-10	1.2
NCOA7	3.97E-14	-0.190603361	0.597	0.695	9.58E-10	1.2
CCDC170	4.12E-14	-0.15869911	0.014	0.053	9.93E-10	1.2
PTK2	4.17E-14	0.25549576	0.609	0.552	1.01E-09	1.2
HIST1H4E	4.68E-14	-0.199997512	0.059	0.118	1.13E-09	1.2
NR1D2	5.19E-14	-0.190978429	0.112	0.188	1.25E-09	1.2
TGM2	5.29E-14	-0.152059352	0.047	0.103	1.28E-09	1.2
GPRC5A	5.39E-14	0.218735317	0.411	0.33	1.30E-09	1.2
UQCRH	5.72E-14	-0.205342945	0.832	0.86	1.38E-09	1.2
CRABP2	5.80E-14	0.226848452	0.123	0.071	1.40E-09	1.2
CDC25B	6.01E-14	-0.162142685	0.007	0.041	1.45E-09	1.2
ATP6V0E1	6.14E-14	0.271067813	0.686	0.662	1.48E-09	1.2
VPS13B	6.33E-14	-0.205610064	0.109	0.182	1.53E-09	1.2
SNED1	6.59E-14	-0.173126712	0.024	0.068	1.59E-09	1.2
NUCKS1	6.78E-14	-0.200499145	0.234	0.329	1.64E-09	1.2
RNF130	6.83E-14	-0.179858882	0.106	0.178	1.65E-09	1.2
USP40	6.86E-14	-0.165736341	0.032	0.081	1.65E-09	1.2
DLG5	6.92E-14	-0.285237371	0.115	0.186	1.67E-09	1.2
HNRNPA1	7.02E-14	-0.21459149	0.862	0.904	1.69E-09	1.2
BID	7.12E-14	0.272353563	0.196	0.133	1.72E-09	1.2
HP	7.19E-14	-0.451520523	0.004	0.036	1.73E-09	1.2
SMAD3	7.40E-14	0.249767018	0.171	0.11	1.78E-09	1.2
NBAT1	7.50E-14	0.22758738	0.121	0.07	1.81E-09	1.2
CITED2	7.59E-14	-0.136606218	0.019	0.062	1.83E-09	1.2
FBXL2	8.48E-14	0.225591445	0.144	0.088	2.05E-09	1.2
MRAS	8.74E-14	-0.15548013	0.037	0.087	2.11E-09	1.2

RASGEF1B	9.02E-14	0.256564018	0.907	0.891	2.17E-09	1.2
RPL15	9.06E-14	-0.15792088	0.748	0.803	2.18E-09	1.2
PDGFC	9.28E-14	-0.236682658	0.211	0.3	2.24E-09	1.2
PLCB4	9.83E-14	-0.185180963	0.073	0.136	2.37E-09	1.2
MDH1	9.99E-14	-0.232513473	0.288	0.384	2.41E-09	1.2
CP	1.00E-13	-0.206059951	0.031	0.079	2.41E-09	1.2
ANKRA2	1.01E-13	-0.150396945	0.03	0.078	2.43E-09	1.2
SCGB2B2	1.04E-13	-0.149795169	0.025	0.07	2.51E-09	1.2
PPIG	1.07E-13	-0.193879436	0.345	0.456	2.59E-09	1.2
LINC01152	1.12E-13	-0.265024912	0.077	0.14	2.70E-09	1.2
PDLIM7	1.12E-13	0.11899511	0.052	0.021	2.71E-09	1.2
PRELID1	1.13E-13	-0.179386827	0.089	0.156	2.72E-09	1.2
CD63	1.20E-13	0.330368861	0.27	0.2	2.89E-09	1.2
DTWD1	1.26E-13	-0.144824164	0.032	0.08	3.03E-09	1.2
CSF3R	1.33E-13	-0.110363979	0.008	0.043	3.22E-09	1.2
GNB2L1	1.34E-13	-0.223968914	0.65	0.73	3.23E-09	1.2
MRPL45	1.44E-13	-0.192923153	0.149	0.229	3.48E-09	1.2
ERGIC3	1.48E-13	-0.239769366	0.294	0.384	3.56E-09	1.2
DSC3	1.69E-13	0.231159672	0.06	0.026	4.08E-09	1.2
TSPAN6	1.77E-13	-0.186302696	0.087	0.152	4.26E-09	1.2
NIPAL3	1.78E-13	-0.147622518	0.039	0.09	4.28E-09	1.2
PCBP2	1.79E-13	-0.222987695	0.534	0.628	4.31E-09	1.2
FAF1	1.82E-13	-0.22405584	0.2	0.29	4.39E-09	1.2
EEPD1	2.01E-13	-0.173377476	0.023	0.066	4.84E-09	1.2
KDM6A	2.03E-13	-0.220063039	0.245	0.339	4.90E-09	1.2
FCHSD2	2.05E-13	-0.178779392	0.061	0.12	4.93E-09	1.2
HIST1H4H	2.07E-13	-0.162675016	0.033	0.081	5.00E-09	1.2
LINC00536	2.10E-13	-0.169598143	0.039	0.09	5.06E-09	1.2
RCAN3	2.15E-13	0.582965356	0.238	0.173	5.18E-09	1.2
DYNC2LI1	2.18E-13	-0.177358599	0.051	0.106	5.25E-09	1.2
SPTLC2	2.25E-13	0.25646062	0.214	0.15	5.43E-09	1.2
SLC7A11	2.35E-13	0.17851029	0.08	0.04	5.66E-09	1.2
PAK1	2.39E-13	-0.206073015	0.131	0.206	5.76E-09	1.2
CASP4	2.42E-13	-0.180242121	0.218	0.311	5.83E-09	1.2
TUBA1A	2.42E-13	0.295507971	0.31	0.238	5.84E-09	1.2
GLIPR2	2.48E-13	-0.190505793	0.079	0.141	5.98E-09	1.2
BCL7A	2.49E-13	-0.144539542	0.016	0.054	6.00E-09	1.2
RPRD2	2.62E-13	-0.213648728	0.097	0.165	6.32E-09	1.2
ZNHIT3	2.68E-13	-0.179858223	0.148	0.225	6.45E-09	1.2
FBXL17	2.84E-13	-0.200112577	0.128	0.203	6.85E-09	1.2
NAALADL2	2.87E-13	-0.230675779	0.271	0.361	6.93E-09	1.2
BCL10	3.16E-13	0.236825859	0.175	0.115	7.63E-09	1.2
PTPRG	3.18E-13	-0.237135083	0.166	0.247	7.66E-09	1.2
F3	3.55E-13	0.278783155	0.098	0.054	8.55E-09	1.2
PITPNC1	3.63E-13	0.450489976	0.265	0.2	8.76E-09	1.2
BACH1	4.10E-13	0.32515399	0.36	0.291	9.90E-09	1.2
HMGA1	4.62E-13	0.385950453	0.182	0.123	1.11E-08	1.2

NBEAL1	4.92E-13	-0.207438881	0.622	0.706	1.19E-08	1.2
CLDN4	5.27E-13	0.38860055	0.269	0.201	1.27E-08	1.2
CSF1	5.33E-13	-0.225950153	0.048	0.101	1.28E-08	1.2
ITGB4	5.33E-13	-0.149751442	0.021	0.062	1.29E-08	1.2
MTURN	5.36E-13	0.230460183	0.148	0.094	1.29E-08	1.2
ABI1	6.16E-13	0.177314919	0.759	0.719	1.49E-08	1.2
ICA1	6.18E-13	-0.189401225	0.215	0.303	1.49E-08	1.2
MBOAT2	6.30E-13	-0.168827218	0.07	0.13	1.52E-08	1.2
ILF2	6.41E-13	0.241328082	0.605	0.55	1.54E-08	1.2
ACTR3	6.53E-13	0.253463734	0.359	0.287	1.58E-08	1.2
SCOC	6.54E-13	-0.205747176	0.199	0.282	1.58E-08	1.2
AKAP12	6.58E-13	0.497761827	0.064	0.03	1.59E-08	1.2
CMTM7	6.85E-13	0.288174075	0.284	0.215	1.65E-08	1.2
SLC25A12	7.28E-13	-0.186342216	0.054	0.108	1.75E-08	1.2
MCFD2	7.39E-13	0.235114244	0.204	0.142	1.78E-08	1.2
FAM189A2	7.56E-13	-0.201807432	0.052	0.105	1.82E-08	1.2
WNT9A	7.79E-13	0.139698064	0.052	0.022	1.88E-08	1.2
IDH1	7.86E-13	-0.145122974	0.034	0.081	1.90E-08	1.2
MIR222HG	7.87E-13	0.260245469	0.139	0.086	1.90E-08	1.2
RP11-437B	7.89E-13	-0.225786338	0.317	0.416	1.90E-08	1.2
ACTR3C	8.01E-13	-0.127938072	0.019	0.058	1.93E-08	1.2
RP11-817J1	8.43E-13	-0.183460671	0.067	0.124	2.03E-08	1.2
ELF1	9.36E-13	0.283330542	0.494	0.431	2.26E-08	1.2
BAZ1A	9.97E-13	0.282818893	0.368	0.305	2.40E-08	1.2
MT-CO1	1.05E-12	-0.169170375	0.991	0.988	2.52E-08	1.2
NEDD9	1.09E-12	-0.340666675	0.211	0.287	2.63E-08	1.2
BANF1	1.11E-12	-0.169434639	0.151	0.229	2.69E-08	1.2
TOMM7	1.12E-12	-0.191396675	0.89	0.912	2.70E-08	1.2
C14orf166	1.13E-12	-0.170008928	0.181	0.263	2.73E-08	1.2
PDCD10	1.15E-12	0.302056544	0.34	0.273	2.77E-08	1.2
DLEU2	1.17E-12	-0.18026536	0.133	0.205	2.83E-08	1.2
MTIF3	1.20E-12	-0.201779802	0.18	0.261	2.90E-08	1.2
TMEM176A	1.24E-12	-0.13204282	0.021	0.061	2.99E-08	1.2
SLC5A1	1.24E-12	0.285192658	0.149	0.097	3.00E-08	1.2
CHKA	1.33E-12	-0.227240224	0.078	0.138	3.20E-08	1.2
LARP7	1.35E-12	-0.168087761	0.119	0.19	3.25E-08	1.2
CLIC1	1.37E-12	0.3005115	0.619	0.585	3.29E-08	1.2
TTC28	1.40E-12	-0.203826921	0.053	0.106	3.38E-08	1.2
THUMPD3-	1.42E-12	0.323492767	0.355	0.291	3.42E-08	1.2
PHF21A	1.42E-12	-0.262138217	0.183	0.262	3.43E-08	1.2
PTPN12	1.49E-12	0.262183335	0.473	0.413	3.60E-08	1.2
TLE4	1.51E-12	0.250375331	0.462	0.389	3.64E-08	1.2
LYPLAL1	1.61E-12	-0.145361477	0.04	0.089	3.89E-08	1.2
KTN1	1.66E-12	0.29776543	0.443	0.384	4.01E-08	1.2
MARCKSL1	1.70E-12	0.26834446	0.133	0.083	4.10E-08	1.2
SLC26A3	1.71E-12	0.256019443	0.791	0.753	4.12E-08	1.2
CARF	1.74E-12	-0.12153815	0.008	0.04	4.19E-08	1.2

CPNE3	1.74E-12	-0.181500759	0.107	0.174	4.20E-08	1.2
LMBRD1	1.79E-12	-0.205293791	0.177	0.256	4.31E-08	1.2
ABLIM3	1.83E-12	0.105354984	0.043	0.016	4.40E-08	1.2
POGZ	1.95E-12	-0.225604953	0.212	0.294	4.69E-08	1.2
ATM	1.96E-12	-0.150829613	0.037	0.084	4.73E-08	1.2
HSP90AB1	2.01E-12	0.192309175	0.948	0.934	4.85E-08	1.2
THSD4-AS1	2.03E-12	-0.194244269	0.38	0.489	4.88E-08	1.2
VCL	2.12E-12	0.256006255	0.38	0.307	5.11E-08	1.2
MYH9	2.22E-12	0.298137528	0.536	0.475	5.34E-08	1.2
UBR5	2.25E-12	-0.243858296	0.336	0.429	5.42E-08	1.2
LDLRAD3	2.25E-12	-0.242835895	0.234	0.319	5.42E-08	1.2
NDUFB1	2.27E-12	-0.208202317	0.276	0.369	5.46E-08	1.2
EGF	2.28E-12	-0.154719256	0.041	0.09	5.49E-08	1.2
TMEM14C	2.30E-12	-0.125899393	0.051	0.103	5.55E-08	1.2
BBIP1	2.31E-12	-0.152849126	0.062	0.118	5.58E-08	1.2
ZBTB43	2.48E-12	0.256302845	0.207	0.146	5.97E-08	1.2
TIMM9	2.66E-12	-0.208559455	0.163	0.237	6.41E-08	1.2
NPC2	2.81E-12	-0.215102441	0.385	0.482	6.77E-08	1.2
CTD-2626G	2.81E-12	-0.124176847	0.014	0.05	6.77E-08	1.2
RASGEF1C	2.84E-12	0.181259651	0.11	0.064	6.86E-08	1.2
FRY	2.87E-12	-0.125301633	0.013	0.048	6.93E-08	1.2
TSLP	2.95E-12	0.198337604	0.112	0.066	7.12E-08	1.2
APTX	3.04E-12	-0.233819762	0.094	0.156	7.33E-08	1.2
FAXDC2	3.12E-12	-0.128506621	0.014	0.05	7.52E-08	1.2
STAT3	3.16E-12	-0.272893641	0.445	0.539	7.62E-08	1.2
C4BPA	3.29E-12	-0.174280002	0.017	0.053	7.92E-08	1.2
FYN	3.29E-12	0.132437043	0.059	0.027	7.94E-08	1.2
KLK7	3.44E-12	0.155993091	0.064	0.03	8.31E-08	1.2
RPL23A	3.47E-12	-0.25288206	0.684	0.734	8.37E-08	1.2
ZNF83	3.51E-12	0.382068327	0.401	0.347	8.46E-08	1.2
ARHGEF10	3.68E-12	0.324796688	0.416	0.355	8.87E-08	1.2
C5orf46	3.79E-12	0.283401765	0.283	0.215	9.14E-08	1.2
TCF7L2	3.93E-12	-0.269495023	0.371	0.467	9.48E-08	1.2
GSTO2	4.00E-12	-0.176520494	0.073	0.13	9.65E-08	1.2
TCEAL4	4.26E-12	-0.197897718	0.168	0.244	1.03E-07	1.2
TRIM38	4.28E-12	-0.178339284	0.063	0.117	1.03E-07	1.2
KIZ-AS1	4.29E-12	0.174367251	0.894	0.89	1.03E-07	1.2
ALDH7A1	4.35E-12	-0.192246791	0.058	0.111	1.05E-07	1.2
RNF181	4.35E-12	0.295605792	0.436	0.371	1.05E-07	1.2
STK10	4.58E-12	0.184869179	0.098	0.056	1.11E-07	1.2
NSD1	4.70E-12	-0.19190913	0.097	0.161	1.13E-07	1.2
TBC1D22B	4.78E-12	0.241823665	0.235	0.171	1.15E-07	1.2
DLEU1	4.89E-12	-0.137798964	0.106	0.174	1.18E-07	1.2
SYCP3	4.94E-12	-0.123305672	0.008	0.038	1.19E-07	1.2
EIF4A2	5.06E-12	0.214512771	0.821	0.811	1.22E-07	1.2
DPYSL2	5.57E-12	-0.1774511	0.08	0.139	1.34E-07	1.2
RNF144B	5.63E-12	-0.187408847	0.279	0.369	1.36E-07	1.2

ARPC2	6.00E-12	0.253546008	0.223	0.163	1.45E-07	1.2
RPA3	6.37E-12	-0.162993931	0.076	0.134	1.54E-07	1.2
RP11-536O	6.62E-12	-0.124707524	0.073	0.132	1.60E-07	1.2
ISG20	6.66E-12	0.247384941	0.077	0.04	1.61E-07	1.2
PGM1	6.77E-12	-0.204780647	0.076	0.134	1.63E-07	1.2
ANKRD36	6.84E-12	0.279202079	0.188	0.131	1.65E-07	1.2
FAM129B	6.85E-12	0.279617946	0.15	0.098	1.65E-07	1.2
PNPLA3	7.11E-12	-0.113173808	0.012	0.045	1.71E-07	1.2
RP11-115D	7.15E-12	-0.143068262	0.008	0.038	1.72E-07	1.2
ACAT2	7.24E-12	-0.253503851	0.229	0.308	1.74E-07	1.2
MIB1	7.40E-12	0.252856444	0.357	0.289	1.78E-07	1.2
ACSL3	7.44E-12	-0.292703278	0.199	0.279	1.79E-07	1.2
IRS2	7.49E-12	0.244872548	0.414	0.346	1.81E-07	1.2
PKD2	7.49E-12	-0.158798695	0.047	0.096	1.81E-07	1.2
NHSL2	7.68E-12	0.285534909	0.379	0.319	1.85E-07	1.2
SREK1	8.23E-12	-0.185533773	0.103	0.167	1.98E-07	1.2
CCDC66	8.24E-12	-0.145221036	0.102	0.166	1.99E-07	1.2
RP11-475O	8.45E-12	-0.174366659	0.022	0.061	2.04E-07	1.2
CCSER1	8.65E-12	-0.246762303	0.459	0.546	2.08E-07	1.2
SLC25A33	8.97E-12	-0.245479926	0.088	0.148	2.16E-07	1.2
CEBPD	9.01E-12	-0.21883969	0.163	0.238	2.17E-07	1.2
HMG20A	9.20E-12	-0.124412862	0.026	0.067	2.22E-07	1.2
THYN1	9.31E-12	-0.109797526	0.016	0.051	2.25E-07	1.2
PCM1	9.85E-12	-0.174404628	0.135	0.206	2.38E-07	1.2
C8orf4	1.08E-11	-0.29249722	0.108	0.17	2.60E-07	1.2
LRIG3	1.09E-11	-0.132932824	0.025	0.065	2.62E-07	1.2
WDFY3	1.14E-11	-0.2023221	0.188	0.264	2.76E-07	1.2
AKAP13	1.17E-11	0.258556662	0.558	0.508	2.82E-07	1.2
MRPS18B	1.21E-11	-0.154099014	0.103	0.167	2.91E-07	1.2
AC058791.	1.22E-11	0.258135542	0.257	0.195	2.95E-07	1.2
ATP5C1	1.23E-11	-0.177815732	0.282	0.374	2.97E-07	1.2
SDCCAG8	1.27E-11	-0.206299166	0.13	0.199	3.07E-07	1.2
CHORDC1	1.29E-11	0.254734194	0.134	0.086	3.11E-07	1.2
TOP1	1.30E-11	-0.22941465	0.274	0.362	3.14E-07	1.2
RP11-7K24	1.30E-11	0.108453287	0.038	0.014	3.14E-07	1.2
MTMR3	1.31E-11	-0.194145311	0.115	0.181	3.16E-07	1.2
PIWIL4	1.35E-11	-0.156853049	0.03	0.072	3.24E-07	1.2
COMMD6	1.36E-11	-0.18958306	0.342	0.435	3.28E-07	1.2
SLC35F3	1.44E-11	0.194384294	0.082	0.044	3.48E-07	1.2
MORF4L1	1.46E-11	0.219634899	0.71	0.689	3.52E-07	1.2
TMEM176E	1.48E-11	-0.126461317	0.02	0.057	3.58E-07	1.2
MCC	1.53E-11	-0.166252096	0.071	0.126	3.70E-07	1.2
TLR5	1.56E-11	-0.116681757	0.021	0.059	3.76E-07	1.2
ELOVL6	1.60E-11	-0.193143431	0.046	0.093	3.85E-07	1.2
UST	1.60E-11	-0.303861779	0.189	0.264	3.87E-07	1.2
CNNM2	1.63E-11	-0.131395954	0.024	0.063	3.93E-07	1.2
PBRM1	1.68E-11	-0.1878962	0.141	0.213	4.06E-07	1.2

SH3BGRL	1.74E-11	-0.184634806	0.181	0.258	4.20E-07	1.2
PAPD4	1.80E-11	-0.204384706	0.229	0.312	4.34E-07	1.2
HIST1H2BG	1.83E-11	-0.152851123	0.065	0.119	4.40E-07	1.2
SNRPG	1.84E-11	0.298861077	0.578	0.535	4.43E-07	1.2
ZRANB1	1.89E-11	0.227544808	0.203	0.144	4.55E-07	1.2
PLA2G4C	1.99E-11	-0.118749129	0.027	0.066	4.80E-07	1.2
MT-ATP6	2.02E-11	-0.142298142	0.991	0.986	4.88E-07	1.2
FUT9	2.03E-11	-0.118644069	0.009	0.039	4.89E-07	1.2
MOB3B	2.06E-11	-0.191423226	0.182	0.262	4.96E-07	1.2
HSDL2	2.06E-11	-0.155704577	0.065	0.118	4.97E-07	1.2
BNIP3	2.10E-11	0.212029902	0.156	0.103	5.07E-07	1.2
EHD2	2.12E-11	0.128645778	0.075	0.039	5.12E-07	1.2
PER2	2.21E-11	-0.163604793	0.06	0.112	5.33E-07	1.2
MTF2	2.23E-11	-0.203892696	0.158	0.23	5.38E-07	1.2
SERAC1	2.29E-11	-0.203631791	0.044	0.089	5.52E-07	1.2
UFC1	2.36E-11	-0.195884131	0.441	0.53	5.70E-07	1.2
FAM49B	2.39E-11	0.23008128	0.287	0.224	5.76E-07	1.2
MTHFD1L	2.45E-11	0.353817108	0.329	0.274	5.91E-07	1.2
CTPS2	2.81E-11	-0.139487697	0.035	0.077	6.77E-07	1.2
ANTXR1	2.86E-11	-0.11384583	0.026	0.065	6.89E-07	1.2
CAV2	2.97E-11	0.168331819	0.098	0.057	7.16E-07	1.2
LINC01515	3.04E-11	-0.126789591	0.012	0.044	7.34E-07	1.2
C21orf91	3.08E-11	-0.134935924	0.033	0.075	7.44E-07	1.2
CORO1C	3.10E-11	0.183048586	0.291	0.221	7.47E-07	1.2
EGOT	3.13E-11	-0.152281729	0.039	0.082	7.56E-07	1.2
PDLIM3	3.14E-11	-0.16662426	0.031	0.071	7.57E-07	1.2
C1R	3.32E-11	-0.175437462	0.041	0.086	8.00E-07	1.2
MAP3K2	3.32E-11	-0.204310867	0.132	0.197	8.01E-07	1.2
DDAH1	3.35E-11	0.27016457	0.133	0.086	8.07E-07	1.2
TJP2	3.36E-11	0.324052695	0.456	0.405	8.09E-07	1.2
HIST1H2BN	3.39E-11	-0.101784934	0.013	0.045	8.17E-07	1.2
FNBP1	3.40E-11	-0.28482127	0.416	0.507	8.19E-07	1.2
RAB29	3.43E-11	-0.123218875	0.045	0.092	8.27E-07	1.2
LRP6	3.45E-11	-0.283655989	0.232	0.308	8.33E-07	1.2
IGBP1	3.64E-11	-0.180469428	0.382	0.476	8.77E-07	1.2
RPL12	3.72E-11	-0.174011312	0.326	0.413	8.96E-07	1.2
NUDT2	3.81E-11	-0.161811705	0.097	0.157	9.20E-07	1.2
MAP2K5	4.01E-11	-0.140182076	0.082	0.14	9.66E-07	1.2
TMEM27	4.17E-11	0.123006984	0.073	0.038	1.00E-06	1.2
LIFR	4.17E-11	-0.218530392	0.071	0.124	1.01E-06	1.2
EFNA1	4.19E-11	-0.134251147	0.069	0.123	1.01E-06	1.2
PIK3IP1	4.19E-11	-0.118445437	0.034	0.076	1.01E-06	1.2
USP13	4.20E-11	-0.149559468	0.053	0.102	1.01E-06	1.2
TTTY14	4.21E-11	0.356113857	0.351	0.289	1.02E-06	1.2
TUBB4B	4.24E-11	0.21472241	0.084	0.047	1.02E-06	1.2
DZIP3	4.25E-11	-0.117792541	0.016	0.05	1.02E-06	1.2
TNFRSF12A	4.42E-11	0.110574102	0.046	0.02	1.07E-06	1.2

LYST	4.75E-11	0.26817596	0.211	0.155	1.15E-06	1.2
HIST1H2BJ	4.87E-11	-0.18098524	0.054	0.103	1.17E-06	1.2
GRIP1	4.96E-11	-0.216588832	0.146	0.215	1.20E-06	1.2
SEC24A	5.05E-11	0.199258303	0.268	0.204	1.22E-06	1.2
PCNXL2	5.23E-11	0.300378956	0.813	0.813	1.26E-06	1.2
DNAJA1	5.33E-11	0.244257422	0.297	0.233	1.28E-06	1.2
ENO2	5.37E-11	0.135047697	0.093	0.053	1.30E-06	1.2
COPS4	5.42E-11	-0.165098494	0.086	0.144	1.31E-06	1.2
VPS13C	5.51E-11	-0.17751761	0.165	0.235	1.33E-06	1.2
MAT2A	5.70E-11	0.186551787	0.092	0.054	1.37E-06	1.2
RCOR3	5.81E-11	-0.139679212	0.057	0.107	1.40E-06	1.2
NHSL1	5.89E-11	0.328105061	0.25	0.189	1.42E-06	1.2
PEX2	5.95E-11	-0.1177699	0.057	0.107	1.44E-06	1.2
GPRIN3	6.09E-11	-0.120938583	0.009	0.038	1.47E-06	1.2
DDX1	6.11E-11	-0.14981639	0.082	0.138	1.47E-06	1.2
SPPL3	6.17E-11	-0.187759031	0.191	0.268	1.49E-06	1.2
LIF	6.31E-11	0.185570675	0.056	0.027	1.52E-06	1.2
SPATA5	6.54E-11	0.279251463	0.176	0.123	1.58E-06	1.2
CALU	6.62E-11	0.289188548	0.337	0.281	1.60E-06	1.2
VPS25	6.86E-11	-0.150424777	0.084	0.141	1.65E-06	1.2
BNIP3L	6.95E-11	0.236098606	0.359	0.293	1.68E-06	1.2
LINC00152	7.08E-11	0.204638313	0.396	0.329	1.71E-06	1.2
SNX9	7.26E-11	0.289388166	0.491	0.439	1.75E-06	1.2
SLX4IP	7.26E-11	-0.11973866	0.015	0.048	1.75E-06	1.2
ELMSAN1	7.32E-11	-0.162514411	0.046	0.091	1.77E-06	1.2
ZDHHC20	7.55E-11	-0.17145241	0.102	0.163	1.82E-06	1.2
MYOF	7.65E-11	0.287165517	0.552	0.509	1.84E-06	1.2
PDE4B	7.70E-11	-0.210027187	0.864	0.926	1.86E-06	1.2
AC073283.	7.73E-11	-0.12072255	0.072	0.126	1.86E-06	1.2
QRSL1	7.81E-11	-0.134064908	0.038	0.081	1.88E-06	1.2
MSH3	7.89E-11	-0.194171863	0.065	0.116	1.90E-06	1.2
RASGEF1A	7.94E-11	-0.132914049	0.015	0.047	1.92E-06	1.2
PPIL3	8.06E-11	-0.120420926	0.067	0.12	1.94E-06	1.2
UBE2N	8.56E-11	0.267455086	0.263	0.206	2.06E-06	1.2
MACROD2	8.68E-11	-0.243847647	0.205	0.279	2.09E-06	1.2
COX6B1	8.98E-11	-0.179673767	0.684	0.753	2.17E-06	1.2
TSC22D1	9.14E-11	-0.260636329	0.2	0.272	2.20E-06	1.2
PPP1R21	9.29E-11	0.24128304	0.127	0.082	2.24E-06	1.2
KLK10	9.76E-11	0.199203574	0.089	0.051	2.35E-06	1.2
DHRS3	9.95E-11	-0.160166787	0.054	0.102	2.40E-06	1.2
RAPGEF5	1.00E-10	-0.207251656	0.413	0.501	2.42E-06	1.2
PARG	1.08E-10	-0.159323797	0.055	0.103	2.62E-06	1.2
TMED10	1.25E-10	-0.199831153	0.252	0.333	3.00E-06	1.2
NDUFB3	1.26E-10	-0.165982784	0.162	0.233	3.05E-06	1.2
RP11-131L	1.28E-10	0.279843172	0.108	0.067	3.08E-06	1.2
LINC01482	1.29E-10	-0.127399872	0.008	0.036	3.11E-06	1.2
DAAM1	1.31E-10	0.185861397	0.561	0.502	3.16E-06	1.2

ZNF226	1.31E-10	-0.11954884	0.044	0.088	3.17E-06	1.2
SH3PXD2A	1.32E-10	0.28228569	0.19	0.136	3.18E-06	1.2
TIMM10	1.33E-10	-0.165907734	0.194	0.269	3.20E-06	1.2
ALDH1A2	1.34E-10	-0.12638715	0.015	0.047	3.23E-06	1.2
YWHAB	1.35E-10	0.252832742	0.326	0.267	3.26E-06	1.2
RPS13	1.36E-10	-0.280338022	0.84	0.851	3.28E-06	1.2
NFATC2	1.42E-10	0.196074615	0.191	0.133	3.42E-06	1.2
PARD3	1.42E-10	0.225931933	0.672	0.638	3.42E-06	1.2
TSC22D2	1.42E-10	-0.457546049	0.275	0.337	3.43E-06	1.2
MAP2K3	1.46E-10	0.168260733	0.094	0.055	3.52E-06	1.2
PDSS2	1.46E-10	-0.187680715	0.092	0.149	3.53E-06	1.2
CHCHD3	1.47E-10	-0.159604027	0.857	0.891	3.53E-06	1.2
OARD1	1.52E-10	-0.128819942	0.061	0.111	3.65E-06	1.2
PAWR	1.57E-10	0.197525014	0.525	0.468	3.79E-06	1.2
MTHFD2L	1.58E-10	0.438146664	0.646	0.607	3.81E-06	1.2
FDFT1	1.60E-10	-0.23898055	0.279	0.356	3.85E-06	1.2
TGFBR3	1.66E-10	-0.248120925	0.14	0.207	4.00E-06	1.2
FAM210B	1.66E-10	-0.114929599	0.03	0.069	4.01E-06	1.2
TIMM17A	1.70E-10	0.264877092	0.265	0.209	4.09E-06	1.2
DIMT1	1.72E-10	-0.119574266	0.035	0.076	4.15E-06	1.2
FBLIM1	1.74E-10	0.210087966	0.188	0.134	4.21E-06	1.2
CCDC122	1.75E-10	-0.108695492	0.021	0.056	4.21E-06	1.2
CTD-2020K	1.76E-10	0.110109763	0.037	0.014	4.23E-06	1.2
MTRNR2L1	1.78E-10	0.303345405	0.328	0.268	4.30E-06	1.2
MYO1B	1.81E-10	0.285226315	0.379	0.319	4.35E-06	1.2
SKAP2	1.85E-10	-0.171945172	0.258	0.343	4.46E-06	1.2
FAM151B	1.88E-10	-0.123430449	0.021	0.056	4.53E-06	1.2
NDUFA12	1.89E-10	-0.162372301	0.176	0.249	4.55E-06	1.2
TMLHE	1.93E-10	-0.131989882	0.029	0.067	4.66E-06	1.2
CX3CL1	2.02E-10	-0.261892305	0.209	0.282	4.87E-06	1.2
RB1CC1	2.07E-10	0.252850975	0.445	0.393	4.99E-06	1.2
UVRAG	2.10E-10	-0.240351129	0.352	0.435	5.06E-06	1.2
OTUD6B-AS	2.14E-10	-0.136532821	0.144	0.214	5.16E-06	1.2
AUTS2	2.15E-10	-0.218304889	0.495	0.582	5.18E-06	1.2
AP006222.	2.16E-10	0.129340396	0.063	0.032	5.22E-06	1.2
COTL1	2.20E-10	0.149251969	0.066	0.035	5.30E-06	1.2
PDZD11	2.28E-10	-0.123293999	0.057	0.104	5.51E-06	1.2
CYP27A1	2.30E-10	-0.141126884	0.048	0.093	5.55E-06	1.2
MRRF	2.31E-10	-0.15500331	0.121	0.184	5.56E-06	1.2
SSR3	2.53E-10	0.223169296	0.613	0.586	6.10E-06	1.2
RP11-371F	2.58E-10	0.157601449	0.056	0.028	6.21E-06	1.2
BTAF1	2.58E-10	-0.265147772	0.195	0.262	6.23E-06	1.2
KLC1	2.69E-10	0.153311821	0.069	0.037	6.49E-06	1.2
RAD51C	2.71E-10	-0.10224927	0.024	0.059	6.53E-06	1.2
JARID2	2.71E-10	-0.256925498	0.199	0.271	6.54E-06	1.2
PTPRM	2.73E-10	-0.357167944	0.127	0.187	6.59E-06	1.2
MICAL2	2.75E-10	-0.176855617	0.034	0.073	6.62E-06	1.2

TFDP2	2.78E-10	-0.175729236	0.173	0.243	6.72E-06	1.2
CNIH4	2.83E-10	0.257162544	0.422	0.37	6.83E-06	1.2
PREPL	2.87E-10	-0.195195883	0.088	0.142	6.92E-06	1.2
CDC27	2.99E-10	-0.227289313	0.187	0.256	7.20E-06	1.2
EPB41L3	3.11E-10	0.143744802	0.039	0.016	7.50E-06	1.2
FRMD4A	3.19E-10	-0.339421703	0.419	0.494	7.68E-06	1.2
SLTM	3.23E-10	-0.175237307	0.203	0.276	7.80E-06	1.2
CD163L1	3.36E-10	-0.125072616	0.034	0.073	8.10E-06	1.2
B4GALNT3	3.36E-10	0.128472368	0.077	0.042	8.10E-06	1.2
DUSP5	3.38E-10	0.156613431	0.1	0.061	8.15E-06	1.2
SRI	3.39E-10	0.191856466	0.088	0.051	8.17E-06	1.2
HIVEP3	3.40E-10	-0.227902944	0.254	0.337	8.20E-06	1.2
SCGB1D2	3.42E-10	0.37206892	0.13	0.084	8.25E-06	1.2
DIAPH2	3.44E-10	-0.184283454	0.216	0.291	8.30E-06	1.2
ADH5	3.44E-10	-0.141991304	0.081	0.134	8.31E-06	1.2
LSAMP	3.53E-10	0.259762332	0.297	0.236	8.52E-06	1.2
MSI2	3.55E-10	-0.168399149	0.146	0.212	8.55E-06	1.2
CETN3	3.55E-10	-0.106091975	0.021	0.054	8.56E-06	1.2
KCTD9	3.68E-10	0.240885673	0.35	0.287	8.87E-06	1.2
RAD50	3.81E-10	-0.13895993	0.062	0.111	9.19E-06	1.2
ARHGDIB	3.84E-10	-0.195023729	0.131	0.193	9.25E-06	1.2
PTPN4	3.91E-10	-0.136134979	0.055	0.102	9.43E-06	1.2
LINC01138	4.02E-10	-0.19639104	0.177	0.246	9.69E-06	1.2
ST14	4.03E-10	0.226018536	0.223	0.167	9.71E-06	1.2
ANKRD26	4.04E-10	-0.126152139	0.042	0.084	9.74E-06	1.2
TARS	4.12E-10	0.249887403	0.239	0.183	9.93E-06	1.2
RUBCN	4.13E-10	0.148665784	0.098	0.058	9.96E-06	1.2
ARPP19	4.23E-10	0.203206753	0.382	0.32	1.02E-05	1.2
ATG14	4.34E-10	-0.181879598	0.063	0.11	1.05E-05	1.2
CCT3	4.45E-10	-0.137447404	0.368	0.461	1.07E-05	1.2
MFSD11	4.49E-10	-0.134478275	0.04	0.081	1.08E-05	1.2
MT-ND4	4.75E-10	-0.104649568	0.994	0.989	1.15E-05	1.2
KCNK1	4.79E-10	0.245526587	0.215	0.161	1.15E-05	1.2
MLF1	4.80E-10	-0.176765258	0.176	0.244	1.16E-05	1.2
DISC1	4.83E-10	-0.15859098	0.064	0.113	1.16E-05	1.2
TMEM126E	4.96E-10	-0.136035421	0.08	0.133	1.20E-05	1.2
S100A2	5.15E-10	0.577460346	0.136	0.091	1.24E-05	1.2
ABCC4	5.45E-10	-0.133338361	0.013	0.043	1.31E-05	1.2
NPC1	5.55E-10	0.240496309	0.372	0.31	1.34E-05	1.2
RPL14	5.57E-10	-0.147934215	0.989	0.979	1.34E-05	1.2
ODAM	5.59E-10	-0.208358369	0.006	0.031	1.35E-05	1.2
ZDHHC14	5.61E-10	0.23473099	0.138	0.093	1.35E-05	1.2
TFCP2	5.69E-10	-0.163572901	0.093	0.149	1.37E-05	1.2
PSMC5	5.73E-10	-0.124194225	0.063	0.112	1.38E-05	1.2
SEC24D	5.89E-10	-0.268686136	0.242	0.315	1.42E-05	1.2
ATP5G1	6.02E-10	-0.165200189	0.156	0.221	1.45E-05	1.2
ADAMTS9-/	6.07E-10	0.124927005	0.045	0.02	1.46E-05	1.2

SPIRE1	6.13E-10	-0.201961145	0.334	0.418	1.48E-05	1.2
PCLO	6.22E-10	-0.142037102	0.02	0.052	1.50E-05	1.2
SLC19A2	6.56E-10	-0.145953225	0.055	0.1	1.58E-05	1.2
GABRG3	6.62E-10	-0.134964841	0.016	0.046	1.60E-05	1.2
NOL3	6.72E-10	-0.117268039	0.024	0.058	1.62E-05	1.2
ITGB8	6.72E-10	0.221399236	0.748	0.733	1.62E-05	1.2
HPCAL1	7.21E-10	0.249659523	0.145	0.099	1.74E-05	1.2
MBD5	7.27E-10	-0.193651229	0.209	0.283	1.75E-05	1.2
CTNNA1	7.31E-10	0.249755989	0.528	0.49	1.76E-05	1.2
SNCAIP	7.53E-10	-0.122374666	0.009	0.035	1.82E-05	1.2
SOX5	8.02E-10	-0.156573653	0.017	0.047	1.93E-05	1.2
C1QTNF1	8.04E-10	-0.119128511	0.02	0.053	1.94E-05	1.2
FZD7	8.14E-10	-0.124236114	0.046	0.088	1.96E-05	1.2
EVL	8.27E-10	-0.15699231	0.032	0.07	1.99E-05	1.2
TMEM126A	8.31E-10	-0.126566119	0.083	0.136	2.00E-05	1.2
BACH2	8.40E-10	0.159996572	0.461	0.399	2.03E-05	1.2
BMP2K	9.01E-10	0.160610077	0.09	0.053	2.17E-05	1.2
B4GALT5	9.21E-10	0.256894057	0.379	0.321	2.22E-05	1.2
EML4	9.36E-10	-0.177019531	0.208	0.281	2.26E-05	1.2
POLR2F	9.40E-10	-0.14197671	0.039	0.079	2.27E-05	1.2
RNASE1	9.55E-10	-0.119728578	0.017	0.047	2.30E-05	1.2
CTD-3252C	9.80E-10	0.175060011	0.123	0.08	2.36E-05	1.2
BAMBI	1.00E-09	-0.132807525	0.046	0.088	2.41E-05	1.2
EFNA5	1.02E-09	-0.237366483	0.437	0.514	2.46E-05	1.2
CLIC4	1.02E-09	0.21322012	0.685	0.647	2.47E-05	1.2
DDX5	1.02E-09	0.18563672	0.544	0.495	2.47E-05	1.2
MBNL1	1.08E-09	-0.205520051	0.358	0.444	2.59E-05	1.2
CREB5	1.08E-09	0.429166203	0.36	0.307	2.61E-05	1.2
ARID2	1.12E-09	-0.190671823	0.239	0.316	2.70E-05	1.2
CHDH	1.12E-09	-0.126491299	0.027	0.062	2.70E-05	1.2
DUSP14	1.14E-09	0.146720257	0.063	0.033	2.75E-05	1.2
TNS3	1.16E-09	-0.190657314	0.048	0.09	2.79E-05	1.2
IP6K2	1.17E-09	-0.200333649	0.163	0.229	2.81E-05	1.2
SULF2	1.19E-09	-0.160967431	0.064	0.111	2.86E-05	1.2
APOO	1.19E-09	-0.122983733	0.988	0.98	2.87E-05	1.2
ITPR3	1.25E-09	0.169868225	0.074	0.042	3.01E-05	1.2
TRIM22	1.25E-09	-0.139238944	0.07	0.118	3.01E-05	1.2
CRNDE	1.26E-09	-0.145261749	0.041	0.081	3.05E-05	1.2
USP47	1.33E-09	-0.14616586	0.253	0.33	3.21E-05	1.2
CASC4	1.33E-09	-0.177702818	0.136	0.198	3.22E-05	1.2
ITGB3BP	1.34E-09	-0.154156078	0.06	0.105	3.24E-05	1.2
UQCR10	1.39E-09	-0.142582584	0.336	0.419	3.35E-05	1.2
EPB41L2	1.39E-09	-0.257606789	0.144	0.206	3.36E-05	1.2
BCL11A	1.41E-09	-0.11767747	0.032	0.069	3.40E-05	1.2
CCDC57	1.42E-09	-0.144361488	0.036	0.075	3.43E-05	1.2
ATP5A1	1.43E-09	-0.130433246	0.133	0.195	3.45E-05	1.2
TCEAL8	1.44E-09	-0.152320966	0.267	0.348	3.46E-05	1.2

EPC1	1.44E-09	-0.142209447	0.099	0.155	3.47E-05	1.2
GOLGA3	1.52E-09	0.140991125	0.076	0.043	3.65E-05	1.2
TMEM230	1.55E-09	-0.135343485	0.039	0.078	3.74E-05	1.2
HAUS1	1.57E-09	-0.112398891	0.036	0.074	3.78E-05	1.2
NR4A2	1.58E-09	-0.142774702	0.029	0.064	3.82E-05	1.2
ATRNL1	1.60E-09	-0.194344445	0.046	0.087	3.87E-05	1.2
PSME1	1.62E-09	-0.134462949	0.15	0.214	3.92E-05	1.2
GSTK1	1.68E-09	-0.142314224	0.047	0.088	4.04E-05	1.2
BACE2	1.74E-09	0.242687589	0.498	0.447	4.19E-05	1.2
PPM1H	1.82E-09	-0.189072389	0.315	0.398	4.39E-05	1.2
SCMH1	1.86E-09	-0.122594458	0.163	0.232	4.50E-05	1.2
CATSPER2	1.88E-09	-0.140570283	0.053	0.097	4.53E-05	1.2
FAM20A	1.92E-09	-0.133006301	0.04	0.079	4.62E-05	1.2
ANKLE2	1.94E-09	0.156748757	0.152	0.105	4.68E-05	1.2
GNPTAB	1.97E-09	-0.182014501	0.074	0.122	4.75E-05	1.2
STK17B	2.03E-09	-0.186063513	0.083	0.134	4.90E-05	1.2
CXCL6	2.04E-09	-0.165820901	0.015	0.044	4.92E-05	1.2
VGLL4	2.07E-09	-0.206303812	0.213	0.283	5.00E-05	1.2
HIP1	2.09E-09	-0.145356314	0.057	0.102	5.04E-05	1.2
ANXA4	2.09E-09	-0.133001567	0.07	0.119	5.05E-05	1.2
PMS1	2.11E-09	-0.120017804	0.023	0.056	5.08E-05	1.2
DIS3L2	2.12E-09	-0.105834141	0.068	0.116	5.11E-05	1.2
DRAM2	2.18E-09	-0.16236066	0.233	0.308	5.26E-05	1.2
VPS45	2.23E-09	-0.132012756	0.055	0.098	5.37E-05	1.2
C3orf35	2.23E-09	0.175990599	0.067	0.037	5.38E-05	1.2
ALOX5	2.24E-09	0.212136501	0.17	0.122	5.40E-05	1.2
SH3D19	2.25E-09	0.250892492	0.388	0.332	5.42E-05	1.2
SUSD4	2.26E-09	-0.100245692	0.013	0.041	5.44E-05	1.2
PFN1	2.28E-09	0.212954815	0.218	0.166	5.50E-05	1.2
C2orf76	2.33E-09	-0.120384446	0.034	0.071	5.63E-05	1.2
SHOC2	2.34E-09	0.192108818	0.264	0.208	5.65E-05	1.2
AASDH	2.35E-09	-0.101422257	0.029	0.064	5.67E-05	1.2
NACA2	2.37E-09	-0.209319554	0.586	0.651	5.72E-05	1.2
MFSD14A	2.43E-09	-0.188456795	0.096	0.148	5.85E-05	1.2
CARS	2.44E-09	0.186525527	0.124	0.082	5.88E-05	1.2
CXADR	2.44E-09	-0.199574473	0.18	0.244	5.88E-05	1.2
SIPA1L1	2.46E-09	-0.158879427	0.294	0.374	5.94E-05	1.2
S100A11	2.53E-09	0.196639528	0.758	0.691	6.11E-05	1.2
EYA2	2.59E-09	-0.15066588	0.154	0.219	6.25E-05	1.2
EFHD1	2.70E-09	-0.124248723	0.025	0.058	6.52E-05	1.2
AC004231.	2.79E-09	0.184202701	0.091	0.055	6.73E-05	1.2
RP11-290O	2.86E-09	-0.175077676	0.061	0.106	6.90E-05	1.2
ARHGAP10	2.89E-09	0.245620101	0.173	0.125	6.97E-05	1.2
AC016831.	2.92E-09	0.228269815	0.301	0.243	7.04E-05	1.2
PLRG1	2.93E-09	-0.123851503	0.08	0.131	7.08E-05	1.2
KLRD1	2.95E-09	0.193168862	0.102	0.064	7.11E-05	1.2
BHLHE40	3.01E-09	-0.168455149	0.067	0.112	7.25E-05	1.2

CRYAB	3.04E-09	-0.210225446	0.455	0.53	7.32E-05	1.2
CRY2	3.09E-09	-0.157626012	0.038	0.076	7.45E-05	1.2
AK3	3.19E-09	-0.1258924	0.092	0.145	7.68E-05	1.2
DMTF1	3.42E-09	0.21568874	0.154	0.109	8.24E-05	1.2
CRADD	3.45E-09	-0.145244292	0.065	0.111	8.33E-05	1.2
KCNMA1	3.47E-09	0.243639513	0.068	0.038	8.36E-05	1.2
IL20	3.49E-09	0.139802196	0.027	0.009	8.42E-05	1.2
RASSF8	3.53E-09	0.218119715	0.196	0.146	8.52E-05	1.2
R3HDM1	3.58E-09	-0.126711184	0.033	0.069	8.64E-05	1.2
DEPTOR	3.60E-09	-0.119757767	0.013	0.04	8.67E-05	1.2
POLK	3.69E-09	-0.157961215	0.098	0.152	8.89E-05	1.2
RELA	3.70E-09	0.151782992	0.124	0.082	8.92E-05	1.2
BEND7	3.74E-09	0.155732234	0.113	0.073	9.01E-05	1.2
ZNF281	3.81E-09	0.154905991	0.124	0.082	9.18E-05	1.2
NEK9	3.81E-09	-0.104267157	0.04	0.079	9.20E-05	1.2
RIPK2	3.85E-09	-0.272523361	0.344	0.412	9.28E-05	1.2
VCAM1	3.85E-09	-0.110939734	0.003	0.022	9.29E-05	1.2
ATP5H	3.90E-09	-0.14496024	0.311	0.395	9.41E-05	1.2
NFAT5	3.92E-09	-0.203904131	0.566	0.643	9.46E-05	1.2
C18orf8	3.94E-09	0.174963886	0.097	0.06	9.51E-05	1.2
CNTNAP3	4.01E-09	-0.153103284	0.034	0.07	9.66E-05	1.2
IARS	4.07E-09	0.255246503	0.172	0.125	9.82E-05	1.2
SMAD1	4.14E-09	-0.12235475	0.054	0.097	9.99E-05	1.2
DPH6	4.23E-09	-0.145192218	0.04	0.078	0.000102	1.2
MKL2	4.29E-09	-0.190442865	0.204	0.275	0.0001035	1.2
CREB3L2	4.32E-09	-0.154843208	0.077	0.125	0.0001041	1.2
TBC1D32	4.39E-09	-0.139982722	0.028	0.062	0.0001058	1.2
NASP	4.45E-09	-0.147301658	0.134	0.195	0.0001073	1.2
MBNL2	4.46E-09	-0.191336254	0.345	0.424	0.0001075	1.2
VPS50	4.47E-09	-0.114422468	0.031	0.066	0.0001078	1.2
COG7	4.69E-09	-0.127699068	0.047	0.087	0.0001131	1.2
IFI16	4.77E-09	-0.175800774	0.222	0.291	0.000115	1.2
KHDRBS3	4.91E-09	0.216651923	0.161	0.115	0.0001184	1.2
PRELID2	5.12E-09	0.191607887	0.107	0.068	0.0001234	1.2
MB21D2	5.21E-09	-0.147908717	0.043	0.082	0.0001257	1.2
CFL1	5.24E-09	0.300558452	0.286	0.231	0.0001263	1.2
C6orf89	5.41E-09	-0.109652161	0.048	0.089	0.0001306	1.2
LARGE	5.49E-09	0.244208562	0.276	0.222	0.0001324	1.2
XRCC4	5.55E-09	-0.106447016	0.027	0.06	0.0001338	1.2
ACTR2	5.60E-09	0.259812577	0.255	0.205	0.0001351	1.2
ICE2	5.69E-09	-0.107294574	0.026	0.058	0.0001371	1.2
MRPS14	5.71E-09	-0.117965636	0.115	0.172	0.0001378	1.2
ADARB1	5.72E-09	-0.105741887	0.031	0.066	0.0001379	1.2
LINC00342	5.73E-09	0.168458137	0.092	0.056	0.0001381	1.2
KALRN	5.79E-09	-0.174095126	0.09	0.141	0.0001395	1.2
C14orf159	5.90E-09	-0.116493863	0.038	0.076	0.0001424	1.2
HADHA	6.01E-09	-0.178540149	0.294	0.371	0.000145	1.2

NAA38	6.06E-09	-0.121574607	0.035	0.071	0.0001462	1.2
CHIC2	6.29E-09	0.196995835	0.274	0.219	0.0001518	1.2
CLASP2	6.49E-09	-0.160319392	0.18	0.247	0.0001566	1.2
GALK2	6.51E-09	-0.145586403	0.046	0.085	0.0001569	1.2
ATF4	6.59E-09	0.227691714	0.404	0.35	0.0001588	1.2
FBXO9	6.60E-09	-0.108720792	0.041	0.079	0.0001592	1.2
HDGF	6.68E-09	0.265860134	0.303	0.253	0.0001612	1.2
MARS	6.69E-09	0.198798826	0.172	0.124	0.0001612	1.2
ZSWIM7	6.77E-09	-0.135296222	0.043	0.081	0.0001632	1.2
C7orf73	6.77E-09	-0.12585074	0.078	0.127	0.0001633	1.2
IGFBP3	6.88E-09	0.251799239	0.153	0.107	0.0001659	1.2
S100A9	6.93E-09	-0.149994645	0.039	0.076	0.000167	1.2
MAP2K6	7.06E-09	-0.118723561	0.012	0.037	0.0001702	1.2
HCFC2	7.11E-09	-0.107380858	0.016	0.043	0.0001713	1.2
C3orf14	7.13E-09	-0.137571683	0.048	0.087	0.000172	1.2
CISD1	7.15E-09	-0.133471854	0.172	0.239	0.0001724	1.2
GK5	7.19E-09	-0.106625573	0.039	0.076	0.0001733	1.2
ANAPC13	7.28E-09	-0.122970426	0.086	0.138	0.0001756	1.2
PSMB8	7.29E-09	-0.121290246	0.076	0.124	0.0001758	1.2
COBLL1	7.47E-09	-0.116447144	0.09	0.141	0.0001802	1.2
SLC44A1	7.59E-09	-0.121805655	0.058	0.101	0.000183	1.2
HNRNPA1L	7.60E-09	-0.103698174	0.042	0.079	0.0001833	1.2
LYRM2	7.64E-09	-0.101616392	0.073	0.12	0.0001843	1.2
LIAS	7.67E-09	-0.113366809	0.036	0.073	0.000185	1.2
PFDN5	7.76E-09	-0.143960108	0.887	0.901	0.0001871	1.2
ATP1B3	7.80E-09	0.238913566	0.31	0.256	0.0001881	1.2
NSF	7.84E-09	-0.170918071	0.114	0.169	0.000189	1.2
SF3B6	7.86E-09	0.251629071	0.673	0.662	0.0001895	1.2
RPS7	7.87E-09	-0.124499286	0.978	0.966	0.0001898	1.2
NBN	7.90E-09	-0.103775542	0.038	0.075	0.0001904	1.2
SPTBN1	7.93E-09	-0.184076678	0.154	0.215	0.0001913	1.2
RPS6KA3	7.97E-09	0.19382421	0.392	0.331	0.0001923	1.2
CCDC90B	8.02E-09	-0.124578252	0.063	0.107	0.0001933	1.2
AKAP9	8.02E-09	-0.152841866	0.3	0.38	0.0001933	1.2
EIF3H	8.23E-09	-0.150909608	0.641	0.715	0.0001985	1.2
NUBPL	8.38E-09	-0.131537639	0.035	0.07	0.000202	1.2
CAMKMT	8.63E-09	-0.148334951	0.118	0.174	0.0002082	1.2
PEAK1	8.63E-09	0.207788639	0.22	0.169	0.0002082	1.2
HOMER2	8.67E-09	-0.2073762	0.168	0.229	0.0002091	1.2
SGCE	8.69E-09	-0.101424989	0.037	0.074	0.0002094	1.2
TRIM2	8.72E-09	-0.174991445	0.247	0.319	0.0002102	1.2
DENND4C	8.75E-09	-0.140876579	0.165	0.229	0.0002111	1.2
XPR1	8.76E-09	-0.124565997	0.084	0.133	0.0002112	1.2
GTF2H5	8.86E-09	-0.119621508	0.173	0.24	0.0002136	1.2
INTS12	8.91E-09	-0.187208378	0.148	0.206	0.0002148	1.2
RABGAP1L	9.04E-09	-0.13525355	0.177	0.241	0.0002179	1.2
NHS	9.12E-09	-0.164714695	0.142	0.203	0.0002198	1.2

COL6A1	9.20E-09	0.222630094	0.069	0.039	0.0002217	1.2
FAM208A	9.51E-09	-0.104498111	0.042	0.08	0.0002293	1.2
DDIT3	9.58E-09	-0.27876995	0.088	0.136	0.0002311	1.2
RPS24	9.65E-09	-0.109575427	0.988	0.981	0.0002327	1.2
APBB2	9.69E-09	-0.123400716	0.071	0.117	0.0002337	1.2
GPAT3	9.81E-09	0.143536303	0.116	0.076	0.0002365	1.2
CAB39	1.01E-08	0.303815258	0.317	0.266	0.000244	1.2
ACADM	1.03E-08	-0.101573018	0.051	0.092	0.0002478	1.2
ZBTB10	1.04E-08	-0.168694655	0.125	0.181	0.0002506	1.2
TRMT10B	1.06E-08	-0.107097754	0.076	0.124	0.0002552	1.2
ENSA	1.06E-08	-0.148192797	0.318	0.4	0.0002567	1.2
MBOAT1	1.08E-08	-0.174069435	0.04	0.076	0.0002604	1.2
SNTB1	1.09E-08	-0.122434206	0.035	0.071	0.000262	1.2
DRAM1	1.11E-08	-0.139978967	0.124	0.181	0.0002684	1.2
RRAS2	1.15E-08	0.20712142	0.267	0.214	0.0002762	1.2
IRF1	1.16E-08	-0.16013306	0.106	0.16	0.0002807	1.2
ARHGEF3	1.18E-08	-0.197706206	0.254	0.326	0.0002846	1.2
ATR	1.20E-08	-0.142700957	0.124	0.181	0.0002895	1.2
MRPL27	1.21E-08	-0.112146628	0.126	0.184	0.0002907	1.2
HDAC8	1.21E-08	-0.190774206	0.178	0.241	0.0002923	1.2
UBE2H	1.22E-08	0.133309862	0.743	0.709	0.0002937	1.2
EPHA4	1.22E-08	-0.119530192	0.025	0.057	0.0002937	1.2
PGAP1	1.24E-08	-0.113226594	0.038	0.075	0.0002985	1.2
NAP1L1	1.31E-08	-0.154609831	0.379	0.462	0.0003153	1.2
UBD	1.34E-08	-0.14646676	0.125	0.181	0.0003227	1.2
SF3A3	1.34E-08	-0.111671622	0.05	0.09	0.0003233	1.2
HDAC7	1.37E-08	0.212207771	0.167	0.121	0.00033	1.2
IER3	1.39E-08	-0.169076361	0.106	0.159	0.0003352	1.2
ERBB4	1.40E-08	-0.174621699	0.078	0.124	0.0003371	1.2
TBCK	1.47E-08	-0.154095697	0.088	0.136	0.0003539	1.2
EIF1	1.49E-08	0.293194202	0.466	0.418	0.0003593	1.2
DCTN4	1.52E-08	-0.168597254	0.128	0.183	0.0003667	1.2
VPS13D	1.57E-08	-0.108985081	0.265	0.343	0.0003787	1.2
OST4	1.60E-08	-0.11586617	0.293	0.37	0.0003848	1.2
CCDC64	1.64E-08	-0.105000788	0.014	0.039	0.0003959	1.2
ARMT1	1.75E-08	-0.114055404	0.057	0.099	0.0004224	1.2
WDR7	1.81E-08	-0.12808237	0.027	0.059	0.0004365	1.2
EEF1A1	1.84E-08	-0.103350723	0.987	0.982	0.0004446	1.2
TTC14	1.88E-08	-0.1276366	0.045	0.083	0.0004537	1.2
MORF4L2	1.89E-08	0.168077607	0.754	0.744	0.0004549	1.2
HMGN2	1.90E-08	-0.112355681	0.036	0.071	0.0004578	1.2
IRX2	1.93E-08	-0.107023287	0.049	0.088	0.0004643	1.2
HMGB1	2.01E-08	-0.153650676	0.48	0.557	0.0004858	1.2
MTERF4	2.08E-08	-0.10147136	0.055	0.095	0.0005026	1.2
NRDC	2.13E-08	-0.15547303	0.133	0.189	0.0005143	1.2
SPP1	2.15E-08	-0.35958357	0.006	0.026	0.000518	1.2
ZMYM5	2.19E-08	-0.148720097	0.129	0.184	0.0005272	1.2

MAP3K9	2.19E-08	0.19349436	0.212	0.162	0.0005286	1.2
MSN	2.21E-08	0.165972193	0.495	0.457	0.000533	1.2
OXCT1	2.25E-08	-0.11137108	0.021	0.049	0.0005422	1.2
FOS	2.32E-08	-0.246435183	0.195	0.258	0.0005597	1.2
KRIT1	2.34E-08	-0.149842843	0.086	0.133	0.0005652	1.2
RALGAPA1	2.37E-08	-0.166640983	0.186	0.25	0.0005715	1.2
GNAI3	2.40E-08	0.227003247	0.197	0.149	0.0005787	1.2
STEAP3	2.55E-08	-0.108531337	0.022	0.051	0.0006144	1.2
PTPRF	2.60E-08	-0.152261181	0.059	0.099	0.000627	1.2
HINT3	2.67E-08	-0.102418492	0.033	0.067	0.0006438	1.2
PSMB2	2.67E-08	-0.129014124	0.1	0.15	0.000644	1.2
MSMO1	2.72E-08	-0.141547585	0.216	0.285	0.0006548	1.2
GMPR2	2.72E-08	-0.111048966	0.052	0.091	0.0006566	1.2
RSRC1	2.76E-08	-0.131874896	0.203	0.273	0.0006664	1.2
C5orf17	2.77E-08	-0.179953277	0.038	0.073	0.0006688	1.2
HSF2	2.79E-08	-0.155064603	0.054	0.093	0.0006724	1.2
TPD52L1	2.83E-08	-0.178421622	0.178	0.239	0.0006827	1.2
PGM2L1	3.01E-08	0.185623884	0.141	0.1	0.0007265	1.2
LCORL	3.06E-08	-0.15806031	0.083	0.129	0.0007379	1.2
ATXN7L1	3.08E-08	-0.126951518	0.035	0.068	0.0007423	1.2
RSL24D1	3.11E-08	0.180958166	0.759	0.741	0.0007492	1.2
PHB2	3.20E-08	-0.112753881	0.077	0.123	0.0007721	1.2
BTBD11	3.23E-08	-0.13803502	0.046	0.084	0.0007797	1.2
CAP2	3.34E-08	-0.130563988	0.065	0.107	0.0008049	1.2
RNF213	3.39E-08	-0.138969212	0.136	0.193	0.0008179	1.2
PDXK	3.41E-08	-0.169170257	0.119	0.173	0.0008222	1.2
NDUFB9	3.46E-08	-0.117777719	0.152	0.211	0.0008334	1.2
ZFHX3	3.53E-08	-0.106451783	0.08	0.126	0.0008522	1.2
ENOX2	3.61E-08	-0.108294048	0.031	0.063	0.0008695	1.2
DHFR	3.66E-08	0.198616544	0.862	0.845	0.0008816	1.2
CBY1	3.66E-08	-0.103327878	0.044	0.081	0.0008823	1.2
INO80D	3.71E-08	0.197524264	0.546	0.488	0.0008956	1.2
PPIP5K1	3.75E-08	-0.10747031	0.023	0.052	0.0009032	1.2
C11orf80	3.83E-08	-0.146286049	0.171	0.232	0.000923	1.2
TOP2B	3.88E-08	-0.166705254	0.135	0.19	0.0009348	1.2
DDI2	3.90E-08	-0.102822106	0.07	0.115	0.0009412	1.2
HIVEP1	3.92E-08	0.198122722	0.196	0.149	0.0009463	1.2
ATP6V1G1	3.97E-08	-0.117286125	0.299	0.378	0.0009575	1.2
NRG2	3.97E-08	-0.105344369	0.034	0.067	0.0009578	1.2
KMT5B	3.98E-08	-0.136245895	0.119	0.173	0.0009587	1.2
MLLT10	4.03E-08	-0.156288585	0.122	0.177	0.0009723	1.2
DPYD-AS1	4.06E-08	-0.127912293	0.059	0.1	0.0009792	1.2
PPP2R5A	4.08E-08	-0.142960081	0.109	0.16	0.0009841	1.2
MLH3	4.11E-08	-0.117604178	0.067	0.11	0.000991	1.2
DNTTIP2	4.19E-08	0.263647208	0.252	0.206	0.0010104	1.2
SFN	4.20E-08	0.116475239	0.033	0.014	0.0010122	1.2
EEF2K	4.31E-08	-0.10159041	0.017	0.043	0.0010388	1.2

MON2	4.32E-08	-0.140204943	0.207	0.272	0.0010414	1.2
CACNA2D1	4.42E-08	-0.127127838	0.022	0.051	0.0010668	1.2
AC005042.	4.53E-08	-0.150912303	0.044	0.081	0.0010934	1.2
KRT7	4.59E-08	0.217973486	0.321	0.265	0.0011076	1.2
RAD23B	4.67E-08	-0.280440317	0.369	0.434	0.0011252	1.2
PGBD5	4.78E-08	0.153490724	0.108	0.072	0.0011515	1.2
TNFAIP8	4.81E-08	0.323201211	0.438	0.393	0.0011607	1.2
SMARCA1	4.82E-08	0.193083128	0.181	0.135	0.001163	1.2
CKS1B	4.84E-08	-0.113203118	0.326	0.409	0.0011663	1.2
RALY	4.87E-08	0.198118456	0.191	0.145	0.0011751	1.2
POMP	5.13E-08	0.228929784	0.677	0.656	0.0012366	1.2
MYCBP2	5.19E-08	-0.144182067	0.13	0.185	0.0012516	1.2
YY1AP1	5.29E-08	-0.137062789	0.127	0.18	0.0012749	1.2
SOX9	5.33E-08	0.221129547	0.237	0.189	0.0012847	1.2
AKAP1	5.68E-08	-0.102951238	0.018	0.044	0.0013688	1.2
CLEC2B	5.73E-08	-0.19967281	0.081	0.126	0.0013815	1.2
PARN	5.98E-08	-0.110686046	0.054	0.093	0.0014432	1.2
RAD21	5.99E-08	-0.214837835	0.309	0.374	0.0014452	1.2
SNX10	6.01E-08	-0.110466815	0.029	0.06	0.00145	1.2
FAM213A	6.16E-08	-0.103955613	0.05	0.087	0.0014863	1.2
HEY2	6.16E-08	-0.11069668	0.015	0.04	0.0014865	1.2
IAH1	6.19E-08	0.215061158	0.171	0.127	0.001493	1.2
AC097724.	6.28E-08	-0.124425828	0.056	0.095	0.001515	1.2
NEK10	6.45E-08	0.198767065	0.154	0.111	0.0015551	1.2
CDC26	6.52E-08	-0.121773514	0.173	0.235	0.0015716	1.2
ZNF235	6.55E-08	-0.123047695	0.047	0.084	0.0015783	1.2
VASP	6.62E-08	0.153300657	0.065	0.038	0.001597	1.2
RERG	6.71E-08	-0.174410386	0.117	0.169	0.001617	1.2
TAF4B	6.77E-08	-0.135221757	0.041	0.075	0.0016313	1.2
GHITM	6.81E-08	-0.119425506	0.415	0.5	0.0016413	1.2
SHFM1	6.88E-08	-0.14767396	0.577	0.643	0.0016599	1.2
TMEM123	7.01E-08	-0.217056923	0.367	0.44	0.0016907	1.2
WDR33	7.07E-08	-0.145586013	0.258	0.328	0.0017052	1.2
LSM3	7.39E-08	-0.157588565	0.269	0.335	0.001781	1.2
TBC1D3P1-	7.43E-08	-0.169965515	0.72	0.76	0.0017912	1.2
ATF2	7.53E-08	-0.157043761	0.097	0.143	0.0018157	1.2
ROR1	7.57E-08	-0.147276128	0.018	0.044	0.0018259	1.2
SOD2	7.61E-08	-0.156183417	0.955	0.942	0.0018342	1.2
PERP	7.81E-08	0.223351055	0.364	0.318	0.0018831	1.2
CCDC82	7.83E-08	0.198475544	0.29	0.239	0.0018892	1.2
WAC	7.98E-08	-0.257829572	0.579	0.642	0.0019234	1.2
STIP1	7.99E-08	0.129278299	0.078	0.047	0.0019274	1.2
HGSNAT	8.05E-08	-0.131324929	0.125	0.179	0.0019399	1.2
TEAD2	8.05E-08	-0.100389573	0.045	0.081	0.0019417	1.2
PLCH1	8.07E-08	-0.101708697	0.018	0.044	0.0019465	1.2
ADGRL3-AS	8.20E-08	0.198482887	0.68	0.662	0.0019782	1.2
DCAF5	8.36E-08	-0.125628149	0.088	0.134	0.0020147	1.2

CCL20	8.42E-08	0.907081571	0.338	0.293	0.0020308	1.2
EIF5B	8.45E-08	0.255218269	0.256	0.212	0.0020385	1.2
S100A13	8.52E-08	-0.146766527	0.546	0.622	0.0020543	1.2
EMC2	8.60E-08	-0.116840267	0.152	0.209	0.0020734	1.2
PDK1	8.60E-08	-0.212884208	0.085	0.128	0.0020747	1.2
MTX2	8.90E-08	-0.101759123	0.043	0.078	0.0021467	1.2
ZBTB20	9.04E-08	-0.183722366	0.423	0.498	0.0021807	1.2
DUSP6	9.18E-08	-0.105394478	0.023	0.051	0.0022125	1.2
NEO1	9.25E-08	-0.132762946	0.055	0.093	0.0022307	1.2
HPS5	9.47E-08	-0.142591821	0.178	0.238	0.0022839	1.2
ESRRG	9.63E-08	-0.103822501	0.014	0.038	0.0023231	1.2
RPS18	9.72E-08	-0.121208082	0.613	0.695	0.0023437	1.2
TPM3	9.84E-08	0.163631137	0.142	0.102	0.0023723	1.2
RP11-318C	9.88E-08	-0.10095925	0.024	0.053	0.002382	1.2
AKR1B1	1.01E-07	-0.183765278	0.044	0.079	0.0024375	1.2
EPB42	1.01E-07	0.208952806	0.923	0.928	0.0024406	1.2
DLG2	1.02E-07	-0.172774057	0.126	0.179	0.0024683	1.2
FTL	1.03E-07	0.758647975	0.541	0.535	0.0024811	1.2
GANAB	1.03E-07	-0.121203632	0.07	0.111	0.0024854	1.2
PVT1	1.03E-07	0.319406513	0.422	0.381	0.0024944	1.2
HDAC9	1.04E-07	0.234834885	0.274	0.224	0.0025166	1.2
GBF1	1.05E-07	-0.127790372	0.164	0.225	0.0025363	1.2
IRF2BP2	1.12E-07	-0.1432273	0.096	0.143	0.0026972	1.2
MID1	1.13E-07	-0.152627981	0.085	0.129	0.0027289	1.2
ORC3	1.15E-07	-0.106997158	0.052	0.09	0.0027707	1.2
HNRNPM	1.17E-07	-0.194105561	0.153	0.206	0.0028092	1.2
ATP6V1D	1.19E-07	0.274960369	0.326	0.283	0.0028577	1.2
RPL5	1.21E-07	-0.117152742	0.977	0.973	0.0029214	1.2
FAM172A	1.22E-07	0.207603844	0.755	0.746	0.0029514	1.2
NAA25	1.23E-07	-0.245823751	0.268	0.332	0.0029645	1.2
ATXN3	1.26E-07	-0.149144046	0.127	0.178	0.0030335	1.2
PIGR	1.27E-07	-0.302071983	0.467	0.533	0.0030625	1.2
UQCRHL	1.29E-07	-0.14905157	0.229	0.294	0.0030996	1.2
FRYL	1.31E-07	0.220754073	0.312	0.263	0.0031665	1.2
TAX1BP1	1.34E-07	0.161608154	0.679	0.666	0.0032198	1.2
GSK3B	1.34E-07	0.260376884	0.331	0.287	0.0032213	1.2
ACBD6	1.34E-07	-0.122238733	0.044	0.078	0.003243	1.2
RPS2	1.35E-07	-0.145329749	0.322	0.393	0.003249	1.2
KIF13B	1.35E-07	0.205617742	0.336	0.283	0.0032593	1.2
ACOT9	1.37E-07	0.166371156	0.11	0.075	0.0032923	1.2
ASCC3	1.39E-07	-0.148567765	0.163	0.222	0.0033618	1.2
PDIA3	1.40E-07	-0.168603336	0.22	0.285	0.003381	1.2
PPP2R1B	1.42E-07	-0.139717969	0.071	0.112	0.003415	1.2
SMARCA4	1.44E-07	-0.110559087	0.105	0.153	0.0034757	1.2
KRT19	1.53E-07	0.274603162	0.359	0.305	0.0036844	1.2
METTL15	1.53E-07	-0.115322409	0.095	0.142	0.0036846	1.2
KLF10	1.53E-07	-0.142609962	0.074	0.116	0.0036892	1.2

MFSD1	1.55E-07	-0.102935317	0.032	0.062	0.0037419	1.2
WDR70	1.57E-07	-0.17153294	0.095	0.141	0.00379	1.2
MYH14	1.57E-07	0.181077683	0.205	0.16	0.0037942	1.2
CTC-490E2	1.59E-07	0.109962826	0.066	0.039	0.0038313	1.2
BDNF-AS	1.62E-07	-0.13839875	0.629	0.69	0.0039018	1.2
CRK	1.63E-07	0.166057005	0.163	0.121	0.0039414	1.2
NAV1	1.65E-07	0.12187513	0.065	0.038	0.0039675	1.2
HSD11B1	1.67E-07	-0.181366915	0.154	0.209	0.0040255	1.2
SECISBP2	1.70E-07	-0.108622445	0.051	0.088	0.0041074	1.2
NUTF2	1.74E-07	0.13830262	0.094	0.062	0.0041881	1.2
MIS18BP1	1.89E-07	-0.117220337	0.093	0.14	0.0045666	1.2
GRB2	1.92E-07	0.19005044	0.161	0.121	0.0046405	1.2
RBP1	1.94E-07	0.145023235	0.064	0.038	0.0046806	1.2
RP11-774D	1.95E-07	-0.116385235	0.036	0.068	0.0047123	1.2
CEBPB	1.96E-07	-0.133818719	0.108	0.156	0.0047381	1.2
EMSY	1.98E-07	-0.118654287	0.094	0.141	0.004774	1.2
ADAM17	1.98E-07	0.226223931	0.456	0.412	0.0047748	1.2
ATP6V1H	2.00E-07	-0.126959404	0.066	0.106	0.0048238	1.2
SAA4	2.06E-07	0.2841372	0.313	0.268	0.0049744	1.2
SPINK5	2.18E-07	-0.136995265	0.006	0.024	0.005248	1.2
CD83	2.23E-07	-0.177720079	0.069	0.108	0.0053684	1.2
RBMS1	2.23E-07	0.228344436	0.302	0.256	0.0053849	1.2
TBC1D8	2.26E-07	-0.197284971	0.352	0.421	0.0054541	1.2
SRSF2	2.32E-07	0.156015391	0.114	0.078	0.0055959	1.2
LGALS2	2.33E-07	-0.13779496	0.019	0.045	0.0056172	1.2
BZW1	2.34E-07	0.193749173	0.187	0.144	0.0056499	1.2
DAPL1	2.35E-07	-0.139388272	0.028	0.056	0.0056675	1.2
ANKRD37	2.42E-07	-0.126581588	0.04	0.073	0.0058324	1.2
AHI1	2.46E-07	-0.173467229	0.164	0.219	0.0059214	1.2
C10orf76	2.48E-07	-0.140047914	0.097	0.144	0.0059702	1.2
LSM14A	2.48E-07	-0.17399467	0.299	0.365	0.0059876	1.2
TMEM99	2.55E-07	-0.102971472	0.051	0.086	0.0061557	1.2
C9orf3	2.58E-07	-0.147038286	0.127	0.179	0.0062256	1.2
S100A7	2.60E-07	-0.496953042	0.012	0.034	0.0062743	1.2
CCPG1	2.60E-07	-0.103924846	0.04	0.073	0.0062786	1.2
TMEM45A	2.61E-07	-0.148954967	0.119	0.168	0.0062864	1.2
DPH5	2.69E-07	-0.107110484	0.056	0.092	0.0064812	1.2
SMURF1	2.70E-07	0.204337037	0.302	0.255	0.006505	1.2
SCLT1	2.70E-07	-0.113614436	0.026	0.053	0.0065054	1.2
SUSD6	2.73E-07	-0.133107382	0.173	0.231	0.0065827	1.2
PRKAG1	2.74E-07	-0.104909233	0.123	0.174	0.0066044	1.2
USP9X	2.75E-07	-0.158636478	0.275	0.343	0.0066285	1.2
MITD1	2.78E-07	-0.107191744	0.082	0.125	0.0067021	1.2
PICALM	2.82E-07	0.187633553	0.447	0.403	0.0068069	1.2
RFTN2	2.86E-07	-0.103894937	0.006	0.024	0.0069032	1.2
WRN	2.91E-07	-0.154422531	0.146	0.198	0.0070191	1.2
LINC00511	3.10E-07	-0.206588111	0.087	0.13	0.0074659	1.2

RP11-141M	3.13E-07	-0.146195928	0.042	0.074	0.007552	1.2
RAP1A	3.15E-07	0.196612952	0.247	0.201	0.0075851	1.2
CFL2	3.24E-07	0.196830669	0.148	0.109	0.0078115	1.2
MED13L	3.24E-07	-0.204416081	0.625	0.692	0.0078219	1.2
TNFAIP1	3.30E-07	0.138742115	0.105	0.072	0.0079581	1.2
JMY	3.32E-07	-0.134141257	0.095	0.139	0.0079959	1.2
SH3BP5	3.38E-07	-0.138371897	0.068	0.107	0.008145	1.2
ZFC3H1	3.45E-07	0.213784744	0.237	0.19	0.0083103	1.2
ZNF438	3.55E-07	-0.104059619	0.044	0.078	0.0085639	1.2
CNIH1	3.64E-07	-0.143931279	0.236	0.3	0.0087802	1.2
LYRM5	3.65E-07	-0.10259824	0.078	0.12	0.0087927	1.2
CABIN1	3.68E-07	-0.119876927	0.049	0.083	0.0088641	1.2
ACP1	3.76E-07	-0.122010354	0.347	0.421	0.0090598	1.2
MOB4	3.76E-07	-0.11911834	0.155	0.21	0.0090619	1.2
PFKP	3.81E-07	0.134089365	0.051	0.028	0.0091932	1.2
USP33	3.85E-07	-0.146518696	0.176	0.232	0.0092906	1.2
ARL15	3.91E-07	-0.158429316	0.167	0.221	0.0094235	1.2
SURF4	3.96E-07	0.131025311	0.068	0.041	0.0095501	1.2
ZNF652	4.03E-07	-0.153836575	0.522	0.593	0.0097286	1.2
TMEM30A	4.05E-07	0.194365672	0.19	0.148	0.0097566	1.2
PRELID3B	4.05E-07	0.201628391	0.318	0.275	0.0097597	1.2
AEBP2	4.06E-07	0.249364835	0.279	0.234	0.0097834	1.2
STX8	4.11E-07	-0.14530545	0.159	0.212	0.009914	1.2
MRPS31	4.11E-07	-0.113014658	0.084	0.126	0.0099194	1.2
KPNA4	4.15E-07	0.162303025	0.183	0.139	0.0100122	1.2
RANBP2	4.15E-07	-0.142428491	0.229	0.29	0.0100142	1.2
SERPINB9	4.28E-07	0.241398997	0.195	0.148	0.0103107	1.2
SLC15A4	4.44E-07	0.119689272	0.091	0.06	0.0107131	1.2
MAP7	4.48E-07	0.226995023	0.364	0.323	0.0107941	1.2
PHIP	4.49E-07	-0.115299884	0.361	0.437	0.0108276	1.2
BCOR	4.50E-07	0.206459576	0.47	0.414	0.01084	1.2
ETS1	4.60E-07	0.167329052	0.174	0.132	0.0110949	1.2
FRAS1	4.63E-07	0.209139054	0.088	0.058	0.0111743	1.2
BDP1	4.64E-07	-0.159247392	0.168	0.221	0.0111917	1.2
RPL22L1	4.71E-07	-0.114468414	0.501	0.579	0.011349	1.2
ARHGEF28	4.84E-07	-0.3935054	0.342	0.396	0.0116703	1.2
BCL2L14	4.99E-07	-0.134697196	0.111	0.157	0.0120361	1.2
LRRC75A	5.00E-07	-0.145355087	0.161	0.215	0.0120554	1.2
PIP5K1B	5.01E-07	-0.139531929	0.04	0.071	0.0120705	1.2
EXOSC8	5.16E-07	-0.120358397	0.112	0.16	0.0124499	1.2
RNF152	5.24E-07	-0.124152602	0.026	0.052	0.0126334	1.2
MRPL48	5.24E-07	-0.114267712	0.084	0.125	0.0126347	1.2
PLAU	5.26E-07	0.10879838	0.046	0.024	0.0126857	1.2
TMEM165	5.32E-07	0.199682471	0.51	0.467	0.0128394	1.2
CIR1	5.35E-07	-0.114433474	0.246	0.31	0.0129076	1.2
NBEA	5.39E-07	-0.151472055	0.079	0.119	0.0129851	1.2
AIMP1	5.44E-07	-0.156287591	0.368	0.436	0.0131069	1.2

GPM6B	5.45E-07	0.202141308	0.242	0.196	0.0131534	1.2
RAPGEF2	5.54E-07	0.184888125	0.442	0.397	0.0133483	1.2
SIK2	5.70E-07	-0.203845091	0.281	0.339	0.0137526	1.2
C14orf119	5.86E-07	0.214245895	0.228	0.186	0.014125	1.2
TGFBR2	5.93E-07	-0.175578546	0.093	0.136	0.014304	1.2
OTUD3	6.00E-07	-0.132326431	0.027	0.054	0.014476	1.2
SCAF8	6.10E-07	-0.143426779	0.187	0.244	0.0147166	1.2
GAPDH	6.11E-07	-0.121400663	0.543	0.622	0.0147428	1.2
ASCC1	6.14E-07	-0.114886624	0.133	0.183	0.0148048	1.2
FUT8	6.15E-07	-0.138135831	0.064	0.101	0.0148339	1.2
PELI1	6.19E-07	-0.177806295	0.399	0.47	0.0149333	1.2
LRBA	6.26E-07	-0.11615203	0.398	0.48	0.0151027	1.2
IDO1	6.27E-07	-0.192234369	0.02	0.044	0.0151198	1.2
HAX1	6.33E-07	-0.1124544	0.279	0.348	0.0152577	1.2
CEP57	6.35E-07	-0.115932879	0.075	0.115	0.0153222	1.2
CDH3	6.42E-07	0.18811554	0.18	0.138	0.0154896	1.2
UBE2E2	6.50E-07	0.174247683	0.438	0.392	0.015685	1.2
ZNRF2	6.59E-07	0.234679961	0.191	0.151	0.0158823	1.2
RBM39	6.60E-07	-0.133297252	0.401	0.476	0.0159195	1.2
KCNQ1	6.65E-07	-0.114131218	0.067	0.105	0.0160383	1.2
ROR2	6.76E-07	-0.190752242	0.09	0.132	0.0162988	1.2
PFDN4	6.81E-07	-0.119249985	0.453	0.529	0.0164275	1.2
RLF	6.91E-07	-0.307911701	0.307	0.361	0.0166607	1.2
DAPK2	7.01E-07	0.153314812	0.48	0.436	0.0169057	1.2
BPTF	7.22E-07	-0.147020242	0.283	0.35	0.0174193	1.2
RGCC	7.41E-07	-0.12376424	0.032	0.06	0.0178727	1.2
VIM	7.62E-07	0.241200521	0.242	0.199	0.0183831	1.2
MFHAS1	7.69E-07	0.153644561	0.075	0.047	0.0185475	1.2
MOB1A	7.77E-07	0.131170899	0.108	0.075	0.0187457	1.2
ZDHHC13	7.79E-07	0.163175926	0.134	0.097	0.0187863	1.2
TTLL5	7.88E-07	-0.121052884	0.136	0.186	0.0190115	1.2
FBL	7.94E-07	-0.10956497	0.174	0.229	0.0191355	1.2
TATDN1	7.96E-07	-0.157443129	0.26	0.322	0.0192015	1.2
PARK7	7.99E-07	-0.121753624	0.419	0.494	0.0192562	1.2
VCP	8.27E-07	0.172056863	0.14	0.103	0.0199402	1.2
KCNQ5	8.32E-07	0.205594977	0.093	0.062	0.0200639	1.2
RP11-631N	8.47E-07	0.138204378	0.098	0.067	0.0204306	1.2
GS1-114I9.	8.60E-07	0.180853147	0.157	0.118	0.0207468	1.2
USP12	8.72E-07	0.163554311	0.161	0.121	0.0210216	1.2
SNX29	9.10E-07	-0.134390232	0.075	0.114	0.0219451	1.2
SUPT20H	9.12E-07	-0.102698105	0.055	0.09	0.0219806	1.2
CTTN	9.18E-07	0.178732571	0.209	0.165	0.0221243	1.2
KANK1	9.32E-07	0.209441088	0.252	0.208	0.0224779	1.2
ZSWIM6	9.46E-07	0.196243988	0.382	0.337	0.0228224	1.2
ROPN1	9.50E-07	-0.141323548	0.043	0.074	0.022903	1.2
SLC11A2	9.52E-07	-0.120385162	0.371	0.446	0.0229458	1.2
NSUN6	9.52E-07	-0.103194319	0.065	0.102	0.0229657	1.2

THAP6	9.58E-07	-0.106373088	0.047	0.079	0.0230999	1.2
ATAD2B	9.64E-07	-0.112244239	0.115	0.162	0.0232552	1.2
RP11-711K	9.72E-07	-0.122248747	0.038	0.068	0.0234289	1.2
CAMSAP1	9.80E-07	0.127505646	0.102	0.07	0.0236223	1.2
RAB3GAP2	1.04E-06	-0.131346976	0.145	0.196	0.0249824	1.2
MAPK14	1.05E-06	-0.15471932	0.14	0.189	0.025305	1.2
PTEN	1.06E-06	0.185234916	0.851	0.85	0.0255575	1.2
ARHGAP5	1.07E-06	0.229812803	0.419	0.381	0.0257254	1.2
ELF2	1.07E-06	-0.138221317	0.273	0.338	0.0257751	1.2
ARHGEF38	1.08E-06	-0.118955914	0.424	0.5	0.0259963	1.2
HNRNPAB	1.08E-06	0.164994379	0.107	0.075	0.0260161	1.2
PIGP	1.09E-06	-0.126644128	0.124	0.171	0.0262368	1.2
ZDHHC9	1.10E-06	0.152026097	0.161	0.122	0.0264937	1.2
PHC3	1.14E-06	-0.114301012	0.145	0.195	0.0275979	1.2
UBR1	1.16E-06	-0.107026375	0.096	0.139	0.0280572	1.2
SCRN1	1.17E-06	0.116372066	0.088	0.058	0.0282162	1.2
CACUL1	1.19E-06	-0.140716082	0.344	0.417	0.0286079	1.2
MYC	1.20E-06	0.179768958	0.181	0.139	0.028878	1.2
VRK2	1.22E-06	-0.10957977	0.141	0.192	0.0293397	1.2
LINC01492	1.22E-06	0.175547098	0.162	0.124	0.0293504	1.2
CAPZB	1.22E-06	0.198914196	0.171	0.133	0.0294972	1.2
ZNF385D	1.22E-06	-0.134341431	0.042	0.073	0.0295365	1.2
HLCS	1.24E-06	-0.108213664	0.058	0.093	0.0299285	1.2
PPP2R5E	1.27E-06	-0.180374245	0.23	0.287	0.030616	1.2
RP11-293M	1.28E-06	-0.121745142	0.035	0.064	0.0309004	1.2
STEAP1B	1.28E-06	0.189328408	0.947	0.955	0.0309704	1.2
AMD1	1.31E-06	0.19096636	0.277	0.232	0.0316689	1.2
CATSPERB	1.33E-06	-0.131468402	0.201	0.259	0.0319575	1.2
ZNF165	1.33E-06	0.156694363	0.086	0.057	0.0319863	1.2
RASAL2	1.44E-06	0.11975881	0.57	0.534	0.0347482	1.2
GLIS3	1.46E-06	0.292752017	0.321	0.278	0.0351591	1.2
HADHB	1.46E-06	-0.115784323	0.24	0.305	0.035306	1.2
LURAP1L-A	1.49E-06	0.126911595	0.118	0.084	0.0358306	1.2
PTAR1	1.51E-06	-0.121479058	0.067	0.103	0.0364863	1.2
CNOT6L	1.52E-06	-0.110382338	0.058	0.093	0.0367511	1.2
RALGAPA2	1.60E-06	-0.149905799	0.151	0.202	0.0386002	1.2
RP11-368L	1.62E-06	0.124084942	0.104	0.072	0.0390103	1.2
KCNH8	1.64E-06	0.148084863	0.061	0.037	0.0395479	1.2
MACF1	1.67E-06	0.21786585	0.641	0.632	0.0402803	1.2
TNRC18	1.73E-06	-0.101761973	0.038	0.067	0.041708	1.2
ZC3HAV1	1.77E-06	0.16041627	0.142	0.106	0.0426259	1.2
SFT2D2	1.80E-06	-0.11004835	0.183	0.238	0.0433711	1.2
KIDINS220	1.85E-06	-0.115622361	0.139	0.189	0.0446364	1.2
RBM47	1.86E-06	-0.137604039	0.541	0.604	0.0448099	1.2
ENO1	1.89E-06	-0.100204139	0.462	0.531	0.0455711	1.2
MRPL51	2.02E-06	-0.126682037	0.37	0.441	0.0487086	1.2
PIKFYVE	2.02E-06	-0.124116682	0.073	0.109	0.0488009	1.2

TMEM184E	2.02E-06	0.166606927	0.165	0.126	0.0488274	1.2
KIF9-AS1	2.04E-06	-0.10451954	0.028	0.054	0.0491712	1.2
SQSTM1	2.05E-06	-0.130249123	0.234	0.294	0.0494717	1.2
BARD1	2.09E-06	0.136918301	0.1	0.069	0.0504524	1.2
FRK	2.10E-06	0.192804936	0.198	0.158	0.0507339	1.2
KIF13A	2.11E-06	-0.207508223	0.229	0.283	0.0507817	1.2
GBP2	2.11E-06	0.176436349	0.757	0.753	0.0508242	1.2
HNRNPLL	2.12E-06	-0.110721413	0.083	0.121	0.0510703	1.2
COL22A1	2.13E-06	0.103543497	0.051	0.03	0.0513215	1.2
MAPK6	2.14E-06	0.175876482	0.248	0.204	0.0515747	1.2
SGMS2	2.15E-06	0.163639189	0.213	0.169	0.0518532	1.2
GIGYF2	2.21E-06	-0.124591956	0.206	0.263	0.053393	1.2
TOB1	2.22E-06	-0.129205844	0.066	0.101	0.0535284	1.2
CPSF6	2.24E-06	0.157631395	0.167	0.129	0.0539574	1.2
TPTEP1	2.28E-06	0.200101065	0.266	0.223	0.055092	1.2
GS1-24F4.2	2.32E-06	-0.15286133	0.078	0.117	0.0559145	1.2
CEP63	2.33E-06	-0.10117435	0.057	0.091	0.0561339	1.2
PLEKHA8	2.34E-06	0.121048511	0.099	0.068	0.056432	1.2
PPP4R3A	2.35E-06	-0.117059947	0.125	0.169	0.0566503	1.2
RBL2	2.37E-06	-0.122502526	0.069	0.105	0.0571343	1.2
USP16	2.39E-06	-0.115518568	0.156	0.206	0.0576814	1.2
CSNK1E	2.44E-06	0.24603808	0.291	0.251	0.0588191	1.2
LRRC28	2.45E-06	-0.114885212	0.046	0.076	0.0590104	1.2
CDC42EP4	2.45E-06	-0.109371458	0.049	0.081	0.0590107	1.2
BTBD10	2.49E-06	0.145320708	0.086	0.058	0.0600403	1.2
FANK1	2.52E-06	0.151721619	0.096	0.066	0.0606442	1.2
IGFBP7	2.54E-06	-0.10352434	0.01	0.028	0.0613237	1.2
POLR1D	2.55E-06	-0.127184714	0.267	0.329	0.0615134	1.2
RPL13	2.64E-06	-0.130763249	0.445	0.515	0.0635853	1.2
SLC25A36	2.71E-06	-0.106105848	0.137	0.185	0.065424	1.2
KIAA1468	2.75E-06	-0.110295653	0.08	0.119	0.0662205	1.2
STAG1	2.77E-06	0.172942607	0.504	0.474	0.0669057	1.2
UBR3	2.79E-06	-0.131982722	0.162	0.212	0.0671721	1.2
FARP2	2.84E-06	0.134552352	0.107	0.075	0.0685162	1.2
POLG2	2.91E-06	-0.128418372	0.052	0.083	0.0701626	1.2
EML5	2.93E-06	-0.10511864	0.079	0.117	0.0707442	1.2
ARID4A	2.97E-06	-0.120648221	0.137	0.183	0.071624	1.2
HSPD1	3.03E-06	-0.131082821	0.286	0.351	0.0731305	1.2
FBXW4	3.04E-06	-0.101678448	0.053	0.085	0.073325	1.2
PPP1R13L	3.06E-06	0.156099338	0.132	0.097	0.0737441	1.2
FAM78B	3.07E-06	0.183935414	0.097	0.067	0.0740126	1.2
AZGP1	3.11E-06	-0.239431246	0.296	0.354	0.0749172	1.2
EIF2B3	3.11E-06	-0.128671434	0.05	0.08	0.0749902	1.2
TMEM51	3.16E-06	0.165616292	0.206	0.166	0.0761603	1.2
COPB1	3.21E-06	0.181073074	0.244	0.204	0.0773379	1.2
EIF3M	3.26E-06	-0.128634083	0.433	0.502	0.0786609	1.2
TLR2	3.30E-06	-0.110149584	0.211	0.27	0.0794918	1.2

LINC00475	3.37E-06	0.18728031	0.11	0.079	0.0813173	1.2
NLK	3.38E-06	0.174994405	0.128	0.095	0.0815998	1.2
BBC3	3.48E-06	0.105982846	0.091	0.062	0.0838618	1.2
HSPH1	3.52E-06	0.213404213	0.151	0.115	0.0848646	1.2
SPOPL	3.55E-06	-0.111143722	0.116	0.16	0.0857043	1.2
RPS25	3.56E-06	-0.138926619	0.851	0.87	0.0857651	1.2
CPAMD8	3.67E-06	0.208830033	0.146	0.11	0.0885691	1.2
KRAS	3.68E-06	0.144835633	0.174	0.134	0.0886212	1.2
MPRIP-AS1	3.83E-06	0.11343397	0.077	0.05	0.0923595	1.2
PEX13	4.00E-06	-0.116109193	0.09	0.129	0.0963794	1.2
CTBP2	4.07E-06	0.160257346	0.347	0.303	0.0981646	1.2
TIPARP	4.09E-06	0.135182489	0.127	0.093	0.0987231	1.2
ZNF22	4.15E-06	-0.101081228	0.192	0.247	0.0999624	1.2
ARHGEF2	4.36E-06	0.105979093	0.106	0.075	0.1051852	1.2
SPTAN1	4.37E-06	0.185691527	0.213	0.173	0.10545	1.2
OLFM4	4.39E-06	-0.228083966	0.022	0.045	0.1057867	1.2
HNRNPA2B	4.40E-06	-0.117844269	0.368	0.437	0.1061436	1.2
TBC1D9	4.42E-06	-0.124659018	0.184	0.236	0.1066257	1.2
CCDC109B	4.46E-06	0.151482481	0.082	0.055	0.1075278	1.2
DRG1	4.47E-06	-0.112026929	0.187	0.24	0.1078579	1.2
SLC25A6	4.63E-06	-0.143543998	0.107	0.148	0.1115515	1.2
RP11-231C	4.66E-06	0.163898962	0.226	0.184	0.1124406	1.2
BAG5	4.70E-06	0.14598833	0.145	0.109	0.1134234	1.2
ZZZ3	4.76E-06	-0.122862173	0.14	0.188	0.1147675	1.2
RAP1GAP2	4.76E-06	-0.174498922	0.133	0.177	0.1147868	1.2
MAP7D1	4.87E-06	0.111606207	0.163	0.124	0.1175176	1.2
CTD-2337A	4.88E-06	-0.158988249	0.154	0.201	0.1176778	1.2
SNRPD2	5.01E-06	-0.134985637	0.706	0.756	0.1207137	1.2
TYW3	5.03E-06	-0.110875809	0.055	0.086	0.1212261	1.2
REEP3	5.03E-06	0.19380278	0.279	0.236	0.12123	1.2
TTLL4	5.06E-06	-0.11291241	0.135	0.18	0.1219827	1.2
PDE4D	5.06E-06	-0.137251571	0.078	0.114	0.1220437	1.2
EIF4E	5.10E-06	0.170877588	0.415	0.376	0.1229524	1.2
RPS9	5.11E-06	-0.112887615	0.251	0.312	0.1233241	1.2
WDFY2	5.12E-06	-0.113284132	0.157	0.206	0.1235417	1.2
CTD-3088G	5.16E-06	0.190333612	0.074	0.049	0.1244651	1.2
BCL2L11	5.20E-06	-0.109208503	0.036	0.063	0.1253147	1.2
ZNF708	5.21E-06	-0.149538232	0.064	0.098	0.1256319	1.2
SC5D	5.24E-06	-0.100624551	0.066	0.1	0.126253	1.2
PHF14	5.24E-06	-0.118933739	0.112	0.154	0.1264346	1.2
LUC7L3	5.30E-06	0.185775688	0.333	0.289	0.1278966	1.2
FGGY	5.32E-06	-0.12745147	0.191	0.242	0.1283583	1.2
NDUFB2	5.33E-06	0.10136414	0.041	0.022	0.128522	1.2
IDE	5.34E-06	-0.104129412	0.052	0.083	0.1288121	1.2
MAP2K4	5.65E-06	0.224166562	0.397	0.359	0.1361606	1.2
SFPQ	5.71E-06	0.195747772	0.251	0.211	0.1377577	1.2
TMA7	5.73E-06	-0.106729779	0.534	0.6	0.1381027	1.2

CLIC6	6.14E-06	-0.117439633	0.081	0.118	0.147957	1.2
SENP6	6.25E-06	-0.126564568	0.285	0.346	0.1507373	1.2
SESN3	6.29E-06	-0.147594199	0.057	0.089	0.1516168	1.2
RBM5	6.31E-06	-0.111360051	0.124	0.168	0.1521165	1.2
CABLES1	6.88E-06	-0.101136358	0.044	0.073	0.1658145	1.2
RPL22	7.11E-06	-0.132536681	0.755	0.806	0.1714153	1.2
KMT2E	7.11E-06	-0.150692623	0.355	0.416	0.1715254	1.2
ACTN4	7.14E-06	0.268599784	0.367	0.326	0.1721607	1.2
HEATR5B	7.22E-06	-0.101464089	0.088	0.126	0.1741148	1.2
ZCCHC8	7.23E-06	-0.13809618	0.102	0.141	0.1744434	1.2
RPS6KA2	7.24E-06	0.113819738	0.132	0.099	0.1745548	1.2
KIAA0355	7.26E-06	0.157097136	0.16	0.124	0.1749915	1.2
FGD4	7.26E-06	0.193512782	0.3	0.257	0.1750224	1.2
AK6	7.27E-06	0.172151473	0.239	0.197	0.1751855	1.2
SPARCL1	7.42E-06	-0.109556221	0.052	0.082	0.1789582	1.2
PABPC1	7.75E-06	0.152743198	0.796	0.787	0.1868537	1.2
BNIP2	7.85E-06	0.172561945	0.142	0.109	0.1892695	1.2
MTMR2	8.00E-06	-0.102852958	0.082	0.119	0.1928149	1.2
SGCD	8.02E-06	-0.127045887	0.037	0.064	0.1934573	1.2
DOCK5	8.04E-06	0.200339117	0.251	0.21	0.1938843	1.2
HEBP2	8.64E-06	0.172839649	0.205	0.167	0.2082867	1.2
CH17-189H	8.65E-06	-0.110022251	0.1	0.14	0.2084958	1.2
GNL3	9.17E-06	0.197725505	0.305	0.269	0.2210687	1.2
TMED5	9.19E-06	0.153918282	0.131	0.098	0.2216186	1.2
TTC37	9.19E-06	-0.108803697	0.13	0.174	0.2216294	1.2
REL	9.35E-06	-0.133709641	0.199	0.249	0.225574	1.2
SPIN1	9.38E-06	-0.127469134	0.152	0.197	0.2261511	1.2
GJA1	9.48E-06	0.127127648	0.078	0.052	0.2285004	1.2
CTNND1	9.80E-06	0.174941106	0.347	0.311	0.2362289	1.2
DCLK2	9.88E-06	-0.115324619	0.022	0.043	0.2382905	1.2
OXSR1	1.01E-05	0.141580513	0.267	0.224	0.2430349	1.2
FAM177B	1.02E-05	0.158825347	0.667	0.635	0.2450714	1.2
CAPZA1	1.03E-05	0.182056979	0.471	0.439	0.2483828	1.2
TCAF2	1.04E-05	-0.112531723	0.104	0.143	0.2499509	1.2
CLDND1	1.05E-05	0.183476929	0.245	0.206	0.2523367	1.2
CTNNAL1	1.07E-05	-0.100412793	0.079	0.115	0.2581704	1.2
CAPRIN1	1.10E-05	-0.11974567	0.196	0.246	0.2645465	1.2
TIAM2	1.12E-05	0.222283113	0.272	0.236	0.2693424	1.2
STRBP	1.12E-05	-0.120383253	0.154	0.201	0.2698078	1.2
YARS	1.14E-05	0.117747045	0.315	0.271	0.2739145	1.2
DIP2A	1.15E-05	0.14163834	0.127	0.095	0.2771025	1.2
RAB22A	1.16E-05	0.156744081	0.116	0.086	0.2796509	1.2
LIPG	1.16E-05	0.117547163	0.046	0.027	0.280384	1.2
HAT1	1.18E-05	-0.105487667	0.133	0.176	0.2833754	1.2
ACACA	1.21E-05	-0.103245075	0.121	0.164	0.2913978	1.2
11-Sep	1.22E-05	0.158813355	0.145	0.112	0.2951133	1.2
ZNF277	1.27E-05	-0.106116991	0.174	0.221	0.3056971	1.2

MECOM	1.27E-05	-0.108913135	0.863	0.896	0.3062574	1.2
SNHG12	1.27E-05	0.109375817	0.089	0.062	0.3067816	1.2
RBMX	1.28E-05	-0.106283329	0.204	0.257	0.309377	1.2
DOPEY1	1.29E-05	-0.102911514	0.095	0.133	0.3110539	1.2
BCAR3	1.30E-05	0.185382594	0.116	0.086	0.3132971	1.2
TJP1	1.35E-05	0.185764652	0.417	0.382	0.3262234	1.2
ELP2	1.36E-05	-0.114674283	0.134	0.177	0.3275095	1.2
RBM8A	1.36E-05	0.18536813	0.529	0.506	0.3280268	1.2
RP11-277P	1.37E-05	0.180191562	0.092	0.065	0.3311099	1.2
TACC1	1.38E-05	-0.189040487	0.198	0.246	0.3316661	1.2
NIN	1.39E-05	-0.116126734	0.076	0.11	0.3358352	1.2
TAF15	1.40E-05	-0.108516786	0.272	0.331	0.3363807	1.2
RARS	1.40E-05	-0.112821044	0.243	0.299	0.3364323	1.2
GPBP1	1.40E-05	-0.186620858	0.571	0.63	0.3368478	1.2
PCDH11X	1.43E-05	0.174134706	0.068	0.045	0.3452439	1.2
CKAP4	1.43E-05	-0.101495399	0.133	0.175	0.3454639	1.2
YWHAG	1.55E-05	0.156568147	0.263	0.223	0.3725963	1.2
C11orf1	1.55E-05	-0.115597508	0.146	0.19	0.3727336	1.2
STK39	1.57E-05	-0.349550558	0.103	0.138	0.3775105	1.2
FER	1.62E-05	0.187835073	0.243	0.206	0.3907467	1.2
HELZ	1.66E-05	-0.185350934	0.287	0.336	0.4000794	1.2
CCDC146	1.69E-05	0.185820584	0.216	0.178	0.4078826	1.2
SNX2	1.72E-05	-0.1093205	0.16	0.206	0.4149769	1.2
GAB2	1.73E-05	0.198216275	0.25	0.213	0.4177813	1.2
PVRL2	1.74E-05	0.16334793	0.206	0.168	0.4191478	1.2
PSMA4	1.74E-05	-0.157971742	0.45	0.516	0.420319	1.2
VOPP1	1.75E-05	0.109924902	0.087	0.06	0.4231467	1.2
PRSS8	1.81E-05	0.129946059	0.081	0.056	0.4362292	1.2
CLEC2D	1.84E-05	-0.104833484	0.089	0.124	0.4435262	1.2
SLC39A11	1.89E-05	-0.134280987	0.054	0.083	0.454874	1.2
LMO7	1.91E-05	0.129998548	0.099	0.071	0.4604526	1.2
PHF20	1.92E-05	0.205715395	0.309	0.272	0.4623473	1.2
NFX1	1.93E-05	-0.114695801	0.131	0.173	0.4652549	1.2
CHD9	1.93E-05	0.155198165	0.494	0.462	0.4665819	1.2
CLSTN3	2.02E-05	-0.107269565	0.035	0.06	0.4876415	1.2
DPP10	2.04E-05	-0.100441011	0.032	0.055	0.4926909	1.2
EIF2S3	2.04E-05	-0.11348617	0.345	0.409	0.4930708	1.2
PROM1	2.05E-05	-0.129482115	0.404	0.472	0.4937096	1.2
EIF2S2	2.06E-05	0.26262699	0.298	0.267	0.4959997	1.2
TNFRSF10D	2.06E-05	0.106550946	0.065	0.042	0.4972528	1.2
GALNT18	2.07E-05	0.120710099	0.07	0.047	0.4993658	1.2
USP31	2.09E-05	0.1443347	0.157	0.122	0.5038599	1.2
WDR19	2.22E-05	-0.107772075	0.055	0.084	0.534335	1.2
TBC1D1	2.27E-05	0.196389266	0.219	0.184	0.5469267	1.2
CBFA2T2	2.32E-05	-0.124854397	0.111	0.149	0.560576	1.2
TNFRSF8	2.35E-05	0.103264415	0.161	0.126	0.5677484	1.2
EIF4E2	2.35E-05	-0.109208084	0.347	0.412	0.5678353	1.2

MGST1	2.36E-05	-0.114030249	0.876	0.886	0.5701689	1.2
SERPINE2	2.37E-05	-0.104763816	0.034	0.058	0.5717889	1.2
NFIC	2.39E-05	-0.103584328	0.139	0.183	0.576724	1.2
RP11-273G	2.43E-05	0.165250669	0.094	0.068	0.5852634	1.2
IFIH1	2.44E-05	-0.10085574	0.074	0.107	0.5874019	1.2
MEGF11	2.46E-05	0.132620186	0.02	0.008	0.5921243	1.2
PRDX1	2.56E-05	0.186306369	0.732	0.714	0.6161021	1.2
LUZP1	2.56E-05	0.156740806	0.115	0.086	0.6182902	1.2
NCEH1	2.62E-05	-0.132497134	0.184	0.231	0.6321564	1.2
FBXL20	2.64E-05	-0.132151419	0.217	0.266	0.637191	1.2
RBM3	2.73E-05	-0.121203948	0.453	0.518	0.6573398	1.2
ALG13	2.75E-05	-0.108281107	0.076	0.108	0.6625382	1.2
SLC25A26	2.76E-05	-0.102104253	0.067	0.098	0.6649987	1.2
LRRC4C	2.82E-05	-0.109940015	0.038	0.063	0.6810394	1.2
LINGO2	2.84E-05	0.141496515	0.087	0.061	0.6851889	1.2
RP11-841O	2.88E-05	0.109058076	0.09	0.064	0.6941059	1.2
CUL3	2.92E-05	-0.10353318	0.3	0.36	0.7034254	1.2
LPIN1	3.01E-05	0.263709865	0.367	0.336	0.725961	1.2
SUMO1	3.03E-05	0.184310138	0.543	0.529	0.7297487	1.2
TTC39C	3.05E-05	-0.104033775	0.104	0.143	0.734523	1.2
RSL1D1	3.05E-05	-0.136624161	0.582	0.644	0.7356024	1.2
KAT6B	3.10E-05	-0.118376253	0.104	0.141	0.7475549	1.2
ACYP2	3.12E-05	-0.12365662	0.188	0.235	0.7522792	1.2
PPFIA1	3.25E-05	0.199799901	0.3	0.264	0.7828104	1.2
PTPRJ	3.30E-05	0.128653186	0.357	0.312	0.7955184	1.2
TMEM41B	3.32E-05	-0.123740087	0.163	0.207	0.8006384	1.2
CLOCK	3.37E-05	0.226978149	0.214	0.18	0.8118444	1.2
SNX8	3.38E-05	-0.112503316	0.056	0.084	0.8157462	1.2
CCDC91	3.38E-05	-0.109435113	0.311	0.368	0.8159528	1.2
UBASH3B	3.39E-05	0.12065124	0.083	0.058	0.8177548	1.2
LHFPL2	3.40E-05	-0.278737539	0.366	0.41	0.8199084	1.2
SYNPO2	3.41E-05	-0.16964158	0.169	0.213	0.8212402	1.2
LDHC	3.48E-05	-0.109032765	0.013	0.03	0.8400578	1.2
SPNS2	3.49E-05	-0.101737822	0.047	0.073	0.8407421	1.2
MIER1	3.53E-05	-0.15015256	0.153	0.194	0.8511179	1.2
PDP1	3.54E-05	0.152288398	0.11	0.082	0.8544306	1.2
SHROOM3	3.56E-05	-0.195235458	0.479	0.536	0.8577287	1.2
LAMP2	3.65E-05	0.16823978	0.279	0.245	0.879003	1.2
ERRFI1	3.70E-05	-0.122805728	0.544	0.621	0.8913674	1.2
MTR	3.79E-05	-0.102684425	0.049	0.076	0.9137693	1.2
ATP13A3	3.94E-05	0.181708696	0.372	0.335	0.9495047	1.2
IST1	3.98E-05	0.185615594	0.314	0.281	0.9598527	1.2
CENPW	4.00E-05	0.149786894	0.154	0.122	0.9635118	1.2
SENP7	4.01E-05	-0.116537583	0.095	0.129	0.9662476	1.2
PLEKHH2	4.14E-05	0.123101272	0.155	0.122	0.9984827	1.2
ZNF267	4.22E-05	0.143555356	0.205	0.169	1	1.2
SPAG1	4.32E-05	0.203408281	0.18	0.146	1	1.2

PCNP	4.32E-05	0.173804084	0.395	0.364	1	1.2
GPR75-ASB	4.42E-05	0.248451654	0.174	0.141	1	1.2
RP11-431K	4.54E-05	-0.133563448	0.035	0.058	1	1.2
SLC1A5	4.58E-05	0.10091214	0.063	0.042	1	1.2
PELI2	4.65E-05	-0.117824206	0.066	0.096	1	1.2
GRPEL1	4.70E-05	0.109380177	0.08	0.056	1	1.2
MAGI2	4.81E-05	-0.142856896	0.072	0.103	1	1.2
SGK223	4.81E-05	-0.103553937	0.056	0.084	1	1.2
TNFRSF10A	4.86E-05	0.150124021	0.096	0.07	1	1.2
IQGAP1	4.96E-05	0.15129918	0.461	0.426	1	1.2
FARS2	4.97E-05	-0.106353404	0.061	0.09	1	1.2
OPHN1	5.10E-05	0.219174761	0.25	0.217	1	1.2
ERN1	5.29E-05	0.154242188	0.167	0.135	1	1.2
UHRF1BP1I	5.42E-05	-0.106319405	0.161	0.204	1	1.2
OSBPL10	5.47E-05	0.140416307	0.16	0.127	1	1.2
CDV3	5.63E-05	0.13555099	0.129	0.1	1	1.2
NDRG2	5.66E-05	0.228607036	0.283	0.248	1	1.2
C14orf2	5.81E-05	-0.101068177	0.671	0.73	1	1.2
ARHGAP12	5.95E-05	0.1763968	0.367	0.332	1	1.2
MGLL	6.09E-05	0.120039688	0.086	0.061	1	1.2
ACTN1	6.12E-05	0.106468004	0.309	0.266	1	1.2
PTCHD1	6.20E-05	0.123423345	0.105	0.078	1	1.2
PLCG2	6.52E-05	-0.100393215	0.046	0.071	1	1.2
CTSB	6.54E-05	-0.119225702	0.266	0.314	1	1.2
SCARB1	6.63E-05	-0.105861891	0.033	0.054	1	1.2
CA12	6.66E-05	0.206637865	0.262	0.224	1	1.2
C5orf56	6.80E-05	-0.136098488	0.099	0.132	1	1.2
TGFA	6.82E-05	0.120736921	0.114	0.086	1	1.2
CDKL5	7.46E-05	0.181044126	0.109	0.082	1	1.2
ASNS	7.56E-05	0.118037175	0.103	0.076	1	1.2
STK38L	7.71E-05	0.159174317	0.168	0.135	1	1.2
ERGIC1	7.75E-05	0.152696203	0.118	0.09	1	1.2
IRAK2	7.78E-05	-0.113315025	0.113	0.15	1	1.2
STK24	7.84E-05	0.137875933	0.168	0.136	1	1.2
RNF114	7.93E-05	0.146325802	0.173	0.141	1	1.2
RHOC	7.94E-05	0.153767345	0.072	0.05	1	1.2
SUZ12	7.99E-05	-0.11154728	0.152	0.192	1	1.2
TCN1	7.99E-05	-0.133896894	0.021	0.04	1	1.2
SERPINB1	8.23E-05	0.156410351	0.127	0.099	1	1.2
TCF12	8.28E-05	-0.152648324	0.451	0.51	1	1.2
RSBN1L	8.34E-05	-0.142636739	0.097	0.13	1	1.2
ELF3	8.44E-05	-0.16682916	0.488	0.551	1	1.2
OSBPL9	8.63E-05	-0.316119027	0.426	0.473	1	1.2
PMAIP1	8.77E-05	0.109885558	0.108	0.081	1	1.2
LINC01420	8.96E-05	0.101370415	0.318	0.383	1	1.2
STMN1	9.02E-05	0.194390958	0.219	0.188	1	1.2
RFWD2	9.07E-05	0.162391975	0.318	0.287	1	1.2

GLRX	9.08E-05	0.439180391	0.503	0.497	1	1.2
SLC39A10	9.14E-05	0.141061465	0.131	0.102	1	1.2
NACA	9.19E-05	-0.100300884	0.968	0.961	1	1.2
BIRC2	9.23E-05	0.150534462	0.173	0.141	1	1.2
OTUD7A	9.79E-05	0.109157162	0.085	0.061	1	1.2
SLC41A2	0.000101	0.112253078	0.078	0.056	1	1.2
STRN	0.000102	-0.184977529	0.357	0.409	1	1.2
PNISR	0.000102	-0.113953361	0.737	0.773	1	1.2
LAMTOR5	0.000104	0.142914508	0.623	0.607	1	1.2
MT1E	0.000104	-0.351269741	0.026	0.045	1	1.2
TLE1	0.000105	0.179629753	0.254	0.22	1	1.2
HNRNPA3	0.000105	-0.111491209	0.299	0.353	1	1.2
NEB	0.000106	0.101409879	0.043	0.027	1	1.2
POLD3	0.000106	-0.105090046	0.057	0.083	1	1.2
DST	0.000109	-0.137421168	0.548	0.607	1	1.2
RHOQ	0.00011	0.157242342	0.325	0.291	1	1.2
REPS1	0.000112	-0.118506642	0.232	0.279	1	1.2
DNTTIP1	0.000116	0.1125211	0.088	0.064	1	1.2
RP1-313I6.	0.000117	0.119013637	0.082	0.059	1	1.2
HLA-B	0.000117	0.168499079	0.636	0.638	1	1.2
AKIRIN1	0.000118	0.121166083	0.123	0.095	1	1.2
RNF19A	0.000118	0.257087617	0.621	0.626	1	1.2
ZNF791	0.00012	0.133986007	0.134	0.105	1	1.2
CDC42BPA	0.00012	0.171002549	0.31	0.28	1	1.2
EIF4EBP2	0.000121	0.159145477	0.14	0.112	1	1.2
PCMT1	0.000129	0.144275775	0.13	0.103	1	1.2
RIC3	0.000131	0.240582579	0.11	0.084	1	1.2
LPIN2	0.000131	0.139848726	0.121	0.093	1	1.2
SMIM14	0.000131	-0.102748632	0.127	0.164	1	1.2
BCCIP	0.000131	0.117530826	0.111	0.084	1	1.2
EXT1	0.000132	0.242701008	0.571	0.56	1	1.2
ZC3H11A	0.000134	0.151394794	0.269	0.233	1	1.2
PLIN2	0.000136	-0.103320628	0.125	0.161	1	1.2
UBC	0.000147	-0.601117359	0.949	0.95	1	1.2
RNF19B	0.00015	0.205168819	0.196	0.165	1	1.2
MEX3A	0.000151	0.104703867	0.075	0.053	1	1.2
CCT5	0.000153	0.248084052	0.391	0.371	1	1.2
SLCO3A1	0.000164	0.208340974	0.28	0.247	1	1.2
AC008074.	0.000164	0.137743493	0.101	0.076	1	1.2
PRKX	0.000164	0.170830645	0.2	0.169	1	1.2
SCGB2A1	0.000165	0.296628633	0.059	0.04	1	1.2
PRMT1	0.000165	0.154101459	0.044	0.027	1	1.2
BAZ2B	0.000168	-0.1008205	0.391	0.455	1	1.2
ASAP2	0.000172	0.126901909	0.081	0.059	1	1.2
CPD	0.000177	-0.129222469	0.189	0.23	1	1.2
NUFIP2	0.000188	0.167247367	0.236	0.205	1	1.2
GABARAP	0.000189	0.162880798	0.33	0.297	1	1.2

RP11-530C	0.000189	0.105296753	0.057	0.038	1	1.2
ARPC5	0.000189	0.135931331	0.123	0.097	1	1.2
HNRNPU	0.000191	-0.119377551	0.08	0.109	1	1.2
SRRM2	0.000192	-0.10410475	0.168	0.209	1	1.2
NARS	0.000193	0.208819452	0.31	0.284	1	1.2
MPP5	0.000198	0.13647034	0.269	0.235	1	1.2
AC026202.	0.000202	-0.12724148	0.166	0.205	1	1.2
SETD5	0.000204	-0.13195336	0.472	0.532	1	1.2
ARFGAP3	0.000206	0.149633382	0.206	0.175	1	1.2
SMYD2	0.000206	0.128915126	0.069	0.049	1	1.2
CXCL2	0.000213	-0.161249489	0.223	0.268	1	1.2
TMSB10	0.000221	0.372783036	0.619	0.63	1	1.2
MAPKBP1	0.000221	0.13512438	0.117	0.091	1	1.2
YWHAQ	0.000228	0.12487685	0.4	0.368	1	1.2
PSMD12	0.000233	0.145631312	0.145	0.116	1	1.2
FSTL1	0.000234	0.123190545	0.136	0.109	1	1.2
SOS2	0.000235	0.183340022	0.483	0.465	1	1.2
SERINC1	0.00024	0.177103274	0.329	0.301	1	1.2
EIF4B	0.00024	-0.100826057	0.436	0.5	1	1.2
PIN4	0.000243	-0.1001041	0.255	0.302	1	1.2
SLC38A2	0.00025	0.159429725	0.2	0.169	1	1.2
FRMD5	0.000252	0.243891528	0.192	0.163	1	1.2
PTMA	0.000255	-0.156660237	0.544	0.599	1	1.2
MAD1L1	0.000259	0.113384758	0.076	0.055	1	1.2
IGF1R	0.000267	-0.114485637	0.372	0.426	1	1.2
NLRC5	0.000274	0.104205346	0.052	0.035	1	1.2
FHL2	0.000275	0.110306001	0.1	0.076	1	1.2
MAPRE1	0.000282	0.132211557	0.182	0.151	1	1.2
FOXK2	0.000285	-0.107205815	0.264	0.312	1	1.2
UBR2	0.000287	-0.124964114	0.278	0.328	1	1.2
GGACT	0.000297	0.155061379	0.193	0.163	1	1.2
LCN2	0.000306	-0.120127071	0.023	0.04	1	1.2
ACSS1	0.000306	-0.124100636	0.007	0.019	1	1.2
TMED9	0.000314	0.103745878	0.093	0.07	1	1.2
PCBP1	0.000315	-0.124453806	0.435	0.491	1	1.2
HSP90AA1	0.000318	0.226961462	0.914	0.917	1	1.2
ENY2	0.00032	0.232824459	0.432	0.418	1	1.2
ASH1L	0.000328	-0.10717425	0.455	0.511	1	1.2
RP11-795H	0.000341	0.163641391	0.458	0.436	1	1.2
ALPL	0.000343	0.14515174	0.075	0.054	1	1.2
HIF1A	0.000344	-0.160052356	0.354	0.403	1	1.2
NCOA3	0.000351	0.165593833	0.313	0.282	1	1.2
BSDC1	0.000357	0.155398751	0.201	0.173	1	1.2
POLE2	0.000362	0.172428486	0.238	0.208	1	1.2
ROBO2	0.000364	0.100087947	0.07	0.05	1	1.2
PKP4	0.000366	-0.107806987	0.395	0.451	1	1.2
RNF24	0.000369	-0.102234624	0.379	0.432	1	1.2

HIF1A-AS2	0.000372	0.27810962	0.165	0.138	1	1.2
ARF6	0.000377	0.123033302	0.213	0.182	1	1.2
SPATS2L	0.000378	0.173438317	0.302	0.27	1	1.2
GNA12	0.000378	0.160003138	0.379	0.352	1	1.2
RAB5A	0.00038	0.123502649	0.246	0.214	1	1.2
UBR4	0.000413	0.156820557	0.215	0.184	1	1.2
IPO5	0.000425	0.14032658	0.182	0.152	1	1.2
TOR1AIP1	0.000436	0.1475544	0.109	0.085	1	1.2
SEC62	0.000436	0.133887342	0.624	0.626	1	1.2
FBXO28	0.000439	0.16358345	0.225	0.195	1	1.2
BLVRA	0.000444	0.1166073	0.108	0.084	1	1.2
ANKRD11	0.000453	0.234111909	0.313	0.287	1	1.2
PTBP3	0.000457	0.146061264	0.377	0.35	1	1.2
NDUFS4	0.000459	-0.105976515	0.415	0.475	1	1.2
HSD17B6	0.000459	0.214544688	0.048	0.032	1	1.2
SGK1	0.00047	0.172436778	0.201	0.171	1	1.2
YWHAE	0.000481	0.100536584	0.635	0.614	1	1.2
FGD6	0.000503	0.202069693	0.274	0.247	1	1.2
SCD5	0.000503	0.114026404	0.078	0.058	1	1.2
SGMS1	0.000504	0.206719655	0.296	0.268	1	1.2
NEK1	0.00051	-0.11040201	0.067	0.092	1	1.2
TANC1	0.000519	-0.111346889	0.224	0.268	1	1.2
C8orf37-AS	0.000527	0.15238112	0.293	0.262	1	1.2
RAB3GAP1	0.000533	0.127058624	0.323	0.291	1	1.2
HNRNPK	0.000552	0.176075072	0.481	0.471	1	1.2
SRSF11	0.000557	0.13350824	0.492	0.475	1	1.2
PFDN2	0.000589	0.151450036	0.095	0.073	1	1.2
STX7	0.000593	0.144033483	0.182	0.153	1	1.2
NDUFA1	0.000602	0.149034301	0.567	0.559	1	1.2
PPP2R3A	0.000611	-0.114970042	0.373	0.42	1	1.2
ZFAND5	0.000612	-0.109397708	0.306	0.354	1	1.2
SRPK2	0.000616	0.142962339	0.4	0.372	1	1.2
CPNE4	0.000617	0.11280253	0.059	0.041	1	1.2
RP11-66B2	0.000628	0.112518788	0.1	0.078	1	1.2
PRPSAP1	0.000634	0.127501573	0.138	0.112	1	1.2
CKS2	0.000634	0.152171933	0.164	0.137	1	1.2
NUP88	0.000661	0.136257346	0.217	0.187	1	1.2
RP11-417F	0.000666	0.126861691	0.136	0.111	1	1.2
FBXW11	0.000673	0.160052375	0.343	0.318	1	1.2
ARIH2	0.000682	0.140888008	0.148	0.121	1	1.2
PGM3	0.000699	0.136626862	0.13	0.106	1	1.2
ESYT2	0.000699	0.102615389	0.527	0.507	1	1.2
EFTUD1	0.000719	0.178017848	0.122	0.099	1	1.2
LGALS3	0.000721	0.240440161	0.203	0.176	1	1.2
GLRX3	0.000726	0.126612495	0.118	0.094	1	1.2
LIMS1	0.000735	0.101661917	0.342	0.309	1	1.2
GPR87	0.000738	0.109949358	0.071	0.052	1	1.2

MEAF6	0.000747	-0.109883337	0.123	0.154	1	1.2
IQCJ-SCHIP	0.000749	0.120943059	0.093	0.071	1	1.2
TUBB	0.00075	0.15031325	0.369	0.344	1	1.2
HERC3	0.000754	-0.136328178	0.131	0.162	1	1.2
DNER	0.000757	-0.116134072	0.055	0.077	1	1.2
NET1	0.000796	-0.130097503	0.165	0.197	1	1.2
ADRBK2	0.000813	0.128615279	0.124	0.1	1	1.2
RAB3IP	0.000828	0.120039968	0.138	0.114	1	1.2
CXCL3	0.000859	-0.138103802	0.072	0.097	1	1.2
PLSCR1	0.000875	0.159664382	0.31	0.285	1	1.2
CEBPZ	0.000878	0.167707922	0.196	0.171	1	1.2
SAR1B	0.000937	0.133742084	0.478	0.449	1	1.2
GSPT1	0.000942	0.142316086	0.238	0.21	1	1.2
SOS1	0.000954	-0.137197753	0.349	0.392	1	1.2
SMURF2	0.000954	0.145849631	0.383	0.36	1	1.2
UBAP1	0.00098	-0.113711244	0.302	0.346	1	1.2
GRIK1	0.000984	0.11944263	0.068	0.05	1	1.2
TMEM2	0.000991	0.122111817	0.189	0.159	1	1.2
L3MBTL1	0.001009	0.10100685	0.05	0.035	1	1.2
UCHL3	0.001081	0.134982983	0.161	0.135	1	1.2
CXCL16	0.001101	0.134455693	0.221	0.193	1	1.2
RP11-452H	0.001126	0.108389557	0.105	0.082	1	1.2
RGS6	0.001132	0.210547807	0.165	0.14	1	1.2
CD9	0.001179	0.171474815	0.273	0.25	1	1.2
TMEM181	0.001188	0.171466259	0.262	0.236	1	1.2
TMBIM1	0.001198	0.12143816	0.155	0.13	1	1.2
TRA2B	0.001209	-0.103369885	0.171	0.206	1	1.2
EIF4A1	0.001215	0.154446578	0.595	0.582	1	1.2
VAT1	0.001231	0.129751058	0.139	0.116	1	1.2
ZNF431	0.001266	0.141424666	0.154	0.129	1	1.2
CDK6	0.001277	0.151840799	0.251	0.223	1	1.2
KLF5	0.001354	0.117886075	0.251	0.222	1	1.2
RPS27L	0.001362	0.241673583	0.528	0.53	1	1.2
CTIF	0.001365	0.106151043	0.087	0.067	1	1.2
SARS	0.001401	0.145380908	0.226	0.198	1	1.2
SLC6A14	0.001407	0.170098361	0.235	0.206	1	1.2
DDIT4	0.00143	-0.129433902	0.184	0.219	1	1.2
TOR1AIP2	0.001528	0.14797757	0.259	0.234	1	1.2
ABHD3	0.001528	0.159568121	0.163	0.138	1	1.2
HN1	0.001532	0.106663191	0.067	0.049	1	1.2
CD58	0.001596	0.144940003	0.105	0.085	1	1.2
UBE2D3	0.001597	0.154625588	0.567	0.561	1	1.2
KCNQ1OT1	0.001645	0.109693533	0.117	0.094	1	1.2
DCAF6	0.00171	0.143264545	0.469	0.452	1	1.2
MAPK1	0.001722	0.124960748	0.173	0.147	1	1.2
NUP153	0.001726	-0.156566239	0.244	0.278	1	1.2
STK17A	0.001759	0.106657973	0.087	0.067	1	1.2

POLR2K	0.001762	0.164470131	0.347	0.329	1	1.2
RPS15	0.001764	-0.144987179	0.146	0.178	1	1.2
COX7B	0.001819	0.126084875	0.656	0.657	1	1.2
HNRNPH2	0.001831	0.138024709	0.246	0.22	1	1.2
TXN	0.00189	0.213364538	0.7	0.709	1	1.2
CLTC	0.001918	0.146736514	0.224	0.2	1	1.2
TAGLN	0.001994	0.324033861	0.035	0.022	1	1.2
CHCHD2	0.002049	0.169026145	0.254	0.228	1	1.2
PHACTR4	0.002076	0.138473917	0.41	0.391	1	1.2
GAN	0.002096	0.132228557	0.159	0.136	1	1.2
CDYL	0.002115	0.160432764	0.247	0.222	1	1.2
DENND4A	0.002177	0.178323822	0.528	0.519	1	1.2
FAM219A	0.002241	0.127212875	0.099	0.079	1	1.2
AIM1	0.002285	-0.111673413	0.283	0.323	1	1.2
PXN	0.002306	0.104456918	0.105	0.085	1	1.2
SPAG9	0.002369	0.106143523	0.359	0.333	1	1.2
RAB18	0.002382	0.139729558	0.307	0.28	1	1.2
FABP6	0.002399	-0.132298795	0.034	0.05	1	1.2
UBE2L3	0.00251	0.140415693	0.463	0.452	1	1.2
MFAP1	0.002596	0.134718377	0.177	0.153	1	1.2
BCL2L1	0.002616	0.149560139	0.112	0.092	1	1.2
GATAD2B	0.002685	0.111844791	0.249	0.224	1	1.2
SESTD1	0.002713	0.105915072	0.619	0.625	1	1.2
RNF38	0.002824	0.137585525	0.243	0.219	1	1.2
TEC	0.002827	0.125965146	0.075	0.058	1	1.2
UGCG	0.0029	-0.151751421	0.3	0.337	1	1.2
CDKL1	0.002909	0.113732969	0.071	0.054	1	1.2
VEZT	0.002914	0.146647434	0.356	0.334	1	1.2
SLC36A4	0.002937	0.104996303	0.084	0.066	1	1.2
EYS	0.003053	0.102749968	0.084	0.066	1	1.2
RPL9	0.003056	0.13035009	0.666	0.673	1	1.2
ATP8B1	0.003082	-0.168251499	0.304	0.339	1	1.2
LAPTM4A	0.003147	0.151125478	0.256	0.233	1	1.2
RND3	0.003159	0.120679865	0.322	0.295	1	1.2
HSPA1A	0.003174	0.11637874	0.063	0.047	1	1.2
BIRC6-AS2	0.003185	0.16289346	0.25	0.226	1	1.2
EIF1AX	0.003217	0.12527578	0.257	0.232	1	1.2
TNFRSF10E	0.003219	-0.101061702	0.172	0.204	1	1.2
MKL1	0.003298	-0.235217718	0.46	0.496	1	1.2
ETS2	0.003394	0.1101291	0.143	0.121	1	1.2
WFDC2	0.003436	0.102906439	0.513	0.496	1	1.2
PCSK5	0.003466	0.121971526	0.062	0.046	1	1.2
NUB1	0.003541	-0.122808364	0.261	0.301	1	1.2
GFPT1	0.003608	0.150363542	0.226	0.203	1	1.2
TNFAIP6	0.003616	0.187160267	0.377	0.43	1	1.2
GRB14	0.003751	0.163479019	0.564	0.562	1	1.2
MARK3	0.003762	0.125406688	0.446	0.429	1	1.2

OFD1	0.003924	0.180688636	0.319	0.301	1	1.2
OGDH	0.003951	0.14598799	0.125	0.104	1	1.2
RAB12	0.004069	0.109411814	0.217	0.192	1	1.2
NUTM2B-A	0.004107	0.166375411	0.315	0.293	1	1.2
FBXW7	0.004149	-0.107982783	0.291	0.331	1	1.2
DCUN1D4	0.004287	0.12931183	0.193	0.17	1	1.2
STARD13	0.004413	0.220030649	0.219	0.198	1	1.2
NCAM1	0.004416	0.101789723	0.049	0.035	1	1.2
ARL4C	0.004464	0.112753536	0.237	0.211	1	1.2
RANBP17	0.004487	0.186238527	0.194	0.17	1	1.2
BRD2	0.004707	0.14159862	0.241	0.219	1	1.2
CCT2	0.004732	0.174574684	0.415	0.405	1	1.2
CSTB	0.004773	-0.141621927	0.166	0.194	1	1.2
MAP3K8	0.004822	0.1050114	0.301	0.275	1	1.2
ALDOA	0.004825	0.349217553	0.136	0.116	1	1.2
ACTG2	0.004897	0.118243473	0.023	0.014	1	1.2
UBE3C	0.00502	0.135568104	0.289	0.27	1	1.2
UBXN2A	0.005095	-0.121699826	0.069	0.089	1	1.2
TMPRSS2	0.005237	0.138919138	0.152	0.131	1	1.2
PPARG	0.005357	0.103431002	0.073	0.057	1	1.2
UBA6	0.005414	0.117671514	0.351	0.331	1	1.2
TRAPPC3	0.005505	0.113010479	0.087	0.07	1	1.2
IFNAR2	0.005515	0.169483178	0.315	0.299	1	1.2
SNX24	0.005749	0.11419784	0.224	0.202	1	1.2
PUM2	0.005793	-0.111091657	0.327	0.364	1	1.2
SEC31A	0.005795	0.143401864	0.462	0.45	1	1.2
GPR160	0.005872	0.102442263	0.07	0.054	1	1.2
CTC-444N2	0.005984	0.12449991	0.097	0.079	1	1.2
TUFT1	0.00601	-0.108453638	0.131	0.155	1	1.2
TRNAU1AP	0.006056	0.12729934	0.289	0.269	1	1.2
TM2D1	0.006078	0.102686369	0.109	0.09	1	1.2
LAMTOR3	0.006122	0.100659053	0.158	0.136	1	1.2
FYTTD1	0.006203	0.115057341	0.13	0.11	1	1.2
RAPGEF4	0.006373	0.100639755	0.112	0.093	1	1.2
CSDE1	0.00639	0.136429681	0.579	0.586	1	1.2
S100A16	0.006441	0.206326877	0.116	0.096	1	1.2
KPNA2	0.006642	0.126501259	0.185	0.164	1	1.2
MAGT1	0.006878	0.12839475	0.196	0.174	1	1.2
WDR43	0.006941	0.10532552	0.126	0.106	1	1.2
TMEM65	0.007026	0.106168917	0.197	0.175	1	1.2
PRRG4	0.00705	0.146197323	0.215	0.195	1	1.2
TMED2	0.007107	0.119897457	0.172	0.15	1	1.2
EXT2	0.007113	0.107497796	0.15	0.128	1	1.2
TMEM159	0.007206	0.144928395	0.432	0.428	1	1.2
ABL2	0.007303	0.135398099	0.219	0.2	1	1.2
YPEL5	0.007352	0.117029081	0.395	0.379	1	1.2
STK40	0.00777	0.10261378	0.146	0.126	1	1.2

GOLGA4	0.007837	-0.110728254	0.607	0.662	1	1.2
ALCAM	0.00796	0.142903983	0.197	0.174	1	1.2
CCDC93	0.007993	0.102896699	0.135	0.115	1	1.2
ASPH	0.007995	0.133981207	0.13	0.112	1	1.2
LMBR1	0.008031	0.107145934	0.137	0.117	1	1.2
IFFO2	0.008353	0.10512592	0.159	0.137	1	1.2
MAP1LC3B	0.008387	0.122710793	0.123	0.104	1	1.2
PPP2CB	0.008464	-0.134290169	0.285	0.315	1	1.2
GTF2B	0.008476	0.143856849	0.236	0.218	1	1.2
MRPS6	0.008796	0.177574838	0.209	0.186	1	1.2
GPATCH2L	0.008981	0.115547382	0.138	0.118	1	1.2
UBE2K	0.009006	0.10452501	0.343	0.322	1	1.2
MAX	0.009103	0.111353369	0.197	0.176	1	1.2
AMOTL1	0.009164	0.124329568	0.1	0.083	1	1.2
TAOK1	0.009261	0.128768389	0.268	0.251	1	1.2
MFN1	0.00932	0.103730359	0.168	0.147	1	1.2
NR2C2	0.009416	0.113126724	0.154	0.133	1	1.2
CEACAM1	0.009448	0.163225794	0.131	0.113	1	1.2
SRSF5	0.009494	0.101481838	0.335	0.316	1	1.2
SRP54	0.009801	0.138060352	0.29	0.273	1	1.2
FLNB	0.009859	-0.145409387	0.358	0.385	1	1.2
PIK3CA	0.009902	0.108626236	0.239	0.218	1	1.2
PDIA6	0.009956	-0.164095972	0.226	0.251	1	1.2
SLIRP	0.009981	0.172002249	0.348	0.333	1	1.2
ANXA6	0.010333	0.112400331	0.095	0.079	1	1.2
GATAD2A	0.010516	0.100501541	0.118	0.099	1	1.2
COCH	0.01069	0.121019033	0.117	0.1	1	1.2
SNRPB	0.011182	0.118319685	0.094	0.078	1	1.2
UAP1	0.011437	0.152286833	0.257	0.237	1	1.2
KIF1B	0.011438	0.208014248	0.484	0.481	1	1.2
XRRA1	0.011513	0.118092833	0.125	0.108	1	1.2
CBR4	0.012084	0.133374451	0.28	0.262	1	1.2
TUBA1B	0.01258	0.108316766	0.289	0.267	1	1.2
KIAA0232	0.014239	0.104754461	0.151	0.133	1	1.2
IWS1	0.014301	0.106422321	0.164	0.146	1	1.2
TFG	0.015049	-0.104390868	0.288	0.318	1	1.2
RBM25	0.015051	0.122095043	0.338	0.323	1	1.2
GADD45A	0.015399	-0.173668304	0.213	0.238	1	1.2
UXS1	0.015494	0.125271298	0.11	0.094	1	1.2
SEC11A	0.01562	0.108985531	0.532	0.534	1	1.2
ANKIB1	0.01608	0.125674087	0.33	0.315	1	1.2
NF1	0.017263	0.116235737	0.465	0.455	1	1.2
GLO1	0.017333	0.109717844	0.117	0.101	1	1.2
PTPN14	0.018942	-0.185384178	0.428	0.465	1	1.2
KCMF1	0.019199	-0.128077508	0.28	0.309	1	1.2
FAM46A	0.019767	0.134939372	0.123	0.107	1	1.2
SIAH2	0.020482	0.190172686	0.069	0.056	1	1.2

PPP1R14C	0.020482	0.1139345	0.208	0.191	1	1.2
AC074391.	0.020515	-0.1141376	0.045	0.058	1	1.2
EIF5A	0.020713	0.101035335	0.072	0.059	1	1.2
GTF2I	0.021276	0.138327266	0.441	0.433	1	1.2
NAA15	0.021334	0.10028143	0.16	0.142	1	1.2
PMM2	0.02137	0.11177077	0.105	0.089	1	1.2
SRFBP1	0.021938	0.119021554	0.21	0.193	1	1.2
DDX24	0.022248	0.117624335	0.612	0.63	1	1.2
EPS15	0.022307	0.10886738	0.252	0.233	1	1.2
N4BP1	0.022337	0.135710191	0.205	0.187	1	1.2
PTTG1IP	0.022715	0.113533969	0.154	0.136	1	1.2
SKIL	0.022732	0.102174176	0.105	0.09	1	1.2
SRPRA	0.023074	0.102611828	0.08	0.067	1	1.2
RNASE4	0.023278	0.110632078	0.082	0.069	1	1.2
SAV1	0.023977	0.1144822	0.382	0.374	1	1.2
ATF3	0.024042	-0.227779642	0.327	0.349	1	1.2
ANKUB1	0.024228	0.165975692	0.15	0.134	1	1.2
WHSC1	0.024347	0.103465578	0.132	0.116	1	1.2
PLIN3	0.024501	0.111154343	0.122	0.106	1	1.2
NME7	0.025386	0.104196129	0.106	0.091	1	1.2
TNF	0.027731	0.166926976	0.115	0.1	1	1.2
NUMB	0.02824	0.104732757	0.452	0.443	1	1.2
DAD1	0.028293	0.123693949	0.513	0.52	1	1.2
GSAP	0.028648	0.132130271	0.165	0.149	1	1.2
KIF5B	0.029337	0.115016094	0.269	0.255	1	1.2
EIF3J	0.03018	0.102271291	0.165	0.148	1	1.2
NDUFV2	0.030364	0.108816278	0.167	0.15	1	1.2
VPS35	0.031515	0.140360515	0.294	0.282	1	1.2
FUS	0.031544	0.100231401	0.308	0.291	1	1.2
BCYRN1	0.031566	0.10160614	0.065	0.053	1	1.2
USP32	0.031632	0.103819733	0.213	0.197	1	1.2
MAPK8	0.032891	0.130222419	0.228	0.212	1	1.2
PPP1R12A	0.033964	0.123520144	0.223	0.208	1	1.2
ATP5EP2	0.034113	0.120868209	0.219	0.202	1	1.2
RAPH1	0.034423	0.142212462	0.204	0.19	1	1.2
EIF3A	0.035245	0.127156728	0.353	0.342	1	1.2
DCAF13	0.035667	0.101987058	0.095	0.082	1	1.2
CDK7	0.036281	0.109357494	0.129	0.115	1	1.2
ANKRD28	0.036355	-0.100095575	0.283	0.311	1	1.2
STK3	0.036688	0.120926224	0.381	0.372	1	1.2
SERINC5	0.037101	0.12188938	0.251	0.237	1	1.2
EGFR	0.037728	0.137326057	0.345	0.333	1	1.2
HSPA4	0.037746	0.111500411	0.122	0.108	1	1.2
RASA1	0.039769	0.113991739	0.215	0.199	1	1.2
LINC00662	0.039956	0.107799532	0.095	0.082	1	1.2
KEAP1	0.040902	0.131614515	0.049	0.039	1	1.2
LRRC49	0.041254	0.153322837	0.135	0.121	1	1.2

HMGCS1	0.041934	0.10768506	0.247	0.232	1	1.2
KIAA0368	0.043384	0.142033413	0.239	0.226	1	1.2
HECA	0.044863	0.102019084	0.118	0.105	1	1.2
UBN2	0.045761	0.100534249	0.159	0.144	1	1.2
SH3PXD2B	0.047121	0.100954468	0.144	0.129	1	1.2
DPM1	0.047217	0.110737565	0.282	0.27	1	1.2
ABHD2	0.047667	0.102918513	0.111	0.097	1	1.2
VDAC1	0.049005	0.108953802	0.228	0.214	1	1.2
SLC16A13	0.050452	-0.144885935	0.044	0.055	1	1.2
GCC2-AS1	0.051159	0.167671328	0.049	0.04	1	1.2
STAG2	0.053673	0.115879687	0.373	0.371	1	1.2
ENAH	0.054271	-0.157110679	0.462	0.495	1	1.2
BPGM	0.055761	0.117282742	0.176	0.162	1	1.2
RAN	0.055812	0.12454699	0.593	0.607	1	1.2
LINC01588	0.05748	0.122338132	0.101	0.089	1	1.2
FAM134B	0.057934	0.118738873	0.161	0.148	1	1.2
TRMT10C	0.058661	0.106499231	0.137	0.125	1	1.2
FARP1	0.060337	0.110078576	0.322	0.309	1	1.2
SET	0.060835	0.163904215	0.482	0.488	1	1.2
HK2	0.060942	-0.125064253	0.16	0.175	1	1.2
PSME4	0.061987	-0.165956348	0.68	0.706	1	1.2
CYP24A1	0.063619	0.171192982	0.208	0.236	1	1.2
MED6	0.064029	0.103492518	0.183	0.17	1	1.2
OTUD7B	0.064418	-0.111021639	0.156	0.173	1	1.2
CCNI	0.064622	0.107387243	0.578	0.601	1	1.2
LUCAT1	0.066549	0.152643922	0.408	0.397	1	1.2
C5orf28	0.06691	0.101414253	0.19	0.178	1	1.2
GABARAPL	0.070019	0.103240504	0.163	0.152	1	1.2
UBE2R2	0.071546	0.101364526	0.337	0.327	1	1.2
XDH	0.074113	0.113962592	0.126	0.114	1	1.2
EDF1	0.074504	0.142179051	0.086	0.075	1	1.2
HK1	0.075624	0.10984128	0.13	0.118	1	1.2
SH2B1	0.077871	-0.105168886	0.019	0.025	1	1.2
ACSS2	0.07899	0.100315743	0.188	0.176	1	1.2
FBXO34	0.079886	0.1291744	0.198	0.188	1	1.2
GBP1	0.081236	-0.157068462	0.179	0.194	1	1.2
STX5	0.082306	0.10209705	0.199	0.186	1	1.2
NCL	0.082944	0.175695985	0.215	0.203	1	1.2
MXD1	0.083321	-0.101035134	0.148	0.164	1	1.2
ORMDL2	0.085414	0.108518024	0.172	0.16	1	1.2
TAF7	0.085435	0.107823014	0.156	0.144	1	1.2
TCEANC2	0.085583	0.126224249	0.222	0.211	1	1.2
ITSN1	0.091127	0.112079246	0.14	0.129	1	1.2
NBPF19	0.091315	0.119407266	0.195	0.183	1	1.2
KCND2	0.092829	-0.111118939	0.079	0.091	1	1.2
DENND5A	0.096254	-0.105672471	0.339	0.36	1	1.2
DHRSX	0.098053	0.100314518	0.232	0.221	1	1.2

SEC61B	0.098715	0.167654597	0.12	0.11	1	1.2
HSPA5	0.104695	-0.296479599	0.383	0.399	1	1.2
TNPO1	0.105524	0.138132927	0.228	0.219	1	1.2
ZNF609	0.109731	0.136460477	0.522	0.536	1	1.2
IRF2	0.11169	0.123740622	0.301	0.296	1	1.2
MT-ND4L	0.112971	0.13200994	0.519	0.538	1	1.2
LTBP1	0.113785	0.162931561	0.233	0.223	1	1.2
CCNC	0.11414	0.100651093	0.27	0.262	1	1.2
C6orf62	0.118424	0.103588358	0.182	0.173	1	1.2
NUDC	0.125106	0.100190291	0.063	0.055	1	1.2
FAM35A	0.141312	0.126082357	0.123	0.113	1	1.2
CCNY	0.156748	-0.132744609	0.248	0.264	1	1.2
TRPM7	0.168361	0.106833792	0.194	0.186	1	1.2
MT2A	0.169354	-0.335560497	0.068	0.077	1	1.2
G3BP1	0.169506	0.107355589	0.18	0.172	1	1.2
UBE2D2	0.172401	0.100153304	0.223	0.215	1	1.2
TBC1D15	0.181377	0.104124668	0.289	0.284	1	1.2
ICAM1	0.182365	0.117644427	0.165	0.157	1	1.2
HERC1	0.185207	-0.105540001	0.38	0.397	1	1.2
FGFR1	0.185512	-0.185713596	0.161	0.169	1	1.2
LALBA	0.194028	-0.245960333	0.011	0.014	1	1.2
SENP5	0.202507	0.113341938	0.211	0.206	1	1.2
UBE2J1	0.206269	0.102435067	0.199	0.191	1	1.2
ADGRA3	0.207639	0.118539757	0.139	0.131	1	1.2
RP11-122M	0.212832	0.103420709	0.022	0.018	1	1.2
CCNG2	0.234099	0.10085037	0.157	0.151	1	1.2
ARHGAP21	0.23433	0.123298568	0.246	0.241	1	1.2
PTGES3	0.242866	0.102469278	0.216	0.211	1	1.2
USP25	0.244289	0.102976481	0.163	0.155	1	1.2
MYO5C	0.245174	0.120130915	0.111	0.104	1	1.2
ASTN2	0.252441	0.110704909	0.225	0.221	1	1.2
CLIP1	0.28245	0.119043407	0.382	0.384	1	1.2
C2orf88	0.288309	0.14609844	0.377	0.389	1	1.2
DTNA	0.305458	-0.24817951	0.723	0.721	1	1.2
CARHSP1	0.331032	0.127587417	0.274	0.272	1	1.2
ZNF462	0.350778	0.124606972	0.343	0.35	1	1.2
RP11-37B2	0.353955	0.175133723	0.287	0.294	1	1.2
NCOA2	0.362103	0.149789872	0.487	0.502	1	1.2
URB1	0.362238	0.147917737	0.054	0.06	1	1.2
PLEKHG1	0.418354	0.119405468	0.178	0.174	1	1.2
OSER1	0.424716	-0.331542288	0.581	0.541	1	1.2
BRINP1	0.438431	0.181578558	0.191	0.205	1	1.2
ZC3H7A	0.441667	0.217564836	0.182	0.195	1	1.2
EIF2S1	0.45841	0.136431487	0.257	0.262	1	1.2
LRCH3	0.481092	0.107355518	0.358	0.368	1	1.2
CXCL1	0.487443	0.126014295	0.255	0.251	1	1.2
ZCCHC2	0.506449	0.127019753	0.307	0.31	1	1.2

PPIB	0.526307	0.136895052	0.12	0.118	1	1.2
RP11-356C	0.559809	0.124514941	0.251	0.253	1	1.2
CYSTM1	0.572288	0.101465069	0.164	0.163	1	1.2
MMP7	0.58555	0.165731507	0.297	0.317	1	1.2
LGALS1	0.601926	0.153423393	0.078	0.076	1	1.2
RPL18	0.617245	0.100743618	0.123	0.12	1	1.2
SPECC1	0.634177	0.110802495	0.058	0.056	1	1.2
TGFB2	0.69982	0.106615778	0.244	0.244	1	1.2
MRPL33	0.703385	0.105352042	0.412	0.434	1	1.2
PLOD2	0.739075	0.124545399	0.16	0.16	1	1.2
CCL4	0.755717	0.220276026	0.085	0.088	1	1.2
FAU	0.850615	0.151888053	0.205	0.211	1	1.2
CYR61	0.952452	-0.43113963	0.291	0.283	1	1.2
MGST1	7.87E-272	0.909606936	0.958	0.858	1.90E-267	1.3
LRRFIP2	4.79E-215	-1.369296875	0.694	0.855	1.15E-210	1.3
CD59	4.19E-202	0.752855186	0.966	0.83	1.01E-197	1.3
S100A11	2.05E-193	0.959979558	0.879	0.646	4.95E-189	1.3
FDPS	2.29E-181	1.01505751	0.681	0.387	5.52E-177	1.3
SAA1	1.80E-179	0.868588867	0.99	0.942	4.34E-175	1.3
RP5-1198O	1.29E-156	0.779867277	0.332	0.099	3.11E-152	1.3
CYP24A1	3.30E-149	0.979631205	0.422	0.164	7.95E-145	1.3
LUCAT1	3.92E-143	0.800071959	0.621	0.323	9.45E-139	1.3
OSER1	8.91E-138	-1.456712036	0.393	0.604	2.15E-133	1.3
AKR1C3	2.89E-137	0.731871912	0.365	0.132	6.97E-133	1.3
PRDX1	7.40E-136	0.842829984	0.838	0.677	1.78E-131	1.3
SLC12A2	4.73E-131	0.92244878	0.731	0.474	1.14E-126	1.3
RARRES1	7.47E-130	0.683158979	0.624	0.324	1.80E-125	1.3
DTNA	1.34E-128	-1.064242759	0.602	0.762	3.23E-124	1.3
DEFB1	2.32E-128	0.902077187	0.773	0.538	5.59E-124	1.3
HSPA8	2.04E-122	0.820825263	0.71	0.5	4.91E-118	1.3
SAT1	4.81E-118	0.652997042	0.979	0.956	1.16E-113	1.3
MLLT4	1.82E-115	-0.971010394	0.538	0.705	4.38E-111	1.3
CXCL17	1.41E-110	0.961681925	0.334	0.133	3.40E-106	1.3
MYO5B	2.32E-108	-1.181257377	0.471	0.641	5.60E-104	1.3
PDLIM5	4.54E-108	-0.70531712	0.765	0.864	1.09E-103	1.3
SOD2	1.80E-106	0.488349461	0.996	0.928	4.35E-102	1.3
TSHZ2	3.33E-106	0.783662391	0.649	0.402	8.03E-102	1.3
PI3	2.09E-101	1.067994074	0.467	0.237	5.05E-97	1.3
ZMYND8	8.36E-100	0.664410584	0.428	0.203	2.02E-95	1.3
TRIO	1.74E-97	-1.060024761	0.683	0.764	4.20E-93	1.3
PDE4B	4.57E-93	-0.661583648	0.885	0.922	1.10E-88	1.3
DHFR	1.67E-91	-0.645924242	0.785	0.87	4.03E-87	1.3
FTX	1.72E-85	0.465197642	0.738	0.476	4.15E-81	1.3
ANKRD36C	6.20E-84	0.650409755	0.72	0.505	1.50E-79	1.3
HSPA5	1.41E-83	-1.521688578	0.255	0.443	3.40E-79	1.3
ABI1	5.30E-83	-0.715510105	0.645	0.757	1.28E-78	1.3
TM4SF18	2.06E-82	0.532091193	0.183	0.054	4.96E-78	1.3

LINC-PINT	1.13E-79	0.654311425	0.703	0.491	2.72E-75	1.3
LINC01152	6.20E-79	0.634060681	0.238	0.088	1.49E-74	1.3
OVOS2	3.24E-78	0.730758294	0.656	0.448	7.82E-74	1.3
FNBP1	6.68E-77	-0.774207986	0.357	0.531	1.61E-72	1.3
EIF4G3	1.07E-76	0.54448063	0.481	0.271	2.57E-72	1.3
MYO6	1.08E-76	-0.81751344	0.552	0.679	2.59E-72	1.3
EBP	1.78E-75	0.423024195	0.205	0.07	4.29E-71	1.3
BICD1	2.24E-73	0.527770572	0.323	0.151	5.39E-69	1.3
DPYD	2.25E-73	0.587196699	0.704	0.473	5.42E-69	1.3
PLCB1	3.64E-73	0.526646707	0.559	0.343	8.78E-69	1.3
PSME4	1.57E-72	-0.828540968	0.634	0.723	3.80E-68	1.3
TBC1D5	1.76E-72	0.538097041	0.511	0.303	4.25E-68	1.3
AKR1C1	4.78E-71	0.689754587	0.228	0.087	1.15E-66	1.3
TACC1	6.48E-71	0.514405995	0.372	0.189	1.56E-66	1.3
ZFAS1	1.20E-70	-0.390086062	0.949	0.967	2.90E-66	1.3
AC005152.	6.26E-70	0.66483992	0.287	0.128	1.51E-65	1.3
UBC	1.20E-69	-1.098308066	0.941	0.953	2.90E-65	1.3
FDFT1	2.64E-69	0.561207066	0.482	0.29	6.37E-65	1.3
TMTC2	5.97E-69	0.539944479	0.46	0.26	1.44E-64	1.3
VAMP8	1.23E-68	0.447463814	0.802	0.665	2.96E-64	1.3
ZBTB38	6.32E-68	0.469584513	0.47	0.269	1.52E-63	1.3
DAAM1	3.29E-67	-0.85855649	0.41	0.551	7.93E-63	1.3
DHCR24	6.61E-67	0.413392108	0.286	0.129	1.59E-62	1.3
CYR61	1.18E-66	-1.661416152	0.161	0.327	2.84E-62	1.3
IER3	2.40E-66	0.570646623	0.256	0.11	5.79E-62	1.3
ACAT2	3.13E-66	0.598996845	0.426	0.245	7.55E-62	1.3
RASA2	6.02E-66	-0.892627185	0.558	0.656	1.45E-61	1.3
APP	6.51E-66	0.498605986	0.866	0.737	1.57E-61	1.3
MSMO1	3.32E-65	0.420090594	0.41	0.222	8.00E-61	1.3
SC5D	3.26E-63	0.359004719	0.18	0.062	7.86E-59	1.3
HNRNPC	1.04E-62	-0.549644157	0.757	0.822	2.50E-58	1.3
PIK3R1	4.09E-62	0.533829464	0.401	0.221	9.87E-58	1.3
SPIDR	5.19E-62	0.444685965	0.746	0.539	1.25E-57	1.3
FOXP1	7.12E-62	0.423196552	0.713	0.495	1.72E-57	1.3
KRT15	8.79E-62	-1.009755143	0.218	0.383	2.12E-57	1.3
NPAS2	2.62E-61	0.50793468	0.632	0.435	6.32E-57	1.3
TPD52L1	2.74E-61	0.48415305	0.349	0.183	6.60E-57	1.3
ST8SIA1	7.24E-61	0.488463965	0.297	0.142	1.75E-56	1.3
MRPS6	2.44E-60	0.483106742	0.308	0.151	5.88E-56	1.3
OSBPL9	3.33E-60	-0.861900156	0.358	0.498	8.03E-56	1.3
EHBP1	6.27E-60	0.482334102	0.482	0.296	1.51E-55	1.3
TNFSF10	8.95E-60	0.505537417	0.638	0.442	2.16E-55	1.3
S100A4	1.58E-58	0.394540073	0.183	0.068	3.81E-54	1.3
RPL34	2.74E-58	-0.267561341	0.977	0.987	6.60E-54	1.3
VNN1	3.52E-58	0.514374533	0.387	0.215	8.49E-54	1.3
TUBA1B	1.07E-57	0.497547323	0.4	0.228	2.58E-53	1.3
DYNLT1	2.33E-57	0.412750724	0.74	0.575	5.63E-53	1.3

IDI1	2.87E-57	0.434943763	0.333	0.175	6.91E-53	1.3
TSC22D2	4.11E-56	-0.911516523	0.212	0.362	9.91E-52	1.3
SMYD3	5.94E-56	0.536233997	0.348	0.191	1.43E-51	1.3
ADGRF1	1.38E-55	0.327639513	0.129	0.038	3.32E-51	1.3
HSP90B1	1.43E-55	-0.813704906	0.363	0.504	3.44E-51	1.3
AZGP1	1.40E-54	0.695399747	0.465	0.299	3.38E-50	1.3
RP4-678D1	3.57E-54	0.464755154	0.264	0.125	8.62E-50	1.3
ARPC3	3.77E-53	0.423523518	0.766	0.634	9.09E-49	1.3
SOS1	5.59E-53	0.479922767	0.511	0.339	1.35E-48	1.3
CTSB	6.32E-53	0.558284526	0.423	0.263	1.52E-48	1.3
HNRNPA1	7.13E-53	-0.503102672	0.874	0.901	1.72E-48	1.3
ECHDC1	7.95E-53	0.385116168	0.401	0.233	1.92E-48	1.3
USP39	2.95E-52	0.398307809	0.487	0.306	7.11E-48	1.3
SORBS1	4.90E-52	0.422452939	0.478	0.298	1.18E-47	1.3
GBP2	8.94E-52	-0.523954941	0.701	0.772	2.16E-47	1.3
WFDC2	1.11E-51	0.838350915	0.642	0.451	2.67E-47	1.3
AKR1B1	1.43E-51	0.497458366	0.14	0.047	3.46E-47	1.3
SAA2-SAA4	1.76E-51	0.386284891	0.52	0.326	4.24E-47	1.3
EEF1A1	2.07E-51	-0.258412846	0.978	0.985	4.99E-47	1.3
CCL2	4.51E-51	1.001725184	0.549	0.392	1.09E-46	1.3
USP53	4.57E-51	-0.946377647	0.627	0.683	1.10E-46	1.3
RICTOR	5.67E-51	-0.64766293	0.431	0.558	1.37E-46	1.3
ANXA3	6.97E-51	0.456360934	0.541	0.37	1.68E-46	1.3
LTBP1	9.41E-51	0.488276254	0.336	0.187	2.27E-46	1.3
ATP1B1	1.18E-50	0.743560284	0.804	0.679	2.85E-46	1.3
CLIP2	1.54E-50	0.422140104	0.295	0.151	3.72E-46	1.3
RPS4X	1.56E-50	-0.265279358	0.977	0.986	3.76E-46	1.3
SFRP1	4.27E-50	0.420937174	0.596	0.413	1.03E-45	1.3
CYP7B1	7.13E-50	0.466017978	0.512	0.329	1.72E-45	1.3
AFF1	6.92E-49	0.41901328	0.418	0.256	1.67E-44	1.3
CHD2	7.62E-49	-0.671750533	0.333	0.466	1.84E-44	1.3
EFNA5	7.95E-49	0.403137626	0.642	0.447	1.92E-44	1.3
TMBIM6	9.40E-49	0.480381745	0.859	0.779	2.27E-44	1.3
EGFR	1.09E-48	0.455301605	0.461	0.292	2.62E-44	1.3
GADD45A	1.17E-48	-0.635874235	0.128	0.268	2.82E-44	1.3
RNF145	1.98E-48	-0.71396853	0.487	0.589	4.76E-44	1.3
L3MBTL4	2.20E-48	0.431805798	0.47	0.296	5.29E-44	1.3
COBL	3.93E-48	-0.749640079	0.404	0.529	9.47E-44	1.3
GBP1	4.75E-48	-0.683533419	0.092	0.225	1.14E-43	1.3
ATP1A1	1.86E-47	0.429142461	0.635	0.469	4.48E-43	1.3
BACH2	2.56E-47	-0.714948465	0.308	0.448	6.17E-43	1.3
LINC01184	3.69E-47	0.418384427	0.326	0.183	8.89E-43	1.3
TRPS1	4.78E-47	0.339154437	0.846	0.674	1.15E-42	1.3
NOP10	5.50E-47	0.386355109	0.591	0.429	1.33E-42	1.3
FAM129A	7.61E-47	-0.682799858	0.374	0.515	1.83E-42	1.3
S100A14	9.22E-47	0.359942212	0.774	0.63	2.22E-42	1.3
SCD	1.41E-46	0.289139639	0.157	0.06	3.41E-42	1.3

H2AFZ	2.08E-46	0.379715989	0.719	0.579	5.02E-42	1.3
FLNB	8.88E-46	-0.694234306	0.277	0.414	2.14E-41	1.3
NFIB	1.56E-45	0.337823812	0.932	0.824	3.77E-41	1.3
LRP1B	7.46E-45	0.861304321	0.306	0.171	1.80E-40	1.3
ATP5G2	8.29E-45	-0.501910944	0.743	0.799	2.00E-40	1.3
ARL15	1.81E-44	0.384788871	0.313	0.173	4.37E-40	1.3
GHITM	2.55E-44	0.424408382	0.595	0.442	6.14E-40	1.3
C4orf3	3.32E-44	-0.640704616	0.557	0.65	8.01E-40	1.3
GAB1	4.07E-44	0.415042244	0.486	0.32	9.82E-40	1.3
RPL22	7.21E-44	-0.411910817	0.757	0.807	1.74E-39	1.3
BIRC3	8.59E-44	0.410788593	0.886	0.804	2.07E-39	1.3
S100A6	8.78E-44	0.687770187	0.599	0.42	2.12E-39	1.3
MT-ATP6	1.25E-43	0.295924757	0.988	0.986	3.00E-39	1.3
RBX1	1.28E-43	0.374848067	0.575	0.412	3.09E-39	1.3
PPIA	1.28E-43	0.296451468	0.909	0.845	3.10E-39	1.3
BTG1	1.60E-43	0.441035905	0.589	0.428	3.87E-39	1.3
CALM1	1.74E-43	0.367492775	0.336	0.192	4.21E-39	1.3
LHFPL2	1.44E-42	-0.685801624	0.307	0.433	3.46E-38	1.3
CDH1	2.55E-42	-0.604852819	0.437	0.544	6.14E-38	1.3
SRP14	2.99E-42	0.310937891	0.886	0.83	7.21E-38	1.3
EIF2AK3	3.89E-42	-0.908204364	0.209	0.331	9.37E-38	1.3
WAC	4.63E-42	-0.552976799	0.572	0.647	1.12E-37	1.3
GOLGA4	4.86E-42	-0.521511599	0.593	0.669	1.17E-37	1.3
SRRM1	5.18E-42	-0.516776273	0.608	0.676	1.25E-37	1.3
SLPI	5.36E-42	0.335167674	0.891	0.788	1.29E-37	1.3
RERG	6.38E-42	0.392095061	0.248	0.126	1.54E-37	1.3
SLC26A2	1.03E-41	0.401771238	0.251	0.129	2.48E-37	1.3
EIF4E2	1.57E-41	0.362916609	0.518	0.357	3.79E-37	1.3
MAP3K5	1.58E-41	0.390851477	0.439	0.282	3.81E-37	1.3
CHPT1	2.01E-41	0.434594086	0.507	0.347	4.84E-37	1.3
NTN4	2.10E-41	0.461026841	0.272	0.147	5.06E-37	1.3
RIPK2	3.36E-41	-0.638962415	0.299	0.431	8.10E-37	1.3
SIK3	6.29E-41	-0.295238835	0.977	0.986	1.52E-36	1.3
AUH	9.62E-41	-0.671647501	0.245	0.37	2.32E-36	1.3
MRPL27	1.51E-40	0.300994635	0.263	0.14	3.65E-36	1.3
RSU1	4.45E-40	0.364331558	0.292	0.164	1.07E-35	1.3
RPL30	5.78E-40	-0.235684876	0.973	0.983	1.39E-35	1.3
TMEM126E	6.71E-40	0.270433113	0.2	0.095	1.62E-35	1.3
CAMTA1	7.46E-40	-0.548660901	0.442	0.551	1.80E-35	1.3
FGFR1	1.42E-39	-0.645130073	0.083	0.196	3.42E-35	1.3
FABP7	1.56E-39	-1.162799325	0.13	0.253	3.76E-35	1.3
RBM47	1.59E-39	0.342414846	0.722	0.545	3.84E-35	1.3
SYNPO2	2.07E-39	-0.711599336	0.111	0.235	4.99E-35	1.3
ZCCHC17	3.70E-39	0.34924662	0.424	0.274	8.93E-35	1.3
TPCN1	4.32E-39	0.395875674	0.197	0.094	1.04E-34	1.3
DIAPH2	2.63E-38	0.375631704	0.38	0.238	6.34E-34	1.3
SELK	2.81E-38	-0.598001225	0.632	0.69	6.77E-34	1.3

GGPS1	3.31E-38	0.299568269	0.299	0.17	7.98E-34	1.3
STAG1	5.72E-38	0.343467492	0.605	0.439	1.38E-33	1.3
SIPA1L1	7.42E-38	0.353112695	0.471	0.317	1.79E-33	1.3
SGK1	1.83E-37	0.341812593	0.267	0.147	4.40E-33	1.3
5-Mar	2.87E-37	-0.57513259	0.144	0.263	6.92E-33	1.3
NUDT5	3.73E-37	0.306514256	0.273	0.153	9.00E-33	1.3
HIST1H2AC	3.76E-37	0.330779409	0.432	0.28	9.08E-33	1.3
EVA1C	5.97E-37	0.231469963	0.589	0.401	1.44E-32	1.3
ENAH	5.97E-37	-0.651779027	0.417	0.512	1.44E-32	1.3
MRPL13	6.70E-37	0.295607391	0.27	0.149	1.62E-32	1.3
ADAM9	7.43E-37	-0.633244599	0.522	0.598	1.79E-32	1.3
IDH1	9.59E-37	0.223622305	0.13	0.051	2.31E-32	1.3
DTNB	1.32E-36	0.315135342	0.705	0.536	3.17E-32	1.3
C15orf48	1.58E-36	0.577845542	0.593	0.451	3.80E-32	1.3
NAPG	2.79E-36	0.28947812	0.283	0.16	6.72E-32	1.3
TAF1D	3.06E-36	-0.527167592	0.229	0.354	7.39E-32	1.3
GLIS3	4.17E-36	0.308274423	0.397	0.25	1.00E-31	1.3
HIST1H2BB	6.27E-36	0.391838881	0.214	0.111	1.51E-31	1.3
HCAR2	1.30E-35	-0.731332025	0.117	0.23	3.14E-31	1.3
LINGO2	2.86E-35	0.596330578	0.122	0.048	6.89E-31	1.3
MED31	3.56E-35	0.290361461	0.28	0.16	8.58E-31	1.3
BTBD9	4.41E-35	0.348921873	0.288	0.167	1.06E-30	1.3
RPS24	4.88E-35	-0.214984943	0.975	0.985	1.18E-30	1.3
CACHD1	1.20E-34	0.280092551	0.154	0.069	2.90E-30	1.3
TUBA1A	1.32E-34	0.437855808	0.349	0.221	3.19E-30	1.3
PLA2R1	1.95E-34	0.311667006	0.337	0.205	4.69E-30	1.3
ATP1B3	2.71E-34	0.340718609	0.367	0.234	6.55E-30	1.3
AKR1C2	3.17E-34	0.325055749	0.135	0.057	7.64E-30	1.3
CRIM1	3.68E-34	-0.589691952	0.431	0.527	8.86E-30	1.3
RPL31	4.69E-34	-0.259393866	0.959	0.966	1.13E-29	1.3
PLXDC2	6.94E-34	0.324113963	0.333	0.206	1.67E-29	1.3
ARHGAP44	8.57E-34	0.359575004	0.338	0.21	2.07E-29	1.3
PAPSS1	1.34E-33	0.33655626	0.483	0.339	3.23E-29	1.3
DOCK1	2.17E-33	0.34597591	0.406	0.265	5.22E-29	1.3
GUCY1A3	2.60E-33	0.306851879	0.326	0.198	6.28E-29	1.3
UBE2H	3.31E-33	-0.431694708	0.672	0.732	7.97E-29	1.3
TXNIP	4.55E-33	0.224173163	0.593	0.419	1.10E-28	1.3
PPP2R3A	5.94E-33	0.304561016	0.525	0.37	1.43E-28	1.3
TOMM7	6.14E-33	-0.304758561	0.888	0.913	1.48E-28	1.3
DENND5A	6.23E-33	-0.483861016	0.267	0.385	1.50E-28	1.3
ACACA	6.78E-33	0.285617218	0.234	0.128	1.64E-28	1.3
RSRC1	7.63E-33	0.314682688	0.354	0.224	1.84E-28	1.3
RPS27L	7.76E-33	0.373533632	0.633	0.494	1.87E-28	1.3
BPTF	1.46E-32	0.29587819	0.444	0.298	3.51E-28	1.3
S100A13	2.07E-32	0.358656008	0.694	0.575	4.99E-28	1.3
PDZK1IP1	2.52E-32	0.349103979	0.254	0.144	6.08E-28	1.3
RERE	5.76E-32	0.314269306	0.537	0.394	1.39E-27	1.3

PKM	6.01E-32	0.373188848	0.349	0.223	1.45E-27	1.3
RPS15A	6.75E-32	-0.240044946	0.958	0.974	1.63E-27	1.3
ARID1B	7.53E-32	0.294287881	0.522	0.369	1.82E-27	1.3
COA1	7.99E-32	0.295708305	0.373	0.239	1.93E-27	1.3
IRF2BPL	9.04E-32	0.252488361	0.199	0.104	2.18E-27	1.3
LTF	9.56E-32	0.148245099	0.803	0.619	2.31E-27	1.3
MKL1	1.01E-31	-0.599498321	0.426	0.509	2.42E-27	1.3
PHLDA1	1.31E-31	0.282148501	0.253	0.144	3.17E-27	1.3
PSMB1	1.37E-31	0.345902333	0.565	0.427	3.30E-27	1.3
MGST3	1.42E-31	0.309513864	0.598	0.457	3.42E-27	1.3
FZD7	1.42E-31	0.238930215	0.136	0.06	3.43E-27	1.3
RTN4	2.42E-31	-0.475002521	0.577	0.645	5.83E-27	1.3
ARG2	3.79E-31	0.209268674	0.232	0.127	9.14E-27	1.3
GRHL2	4.01E-31	0.342377807	0.373	0.242	9.68E-27	1.3
TULP4	4.99E-31	-0.533643118	0.426	0.52	1.20E-26	1.3
TUBB	5.82E-31	0.371053162	0.449	0.316	1.40E-26	1.3
DMKN	6.14E-31	0.255111355	0.175	0.087	1.48E-26	1.3
SREBF2	6.93E-31	0.314578373	0.465	0.325	1.67E-26	1.3
SKAP2	1.23E-30	0.317580819	0.426	0.289	2.96E-26	1.3
SLC20A2	1.57E-30	-0.611206422	0.309	0.412	3.80E-26	1.3
FADS1	1.59E-30	0.169667383	0.064	0.017	3.83E-26	1.3
RALGAPA2	1.75E-30	0.24862589	0.275	0.162	4.23E-26	1.3
CHMP5	3.01E-30	0.331615997	0.44	0.31	7.27E-26	1.3
RPL37	3.33E-30	-0.226819764	0.967	0.976	8.03E-26	1.3
GPBP1	3.41E-30	-0.473650121	0.57	0.633	8.23E-26	1.3
EXOC4	4.31E-30	0.312571139	0.391	0.261	1.04E-25	1.3
SRPX2	4.32E-30	0.185092417	0.085	0.029	1.04E-25	1.3
ATP6V0E1	4.71E-30	0.266683543	0.756	0.636	1.13E-25	1.3
PRKCE	5.49E-30	0.352802083	0.289	0.177	1.32E-25	1.3
C14orf119	5.54E-30	0.2538092	0.28	0.167	1.34E-25	1.3
MT-CO2	5.80E-30	0.247992338	0.986	0.985	1.40E-25	1.3
STEAP1	6.34E-30	0.177492743	0.091	0.033	1.53E-25	1.3
TXN	9.09E-30	0.325363222	0.771	0.685	2.19E-25	1.3
LRP6	9.24E-30	-0.52505168	0.212	0.319	2.23E-25	1.3
ARHGEF28	1.11E-29	-0.679665872	0.307	0.41	2.68E-25	1.3
MAML3	1.17E-29	0.339400887	0.33	0.212	2.83E-25	1.3
CALD1	1.47E-29	-0.60065563	0.541	0.618	3.53E-25	1.3
RPS3A	1.64E-29	-0.229594145	0.941	0.956	3.95E-25	1.3
CCAR1	1.69E-29	0.298236715	0.348	0.224	4.07E-25	1.3
ARHGAP29	1.72E-29	-0.842506375	0.425	0.503	4.14E-25	1.3
RLF	2.26E-29	-0.629699966	0.275	0.374	5.46E-25	1.3
NEDD4L	3.08E-29	-0.500800902	0.485	0.569	7.44E-25	1.3
SQLE	3.09E-29	0.158438607	0.103	0.04	7.45E-25	1.3
ATRNL1	3.35E-29	0.317631095	0.132	0.059	8.08E-25	1.3
SLC34A2	3.59E-29	0.319644589	0.382	0.253	8.66E-25	1.3
TMEM59	3.63E-29	0.262319392	0.442	0.305	8.75E-25	1.3
AC005863.	3.83E-29	0.10934593	0.047	0.01	9.24E-25	1.3

MGP	3.96E-29	-0.70065932	0.831	0.881	9.55E-25	1.3
FAAH2	8.29E-29	0.313473351	0.294	0.183	2.00E-24	1.3
GABRP	8.74E-29	-0.442211348	0.487	0.571	2.11E-24	1.3
RNF13	1.50E-28	0.294501897	0.395	0.267	3.62E-24	1.3
GK5	1.71E-28	0.221123901	0.118	0.051	4.13E-24	1.3
ATP5E	1.75E-28	0.319143858	0.555	0.427	4.21E-24	1.3
EPB41L4A	1.81E-28	0.194194008	0.082	0.029	4.38E-24	1.3
KMT2C	2.25E-28	-0.499701512	0.58	0.626	5.43E-24	1.3
ZNF385D	3.94E-28	0.264628239	0.116	0.049	9.49E-24	1.3
BACH1	4.09E-28	0.310513589	0.4	0.274	9.85E-24	1.3
SOX5	4.85E-28	0.243609404	0.079	0.027	1.17E-23	1.3
MXD1	5.54E-28	-0.467451957	0.091	0.184	1.33E-23	1.3
SERPINE2	7.52E-28	0.230756824	0.097	0.038	1.81E-23	1.3
TNFRSF11E	7.73E-28	0.350721365	0.165	0.084	1.86E-23	1.3
S100P	7.81E-28	0.208182789	0.068	0.021	1.88E-23	1.3
C3	8.17E-28	0.288349239	0.399	0.271	1.97E-23	1.3
NSF	8.44E-28	0.314575652	0.229	0.133	2.04E-23	1.3
ANK3	8.61E-28	0.275956825	0.449	0.308	2.08E-23	1.3
RPL24	1.01E-27	-0.23826533	0.942	0.958	2.45E-23	1.3
HSD17B12	1.02E-27	0.314034472	0.292	0.182	2.46E-23	1.3
GPRC5A	1.80E-27	-0.802617583	0.28	0.371	4.34E-23	1.3
CORO1C	1.84E-27	-0.496661668	0.162	0.261	4.45E-23	1.3
RP11-123O	1.90E-27	0.287271034	0.299	0.188	4.57E-23	1.3
MYO1B	2.08E-27	0.306576841	0.429	0.299	5.01E-23	1.3
BCL2L1	2.12E-27	0.22647888	0.155	0.077	5.11E-23	1.3
STX12	2.31E-27	-0.566446013	0.386	0.47	5.57E-23	1.3
OSMR	2.48E-27	0.238432761	0.482	0.337	5.99E-23	1.3
IRAK2	2.52E-27	0.300457031	0.21	0.118	6.08E-23	1.3
WFDC3	2.73E-27	0.198182593	0.165	0.083	6.59E-23	1.3
FAM177B	4.49E-27	0.355165897	0.757	0.603	1.08E-22	1.3
LRRC16A	4.54E-27	0.300279987	0.253	0.152	1.09E-22	1.3
PDSS2	4.69E-27	0.262159153	0.204	0.114	1.13E-22	1.3
MT-CO3	5.86E-27	0.182599153	0.992	0.995	1.41E-22	1.3
HELB	6.98E-27	0.259180673	0.186	0.1	1.68E-22	1.3
RBM3	8.73E-27	-0.431172512	0.446	0.523	2.10E-22	1.3
NPM1	8.79E-27	-0.294542847	0.902	0.915	2.12E-22	1.3
PSMA4	9.03E-27	0.40395165	0.589	0.472	2.18E-22	1.3
PTPRG	1.11E-26	0.354320577	0.31	0.201	2.69E-22	1.3
PGK1	1.15E-26	0.33781398	0.734	0.624	2.78E-22	1.3
THRB	1.15E-26	0.246615869	0.392	0.265	2.78E-22	1.3
OTUD6B-AS	1.29E-26	0.233967894	0.279	0.171	3.12E-22	1.3
NOCT	1.45E-26	-0.397392632	0.075	0.16	3.50E-22	1.3
LRBA	1.57E-26	0.284202853	0.569	0.425	3.79E-22	1.3
TNS3	1.61E-26	0.279769098	0.132	0.063	3.88E-22	1.3
CADPS2	1.86E-26	0.276916137	0.407	0.278	4.49E-22	1.3
FAM3C	2.08E-26	0.280629131	0.227	0.133	5.03E-22	1.3
RPS6	2.24E-26	-0.17896937	0.979	0.986	5.40E-22	1.3

HSBP1	2.25E-26	0.255409211	0.584	0.449	5.42E-22	1.3
TTC1	2.43E-26	0.277666868	0.42	0.296	5.85E-22	1.3
RCAN1	2.87E-26	0.244378068	0.755	0.632	6.92E-22	1.3
TMCO1	2.89E-26	0.271544817	0.422	0.298	6.96E-22	1.3
UBASH3B	3.05E-26	0.251324437	0.11	0.047	7.35E-22	1.3
MRPL33	3.19E-26	0.261103325	0.53	0.395	7.70E-22	1.3
RPL21	3.30E-26	-0.223238281	0.948	0.962	7.96E-22	1.3
ASS1	3.48E-26	0.302368142	0.23	0.136	8.39E-22	1.3
RRAS2	4.12E-26	0.279019797	0.309	0.197	9.94E-22	1.3
RPL35A	4.43E-26	-0.228895499	0.975	0.983	1.07E-21	1.3
GALNT15	6.64E-26	0.455446108	0.209	0.121	1.60E-21	1.3
PSMB5	8.52E-26	0.260082644	0.315	0.205	2.06E-21	1.3
RNASE1	9.59E-26	0.243970911	0.078	0.028	2.31E-21	1.3
PRKD1	9.96E-26	0.312244337	0.232	0.139	2.40E-21	1.3
PPM1H	1.02E-25	0.24913055	0.481	0.345	2.45E-21	1.3
ZBTB20	1.16E-25	0.273278687	0.586	0.446	2.81E-21	1.3
HTATIP2	1.17E-25	0.205743774	0.161	0.084	2.82E-21	1.3
XKR6	1.36E-25	0.291926894	0.245	0.149	3.28E-21	1.3
TPI1	2.19E-25	0.282913233	0.467	0.339	5.28E-21	1.3
PFDN5	3.09E-25	-0.264400903	0.888	0.901	7.45E-21	1.3
MAN1A1	3.09E-25	0.25551151	0.205	0.116	7.46E-21	1.3
UGP2	3.95E-25	0.253776068	0.627	0.489	9.54E-21	1.3
PPP2CB	4.08E-25	-0.47476335	0.239	0.332	9.84E-21	1.3
ATF7IP	4.35E-25	0.253207279	0.247	0.149	1.05E-20	1.3
TXNDC9	4.72E-25	0.254113164	0.215	0.124	1.14E-20	1.3
CDK14	5.05E-25	0.280131351	0.498	0.371	1.22E-20	1.3
UQCR10	5.66E-25	0.26137702	0.493	0.369	1.36E-20	1.3
KITLG	5.94E-25	0.143268173	0.055	0.016	1.43E-20	1.3
MT-ND1	6.17E-25	0.245818331	0.977	0.971	1.49E-20	1.3
RABGAP1L	6.55E-25	0.316199848	0.306	0.2	1.58E-20	1.3
PTGR1	7.87E-25	0.257400687	0.343	0.231	1.90E-20	1.3
FKBP5	8.74E-25	0.302250792	0.37	0.252	2.11E-20	1.3
HDAC9	8.94E-25	0.261147679	0.318	0.206	2.16E-20	1.3
NACA	1.10E-24	-0.22044048	0.951	0.967	2.65E-20	1.3
ETV6	1.12E-24	-0.362435658	0.648	0.689	2.69E-20	1.3
HMGB1	1.21E-24	0.268788052	0.63	0.509	2.92E-20	1.3
NMT1	1.26E-24	0.254813204	0.267	0.165	3.03E-20	1.3
MAGED1	1.39E-24	0.249664262	0.166	0.089	3.36E-20	1.3
ARHGAP32	1.45E-24	0.256190682	0.368	0.249	3.51E-20	1.3
EIF3E	2.28E-24	-0.32190458	0.784	0.803	5.51E-20	1.3
CD2AP	2.47E-24	-0.534872947	0.319	0.407	5.95E-20	1.3
PLEKHA7	2.60E-24	-0.155772075	0.977	0.987	6.27E-20	1.3
PSMC1	5.04E-24	0.305047401	0.45	0.333	1.21E-19	1.3
VPS25	5.83E-24	0.205590431	0.19	0.107	1.41E-19	1.3
SMC1A	7.01E-24	0.184838802	0.129	0.062	1.69E-19	1.3
PSMB7	9.67E-24	0.327111818	0.659	0.557	2.33E-19	1.3
PLEKHA5	9.75E-24	0.259130089	0.521	0.386	2.35E-19	1.3

NAV2	1.00E-23	-0.556741169	0.367	0.46	2.41E-19	1.3
PTPN2	1.05E-23	0.231040279	0.473	0.339	2.53E-19	1.3
INSIG1	1.06E-23	0.214059663	0.164	0.088	2.56E-19	1.3
SPDYE2	1.37E-23	0.226461156	0.263	0.165	3.31E-19	1.3
INSR	1.44E-23	-0.396397243	0.607	0.666	3.48E-19	1.3
TKT	1.55E-23	0.247233854	0.197	0.114	3.75E-19	1.3
SLC47A1	1.76E-23	-0.202655504	0.933	0.951	4.23E-19	1.3
CDC42BPA	2.12E-23	0.278820435	0.371	0.258	5.12E-19	1.3
KIZ-AS1	2.23E-23	-0.310600282	0.866	0.899	5.38E-19	1.3
SSBP1	2.25E-23	0.31422255	0.532	0.412	5.44E-19	1.3
ANO10	2.34E-23	0.257798365	0.314	0.208	5.64E-19	1.3
PCNXL4	2.59E-23	0.208257943	0.155	0.082	6.25E-19	1.3
CXCL6	2.93E-23	0.25911732	0.072	0.026	7.05E-19	1.3
MTHFD2L	2.99E-23	0.177150197	0.721	0.58	7.20E-19	1.3
MRPL50	3.07E-23	0.181100754	0.199	0.114	7.40E-19	1.3
MMADHC	3.78E-23	0.238689417	0.466	0.344	9.12E-19	1.3
CTDSPL	3.85E-23	0.174762096	0.107	0.048	9.29E-19	1.3
CLLU1OS	3.86E-23	0.232390906	0.141	0.073	9.31E-19	1.3
MEF2A	4.62E-23	-0.516735764	0.325	0.409	1.11E-18	1.3
N4BP2L2	4.74E-23	0.202582545	0.825	0.689	1.14E-18	1.3
ARID5B	5.06E-23	-0.429973651	0.726	0.752	1.22E-18	1.3
ANXA7	5.18E-23	0.245153135	0.378	0.266	1.25E-18	1.3
ABRACL	5.27E-23	0.257212596	0.474	0.355	1.27E-18	1.3
ENOSF1	5.49E-23	0.242434743	0.297	0.195	1.32E-18	1.3
LINC01191	5.65E-23	0.197595288	0.098	0.042	1.36E-18	1.3
PSMC5	5.93E-23	0.173867169	0.156	0.082	1.43E-18	1.3
CTSC	6.06E-23	0.162104947	0.096	0.041	1.46E-18	1.3
HIST1H2BJ	6.52E-23	0.21350971	0.144	0.074	1.57E-18	1.3
SNX24	7.14E-23	0.261685212	0.279	0.182	1.72E-18	1.3
PSMD11	7.36E-23	0.274052919	0.386	0.273	1.77E-18	1.3
ITPR2	7.83E-23	0.215613012	0.642	0.511	1.89E-18	1.3
STEAP1B	8.68E-23	-0.345455959	0.944	0.957	2.09E-18	1.3
RBFOX2	9.14E-23	-0.307407979	0.956	0.966	2.20E-18	1.3
CTD-2015G	9.24E-23	0.218707138	0.251	0.157	2.23E-18	1.3
PTPRJ	9.60E-23	-0.476727917	0.256	0.345	2.31E-18	1.3
SLC6A14	9.81E-23	0.299444877	0.287	0.187	2.37E-18	1.3
DHX32	1.01E-22	0.26265564	0.293	0.194	2.43E-18	1.3
ARRDC3	1.08E-22	0.195355523	0.737	0.62	2.60E-18	1.3
OLMALINC	1.09E-22	0.150614803	0.077	0.029	2.63E-18	1.3
SRSF5	1.13E-22	0.239359103	0.408	0.29	2.72E-18	1.3
RNF19A	1.23E-22	-0.497544765	0.582	0.639	2.96E-18	1.3
NIN	1.37E-22	0.212554604	0.156	0.085	3.31E-18	1.3
GTF2H5	1.50E-22	0.240752126	0.3	0.199	3.62E-18	1.3
UBL5	1.67E-22	0.258051695	0.466	0.349	4.02E-18	1.3
KNOP1	1.83E-22	0.214503119	0.232	0.142	4.42E-18	1.3
ZNF521	2.38E-22	0.216806233	0.254	0.16	5.74E-18	1.3
RIOK3	2.44E-22	-0.455200255	0.232	0.32	5.88E-18	1.3

RUNX1	2.60E-22	0.228590458	0.666	0.529	6.27E-18	1.3
SLC6A9	2.80E-22	0.114289724	0.046	0.012	6.74E-18	1.3
SYTL2	2.87E-22	0.249514324	0.205	0.122	6.92E-18	1.3
ANKS1B	2.94E-22	0.247002084	0.356	0.243	7.10E-18	1.3
PLEKHA6	3.01E-22	-0.223082745	0.916	0.926	7.25E-18	1.3
SRP9	3.12E-22	0.238539478	0.502	0.377	7.52E-18	1.3
CAMKMT	3.19E-22	0.265952622	0.227	0.14	7.69E-18	1.3
NUP153	3.19E-22	-0.44632724	0.205	0.293	7.69E-18	1.3
ACSS2	3.63E-22	0.23845196	0.248	0.155	8.75E-18	1.3
ROPN1B	3.76E-22	-0.383979989	0.077	0.151	9.06E-18	1.3
TMEM87B	4.54E-22	0.215895008	0.137	0.07	1.09E-17	1.3
XRCC5	4.75E-22	0.256778137	0.44	0.324	1.14E-17	1.3
KIAA0922	7.46E-22	-0.666673823	0.197	0.283	1.80E-17	1.3
TLDC1	7.59E-22	0.186984327	0.16	0.088	1.83E-17	1.3
THSD4	8.58E-22	0.322297788	0.503	0.386	2.07E-17	1.3
CASC15	8.63E-22	-0.538401287	0.282	0.378	2.08E-17	1.3
DHRS7	9.61E-22	0.166117589	0.096	0.043	2.32E-17	1.3
CDC26	1.14E-21	0.212927052	0.296	0.196	2.76E-17	1.3
TLN2	1.29E-21	0.27911507	0.157	0.087	3.11E-17	1.3
IGFBP5	1.35E-21	0.27889292	0.107	0.051	3.25E-17	1.3
TMEM167A	1.39E-21	0.257308011	0.345	0.24	3.34E-17	1.3
CYB5R2	1.52E-21	0.159206891	0.103	0.048	3.66E-17	1.3
PPP2R2A	1.61E-21	-0.440192639	0.355	0.429	3.87E-17	1.3
ILF2	1.76E-21	-0.41851023	0.518	0.577	4.24E-17	1.3
RPL38	2.13E-21	-0.211545222	0.926	0.929	5.13E-17	1.3
RAB11FIP1	2.31E-21	-0.514051978	0.469	0.532	5.57E-17	1.3
AC026202.	2.42E-21	-0.402001254	0.132	0.218	5.82E-17	1.3
DDX21	2.43E-21	-0.471662268	0.599	0.635	5.85E-17	1.3
DAZAP2	2.60E-21	0.223218336	0.461	0.345	6.27E-17	1.3
SSH2	2.82E-21	0.26745181	0.425	0.309	6.80E-17	1.3
ANKRD36	3.00E-21	0.281542104	0.204	0.123	7.23E-17	1.3
LINC00511	3.09E-21	0.237943279	0.177	0.101	7.44E-17	1.3
ARHGAP17	3.10E-21	-0.437730853	0.156	0.239	7.48E-17	1.3
VPS13D	3.15E-21	0.231391972	0.414	0.296	7.60E-17	1.3
EPRS	3.26E-21	0.198475608	0.215	0.13	7.87E-17	1.3
LINC00693	3.29E-21	0.15546337	0.054	0.017	7.92E-17	1.3
FAM134B	3.95E-21	0.280872654	0.212	0.13	9.52E-17	1.3
SEC24D	3.99E-21	-0.457454688	0.234	0.321	9.63E-17	1.3
SERGEF	4.07E-21	0.136260309	0.067	0.025	9.81E-17	1.3
PSMD1	4.43E-21	0.216383232	0.202	0.121	1.07E-16	1.3
LINC00152	4.48E-21	-0.589003808	0.289	0.362	1.08E-16	1.3
GNE	4.58E-21	0.242146645	0.244	0.155	1.10E-16	1.3
ESYT2	4.73E-21	-0.47129167	0.463	0.528	1.14E-16	1.3
FNBP1L	5.56E-21	-0.493515254	0.38	0.453	1.34E-16	1.3
HSD17B7	5.62E-21	0.244946426	0.17	0.097	1.36E-16	1.3
RP11-314N	6.17E-21	-0.449815339	0.089	0.166	1.49E-16	1.3
JMJD1C	6.95E-21	-0.365480209	0.648	0.689	1.68E-16	1.3

SHFM1	6.98E-21	0.24551159	0.706	0.602	1.68E-16	1.3
CHCHD7	7.10E-21	0.16904946	0.118	0.058	1.71E-16	1.3
MECOM	7.51E-21	-0.251187209	0.883	0.89	1.81E-16	1.3
UBAC2	7.59E-21	0.231428004	0.284	0.19	1.83E-16	1.3
MACC1	7.67E-21	-0.568071056	0.342	0.425	1.85E-16	1.3
CNIH4	7.74E-21	0.212863872	0.472	0.351	1.87E-16	1.3
AC159540.	8.00E-21	0.282713829	0.241	0.152	1.93E-16	1.3
RAP1GAP2	8.35E-21	0.284813927	0.231	0.146	2.01E-16	1.3
ZNF33A	8.62E-21	0.228489905	0.326	0.223	2.08E-16	1.3
ELOVL6	1.16E-20	0.200766795	0.13	0.067	2.80E-16	1.3
ATF3	1.21E-20	-0.533907837	0.283	0.365	2.92E-16	1.3
ELL2	1.22E-20	-0.321065063	0.645	0.694	2.94E-16	1.3
CCDC91	1.22E-20	0.209019304	0.443	0.326	2.94E-16	1.3
TMSB10	1.54E-20	0.301592132	0.695	0.604	3.72E-16	1.3
KIAA1551	1.56E-20	0.199356039	0.192	0.113	3.76E-16	1.3
COMMD10	1.71E-20	0.252594499	0.281	0.186	4.12E-16	1.3
CCT5	1.88E-20	0.250123582	0.462	0.346	4.54E-16	1.3
DOCK5	2.06E-20	0.239550901	0.292	0.194	4.96E-16	1.3
COX20	2.29E-20	0.174950525	0.165	0.093	5.53E-16	1.3
AGFG1	2.33E-20	-0.41328613	0.348	0.43	5.61E-16	1.3
STIM1	2.37E-20	0.207001522	0.279	0.183	5.71E-16	1.3
CNTNAP3B	2.37E-20	0.193275615	0.167	0.095	5.73E-16	1.3
SCNN1A	2.43E-20	0.185397878	0.121	0.061	5.86E-16	1.3
QKI	2.44E-20	-0.463489526	0.492	0.551	5.89E-16	1.3
CAPZA1	2.66E-20	0.227833896	0.535	0.416	6.41E-16	1.3
PHF21A	2.67E-20	0.256521131	0.317	0.22	6.43E-16	1.3
JKAMP	2.72E-20	0.195591937	0.129	0.067	6.55E-16	1.3
MAPK14	3.09E-20	0.227034024	0.244	0.156	7.45E-16	1.3
METTL17	3.20E-20	-0.250915244	0.024	0.078	7.72E-16	1.3
RSL1D1	3.27E-20	-0.375406034	0.603	0.639	7.88E-16	1.3
RPL11	3.29E-20	-0.188448421	0.972	0.983	7.94E-16	1.3
ORMDL2	3.44E-20	0.200235675	0.226	0.142	8.29E-16	1.3
BRK1	3.72E-20	0.230641974	0.761	0.668	8.97E-16	1.3
SOX10	3.99E-20	0.225687463	0.184	0.109	9.62E-16	1.3
SAMD12	4.27E-20	0.227888449	0.341	0.236	1.03E-15	1.3
ROPN1	4.46E-20	-0.245099289	0.026	0.081	1.07E-15	1.3
OSBPL3	4.77E-20	0.179441681	0.101	0.048	1.15E-15	1.3
LIMCH1	4.82E-20	0.222660113	0.348	0.241	1.16E-15	1.3
ST6GALNA(	5.16E-20	0.243858239	0.476	0.358	1.24E-15	1.3
PDIA6	5.33E-20	-0.440584394	0.186	0.266	1.29E-15	1.3
AGAP1	5.81E-20	0.169638121	0.72	0.583	1.40E-15	1.3
SGPP2	6.44E-20	0.22376845	0.259	0.169	1.55E-15	1.3
MFGE8	6.46E-20	0.378819814	0.252	0.166	1.56E-15	1.3
CFL1	7.41E-20	0.298122711	0.315	0.219	1.79E-15	1.3
PSMD7	8.04E-20	0.196732634	0.209	0.129	1.94E-15	1.3
MTIF3	8.11E-20	0.23565662	0.316	0.218	1.96E-15	1.3
RAD23B	9.19E-20	-0.471166931	0.371	0.437	2.22E-15	1.3

SCMH1	9.82E-20	0.263440645	0.287	0.192	2.37E-15	1.3
ST6GAL1	1.14E-19	0.230848664	0.422	0.308	2.75E-15	1.3
HDAC8	1.35E-19	0.233537113	0.3	0.202	3.25E-15	1.3
SEMA4B	1.40E-19	0.250403674	0.225	0.144	3.37E-15	1.3
ERH	1.45E-19	0.193298111	0.658	0.535	3.49E-15	1.3
DOCK7	1.66E-19	0.223772515	0.316	0.219	4.00E-15	1.3
IFITM3	1.73E-19	0.272780655	0.328	0.229	4.18E-15	1.3
FANCC	2.09E-19	0.198817177	0.185	0.11	5.05E-15	1.3
RPL32	2.11E-19	-0.197379169	0.979	0.987	5.08E-15	1.3
RIN2	2.13E-19	0.234441866	0.392	0.284	5.14E-15	1.3
TCF12	2.13E-19	0.210509323	0.587	0.467	5.15E-15	1.3
OOEP	2.41E-19	-0.172668397	0.972	0.984	5.82E-15	1.3
TBC1D4	2.52E-19	0.251853122	0.2	0.123	6.07E-15	1.3
ATG5	2.53E-19	0.1769987	0.163	0.094	6.11E-15	1.3
NEAT1	2.83E-19	0.147143023	0.993	0.955	6.82E-15	1.3
PSMB2	2.98E-19	0.208049058	0.195	0.12	7.18E-15	1.3
MITD1	3.00E-19	0.193566135	0.168	0.098	7.23E-15	1.3
SERPINB3	3.13E-19	0.322505831	0.129	0.069	7.54E-15	1.3
DENND2D	3.13E-19	0.196521367	0.19	0.116	7.56E-15	1.3
UBE2L3	3.17E-19	0.243624581	0.537	0.426	7.64E-15	1.3
PDCD10	3.45E-19	0.212744163	0.368	0.26	8.32E-15	1.3
ITFG1	3.58E-19	0.261566863	0.316	0.221	8.64E-15	1.3
ENO1	3.62E-19	0.373825544	0.596	0.488	8.72E-15	1.3
PRICKLE1	4.42E-19	0.175074385	0.155	0.087	1.06E-14	1.3
FBXO2	4.64E-19	0.115506865	0.049	0.016	1.12E-14	1.3
HMGCR	5.64E-19	0.166732357	0.104	0.05	1.36E-14	1.3
S100A8	6.02E-19	0.198430362	0.282	0.194	1.45E-14	1.3
VNN3	6.34E-19	0.218746866	0.351	0.244	1.53E-14	1.3
PDE7A	6.42E-19	0.232429549	0.339	0.238	1.55E-14	1.3
MAST4	6.56E-19	0.153032422	0.475	0.352	1.58E-14	1.3
YTHDC1	6.73E-19	-0.391073264	0.222	0.303	1.62E-14	1.3
IGF2BP2	7.79E-19	-0.512821312	0.473	0.524	1.88E-14	1.3
SLC25A3	7.85E-19	0.185517908	0.263	0.174	1.89E-14	1.3
EIF4A2	7.91E-19	-0.271884274	0.788	0.822	1.91E-14	1.3
BTAF1	8.05E-19	-0.386094333	0.186	0.268	1.94E-14	1.3
TCEB1	9.04E-19	0.19714182	0.375	0.268	2.18E-14	1.3
SUB1	9.58E-19	0.226062656	0.809	0.735	2.31E-14	1.3
LDLRAD4	9.94E-19	0.271459248	0.237	0.153	2.40E-14	1.3
SLC11A2	1.09E-18	0.207687965	0.518	0.399	2.64E-14	1.3
RAPGEF2	1.13E-18	-0.464725986	0.355	0.425	2.73E-14	1.3
AFTPH	1.17E-18	-0.408657759	0.258	0.335	2.83E-14	1.3
CCT3	1.19E-18	0.204589015	0.529	0.411	2.88E-14	1.3
USP54	1.23E-18	-0.467703488	0.471	0.528	2.98E-14	1.3
FAM213A	1.27E-18	0.174806877	0.122	0.064	3.05E-14	1.3
C2orf88	1.31E-18	-0.393457604	0.33	0.406	3.15E-14	1.3
ZBTB8OS	1.33E-18	0.186862289	0.194	0.119	3.21E-14	1.3
SPATA5	1.42E-18	0.189912697	0.191	0.116	3.42E-14	1.3

UBE2V2	1.46E-18	0.189212405	0.368	0.263	3.52E-14	1.3
RTTN	1.47E-18	0.185417854	0.107	0.054	3.54E-14	1.3
ATG7	1.62E-18	0.212309958	0.291	0.198	3.91E-14	1.3
C15orf52	1.72E-18	0.137595582	0.079	0.035	4.15E-14	1.3
MIR4435-2	1.84E-18	-0.52541931	0.444	0.499	4.44E-14	1.3
ATP6V1E1	2.10E-18	0.23465898	0.352	0.254	5.07E-14	1.3
APBB2	2.13E-18	0.21102825	0.156	0.09	5.14E-14	1.3
GLUL	2.15E-18	0.182625116	0.408	0.296	5.19E-14	1.3
TIMP1	2.28E-18	0.178454661	0.096	0.046	5.49E-14	1.3
SEMA6A	2.39E-18	0.26217075	0.503	0.384	5.76E-14	1.3
STARD13	2.52E-18	0.157370416	0.27	0.18	6.08E-14	1.3
PSAT1	2.62E-18	0.104578553	0.046	0.015	6.32E-14	1.3
PERP	2.82E-18	0.22849749	0.406	0.302	6.80E-14	1.3
FTH1	3.06E-18	0.281167546	0.915	0.86	7.37E-14	1.3
SNRNP35	3.13E-18	0.166677053	0.125	0.067	7.54E-14	1.3
ANKRD36B	3.16E-18	0.232731106	0.169	0.1	7.61E-14	1.3
FMN1	3.42E-18	0.203093938	0.228	0.148	8.26E-14	1.3
EPB41L1	3.54E-18	0.194977941	0.152	0.087	8.53E-14	1.3
ADARB1	3.68E-18	0.163237406	0.095	0.046	8.88E-14	1.3
INVS	3.87E-18	0.213974526	0.187	0.115	9.34E-14	1.3
CD9	4.02E-18	0.244967275	0.325	0.231	9.69E-14	1.3
SDCBP	4.03E-18	0.280942558	0.779	0.694	9.73E-14	1.3
RP11-903H	4.15E-18	0.12677677	0.042	0.012	1.00E-13	1.3
ZNF608	4.16E-18	0.219887163	0.225	0.145	1.00E-13	1.3
CEBPZOS	4.19E-18	0.203518458	0.199	0.124	1.01E-13	1.3
UAP1	4.30E-18	0.242416886	0.311	0.218	1.04E-13	1.3
HK2	4.65E-18	-0.389895244	0.117	0.19	1.12E-13	1.3
NPEPPS	4.72E-18	-0.499591869	0.405	0.469	1.14E-13	1.3
HIP1	4.90E-18	0.202195983	0.137	0.076	1.18E-13	1.3
FAM208A	5.20E-18	0.160576949	0.111	0.058	1.25E-13	1.3
VMP1	5.23E-18	0.217684031	0.838	0.759	1.26E-13	1.3
DNMBP	5.43E-18	0.136346415	0.068	0.028	1.31E-13	1.3
CASC4	5.70E-18	0.204181966	0.248	0.163	1.38E-13	1.3
MT-ND6	6.27E-18	0.181829377	0.221	0.142	1.51E-13	1.3
SLMAP	6.28E-18	-0.339277009	0.594	0.643	1.51E-13	1.3
PPP1R13B	6.30E-18	0.218455933	0.187	0.115	1.52E-13	1.3
S100A9	6.44E-18	0.438499658	0.107	0.055	1.55E-13	1.3
PRIM2	6.92E-18	0.230813322	0.119	0.063	1.67E-13	1.3
C5orf46	7.02E-18	0.213420531	0.298	0.207	1.69E-13	1.3
HSDL2	7.81E-18	0.176075145	0.155	0.09	1.88E-13	1.3
RSRC2	8.44E-18	-0.424477983	0.581	0.611	2.03E-13	1.3
PARD3B	8.64E-18	0.195582791	0.186	0.113	2.08E-13	1.3
LLPH	9.01E-18	0.150035279	0.191	0.116	2.17E-13	1.3
RPS29	9.26E-18	-0.205970413	0.958	0.968	2.23E-13	1.3
ACBD6	9.80E-18	0.224589925	0.11	0.057	2.36E-13	1.3
C1D	1.04E-17	0.156872546	0.193	0.119	2.51E-13	1.3
HIST1H2BD	1.07E-17	0.16540926	0.357	0.256	2.57E-13	1.3

CTCF	1.07E-17	0.144712856	0.116	0.061	2.59E-13	1.3
BCL2A1	1.09E-17	-0.564962938	0.105	0.176	2.63E-13	1.3
GAPDH	1.23E-17	0.365821566	0.682	0.578	2.95E-13	1.3
THADA	1.34E-17	0.271821248	0.345	0.249	3.23E-13	1.3
TMEM33	1.41E-17	0.166730954	0.137	0.076	3.39E-13	1.3
PHLPP1	1.42E-17	-0.496429009	0.418	0.474	3.42E-13	1.3
CHKA	1.49E-17	0.217319627	0.176	0.107	3.59E-13	1.3
CD83	1.49E-17	-0.338177836	0.055	0.115	3.60E-13	1.3
PROM1	1.50E-17	0.177432225	0.549	0.426	3.62E-13	1.3
KLHL2	1.52E-17	0.184660231	0.105	0.054	3.67E-13	1.3
RHOJ	1.61E-17	0.119024051	0.062	0.025	3.89E-13	1.3
CYLD	1.66E-17	-0.340814383	0.181	0.262	3.99E-13	1.3
SMS	1.72E-17	-0.600977211	0.349	0.404	4.15E-13	1.3
GCNT2	1.81E-17	0.172641018	0.45	0.335	4.36E-13	1.3
NEURL3	1.90E-17	0.178302841	0.099	0.049	4.57E-13	1.3
CEBPB	2.05E-17	0.229649059	0.2	0.127	4.93E-13	1.3
JPX	2.07E-17	0.188108041	0.331	0.234	5.00E-13	1.3
ASCC1	2.22E-17	0.200604129	0.231	0.152	5.36E-13	1.3
S100B	2.31E-17	-0.220620126	0.019	0.064	5.57E-13	1.3
TES	2.38E-17	-0.382513785	0.267	0.34	5.73E-13	1.3
TBC1D22A	2.46E-17	0.244898646	0.183	0.113	5.93E-13	1.3
KIAA0319L	2.54E-17	0.253847198	0.22	0.145	6.13E-13	1.3
PPM1L	2.65E-17	0.200142832	0.114	0.06	6.40E-13	1.3
FAM63B	2.67E-17	0.190245989	0.184	0.114	6.45E-13	1.3
NIPAL3	2.92E-17	0.164146048	0.12	0.065	7.05E-13	1.3
RND3	3.01E-17	-0.407316424	0.242	0.321	7.26E-13	1.3
PARP1	3.03E-17	0.158254569	0.132	0.074	7.30E-13	1.3
BTD	3.28E-17	0.127020193	0.092	0.044	7.91E-13	1.3
DST	3.39E-17	0.222576145	0.669	0.569	8.17E-13	1.3
NEBL	3.45E-17	0.244612785	0.591	0.472	8.31E-13	1.3
CSGALNAC	3.88E-17	0.243927111	0.133	0.075	9.35E-13	1.3
NDUFB3	3.92E-17	0.172641401	0.284	0.195	9.45E-13	1.3
BTBD11	4.50E-17	0.226248951	0.116	0.062	1.09E-12	1.3
MORC3	4.52E-17	-0.360179941	0.17	0.244	1.09E-12	1.3
PLEKHS1	4.75E-17	0.1923333	0.333	0.237	1.15E-12	1.3
TNFRSF21	4.83E-17	0.159337954	0.107	0.055	1.17E-12	1.3
MRPS18C	5.13E-17	0.168695883	0.254	0.171	1.24E-12	1.3
MRPS14	5.20E-17	0.173160705	0.217	0.14	1.25E-12	1.3
TRIM2	5.30E-17	0.147985774	0.384	0.276	1.28E-12	1.3
COPS2	5.45E-17	-0.328766165	0.3	0.373	1.31E-12	1.3
VWA8	6.26E-17	0.183459465	0.104	0.054	1.51E-12	1.3
ST3GAL1	6.36E-17	0.183457985	0.255	0.172	1.53E-12	1.3
BCAP29	6.39E-17	0.191007702	0.213	0.138	1.54E-12	1.3
DLG2	6.55E-17	0.297630314	0.224	0.148	1.58E-12	1.3
C15orf41	7.45E-17	0.188767662	0.112	0.06	1.80E-12	1.3
GPR87	7.50E-17	0.138154315	0.091	0.044	1.81E-12	1.3
LIPH	7.92E-17	-0.813501577	0.44	0.479	1.91E-12	1.3

PPP2R5A	8.21E-17	0.206899006	0.202	0.13	1.98E-12	1.3
MBD5	8.63E-17	0.214396365	0.337	0.242	2.08E-12	1.3
TRAPPC9	8.98E-17	0.240041767	0.236	0.158	2.17E-12	1.3
CARD6	9.32E-17	0.163262109	0.088	0.043	2.25E-12	1.3
ACP1	9.80E-17	0.206348646	0.485	0.377	2.36E-12	1.3
SEMA6A-AS	1.00E-16	0.227234591	0.324	0.229	2.42E-12	1.3
PARP4	1.03E-16	0.220268672	0.257	0.175	2.49E-12	1.3
C11orf80	1.05E-16	0.208423128	0.283	0.197	2.53E-12	1.3
VTI1A	1.05E-16	0.257104088	0.225	0.148	2.54E-12	1.3
SLC9A7	1.08E-16	-0.424047754	0.235	0.309	2.61E-12	1.3
C1GALT1C1	1.09E-16	0.121036921	0.079	0.036	2.63E-12	1.3
PSMA3	1.11E-16	0.260623899	0.517	0.413	2.67E-12	1.3
FLRT3	1.14E-16	0.11639784	0.049	0.017	2.74E-12	1.3
KCMF1	1.17E-16	-0.42631598	0.251	0.32	2.83E-12	1.3
CXCL8	1.19E-16	0.274074552	0.428	0.334	2.87E-12	1.3
SLC36A4	1.24E-16	0.142770111	0.108	0.057	2.99E-12	1.3
MGAM2	1.32E-16	0.200540729	0.419	0.311	3.19E-12	1.3
UBE4B	1.32E-16	0.173165014	0.276	0.19	3.19E-12	1.3
RAB31	1.34E-16	0.199862819	0.156	0.093	3.22E-12	1.3
KDM5B	1.42E-16	-0.368798706	0.531	0.568	3.42E-12	1.3
S100A10	1.43E-16	0.253003249	0.384	0.283	3.46E-12	1.3
EPB42	1.45E-16	-0.250292122	0.907	0.934	3.50E-12	1.3
CRY1	1.49E-16	-0.478433863	0.288	0.363	3.59E-12	1.3
GPRIN3	1.52E-16	0.132412349	0.058	0.023	3.65E-12	1.3
VBP1	1.59E-16	0.182296087	0.206	0.133	3.83E-12	1.3
RFWD2	1.65E-16	0.21435313	0.368	0.268	3.97E-12	1.3
SYNE2	1.67E-16	0.190430866	0.599	0.479	4.02E-12	1.3
HNRNPA2B	1.78E-16	0.236127615	0.502	0.395	4.30E-12	1.3
CPEB3	1.82E-16	-0.507960722	0.282	0.352	4.38E-12	1.3
TFB1M	2.10E-16	0.143084409	0.098	0.05	5.05E-12	1.3
CBX1	2.21E-16	-0.295001803	0.092	0.157	5.33E-12	1.3
GNG12	2.23E-16	-0.393578911	0.25	0.323	5.37E-12	1.3
SLC4A7	2.28E-16	0.223493199	0.408	0.311	5.49E-12	1.3
ACLY	2.28E-16	0.12526465	0.157	0.093	5.51E-12	1.3
PRR13	2.38E-16	0.154746148	0.129	0.073	5.73E-12	1.3
WWOX	2.48E-16	0.308030895	0.286	0.202	5.98E-12	1.3
FRMD5	2.69E-16	0.199107137	0.226	0.15	6.49E-12	1.3
THSD4-AS1	2.73E-16	0.174419245	0.553	0.435	6.59E-12	1.3
ELF1	2.75E-16	-0.382600899	0.398	0.46	6.62E-12	1.3
HIST1H4H	3.01E-16	0.144670494	0.109	0.058	7.27E-12	1.3
ITGB6	3.77E-16	-0.345608184	0.255	0.335	9.08E-12	1.3
PSMA5	3.95E-16	0.176696407	0.281	0.196	9.53E-12	1.3
MEIS2	4.22E-16	0.216263458	0.324	0.232	1.02E-11	1.3
PPTC7	4.24E-16	-0.387417513	0.161	0.236	1.02E-11	1.3
MRPL51	4.26E-16	0.224042535	0.502	0.399	1.03E-11	1.3
UQCRB	4.38E-16	-0.235120306	0.813	0.832	1.06E-11	1.3
CNN3	4.39E-16	-0.443793813	0.424	0.475	1.06E-11	1.3

FOCAD	4.58E-16	0.208728011	0.238	0.16	1.10E-11	1.3
DPYD-AS1	4.59E-16	0.180391762	0.134	0.076	1.11E-11	1.3
LYN	4.79E-16	0.106235439	0.8	0.65	1.16E-11	1.3
CYB5B	4.80E-16	0.166184193	0.22	0.144	1.16E-11	1.3
TFCP2	4.95E-16	0.170955768	0.188	0.119	1.19E-11	1.3
FOXN3	5.43E-16	0.211047205	0.297	0.211	1.31E-11	1.3
EXT1	5.49E-16	-0.417442818	0.527	0.575	1.32E-11	1.3
GULP1	5.58E-16	0.188461588	0.249	0.169	1.35E-11	1.3
MAP1B	5.61E-16	0.165546792	0.491	0.38	1.35E-11	1.3
PTPN4	5.73E-16	0.182347763	0.134	0.077	1.38E-11	1.3
TM4SF1	6.05E-16	-0.68641213	0.889	0.9	1.46E-11	1.3
TALDO1	6.05E-16	0.179560247	0.123	0.069	1.46E-11	1.3
YBX1	6.50E-16	0.221102178	0.413	0.316	1.57E-11	1.3
ARHGAP24	6.67E-16	0.201453373	0.08	0.038	1.61E-11	1.3
HES1	7.02E-16	0.212506804	0.314	0.226	1.69E-11	1.3
CYP20A1	7.43E-16	0.155265895	0.107	0.057	1.79E-11	1.3
GLYATL2	7.50E-16	0.127348404	0.113	0.061	1.81E-11	1.3
PLOD2	7.59E-16	0.206327263	0.215	0.141	1.83E-11	1.3
EIF2S2	7.80E-16	0.190063628	0.343	0.251	1.88E-11	1.3
GABARAP	8.12E-16	-0.393482712	0.25	0.323	1.96E-11	1.3
KMT2A	8.28E-16	-0.308211752	0.347	0.42	2.00E-11	1.3
UBR5	8.48E-16	-0.343732672	0.355	0.427	2.04E-11	1.3
IFRD1	8.53E-16	-0.411667813	0.328	0.397	2.06E-11	1.3
LARP7	8.56E-16	0.166699143	0.231	0.155	2.06E-11	1.3
FAF2	9.03E-16	0.165281976	0.228	0.152	2.18E-11	1.3
NFAT5	9.08E-16	-0.311253585	0.597	0.636	2.19E-11	1.3
NARS2	9.23E-16	0.160093894	0.099	0.052	2.23E-11	1.3
OSBPL10	9.53E-16	0.230164566	0.185	0.117	2.30E-11	1.3
HSP90AB1	9.53E-16	-0.220145483	0.928	0.939	2.30E-11	1.3
EMC3	9.73E-16	0.167270788	0.212	0.138	2.35E-11	1.3
HIST1H2BG	1.08E-15	0.150997933	0.153	0.092	2.60E-11	1.3
STOX2	1.13E-15	0.176665403	0.237	0.158	2.72E-11	1.3
MEAF6	1.16E-15	-0.313751008	0.099	0.163	2.80E-11	1.3
PPP1R7	1.16E-15	0.11419964	0.081	0.038	2.81E-11	1.3
MMP7	1.26E-15	0.325065806	0.38	0.289	3.04E-11	1.3
RPRD2	1.35E-15	0.21433919	0.203	0.132	3.26E-11	1.3
LSS	1.39E-15	0.144922498	0.081	0.039	3.36E-11	1.3
MGA	1.45E-15	0.165683055	0.107	0.057	3.49E-11	1.3
PRDM1	1.74E-15	0.163499748	0.104	0.056	4.19E-11	1.3
CKS1B	1.86E-15	0.209057526	0.467	0.364	4.48E-11	1.3
DCAF13	1.87E-15	0.14637281	0.125	0.071	4.50E-11	1.3
PPP1R9A	1.91E-15	0.19289214	0.265	0.186	4.60E-11	1.3
SIPA1L3	1.95E-15	0.202560856	0.237	0.16	4.71E-11	1.3
C1QTNF3-A	2.00E-15	0.197900158	0.279	0.196	4.83E-11	1.3
TAGLN2	2.01E-15	0.218150511	0.244	0.166	4.84E-11	1.3
RP11-634B	2.04E-15	0.105473506	0.044	0.016	4.93E-11	1.3
C11orf54	2.12E-15	0.153722172	0.144	0.085	5.11E-11	1.3

MYH9	2.13E-15	-0.281951073	0.435	0.507	5.13E-11	1.3
NCBP2	2.26E-15	0.150705946	0.143	0.085	5.45E-11	1.3
PSMD13	2.43E-15	0.144139939	0.118	0.066	5.86E-11	1.3
CD63	2.44E-15	0.266746066	0.273	0.195	5.88E-11	1.3
BRE	2.46E-15	0.182948049	0.229	0.154	5.93E-11	1.3
FIGN	2.62E-15	0.228306357	0.182	0.116	6.31E-11	1.3
RP11-519G	3.46E-15	-0.311568348	0.053	0.106	8.33E-11	1.3
RP11-37B2	3.53E-15	-0.426004549	0.24	0.311	8.50E-11	1.3
LYRM2	3.68E-15	0.13141411	0.156	0.094	8.87E-11	1.3
ACTR6	3.69E-15	0.135119799	0.23	0.153	8.89E-11	1.3
HELZ	3.71E-15	-0.369108704	0.272	0.344	8.95E-11	1.3
TNRC18	3.80E-15	0.158188288	0.094	0.049	9.16E-11	1.3
PLD1	4.01E-15	0.143229953	0.141	0.083	9.66E-11	1.3
CLASP2	4.09E-15	0.175890402	0.297	0.21	9.86E-11	1.3
TCEB2	4.09E-15	0.200609618	0.069	0.032	9.87E-11	1.3
SGPL1	4.10E-15	0.140174388	0.166	0.103	9.89E-11	1.3
SNRPD3	4.17E-15	0.186043256	0.423	0.325	1.00E-10	1.3
CAP2	4.28E-15	0.183621634	0.139	0.083	1.03E-10	1.3
FAM114A1	4.47E-15	0.206472407	0.2	0.132	1.08E-10	1.3
MLLT10	4.52E-15	0.178927936	0.22	0.146	1.09E-10	1.3
MT-ND3	4.57E-15	0.134731377	0.984	0.984	1.10E-10	1.3
TEFM	4.62E-15	0.149651356	0.08	0.039	1.11E-10	1.3
BBX	4.85E-15	0.191913459	0.469	0.365	1.17E-10	1.3
VAT1	5.00E-15	0.160060509	0.168	0.105	1.20E-10	1.3
BRINP1	5.09E-15	0.21302626	0.26	0.182	1.23E-10	1.3
RORA	5.23E-15	-0.357420711	0.443	0.5	1.26E-10	1.3
STXBP5	5.32E-15	0.168740332	0.139	0.083	1.28E-10	1.3
RAPGEF5	5.50E-15	0.153858482	0.573	0.451	1.33E-10	1.3
NFATC2	5.82E-15	-0.35904062	0.098	0.162	1.40E-10	1.3
LRRC49	5.86E-15	0.187631397	0.171	0.108	1.41E-10	1.3
PSMD14	5.93E-15	0.134561323	0.103	0.055	1.43E-10	1.3
TUBA1C	6.21E-15	0.153412702	0.436	0.331	1.50E-10	1.3
GSDMC	6.45E-15	0.115889261	0.064	0.028	1.56E-10	1.3
GJA1	6.60E-15	-0.237827271	0.026	0.069	1.59E-10	1.3
TGOLN2	6.92E-15	0.14724287	0.157	0.097	1.67E-10	1.3
SLTM	7.55E-15	0.200503978	0.325	0.238	1.82E-10	1.3
PRKDC	7.78E-15	0.15436087	0.16	0.098	1.88E-10	1.3
GRIP1	8.49E-15	0.211812108	0.257	0.18	2.05E-10	1.3
BPNT1	9.54E-15	0.132232775	0.093	0.049	2.30E-10	1.3
TPT1-AS1	9.65E-15	-0.330050728	0.36	0.423	2.33E-10	1.3
CXCL1	9.99E-15	0.198765181	0.314	0.231	2.41E-10	1.3
RAD21	1.04E-14	-0.395902846	0.315	0.375	2.51E-10	1.3
LRP10	1.07E-14	0.174165433	0.154	0.095	2.59E-10	1.3
FAS	1.08E-14	-0.293449651	0.144	0.214	2.60E-10	1.3
RNF130	1.16E-14	0.183800676	0.213	0.144	2.79E-10	1.3
C10orf10	1.19E-14	0.129962296	0.256	0.176	2.86E-10	1.3
NR2F2	1.22E-14	0.155582192	0.134	0.08	2.93E-10	1.3

ATR	1.22E-14	0.157361376	0.223	0.15	2.94E-10	1.3
IFI16	1.23E-14	0.172785768	0.343	0.253	2.97E-10	1.3
PTPRE	1.29E-14	0.16266305	0.179	0.114	3.11E-10	1.3
LCN2	1.32E-14	0.261228014	0.062	0.028	3.18E-10	1.3
SUGT1	1.32E-14	0.158306526	0.205	0.135	3.19E-10	1.3
ZNF69	1.32E-14	0.13360041	0.089	0.046	3.19E-10	1.3
SPRED2	1.33E-14	0.158627621	0.163	0.102	3.21E-10	1.3
KCCAT211	1.38E-14	0.198155184	0.086	0.044	3.34E-10	1.3
CLIC6	1.40E-14	0.184055087	0.153	0.095	3.38E-10	1.3
ATE1	1.44E-14	0.139627269	0.153	0.094	3.47E-10	1.3
WDR70	1.45E-14	0.214608914	0.178	0.115	3.51E-10	1.3
RP11-96H1	1.47E-14	0.215078726	0.158	0.098	3.55E-10	1.3
SMAD1	1.49E-14	0.152571387	0.127	0.074	3.59E-10	1.3
NBR1	1.51E-14	0.177200004	0.225	0.153	3.63E-10	1.3
UBE2E3	1.56E-14	0.164222464	0.23	0.157	3.77E-10	1.3
FBLN5	1.72E-14	0.190367177	0.407	0.311	4.16E-10	1.3
PM20D2	1.74E-14	0.146480815	0.185	0.12	4.19E-10	1.3
LINC01235	1.74E-14	0.20666665	0.253	0.177	4.19E-10	1.3
GABRE	1.80E-14	0.13380126	0.097	0.052	4.35E-10	1.3
ZNF609	1.82E-14	0.192089454	0.609	0.507	4.38E-10	1.3
MBNL1	1.84E-14	0.21224418	0.504	0.399	4.43E-10	1.3
ARL6IP5	1.86E-14	0.166214019	0.568	0.461	4.47E-10	1.3
OXSR1	1.99E-14	-0.361337561	0.184	0.251	4.79E-10	1.3
NUP107	2.01E-14	0.13844862	0.205	0.136	4.84E-10	1.3
SLC28A3	2.12E-14	0.15536171	0.44	0.333	5.11E-10	1.3
C5orf63	2.25E-14	0.109560195	0.061	0.027	5.42E-10	1.3
PTRH2	2.26E-14	0.174395674	0.221	0.148	5.46E-10	1.3
AMN1	2.35E-14	0.168590285	0.176	0.113	5.67E-10	1.3
CDC42SE1	2.37E-14	0.187059016	0.207	0.138	5.71E-10	1.3
RARRES3	2.37E-14	-0.370835688	0.265	0.334	5.72E-10	1.3
LDLRAD3	2.39E-14	0.182116494	0.369	0.277	5.76E-10	1.3
DNAJC8	2.40E-14	0.158665312	0.29	0.207	5.80E-10	1.3
NSD1	2.49E-14	0.161894742	0.198	0.13	6.00E-10	1.3
FAM32A	2.52E-14	0.111657856	0.106	0.058	6.09E-10	1.3
CNTN3	2.54E-14	0.133496978	0.048	0.019	6.12E-10	1.3
BCL2L14	2.66E-14	-0.268301985	0.101	0.163	6.41E-10	1.3
ICA1	3.00E-14	0.191321034	0.351	0.26	7.24E-10	1.3
SERPINB4	3.00E-14	0.173392662	0.137	0.082	7.24E-10	1.3
ARHGEF3	3.07E-14	0.158725751	0.381	0.286	7.41E-10	1.3
RPS23	3.19E-14	-0.168729216	0.977	0.986	7.69E-10	1.3
NFATC3	3.24E-14	0.170038403	0.211	0.143	7.81E-10	1.3
STAMBP	3.30E-14	0.1731076	0.241	0.167	7.95E-10	1.3
SOCS6	3.30E-14	0.147023096	0.169	0.107	7.96E-10	1.3
RBL2	3.36E-14	0.191679603	0.138	0.083	8.11E-10	1.3
RAP2B	3.63E-14	-0.329017734	0.142	0.207	8.76E-10	1.3
MOB4	3.71E-14	0.152167023	0.256	0.178	8.95E-10	1.3
RNF217	3.73E-14	0.190242231	0.135	0.082	9.00E-10	1.3

THUMPD3-	3.75E-14	0.162943566	0.373	0.282	9.04E-10	1.3
SLC24A3	4.03E-14	0.19498773	0.215	0.144	9.72E-10	1.3
CLDN8	4.12E-14	0.125873205	0.127	0.074	9.93E-10	1.3
NFIC	4.23E-14	0.169712842	0.228	0.155	1.02E-09	1.3
XPR1	4.29E-14	0.151912711	0.168	0.107	1.03E-09	1.3
MYCBP2	4.34E-14	0.211806199	0.225	0.155	1.05E-09	1.3
FAF1	4.38E-14	0.222200773	0.335	0.248	1.06E-09	1.3
LNX1	4.42E-14	0.193706317	0.134	0.081	1.07E-09	1.3
LYPLA1	4.45E-14	0.165771193	0.15	0.093	1.07E-09	1.3
ANKIB1	4.49E-14	0.174073686	0.39	0.294	1.08E-09	1.3
BMS1	4.87E-14	0.161399064	0.145	0.089	1.17E-09	1.3
MKL2	4.91E-14	0.185406709	0.325	0.238	1.18E-09	1.3
RP11-739G	5.22E-14	-0.26459426	0.014	0.05	1.26E-09	1.3
PSMC2	5.28E-14	0.188000961	0.352	0.263	1.27E-09	1.3
FAM20C	5.34E-14	0.229853381	0.142	0.087	1.29E-09	1.3
ZNF98	5.37E-14	0.153540578	0.13	0.077	1.29E-09	1.3
RPL39	5.41E-14	-0.182432848	0.932	0.947	1.30E-09	1.3
STRN	5.55E-14	-0.371074534	0.355	0.412	1.34E-09	1.3
CLSTN1	6.06E-14	0.15959708	0.114	0.065	1.46E-09	1.3
NFE2L2	6.66E-14	0.160496303	0.431	0.331	1.61E-09	1.3
FANCL	6.77E-14	0.152948389	0.117	0.068	1.63E-09	1.3
NUDCD3	7.03E-14	0.174987672	0.149	0.092	1.69E-09	1.3
PGD	7.25E-14	0.14851954	0.1	0.055	1.75E-09	1.3
KANK1	7.37E-14	0.166159007	0.279	0.197	1.78E-09	1.3
SOX4	7.53E-14	-0.372446674	0.711	0.749	1.82E-09	1.3
APEX1	7.55E-14	-0.319428882	0.21	0.276	1.82E-09	1.3
R3HDM2	7.61E-14	0.202000898	0.203	0.136	1.84E-09	1.3
MED13	8.61E-14	-0.321960918	0.3	0.366	2.08E-09	1.3
RBM6	8.62E-14	0.17165002	0.404	0.307	2.08E-09	1.3
QDPR	8.63E-14	0.131819945	0.095	0.052	2.08E-09	1.3
MFSD6	9.28E-14	0.140758369	0.128	0.076	2.24E-09	1.3
LLGL2	9.42E-14	0.136118945	0.117	0.068	2.27E-09	1.3
RAP1B	9.48E-14	-0.321166982	0.368	0.429	2.29E-09	1.3
POMP	9.53E-14	0.242379746	0.713	0.642	2.30E-09	1.3
KIAA1671	9.72E-14	0.176321955	0.238	0.165	2.34E-09	1.3
CMTM8	9.80E-14	0.180554914	0.251	0.177	2.36E-09	1.3
HLA-DRA	1.01E-13	-0.459341375	0.111	0.172	2.43E-09	1.3
PUDP	1.04E-13	0.104903133	0.063	0.029	2.50E-09	1.3
NARS	1.08E-13	0.171293559	0.358	0.266	2.61E-09	1.3
HGSNAT	1.10E-13	0.155725114	0.219	0.15	2.65E-09	1.3
RDH10	1.11E-13	0.105039748	0.048	0.019	2.67E-09	1.3
RP11-138A	1.12E-13	0.151810297	0.169	0.107	2.69E-09	1.3
ANGPT2	1.16E-13	0.128529297	0.073	0.036	2.79E-09	1.3
VDR	1.16E-13	0.177315816	0.135	0.082	2.79E-09	1.3
MUC15	1.16E-13	0.131461177	0.077	0.038	2.80E-09	1.3
BBIP1	1.17E-13	0.137589439	0.147	0.091	2.81E-09	1.3
S100A16	1.24E-13	0.200399538	0.14	0.087	2.99E-09	1.3

PLSCR1	1.25E-13	0.147446173	0.358	0.267	3.01E-09	1.3
RAB18	1.47E-13	-0.339122008	0.238	0.303	3.55E-09	1.3
DNAJC10	1.49E-13	0.130409153	0.084	0.044	3.59E-09	1.3
FXYD3	1.55E-13	0.196104338	0.596	0.498	3.73E-09	1.3
KIAA0196	1.59E-13	0.123484646	0.081	0.042	3.84E-09	1.3
NBEA	1.67E-13	0.196996021	0.152	0.096	4.02E-09	1.3
PARK7	1.75E-13	0.212621988	0.545	0.455	4.22E-09	1.3
HOOK2	1.76E-13	0.175410636	0.214	0.146	4.25E-09	1.3
RSRP1	1.77E-13	0.167879062	0.313	0.228	4.26E-09	1.3
ALOX5	2.05E-13	0.162065632	0.179	0.117	4.95E-09	1.3
ARHGEF38	2.06E-13	-0.350675811	0.443	0.497	4.97E-09	1.3
SLC35F3	2.15E-13	0.134011573	0.082	0.042	5.19E-09	1.3
TOM1L1	2.15E-13	0.173678211	0.267	0.19	5.19E-09	1.3
OTUD7B	2.18E-13	-0.309609681	0.123	0.185	5.27E-09	1.3
SEC24A	2.21E-13	-0.340401976	0.169	0.234	5.32E-09	1.3
VLDLR-AS1	2.40E-13	0.117210716	0.049	0.02	5.78E-09	1.3
MCPH1	2.51E-13	0.158122094	0.219	0.15	6.06E-09	1.3
7-Mar	2.52E-13	-0.321426193	0.232	0.298	6.08E-09	1.3
RARB	2.52E-13	0.111759561	0.095	0.051	6.09E-09	1.3
CHST9	2.58E-13	0.133636473	0.072	0.035	6.21E-09	1.3
CRIPT	2.64E-13	0.153231157	0.276	0.199	6.36E-09	1.3
SLC25A13	2.70E-13	0.172199532	0.107	0.061	6.52E-09	1.3
WWC1	2.78E-13	0.237176831	0.523	0.429	6.70E-09	1.3
EPAS1	2.79E-13	0.143398817	0.119	0.07	6.73E-09	1.3
HIPK3	2.88E-13	0.190924618	0.236	0.165	6.94E-09	1.3
TANC1	3.03E-13	0.192880885	0.319	0.237	7.31E-09	1.3
EXOC6	3.15E-13	0.155343202	0.115	0.067	7.60E-09	1.3
NNMT	3.18E-13	0.321358293	0.119	0.071	7.66E-09	1.3
GLIPR1	3.22E-13	-0.544990153	0.311	0.358	7.77E-09	1.3
OCLN	3.46E-13	-0.351915682	0.268	0.33	8.34E-09	1.3
ADK	3.48E-13	0.199996209	0.533	0.43	8.38E-09	1.3
CYYR1	3.49E-13	0.132524554	0.113	0.065	8.43E-09	1.3
MICU2	3.50E-13	0.156061343	0.176	0.116	8.45E-09	1.3
DDX18	3.69E-13	0.170737294	0.436	0.345	8.91E-09	1.3
RNF149	3.72E-13	-0.329465618	0.249	0.315	8.97E-09	1.3
TMEM135	3.73E-13	0.143469863	0.113	0.065	8.98E-09	1.3
NFYB	3.79E-13	0.127564147	0.1	0.056	9.13E-09	1.3
UFD1L	3.80E-13	0.108450739	0.104	0.058	9.16E-09	1.3
PBRM1	3.81E-13	0.128918092	0.254	0.178	9.18E-09	1.3
ABCC3	3.85E-13	0.137162021	0.045	0.018	9.28E-09	1.3
MTRNR2L8	3.90E-13	-0.256953822	0.153	0.218	9.41E-09	1.3
DLD	4.29E-13	0.189472125	0.179	0.118	1.03E-08	1.3
VPS45	4.31E-13	0.118881802	0.127	0.076	1.04E-08	1.3
ALG14	4.33E-13	0.127308566	0.127	0.075	1.05E-08	1.3
MPP6	4.35E-13	0.129609786	0.11	0.063	1.05E-08	1.3
KPNA1	4.38E-13	-0.357027717	0.225	0.287	1.06E-08	1.3
ABLIM1	4.44E-13	0.1661019	0.325	0.241	1.07E-08	1.3

ZNF708	4.45E-13	0.143237181	0.128	0.077	1.07E-08	1.3
PRDX5	4.97E-13	0.192203879	0.097	0.054	1.20E-08	1.3
HAX1	5.14E-13	0.172377786	0.401	0.309	1.24E-08	1.3
DNAJB6	5.18E-13	-0.365432893	0.307	0.365	1.25E-08	1.3
IDH3A	5.48E-13	0.152515174	0.111	0.065	1.32E-08	1.3
EXOC2	5.52E-13	0.135809024	0.108	0.062	1.33E-08	1.3
AVL9	5.66E-13	0.183364032	0.232	0.163	1.36E-08	1.3
VPS13C	5.69E-13	0.190082306	0.276	0.201	1.37E-08	1.3
MPZL2	6.20E-13	0.139976071	0.173	0.113	1.49E-08	1.3
B4GALT6	6.29E-13	0.100355448	0.066	0.032	1.52E-08	1.3
RAI14	6.34E-13	-0.330789307	0.126	0.186	1.53E-08	1.3
MTRNR2L1	6.39E-13	-0.321497349	0.231	0.298	1.54E-08	1.3
RASD1	6.47E-13	0.104080065	0.054	0.024	1.56E-08	1.3
FUT3	6.47E-13	0.107620627	0.049	0.021	1.56E-08	1.3
PPID	6.51E-13	0.119140415	0.112	0.065	1.57E-08	1.3
TNFAIP6	6.54E-13	0.135205515	0.494	0.393	1.58E-08	1.3
RNF213	6.65E-13	0.181960175	0.233	0.163	1.60E-08	1.3
TNPO3	6.68E-13	0.154804405	0.18	0.119	1.61E-08	1.3
EGLN3	7.09E-13	0.14414032	0.159	0.101	1.71E-08	1.3
TRMT112	7.27E-13	0.13488602	0.106	0.061	1.75E-08	1.3
EVI5	7.49E-13	0.184964338	0.183	0.122	1.81E-08	1.3
HS3ST3B1	7.72E-13	0.118585959	0.047	0.02	1.86E-08	1.3
SSR1	7.76E-13	0.15522347	0.231	0.161	1.87E-08	1.3
DYNLRB1	7.92E-13	0.140608458	0.33	0.246	1.91E-08	1.3
RPL27	8.48E-13	-0.142053998	0.942	0.959	2.04E-08	1.3
SPINT1	9.05E-13	0.135930835	0.167	0.11	2.18E-08	1.3
PTPN3	9.06E-13	0.105262235	0.106	0.06	2.18E-08	1.3
GRIK1	9.06E-13	0.123895122	0.084	0.044	2.19E-08	1.3
ESRRA	9.14E-13	0.13261337	0.1	0.056	2.20E-08	1.3
PPP2R3C	9.69E-13	0.141443014	0.127	0.076	2.34E-08	1.3
SRGAP2	9.75E-13	0.140487897	0.159	0.103	2.35E-08	1.3
ZNF680	9.84E-13	0.105384177	0.137	0.084	2.37E-08	1.3
TEX2	9.97E-13	0.116911606	0.095	0.053	2.40E-08	1.3
PAWR	1.03E-12	-0.328452319	0.449	0.491	2.48E-08	1.3
CDH13	1.05E-12	0.132548003	0.077	0.039	2.53E-08	1.3
INADL	1.12E-12	0.137513695	0.876	0.773	2.69E-08	1.3
CCND3	1.13E-12	0.168668937	0.43	0.333	2.71E-08	1.3
PHF14	1.21E-12	0.156928148	0.191	0.129	2.91E-08	1.3
DGKH	1.26E-12	0.171061698	0.206	0.141	3.04E-08	1.3
KIFC3	1.36E-12	0.123327933	0.07	0.035	3.27E-08	1.3
TLK1	1.36E-12	0.167729348	0.263	0.188	3.29E-08	1.3
FBN1	1.41E-12	-0.180972926	0.022	0.059	3.40E-08	1.3
BCAP31	1.53E-12	0.125775745	0.101	0.058	3.69E-08	1.3
CX3CL1	1.59E-12	0.254654494	0.326	0.246	3.83E-08	1.3
FAM151B	1.59E-12	0.112488036	0.075	0.039	3.84E-08	1.3
PKD2	1.66E-12	0.161435387	0.121	0.073	4.01E-08	1.3
CCDC90B	1.82E-12	0.126481672	0.135	0.084	4.40E-08	1.3

TRABD2B	1.86E-12	0.229009338	0.144	0.091	4.50E-08	1.3
TTLL4	1.94E-12	0.168361568	0.22	0.153	4.67E-08	1.3
SS18L2	2.05E-12	0.133382169	0.185	0.124	4.93E-08	1.3
RNF24	2.10E-12	0.168122916	0.496	0.395	5.07E-08	1.3
LZIC	2.15E-12	0.122604303	0.129	0.079	5.18E-08	1.3
DOCK9	2.32E-12	0.16439819	0.217	0.151	5.59E-08	1.3
PPM1K	2.34E-12	0.160607617	0.125	0.077	5.63E-08	1.3
RPS6KA3	2.37E-12	-0.411808204	0.305	0.358	5.71E-08	1.3
PON2	2.53E-12	0.118174868	0.087	0.048	6.09E-08	1.3
NOL3	2.55E-12	0.116431786	0.078	0.041	6.14E-08	1.3
NFIL3	2.68E-12	-0.297893153	0.115	0.169	6.46E-08	1.3
EPS8	2.71E-12	0.142241783	0.526	0.423	6.53E-08	1.3
RAN	2.74E-12	0.153668105	0.669	0.582	6.60E-08	1.3
TCF20	2.76E-12	0.152898563	0.151	0.097	6.65E-08	1.3
TBCA	2.88E-12	0.201482634	0.737	0.658	6.94E-08	1.3
EXOC6B	2.91E-12	0.203549929	0.245	0.175	7.02E-08	1.3
ST3GAL6	2.94E-12	0.187462536	0.16	0.105	7.09E-08	1.3
SDHC	2.99E-12	0.132132941	0.196	0.134	7.21E-08	1.3
FAM217B	2.99E-12	0.10516462	0.063	0.031	7.21E-08	1.3
RHBDL2	3.03E-12	0.1213982	0.069	0.035	7.30E-08	1.3
6-Mar	3.04E-12	-0.318480419	0.302	0.358	7.33E-08	1.3
SDF2	3.11E-12	0.112417183	0.136	0.084	7.49E-08	1.3
GSK3B	3.12E-12	0.175505829	0.36	0.275	7.52E-08	1.3
BMPR1A	3.12E-12	0.174605754	0.346	0.263	7.53E-08	1.3
CISD1	3.14E-12	0.156874363	0.281	0.205	7.57E-08	1.3
RTN3	3.18E-12	0.148442921	0.248	0.177	7.67E-08	1.3
MFAP1	3.34E-12	0.122141947	0.208	0.142	8.05E-08	1.3
SLC22A23	3.35E-12	0.169356898	0.214	0.149	8.07E-08	1.3
KIT	3.49E-12	0.173456858	0.436	0.343	8.40E-08	1.3
SPIRE1	3.56E-12	0.176025345	0.469	0.375	8.57E-08	1.3
PEX2	3.56E-12	0.122578081	0.133	0.083	8.58E-08	1.3
DDIT3	3.57E-12	-0.366596199	0.087	0.138	8.60E-08	1.3
TBL1X	3.61E-12	0.15304765	0.213	0.148	8.70E-08	1.3
CALU	3.93E-12	0.140326917	0.358	0.271	9.46E-08	1.3
THBS1	3.97E-12	-0.251824642	0.042	0.085	9.58E-08	1.3
SUMF1	4.00E-12	0.169257835	0.157	0.102	9.63E-08	1.3
GNAQ	4.02E-12	0.188969745	0.375	0.291	9.70E-08	1.3
MTRNR2L1	4.19E-12	-0.279292113	0.137	0.196	1.01E-07	1.3
MAP2K5	4.56E-12	0.13341324	0.17	0.112	1.10E-07	1.3
FBXL5	4.76E-12	0.161960211	0.172	0.115	1.15E-07	1.3
TUSC3	5.04E-12	0.155023345	0.192	0.131	1.21E-07	1.3
NOS1AP	5.30E-12	0.136635051	0.208	0.144	1.28E-07	1.3
PHB	5.37E-12	0.149860913	0.057	0.027	1.30E-07	1.3
BBS9	5.57E-12	0.139243661	0.096	0.055	1.34E-07	1.3
PRLR	5.88E-12	0.142609923	0.176	0.118	1.42E-07	1.3
EGF	6.00E-12	0.121167499	0.113	0.067	1.45E-07	1.3
RAB1A	6.05E-12	-0.277765806	0.417	0.467	1.46E-07	1.3

VPS13B	6.17E-12	0.176425179	0.213	0.15	1.49E-07	1.3
PSMB6	6.42E-12	0.128891957	0.144	0.092	1.55E-07	1.3
PLRG1	6.80E-12	0.128573257	0.161	0.106	1.64E-07	1.3
TTC14	7.03E-12	0.137697274	0.107	0.063	1.69E-07	1.3
PSMA7	7.25E-12	0.132316259	0.235	0.167	1.75E-07	1.3
STAC	7.30E-12	0.120361817	0.069	0.035	1.76E-07	1.3
PXDN	7.50E-12	0.15631094	0.157	0.103	1.81E-07	1.3
LAMTOR5	7.62E-12	0.147225522	0.681	0.587	1.84E-07	1.3
FBXL4	7.62E-12	0.148804695	0.12	0.073	1.84E-07	1.3
PDIA3	7.66E-12	-0.369010164	0.226	0.286	1.85E-07	1.3
RP11-20I2(	7.87E-12	0.10063953	0.055	0.026	1.90E-07	1.3
TMC5	7.97E-12	0.123947091	0.077	0.041	1.92E-07	1.3
FAM126B	8.09E-12	-0.302381547	0.117	0.172	1.95E-07	1.3
NAA20	8.14E-12	0.150372108	0.172	0.115	1.96E-07	1.3
PSMB3	8.48E-12	0.145872718	0.104	0.061	2.04E-07	1.3
PRMT2	8.96E-12	0.166857286	0.169	0.113	2.16E-07	1.3
KATNBL1	9.02E-12	0.172127928	0.189	0.13	2.18E-07	1.3
DPY30	9.14E-12	0.150086638	0.213	0.149	2.20E-07	1.3
PPARA	9.43E-12	0.140216549	0.112	0.067	2.27E-07	1.3
ANAPC13	9.97E-12	0.149054711	0.168	0.112	2.40E-07	1.3
USP6NL	1.04E-11	-0.295787216	0.162	0.222	2.51E-07	1.3
ARHGEF11	1.04E-11	0.127116048	0.077	0.041	2.51E-07	1.3
EMP1	1.07E-11	-0.685392178	0.738	0.712	2.59E-07	1.3
LPIN1	1.08E-11	-0.422258599	0.306	0.356	2.60E-07	1.3
FAM160A1	1.14E-11	-0.282658772	0.687	0.705	2.74E-07	1.3
PRKAR2A	1.15E-11	0.152014059	0.166	0.11	2.77E-07	1.3
RP11-711K	1.15E-11	0.155295825	0.091	0.051	2.78E-07	1.3
IRX3	1.15E-11	0.112836314	0.078	0.042	2.78E-07	1.3
COX6C	1.21E-11	0.182652826	0.689	0.606	2.91E-07	1.3
MIR222HG	1.24E-11	-0.26866734	0.063	0.11	2.99E-07	1.3
NFKB1	1.26E-11	-0.404348799	0.364	0.414	3.03E-07	1.3
GLCE	1.26E-11	0.109427735	0.077	0.041	3.03E-07	1.3
ATP5EP2	1.28E-11	0.179553499	0.256	0.188	3.09E-07	1.3
DIAPH2-AS	1.31E-11	0.12175121	0.064	0.032	3.17E-07	1.3
DIAPH3	1.32E-11	-0.320555306	0.088	0.14	3.18E-07	1.3
PVRL2	1.34E-11	-0.236646168	0.133	0.191	3.23E-07	1.3
GTF2A2	1.37E-11	0.110319984	0.125	0.077	3.30E-07	1.3
ADAMTS9-,	1.37E-11	0.129080769	0.385	0.298	3.30E-07	1.3
LCORL	1.45E-11	0.162800885	0.158	0.105	3.51E-07	1.3
MAP3K13	1.47E-11	-0.300564792	0.838	0.826	3.55E-07	1.3
AKAP6	1.49E-11	0.124972675	0.079	0.043	3.60E-07	1.3
C20orf24	1.49E-11	0.122465102	0.095	0.055	3.60E-07	1.3
DENND1B	1.55E-11	0.158436499	0.176	0.118	3.74E-07	1.3
FOS	1.68E-11	0.295991005	0.297	0.226	4.06E-07	1.3
RPL37A	1.72E-11	-0.127464633	0.973	0.986	4.14E-07	1.3
MEST	1.72E-11	-0.156098832	0.015	0.045	4.16E-07	1.3
ZNF480	1.80E-11	0.111722192	0.079	0.043	4.34E-07	1.3

EIF3I	1.94E-11	0.177498555	0.717	0.642	4.69E-07	1.3
NR3C2	1.95E-11	0.147022068	0.16	0.107	4.70E-07	1.3
FBXL20	2.09E-11	0.20521462	0.309	0.236	5.05E-07	1.3
LRSAM1	2.09E-11	0.147946927	0.155	0.102	5.05E-07	1.3
WEE1	2.11E-11	-0.304456655	0.221	0.28	5.08E-07	1.3
SNRPG	2.13E-11	0.154227382	0.613	0.521	5.13E-07	1.3
C14orf1	2.18E-11	0.112205464	0.085	0.047	5.26E-07	1.3
ASNS	2.29E-11	0.146866839	0.115	0.071	5.51E-07	1.3
CIAPIN1	2.34E-11	0.120638691	0.122	0.076	5.65E-07	1.3
CBFA2T2	2.40E-11	0.166634316	0.183	0.126	5.80E-07	1.3
KIAA0556	2.44E-11	0.117312593	0.123	0.076	5.89E-07	1.3
CAP1	2.47E-11	0.147000238	0.283	0.209	5.96E-07	1.3
TRIB2	2.53E-11	0.112604461	0.084	0.047	6.10E-07	1.3
SEC61G	2.56E-11	0.200726767	0.748	0.686	6.18E-07	1.3
IKZF2	2.61E-11	0.107758747	0.073	0.039	6.29E-07	1.3
9-Sep	2.62E-11	0.152488381	0.167	0.112	6.32E-07	1.3
GS1-24F4.2	2.82E-11	0.136286116	0.147	0.095	6.79E-07	1.3
SPAG9	2.82E-11	-0.324130664	0.298	0.353	6.80E-07	1.3
SPG7	2.84E-11	0.122334858	0.086	0.048	6.84E-07	1.3
BTF3L4	2.86E-11	0.135886509	0.344	0.265	6.89E-07	1.3
PRELID1	3.03E-11	0.146470985	0.184	0.127	7.30E-07	1.3
PTCHD1-AS	3.05E-11	0.178045461	0.143	0.093	7.34E-07	1.3
WIPF2	3.12E-11	0.125022922	0.142	0.092	7.52E-07	1.3
R3HCC1L	3.13E-11	0.157860555	0.166	0.112	7.55E-07	1.3
ECHDC2	3.14E-11	0.170318878	0.156	0.103	7.58E-07	1.3
JAK2	3.26E-11	0.184232212	0.116	0.072	7.86E-07	1.3
C2orf68	3.26E-11	0.13433801	0.095	0.055	7.87E-07	1.3
HILPDA	3.34E-11	-0.441718822	0.202	0.263	8.04E-07	1.3
ADAM17	3.40E-11	-0.334342982	0.386	0.434	8.19E-07	1.3
SERPINA3	3.47E-11	0.175244912	0.27	0.2	8.37E-07	1.3
SLC7A11	3.48E-11	0.157697184	0.075	0.04	8.40E-07	1.3
C6orf62	3.54E-11	0.135562212	0.222	0.158	8.53E-07	1.3
URM1	3.78E-11	0.100347405	0.1	0.058	9.11E-07	1.3
UVRAG	3.84E-11	0.171844727	0.486	0.393	9.26E-07	1.3
ZNF226	3.97E-11	0.126154604	0.11	0.067	9.58E-07	1.3
FAM107B	4.02E-11	0.146329255	0.302	0.225	9.70E-07	1.3
CAAP1	4.05E-11	0.120916865	0.091	0.052	9.78E-07	1.3
AK1	4.16E-11	0.104096855	0.031	0.011	1.00E-06	1.3
FER	4.16E-11	0.158880861	0.267	0.196	1.00E-06	1.3
CRTC3	4.56E-11	0.147737269	0.313	0.239	1.10E-06	1.3
P4HA2	4.58E-11	0.113952357	0.106	0.063	1.10E-06	1.3
RARS	4.73E-11	0.138226565	0.347	0.266	1.14E-06	1.3
KIAA1147	4.83E-11	0.13754498	0.12	0.074	1.17E-06	1.3
NRG1	4.95E-11	0.195074436	0.166	0.112	1.19E-06	1.3
NT5C2	4.97E-11	0.133252908	0.443	0.351	1.20E-06	1.3
HIVEP2	4.97E-11	-0.393283879	0.332	0.38	1.20E-06	1.3
C15orf57	5.00E-11	0.112802045	0.1	0.059	1.21E-06	1.3

RBM8A	5.42E-11	0.153817059	0.577	0.488	1.31E-06	1.3
PACS1	5.44E-11	0.120301857	0.363	0.281	1.31E-06	1.3
EZR	5.82E-11	-0.333363766	0.272	0.324	1.40E-06	1.3
SLC1A5	5.94E-11	0.100514039	0.072	0.038	1.43E-06	1.3
NSUN4	6.18E-11	0.116011349	0.111	0.068	1.49E-06	1.3
NSUN3	6.22E-11	0.10274507	0.077	0.042	1.50E-06	1.3
ADAM10	6.57E-11	0.139006309	0.298	0.222	1.58E-06	1.3
MPHOSPH8	6.81E-11	0.144976163	0.214	0.152	1.64E-06	1.3
WWP1	6.82E-11	0.132474804	0.238	0.172	1.65E-06	1.3
HNRNPF	6.92E-11	0.132316234	0.175	0.119	1.67E-06	1.3
TCF25	7.04E-11	0.19385947	0.15	0.099	1.70E-06	1.3
ZNF143	7.12E-11	-0.258770437	0.111	0.163	1.72E-06	1.3
VPS36	7.18E-11	0.151248588	0.145	0.096	1.73E-06	1.3
TMEM87A	7.24E-11	0.133430843	0.43	0.339	1.75E-06	1.3
MT2A	7.27E-11	0.912753112	0.105	0.065	1.75E-06	1.3
TNC	7.37E-11	0.291539284	0.151	0.101	1.78E-06	1.3
CNTNAP3	7.48E-11	0.117937904	0.09	0.052	1.80E-06	1.3
AP3B1	7.79E-11	0.160151022	0.233	0.17	1.88E-06	1.3
SUPT3H	7.97E-11	0.139022104	0.204	0.144	1.92E-06	1.3
SLC39A8	8.06E-11	0.103210642	0.193	0.134	1.94E-06	1.3
TATDN1	8.09E-11	-0.261253196	0.267	0.323	1.95E-06	1.3
HS6ST3	8.72E-11	0.135526273	0.062	0.031	2.10E-06	1.3
PHKB	8.84E-11	0.162801876	0.246	0.18	2.13E-06	1.3
ZBTB44	9.18E-11	0.14187682	0.143	0.094	2.21E-06	1.3
RPL7L1	9.41E-11	0.122696352	0.173	0.119	2.27E-06	1.3
SNAP23	9.76E-11	0.127751125	0.192	0.133	2.35E-06	1.3
FAM13A	9.85E-11	-0.532587516	0.346	0.388	2.38E-06	1.3
ANKRD33B	1.01E-10	-0.185777948	0.029	0.064	2.44E-06	1.3
RFX3	1.02E-10	0.170478142	0.204	0.146	2.45E-06	1.3
CREB5	1.02E-10	-0.450912327	0.279	0.332	2.46E-06	1.3
POLR2L	1.03E-10	0.142732865	0.057	0.028	2.49E-06	1.3
TTLL5	1.05E-10	0.12763139	0.221	0.16	2.52E-06	1.3
TWF1	1.06E-10	0.10175795	0.111	0.068	2.55E-06	1.3
ZNHIT3	1.08E-10	0.14930821	0.258	0.191	2.61E-06	1.3
GNA13	1.08E-10	-0.263375848	0.157	0.213	2.61E-06	1.3
UXS1	1.11E-10	0.129747946	0.133	0.085	2.68E-06	1.3
ATXN10	1.20E-10	0.16311494	0.201	0.144	2.89E-06	1.3
AP1B1	1.23E-10	0.15302818	0.056	0.028	2.96E-06	1.3
DDX52	1.24E-10	0.133478722	0.151	0.101	2.98E-06	1.3
ZNF235	1.24E-10	0.152748747	0.107	0.065	2.99E-06	1.3
HDGF	1.26E-10	0.130179871	0.321	0.244	3.05E-06	1.3
STK38L	1.29E-10	-0.291395796	0.105	0.155	3.10E-06	1.3
CDC42SE2	1.30E-10	0.166878582	0.276	0.207	3.14E-06	1.3
C1orf21	1.33E-10	-0.329912094	0.184	0.238	3.20E-06	1.3
ENOX2	1.33E-10	0.126863443	0.083	0.047	3.20E-06	1.3
RP11-712B	1.35E-10	0.14639246	0.081	0.046	3.25E-06	1.3
RAD50	1.41E-10	0.153814187	0.135	0.088	3.41E-06	1.3

MORF4L2	1.46E-10	-0.227760589	0.738	0.749	3.53E-06	1.3
SSB	1.49E-10	0.13667571	0.221	0.159	3.59E-06	1.3
DSTN	1.50E-10	0.212126588	0.545	0.465	3.61E-06	1.3
CARHSP1	1.62E-10	0.196931303	0.328	0.254	3.92E-06	1.3
PSMC6	1.65E-10	0.147984551	0.39	0.31	3.99E-06	1.3
ARL1	1.66E-10	0.155774498	0.343	0.266	4.00E-06	1.3
INTS10	1.66E-10	0.151564549	0.148	0.099	4.00E-06	1.3
NIF3L1	1.67E-10	0.11635598	0.091	0.054	4.03E-06	1.3
NRDC	1.68E-10	0.13586048	0.222	0.161	4.06E-06	1.3
MCF2L2	1.75E-10	0.145045442	0.205	0.145	4.22E-06	1.3
IQCG	1.76E-10	-0.328749159	0.178	0.232	4.24E-06	1.3
PGM1	1.77E-10	0.214421383	0.16	0.108	4.26E-06	1.3
MSI2	1.78E-10	0.170948227	0.246	0.181	4.29E-06	1.3
GLUD1	1.86E-10	-0.319846364	0.125	0.176	4.48E-06	1.3
LAMC2	1.91E-10	0.115020415	0.354	0.275	4.60E-06	1.3
CRADD	1.95E-10	0.118969038	0.136	0.089	4.71E-06	1.3
PUM1	1.98E-10	-0.28062347	0.492	0.521	4.77E-06	1.3
TLE4	2.01E-10	-0.333498857	0.368	0.418	4.85E-06	1.3
FCHO2	2.01E-10	0.128221221	0.192	0.135	4.86E-06	1.3
FOSL2	2.02E-10	0.145360215	0.226	0.165	4.87E-06	1.3
PSTPIP2	2.02E-10	0.107948316	0.427	0.334	4.87E-06	1.3
NCEH1	2.04E-10	0.160740754	0.271	0.203	4.92E-06	1.3
ODAM	2.14E-10	0.110103899	0.043	0.019	5.17E-06	1.3
EFNA1	2.29E-10	0.114058629	0.149	0.099	5.52E-06	1.3
RP5-945F2.	2.29E-10	-0.368260987	0.048	0.087	5.53E-06	1.3
SESN3	2.31E-10	0.188048242	0.114	0.071	5.58E-06	1.3
CCDC6	2.44E-10	-0.344094714	0.305	0.353	5.88E-06	1.3
LINC00342	2.48E-10	0.122606182	0.092	0.055	5.97E-06	1.3
LINC01549	2.54E-10	0.155513617	0.057	0.029	6.13E-06	1.3
PTPN6	2.57E-10	0.104501638	0.07	0.038	6.19E-06	1.3
C2CD2	2.73E-10	0.108649513	0.077	0.043	6.59E-06	1.3
RP11-83A2	2.78E-10	0.149705343	0.156	0.106	6.71E-06	1.3
AHI1	2.80E-10	0.152447246	0.257	0.19	6.75E-06	1.3
GAPVD1	2.80E-10	-0.246346892	0.168	0.223	6.75E-06	1.3
HERC1	2.82E-10	-0.344557789	0.362	0.403	6.81E-06	1.3
SLC2A1	2.86E-10	-0.251774837	0.05	0.09	6.89E-06	1.3
CEP63	2.92E-10	0.136685412	0.115	0.072	7.04E-06	1.3
SEC62	2.96E-10	-0.291021972	0.611	0.631	7.14E-06	1.3
CXCL16	2.98E-10	0.11228145	0.248	0.183	7.19E-06	1.3
CXCR4	2.99E-10	-0.572128612	0.262	0.311	7.20E-06	1.3
KLK10	2.99E-10	0.125509162	0.086	0.05	7.22E-06	1.3
SLC25A5	3.07E-10	0.160799019	0.195	0.139	7.41E-06	1.3
KIAA2026	3.09E-10	0.11175734	0.188	0.132	7.45E-06	1.3
ZNF121	3.10E-10	0.134213991	0.121	0.077	7.47E-06	1.3
EIF4E	3.23E-10	-0.320486237	0.352	0.396	7.79E-06	1.3
NSRP1	3.24E-10	0.151179459	0.33	0.256	7.81E-06	1.3
PNISR	3.30E-10	0.118386759	0.837	0.741	7.96E-06	1.3

HPS5	3.40E-10	0.114275334	0.277	0.207	8.19E-06	1.3
CCNB1IP1	3.43E-10	-0.260483742	0.331	0.382	8.27E-06	1.3
USP34	3.46E-10	0.169186303	0.654	0.562	8.33E-06	1.3
RFX7	3.55E-10	0.168529112	0.19	0.135	8.56E-06	1.3
AHR	3.57E-10	0.171615313	0.254	0.189	8.61E-06	1.3
CUL5	3.59E-10	0.114899451	0.174	0.12	8.66E-06	1.3
SERPINB1	3.62E-10	0.159129505	0.14	0.093	8.73E-06	1.3
GMFB	3.68E-10	0.113363845	0.132	0.086	8.87E-06	1.3
TLR2	3.83E-10	0.113192944	0.314	0.237	9.23E-06	1.3
PRRG4	3.96E-10	0.114345129	0.248	0.183	9.54E-06	1.3
RGS2	3.99E-10	-0.693001891	0.174	0.224	9.61E-06	1.3
TMCC1	4.01E-10	0.134403623	0.333	0.257	9.66E-06	1.3
ACTL6A	4.09E-10	0.111245148	0.086	0.05	9.86E-06	1.3
ZFP36L1	4.16E-10	0.194676561	0.589	0.503	1.00E-05	1.3
SERAC1	4.22E-10	-0.229940354	0.05	0.089	1.02E-05	1.3
MFSD11	4.28E-10	0.134200506	0.102	0.062	1.03E-05	1.3
SIPA1L2	4.31E-10	0.110453286	0.104	0.064	1.04E-05	1.3
FRMD3	4.63E-10	0.155281954	0.125	0.081	1.12E-05	1.3
SLC38A2	4.64E-10	0.102434674	0.222	0.16	1.12E-05	1.3
TNRC6A	4.77E-10	-0.268545631	0.211	0.266	1.15E-05	1.3
GNPTAB	4.98E-10	0.145986105	0.147	0.099	1.20E-05	1.3
RPA1	5.09E-10	0.120196118	0.09	0.053	1.23E-05	1.3
LINC01183	5.10E-10	0.129058554	0.087	0.051	1.23E-05	1.3
NF1	5.11E-10	0.136450586	0.529	0.432	1.23E-05	1.3
TMED3	5.15E-10	0.119407148	0.1	0.061	1.24E-05	1.3
ELMOD2	5.42E-10	0.107068293	0.083	0.048	1.31E-05	1.3
ZNF518A	5.59E-10	0.132949331	0.234	0.171	1.35E-05	1.3
GSTP1	5.66E-10	0.248739127	0.107	0.067	1.37E-05	1.3
MICAL3	5.74E-10	-0.351768403	0.263	0.315	1.38E-05	1.3
SCFD2	5.75E-10	0.104545459	0.067	0.036	1.39E-05	1.3
TACC2	5.77E-10	0.130337097	0.19	0.135	1.39E-05	1.3
SEL1L	6.18E-10	-0.232407945	0.061	0.102	1.49E-05	1.3
IQCB1	6.27E-10	0.128562284	0.133	0.087	1.51E-05	1.3
MIRLET7BH	6.37E-10	0.100501936	0.055	0.028	1.54E-05	1.3
C16orf72	6.41E-10	-0.247381347	0.108	0.156	1.55E-05	1.3
UBR1	6.54E-10	0.140172536	0.169	0.117	1.58E-05	1.3
YWHAB	6.92E-10	0.119815679	0.337	0.26	1.67E-05	1.3
WIPF3	6.94E-10	0.103236253	0.073	0.041	1.67E-05	1.3
LGALS3	6.99E-10	0.193757859	0.225	0.167	1.68E-05	1.3
PDE1C	7.09E-10	0.148104832	0.111	0.07	1.71E-05	1.3
LBP	7.15E-10	-0.178609459	0.014	0.041	1.72E-05	1.3
VPS50	7.17E-10	0.121093737	0.085	0.05	1.73E-05	1.3
TTC9C	7.20E-10	0.135501229	0.191	0.135	1.74E-05	1.3
SLC30A6	7.21E-10	0.112363274	0.084	0.049	1.74E-05	1.3
AGO4	7.59E-10	0.132997029	0.14	0.094	1.83E-05	1.3
PTPRA	7.90E-10	0.139235932	0.192	0.136	1.90E-05	1.3
RNF114	8.12E-10	-0.253192527	0.112	0.16	1.96E-05	1.3

BBS4	8.26E-10	0.114768377	0.076	0.043	1.99E-05	1.3
CAMK1D	8.30E-10	-0.328653809	0.114	0.162	2.00E-05	1.3
LDLR	8.37E-10	0.160217563	0.212	0.154	2.02E-05	1.3
CLK1	8.43E-10	-0.297120842	0.355	0.404	2.03E-05	1.3
OPHN1	8.68E-10	0.121713715	0.275	0.207	2.09E-05	1.3
SNX14	8.73E-10	0.116820244	0.124	0.08	2.11E-05	1.3
PITPNC1	8.78E-10	0.138834796	0.262	0.198	2.12E-05	1.3
RPS6KC1	8.99E-10	0.122094277	0.12	0.077	2.17E-05	1.3
HERPUD1	9.26E-10	-0.306124464	0.157	0.209	2.23E-05	1.3
HIST1H4E	1.01E-09	0.108043558	0.14	0.093	2.44E-05	1.3
TFG	1.04E-09	-0.262547489	0.273	0.325	2.50E-05	1.3
TIMM9	1.04E-09	-0.255336224	0.182	0.234	2.52E-05	1.3
ASXL2	1.05E-09	0.14267291	0.107	0.067	2.53E-05	1.3
TTC9	1.08E-09	0.148777857	0.241	0.18	2.60E-05	1.3
ITCH	1.11E-09	0.130930914	0.42	0.333	2.67E-05	1.3
MDM4	1.11E-09	0.136391692	0.325	0.253	2.67E-05	1.3
HEATR5A	1.13E-09	0.117519638	0.088	0.053	2.71E-05	1.3
ALG13	1.15E-09	0.146377906	0.134	0.09	2.76E-05	1.3
COBLL1	1.15E-09	0.156788812	0.167	0.117	2.78E-05	1.3
C5orf56	1.16E-09	-0.192275295	0.089	0.137	2.80E-05	1.3
SEL1L3	1.17E-09	0.128074935	0.081	0.047	2.82E-05	1.3
SMG6	1.19E-09	0.134609388	0.202	0.145	2.86E-05	1.3
SYPL1	1.23E-09	0.115137545	0.478	0.388	2.97E-05	1.3
IK	1.24E-09	0.134913021	0.27	0.206	2.99E-05	1.3
INO80	1.28E-09	0.122903031	0.206	0.149	3.10E-05	1.3
DPH3	1.29E-09	0.11505284	0.15	0.102	3.11E-05	1.3
PSMB4	1.36E-09	0.140339137	0.185	0.132	3.29E-05	1.3
MAPKAP1	1.40E-09	0.117955097	0.133	0.088	3.37E-05	1.3
ARHGEF10	1.49E-09	-0.330057289	0.335	0.38	3.60E-05	1.3
GDE1	1.58E-09	0.115598322	0.098	0.06	3.80E-05	1.3
ARHGAP21	1.59E-09	0.131409667	0.292	0.225	3.83E-05	1.3
PILRB	1.60E-09	0.151939725	0.081	0.047	3.86E-05	1.3
LGALS8	1.62E-09	0.15000078	0.159	0.11	3.91E-05	1.3
MYO5C	1.62E-09	0.117221895	0.14	0.094	3.92E-05	1.3
RPL4	1.65E-09	-0.164511398	0.951	0.959	3.98E-05	1.3
RP11-142C	1.68E-09	0.137033556	0.157	0.109	4.05E-05	1.3
SNX29	1.71E-09	0.139248531	0.14	0.094	4.12E-05	1.3
DARS	1.71E-09	0.122604372	0.391	0.31	4.13E-05	1.3
VPS29	1.75E-09	0.151518047	0.45	0.368	4.21E-05	1.3
RP11-793A	1.75E-09	0.120853965	0.063	0.034	4.22E-05	1.3
STAT5B	1.84E-09	0.148908399	0.229	0.17	4.44E-05	1.3
GCC2	1.85E-09	0.110630195	0.387	0.306	4.47E-05	1.3
FAM193A	1.94E-09	0.127942621	0.188	0.134	4.67E-05	1.3
IRF6	2.06E-09	-0.301450249	0.185	0.234	4.96E-05	1.3
MSH3	2.17E-09	0.130802217	0.138	0.093	5.24E-05	1.3
MTMR2	2.20E-09	0.120165714	0.146	0.099	5.31E-05	1.3
NAALADL2	2.20E-09	0.204634666	0.394	0.323	5.31E-05	1.3

LSM3	2.26E-09	0.146643812	0.377	0.302	5.44E-05	1.3
RSBN1L	2.26E-09	-0.239535174	0.089	0.134	5.45E-05	1.3
TMC1	2.27E-09	0.100847123	0.053	0.027	5.48E-05	1.3
DYRK1A	2.31E-09	-0.325451997	0.326	0.37	5.58E-05	1.3
GRB14	2.33E-09	-0.297915553	0.54	0.57	5.61E-05	1.3
MKLN1	2.47E-09	0.181127761	0.686	0.616	5.95E-05	1.3
TAF1B	2.47E-09	0.117017971	0.127	0.083	5.97E-05	1.3
RPSA	2.48E-09	-0.197685965	0.92	0.921	5.97E-05	1.3
FSTL1	2.49E-09	0.128765585	0.15	0.103	6.01E-05	1.3
TFCP2L1	2.49E-09	0.185741024	0.191	0.138	6.01E-05	1.3
DIRC2	2.52E-09	0.129026942	0.097	0.061	6.07E-05	1.3
SLC16A1-A:	2.53E-09	0.150884856	0.149	0.103	6.11E-05	1.3
ITGB4	2.62E-09	0.122732674	0.077	0.045	6.31E-05	1.3
TRAPPC13	2.63E-09	0.105654691	0.085	0.05	6.35E-05	1.3
STX8	2.64E-09	0.134623581	0.245	0.185	6.37E-05	1.3
SLCO3A1	2.65E-09	0.108615899	0.307	0.236	6.40E-05	1.3
MCTP2	2.66E-09	0.134525495	0.19	0.136	6.42E-05	1.3
DENND1A	2.67E-09	0.133743647	0.265	0.201	6.45E-05	1.3
SLIT2	2.72E-09	0.107080538	0.066	0.037	6.56E-05	1.3
CTDSP2	2.97E-09	0.113741142	0.234	0.173	7.16E-05	1.3
PUM2	3.03E-09	-0.260529461	0.319	0.369	7.31E-05	1.3
CPD	3.08E-09	0.140924262	0.267	0.205	7.43E-05	1.3
SERPINA1	3.20E-09	0.12038309	0.055	0.029	7.71E-05	1.3
CMTM4	3.28E-09	0.10737413	0.135	0.091	7.90E-05	1.3
AK4	3.33E-09	0.113777355	0.24	0.179	8.03E-05	1.3
RAPH1	3.36E-09	0.103861823	0.238	0.177	8.09E-05	1.3
FBXL17	3.41E-09	0.101896493	0.231	0.172	8.22E-05	1.3
PDS5B	3.43E-09	0.129656386	0.338	0.265	8.28E-05	1.3
ATXN1	3.52E-09	-0.341238964	0.329	0.374	8.50E-05	1.3
ARL8B	3.59E-09	-0.258722301	0.215	0.266	8.65E-05	1.3
NDUFA12	3.60E-09	0.127846976	0.281	0.217	8.69E-05	1.3
VPS8	3.67E-09	0.119441086	0.114	0.074	8.85E-05	1.3
SGMS2	3.76E-09	-0.298256626	0.143	0.191	9.07E-05	1.3
C20orf194	3.79E-09	0.149258017	0.2	0.147	9.15E-05	1.3
TGM2	3.81E-09	0.116555394	0.121	0.08	9.19E-05	1.3
CBLB	3.97E-09	0.154777598	0.279	0.215	9.56E-05	1.3
RPL5	4.03E-09	-0.134788706	0.968	0.976	9.71E-05	1.3
TTC17	4.11E-09	0.149468555	0.338	0.266	9.90E-05	1.3
EDN1	4.17E-09	-0.348448628	0.177	0.225	0.0001005	1.3
ZFYVE9	4.17E-09	0.144404508	0.143	0.098	0.0001006	1.3
ZBTB1	4.31E-09	0.113827907	0.12	0.079	0.0001039	1.3
IDE	4.38E-09	0.128014583	0.104	0.067	0.0001057	1.3
TCF7L1	4.78E-09	-0.308626508	0.299	0.348	0.0001152	1.3
TSG101	4.82E-09	0.124346684	0.219	0.162	0.0001161	1.3
YARS	4.84E-09	-0.299775552	0.245	0.292	0.0001167	1.3
XPO4	5.08E-09	0.13524102	0.173	0.124	0.0001225	1.3
ZNF33B	5.09E-09	0.142215534	0.228	0.171	0.0001226	1.3

CPEB4	5.22E-09	0.138749053	0.226	0.168	0.0001258	1.3
ZNF267	5.40E-09	-0.21716213	0.14	0.189	0.0001301	1.3
FBXO32	5.42E-09	0.171924705	0.469	0.396	0.0001307	1.3
YBX3	5.45E-09	0.159651143	0.329	0.26	0.0001315	1.3
PIM1	5.57E-09	-0.10071851	0.01	0.033	0.0001343	1.3
C5orf17	6.02E-09	0.149417927	0.091	0.056	0.0001452	1.3
TMEM65	6.33E-09	0.124501695	0.222	0.165	0.0001526	1.3
ANKS1A	6.45E-09	0.131340131	0.173	0.123	0.0001555	1.3
HNRNPH2	6.57E-09	0.113383676	0.274	0.209	0.0001585	1.3
CACNA2D1	6.70E-09	0.124279234	0.066	0.037	0.0001616	1.3
BROX	6.82E-09	0.101402144	0.173	0.123	0.0001645	1.3
SERTAD2	6.83E-09	-0.282358949	0.169	0.218	0.0001646	1.3
SAR1B	6.89E-09	-0.42280559	0.428	0.465	0.000166	1.3
FXR1	7.10E-09	-0.25438483	0.401	0.437	0.0001712	1.3
MRPL20	7.12E-09	0.109390808	0.096	0.06	0.0001718	1.3
BBOX1	7.22E-09	-0.3369786	0.226	0.278	0.000174	1.3
PWWP2A	7.44E-09	0.1207204	0.096	0.061	0.0001793	1.3
RAP1GDS1	7.54E-09	0.121307906	0.114	0.075	0.0001818	1.3
MAP4K4	8.24E-09	0.121929503	0.414	0.332	0.0001986	1.3
ZZZ3	8.29E-09	0.104711163	0.22	0.163	0.0001998	1.3
CYB5R3	8.50E-09	0.108485594	0.104	0.067	0.000205	1.3
MLLT3	8.70E-09	0.130416519	0.19	0.139	0.0002099	1.3
MAN1A2	8.73E-09	0.152765523	0.222	0.167	0.0002105	1.3
C10orf76	8.96E-09	0.170531119	0.169	0.122	0.000216	1.3
UBE2N	8.99E-09	0.101294	0.267	0.202	0.0002168	1.3
NALCN	9.25E-09	0.134679326	0.193	0.141	0.0002232	1.3
NUB1	9.29E-09	0.335842264	0.336	0.277	0.000224	1.3
RASSF3	9.62E-09	-0.227752029	0.115	0.161	0.000232	1.3
ERP29	9.64E-09	0.130986014	0.118	0.078	0.0002325	1.3
RP11-266O	9.76E-09	-0.195255468	0.028	0.058	0.0002353	1.3
SHANK2	1.06E-08	0.123733232	0.242	0.181	0.000256	1.3
DENND4A	1.07E-08	-0.312268852	0.496	0.529	0.0002579	1.3
LRIG1	1.08E-08	0.111784598	0.294	0.229	0.0002598	1.3
CTC-471J1.	1.09E-08	-0.232862025	0.765	0.754	0.0002623	1.3
BAIAP2L1	1.09E-08	-0.204277216	0.958	0.97	0.0002625	1.3
GPX1	1.11E-08	0.126840269	0.064	0.036	0.0002684	1.3
RPL14	1.11E-08	-0.137593671	0.975	0.983	0.0002685	1.3
IFFO2	1.14E-08	-0.234748312	0.108	0.154	0.0002759	1.3
PIAS1	1.17E-08	-0.263834558	0.388	0.433	0.0002815	1.3
RAB13	1.20E-08	0.143482912	0.179	0.131	0.0002887	1.3
MTHFD1L	1.22E-08	0.116385815	0.341	0.268	0.0002939	1.3
EZH1	1.22E-08	0.114041415	0.129	0.087	0.000294	1.3
TXNRD1	1.24E-08	0.213408094	0.466	0.385	0.000299	1.3
TOP1	1.26E-08	-0.280739557	0.309	0.354	0.0003038	1.3
CDK5RAP2	1.28E-08	0.109077491	0.086	0.053	0.0003076	1.3
KDSR	1.29E-08	0.119792791	0.141	0.097	0.000311	1.3
STK38	1.38E-08	-0.241471287	0.216	0.266	0.0003331	1.3

ATP2A2	1.41E-08	0.137248128	0.3	0.233	0.0003402	1.3
ZNF43	1.43E-08	0.133085511	0.178	0.129	0.0003444	1.3
HMGCS1	1.43E-08	0.166683409	0.281	0.219	0.0003447	1.3
CAPNS1	1.48E-08	0.145198485	0.088	0.055	0.0003571	1.3
SYNJ2	1.50E-08	0.123158088	0.366	0.294	0.0003613	1.3
ZNF721	1.51E-08	0.133726102	0.26	0.2	0.0003632	1.3
KANSL1	1.53E-08	0.150586368	0.444	0.361	0.0003686	1.3
GPATCH8	1.58E-08	0.119064158	0.306	0.239	0.0003798	1.3
TEAD1	1.58E-08	-0.302774098	0.339	0.38	0.0003818	1.3
SND1	1.59E-08	0.121817389	0.374	0.301	0.0003833	1.3
MRPL47	1.66E-08	0.107255561	0.23	0.172	0.0004012	1.3
RPS9	1.72E-08	0.26196762	0.349	0.282	0.0004137	1.3
ADNP	1.73E-08	0.129686067	0.367	0.295	0.0004164	1.3
SASH1	1.73E-08	0.129963432	0.371	0.298	0.0004169	1.3
WDR7	1.73E-08	0.114067047	0.075	0.044	0.0004171	1.3
ATP5J	1.78E-08	0.138814875	0.561	0.475	0.000428	1.3
LGALS1	1.82E-08	0.131040397	0.103	0.067	0.0004393	1.3
LINC00657	1.83E-08	0.111333827	0.192	0.141	0.000441	1.3
PAN3	1.84E-08	-0.229912225	0.558	0.581	0.0004426	1.3
IFIT2	1.87E-08	-0.283220225	0.02	0.047	0.0004517	1.3
RBM39	1.88E-08	-0.19794752	0.436	0.468	0.0004539	1.3
EPS15	1.90E-08	0.111900086	0.284	0.221	0.0004572	1.3
HS2ST1	1.90E-08	0.106508779	0.056	0.03	0.0004582	1.3
ZNF638	1.95E-08	0.138886639	0.485	0.402	0.0004708	1.3
GLTSCR1L	1.96E-08	0.144853611	0.178	0.13	0.0004722	1.3
MPRIP	1.97E-08	-0.303962379	0.195	0.239	0.0004752	1.3
FOXO1	1.98E-08	0.125228932	0.299	0.235	0.0004768	1.3
SERINC3	2.08E-08	0.132778551	0.222	0.168	0.0005004	1.3
MAT2A	2.27E-08	0.103438106	0.087	0.054	0.0005472	1.3
DISC1	2.35E-08	0.125097666	0.134	0.092	0.0005671	1.3
RBPMS	2.36E-08	-0.207975096	0.794	0.787	0.0005691	1.3
ARID4B	2.38E-08	-0.244126739	0.478	0.512	0.0005745	1.3
TMEM245	2.43E-08	0.110181724	0.13	0.088	0.000587	1.3
ATXN3	2.46E-08	0.12225695	0.207	0.153	0.0005926	1.3
FEM1B	2.50E-08	-0.212848059	0.094	0.136	0.0006034	1.3
GTPBP10	2.65E-08	0.100549137	0.133	0.091	0.00064	1.3
RP11-481C	2.70E-08	-0.149211955	0.039	0.071	0.0006516	1.3
DNER	2.74E-08	0.187863951	0.098	0.064	0.000661	1.3
FNBP4	2.74E-08	-0.24072931	0.428	0.466	0.0006618	1.3
PIN4	2.81E-08	0.116891891	0.342	0.275	0.0006781	1.3
SMARCAD1	2.82E-08	0.108159033	0.094	0.06	0.000679	1.3
NFKBIZ	2.87E-08	-0.197866967	0.798	0.796	0.0006932	1.3
EIF2AK2	2.93E-08	0.124204265	0.137	0.096	0.0007055	1.3
ATL3	2.95E-08	0.10886805	0.151	0.106	0.0007118	1.3
SMG1	2.96E-08	-0.301500394	0.225	0.27	0.0007129	1.3
ALDOA	3.00E-08	0.238654758	0.153	0.11	0.0007243	1.3
CSTB	3.07E-08	0.236738977	0.226	0.175	0.0007407	1.3

MT1E	3.12E-08	0.402267897	0.06	0.034	0.0007533	1.3
DDX5	3.27E-08	0.100834804	0.574	0.483	0.0007881	1.3
DYNC1I2	3.35E-08	0.123253134	0.431	0.358	0.0008085	1.3
MAPKAPK2	3.40E-08	-0.192730961	0.095	0.138	0.0008189	1.3
KIAA1143	3.47E-08	0.10101561	0.15	0.105	0.0008367	1.3
ZRANB2	3.51E-08	0.118755106	0.394	0.321	0.0008452	1.3
TRA2A	3.56E-08	-0.265072399	0.385	0.421	0.0008574	1.3
CASP7	3.56E-08	0.114022089	0.205	0.152	0.0008574	1.3
ZRANB1	3.78E-08	-0.208206235	0.124	0.169	0.0009111	1.3
ATP11A	3.79E-08	0.130211478	0.108	0.071	0.000915	1.3
NFE2L3	3.84E-08	-0.251554091	0.065	0.102	0.0009258	1.3
NHSL1	3.89E-08	0.170116007	0.245	0.188	0.0009382	1.3
WDR48	4.08E-08	-0.225166542	0.152	0.198	0.0009842	1.3
PCCA	4.11E-08	0.159488089	0.198	0.147	0.0009921	1.3
FGFBP1	4.24E-08	0.238518066	0.1	0.066	0.001022	1.3
PPL	4.27E-08	0.139961552	0.127	0.087	0.0010284	1.3
CCNG2	4.34E-08	-0.225860478	0.119	0.163	0.0010463	1.3
ZNF407	4.34E-08	0.145112203	0.146	0.103	0.0010467	1.3
C3orf52	4.36E-08	-0.171961865	0.044	0.077	0.0010522	1.3
ARHGAP10	4.42E-08	-0.252250314	0.104	0.146	0.001065	1.3
SAMD5	4.46E-08	0.117741654	0.085	0.053	0.0010746	1.3
TRNAU1AP	4.46E-08	0.119620201	0.322	0.257	0.0010749	1.3
DLG5	4.47E-08	0.135179825	0.211	0.157	0.0010789	1.3
GPCPD1	4.63E-08	0.159957029	0.158	0.115	0.0011169	1.3
LGR4	4.73E-08	0.103054881	0.374	0.305	0.0011395	1.3
ZFR	4.74E-08	0.114536468	0.438	0.361	0.001142	1.3
KDM3B	4.93E-08	0.154163004	0.216	0.164	0.0011882	1.3
KLF3	5.01E-08	0.132808631	0.148	0.104	0.0012073	1.3
PTEN	5.02E-08	-0.194605691	0.838	0.854	0.0012109	1.3
TASP1	5.03E-08	0.127051392	0.189	0.139	0.0012138	1.3
H1F0	5.05E-08	0.125640713	0.21	0.158	0.0012182	1.3
ANPEP	5.17E-08	0.125031155	0.061	0.035	0.0012473	1.3
MINK1	5.17E-08	0.122597941	0.177	0.13	0.0012476	1.3
BIRC6-AS2	5.21E-08	0.115594741	0.277	0.216	0.0012563	1.3
ARF6	5.25E-08	-0.26955944	0.156	0.2	0.0012648	1.3
SPEN	5.46E-08	-0.220113048	0.236	0.284	0.0013165	1.3
ACAP2	5.57E-08	0.1009194	0.191	0.142	0.0013421	1.3
HERC2	5.68E-08	0.116947745	0.11	0.074	0.0013692	1.3
MTCH2	5.68E-08	0.107344369	0.101	0.066	0.0013693	1.3
ELP2	5.94E-08	-0.197414516	0.134	0.179	0.001432	1.3
CDKAL1	5.95E-08	0.120944092	0.292	0.23	0.0014343	1.3
PPP6R3	6.04E-08	-0.250993409	0.404	0.439	0.0014564	1.3
NHP2	6.38E-08	0.100127704	0.042	0.021	0.0015395	1.3
APOO	6.40E-08	-0.123784034	0.976	0.984	0.0015443	1.3
ZNF592	6.63E-08	-0.208751582	0.094	0.134	0.0015995	1.3
TMCO4	6.68E-08	0.112449062	0.127	0.088	0.0016113	1.3
FBXO11	6.77E-08	-0.274604208	0.355	0.396	0.0016327	1.3

RBPJ	6.95E-08	-0.245731325	0.373	0.412	0.0016758	1.3
CNNM2	7.31E-08	0.10349407	0.077	0.047	0.0017617	1.3
RP11-608O	7.62E-08	-0.202128539	0.736	0.737	0.0018374	1.3
C1orf56	7.65E-08	0.117073311	0.197	0.148	0.0018445	1.3
COX7C	7.73E-08	-0.139822581	0.887	0.888	0.0018646	1.3
MYO5A	7.97E-08	0.12844197	0.113	0.076	0.0019207	1.3
ELMO2	8.20E-08	-0.11236194	0.041	0.072	0.0019768	1.3
TBC1D8	8.21E-08	-0.263729035	0.375	0.416	0.0019806	1.3
NUMB	8.32E-08	0.130081505	0.501	0.426	0.0020054	1.3
GJC3	8.41E-08	-0.201798131	0.06	0.094	0.0020282	1.3
SDK1	8.88E-08	0.156851928	0.117	0.079	0.0021411	1.3
ACTN1	8.91E-08	-0.324448371	0.242	0.287	0.002148	1.3
KIAA1328	8.98E-08	0.123559382	0.117	0.079	0.0021645	1.3
AUTS2	9.05E-08	0.136165789	0.628	0.541	0.002182	1.3
MYH14	9.05E-08	0.12212835	0.207	0.157	0.0021831	1.3
KLHL5	9.53E-08	0.134556296	0.216	0.164	0.0022979	1.3
KCTD3	9.72E-08	-0.260617563	0.167	0.212	0.0023432	1.3
WDR60	9.83E-08	0.150627538	0.156	0.113	0.0023711	1.3
CBR4	9.99E-08	0.115327576	0.314	0.25	0.0024096	1.3
LURAP1L	1.02E-07	0.111915534	0.267	0.206	0.0024493	1.3
CLEC7A	1.06E-07	0.101574531	0.162	0.116	0.0025668	1.3
EGR1	1.12E-07	0.195047467	0.102	0.068	0.0027125	1.3
C9orf3	1.13E-07	0.101700418	0.207	0.154	0.002723	1.3
ATP5L	1.13E-07	0.114967595	0.879	0.849	0.0027238	1.3
NSMAF	1.15E-07	0.106820054	0.122	0.084	0.002769	1.3
HDAC1	1.19E-07	0.102166938	0.144	0.102	0.0028756	1.3
MYO1C	1.22E-07	0.110350712	0.056	0.032	0.0029325	1.3
DAPL1	1.26E-07	-0.100798462	0.029	0.057	0.0030464	1.3
AKAP12	1.31E-07	-0.343563266	0.02	0.043	0.0031684	1.3
PLA2G16	1.31E-07	0.12333326	0.162	0.118	0.0031705	1.3
RAB10	1.35E-07	0.140706981	0.325	0.262	0.0032562	1.3
CTNND1	1.35E-07	-0.246414499	0.291	0.329	0.003259	1.3
UBE3A	1.37E-07	-0.21739843	0.291	0.338	0.0032921	1.3
PITPNA	1.42E-07	0.125772648	0.178	0.131	0.0034326	1.3
ADGRL3-AS	1.45E-07	-0.208710627	0.651	0.671	0.003492	1.3
RGS6	1.46E-07	0.125063342	0.18	0.134	0.0035324	1.3
LSAMP	1.48E-07	0.128594019	0.294	0.234	0.003572	1.3
TCN1	1.50E-07	0.212745644	0.053	0.03	0.0036095	1.3
SEC24B	1.51E-07	-0.249218848	0.267	0.311	0.0036319	1.3
KIF13B	1.53E-07	-0.325097847	0.265	0.305	0.0036913	1.3
VPS41	1.55E-07	0.10728014	0.106	0.071	0.0037289	1.3
UACA	1.56E-07	0.126656371	0.203	0.154	0.0037537	1.3
IER2	1.57E-07	0.179356024	0.09	0.059	0.0037962	1.3
IFNAR2	1.58E-07	0.12565546	0.354	0.285	0.0038025	1.3
ZNF254	1.59E-07	0.110182966	0.197	0.148	0.0038415	1.3
ZCCHC2	1.61E-07	0.125557156	0.358	0.292	0.0038891	1.3
CCL4	1.63E-07	-1.269192278	0.062	0.096	0.0039224	1.3

WDR43	1.71E-07	-0.219190372	0.083	0.12	0.0041177	1.3
CCDC174	1.72E-07	0.103927244	0.224	0.171	0.0041562	1.3
RP11-511B	1.74E-07	-0.206723487	0.574	0.595	0.0041871	1.3
KHDRBS1	1.75E-07	-0.229253646	0.26	0.306	0.0042312	1.3
DIS3L2	1.76E-07	0.101162075	0.135	0.095	0.004243	1.3
ZNF544	1.78E-07	0.114513022	0.129	0.09	0.0042825	1.3
APPL2	1.78E-07	0.105588594	0.098	0.065	0.004293	1.3
HS3ST4	1.79E-07	0.145364389	0.398	0.328	0.0043165	1.3
PRR14L	1.86E-07	0.110206304	0.115	0.079	0.0044965	1.3
MBOAT1	1.90E-07	-0.163295195	0.045	0.076	0.0045751	1.3
BAZ2B	1.90E-07	0.128771133	0.503	0.42	0.0045908	1.3
TNFAIP3	1.92E-07	-0.297684346	0.22	0.265	0.0046342	1.3
SPDYE16	1.93E-07	0.108109513	0.127	0.089	0.0046467	1.3
SBF2	1.98E-07	-0.242406126	0.658	0.658	0.0047799	1.3
APTR	2.00E-07	-0.16139584	0.057	0.09	0.0048309	1.3
ODF2L	2.02E-07	0.113463577	0.176	0.13	0.0048631	1.3
TAF3	2.06E-07	0.107928025	0.087	0.056	0.0049681	1.3
TRPC1	2.07E-07	0.110739376	0.067	0.04	0.0049843	1.3
BCL2	2.07E-07	-0.22651586	0.099	0.139	0.0049876	1.3
DHRS3	2.09E-07	0.104062174	0.119	0.082	0.0050392	1.3
SS18	2.11E-07	0.140152031	0.183	0.138	0.0050838	1.3
ZBTB16	2.13E-07	0.131313724	0.322	0.262	0.0051474	1.3
RUFY3	2.14E-07	0.103982042	0.213	0.163	0.005166	1.3
USP32	2.15E-07	0.133439792	0.24	0.187	0.0051792	1.3
AFF4	2.16E-07	-0.23458745	0.406	0.438	0.0052152	1.3
RP11-286E	2.18E-07	-0.169730609	0.04	0.069	0.0052547	1.3
NCK2	2.21E-07	0.104294111	0.202	0.152	0.0053371	1.3
RABGAP1	2.24E-07	0.114598639	0.356	0.285	0.0054126	1.3
FLOT1	2.35E-07	0.120109103	0.143	0.103	0.0056554	1.3
RUBCN	2.35E-07	-0.110215599	0.044	0.075	0.0056767	1.3
RAB6A	2.46E-07	0.100442212	0.342	0.276	0.0059209	1.3
MAP7D1	2.51E-07	-0.193316988	0.103	0.143	0.0060598	1.3
SCFD1	2.53E-07	0.114781169	0.269	0.212	0.0061119	1.3
CACNB2	2.55E-07	0.135359506	0.176	0.132	0.0061368	1.3
MVP	2.55E-07	0.11168171	0.092	0.061	0.0061592	1.3
UBAP2	2.57E-07	0.118962147	0.228	0.175	0.0062044	1.3
SPINK5	2.58E-07	-0.145040622	0.007	0.024	0.0062199	1.3
LRRC37A3	2.67E-07	0.110083885	0.153	0.112	0.0064357	1.3
NFIA	2.77E-07	0.116061938	0.161	0.118	0.0066863	1.3
ASH1L	2.79E-07	0.118588267	0.562	0.477	0.0067218	1.3
PHC3	2.84E-07	0.124124176	0.222	0.171	0.0068496	1.3
CHMP2A	2.92E-07	0.101633532	0.081	0.052	0.007051	1.3
CDK13	2.95E-07	0.111868313	0.404	0.331	0.0071071	1.3
NTRK2	2.95E-07	-0.219206632	0.156	0.199	0.0071102	1.3
CLCN3	2.97E-07	0.11936057	0.226	0.175	0.007163	1.3
FAM172A	3.06E-07	-0.273452144	0.752	0.747	0.0073765	1.3
HIST2H2BE	3.09E-07	0.105961289	0.121	0.085	0.007439	1.3

WSB1	3.09E-07	-0.248120014	0.309	0.347	0.007452	1.3
MAGT1	3.16E-07	-0.221308949	0.148	0.19	0.00761	1.3
PTK7	3.16E-07	0.105431666	0.119	0.083	0.0076298	1.3
AGO3	3.28E-07	0.104453697	0.291	0.233	0.0079138	1.3
KLF10	3.30E-07	0.10047245	0.136	0.097	0.0079567	1.3
REL	3.34E-07	-0.248396413	0.207	0.248	0.0080447	1.3
GBAS	3.40E-07	0.104658407	0.231	0.178	0.0082021	1.3
OAT	3.51E-07	0.101079545	0.425	0.355	0.0084593	1.3
C8orf4	3.52E-07	0.101808247	0.191	0.145	0.0084862	1.3
TBCK	3.58E-07	0.124037866	0.156	0.115	0.0086325	1.3
NPC1	3.62E-07	-0.286842206	0.296	0.333	0.0087271	1.3
DDR2	3.67E-07	0.135773268	0.147	0.107	0.008843	1.3
TMEM57	3.75E-07	-0.213345873	0.141	0.182	0.0090316	1.3
SH3GL1	3.76E-07	-0.179009685	0.065	0.098	0.0090749	1.3
RPS27A	3.84E-07	-0.102773232	0.979	0.988	0.0092477	1.3
RP11-65I12	3.85E-07	0.133125381	0.074	0.046	0.0092751	1.3
ADCY9	3.95E-07	0.112204742	0.047	0.026	0.0095263	1.3
TNRC6B	3.98E-07	0.109659138	0.475	0.399	0.0095972	1.3
FTO	4.08E-07	0.133250556	0.279	0.222	0.0098403	1.3
RGS10	4.09E-07	0.11069357	0.15	0.109	0.0098528	1.3
PHYKPL	4.16E-07	0.103884699	0.075	0.047	0.0100242	1.3
MPRIP-AS1	4.17E-07	-0.150461361	0.036	0.063	0.0100535	1.3
SDCCAG8	4.29E-07	0.117884676	0.221	0.171	0.0103421	1.3
PTP4A2	4.41E-07	-0.182178966	0.088	0.124	0.0106405	1.3
RAD51B	4.45E-07	0.144818002	0.266	0.21	0.0107361	1.3
TRIM56	4.53E-07	0.103635378	0.244	0.19	0.0109119	1.3
ARHGEF7	4.57E-07	-0.229029862	0.142	0.183	0.0110224	1.3
RHBDD1	4.57E-07	0.103704381	0.158	0.116	0.011027	1.3
MBOAT2	4.75E-07	0.101667494	0.147	0.107	0.0114455	1.3
IKBKB	4.80E-07	0.132072591	0.147	0.107	0.0115797	1.3
LARP1	4.86E-07	0.104888337	0.215	0.167	0.0117248	1.3
NEDD4	4.91E-07	0.134543858	0.111	0.077	0.0118507	1.3
CLASP1	5.17E-07	0.130167268	0.3	0.242	0.012468	1.3
COMT	5.27E-07	0.106724391	0.075	0.047	0.0127151	1.3
HDAC4	5.36E-07	0.141613383	0.06	0.036	0.0129178	1.3
PARVA	5.42E-07	0.115016358	0.176	0.132	0.0130698	1.3
RNF150	5.47E-07	-0.219247993	0.107	0.147	0.0131818	1.3
SETD5	5.70E-07	-0.231601999	0.504	0.524	0.0137561	1.3
MLH3	5.81E-07	0.106989236	0.128	0.091	0.0140052	1.3
MSRA	5.87E-07	0.118872601	0.093	0.062	0.0141565	1.3
FGD6	6.10E-07	0.144535176	0.293	0.239	0.0147009	1.3
MYL12A	6.17E-07	-0.261760234	0.86	0.858	0.0148716	1.3
CASP4	6.31E-07	0.112768899	0.339	0.274	0.0152204	1.3
RNF168	6.34E-07	0.107631629	0.103	0.071	0.015283	1.3
FERMT2	6.42E-07	-0.206433805	0.132	0.172	0.0154837	1.3
REEP3	6.45E-07	0.101654542	0.288	0.231	0.0155567	1.3
MYO9B	6.57E-07	-0.25491422	0.172	0.213	0.0158384	1.3

FMO2	6.81E-07	-0.16874332	0.089	0.127	0.0164266	1.3
RPL26L1	6.91E-07	0.103724727	0.215	0.165	0.0166587	1.3
B4GALT1	6.94E-07	0.115731645	0.46	0.385	0.0167241	1.3
CHMP4B	6.94E-07	-0.195159732	0.096	0.133	0.0167391	1.3
EP300	7.27E-07	0.136026358	0.175	0.133	0.0175404	1.3
AC072062.	7.43E-07	-0.221417124	0.508	0.527	0.0179215	1.3
ERBB2IP	7.54E-07	0.114761553	0.318	0.257	0.0181699	1.3
EIF3L	8.09E-07	-0.169375707	0.763	0.761	0.0194956	1.3
XRN1	8.28E-07	-0.229658097	0.312	0.35	0.0199758	1.3
ALPL	8.46E-07	-0.157103707	0.038	0.066	0.0203924	1.3
PFDN4	8.59E-07	0.11072783	0.568	0.493	0.0207175	1.3
KLRD1	8.69E-07	-0.167412566	0.05	0.08	0.0209473	1.3
EEF2	8.80E-07	0.212241554	0.495	0.429	0.0212291	1.3
C11orf1	8.92E-07	-0.20468772	0.151	0.19	0.0215102	1.3
SNHG8	8.96E-07	-0.221810094	0.287	0.328	0.0215951	1.3
PTPRM	9.14E-07	0.170175023	0.208	0.162	0.0220497	1.3
RP11-138A	9.34E-07	0.103060235	0.09	0.06	0.0225301	1.3
JAZF1	9.39E-07	-0.153209938	0.094	0.131	0.0226375	1.3
MT-ND4L	9.43E-07	-0.220378496	0.528	0.536	0.0227392	1.3
NEK10	9.55E-07	0.117742896	0.151	0.11	0.0230239	1.3
ARF4	1.01E-06	-0.234452028	0.648	0.654	0.0244071	1.3
MCTS1	1.05E-06	0.105795649	0.336	0.274	0.0254026	1.3
GCNT1	1.08E-06	-0.218723982	0.068	0.1	0.0260113	1.3
ARPP19	1.09E-06	-0.281979097	0.313	0.341	0.0263105	1.3
LDHC	1.11E-06	-0.118543479	0.012	0.031	0.0266476	1.3
ABCC4	1.14E-06	-0.122403802	0.02	0.042	0.027404	1.3
CCNL1	1.14E-06	-0.209497922	0.368	0.407	0.0274478	1.3
ZNF106	1.21E-06	0.111844775	0.148	0.109	0.0290661	1.3
RNF144B	1.23E-06	0.107788112	0.398	0.333	0.0297153	1.3
REPS1	1.23E-06	0.108992509	0.313	0.254	0.0297268	1.3
SLC25A25	1.26E-06	0.152861694	0.186	0.143	0.0304474	1.3
RHPN2	1.26E-06	0.103688927	0.332	0.271	0.0304486	1.3
KLHL20	1.28E-06	0.124202263	0.098	0.067	0.0309739	1.3
NLGN4X	1.32E-06	0.12329379	0.102	0.071	0.0318246	1.3
WWTR1	1.35E-06	-0.225241872	0.385	0.416	0.0325328	1.3
WASF1	1.36E-06	0.104093385	0.077	0.05	0.0328199	1.3
AGTPBP1	1.37E-06	0.11501159	0.107	0.074	0.0330125	1.3
SMAD2	1.38E-06	0.138128929	0.277	0.225	0.0332124	1.3
PSME2	1.44E-06	0.190325125	0.395	0.339	0.0347737	1.3
RAB8B	1.46E-06	-0.230685974	0.137	0.175	0.0352663	1.3
PYGB	1.47E-06	0.110906435	0.072	0.046	0.0353864	1.3
SNX18	1.49E-06	-0.126025464	0.04	0.067	0.0359265	1.3
ZCCHC7	1.50E-06	0.123480828	0.441	0.376	0.0361276	1.3
ROCK1	1.54E-06	0.118682527	0.244	0.194	0.0370655	1.3
CCL5	1.57E-06	-0.105180136	0.012	0.03	0.0378992	1.3
SUCLG2	1.83E-06	0.129116318	0.124	0.089	0.0440697	1.3
TBC1D9	1.83E-06	0.112036253	0.264	0.211	0.0442196	1.3

IMMP2L	1.88E-06	0.151368036	0.361	0.302	0.0453829	1.3
CLDN1	1.96E-06	-0.49392221	0.397	0.418	0.0472789	1.3
ZCCHC8	2.03E-06	-0.179135057	0.106	0.142	0.048986	1.3
RP11-241G	2.07E-06	-0.103694773	0.025	0.048	0.0499049	1.3
CEPT1	2.12E-06	0.11128851	0.329	0.271	0.051013	1.3
IDH2	2.12E-06	0.103540076	0.112	0.079	0.0511485	1.3
UPF2	2.13E-06	0.108557656	0.328	0.27	0.0512583	1.3
AC016831.	2.14E-06	0.137778852	0.298	0.241	0.051558	1.3
CHD1	2.19E-06	-0.202819538	0.202	0.242	0.052799	1.3
UBE2E2	2.19E-06	0.126884862	0.453	0.384	0.0528253	1.3
C1orf132	2.22E-06	0.111923716	0.089	0.06	0.0534844	1.3
RP11-486O	2.34E-06	-0.12739668	0.036	0.061	0.0564813	1.3
CHD6	2.40E-06	-0.216233876	0.268	0.304	0.0578625	1.3
MYO1D	2.41E-06	0.11337736	0.31	0.252	0.0580912	1.3
SWAP70	2.45E-06	-0.26386043	0.278	0.313	0.0590958	1.3
R3HDM1	2.47E-06	0.116349892	0.081	0.054	0.0595198	1.3
BTF3	2.49E-06	-0.173221567	0.948	0.946	0.0600352	1.3
TTTY14	2.51E-06	-0.288260526	0.275	0.312	0.0605945	1.3
MYO9A	2.51E-06	0.107858204	0.34	0.278	0.0606295	1.3
PPA2	2.54E-06	0.103773019	0.239	0.19	0.0612691	1.3
CDKL5	2.60E-06	-0.204670059	0.065	0.096	0.0627031	1.3
RCAN3	2.60E-06	-0.169328015	0.226	0.174	0.0627644	1.3
ARIH1	2.62E-06	-0.205177835	0.394	0.431	0.0632261	1.3
DYRK3	2.70E-06	-0.145385141	0.054	0.083	0.0651895	1.3
MED15	2.75E-06	-0.14735718	0.114	0.151	0.0663144	1.3
ZNF37A	2.79E-06	0.110828076	0.117	0.084	0.0673687	1.3
CRNDE	2.84E-06	0.118552954	0.094	0.065	0.0684002	1.3
SH3PXD2B	2.88E-06	0.102114182	0.162	0.122	0.0695437	1.3
PIGA	3.12E-06	-0.149677821	0.061	0.091	0.0752991	1.3
RIC1	3.23E-06	0.118352225	0.136	0.1	0.0779729	1.3
RALGAPA1	3.24E-06	0.127022097	0.274	0.223	0.0780303	1.3
SNRPB	3.24E-06	0.110523212	0.105	0.074	0.0781533	1.3
RPF2	3.56E-06	-0.187225859	0.154	0.192	0.0857948	1.3
METTL5	3.64E-06	0.107870381	0.138	0.103	0.0877139	1.3
PLCG2	3.89E-06	0.131505002	0.086	0.058	0.0939183	1.3
SHROOM3	4.00E-06	-0.269536228	0.515	0.526	0.0965577	1.3
NAA25	4.03E-06	-0.348606336	0.3	0.325	0.0972856	1.3
MACROD2	4.06E-06	0.109510247	0.303	0.249	0.0978257	1.3
ATP6V0A1	4.33E-06	-0.167549868	0.143	0.182	0.1044949	1.3
UBAP1	4.40E-06	-0.229648629	0.31	0.345	0.1059851	1.3
FARS2	4.42E-06	0.121577324	0.107	0.076	0.1065442	1.3
AKT3	4.50E-06	-0.337794123	0.466	0.48	0.1085803	1.3
PRKRIP1	4.55E-06	0.110708838	0.17	0.13	0.1096871	1.3
RPL41	4.58E-06	-0.199856716	0.952	0.955	0.110386	1.3
RNGTT	4.66E-06	0.104745461	0.082	0.055	0.1124384	1.3
SCAF4	5.08E-06	-0.137010208	0.061	0.09	0.1225176	1.3
CHD8	5.20E-06	0.103210333	0.117	0.085	0.1254107	1.3

NIPBL	5.25E-06	-0.206536781	0.461	0.483	0.1266947	1.3
ELMOD3	5.59E-06	0.115833471	0.112	0.081	0.1348912	1.3
SMARCA2	5.70E-06	0.103474794	0.347	0.286	0.1374522	1.3
RANBP2	5.76E-06	-0.242183894	0.251	0.286	0.1389174	1.3
RP11-84A1	6.09E-06	-0.10994154	0.025	0.047	0.1467937	1.3
PTN	6.17E-06	-0.497518213	0.203	0.241	0.1487797	1.3
METTL15	6.64E-06	0.109674579	0.16	0.122	0.160012	1.3
TFDP2	6.72E-06	0.135153282	0.264	0.216	0.1620176	1.3
MB21D2	6.76E-06	-0.149816681	0.053	0.08	0.163052	1.3
TIAM1	6.87E-06	-0.370031037	0.29	0.318	0.1657068	1.3
EHF	6.91E-06	-0.265405392	0.607	0.595	0.1665475	1.3
TAPBP	6.93E-06	0.147871861	0.151	0.116	0.1669987	1.3
ZDHHC17	6.94E-06	0.106785087	0.118	0.086	0.1672883	1.3
TMEM159	6.97E-06	-0.21852813	0.415	0.433	0.1681093	1.3
RPS19	7.03E-06	0.265124466	0.3	0.252	0.1694768	1.3
GAREM1	7.31E-06	0.135899016	0.305	0.253	0.1761871	1.3
DAPP1	7.74E-06	-0.328438828	0.399	0.418	0.1865838	1.3
RPL7A	7.91E-06	-0.118714493	0.966	0.979	0.1907172	1.3
PISD	8.03E-06	-0.188435075	0.149	0.185	0.1936466	1.3
GTF3C2	8.20E-06	0.100964716	0.091	0.063	0.1977687	1.3
RPL22L1	8.22E-06	0.12179071	0.605	0.547	0.1982944	1.3
MTF2	8.32E-06	-0.180273333	0.186	0.224	0.2006604	1.3
MGEA5	8.56E-06	-0.209460917	0.379	0.408	0.2064906	1.3
ARMC9	8.63E-06	0.15081822	0.127	0.094	0.2080124	1.3
ZNF292	8.92E-06	-0.213067829	0.633	0.636	0.2152024	1.3
SERF2	9.02E-06	0.185779075	0.301	0.251	0.2175768	1.3
SERINC2	9.08E-06	0.111030358	0.107	0.077	0.219039	1.3
PFN1	9.12E-06	0.103077798	0.209	0.167	0.2199016	1.3
PIP5K1A	9.22E-06	-0.216359763	0.28	0.312	0.2224385	1.3
BCL10	9.29E-06	-0.178673977	0.104	0.137	0.224049	1.3
PNPLA8	9.43E-06	-0.283053565	0.267	0.297	0.2274216	1.3
NDUFA1	9.54E-06	0.103505978	0.61	0.544	0.2300157	1.3
RBM26	9.74E-06	0.120749749	0.242	0.195	0.2348623	1.3
ZNF91	9.89E-06	0.105653019	0.176	0.136	0.2384304	1.3
ABI2	9.93E-06	0.100188542	0.179	0.14	0.2393369	1.3
DPH5	1.01E-05	-0.108698852	0.062	0.092	0.2426044	1.3
TMEM161E	1.03E-05	0.110568239	0.124	0.091	0.2480066	1.3
DTX2	1.03E-05	0.112602179	0.127	0.095	0.2484014	1.3
MAP2K4	1.04E-05	0.10809857	0.412	0.352	0.25073	1.3
KLF5	1.13E-05	-0.203158802	0.202	0.238	0.2728432	1.3
RNPC3	1.14E-05	0.107201768	0.098	0.07	0.2757225	1.3
SRPK2	1.17E-05	-0.231592012	0.358	0.385	0.2814832	1.3
C11orf49	1.17E-05	0.115722429	0.172	0.132	0.2827864	1.3
KPNA6	1.17E-05	-0.221657347	0.251	0.286	0.2830519	1.3
NEDD9	1.20E-05	-0.254063371	0.246	0.279	0.2899981	1.3
TOP2B	1.25E-05	-0.205406987	0.152	0.187	0.3019751	1.3
DDIT4	1.27E-05	0.142024306	0.243	0.2	0.3069821	1.3

STRBP	1.29E-05	0.113518689	0.223	0.18	0.3112557	1.3
MARK3	1.30E-05	-0.187047414	0.41	0.44	0.3136113	1.3
UHRF1BP1I	1.30E-05	-0.218026874	0.168	0.203	0.3143587	1.3
GNL2	1.31E-05	-0.149355328	0.08	0.11	0.3160612	1.3
AC016995.	1.33E-05	-0.235880106	0.171	0.207	0.3208763	1.3
KMT5B	1.39E-05	0.102190824	0.192	0.151	0.3342762	1.3
SLC25A6	1.42E-05	0.156542465	0.166	0.13	0.34127	1.3
CAMSAP2	1.47E-05	0.102253617	0.155	0.119	0.3535104	1.3
RPS15	1.52E-05	0.219518873	0.202	0.16	0.366633	1.3
COX7A2	1.56E-05	0.132258529	0.809	0.795	0.3749979	1.3
PHC2	1.56E-05	0.124351873	0.113	0.083	0.375758	1.3
NFKBIA	1.57E-05	-0.300129017	0.63	0.62	0.3787683	1.3
ZFAND1	1.63E-05	-0.21281422	0.255	0.286	0.3937844	1.3
TAGLN	1.64E-05	0.166529877	0.037	0.021	0.3960258	1.3
HNRNPM	1.71E-05	-0.177884039	0.167	0.203	0.4128125	1.3
ANXA11	1.81E-05	-0.239220245	0.221	0.254	0.4366025	1.3
ACTN4	1.81E-05	-0.230499349	0.311	0.343	0.436703	1.3
FRMD6	1.81E-05	-0.201488011	0.169	0.205	0.4376388	1.3
FAM60A	1.87E-05	0.13506465	0.441	0.379	0.4502609	1.3
ELF3	2.14E-05	-0.246474196	0.529	0.54	0.5159851	1.3
PRDM2	2.23E-05	-0.204880391	0.29	0.321	0.5369989	1.3
TCEB3	2.31E-05	-0.102586156	0.053	0.079	0.5567311	1.3
EFR3A	2.33E-05	-0.208999004	0.139	0.173	0.5618065	1.3
DENND2C	2.37E-05	-0.118781702	0.026	0.046	0.5724849	1.3
FASTKD1	2.50E-05	0.105768448	0.07	0.047	0.6037989	1.3
EIF1AX	2.53E-05	-0.210229429	0.214	0.246	0.6093072	1.3
LINGO1	2.55E-05	-0.1622208	0.883	0.884	0.6137231	1.3
OTUD3	2.56E-05	-0.111732988	0.032	0.054	0.6183745	1.3
CXADR	2.60E-05	0.121876312	0.264	0.219	0.6257827	1.3
LINC01344	2.61E-05	-0.13395728	0.027	0.047	0.6286519	1.3
RP11-290O	2.68E-05	0.118774438	0.118	0.088	0.6459634	1.3
EEF1B2	2.71E-05	-0.170786648	0.876	0.886	0.6529694	1.3
PVT1	2.72E-05	-0.224167959	0.372	0.396	0.6564209	1.3
MARCKS	2.86E-05	-0.242421944	0.414	0.434	0.689218	1.3
ITGB1	2.87E-05	-0.293554816	0.339	0.363	0.6920236	1.3
ARFGEF3	2.93E-05	-0.230444399	0.203	0.237	0.7056734	1.3
LAMC1	3.00E-05	-0.209616044	0.242	0.274	0.7237356	1.3
SSR3	3.24E-05	-0.225079885	0.596	0.591	0.7813899	1.3
KLHL24	3.27E-05	-0.2133207	0.256	0.285	0.7874781	1.3
IL32	3.34E-05	0.115121845	0.091	0.065	0.8053638	1.3
USMG5	3.48E-05	0.143366939	0.624	0.575	0.8397929	1.3
KDM3A	3.63E-05	-0.137832511	0.087	0.117	0.8764078	1.3
SERPINB9	3.64E-05	-0.475281252	0.134	0.167	0.8782796	1.3
RAB5A	3.66E-05	-0.171828868	0.195	0.23	0.8833827	1.3
KIDINS220	3.95E-05	-0.132410525	0.152	0.187	0.9520392	1.3
EEF1D	3.98E-05	0.107667891	0.148	0.114	0.9604206	1.3
F11R	4.01E-05	-0.182091783	0.152	0.184	0.9673449	1.3

TUFT1	4.03E-05	-0.130348318	0.125	0.159	0.9719897	1.3
VGLL4	4.07E-05	0.105891177	0.304	0.255	0.9815294	1.3
HBS1L	4.18E-05	-0.19544919	0.171	0.204	1	1.3
PPP2R2D	4.29E-05	-0.178425885	0.104	0.134	1	1.3
ESD	4.44E-05	-0.208977305	0.448	0.459	1	1.3
TMEM258	4.58E-05	-0.163315514	0.628	0.631	1	1.3
ACTB	4.66E-05	-0.282595937	0.612	0.617	1	1.3
KLK5	4.67E-05	-0.194280527	0.052	0.075	1	1.3
IPO7	4.68E-05	-0.242966637	0.272	0.297	1	1.3
RASGEF1B	4.89E-05	-0.154293438	0.894	0.894	1	1.3
RPLP2	4.93E-05	0.180541698	0.265	0.223	1	1.3
CHSY1	5.16E-05	-0.14538849	0.077	0.104	1	1.3
MAMDC2	5.18E-05	-0.136647634	0.941	0.939	1	1.3
EML4	5.27E-05	-0.182947641	0.24	0.273	1	1.3
SMIM14	5.40E-05	0.100809277	0.183	0.147	1	1.3
RPS25	5.46E-05	-0.183522288	0.876	0.862	1	1.3
EDF1	5.46E-05	0.132306587	0.097	0.071	1	1.3
USP47	5.48E-05	-0.201087514	0.29	0.321	1	1.3
ASCC3	5.61E-05	0.106360631	0.241	0.198	1	1.3
NOD2	5.73E-05	-0.109366455	0.06	0.086	1	1.3
KYNU	5.76E-05	-0.210831029	0.444	0.358	1	1.3
PJA2	6.13E-05	-0.196538431	0.205	0.237	1	1.3
CDK6	6.14E-05	0.120686694	0.263	0.218	1	1.3
SYMPK	6.74E-05	0.101688063	0.086	0.062	1	1.3
YY1AP1	6.86E-05	-0.149496106	0.145	0.176	1	1.3
IL6R	6.91E-05	0.105292875	0.094	0.069	1	1.3
CSRP1	6.94E-05	-0.152297047	0.115	0.145	1	1.3
TNFRSF10E	6.97E-05	-0.185002542	0.173	0.205	1	1.3
LATS2	7.17E-05	-0.153094502	0.098	0.128	1	1.3
RREB1	7.74E-05	0.101583976	0.3	0.253	1	1.3
MAMLD1	7.76E-05	-0.134176694	0.042	0.063	1	1.3
DDX46	7.78E-05	0.109354459	0.125	0.096	1	1.3
PITPNB	7.90E-05	-0.22072882	0.295	0.322	1	1.3
GPM6B	7.94E-05	-0.196099115	0.182	0.214	1	1.3
HLA-E	8.17E-05	0.106829567	0.142	0.111	1	1.3
SESN2	8.21E-05	-0.174292246	0.085	0.112	1	1.3
ERO1A	8.30E-05	-0.31585306	0.336	0.357	1	1.3
EIF2A	8.34E-05	-0.197042237	0.423	0.437	1	1.3
CAMSAP1	8.38E-05	-0.155650806	0.059	0.083	1	1.3
RTCB	8.66E-05	-0.213573126	0.44	0.456	1	1.3
INSIG2	8.88E-05	-0.24493601	0.215	0.243	1	1.3
FAM19A2	9.03E-05	-0.201908572	0.625	0.638	1	1.3
CYFIP1	9.10E-05	0.113430762	0.148	0.115	1	1.3
BCAS3	9.12E-05	-0.101138619	0.849	0.838	1	1.3
APOLD1	9.49E-05	-0.109406267	0.046	0.068	1	1.3
HNRNPD	9.66E-05	-0.193824305	0.267	0.297	1	1.3
DTNBP1	9.81E-05	0.106766307	0.074	0.052	1	1.3

EPN2	9.91E-05	0.101938796	0.158	0.125	1	1.3
CD47	0.0001	-0.175065092	0.418	0.443	1	1.3
MORN2	0.000107	-0.107407546	0.038	0.058	1	1.3
NUBPL	0.000108	0.100321047	0.079	0.057	1	1.3
TBC1D3P1-	0.000108	-0.186632137	0.745	0.753	1	1.3
RP11-66B2	0.000111	0.124572699	0.102	0.076	1	1.3
ZC3H15	0.000111	-0.166509391	0.246	0.276	1	1.3
TM9SF3	0.000115	-0.185743947	0.258	0.288	1	1.3
CEBPD	0.000117	0.121238792	0.252	0.211	1	1.3
MACF1	0.000122	-0.23113628	0.642	0.631	1	1.3
DSG2	0.000126	-0.169335455	0.338	0.366	1	1.3
RP11-68E1	0.000127	-0.112122451	0.039	0.059	1	1.3
GTF2IRD1	0.000127	-0.16690757	0.125	0.154	1	1.3
UBA6	0.000128	-0.165299855	0.314	0.343	1	1.3
IQGAP1	0.00013	-0.181056908	0.42	0.439	1	1.3
SH3BP5	0.000135	-0.104637315	0.078	0.105	1	1.3
OLFM4	0.000138	0.160950125	0.053	0.035	1	1.3
ZBTB43	0.00014	-0.15635165	0.137	0.167	1	1.3
ABL1	0.000141	0.113790915	0.156	0.125	1	1.3
PALLD	0.000142	-0.212148377	0.51	0.517	1	1.3
RABGGTB	0.000142	-0.139198836	0.075	0.1	1	1.3
SEC63	0.000142	-0.165716508	0.229	0.259	1	1.3
ASXL1	0.000147	-0.13246999	0.241	0.273	1	1.3
SESTD1	0.000148	-0.153864498	0.619	0.626	1	1.3
PIWIL1	0.000149	-0.126564873	0.042	0.063	1	1.3
MAX	0.00015	-0.100456681	0.156	0.189	1	1.3
MBD2	0.000157	-0.227287381	0.265	0.29	1	1.3
TAB2	0.000158	-0.223457729	0.252	0.277	1	1.3
HNRNPU	0.000159	-0.125206421	0.084	0.109	1	1.3
SSBP2	0.000177	-0.361941551	0.332	0.35	1	1.3
VCL	0.000184	-0.166731215	0.302	0.331	1	1.3
CRABP2	0.000184	-0.123852767	0.065	0.089	1	1.3
DAPK2	0.000189	-0.289322611	0.443	0.447	1	1.3
RNF115	0.000208	-0.230111587	0.219	0.246	1	1.3
SLC26A3	0.000209	-0.141290546	0.747	0.766	1	1.3
KRT80	0.000218	-0.110289859	0.029	0.046	1	1.3
ACTG1	0.00022	0.118637796	0.723	0.688	1	1.3
FOXK1	0.000222	-0.140382265	0.125	0.154	1	1.3
RHEB	0.00023	-0.197365952	0.23	0.258	1	1.3
TRAK2	0.000233	0.103290357	0.052	0.034	1	1.3
ARHGAP26	0.000236	-0.215745064	0.21	0.24	1	1.3
RIF1	0.000243	-0.150639953	0.306	0.334	1	1.3
RP11-356C	0.000245	-0.243978131	0.231	0.26	1	1.3
PHF3	0.000258	-0.172319986	0.322	0.351	1	1.3
SCGB1D2	0.000287	-0.589157909	0.076	0.101	1	1.3
NDUFV2	0.000311	-0.172782215	0.134	0.161	1	1.3
KIAA1324	0.000311	-0.138145578	0.089	0.114	1	1.3

RTKN2	0.000317	-0.104631428	0.024	0.04	1	1.3
MAP3K8	0.000329	-0.20142065	0.261	0.287	1	1.3
CHCHD3	0.00033	-0.136953749	0.89	0.881	1	1.3
ARL5B	0.000333	-0.122032973	0.056	0.078	1	1.3
ACTR3	0.000339	-0.170861264	0.284	0.309	1	1.3
MCTP1	0.000342	0.148654581	0.125	0.099	1	1.3
INTS12	0.000343	-0.192828479	0.173	0.2	1	1.3
TCF7L2	0.000347	-0.229326032	0.436	0.45	1	1.3
CCDC85C	0.000349	-0.103018895	0.041	0.06	1	1.3
LYST	0.000349	-0.181729228	0.147	0.174	1	1.3
ZFP36L2	0.000351	-0.322176723	0.35	0.367	1	1.3
ATP13A5	0.000352	-0.104457814	0.033	0.051	1	1.3
PTBP2	0.000361	-0.185889986	0.446	0.459	1	1.3
RP11-496N	0.000374	-0.153066024	0.009	0.02	1	1.3
IFT57	0.00038	-0.158397753	0.107	0.133	1	1.3
MRPS33	0.000381	-0.179353773	0.29	0.315	1	1.3
OLA1	0.000383	0.100861874	0.276	0.235	1	1.3
NBAT1	0.000391	-0.126436857	0.065	0.087	1	1.3
SCARA3	0.0004	-0.108549217	0.05	0.071	1	1.3
LINC00866	0.00041	-0.110907327	0.01	0.022	1	1.3
ATP13A3	0.000419	-0.262638333	0.33	0.348	1	1.3
HPCAL1	0.000423	-0.138609936	0.09	0.115	1	1.3
CCDC170	0.000426	-0.109057879	0.032	0.049	1	1.3
PAFAH1B1	0.000432	-0.189048712	0.355	0.374	1	1.3
LRP2	0.00044	-0.201826101	0.212	0.239	1	1.3
CSNK1A1	0.000442	-0.138288505	0.596	0.597	1	1.3
RPL8	0.000442	0.202792817	0.213	0.179	1	1.3
PAFAH1B2	0.000451	-0.188090707	0.215	0.24	1	1.3
STAT3	0.000452	-0.194670903	0.517	0.518	1	1.3
LRRC23	0.000453	-0.100630743	0.121	0.094	1	1.3
TNFRSF11A	0.000469	-0.15965486	0.113	0.139	1	1.3
IRF1	0.000469	-0.125461963	0.127	0.156	1	1.3
CHIC2	0.00047	-0.201186686	0.21	0.238	1	1.3
TMEM263	0.00048	-0.123773074	0.088	0.113	1	1.3
PLIN2	0.000481	-0.276981348	0.135	0.159	1	1.3
RP11-779O	0.000487	-0.186322951	0.193	0.222	1	1.3
TMEM41A	0.000487	-0.155632851	0.191	0.218	1	1.3
BBC3	0.000488	-0.124669217	0.053	0.073	1	1.3
FAM78B	0.000501	-0.108044901	0.058	0.079	1	1.3
TJP1	0.000506	-0.210802692	0.378	0.394	1	1.3
ARID4A	0.000511	-0.125659909	0.152	0.181	1	1.3
POLG2	0.000513	-0.173236686	0.061	0.082	1	1.3
GRIN2A	0.000519	-0.157787815	0.043	0.062	1	1.3
RPS5	0.00052	0.227366439	0.319	0.28	1	1.3
MYC	0.000532	-0.20208448	0.13	0.155	1	1.3
NDRG1	0.000541	-0.344740413	0.452	0.455	1	1.3
TOR1AIP2	0.000542	-0.226020533	0.223	0.245	1	1.3

LMAN1	0.000545	-0.146793089	0.182	0.21	1	1.3
COX7B	0.00056	-0.14460301	0.661	0.656	1	1.3
DSG3	0.000562	-0.487850247	0.055	0.075	1	1.3
RPL29	0.00058	0.223880885	0.329	0.289	1	1.3
S100A2	0.00058	-0.119793156	0.121	0.094	1	1.3
PTGS2	0.000586	-0.132835105	0.016	0.029	1	1.3
RAB21	0.000589	-0.1493345	0.176	0.204	1	1.3
FABP6	0.000612	-0.162802867	0.033	0.05	1	1.3
LAMB3	0.000625	-0.17951865	0.332	0.274	1	1.3
DECR1	0.000625	-0.121968778	0.125	0.152	1	1.3
KRAS	0.000633	-0.124581598	0.123	0.15	1	1.3
LINC00887	0.000693	-0.138281991	0.052	0.072	1	1.3
RP11-795H	0.000696	-0.205090059	0.425	0.446	1	1.3
GTF2I	0.000719	-0.22137809	0.427	0.437	1	1.3
SH3BGRL	0.000747	-0.17984933	0.222	0.248	1	1.3
KRT7	0.00075	-0.194490292	0.259	0.284	1	1.3
CA5B	0.000758	-0.174811178	0.116	0.14	1	1.3
CCDC59	0.000798	-0.185502008	0.163	0.187	1	1.3
TP53BP2	0.000803	-0.133845646	0.139	0.165	1	1.3
PCBP1	0.000836	-0.232910236	0.484	0.477	1	1.3
GSTA1	0.000853	-0.225704612	0.072	0.095	1	1.3
C12orf60	0.00086	-0.119371004	0.079	0.101	1	1.3
RAB9A	0.000883	-0.206953991	0.188	0.213	1	1.3
PEAK1	0.000886	-0.225214211	0.161	0.187	1	1.3
NOP58	0.000906	-0.157637896	0.196	0.224	1	1.3
PDK1	0.000911	-0.179748971	0.101	0.124	1	1.3
RPL28	0.000925	0.294961481	0.148	0.123	1	1.3
TPT1	0.000926	0.143402872	0.857	0.825	1	1.3
ETS2	0.000974	-0.108897646	0.107	0.132	1	1.3
SGCZ	0.000976	-0.176949014	0.069	0.091	1	1.3
LPP	0.000999	0.101671244	0.918	0.879	1	1.3
MREG	0.001007	-0.130480475	0.078	0.101	1	1.3
LAPTM4A	0.001032	-0.148566996	0.217	0.245	1	1.3
FEZ2	0.001047	-0.242945306	0.375	0.389	1	1.3
RAB14	0.001051	-0.13562987	0.104	0.128	1	1.3
FDCSP	0.00108	-0.138723052	0.231	0.196	1	1.3
ANXA1	0.001197	-0.203048369	0.932	0.897	1	1.3
UBD	0.001235	-0.353074176	0.148	0.176	1	1.3
ARHGEF2	0.001241	-0.101905968	0.067	0.087	1	1.3
OTUD4	0.001258	-0.11176478	0.113	0.137	1	1.3
RASGEF1C	0.001259	-0.143443911	0.06	0.079	1	1.3
HNRNPDL	0.001335	-0.155444634	0.287	0.307	1	1.3
CEP95	0.001394	-0.132886842	0.133	0.157	1	1.3
GSTO2	0.001394	-0.105245826	0.1	0.123	1	1.3
FAM49A	0.001413	-0.118744281	0.047	0.064	1	1.3
FOXJ3	0.001454	-0.164143174	0.276	0.3	1	1.3
RPL18	0.00149	0.145005917	0.139	0.114	1	1.3

MAP2	0.001499	-0.206920457	0.15	0.173	1	1.3
CUL3	0.001513	-0.187097097	0.333	0.351	1	1.3
PCGF5	0.001569	-0.161145954	0.163	0.187	1	1.3
RPS16	0.001594	0.159280385	0.388	0.347	1	1.3
BCOR	0.001597	-0.170876258	0.407	0.433	1	1.3
FBXO42	0.001653	-0.1646817	0.156	0.179	1	1.3
YWHAH	0.001712	-0.193695544	0.205	0.224	1	1.3
EIF4B	0.001726	-0.138506394	0.477	0.489	1	1.3
RPL36	0.001756	0.123238448	0.268	0.234	1	1.3
SNRPD2	0.00176	-0.145579059	0.758	0.741	1	1.3
CXCL3	0.001798	-0.216602806	0.077	0.097	1	1.3
MYSM1	0.001812	-0.123479418	0.112	0.135	1	1.3
RPS27	0.001824	-0.202541846	0.801	0.789	1	1.3
MAP4K5	0.001834	-0.213669072	0.355	0.368	1	1.3
RP11-840I1	0.001856	-0.118623071	0.077	0.098	1	1.3
ZNF706	0.001857	-0.118675391	0.105	0.128	1	1.3
GHR	0.001945	-0.212465196	0.121	0.144	1	1.3
CLINT1	0.001959	-0.195280473	0.361	0.371	1	1.3
LAMP2	0.001994	-0.191193547	0.238	0.258	1	1.3
PRKAG2	0.002031	-0.107738576	0.149	0.172	1	1.3
IFIH1	0.002043	-0.135538256	0.085	0.105	1	1.3
C6orf132	0.002195	-0.13561374	0.148	0.171	1	1.3
RAB11A	0.002197	-0.258601821	0.615	0.595	1	1.3
EIF3M	0.002314	-0.162798513	0.489	0.487	1	1.3
RAB3IP	0.002334	-0.127774736	0.104	0.124	1	1.3
GGA2	0.002347	-0.139308746	0.134	0.156	1	1.3
ATP2B4	0.002429	-0.270516895	0.259	0.278	1	1.3
UBQLN1	0.002441	-0.141544265	0.233	0.255	1	1.3
RPS13	0.002478	-0.136820229	0.854	0.847	1	1.3
RPLP1	0.002525	0.222051457	0.292	0.26	1	1.3
UBE2G1	0.00257	-0.160084958	0.17	0.193	1	1.3
PTRF	0.002752	-0.191123498	0.161	0.182	1	1.3
STX5	0.002791	-0.121185704	0.17	0.195	1	1.3
ROR1	0.002901	0.107307306	0.048	0.034	1	1.3
DCP1A	0.002918	-0.168691642	0.244	0.264	1	1.3
MPP5	0.002925	-0.170870397	0.227	0.248	1	1.3
CREBRF	0.002943	-0.130840922	0.201	0.225	1	1.3
SEC31A	0.002985	-0.159239926	0.448	0.454	1	1.3
MGAT5	0.003097	-0.177448446	0.296	0.315	1	1.3
GPR107	0.003107	-0.125344118	0.137	0.158	1	1.3
DAPK1	0.003179	-0.162532995	0.673	0.663	1	1.3
BFAR	0.003258	-0.133110635	0.179	0.202	1	1.3
ABTB2	0.003261	-0.232096574	0.225	0.246	1	1.3
ANKRD12	0.003339	-0.206672351	0.567	0.562	1	1.3
CALR	0.003387	-0.144482968	0.17	0.194	1	1.3
EIF3H	0.003734	-0.123997491	0.704	0.697	1	1.3
MPZL1	0.003735	-0.154698679	0.476	0.48	1	1.3

CA8	0.003818	-0.246795547	0.44	0.439	1	1.3
NACA2	0.003841	-0.153868211	0.644	0.634	1	1.3
SRSF3	0.003888	-0.183002367	0.605	0.598	1	1.3
OGT	0.003911	-0.18386641	0.362	0.371	1	1.3
CCDC50	0.004068	-0.148799551	0.141	0.161	1	1.3
ZNFX1	0.004138	-0.118367429	0.117	0.139	1	1.3
TNF	0.004222	-0.172387353	0.088	0.108	1	1.3
TBC1D22B	0.004331	-0.162247926	0.17	0.19	1	1.3
OAZ2	0.004398	-0.105537686	0.071	0.089	1	1.3
YWHAE	0.004401	-0.125080333	0.62	0.618	1	1.3
IRS2	0.004464	-0.112926122	0.347	0.366	1	1.3
RDX	0.004622	-0.156767378	0.2	0.221	1	1.3
NDUFS4	0.004767	-0.129257039	0.458	0.464	1	1.3
RPL9	0.004882	-0.103162553	0.67	0.672	1	1.3
PRKCA	0.004956	-0.154218609	0.077	0.095	1	1.3
NAA50	0.005102	-0.146578284	0.2	0.221	1	1.3
SERP1	0.005106	-0.122922515	0.259	0.279	1	1.3
ZFC3H1	0.005235	-0.135919929	0.183	0.207	1	1.3
NCOA2	0.005543	-0.188373599	0.504	0.496	1	1.3
SNRPF	0.005762	-0.153349738	0.235	0.254	1	1.3
GALNT3	0.006008	-0.210530832	0.2	0.217	1	1.3
TRA2B	0.006045	-0.1775377	0.185	0.203	1	1.3
HSPA9	0.006202	-0.163412925	0.197	0.216	1	1.3
KLF6	0.006332	-0.145940383	0.52	0.528	1	1.3
RPS28	0.00637	0.100756643	0.129	0.108	1	1.3
NT5C3A	0.006581	-0.163731468	0.176	0.197	1	1.3
ISG20	0.006672	-0.101878276	0.038	0.052	1	1.3
VAPA	0.006731	-0.13075311	0.486	0.479	1	1.3
WTAP	0.006745	-0.29553274	0.676	0.659	1	1.3
TNFAIP8	0.006828	-0.107147215	0.447	0.387	1	1.3
IDO1	0.006957	-0.146093343	0.029	0.042	1	1.3
PIGP	0.006978	-0.115604797	0.146	0.166	1	1.3
SERINC1	0.007154	-0.175890097	0.299	0.31	1	1.3
CD74	0.007607	-0.12779599	0.141	0.162	1	1.3
LDHB	0.00774	-0.125607864	0.762	0.745	1	1.3
FBL	0.007864	-0.102653288	0.2	0.222	1	1.3
CDK17	0.007936	-0.109762617	0.123	0.143	1	1.3
PRPSAP1	0.007957	-0.126020822	0.104	0.122	1	1.3
ZNF195	0.008057	-0.107008367	0.1	0.119	1	1.3
NUDT2	0.008353	-0.112072608	0.13	0.148	1	1.3
LMBRD1	0.008362	-0.148491376	0.225	0.243	1	1.3
CPAMD8	0.008393	-0.105587209	0.104	0.122	1	1.3
RAB7A	0.008706	-0.158225951	0.386	0.391	1	1.3
RNF10	0.008714	-0.122735747	0.156	0.174	1	1.3
TNIP1	0.008759	-0.125419063	0.163	0.183	1	1.3
WAPL	0.008841	-0.154082887	0.16	0.177	1	1.3
ATG14	0.008894	-0.105072363	0.087	0.104	1	1.3

SCYL2	0.009239	-0.166461269	0.244	0.26	1	1.3
GNA12	0.009482	-0.13712384	0.345	0.362	1	1.3
CTNNAL1	0.009563	-0.150336738	0.094	0.111	1	1.3
PPP1R14C	0.010813	-0.15218319	0.182	0.199	1	1.3
BRIX1	0.011089	-0.151870588	0.111	0.127	1	1.3
MBIP	0.011175	-0.129386489	0.15	0.167	1	1.3
MED13L	0.01214	-0.144043083	0.7	0.669	1	1.3
SORBS2	0.012148	-0.150348598	0.635	0.559	1	1.3
NBPF19	0.012421	-0.145489211	0.172	0.19	1	1.3
ATP11B	0.01274	-0.145661555	0.183	0.201	1	1.3
CLIP1	0.012978	-0.12378909	0.375	0.387	1	1.3
FGD4	0.013108	-0.144908928	0.255	0.27	1	1.3
RP11-574F:	0.013191	-0.103310635	0.084	0.1	1	1.3
WDR26	0.013211	-0.131286206	0.196	0.214	1	1.3
NR3C1	0.013294	-0.153471832	0.237	0.253	1	1.3
CCNY	0.013396	-0.357454439	0.254	0.263	1	1.3
RP1-78O14	0.013408	-0.173610186	0.058	0.071	1	1.3
CALCOCO2	0.013581	-0.138147841	0.319	0.333	1	1.3
CELF1	0.013893	-0.117010771	0.333	0.344	1	1.3
PELI1	0.013974	-0.193112652	0.459	0.453	1	1.3
UQCRC2	0.014582	-0.141927336	0.45	0.448	1	1.3
ADAM32	0.014628	-0.195232026	0.113	0.128	1	1.3
CSDE1	0.014685	-0.126352075	0.596	0.58	1	1.3
ELK3	0.014773	-0.147341843	0.171	0.187	1	1.3
KRT17	0.014898	-0.125505096	0.022	0.032	1	1.3
H3F3B	0.01491	0.122486778	0.426	0.388	1	1.3
LHFPL3	0.015272	-0.138087019	0.303	0.313	1	1.3
NUP88	0.015549	-0.141130247	0.181	0.198	1	1.3
CTC-425F1.	0.015588	-0.106285712	0.071	0.086	1	1.3
TSLP	0.015855	-0.105364309	0.065	0.079	1	1.3
SNX1	0.015923	-0.140881558	0.128	0.144	1	1.3
ZNF165	0.016409	-0.134473136	0.053	0.066	1	1.3
KIF1B	0.017468	-0.167036654	0.479	0.482	1	1.3
ZNF207	0.018597	-0.103342636	0.487	0.486	1	1.3
FABP3	0.018916	-0.146019155	0.01	0.017	1	1.3
TBC1D10A	0.018989	-0.310528518	0.105	0.12	1	1.3
SVIL	0.019001	-0.114413659	0.856	0.827	1	1.3
HERC3	0.019489	-0.167338596	0.143	0.16	1	1.3
JAK1	0.019535	-0.124685854	0.331	0.343	1	1.3
SDK2	0.019769	-0.176743895	0.059	0.073	1	1.3
COPB1	0.020164	-0.151275851	0.202	0.216	1	1.3
RNF19B	0.020848	-0.139120199	0.159	0.176	1	1.3
NONO	0.021408	-0.122474215	0.394	0.397	1	1.3
UBA2	0.021638	-0.116167149	0.129	0.146	1	1.3
CSNK2A1	0.021656	-0.118726661	0.189	0.205	1	1.3
2-Sep	0.021817	-0.122955712	0.285	0.298	1	1.3
RIC3	0.021944	-0.271781361	0.08	0.093	1	1.3

RAC1	0.02208	-0.12100184	0.494	0.486	1	1.3
ZMYM5	0.022774	-0.112914901	0.16	0.176	1	1.3
TGFBR3	0.022834	0.104801219	0.211	0.186	1	1.3
IST1	0.023595	-0.140682803	0.281	0.291	1	1.3
TJP2	0.023648	-0.166905712	0.417	0.416	1	1.3
FHIT	0.023689	-0.129327421	0.375	0.39	1	1.3
MARCO	0.023992	-0.114547265	0.088	0.103	1	1.3
MAFF	0.02401	-0.141134825	0.192	0.206	1	1.3
C8orf37-AS	0.024324	-0.132063168	0.258	0.272	1	1.3
AGO2	0.024899	-0.135111205	0.166	0.183	1	1.3
EPB41L4B	0.025293	-0.126163932	0.101	0.116	1	1.3
CD55	0.025342	-0.176956208	0.378	0.38	1	1.3
MARCKSL1	0.025559	-0.122452657	0.084	0.098	1	1.3
UBE2D3	0.025575	-0.117051125	0.567	0.561	1	1.3
OFD1	0.0256	-0.18541137	0.298	0.307	1	1.3
FAM53C	0.026074	-0.133056782	0.201	0.215	1	1.3
CCL28	0.026565	-0.157416315	0.708	0.706	1	1.3
ELF5	0.02678	-0.181336146	0.293	0.3	1	1.3
BTG3	0.027232	-0.121680939	0.183	0.198	1	1.3
AREG	0.02754	-0.127155756	0.088	0.102	1	1.3
KRT19	0.02789	-0.295903797	0.313	0.318	1	1.3
DDX24	0.028191	-0.153043534	0.644	0.62	1	1.3
ACSL3	0.02861	-0.173760373	0.253	0.264	1	1.3
NDRG2	0.028669	-0.161473342	0.246	0.259	1	1.3
NDUFB1	0.029423	-0.117506425	0.34	0.351	1	1.3
RNMT	0.030246	-0.172468565	0.372	0.373	1	1.3
ZNF83	0.031482	-0.1987394	0.357	0.359	1	1.3
STRAP	0.031653	-0.112978254	0.114	0.129	1	1.3
YES1	0.032343	-0.106976271	0.198	0.213	1	1.3
DAB2IP	0.032656	-0.103726119	0.103	0.118	1	1.3
FOSL1	0.032874	-0.119927669	0.089	0.103	1	1.3
PCNX	0.033281	-0.110848496	0.18	0.196	1	1.3
FAM46A	0.033457	-0.130171982	0.1	0.114	1	1.3
NCKAP1	0.033729	-0.131796406	0.41	0.409	1	1.3
MYO1E	0.03423	-0.188885585	0.498	0.484	1	1.3
SPATS2L	0.034596	-0.135870601	0.267	0.28	1	1.3
RPL10A	0.034775	-0.114051583	0.791	0.784	1	1.3
RALA	0.034792	-0.100970386	0.163	0.179	1	1.3
RP1-313I6.	0.036005	-0.111079277	0.055	0.067	1	1.3
DIP2B	0.03746	-0.164305739	0.319	0.325	1	1.3
WBP11	0.038432	-0.108292572	0.118	0.132	1	1.3
FOSB	0.040701	-0.145338092	0.421	0.427	1	1.3
NEK7	0.041657	-0.108126589	0.192	0.206	1	1.3
INTS6-AS1	0.041949	-0.100257035	0.161	0.174	1	1.3
SLC16A13	0.042505	-0.194855563	0.045	0.055	1	1.3
CRYAB	0.042627	-0.10440277	0.55	0.501	1	1.3
BNIP3L	0.042963	-0.116865466	0.298	0.311	1	1.3

SF3B1	0.04448	-0.125166753	0.355	0.359	1	1.3
UQCRH	0.044953	-0.104198806	0.868	0.849	1	1.3
MIER1	0.045266	-0.105065552	0.174	0.188	1	1.3
NSA2	0.047063	-0.125472842	0.42	0.419	1	1.3
RCC1	0.047696	-0.195976131	0.358	0.354	1	1.3
LIMS1	0.047918	-0.17006547	0.312	0.318	1	1.3
GALNT1	0.048387	-0.11087984	0.14	0.154	1	1.3
FBLIM1	0.048484	-0.125464349	0.137	0.149	1	1.3
CYTH3	0.048561	-0.127309911	0.077	0.088	1	1.3
HIST1H4C	0.048655	-0.15512297	0.218	0.229	1	1.3
ERGIC3	0.048732	-0.15310862	0.364	0.364	1	1.3
MCL1	0.0488	-0.11886068	0.268	0.279	1	1.3
CCNK	0.049332	-0.114312826	0.114	0.127	1	1.3
KDM7A	0.049577	-0.124600965	0.341	0.344	1	1.3
CAST	0.049892	-0.160026101	0.561	0.538	1	1.3
HADH	0.051891	-0.209834486	0.197	0.208	1	1.3
TNKS2	0.053875	-0.128805746	0.252	0.261	1	1.3
RBMS2	0.05448	-0.119680614	0.143	0.156	1	1.3
UGCG	0.055284	-0.176815364	0.325	0.33	1	1.3
TPTEP1	0.057066	-0.130791355	0.225	0.235	1	1.3
AMD1	0.058021	-0.184436106	0.236	0.244	1	1.3
GPBP1L1	0.058039	-0.137369156	0.274	0.281	1	1.3
SKP1	0.060748	-0.12119625	0.851	0.833	1	1.3
ZCCHC6	0.060859	-0.148841686	0.344	0.345	1	1.3
HMGXB4	0.061701	-0.11383772	0.245	0.257	1	1.3
CLMN	0.062346	-0.175927311	0.613	0.583	1	1.3
PPP1CB	0.066668	-0.131666	0.427	0.419	1	1.3
SAP18	0.06669	-0.120161486	0.619	0.598	1	1.3
PPFIA1	0.067928	-0.122039247	0.266	0.274	1	1.3
LRCH3	0.06804	-0.124425041	0.365	0.366	1	1.3
AKAP9	0.070473	-0.108570544	0.355	0.364	1	1.3
PTPN12	0.071363	-0.134275299	0.43	0.425	1	1.3
DOCK4	0.072922	-0.145482405	0.395	0.344	1	1.3
SUN1	0.074307	-0.101081035	0.101	0.111	1	1.3
SMURF2	0.075276	-0.112548625	0.362	0.366	1	1.3
ATF2	0.076388	-0.108155782	0.125	0.136	1	1.3
KEAP1	0.076776	-0.105291105	0.035	0.043	1	1.3
ZBTB10	0.07798	-0.103459951	0.16	0.172	1	1.3
KPNB1	0.07803	-0.160216089	0.455	0.444	1	1.3
ASTN2	0.079857	-0.11011105	0.213	0.224	1	1.3
BAZ1A	0.079973	-0.126803442	0.317	0.32	1	1.3
CDC27	0.08368	-0.17469077	0.236	0.242	1	1.3
FMNL2	0.084814	-0.146772194	0.451	0.438	1	1.3
SEPP1	0.085675	-0.16604654	0.362	0.363	1	1.3
RNF11	0.092001	-0.116018055	0.15	0.159	1	1.3
NKX3-1	0.095138	-0.107164434	0.081	0.092	1	1.3
WDR33	0.095208	-0.103305083	0.31	0.314	1	1.3

RUSC2	0.095387	-0.127449697	0.113	0.124	1	1.3
MON2	0.098904	-0.125521836	0.253	0.259	1	1.3
RP11-131L1	0.098911	-0.110359502	0.069	0.078	1	1.3
ELAVL1	0.099971	-0.153131273	0.146	0.158	1	1.3
CLU	0.102397	-0.149968053	0.101	0.113	1	1.3
SGMS1	0.103054	-0.156803896	0.271	0.275	1	1.3
CACUL1	0.103441	-0.126093434	0.404	0.4	1	1.3
LMTK2	0.104453	-0.14065558	0.15	0.158	1	1.3
ARHGEF12	0.10664	-0.134466603	0.438	0.431	1	1.3
UFM1	0.108839	-0.121680948	0.352	0.352	1	1.3
F3	0.109038	-0.112450905	0.057	0.066	1	1.3
B4GALT5	0.11179	-0.122963273	0.332	0.335	1	1.3
RAB3GAP1	0.112165	-0.118311663	0.293	0.299	1	1.3
TPK1	0.112921	-0.120020113	0.257	0.263	1	1.3
ITGA2	0.113621	-0.325467404	0.482	0.455	1	1.3
YWHAQ	0.114034	-0.166536841	0.378	0.374	1	1.3
DNAJB1	0.120134	-0.152220124	0.098	0.106	1	1.3
METAP1	0.132021	-0.101861083	0.085	0.093	1	1.3
NCOA7	0.132849	-0.15069037	0.702	0.664	1	1.3
BDNF-AS	0.133654	-0.148303336	0.7	0.668	1	1.3
MAP3K14	0.133709	-0.101690876	0.069	0.078	1	1.3
CTSV	0.137251	-0.267887329	0.241	0.213	1	1.3
LRCH1	0.141593	-0.10573635	0.352	0.353	1	1.3
AIMP1	0.142028	-0.113321913	0.425	0.42	1	1.3
MPZL3	0.142606	-0.125866368	0.305	0.307	1	1.3
RP11-519G	0.143167	-0.228845161	0.562	0.477	1	1.3
TXLNG	0.144294	-0.110279329	0.105	0.113	1	1.3
SRGAP1	0.149741	-0.146963514	0.321	0.322	1	1.3
CTTNBP2NI	0.15029	-0.132658583	0.283	0.283	1	1.3
GADD45B	0.155528	-0.109129814	0.048	0.056	1	1.3
DSC2	0.157854	-0.114489737	0.235	0.242	1	1.3
KMT2E	0.159727	-0.101955275	0.404	0.403	1	1.3
MAP3K1	0.161651	-0.12392311	0.207	0.214	1	1.3
ZHX2	0.166334	-0.105795917	0.35	0.349	1	1.3
MYLK	0.168474	0.107665999	0.022	0.018	1	1.3
SLU7	0.174717	-0.100889567	0.134	0.142	1	1.3
CHI3L1	0.179541	-0.174868577	0.213	0.197	1	1.3
FGF13	0.181308	-0.191397306	0.214	0.219	1	1.3
URB1	0.182776	-0.17412545	0.065	0.057	1	1.3
STOM	0.184159	-0.101670966	0.192	0.199	1	1.3
JARID2	0.188879	-0.123822514	0.253	0.256	1	1.3
EIF5	0.189011	-0.137865586	0.199	0.204	1	1.3
MAP4	0.190375	-0.107554436	0.437	0.43	1	1.3
MAP3K9	0.190507	-0.101953591	0.167	0.175	1	1.3
MTM1	0.19183	-0.178823831	0.195	0.197	1	1.3
GNAI3	0.193547	-0.104466299	0.155	0.161	1	1.3
NCOR1	0.194465	-0.105373043	0.365	0.362	1	1.3

AC074391.	0.200004	-0.152244731	0.05	0.057	1	1.3
TTC19	0.221313	-0.116133131	0.208	0.212	1	1.3
YWHAG	0.227006	-0.120721593	0.231	0.232	1	1.3
SPCS2	0.227312	-0.121710926	0.318	0.315	1	1.3
EEA1	0.229739	-0.102381853	0.159	0.165	1	1.3
PDZD2	0.232622	-0.103911475	0.555	0.544	1	1.3
FAM208B	0.237021	-0.107730021	0.186	0.19	1	1.3
GNAS	0.250778	-0.110993944	0.619	0.593	1	1.3
WASF2	0.26175	-0.102416279	0.214	0.216	1	1.3
CXCL2	0.275055	-0.165074992	0.274	0.252	1	1.3
PIGR	0.278624	-0.205394806	0.524	0.517	1	1.3
EIF3K	0.291552	-0.142112203	0.489	0.468	1	1.3
HP	0.293275	-0.131425917	0.026	0.03	1	1.3
ATP8B1	0.294776	-0.202357906	0.338	0.329	1	1.3
CFLAR	0.297669	-0.106830749	0.505	0.458	1	1.3
KIF13A	0.303716	-0.174522192	0.273	0.271	1	1.3
AC090498.	0.308283	-0.102843981	0.399	0.386	1	1.3
DPP6	0.311322	-0.10227422	0.16	0.164	1	1.3
DYNLL1	0.31997	-0.137884068	0.231	0.231	1	1.3
TPD52	0.320195	-0.112436195	0.167	0.171	1	1.3
ELF2	0.321147	-0.110603811	0.326	0.323	1	1.3
COPA	0.321307	-0.104543338	0.354	0.345	1	1.3
MUCL1	0.342775	-0.112208542	0.15	0.154	1	1.3
KAT6A	0.36768	-0.123601329	0.249	0.249	1	1.3
MLF1	0.377793	-0.101124482	0.227	0.229	1	1.3
RP11-659O	0.414641	-0.106411219	0.187	0.188	1	1.3
MET	0.41854	-0.178137481	0.365	0.349	1	1.3
P4HA1	0.420909	-0.185542745	0.274	0.269	1	1.3
DBI	0.424743	-0.11072512	0.74	0.679	1	1.3
SCGB2A2	0.452876	-0.364408525	0.199	0.187	1	1.3
SAA4	0.464946	-0.155873096	0.283	0.277	1	1.3
SMCHD1	0.470536	-0.10193438	0.295	0.288	1	1.3
SOS2	0.471498	-0.101970188	0.489	0.462	1	1.3
STK39	0.494813	-0.317533156	0.129	0.131	1	1.3
RASAL2	0.530015	-0.259003074	0.569	0.532	1	1.3
CXCL13	0.540926	-0.104075976	0.099	0.101	1	1.3
PRSS8	0.554475	-0.164229697	0.059	0.062	1	1.3
HMOX1	0.567962	-0.10664252	0.061	0.064	1	1.3
KCTD9	0.577169	-0.120373797	0.306	0.299	1	1.3
DHX15	0.580352	-0.115010062	0.214	0.208	1	1.3
CYCS	0.594623	-0.146418714	0.565	0.537	1	1.3
PDK3	0.606609	-0.12712807	0.295	0.277	1	1.3
DFNB59	0.618974	-0.145919859	0.019	0.02	1	1.3
SLC25A33	0.620469	-0.135755517	0.134	0.135	1	1.3
HSD17B6	0.670151	-0.11423179	0.034	0.036	1	1.3
HIF1A-AS2	0.675796	-0.100502068	0.149	0.142	1	1.3
LALBA	0.692904	-0.22877235	0.014	0.013	1	1.3

PTP4A1	0.780262	-0.140906752	0.218	0.208	1	1.3
LDHA	0.78405	-0.269790041	0.766	0.703	1	1.3
SNX9	0.805627	-0.116831093	0.472	0.443	1	1.3
KRT23	0.815313	-0.178467906	0.249	0.24	1	1.3
SH3RF1	0.837511	-0.102205949	0.248	0.239	1	1.3
COL6A2	0.849262	-0.136125738	0.145	0.14	1	1.3
PLAUR	0.856633	-0.166045286	0.19	0.183	1	1.3
EPB41L2	0.877673	-0.189438418	0.198	0.19	1	1.3
GEMIN5	0.882807	-0.106501394	0.025	0.024	1	1.3
RHOA	0.926974	-0.118931397	0.443	0.414	1	1.3
LRRFIP1	0.966236	-0.161086036	0.55	0.504	1	1.3
TMEM41B	0.973662	-0.124614428	0.205	0.195	1	1.3
TGFB2	0.975122	-0.137075598	0.251	0.241	1	1.3
DMD	0.980325	-0.139883771	0.494	0.463	1	1.3
PPP3CA	0.998419	-0.152103461	0.433	0.406	1	1.3
KYNU	0	2.205536398	0.777	0.24	0	1.4
LTF	0	1.592857415	0.932	0.572	0	1.4
RARRES1	0	1.344866824	0.716	0.289	0	1.4
ARHGAP26	0	1.283934291	0.982	0.782	0	1.4
DOCK4	0	1.495407426	0.699	0.237	0	1.4
SAA1	0	1.461089045	0.995	0.94	0	1.4
FRMD4A	0	1.403947291	0.796	0.366	0	1.4
FBLN5	0	1.528613996	0.664	0.219	0	1.4
RP11-519G	0	1.243928886	0.816	0.386	0	1.4
ANXA2	0	-1.427126542	0.851	0.944	0	1.4
PSTPIP2	0	1.189775516	0.67	0.247	0	1.4
EVA1C	0	1.402371579	0.802	0.324	0	1.4
ST5	7.67E-307	1.23631877	0.693	0.285	1.85E-302	1.4
TXNIP	2.20E-293	1.323714407	0.745	0.364	5.31E-289	1.4
MYL6	1.41E-285	-1.007230595	0.892	0.955	3.40E-281	1.4
CHI3L1	8.88E-284	1.796617321	0.453	0.112	2.14E-279	1.4
USP53	1.94E-279	-1.909307633	0.444	0.747	4.67E-275	1.4
ALPK1	6.28E-277	1.057591189	0.575	0.203	1.51E-272	1.4
OVOS2	1.93E-276	1.449916928	0.769	0.406	4.66E-272	1.4
VNN3	3.14E-276	1.281850715	0.539	0.176	7.58E-272	1.4
ANK3	6.93E-273	1.086414117	0.626	0.244	1.67E-268	1.4
TRIO	5.86E-272	-1.549327476	0.561	0.808	1.41E-267	1.4
CHODL	7.11E-271	1.202824057	0.446	0.115	1.71E-266	1.4
CHI3L2	2.51E-262	1.393928747	0.761	0.407	6.06E-258	1.4
SAA2-SAA4	9.54E-261	1.032678828	0.661	0.274	2.30E-256	1.4
LYN	5.34E-257	0.940933141	0.922	0.606	1.29E-252	1.4
DCHS2	4.85E-254	1.059778792	0.362	0.074	1.17E-249	1.4
MYL12A	5.40E-253	-1.162754829	0.775	0.888	1.30E-248	1.4
PLEKHS1	2.12E-249	1.015432652	0.521	0.17	5.11E-245	1.4
TRPS1	1.60E-243	1.016337323	0.909	0.651	3.86E-239	1.4
MGAM2	1.70E-236	1.139348159	0.603	0.245	4.10E-232	1.4
SLC34A2	3.64E-235	0.944767307	0.542	0.195	8.78E-231	1.4

TNFAIP2	2.56E-229	1.318299826	0.555	0.207	6.18E-225	1.4
CNTN4	1.30E-227	1.14523347	0.419	0.118	3.14E-223	1.4
RASGRP1	6.41E-220	0.835476927	0.33	0.071	1.55E-215	1.4
CCND3	1.64E-219	0.956961066	0.62	0.265	3.95E-215	1.4
CYP1B1	7.57E-219	0.909108641	0.308	0.061	1.83E-214	1.4
ANXA1	9.01E-217	-1.30147723	0.841	0.929	2.17E-212	1.4
LDHA	3.23E-216	-1.329112757	0.568	0.772	7.78E-212	1.4
AGAP1	2.55E-213	1.005961861	0.834	0.542	6.14E-209	1.4
SERPINB7	2.38E-212	1.243590792	0.41	0.121	5.75E-208	1.4
ITGA2	7.14E-208	-1.631295003	0.218	0.548	1.72E-203	1.4
CYP7B1	1.10E-206	1.046731403	0.636	0.284	2.64E-202	1.4
3-Mar	9.13E-202	1.103563983	0.424	0.134	2.20E-197	1.4
C15orf48	1.67E-198	1.267512275	0.722	0.404	4.03E-194	1.4
NR2F2-AS1	9.53E-194	0.820775618	0.322	0.077	2.30E-189	1.4
ITPR2	6.99E-187	0.935433084	0.774	0.463	1.68E-182	1.4
OSER1	1.01E-185	-1.642478526	0.365	0.615	2.43E-181	1.4
CCL2	1.05E-183	0.907035226	0.676	0.346	2.54E-179	1.4
FOXP1	2.49E-183	0.83340617	0.786	0.468	6.00E-179	1.4
THADA	4.11E-178	1.049638232	0.493	0.196	9.90E-174	1.4
PIK3R1	1.24E-177	0.911320564	0.486	0.189	2.98E-173	1.4
RASA2	7.49E-177	-1.274193399	0.457	0.693	1.81E-172	1.4
CHPT1	1.91E-173	1.045480389	0.614	0.307	4.60E-169	1.4
SOD2	7.89E-173	0.568047328	0.997	0.927	1.90E-168	1.4
ZNF518A	2.47E-172	0.755402468	0.377	0.12	5.96E-168	1.4
PADI2	3.06E-171	0.775390635	0.368	0.113	7.38E-167	1.4
CFB	3.02E-169	0.718002257	0.303	0.077	7.29E-165	1.4
FBN1	5.27E-169	0.579653982	0.156	0.012	1.27E-164	1.4
TM4SF1	3.08E-165	-1.340895275	0.85	0.914	7.44E-161	1.4
UBC	1.17E-164	-1.392130768	0.919	0.96	2.81E-160	1.4
KRT15	1.78E-161	-1.483842828	0.124	0.418	4.29E-157	1.4
PBX1	7.93E-161	0.845382597	0.418	0.155	1.91E-156	1.4
SLFN5	1.48E-160	0.686759334	0.56	0.252	3.56E-156	1.4
PDZRN3	3.81E-160	0.803377768	0.37	0.121	9.20E-156	1.4
SORBS2	1.09E-158	0.979730565	0.78	0.507	2.64E-154	1.4
TNFAIP6	1.74E-157	0.932259801	0.654	0.336	4.18E-153	1.4
MAST4	3.06E-157	1.006728959	0.595	0.308	7.38E-153	1.4
FABP7	4.74E-156	2.096895793	0.4	0.159	1.14E-151	1.4
CA8	7.11E-155	0.800825935	0.665	0.36	1.71E-150	1.4
SCARA3	3.94E-154	0.596998726	0.181	0.025	9.50E-150	1.4
CLU	7.10E-154	1.079467796	0.256	0.059	1.71E-149	1.4
ANKS1B	1.99E-153	0.798850521	0.479	0.199	4.79E-149	1.4
MYO5B	3.25E-151	-1.353146214	0.433	0.656	7.84E-147	1.4
MLLT4	1.62E-149	-1.100780721	0.52	0.712	3.90E-145	1.4
SLC25A37	1.06E-148	0.663869602	0.892	0.638	2.57E-144	1.4
C4orf19	1.78E-148	0.779771402	0.365	0.126	4.28E-144	1.4
HSD11B1	9.21E-147	0.811061142	0.377	0.133	2.22E-142	1.4
RP11-739G	1.14E-146	0.700411086	0.131	0.009	2.76E-142	1.4

DDX21	4.33E-146	-1.038512022	0.49	0.674	1.05E-141	1.4
RP11-114H	1.57E-143	1.027597336	0.357	0.125	3.78E-139	1.4
LRIG1	5.96E-143	0.828406575	0.438	0.178	1.44E-138	1.4
DPYD	3.92E-142	0.679368822	0.755	0.453	9.45E-138	1.4
SORBS1	8.35E-141	0.71207925	0.555	0.27	2.01E-136	1.4
CADPS2	1.27E-139	0.71538376	0.517	0.238	3.06E-135	1.4
C3	1.32E-139	0.715057473	0.508	0.232	3.19E-135	1.4
PTPN2	3.14E-138	0.715132056	0.578	0.301	7.57E-134	1.4
SELK	4.32E-138	-0.999683871	0.551	0.718	1.04E-133	1.4
RIN2	2.62E-137	0.725522443	0.514	0.24	6.31E-133	1.4
PODXL	4.08E-137	0.67004918	0.36	0.128	9.85E-133	1.4
C10orf90	1.22E-136	0.615399565	0.25	0.063	2.94E-132	1.4
SLC39A8	1.74E-134	0.81244753	0.302	0.095	4.19E-130	1.4
SIK2	3.67E-134	0.755804819	0.526	0.256	8.84E-130	1.4
JARID2	7.66E-134	0.799444146	0.44	0.19	1.85E-129	1.4
RAPGEF5	2.15E-133	0.718087245	0.694	0.407	5.18E-129	1.4
FMN1	2.33E-133	0.708524212	0.331	0.111	5.62E-129	1.4
SKP1	1.13E-132	-0.749969605	0.766	0.863	2.74E-128	1.4
EMP1	1.25E-132	-1.309343644	0.62	0.754	3.02E-128	1.4
PLPP3	1.52E-132	0.815886702	0.404	0.163	3.68E-128	1.4
MARCO	8.11E-132	0.629233114	0.227	0.054	1.95E-127	1.4
SMARCA2	2.13E-131	0.690689632	0.495	0.233	5.13E-127	1.4
FDCSP	3.87E-130	2.56175661	0.364	0.149	9.33E-126	1.4
GLIPR1	1.42E-129	-1.212591426	0.16	0.412	3.42E-125	1.4
FAAH2	5.10E-129	0.665447976	0.385	0.15	1.23E-124	1.4
ACTB	3.51E-128	-1.061329435	0.475	0.665	8.46E-124	1.4
FTX	4.08E-128	0.698161373	0.755	0.468	9.83E-124	1.4
PLXDC2	7.22E-128	0.682492661	0.42	0.175	1.74E-123	1.4
MYL12B	4.01E-127	-0.885450703	0.614	0.75	9.66E-123	1.4
S100A8	1.22E-126	2.060650802	0.381	0.158	2.95E-122	1.4
RP11-519G	1.64E-126	0.722076503	0.214	0.05	3.95E-122	1.4
SLC26A2	9.77E-126	0.767763552	0.313	0.106	2.36E-121	1.4
ZBTB20	4.06E-125	0.683274831	0.691	0.408	9.79E-121	1.4
ITFG1	4.22E-125	0.65489094	0.426	0.181	1.02E-120	1.4
ZNF521	1.30E-124	0.726441773	0.345	0.128	3.13E-120	1.4
C1QTNF3-A	3.29E-124	0.653236698	0.391	0.156	7.93E-120	1.4
GCNT2	7.38E-124	0.745029061	0.56	0.295	1.78E-119	1.4
ESR1	4.04E-122	0.63910071	0.277	0.086	9.75E-118	1.4
NOS1AP	1.35E-121	0.782196426	0.31	0.107	3.25E-117	1.4
OSMR	2.35E-121	0.685241537	0.574	0.304	5.67E-117	1.4
HSP90AB1	4.28E-121	-0.58566899	0.912	0.946	1.03E-116	1.4
RP11-266O	1.64E-120	0.648703098	0.14	0.019	3.94E-116	1.4
LBP	2.08E-120	0.53896989	0.11	0.008	5.01E-116	1.4
AUTS2	4.34E-120	0.641317485	0.757	0.495	1.05E-115	1.4
TLR2	2.33E-119	0.638948194	0.436	0.194	5.62E-115	1.4
TNFSF10	5.64E-119	0.675101456	0.686	0.424	1.36E-114	1.4
ADAM9	1.96E-118	-0.982434767	0.43	0.631	4.72E-114	1.4

TMSB4X	1.45E-117	-0.866554854	0.83	0.909	3.50E-113	1.4
HSPA5	2.72E-116	-1.688263564	0.23	0.454	6.57E-112	1.4
KIT	1.15E-114	0.646473011	0.565	0.297	2.78E-110	1.4
N4BP2L2	1.19E-114	0.545888163	0.883	0.667	2.87E-110	1.4
RORA	3.65E-114	0.706667708	0.668	0.421	8.79E-110	1.4
TBC1D5	1.35E-113	0.647060057	0.547	0.288	3.26E-109	1.4
OSMR-AS1	4.27E-113	0.604739725	0.356	0.141	1.03E-108	1.4
TNIP3	9.50E-113	0.481925146	0.185	0.041	2.29E-108	1.4
CLEC7A	2.58E-110	0.558522709	0.258	0.082	6.23E-106	1.4
CTD-2015G	5.21E-110	0.57802008	0.332	0.127	1.26E-105	1.4
RP11-449D	6.29E-108	0.599225868	0.198	0.049	1.52E-103	1.4
CACNB2	1.93E-107	0.602938631	0.28	0.095	4.66E-103	1.4
COL27A1	7.96E-107	0.647449616	0.214	0.058	1.92E-102	1.4
FHIT	2.24E-106	0.729788336	0.57	0.322	5.40E-102	1.4
C1S	2.53E-106	0.465654162	0.24	0.072	6.10E-102	1.4
LINC01344	2.04E-104	0.442643159	0.119	0.015	4.91E-100	1.4
TPST1	2.14E-104	0.634399034	0.282	0.098	5.16E-100	1.4
LRRFIP2	2.34E-104	-1.068995989	0.767	0.83	5.65E-100	1.4
GLIPR2	1.16E-103	0.548689946	0.253	0.083	2.80E-99	1.4
MAP3K5	5.81E-103	0.631005058	0.502	0.258	1.40E-98	1.4
THRB	1.25E-101	0.608114251	0.473	0.235	3.02E-97	1.4
CDC42	1.92E-101	-0.735265224	0.612	0.733	4.62E-97	1.4
GLUL	1.93E-101	0.787461681	0.494	0.265	4.65E-97	1.4
TRABD2B	2.48E-101	0.606323492	0.22	0.064	5.97E-97	1.4
PPP2R3A	2.46E-99	0.606734183	0.593	0.345	5.94E-95	1.4
UST	2.23E-98	0.66245029	0.409	0.19	5.38E-94	1.4
CNN3	4.34E-98	-0.876943508	0.312	0.515	1.05E-93	1.4
PARK2	1.90E-97	0.575271753	0.305	0.117	4.58E-93	1.4
LRRFIP1	2.29E-97	-0.851703851	0.381	0.563	5.52E-93	1.4
CYCS	3.00E-97	-0.886799134	0.407	0.592	7.22E-93	1.4
PPP1R9A	9.32E-97	0.59711336	0.355	0.153	2.25E-92	1.4
SPIDR	5.46E-96	0.579679874	0.769	0.529	1.32E-91	1.4
GHR	6.47E-96	0.658464803	0.264	0.093	1.56E-91	1.4
RGL1	8.73E-95	0.468252491	0.198	0.055	2.11E-90	1.4
PROM1	1.52E-94	0.608393092	0.64	0.392	3.67E-90	1.4
IFITM3	1.81E-94	0.65695729	0.408	0.2	4.37E-90	1.4
DENND2D	2.19E-94	0.465997416	0.259	0.091	5.28E-90	1.4
SERPINB4	2.24E-94	1.419171832	0.202	0.059	5.40E-90	1.4
CYR61	4.72E-94	-1.804936101	0.135	0.338	1.14E-89	1.4
PLA2R1	4.73E-94	0.621531037	0.393	0.184	1.14E-89	1.4
AC016995.	5.90E-94	0.685799268	0.339	0.148	1.42E-89	1.4
RP11-142C	2.96E-93	0.535513762	0.239	0.08	7.13E-89	1.4
LIPH	1.63E-92	-1.207928839	0.32	0.522	3.94E-88	1.4
KIAA1217	3.12E-92	0.498824678	0.932	0.784	7.51E-88	1.4
SLC5A6	5.30E-92	0.491999625	0.255	0.09	1.28E-87	1.4
HSP90B1	6.92E-92	-1.006908695	0.33	0.517	1.67E-87	1.4
HSP90AA1	7.98E-92	-0.687717744	0.901	0.922	1.92E-87	1.4

CALD1	2.00E-91	-1.046620739	0.474	0.642	4.82E-87	1.4
PPM1H	3.07E-91	0.623011877	0.555	0.318	7.41E-87	1.4
HIVEP3	3.23E-91	0.73338925	0.484	0.26	7.78E-87	1.4
HS3ST4	4.28E-91	0.611879276	0.516	0.286	1.03E-86	1.4
FBXL17	1.10E-90	0.592333055	0.327	0.137	2.66E-86	1.4
RAD51B	2.29E-90	0.585556293	0.373	0.172	5.53E-86	1.4
HIST1H2AC	6.90E-90	0.649973128	0.483	0.261	1.66E-85	1.4
SAMD12	1.04E-89	0.607835067	0.421	0.207	2.52E-85	1.4
CLDN1	1.79E-89	-1.246775364	0.268	0.464	4.32E-85	1.4
UBD	3.42E-88	1.011053808	0.301	0.123	8.24E-84	1.4
SERPINA3	4.69E-88	0.733551482	0.365	0.166	1.13E-83	1.4
GBE1	1.72E-87	-1.087608225	0.248	0.451	4.15E-83	1.4
HMGN3	1.27E-86	0.481091069	0.299	0.122	3.06E-82	1.4
TSC22D2	2.56E-86	-1.057367386	0.179	0.375	6.17E-82	1.4
SLC11A2	5.08E-86	0.517876414	0.607	0.367	1.22E-81	1.4
NALCN	2.64E-85	0.512235138	0.279	0.11	6.37E-81	1.4
MAP1B	3.58E-85	-0.869763395	0.259	0.461	8.63E-81	1.4
EIF2AK3	1.04E-84	-1.120876932	0.157	0.35	2.50E-80	1.4
LINC01138	1.64E-84	0.526834186	0.376	0.179	3.96E-80	1.4
KREMEN1	2.58E-84	0.402982285	0.164	0.042	6.22E-80	1.4
RPL24	4.06E-84	-0.42507193	0.936	0.96	9.80E-80	1.4
CORO1C	5.79E-84	-0.788855897	0.097	0.285	1.40E-79	1.4
MEIS2	1.84E-83	0.563582149	0.405	0.203	4.44E-79	1.4
ABCC4	2.23E-83	0.396583195	0.1	0.014	5.37E-79	1.4
ARID1B	2.77E-83	0.528543671	0.575	0.349	6.69E-79	1.4
SLC28A3	3.33E-83	0.49979719	0.536	0.299	8.03E-79	1.4
LDLRAD4	4.30E-83	0.565029271	0.304	0.129	1.04E-78	1.4
LINC00152	8.31E-83	-0.959102302	0.204	0.393	2.00E-78	1.4
RELB	1.58E-82	0.48706496	0.272	0.107	3.80E-78	1.4
SMS	7.39E-82	-0.985945056	0.252	0.439	1.78E-77	1.4
SMARCD3	2.72E-81	0.32576209	0.099	0.014	6.55E-77	1.4
FAM20A	2.83E-80	0.403462338	0.156	0.04	6.83E-76	1.4
EXOC4	4.86E-80	0.54211448	0.448	0.24	1.17E-75	1.4
GMDS-AS1	8.52E-80	0.581798458	0.224	0.079	2.05E-75	1.4
CCDC170	8.92E-80	0.358847176	0.113	0.02	2.15E-75	1.4
TMTC2	1.24E-79	0.522706784	0.47	0.255	2.99E-75	1.4
CP	1.46E-79	0.460164489	0.153	0.039	3.53E-75	1.4
CCSER1	7.83E-79	0.54820662	0.685	0.471	1.89E-74	1.4
NCOA7	7.89E-79	0.515878293	0.813	0.624	1.90E-74	1.4
HSD17B4	1.97E-78	0.468054123	0.284	0.118	4.75E-74	1.4
GAB1	3.02E-78	0.665384326	0.515	0.308	7.29E-74	1.4
CRYAB	5.72E-78	-1.040071206	0.391	0.556	1.38E-73	1.4
OSBPL9	1.27E-77	-0.981591345	0.342	0.505	3.07E-73	1.4
PNISR	2.36E-77	0.462098204	0.879	0.725	5.68E-73	1.4
CDH1	2.76E-77	-0.769842975	0.391	0.561	6.65E-73	1.4
OSBPL1A	2.81E-77	0.453411406	0.266	0.107	6.77E-73	1.4
ENOSF1	2.93E-77	0.522042275	0.358	0.173	7.06E-73	1.4

BRE	5.21E-77	0.525757522	0.297	0.13	1.26E-72	1.4
C1RL	6.21E-77	0.476757602	0.229	0.084	1.50E-72	1.4
NCOA1	6.22E-77	0.571271598	0.561	0.347	1.50E-72	1.4
MTHFD2L	6.75E-77	0.430513775	0.773	0.56	1.63E-72	1.4
MBD5	8.90E-77	0.506051185	0.414	0.215	2.15E-72	1.4
C1R	1.76E-76	0.403903916	0.162	0.046	4.25E-72	1.4
GNE	4.48E-76	0.491309119	0.303	0.133	1.08E-71	1.4
SERPINB3	7.02E-76	1.164295692	0.174	0.053	1.69E-71	1.4
MOB3B	5.04E-75	0.456156348	0.386	0.194	1.21E-70	1.4
RTN4	2.46E-74	-0.683956824	0.532	0.661	5.93E-70	1.4
GXYLT2	3.19E-74	0.397906702	0.177	0.055	7.68E-70	1.4
PSME4	3.46E-74	-0.830748176	0.635	0.724	8.34E-70	1.4
PDZK1IP1	3.80E-74	0.603098643	0.294	0.129	9.17E-70	1.4
DLEU2	1.05E-73	0.47300547	0.315	0.145	2.53E-69	1.4
ARHGAP26	1.32E-73	0.565797916	0.371	0.184	3.19E-69	1.4
CSF3R	1.59E-73	0.264817273	0.094	0.014	3.84E-69	1.4
SYNM	2.10E-73	0.587194632	0.275	0.117	5.07E-69	1.4
GANC	8.08E-73	0.402588408	0.202	0.07	1.95E-68	1.4
PGK1	4.27E-72	-0.686293986	0.556	0.686	1.03E-67	1.4
SPG11	4.99E-72	0.49172189	0.433	0.234	1.20E-67	1.4
WWOX	9.92E-72	0.763014785	0.352	0.179	2.39E-67	1.4
SLC30A4	2.00E-71	0.42820092	0.237	0.092	4.83E-67	1.4
RUNX1	2.32E-71	0.555422917	0.718	0.509	5.59E-67	1.4
SGCZ	2.37E-71	0.612846557	0.173	0.054	5.71E-67	1.4
ENPP6	4.21E-71	0.349308687	0.12	0.027	1.02E-66	1.4
MYO6	7.16E-71	-0.807218507	0.581	0.67	1.73E-66	1.4
UCK2	8.85E-71	0.531369889	0.288	0.129	2.13E-66	1.4
JPX	1.71E-70	0.501294299	0.395	0.211	4.12E-66	1.4
GPHN	3.65E-70	0.433090672	0.442	0.24	8.80E-66	1.4
CLLU1OS	7.60E-70	0.360191621	0.18	0.059	1.83E-65	1.4
RP11-290O	2.81E-69	0.507290763	0.188	0.063	6.78E-65	1.4
PRELID1	2.89E-69	0.402523588	0.25	0.103	6.97E-65	1.4
TMEM165	5.03E-69	0.4685835	0.633	0.421	1.21E-64	1.4
RNF213	7.40E-69	0.416913974	0.302	0.138	1.78E-64	1.4
AKR1C3	1.23E-68	0.426305647	0.317	0.147	2.97E-64	1.4
RPS6KA5	1.43E-68	0.496862552	0.335	0.165	3.45E-64	1.4
RPL36AL	2.41E-68	-0.411755318	0.9	0.919	5.82E-64	1.4
AL109761.!	2.66E-68	0.278058571	0.105	0.021	6.43E-64	1.4
CYP1B1-AS	3.17E-68	0.288670647	0.098	0.018	7.63E-64	1.4
ZNF33A	4.17E-68	0.422454105	0.385	0.201	1.01E-63	1.4
ILF2	6.08E-68	-0.696189738	0.464	0.596	1.47E-63	1.4
ZNHIT6	6.59E-68	0.486566039	0.31	0.147	1.59E-63	1.4
TGM2	8.18E-68	0.330966884	0.179	0.059	1.97E-63	1.4
NEBL	2.81E-67	0.466508696	0.654	0.449	6.77E-63	1.4
GUCY1A3	3.18E-67	0.539762167	0.358	0.185	7.66E-63	1.4
UTRN	7.57E-67	0.531902598	0.342	0.171	1.83E-62	1.4
ZFAS1	7.65E-67	-0.388439071	0.952	0.966	1.85E-62	1.4

C11orf80	8.63E-67	0.447200012	0.347	0.173	2.08E-62	1.4
EFNA5	8.74E-67	0.506842633	0.653	0.442	2.11E-62	1.4
HP	1.01E-66	0.83810209	0.08	0.011	2.44E-62	1.4
SEMA6A	1.17E-66	0.525918941	0.565	0.361	2.81E-62	1.4
IMMP2L	1.24E-66	0.556841993	0.457	0.267	2.99E-62	1.4
TIAM1	1.99E-66	-0.879998542	0.182	0.356	4.80E-62	1.4
ELF5	2.39E-66	0.473756205	0.439	0.249	5.77E-62	1.4
SLC24A3	2.55E-66	0.444120545	0.274	0.122	6.15E-62	1.4
DHCR24	3.99E-66	0.417999371	0.283	0.129	9.62E-62	1.4
RP11-475O	4.35E-66	0.380666994	0.12	0.028	1.05E-61	1.4
DTNB	5.28E-66	0.453695045	0.731	0.525	1.27E-61	1.4
BMPR1A	5.66E-66	0.453238598	0.426	0.235	1.37E-61	1.4
UBE2H	7.68E-66	-0.608905159	0.658	0.738	1.85E-61	1.4
IDH2	1.93E-65	0.360614848	0.173	0.057	4.65E-61	1.4
FOXN3	3.80E-65	0.4873066	0.361	0.188	9.16E-61	1.4
C10orf10	4.13E-65	0.490626274	0.317	0.154	9.97E-61	1.4
ST6GAL1	1.49E-64	0.502974805	0.481	0.286	3.60E-60	1.4
RBM47	1.88E-64	0.445846111	0.732	0.54	4.52E-60	1.4
DEFB1	2.43E-64	0.261634825	0.767	0.538	5.87E-60	1.4
RP11-141O	2.99E-64	0.299809368	0.092	0.017	7.20E-60	1.4
SPX	3.25E-64	0.311680792	0.107	0.023	7.82E-60	1.4
ABCA5	5.88E-64	0.372898892	0.218	0.086	1.42E-59	1.4
CRIM1	6.17E-64	-0.696639534	0.385	0.544	1.49E-59	1.4
PIWIL4	1.26E-63	0.322386571	0.135	0.037	3.03E-59	1.4
DISC1	1.55E-63	0.387544847	0.192	0.07	3.74E-59	1.4
FCHSD2	1.70E-63	0.401324263	0.199	0.074	4.09E-59	1.4
MIR646HG	2.03E-63	0.417806403	0.183	0.065	4.88E-59	1.4
C4orf3	3.66E-63	-0.792590179	0.56	0.649	8.83E-59	1.4
SLC20A2	5.80E-63	-0.790276424	0.266	0.428	1.40E-58	1.4
TRIM38	7.02E-63	0.378283843	0.195	0.073	1.69E-58	1.4
ARF4	7.72E-63	-0.589081708	0.572	0.681	1.86E-58	1.4
LINC01184	1.35E-62	0.415892987	0.346	0.175	3.25E-58	1.4
ATF7IP	1.59E-62	0.459863601	0.287	0.134	3.83E-58	1.4
BOC	1.73E-62	0.300224682	0.096	0.019	4.16E-58	1.4
MT-ND3	3.95E-62	0.319960064	0.986	0.983	9.54E-58	1.4
RASAL2	5.55E-62	-0.834623201	0.442	0.577	1.34E-57	1.4
TSC22D1	6.04E-62	0.561053743	0.382	0.212	1.46E-57	1.4
C21orf91-C	6.57E-62	0.283668033	0.097	0.019	1.58E-57	1.4
EYA2	9.28E-62	0.468452638	0.324	0.162	2.24E-57	1.4
NSD1	1.24E-61	0.413490852	0.251	0.111	3.00E-57	1.4
ITGB1	1.84E-61	-0.749772243	0.238	0.399	4.43E-57	1.4
HGSNAT	2.04E-61	0.435086939	0.277	0.129	4.93E-57	1.4
PDXK	2.15E-61	0.416469601	0.269	0.123	5.18E-57	1.4
CCL4	2.38E-61	1.890387692	0.168	0.059	5.74E-57	1.4
NPM1	3.06E-61	-0.426516858	0.893	0.918	7.38E-57	1.4
SFRP1	3.96E-61	0.469752456	0.609	0.408	9.55E-57	1.4
R3HDM2	6.00E-61	0.427988118	0.258	0.116	1.45E-56	1.4

SCGB2B2	6.76E-61	0.280815165	0.129	0.035	1.63E-56	1.4
LINC01122	7.87E-61	0.413163678	0.138	0.04	1.90E-56	1.4
MAML3	8.94E-61	0.454458709	0.369	0.197	2.16E-56	1.4
ST8SIA1	1.48E-60	0.406950169	0.294	0.142	3.57E-56	1.4
PKP4	1.79E-60	0.479615053	0.583	0.388	4.31E-56	1.4
WFDC2	3.48E-60	0.317261944	0.662	0.442	8.39E-56	1.4
LRP1B	4.55E-60	0.611306395	0.321	0.164	1.10E-55	1.4
NPAS3	7.00E-60	0.493693085	0.268	0.125	1.69E-55	1.4
CX3CL1	1.56E-59	0.513898604	0.394	0.221	3.76E-55	1.4
FKBP5	4.23E-59	0.53675326	0.413	0.236	1.02E-54	1.4
TRIM56	4.39E-59	0.434835378	0.32	0.162	1.06E-54	1.4
ARHGAP29	4.64E-59	-1.033296066	0.379	0.519	1.12E-54	1.4
LRBA	5.78E-59	0.402396904	0.619	0.406	1.39E-54	1.4
TMCO4	7.85E-59	0.338501602	0.184	0.068	1.89E-54	1.4
BTG1	8.44E-59	0.505740863	0.606	0.42	2.03E-54	1.4
HNRNPA1	1.08E-58	-0.501742559	0.879	0.9	2.60E-54	1.4
MAML2	1.12E-58	0.414871387	0.889	0.742	2.70E-54	1.4
CYP27A1	2.05E-58	0.333914467	0.162	0.055	4.93E-54	1.4
PDCD4	2.25E-58	0.507636876	0.341	0.183	5.42E-54	1.4
MT-ATP6	2.42E-58	0.291573284	0.987	0.987	5.84E-54	1.4
VNN1	2.57E-58	0.395477411	0.389	0.212	6.20E-54	1.4
MICAL2	2.79E-58	0.354828128	0.135	0.04	6.73E-54	1.4
MORF4L2	3.50E-58	-0.505428593	0.698	0.763	8.43E-54	1.4
HRSP12	4.91E-58	0.340654159	0.169	0.06	1.18E-53	1.4
IFI16	6.39E-58	0.445120755	0.404	0.231	1.54E-53	1.4
NNMT	7.17E-58	0.414178313	0.162	0.055	1.73E-53	1.4
ATL2	9.92E-58	0.451393558	0.414	0.238	2.39E-53	1.4
HSD17B2	1.30E-57	0.31548656	0.137	0.041	3.13E-53	1.4
INTS10	1.55E-57	0.343800075	0.201	0.08	3.74E-53	1.4
RP11-536O	2.21E-57	0.405522564	0.21	0.086	5.32E-53	1.4
ARHGAP44	2.27E-57	0.383960867	0.369	0.198	5.48E-53	1.4
PCCA	3.69E-57	0.395342072	0.264	0.123	8.89E-53	1.4
PRKCE	4.11E-57	0.494202942	0.32	0.165	9.91E-53	1.4
UVRAG	4.66E-57	0.430408531	0.563	0.365	1.12E-52	1.4
NHS	5.87E-57	0.416767081	0.302	0.15	1.42E-52	1.4
YWHAZ	2.60E-56	-0.567682352	0.514	0.615	6.27E-52	1.4
RSRP1	3.22E-56	0.416067618	0.374	0.206	7.75E-52	1.4
VPS13D	3.22E-56	0.386911537	0.463	0.277	7.76E-52	1.4
KRIT1	3.71E-56	0.334198492	0.214	0.09	8.96E-52	1.4
JAK2	4.62E-56	0.490563495	0.159	0.056	1.11E-51	1.4
CDKAL1	5.11E-56	0.433928021	0.368	0.202	1.23E-51	1.4
IRF2BPL	7.50E-56	0.35947454	0.222	0.095	1.81E-51	1.4
ASS1	8.02E-56	0.387487333	0.262	0.124	1.93E-51	1.4
HSD17B7	8.40E-56	0.384393902	0.205	0.084	2.03E-51	1.4
KIAA0319L	9.44E-56	0.384514289	0.269	0.127	2.28E-51	1.4
PTPRG	1.30E-55	0.429479118	0.35	0.186	3.12E-51	1.4
SKAP2	1.78E-55	0.391322003	0.459	0.276	4.30E-51	1.4

NEAT1	2.22E-55	0.299841435	0.994	0.954	5.36E-51	1.4
PHLDA1	4.13E-55	0.41089177	0.277	0.135	9.97E-51	1.4
MGC32805	7.41E-55	0.19434683	0.063	0.008	1.79E-50	1.4
DAPK2	8.49E-55	-0.699827352	0.337	0.484	2.05E-50	1.4
PTPRK	1.08E-54	0.383028576	0.839	0.673	2.60E-50	1.4
EDN1	1.19E-54	-0.703420742	0.105	0.25	2.88E-50	1.4
LINC01482	1.42E-54	0.280457098	0.076	0.013	3.42E-50	1.4
ATR	1.55E-54	0.395431235	0.272	0.132	3.74E-50	1.4
IDO1	1.97E-54	0.565392067	0.091	0.02	4.74E-50	1.4
BPTF	2.41E-54	0.424554573	0.465	0.289	5.82E-50	1.4
BBOX1	3.84E-54	0.514424275	0.389	0.221	9.25E-50	1.4
NPEPPS	4.54E-54	-0.746065233	0.354	0.488	1.09E-49	1.4
WDPCP	4.96E-54	0.410512523	0.249	0.116	1.19E-49	1.4
FAM157C	6.53E-54	0.359724409	0.257	0.12	1.57E-49	1.4
CEPT1	7.64E-54	0.38633213	0.413	0.241	1.84E-49	1.4
RNF150	1.08E-53	0.441780125	0.232	0.103	2.59E-49	1.4
INSIG2	1.10E-53	-0.641679949	0.129	0.274	2.65E-49	1.4
PTPRM	1.17E-53	0.605267652	0.275	0.138	2.83E-49	1.4
MAP2K5	1.67E-53	0.380049307	0.218	0.095	4.03E-49	1.4
DAAM1	1.69E-53	-0.732334792	0.42	0.549	4.08E-49	1.4
NFE2L2	1.69E-53	0.451452306	0.486	0.311	4.08E-49	1.4
RNF130	1.80E-53	0.354238198	0.264	0.126	4.35E-49	1.4
MYOF	1.93E-53	-0.629769149	0.423	0.553	4.64E-49	1.4
ABI1	3.64E-53	-0.602838157	0.679	0.745	8.77E-49	1.4
MACC1	4.58E-53	-0.796523119	0.293	0.443	1.10E-48	1.4
STX17	4.71E-53	0.307880183	0.239	0.109	1.13E-48	1.4
EIF4A2	5.85E-53	-0.480869576	0.783	0.824	1.41E-48	1.4
TLR5	8.91E-53	0.245202227	0.11	0.029	2.15E-48	1.4
COA1	1.18E-52	0.378403894	0.395	0.23	2.84E-48	1.4
ZNF429	1.35E-52	0.308256384	0.155	0.055	3.27E-48	1.4
DTNA	1.65E-52	-0.78171786	0.674	0.738	3.97E-48	1.4
TES	1.81E-52	-0.617407961	0.211	0.361	4.36E-48	1.4
PLEKHA5	1.98E-52	0.383323817	0.56	0.371	4.76E-48	1.4
RAD23B	2.29E-52	-0.68529675	0.316	0.456	5.52E-48	1.4
ZNF608	2.34E-52	0.405870754	0.267	0.13	5.65E-48	1.4
KCNJ2	2.68E-52	0.187109136	0.065	0.009	6.46E-48	1.4
RP1-28O10	3.04E-52	0.272941211	0.132	0.042	7.33E-48	1.4
SGPP2	5.23E-52	0.413045995	0.299	0.154	1.26E-47	1.4
ZKSCAN1	5.75E-52	0.391103845	0.388	0.221	1.39E-47	1.4
MTUS1	5.84E-52	-0.701852393	0.261	0.409	1.41E-47	1.4
SVIL	7.42E-52	0.401365684	0.909	0.808	1.79E-47	1.4
DPYSL2	1.25E-51	0.373555371	0.215	0.095	3.02E-47	1.4
GPRC5A	1.92E-51	-0.995126031	0.247	0.383	4.63E-47	1.4
BBX	3.01E-51	0.384504687	0.527	0.344	7.25E-47	1.4
CCDC14	5.98E-51	0.326649144	0.236	0.109	1.44E-46	1.4
ARPP19	8.05E-51	-0.595981489	0.225	0.372	1.94E-46	1.4
FLNB	1.13E-50	-0.728598971	0.276	0.416	2.72E-46	1.4

ANXA3	1.18E-50	-0.652037615	0.307	0.451	2.86E-46	1.4
CCL5	1.23E-50	0.271783622	0.067	0.011	2.96E-46	1.4
CATSPERB	2.25E-50	0.358089467	0.366	0.204	5.42E-46	1.4
SUMF1	2.82E-50	0.390947177	0.2	0.086	6.80E-46	1.4
IFNGR1	3.49E-50	0.418968773	0.374	0.216	8.41E-46	1.4
GBP3	3.51E-50	0.36406837	0.258	0.126	8.47E-46	1.4
RP11-115D	3.76E-50	0.272705692	0.077	0.015	9.07E-46	1.4
GRHL2	6.04E-50	0.360418778	0.395	0.233	1.46E-45	1.4
VCAM1	7.75E-50	0.227927743	0.053	0.006	1.87E-45	1.4
KLHL5	8.21E-50	0.370192136	0.279	0.142	1.98E-45	1.4
NAA25	1.19E-49	-0.67350762	0.211	0.356	2.87E-45	1.4
DOCK7	1.21E-49	0.430876634	0.358	0.203	2.91E-45	1.4
MCC	1.50E-49	0.34476593	0.197	0.084	3.62E-45	1.4
UGGT2	2.00E-49	0.475860618	0.271	0.137	4.81E-45	1.4
CAPN2	3.33E-49	-0.583637043	0.19	0.335	8.03E-45	1.4
LINC00536	4.57E-49	0.346861271	0.149	0.054	1.10E-44	1.4
ACTN1	6.93E-49	-0.68890009	0.167	0.314	1.67E-44	1.4
RP11-37B2	8.87E-49	0.381727528	0.418	0.248	2.14E-44	1.4
FGFR1	2.27E-48	-0.695758283	0.074	0.2	5.47E-44	1.4
MICAL3	2.81E-48	-0.702770879	0.197	0.339	6.78E-44	1.4
SMIM14	3.78E-48	0.349531987	0.251	0.123	9.12E-44	1.4
PHLPP1	3.79E-48	-0.756468081	0.366	0.493	9.13E-44	1.4
CTC-340D7	3.96E-48	0.207065364	0.071	0.013	9.55E-44	1.4
UBE2E3	6.69E-48	0.395806029	0.273	0.141	1.61E-43	1.4
SOS1	7.70E-48	0.399410949	0.516	0.335	1.86E-43	1.4
FTH1	9.15E-48	0.313137259	0.939	0.851	2.21E-43	1.4
RANBP17	1.12E-47	0.351178313	0.275	0.14	2.70E-43	1.4
SULF2	1.17E-47	0.326992824	0.179	0.073	2.83E-43	1.4
USP39	2.51E-47	0.331400035	0.484	0.306	6.05E-43	1.4
5-Mar	3.22E-47	-0.634271624	0.133	0.269	7.76E-43	1.4
FGGY	9.85E-47	0.388623962	0.339	0.193	2.38E-42	1.4
C4BPA	1.07E-46	0.282110546	0.098	0.027	2.57E-42	1.4
FAM20C	1.51E-46	0.39015473	0.178	0.074	3.64E-42	1.4
IGFBP7	1.52E-46	0.278912054	0.063	0.011	3.66E-42	1.4
CPD	1.76E-46	0.427683334	0.326	0.183	4.25E-42	1.4
KCNQ3	2.71E-46	0.367437324	0.112	0.034	6.53E-42	1.4
CH17-189H	2.71E-46	0.354192451	0.216	0.101	6.53E-42	1.4
DANT2	3.01E-46	0.29942555	0.155	0.059	7.25E-42	1.4
SRRM1	3.03E-46	-0.538674704	0.612	0.675	7.31E-42	1.4
ZC3H12C	3.03E-46	0.397153979	0.322	0.179	7.31E-42	1.4
YWHAH	4.30E-46	-0.531089708	0.121	0.253	1.04E-41	1.4
SLPI	4.32E-46	0.683956561	0.879	0.791	1.04E-41	1.4
MARK1	8.11E-46	0.286743365	0.157	0.06	1.95E-41	1.4
MINA	8.17E-46	0.261085732	0.143	0.053	1.97E-41	1.4
CATSPER2	1.07E-45	0.277770836	0.158	0.062	2.58E-41	1.4
EVL	1.67E-45	0.283321353	0.121	0.04	4.02E-41	1.4
SPDYE2	1.81E-45	0.320003178	0.29	0.155	4.37E-41	1.4

TBCK	1.83E-45	0.345211361	0.209	0.096	4.42E-41	1.4
SGMS2	2.22E-45	-0.541848982	0.085	0.211	5.35E-41	1.4
RP11-318C	2.36E-45	0.225735013	0.099	0.028	5.70E-41	1.4
ZNF638	2.44E-45	0.342025357	0.555	0.377	5.88E-41	1.4
TNFAIP8	2.65E-45	0.43136599	0.533	0.357	6.40E-41	1.4
FAF1	3.15E-45	0.376220621	0.384	0.23	7.59E-41	1.4
CMPK1	3.77E-45	0.344217057	0.442	0.275	9.10E-41	1.4
TANC2	4.55E-45	-0.641259774	0.19	0.326	1.10E-40	1.4
LAP3	4.75E-45	0.389451422	0.274	0.145	1.15E-40	1.4
NEDD4L	5.66E-45	-0.609368448	0.47	0.575	1.37E-40	1.4
CNKSR3	5.98E-45	0.47403477	0.642	0.468	1.44E-40	1.4
HELB	6.19E-45	0.336516418	0.203	0.093	1.49E-40	1.4
NFIA	6.38E-45	0.369680067	0.212	0.099	1.54E-40	1.4
SNX10	7.15E-45	0.237636797	0.109	0.034	1.72E-40	1.4
IKBKE	8.34E-45	0.234224821	0.091	0.024	2.01E-40	1.4
BAZ2B	9.79E-45	0.343770259	0.571	0.395	2.36E-40	1.4
ABHD17C	1.06E-44	0.291428828	0.125	0.043	2.56E-40	1.4
GLCCI1	1.22E-44	0.290798372	0.15	0.058	2.95E-40	1.4
LINC-PINT	1.68E-44	0.404378431	0.678	0.498	4.05E-40	1.4
ZMYND8	1.71E-44	0.34322513	0.373	0.221	4.12E-40	1.4
PLSCR1	1.76E-44	0.35448506	0.408	0.249	4.25E-40	1.4
KCNQ1	1.78E-44	0.308580449	0.17	0.07	4.30E-40	1.4
ANO10	2.44E-44	0.325476186	0.345	0.196	5.88E-40	1.4
C5orf46	2.46E-44	-0.582772566	0.132	0.265	5.93E-40	1.4
RERE	2.57E-44	0.332380904	0.566	0.383	6.19E-40	1.4
ENAH	4.69E-44	-0.702704091	0.407	0.516	1.13E-39	1.4
METTL15	4.73E-44	0.320131611	0.216	0.102	1.14E-39	1.4
SPPL3	5.10E-44	0.353080718	0.361	0.212	1.23E-39	1.4
CDC25B	5.76E-44	0.257804533	0.078	0.018	1.39E-39	1.4
MIR4435-2	5.92E-44	-0.68454834	0.397	0.516	1.43E-39	1.4
GABRG3	7.13E-44	0.280098874	0.087	0.023	1.72E-39	1.4
C1QTNF1	8.49E-44	0.246475016	0.097	0.027	2.05E-39	1.4
CTSV	9.66E-44	-0.885344304	0.123	0.255	2.33E-39	1.4
LIFR	1.85E-43	0.379221143	0.19	0.085	4.47E-39	1.4
SRD5A3	1.86E-43	0.23259651	0.112	0.036	4.49E-39	1.4
ATM	1.93E-43	0.281273095	0.137	0.051	4.66E-39	1.4
SNCAIP	2.39E-43	0.256257391	0.07	0.014	5.76E-39	1.4
CNDP2	2.44E-43	0.29398141	0.177	0.076	5.89E-39	1.4
AC027119.	2.88E-43	0.169070773	0.052	0.007	6.94E-39	1.4
IGF2BP2	3.29E-43	-0.680907133	0.431	0.539	7.94E-39	1.4
H1F0	3.62E-43	0.354412956	0.264	0.139	8.73E-39	1.4
SLC26A4	5.60E-43	0.322559073	0.058	0.01	1.35E-38	1.4
PARP14	8.57E-43	0.338426689	0.244	0.124	2.07E-38	1.4
CTB-113D1	9.32E-43	0.1459188	0.043	0.004	2.25E-38	1.4
AKR1C1	1.02E-42	0.451217613	0.203	0.095	2.47E-38	1.4
NRG2	2.11E-42	0.286959487	0.117	0.039	5.09E-38	1.4
MAP3K13	2.21E-42	0.261728186	0.916	0.798	5.32E-38	1.4

SDCCAG8	2.36E-42	0.371932257	0.279	0.15	5.68E-38	1.4
FRY	2.41E-42	0.23669099	0.088	0.024	5.80E-38	1.4
RP11-142M	2.50E-42	0.210651206	0.065	0.013	6.02E-38	1.4
PPP2R2A	3.39E-42	-0.584790425	0.318	0.443	8.16E-38	1.4
TPCN1	4.00E-42	0.311572038	0.199	0.093	9.66E-38	1.4
HOOK2	4.49E-42	0.340422063	0.254	0.132	1.08E-37	1.4
PARD3B	7.36E-42	0.352285235	0.213	0.103	1.77E-37	1.4
EIF4E	8.27E-42	-0.565643354	0.291	0.418	1.99E-37	1.4
EZR	1.06E-41	-0.603063973	0.212	0.346	2.55E-37	1.4
SOX10	1.13E-41	0.374935594	0.208	0.1	2.72E-37	1.4
KRT7	1.31E-41	-0.579671257	0.178	0.312	3.16E-37	1.4
MAPK14	1.32E-41	0.344074994	0.27	0.146	3.18E-37	1.4
PLD1	1.68E-41	0.314903646	0.169	0.073	4.04E-37	1.4
DTWD1	2.31E-41	0.258045787	0.13	0.048	5.57E-37	1.4
VPS13C	2.89E-41	0.325138946	0.321	0.184	6.97E-37	1.4
GABPB1-AS	3.59E-41	0.304776073	0.301	0.168	8.65E-37	1.4
RALGAPA2	3.68E-41	0.380672675	0.284	0.157	8.87E-37	1.4
MYCBP2	4.20E-41	0.320362589	0.264	0.14	1.01E-36	1.4
PPM1L	4.63E-41	0.298144251	0.136	0.052	1.12E-36	1.4
CTSB	4.64E-41	0.32197208	0.419	0.263	1.12E-36	1.4
ENTPD1-AS	5.97E-41	0.268188822	0.131	0.049	1.44E-36	1.4
CSF1	7.88E-41	0.347923995	0.157	0.065	1.90E-36	1.4
CYP27B1	8.65E-41	0.22722434	0.091	0.026	2.09E-36	1.4
CCDC125	9.05E-41	0.259498477	0.126	0.046	2.18E-36	1.4
WIPI1	9.41E-41	0.316608394	0.257	0.136	2.27E-36	1.4
S100A14	9.74E-41	-0.626671707	0.612	0.686	2.35E-36	1.4
TIMM23B	1.10E-40	0.340598775	0.302	0.171	2.66E-36	1.4
LINC00998	1.33E-40	0.291101359	0.26	0.137	3.20E-36	1.4
HDAC8	1.43E-40	0.348955678	0.327	0.192	3.44E-36	1.4
SLC27A4	1.78E-40	-0.699317887	0.475	0.595	4.28E-36	1.4
LRRC37A3	1.97E-40	0.269026154	0.201	0.095	4.74E-36	1.4
TRIM22	2.00E-40	0.278557481	0.181	0.082	4.83E-36	1.4
SHPRH	2.53E-40	0.286008351	0.177	0.079	6.11E-36	1.4
TMBIM6	2.66E-40	-0.41721865	0.772	0.809	6.42E-36	1.4
RSRC2	2.76E-40	-0.562460552	0.547	0.624	6.64E-36	1.4
CAST	3.55E-40	-0.562286016	0.482	0.566	8.56E-36	1.4
FMO2	4.36E-40	0.36444686	0.192	0.09	1.05E-35	1.4
CCAR1	4.57E-40	0.309995739	0.361	0.219	1.10E-35	1.4
VPS13B	5.20E-40	0.301759885	0.255	0.134	1.25E-35	1.4
TTC28	7.82E-40	0.338413631	0.162	0.07	1.89E-35	1.4
C4BPB	9.62E-40	0.212912505	0.095	0.029	2.32E-35	1.4
ZC3H6	9.66E-40	0.248523825	0.129	0.049	2.33E-35	1.4
PKD2	1.05E-39	0.280975567	0.151	0.062	2.53E-35	1.4
ZNF124	1.10E-39	0.284534189	0.155	0.066	2.65E-35	1.4
HIBADH	1.13E-39	0.318853613	0.287	0.159	2.72E-35	1.4
CTSS	1.16E-39	0.356501878	0.37	0.227	2.80E-35	1.4
USP40	1.59E-39	0.246439147	0.129	0.049	3.84E-35	1.4

CDK13	1.65E-39	0.335763284	0.465	0.309	3.98E-35	1.4
TCF12	2.11E-39	0.360605896	0.624	0.453	5.09E-35	1.4
AK3	2.14E-39	0.273985681	0.212	0.105	5.15E-35	1.4
TTC39C	2.50E-39	0.34832194	0.214	0.106	6.03E-35	1.4
DIMT1	2.58E-39	0.228941349	0.125	0.046	6.21E-35	1.4
NEO1	3.28E-39	0.287087186	0.15	0.062	7.91E-35	1.4
FAM135A	3.46E-39	0.292883893	0.147	0.06	8.33E-35	1.4
MOB4	3.49E-39	0.300878966	0.292	0.164	8.41E-35	1.4
SIPA1L1	4.96E-39	0.324276467	0.476	0.314	1.20E-34	1.4
CCT2	7.83E-39	-0.531549123	0.32	0.438	1.89E-34	1.4
GPATCH8	8.10E-39	0.334328437	0.36	0.22	1.95E-34	1.4
CLEC1A	1.11E-38	0.158481745	0.066	0.015	2.67E-34	1.4
NBEA	1.31E-38	0.359129475	0.182	0.085	3.16E-34	1.4
ZNF280D	1.38E-38	0.27095519	0.174	0.078	3.32E-34	1.4
NMI	1.58E-38	0.270276245	0.201	0.097	3.81E-34	1.4
CD47	1.87E-38	0.355860664	0.561	0.393	4.50E-34	1.4
TNFRSF1B	2.04E-38	0.22791023	0.135	0.053	4.92E-34	1.4
CARF	2.05E-38	0.200885282	0.074	0.019	4.95E-34	1.4
CHD2	2.49E-38	-0.599393442	0.345	0.462	6.00E-34	1.4
ARHGEF3	2.86E-38	0.335730993	0.421	0.271	6.89E-34	1.4
MAGED1	3.09E-38	0.27228542	0.181	0.083	7.44E-34	1.4
RP11-66B2	3.15E-38	0.269867413	0.171	0.077	7.60E-34	1.4
CCL20	3.30E-38	0.264219633	0.409	0.265	7.96E-34	1.4
BMPR2	3.37E-38	0.333688628	0.334	0.199	8.12E-34	1.4
FDPS	3.68E-38	0.333216609	0.582	0.419	8.86E-34	1.4
CCDC146	4.08E-38	0.355003802	0.277	0.154	9.83E-34	1.4
SCMH1	4.19E-38	0.309701672	0.314	0.182	1.01E-33	1.4
DBI	7.11E-38	0.324615242	0.799	0.657	1.71E-33	1.4
PARP8	8.17E-38	0.310853775	0.201	0.098	1.97E-33	1.4
RPRD2	8.80E-38	0.310590168	0.232	0.121	2.12E-33	1.4
LINC01183	8.90E-38	0.257034276	0.114	0.041	2.15E-33	1.4
TRAPPC9	8.96E-38	0.304288355	0.268	0.146	2.16E-33	1.4
ZNF195	9.34E-38	0.29453306	0.187	0.088	2.25E-33	1.4
MKL2	9.65E-38	0.350803474	0.363	0.223	2.33E-33	1.4
ESYT2	9.92E-38	-0.608254346	0.44	0.536	2.39E-33	1.4
UMAD1	1.08E-37	0.29016208	0.183	0.085	2.61E-33	1.4
PHF21A	1.37E-37	0.35357103	0.346	0.209	3.31E-33	1.4
CCDC66	1.50E-37	0.276197155	0.235	0.123	3.62E-33	1.4
LGALS2	1.73E-37	0.276722118	0.083	0.023	4.18E-33	1.4
GTF2IRD2B	1.78E-37	0.259396088	0.15	0.063	4.30E-33	1.4
SSR3	1.99E-37	-0.49839744	0.534	0.613	4.80E-33	1.4
CSTB	2.26E-37	0.389539661	0.277	0.156	5.46E-33	1.4
PTRF	2.41E-37	-0.442972498	0.094	0.206	5.81E-33	1.4
SNED1	2.50E-37	0.249168933	0.111	0.04	6.03E-33	1.4
PAPSS1	3.14E-37	0.35604092	0.491	0.335	7.57E-33	1.4
RSL24D1	3.19E-37	-0.37672805	0.709	0.758	7.70E-33	1.4
BCL11A	3.49E-37	0.241127344	0.115	0.042	8.41E-33	1.4

CCDC122	3.79E-37	0.200461023	0.097	0.031	9.15E-33	1.4
HIST2H2BE	3.81E-37	0.298559366	0.16	0.071	9.18E-33	1.4
WDR70	4.23E-37	0.303122349	0.208	0.104	1.02E-32	1.4
APLF	6.12E-37	0.257966207	0.146	0.062	1.48E-32	1.4
SEC22A	6.53E-37	0.27922231	0.219	0.111	1.58E-32	1.4
STAC2	7.44E-37	0.255570944	0.098	0.032	1.79E-32	1.4
TGFB2	7.85E-37	-0.630674023	0.153	0.276	1.89E-32	1.4
PTN	8.99E-37	-1.076361798	0.144	0.262	2.17E-32	1.4
RALGAPA1	9.16E-37	0.32072793	0.335	0.201	2.21E-32	1.4
RLF	9.63E-37	-0.688017491	0.264	0.379	2.32E-32	1.4
NBR1	1.07E-36	0.257790124	0.259	0.14	2.57E-32	1.4
SUCLG2	1.09E-36	0.263393659	0.165	0.074	2.62E-32	1.4
PUS10	1.13E-36	0.212587584	0.127	0.049	2.74E-32	1.4
C21orf91	1.17E-36	0.236294962	0.122	0.046	2.82E-32	1.4
TACSTD2	1.34E-36	-0.61390367	0.7	0.728	3.23E-32	1.4
SEMA6A-A!	1.38E-36	0.366648872	0.353	0.218	3.33E-32	1.4
TRMT10B	2.26E-36	0.241341211	0.185	0.088	5.45E-32	1.4
KRT19	2.64E-36	-0.67322222	0.225	0.349	6.37E-32	1.4
RABGAP1	2.67E-36	0.296725695	0.41	0.266	6.45E-32	1.4
ACSL3	3.20E-36	0.358226436	0.361	0.226	7.70E-32	1.4
ECHDC2	3.21E-36	0.26443929	0.189	0.091	7.75E-32	1.4
NDUFS5	3.86E-36	-0.348320775	0.814	0.842	9.32E-32	1.4
AC097724.	3.93E-36	0.259900519	0.148	0.064	9.48E-32	1.4
SLC2A1	4.04E-36	-0.383536309	0.02	0.101	9.75E-32	1.4
PLAUR	4.65E-36	-0.578862565	0.102	0.214	1.12E-31	1.4
H2AFZ	4.85E-36	-0.568460314	0.57	0.63	1.17E-31	1.4
FTL	5.40E-36	-0.906515485	0.477	0.557	1.30E-31	1.4
LIMK2	5.84E-36	0.302967501	0.203	0.102	1.41E-31	1.4
TTLL5	7.32E-36	0.327221398	0.261	0.145	1.77E-31	1.4
HILPDA	7.52E-36	-0.759649148	0.158	0.279	1.81E-31	1.4
C21orf62-A	9.42E-36	0.159939565	0.07	0.018	2.27E-31	1.4
DHRS7B	9.95E-36	0.237991013	0.123	0.048	2.40E-31	1.4
PARN	1.02E-35	0.246152082	0.146	0.063	2.46E-31	1.4
RBP5	1.11E-35	0.185640684	0.088	0.027	2.67E-31	1.4
PHIP	1.39E-35	0.315492955	0.538	0.379	3.36E-31	1.4
MGP	1.48E-35	-1.081573249	0.857	0.873	3.57E-31	1.4
RNASEH2B	1.49E-35	0.175412298	0.085	0.026	3.60E-31	1.4
CUX1	1.49E-35	0.293868249	0.323	0.194	3.60E-31	1.4
ATAD2B	1.60E-35	0.293734839	0.232	0.123	3.87E-31	1.4
STAT1	4.15E-35	0.211904664	0.135	0.056	1.00E-30	1.4
H6PD	4.63E-35	0.210799585	0.098	0.033	1.12E-30	1.4
SS18	4.92E-35	0.25579761	0.229	0.121	1.19E-30	1.4
IGFBP3	5.00E-35	-0.559576623	0.048	0.141	1.21E-30	1.4
SERTAD2	5.44E-35	-0.515899521	0.123	0.234	1.31E-30	1.4
ANKRA2	5.89E-35	0.204069031	0.122	0.048	1.42E-30	1.4
RAPGEF2	6.23E-35	-0.649406074	0.327	0.435	1.50E-30	1.4
DARS	7.45E-35	0.321765915	0.438	0.293	1.80E-30	1.4

NFATC2	1.08E-34	-0.557562618	0.072	0.172	2.61E-30	1.4
RICTOR	1.14E-34	-0.585137297	0.465	0.547	2.75E-30	1.4
KLK5	1.56E-34	-0.357759689	0.015	0.089	3.76E-30	1.4
ZNF544	1.67E-34	0.23359406	0.166	0.077	4.03E-30	1.4
C1orf147	1.84E-34	0.187897636	0.071	0.019	4.43E-30	1.4
MET	1.89E-34	-0.611740068	0.268	0.383	4.56E-30	1.4
NR2F2	2.12E-34	0.228363065	0.157	0.071	5.11E-30	1.4
TAF1D	2.13E-34	-0.578410416	0.241	0.352	5.14E-30	1.4
RP11-759A	2.58E-34	0.173624032	0.072	0.02	6.22E-30	1.4
ICE2	2.67E-34	0.192883021	0.099	0.034	6.44E-30	1.4
SLC25A12	3.24E-34	0.273862086	0.161	0.073	7.80E-30	1.4
IGF1R	3.27E-34	0.350570667	0.525	0.375	7.88E-30	1.4
N4BP2L1	3.98E-34	0.259894735	0.176	0.084	9.60E-30	1.4
BACH2	4.30E-34	-0.683167644	0.334	0.44	1.04E-29	1.4
KALRN	4.36E-34	0.28089654	0.203	0.103	1.05E-29	1.4
LHFPL2	4.83E-34	-0.574273102	0.321	0.428	1.17E-29	1.4
LINC01137	5.09E-34	0.21547597	0.111	0.042	1.23E-29	1.4
DRAM2	5.38E-34	0.281547409	0.395	0.255	1.30E-29	1.4
ASCC3	6.76E-34	0.307918032	0.299	0.177	1.63E-29	1.4
CDR2	6.93E-34	0.275717934	0.216	0.113	1.67E-29	1.4
RCOR3	7.34E-34	0.284719478	0.159	0.074	1.77E-29	1.4
RASGEF1A	7.80E-34	0.194181793	0.082	0.025	1.88E-29	1.4
PNRC1	9.09E-34	0.285396388	0.649	0.497	2.19E-29	1.4
SAA4	9.15E-34	0.223071313	0.385	0.24	2.21E-29	1.4
KCTD9	1.01E-33	-0.604364786	0.216	0.331	2.42E-29	1.4
C9orf91	1.03E-33	0.187749027	0.073	0.02	2.49E-29	1.4
SF3B6	1.12E-33	-0.430667624	0.63	0.677	2.70E-29	1.4
ZNF397	1.42E-33	0.247592694	0.184	0.091	3.42E-29	1.4
TMEM87A	1.53E-33	0.322562043	0.468	0.325	3.68E-29	1.4
SCAPER	1.67E-33	0.298514206	0.311	0.188	4.02E-29	1.4
CRTC3	1.72E-33	0.296230344	0.355	0.224	4.14E-29	1.4
PHF14	1.89E-33	0.324896866	0.22	0.119	4.57E-29	1.4
RPL22L1	1.96E-33	-0.526802362	0.518	0.578	4.72E-29	1.4
ALDH9A1	2.12E-33	0.227097407	0.146	0.064	5.11E-29	1.4
RP1-167A1	2.29E-33	0.232330969	0.106	0.039	5.52E-29	1.4
PARP4	2.54E-33	0.283054205	0.283	0.165	6.11E-29	1.4
MSH3	2.74E-33	0.312552695	0.17	0.082	6.60E-29	1.4
ISLR	3.01E-33	0.119580833	0.042	0.006	7.25E-29	1.4
WAC	3.04E-33	-0.480394596	0.581	0.645	7.33E-29	1.4
CEP192	4.27E-33	0.278884181	0.154	0.071	1.03E-28	1.4
ANKRD26	4.88E-33	0.225961264	0.131	0.055	1.18E-28	1.4
CASC4	5.01E-33	0.29093583	0.269	0.155	1.21E-28	1.4
NUDT7	5.02E-33	0.171816105	0.09	0.03	1.21E-28	1.4
DIAPH2	5.33E-33	0.329588428	0.373	0.239	1.29E-28	1.4
ATXN3	5.65E-33	0.286107645	0.247	0.139	1.36E-28	1.4
SLC12A2	6.88E-33	0.289981733	0.657	0.498	1.66E-28	1.4
PRKD1	7.51E-33	0.294788383	0.243	0.134	1.81E-28	1.4

EPB41L1	7.56E-33	0.314395655	0.168	0.081	1.82E-28	1.4
ZFP14	8.20E-33	0.20329349	0.126	0.053	1.98E-28	1.4
FLOT2	8.67E-33	0.239053395	0.106	0.04	2.09E-28	1.4
PAPD4	8.80E-33	0.302358538	0.392	0.259	2.12E-28	1.4
USP13	9.76E-33	0.274869479	0.152	0.07	2.35E-28	1.4
CD2AP	1.08E-32	-0.592699513	0.306	0.412	2.60E-28	1.4
CASP4	1.14E-32	0.277243601	0.39	0.255	2.76E-28	1.4
PTPRJ	1.32E-32	-0.554324197	0.24	0.351	3.18E-28	1.4
ADGRL2	1.36E-32	0.370304931	0.195	0.1	3.28E-28	1.4
RFX3-AS1	1.44E-32	0.250869672	0.126	0.053	3.48E-28	1.4
C10orf76	1.55E-32	0.223978777	0.208	0.108	3.74E-28	1.4
OGT	1.56E-32	-0.510425995	0.287	0.397	3.75E-28	1.4
COL6A2	1.70E-32	-0.551483691	0.071	0.166	4.10E-28	1.4
NAMPT	1.75E-32	0.413364875	0.687	0.548	4.21E-28	1.4
FBXO9	1.76E-32	0.191588649	0.126	0.052	4.25E-28	1.4
PLCB4	2.03E-32	0.277321686	0.192	0.098	4.88E-28	1.4
FUT8	2.51E-32	0.284675373	0.154	0.071	6.06E-28	1.4
PDE7A	2.63E-32	0.362255126	0.357	0.231	6.33E-28	1.4
TBC1D32	3.24E-32	0.261704598	0.102	0.038	7.81E-28	1.4
DOCK1	3.28E-32	0.301562674	0.401	0.265	7.91E-28	1.4
ITGB4	3.48E-32	0.2408438	0.1	0.037	8.38E-28	1.4
KIAA1324	3.53E-32	0.317131108	0.173	0.085	8.50E-28	1.4
MAGI2	3.79E-32	0.349595505	0.158	0.074	9.13E-28	1.4
MORF4L1	3.80E-32	-0.370702995	0.667	0.703	9.16E-28	1.4
TYW5	4.24E-32	0.204934277	0.117	0.047	1.02E-27	1.4
COBL	4.31E-32	-0.643741627	0.433	0.52	1.04E-27	1.4
FANCL	4.34E-32	0.209528414	0.138	0.06	1.05E-27	1.4
MALAT1	5.06E-32	-0.23928702	1	1	1.22E-27	1.4
RABGAP1L	5.25E-32	0.26170893	0.319	0.195	1.27E-27	1.4
MYBL1	7.22E-32	0.209505038	0.085	0.028	1.74E-27	1.4
CCDC91	7.26E-32	0.299056746	0.463	0.318	1.75E-27	1.4
PIP5K1B	8.62E-32	0.282823676	0.115	0.047	2.08E-27	1.4
CSGALNAC	8.80E-32	0.241875872	0.15	0.069	2.12E-27	1.4
C14orf159	8.94E-32	0.195712701	0.12	0.049	2.15E-27	1.4
WDR11	9.55E-32	0.226325112	0.136	0.06	2.30E-27	1.4
PIK3IP1	1.03E-31	0.191360971	0.119	0.049	2.48E-27	1.4
MICU1	1.06E-31	0.284775231	0.307	0.187	2.56E-27	1.4
PDIA6	1.28E-31	-0.52060487	0.166	0.274	3.08E-27	1.4
FBXW4	1.29E-31	0.246066367	0.134	0.058	3.12E-27	1.4
THAP6	1.48E-31	0.202401691	0.126	0.053	3.57E-27	1.4
PLA2G4C	1.69E-31	0.217296017	0.106	0.041	4.08E-27	1.4
YWHAE	1.82E-31	-0.419404684	0.569	0.636	4.40E-27	1.4
ATF3	1.84E-31	-0.628753191	0.266	0.371	4.45E-27	1.4
TAF15	2.18E-31	0.310150203	0.418	0.282	5.26E-27	1.4
CTB-91J4.1	2.48E-31	0.1644169	0.035	0.004	5.98E-27	1.4
SMC6	2.56E-31	0.211815615	0.131	0.057	6.17E-27	1.4
ASH1L	2.67E-31	0.294376443	0.607	0.461	6.44E-27	1.4

RP11-867G	3.07E-31	0.212378991	0.078	0.025	7.40E-27	1.4
OXSR1	3.07E-31	-0.462843101	0.154	0.262	7.41E-27	1.4
ACTR3C	3.13E-31	0.227024161	0.094	0.034	7.54E-27	1.4
ZNF254	3.14E-31	0.259076799	0.238	0.134	7.58E-27	1.4
SLC15A1	3.29E-31	0.192548779	0.069	0.02	7.95E-27	1.4
DIS3L2	3.83E-31	0.244326556	0.169	0.083	9.24E-27	1.4
CTPS2	3.94E-31	0.219832862	0.12	0.05	9.50E-27	1.4
CRADD	4.59E-31	0.300942674	0.163	0.079	1.11E-26	1.4
GARS	4.72E-31	-0.405949104	0.11	0.213	1.14E-26	1.4
GALNT15	5.11E-31	0.357518325	0.216	0.118	1.23E-26	1.4
CTD-2315E	5.64E-31	0.140430372	0.05	0.01	1.36E-26	1.4
STRA6	6.95E-31	0.106460141	0.031	0.003	1.68E-26	1.4
SLC6A16	7.90E-31	0.193429471	0.091	0.032	1.91E-26	1.4
AKR1C2	8.44E-31	0.255922504	0.131	0.058	2.04E-26	1.4
SAMD9L	8.61E-31	0.159811443	0.063	0.017	2.08E-26	1.4
NFIB	8.83E-31	0.2153676	0.925	0.826	2.13E-26	1.4
TLR1	9.18E-31	0.238230223	0.107	0.042	2.21E-26	1.4
L3MBTL4	9.28E-31	0.280729767	0.446	0.303	2.24E-26	1.4
TMCC1	9.83E-31	0.308329764	0.371	0.243	2.37E-26	1.4
ARFGEF2	1.08E-30	0.255219013	0.439	0.295	2.62E-26	1.4
RP11-692D	1.23E-30	0.184783134	0.069	0.02	2.97E-26	1.4
IQCK	1.42E-30	0.215801163	0.101	0.038	3.43E-26	1.4
SLCO1A2	1.54E-30	0.186537041	0.063	0.017	3.71E-26	1.4
SEMA4B	1.69E-30	0.314842134	0.24	0.137	4.08E-26	1.4
PLA2G4A	1.73E-30	0.189255134	0.093	0.034	4.18E-26	1.4
ZCCHC6	2.01E-30	0.296924782	0.447	0.309	4.85E-26	1.4
ABCA1	2.04E-30	0.11765816	0.051	0.011	4.91E-26	1.4
RAB11FIP1	2.05E-30	-0.506712114	0.448	0.54	4.93E-26	1.4
LIMCH1	2.06E-30	0.256511853	0.366	0.234	4.96E-26	1.4
ZNF438	2.14E-30	0.219252731	0.122	0.052	5.16E-26	1.4
ADD3	2.25E-30	0.295439889	0.234	0.133	5.44E-26	1.4
FOXO1	2.30E-30	0.264816622	0.343	0.219	5.54E-26	1.4
ANTXR1	2.44E-30	0.212563764	0.103	0.04	5.88E-26	1.4
RP11-1069	2.52E-30	0.160567469	0.067	0.019	6.08E-26	1.4
CLIC6	3.19E-30	0.257906033	0.174	0.087	7.69E-26	1.4
NIPAL3	3.40E-30	0.24447697	0.133	0.06	8.19E-26	1.4
IQGAP2	3.41E-30	0.205270231	0.129	0.057	8.22E-26	1.4
ZFHX3	3.47E-30	0.269611035	0.181	0.093	8.36E-26	1.4
ZNF43	3.76E-30	0.248324808	0.213	0.116	9.08E-26	1.4
FOSL1	5.27E-30	-0.396660124	0.04	0.12	1.27E-25	1.4
ATG10	5.42E-30	0.308057852	0.211	0.115	1.31E-25	1.4
DMGDH	6.32E-30	0.183202569	0.08	0.026	1.52E-25	1.4
FBXO32	6.52E-30	0.420991399	0.519	0.378	1.57E-25	1.4
SAP18	7.02E-30	-0.386905814	0.557	0.62	1.69E-25	1.4
MT-CO3	8.16E-30	0.17434485	0.99	0.996	1.97E-25	1.4
RAI14	8.57E-30	-0.445685479	0.098	0.197	2.07E-25	1.4
MGAT3	9.23E-30	0.135470738	0.048	0.01	2.23E-25	1.4

TSFM	9.30E-30	0.184369239	0.1	0.038	2.24E-25	1.4
CLASP2	9.80E-30	0.291083322	0.319	0.202	2.36E-25	1.4
PIK3C2B	9.95E-30	0.198863524	0.074	0.024	2.40E-25	1.4
ZNF235	1.04E-29	0.215634824	0.129	0.057	2.51E-25	1.4
ATP13A5	1.05E-29	0.289230442	0.089	0.032	2.54E-25	1.4
SUSD4	1.44E-29	0.149492387	0.071	0.022	3.48E-25	1.4
FAM129A	1.46E-29	0.337082786	0.587	0.44	3.52E-25	1.4
NAIP	1.63E-29	0.235940315	0.112	0.046	3.92E-25	1.4
FBXL20	1.66E-29	0.253533175	0.347	0.222	3.99E-25	1.4
PNPLA3	1.75E-29	0.188493175	0.075	0.024	4.23E-25	1.4
C2	1.81E-29	0.155284081	0.065	0.018	4.36E-25	1.4
RASSF4	1.93E-29	0.127289822	0.054	0.013	4.66E-25	1.4
FGFR2	2.06E-29	0.204578104	0.079	0.026	4.96E-25	1.4
KLF6	2.19E-29	-0.505068258	0.47	0.546	5.27E-25	1.4
CGNL1	2.39E-29	0.262281232	0.201	0.109	5.76E-25	1.4
TANK	2.45E-29	0.285901589	0.739	0.597	5.91E-25	1.4
MCPH1	2.91E-29	0.289902697	0.243	0.141	7.02E-25	1.4
NEK9	2.92E-29	0.203284822	0.121	0.052	7.04E-25	1.4
RPS6	2.96E-29	-0.188040835	0.975	0.987	7.13E-25	1.4
TTLL4	3.27E-29	0.259290459	0.246	0.143	7.88E-25	1.4
ZNF630	3.38E-29	0.169168884	0.063	0.018	8.16E-25	1.4
ARFGEF3	3.41E-29	-0.486805069	0.153	0.255	8.22E-25	1.4
CTD-2337A	3.42E-29	0.327152093	0.272	0.162	8.24E-25	1.4
ASCC1	3.78E-29	0.252496319	0.248	0.145	9.11E-25	1.4
RP11-774D	3.87E-29	0.18843986	0.108	0.044	9.34E-25	1.4
SPP1	4.18E-29	0.566420893	0.05	0.011	1.01E-24	1.4
TUBA1A	4.30E-29	-0.520443846	0.176	0.281	1.04E-24	1.4
CCDC6	4.41E-29	-0.503103602	0.264	0.368	1.06E-24	1.4
SRGAP2	4.71E-29	0.258984453	0.181	0.094	1.14E-24	1.4
PITPNA	4.78E-29	0.253526841	0.214	0.118	1.15E-24	1.4
DPP8	5.17E-29	0.24178824	0.142	0.066	1.25E-24	1.4
ZNF618	6.41E-29	0.132355315	0.056	0.014	1.55E-24	1.4
SYNE2	6.53E-29	0.288857388	0.617	0.472	1.57E-24	1.4
ERBB4	6.79E-29	0.310436224	0.177	0.092	1.64E-24	1.4
RPL34	6.99E-29	-0.18866723	0.975	0.988	1.69E-24	1.4
B4GALT1	7.29E-29	0.316821102	0.506	0.369	1.76E-24	1.4
SLC9A8	7.71E-29	0.217862861	0.147	0.07	1.86E-24	1.4
SLC35E3	8.02E-29	0.254653462	0.155	0.076	1.93E-24	1.4
TC2N	1.00E-28	0.270674136	0.454	0.313	2.42E-24	1.4
KCCAT211	1.04E-28	0.267666155	0.1	0.039	2.51E-24	1.4
PIBF1	1.09E-28	0.267900087	0.294	0.183	2.64E-24	1.4
ENSA	1.14E-28	0.285881976	0.484	0.346	2.75E-24	1.4
TXN	1.23E-28	-0.40311663	0.683	0.715	2.97E-24	1.4
ZNF710	1.29E-28	0.23303863	0.161	0.08	3.11E-24	1.4
TTBK2	1.43E-28	0.247278741	0.183	0.096	3.44E-24	1.4
BICD1	1.61E-28	0.331662074	0.274	0.167	3.89E-24	1.4
AKAP6	1.70E-28	0.217270422	0.096	0.037	4.09E-24	1.4

SEC62	1.70E-28	-0.48511492	0.587	0.639	4.09E-24	1.4
RNF175	1.76E-28	0.217189909	0.093	0.035	4.25E-24	1.4
ERBB2IP	1.79E-28	0.297228048	0.362	0.241	4.33E-24	1.4
SCLT1	1.81E-28	0.239330258	0.089	0.033	4.36E-24	1.4
ORC3	1.89E-28	0.207092898	0.135	0.062	4.55E-24	1.4
APTX	2.03E-28	0.23702919	0.212	0.118	4.89E-24	1.4
FAM177B	2.32E-28	0.348977877	0.749	0.604	5.59E-24	1.4
APOL1	2.41E-28	0.153644917	0.069	0.021	5.81E-24	1.4
SP1	2.53E-28	0.23945214	0.209	0.116	6.10E-24	1.4
VWA5A	2.63E-28	0.206403651	0.17	0.087	6.33E-24	1.4
TTC14	2.67E-28	0.223555428	0.126	0.056	6.43E-24	1.4
DYNLL1	2.78E-28	-0.501088716	0.157	0.257	6.69E-24	1.4
TMEM176B	3.00E-28	0.188283219	0.091	0.034	7.23E-24	1.4
FAM120B	3.07E-28	0.242758175	0.176	0.092	7.40E-24	1.4
LARP7	3.28E-28	0.24331695	0.25	0.148	7.90E-24	1.4
ZDHHC20	3.33E-28	0.283749393	0.22	0.125	8.02E-24	1.4
SMPDL3A	3.47E-28	0.130597629	0.051	0.012	8.36E-24	1.4
MCEE	3.47E-28	0.15433946	0.069	0.021	8.37E-24	1.4
GSAP	3.54E-28	0.254937391	0.224	0.127	8.54E-24	1.4
RBM6	3.86E-28	0.267918861	0.432	0.296	9.30E-24	1.4
SLC38A1	4.03E-28	-0.434190214	0.209	0.314	9.71E-24	1.4
LPL	4.12E-28	0.212546626	0.102	0.041	9.93E-24	1.4
POC1B	5.08E-28	0.204550557	0.156	0.077	1.22E-23	1.4
CHSY3	5.15E-28	0.197235973	0.045	0.009	1.24E-23	1.4
IRAK3	5.35E-28	0.122992666	0.049	0.011	1.29E-23	1.4
DYNLT1	5.46E-28	0.232036926	0.735	0.576	1.32E-23	1.4
LRIG3	7.45E-28	0.183688686	0.101	0.04	1.80E-23	1.4
WWC1	7.62E-28	0.353480436	0.555	0.417	1.84E-23	1.4
AC073283.[illegible]	7.62E-28	0.250228135	0.176	0.092	1.84E-23	1.4
PLEKHA7	9.09E-28	-0.168032614	0.979	0.987	2.19E-23	1.4
TTC17	9.40E-28	0.265479612	0.376	0.252	2.27E-23	1.4
RP5-1198O[illegible]	1.10E-27	0.331657972	0.23	0.133	2.66E-23	1.4
NAV2	1.12E-27	-0.763909509	0.384	0.455	2.69E-23	1.4
RAB29	1.12E-27	0.178628795	0.135	0.063	2.69E-23	1.4
CLK4	1.19E-27	0.202651679	0.115	0.05	2.88E-23	1.4
ARL4C	1.21E-27	-0.461605252	0.143	0.242	2.92E-23	1.4
SCGB1D2	1.35E-27	-1.368524629	0.039	0.114	3.25E-23	1.4
CIR1	1.39E-27	0.248955233	0.39	0.263	3.35E-23	1.4
RAP1GAP2	1.43E-27	0.315468357	0.24	0.142	3.46E-23	1.4
SLC16A1-A[illegible]	1.93E-27	0.252344092	0.176	0.092	4.66E-23	1.4
PHC3	2.34E-27	0.230271433	0.26	0.157	5.63E-23	1.4
CEP295	2.56E-27	0.168701192	0.105	0.043	6.18E-23	1.4
PACRGL	2.68E-27	0.140425771	0.074	0.025	6.46E-23	1.4
ZNF718	2.84E-27	0.197275823	0.105	0.044	6.85E-23	1.4
SYCP2	2.96E-27	0.166961225	0.066	0.02	7.14E-23	1.4
ATXN7L1	2.99E-27	0.190350784	0.107	0.045	7.21E-23	1.4
ELOVL6	3.21E-27	0.28485944	0.135	0.064	7.74E-23	1.4

RP5-1101C	3.25E-27	0.189975033	0.095	0.037	7.83E-23	1.4
EIF3E	3.88E-27	-0.320064499	0.78	0.805	9.35E-23	1.4
DDX5	4.66E-27	0.298578121	0.617	0.467	1.12E-22	1.4
TFCP2	4.97E-27	0.260732865	0.203	0.113	1.20E-22	1.4
GPRC5B	5.65E-27	0.230904993	0.112	0.049	1.36E-22	1.4
RBM41	5.93E-27	0.166288274	0.143	0.069	1.43E-22	1.4
LAMTOR5	6.07E-27	-0.380043873	0.572	0.625	1.46E-22	1.4
GPD2	6.89E-27	0.228617358	0.329	0.209	1.66E-22	1.4
SEPP1	6.99E-27	0.204537135	0.468	0.326	1.69E-22	1.4
DDX60	7.90E-27	0.148759957	0.055	0.015	1.91E-22	1.4
HMG20A	7.96E-27	0.194768523	0.102	0.042	1.92E-22	1.4
RFWD2	8.96E-27	0.243596374	0.387	0.26	2.16E-22	1.4
RPL14	9.28E-27	-0.247810406	0.97	0.985	2.24E-22	1.4
NSF	9.42E-27	0.248414491	0.228	0.132	2.27E-22	1.4
LYPLAL1	1.10E-26	0.198370027	0.129	0.06	2.66E-22	1.4
ELMOD3	1.11E-26	0.182606908	0.144	0.07	2.67E-22	1.4
PPP2CB	1.17E-26	-0.491946448	0.237	0.334	2.82E-22	1.4
KCMF1	1.20E-26	-0.519749093	0.232	0.328	2.88E-22	1.4
RBPMS	1.20E-26	0.261857892	0.864	0.763	2.89E-22	1.4
SLC47A1	1.22E-26	-0.219378747	0.932	0.951	2.94E-22	1.4
UBR1	1.27E-26	0.234661382	0.194	0.107	3.06E-22	1.4
MTX2	1.29E-26	0.215392498	0.118	0.053	3.11E-22	1.4
S100A10	1.33E-26	-0.603726282	0.235	0.335	3.20E-22	1.4
MARCKS	1.40E-26	0.318969808	0.526	0.395	3.37E-22	1.4
NAA38	1.59E-26	0.215066291	0.109	0.047	3.83E-22	1.4
NBN	2.01E-26	0.187101692	0.115	0.05	4.84E-22	1.4
SLC30A7	2.12E-26	0.261447482	0.18	0.097	5.11E-22	1.4
THUMPD1	2.35E-26	0.198193625	0.135	0.064	5.67E-22	1.4
RAD21	2.37E-26	-0.492233106	0.291	0.384	5.71E-22	1.4
ZNF22	2.37E-26	0.246903795	0.318	0.205	5.72E-22	1.4
KPNA1	2.45E-26	-0.474694448	0.199	0.297	5.91E-22	1.4
RP11-96H1	2.47E-26	0.288958288	0.172	0.092	5.96E-22	1.4
ZNF143	2.75E-26	-0.390251481	0.086	0.172	6.64E-22	1.4
BBS4	2.85E-26	0.171708369	0.093	0.037	6.88E-22	1.4
BBIP1	2.87E-26	0.207093745	0.164	0.085	6.91E-22	1.4
TAPBP	3.00E-26	0.244743237	0.188	0.102	7.24E-22	1.4
TTC39B	3.05E-26	0.223912538	0.16	0.083	7.36E-22	1.4
SEC61G	3.10E-26	-0.411256399	0.683	0.708	7.48E-22	1.4
ATP6AP1L	3.29E-26	0.129479559	0.063	0.019	7.94E-22	1.4
PACS1	3.37E-26	0.288215918	0.391	0.27	8.13E-22	1.4
RPS6KA3	4.00E-26	-0.512558665	0.272	0.371	9.64E-22	1.4
GBAS	4.28E-26	0.209577966	0.268	0.164	1.03E-21	1.4
ACSL1	4.43E-26	0.270925009	0.316	0.203	1.07E-21	1.4
DNAJC1	4.43E-26	0.220386885	0.286	0.179	1.07E-21	1.4
TEAD1	4.47E-26	-0.476712079	0.296	0.395	1.08E-21	1.4
PCBP1	4.71E-26	-0.45759979	0.429	0.497	1.14E-21	1.4
FAM63B	4.77E-26	0.230541368	0.195	0.109	1.15E-21	1.4

DHFR	5.08E-26	-0.381144681	0.832	0.854	1.23E-21	1.4
TRAF3IP2	5.44E-26	0.239259573	0.248	0.149	1.31E-21	1.4
WEE1	5.89E-26	-0.451550619	0.193	0.291	1.42E-21	1.4
PAWR	6.44E-26	-0.498931827	0.425	0.5	1.55E-21	1.4
RHBDF2	6.51E-26	0.149775096	0.115	0.05	1.57E-21	1.4
CXCL1	6.72E-26	0.366999677	0.334	0.223	1.62E-21	1.4
ATG7	7.60E-26	0.234973728	0.303	0.193	1.83E-21	1.4
S100A9	8.25E-26	0.416156642	0.115	0.051	1.99E-21	1.4
SMYD3	9.67E-26	0.314622875	0.311	0.202	2.33E-21	1.4
PTPN4	1.07E-25	0.20281408	0.146	0.072	2.59E-21	1.4
KCTD1	1.21E-25	0.183833036	0.085	0.032	2.92E-21	1.4
ALDH1A2	1.29E-25	0.213721255	0.076	0.027	3.12E-21	1.4
MPHOSPH8	1.33E-25	0.226789886	0.239	0.143	3.20E-21	1.4
RP11-39M2	1.33E-25	0.162986648	0.08	0.029	3.20E-21	1.4
HK2	1.34E-25	-0.432613719	0.106	0.195	3.24E-21	1.4
IL15RA	1.37E-25	0.196506215	0.102	0.043	3.31E-21	1.4
FTO	1.47E-25	0.230103462	0.321	0.207	3.55E-21	1.4
MIR222HG	1.48E-25	-0.400229012	0.044	0.117	3.56E-21	1.4
HIST1H2BB	1.51E-25	0.245174025	0.202	0.114	3.65E-21	1.4
PTGR1	1.60E-25	0.235067082	0.347	0.229	3.87E-21	1.4
ZNF135	1.78E-25	0.122054988	0.061	0.019	4.28E-21	1.4
PPA2	1.78E-25	0.248433923	0.28	0.175	4.29E-21	1.4
MAMDC2	1.79E-25	-0.287109811	0.928	0.944	4.32E-21	1.4
COX7B	1.83E-25	-0.372094533	0.629	0.667	4.42E-21	1.4
UBAC2	2.25E-25	0.2417605	0.294	0.186	5.44E-21	1.4
WDSUB1	2.33E-25	0.180655864	0.101	0.043	5.62E-21	1.4
CHDH	2.35E-25	0.19075221	0.096	0.039	5.66E-21	1.4
TUBA1B	2.43E-25	-0.456553322	0.2	0.297	5.86E-21	1.4
ELK3	2.49E-25	-0.392959132	0.117	0.207	6.00E-21	1.4
RPL35A	2.85E-25	-0.223268114	0.971	0.985	6.86E-21	1.4
LINC01235	2.96E-25	0.240322406	0.272	0.17	7.13E-21	1.4
MSI2	3.18E-25	0.254201507	0.273	0.171	7.68E-21	1.4
ZNF248	3.30E-25	0.193665549	0.09	0.036	7.96E-21	1.4
RNF144B	3.39E-25	0.303791737	0.44	0.318	8.17E-21	1.4
AMY2B	3.89E-25	0.140307604	0.078	0.029	9.37E-21	1.4
AC007566.	4.20E-25	0.146112734	0.077	0.028	1.01E-20	1.4
CAMK1D	4.33E-25	-0.460767895	0.088	0.172	1.04E-20	1.4
VTI1A	4.43E-25	0.222814546	0.239	0.143	1.07E-20	1.4
RCC1	4.66E-25	-0.467911249	0.289	0.378	1.12E-20	1.4
ARF6	5.00E-25	-0.413988479	0.123	0.212	1.21E-20	1.4
MEAF6	6.63E-25	-0.400056086	0.086	0.169	1.60E-20	1.4
ACVR2A	6.79E-25	0.194421705	0.089	0.036	1.64E-20	1.4
CADM1	6.86E-25	0.25263292	0.139	0.069	1.65E-20	1.4
HLCS	7.00E-25	0.231988243	0.137	0.067	1.69E-20	1.4
C1RL-AS1	7.16E-25	0.153491172	0.06	0.019	1.73E-20	1.4
ANXA11	7.36E-25	-0.442001755	0.176	0.27	1.77E-20	1.4
CTA-292E1	7.65E-25	0.225034878	0.17	0.091	1.84E-20	1.4

USP33	8.62E-25	0.2502686	0.299	0.192	2.08E-20	1.4
RNF170	8.72E-25	0.170592908	0.098	0.041	2.10E-20	1.4
RHOA	9.00E-25	-0.481756968	0.364	0.441	2.17E-20	1.4
MTERF1	9.93E-25	0.191231641	0.128	0.061	2.39E-20	1.4
THBS1	1.04E-24	-0.396015604	0.027	0.09	2.50E-20	1.4
BTBD9	1.19E-24	0.270274214	0.272	0.171	2.88E-20	1.4
ROCK2	1.28E-24	0.280905337	0.344	0.233	3.08E-20	1.4
NACA	1.39E-24	-0.214712161	0.953	0.966	3.36E-20	1.4
CEBPB	1.49E-24	0.203167095	0.212	0.123	3.60E-20	1.4
C5orf17	1.62E-24	0.26195418	0.11	0.049	3.90E-20	1.4
NCEH1	1.74E-24	0.335105221	0.298	0.193	4.19E-20	1.4
TAF1	1.74E-24	0.212809581	0.191	0.107	4.20E-20	1.4
SHROOM3	1.81E-24	-0.477459848	0.47	0.542	4.37E-20	1.4
INVS	1.82E-24	0.244978777	0.195	0.111	4.39E-20	1.4
ATP1B1	1.83E-24	-0.506744462	0.674	0.724	4.42E-20	1.4
MAP2	1.84E-24	-0.455362658	0.104	0.19	4.45E-20	1.4
C16orf45	2.14E-24	0.195987843	0.102	0.044	5.16E-20	1.4
PDSS2	2.14E-24	0.261196988	0.2	0.114	5.17E-20	1.4
PBRM1	2.26E-24	0.265281308	0.271	0.171	5.45E-20	1.4
RP11-367G	2.37E-24	0.112131041	0.043	0.01	5.71E-20	1.4
NIFK-AS1	2.37E-24	0.154969203	0.079	0.03	5.72E-20	1.4
ARHGEF28	2.39E-24	-0.679041462	0.324	0.405	5.76E-20	1.4
SIDT1	2.42E-24	0.125107544	0.039	0.008	5.83E-20	1.4
CUL5	2.63E-24	0.24136928	0.195	0.112	6.35E-20	1.4
TPM4	2.69E-24	-0.405025277	0.44	0.517	6.49E-20	1.4
YARS	2.70E-24	-0.392005712	0.209	0.305	6.51E-20	1.4
GRIP1	2.83E-24	0.24550475	0.275	0.173	6.83E-20	1.4
FBXL4	2.84E-24	0.185128336	0.136	0.067	6.85E-20	1.4
CEBPD	2.91E-24	0.221281642	0.3	0.193	7.03E-20	1.4
SLC39A14	3.02E-24	0.271508846	0.438	0.311	7.29E-20	1.4
DIRC2	3.31E-24	0.193557718	0.116	0.053	7.98E-20	1.4
RCAN1	3.37E-24	-0.392675356	0.607	0.683	8.12E-20	1.4
TEAD2	3.43E-24	0.180651681	0.12	0.057	8.26E-20	1.4
TRMT1L	3.77E-24	0.116183602	0.054	0.016	9.10E-20	1.4
PGM1	3.81E-24	0.193191046	0.181	0.1	9.20E-20	1.4
ZBED5	3.83E-24	0.19292129	0.125	0.06	9.23E-20	1.4
CH507-528	3.86E-24	0.128960651	0.063	0.02	9.30E-20	1.4
MFSD11	4.12E-24	0.184266812	0.119	0.056	9.93E-20	1.4
SSR2	4.25E-24	-0.313397963	0.759	0.766	1.02E-19	1.4
GOLGA4	4.27E-24	-0.419750418	0.626	0.659	1.03E-19	1.4
MXD1	4.40E-24	-0.431797109	0.097	0.182	1.06E-19	1.4
ACTN4	4.95E-24	-0.502478234	0.267	0.359	1.19E-19	1.4
RPL21	5.30E-24	-0.201902751	0.948	0.962	1.28E-19	1.4
SGMS1	5.76E-24	0.191879628	0.363	0.243	1.39E-19	1.4
FEM1B	5.78E-24	-0.332169987	0.068	0.146	1.39E-19	1.4
ZER1	5.80E-24	0.216408457	0.161	0.086	1.40E-19	1.4
SIPA1L3	6.20E-24	0.267590527	0.248	0.155	1.50E-19	1.4

NTN1	6.51E-24	0.225576403	0.22	0.131	1.57E-19	1.4
FAM120C	6.55E-24	0.19047383	0.095	0.04	1.58E-19	1.4
TNKS	6.72E-24	0.224549694	0.359	0.242	1.62E-19	1.4
ZNF780B	6.88E-24	0.14548286	0.077	0.029	1.66E-19	1.4
DPH6	7.45E-24	0.21957369	0.115	0.053	1.80E-19	1.4
ZNF708	7.74E-24	0.288911367	0.142	0.072	1.87E-19	1.4
UACA	7.97E-24	0.251498917	0.235	0.143	1.92E-19	1.4
MUC16	8.08E-24	0.222817614	0.091	0.038	1.95E-19	1.4
NCOA2	8.79E-24	0.229185955	0.599	0.463	2.12E-19	1.4
SNX29	8.96E-24	0.265061126	0.16	0.086	2.16E-19	1.4
CMC1	9.59E-24	0.167893206	0.089	0.036	2.31E-19	1.4
SLC9A7	1.02E-23	-0.506442158	0.223	0.314	2.46E-19	1.4
RHBDD1	1.03E-23	0.213910817	0.188	0.106	2.48E-19	1.4
TPM1	1.03E-23	0.259969898	0.693	0.574	2.49E-19	1.4
SMAD1	1.09E-23	0.1832332	0.138	0.07	2.62E-19	1.4
THUMPD3-	1.12E-23	0.204013121	0.396	0.273	2.70E-19	1.4
ZNF44	1.13E-23	0.145315853	0.095	0.04	2.73E-19	1.4
DLEU1	1.14E-23	0.19828443	0.226	0.135	2.75E-19	1.4
LRRC4C	1.18E-23	0.199170083	0.099	0.042	2.83E-19	1.4
PRLR	1.19E-23	0.248690979	0.194	0.111	2.87E-19	1.4
TFAP2C	1.21E-23	0.210913154	0.183	0.103	2.93E-19	1.4
SP110	1.26E-23	0.12488863	0.05	0.014	3.03E-19	1.4
OTUD6B-AS	1.40E-23	0.19616365	0.274	0.172	3.37E-19	1.4
NFIC	1.45E-23	0.250920304	0.242	0.149	3.50E-19	1.4
ZNF586	1.48E-23	0.202785262	0.154	0.081	3.57E-19	1.4
TBC1D14	1.50E-23	0.20752982	0.124	0.06	3.62E-19	1.4
STOX2	1.51E-23	0.239342917	0.247	0.154	3.63E-19	1.4
GTF2IRD2	1.74E-23	0.221976691	0.142	0.073	4.19E-19	1.4
COBLL1	1.83E-23	0.227786649	0.191	0.109	4.42E-19	1.4
IFT43	1.94E-23	0.200756876	0.134	0.066	4.67E-19	1.4
USP34	1.96E-23	0.216415711	0.695	0.547	4.73E-19	1.4
GS1-24F4.2	2.39E-23	0.29460106	0.163	0.089	5.76E-19	1.4
ZNFX1	2.50E-23	0.229331922	0.194	0.112	6.03E-19	1.4
ELL2	2.61E-23	-0.469677476	0.652	0.692	6.30E-19	1.4
ITSN2	2.68E-23	0.275171381	0.412	0.293	6.47E-19	1.4
TRIM13	2.82E-23	0.147080053	0.136	0.068	6.79E-19	1.4
KPNA3	2.87E-23	0.197741139	0.169	0.093	6.93E-19	1.4
NRG3	2.88E-23	0.250033015	0.151	0.08	6.96E-19	1.4
IL4R	3.03E-23	0.190439007	0.198	0.114	7.31E-19	1.4
PARVA	3.22E-23	0.224038623	0.206	0.121	7.77E-19	1.4
ATP9B	3.27E-23	0.156128574	0.128	0.062	7.88E-19	1.4
TCTN3	3.28E-23	0.139171861	0.072	0.026	7.90E-19	1.4
PTCH1	3.63E-23	0.11157874	0.038	0.008	8.75E-19	1.4
RPS4X	3.79E-23	-0.181836147	0.973	0.988	9.15E-19	1.4
HIBCH	3.82E-23	0.234015359	0.209	0.124	9.21E-19	1.4
FAM179B	3.84E-23	0.195030409	0.126	0.062	9.26E-19	1.4
SENP5	4.09E-23	0.224738743	0.282	0.181	9.86E-19	1.4

YIPF4	4.10E-23	0.20463616	0.147	0.077	9.88E-19	1.4
KDM3B	4.34E-23	0.191266696	0.246	0.153	1.05E-18	1.4
GALK2	4.51E-23	0.202021401	0.124	0.06	1.09E-18	1.4
PCM1	4.89E-23	0.254767357	0.26	0.166	1.18E-18	1.4
COMMD10	5.67E-23	0.227125671	0.283	0.185	1.37E-18	1.4
TYW1	6.34E-23	0.154062894	0.132	0.065	1.53E-18	1.4
ARHGEF9	6.66E-23	0.155819615	0.087	0.036	1.61E-18	1.4
IFITM2	6.91E-23	0.13770132	0.083	0.033	1.67E-18	1.4
PURA	7.55E-23	0.149526099	0.11	0.051	1.82E-18	1.4
SRSF3	7.61E-23	-0.431574126	0.555	0.615	1.84E-18	1.4
NSMCE1	7.66E-23	0.193700305	0.172	0.096	1.85E-18	1.4
POLR2F	8.09E-23	0.210236407	0.115	0.055	1.95E-18	1.4
KCND2	8.93E-23	0.407335847	0.137	0.071	2.15E-18	1.4
DMXL1	9.51E-23	0.171564932	0.18	0.101	2.29E-18	1.4
ERCC6L2	9.55E-23	0.194315483	0.139	0.071	2.30E-18	1.4
PELI2	9.60E-23	0.236880658	0.139	0.072	2.31E-18	1.4
TNRC6B	1.11E-22	0.228473328	0.52	0.382	2.68E-18	1.4
IL12RB1	1.13E-22	0.123024332	0.066	0.023	2.72E-18	1.4
C5orf28	1.20E-22	0.234110242	0.249	0.156	2.90E-18	1.4
SLC18B1	1.46E-22	0.24189565	0.274	0.175	3.52E-18	1.4
NUB1	1.50E-22	0.31433404	0.374	0.263	3.62E-18	1.4
TPI1	1.56E-22	-0.40907452	0.306	0.395	3.76E-18	1.4
MED13L	1.68E-22	0.272738302	0.763	0.647	4.05E-18	1.4
TAPT1-AS1	1.69E-22	0.175802746	0.109	0.05	4.08E-18	1.4
WDR7	1.79E-22	0.172974435	0.091	0.038	4.32E-18	1.4
TLK1	1.84E-22	0.238816618	0.28	0.182	4.45E-18	1.4
VRK2	1.85E-22	0.209860815	0.251	0.156	4.46E-18	1.4
DPH6-AS1	1.95E-22	0.125986577	0.05	0.015	4.71E-18	1.4
VPS54	2.05E-22	0.24002054	0.308	0.204	4.95E-18	1.4
MCTP2	2.09E-22	0.228286275	0.212	0.127	5.05E-18	1.4
VPS45	2.22E-22	0.206371876	0.138	0.071	5.35E-18	1.4
C11orf54	2.51E-22	0.198079508	0.152	0.082	6.05E-18	1.4
SOS2	2.74E-22	0.204325049	0.567	0.434	6.60E-18	1.4
RP11-16D2	2.76E-22	0.142503248	0.046	0.013	6.65E-18	1.4
NMRK1	3.13E-22	0.122244481	0.06	0.02	7.55E-18	1.4
NHLRC2	3.16E-22	0.164878627	0.108	0.05	7.62E-18	1.4
RP1-122P2	3.56E-22	0.166761273	0.067	0.024	8.59E-18	1.4
NUP107	3.61E-22	0.211136345	0.216	0.131	8.72E-18	1.4
ARMT1	3.63E-22	0.174901922	0.14	0.072	8.76E-18	1.4
ST3GAL6	4.70E-22	0.191001288	0.176	0.099	1.13E-17	1.4
WBP1L	4.88E-22	0.179886536	0.105	0.049	1.18E-17	1.4
AP1S3	4.90E-22	-0.283644188	0.03	0.09	1.18E-17	1.4
MAPRE1	5.37E-22	-0.33707737	0.099	0.179	1.29E-17	1.4
AMOT	5.37E-22	0.146264083	0.089	0.038	1.29E-17	1.4
RPL31	5.71E-22	-0.204268014	0.951	0.97	1.38E-17	1.4
KIF9-AS1	5.84E-22	0.182219031	0.085	0.035	1.41E-17	1.4
CR1L	6.29E-22	0.13960654	0.069	0.025	1.52E-17	1.4

DRAM1	6.46E-22	0.2508361	0.234	0.146	1.56E-17	1.4
PRPF3	6.48E-22	0.212168915	0.177	0.101	1.56E-17	1.4
MKL1	6.50E-22	-0.542978313	0.435	0.507	1.57E-17	1.4
PRKAG2	6.55E-22	0.283498793	0.23	0.144	1.58E-17	1.4
RP11-711K	6.80E-22	0.208236001	0.102	0.047	1.64E-17	1.4
ANKRD13C	7.04E-22	0.223465572	0.181	0.104	1.70E-17	1.4
AMD1	7.13E-22	-0.429881093	0.179	0.265	1.72E-17	1.4
ZNF133	7.32E-22	0.125147165	0.067	0.024	1.76E-17	1.4
RIOK3	7.46E-22	-0.461786709	0.236	0.319	1.80E-17	1.4
HP1BP3	7.47E-22	0.21569178	0.265	0.172	1.80E-17	1.4
PLEKHA6	7.64E-22	-0.219698602	0.916	0.926	1.84E-17	1.4
MLLT6	7.74E-22	0.201983023	0.121	0.06	1.87E-17	1.4
STPG2	9.27E-22	0.210934216	0.096	0.043	2.23E-17	1.4
MYC	9.90E-22	-0.377549216	0.091	0.169	2.39E-17	1.4
DDIT3	1.01E-21	-0.459141699	0.072	0.144	2.43E-17	1.4
ATPAF1	1.01E-21	0.114776483	0.056	0.018	2.44E-17	1.4
BCL6	1.07E-21	0.292577808	0.427	0.31	2.58E-17	1.4
AFF1	1.08E-21	0.178571202	0.384	0.266	2.60E-17	1.4
CKAP4	1.11E-21	0.218707356	0.23	0.143	2.68E-17	1.4
SECISBP2	1.15E-21	0.175845569	0.126	0.063	2.76E-17	1.4
ANXA5	1.20E-21	-0.473726586	0.187	0.274	2.88E-17	1.4
BPI	1.21E-21	0.155308217	0.077	0.031	2.92E-17	1.4
SWAP70	1.30E-21	-0.421530833	0.24	0.327	3.14E-17	1.4
SETD7	1.32E-21	0.221361362	0.16	0.089	3.18E-17	1.4
LIMS1	1.36E-21	-0.534552234	0.256	0.338	3.27E-17	1.4
RAB27A	1.59E-21	0.163910925	0.12	0.059	3.83E-17	1.4
TJP3	1.62E-21	0.134525659	0.066	0.024	3.90E-17	1.4
ZNF106	1.65E-21	0.173144416	0.175	0.099	3.97E-17	1.4
ARHGAP17	1.83E-21	-0.464577301	0.157	0.24	4.41E-17	1.4
SLC1A3	1.98E-21	0.161162276	0.09	0.039	4.77E-17	1.4
RPS3A	2.11E-21	-0.203042123	0.942	0.956	5.08E-17	1.4
PLS3	2.27E-21	-0.28940504	0.059	0.129	5.48E-17	1.4
LPIN1	2.50E-21	-0.518158239	0.283	0.364	6.02E-17	1.4
RAB8B	2.52E-21	-0.392689755	0.107	0.186	6.07E-17	1.4
FIGN	2.69E-21	0.2268025	0.191	0.113	6.50E-17	1.4
NHLRC3	2.70E-21	0.110833163	0.049	0.015	6.51E-17	1.4
CNTNAP3	2.92E-21	0.199306076	0.102	0.047	7.03E-17	1.4
PPP2R5A	2.95E-21	0.211813486	0.209	0.127	7.11E-17	1.4
CCT5	3.04E-21	-0.410412369	0.313	0.398	7.34E-17	1.4
PCLO	3.08E-21	0.206998361	0.08	0.033	7.42E-17	1.4
HNMT	3.33E-21	0.215032089	0.301	0.2	8.03E-17	1.4
RPL30	3.34E-21	-0.18172914	0.969	0.984	8.06E-17	1.4
KDM5B	3.36E-21	-0.438175255	0.533	0.567	8.11E-17	1.4
GALNT11	3.42E-21	0.190372506	0.131	0.068	8.26E-17	1.4
MTR	3.67E-21	0.18713407	0.113	0.055	8.84E-17	1.4
XPO4	3.67E-21	0.187258285	0.195	0.115	8.85E-17	1.4
ZNF37A	4.13E-21	0.178894215	0.141	0.075	9.97E-17	1.4

ITM2B	4.14E-21	0.222813637	0.331	0.227	9.98E-17	1.4
ZNF302	4.18E-21	0.115799126	0.062	0.022	1.01E-16	1.4
PLGRKT	4.46E-21	0.147050423	0.081	0.033	1.08E-16	1.4
SUPT20H	4.76E-21	0.171889578	0.129	0.066	1.15E-16	1.4
RP11-514P	4.79E-21	0.125181801	0.071	0.027	1.15E-16	1.4
R3HDM1	4.96E-21	0.165440307	0.101	0.047	1.19E-16	1.4
PHKB	5.03E-21	0.221708702	0.265	0.173	1.21E-16	1.4
YWHAQ	5.11E-21	-0.43958891	0.316	0.396	1.23E-16	1.4
NNT	5.35E-21	0.198559099	0.154	0.085	1.29E-16	1.4
HSPD1	6.05E-21	0.252878317	0.418	0.308	1.46E-16	1.4
LITAF	6.17E-21	0.225008488	0.592	0.464	1.49E-16	1.4
RP11-66B2	6.30E-21	0.132252333	0.062	0.022	1.52E-16	1.4
NT5C2	7.48E-21	0.236296859	0.462	0.344	1.80E-16	1.4
ACP6	7.77E-21	0.106888042	0.049	0.015	1.87E-16	1.4
ANKRD36C	7.79E-21	-0.456152489	0.508	0.578	1.88E-16	1.4
SLC25A3	8.79E-21	0.196854709	0.266	0.172	2.12E-16	1.4
SLC38A6	9.47E-21	0.132734888	0.071	0.028	2.28E-16	1.4
GATAD1	9.77E-21	0.133947638	0.073	0.029	2.36E-16	1.4
MTERF4	1.01E-20	0.173971832	0.133	0.07	2.45E-16	1.4
UFL1	1.07E-20	0.180139899	0.107	0.051	2.59E-16	1.4
CBR4	1.14E-20	0.182471372	0.347	0.238	2.76E-16	1.4
MIS18BP1	1.18E-20	0.205015721	0.186	0.11	2.86E-16	1.4
LURAP1L	1.22E-20	-0.370735011	0.159	0.244	2.94E-16	1.4
GOSR1	1.32E-20	0.17784048	0.237	0.149	3.19E-16	1.4
MACROD2	1.39E-20	0.293816482	0.336	0.236	3.35E-16	1.4
FAM78B	1.40E-20	0.181938854	0.117	0.058	3.38E-16	1.4
UBXN4	1.41E-20	0.225217099	0.357	0.252	3.39E-16	1.4
CAMKMT	1.52E-20	0.233797386	0.225	0.14	3.66E-16	1.4
DENND4C	1.54E-20	0.219871019	0.286	0.19	3.71E-16	1.4
RPS19BP1	1.57E-20	0.141918172	0.051	0.016	3.79E-16	1.4
KIAA1109	1.59E-20	0.192272855	0.176	0.102	3.84E-16	1.4
CECR7	1.66E-20	0.17850277	0.134	0.07	4.00E-16	1.4
MAL2	1.73E-20	-0.411168183	0.185	0.269	4.16E-16	1.4
FAM217B	1.78E-20	0.112203016	0.071	0.028	4.29E-16	1.4
GNAQ	1.89E-20	0.208655381	0.398	0.283	4.55E-16	1.4
SPDYE16	1.99E-20	0.184259786	0.148	0.081	4.79E-16	1.4
CCDC64	2.03E-20	0.154554009	0.063	0.023	4.89E-16	1.4
ZNF33B	2.06E-20	0.20258046	0.251	0.162	4.96E-16	1.4
ZNF274	2.08E-20	0.178014771	0.133	0.07	5.02E-16	1.4
ZC3H13	2.08E-20	0.199729211	0.247	0.158	5.02E-16	1.4
ZBTB8OS	2.12E-20	0.193727599	0.195	0.118	5.10E-16	1.4
GPBP1	2.19E-20	-0.38165135	0.593	0.626	5.29E-16	1.4
RASSF3	2.36E-20	-0.336201912	0.094	0.169	5.68E-16	1.4
MLH3	2.45E-20	0.175353683	0.15	0.083	5.92E-16	1.4
ARL15	2.48E-20	0.23694511	0.278	0.185	5.98E-16	1.4
C5orf56	2.62E-20	0.177584269	0.181	0.105	6.32E-16	1.4
EML5	2.70E-20	0.204707406	0.16	0.09	6.52E-16	1.4

STRBP	2.71E-20	0.206116081	0.258	0.167	6.53E-16	1.4
KLK7	2.75E-20	-0.200689782	0.007	0.049	6.62E-16	1.4
KMT5B	3.10E-20	0.193587614	0.224	0.139	7.46E-16	1.4
VPS50	3.11E-20	0.160172599	0.097	0.045	7.50E-16	1.4
RP11-499P	3.35E-20	0.133549819	0.071	0.028	8.07E-16	1.4
LINC01191	3.83E-20	0.13563227	0.094	0.043	9.23E-16	1.4
CLEC16A	4.24E-20	0.176083652	0.222	0.137	1.02E-15	1.4
MIR3142H(	4.52E-20	0.101734142	0.047	0.014	1.09E-15	1.4
RIC1	4.61E-20	0.175722298	0.161	0.091	1.11E-15	1.4
PRKRIP1	5.23E-20	0.18210266	0.199	0.12	1.26E-15	1.4
ELF1	5.25E-20	-0.421076883	0.393	0.463	1.27E-15	1.4
IDE	5.37E-20	0.167489009	0.12	0.061	1.29E-15	1.4
CYFIP1	5.68E-20	0.169939287	0.179	0.104	1.37E-15	1.4
NPAS2	5.78E-20	0.232511238	0.583	0.451	1.39E-15	1.4
TMEM108	5.89E-20	0.183844777	0.042	0.012	1.42E-15	1.4
SPOP	6.15E-20	0.145442151	0.137	0.073	1.48E-15	1.4
PTK2B	6.18E-20	0.156299193	0.096	0.045	1.49E-15	1.4
ROR1	6.52E-20	0.19413153	0.069	0.027	1.57E-15	1.4
DSG3	6.55E-20	-0.696081309	0.029	0.084	1.58E-15	1.4
IKBKB	6.77E-20	0.197499905	0.17	0.098	1.63E-15	1.4
FARS2	6.78E-20	0.195191243	0.129	0.068	1.63E-15	1.4
CLCN3	7.04E-20	0.199438278	0.253	0.165	1.70E-15	1.4
SFT2D2	7.07E-20	0.224420683	0.297	0.201	1.70E-15	1.4
PLD3	7.18E-20	0.147504885	0.079	0.033	1.73E-15	1.4
SUMO1	7.38E-20	-0.353730097	0.495	0.545	1.78E-15	1.4
XRN1	7.43E-20	0.221719939	0.425	0.31	1.79E-15	1.4
RBFOX2	7.64E-20	-0.294700993	0.953	0.968	1.84E-15	1.4
ZNF260	7.73E-20	0.141084521	0.138	0.074	1.86E-15	1.4
PCMTD1	8.24E-20	0.195841405	0.397	0.285	1.99E-15	1.4
AHCYL2	8.48E-20	0.186400232	0.126	0.065	2.04E-15	1.4
RFX3	8.51E-20	0.214301151	0.222	0.139	2.05E-15	1.4
ADAM10	8.56E-20	0.219091218	0.312	0.216	2.06E-15	1.4
IL34	8.69E-20	0.199757769	0.228	0.142	2.10E-15	1.4
ESR2	8.87E-20	0.186606033	0.165	0.095	2.14E-15	1.4
TMEM245	9.11E-20	0.168244059	0.148	0.082	2.20E-15	1.4
AASDH	9.60E-20	0.133849453	0.093	0.043	2.32E-15	1.4
PPP2R1B	1.02E-19	0.170968105	0.154	0.086	2.45E-15	1.4
OTUD7B	1.05E-19	-0.361891626	0.113	0.189	2.52E-15	1.4
POLK	1.06E-19	0.175480503	0.198	0.119	2.55E-15	1.4
STAT5B	1.06E-19	0.204760622	0.25	0.162	2.56E-15	1.4
SPATA6	1.10E-19	0.150587726	0.071	0.029	2.64E-15	1.4
MFSD6	1.14E-19	0.183679611	0.135	0.073	2.75E-15	1.4
DDR2	1.18E-19	0.233014485	0.17	0.099	2.85E-15	1.4
LHPP	1.20E-19	0.109613814	0.046	0.014	2.89E-15	1.4
PARG	1.21E-19	0.166490743	0.14	0.076	2.92E-15	1.4
ERO1A	1.29E-19	-0.462489183	0.293	0.372	3.12E-15	1.4
AGO4	1.38E-19	0.169345847	0.156	0.088	3.33E-15	1.4

PHC2	1.43E-19	0.174911741	0.137	0.074	3.44E-15	1.4
KLHDC1	1.52E-19	0.135342308	0.059	0.021	3.67E-15	1.4
ATP13A3	1.58E-19	-0.461935914	0.287	0.363	3.81E-15	1.4
CPEB4	1.63E-19	0.172914075	0.249	0.16	3.94E-15	1.4
HERC1	1.93E-19	-0.467006675	0.338	0.412	4.65E-15	1.4
WBP2NL	2.03E-19	0.148270267	0.088	0.04	4.89E-15	1.4
DNAH14	2.10E-19	0.171257792	0.107	0.052	5.06E-15	1.4
MAK	2.19E-19	0.180125623	0.135	0.073	5.29E-15	1.4
METAP2	2.38E-19	0.201729662	0.439	0.328	5.75E-15	1.4
LPCAT3	2.52E-19	0.129265998	0.09	0.041	6.09E-15	1.4
TAMM41	2.54E-19	0.132372146	0.076	0.032	6.13E-15	1.4
ENOX2	2.74E-19	0.182570468	0.093	0.043	6.61E-15	1.4
CCDC174	2.86E-19	0.179175044	0.25	0.161	6.89E-15	1.4
SYNJ2	3.00E-19	0.243025763	0.393	0.284	7.23E-15	1.4
FAM3B	3.00E-19	0.164660157	0.116	0.06	7.24E-15	1.4
RP11-106M	3.06E-19	0.130425606	0.076	0.032	7.39E-15	1.4
SP100	3.36E-19	0.206262251	0.206	0.129	8.11E-15	1.4
ANKH	3.37E-19	0.22944382	0.114	0.058	8.12E-15	1.4
ALG13	3.42E-19	0.161897904	0.15	0.084	8.24E-15	1.4
TMEM164	3.50E-19	0.132648451	0.077	0.033	8.45E-15	1.4
FOXO3	3.63E-19	0.178871935	0.409	0.299	8.76E-15	1.4
CPEB3	3.68E-19	-0.539987259	0.284	0.352	8.87E-15	1.4
RBM5	3.95E-19	0.187448991	0.218	0.137	9.53E-15	1.4
ATE1	4.02E-19	0.196047521	0.159	0.091	9.70E-15	1.4
C1orf21	4.14E-19	-0.446673539	0.166	0.245	9.97E-15	1.4
ZNF565	4.71E-19	0.180820529	0.24	0.154	1.14E-14	1.4
NUP153	4.95E-19	-0.422016127	0.211	0.291	1.19E-14	1.4
STIM1	5.07E-19	0.227647999	0.274	0.185	1.22E-14	1.4
ANXA4	5.58E-19	0.169446068	0.158	0.09	1.35E-14	1.4
FAM171A1	5.80E-19	0.176994255	0.112	0.057	1.40E-14	1.4
GNA13	5.99E-19	-0.377987434	0.142	0.219	1.44E-14	1.4
GIGYF2	6.16E-19	0.212690255	0.323	0.224	1.49E-14	1.4
ST3GAL1	6.76E-19	0.191706556	0.259	0.17	1.63E-14	1.4
FMO4	6.92E-19	0.136298944	0.074	0.031	1.67E-14	1.4
FAM208A	6.99E-19	0.146822342	0.112	0.057	1.69E-14	1.4
ZADH2	7.58E-19	0.122850064	0.064	0.025	1.83E-14	1.4
RGS10	8.07E-19	0.191186944	0.171	0.101	1.95E-14	1.4
SLC25A27	8.34E-19	0.1196361	0.058	0.021	2.01E-14	1.4
PTPRF	9.01E-19	0.197411765	0.136	0.074	2.17E-14	1.4
KPNB1	9.08E-19	-0.390376749	0.403	0.462	2.19E-14	1.4
CFLAR	9.20E-19	0.205543888	0.566	0.435	2.22E-14	1.4
CUL4A	9.84E-19	0.198921271	0.195	0.119	2.37E-14	1.4
MAP7D1	1.03E-18	-0.305648241	0.082	0.151	2.47E-14	1.4
RP11-123O	1.07E-18	0.201469919	0.284	0.192	2.57E-14	1.4
PLEKHM3	1.10E-18	0.12422957	0.082	0.036	2.64E-14	1.4
RTP4	1.14E-18	0.146395517	0.13	0.07	2.74E-14	1.4
GGA1	1.16E-18	0.145179294	0.077	0.033	2.80E-14	1.4

RAD9A	1.22E-18	0.157772329	0.155	0.089	2.95E-14	1.4
HLA-A	1.23E-18	0.217891989	0.412	0.301	2.98E-14	1.4
AC009313.	1.26E-18	0.160939385	0.185	0.111	3.05E-14	1.4
SEC24A	1.33E-18	-0.414128487	0.163	0.237	3.20E-14	1.4
EVI5	1.47E-18	0.208294523	0.192	0.118	3.54E-14	1.4
RP11-796E	1.58E-18	0.136406842	0.057	0.021	3.82E-14	1.4
CREB3L2	1.67E-18	0.225111448	0.164	0.096	4.03E-14	1.4
SRSF5	1.68E-18	0.181566609	0.403	0.29	4.05E-14	1.4
IFFO2	1.73E-18	-0.338811592	0.09	0.161	4.17E-14	1.4
PDE8A	1.77E-18	0.290219087	0.555	0.448	4.26E-14	1.4
PPM1K	1.80E-18	0.15969123	0.134	0.073	4.34E-14	1.4
LRP2	1.83E-18	0.248789904	0.302	0.208	4.41E-14	1.4
MRAS	1.85E-18	0.168065082	0.117	0.061	4.46E-14	1.4
LNX1	1.90E-18	0.173457925	0.14	0.078	4.58E-14	1.4
SLX4IP	1.96E-18	0.143923997	0.071	0.03	4.72E-14	1.4
TMOD1	1.99E-18	0.1851269	0.179	0.108	4.80E-14	1.4
BTBD11	2.01E-18	0.172026331	0.117	0.061	4.84E-14	1.4
RCAN3	2.02E-18	-0.746791379	0.133	0.207	4.88E-14	1.4
IPO7	2.22E-18	-0.371761515	0.231	0.311	5.36E-14	1.4
AHNAK	2.26E-18	-0.379908368	0.23	0.308	5.44E-14	1.4
PHLDB2	2.29E-18	0.243358808	0.321	0.228	5.52E-14	1.4
ATP1A1	2.34E-18	0.190547175	0.612	0.476	5.64E-14	1.4
CRY1	2.35E-18	-0.512999627	0.291	0.363	5.68E-14	1.4
RPL11	2.39E-18	-0.183404006	0.973	0.983	5.76E-14	1.4
VWA8	2.42E-18	0.180075085	0.105	0.053	5.84E-14	1.4
MCCC1	2.50E-18	0.152959953	0.155	0.089	6.03E-14	1.4
CHKA	2.61E-18	0.202727157	0.177	0.106	6.29E-14	1.4
TYW1B	2.73E-18	0.213366573	0.209	0.132	6.59E-14	1.4
ANKIB1	2.89E-18	0.183474343	0.398	0.291	6.96E-14	1.4
RUFY3	2.91E-18	0.224281335	0.236	0.154	7.02E-14	1.4
EMC2	2.94E-18	0.15443879	0.263	0.173	7.08E-14	1.4
BAMBI	3.10E-18	0.167685029	0.12	0.064	7.49E-14	1.4
RPL5	3.32E-18	-0.183275922	0.965	0.977	8.02E-14	1.4
DDAH1	3.39E-18	-0.301035121	0.052	0.112	8.18E-14	1.4
MTIF3	3.46E-18	0.189922641	0.316	0.218	8.35E-14	1.4
BRAF	4.10E-18	0.182622777	0.421	0.308	9.89E-14	1.4
CAP2	4.11E-18	0.194410715	0.143	0.081	9.90E-14	1.4
RFTN2	4.44E-18	0.151422669	0.042	0.013	1.07E-13	1.4
RPS17	4.91E-18	-0.151291836	0.952	0.967	1.18E-13	1.4
PTAR1	4.99E-18	0.171887686	0.14	0.079	1.20E-13	1.4
PMS1	5.02E-18	0.154713594	0.081	0.037	1.21E-13	1.4
TMCO1	5.16E-18	0.162315539	0.412	0.3	1.24E-13	1.4
OVOL2	5.22E-18	0.143889557	0.103	0.051	1.26E-13	1.4
ANAPC5	5.56E-18	0.114932675	0.164	0.096	1.34E-13	1.4
SYCP3	5.59E-18	0.138546769	0.058	0.022	1.35E-13	1.4
CNNM2	5.95E-18	0.13807172	0.09	0.042	1.44E-13	1.4
TMEM117	6.26E-18	0.150450854	0.07	0.03	1.51E-13	1.4

ACO1	6.31E-18	0.157491145	0.094	0.046	1.52E-13	1.4
TMEM135	6.42E-18	0.173715324	0.118	0.063	1.55E-13	1.4
C7orf73	6.66E-18	0.183217161	0.165	0.099	1.61E-13	1.4
NUBPL	6.77E-18	0.17166614	0.099	0.049	1.63E-13	1.4
B4GALT6	6.78E-18	0.148151187	0.071	0.03	1.63E-13	1.4
PIKFYVE	7.05E-18	0.172138171	0.148	0.085	1.70E-13	1.4
PAXBP1	7.24E-18	0.166766004	0.14	0.079	1.75E-13	1.4
GTF2H2	7.65E-18	0.124935032	0.077	0.034	1.85E-13	1.4
GLTP	8.04E-18	0.193796495	0.238	0.156	1.94E-13	1.4
USMG5	8.67E-18	-0.368395941	0.557	0.599	2.09E-13	1.4
RPAP2	8.68E-18	0.187780786	0.232	0.15	2.09E-13	1.4
CCDC25	8.76E-18	0.13955899	0.164	0.096	2.11E-13	1.4
TNFRSF11E	8.86E-18	-0.380439283	0.06	0.121	2.14E-13	1.4
ZNF720	9.09E-18	0.136702508	0.128	0.069	2.19E-13	1.4
ZFYVE9	9.12E-18	0.16612414	0.158	0.093	2.20E-13	1.4
VPS36	9.12E-18	0.166153863	0.157	0.091	2.20E-13	1.4
UGDH-AS1	9.15E-18	0.123443475	0.084	0.039	2.21E-13	1.4
GBP4	9.24E-18	0.105501389	0.045	0.015	2.23E-13	1.4
GLG1	9.47E-18	0.194390081	0.266	0.179	2.28E-13	1.4
DPYD-AS1	9.54E-18	0.195489732	0.135	0.075	2.30E-13	1.4
ADAMTS9	9.97E-18	-0.493828425	0.379	0.457	2.40E-13	1.4
RP11-286N	1.01E-17	0.12834154	0.082	0.037	2.43E-13	1.4
JMJD1C	1.05E-17	-0.358057391	0.666	0.683	2.53E-13	1.4
RHOXF1-AS	1.09E-17	0.10066448	0.043	0.014	2.62E-13	1.4
CEP350	1.12E-17	0.199867152	0.258	0.174	2.69E-13	1.4
ADARB1	1.14E-17	0.139401763	0.094	0.046	2.76E-13	1.4
CCPG1	1.15E-17	0.151047642	0.103	0.052	2.77E-13	1.4
PVT1	1.17E-17	-0.449831941	0.339	0.408	2.81E-13	1.4
P3H2	1.17E-17	0.238391925	0.186	0.114	2.83E-13	1.4
RUFY1	1.20E-17	0.187272682	0.118	0.063	2.89E-13	1.4
RP11-353M	1.23E-17	0.126952406	0.045	0.014	2.96E-13	1.4
KDM4C	1.24E-17	0.149719346	0.107	0.055	2.98E-13	1.4
GS1-114I9.	1.25E-17	0.169734033	0.179	0.109	3.01E-13	1.4
DNAJB14	1.32E-17	0.180027322	0.139	0.078	3.19E-13	1.4
SLC25A46	1.33E-17	0.122689821	0.07	0.03	3.21E-13	1.4
DDHD1	1.36E-17	0.119901275	0.055	0.02	3.28E-13	1.4
TUG1	1.40E-17	0.169357437	0.167	0.1	3.37E-13	1.4
PARP9	1.42E-17	0.109886363	0.089	0.042	3.42E-13	1.4
FGF2	1.43E-17	-0.340279754	0.047	0.104	3.45E-13	1.4
TLE4	1.45E-17	-0.41797361	0.355	0.422	3.50E-13	1.4
VDR	1.50E-17	0.144294846	0.141	0.08	3.61E-13	1.4
CTSO	1.63E-17	0.105539382	0.054	0.02	3.93E-13	1.4
STAT5A	1.64E-17	0.149747263	0.11	0.057	3.95E-13	1.4
TRMT11	1.66E-17	0.160466814	0.191	0.118	4.01E-13	1.4
EGFR	1.67E-17	0.188329445	0.418	0.306	4.02E-13	1.4
SYNRG	1.77E-17	0.162637346	0.093	0.045	4.28E-13	1.4
DHTKD1	1.84E-17	0.108470564	0.065	0.026	4.44E-13	1.4

SLC37A1	1.95E-17	0.133821255	0.084	0.039	4.69E-13	1.4
ZBTB1	1.97E-17	0.144060151	0.133	0.074	4.75E-13	1.4
PSPC1	1.99E-17	0.193239174	0.223	0.145	4.79E-13	1.4
GTF2H5	2.03E-17	0.17057159	0.293	0.201	4.90E-13	1.4
GJC3	2.12E-17	-0.263936093	0.044	0.1	5.11E-13	1.4
ZZZ3	2.16E-17	0.217630622	0.236	0.157	5.20E-13	1.4
IFNAR2	2.32E-17	0.152542317	0.381	0.275	5.58E-13	1.4
RP11-541G	2.37E-17	0.110704515	0.039	0.011	5.71E-13	1.4
DENND1A	2.46E-17	0.197427488	0.283	0.195	5.94E-13	1.4
TBC1D19	2.52E-17	0.12010762	0.049	0.017	6.09E-13	1.4
ZNF24	2.57E-17	0.187273585	0.299	0.209	6.19E-13	1.4
GRIN2B	2.64E-17	0.139175123	0.061	0.024	6.37E-13	1.4
INO80	2.67E-17	0.199325118	0.22	0.144	6.44E-13	1.4
TDRD3	2.80E-17	0.163117917	0.116	0.062	6.76E-13	1.4
MRPS6	3.17E-17	-0.383367385	0.137	0.21	7.65E-13	1.4
ZEB1	3.27E-17	0.170262123	0.082	0.038	7.89E-13	1.4
C3orf52	3.33E-17	-0.234973215	0.031	0.082	8.04E-13	1.4
RP11-544A	3.37E-17	0.132566409	0.07	0.03	8.13E-13	1.4
YLPM1	3.53E-17	0.186098637	0.177	0.109	8.51E-13	1.4
RNF181	3.70E-17	-0.398946657	0.335	0.403	8.92E-13	1.4
SMIM12	3.73E-17	0.12710406	0.068	0.029	8.99E-13	1.4
IFT88	3.95E-17	0.148324037	0.088	0.042	9.52E-13	1.4
MACF1	4.00E-17	-0.416184146	0.615	0.641	9.64E-13	1.4
SERP2	4.17E-17	0.137930394	0.042	0.013	1.00E-12	1.4
WDR27	4.17E-17	0.134744324	0.073	0.032	1.01E-12	1.4
CASD1	4.42E-17	0.151683215	0.096	0.048	1.07E-12	1.4
UBA6-AS1	4.43E-17	0.187492337	0.132	0.074	1.07E-12	1.4
KRCC1	4.48E-17	0.141108506	0.119	0.064	1.08E-12	1.4
DDI2	4.51E-17	0.173337492	0.151	0.089	1.09E-12	1.4
VPS29	4.55E-17	-0.352158117	0.335	0.408	1.10E-12	1.4
STK17B	4.58E-17	0.196078401	0.172	0.105	1.10E-12	1.4
PNPLA8	4.69E-17	-0.433574722	0.233	0.309	1.13E-12	1.4
EGFLAM	4.73E-17	0.130988726	0.052	0.019	1.14E-12	1.4
MT-ND2	4.77E-17	0.132734063	0.977	0.975	1.15E-12	1.4
CDC42EP5	4.84E-17	0.124008782	0.061	0.024	1.17E-12	1.4
FERMT2	5.58E-17	-0.342373808	0.111	0.18	1.34E-12	1.4
NFATC3	5.62E-17	0.195269895	0.217	0.14	1.36E-12	1.4
RPS27A	6.08E-17	-0.157639546	0.978	0.988	1.47E-12	1.4
APPBP2	6.20E-17	0.16925487	0.157	0.093	1.49E-12	1.4
CD46	6.28E-17	0.235469495	0.51	0.399	1.52E-12	1.4
MTHFD2	6.38E-17	-0.223171509	0.046	0.102	1.54E-12	1.4
C7orf49	6.42E-17	0.124794718	0.055	0.021	1.55E-12	1.4
DHRS3	6.61E-17	0.204492234	0.134	0.076	1.59E-12	1.4
TACC1	6.88E-17	0.272114578	0.3	0.213	1.66E-12	1.4
RP3-325F2:	6.90E-17	0.119052061	0.061	0.025	1.66E-12	1.4
C1orf132	6.97E-17	0.161582341	0.105	0.054	1.68E-12	1.4
CCNY	7.03E-17	-0.5377933	0.206	0.28	1.70E-12	1.4

TCHP	7.65E-17	0.214898626	0.07	0.03	1.84E-12	1.4
ZNF226	7.74E-17	0.141194172	0.118	0.064	1.87E-12	1.4
IFRD1	7.81E-17	-0.433615316	0.327	0.397	1.88E-12	1.4
ODAM	7.90E-17	0.309744965	0.048	0.017	1.91E-12	1.4
MMP24	8.00E-17	0.145591912	0.153	0.09	1.93E-12	1.4
HYDIN	8.14E-17	0.143379095	0.052	0.019	1.96E-12	1.4
SQRDL	8.15E-17	0.145615229	0.243	0.161	1.97E-12	1.4
SYT7	8.58E-17	0.106106929	0.058	0.023	2.07E-12	1.4
SNRPD1	8.68E-17	-0.379690541	0.363	0.421	2.09E-12	1.4
CCL28	8.86E-17	0.243923437	0.78	0.681	2.14E-12	1.4
GNG12	9.10E-17	-0.382837749	0.252	0.323	2.19E-12	1.4
SLC30A5	9.51E-17	0.137872545	0.104	0.053	2.29E-12	1.4
DZIP3	9.54E-17	0.114737704	0.072	0.032	2.30E-12	1.4
NR2C1	9.58E-17	0.147833741	0.095	0.048	2.31E-12	1.4
DCAF10	9.60E-17	0.167724652	0.247	0.166	2.31E-12	1.4
CEP162	1.00E-16	0.122250195	0.069	0.03	2.41E-12	1.4
ZNF791	1.00E-16	0.153187639	0.159	0.095	2.42E-12	1.4
MAMLD1	1.02E-16	-0.21580363	0.024	0.07	2.45E-12	1.4
ZNF107	1.08E-16	0.149202087	0.114	0.061	2.61E-12	1.4
S100A7	1.10E-16	0.787468987	0.054	0.02	2.66E-12	1.4
SCYL3	1.15E-16	0.13287501	0.058	0.023	2.76E-12	1.4
KB-1562D1	1.16E-16	0.137656774	0.071	0.031	2.80E-12	1.4
LINC01135	1.27E-16	0.102987026	0.035	0.01	3.07E-12	1.4
EEF1A1	1.31E-16	-0.144316973	0.975	0.986	3.16E-12	1.4
EIF2AK2	1.31E-16	0.159483143	0.152	0.09	3.17E-12	1.4
AP000998.	1.37E-16	0.124987592	0.028	0.006	3.30E-12	1.4
DICER1	1.38E-16	0.178299422	0.225	0.147	3.32E-12	1.4
NPC2	1.41E-16	0.245391659	0.541	0.432	3.41E-12	1.4
XPR1	1.41E-16	0.179690723	0.172	0.105	3.41E-12	1.4
CARD14	1.43E-16	0.109331845	0.059	0.023	3.46E-12	1.4
NDUFAF6	1.44E-16	0.123107206	0.082	0.039	3.47E-12	1.4
SH3GL1	1.57E-16	-0.251398128	0.049	0.104	3.78E-12	1.4
ZNF131	1.57E-16	0.175241421	0.193	0.123	3.79E-12	1.4
WNK1	1.62E-16	0.188817808	0.24	0.162	3.90E-12	1.4
FOCAD	1.62E-16	0.166694335	0.239	0.159	3.91E-12	1.4
C9orf72	1.63E-16	0.143472582	0.126	0.07	3.93E-12	1.4
METTL17	1.64E-16	-0.223463614	0.029	0.077	3.94E-12	1.4
RSPH3	1.70E-16	0.141059069	0.103	0.053	4.11E-12	1.4
TIMMDC1	1.72E-16	0.129356416	0.086	0.041	4.14E-12	1.4
RP11-83A2	1.72E-16	0.148107163	0.168	0.102	4.14E-12	1.4
OCLN	1.79E-16	-0.36749721	0.262	0.333	4.32E-12	1.4
TMEM9B-A	1.83E-16	0.109999466	0.043	0.014	4.42E-12	1.4
RAD50	1.87E-16	0.153353745	0.145	0.084	4.51E-12	1.4
TATDN3	1.88E-16	0.143348259	0.059	0.024	4.54E-12	1.4
CREB5	1.90E-16	-0.474894457	0.262	0.338	4.57E-12	1.4
HMGCL	1.93E-16	0.102972188	0.049	0.018	4.65E-12	1.4
APOO	2.03E-16	-0.175878428	0.973	0.985	4.89E-12	1.4

SSPN	2.06E-16	0.15136013	0.076	0.035	4.96E-12	1.4
ZPLD1	2.07E-16	-0.495493328	0.081	0.143	4.98E-12	1.4
INTS8	2.07E-16	0.110469797	0.06	0.024	4.99E-12	1.4
UBASH3B	2.08E-16	-0.243553335	0.028	0.076	5.03E-12	1.4
RP11-420A	2.15E-16	0.122617312	0.06	0.024	5.17E-12	1.4
KRT80	2.26E-16	-0.164217341	0.013	0.052	5.45E-12	1.4
CASK	2.28E-16	0.166862011	0.365	0.263	5.49E-12	1.4
TNFSF15	2.30E-16	0.120910843	0.054	0.021	5.54E-12	1.4
RALGPS2	2.49E-16	-0.397051469	0.113	0.178	6.00E-12	1.4
PRR14L	2.49E-16	0.165967986	0.13	0.074	6.01E-12	1.4
TRAPPC11	2.54E-16	0.145000282	0.1	0.052	6.13E-12	1.4
MFSD8	2.61E-16	0.127251349	0.088	0.043	6.29E-12	1.4
APBA2	2.62E-16	0.128613467	0.093	0.046	6.32E-12	1.4
ST3GAL4	2.63E-16	0.105988856	0.051	0.019	6.33E-12	1.4
RBM23	2.69E-16	0.150239059	0.129	0.073	6.47E-12	1.4
MITD1	2.69E-16	0.141704982	0.163	0.099	6.49E-12	1.4
KLHL20	2.74E-16	0.129111918	0.113	0.061	6.60E-12	1.4
RAP2B	2.89E-16	-0.38952505	0.141	0.208	6.98E-12	1.4
CMTM8	2.95E-16	0.208469329	0.255	0.175	7.12E-12	1.4
TRAF3	3.08E-16	0.214806136	0.186	0.118	7.43E-12	1.4
FXR1	3.10E-16	-0.357501014	0.384	0.443	7.47E-12	1.4
ROBO1	3.24E-16	0.147551129	0.114	0.062	7.81E-12	1.4
LINC00623	3.25E-16	0.135266997	0.073	0.033	7.84E-12	1.4
SRP14	3.32E-16	-0.227533597	0.851	0.841	8.02E-12	1.4
AVIL	3.42E-16	0.116855192	0.079	0.037	8.24E-12	1.4
LRIF1	3.55E-16	0.125477729	0.106	0.056	8.56E-12	1.4
CTD-2561J	3.57E-16	0.124213429	0.072	0.032	8.61E-12	1.4
RP11-356C	3.74E-16	0.148036255	0.325	0.227	9.02E-12	1.4
CDC42BPA	3.87E-16	0.18026393	0.359	0.261	9.33E-12	1.4
CD24	3.96E-16	0.323433695	0.641	0.544	9.55E-12	1.4
FOSL2	3.98E-16	0.152838697	0.24	0.16	9.60E-12	1.4
RABEP1	4.02E-16	0.148288459	0.15	0.089	9.69E-12	1.4
TMEM161E	4.04E-16	0.144644889	0.144	0.084	9.74E-12	1.4
ARL17A	4.10E-16	0.13895006	0.099	0.051	9.87E-12	1.4
RP11-499F	4.33E-16	0.135863599	0.035	0.01	1.04E-11	1.4
ROPN1	4.42E-16	-0.221629268	0.032	0.079	1.07E-11	1.4
LCORL	4.45E-16	0.168299044	0.167	0.102	1.07E-11	1.4
FZD6	4.49E-16	0.145919829	0.085	0.042	1.08E-11	1.4
CLDN8	4.56E-16	0.148580274	0.129	0.073	1.10E-11	1.4
MAP4K3	4.58E-16	0.188810805	0.235	0.157	1.10E-11	1.4
NEDD9	4.58E-16	-0.438657088	0.219	0.289	1.10E-11	1.4
RHOT1	4.64E-16	0.163222696	0.135	0.077	1.12E-11	1.4
HELZ	4.65E-16	-0.37538508	0.277	0.343	1.12E-11	1.4
TASP1	4.69E-16	0.140261017	0.206	0.133	1.13E-11	1.4
RP5-945F2	4.83E-16	-0.394559442	0.039	0.091	1.16E-11	1.4
BRINP1	4.89E-16	0.123037025	0.264	0.18	1.18E-11	1.4
ACAD10	4.96E-16	0.106933657	0.053	0.021	1.20E-11	1.4

LINC01572	5.06E-16	0.119512221	0.07	0.031	1.22E-11	1.4
MYH9	5.16E-16	-0.350524754	0.44	0.505	1.24E-11	1.4
ANAPC13	5.16E-16	0.142646396	0.176	0.109	1.24E-11	1.4
LRRC8B	5.27E-16	0.109842123	0.203	0.129	1.27E-11	1.4
MASTL	5.32E-16	0.143064	0.083	0.04	1.28E-11	1.4
RPS15A	5.39E-16	-0.165053965	0.96	0.973	1.30E-11	1.4
TTC23	5.45E-16	0.126500393	0.102	0.053	1.31E-11	1.4
ACTR3	5.55E-16	-0.348993863	0.249	0.322	1.34E-11	1.4
HSDL2	5.73E-16	0.167029705	0.151	0.091	1.38E-11	1.4
RARRES3	6.22E-16	0.185797206	0.39	0.29	1.50E-11	1.4
GSDMC	6.23E-16	0.111948294	0.064	0.028	1.50E-11	1.4
SLC39A11	6.51E-16	0.176000764	0.115	0.063	1.57E-11	1.4
PTP4A1	6.54E-16	-0.397798136	0.158	0.229	1.58E-11	1.4
RP13-726E	6.58E-16	0.105819722	0.047	0.017	1.59E-11	1.4
MSRA	6.62E-16	0.148859128	0.107	0.057	1.60E-11	1.4
AFTPH	6.69E-16	-0.369218439	0.263	0.333	1.61E-11	1.4
PDLIM5	6.78E-16	-0.33634448	0.85	0.834	1.63E-11	1.4
EIF4G3	7.67E-16	0.166664558	0.401	0.297	1.85E-11	1.4
DCLK2	7.68E-16	0.120515405	0.066	0.029	1.85E-11	1.4
CNOT4	7.75E-16	0.176859164	0.427	0.322	1.87E-11	1.4
DMD	7.78E-16	0.207298661	0.554	0.442	1.88E-11	1.4
NFX1	7.84E-16	0.144064397	0.22	0.144	1.89E-11	1.4
NSMCE2	8.25E-16	0.165627106	0.509	0.397	1.99E-11	1.4
SNX3	8.35E-16	0.155707773	0.25	0.17	2.01E-11	1.4
SCGB2A2	8.69E-16	-1.744774555	0.141	0.207	2.10E-11	1.4
LINC00869	8.80E-16	0.140461444	0.085	0.042	2.12E-11	1.4
MVP	8.90E-16	0.128428971	0.105	0.056	2.15E-11	1.4
LRCH1	9.16E-16	0.217084654	0.429	0.326	2.21E-11	1.4
ZRANB2	9.29E-16	0.187714253	0.416	0.312	2.24E-11	1.4
LINC00853	9.54E-16	0.108874076	0.056	0.023	2.30E-11	1.4
CETN3	9.97E-16	0.124268565	0.077	0.036	2.40E-11	1.4
CCDC71L	1.01E-15	0.106612874	0.069	0.03	2.44E-11	1.4
MAGI3	1.02E-15	0.162918544	0.243	0.165	2.45E-11	1.4
RSL1D1	1.05E-15	-0.33061843	0.621	0.633	2.54E-11	1.4
LINC01515	1.06E-15	0.130246102	0.063	0.027	2.56E-11	1.4
ZMYND11	1.08E-15	0.14236296	0.158	0.096	2.61E-11	1.4
ARL17B	1.08E-15	0.190354487	0.117	0.064	2.61E-11	1.4
HIPK3	1.09E-15	0.160202138	0.241	0.163	2.63E-11	1.4
MBD2	1.16E-15	-0.387673432	0.233	0.301	2.79E-11	1.4
EIF4E2	1.20E-15	0.163256871	0.481	0.368	2.89E-11	1.4
YTHDC1	1.21E-15	-0.366680818	0.231	0.3	2.92E-11	1.4
GTF3C3	1.22E-15	0.126813847	0.103	0.055	2.95E-11	1.4
S100PBP	1.24E-15	0.175552271	0.159	0.097	2.99E-11	1.4
ICAM1	1.24E-15	0.142842818	0.215	0.139	3.00E-11	1.4
SMARCA4	1.26E-15	0.170883406	0.193	0.124	3.05E-11	1.4
TARS	1.30E-15	-0.290500729	0.144	0.214	3.13E-11	1.4
KIAA1958	1.43E-15	0.16611695	0.134	0.078	3.46E-11	1.4

TBL1X	1.51E-15	0.183268653	0.22	0.146	3.63E-11	1.4
LAMA1	1.51E-15	0.106006412	0.052	0.02	3.64E-11	1.4
ZNF146	1.53E-15	0.143849932	0.164	0.102	3.69E-11	1.4
HOMER2	1.55E-15	0.200901029	0.277	0.194	3.75E-11	1.4
SNX9	1.58E-15	-0.384997563	0.407	0.466	3.80E-11	1.4
LRSAM1	1.58E-15	0.161864145	0.162	0.099	3.82E-11	1.4
RAB28	1.64E-15	0.122895153	0.081	0.039	3.96E-11	1.4
SEL1L	1.68E-15	-0.26270298	0.052	0.105	4.05E-11	1.4
KCTD7	1.73E-15	0.167451441	0.176	0.111	4.16E-11	1.4
QRSL1	1.75E-15	0.128767535	0.108	0.058	4.21E-11	1.4
CALCOCO1	1.76E-15	0.130800302	0.119	0.066	4.23E-11	1.4
RALGDS	1.77E-15	0.122155206	0.111	0.06	4.26E-11	1.4
FANCC	1.81E-15	0.158076788	0.178	0.112	4.37E-11	1.4
PAK1	1.89E-15	0.133634272	0.248	0.169	4.55E-11	1.4
TRIP11	1.92E-15	0.157575662	0.205	0.134	4.64E-11	1.4
IRAK4	1.99E-15	0.116131621	0.062	0.026	4.79E-11	1.4
ACAP2	2.00E-15	0.205920445	0.207	0.136	4.83E-11	1.4
CTTNBP2NI	2.03E-15	-0.365049	0.233	0.301	4.88E-11	1.4
SLF2	2.08E-15	0.133887307	0.066	0.029	5.03E-11	1.4
RARS	2.09E-15	0.18619365	0.356	0.262	5.05E-11	1.4
SLC38A9	2.18E-15	0.174804441	0.094	0.049	5.26E-11	1.4
MRPS14	2.21E-15	0.136402908	0.214	0.141	5.32E-11	1.4
TOR1AIP2	2.21E-15	-0.341004547	0.189	0.257	5.33E-11	1.4
RPL6	2.22E-15	0.123925311	0.967	0.969	5.35E-11	1.4
PEX2	2.28E-15	0.141716927	0.137	0.081	5.50E-11	1.4
RBMX	2.30E-15	0.157417329	0.31	0.223	5.55E-11	1.4
CTNND1	2.36E-15	-0.334628263	0.269	0.337	5.69E-11	1.4
TBC1D9	2.43E-15	0.189034926	0.286	0.202	5.85E-11	1.4
PTPRA	2.43E-15	0.18059686	0.202	0.132	5.86E-11	1.4
SLC30A6	2.59E-15	0.13994393	0.091	0.046	6.24E-11	1.4
MINK1	2.79E-15	0.194220307	0.191	0.124	6.74E-11	1.4
NUP214	2.81E-15	0.152878692	0.115	0.064	6.78E-11	1.4
IWS1	2.85E-15	0.156792526	0.202	0.131	6.86E-11	1.4
GPAT3	2.86E-15	-0.266151976	0.047	0.098	6.90E-11	1.4
GFOD2	3.03E-15	0.12227948	0.073	0.034	7.30E-11	1.4
CUL1	3.30E-15	0.166032465	0.153	0.093	7.95E-11	1.4
TBC1D22B	3.32E-15	-0.312685618	0.135	0.203	8.00E-11	1.4
CTDSP2	3.47E-15	0.156074309	0.247	0.168	8.36E-11	1.4
GCC2	3.52E-15	0.176063413	0.398	0.301	8.48E-11	1.4
CNOT6L	3.63E-15	0.146442242	0.124	0.071	8.74E-11	1.4
TRIB2	3.79E-15	0.131811149	0.088	0.045	9.13E-11	1.4
MDM4	3.89E-15	0.217586553	0.336	0.248	9.38E-11	1.4
PML	3.89E-15	0.171115059	0.16	0.099	9.39E-11	1.4
LINC00907	3.90E-15	0.112941003	0.05	0.019	9.40E-11	1.4
TUBB6	4.03E-15	-0.170538615	0.023	0.066	9.71E-11	1.4
RCSD1	4.26E-15	0.110320237	0.038	0.012	1.03E-10	1.4
PPIP5K1	4.34E-15	0.133632602	0.075	0.036	1.05E-10	1.4

RSRC1	4.44E-15	0.151650562	0.325	0.233	1.07E-10	1.4
TRAFD1	4.55E-15	0.160089493	0.135	0.079	1.10E-10	1.4
ADAM17	4.69E-15	-0.382510199	0.379	0.437	1.13E-10	1.4
ADM	4.82E-15	-0.14447985	0.003	0.033	1.16E-10	1.4
CDC42EP1	4.88E-15	0.198310647	0.088	0.045	1.18E-10	1.4
SH3RF1	5.03E-15	-0.336467111	0.187	0.26	1.21E-10	1.4
MPV17	5.03E-15	0.113461141	0.073	0.034	1.21E-10	1.4
SLCO3A1	5.24E-15	0.180744856	0.319	0.231	1.26E-10	1.4
ZBTB44	5.43E-15	0.166212906	0.149	0.091	1.31E-10	1.4
VNN2	5.53E-15	0.132904418	0.065	0.029	1.33E-10	1.4
ZBTB10	5.76E-15	0.159360101	0.223	0.15	1.39E-10	1.4
SPATS2	5.82E-15	0.175968438	0.153	0.094	1.40E-10	1.4
AHI1	5.85E-15	0.220721244	0.265	0.187	1.41E-10	1.4
S100B	6.22E-15	-0.21828314	0.022	0.063	1.50E-10	1.4
PTPRE	6.39E-15	0.156167163	0.179	0.114	1.54E-10	1.4
KLF9	6.50E-15	0.164847797	0.177	0.113	1.57E-10	1.4
TMEM14C	6.63E-15	0.129703743	0.132	0.077	1.60E-10	1.4
IRAK1BP1	6.85E-15	0.163299599	0.11	0.061	1.65E-10	1.4
QKI	6.87E-15	-0.486046073	0.52	0.542	1.66E-10	1.4
MGME1	6.93E-15	0.127436123	0.1	0.054	1.67E-10	1.4
IFT74	6.95E-15	0.129866195	0.083	0.041	1.68E-10	1.4
PHF11	6.97E-15	0.143535899	0.12	0.068	1.68E-10	1.4
PSMF1	7.38E-15	0.142502022	0.134	0.079	1.78E-10	1.4
SENP6	7.42E-15	0.154288225	0.407	0.306	1.79E-10	1.4
CASP8AP2	7.44E-15	0.11469506	0.078	0.038	1.79E-10	1.4
CLTA	7.49E-15	0.138336606	0.213	0.141	1.81E-10	1.4
KRT23	7.50E-15	-0.481252464	0.193	0.26	1.81E-10	1.4
ANXA7	7.56E-15	0.163691005	0.363	0.27	1.82E-10	1.4
HIVEP1	7.57E-15	0.142579036	0.213	0.141	1.83E-10	1.4
TXNDC16	7.69E-15	0.113203919	0.067	0.03	1.85E-10	1.4
AL592183.:	7.80E-15	0.152123002	0.126	0.073	1.88E-10	1.4
PDE4DIP	7.82E-15	0.11448048	0.1	0.053	1.89E-10	1.4
FOXP2	8.11E-15	0.11144881	0.056	0.023	1.95E-10	1.4
AKAP12	8.16E-15	-0.418736142	0.011	0.046	1.97E-10	1.4
EIF3I	8.98E-15	-0.280338323	0.649	0.665	2.17E-10	1.4
MON2	9.16E-15	0.128704199	0.324	0.234	2.21E-10	1.4
NMT1	9.88E-15	0.153825942	0.247	0.172	2.38E-10	1.4
MYO9A	9.91E-15	0.173154763	0.365	0.269	2.39E-10	1.4
RP5-1180E	1.02E-14	0.114066383	0.064	0.029	2.46E-10	1.4
RAB24	1.06E-14	0.113280959	0.058	0.025	2.55E-10	1.4
RAB12	1.11E-14	0.184015269	0.254	0.177	2.67E-10	1.4
CPSF2	1.11E-14	0.130261554	0.077	0.037	2.67E-10	1.4
GABRP	1.11E-14	0.162076736	0.628	0.522	2.67E-10	1.4
RAN	1.17E-14	-0.301530913	0.591	0.608	2.81E-10	1.4
C6orf89	1.24E-14	0.12075063	0.118	0.067	2.99E-10	1.4
AHCYL1	1.24E-14	0.158295688	0.353	0.258	3.00E-10	1.4
MCTP1	1.24E-14	0.183289935	0.148	0.09	3.00E-10	1.4

HIVEP2	1.28E-14	-0.418803732	0.317	0.385	3.08E-10	1.4
RASGEF1C	1.30E-14	-0.235588009	0.039	0.087	3.13E-10	1.4
FBLIM1	1.31E-14	-0.255530158	0.1	0.162	3.16E-10	1.4
LUC7L	1.32E-14	0.150316763	0.137	0.082	3.19E-10	1.4
TUBA1C	1.36E-14	-0.327968435	0.31	0.375	3.27E-10	1.4
DYM	1.36E-14	0.136595918	0.311	0.223	3.27E-10	1.4
PBX3	1.40E-14	0.148002837	0.077	0.038	3.38E-10	1.4
ROPN1B	1.45E-14	-0.311036396	0.089	0.148	3.50E-10	1.4
CITED2	1.47E-14	0.128355398	0.082	0.041	3.55E-10	1.4
C2orf68	1.53E-14	0.128843868	0.099	0.053	3.70E-10	1.4
NDUFAF7	1.62E-14	0.123244091	0.099	0.053	3.90E-10	1.4
RP11-418J1	1.62E-14	0.139696645	0.071	0.033	3.91E-10	1.4
COG7	1.67E-14	0.158117444	0.115	0.065	4.04E-10	1.4
ATAD2	1.70E-14	0.129670745	0.103	0.056	4.10E-10	1.4
MAPKAPK2	1.74E-14	-0.248299003	0.084	0.142	4.20E-10	1.4
RP11-577H	1.76E-14	0.138485218	0.104	0.057	4.24E-10	1.4
MLLT3	1.81E-14	0.182071082	0.201	0.134	4.36E-10	1.4
NSUN4	1.91E-14	0.106505038	0.116	0.066	4.60E-10	1.4
BPGM	1.94E-14	0.179101094	0.217	0.146	4.68E-10	1.4
ZNF714	1.96E-14	0.109842308	0.06	0.027	4.72E-10	1.4
C2CD5	1.96E-14	0.102148588	0.049	0.02	4.73E-10	1.4
SLC25A6	1.97E-14	0.222543851	0.186	0.122	4.75E-10	1.4
ALOX12-AS	2.01E-14	0.132042885	0.104	0.057	4.85E-10	1.4
CBFA2T2	2.02E-14	0.17030593	0.188	0.124	4.86E-10	1.4
SPICE1	2.13E-14	0.130857973	0.082	0.041	5.13E-10	1.4
HUWE1	2.14E-14	0.122948896	0.413	0.309	5.16E-10	1.4
TYMP	2.15E-14	0.121089207	0.052	0.021	5.19E-10	1.4
S100A6	2.19E-14	-0.794251537	0.423	0.481	5.28E-10	1.4
STYXL1	2.19E-14	0.12277225	0.092	0.048	5.28E-10	1.4
STRN	2.30E-14	-0.375772739	0.355	0.412	5.54E-10	1.4
RABGGTB	2.32E-14	-0.233527845	0.055	0.107	5.59E-10	1.4
GLYATL2	2.34E-14	0.113009219	0.11	0.061	5.65E-10	1.4
RANBP10	2.38E-14	0.148553219	0.09	0.046	5.73E-10	1.4
RP11-141M	2.39E-14	0.182514764	0.101	0.055	5.77E-10	1.4
FDFT1	2.46E-14	0.218149475	0.408	0.314	5.93E-10	1.4
CCDC130	2.48E-14	0.106815393	0.063	0.028	5.99E-10	1.4
CDC26	2.66E-14	0.15135939	0.282	0.2	6.41E-10	1.4
YWHAG	2.69E-14	-0.309545559	0.182	0.249	6.48E-10	1.4
CD164	2.77E-14	0.160785355	0.236	0.161	6.68E-10	1.4
SERAC1	2.88E-14	-0.259026985	0.044	0.092	6.94E-10	1.4
RAB30-AS1	2.89E-14	0.161267371	0.14	0.085	6.98E-10	1.4
DECR1	2.90E-14	0.137818838	0.196	0.127	6.98E-10	1.4
CACHD1	2.92E-14	0.163205865	0.13	0.077	7.05E-10	1.4
FAM172A	3.32E-14	-0.359071172	0.737	0.752	8.01E-10	1.4
SEL1L3	3.46E-14	0.125046582	0.086	0.045	8.35E-10	1.4
CLIC1	3.53E-14	-0.304826742	0.571	0.6	8.52E-10	1.4
UGCG	3.55E-14	0.207755685	0.398	0.305	8.57E-10	1.4

CEP95	3.82E-14	0.139708696	0.202	0.133	9.20E-10	1.4
RAB13	4.02E-14	0.151085608	0.192	0.126	9.69E-10	1.4
FBXO38	4.31E-14	0.104683206	0.086	0.044	1.04E-09	1.4
ATRNL1	4.49E-14	0.18028237	0.114	0.065	1.08E-09	1.4
UBE2N	4.57E-14	-0.319098037	0.17	0.236	1.10E-09	1.4
CEP112	4.66E-14	0.105828735	0.069	0.033	1.12E-09	1.4
GRAMD1C	4.68E-14	0.128550842	0.074	0.036	1.13E-09	1.4
RP11-513G	4.78E-14	0.102076878	0.038	0.013	1.15E-09	1.4
ACBD6	4.92E-14	0.127333076	0.105	0.059	1.19E-09	1.4
LARS2	4.93E-14	0.130388721	0.074	0.036	1.19E-09	1.4
FBXW11	4.98E-14	0.129776213	0.396	0.298	1.20E-09	1.4
RP11-286E	5.02E-14	-0.205069993	0.03	0.073	1.21E-09	1.4
DNAJC16	5.02E-14	0.117951427	0.088	0.046	1.21E-09	1.4
SESN2	5.16E-14	-0.258140772	0.066	0.119	1.25E-09	1.4
SLC44A1	5.58E-14	0.13316854	0.131	0.078	1.34E-09	1.4
STEAP3	6.22E-14	0.13606255	0.071	0.035	1.50E-09	1.4
ATP6V1G1	6.27E-14	0.130218016	0.438	0.334	1.51E-09	1.4
PDK1	6.32E-14	-0.268540803	0.077	0.133	1.52E-09	1.4
XKR6	6.32E-14	0.178372862	0.226	0.155	1.52E-09	1.4
IQGAP1	6.35E-14	-0.315947284	0.389	0.45	1.53E-09	1.4
CEP152	6.42E-14	0.102742665	0.05	0.021	1.55E-09	1.4
MFF	6.81E-14	0.123079841	0.124	0.073	1.64E-09	1.4
PDXDC1	6.83E-14	0.139173396	0.369	0.273	1.65E-09	1.4
SARNP	6.87E-14	-0.347696815	0.179	0.243	1.66E-09	1.4
KIZ	6.94E-14	0.108087033	0.059	0.026	1.67E-09	1.4
CFAP44	6.95E-14	0.104699913	0.055	0.023	1.68E-09	1.4
COX7A2	7.02E-14	-0.217606619	0.805	0.797	1.69E-09	1.4
MBTD1	7.08E-14	0.120368435	0.224	0.152	1.71E-09	1.4
CSPP1	7.23E-14	0.171182736	0.172	0.111	1.74E-09	1.4
GLMN	7.33E-14	0.133803344	0.086	0.045	1.77E-09	1.4
CDK12	7.54E-14	0.17839881	0.142	0.088	1.82E-09	1.4
FAF2	7.65E-14	0.179434995	0.223	0.154	1.85E-09	1.4
STMN1	7.80E-14	-0.335391628	0.147	0.212	1.88E-09	1.4
WWP1	8.21E-14	0.171011625	0.243	0.17	1.98E-09	1.4
PCID2	8.22E-14	0.136778502	0.158	0.1	1.98E-09	1.4
PIK3IP1-AS	8.24E-14	0.130293467	0.081	0.041	1.99E-09	1.4
EPB42	8.53E-14	-0.259306794	0.915	0.931	2.06E-09	1.4
DHRSX	8.57E-14	0.173965997	0.283	0.203	2.07E-09	1.4
DOPEY1	8.69E-14	0.134917139	0.17	0.109	2.10E-09	1.4
DDX58	8.86E-14	0.125606588	0.114	0.065	2.14E-09	1.4
GRB14	8.93E-14	-0.371762339	0.53	0.573	2.15E-09	1.4
TRAPPC13	9.30E-14	0.116301892	0.091	0.048	2.24E-09	1.4
STEAP1B	9.68E-14	-0.256308751	0.947	0.956	2.34E-09	1.4
ZBTB38	9.90E-14	-0.293674141	0.269	0.338	2.39E-09	1.4
DENND5A	1.02E-13	-0.414101803	0.315	0.37	2.45E-09	1.4
ZBTB46	1.04E-13	0.106400076	0.099	0.054	2.51E-09	1.4
DLG2	1.04E-13	0.155303309	0.22	0.149	2.52E-09	1.4

MTM1	1.06E-13	-0.37129548	0.15	0.213	2.56E-09	1.4
AC013461.	1.10E-13	0.127466102	0.268	0.19	2.64E-09	1.4
ENO2	1.10E-13	-0.163938284	0.03	0.073	2.65E-09	1.4
BET1	1.11E-13	0.1436039	0.196	0.13	2.68E-09	1.4
MAP2K1	1.11E-13	-0.324017866	0.163	0.228	2.68E-09	1.4
GNL3	1.14E-13	-0.325120051	0.227	0.294	2.74E-09	1.4
CFL1	1.15E-13	-0.355773472	0.194	0.261	2.78E-09	1.4
IFT81	1.24E-13	0.107042533	0.064	0.03	3.00E-09	1.4
EFNA1	1.29E-13	0.1604206	0.153	0.097	3.10E-09	1.4
CCDC57	1.30E-13	0.143811751	0.099	0.055	3.13E-09	1.4
AVL9	1.30E-13	0.15010562	0.234	0.162	3.13E-09	1.4
N4BP1	1.33E-13	0.154710155	0.245	0.172	3.21E-09	1.4
PDS5A	1.39E-13	0.129240651	0.435	0.335	3.36E-09	1.4
LDLR	1.43E-13	0.172748106	0.22	0.151	3.45E-09	1.4
TACC2	1.43E-13	0.14995042	0.196	0.133	3.46E-09	1.4
BNIP3	1.54E-13	-0.244145707	0.074	0.129	3.72E-09	1.4
ITPR1	1.55E-13	0.117216556	0.068	0.032	3.75E-09	1.4
CFAP161	1.56E-13	0.111620555	0.058	0.026	3.75E-09	1.4
SUB1	1.56E-13	-0.268331363	0.75	0.755	3.75E-09	1.4
PTPN14	1.61E-13	-0.445585203	0.424	0.469	3.88E-09	1.4
NIN	1.66E-13	0.154205288	0.144	0.088	4.01E-09	1.4
RCL1	1.69E-13	0.111101179	0.06	0.027	4.08E-09	1.4
EPC2	1.70E-13	0.114021285	0.096	0.053	4.09E-09	1.4
KHDRBS3	1.86E-13	-0.264718356	0.083	0.139	4.48E-09	1.4
B2M	1.88E-13	0.195693726	0.981	0.987	4.52E-09	1.4
ZNF440	1.89E-13	0.105341965	0.057	0.025	4.55E-09	1.4
CCDC149	1.99E-13	0.113263193	0.054	0.023	4.79E-09	1.4
MEF2A	2.06E-13	-0.437417641	0.351	0.401	4.96E-09	1.4
CEBPZOS	2.08E-13	0.11683634	0.19	0.126	5.01E-09	1.4
AAMDC	2.09E-13	0.171765972	0.218	0.15	5.03E-09	1.4
PKNOX1	2.10E-13	0.115046614	0.067	0.032	5.05E-09	1.4
TMEM27	2.24E-13	-0.16062046	0.019	0.056	5.40E-09	1.4
TTF1	2.24E-13	0.12316523	0.167	0.107	5.40E-09	1.4
ARHGEF6	2.28E-13	0.127921871	0.093	0.05	5.49E-09	1.4
MMP24-AS	2.31E-13	0.106914081	0.07	0.034	5.58E-09	1.4
PPID	2.47E-13	0.118143754	0.113	0.065	5.96E-09	1.4
PPFIBP2	2.49E-13	0.117964321	0.138	0.084	6.01E-09	1.4
CBX1	2.50E-13	-0.300493605	0.099	0.155	6.02E-09	1.4
LRP10	2.53E-13	0.127642047	0.151	0.096	6.10E-09	1.4
CFLAR-AS1	2.62E-13	0.116092411	0.122	0.072	6.31E-09	1.4
SUMF2	2.63E-13	0.127207204	0.103	0.058	6.34E-09	1.4
ZBTB7C	2.83E-13	0.127616313	0.064	0.03	6.83E-09	1.4
KIF16B	2.97E-13	0.114053791	0.122	0.072	7.17E-09	1.4
SUPT3H	2.97E-13	0.174428106	0.208	0.142	7.17E-09	1.4
PPARA	3.07E-13	0.119807257	0.114	0.066	7.39E-09	1.4
RHOU	3.10E-13	0.111327751	0.065	0.031	7.47E-09	1.4
CTD-2369P	3.66E-13	0.109485009	0.048	0.02	8.82E-09	1.4

MT-CO2	3.73E-13	0.140040734	0.982	0.986	8.99E-09	1.4
FAM151B	3.73E-13	0.130394709	0.076	0.038	8.99E-09	1.4
SYNPO2	3.79E-13	0.315873603	0.257	0.185	9.14E-09	1.4
SNRPD2	3.84E-13	-0.264723642	0.75	0.743	9.27E-09	1.4
LINC00894	3.97E-13	0.128639356	0.096	0.053	9.58E-09	1.4
BTBD3	4.03E-13	0.165229985	0.152	0.097	9.71E-09	1.4
UPF2	4.06E-13	0.123540267	0.349	0.262	9.79E-09	1.4
PRMT2	4.12E-13	0.133499832	0.171	0.112	9.93E-09	1.4
RPA3	4.14E-13	0.114728995	0.165	0.106	9.97E-09	1.4
RPL27	4.25E-13	-0.135677443	0.948	0.957	1.02E-08	1.4
EIF3J-AS1	4.29E-13	0.103339421	0.095	0.052	1.03E-08	1.4
RPS6KC1	4.37E-13	0.150379698	0.125	0.075	1.05E-08	1.4
POMP	4.40E-13	-0.271393907	0.645	0.666	1.06E-08	1.4
RAD51C	4.49E-13	0.10410824	0.08	0.041	1.08E-08	1.4
MFSD14C	4.64E-13	0.127332588	0.275	0.197	1.12E-08	1.4
MBOAT2	4.69E-13	0.152946574	0.159	0.102	1.13E-08	1.4
C5orf45	4.78E-13	0.131408411	0.068	0.033	1.15E-08	1.4
NDRG1	5.02E-13	-0.561709871	0.426	0.464	1.21E-08	1.4
PSMB8	5.14E-13	0.1299567	0.155	0.099	1.24E-08	1.4
SARS	5.20E-13	-0.262414664	0.158	0.221	1.25E-08	1.4
TMEM176A	5.22E-13	0.138187582	0.081	0.043	1.26E-08	1.4
EFCAB2	5.44E-13	0.13820498	0.071	0.036	1.31E-08	1.4
NRG1	5.57E-13	0.153734796	0.17	0.111	1.34E-08	1.4
XRCC4	5.57E-13	0.13126519	0.081	0.042	1.34E-08	1.4
DNAJC6	5.83E-13	-0.267896739	0.063	0.114	1.41E-08	1.4
ATXN10	5.95E-13	0.147379186	0.207	0.141	1.44E-08	1.4
ZNF91	6.03E-13	0.135510874	0.194	0.13	1.45E-08	1.4
NCOA6	6.08E-13	0.161898648	0.201	0.137	1.47E-08	1.4
HIPK2	6.16E-13	0.122821572	0.3	0.219	1.49E-08	1.4
SPAG9	6.20E-13	-0.375918813	0.297	0.354	1.50E-08	1.4
PHF20L1	6.24E-13	0.161531515	0.176	0.116	1.50E-08	1.4
ANO6	6.26E-13	-0.381158961	0.312	0.368	1.51E-08	1.4
LRRC28	6.31E-13	0.16184803	0.102	0.058	1.52E-08	1.4
EP400	6.51E-13	0.126907847	0.118	0.07	1.57E-08	1.4
SRGN	6.96E-13	0.31699709	0.057	0.026	1.68E-08	1.4
UBE3B	7.02E-13	0.101961752	0.063	0.03	1.69E-08	1.4
TBC1D4	7.13E-13	0.157739313	0.187	0.126	1.72E-08	1.4
MORC3	7.28E-13	-0.330039137	0.18	0.242	1.75E-08	1.4
KIAA1468	7.44E-13	0.114818267	0.152	0.096	1.79E-08	1.4
BCL2	7.49E-13	0.176684592	0.173	0.113	1.81E-08	1.4
RPS24	7.71E-13	-0.1346204	0.975	0.985	1.86E-08	1.4
MNAT1	7.74E-13	0.158694676	0.391	0.301	1.87E-08	1.4
KIAA2026	8.05E-13	0.15085591	0.192	0.13	1.94E-08	1.4
SMIM8	8.09E-13	0.141222576	0.126	0.077	1.95E-08	1.4
NOL3	8.19E-13	0.12286985	0.078	0.041	1.97E-08	1.4
PRNP	8.45E-13	-0.265855702	0.093	0.148	2.04E-08	1.4
RBM3	8.98E-13	-0.323415623	0.478	0.513	2.16E-08	1.4

UBE2L3	9.58E-13	-0.299355582	0.42	0.466	2.31E-08	1.4
IFIH1	1.01E-12	0.119779666	0.139	0.086	2.44E-08	1.4
KRT81	1.02E-12	-0.3126976	0.011	0.042	2.47E-08	1.4
SLC35F5	1.07E-12	0.13109147	0.141	0.088	2.59E-08	1.4
BTF3	1.13E-12	-0.218897779	0.937	0.95	2.72E-08	1.4
DDX17	1.15E-12	0.18074151	0.508	0.403	2.79E-08	1.4
RNF180	1.16E-12	0.123935166	0.059	0.027	2.80E-08	1.4
SF3B3	1.17E-12	0.154457666	0.267	0.193	2.83E-08	1.4
GLYR1	1.20E-12	0.136391866	0.16	0.104	2.89E-08	1.4
RPGR	1.21E-12	0.115276411	0.085	0.046	2.91E-08	1.4
RP11-701H	1.24E-12	0.140778368	0.077	0.04	2.98E-08	1.4
ARGLU1	1.24E-12	0.170282461	0.227	0.159	2.99E-08	1.4
EPPK1	1.28E-12	-0.187151143	0.032	0.072	3.09E-08	1.4
ACADSB	1.29E-12	0.100787524	0.05	0.022	3.10E-08	1.4
TFCP2L1	1.35E-12	-0.405651001	0.11	0.166	3.24E-08	1.4
ABCA3	1.38E-12	0.113780247	0.065	0.032	3.32E-08	1.4
ZFAND5	1.39E-12	0.177851416	0.408	0.321	3.34E-08	1.4
ZNF76	1.41E-12	0.124876205	0.052	0.023	3.39E-08	1.4
SCAF11	1.41E-12	0.164269793	0.359	0.272	3.40E-08	1.4
SGCE	1.45E-12	0.140839166	0.097	0.055	3.49E-08	1.4
WDR19	1.54E-12	0.13554061	0.111	0.065	3.71E-08	1.4
MCCC2	1.59E-12	0.127753843	0.157	0.102	3.83E-08	1.4
ZNF529-AS	1.62E-12	0.107620882	0.08	0.042	3.90E-08	1.4
TCF7L1	1.62E-12	0.140869068	0.405	0.311	3.90E-08	1.4
PPIA	1.64E-12	-0.199077757	0.879	0.855	3.95E-08	1.4
UBAP1	1.70E-12	-0.327500114	0.295	0.351	4.09E-08	1.4
GNPTAB	1.72E-12	0.147940006	0.151	0.097	4.15E-08	1.4
TCAIM	1.74E-12	0.12118997	0.095	0.053	4.20E-08	1.4
NRDC	1.76E-12	0.131225725	0.228	0.158	4.25E-08	1.4
SPCS2	1.84E-12	-0.314465264	0.271	0.331	4.45E-08	1.4
INTS3	1.88E-12	0.106261957	0.093	0.052	4.53E-08	1.4
KIAA1551	1.89E-12	0.134240362	0.177	0.118	4.55E-08	1.4
TAF4B	1.89E-12	0.139727652	0.099	0.056	4.56E-08	1.4
FGF13	1.94E-12	-0.447226849	0.173	0.234	4.68E-08	1.4
FUT9	2.00E-12	0.112427381	0.055	0.025	4.82E-08	1.4
DUSP6	2.03E-12	0.102455662	0.071	0.036	4.89E-08	1.4
CLMN	2.06E-12	-0.347660331	0.572	0.597	4.97E-08	1.4
NRIP1	2.13E-12	0.114278091	0.168	0.109	5.14E-08	1.4
HAGH	2.14E-12	0.105153275	0.055	0.025	5.15E-08	1.4
SIL1	2.24E-12	0.175212229	0.147	0.094	5.41E-08	1.4
KRT16	2.25E-12	-0.182375394	0.004	0.028	5.42E-08	1.4
CISD1	2.32E-12	0.138365346	0.28	0.205	5.59E-08	1.4
ABLIM1	2.35E-12	0.166140368	0.321	0.242	5.66E-08	1.4
EPG5	2.35E-12	0.11489131	0.127	0.078	5.66E-08	1.4
SYTL3	2.35E-12	0.122941379	0.092	0.052	5.66E-08	1.4
SH3RF2	2.39E-12	-0.120591118	0.004	0.029	5.76E-08	1.4
PPA1	2.47E-12	0.161314501	0.32	0.24	5.96E-08	1.4

CTSC	2.56E-12	0.121852379	0.084	0.045	6.18E-08	1.4
NEMF	2.57E-12	0.146568077	0.39	0.299	6.19E-08	1.4
TBC1D1	2.57E-12	0.189638776	0.243	0.173	6.19E-08	1.4
COG6	2.58E-12	0.105684889	0.053	0.024	6.22E-08	1.4
HPS4	2.66E-12	0.117448995	0.069	0.034	6.41E-08	1.4
HERC2	2.80E-12	0.141375074	0.117	0.071	6.75E-08	1.4
FAM196A	2.81E-12	0.11737608	0.099	0.057	6.78E-08	1.4
SUGP2	2.81E-12	0.112015967	0.082	0.044	6.78E-08	1.4
MFGE8	2.95E-12	0.230385425	0.237	0.17	7.11E-08	1.4
LYRM2	3.05E-12	0.131203806	0.149	0.096	7.37E-08	1.4
OLFM4	3.06E-12	0.28152371	0.064	0.031	7.38E-08	1.4
ATXN7	3.23E-12	0.125439845	0.192	0.131	7.80E-08	1.4
ZCCHC7	3.29E-12	0.14536665	0.467	0.367	7.94E-08	1.4
TTN-AS1	3.31E-12	0.128961938	0.086	0.047	7.97E-08	1.4
TMLHE	3.32E-12	0.103350092	0.088	0.048	8.02E-08	1.4
MARK2	3.35E-12	0.142104158	0.131	0.082	8.08E-08	1.4
ZCCHC9	3.38E-12	0.104316043	0.096	0.054	8.15E-08	1.4
SMARCAD1	3.41E-12	0.116303219	0.1	0.057	8.21E-08	1.4
HMBOX1	3.42E-12	0.133910939	0.275	0.2	8.24E-08	1.4
NEK1	3.69E-12	0.14923476	0.121	0.074	8.91E-08	1.4
RBL2	3.72E-12	0.114240011	0.135	0.084	8.97E-08	1.4
RP11-277P	3.76E-12	0.104128453	0.104	0.06	9.07E-08	1.4
TSG101	3.98E-12	0.126595476	0.227	0.159	9.60E-08	1.4
AEBP2	4.16E-12	0.116212936	0.303	0.222	1.00E-07	1.4
RHPN2	4.21E-12	0.13289551	0.35	0.264	1.02E-07	1.4
EIF2A	4.22E-12	-0.310128902	0.404	0.444	1.02E-07	1.4
ELF2	4.22E-12	0.182555512	0.388	0.301	1.02E-07	1.4
C5orf42	4.28E-12	0.101356695	0.073	0.038	1.03E-07	1.4
TRIM44	4.34E-12	0.150268342	0.193	0.133	1.05E-07	1.4
TNS3	4.35E-12	0.163735554	0.114	0.069	1.05E-07	1.4
SFI1	4.46E-12	0.123751547	0.082	0.044	1.07E-07	1.4
RP11-382A	4.51E-12	0.102831943	0.08	0.042	1.09E-07	1.4
CAPN7	4.60E-12	0.104193739	0.178	0.119	1.11E-07	1.4
DAB1	4.64E-12	0.12086135	0.094	0.053	1.12E-07	1.4
UBR3	4.73E-12	0.136333192	0.255	0.182	1.14E-07	1.4
ANKRD37	4.88E-12	-0.186305266	0.036	0.076	1.18E-07	1.4
SDHC	5.01E-12	0.129079732	0.196	0.133	1.21E-07	1.4
RAB11FIP2	5.12E-12	0.106703044	0.062	0.03	1.23E-07	1.4
PAFAH1B2	5.22E-12	-0.285223104	0.191	0.248	1.26E-07	1.4
KIZ-AS1	5.27E-12	-0.180593364	0.878	0.895	1.27E-07	1.4
GCA	5.41E-12	0.113599136	0.097	0.056	1.30E-07	1.4
HIP1	5.41E-12	0.148224913	0.128	0.079	1.30E-07	1.4
GTPBP10	5.59E-12	0.14613284	0.139	0.088	1.35E-07	1.4
DNAJC11	5.63E-12	0.102302079	0.078	0.041	1.36E-07	1.4
MT-ND6	5.64E-12	0.154398901	0.209	0.146	1.36E-07	1.4
SIRPA	5.66E-12	0.121138676	0.094	0.054	1.36E-07	1.4
SLC12A8	5.67E-12	0.100205184	0.072	0.037	1.37E-07	1.4

SSH2	5.82E-12	0.135417518	0.406	0.315	1.40E-07	1.4
TSTD2	5.85E-12	0.11007498	0.083	0.045	1.41E-07	1.4
CNTNAP3B	6.11E-12	0.146027853	0.152	0.099	1.47E-07	1.4
CEP290	6.34E-12	0.133657224	0.102	0.06	1.53E-07	1.4
DAD1	6.36E-12	-0.308981791	0.499	0.525	1.53E-07	1.4
BCL2L11	6.46E-12	0.114927565	0.085	0.047	1.56E-07	1.4
GLUD1	6.52E-12	-0.321228472	0.122	0.178	1.57E-07	1.4
ANGPTL1	6.65E-12	-0.232589034	0.044	0.086	1.60E-07	1.4
PPP1R13B	6.91E-12	0.109626972	0.176	0.118	1.67E-07	1.4
XPNPEP3	7.19E-12	0.114371131	0.1	0.058	1.73E-07	1.4
ANKRD49	7.78E-12	0.10823647	0.092	0.052	1.88E-07	1.4
GALNT3	7.81E-12	-0.300663858	0.168	0.228	1.88E-07	1.4
CASP7	8.22E-12	0.11736325	0.214	0.149	1.98E-07	1.4
RND3	8.30E-12	-0.377401545	0.259	0.316	2.00E-07	1.4
TERF2IP	8.47E-12	0.1449542	0.189	0.13	2.04E-07	1.4
TRIP4	8.79E-12	0.125497365	0.073	0.038	2.12E-07	1.4
MRPS31	9.09E-12	0.114727272	0.157	0.103	2.19E-07	1.4
STK38L	9.17E-12	-0.312704465	0.103	0.156	2.21E-07	1.4
SPINT1	9.62E-12	0.163676243	0.165	0.11	2.32E-07	1.4
SNRPE	9.73E-12	-0.250055178	0.598	0.618	2.35E-07	1.4
CBY1	9.88E-12	0.122536442	0.104	0.062	2.38E-07	1.4
ANAPC16	9.89E-12	0.125710164	0.349	0.265	2.38E-07	1.4
CRLF3	9.99E-12	0.150431794	0.167	0.111	2.41E-07	1.4
IRAK2	1.02E-11	0.146224024	0.185	0.126	2.46E-07	1.4
HADH	1.04E-11	-0.334405278	0.162	0.22	2.50E-07	1.4
CLSTN3	1.04E-11	0.15779409	0.082	0.045	2.51E-07	1.4
NMB	1.08E-11	-0.127954106	0.008	0.035	2.60E-07	1.4
ECHDC1	1.09E-11	0.161155948	0.333	0.255	2.64E-07	1.4
XIST	1.18E-11	0.141887156	0.766	0.659	2.84E-07	1.4
STXBP5	1.20E-11	0.107079713	0.133	0.084	2.88E-07	1.4
STAT2	1.22E-11	0.139028695	0.13	0.082	2.95E-07	1.4
MAPRE2	1.24E-11	0.166148189	0.181	0.123	2.99E-07	1.4
SLTM	1.30E-11	0.161679418	0.316	0.24	3.13E-07	1.4
HIST2H2BF	1.34E-11	0.105489723	0.063	0.032	3.24E-07	1.4
TPRG1	1.35E-11	0.178491833	0.129	0.081	3.25E-07	1.4
NFKBIZ	1.36E-11	-0.246548443	0.803	0.794	3.28E-07	1.4
MED27	1.36E-11	0.123639908	0.09	0.051	3.29E-07	1.4
DNAJC9	1.38E-11	0.106217348	0.088	0.049	3.34E-07	1.4
TECPR2	1.42E-11	0.122544512	0.07	0.036	3.41E-07	1.4
HNRNPU-A	1.43E-11	0.119128605	0.111	0.067	3.45E-07	1.4
KDM2B	1.48E-11	0.113475122	0.076	0.041	3.57E-07	1.4
CD44	1.51E-11	0.124732739	0.556	0.455	3.65E-07	1.4
FCHO2	1.55E-11	0.125244922	0.194	0.134	3.75E-07	1.4
TCF7L2	1.55E-11	0.14530589	0.525	0.418	3.75E-07	1.4
FAM208B	1.56E-11	0.129163854	0.239	0.172	3.76E-07	1.4
ZNF407	1.59E-11	0.113182217	0.153	0.1	3.83E-07	1.4
WDR43	1.59E-11	-0.258689812	0.075	0.123	3.84E-07	1.4

TMEM63A	1.69E-11	0.17308227	0.101	0.06	4.08E-07	1.4
JUP	1.69E-11	-0.295214264	0.177	0.235	4.09E-07	1.4
PRPF40B	1.70E-11	0.135163516	0.058	0.028	4.09E-07	1.4
PON2	1.77E-11	0.101497371	0.086	0.048	4.26E-07	1.4
UPP2	1.77E-11	0.102969114	0.052	0.024	4.27E-07	1.4
RNF19B	1.92E-11	0.130369274	0.219	0.155	4.63E-07	1.4
ZSCAN5A	1.96E-11	0.10035426	0.055	0.026	4.72E-07	1.4
LRRC49	1.98E-11	0.112836889	0.165	0.11	4.77E-07	1.4
HERC3	2.01E-11	-0.332807896	0.117	0.169	4.85E-07	1.4
WDR37	2.06E-11	0.120553429	0.121	0.075	4.96E-07	1.4
C20orf194	2.06E-11	0.164132322	0.207	0.144	4.98E-07	1.4
NUCKS1	2.11E-11	0.143303371	0.37	0.286	5.08E-07	1.4
NT5DC1	2.11E-11	0.123087036	0.102	0.06	5.10E-07	1.4
NCK1	2.17E-11	0.126995539	0.207	0.144	5.24E-07	1.4
LHFP	2.19E-11	0.12314323	0.058	0.028	5.29E-07	1.4
SRGAP3	2.23E-11	0.152151969	0.096	0.056	5.39E-07	1.4
ADRBK2	2.27E-11	0.14123005	0.142	0.092	5.47E-07	1.4
BACE2	2.29E-11	-0.339403404	0.427	0.469	5.52E-07	1.4
RP11-84A1	2.33E-11	-0.144725352	0.018	0.049	5.62E-07	1.4
ABHD2	2.38E-11	-0.245561144	0.066	0.112	5.75E-07	1.4
PDLIM3	2.44E-11	0.14060548	0.091	0.052	5.89E-07	1.4
SLC10A7	2.45E-11	0.132963444	0.088	0.05	5.90E-07	1.4
C16orf72	2.53E-11	-0.23125353	0.104	0.158	6.09E-07	1.4
GBP1	2.54E-11	-0.449140887	0.151	0.205	6.12E-07	1.4
HNRNPK	2.56E-11	-0.294663968	0.446	0.483	6.18E-07	1.4
NASP	2.57E-11	0.129418004	0.23	0.165	6.18E-07	1.4
ODF2L	2.60E-11	0.154205555	0.184	0.127	6.26E-07	1.4
TFAP2A	2.63E-11	0.113332904	0.097	0.057	6.34E-07	1.4
PPP6R2	2.69E-11	0.164421506	0.143	0.093	6.48E-07	1.4
SETDB1	2.71E-11	0.125809781	0.121	0.075	6.54E-07	1.4
ROCK1	2.74E-11	0.117428058	0.259	0.188	6.60E-07	1.4
DNER	2.81E-11	0.162479436	0.103	0.062	6.78E-07	1.4
BCAP29	2.99E-11	0.159538591	0.202	0.141	7.21E-07	1.4
UBLCP1	3.03E-11	0.101506868	0.1	0.059	7.29E-07	1.4
SAR1B	3.07E-11	-0.338619791	0.423	0.467	7.41E-07	1.4
LINGO1	3.08E-11	-0.233345784	0.867	0.89	7.42E-07	1.4
EZH1	3.15E-11	0.122359598	0.134	0.085	7.60E-07	1.4
EP300	3.22E-11	0.122257374	0.187	0.128	7.75E-07	1.4
CEP70	3.29E-11	0.114222172	0.113	0.069	7.94E-07	1.4
ZNHIT3	3.37E-11	0.104982619	0.26	0.19	8.13E-07	1.4
RP11-767I2	3.42E-11	0.105915879	0.023	0.007	8.25E-07	1.4
SPRED1	3.49E-11	0.131614695	0.107	0.065	8.41E-07	1.4
SNU13	3.54E-11	-0.316939598	0.459	0.491	8.55E-07	1.4
DPP10	3.59E-11	0.146963009	0.076	0.041	8.66E-07	1.4
CXorf23	3.61E-11	0.100619787	0.082	0.046	8.71E-07	1.4
KRT17	3.61E-11	-0.217555444	0.01	0.036	8.71E-07	1.4
FYN	3.63E-11	-0.116586317	0.013	0.042	8.75E-07	1.4

NBPF12	3.71E-11	0.117600423	0.093	0.054	8.95E-07	1.4
MTHFD1L	3.92E-11	-0.297525261	0.243	0.302	9.46E-07	1.4
SORL1	3.95E-11	0.104284722	0.104	0.062	9.53E-07	1.4
PSMB7	3.96E-11	-0.297864786	0.571	0.588	9.54E-07	1.4
ATN1	3.98E-11	0.105523405	0.083	0.046	9.59E-07	1.4
PNP	4.00E-11	-0.156423418	0.03	0.066	9.65E-07	1.4
TRIM5	4.09E-11	0.183163258	0.19	0.133	9.86E-07	1.4
ESCO1	4.10E-11	0.118335822	0.134	0.086	9.88E-07	1.4
SNHG12	4.17E-11	-0.190711206	0.039	0.078	1.01E-06	1.4
ITPKB	4.31E-11	0.104720025	0.091	0.052	1.04E-06	1.4
FAM107B	4.40E-11	-0.277363245	0.201	0.26	1.06E-06	1.4
VCL	4.68E-11	-0.282859311	0.28	0.339	1.13E-06	1.4
CDC42SE1	4.76E-11	0.124314817	0.2	0.14	1.15E-06	1.4
ASXL2	4.77E-11	0.111533942	0.109	0.066	1.15E-06	1.4
ERBB2	4.82E-11	0.108526329	0.092	0.053	1.16E-06	1.4
CHIC2	5.10E-11	-0.345863308	0.192	0.245	1.23E-06	1.4
FKBP15	5.28E-11	0.118338674	0.088	0.051	1.27E-06	1.4
KIAA0430	5.40E-11	0.118748778	0.103	0.062	1.30E-06	1.4
KAZN	5.45E-11	0.124387563	0.192	0.133	1.31E-06	1.4
FOPNL	5.48E-11	0.103509687	0.095	0.056	1.32E-06	1.4
PDGFRL	5.61E-11	0.219823814	0.082	0.046	1.35E-06	1.4
UEVLD	5.87E-11	0.119893206	0.092	0.054	1.42E-06	1.4
CRHR1	5.93E-11	0.132235885	0.079	0.044	1.43E-06	1.4
MTF2	5.99E-11	0.110563206	0.267	0.196	1.44E-06	1.4
CSTF3	6.14E-11	0.12529894	0.175	0.12	1.48E-06	1.4
HSPG2	6.18E-11	0.128528268	0.155	0.104	1.49E-06	1.4
EIF1AX	6.43E-11	-0.316156243	0.198	0.252	1.55E-06	1.4
NR3C2	6.49E-11	0.125680873	0.159	0.107	1.57E-06	1.4
CXCL8	6.53E-11	-1.039639064	0.316	0.372	1.57E-06	1.4
SLMAP	6.54E-11	-0.327703004	0.628	0.631	1.58E-06	1.4
RPL27A	6.74E-11	0.119672495	0.531	0.442	1.62E-06	1.4
MRE11A	6.80E-11	0.128510393	0.107	0.065	1.64E-06	1.4
TAF3	6.97E-11	0.10387772	0.093	0.054	1.68E-06	1.4
FAM49B	7.02E-11	0.158604649	0.29	0.22	1.69E-06	1.4
HES1	7.04E-11	0.138762333	0.304	0.229	1.70E-06	1.4
RPP38	7.33E-11	0.13961881	0.124	0.079	1.77E-06	1.4
RUBCN	7.39E-11	-0.221888437	0.039	0.077	1.78E-06	1.4
PRELID3B	7.44E-11	-0.272168127	0.246	0.298	1.79E-06	1.4
LETMD1	7.66E-11	0.108561108	0.122	0.077	1.85E-06	1.4
PRR5L	7.68E-11	0.103568038	0.053	0.026	1.85E-06	1.4
CAAP1	7.74E-11	0.103159393	0.09	0.052	1.87E-06	1.4
TMC4	7.92E-11	0.116583829	0.063	0.032	1.91E-06	1.4
RAB18	8.11E-11	-0.315400777	0.248	0.3	1.96E-06	1.4
BCL3	8.28E-11	0.100610322	0.116	0.072	2.00E-06	1.4
UBE4B	8.59E-11	0.106175086	0.264	0.194	2.07E-06	1.4
ZNF644	8.72E-11	0.152064293	0.404	0.317	2.10E-06	1.4
HIF1A-AS2	9.72E-11	-0.356593352	0.106	0.157	2.34E-06	1.4

COX7C	1.03E-10	-0.172401727	0.882	0.889	2.47E-06	1.4
CCDC90B	1.08E-10	0.118698505	0.132	0.085	2.59E-06	1.4
FAM213A	1.09E-10	0.102796802	0.11	0.068	2.63E-06	1.4
TM2D2	1.09E-10	-0.161046586	0.033	0.069	2.63E-06	1.4
H2AFJ	1.10E-10	0.125199814	0.056	0.028	2.64E-06	1.4
HERPUD1	1.13E-10	-0.317976679	0.157	0.21	2.72E-06	1.4
ROR2	1.21E-10	0.100037536	0.162	0.109	2.91E-06	1.4
OGFRL1	1.22E-10	0.124679529	0.298	0.225	2.95E-06	1.4
ATP5L	1.25E-10	-0.167165782	0.86	0.855	3.01E-06	1.4
RASSF8	1.25E-10	-0.270476889	0.118	0.17	3.02E-06	1.4
TAF7	1.26E-10	0.125374577	0.188	0.132	3.04E-06	1.4
TMEM217	1.29E-10	-0.192475226	0.041	0.08	3.12E-06	1.4
UQCRB	1.30E-10	-0.188887327	0.83	0.827	3.15E-06	1.4
MAP4	1.34E-10	-0.300448707	0.398	0.443	3.23E-06	1.4
ARL6IP5	1.36E-10	0.10980698	0.565	0.461	3.27E-06	1.4
ZNF165	1.37E-10	-0.202282351	0.036	0.073	3.30E-06	1.4
ABI2	1.41E-10	0.111743561	0.194	0.135	3.41E-06	1.4
10-Sep	1.42E-10	0.116487119	0.192	0.135	3.42E-06	1.4
ELOVL7	1.43E-10	0.126713283	0.116	0.073	3.45E-06	1.4
PTEN	1.48E-10	-0.273368214	0.849	0.851	3.56E-06	1.4
PCNXL4	1.49E-10	0.121840531	0.135	0.088	3.59E-06	1.4
DNAJC7	1.51E-10	0.113147256	0.263	0.194	3.64E-06	1.4
HBEGF	1.52E-10	-0.115124265	0.008	0.032	3.66E-06	1.4
BRD8	1.52E-10	0.131432294	0.1	0.06	3.67E-06	1.4
SETD2	1.57E-10	0.127643593	0.344	0.267	3.77E-06	1.4
CHM	1.60E-10	0.110799186	0.107	0.066	3.86E-06	1.4
CWF19L2	1.70E-10	0.108067981	0.181	0.125	4.09E-06	1.4
VEGFA	1.85E-10	-0.296440928	0.18	0.236	4.45E-06	1.4
C7orf60	1.85E-10	0.111543858	0.124	0.079	4.47E-06	1.4
MICA	1.95E-10	-0.22300397	0.098	0.147	4.70E-06	1.4
OXR1	1.98E-10	0.116054616	0.234	0.171	4.76E-06	1.4
RUSC2	2.02E-10	-0.245210135	0.086	0.133	4.88E-06	1.4
PIGN	2.07E-10	0.12529422	0.146	0.097	4.98E-06	1.4
RNPC3	2.07E-10	0.104066064	0.107	0.066	4.99E-06	1.4
LRRC37B	2.10E-10	0.107411577	0.066	0.035	5.08E-06	1.4
PSMD4	2.10E-10	0.109930596	0.129	0.083	5.08E-06	1.4
EGF	2.16E-10	0.136381287	0.109	0.068	5.21E-06	1.4
SPRED2	2.36E-10	0.146456054	0.154	0.104	5.68E-06	1.4
ICA1	2.56E-10	0.148077711	0.337	0.264	6.18E-06	1.4
SREBF2	2.57E-10	0.100101642	0.427	0.338	6.20E-06	1.4
ZFYVE16	2.65E-10	0.126306398	0.116	0.073	6.39E-06	1.4
CEP57	2.81E-10	0.119468837	0.141	0.093	6.77E-06	1.4
WWTR1	2.82E-10	-0.343303057	0.378	0.419	6.79E-06	1.4
C9orf3	2.92E-10	0.213820211	0.208	0.153	7.04E-06	1.4
PIM1	2.93E-10	-0.100981023	0.009	0.034	7.07E-06	1.4
LARP4	2.95E-10	0.116139022	0.157	0.106	7.10E-06	1.4
EYA3	2.99E-10	0.123634634	0.132	0.086	7.20E-06	1.4

NDUFA4	2.99E-10	-0.229832191	0.809	0.798	7.20E-06	1.4
ARSB	3.08E-10	0.121806275	0.071	0.039	7.44E-06	1.4
NHSL2	3.22E-10	-0.273977453	0.294	0.346	7.76E-06	1.4
ABCD3	3.22E-10	0.107387303	0.105	0.065	7.76E-06	1.4
PRKCI	3.25E-10	-0.283395311	0.198	0.252	7.84E-06	1.4
CLEC2D	3.28E-10	0.112684791	0.154	0.103	7.91E-06	1.4
NSMAF	3.42E-10	0.111058952	0.127	0.082	8.25E-06	1.4
DDX52	3.46E-10	0.105668791	0.151	0.101	8.34E-06	1.4
ALPL	3.47E-10	0.105652499	0.085	0.049	8.37E-06	1.4
SLC7A1	3.53E-10	-0.176192348	0.066	0.109	8.52E-06	1.4
GLTSCR1L	3.71E-10	0.112178864	0.184	0.127	8.94E-06	1.4
TRIM27	3.78E-10	0.104867737	0.126	0.082	9.12E-06	1.4
NKTR	3.91E-10	0.13731745	0.362	0.282	9.42E-06	1.4
PDZD2	3.96E-10	0.133533484	0.618	0.521	9.55E-06	1.4
TFDP2	3.97E-10	0.119602546	0.277	0.21	9.57E-06	1.4
CYB5D2	4.04E-10	0.123839828	0.06	0.031	9.74E-06	1.4
ARHGEF10I	4.12E-10	0.135581362	0.284	0.215	9.94E-06	1.4
IARS	4.26E-10	-0.247081476	0.1	0.148	1.03E-05	1.4
RNF152	4.43E-10	0.143297586	0.07	0.038	1.07E-05	1.4
ZNF141	4.45E-10	0.106240878	0.091	0.054	1.07E-05	1.4
STAU2	4.48E-10	0.121316258	0.129	0.084	1.08E-05	1.4
BCKDHB	4.54E-10	0.122958911	0.077	0.044	1.09E-05	1.4
NF1	4.76E-10	0.121549623	0.525	0.433	1.15E-05	1.4
PIP5K1A	5.08E-10	-0.31774439	0.267	0.317	1.23E-05	1.4
PXN	5.13E-10	-0.182022237	0.059	0.1	1.24E-05	1.4
RELL1	5.30E-10	0.101195566	0.073	0.041	1.28E-05	1.4
VPS51	5.38E-10	0.104077883	0.083	0.048	1.30E-05	1.4
MYADM	5.47E-10	-0.13526978	0.022	0.053	1.32E-05	1.4
ARAP2	5.80E-10	0.101668004	0.181	0.127	1.40E-05	1.4
IGF2BP2-AS	5.84E-10	-0.16708236	0.025	0.056	1.41E-05	1.4
SGK223	5.89E-10	0.124413285	0.107	0.067	1.42E-05	1.4
APBB2	5.94E-10	0.134495073	0.141	0.095	1.43E-05	1.4
TGOLN2	6.00E-10	0.113349617	0.148	0.1	1.45E-05	1.4
IRF1	6.03E-10	0.100697789	0.191	0.133	1.46E-05	1.4
RPL38	6.12E-10	-0.138675409	0.927	0.928	1.48E-05	1.4
IPO11	6.30E-10	0.101756468	0.06	0.031	1.52E-05	1.4
SPATS2L	6.36E-10	0.151929321	0.33	0.258	1.53E-05	1.4
PHF8	6.51E-10	0.107616992	0.099	0.06	1.57E-05	1.4
AKAP1	6.73E-10	0.103261871	0.059	0.031	1.62E-05	1.4
GLIS3	6.81E-10	-0.390016159	0.251	0.3	1.64E-05	1.4
TCEB1	6.95E-10	-0.322146501	0.259	0.308	1.68E-05	1.4
USP6NL	7.22E-10	-0.329589629	0.17	0.22	1.74E-05	1.4
PSMB2	7.26E-10	0.103784005	0.179	0.125	1.75E-05	1.4
ZNF680	7.34E-10	0.14863922	0.13	0.086	1.77E-05	1.4
NFIX	7.39E-10	0.182744985	0.154	0.106	1.78E-05	1.4
TULP4	7.51E-10	-0.343979544	0.476	0.503	1.81E-05	1.4
UFM1	7.81E-10	-0.298371254	0.317	0.364	1.88E-05	1.4

HMGXB4	7.83E-10	0.109198586	0.308	0.235	1.89E-05	1.4
ZNF562	7.86E-10	0.121260545	0.288	0.22	1.90E-05	1.4
CLASP1	7.97E-10	0.124891671	0.309	0.238	1.92E-05	1.4
WDFY3	8.24E-10	0.103739948	0.299	0.229	1.99E-05	1.4
RP11-91P2	8.34E-10	0.117549181	0.07	0.039	2.01E-05	1.4
THOC1	8.39E-10	0.108725538	0.135	0.09	2.02E-05	1.4
TTC3	8.79E-10	0.107393976	0.352	0.273	2.12E-05	1.4
SOSTDC1	9.59E-10	-0.120107364	0.007	0.029	2.31E-05	1.4
FBXO11	9.64E-10	-0.313545187	0.354	0.397	2.32E-05	1.4
TPTEP1	1.00E-09	0.135696452	0.281	0.215	2.41E-05	1.4
SLC35F3	1.01E-09	-0.163697431	0.029	0.061	2.43E-05	1.4
GOLGB1	1.01E-09	0.123485368	0.442	0.355	2.43E-05	1.4
PREP	1.02E-09	-0.203847893	0.075	0.119	2.45E-05	1.4
PUM1	1.04E-09	-0.263623138	0.492	0.521	2.51E-05	1.4
FAM46B	1.04E-09	-0.195961865	0.045	0.083	2.51E-05	1.4
TTC9	1.07E-09	0.119576948	0.242	0.179	2.58E-05	1.4
HIST1H2BJ	1.10E-09	0.135641673	0.124	0.081	2.66E-05	1.4
ATP6V1B2	1.12E-09	0.14028944	0.19	0.138	2.71E-05	1.4
ZRANB1	1.14E-09	-0.263530796	0.122	0.17	2.75E-05	1.4
IDH1	1.18E-09	0.102463587	0.099	0.061	2.85E-05	1.4
LNPEP	1.21E-09	0.11182239	0.141	0.095	2.92E-05	1.4
MRPL19	1.22E-09	0.108040684	0.085	0.05	2.95E-05	1.4
CD74	1.25E-09	0.12487897	0.198	0.142	3.02E-05	1.4
RPTOR	1.26E-09	0.102556235	0.088	0.052	3.04E-05	1.4
CYP20A1	1.29E-09	0.101758911	0.097	0.06	3.11E-05	1.4
CAPRIN1	1.30E-09	0.109556967	0.286	0.217	3.13E-05	1.4
BOD1L1	1.31E-09	0.133950454	0.139	0.094	3.15E-05	1.4
AC005042.	1.36E-09	0.13245026	0.101	0.063	3.27E-05	1.4
R3HCC1L	1.39E-09	0.120030408	0.163	0.113	3.35E-05	1.4
PI4KA	1.40E-09	0.117710227	0.124	0.081	3.37E-05	1.4
RPL13A	1.42E-09	0.173009347	0.607	0.513	3.44E-05	1.4
AATF	1.45E-09	0.107629879	0.062	0.033	3.51E-05	1.4
SMYD4	1.46E-09	0.118605463	0.05	0.025	3.51E-05	1.4
NR2C2	1.48E-09	0.126960986	0.176	0.124	3.57E-05	1.4
GPR87	1.51E-09	-0.169021797	0.032	0.064	3.63E-05	1.4
LINC01481	1.61E-09	0.108003131	0.079	0.046	3.87E-05	1.4
RSF1	1.63E-09	0.110960424	0.384	0.303	3.93E-05	1.4
HNRNPLL	1.66E-09	0.128661866	0.147	0.101	4.00E-05	1.4
FAM174B	1.66E-09	0.118197619	0.082	0.049	4.01E-05	1.4
BCL10	1.69E-09	-0.231803883	0.095	0.14	4.07E-05	1.4
DLGAP4	1.69E-09	0.140272277	0.145	0.098	4.08E-05	1.4
ATP6V1D	1.90E-09	-0.333668902	0.259	0.304	4.59E-05	1.4
ZNF592	1.91E-09	-0.238024031	0.091	0.135	4.60E-05	1.4
IDS	1.92E-09	-0.11227961	0.022	0.052	4.64E-05	1.4
SCOC	1.93E-09	0.115393891	0.317	0.245	4.65E-05	1.4
CDK14	1.98E-09	-0.313787075	0.375	0.413	4.78E-05	1.4
PTPN12	2.06E-09	-0.277833687	0.399	0.435	4.98E-05	1.4

RRAS2	2.20E-09	-0.30314704	0.188	0.239	5.30E-05	1.4
JKAMP	2.21E-09	0.103780461	0.113	0.073	5.34E-05	1.4
TTC9C	2.23E-09	0.108002372	0.189	0.136	5.38E-05	1.4
APPL2	2.27E-09	0.116569287	0.101	0.064	5.48E-05	1.4
OCIAD1	2.31E-09	0.108892375	0.331	0.258	5.58E-05	1.4
RP1-78O14	2.34E-09	-0.260495356	0.042	0.077	5.64E-05	1.4
DROSHA	2.34E-09	0.107221395	0.063	0.034	5.65E-05	1.4
CENPC	2.46E-09	0.104745034	0.098	0.061	5.93E-05	1.4
INADL	2.49E-09	0.108825101	0.872	0.774	6.01E-05	1.4
HCG17	2.51E-09	0.109555546	0.095	0.058	6.06E-05	1.4
PLSCR4	2.57E-09	0.133168191	0.109	0.07	6.19E-05	1.4
RPS2	2.57E-09	0.186731182	0.434	0.358	6.19E-05	1.4
PRKRA	2.71E-09	0.112156732	0.109	0.07	6.54E-05	1.4
ELF3	2.78E-09	-0.351745756	0.517	0.544	6.71E-05	1.4
DSG2	2.81E-09	-0.273121722	0.33	0.369	6.77E-05	1.4
ZSWIM7	2.88E-09	0.138191329	0.1	0.063	6.96E-05	1.4
NXN	3.01E-09	0.16028982	0.206	0.151	7.26E-05	1.4
TRAM1	3.02E-09	-0.226212512	0.178	0.229	7.28E-05	1.4
ZNF721	3.15E-09	0.141004089	0.262	0.199	7.60E-05	1.4
TMA16	3.18E-09	0.100761362	0.074	0.043	7.66E-05	1.4
CREBBP	3.22E-09	0.125854179	0.289	0.222	7.77E-05	1.4
CBWD7	3.23E-09	0.105260922	0.102	0.064	7.79E-05	1.4
ABL1	3.25E-09	0.113310814	0.169	0.12	7.83E-05	1.4
HIST1H4H	3.30E-09	0.104077092	0.098	0.061	7.97E-05	1.4
FGD4	3.33E-09	-0.338099044	0.231	0.279	8.03E-05	1.4
LCN2	3.38E-09	0.131717131	0.056	0.03	8.15E-05	1.4
RPL23	3.40E-09	-0.103916171	0.961	0.964	8.20E-05	1.4
RP11-415J8	3.41E-09	0.109568048	0.087	0.052	8.22E-05	1.4
GRIN2A	3.43E-09	-0.244773987	0.033	0.065	8.26E-05	1.4
SMG6	3.49E-09	0.125850149	0.2	0.145	8.41E-05	1.4
GTF2IRD1	3.52E-09	-0.249119054	0.113	0.159	8.49E-05	1.4
MT-CO1	3.60E-09	0.177686824	0.983	0.991	8.69E-05	1.4
NOL4	3.67E-09	0.113181428	0.067	0.038	8.85E-05	1.4
AK9	3.68E-09	0.108212864	0.083	0.05	8.87E-05	1.4
CTNNA1	3.69E-09	-0.258103776	0.478	0.506	8.91E-05	1.4
WHSC1L1	3.71E-09	0.125609579	0.234	0.176	8.95E-05	1.4
CTGF	3.84E-09	-0.188006098	0.016	0.041	9.26E-05	1.4
LIPE-AS1	3.95E-09	0.109843855	0.113	0.073	9.53E-05	1.4
ARID2	4.03E-09	0.129911885	0.352	0.28	9.72E-05	1.4
CLNS1A	4.07E-09	0.109007834	0.121	0.079	9.82E-05	1.4
NSUN3	4.17E-09	0.11212241	0.074	0.043	0.0001006	1.4
GCH1	4.18E-09	0.172646883	0.121	0.08	0.0001007	1.4
STOM	4.20E-09	0.128377407	0.241	0.182	0.0001013	1.4
RP11-68E1	4.21E-09	-0.149092731	0.031	0.062	0.0001016	1.4
GK	4.23E-09	0.106967531	0.074	0.043	0.000102	1.4
IRF6	4.25E-09	-0.290950435	0.186	0.234	0.0001024	1.4
TANC1	4.31E-09	0.13554785	0.308	0.24	0.0001039	1.4

HNRNPC	4.37E-09	-0.264155269	0.823	0.8	0.0001053	1.4
UQCRH	4.42E-09	-0.185102153	0.85	0.856	0.0001066	1.4
PFKFB4	4.46E-09	-0.144532835	0.019	0.047	0.0001075	1.4
SLC6A14	4.57E-09	-0.299496032	0.173	0.226	0.0001102	1.4
RP11-479O	4.75E-09	0.113008114	0.088	0.053	0.0001146	1.4
LAMP2	4.77E-09	-0.311083949	0.219	0.265	0.0001149	1.4
KIAA1328	4.79E-09	0.117413544	0.118	0.078	0.0001155	1.4
LARGE	4.82E-09	0.107520935	0.284	0.217	0.0001163	1.4
IMMP1L	5.24E-09	0.114747688	0.11	0.072	0.0001265	1.4
SET	5.31E-09	-0.25768343	0.464	0.495	0.0001281	1.4
FRK	5.34E-09	0.117600503	0.207	0.153	0.0001289	1.4
PPM1B	5.43E-09	0.118095149	0.221	0.164	0.000131	1.4
EXOC2	5.68E-09	0.102514097	0.101	0.064	0.000137	1.4
KMT2C	5.75E-09	-0.349855555	0.624	0.61	0.0001386	1.4
AQR	5.79E-09	0.12128865	0.113	0.073	0.0001397	1.4
SEC11A	5.80E-09	-0.238194593	0.52	0.539	0.0001399	1.4
PER2	6.39E-09	0.121849736	0.133	0.09	0.000154	1.4
NCK2	6.82E-09	0.139093207	0.204	0.15	0.0001644	1.4
ADGRL3-AS	6.87E-09	-0.214545536	0.641	0.674	0.0001656	1.4
DIAPH3	6.90E-09	-0.22560213	0.093	0.138	0.0001664	1.4
CABIN1	7.10E-09	0.109449738	0.104	0.066	0.0001711	1.4
KLHL28	7.14E-09	0.100500743	0.104	0.066	0.0001722	1.4
BMPR1B	7.26E-09	-0.220569121	0.015	0.039	0.000175	1.4
HSPA9	7.27E-09	-0.247306305	0.176	0.223	0.0001754	1.4
SUN1	7.28E-09	-0.184884547	0.078	0.119	0.0001756	1.4
CDCP1	7.29E-09	-0.322459886	0.347	0.387	0.0001758	1.4
NPC1	7.34E-09	-0.340194719	0.293	0.334	0.0001769	1.4
PRKCA	7.78E-09	-0.218453498	0.062	0.1	0.0001877	1.4
REST	7.83E-09	0.108465793	0.099	0.063	0.0001888	1.4
RP11-613M	7.87E-09	0.111603162	0.091	0.056	0.0001898	1.4
LMBR1	8.15E-09	0.102224244	0.156	0.109	0.0001966	1.4
AHR	8.22E-09	-0.265635077	0.169	0.218	0.0001982	1.4
ZNF281	8.24E-09	-0.177928324	0.063	0.101	0.0001987	1.4
ARFIP1	8.27E-09	0.109271719	0.19	0.137	0.0001994	1.4
FABP3	8.30E-09	-0.189545888	0.003	0.02	0.0002001	1.4
NUP58	8.54E-09	-0.178695154	0.075	0.117	0.0002059	1.4
KANK1	8.57E-09	-0.223332234	0.179	0.232	0.0002067	1.4
ZNF814	8.93E-09	0.100170248	0.123	0.082	0.0002152	1.4
IGBP1	9.01E-09	0.100908391	0.524	0.432	0.0002173	1.4
RAB11FIP3	9.31E-09	0.109211355	0.107	0.069	0.0002244	1.4
MT1E	9.46E-09	0.267280308	0.06	0.033	0.000228	1.4
CAMTA1	9.47E-09	-0.315259184	0.506	0.529	0.0002283	1.4
RPL7A	9.84E-09	-0.160463885	0.967	0.979	0.0002372	1.4
FHL2	9.84E-09	-0.162133436	0.054	0.09	0.0002372	1.4
DGKH	1.02E-08	0.128757959	0.196	0.144	0.0002469	1.4
GALNT1	1.03E-08	-0.243411341	0.118	0.162	0.0002479	1.4
ANKRD6	1.07E-08	0.106282135	0.118	0.078	0.000258	1.4

EIF2S1	1.08E-08	-0.291602412	0.227	0.273	0.0002599	1.4
PRCC	1.08E-08	0.104767591	0.097	0.062	0.0002603	1.4
DTX2	1.09E-08	0.100285694	0.135	0.092	0.0002626	1.4
HPRT1	1.09E-08	0.116944993	0.095	0.06	0.0002637	1.4
DUSP14	1.11E-08	-0.130950755	0.02	0.046	0.0002673	1.4
NR4A1	1.12E-08	-0.109840665	0.01	0.032	0.0002696	1.4
STC2	1.12E-08	0.127594776	0.058	0.032	0.0002703	1.4
TEX9	1.13E-08	0.111542874	0.074	0.044	0.0002736	1.4
EHBP1	1.16E-08	0.105910949	0.4	0.323	0.0002792	1.4
S100P	1.16E-08	-0.13695774	0.015	0.039	0.0002807	1.4
PFDN1	1.19E-08	-0.234938567	0.198	0.247	0.0002869	1.4
RPS23	1.21E-08	-0.146191268	0.975	0.987	0.0002919	1.4
AP001439.	1.25E-08	-0.202836969	0.063	0.101	0.0003009	1.4
SAR1A	1.25E-08	-0.23725903	0.178	0.225	0.0003017	1.4
GCNT1	1.28E-08	-0.245708228	0.064	0.101	0.0003086	1.4
RPLP1	1.29E-08	0.110281029	0.318	0.251	0.0003118	1.4
KIAA1033	1.35E-08	0.130541398	0.139	0.096	0.0003257	1.4
GNL2	1.37E-08	-0.175259177	0.073	0.113	0.0003302	1.4
LONP2	1.40E-08	0.105430684	0.266	0.203	0.000337	1.4
HMGA1	1.40E-08	-0.345824017	0.104	0.147	0.0003377	1.4
KCNQ5	1.41E-08	-0.217874602	0.044	0.078	0.0003398	1.4
OPA1	1.46E-08	0.136676222	0.144	0.101	0.0003531	1.4
FOS	1.50E-08	0.136897092	0.291	0.227	0.0003622	1.4
CCZ1B	1.54E-08	0.10172775	0.066	0.038	0.0003724	1.4
GTF2H2C	1.56E-08	0.107280307	0.081	0.049	0.0003757	1.4
CELF2	1.61E-08	-0.298277682	0.214	0.262	0.000388	1.4
RP11-481C	1.62E-08	-0.178134114	0.039	0.072	0.0003916	1.4
CA12	1.63E-08	-0.339913411	0.197	0.245	0.0003927	1.4
SRPK2	1.64E-08	-0.311208358	0.351	0.388	0.0003948	1.4
RPL3	1.68E-08	0.137969075	0.42	0.343	0.0004058	1.4
DNAJC8	1.68E-08	-0.256235388	0.194	0.24	0.000406	1.4
PPM1A	1.70E-08	0.108929349	0.164	0.117	0.0004089	1.4
ZHX2	1.71E-08	0.10162388	0.409	0.328	0.000412	1.4
SUZ12	1.73E-08	0.13401231	0.224	0.169	0.0004179	1.4
SPPL2A	1.78E-08	0.102341799	0.244	0.185	0.0004303	1.4
METTL16	1.79E-08	0.116320753	0.229	0.172	0.000431	1.4
GNPAT	1.80E-08	0.104843241	0.085	0.053	0.0004331	1.4
ERC2	1.86E-08	0.10664882	0.121	0.081	0.0004486	1.4
TBC1D10A	1.91E-08	-0.447280805	0.087	0.127	0.0004602	1.4
SH3BGRL3	2.05E-08	-0.24902234	0.03	0.059	0.0004953	1.4
ARHGAP24	2.11E-08	0.105721097	0.07	0.041	0.0005092	1.4
RNMT	2.13E-08	-0.274543645	0.344	0.383	0.0005127	1.4
INSR	2.27E-08	0.101196668	0.717	0.627	0.0005466	1.4
NFATC2IP	2.35E-08	0.106202955	0.1	0.065	0.0005675	1.4
NFIL3	2.35E-08	-0.251133765	0.124	0.167	0.0005676	1.4
SPG7	2.40E-08	0.112161163	0.081	0.049	0.0005785	1.4
PLXNA2	2.63E-08	0.1008494	0.06	0.034	0.0006339	1.4

XDH	3.50E-08	0.190017932	0.149	0.105	0.0008435	1.4
OTUD3	3.51E-08	-0.146717075	0.027	0.055	0.0008469	1.4
CHD9	3.64E-08	0.119104395	0.532	0.447	0.0008777	1.4
DAPL1	3.73E-08	-0.197269365	0.029	0.057	0.0008987	1.4
TNFRSF11A	3.74E-08	-0.272240771	0.103	0.143	0.0009022	1.4
GGACT	3.76E-08	-0.215674296	0.137	0.181	0.0009055	1.4
P4HA1	3.79E-08	-0.368464865	0.238	0.282	0.0009142	1.4
TPR	3.95E-08	0.101502606	0.195	0.145	0.0009532	1.4
TBX19	4.21E-08	0.106482756	0.127	0.087	0.0010163	1.4
WSB1	4.25E-08	-0.408926188	0.31	0.347	0.001024	1.4
RPL32	4.31E-08	-0.142917165	0.975	0.989	0.00104	1.4
NTRK2	4.35E-08	0.113562928	0.23	0.173	0.0010496	1.4
SOCS5	4.69E-08	0.123555055	0.206	0.154	0.0011311	1.4
CHMP2B	4.73E-08	-0.202459797	0.086	0.125	0.0011414	1.4
CAPZA2	5.09E-08	-0.275726324	0.298	0.34	0.0012274	1.4
F11R	5.48E-08	-0.215796128	0.143	0.187	0.001322	1.4
MICU2	5.54E-08	0.101197094	0.166	0.119	0.0013359	1.4
CEBPG	5.66E-08	-0.102265456	0.024	0.05	0.0013643	1.4
HM13	5.78E-08	0.10415994	0.194	0.143	0.0013929	1.4
CRABP2	5.89E-08	-0.170983363	0.057	0.092	0.0014205	1.4
SCFD1	5.94E-08	0.12191534	0.27	0.211	0.0014334	1.4
SMG1	5.95E-08	-0.284540405	0.227	0.269	0.0014339	1.4
KLK10	6.23E-08	-0.174988196	0.037	0.067	0.0015012	1.4
SLK	6.24E-08	-0.212198899	0.104	0.145	0.0015056	1.4
EEF2	6.30E-08	0.160313013	0.503	0.425	0.0015198	1.4
BNIP3L	6.47E-08	-0.242722761	0.275	0.319	0.0015607	1.4
MLLT10	6.52E-08	0.108142711	0.203	0.151	0.0015716	1.4
H3F3B	6.52E-08	0.112554889	0.456	0.376	0.0015727	1.4
MIRLET7BH	6.81E-08	0.121493509	0.052	0.029	0.0016413	1.4
KIAA0040	6.86E-08	0.120782031	0.086	0.054	0.0016533	1.4
NFAT5	7.05E-08	-0.255819096	0.624	0.627	0.0017003	1.4
RNF13	7.07E-08	0.10131144	0.351	0.282	0.0017039	1.4
XYLT1	8.10E-08	0.168894915	0.147	0.105	0.0019541	1.4
NRF1	8.12E-08	0.108311694	0.158	0.114	0.001958	1.4
ESRRG	8.52E-08	0.120273143	0.05	0.027	0.0020543	1.4
TUBB4B	8.62E-08	-0.137435554	0.034	0.063	0.0020782	1.4
PFN1	8.74E-08	-0.223850084	0.147	0.188	0.0021067	1.4
DLG5	9.12E-08	0.138961485	0.207	0.158	0.0022	1.4
MYO9B	9.46E-08	-0.273086392	0.17	0.213	0.0022803	1.4
MAPKAP1	9.47E-08	0.106119301	0.128	0.089	0.0022827	1.4
ATXN2	9.69E-08	0.113003645	0.333	0.264	0.0023366	1.4
CLDND1	1.00E-07	-0.207464048	0.181	0.227	0.0024187	1.4
AUH	1.01E-07	-0.419073202	0.317	0.346	0.002439	1.4
B3GALT5	1.03E-07	0.100179173	0.077	0.048	0.0024912	1.4
C11orf49	1.05E-07	0.101289088	0.176	0.13	0.0025406	1.4
SMARCC1	1.06E-07	0.147973653	0.297	0.237	0.0025482	1.4
RBBP4	1.14E-07	0.104155573	0.206	0.155	0.0027491	1.4

CHD8	1.14E-07	0.107046472	0.121	0.083	0.0027515	1.4
RPL39	1.16E-07	-0.105148307	0.933	0.947	0.0027943	1.4
SPINK5	1.17E-07	-0.143497044	0.007	0.024	0.0028249	1.4
MAPK6	1.20E-07	-0.230750624	0.181	0.225	0.0028858	1.4
AKT3	1.24E-07	-0.343795906	0.46	0.482	0.0029833	1.4
PATL1	1.28E-07	0.106065194	0.185	0.138	0.0030917	1.4
PTPN9	1.29E-07	0.100289918	0.098	0.065	0.0031092	1.4
CALU	1.31E-07	-0.263154707	0.263	0.304	0.0031616	1.4
ATP5G1	1.34E-07	-0.249707486	0.175	0.218	0.0032422	1.4
RPF2	1.37E-07	-0.228557138	0.151	0.193	0.003307	1.4
KHDRBS1	1.41E-07	-0.248179716	0.265	0.304	0.0034082	1.4
IQCB1	1.53E-07	0.117150406	0.127	0.089	0.003696	1.4
SLIRP	1.61E-07	-0.282250082	0.309	0.345	0.003894	1.4
FAM162A	1.65E-07	-0.193703964	0.113	0.154	0.0039686	1.4
AGO3	1.66E-07	0.108670224	0.294	0.232	0.0040047	1.4
TNFAIP3	1.68E-07	-0.304101095	0.222	0.265	0.004052	1.4
GPS2	1.71E-07	-0.200718515	0.087	0.124	0.004118	1.4
TCF25	1.71E-07	0.101865326	0.143	0.102	0.0041289	1.4
PCGF5	1.74E-07	-0.238906262	0.15	0.192	0.0041908	1.4
LINC00511	1.77E-07	0.196759811	0.15	0.11	0.0042669	1.4
TRIM2	1.79E-07	-0.225050351	0.269	0.316	0.0043183	1.4
ACTG1	1.94E-07	-0.342293896	0.689	0.7	0.00468	1.4
GRB10	1.96E-07	0.194746285	0.168	0.126	0.0047224	1.4
MYO1B	1.96E-07	0.10977287	0.384	0.314	0.0047347	1.4
ZDHHC17	2.02E-07	0.114909902	0.122	0.084	0.0048625	1.4
DCTN4	2.02E-07	0.106553606	0.207	0.158	0.0048816	1.4
SCAI	2.14E-07	0.119704239	0.111	0.076	0.005152	1.4
ACTR10	2.16E-07	0.107655579	0.16	0.117	0.0052203	1.4
RAB2A	2.20E-07	0.10873471	0.32	0.256	0.0053022	1.4
WBP5	2.23E-07	-0.265899939	0.374	0.403	0.0053864	1.4
VDAC1	2.24E-07	-0.21789625	0.186	0.228	0.0054122	1.4
CAP1	2.25E-07	-0.231191733	0.197	0.239	0.0054261	1.4
SHOC2	2.30E-07	0.116139788	0.261	0.206	0.0055422	1.4
PRELID2	2.38E-07	-0.209851981	0.053	0.085	0.0057389	1.4
RREB1	2.51E-07	0.105167443	0.31	0.248	0.0060422	1.4
KDM7A	2.54E-07	0.133641642	0.394	0.325	0.0061359	1.4
DENND2C	2.55E-07	-0.129586539	0.023	0.048	0.0061382	1.4
GJA1	2.55E-07	-0.211790354	0.038	0.065	0.0061545	1.4
RAB7A	2.67E-07	-0.262820037	0.365	0.398	0.0064296	1.4
MRPL47	2.70E-07	-0.212084199	0.156	0.198	0.0065081	1.4
KRAS	2.73E-07	-0.199198839	0.113	0.153	0.0065874	1.4
MT2A	2.75E-07	0.18764221	0.1	0.067	0.0066291	1.4
DFNB59	2.76E-07	0.146386066	0.033	0.015	0.0066657	1.4
NDUFAF4	2.89E-07	-0.190953031	0.085	0.121	0.0069614	1.4
TSLP	2.89E-07	-0.173550079	0.052	0.084	0.0069659	1.4
BRIX1	2.92E-07	-0.221012414	0.095	0.133	0.0070478	1.4
ANKLE2	2.93E-07	-0.155908581	0.088	0.125	0.0070613	1.4

NBEAL1	2.95E-07	0.140256849	0.742	0.668	0.0071117	1.4
TLE1	2.98E-07	-0.230176179	0.194	0.239	0.0071805	1.4
FAM160A1	3.10E-07	-0.286689352	0.716	0.695	0.0074744	1.4
AFF4	3.18E-07	-0.27571417	0.412	0.436	0.0076706	1.4
PSEN1	3.23E-07	0.131233758	0.257	0.203	0.0077913	1.4
RASGEF1B	3.31E-07	-0.218852003	0.901	0.892	0.00797	1.4
TMX4	3.43E-07	0.107164796	0.066	0.04	0.0082598	1.4
FAR1	3.46E-07	-0.177805826	0.084	0.121	0.0083486	1.4
LATS2	3.52E-07	-0.176806495	0.092	0.13	0.008487	1.4
LURAP1L-A	3.57E-07	-0.164333755	0.066	0.1	0.0086131	1.4
HMOX1	3.58E-07	-0.28550577	0.042	0.071	0.0086278	1.4
LRRC59	3.91E-07	-0.108603368	0.032	0.058	0.0094324	1.4
KANSL3	4.25E-07	0.104379004	0.085	0.056	0.010257	1.4
HNRNPA3	4.51E-07	-0.238354003	0.315	0.35	0.0108719	1.4
HMGB1	4.63E-07	0.113441656	0.599	0.519	0.0111538	1.4
RP11-511B	4.69E-07	-0.193200545	0.585	0.591	0.0113065	1.4
GTPBP4	4.74E-07	-0.217972635	0.109	0.147	0.0114309	1.4
FAM193A	5.04E-07	0.104572362	0.181	0.136	0.0121505	1.4
PIP	5.41E-07	-0.184262242	0.032	0.058	0.0130376	1.4
ARHGAP12	6.27E-07	-0.279379973	0.314	0.348	0.0151073	1.4
UBR2	6.47E-07	0.123899317	0.365	0.3	0.0155947	1.4
ZBTB43	6.58E-07	-0.239350234	0.131	0.169	0.0158718	1.4
RTKN2	6.84E-07	-0.120329389	0.019	0.041	0.0164913	1.4
CA2	7.23E-07	-0.177216981	0.047	0.076	0.0174291	1.4
HNRNPDL	8.32E-07	-0.219916875	0.273	0.313	0.0200601	1.4
ATP6V0A1	8.42E-07	-0.22169291	0.143	0.182	0.0203021	1.4
LDHC	8.83E-07	-0.107455757	0.012	0.031	0.0212902	1.4
CXCR4	8.89E-07	-0.540965679	0.275	0.307	0.0214317	1.4
ARL5B	9.29E-07	-0.158766604	0.051	0.08	0.0224017	1.4
PFDN2	9.86E-07	-0.158320864	0.055	0.086	0.0237785	1.4
KIAA0825	1.02E-06	0.100781077	0.071	0.045	0.0244845	1.4
NMNAT2	1.03E-06	0.120800759	0.05	0.029	0.0248975	1.4
PERP	1.08E-06	-0.246515401	0.306	0.336	0.0259563	1.4
CSNK1G3	1.09E-06	0.103363714	0.125	0.089	0.0262912	1.4
CA5B	1.10E-06	-0.195479451	0.107	0.143	0.0264696	1.4
SEMA3C	1.12E-06	-0.198568982	0.046	0.075	0.0271236	1.4
PUM2	1.13E-06	-0.267587472	0.335	0.363	0.0273575	1.4
NF2	1.14E-06	-0.122165304	0.046	0.075	0.0275528	1.4
HNRNPD	1.18E-06	-0.242402631	0.264	0.298	0.0283596	1.4
AC037445.	1.25E-06	-0.1051684	0.016	0.037	0.030031	1.4
BTAF1	1.27E-06	-0.268966043	0.219	0.257	0.0306978	1.4
SPTBN1	1.27E-06	0.111389405	0.239	0.189	0.0307026	1.4
PRKDC	1.34E-06	0.109631593	0.141	0.104	0.0322515	1.4
MPRIP	1.34E-06	-0.224927642	0.198	0.239	0.0323506	1.4
WDR1	1.37E-06	-0.156470677	0.114	0.152	0.0330604	1.4
DSC3	1.43E-06	-0.151716516	0.019	0.039	0.0345553	1.4
ETS2	1.54E-06	-0.203421868	0.099	0.135	0.0370498	1.4

RPS5	1.59E-06	0.146901687	0.333	0.275	0.0383272	1.4
SLC7A11	1.74E-06	-0.123935788	0.031	0.055	0.0419093	1.4
STX3	1.74E-06	-0.172539085	0.075	0.108	0.0419332	1.4
SERINC1	1.77E-06	-0.226276668	0.28	0.316	0.0426303	1.4
ACTG2	1.81E-06	-0.137326837	0.005	0.02	0.043619	1.4
SENP7	1.81E-06	0.124789926	0.15	0.112	0.0437094	1.4
DIAPH1	1.82E-06	-0.229065292	0.183	0.224	0.0438698	1.4
DDX47	1.90E-06	-0.131722274	0.029	0.053	0.0458174	1.4
ITGB5-AS1	1.92E-06	-0.21707117	0.024	0.046	0.0463998	1.4
MAP4K5	2.02E-06	-0.305846733	0.343	0.372	0.04865	1.4
TMOD3	2.12E-06	-0.230738294	0.258	0.297	0.0511513	1.4
RP4-605O3	2.17E-06	0.121281995	0.124	0.09	0.052305	1.4
CAV1	2.19E-06	-0.167587759	0.041	0.067	0.0527907	1.4
TMED2	2.24E-06	-0.174894543	0.127	0.165	0.053913	1.4
ARHGEF12	2.28E-06	-0.262331831	0.412	0.44	0.0549963	1.4
CYYR1	2.30E-06	0.100357458	0.1	0.069	0.0554773	1.4
KRT5	2.41E-06	-0.101729655	0.015	0.034	0.0580676	1.4
PSMA4	2.59E-06	0.109481897	0.565	0.479	0.0623979	1.4
MED21	2.63E-06	-0.259180039	0.212	0.247	0.0633242	1.4
TUFT1	2.87E-06	-0.200314664	0.124	0.159	0.0691634	1.4
PABPC1	2.97E-06	-0.163149974	0.811	0.782	0.0715575	1.4
CXADR	3.00E-06	0.106694942	0.269	0.216	0.072295	1.4
PDE1C	3.02E-06	0.125626371	0.103	0.072	0.0728859	1.4
ASNS	3.21E-06	-0.134628767	0.06	0.09	0.0773651	1.4
GNB1	3.23E-06	-0.201122892	0.262	0.299	0.0779224	1.4
EXOSC8	3.61E-06	-0.186651075	0.122	0.159	0.086996	1.4
ATP5G2	3.62E-06	-0.260099036	0.819	0.773	0.0872989	1.4
CDH3	3.63E-06	0.103889883	0.177	0.137	0.087614	1.4
NAA50	3.64E-06	-0.225667902	0.187	0.225	0.0878752	1.4
AKAP10	3.81E-06	0.112874117	0.136	0.1	0.091887	1.4
GNAS	3.83E-06	-0.214870109	0.603	0.598	0.0922928	1.4
SGK1	3.83E-06	-0.256949234	0.151	0.187	0.0923893	1.4
EFNB2	4.04E-06	-0.151424181	0.098	0.133	0.0974015	1.4
SAV1	4.14E-06	0.123124792	0.421	0.359	0.0998113	1.4
TGFBR2	4.18E-06	-0.200049712	0.102	0.135	0.1007216	1.4
MGLL	4.27E-06	-0.124894504	0.047	0.074	0.1029765	1.4
PDIA3	4.31E-06	-0.290767731	0.246	0.279	0.1039585	1.4
KTN1	4.41E-06	-0.242365168	0.384	0.402	0.1064008	1.4
PITPNB	4.50E-06	-0.255429785	0.293	0.323	0.1085325	1.4
OSBPL10	4.70E-06	-0.182799887	0.108	0.143	0.1132396	1.4
KRT6B	4.79E-06	-0.358305061	0.096	0.128	0.1154238	1.4
MRPL24	4.83E-06	-0.13637813	0.044	0.069	0.1163963	1.4
MEST	4.87E-06	-0.124012147	0.022	0.043	0.1173503	1.4
TFG	5.12E-06	-0.259372977	0.289	0.32	0.1235392	1.4
RANBP2	5.24E-06	-0.249597524	0.253	0.286	0.1262834	1.4
KIF13B	5.35E-06	-0.259807744	0.269	0.304	0.1290196	1.4
HSPH1	5.59E-06	-0.222891139	0.099	0.132	0.1349004	1.4

POU2F3	5.92E-06	0.111295474	0.2	0.157	0.1428609	1.4
PRSS8	6.12E-06	-0.302858411	0.043	0.068	0.147548	1.4
ARID1A	6.35E-06	0.100238518	0.245	0.197	0.153131	1.4
COTL1	6.40E-06	-0.109544659	0.026	0.047	0.1544203	1.4
MTRNR2L1	6.59E-06	-0.260839942	0.255	0.29	0.1588038	1.4
ENY2	6.88E-06	-0.246918566	0.408	0.425	0.1658168	1.4
EPC1	7.09E-06	0.118777038	0.172	0.132	0.1709507	1.4
PPP3CA	7.27E-06	-0.286787423	0.396	0.419	0.1752154	1.4
DNAJB6	7.62E-06	-0.267560924	0.329	0.358	0.1837338	1.4
LMTK2	7.96E-06	-0.196037636	0.13	0.165	0.1919124	1.4
ZFAND1	7.96E-06	-0.218600827	0.253	0.287	0.1920199	1.4
NARS	8.70E-06	-0.236165913	0.266	0.298	0.2098799	1.4
TIPARP	8.82E-06	-0.138709164	0.078	0.109	0.2125588	1.4
RPL4	8.97E-06	-0.126261529	0.959	0.957	0.2162926	1.4
PDCD10	1.04E-05	-0.202571784	0.26	0.297	0.2518126	1.4
LINC00866	1.06E-05	-0.157003008	0.008	0.023	0.2560274	1.4
RNF115	1.06E-05	-0.345781681	0.217	0.247	0.2563323	1.4
MSMO1	1.07E-05	-0.256440647	0.244	0.279	0.2591873	1.4
LAMTOR3	1.08E-05	-0.167947553	0.117	0.15	0.2592426	1.4
STX12	1.09E-05	-0.328410473	0.44	0.452	0.2639942	1.4
PCNP	1.12E-05	-0.222785796	0.354	0.377	0.2697448	1.4
LDLRAD3	1.14E-05	0.117319961	0.341	0.287	0.2756902	1.4
ATXN1	1.19E-05	-0.349310028	0.347	0.368	0.2863534	1.4
ZNF219	1.19E-05	-0.103201874	0.04	0.064	0.2864141	1.4
BAG5	1.19E-05	-0.13793231	0.093	0.126	0.2867439	1.4
CTR9	1.19E-05	-0.175654866	0.071	0.1	0.2876382	1.4
VIM	1.19E-05	-0.239948528	0.183	0.218	0.2876977	1.4
SCD5	1.28E-05	-0.143373995	0.044	0.069	0.3084956	1.4
ATP8B1	1.29E-05	-0.338310806	0.312	0.339	0.3113301	1.4
CD55	1.29E-05	-0.252591969	0.359	0.386	0.3119391	1.4
PGM3	1.30E-05	-0.175197427	0.089	0.119	0.3138373	1.4
PGM2L1	1.32E-05	-0.153925726	0.086	0.117	0.3172234	1.4
AP006222.	1.32E-05	-0.100653347	0.024	0.044	0.3181298	1.4
FBXL2	1.32E-05	-0.198689382	0.079	0.108	0.3186927	1.4
NXF1	1.43E-05	-0.133078429	0.062	0.09	0.3448481	1.4
SLC26A3	1.55E-05	-0.191319571	0.76	0.762	0.3734841	1.4
IGFBP5	1.55E-05	-0.199984348	0.047	0.072	0.3739465	1.4
CBL	1.56E-05	-0.139889014	0.071	0.1	0.3771519	1.4
MARS	1.66E-05	-0.143582642	0.11	0.143	0.3992345	1.4
KANSL1	1.66E-05	0.105559773	0.425	0.367	0.400547	1.4
COL6A1	1.68E-05	-0.146827119	0.03	0.051	0.4052667	1.4
CXCL3	1.87E-05	-0.249475633	0.071	0.099	0.4506227	1.4
PARD6B	1.93E-05	-0.142751042	0.118	0.151	0.4664881	1.4
ZDHHC9	1.97E-05	-0.181651066	0.107	0.139	0.4740401	1.4
CCDC126	2.00E-05	-0.136406772	0.05	0.075	0.4816614	1.4
RAB9A	2.06E-05	-0.19423053	0.181	0.216	0.4956925	1.4
TAB2	2.11E-05	-0.215064933	0.245	0.279	0.5093502	1.4

ISG20	2.21E-05	-0.136240646	0.033	0.054	0.5323883	1.4
TIMM17A	2.33E-05	-0.222090287	0.198	0.23	0.5611025	1.4
SCAF4	2.40E-05	-0.128812184	0.063	0.09	0.5788317	1.4
TUBB	2.43E-05	-0.229655762	0.328	0.357	0.5849251	1.4
FUT11	2.43E-05	-0.123774769	0.057	0.083	0.5849432	1.4
ALKBH5	2.43E-05	-0.111710572	0.051	0.076	0.5867652	1.4
RAB14	2.43E-05	-0.172389539	0.099	0.13	0.5871271	1.4
RP1-313I6.	2.45E-05	-0.146982745	0.046	0.07	0.5899839	1.4
RNF10	2.47E-05	-0.170990775	0.145	0.178	0.5967028	1.4
SMAD3	2.61E-05	-0.174404629	0.101	0.132	0.6287793	1.4
CTD-2528L	2.63E-05	-0.115379481	0.024	0.042	0.6338329	1.4
NHSL1	2.75E-05	-0.407254635	0.177	0.212	0.6630355	1.4
INTS12	2.76E-05	-0.231236786	0.17	0.202	0.664583	1.4
CAMSAP1	2.81E-05	-0.140948224	0.058	0.084	0.6786976	1.4
METAP1	2.82E-05	-0.139030607	0.07	0.098	0.680755	1.4
LAMC2	2.87E-05	-0.304772884	0.275	0.302	0.6922336	1.4
CDA	2.90E-05	-0.153192712	0.005	0.017	0.6995426	1.4
GNB2L1	3.04E-05	0.100867236	0.756	0.697	0.7329328	1.4
RIPK2	3.10E-05	-0.318204831	0.382	0.402	0.748401	1.4
CALR	3.11E-05	-0.239907622	0.165	0.196	0.7501595	1.4
PRDM10	3.13E-05	-0.133569825	0.061	0.088	0.7538247	1.4
TOMM7	3.14E-05	-0.139931853	0.918	0.903	0.7561873	1.4
FAM13A	3.18E-05	-0.467505388	0.371	0.379	0.7670643	1.4
TCEB3	3.20E-05	-0.122223382	0.054	0.079	0.7711068	1.4
SNRPB	3.23E-05	-0.162406057	0.063	0.089	0.7788738	1.4
CCNB1IP1	3.30E-05	-0.227816958	0.355	0.374	0.7966473	1.4
MAGT1	3.38E-05	-0.196654287	0.156	0.187	0.8143963	1.4
C4orf32	3.39E-05	-0.142625089	0.044	0.067	0.8179906	1.4
THSD4	3.40E-05	-0.319102063	0.403	0.421	0.8186916	1.4
UCHL3	3.47E-05	-0.156942369	0.117	0.149	0.836071	1.4
PSME2	3.48E-05	0.155092953	0.393	0.339	0.8392941	1.4
S100A16	3.59E-05	-0.21195727	0.08	0.108	0.8648809	1.4
LALBA	3.70E-05	-0.402384212	0.005	0.016	0.8915069	1.4
NOLC1	3.70E-05	-0.122696223	0.057	0.082	0.8931228	1.4
PLIN2	3.78E-05	-0.315256666	0.131	0.161	0.9111092	1.4
TMEM57	3.86E-05	-0.220147203	0.149	0.179	0.9305384	1.4
RP11-111E	3.89E-05	-0.10579965	0.045	0.069	0.9375248	1.4
B4GALNT3	4.07E-05	-0.172901061	0.034	0.055	0.9815425	1.4
ZC3H15	4.26E-05	-0.201891262	0.245	0.276	1	1.4
COPS2	4.32E-05	-0.230083339	0.34	0.359	1	1.4
CSMD1	4.38E-05	0.124408467	0.097	0.07	1	1.4
RAC1	4.69E-05	-0.188385819	0.479	0.491	1	1.4
ATP2B4	4.79E-05	-0.338985311	0.256	0.279	1	1.4
TPD52	4.79E-05	-0.190699589	0.147	0.178	1	1.4
MAFF	4.89E-05	-0.199549359	0.179	0.211	1	1.4
TMSB10	5.35E-05	-0.370927195	0.637	0.624	1	1.4
RP11-317G	5.55E-05	-0.109469282	0.036	0.057	1	1.4

MYO1E	5.63E-05	-0.301798222	0.483	0.49	1	1.4
ARMCX3	5.87E-05	-0.155202989	0.126	0.158	1	1.4
IPO5	6.44E-05	-0.183283157	0.137	0.167	1	1.4
PEAK1	6.47E-05	-0.249607626	0.158	0.188	1	1.4
JUND	6.53E-05	-0.140272096	0.055	0.078	1	1.4
PAK3	6.53E-05	0.133127195	0.106	0.079	1	1.4
RBBP7	6.56E-05	-0.101625623	0.061	0.086	1	1.4
PGAM1	6.89E-05	-0.114410887	0.083	0.112	1	1.4
KLF5	6.91E-05	-0.197064237	0.204	0.237	1	1.4
KLRD1	6.99E-05	-0.170277718	0.055	0.079	1	1.4
CLCA4	7.10E-05	-0.11376216	0.022	0.04	1	1.4
WDR20	7.10E-05	0.104973395	0.108	0.081	1	1.4
C1orf198	7.55E-05	-0.128629928	0.048	0.07	1	1.4
CAV2	8.35E-05	-0.11976351	0.049	0.072	1	1.4
CYTH3	8.48E-05	-0.158698353	0.067	0.092	1	1.4
FEZ2	8.55E-05	-0.251008076	0.373	0.39	1	1.4
LINGO2	8.91E-05	-0.276080644	0.05	0.073	1	1.4
EIF5A	9.01E-05	-0.140144997	0.046	0.067	1	1.4
ARHGEF26	9.66E-05	-0.19004955	0.055	0.079	1	1.4
PIGA	9.69E-05	-0.153472275	0.066	0.09	1	1.4
TAGLN	0.000103	-0.22072563	0.014	0.029	1	1.4
NUTF2	0.000103	-0.108650206	0.052	0.075	1	1.4
AREG	0.000103	-0.16085501	0.079	0.105	1	1.4
TPM3	0.000109	-0.125011534	0.091	0.118	1	1.4
FOXK1	0.000111	-0.145093367	0.125	0.155	1	1.4
COPB1	0.000112	-0.175030439	0.192	0.22	1	1.4
RP11-557C	0.000118	-0.144685833	0.045	0.066	1	1.4
PPTC7	0.00012	-0.285625385	0.197	0.224	1	1.4
PALLD	0.000122	-0.208724911	0.511	0.516	1	1.4
RNF145	0.000122	-0.359126384	0.582	0.557	1	1.4
PJA2	0.000126	-0.234654149	0.208	0.236	1	1.4
RPL22	0.000127	-0.150124032	0.816	0.787	1	1.4
LIMA1	0.00013	-0.233559292	0.362	0.379	1	1.4
DHX15	0.000132	-0.177946924	0.187	0.217	1	1.4
COX6C	0.000135	-0.167613698	0.632	0.625	1	1.4
PCNXL2	0.000138	-0.166097766	0.811	0.813	1	1.4
PTP4A2	0.000145	-0.146936903	0.095	0.122	1	1.4
CCDC82	0.000149	-0.199329563	0.229	0.258	1	1.4
S100A1	0.00015	-0.115056057	0.065	0.09	1	1.4
F3	0.000151	-0.155656879	0.047	0.069	1	1.4
USP54	0.000157	-0.11522363	0.581	0.49	1	1.4
OSBPL6	0.000166	-0.100917289	0.025	0.041	1	1.4
SEC24B	0.000169	-0.240714262	0.285	0.305	1	1.4
CCDC50	0.00017	-0.158596563	0.134	0.164	1	1.4
PAFAH1B1	0.000172	-0.225817274	0.354	0.374	1	1.4
PFDN5	0.000175	-0.119781077	0.901	0.896	1	1.4
CENPW	0.000176	-0.171673188	0.109	0.136	1	1.4

CDC27	0.000195	-0.244618647	0.221	0.248	1	1.4
BCCIP	0.000197	-0.141919171	0.072	0.096	1	1.4
CCDC59	0.000202	-0.158880537	0.159	0.189	1	1.4
RP11-314N	0.000207	-0.228200016	0.126	0.153	1	1.4
AIMP1	0.000208	-0.209062361	0.407	0.426	1	1.4
EIF4A1	0.000211	-0.172794341	0.582	0.585	1	1.4
EIF3H	0.000226	-0.146041661	0.721	0.691	1	1.4
DDX24	0.000228	-0.188511103	0.639	0.621	1	1.4
COX4I1	0.000232	-0.145156086	0.807	0.775	1	1.4
KCNMA1	0.000234	-0.146766195	0.031	0.049	1	1.4
RPS19	0.000235	0.206283231	0.294	0.253	1	1.4
TNC	0.000236	0.135363583	0.135	0.107	1	1.4
UBB	0.000251	-0.214440395	0.802	0.767	1	1.4
SPATA5	0.000251	-0.164808848	0.114	0.142	1	1.4
BRK1	0.000253	-0.126284366	0.703	0.688	1	1.4
KRT18	0.000258	-0.142501802	0.067	0.09	1	1.4
RP11-608O	0.000258	-0.1140207	0.739	0.737	1	1.4
IQCJ-SCHIP	0.000259	-0.114467441	0.059	0.082	1	1.4
PFKFB3	0.000267	-0.103350327	0.059	0.081	1	1.4
DYNLRB1	0.000268	-0.180167818	0.248	0.274	1	1.4
SEC24D	0.000274	-0.291614195	0.285	0.304	1	1.4
MT-ND4L	0.000291	-0.190742988	0.534	0.534	1	1.4
POLR2K	0.0003	-0.222624745	0.32	0.338	1	1.4
RRP15	0.000301	-0.100662924	0.053	0.075	1	1.4
CCT8	0.000305	-0.191385341	0.369	0.383	1	1.4
ZBTB11	0.000313	-0.100789246	0.045	0.065	1	1.4
CHCHD2	0.000315	-0.208266254	0.212	0.241	1	1.4
TPRKB	0.000341	-0.178692878	0.193	0.221	1	1.4
ALDH1A3	0.000341	-0.215205183	0.533	0.544	1	1.4
RPL9	0.000352	-0.15425259	0.681	0.668	1	1.4
GADD45B	0.00037	-0.173342823	0.04	0.059	1	1.4
STK39	0.000376	0.171393225	0.151	0.123	1	1.4
VPS37B	0.000381	-0.119357667	0.134	0.162	1	1.4
PTMA	0.000381	0.174144544	0.627	0.573	1	1.4
ATXN2L	0.000394	-0.104531406	0.061	0.083	1	1.4
S100A2	0.000397	-0.551945227	0.084	0.107	1	1.4
ORMDL2	0.000399	-0.171192465	0.144	0.17	1	1.4
KCNK1	0.000399	-0.178955628	0.152	0.18	1	1.4
EIF5B	0.000421	-0.18953037	0.202	0.229	1	1.4
IQCG	0.000426	-0.245837174	0.201	0.225	1	1.4
APP	0.000428	-0.117050291	0.787	0.764	1	1.4
FRMD6	0.000428	-0.251413373	0.178	0.202	1	1.4
GTF2F2	0.000429	-0.142519684	0.125	0.152	1	1.4
FRAS1	0.00043	-0.104993593	0.049	0.07	1	1.4
LAMC1	0.000449	-0.254545388	0.249	0.272	1	1.4
ARPC2	0.000456	-0.17234426	0.157	0.183	1	1.4
PKIG	0.000474	-0.117469976	0.044	0.063	1	1.4

TMEM41B	0.000489	-0.168532044	0.177	0.204	1	1.4
ABTB2	0.00049	-0.212159061	0.22	0.248	1	1.4
PTCHD1-AS	0.000502	-0.164566571	0.088	0.112	1	1.4
MPRIP-AS1	0.000517	-0.105457142	0.042	0.061	1	1.4
DDX18	0.000538	-0.211501101	0.356	0.372	1	1.4
HNRNPM	0.000538	-0.177641567	0.175	0.2	1	1.4
RHEB	0.000538	-0.186593919	0.233	0.257	1	1.4
GNAI3	0.000546	-0.148313822	0.14	0.166	1	1.4
GPC5	0.000566	-0.139155995	0.028	0.044	1	1.4
TMEM258	0.000591	-0.129214902	0.627	0.632	1	1.4
RPSA	0.000619	-0.134431444	0.928	0.918	1	1.4
DOCK5	0.00063	-0.205809516	0.199	0.226	1	1.4
RAB1A	0.000658	-0.216776519	0.456	0.454	1	1.4
TRA2B	0.000663	-0.202181014	0.179	0.205	1	1.4
YBX1	0.000671	-0.157017404	0.323	0.347	1	1.4
SDK2	0.000676	-0.216261946	0.055	0.074	1	1.4
RP11-231C	0.000679	-0.173160564	0.175	0.2	1	1.4
BLVRA	0.000718	-0.11087841	0.074	0.095	1	1.4
NUDCD1	0.00076	-0.14169757	0.102	0.126	1	1.4
MBIP	0.000765	-0.158046347	0.144	0.169	1	1.4
RHOB	0.000799	-0.104454088	0.038	0.055	1	1.4
MRPL42	0.000811	-0.145551017	0.165	0.191	1	1.4
FAM126B	0.000822	-0.207380048	0.141	0.165	1	1.4
PPP2R2D	0.000824	-0.150337832	0.108	0.132	1	1.4
EPCAM	0.000886	-0.160527542	0.176	0.201	1	1.4
ITGA3	0.000902	-0.151496629	0.1	0.124	1	1.4
SERINC5	0.000948	-0.191397344	0.221	0.247	1	1.4
NFE2L3	0.000957	-0.215351442	0.077	0.098	1	1.4
ATP6V0E1	0.000982	-0.161778624	0.691	0.659	1	1.4
SPTAN1	0.000987	-0.166656747	0.163	0.189	1	1.4
PLCB1	0.00102	-0.282597438	0.388	0.402	1	1.4
UBR4	0.001034	-0.149134405	0.172	0.198	1	1.4
RPL10	0.001044	-0.137208987	0.954	0.968	1	1.4
RPS15	0.001057	0.182552985	0.192	0.163	1	1.4
CD63	0.00108	-0.220682431	0.198	0.221	1	1.4
RGCC	0.001089	-0.104505793	0.041	0.058	1	1.4
SLIT2	0.001133	-0.13352274	0.033	0.048	1	1.4
EIF3M	0.001135	-0.184059032	0.492	0.486	1	1.4
AIM1	0.001171	-0.25382156	0.304	0.318	1	1.4
NKX3-1	0.001171	-0.183472277	0.074	0.094	1	1.4
SERPINB9	0.001176	-0.439974474	0.139	0.165	1	1.4
BLZF1	0.001208	-0.129831649	0.077	0.097	1	1.4
AK6	0.001251	-0.178684167	0.188	0.212	1	1.4
TNFRSF8	0.001277	-0.146208101	0.116	0.14	1	1.4
CXCL10	0.001341	0.148292021	0.013	0.006	1	1.4
ATP5B	0.001415	-0.171239882	0.213	0.236	1	1.4
HCAR2	0.00142	-0.18898052	0.232	0.191	1	1.4

NUS1	0.001433	-0.143298665	0.102	0.124	1	1.4
FAM46A	0.001489	-0.148714576	0.094	0.116	1	1.4
VCP	0.00152	-0.14094912	0.095	0.116	1	1.4
TMEM263	0.001543	-0.112100254	0.09	0.112	1	1.4
RPL29	0.001545	0.159824756	0.328	0.289	1	1.4
PAICS	0.001589	-0.118784506	0.193	0.218	1	1.4
COG5	0.001629	-0.238802754	0.463	0.458	1	1.4
BAIAP2L1	0.001639	-0.104819914	0.96	0.969	1	1.4
FNBP1	0.001648	-0.124532878	0.552	0.464	1	1.4
ARL4A	0.001676	-0.18005439	0.116	0.139	1	1.4
CDV3	0.001709	-0.11751194	0.091	0.112	1	1.4
NDUFA1	0.00173	-0.147239011	0.563	0.56	1	1.4
ATP2A2	0.001745	-0.17475149	0.234	0.256	1	1.4
KDM5C	0.001753	-0.101840731	0.07	0.09	1	1.4
MRPL22	0.001756	-0.179536529	0.245	0.264	1	1.4
WASF2	0.001767	-0.132619329	0.196	0.222	1	1.4
ARHGAP10	0.001818	-0.179118063	0.119	0.141	1	1.4
SEMA3E	0.00184	-0.103835999	0.058	0.077	1	1.4
USP31	0.001958	-0.138490439	0.113	0.136	1	1.4
LRP6	0.001981	-0.251365642	0.28	0.295	1	1.4
PRDX1	0.001996	-0.206680077	0.734	0.713	1	1.4
EIF4H	0.00205	-0.117388144	0.077	0.096	1	1.4
DYNLT3	0.002077	-0.1656503	0.202	0.223	1	1.4
PPP4R1	0.0021	-0.137097077	0.187	0.21	1	1.4
VAT1	0.002113	-0.119430062	0.104	0.127	1	1.4
DPM1	0.002113	-0.18125746	0.258	0.278	1	1.4
NEK10	0.002146	-0.138421813	0.104	0.126	1	1.4
DSTN	0.002192	-0.188024342	0.486	0.485	1	1.4
ZNF292	0.0023	-0.180084264	0.656	0.628	1	1.4
LDHB	0.002335	-0.166063205	0.78	0.739	1	1.4
PPP1R13L	0.002341	-0.135999061	0.089	0.11	1	1.4
MBNL2	0.002342	-0.161674484	0.396	0.411	1	1.4
FBXO42	0.002368	-0.181604916	0.157	0.179	1	1.4
ZSWIM6	0.002368	-0.216749514	0.336	0.35	1	1.4
HSBP1	0.00238	-0.172396085	0.489	0.481	1	1.4
DSC2	0.002383	-0.203497165	0.225	0.245	1	1.4
TNFRSF10B	0.002438	-0.173737627	0.18	0.203	1	1.4
S100A13	0.002489	-0.156584868	0.621	0.599	1	1.4
DNAJB1	0.002536	-0.188584288	0.09	0.109	1	1.4
MPP5	0.002548	-0.17692552	0.226	0.248	1	1.4
CSDE1	0.002629	-0.137010845	0.592	0.581	1	1.4
DNAJB9	0.002642	-0.104215028	0.049	0.066	1	1.4
ASAP1	0.002783	-0.214181615	0.228	0.248	1	1.4
PSMB6	0.002822	-0.107754074	0.09	0.11	1	1.4
LINC01420	0.003	-0.142358796	0.409	0.355	1	1.4
SLC38A2	0.003063	-0.104163208	0.158	0.182	1	1.4
ATP5J	0.003144	-0.171941817	0.5	0.495	1	1.4

FNBP1L	0.003241	-0.260921793	0.435	0.434	1	1.4
SPTLC2	0.003332	-0.193557254	0.149	0.169	1	1.4
BCL2A1	0.00336	-0.216438648	0.142	0.164	1	1.4
VDAC2	0.003502	-0.119160207	0.108	0.129	1	1.4
SRFBP1	0.003601	-0.172020975	0.181	0.202	1	1.4
UFC1	0.003625	-0.154806406	0.514	0.509	1	1.4
FBXO3	0.003658	-0.120661476	0.085	0.104	1	1.4
B3GNT5	0.00372	-0.121719089	0.088	0.108	1	1.4
TATDN1	0.003851	-0.187655779	0.301	0.311	1	1.4
PHACTR4	0.003908	-0.185493092	0.39	0.398	1	1.4
PPARG	0.00394	-0.106991312	0.049	0.065	1	1.4
NOP58	0.003997	-0.142947486	0.203	0.222	1	1.4
S100A4	0.004029	-0.125731451	0.083	0.102	1	1.4
GAB2	0.004214	-0.174551839	0.206	0.226	1	1.4
MED13	0.004316	-0.199930177	0.343	0.352	1	1.4
RNF11	0.004434	-0.107884797	0.14	0.163	1	1.4
PIWIL1	0.00467	-0.129832763	0.047	0.062	1	1.4
NPTN	0.004867	-0.123150415	0.105	0.124	1	1.4
CAPZB	0.005348	-0.118811339	0.126	0.147	1	1.4
CMTM7	0.005379	-0.182419247	0.216	0.235	1	1.4
HDAC2	0.005464	-0.138318717	0.194	0.215	1	1.4
MCFD2	0.00557	-0.114158362	0.14	0.162	1	1.4
RP11-795H	0.005632	-0.182614196	0.434	0.443	1	1.4
WBP4	0.005694	-0.141242147	0.11	0.129	1	1.4
DCTN6	0.005911	-0.168695684	0.336	0.348	1	1.4
RPS25	0.005919	-0.101172738	0.872	0.864	1	1.4
EIF3K	0.006001	-0.220430995	0.484	0.47	1	1.4
VPS35	0.006002	-0.172467582	0.274	0.288	1	1.4
RPS9	0.006428	0.164577838	0.324	0.29	1	1.4
ATP11B	0.006447	-0.168807442	0.182	0.201	1	1.4
YEATS2	0.006619	-0.123019568	0.129	0.149	1	1.4
TSHZ2	0.006675	-0.242028332	0.461	0.467	1	1.4
UGP2	0.006702	-0.146371053	0.532	0.522	1	1.4
FAM219A	0.0069	-0.109950675	0.071	0.087	1	1.4
MLF1	0.007022	-0.160316647	0.214	0.234	1	1.4
INSIG1	0.007037	-0.1095721	0.094	0.112	1	1.4
RBM8A	0.007042	-0.182020348	0.52	0.508	1	1.4
PDLIM1	0.007073	-0.10327612	0.074	0.092	1	1.4
CHD6	0.007353	-0.198077068	0.285	0.299	1	1.4
CLIP2	0.007478	-0.115009987	0.171	0.193	1	1.4
FAM3C	0.00761	-0.132728702	0.143	0.162	1	1.4
EIF5	0.007818	-0.178595819	0.189	0.207	1	1.4
MYO3B	0.008043	-0.17377893	0.191	0.212	1	1.4
SH3D19	0.008112	-0.165409204	0.337	0.347	1	1.4
ATG14	0.008116	-0.120959624	0.087	0.104	1	1.4
RPL8	0.008381	0.1376344	0.206	0.181	1	1.4
IAH1	0.008436	-0.12859213	0.123	0.142	1	1.4

EIF3J	0.008834	-0.154554037	0.138	0.156	1	1.4
TM9SF3	0.008839	-0.173604115	0.272	0.284	1	1.4
FUNDC2	0.008909	-0.154598931	0.133	0.149	1	1.4
ACAT2	0.009015	-0.181284887	0.28	0.295	1	1.4
RAB5A	0.009239	-0.144085951	0.208	0.226	1	1.4
RNF149	0.009345	-0.200922388	0.291	0.3	1	1.4
SLU7	0.00937	-0.128579784	0.126	0.144	1	1.4
PIGR	0.009384	-0.195163623	0.516	0.52	1	1.4
AGFG1	0.009394	-0.221408464	0.412	0.408	1	1.4
RSBN1L	0.009471	-0.168840835	0.11	0.127	1	1.4
HSPA1A	0.009522	-0.198323756	0.041	0.054	1	1.4
NCKAP1	0.009572	-0.184594597	0.405	0.411	1	1.4
PTGES3	0.009637	-0.136656985	0.198	0.217	1	1.4
NDUFV2	0.009712	-0.128978699	0.14	0.159	1	1.4
NDUFS4	0.009946	-0.164581987	0.474	0.458	1	1.4
CDC5L	0.009971	-0.138597523	0.238	0.256	1	1.4
SRSF11	0.010003	-0.132348223	0.486	0.477	1	1.4
HMGCS1	0.010009	-0.148898508	0.221	0.24	1	1.4
SPOPL	0.010082	-0.117414556	0.136	0.155	1	1.4
7-Mar	0.010135	-0.164428809	0.272	0.285	1	1.4
ARL8B	0.010294	-0.202669963	0.244	0.256	1	1.4
TOP2B	0.010364	-0.131192887	0.164	0.183	1	1.4
ZCCHC2	0.010567	-0.209556025	0.301	0.312	1	1.4
ANKRD11	0.010941	-0.168018107	0.282	0.296	1	1.4
FYTTD1	0.011009	-0.114219481	0.103	0.119	1	1.4
FAM129B	0.011463	-0.133170052	0.097	0.114	1	1.4
FOSB	0.011558	-0.201086377	0.423	0.427	1	1.4
ARG2	0.011636	-0.143553508	0.14	0.159	1	1.4
SON	0.011719	-0.192890706	0.708	0.683	1	1.4
GSTA1	0.012298	-0.318617601	0.078	0.093	1	1.4
EGLN3	0.012338	-0.192539134	0.103	0.12	1	1.4
BBC3	0.012582	-0.103483521	0.058	0.072	1	1.4
HLA-DRB1	0.012888	-0.113365988	0.054	0.068	1	1.4
PKM	0.013282	-0.175425229	0.242	0.259	1	1.4
GRAMD3	0.013479	-0.192238271	0.368	0.375	1	1.4
MAPKBP1	0.013853	-0.12052095	0.085	0.101	1	1.4
PEG10	0.014052	-0.102042237	0.054	0.068	1	1.4
KPNA6	0.014498	-0.168904305	0.268	0.281	1	1.4
CLIP4	0.01468	-0.163059274	0.223	0.239	1	1.4
GABARAPL	0.014808	-0.117448849	0.142	0.159	1	1.4
RP11-659O	0.015161	-0.152833996	0.176	0.192	1	1.4
SLC16A13	0.015256	-0.203514791	0.044	0.056	1	1.4
LINC01492	0.01538	-0.114109289	0.121	0.136	1	1.4
MPDZ	0.015567	-0.154472492	0.168	0.184	1	1.4
ERGIC1	0.015767	-0.108372378	0.085	0.101	1	1.4
RGS2	0.015937	-0.136766736	0.233	0.204	1	1.4
RP11-496N	0.016216	-0.113128909	0.011	0.019	1	1.4

LMNA	0.016234	-0.166916487	0.193	0.21	1	1.4
RP11-779O	0.016239	-0.203729569	0.205	0.218	1	1.4
C6orf132	0.01648	-0.106252096	0.151	0.17	1	1.4
CHD1	0.016526	-0.169699695	0.221	0.236	1	1.4
TAX1BP1	0.016593	-0.10898032	0.693	0.661	1	1.4
KIAA0922	0.016689	-0.414951075	0.258	0.263	1	1.4
RAB11A	0.017308	-0.18362158	0.613	0.596	1	1.4
MTURN	0.017511	-0.101400043	0.094	0.11	1	1.4
TOMM20	0.017541	-0.144793185	0.24	0.253	1	1.4
HLA-DRA	0.017745	-0.219843732	0.144	0.161	1	1.4
IRS2	0.018292	-0.149600083	0.354	0.364	1	1.4
DSP	0.018482	-0.169038337	0.34	0.344	1	1.4
YPEL5	0.018576	-0.161917019	0.38	0.383	1	1.4
KIF13A	0.018977	-0.22805182	0.263	0.274	1	1.4
PPFIBP1	0.019039	-0.127579059	0.159	0.176	1	1.4
UBQLN1	0.01934	-0.153654765	0.239	0.252	1	1.4
GCLM	0.019837	-0.174437738	0.145	0.16	1	1.4
SRSF10	0.019882	-0.112199241	0.132	0.149	1	1.4
SCYL2	0.020785	-0.163002754	0.247	0.259	1	1.4
RAB3IP	0.021492	-0.133075364	0.108	0.123	1	1.4
LGALS3	0.021549	-0.245446836	0.172	0.185	1	1.4
DYRK1A	0.021745	-0.198057223	0.359	0.359	1	1.4
CKS1B	0.021782	-0.166421706	0.393	0.39	1	1.4
STK40	0.0221	-0.135637277	0.118	0.134	1	1.4
BFAR	0.022312	-0.123764706	0.186	0.2	1	1.4
COPB2	0.022351	-0.140324463	0.198	0.213	1	1.4
CNIH4	0.022957	-0.136459718	0.378	0.383	1	1.4
MARK3	0.024081	-0.153542776	0.437	0.431	1	1.4
RUVBL1	0.024322	-0.111511301	0.117	0.132	1	1.4
LARS	0.02541	-0.139742639	0.235	0.245	1	1.4
MAP3K9	0.025626	-0.136548862	0.161	0.177	1	1.4
AC090498.	0.025715	-0.148566339	0.388	0.39	1	1.4
CLIC4	0.026481	-0.133780888	0.668	0.651	1	1.4
EIF4G2	0.027329	-0.15104	0.322	0.329	1	1.4
BTG3	0.02741	-0.12221851	0.184	0.198	1	1.4
NTN4	0.027551	-0.19815721	0.168	0.182	1	1.4
ADAM32	0.028129	-0.17701695	0.114	0.128	1	1.4
GAREM1	0.029615	-0.139723399	0.258	0.269	1	1.4
CHMP4B	0.030791	-0.137769801	0.114	0.127	1	1.4
PID1	0.030893	-0.148455267	0.127	0.142	1	1.4
YY1	0.030964	-0.122983523	0.159	0.175	1	1.4
SSBP2	0.03123	-0.333163587	0.348	0.344	1	1.4
MAP3K8	0.031647	-0.172565123	0.272	0.283	1	1.4
ATF4	0.031894	-0.154262782	0.36	0.362	1	1.4
OSTC	0.032384	-0.155042377	0.516	0.501	1	1.4
ARCN1	0.032523	-0.118543672	0.093	0.106	1	1.4
CCNK	0.032814	-0.114956835	0.113	0.127	1	1.4

WDR45B	0.033116	-0.158487832	0.281	0.289	1	1.4
SNRPG	0.033877	-0.150463696	0.564	0.538	1	1.4
ETF1	0.034532	-0.122872715	0.136	0.151	1	1.4
NACA2	0.034715	-0.125007907	0.647	0.633	1	1.4
CANX	0.036038	-0.121025072	0.33	0.339	1	1.4
TM9SF2	0.036078	-0.157609086	0.18	0.192	1	1.4
PHF3	0.036147	-0.145829037	0.342	0.345	1	1.4
IFIT2	0.036631	-0.18034343	0.033	0.042	1	1.4
VPS26A	0.037112	-0.116755274	0.155	0.169	1	1.4
CMIP	0.037362	-0.185393575	0.36	0.36	1	1.4
ACSS2	0.037492	-0.122550212	0.168	0.182	1	1.4
DNTTIP2	0.037795	-0.160656724	0.208	0.219	1	1.4
ZC3H11A	0.03877	-0.136273495	0.232	0.244	1	1.4
REEP3	0.039093	-0.167130758	0.238	0.248	1	1.4
HNRNPU	0.04064	-0.113285489	0.093	0.106	1	1.4
RP11-273G	0.040707	-0.111472898	0.065	0.077	1	1.4
TXLNG	0.04124	-0.102408561	0.101	0.115	1	1.4
GLS	0.042417	-0.203950998	0.253	0.26	1	1.4
NAA15	0.042664	-0.128307758	0.136	0.149	1	1.4
ATP5EP2	0.042952	-0.155788476	0.197	0.209	1	1.4
SPEN	0.043356	-0.180723574	0.266	0.274	1	1.4
NSA2	0.043846	-0.149590911	0.427	0.416	1	1.4
FOXJ3	0.043884	-0.17238604	0.292	0.295	1	1.4
NCL	0.044127	-0.172186729	0.197	0.209	1	1.4
C2orf88	0.045281	-0.19176764	0.394	0.384	1	1.4
GSPT1	0.045797	-0.128993504	0.207	0.219	1	1.4
CYP24A1	0.046983	-0.295590085	0.221	0.232	1	1.4
PSMD11	0.050921	-0.131751452	0.295	0.304	1	1.4
CTC-425F1.	0.052391	-0.108006092	0.074	0.085	1	1.4
VBP1	0.052954	-0.10152774	0.142	0.155	1	1.4
PVRL2	0.053242	-0.134390277	0.167	0.18	1	1.4
NR3C1	0.054184	-0.106462906	0.24	0.252	1	1.4
DUSP1	0.056112	-0.140886109	0.18	0.192	1	1.4
RGS6	0.056751	-0.1289029	0.135	0.149	1	1.4
TCEANC2	0.057508	-0.14669676	0.206	0.216	1	1.4
ATAD1	0.05757	-0.142132634	0.15	0.161	1	1.4
PSMC1	0.057684	-0.170851373	0.365	0.362	1	1.4
HBS1L	0.059509	-0.130809921	0.189	0.198	1	1.4
CD59	0.061328	-0.218468189	0.952	0.834	1	1.4
ARHGAP21	0.061597	-0.15437609	0.236	0.244	1	1.4
MAP7	0.061729	-0.157840565	0.331	0.332	1	1.4
MCF2L2	0.061861	-0.102295812	0.15	0.164	1	1.4
VEZT	0.063318	-0.129891437	0.337	0.34	1	1.4
WDR48	0.064704	-0.15454176	0.179	0.189	1	1.4
MAGOH	0.065043	-0.126090856	0.18	0.192	1	1.4
HSD17B6	0.065056	-0.154644588	0.029	0.037	1	1.4
6-Mar	0.067706	-0.153880639	0.345	0.343	1	1.4

GAPVD1	0.068337	-0.118438215	0.201	0.212	1	1.4
CTNNAL1	0.069481	-0.126752703	0.099	0.11	1	1.4
AKAP13	0.071807	-0.201889588	0.54	0.512	1	1.4
MED15	0.071813	-0.112833775	0.133	0.144	1	1.4
GABARAP	0.072384	-0.204179132	0.305	0.304	1	1.4
COCH	0.073096	-0.102224434	0.096	0.106	1	1.4
PSMD7	0.073432	-0.101698094	0.141	0.152	1	1.4
ZFP36L2	0.074135	-0.182892781	0.394	0.352	1	1.4
URB1	0.074607	-0.108231703	0.067	0.056	1	1.4
SNRPF	0.076259	-0.14196084	0.243	0.251	1	1.4
CHORDC1	0.076921	-0.100282363	0.089	0.1	1	1.4
STRAP	0.077323	-0.101114053	0.117	0.128	1	1.4
CCNG2	0.081142	-0.151024611	0.145	0.155	1	1.4
FASTKD2	0.081426	-0.108735622	0.095	0.106	1	1.4
SESTD1	0.082991	-0.108742161	0.654	0.613	1	1.4
HNRNPH1	0.084638	-0.136025866	0.418	0.414	1	1.4
BIRC3	0.084689	-0.226348343	0.862	0.812	1	1.4
GFPT1	0.085587	-0.153700457	0.203	0.21	1	1.4
ANKRD28	0.088165	-0.169397848	0.303	0.305	1	1.4
ATP1B3	0.088658	-0.144779745	0.264	0.27	1	1.4
AFAP1	0.090427	-0.102691157	0.118	0.13	1	1.4
CCT4	0.091049	-0.118824689	0.213	0.221	1	1.4
HBP1	0.09473	-0.117929203	0.221	0.229	1	1.4
SSBP1	0.096629	-0.137256601	0.45	0.439	1	1.4
MGAT5	0.101287	-0.169826675	0.31	0.31	1	1.4
UHRF1BP1I	0.10359	-0.112842441	0.188	0.196	1	1.4
ARL1	0.107594	-0.126088819	0.283	0.287	1	1.4
ARHGEF7	0.109781	-0.117318171	0.165	0.175	1	1.4
TBC1D8	0.113867	-0.166431368	0.412	0.403	1	1.4
EEF1B2	0.114092	-0.134118362	0.902	0.877	1	1.4
ZNF83	0.114887	-0.203792591	0.365	0.357	1	1.4
WDR26	0.118761	-0.116586564	0.204	0.212	1	1.4
DYNC1I2	0.118773	-0.110222675	0.382	0.374	1	1.4
AC026202.	0.123538	-0.19581953	0.193	0.197	1	1.4
LRRC23	0.128967	-0.171193389	0.093	0.103	1	1.4
SRP72	0.132663	-0.101882491	0.178	0.187	1	1.4
MTRNR2L8	0.133284	-0.119688966	0.196	0.203	1	1.4
SLC19A2	0.13555	-0.112041441	0.084	0.092	1	1.4
PPHLN1	0.140302	-0.138541211	0.408	0.397	1	1.4
CLIP1	0.143004	-0.147620258	0.39	0.382	1	1.4
FOXK2	0.144921	-0.1475386	0.303	0.301	1	1.4
NOCT	0.144996	-0.155593255	0.133	0.14	1	1.4
PTK2	0.148193	-0.108613102	0.586	0.557	1	1.4
ZNF462	0.152723	-0.140150562	0.354	0.347	1	1.4
FAM53C	0.154969	-0.120621816	0.206	0.213	1	1.4
RBM39	0.157141	-0.130398242	0.468	0.457	1	1.4
RTCB	0.158776	-0.157143194	0.467	0.446	1	1.4

HIF1A	0.160742	-0.202925409	0.396	0.39	1	1.4
TIMM9	0.161061	-0.14885639	0.22	0.221	1	1.4
UBE2D3	0.164417	-0.107920368	0.585	0.554	1	1.4
WDR33	0.167447	-0.135887537	0.314	0.312	1	1.4
MPZL3	0.170345	-0.125422185	0.308	0.307	1	1.4
BCLAF1	0.170823	-0.104635829	0.385	0.379	1	1.4
RNF19A	0.171408	-0.115045167	0.676	0.607	1	1.4
UAP1	0.173108	-0.101040457	0.26	0.235	1	1.4
LYST	0.174898	-0.132639509	0.181	0.162	1	1.4
RPS26	0.175287	-0.111049721	0.164	0.17	1	1.4
AC159540.	0.179723	-0.114476246	0.169	0.177	1	1.4
ACTR2	0.182676	-0.130352746	0.213	0.217	1	1.4
CDK7	0.185612	-0.100621764	0.112	0.12	1	1.4
ARIH2	0.186969	-0.10389648	0.122	0.129	1	1.4
SCGB2A1	0.187562	-0.179516507	0.04	0.046	1	1.4
PPFIA1	0.188587	-0.157398396	0.271	0.272	1	1.4
SERBP1	0.193173	-0.13350287	0.165	0.171	1	1.4
BCL2L14	0.197512	-0.113063461	0.143	0.149	1	1.4
TNRC6A	0.20634	-0.133524657	0.251	0.252	1	1.4
SSB	0.210779	-0.117356818	0.171	0.176	1	1.4
UBTD2	0.212915	-0.120447304	0.129	0.135	1	1.4
LSM3	0.219522	-0.130353051	0.324	0.32	1	1.4
TIMM10	0.220455	-0.100464472	0.25	0.253	1	1.4
NOP10	0.220655	-0.109051659	0.49	0.464	1	1.4
DHX9	0.221821	-0.13057432	0.188	0.192	1	1.4
RNF128	0.225958	-0.107020073	0.093	0.099	1	1.4
ZMAT2	0.232192	-0.121180135	0.224	0.226	1	1.4
BCAS2	0.233149	-0.118384449	0.233	0.236	1	1.4
EIF3A	0.235739	-0.127005685	0.351	0.342	1	1.4
NCOA3	0.239864	-0.154377403	0.291	0.289	1	1.4
MTMR3	0.241072	-0.128773003	0.162	0.168	1	1.4
CLDN4	0.247792	-0.25267577	0.213	0.218	1	1.4
PRPF40A	0.248352	-0.105458696	0.171	0.176	1	1.4
HNRNPH2	0.254502	-0.123883109	0.224	0.226	1	1.4
LMBRD1	0.256104	-0.101283527	0.236	0.239	1	1.4
BARX2	0.258163	-0.108447793	0.383	0.383	1	1.4
DAPP1	0.258163	-0.111289322	0.449	0.4	1	1.4
SERP1	0.258635	-0.130902469	0.277	0.273	1	1.4
RAP1B	0.260285	-0.158737236	0.425	0.409	1	1.4
GNA12	0.260498	-0.171012248	0.37	0.354	1	1.4
ASXL1	0.262146	-0.137000941	0.266	0.265	1	1.4
ATP5E	0.267525	-0.123436719	0.474	0.454	1	1.4
RIC3	0.273231	-0.219408003	0.086	0.091	1	1.4
RDX	0.274968	-0.131372791	0.216	0.216	1	1.4
ABL2	0.277646	-0.105212801	0.201	0.205	1	1.4
KCTD3	0.280481	-0.165792605	0.201	0.2	1	1.4
MYO1D	0.2861	-0.145883703	0.266	0.267	1	1.4

GHITM	0.287035	-0.106012391	0.499	0.475	1	1.4
ST6GALNA(	0.297721	-0.173166781	0.393	0.386	1	1.4
CNOT1	0.298094	-0.113005351	0.183	0.187	1	1.4
CDK6	0.299453	-0.149278899	0.228	0.23	1	1.4
FRMD5	0.310519	-0.131652007	0.168	0.17	1	1.4
PITPNC1	0.312042	-0.128800677	0.212	0.215	1	1.4
UQCRC2	0.317993	-0.116756813	0.463	0.443	1	1.4
TBC1D23	0.327537	-0.120003723	0.155	0.158	1	1.4
SH3PXD2A	0.338917	-0.133285778	0.145	0.149	1	1.4
AZGP1	0.339302	-0.120178947	0.346	0.339	1	1.4
PPP6R3	0.340125	-0.139501917	0.447	0.424	1	1.4
ERC1	0.344632	-0.120381998	0.299	0.295	1	1.4
AP000487.	0.344969	-0.132184206	0.202	0.205	1	1.4
ALDOA	0.358764	-0.286553537	0.118	0.122	1	1.4
ZC3H7A	0.366438	-0.159614377	0.191	0.193	1	1.4
RAB3GAP1	0.370844	-0.100670845	0.301	0.297	1	1.4
EIF1B	0.374136	-0.1151247	0.285	0.28	1	1.4
EDF1	0.374997	-0.132366055	0.074	0.079	1	1.4
NEK7	0.393114	-0.110371352	0.201	0.202	1	1.4
VDAC3	0.410948	-0.111148714	0.354	0.343	1	1.4
ARHGDIB	0.412764	-0.110147823	0.177	0.18	1	1.4
S100A11	0.429251	-0.211490206	0.777	0.68	1	1.4
TJP1	0.442023	-0.144252999	0.401	0.386	1	1.4
RPS3	0.460258	-0.145634971	0.937	0.923	1	1.4
CHP1	0.473546	-0.102586738	0.399	0.382	1	1.4
FBXO28	0.47593	-0.113757145	0.202	0.202	1	1.4
TJP2	0.494721	-0.123414837	0.45	0.404	1	1.4
EIF3D	0.495557	-0.107893232	0.425	0.405	1	1.4
MOK	0.497206	-0.12979513	0.036	0.039	1	1.4
EIF1	0.499119	-0.159914954	0.44	0.424	1	1.4
PI3	0.499573	-0.684558042	0.304	0.293	1	1.4
UBL5	0.539982	-0.115838143	0.393	0.373	1	1.4
ABRACL	0.541358	-0.125217253	0.401	0.38	1	1.4
KRT8	0.556265	-0.138311049	0.21	0.197	1	1.4
ITGB6	0.557371	-0.140492538	0.335	0.308	1	1.4
CTA-293F1	0.558615	-0.119220399	0.125	0.126	1	1.4
EIF2S2	0.559094	-0.115239151	0.28	0.272	1	1.4
HECTD1	0.582216	-0.161935563	0.27	0.262	1	1.4
NCALD	0.589636	-0.226439651	0.223	0.215	1	1.4
SEC63	0.599419	-0.100637744	0.255	0.25	1	1.4
BACH1	0.603818	-0.137399885	0.317	0.303	1	1.4
CTD-3088G	0.620562	-0.112435255	0.053	0.055	1	1.4
APEX1	0.641461	-0.106575285	0.275	0.254	1	1.4
FRYL	0.643267	-0.10620237	0.28	0.271	1	1.4
RAB6A	0.651673	-0.10719306	0.301	0.29	1	1.4
FMNL2	0.655489	-0.118157487	0.469	0.432	1	1.4
TET2	0.674496	-0.102347919	0.215	0.21	1	1.4

HSPA8	0.694134	-0.164520777	0.591	0.54	1	1.4
RAP1A	0.702423	-0.104536099	0.215	0.21	1	1.4
ARID4B	0.721281	-0.113386745	0.533	0.493	1	1.4
TMEM41A	0.724366	-0.104690579	0.215	0.21	1	1.4
UBR5	0.745116	-0.103302211	0.43	0.401	1	1.4
EIF2B5	0.75869	-0.103285785	0.602	0.554	1	1.4
LAPTM4A	0.781781	-0.109024339	0.244	0.236	1	1.4
GREB1L	0.815471	-0.100287922	0.14	0.135	1	1.4
EPB41L2	0.816667	-0.173038258	0.196	0.191	1	1.4
KMT2E	0.817686	-0.102403588	0.427	0.394	1	1.4
PPIB	0.82393	-0.100100945	0.119	0.118	1	1.4
BDP1	0.828718	-0.103019414	0.217	0.206	1	1.4
PDK3	0.859392	-0.120375109	0.291	0.278	1	1.4
GTF2I	0.875103	-0.12636472	0.46	0.426	1	1.4
OLA1	0.889822	-0.103318248	0.253	0.243	1	1.4
SQSTM1	0.891057	-0.108303164	0.289	0.278	1	1.4
GPM6B	0.940081	-0.101508148	0.211	0.204	1	1.4
PAN3	0.942292	-0.106209232	0.61	0.562	1	1.4
TP53BP2	0.959806	-0.107598932	0.162	0.157	1	1.4
FNDC3A	0.969192	-0.113565865	0.343	0.324	1	1.4
LAMB3	0.972559	-0.376321034	0.306	0.283	1	1.4
IDI1	0.988125	-0.117261779	0.222	0.213	1	1.4
SBF2	0.990559	-0.121160493	0.702	0.642	1	1.4
PPIL4	0.991425	-0.106042324	0.162	0.157	1	1.4
